# A metabolite-bridged complex between SerRS and SIRT2 couples NAD⁺ metabolism to translation control

**DOI:** 10.1038/s41467-026-75266-4

**Published:** 2026-07-08

**Authors:** Qian Zhang, Huimin Zhang, Marscha Hirschi, Sheng Li, Gabriel C. Lander, Jie Yang, Xiang-Lei Yang

**Affiliations:** 1 Department of Molecular Medicine, The Scripps Research Institute, La Jolla, CA USA; 2 Department of Integrative Structural and Computational Biology, The Scripps Research Institute, La Jolla, CA USA; 3 Department of Medicine and UCSD DXMS proteomics Resource, University of California, San Diego, La Jolla, CA USA; 4 Department of Biochemistry and Molecular Genetics, University of Virginia School of Medicine, Charlottesville, VA USA; 5 Present Address: Institute of Systems and Physical Biology, Shenzhen Bay Laboratory, Shenzhen, China

**Keywords:** Cryoelectron microscopy, tRNAs, RNA-binding proteins, Enzyme mechanisms, Acetylation

## Abstract

Cellular homeostasis requires tight coordination between metabolic and translational networks. Here we identify a direct molecular link between these processes through a cryo-EM structure of human cytosolic seryl-tRNA synthetase (SerRS) in complex with the NAD⁺-dependent deacetylase SIRT2. This interaction is promoted by the NAD⁺ metabolite ADP-ribose (ADPR), which acts as a molecular bridge between the two enzymes. Within the SIRT2 active site, ADPR engages SerRS residue K414 located in a flexible catalytic-domain loop. Acetylation of K414 is dispensable for binding. Functionally, complex formation inhibits SIRT2 deacetylase activity by blocking substrate access, while SIRT2 association suppresses SerRS aminoacylation activity by preventing tRNA binding. Thus, SerRS and SIRT2 mutually regulate each other, with ADPR enhancing while tRNA attenuating their interaction. Oxidative stress promotes this interaction via a PARP1-dependent pathway, revealing an ADPR-responsive regulatory module that couples metabolic state to translational output. This regulatory module is likely conserved across vertebrates.

## Abstract (web summary)

Cells must coordinate metabolism, translation, and stress signaling to survive. Here, the authors reveal an ADP ribose-bridged SerRS-SIRT2 complex, uncovering crosstalk between translation and NAD⁺ metabolism under oxidative stress and defining a model of metabolite-driven regulation.

## Introduction

**Fig. 1:**
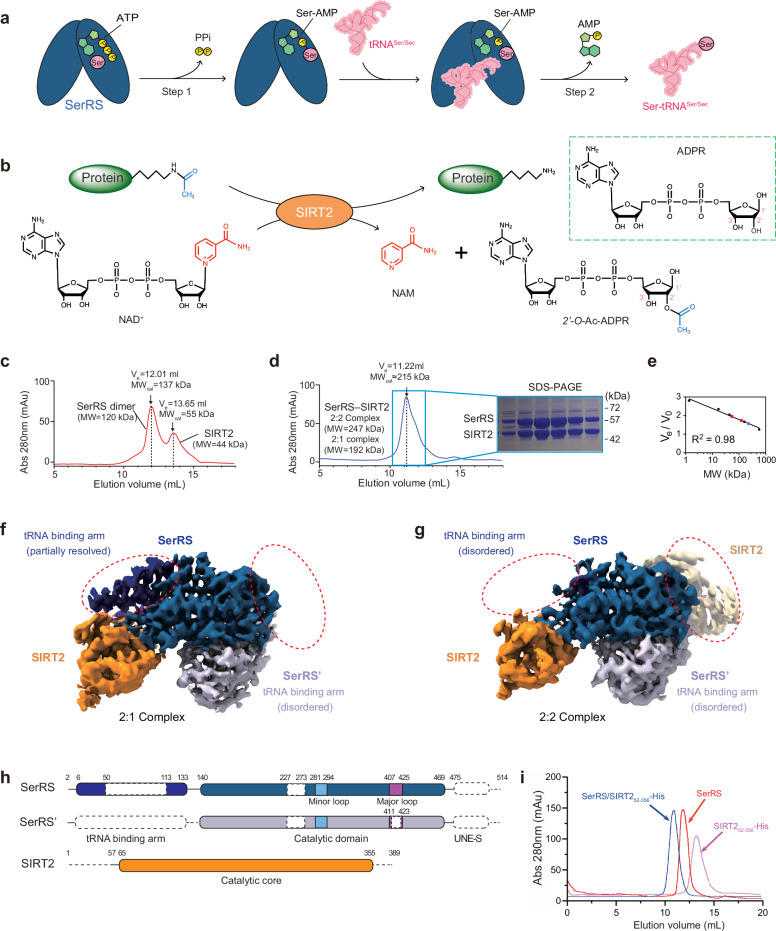
Cryo-EM structures of SerRS–SIRT2 complexes. **a** Two-step tRNA serinylation reaction catalyzed by SerRS. In step 1, ATP and serine bind to the catalytic pocket of SerRS, forming seryl-adenylate intermediate with the release of pyrophosphate (PPi); in step 2, the activated serine is transferred to SerRS-bound tRNA^Ser/Sec^, yielding Ser-tRNA^Ser^ or Ser-tRNA^Sec^ and AMP. **b** SIRT2-mediated NAD^+^-dependent protein deacetylation. The acetyl group from a modified lysine residue of the substrate protein is transferred to the ADP-ribose moiety of NAD^+^, generating 2’-*O*-Ac-ADP-ribose (OAADPR) and a deacetylated protein, with release of nicotinamide (NAM). The chemical structure of ADP-ribose (ADPR; green dashed box) is shown for comparison with OAADPR. **c** Size exclusion chromatography (SEC) of pre-mixed SerRS and SIRT2-His shows no evidence of interaction. Proteins were purified separately. Estimated molecular weight (MW_cal_) was calculated from the elution volume (V_e_) using a standard curve generated with molecular weight marker proteins. Theoretical molecular weight (MW) of SerRS and SIRT2 are also indicated. Source data are provided as a Source Data file. **d** SEC profile of the SerRS–SIRT2 complex formed by co-expression in *E. coli* (left) and SDS-PAGE analysis of the peak fractions (right). Both SerRS and SIRT2 are present in a single asymmetric peak with an estimated MW_cal_ ≈ 215 kDa, intermediate between the predicted 2:2 (247 kDa) and 2:1 (192 kDa) complexes. The experiment was independently repeated 2 times with similar results. Source data are provided as a Source Data file. **e** Standard calibration curve generated using molecular weight standards. Red dots indicate SerRS and SIRT2 peaks from (**c**); the blue dot indicates the SerRS–SIRT2 complex from (**d**). Source data are provided as a Source Data file. **f** Cryo-EM map of the SerRS–SIRT2 2:1 complex at an overall resolution of 3.38 Å. SIRT2 is shown in orange; SerRS subunits are in blue (SerRS) and light purple (SerRS’). Red dashed circles highlight regions differing from the 2:2 complex in (**g**). **g** Cryo-EM map of the SerRS–SIRT2 2:2 complex at an overall resolution of 3.73 Å. The second SIRT2 is shown in wheat. **h** Domain organization of SerRS and SIRT2. Disordered regions in the SerRS–SIRT2 2:1 complex are indicated by dashed lines. **i** SEC profiles showing that SerRS forms stable complex with the catalytic core of SIRT2 (G52-S356).

Aminoacyl-tRNA synthetases (aaRSs) are a family of evolutionarily conserved enzymes that catalyze the ligation of amino acid (aa) to their cognate tRNAs. Each proteinogenic aa requires a specific aaRS for the ligation reaction. Using serine (Ser) as an example, the aminoacylation reaction starts with the aa being selected by seryl-tRNA synthetase (SerRS, encoded by *SARS1*) and activated with energy released from ATP hydrolysis to form the reaction intermediate seryl-adenylate (Ser-AMP). Subsequently, the seryl group is transferred from Ser-AMP to the 3’-end of either serine tRNA (tRNA^Ser^) or selenocysteine tRNA (tRNA^Sec^), resulting in serylated-tRNA^Ser/Sec^ (Fig. 1a). Collectively, these aaRSs-catalyzed reactions create a pool of matched aa-tRNAs, essential for decoding mRNA while providing aa building blocks for ribosomal protein synthesis. In addition, many aaRSs, especially those in higher organisms, have been reported to play regulatory roles in a broad range of biological processes, including metabolism^1–3^.

Sirtuins are lysine deacetylases that utilize nicotinamide adenine dinucleotide (NAD^+^) as the co-substrate to remove posttranslational *N*ε-lysine acetylation from both histone and non-histone proteins (Fig. 1b), thereby regulating protein functions^4^. The level of NAD^+^ in the cell represents its energy potential. As the main oxidizing and reducing agents, NAD^+^ and its reduced form, NADH, facilitate oxidation-reduction (redox) reactions during metabolic processes, extracting ATP and biological building blocks (e.g., nucleotides, phospholipids, and amino acids) from nutrients^5–8^. By using NAD^+^ as the co-substrate, the functions of sirtuin family are intrinsically linked to cellular metabolism and energy homeostasis^9,10^.

A NAD^+^ molecule is composed of two moieties: ADP-ribose (ADPR) and nicotinamide (NAM) (Fig. 1b). When a *N*ε-lysine acetylated substrate protein is engaged by a NAD^+^-bound sirtuin, the acetyl group on lysine forms an intermediate with NAD^+^ and triggers the cleavage of the glycosidic bond between NAM and ADPR. Following the release of NAM from the enzyme, the acetyl-group is transferred from the Nε-lysine to the remaining ADPR moiety, producing *2*’-O-acetyl-ADP-ribose (OAADPR)^11^. Upon release from the sirtuin, OAADPR can be further converted to ADPR in the cell through an esterase or an acetyltransferase^12,13^. In addition to sirtuin-mediated reactions, a major source of cellular ADPR arises from PARP1 activation. During oxidative stress, DNA damage, or replication stress, PARP1 converts NAD⁺ into poly(ADP-ribose) (PAR), which is rapidly degraded by PARG to generate free ADPR monomers^14–16^.

Our previous work identified a direct interaction between human cytosolic SerRS and sirtuin 2 (SIRT2)^17^. Of the seven mammalian family members (SIRT1-7), SIRT2 is the only sirtuin primarily localized in the cytoplasm^10^. Among other targets, SIRT2 deacetylates α-tubulin lysine 40 in response to cellular NAD^+^ levels^18,19^. A small portion of SIRT2 also shuttles into the nucleus in a cell cycle dependent manner^20^, targeting histone H4 lysine 16^20^ and transcription factors^21,22^. Interestingly, a fraction of SerRS is also found in the nucleus due to the presence of a nuclear localization sequence (NLS) in its C-terminal UNE-S domain, which is specific to higher eukaryotes^23^. In the nucleus, SerRS competes with c-Myc for *VEGFA* promoter DNA binding^17^. Moreover, SerRS and SIRT2 act as partners to transcriptionally repress VEGFA expression, thereby playing an essential role in vascular development and angiogenesis regulation^17,23^. However, the details of how SerRS and SIRT2 interact with each other and how this affects their cytosolic functions remain unknown.

Here, we determined the cryo-EM structures of the SerRS–SIRT2 complex. Remarkably, an ADPR molecule was observed in the SIRT2 active site, bridging the two enzymes through their catalytic domains and mediating mutual functional inhibition. SerRS binding occludes the SIRT2 substrate groove, suppressing its deacetylase activity and leading to elevated acetylation of α-tubulin K40 and histone H4K16. Conversely, SIRT2 competes with tRNA for SerRS binding, thereby inhibiting SerRS aminoacylation activity and reducing global protein synthesis. In cells, oxidative stress enhances the SerRS–SIRT2 complex formation via PARP1-mediated ADPR production, and evolutionary analyses suggest that this regulatory mechanism is conserved among vertebrates. Together, these findings establish the SerRS–SIRT2 complex as a stress-sensitive hub that links metabolic and translational control, revealing a previously unrecognized layer of cellular homeostasis.

## Results

### SerRS and SIRT2 interact through their catalytic domain

To enable structural analysis of the SerRS–SIRT2 interaction, we purified recombinant human SerRS and SIRT2 through separate overexpression in *E. coli*. Analytical size-exclusion chromatography (SEC) of pre-mixed SerRS and SIRT2 proteins revealed no detectable interaction, as only two distinct peaks corresponding to dimeric SerRS and monomeric SIRT2 were observed (Fig. 1c). The estimated molecular weight (MW_cal_) of SerRS dimer (137 kDa) and SIRT2 (55 kDa), based on the elution volume (V_e_), were both greater than their theoretical MW values (i.e., 120 kDa and 44 kDa, respectively), presumably due to the flexible conformations of certain domains and regions within the proteins (see below). Considering the complex formation typically requires co-factors and/or co-folding processes in the cell, we co-expressed SerRS (tag-free) with SIRT2 (His-tagged) in *E. coli*. His-tag affinity purification followed by SEC yielded a stable SerRS–SIRT2 complex that eluted as a single peak (Fig. 1d). The presence of both SerRS and SIRT2 in the complex was confirmed by denaturing SDS-PAGE (Fig. 1d). Based on elution volume (V_e_), the peak of SerRS–SIRT2 complex had an estimated molecular weight of approximately 215 kDa (Fig. 1d, e), suggesting a mixture of 2:1 complex (SerRS dimer with 1 SIRT2) and 2:2 complex (SerRS dimer with 2 SIRT2).

The peak fraction was analyzed by single-particle cryo-EM to determine the structure of the SerRS–SIRT2 complex (Supplementary Table 1). Two-dimensional classification of picked particles revealed a mixture of 2:2 and 2:1 SerRS–SIRT2 assemblies (Supplementary Fig. 1a, b), consistent with the size-exclusion chromatography analysis (Fig. 1d). A total of 591,091 particles exhibiting high-resolution secondary structural features were selected for downstream processing (Supplementary Fig. 1b). Reference-free ab initio three-dimensional classification in CryoSPARC separated the particles into distinct 2:2 and 2:1 complexes. Particles corresponding to the 2:2 assembly were selected for non-uniform refinement with C2 symmetry, using the 2:2 ab initio reconstruction as the initial reference. This refinement yielded a three-dimensional reconstruction of the SerRS–SIRT2 2:2 complex at an overall resolution of 3.73 Å (Fig. 1g; Supplementary Fig. 1). Because both the 2:2 and 2:1 ab initio classes contain a common 2:1 region, particles from both classes were combined and subjected to non-uniform refinement with C1 symmetry, using the 2:1 ab initio map as the initial reference. This yielded a reconstruction of the SerRS–SIRT2 2:1 complex at an overall resolution of 3.82 Å. Subsequent local refinement with a mask focused on the 2:1 complex region (excluding density from the second SIRT2 molecule present in the 2:2 complex) further improved the map to 3.38 Å (Fig. 1f; Supplementary Fig. 1). The 2:2 complex contains a dimeric SerRS at the center, with each SerRS subunit engaging one SIRT2 molecule (Fig. 1g). A similar interaction is observed in the 2:1 complex; however, only one SerRS subunit is bound to SIRT2, leaving the other SerRS subunit unbound (Fig. 1f).

In both the 2:2 and 2:1 SerRS–SIRT2 complexes, the N- and C- terminal regions of SIRT2 (M1-E56 and S356-Q389, respectively) are unresolved (Fig. 1h), suggesting they are not involved in the SerRS interaction. Indeed, deletion of both N- and C-terminal regions of SIRT2 does not affect the SerRS–SIRT2 interaction (Fig. 1i). In the 2:1 complex, the N-terminal tRNA binding arm (V2-R133) was partially resolved in one SerRS subunit but remained almost completely disordered in the other (SerRS’) (Fig. 1f, h). Conversely, in the 2:2 complex, the tRNA binding arms of both SerRS subunits remained unresolved (Fig. 1g). Furthermore, the C-terminal UNE-S domains of SerRS (P475-D514) are unresolved in both complexes (Fig. 1h). Consistently, the tRNA binding arm and UNE-S domain of SerRS have been shown to be dispensable for SIRT2 interaction^17^. Therefore, the SerRS–SIRT2 interaction is mediated merely through the catalytic domains of SerRS (H140-K469) and SIRT2 (E65-Q355) (Fig.1h).

### ADPR triggers SerRS–SIRT2 interaction

**Fig. 2:**
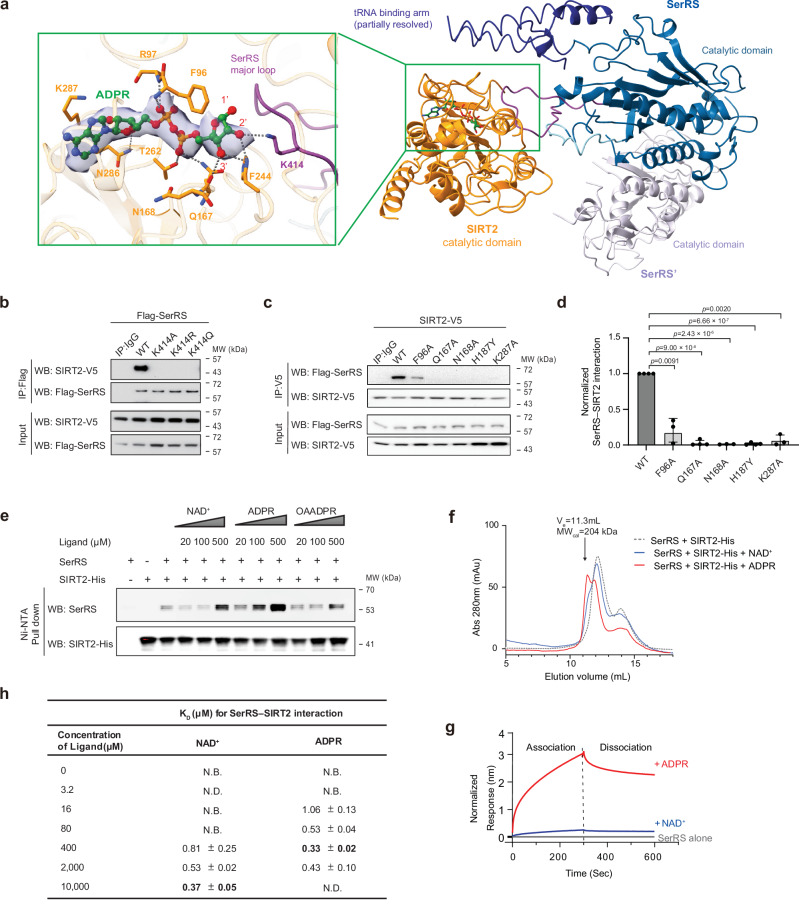
SerRS–SIRT2 interaction is induced by ADP-ribose. **a** Ribbon representation of the overall cryo-EM density-fitted model of SerRS–SIRT2 2:1 complex (right) and a zoomed-in view of the SIRT2 active site (left). SerRS and SIRT2 interact through their catalytic domains. The tRNA-binding arm of the SIRT2-bound SerRS is partially resolved, whereas it is almost completely disordered in the SIRT2-unbound SerRS’. Two SerRS loops involved in SIRT2 interaction are highlighted: the major loop (purple) and minor loop (cyan). The zoomed-in view shows an ADP-ribose (ADPR) molecule captured in the SIRT2 active site (green, stick-and-ball representation) and its interactions with the SIRT2 catalytic core, as well as with K414 from the SerRS major loop, where the 2’-OH in the terminal ribose of ADPR forms a hydrogen bond with the ε-amino group of K414. **b** Co-immunoprecipitation (Co-IP) assay showing the critical role of K414 in the SerRS–SIRT2 interaction. Mutations of K414 abolish the interaction. The experiment was independently repeated 2 times with similar results. Source data are provided as a Source Data file. **c** Representative Co-IP result showing that SIRT2 active site residues involved in ADPR binding are required for the SerRS–SIRT2 interaction. Source data are provided as a Source Data file. **d** Quantification of Co-IP result in (**c**). Data are presented as mean values ± SD. Welch’s two-sided t-test was performed with exact *p* values indicated in the figure. The number of independent experiments (n) is indicated by the individual data points shown in the figure. Source data are provided as a Source Data file. **e** Ni-NTA pull-down assay showing that ADPR promotes SerRS–SIRT2 interaction more effectively than NAD⁺, whereas OAADPR has minimal effect. The experiment was independently repeated 2 times with similar results. Source data are provided as a Source Data file. **f** Size exclusion chromatography (SEC) analysis of separately purified recombinant SerRS and SIRT2-His, in the absence or presence of NAD^+^ or ADPR. The elution volume (V_e_) and estimated molecular weight (MW_cal_) of the ADPR-induced SerRS–SIRT2 complex are indicated. Source data are provided as a Source Data file. **g** Biolayer interferometry analysis showing ADPR induces a stronger SerRS–SIRT2 interaction than NAD^+^ at same concentration (0.5 mM). SIRT2-His was immobilized on Anti-Penta-HIS sensor tips, and background binding of SerRS to SIRT2 in the absence of ligand was subtracted. Source data are provided as a Source Data file. **h** Dissociation constants (K_D_) of the SerRS–SIRT2 complex measured by mass photometry in the absence or presence of NAD⁺ or ADPR. Data are presented as mean values ± SD. *n* = 3 independent mass photometry measurements. Each measurement was performed using 10 nM SerRS dimer and 80 nM SIRT2. “N.D.” indicates not determined, and “N.B.” indicates no detectable binding. Source data are provided as a Source Data file.

Upon zooming into either 2:1 or 2:2 SerRS–SIRT2 complexes, we found extra densities in the active site of SIRT2 corresponding to ADPR and no density for NAM (Fig. 2a; Supplementary Fig. 2a, b). Consistently, the conformation of SIRT2 in complex with SerRS more closely resembles that of SIRT2 bound to ADPR (PDB ID: 5D7O) than any other known SIRT2 states, including the apo form, the NAD^+^-substrate complex, or complexes with intermediate mimics^24–27^ (Supplementary Fig. 2c, d). To confirm that the bound ligand is ADPR, we extracted the ligand from the co-purified SerRS–SIRT2 protein and subjected it to ESI triple quad mass spectrometry analysis. Only ADPR was detected, with no traces of NAD^+^ or OAADPR (Supplementary Fig. 3). In contrast, neither ADPR, OAADPR nor NAD^+^ was detected from the separately purified SIRT2 and SerRS proteins (Supplementary Fig. 3), confirming that ADPR is the co-factor that triggers the formation of a stable complex between SerRS and SIRT2.

Within the SIRT2 active site, ADPR directly interacts with SerRS through a hydrogen bond between 2’-OH of the ribose and the ε-amino group of K414 in SerRS (Fig. 2a). The K414 residue is positioned at the tip of a long loop (R407-M425), which protrudes from the catalytic domain of SerRS and inserts into the substrate-binding groove of SIRT2. Substitution of K414 with either alanine (SerRS^K414A^), arginine (SerRS^K414R^), or glutamine (SerRS^K414Q^) completely abolished the SerRS–SIRT2 interaction (Fig. 2b), confirming the essential role of K414 in mediating this interaction. The SIRT2-binding loop (R407–M425, major loop) adopts a well-resolved conformation in the SIRT2-bound SerRS subunit, whereas the majority of this region (G411–V423) is disordered in SerRS’, the unbound subunit in the 2:1 complex (Fig. 1h), indicating binding-induced stabilization. To further demonstrate that the SerRS–SIRT2 interaction is dependent on the presence of ADPR, we introduced single point substitutions of multiple SIRT2 active site residues that are not directly involved in SerRS binding but important in ADPR binding^28^ (Fig. 2a). All substitutions, including SIRT2^F96A^, SIRT2^Q167A^, SIRT2^N168A^, SIRT2^H187Y^, and SIRT2^K287A^, severely impacted or completely abolished the SerRS–SIRT2 interaction (Fig. 2c, d), confirming the interaction is ligand-dependent.

Interestingly, a pull-down assay demonstrated that NAD^+^ can also induce the SerRS–SIRT2 interaction in vitro, albeit with a weaker effect than ADPR (Fig. 2e). SEC and Bio-layer interferometry (BLI) further confirm a significantly stronger SerRS–SIRT2 interaction in the presence of ADPR compared to NAD^+^ at the same concentration (Fig. 2f, g). Mass photometry^29,30^ was used to determine the binding affinity between SerRS and SIRT2 in the presence of NAD⁺ and ADPR (Fig. 2h, Supplementary Fig. 4). In the absence of ligand, no measurable complex formation was detected. Increasing concentrations of NAD⁺ progressively strengthened the interaction, with the apparent dissociation constant (K_D_) decreasing from 0.81 ± 0.25 µM at 400 µM to 0.37 ± 0.05 µM at 10 mM. ADPR exhibited a similar effect at lower concentrations, reducing the K_D_ from 1.06 ± 0.13 µM at 16 µM to 0.33 ± 0.02 µM at 400 µM. These results indicate that both metabolites promote SerRS–SIRT2 complex formation, with ADPR supporting a more stable SerRS–SIRT2 interaction at comparable concentrations.

### SerRS suppresses SIRT2 deacetylation activity

The insertion of SerRS K414 into the active site of SIRT2 raised the possibility that K414 could be acetylated and serves as a SIRT2 substrate. Accordingly, the structure may capture a deacetylated K414 along with the OAADPR product prior to release from the enzyme. Although the abolished interaction between the acetylation-mimetic mutant SerRS^K414Q^ and SIRT2 (Fig. 2b) and the absence of OAADPR in the co-purified SerRS–SIRT2 complex (Supplementary Fig. 3) argue against this model, we nonetheless evaluated this possibility further.

We subjected both the recombinant SerRS protein alone and the co-purified SerRS–SIRT2 complex to trypsin digestion followed by mass spectrometry analysis. In both cases, a peptide containing the K414 site (^410^YGQTK^414^) was successfully detected (Supplementary Fig. 5a-c); however, no acetylation of this peptide was observed (Supplementary Fig. 5c). In contrast, more than a dozen lysine acetylation sites were identified in SerRS alone and/or in the SerRS–SIRT2 complex (Supplementary Fig. 5d), including the previously identified K323 site from mammalian cells^31^ (Supplementary Fig. 5e). Given that SerRS is acetylated at various sites, we also conducted a deacetylation reaction to assess if SerRS can act as a SIRT2 substrate. An Ac-K40-containing α-tubulin peptide was used as a positive control. In the presence of NAD^+^, SIRT2 efficiently deacetylated the α-tubulin peptide, as evidenced by the detection of the reaction products OAADPR and ADPR by ESI triple quad mass spectrometry (Supplementary Fig. 6). In contrast, no reaction products were detected when SerRS was used as the substrate (Supplementary Fig. 6). Finally, we examined whether OAADPR could promote SerRS–SIRT2 association. In contrast to NAD⁺ or ADPR, OAADPR showed minimal ability to enhance complex formation (Fig. 2e), likely because acetylation of the 2’-OH in OAADPR would disrupt the hydrogen bond observed between ADPR and SerRS K414 (Figs. 1b and 2a). Therefore, although the SerRS–SIRT2 interaction structurally resembles that between SIRT2 and its substrates, we obtain no evidence supporting SerRS as a substrate of SIRT2.

**Fig. 3:**
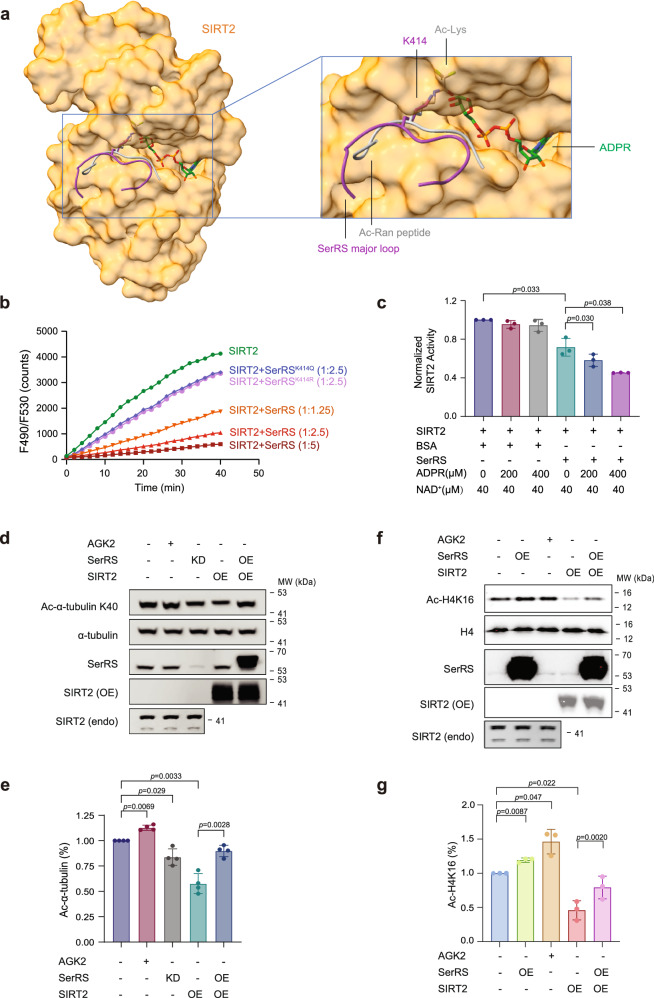
SerRS suppresses SIRT2 deacetylation activity. **a** Superposition of the SerRS-SIRT2 complex with SIRT2 structure bound to a substrate peptide (Ac-Ran peptide; PDB ID: 5FYQ, gray), showing insertion of the SerRS major loop (K414-containing loop, purple) into the substrate-binding groove of SIRT2. SIRT2 is shown in surface representation. **b** In vitro deacetylation assay demonstrating that SerRS, but not SerRS K414 mutants, inhibits SIRT2**-**His activity in a dose-dependent manner. SIRT2 activity was measured by fluorescence intensity (excitation: 490 nm; emission: 530 nm) from the cleaved substrate peptide. Data are presented as mean values of technical duplicates from 1 independent experiment. Source data are provided as a Source Data file. **c** The inhibitory effect of SerRS on SIRT2 deacetylation is enhanced by ADPR in a concentration-dependent manner, as assessed by pre-incubating SerRS and SIRT2 with ADPR prior to substrate addition. SIRT2 activity was measured as in (**b**) and quantified based on the initial reaction rate. BSA was used as a non-specific control. Data are presented as mean values ± SD. Exact *p* values are indicated in the figure. Two-sided paired *t*-test, *n* = 3 independent experiments. Source data are provided as a Source Data file. d Representative western blot showing α-tubulin K40 acetylation levels in HEK293AD cells, evaluating the impact of SerRS knockdown (KD) and overexpression (OE) on SIRT2 activity. A SIRT2 specific inhibitor AGK2 (5 μM) was used as a control. Source data are provided as a Source Data file. **e** Quantification of α-tubulin K40 acetylation levels from (**d**). Data are presented as mean values ± SD. Welch’s two-sided *t*-test was performed. Exact *p* values are indicated in the figure. *n* = 4 independent experiments. Source data are provided as a Source Data file. **f** Representative western blot showing H4K16 acetylation levels in HEK293AD cells, evaluating the effect of SerRS overexpression (OE) on SIRT2 activity. AGK2 (5 μM) was used as a control. Source data are provided as a Source Data file. **g** Quantification of H4K16 acetylation levels from (**f**). Data are presented as mean values ± SD. Exact *p* values are indicated in the figure. Two-sided paired *t*-test, *n* = 3 independent experiments. Source data are provided as a Source Data file.

Because the SIRT2-binding loop of SerRS occludes the substrate-binding groove of SIRT2 (Figs. 2a, 3a), we hypothesized that SerRS binding inhibits rather than enhances SIRT2 deacetylase activity as reported previously^17^. First, we performed an in vitro deacetylation assay using the Ac-K40-containing α-tubulin peptide as the substrate in the absence of added ADPR. Considering that NAD⁺ can promote SerRS–SIRT2 interaction (Fig. 2e, h), which would compete with SIRT2 substrates for binding to SIRT2 (Fig. 3a), and that ADPR is generated during the deacetylation reaction (Supplementary Fig. 6), both factors are likely to contribute to the inhibitory effect of SerRS. Indeed, SerRS inhibited the deacetylase activity of SIRT2 in a concentration-dependent manner (Fig. 3b). In contrast, SerRS mutants unable to bind SIRT2 (K414Q and K414R) showed markedly reduced inhibitory effects (Fig. 3b), demonstrating that the inhibition is specifically mediated by the SerRS–SIRT2 interaction.

Next, we tested the effect of exogenous ADPR. Consistent with previous reports that ADPR is a weak sirtuin inhibitor^32^, the addition of ADPR at 5–10× the NAD⁺ concentration had no effect on SIRT2 activity in the absence of SerRS (Fig. 3c). However, in the presence of SerRS, ADPR enhanced SerRS-mediated inhibition of SIRT2 in a concentration-dependent manner (Fig. 3c), reflecting ADPR-induced stabilization of the SerRS–SIRT2 complex.

To determine whether SerRS regulates SIRT2 activity in cells, we monitored the acetylation of α-tubulin K40 and histone H4K16 in HEK293AD cells under varying SerRS and SIRT2 expression conditions. SerRS knockdown reduced α-tubulin K40 acetylation, consistent with increased SIRT2 deacetylase activity and opposite to the effect of the SIRT2 inhibitor AGK2 (Fig. 3d, e). Conversely, SerRS overexpression increased global H4K16 acetylation, phenocopying AGK2 treatment (Fig. 3f, g). Moreover, co-overexpression of SerRS markedly attenuated the SIRT2-mediated deacetylation of both α-tubulin K40 and H4K16 induced by SIRT2 overexpression alone (Fig. 3d–g). Consistent results were observed with HEK293 cell lysates, where SerRS inhibited SIRT2-dependent deacetylation of α-tubulin K40 (Supplementary Fig. 7). Together, these findings demonstrate that SerRS inhibits SIRT2 deacetylase activity in cells.

### SIRT2 inhibits SerRS **tRNA aminoacylation** activity and global translation

**Fig. 4:**
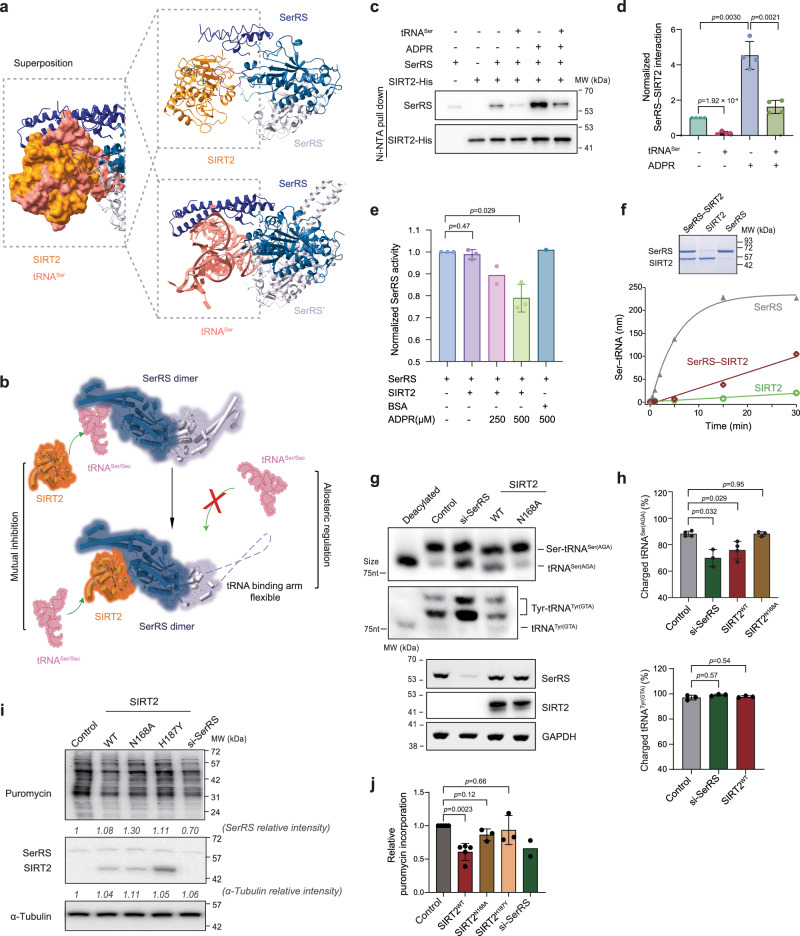
SIRT2 inhibits SerRS tRNA aminoacylation activity and global translation. **a** Superposition of the SerRS–SIRT2 2:1 cryo-EM complex with the SerRS–tRNA^Ser^ crystal structure (PDB ID: 8P7B), revealing overlap between SIRT2 (orange) and tRNA^Ser^ (salmon) binding sites on SerRS. **b** Schematic illustration of mutual inhibition and allosteric regulation between SIRT2 and tRNA^Ser/Sec^ for interaction with SerRS. **c** Ni-NTA pull-down assay showing that the SerRS–SIRT2 interaction is strongly inhibited by tRNA^Ser^ (5 μM, 1:1 mixture of in vitro-transcribed tRNA^Ser(GCT)^ and tRNA^Ser(CGA)^). 250 μM ADPR was used to induce a stable SerRS–SIRT2 interaction. Source data are provided as a Source Data file. **d** Quantification of the pull-down assay from (**c**). Data are presented as mean values ± SD. Welch’s two-sided *t*-test was performed. Exact *p* values are indicated in the figure. *n* = 4 independent experiments. Source data are provided as a Source Data file. **e** In vitro aminoacylation assays showing that the inhibitory effect of SIRT2 on SerRS aminoacylation is induced by ADPR in a concentration-dependent manner. SerRS activity was quantified based on the initial reaction rate. BSA was used as a non-specific control. Data are presented as mean values ± SD. Welch’s two-sided *t*-test was performed with exact *p* values indicated in the figure. The number of independent experiments (n) is indicated by the individual data points shown in the figure. Groups with *n* < 3 are presented as mean values only and excluded from statistical analysis. Source data are provided as a Source Data file. **f** In vitro aminoacylation assays showing that SIRT2 inhibits SerRS aminoacylation activity. Recombinant SerRS, SIRT2 and SerRS–SIRT2 complex used in the assay were analyzed by SDS-PAGE to confirm equal amounts of enzymes were used (top). Data are presented as mean value of duplicate technical replicates from 1 independent experiment. Source data are provided as a Source Data file. **g** Representative acidic gel northern blot analysis showing that SIRT2 specifically inhibits SerRS tRNA charging activity in HEK293AD cells. SerRS knockdown and SIRT2 overexpression were confirmed by western blot. Source data are provided as a Source Data file. **h** Quantification of charged tRNA^Ser(AGA)^ and tRNA^Tyr(GTA)^ levels from (**g**). Data are presented as mean values ± SD. Welch’s two-sided *t*-test was performed with exact *p* values indicated in the figure. The number of independent experiments (n) is indicated by the individual data points shown in the figure. Source data are provided as a Source Data file. **i** Puromycin incorporation assay showing that either SerRS knockdown (65.8% remaining SerRS) or SIRT2 overexpression reduces global protein synthesis in HEK293AD cells, while overexpression of SIRT2 mutants defective in SerRS interaction does not. Source data are provided as a Source Data file. **j** Quantification of puromycin incorporation from (**i**). Data are presented as mean values ± SD. Welch’s two-sided *t*-test was performed with exact *p* values indicated in the figure. The number of independent experiments (n) is indicated by the individual data points shown in the figure. Groups with *n* < 3 are presented as mean values only and excluded from statistical analysis. Source data are provided as a Source Data file.

Comparing the SerRS–SIRT2 and SerRS–tRNA^Ser^ (PDB ID: 8P7B) interactions reveals an overlap between the SIRT2 and tRNA binding sites on SerRS (Fig. 4a), suggesting a mutual exclusion of SIRT2 and tRNA binding. In the 2:1 complex, the tRNA binding arm is partially resolved in the SIRT2-bound SerRS subunit but completely disordered in the SIRT2-free subunit (Figs. 1f, 2a); in the 2:2 complex with two SIRT2 molecules bound, the tRNA-binding arms of both SerRS subunits are entirely disordered (Fig. 1g, Supplementary Fig. 2a), indicating that SIRT2 binding allosterically increases the flexibility of the tRNA-binding arm across the SerRS dimer. Hydrogen deuterium exchange mass spectrometry analysis (HDX-MS, Supplementary Table 2) confirmed increased flexibility of the tRNA-binding arm upon SIRT2 binding. Compared with the separately purified proteins, deuterium incorporation in the SerRS–SIRT2 complex decreased within the catalytic domains of both SIRT2 and SerRS (Supplementary Fig. 8), consistent with binding-induced conformational stabilization. In contrast, the tRNA-binding arm overall showed increased deuterium incorporation (Supplementary Fig. 8a, c), in agreement with the enhanced flexibility inferred from the structures. Together, these results indicate that SIRT2 binding directly blocks tRNA binding to one SerRS subunit and allosterically disrupts tRNA binding to the other subunit (SerRS’) (Fig. 4a, b).

To investigate reciprocal competition between SIRT2 and tRNA, we first performed an in vitro pull-down assay using His-tagged SIRT2. A weak SerRS–SIRT2 interaction was detected in the absence of ADPR, whereas ADPR markedly enhanced complex formation (Fig. 4c, d). Importantly, tRNA^Ser^ inhibited the SerRS–SIRT2 interaction both in the presence and absence of ADPR (Fig. 4c, d). We next assessed the functional impact of SIRT2 on SerRS activity using an in vitro tRNA aminoacylation assay. Addition of SIRT2 or ADPR alone had no effect on SerRS-mediated tRNA aminoacylation (Fig. 4e). However, combined addition of SIRT2 and ADPR inhibited SerRS activity in an ADPR concentration–dependent manner, suggesting that the inhibition is driven by ADPR-enhanced SerRS–SIRT2 interaction (Fig. 4e). Consistently, the co-purified SerRS–SIRT2 complex exhibited reduced tRNA aminoacylation activity compared with SerRS alone (Fig. 4f).

To further assess the inhibitory effect of SIRT2 on SerRS activity in cells, we performed acidic gel northern blot to measure tRNA aminoacylation in HEK293AD cells. Overexpression of SIRT2 reduced the charging level of tRNA^Ser^ from 88% to 76%, comparable to the effect of SerRS knockdown (70% charged) (Fig. 4g, h). In contrast, overexpression of SIRT2^N168A^ mutant, which is unable to interact with SerRS, had no significant effect (Fig. 4g, h). Notably, the inhibitory effect of SIRT2 was specific to tRNA^Ser^, as the charging level of tRNA^Tyr^ was unchanged upon SIRT2 overexpression (Fig. 4g, h); two charged tRNA^Tyr^ species were detected (Fig. 4g), likely reflecting differences in tRNA modification status^33^. Furthermore, SUnSET assays^34^, which monitor global translation via puromycin incorporation, showed that overexpression of WT SIRT2—but not the N168A and H187Y mutants—suppressed global protein synthesis, consistent with the effect of SerRS knockdown (Fig. 4i, j). Collectively, these results suggest that SerRS–SIRT2 interaction suppresses cognate tRNA aminoacylation and inhibits protein synthesis.

### Dual-loop recognition defines SerRS–SIRT2 specificity

**Fig. 5:**
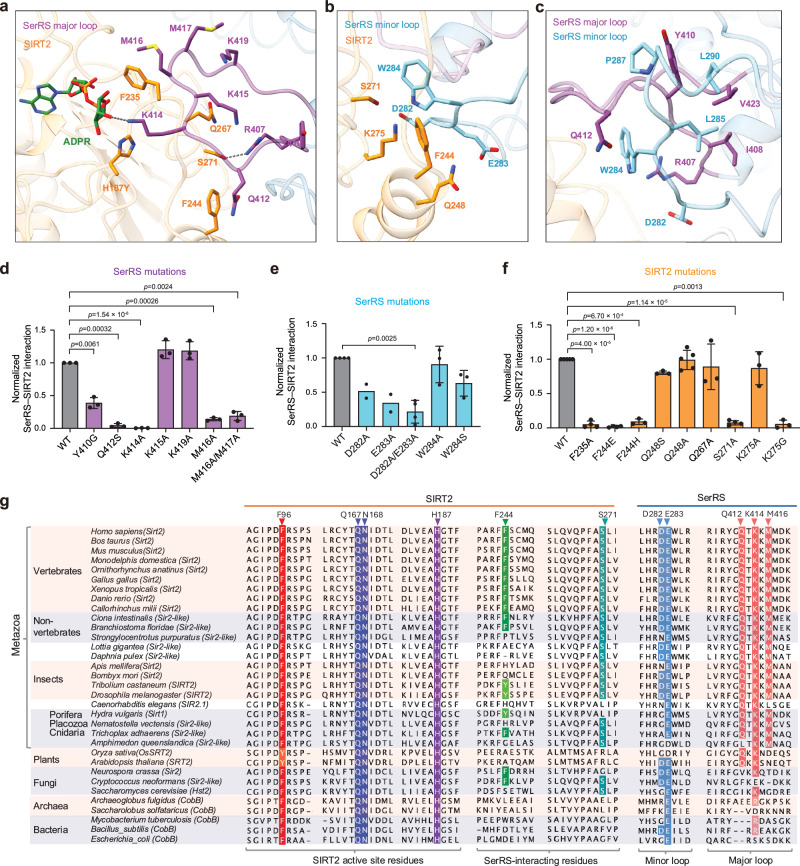
SerRS–SIRT2 interaction, mutagenesis and evolutionary conservation. **a**,**b** SerRS–SIRT2 interface at the major loop (**a**) and minor loop (**b**) of SerRS. Interface residues are shown in sticks. Hydrogen bonds are indicated in gray dashed lines. **c** Structural interactions between the major loop and minor loop of SerRS. **d–f** Quantification of mutagenesis and Co-IP assays shown in Supplementary Fig. 9. Mutations Y410G, Q412S, K414A and M416A in the SerRS major loop (**d**) and D282A and E283A in the SerRS minor loop (**e**) either abolish or weaken SIRT2 binding; mutations F235A, F244E, F244H, S271A, and K275G in SIRT2 (**f**) either abolish or weaken the SerRS binding. Data are presented as mean values ± SD. Welch’s two-sided t-test was performed; exact *p* values are indicated in the figure. The number of independent experiments (n) is indicated by the individual data points shown in the figure. Groups with *n* < 3 are presented as mean values only and excluded from statistical analysis. Source data are provided as a Source Data file. **g** Multiple sequence alignment of human SIRT2 and SerRS with orthologs from representative bacteria, archaea, fungi, plants, early-branching metazoans (including cnidaria, placozoa, porifera, and insects), non-vertebrates, and vertebrates. In the SIRT2 active site, residues involved in ligand binding (F96, Q167, N168, and H187) are highlighted and show a high degree of conservation from bacteria to vertebrates. In addition, SIRT2 residues mediating direct SerRS binding (F244 and S271) are highlighted; notably, F244 is conserved across the vertebrate lineage. From the SerRS perspective, residues in the minor loop (D282 and E283) and major loop (Q412, K414, and M416) that mediate SIRT2 binding are also highlighted and are largely conserved from early metazoans to vertebrates.

In addition to the ADPR-mediated interaction, SerRS engages with SIRT2 through two loops within its catalytic domain (Fig. 2a). The K414-containing SIRT2-binding (major) loop includes several residues that directly contact the SIRT2 substrate-binding groove (Figs. 3a, 5a). Single mutations at Q412 (SerRS^Q412S^) and M416 (SerRS^M416A^), but not at K415 (SerRS^K415A^) or K419 (SerRS^K419A^), abolished the SerRS–SIRT2 interaction (Fig. 5d; Supplementary Fig. 9a, b). A second loop (R281-Y294; the minor loop) also contributes to SIRT2 binding (Fig. 5b), in part by stabilizing the major SIRT2-binding loop (Fig. 5c). Mutations in the minor loop (e.g., D282A and E283A) weaken the SerRS–SIRT2 interaction, although their effects were generally less pronounced than those of mutations in the major loop (Fig. 5e; Supplementary Fig. 9c, d).

On the SIRT2 side, several residues -including F235, F244, and S271- are crucial for interaction with SerRS (Fig. 5a, b, f; Supplementary Fig. 9e–g). SIRT2 F235 forms a cation-π interaction with SerRS K414 and a hydrophobic interaction with SerRS M416; accordingly, the SIRT2^F235A^ mutation disrupted SerRS binding (Fig. 5f, Supplementary Fig. 9f). SIRT2 F244 engages an amide–π stacking with SerRS Q412 (Fig. 5a), and substitutions at this position (SIRT2^F244H^ and SIRT2^F244E^) abolished SerRS binding (Fig. 5f; Supplementary Fig. 9e–g), explaining the specificity of SerRS for SIRT2 over SIRT1^17^, which contains a histidine at the equivalent position (Supplementary Fig. 10). In addition, SIRT2 S271 forms a hydrogen bond with SerRS R407 (Fig. 5a), and the SIRT2^S271A^ substitution also abolished SerRS binding (Fig. 5f; Supplementary Fig. 9g); S271 is conserved in SIRT2 and SIRT3 but absent from other sirtuin family members (Supplementary Fig. 10). Importantly, SIRT2^F244H^ retained partial deacetylase activity, whereas SIRT2^S271A^ maintained full catalytic activity (Supplementary Fig. 9h, i). In contrast, SIRT2 K275G mutation abolished both SerRS binding and SIRT2 enzymatic activity (Fig. 5f, Supplementary Fig. 9g-i). The F244H and S271A mutants thus provide valuable tools to dissect the biological significance of the SerRS–SIRT2 interaction in future studies.

### SerRS–SIRT2 interaction is likely conserved in vertebrates

In contrast to SerRS, which is essential across all domains of life, the evolutionary history of the sirtuin family is more dynamic. Although SIRT2 is broadly conserved among eukaryotes, it is absent in certain lineages, including land plants and some species such as *C. elegans*^35^. Not all SIRT2 orthologs retain a phenylalanine at the position corresponding to F244 in human SIRT2 (Fig. 5g), a residue that is critical for SerRS binding (Fig. 5f; Supplementary Fig. 9e–g). Phylogenetic analysis indicates that F244 emerged during the transition from non-vertebrates to vertebrates and has remained conserved across all vertebrate SIRT2 orthologs (Fig. 5g). In contrast, S271 of SIRT2 and residues within both the major and minor SIRT2-binding loops of SerRS are highly conserved across the animal kingdom (Fig. 5g). Together, this analysis suggests that SerRS–SIRT2 interaction is conserved among vertebrates. Strikingly, the SIRT2-binding loops also mediate SerRS interaction with tRNA^Ser^ and METTL6 (PDB ID: 8P7B), facilitating recognition of tRNA^Ser^ for m³C32 modification^36^ (Supplementary Fig. 11), indicating that SIRT2 binding may inhibit not only SerRS-mediated tRNA aminoacylation but also METTL6-dependent tRNA^Ser^ methylation.

### Oxidative stress induced SerRS–SIRT2 interaction inhibits translation

**Fig. 6:**
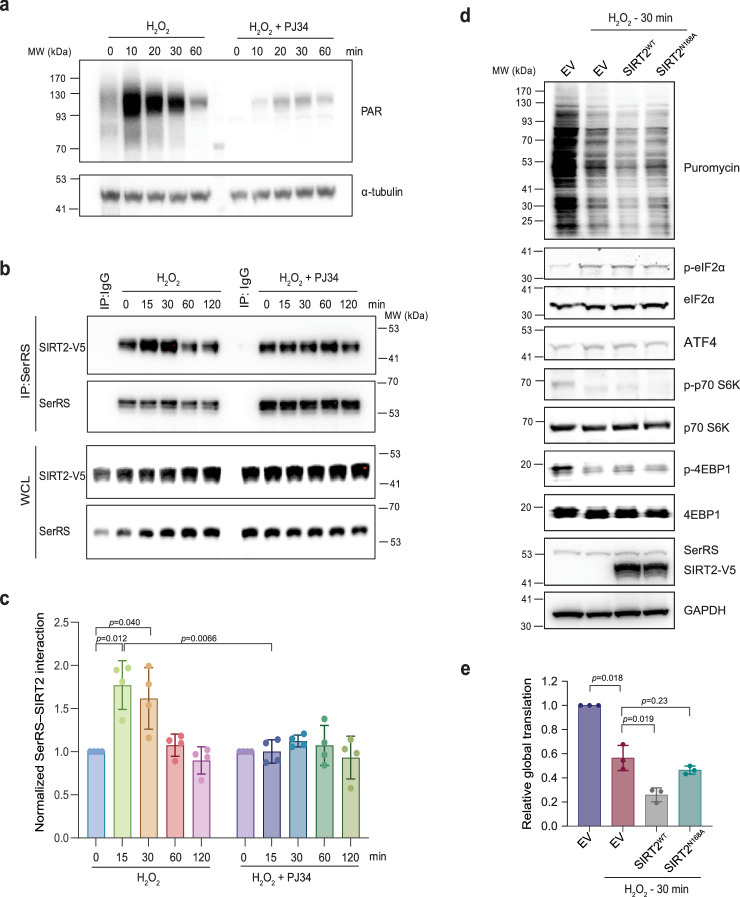
Oxidative stress induced SerRS–SIRT2 interaction inhibits translation. **a** Western blot showing rapid PARP1 activation and accumulation of poly(ADP-ribose) (PAR) within minutes following treatment with H₂O₂ (0.5 mM). PAR polymers are subsequently degraded, dynamically modulating the cellular ADPR pool. Pretreatment with the PARP1 inhibitor PJ34 (10 μM) suppresses PARP1 activation and PAR production. The experiment was independently repeated 1 time with similar results. Source data are provided as a Source Data file. **b** Representative Co-immunoprecipitation (Co-IP) showing that the SerRS–SIRT2 association increased in parallel with PAR degradation following treatment with 0.5 mM H₂O₂. Co-IP was performed in SIRT2-V5–overexpressing HEK293AD cells by immunoprecipitation of SerRS using a home-made anti-SerRS mouse antibody (18A8). Pretreatment with PJ34 (10 μM) attenuated the H₂O₂-induced enhancement of the SerRS–SIRT2 interaction. Source data are provided as a Source Data file. **c** Quantification of SerRS–SIRT2 interactions by Co-IP in (**b**). Data are presented as mean values ± SD. Welch’s two-sided *t*-test was performed. Exact *p* values are indicated in the figure. *n* = 4 independent experiments. Source data are provided as a Source Data file. **d** Puromycin incorporation assay showing that under 30 min of 0.5 mM H₂O₂ treatment, SIRT2 overexpression further reduces nascent global protein synthesis in HEK293AD cells, whereas overexpression of SIRT2 mutant (N168A) defective in SerRS interaction does not. This SIRT2-mediated translational inhibition occurs independently of ISR activation (assessed by p-eIF2α and ATF4) and mTOR inhibition (assessed by p-p70S6K and p-4EBP1). Source data are provided as a Source Data file. **e** Quantification of nascent global protein synthesis in (**d**). Data are presented as mean values ± SD. Welch’s two-sided *t*-test was performed; exact *p* values are indicated in the figure. *n* = 3 independent experiments. Source data are provided as a Source Data file.

Given that ADPR promotes SerRS–SIRT2 interaction in vitro (Fig. 2e–h), we asked whether cellular conditions that elevate ADPR levels can trigger the interaction in cells. Oxidative stress induced by H₂O₂ rapidly activates PARP1 and increases poly(ADP-ribose) (PAR) accumulation in HEK293AD cells, an effect that is blocked by the PARP inhibitor PJ34 (Fig. 6a). PAR would be subsequently degraded by PARG and hydrolases such as ARH3, leading to a transient elevation of cellular ADPR levels^37^. Consistent with this model, SerRS–SIRT2 association increased transiently following H₂O₂ treatment, peaking at 15-30 min and returning to baseline by 60 min (Fig. 6b, c). This stress-induced interaction was abolished by PJ34, confirming that PARP1-dependent ADPR production drives oxidative stress-induced formation of the SerRS–SIRT2 complex (Fig. 6b, c).

Under oxidative stress induced by H₂O₂, ISR activation and mTOR inhibition converge to suppress translation initiation and global protein synthesis. Given that SerRS charging activity is suppressed by SIRT2, we further investigated whether the increased SerRS–SIRT2 interaction under oxidative stress leads to additional translational inhibition. To test this, we treated cells overexpressing SIRT2 (wild-type or a mutant deficient in SerRS binding) with H₂O₂ for 30 min. The oxidative stress treatment per se suppressed translation, as expected from ISR activation (p-eIF2α) and mTOR inhibition (p-p70 S6K and p-4EBP1) (Fig. 6d). The ATF4 protein level was barely enhanced due to the short duration of the treatment^38^. Importantly, overexpression of SIRT2 further reduced nascent protein synthesis whereas overexpressing a SIRT2 mutant (N168A) that is deficient in binding to SerRS had a minimal impact, suggesting that the translation inhibition effect is related to the SerRS–SIRT2 interaction (Fig. 6d, e). Notably, SIRT2 overexpression did not further affect canonical ISR activity or mTOR activity, suggesting that the additional translational suppression operates independently of the initiation-targeting pathways (Fig. 6d). Rather, we propose that reduced charged tRNA^Ser^ availability, resulting from SIRT2-mediated SerRS inhibition, decreases translation elongation rates at serine codons, further potentiating translational inhibition under oxidative stress.

## Discussion

In this work, we determined the cryo-EM structure of the SerRS–SIRT2 complex stabilized by the NAD⁺ metabolite ADPR. Structural, in vitro biochemical, and cell-based analyses demonstrate that the SerRS–SIRT2 interaction results in reciprocal inhibition of both enzymatic activities (Figs. 3 and 4). However, our previous study reported a cooperative role for SerRS and SIRT2 in regulating *VEGFA* transcription^17^. In particular, we observed that SerRS modestly enhanced SIRT2 activity in vitro, which appears inconsistent with the inhibitory effects described here. One likely explanation for this discrepancy lies in differences in experimental reagents. Whereas the present study used purified, untagged SIRT2, our earlier work relied on a commercially sourced GST-tagged SIRT2 protein, which may have interfered with SerRS binding and/or SIRT2 enzymatic activity, thereby masking the inhibitory interaction revealed here. Because these commercially sourced reagents date back over a decade, direct testing is difficult; therefore, the precise cause of the discrepancy remains to be determined.

Consistent with this interpretation, we show that SerRS overexpression increases global H4K16 acetylation (Fig. 3f, g), whereas our prior work reported increased H4K16 acetylation at the *VEGFA* promoter following SerRS knockdown^17^. Together, these findings suggest that SerRS differentially regulates global versus locus-specific H4K16 acetylation. We propose a model in which SerRS restrains SIRT2 activity through complex formation while, at specific genomic loci, SerRS–DNA interactions may weaken this complex and locally release SIRT2 to drive targeted H4K16 deacetylation. Consistent with this model, pull-down assays demonstrate direct competition between SIRT2 and the VEGFA promoter DNA for SerRS binding, while EMSA analysis shows diminished capacity of the SerRS–SIRT2 complex for DNA binding (Supplementary Fig. 12). This model reconciles the global inhibitory effects observed here with the promoter-specific activation reported previously and provides a testable hypothesis for future investigation on the regulation of the SerRS–SIRT2 interaction in the nucleus.

We identify ADPR as a molecular bridge that stabilizes the SerRS–SIRT2 interaction. Given that ADPR accumulates under diverse stress conditions^14–16^, our findings suggest that this interaction is dynamically regulated by cellular stress. Consistent with this idea, we show that oxidative stress enhances SerRS–SIRT2 complex formation through PARP1-mediated ADPR production (Fig. 6), directly linking stress-induced NAD⁺ metabolism to regulation of this complex. It is worth noting that ADPR and OAADPR have previously been implicated as signaling molecules downstream of NAD⁺ consumption. Under stress, major NAD⁺-dependent enzymes such as sirtuins and PARPs are activated, producing NAM together with OAADPR or ADPR during catalysis or following enzymatic hydrolysis^39^. Unlike NAM, which is efficiently recycled into NAD⁺ through the salvage pathway—the predominant route for NAD⁺ biosynthesis^5^—OAADPR and ADPR do not contribute to NAD⁺ regeneration and instead function as second messengers. In mammalian cells, accumulation of free ADPR during oxidative or nitrosative stress activates TRPM2 cation channels, leading to Ca²⁺ influx^40–42^. In yeast, elevated OAADPR/ADPR levels have been linked to metabolic rewiring that reduces endogenous reactive oxygen species and redirects glucose flux from glycolysis toward the pentose phosphate pathway to support NADPH production^43^.

Against this backdrop, our finding that ADPR—but not OAADPR—potently induces SerRS–SIRT2 complex formation (Fig. 2e–h) reveals a previously unrecognized level of specificity in ADPR signaling. This selectivity aligns with cellular metabolism, as PARP activation generates ADPR through PAR synthesis and degradation, whereas OAADPR is produced by sirtuin-catalyzed deacetylation reactions. Together, these observations support a model in which PARP activation promotes SerRS–SIRT2 complex formation via ADPR accumulation, thereby linking PARP activity to SIRT2 inhibition within a shared, stress-responsive NAD⁺-dependent pathway.

Using mass photometry, a solution-based label-free method, we found that SerRS–SIRT2 association was weak in the absence of ligand (estimated K_D_ > 10 µM). NAD⁺ enhanced complex formation only at high concentrations (> 400 µM), whereas ADPR effectively promoted SerRS–SIRT2 complex formation, supporting a low- to sub-micromolar affinity interaction across 16-400 µM ADPR (Fig. 2h). Reported intracellular NAD⁺ concentrations vary widely depending on measurement methods and subcellular compartments. Total cellular NAD⁺ is typically ~200–500 µM^44^, whereas free cytosolic and nuclear NAD⁺ concentrations are generally in the tens to low hundreds of micromolar range^45^. Consequently, the extent to which physiological NAD⁺ levels modulate the SerRS–SIRT2 interaction remains unclear. By contrast, reported intracellular ADPR levels (~ 40–70 µM at basal level)^46^ fall within the range required to support SerRS–SIRT2 interaction and increase substantially upon PARP activation, positioning ADPR as a physiologically relevant regulator of SerRS–SIRT2 interaction.

**Fig. 7:**
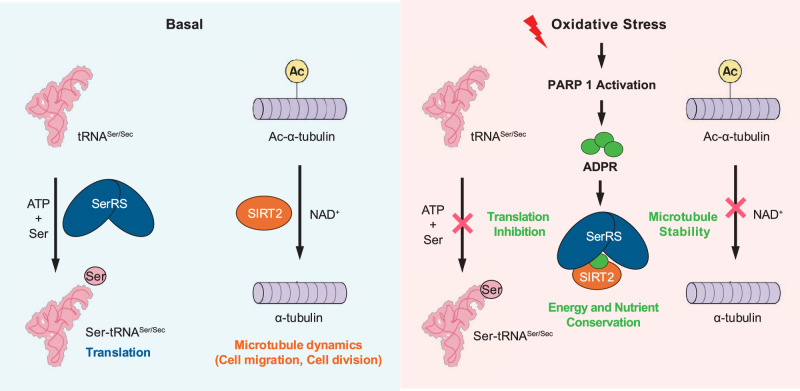
Model for SerRS–SIRT2 interaction as an ADPR-responsive stress sensor. Under basal conditions, abundant tRNA and low ADPR favor SerRS–tRNA engagement, supporting translation, while SIRT2 deacetylates α-tubulin to maintain microtubule dynamics. Under stress, increased ADPR and reduced tRNA promote SerRS–SIRT2 complex formation, leading to mutual inhibition of tRNA serylation and deacetylation, thereby conserving cellular energy and supporting stress adaptation.

We speculate that under basal conditions, when ADPR levels are low, the high abundance of cellular tRNA effectively shields SerRS from interaction with SIRT2. The binding affinity of cognate tRNA–aminoacyl-tRNA synthetase pairs is typically in the K_D_ range of 0.1–1 μM^47–49^, supporting preferential SerRS–tRNA engagement under these conditions. Consequently, SerRS primarily functions in its canonical role, charging tRNA in an ATP-dependent manner to sustain protein synthesis, while SIRT2 continues to utilize NAD⁺ to deacetylate α-tubulin at K40, thereby supporting microtubule dynamics associated with cell migration, division, and intracellular transport (Fig. 7).

During cellular stress, cytosolic tRNA levels decrease through cleavage by angiogenin^50^ and/or retrograde transport from the cytoplasm to the nucleus^51^. Concurrently, elevated ADPR levels favor the formation of a more stable SerRS–SIRT2 complex, resulting in reciprocal inhibition of both enzymatic activities. Formation of this complex is expected to reduce tRNA serylation and dampen global translation (Fig. 4e-j), thereby limiting ATP and amino acid consumption. In parallel, inhibition of SIRT2 activity could attenuate α-tubulin deacetylation, potentially promoting microtubule stabilization. Together, these effects suggest a model in which the SerRS–SIRT2 complex may act as a stress-responsive regulatory node that coordinates translational restraint, NAD⁺ utilization, and cytoskeletal organization to support cellular adaptation and resilience under adverse conditions (Fig. 7).

Reanalysis of a published SIRT2 immunoprecipitation–mass spectrometry dataset revealed enrichment of multiple aminoacyl-tRNA synthetases in the SIRT2 interactome (Supplementary Fig. 13)^52^. Notably, SerRS was the most abundant SIRT2 interactor, supporting a prominent SerRS–SIRT2 association. Among the remaining tRNA synthetases, GlyRS ranked as the top interactor. GlyRS has been independently shown to bind SIRT2—but not SIRT1—and to inhibit SIRT2 deacetylation activity^53^, although whether this interaction reciprocally regulates GlyRS or is modulated by ADPR or cellular stress remains unknown. Interactions between SIRT2 and additional tRNA synthetases, including TyrRS, LysRS, and HisRS, have also been reported^54^, raising the possibility that tRNA synthetases constitute a broader class of SIRT2 regulators. The prominent association of both SerRS and GlyRS with SIRT2 is intriguing, given that serine and glycine are biosynthetically linked and readily interconvertible. Beyond its role in protein synthesis, serine occupies a central position in cellular metabolism, supporting nucleotide and lipid biosynthesis as well as redox homeostasis through production of glutathione and NADPH^55^. These metabolic connections suggest that SerRS–SIRT2 interaction may couple translational control to broader metabolic and redox adaptation during stress.

Despite the apparent breadth of tRNA synthetase interactions with SIRT2, SerRS exhibits a high degree of selectivity for SIRT2 among sirtuin family members. Although SIRT1 and SIRT2 share substantial sequence similarity within their catalytic domains (68%; Supplementary Fig. 10), SerRS interacts with SIRT2 but not SIRT1^17^. Our structural analysis of the SerRS–SIRT2 complex, together with mutagenesis and functional studies, provides a mechanistic basis for this specificity. A key determinant of the interaction, F244, is uniquely conserved in SIRT2 but absent from other sirtuins. Another critical residue, S271, is conserved in SIRT2 and SIRT3 but absent from other sirtuins family members (Fig. 5f; Supplementary Fig. 10). Moreover, consistent with the context-dependent substrate recognition characteristic of sirtuins^56^, analysis of the substrate-mimetic loop of SerRS revealed that Q412 (− 2 position) and M416 (+ 2 position) are present exclusively in SIRT2 substrates. Mutation of either residue markedly weakened the SerRS–SIRT2 interaction (Fig. 5d), further supporting the molecular specificity of SerRS for SIRT2 over other sirtuin family members.

In summary, this study reveals an unexpected, ADPR-dependent regulatory axis linking SerRS and SIRT2, uncovered through structural analysis and integrated biochemical and cellular approaches. By positioning SerRS as a conditional modulator of SIRT2 activity, our findings expand the functional repertoire of aminoacyl-tRNA synthetases beyond translation and identify ADPR as a selective metabolic signal that couples NAD⁺-dependent stress responses to transcriptional, translational and cytoskeletal control.

## Methods

### Protein expression and purification

For protein expression, N-terminal His-SUMO-tagged or tag-free human SerRS construct was generated by subcloning human SerRS gene into a modified pET-28a vector with a SUMO gene inserted at the 5’ of the MSC site. C-terminal His-tagged constructs of human SerRS, SIRT2 and SIRT2_52-356_ (catalytic core region of G52-S356 aa) were subcloned into pET-20b (+) vector (Novagen, Darmstadt, Germany). Recombinant proteins were overexpressed or co-expressed (for the SerRS–SIRT2-His complex) in *E. coli* BL21(DE3) cells. For protein purification, the cells were lysed by M-110L Microfluidizer Processor (Microfluidics) in 25 mM HEPES-Na (pH 7.5), 500 mM NaCl, and 20 mM imidazole (binding buffer). Cell lysates were centrifuged at 95,000 × g for 40 min, and the soluble fractions were applied to a Ni-NTA column (QIAGEN). After washing the Ni-NTA resin with the binding buffer containing 10-40 mM imidazole, bound proteins were eluted with the same buffer containing 250 mM imidazole. The eluted proteins from Ni-NTA were further purified by either a HiTrap Q HP anion exchange column (GE Healthcare, Pittsburgh, PA, USA) for SIRT2-His and SIRT2_52-356_-His, or a HiTrap Heparin HP affinity column (GE Healthcare) for SerRS and SerRS–SIRT2-His complex, using a NaCl gradient from 50 to 500 mM. Final protein purification was achieved by size exclusion chromatography using a HiLoad 16/600 Superdex 200 pg column (GE Healthcare) in 25 mM HEPES-Na (pH 7.5) and 150 mM NaCl. Tag-free SerRS was generated by overnight incubation of the Ni-NTA resin-bound His-SUMO-SerRS protein with homemade Ulp1 enzyme to cleave the His-SUMO tag. The resulting tag-free SerRS was collected the following day in the flow through and further purified by heparin affinity and size exclusion chromatography.

### Sample preparation for cryo-EM

Purified SerRS–SIRT2 complex (0.5 mg/mL) was supplemented with 0.05% Lauryl Maltose Neopentyl Glycol (LMNG) prior to cryo-freezing. The sample (4 µL at 0.5 mg/mL) was applied onto 300 mesh R1.2/1.3 UltraAuFoil Holey Gold grids (Quantifoil) that has been plasma cleaned for 15 s using a Pelco glow discharge cleaning system (TED PELLA, INC.) with atmospheric gases at 15 mA. The sample (4 uL at 0.1 mg/mL) was also applied onto 300 mesh R1.2/1.3 UltraAuFoil Holey Gold grids (Quantifoil) that were coated with a single layer of graphene^57^. The graphene grids were treated with UV ozone for 4 min before usage. Both samples were plunge-frozen using a Thermo Fisher Vitrobot Mark IV at 4 °C, 5 s, 100% humidity, and a blot force of 0 using Whatman #1 blotting paper.

### Cryo-EM data acquisition, processing and reconstruction

Cryo-EM data were collected on a Thermo-Fisher Talos Arctica transmission electron microscope operating at 200 keV using parallel illumination conditions^58,59^. Micrographs were acquired with a Gatan K2 Summit direct electron detector with a total electron exposure of 50e^−^/Å^2^ as a 98-frame dose-fractionated movie during a 9800-ms exposure time. The Leginon data collection software was used to collect 2126 micrographs on unmodified Holey Gold grids and 1394 micrographs on graphene coated Gold grids at 36,000 nominal magnification (1.15 Å/pixel) with a nominal defocus ranging from −0.7 to −1.2 µm. Stage movement was used to target the center of four 1.2-µm holes for focusing, and image shift was used to acquire high magnification images. The Appion image processing wrapper^60^ was used to run MotionCor2 for micrograph frame alignment and dose-weighting in real-time during data collection^61^. All subsequent image processing was done in CryoSPARC (v4.1) for the combined 3520 micrographs^62^. The CTF parameters were estimated using Patch CTF estimation (multi). Representative views of 2D classes were used as templates for template-based particles picking in CryoSPARC and ~5.7 million picked particle coordinates were extracted at 1.15 Å/pixel from the motion-corrected and dose-weighted micrographs with a box size of 256 pixels. These particles were used for reference-free ab initio three-dimensional reconstruction in CryoSPARC, which separated the dataset into three major classes corresponding to a 2:1 SerRS–SIRT2 complex, a 2:2 SerRS–SIRT2 complex, and a junk class. The ab initio reconstruction yielded 241,045 particles corresponding to the 2:1 complex, 253,041 particles corresponding to the 2:2 complex, and 97,005 particles assigned to the junk class, which were excluded from further analysis. Particles assigned to the 2:2 SerRS–SIRT2 complex were subjected to non-uniform refinement with C2 symmetry using the 2:2 ab initio map as the initial reference. This refinement resulted in a three-dimensional reconstruction of the 2:2 complex at an overall resolution of 3.73 Å, based on the gold-standard Fourier shell correlation at the 0.143 criterion. Because both the 2:1 and 2:2 ab initio classes contain a common 2:1 subcomplex, particles from both high-resolution classes were combined and refined using non-uniform refinement with C1 symmetry, using the 2:1 ab initio reconstruction as the initial reference. This refinement produced a reconstruction of the 2:1 SerRS–SIRT2 complex at an overall resolution of 3.82 Å. To further improve map quality, local refinement was performed using a mask focused on the 2:1 complex region, resulting in a final reconstruction at 3.38 Å resolution. All the resolutions were estimated using the 3DFSC server (https://3dfsc.salk.edu)^63^ using a Fourier Shell Correlation (FSC) cutoff at 0.143 (Supplementary Fig. 1). The crystal structure of SerRS dimer (PDB ID: 4L87) and the crystal structure of SIRT2 (PDB ID: 4RMI) were initially docked into the reconstructed density maps using UCSF Chimera (v1.14). Real-space structural refinement and manual model building were performed with Phenix (v1.17.1)^64^ and Coot (v0.8.9.1)^65^, respectively. Phenix real-space refinement includes global minimization, rigid body, local grid search, atomic displacement parameters, and morphing for the first cycle, and was run for 100 iterations, 5 macro cycles, with a target bonds RMSD of 0.01 and a target angles RMSD of 1.0. The refinement settings also include the secondary structure restraints, Ramachandran restraints. Figures of the structures were prepared using UCSF Chimera (v.1.14), and UCSF ChimeraX (v1.8)^66,67^. Supplementary Table 1 includes data collection, refinement, and validation statistics^68^.

### Cell culture and constructs

For cell-based assays, human full-length SIRT2 was cloned into pCDNA6-V5/His-C vector (Life Technologies), while human SerRS was cloned into pFlag-CMV-2 vector (Sigma-Aldrich). These constructs were used to generate the SerRS and SIRT2 mutants tested in this study. All cells were cultured in a humidified incubator at 37 °C with 5% CO_2_. Human HEK293 cell lines were purchased from the American Type Culture Collection (ATCC, #CRL-1573, Manassas, VA, USA) and the human HEK293AD cell line was purchased from Agilent (Agilent, #240085, Santa Clara, CA, USA). Cells were maintained in Dulbecco’s Modified Eagle Medium (ThermoFisher Scientific, Grand Island, NY, USA) supplemented with 10% fetal bovine serum (Omega Scientific, Tarzana, CA, USA) to a final concentration of 10% and 1% Penicillin-Streptomycin (ThermoFisher Scientific). Transient transfections were performed by using Lipofectamine 3000 (ThermoFisher Scientific).

### Coimmunoprecipitation assays and Western blot analysis

For mutagenetic analysis, HEK293AD cells were transiently transfected with Flag-SerRS and SIRT2-V5 for 48 h. Then cells were washed with PBS pH 7.4 and lysed on ice for 5 min using cell lysis buffer (20 mM Tris-HCl pH 7.5, 150 mM NaCl, 1 mM EDTA, 1 mM EGTA, 1% NP40, 0.5% sodium deoxycholate, and freshly added protease inhibitor cocktail). The indicated antibodies (anti-Flag: Cell signaling technology, #8146, 9A3, 1:2000; anti-V5: Cell signaling technology, #80076, E9H8O, 1:2000) were incubated with 20 µL protein-G-conjugated agarose beads (Invitrogen) at 4 °C for 2 h. The supernatants of the cell lysates were collected and incubated with the antibodies coated beads overnight at 4 °C. The beads were then washed three times with ice-cold wash buffer (20 mM Tris-HCl pH 7.5, 150 mM NaCl, 1 mM EDTA, 1 mM EGTA, 0.1% Triton X-100, and freshly added protease inhibitor cocktail). After the final wash, the beads were incubated in SDS loading buffer for 5 min, followed by SDS-PAGE and Western blotting analysis with the indicated antibodies (anti-Flag: Cell signaling technology, #14793, D6W5B, 1:2000; anti-V5: Cell signaling technology, #13202, D3H8Q, 1:2000).

To assess PARP1 activation upon H₂O₂ treatment, HEK293AD cells were treated with 5 mM H₂O₂ for the indicated time periods. As a negative control, cells were pretreated with the PARP1 inhibitor PJ34 (10 μM) for 24 h prior to H₂O₂ treatment. Cells were then washed, lysed in SDS loading buffer, and boiled at 95 °C for 5 min, followed by Western blot analysis with indicated antibodies (anti-poly ADP-ribosylation: Cell signaling technology, #89190, D9P7Z, 1:2000; anti-α-tubulin: Cell signaling technology, #3873, DM1A, 1:2000). For co-immunoprecipitation (co-IP) experiments, SIRT2-V5 was transiently transfected to HEK293AD cells for 48 h before H₂O₂ treatment. Then H₂O₂ and PJ34 were treated as shown. Custom-made anti-SerRS monoclonal mouse antibody (18A8, 3 μg/sample) was incubated with protein-G-conjugated agarose beads (Invitrogen) at 4 °C for 2 h. Then cells were washed and lysed in 1× RIPA Buffer (protease inhibitor cocktail added). The whole cell lysates were collected and incubated with the antibody coated beads at 4 °C for 4 h. The beads were then washed and boiled with SDS loading buffer for 5 min, then immediately followed by SDS-PAGE and Western blotting analysis with the indicated antibodies (anti-SerRS: Bosterbio, #A00501-1, 1 μg/mL; anti-SIRT2: Abcam, #ab211033, EPR20411-105, 1:1000).

### Ni-NTA pull-down assay

The in vitro Ni-NTA pull-down assay was performed in the buffer with 20 mM Hepes (pH 7.5), 150 mM NaCl, 50 mM imidazole (pH 8.0), 10% glycerol and 0.5% NP-40. In each experiment, 350 nM SIRT2-His (3 μg in 200 μL) was pre-bound to 10 µL nickel beads at 4 °C for 2 h, followed by addition of 300 nM SerRS (3 μg in 200 μL) and co-incubation at 4 °C for 2 h. To assess the effect of NAD^+^, ADPR or OAADPR on inducing the SerRS–SIRT2 interaction, SIRT2-His was incubated with varying concentration of these compounds during beads binding. To assess the competition between SIRT2 and in vitro*-*transcribed (IVT) tRNA^Ser^ for SerRS binding, SIRT2-His was incubated with or without ADPR and beads, followed by addition of SerRS and tRNA^ser^ (1:1 mixture of tRNA^Ser(GCT)^ and tRNA^Ser(CGA)^) for co-incubated. To assess the competition between SIRT2 and *VEGFA* promoter DNA for SerRS binding, SIRT2-His was pre-incubated with ADPR and beads. Separately, SerRS was pre-incubated with or without DNA on ice for 1 h prior to mixing and co-incubation. After 3 washes, proteins pulled down by nickel beads were dissolved in sample loading buffer and detected by Western blot with the indicated antibodies (anti-SerRS: Custom-made mouse monoclonal antibody −24C4, 1 μg/mL; anti-SIRT2: Abcam, #ab211033, EPR20411-105, 1:1000).

### Biolayer interferometry

SerRS–SIRT2 interactions were analyzed using Octet RED96 system (Sartorius) at 25 °C with 1× kinetic buffer purchased from Sartorius. SIRT2-His was immobilized on HIS1K biosensor tips for 5 min, equilibrated in buffer containing 0.5 mM NAD^+^,0.5 mM ADPR or no ligand to establish a baseline. The SIRT2 coated tips were then dipped into 180 nM SerRS for 5 min association, followed by 5 min dissociation in ligand-only buffer. Data were processed by subtracting the reference (SerRS alone without ligand) and aligning the association and disassociation curves.

### Mass photometry

The dissociation constants (K_D_) of the SerRS–SIRT2 interaction at varying concentrations of NAD⁺ or ADPR were determined using mass photometry. Protein concentrations of SerRS and SIRT2 were quantified using a BCA assay, diluted to working concentrations, and incubated with NAD⁺ or ADPR for 30 min at room temperature. Immediately prior to measurement, samples were diluted twofold by mixing 10 μL buffer for calibration with 10 μL of the protein mixture. Peaks corresponding to SIRT2, the SerRS dimer, and the SerRS:SIRT2 (2:1) complex were assigned based on their molecular weights. Particle counts for each peak from individual measurements were used to calculate K_D_ values according to previously described protocols^29,30^.

### ESI triple quad mass spectrometry analysis

Calibration standards were prepared by diluting stocks of ADPR, Acetyl-ADPR and NAD^+^ to the appropriate calibration levels. To probe whether SerRS acts as a substrate for SIRT2, the SIRT2 deacetylation assay was performed at room temperature for 3 h in 100 μL of 1 μM SIRT2, 10 μM NAD^+^, and 5 μM acetyl-α-tubulin peptide or SerRS. To facilitate the release of enzyme-bound ligands, 100 μL of 10 μM co-purified SerRS–SIRT2 complex or the individually purified proteins were denatured by boiling at 95 °C for 2 min. All samples were followed by the addition of 400 μL of ice-cold MeOH and incubation at −20 °C for 1 h. After centrifugation at 15,700 × g for 15 min at 4 °C, the supernatants were evaporated overnight in a SpeedVac at 10 °C. Each sample was added with 100 μL of 50:50 MeOH:H_2_O, sonicated for 10 min and centrifuged at 15,000 × g for 10 min, then transferred to autosampler vials for analysis. The samples were run on an Agilent 6495 triple quadrupole mass spectrometer coupled to an Agilent 1290 UPLC stack. The column used was a Waters BEH-amide 2.1×150 mm. The mobile phase composition was as follows: mobile phase A: 10 mM NH_4_OAc, 5 μM deactivator, pH 9, and mobile phase B: 90:10 ACN:H_2_O, 10 mM NH_4_OAc, 5 μM deactivator, pH 9. The flowrate was set to 250 µL/min and the sample injection volume was 5 µL. The gradient was: T0: 0/100, T1: 0/100, T10: 65/35 T13: off, with a 5 min re-equilibration step. Operating in negative ion mode, the source conditions were as follows: Capillary voltage: 2500 V, Gas temp: 120 °C, Gas flow: 11 L/min, Nebulizer pressure: 35 psi. Sheath gas temp: 350 °C, sheath gas flow: 12 L/min. Nozzle voltage was set to 1500 V. Each condition was analyzed as a single measurement. Data was processed using Agilent MassHunter Quantitative Analysis (v12.1). Calibration curves were prepared in concentrations ranging from 10 nM to 5000 nM, with the lower limit of quantitation (LLOQ) set at 10 nM and the upper limit of quantitation (ULOQ) set at 5000 nM.

### Trypsin digestion and mass spectrometry analysis

SDS-PAGE gel bands of SerRS (from recombinant SerRS alone or SerRS–SIRT2 complex) were cut out, destained, reduced (10 mM DTT), alkylated (55 mM iodoacetamide), and digested with 10 ul of trypsin overnight before nano-LC-MS/MS analysis. The analysis was performed using Thermo Scientific Easy nano LC 1200 coupled with Thermo Scientific Q Exactive Plus using Nanospray Flex ion source. 8 μL of digested peptides were separated by reverse-phase chromatography on a 14 cm length x 75 mm inner diameter nano electrospray capillary column, packed in-house with Phenomenex Aqua 3 µm C18 125 Å. Mobile phase A was water including 0.1% formic acid, and mobile phase B was 80% acetonitrile plus 0.1% formic acid. The elution gradient started at 2% B for 5 min, ramped up to 25% B for 100 min, 40% B for 20 min, 95% B for 1 min and held at 95% B for 14 min. Data acquisition was performed using Xcalibur (v4.3). One MS scan of m/z 400–2000 was followed by 10 MS/MS scans on the most abundant ions with application of the dynamic exclusion of 10 sec. MS/MS Raw data was searched against the Custom sequence database which contains the sequence of SerRS and the NCBI Homo sapiens (human) database using Mascot (v2.8.0, Matrix Science). Mascot searches were conducted using a peptide mass tolerance of 10 ppm, a fragment ion mass tolerance of 0.01 Da, fixed modifications of carbamidomethyl (C), variable modifications of Acetylation (K), Myristoylation (K) and Palmitoylation (K), an enzyme of trypsin and a maximum missed cleavage of one. Identification was done with a false discovery rate (FDR) of <2% as determined by using a decoy database search.

### Fluorometric SIRT2 deacetylase activity assay

SIRT2 deacetylase activity was measured using Cyclex SIRT2 Deacetylase Fluorometric Assay Kit (CycLex, Nagano, Japan). The reaction buffer contains 50 mM Tris-HCl (pH 8.8), 0.5 mM DTT, 0.25 mAu/mL Lysylendopeptidase, 1 µM Trichostatin A, 0.8 mM NAD^+^, 20 uM Fluoro-Substrate peptide, 1 µM recombinant SIRT2 and recombinant SerRS at different ratio indicated. For assessing the impact of ADPR, 0.25 µM recombinant SIRT2, 1 µM SerRS or BSA (negative control) were pre-incubated with the indicated concentrations of ADPR at room temperature for 30 min. Subsequently, 4 µM Fluoro-Substrate peptide, 40 µM NAD^+^ were added to initiate the reaction.

### Cell-based SIRT2 deacetylase activity assay

HEK293T or HEK293AD cells were seeded in 6 well plate and allowed to adhere overnight. The following treatments were then applied: 5 µM SIRT2 inhibitor AGK2, SerRS knockdown by siRNA (Santa Cruz), SIRT2 overexpression, SerRS overexpression or combined overexpression of both SIRT2 and SerRS. 3 days post-treatment, 5 µM Tubastin A was applied for 6 h to inhibit HDAC6-mediated deacetylation. cells were then washed with cold PBS (pH 7.4) and lysed on ice with lysis buffer containing freshly added protease inhibitor cocktail (Thermo scientific) for 5 min. All samples were applied to SDS-PAGE followed by Western blot analysis using the indicated antibodies (anti-α-tubulin: Cell signaling technology, #3873, DM1A, 1:2000; anti-acetylated-α-tubulin (K40Ac): Cell signaling technology, #5335, D20G3, 1:1000; anti-H4: Active motif, # 91295, 1:1000; anti-acetylated-H4K16: Active motif, #39167, 1:1000; anti-SerRS: 24C4, 1 μg/mL; anti-SIRT2: Abcam, #ab211033, EPR20411-105, 1:1000).

For the time course investigation, HEK293AD cells were harvested 48 h post-transfection of SerRS, SIRT2, or both. cells were washed and lysed within 150 µL/well cell lysis buffer (freshly added 8 µM Tubastin A and protease inhibitor cocktail) on ice for 5 min. Supernatants were collected and incubated at room temperature for 40 min to stabilize Ac-α-tubulin levels. For each reaction, 100 µL supernatant was used in a 400 µL reaction system containing 50 mM Tris-HCl (pH 9.0), 4 mM MgCl_2_, 0.2 mM DTT, 8 µM Tubastin A, and 2 mM NAD^+^. At each time points, 40 µL reaction mixtures were sampled for western blotting analysis with indicated antibodies (anti-α-tubulin: Cell signaling technology, #3873, DM1A, 1:2000; anti-acetylated-α-tubulin (K40Ac): Cell Signaling Technology, #5335, D20G3, 1:1000; anti-SerRS: 24C4, 1 μg/mL; anti-SIRT2: Cell signaling technology, #12650, D4O5O, 1:1000).

### Hydrogen deuterium exchange mass spectrometry

To initiate hydrogen deuterium exchange (HDX) reactions, 3 μL of each protein stock solution (SerRS, SIRT2 or co-purified SerRS–SIRT2 protein) was mixed with 9 μL of D_2_O buffer (8.3 mM Tris, 150 mM NaCl in D_2_O, pH 7.6), respectively, and incubated for 10, 100, 1000 s at 0 °C. At the designated times, HDX reactions were quenched by adding 18 μL of ice old quench buffer (0.08 M GuHCl, 0.8% formic acid, 16.6% glycerol, pH 2.4) and samples were immediately frozen at –80 °C. Control samples, both non-deuterated and fully deuterated, were also prepared following the previously established method^69^. The 30 μL quenched samples were thawed at 4 °C and immediately passed over the immobilized pepsin column (1 × 20 mm, 40 mg/mL porcine pepsin (Sigma)). The digested peptides were collected on a trap column (Michrom MAGIC C18AQ, 0.2 × 2 mm) for desalting and separated with a Magic C18 column (Michrom, 0.2 × 50 mm, 3 μm, 200 Å) by a 30 min linear acetonitrile gradient of 6.4–38.4%. The accurate peptide mass measurement was performed with an OrbiTrap Elite mass spectrometer (Thermo Fisher Scientific), which was set up for optimal HDX performance with minimum back-exchange rate^70^. Data acquisition was completed in either data-dependent MS/MS or MS1 profile mode and peptide identification was done using Proteome Discoverer software (v1.3.0.339, Thermo Fisher Scientific). A total of 275 peptides with 98.40 % of SIRT2 sequence coverage and 273 peptides with 96.16% of SerRS sequence coverage were employed to track deuterium exchange. Deuterium incorporation levels of each peptide at each time-points were determined by HDExaminer (v2.5.1, Sierra Analytics Inc) with back-exchange correction using above control samples. The mass spectrometry proteomics data have been deposited to the ProteomeXchange^71^ Consortium via the PRIDE^72^ partner repository with the dataset identifier PXD079339.

### In vitro transcription of tRNA

Template DNAs for human tRNA^Ser(CGA)^ and tRNA^Ser(GCT)^ were amplified through PCR. Subsequent in vitro transcription was performed using T7 RNA polymerase (HiScribe T7 High yield RNA Synthesis kit, NEB). The reaction was performed at 37 °C for overnight. The transcription product was then subjected to 10% Urea-PAGE (Life Tech) to assess purity and separate it from proteins and NTPs. The specific tRNA transcripts bands were excised, dissolved in 0.3 M NaOAc (pH 5.0) and 1 mM EDTA, followed by ethanol precipitation of the supernatant. Prior to use in Ni-NTA pull-down assay and aminoacylation assay, in vitro–transcribed (IVT) tRNA^Ser(CGA)^ and tRNA^Ser(GCT)^ were mixed at a 1:1 ratio, incubated at 95 °C for 5 min, and subsequently refolded at 60 °C in the presence of 5 mM MgCl_2_ and gradually cooling down to room temperature.

### Aminoacylation assay

Aminoacylation assays were performed in 50 mM HEPES pH 7.5, 20 mM KCl, 5 mM MgCl_2_, 4 mM ATP, 2 µM [^3^H]-L-serine, 20 µM L-serine, 2 mM DTT, 4 µg/mL pyrophosphatase (Roche), and either 20 µM yeast total tRNA or 5 µM of IVT- tRNA^Ser^. To assess SIRT2-mediated inhibition of SerRS in the presence or absence of ADPR, reactions were performed using 200 nM SerRS with 500 nM SIRT2 (SerRS:SIRT2 = 1:2.5) or PBS as a negative control, with or without 250 or 500 µM ADPR, using 5 µM in vitro-transcribed tRNA^Ser^. To compare the aminoacylation activity of co-purified SerRS–SIRT2 complex with free SerRS, reactions were performed using 200 nM SerRS or SIRT2 or SerRS–SIRT2 complex, with 20 µM yeast total tRNA. The reaction was initiated by addition of tested protein samples. At varying time intervals, 5 µL aliquots were removed and applied to a MultiScreen 96-well filter plate (0.45 µm pore size, hydrophobic, low-protein-binding membrane; Merck Millipore) containing 100 µL quench solution (0.3 M NaOAc pH 3.0, 0.5 mg/mL salmon sperm DNA, 0.1 M EDTA). After all time points were collected, 100 µL of 20% (w/v) trichloroacetic acid (TCA) was added to precipitate nucleic acids. The pellets in the filter plate were then washed 4 times with 200 µL wash solution (100 mM cold serine with 5% TCA) followed by one wash of 200 µL 95% ethanol. 70 µL of freshly prepared 100 mM NaOH was added to each well after the plate was dried out. After 10 min, the solution in the plate was centrifuged into a 96-well flexible PET microplate (PerkinElmer) with 150 µL of Supermix scintillation mixture (PerkinElmer). After mixing, the radioactivity in each well of the plate was measured in a 1450 MicroBeta Micoplate Scintillation and Luminescence Counter (PerkinElmer).

### RNA extraction and acidic gel northern blot

HEK293AD cell lines over-expressing SIRT2^WT^ or SIRT2^N168A^ were selected by 1 μg/mL puromycin for 7 days. SerRS knockdown was achieved by siRNA (Santa Cruz) for 5 days. Cells were harvested for both detection of protein expression level and tRNA charging level. Total RNA was extracted by Trizol (Invitrogen) and all deacetylated samples were dissolved in 10 mM NaOAc (pH 4.5). For deacetylated samples, total RNA was incubated at 37 °C for 1.5 h in 0.2 M Tris-HCl (pH 9.5), followed by ethanol precipitation and resuspension in 10 mM NaOAc (pH 4.5).

Total RNA (5 μg) from each sample was loaded to an acidic 6.5% polyacrylamide/8 M urea gel and electrophoresed in 0.1 M NaOAc (pH 5.0) at constant 500 V for 18 h at 4 °C^73^. After electrophoresis, RNA was transferred onto an Amersham^TM^ Hybond-N^+^ membrane. The membrane was pre-hybridized for 30 min at 65 °C for tRNA^Ser(AGA)^ and 60 °C for tRNA^Tyr(GTA)^, in a buffer containing 20 mM Sodium phosphate (pH 7.2), 0.3 M NaCl and 1% SDS. Hybridization was performed overnight at the corresponding temperature using the same buffer with biotin labeled-DNA probes (tRNA^Ser(AGA)^ probe: 5’-TGGCGTAGTCGGCAGGATTCGAACCTGCGCGGGGARACCCCAATGGATTTCTAGTCCATCGCCTTAACCACTCGGCCACGACTAC-3’, tRNA^Tyr(GTA)^ probe: 5’-TCCTTCGAGCCGGASTCGAACCAGCGACCTAAGGATCTACAGTCCTCCGCTCTACCARCTGAGCTATCGAAGG-3’)^33^. The membrane was washed with buffer containing 20 mM Sodium phosphate (pH 7.2), 0.3 M NaCl, 0.1% SDS and 2 mM EDTA, and subsequently incubated with HRP-streptavidin (Thermo Scientific) at 37 °C for 30 min. The aminoacylation fraction was calculated as the ratio of charged tRNA to the total amount of charged and uncharged tRNAs.

### Puromycin incorporation assay

HEK293AD cells transiently overexpressing WT, N168A or H187Y SIRT2 or with SerRS knockdown by siRNA (Cell signaling) were seeded in 6-well plates. Fourteen hours post-transfection, 10 µg/mL Puromycin was added to each well and incubated for 30 min in the humidified incubator. Cells were then washed once with fresh medium and incubated in fresh medium for an additional 1–1.5 h. After washing with ice cold PBS, cells were lysed in 500 µL/well lysis buffer. Then equal amounts of proteins from each sample were then subjected to SDS-PAGE and analyzed by Western blot with indicated antibodies (anti-puromycin: Abcam, #ab315887, EPR27218-173, 1:2000; anti-SerRS: 24C4, 1 μg/mL; anti-SIRT2: R&D Systems, MAB4358, 1 μg/mL; anti-α-tubulin: Cell signaling technology, #3873, DM1A, 1:2000).

To assess global translation inhibition under oxidative stress, HEK293AD cells seeded in 6-well plates were transiently transfected with empty vector (EV), SIRT2-WT, or SIRT2-N168A. At 48 h post-transfection, cells were treated simultaneously with 10 μg/mL puromycin (Sigma-Aldrich) and 0.5 mM H₂O₂ for 30 min. Cells were then washed twice with PBS, lysed in lysis buffer supplemented with protease and phosphatase inhibitors, and analyzed by Western blot using the indicated antibodies (anti-puromycin: Millipore Sigma, #MABE343, 12D10, 1:3000, anti-GAPDH: Cell signaling technology, #2118, 14C10, 1:3000). ISR pathway activation and mTOR pathway inhibition were assessed in parallel by Western blot using using the indicated antibodies (anti-p-eIF2α: Cell signaling technology, #9721, 1:1000; anti-eIF2α: Cell signaling technology, #9722, 1:1000; anti-ATF4: Cell signaling technology, #11815, D4B8, 1:1000; anti-p-p70 S6K: Cell signaling technology, #9234, 108D2, 1:1000; anti-p70 S6K: Cell signaling technology, #2708, 49D7, 1:1000; anti-p-4EBP1: Cell signaling technology, #2855, 236B4, 1:1000; anti-4EBP1: Cell signaling technology, #9644, 53H11, 1:1000).

### Multiple sequence alignment

Multiple sequence alignment was performed for human SIRT2 and SerRS protein sequences with their orthologs using MUSCLE^74^ with default parameters. Sequences were retrieved from the UniProt database and selected to represent a broad range of taxa, including bacteria, archaea, fungi, plants, early-branching metazoans (including cnidarians, placozoans, poriferans, and insects), non-vertebrates, and vertebrates. Sequence conservation was visualized using either ESPript^75^ or Jalview (v2.11.5.1)^76^. Residues of interest were highlighted based on the requirement.

### Quantification and statistical analysis

The quantification of all Western blot images was done using ImageJ (v2.1.0), Image Lab (v6.0.1, Bio-Rad) or Image Studio (v6.0, LI-COR). GraphPad Prism (v10.5.0) was used for data analysis. Statistical significance was calculated using Welch’s two-sided *t*-test or two-sided paired *t*-test, with a *p* value < 0.05 considered statistically significant. No adjustment was made for multiple comparisons. Data are presented as mean values ± SD.

### Reporting summary

Further information on research design is available in the Nature Portfolio Reporting Summary linked to this article.

## Supplementary information

Supplementary Information

Reporting Summary

Transparent Peer Review file

## Source data

Source Data

## Data Availability

Plasmids, antibodies, and cell lines are available from the corresponding author upon request. The cryo-EM maps and associated atomic models generated in this study have been deposited to the Electron Microscopy Databank (EMDB) and the Protein Databank (PDB), respectively, with the following EMDB and PDB IDs: EMD-47105 and 9DPI for SerRS: SIRT2 (2:2) and EMD-47103 and 9DPD for SerRS: SIRT2 (2:1). The HDX-MS data have been deposited to the ProteomeXchange Consortium via the PRIDE partner repository with the dataset identifier PXD079339. The trypsin digestion and mass spectrometry analysis and ESI triple quad mass spectrometry analysis are included in the Source data file. Source data are provided with this paper.
