# Supplementary material for: A metabolite-bridged complex between SerRS and SIRT2 couples NAD⁺ metabolism to translation control
Source: Nat Commun. 2026 Jul 8;17:8427. doi: 10.1038/s41467-026-75266-4 (PMC13478228; doi:10.1038/s41467-026-75266-4)
Supplement: Supplementary file 1 [file 41467_2026_75266_MOESM1_ESM.pdf]

A metabolite-bridged complex between SerRS and SIRT2 couples NAD$^+$ metabolism to translation control

Qian Zhang[1,5,6], Huimin Zhang[1,2,6], Marscha Hirschi[2], Sheng Li[3], Gabriel C. Lander[2], Jie Yang[2,4,‡], Xiang-Lei Yang[1,2,‡]

[1]Department of Molecular Medicine, The Scripps Research Institute, La Jolla, CA 92037, USA

[2]Department of Integrative Structural and Computational Biology, The Scripps Research Institute, La Jolla, CA 92037, USA.

[3]Department of Medicine and UCSD DXMS proteomics Resource, University of California, San Diego, La Jolla, CA 92023, USA.

[4]Department of Biochemistry and Molecular Genetics, University of Virginia School of Medicine, Charlottesville, VA 22903, USA

[5]Present address: Institute of Systems and Physical Biology, Shenzhen Bay Laboratory, Shenzhen, China.

[6]These authors contributed equally.

[‡]Correspondence: xlyang@scripps.edu; wfw7nc@virginia.edu

Supplementary Table 1. Cryo-EM data collection, refinement, and validation

	**SerRS: SIRT2 (2:2)**<br>**EMDB: EMD-47105**<br>**PDB: 9DPI**	**SerRS: SIRT2 (2:1)**<br>**EMDB: EMD-47103**<br>**PDB: 9DPD**
**Data collection**		
Microscope	Talos Arctica	Talos Arctica
Voltage (keV)	200	200
Detector	K2 Summit (Counting)	K2 Summit (Counting)
Magnification (nominal/calibrated)	36,000× / 43,478×	36,000× / 43,478×
Exposure navigation	Image shift to 16 holes	Image shift to 16 holes
Data acquisition software	Leginon	Leginon
Total electron exposure ($e^-/Å^2$)	50	50
Exposure rate (e^-/pixel/sec)	6.7	6.7
Frame length (ms)<br>Number of frames per micrograph	100<br>98	100<br>98
Pixel size (Å)	1.15	1.15
Defocus range (µm)	-0.7 to -1.3	-0.7 to -1.3
Micrographs collected (no.)	3520	3520
**Reconstruction**		
Image processing package	CryoSPARC	CryoSPARC
Total extracted particles (no.)	5,038,656	5,038,656
Refined particles (no.)	247,287	500,718
Final particles (no.)	247,287	500,718
Symmetry imposed	C2	C1
Resolution (Å)		
FSC 0.5 (unmasked / masked)	3.98 / 3.73	3.87 / 3.38
FSC 0.143 (unmasked / masked)	3.98 / 3.73	3.87 / 3.38
Resolution range (local)	3.3 – 4.5	3.0 – 4.5
3DFSC Sphericity	0.86	0.80
Sharpening B-factor ($Å^2$)	212.6	139.5
**Model Composition**		
Protein residues	1806	1417
Ligands	2	1
**Model Refinement**		
Refinement package	Phenix	Phenix
CC (volume / mask)	0.70 / 0.73	0.69 / 0.71
Bond lengths	0.003	0.003
Bond angles (°)	0.745	0.713
**Validation**		
Map-to-model FSC 0.5 (unmasked)	3.95	3.97
Ramachandran (%)		
Outliers	0.00	0.00
Allowed	6.58	5.47
Favored	93.42	94.35
MolProbity score	2.35	2.18
Poor rotamers (%)	2.19	2.09
Clashscore (all atoms)	14.11	11.05
C-beta deviations	0.00	0.00
CaBLAM Outliers (%)[1]	2.12	1.67
EMRinger	1.08	0.69

Supplementary Table 2. HDX-MS parameters for SerRS–SIRT2 interaction analysis.

Data Set	SerRS	SIRT2	SerRS-SIRT2
HDX reaction details	8.3 mM Tris, 150 mM NaCl, pD_{read} = 7.6, 25 °C	8.3 mM Tris, 150 mM NaCl, pD_{read} = 7.6, 25 °C	8.3 mM Tris, 150 mM NaCl, pD_{read} = 7.6, 25 °C
HDX time course (sec)	10, 100, 1000	10, 100, 1000	10, 100, 1000
HDX control samples	Maximally labeled control (SerRS)	Maximally labeled control (SIRT2)	Maximally labeled control (SerRS, SIRT2)
Back-exchange (mean / IQR)	46.15% / 11.62%	46.22% / 13.31%	46.22% / 13.31%
# of Peptides	273	275	275
Sequence coverage	96%	98%	98%
Average peptide length / Redundancy	18.7/10.3	16.6/15.3	18.7/10.3, 16.6/15.3
Replicates (biological or technical)	1	1	1
Repeatability	N/A	N/A	N/A
Significant differences in HDX (delta HDX > X D)	N/A	N/A	N/A

N/A indicates not applicable.

Supplementary Figures

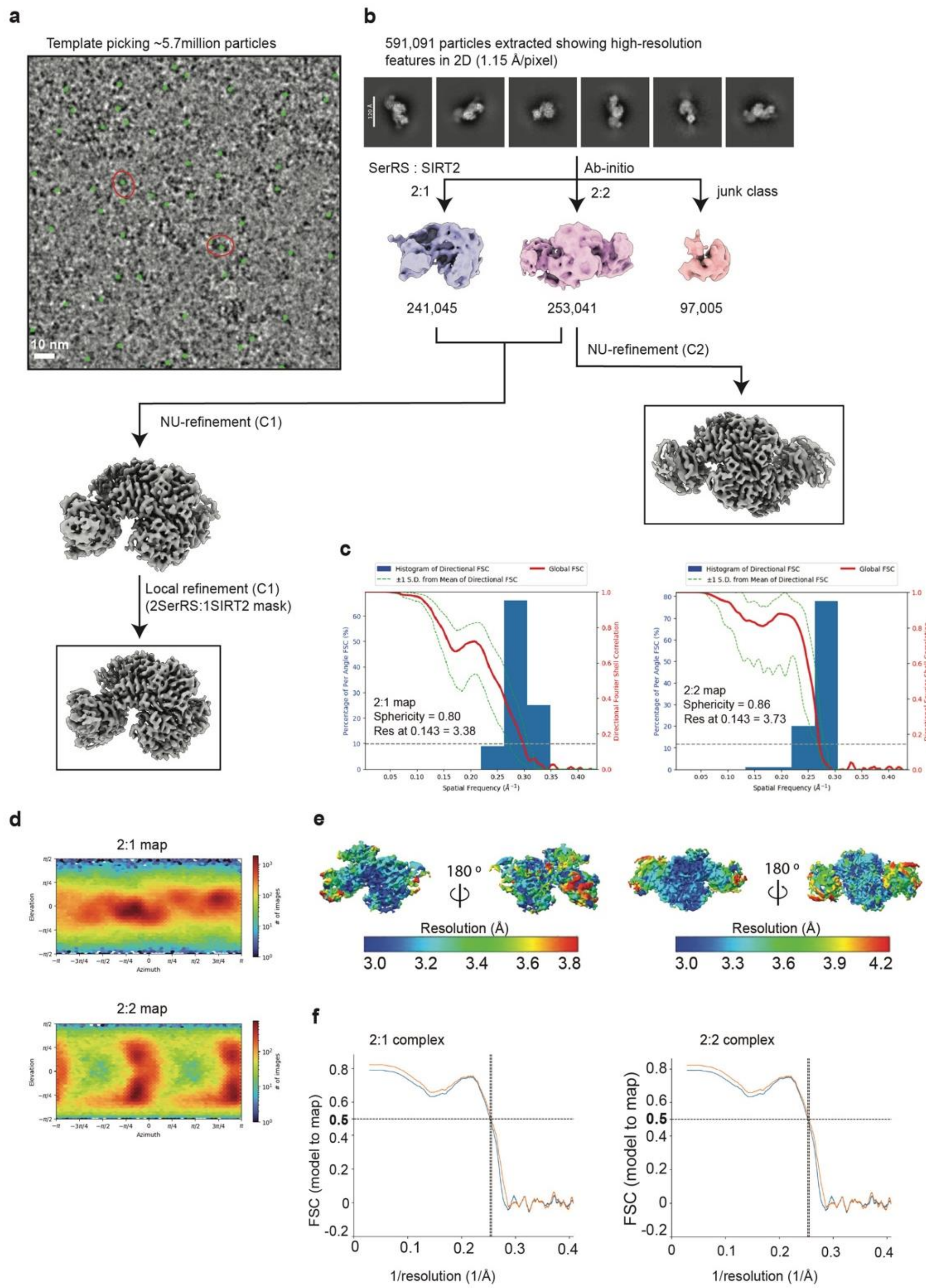

Supplementary Figure 1. Cryo-EM structure determination of SerRS–SIRT2 complex.

(a) Representative micrograph of cryo-EM data collection. Two representative SerRS–SIRT2 complexes are highlighted by red ellipses. (b) Workflow of cryo-EM data

processing using CryoSPARC software. The final 3D reconstruction 2:1 and 2:2 maps were used for model building and refinement. (c) Three-Dimensional Fourier Shell Correlation (3DFSC) of the 2:1, and 2:2 SerRS–SIRT2 reconstructions reporting a global resolution at 3.38 Å and 3.73 Å respectively. (d) Euler angle distribution plot of the 2:1 and 2:2 reconstructions. (e) Final reconstructions in (c) are filtered and colored by local resolutions estimated from CryoSparc. (f) Model-to-map FSC plots for the 2:1 and 2:2 maps from Phenix validation.

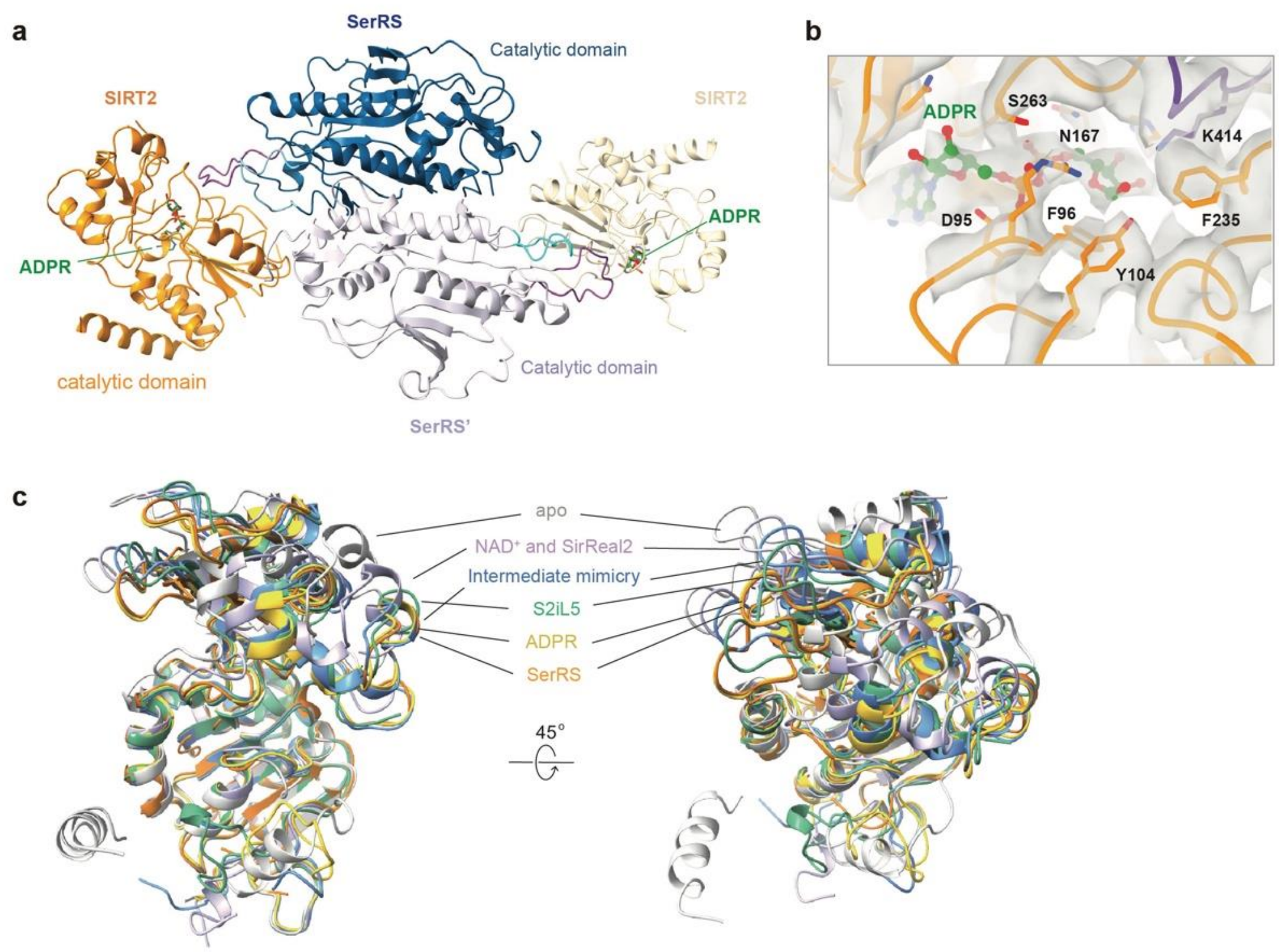

d

Ligand	PDB	RMSD to SIRT2 in complex with SerRS
apo	3ZGO	5.11 Å
NAD⁺ and SirReal2	4RMG	3.06 Å
Intermediate mimicry	4X3O	2.20 Å
S2iL5	4L3O	1.73 Å
ADPR	5D7O	1.26 Å

Supplementary Figure 2. SerRS–SIRT2 2:2 complex and structural comparison with other SIRT2 complexes.

(a) Ribbon representation of the overall cryo-EM density-fitted model of SerRS–SIRT2 2:2 complex. In both SerRS and SIRT2, only the catalytic domains of the two subunits are resolved, whereas the tRNA-binding arms of both SerRS subunits (SerRS and SerRS′) are largely disordered. Two SerRS loops involved in SIRT2 interaction are highlighted: the major loop (purple) and minor loop (cyan). ADPR molecules (green, stick representation) are observed at both SerRS–SIRT2 interfaces. (b) Close-up view of the ADPR binding pocket showing the cryo-EM density surrounding the ligand and adjacent SIRT2 residues. The local resolution in the vicinity of the ADPR ligand is estimated to be approximately 3.2–3.6 Å. (c) Superposition of the SIRT2 structure from the SerRS–SIRT2 complex with apo SIRT2 and ligand-bound SIRT2 structures; corresponding PDB codes are listed in (d).

(d) Root-mean-square deviation (RMSD) values between SIRT2 in the SerRS–SIRT2 complex and other ligand-bound SIRT2 structures.

a

Compound	Precursor ion (MS1)	Quantifier ion transition (MS2)	Qualifier ion transition (MS2)	Collision energy (V)
NAD^+	662.1	540	78.9	22/80
OAADPR	600.07	346	78.9	30/74
ADPR	558.06	346	79	30/74

b

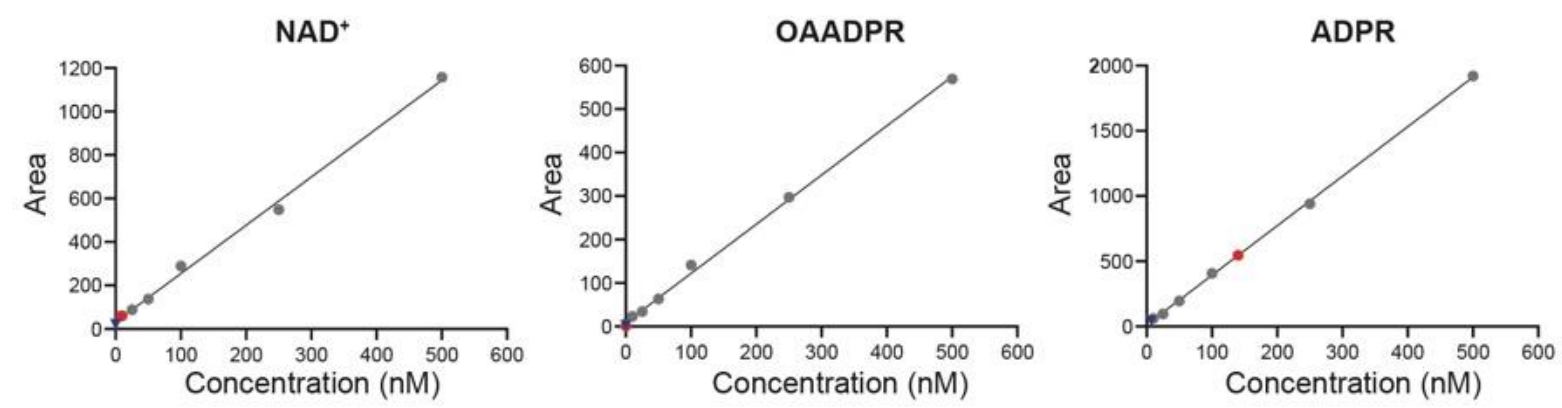

	NAD^+ (nM)	OAADPR (nM)	ADPR (nM)
Co-purified SerRS–SIRT2	< LLOQ	< LLOQ	140.28
Seperately purified SIRT2 and SerRS	< LLOQ	< LLOQ	< LLOQ

The lower limit of quantification (LLOQ) in this experiment was 10 nM.

Supplementary Figure 3. ADPR, but not NAD^+ or OAADPR, is detected in the co-purified SerRS–SIRT2 complex.

(a) Electrospray ionization (ESI) triple quadrupole mass spectrometry parameters used for the detection of NAD^+, OAADPR, and ADPR. (b) Standard curve of NAD^+, OAADPR, and ADPR established through ESI triple quadrupole mass spectrometry (6 points ranging from 10-500nM) for quantifying the concentrations of ligands extracted from the co-purified SerRS–SIRT2 complex (red dots) and from separately purified SIRT2 and SerRS proteins (blue triangles). n=1 independent experiment. Source data are provided as a Source Data file.

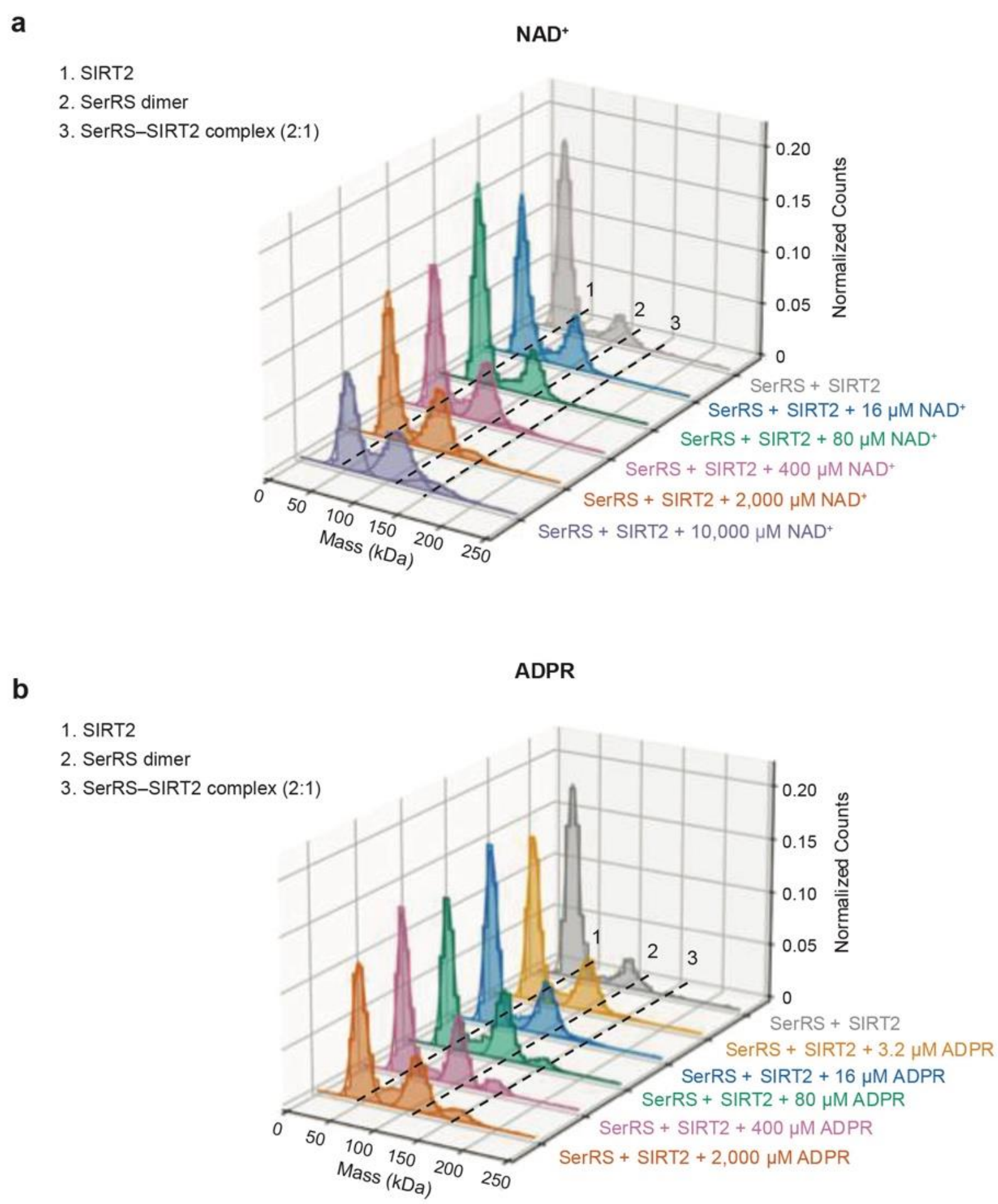

Supplementary Figure 4. Mass photometry profiles of ligand titration to determine K_D of SerRS and SIRT2.

(a,b) Three-dimensional histograms of mass photometry measurements showing peaks corresponding to SIRT2 (peak 1), SerRS dimer (peak 2), and SerRS–SIRT2 complex (peak 3) at increasing concentrations of (a) NAD^+ (16 µM-10 mM) or (b) ADPR (3.2 µM-2 mM). Data were used to determine the dissociation constants (K_D) for SerRS–SIRT2 interaction. Source data are provided as a Source Data file.

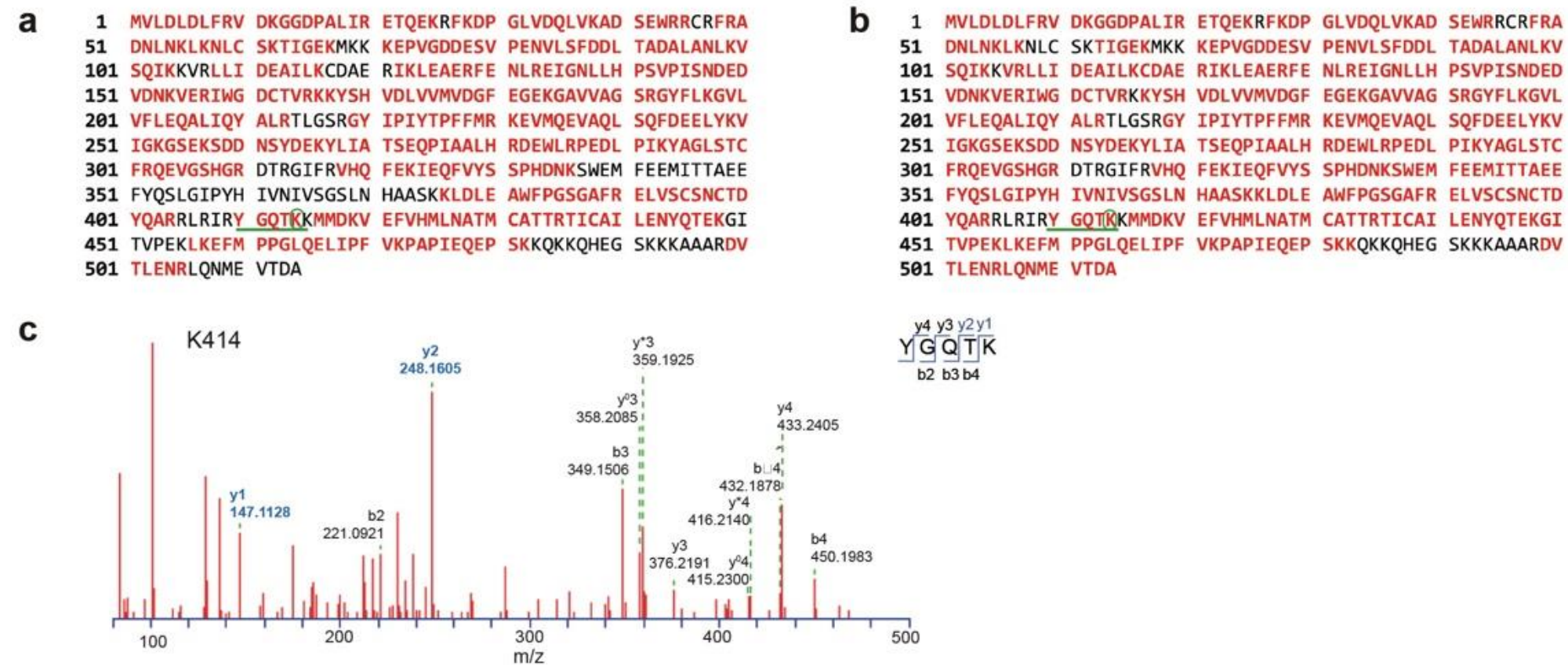

d

Acetylation site	Peptide	SerRS			SerRS/SIRT2		
		Counts of Ac-peptide	Counts of peptide	Acetylation rate	Counts of Ac-peptide	Counts of peptide	Acetylation rate
K12	10-20	15	47	31.91%	1	12	8.33%
K28	27-38	17	376	4.52%	25	407	6.14%
K55	50-57	2	3	66.67%	2	3	66.67%
K57	56-62	1	1	100.00%	---	---	---
K116	108-121	---	---	---	3	5	60.00%
K123	122-128	0	2	0.00%	4	18	22.22%
K154	134-157	0	8	0.00%	2	290	0.69%
K167	167-184	17	834	2.04%	10	562	1.78%
K231	231-249	27	180	15.00%	19	183	10.38%
K253	250-257	---	---	---	1	1	100.00%
K257	254-266	2	11	18.18%	---	---	---
K266	258-281	2	9	22.22%	4	6	66.67%
K293	282-302	1	5	20.00%	1	5	20.00%
K323	318-336	6	13	46.15%	4	11	36.36%
K376	376-390	22	349	6.30%	25	348	7.18%
K419	416-435	47	992	4.74%	48	680	7.06%
K448	436-455	---	---	---	4	8	50.00%
K455	449-457	---	---	---	1	2	50.00%
K457	456-482	0	1	0.00%	7	25	28.00%
Total		159	2831	5.62%	161	2566	6.27%

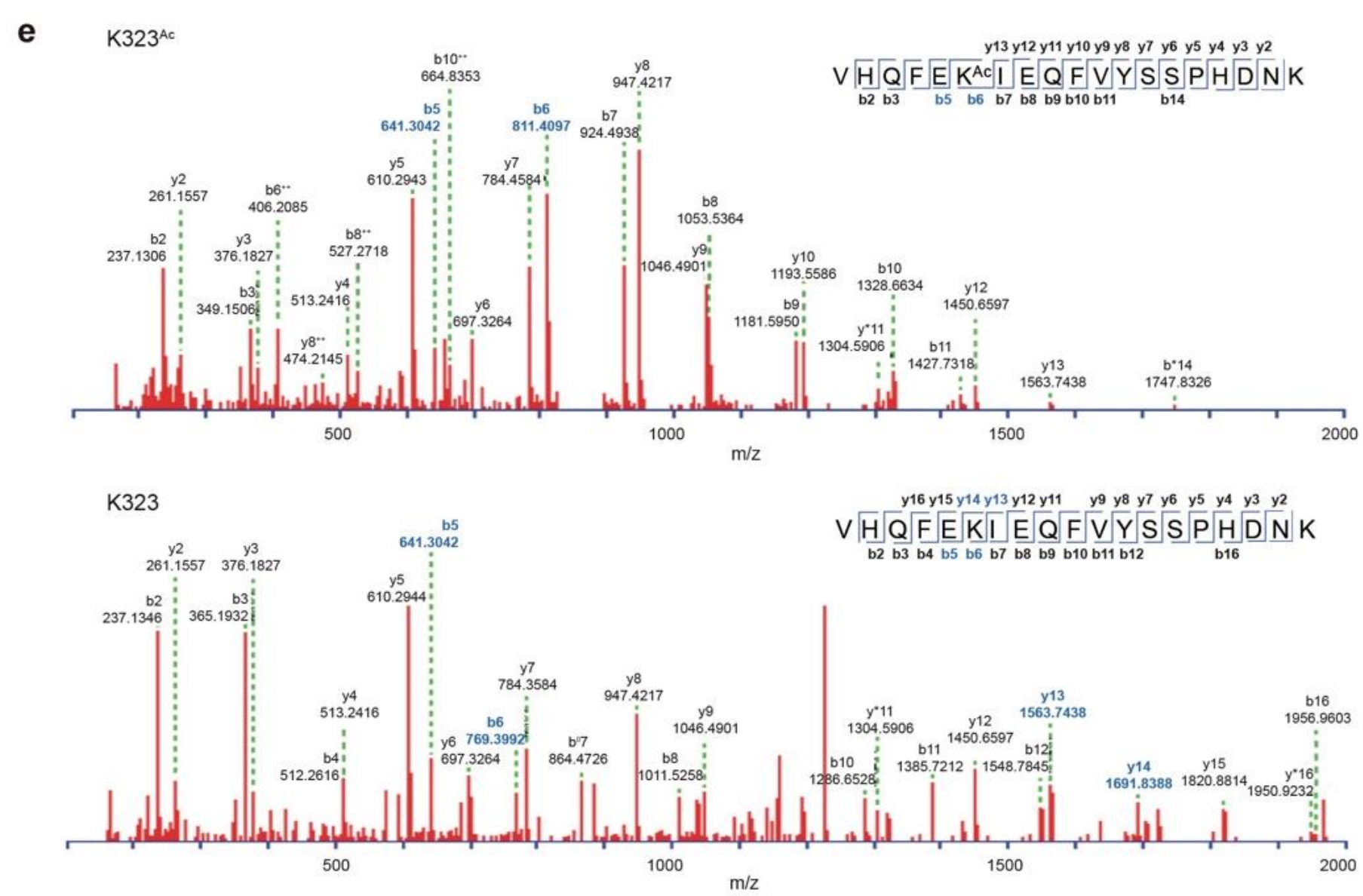

Supplementary Figure 5. Trypsin digestion and mass spectrometry analysis showing that SerRS K414 is not acetylated.

(a,b) Peptides coverage (red) of free recombinant SerRS (a) and SerRS in the co-purified SerRS–SIRT2 complex (b). n=1 independent experiment. The K414-containing peptide

(^{410}YGQTK^{414}) is underlined, and K414 is highlighted by a circle. (c) Tandem mass spectrometry analysis of the K414-containing peptide showing no evidence of K414 acetylation. (d) Summary of lysine acetylation sites in SerRS detected by the mass spectrometry analysis. (e) Representative example of a detected lysine acetylation site (K323) by the mass spectrometry analysis. Source data are provided as separate Supplementary data files.

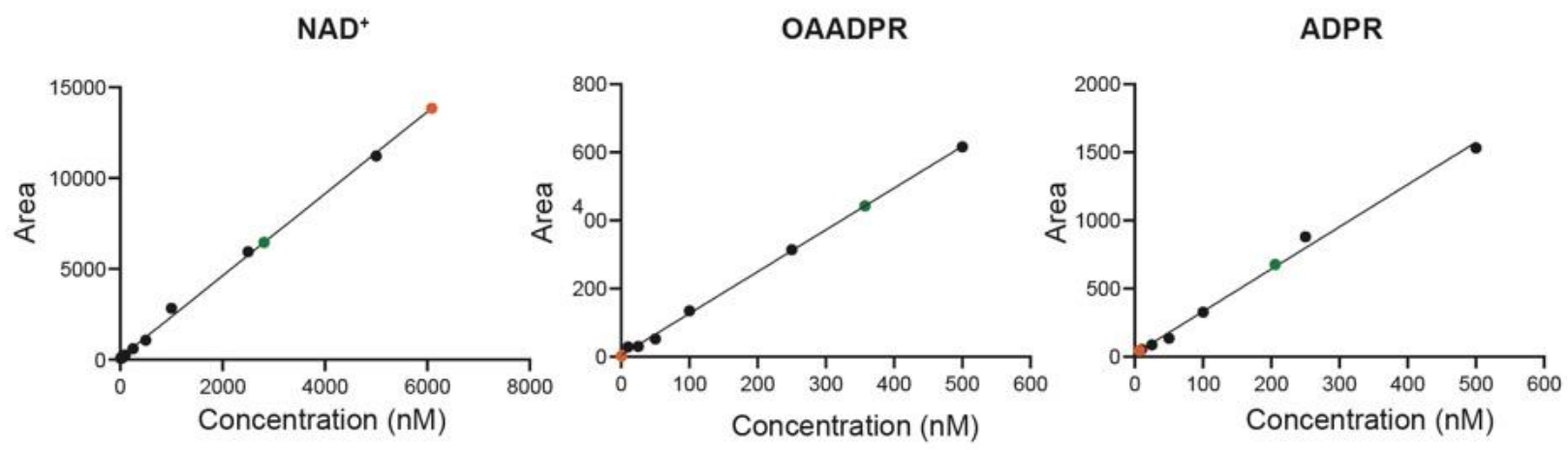

Substrate	NAD^+ (nM)	OAADPR (nM)	ADPR (nM)
5 μM Ac-α-tubulin peptide	2964	357	205
5 μM SerRS	7089*	< LLOQ	< LLOQ

“The lower limit of quantification (LLOQ) in this experiment was 10 nM.

* The value is an estimate, as it exceeds the upper limit of quantification (ULOQ, 5000 nM)

Supplementary Figure 6. Lack of SIRT2 deacetylation activity toward SerRS.

Concentration of ligands extracted from SIRT2 deacetylation reactions using an acetyl-α-tubulin peptide (green dots) or SerRS (orange dots) as the substrate is quantified using standard curves for NAD^+, OAADPR, and ADPR. n=1 independent experiment. While reactions with the acetyl–α-tubulin peptide produced detectable OAADPR and ADPR, no OAADPR or ADPR was detected in reactions using SerRS as the substrate, indicating an absence of SIRT2 deacetylation activity toward SerRS. Source data are provided as a Source Data file.

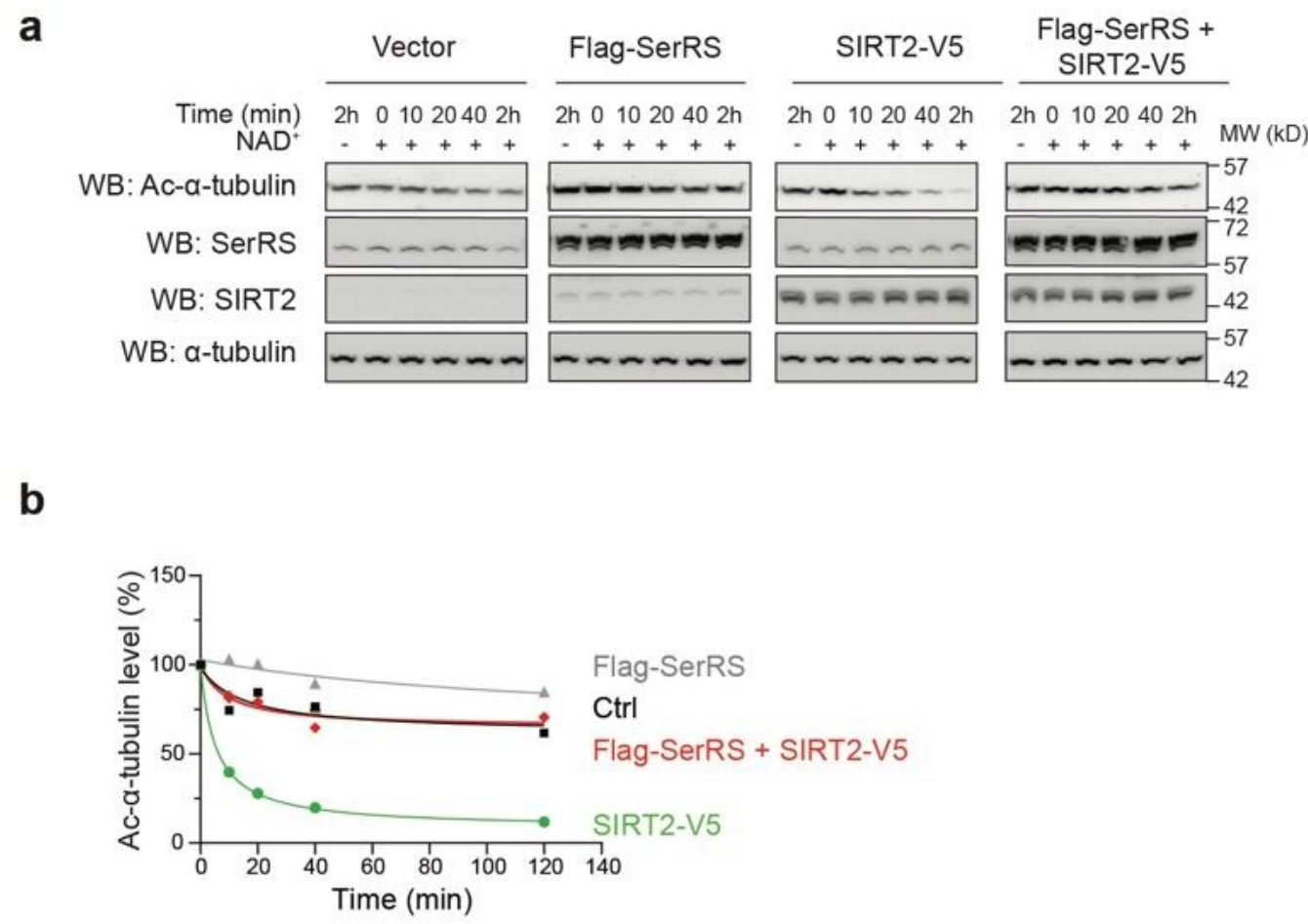

Supplementary Figure 7. SerRS inhibits SIRT2 deacetylation activity in a time-course, cell-based assay.

(a) Representative deacetylation assays using HEK293 cell lysates overexpressing vector control, SerRS, SIRT2 or both SerRS and SIRT2. (b) Quantification of acetyl-α-tubulin level from (a). Acetyl-α-tubulin levels at each time point were normalized to the 0 min time point, which was defined as 100% for each reaction. Data are presented as mean values from n=2 independent experiments. Source data are provided as a Source Data file.

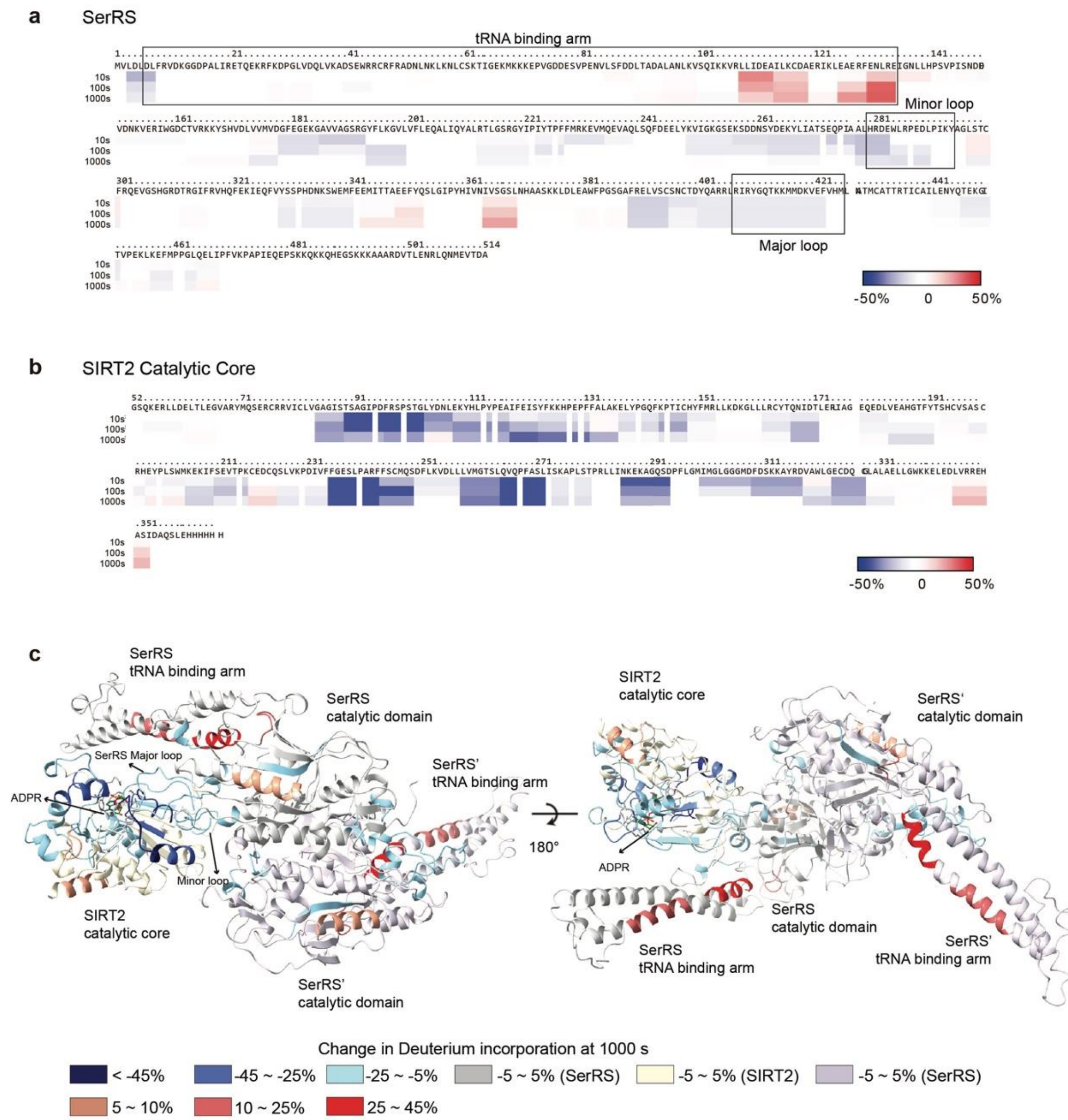

Supplementary Figure 8. Hydrogen-Deuterium Exchange (HDX)-Mass Spectrometry analysis of SerRS–SIRT2 interaction.

(a) Differential deuterium incorporation of SerRS upon binding with SIRT2$_{52\text{-}356}$-His over the time course of 10-1000 s, mapped onto the SerRS sequence. Blue indicates regions with increased protection, while red indicates regions with increased exposure upon SIRT2 binding. n=1 independent experiment. (b) Differential deuterium incorporation of SIRT2$_{52\text{-}356}$-His upon SerRS binding over 10-1000 s, mapped onto the SIRT2 sequence. (c) Differential deuterium incorporations at 1000 s from (a) and (b), mapped onto the SerRS–SIRT2 2:1 complex model. The disordered region of the SerRS tRNA binding arm was

modeled based on the crystal structure of SerRS in complex with a seryl-adenylate analog (PDB ID: 4L87).

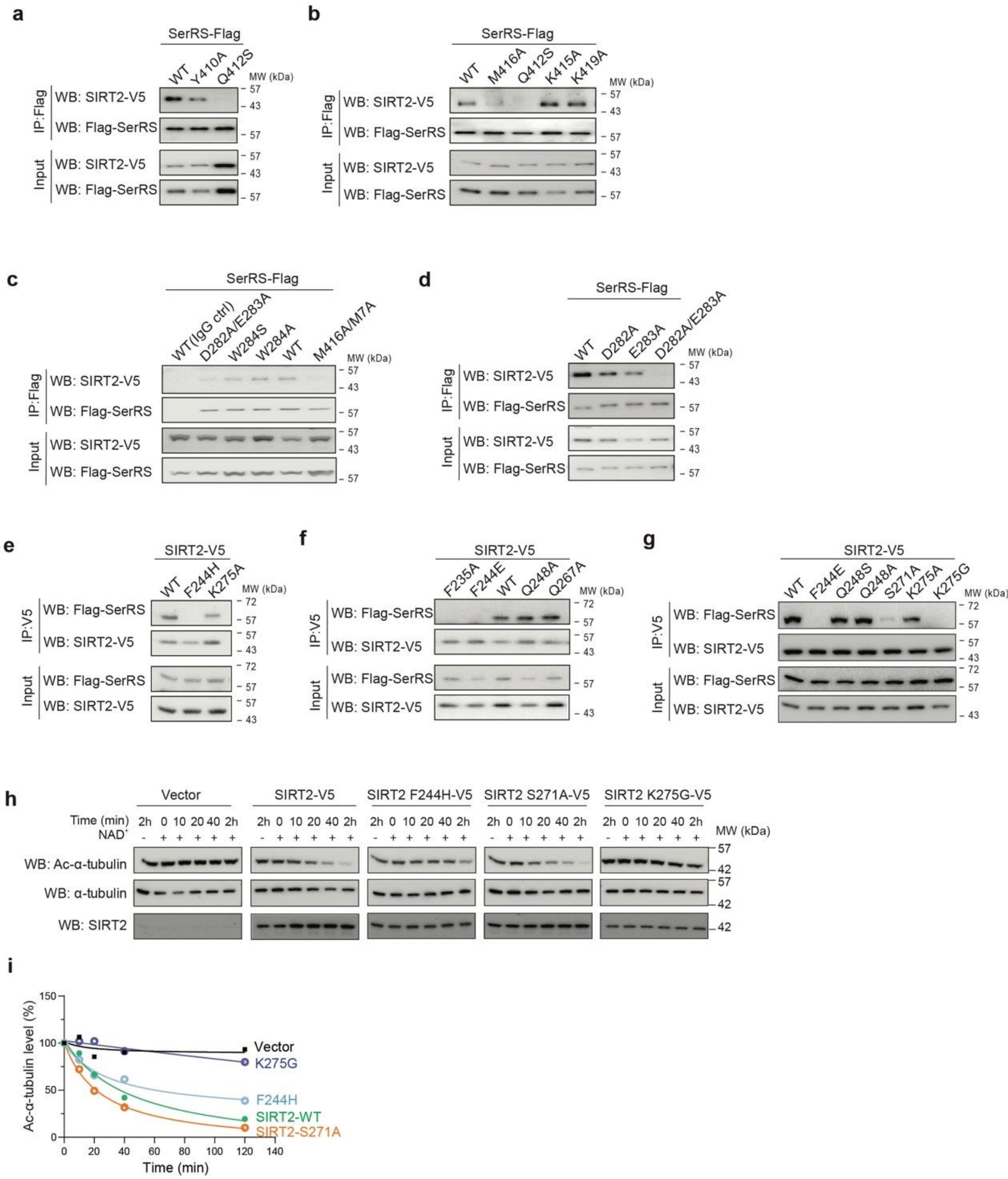

Supplementary Figure 9. Identification of key residues mediating the SerRS–SIRT2 interaction.

(a-g) Mutagenesis and Co-immunoprecipitation (Co-IP) assays identifying key residues in SerRS (a-d) and SIRT2 (e-g) required for the SerRS–SIRT2 interaction (related to Fig.5d-f). (h) Time-course, cell-based deacetylation assays using HEK293 cell lysates overexpressing SIRT2 or SIRT2 mutants. (i) Quantification analysis of acetyl-α-tubulin

levels from (h). Acetyl-α-tubulin levels at each time point were normalized to the 0 min time point, which was defined as 100% for each reaction. Data are presented as mean values from n=2 independent experiments. Source data are provided as a Source Data file.

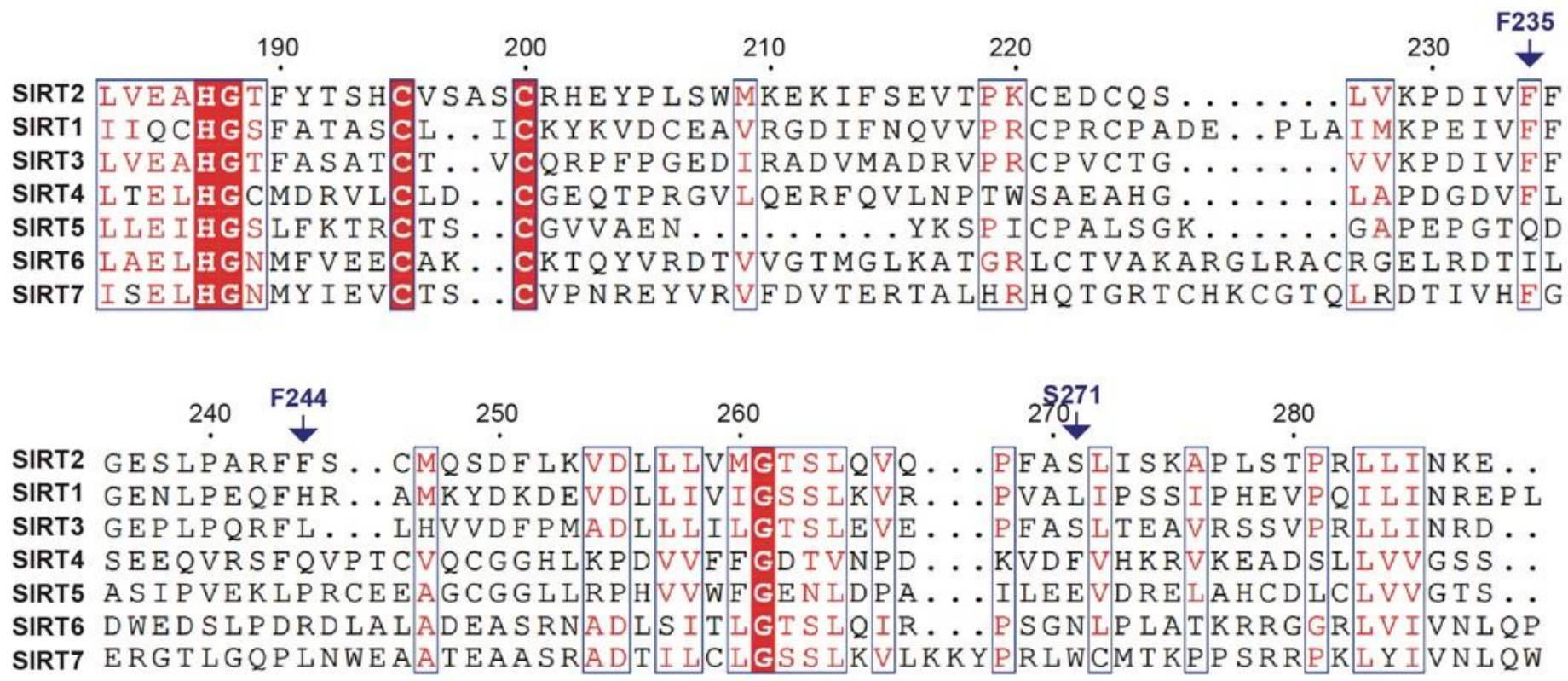

Supplementary Figure 10. Multiple sequence alignment of Sirtuin family proteins. Multiple sequence alignment of SIRT2 with other members of the sirtuin family. Key residues in SIRT2 involved in SerRS binding (F235, F244 and S271) are indicated.

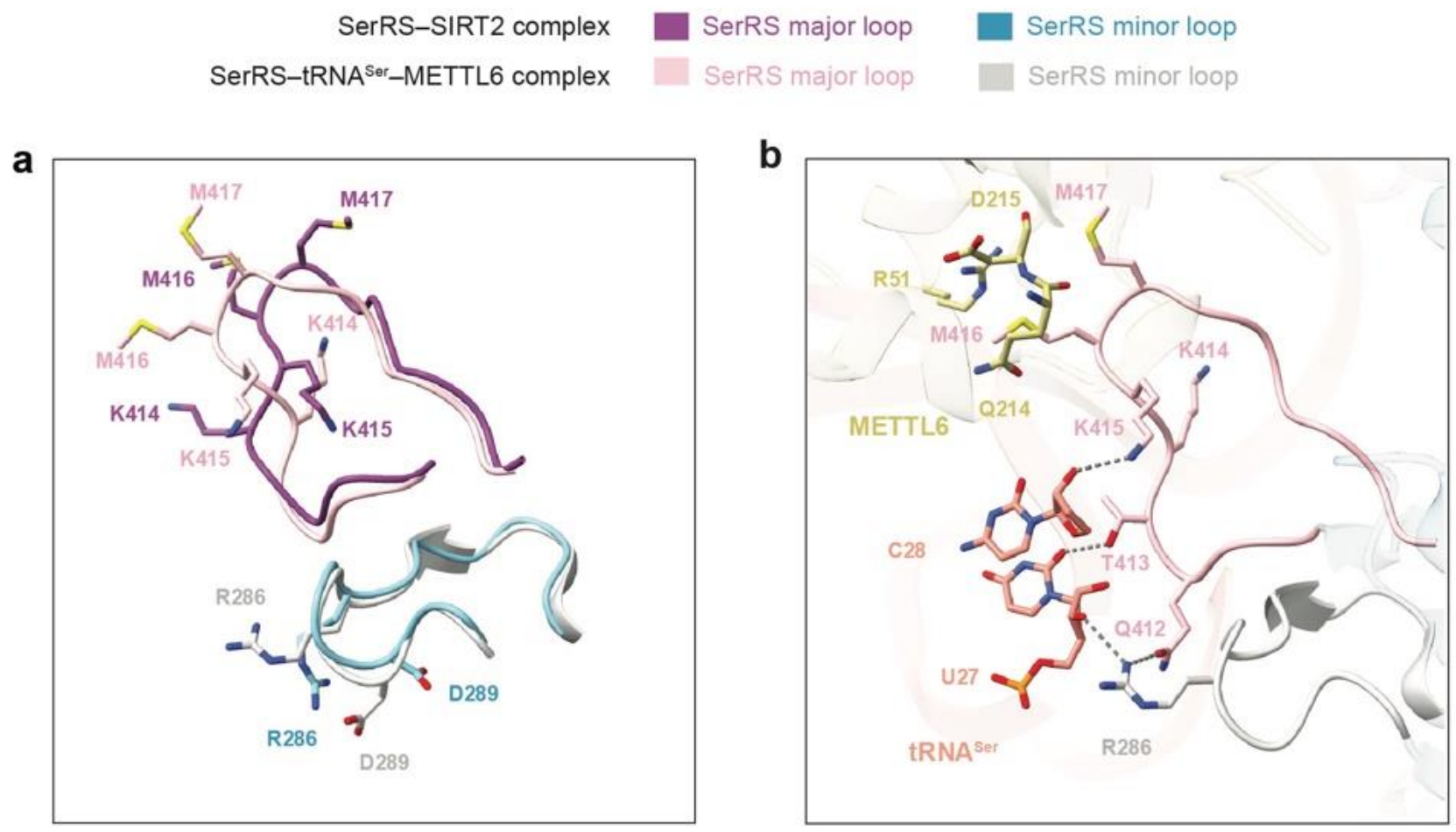

Supplementary Figure 11. Distinct binding modes of SerRS major and minor loops when interacting with SIRT2 or tRNA^{Ser}–METTL6.

(a) Superposition of the SerRS–SIRT2 and SerRS–tRNA^{Ser}–METTL6 complex structures. The major and minor loops of SerRS adopt distinct conformations in each structure, stabilizing alternative binding partners. (b) Close-up view of the interactions mediated by the SerRS major and minor loops in the SerRS–tRNA^{Ser}–METTL6 complex. Residues M416 and M417 in the SerRS major loop participate in METTL6 binding, whereas residues Q412, T413, and K415 are involved in tRNA^{Ser} association; K414 is not involved in these interactions.

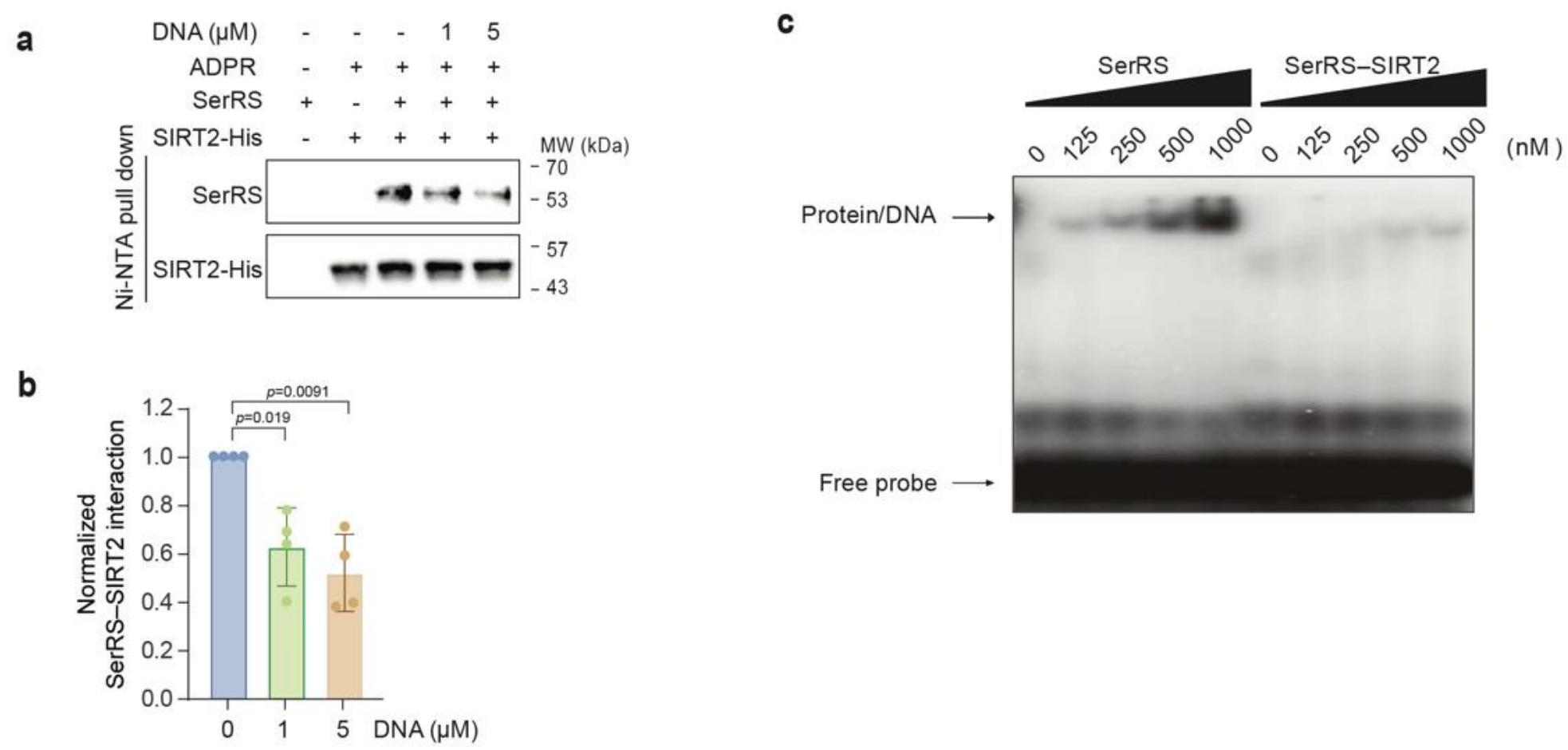

Supplementary Figure 12. **Competition between SIRT2 and VEGFA promoter DNA for SerRS binding.**

(a) Ni-NTA pull-down assay showing that pre-incubation of SerRS with VEGFA promoter DNA suppresses SerRS–SIRT2 interaction in a dose-dependent manner. 200 μM ADPR was used to stabilize SerRS-SIRT2 complex. (b) Quantification of the pull-down assay from (a). Data are presented as mean values ± SD. Welch's two-sided t-test was performed; exact p values are indicated in the figure. n=3 independent experiments. Source data are provided as a Source Data file. (c) Electrophoretic Mobility Shift Assay (EMSA) showing that the SerRS–SIRT2 complex exhibits diminished VEGFA promoter DNA binding capacity.

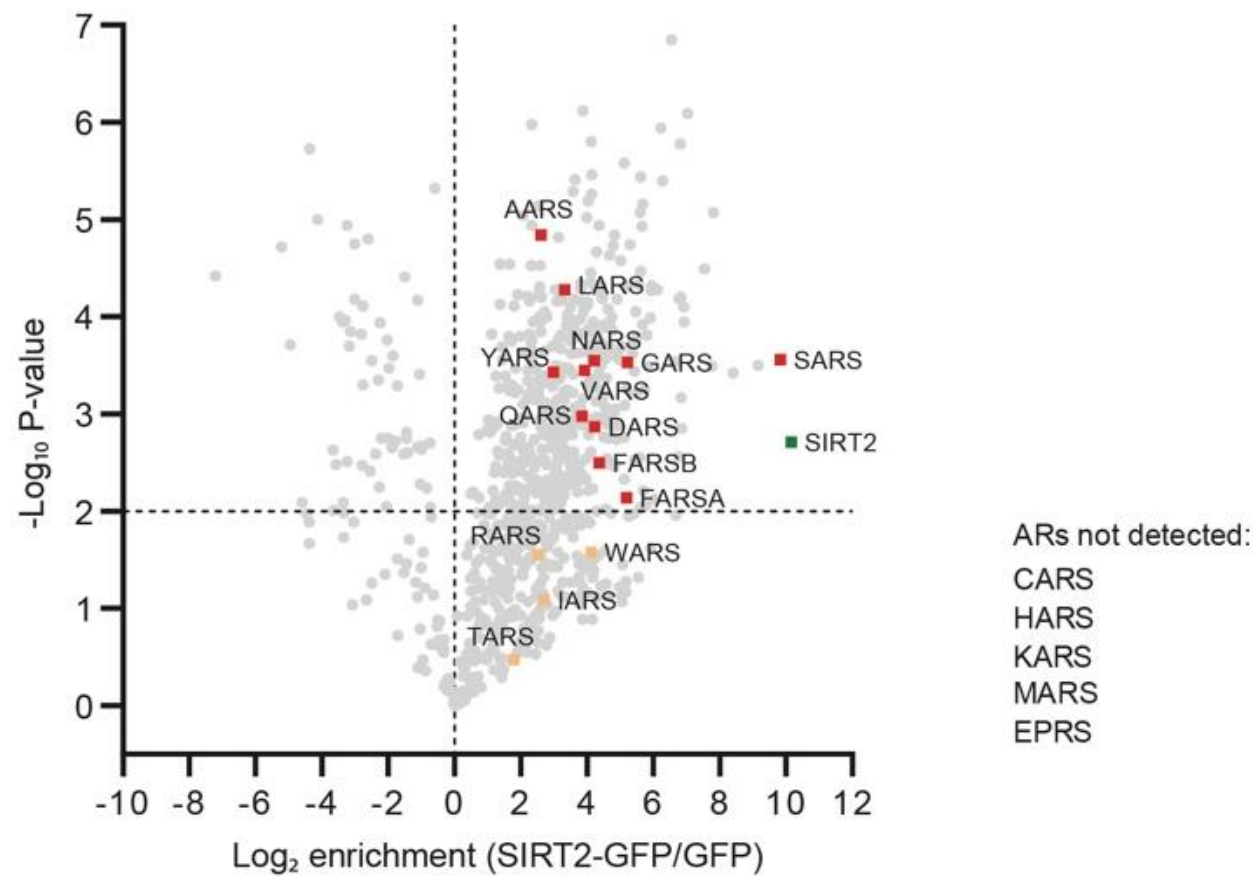

Supplementary Figure 13. Enrichment of tRNA synthetases in the SIRT2 interactome. Interactome data for exogenously expressed SIRT2 were obtained from a previously published study[2]. The volcano plot was re-generated to highlight co-immunoprecipitated tRNA synthetases. tRNA synthetases not detected in the dataset are also listed.

Supplementary References

1. Prisant MG, Williams CJ, Chen VB, Richardson JS, Richardson DC. New Tools in MolProbity Validation: CaBLAM for CryoEM Backbone, UnDowser to Rethink "Waters", and NGL Viewer to Recapture Online 3D Graphics. *Protein Sci*, (2019).

2. Eldridge MJG, Pereira JM, Impens F, Hamon MA. Active nuclear import of the deacetylase Sirtuin-2 is controlled by its C-terminus and importins. *Scientific reports* **10**, 2034 (2020).
