# Supplementary material for: A metabolite-bridged complex between SerRS and SIRT2 couples NAD⁺ metabolism to translation control
Source: Nat Commun. 2026 Jul 8;17:8427. doi: 10.1038/s41467-026-75266-4 (PMC13478228; doi:10.1038/s41467-026-75266-4)
Supplement: Supplementary file 2 [file 41467_2026_75266_MOESM2_ESM.pdf]

nature portfolio

Corresponding author(s): Xiang-Lei Yang

Last updated by author(s): Jun 12, 2026

Reporting Summary

Nature Portfolio wishes to improve the reproducibility of the work that we publish. This form provides structure for consistency and transparency in reporting. For further information on Nature Portfolio policies, see our Editorial Policies and the Editorial Policy Checklist.

Statistics

For all statistical analyses, confirm that the following items are present in the figure legend, table legend, main text, or Methods section.

n/a	Confirmed	
☐	☒	The exact sample size (n) for each experimental group/condition, given as a discrete number and unit of measurement
☐	☒	A statement on whether measurements were taken from distinct samples or whether the same sample was measured repeatedly
☐	☒	The statistical test(s) used AND whether they are one- or two-sided<br>*Only common tests should be described solely by name; describe more complex techniques in the Methods section.*
☒	☐	A description of all covariates tested
☐	☒	A description of any assumptions or corrections, such as tests of normality and adjustment for multiple comparisons
☐	☒	A full description of the statistical parameters including central tendency (e.g. means) or other basic estimates (e.g. regression coefficient) AND variation (e.g. standard deviation) or associated estimates of uncertainty (e.g. confidence intervals)
☐	☒	For null hypothesis testing, the test statistic (e.g. F, t, r) with confidence intervals, effect sizes, degrees of freedom and P value noted<br>*Give P values as exact values whenever suitable.*
☒	☐	For Bayesian analysis, information on the choice of priors and Markov chain Monte Carlo settings
☒	☐	For hierarchical and complex designs, identification of the appropriate level for tests and full reporting of outcomes
☒	☐	Estimates of effect sizes (e.g. Cohen's d, Pearson's r), indicating how they were calculated

Our web collection on statistics for biologists contains articles on many of the points above.

Software and code

Policy information about availability of computer code

Data collection	All data collection software is published and/or publicly and/or commercially available.<br>Cryo-EM data acquisition: Leginon(v3.3)<br>ESI triple quadrupole mass spectrometry acquisition: Agilent MassHunter Acquisition(v12.0).<br>Trypsin digestion and LC–MS/MS data acquisition: Xcalibur (v4.3)<br>Hydrogen–deuterium exchange mass spectrometry data acquisition: Protein Discoverer (v1.3.0.339)
Data analysis	All data analysis software is published and/or publicly and/or commercially available.<br>Cryo-EM data processing: cryoSPARC (v4.1)<br>Model building and refinement: UCSF Chimerav(1.14); ChimeraX (v1.8); Coot (v0.8.9.1); PHENIX (v1.17.1)<br>Multiple sequence alignment: Jalview (v2.11.5.1)<br>Quantification of Western blot images: ImageJ (v2.1.0), Image Lab (v6.0.1, Bio-Rad) or Image Studio (v6.0, LI-COR).<br>Biochemical data analysis and statistics: GraphPad Prism (v10.5.0)<br>ESI triple quadrupole mass spectrometry data analysis: Agilent MassHunter Quantitative Analysis(v12.1)<br>Trypsin digestion and LC–MS/MS data analysis: Mascot (v2.8.0)<br>Hydrogen–deuterium exchange mass spectrometry data analysis:HDExaminer (v2.5.1)

For manuscripts utilizing custom algorithms or software that are central to the research but not yet described in published literature, software must be made available to editors and reviewers. We strongly encourage code deposition in a community repository (e.g. GitHub). See the Nature Portfolio guidelines for submitting code & software for further information.

Data

Policy information about availability of data

All manuscripts must include a data availability statement. This statement should provide the following information, where applicable:

- Accession codes, unique identifiers, or web links for publicly available datasets
- A description of any restrictions on data availability
- For clinical datasets or third party data, please ensure that the statement adheres to our policy

The cryo-EM maps and associated atomic models generated in this study have been deposited to the Electron Microscopy Databank (EMDB) and the Protein Databank (PDB), respectively, with the following EMDB and PDB IDs:
SerRS: SIRT2 (2:2): EMDB: EMD-47105 (https://www.emdataresource.org/EMD-47105) and PDB: 9DPI (https://www.rcsb.org/structure/9DPI);
SerRS: SIRT2 (2:1) EMDB: EMD-47103 (https://www.emdataresource.org/EMD-47103) and PDB: 9DPD (https://www.rcsb.org/structure/9DPD)
The HDX-MS data have been deposited to the ProteomeXchange Consortium via the PRIDE partner repository with the dataset identifier PXD079339.
All data supporting the findings of this study are available within the paper and its Supplementary Information. Source data are provided with this paper. Plasmids, antibodies, and cell lines are available from the corresponding author upon reasonable request.

Research involving human participants, their data, or biological material

Policy information about studies with human participants or human data. See also policy information about sex, gender (identity/presentation), and sexual orientation and race, ethnicity and racism.

Reporting on sex and gender	Not involved
Reporting on race, ethnicity, or other socially relevant groupings	Not involved
Population characteristics	Not involved
Recruitment	Not involved
Ethics oversight	Not involved

Note that full information on the approval of the study protocol must also be provided in the manuscript.

Field-specific reporting

Please select the one below that is the best fit for your research. If you are not sure, read the appropriate sections before making your selection.

☒ Life sciences ☐ Behavioural & social sciences ☐ Ecological, evolutionary & environmental sciences

For a reference copy of the document with all sections, see nature.com/documents/nr-reporting-summary-flat.pdf

Life sciences study design

All studies must disclose on these points even when the disclosure is negative.

Sample size	For cryo-EM experiments, a total of 591,091 particles exhibiting high-resolution secondary structural features were selected for structure determination. For biochemical experiments, sample sizes were chosen based on standard practice in the field, with a minimum of n = 3 independent biological replicates for statistical analysis.
Data exclusions	Through standard cryo-EM procedures, the poor quality or damaged particles were removed to achieve higher resolution<br>This is accepted practice in the cryo-EM field.
Replication	Biochemical and functional experiments were performed with 2–4 independent biological replicates, typically in triplicate. The exact number of replicates is indicated in the corresponding figure legends.
Randomization	Formal randomization was not performed. Samples were allocated into experimental groups based on the experimental design, as the biochemical and cell-based experiments in this study did not require randomization of sample allocation.
Blinding	Blinding was not relevant to this study. All experiments, including cryo-EM data collection and processing, and biochemical assays, were analyzed using objective quantification methods without subjective scoring or group allocation.

Reporting for specific materials, systems and methods

We require information from authors about some types of materials, experimental systems and methods used in many studies. Here, indicate whether each material, system or method listed is relevant to your study. If you are not sure if a list item applies to your research, read the appropriate section before selecting a response.

Materials & experimental systems

n/a	Involved in the study
☐	☒ Antibodies
☐	☒ Eukaryotic cell lines
☒	☐ Palaeontology and archaeology
☒	☐ Animals and other organisms
☒	☐ Clinical data
☒	☐ Dual use research of concern
☒	☐ Plants

Methods

n/a	Involved in the study
☒	☐ ChIP-seq
☒	☐ Flow cytometry
☒	☐ MRI-based neuroimaging

Antibodies

Antibodies used

Custom-made mouse monoclonal anti-human SerRS antibodies (24C4 and 18A8) were raised against human recombinant SerRS and affinity purified. 1μg/mL of 24C4 used for WB, 3 μg of 18A8 was used for each co-IP experiments.
The following commercial antibodies were used:
1. anti-Flag rabbit (Cell Signaling Technology, #14793, D6W5B, 1:2000)
2. anti-Flag mouse (Cell Signaling Technology, #8146, 9A3, 1:2000)
3. anti-V5 rabbit (Cell Signaling Technology, #13202, D3H8Q, 1:2000)
4. anti-V5-mouse (Cell signaling technology, #80076, E9H8O, 1:2000)
5. anti-poly ADP-ribosylation (Cell signaling technology, #89190, D9P7Z, 1:2000)
6. anti-α-tubulin (Cell Signaling Technology, #3873, DM1A, 1:2000)
7.anti-acetylated-α-tubulin (K40Ac) (Cell Signaling Technology, #5335, D20G3, 1:1000)
8. anti-SerRS (Bosterbio, #A00501-1, 1μg/mL)
9. anti-SIRT2 antibody (Cell Signaling Technology, #12650, D4O5O, 1:1000)
10. anti-SIRT2 (Abcam, #ab211033, EPR20411-105, 1:1000)
11. anti-H4 (Active motif, # 91295, 1:1000)
12. anti-acetylated-H4K16 (Active motif, #39167, 1:1000)
13. anti-puromycin (Abcam, #ab315887, EPR27218-173, 1:2000)
14. anti-SIRT2(R&D Systems, MAB4358, 1μg/mL)
15.anti-p-eIF2α (Cell signaling technology, #9721, 1:1000)
16.anti-eIF2α (Cell signaling technology, #9722, 1:1000)
17.anti-ATF4 (Cell signaling technology, #11815, D4B8, 1:1000)
18.anti-p-p70 S6K (Cell signaling technology, #9234, 108D2, 1:1000)
19.anti-p70 S6K (Cell signaling technology, #2708, 49D7, 1:1000)
20.anti-p-4EBP1 (Cell signaling technology, #2855,236B4, 1:1000)
21.anti-4EBP1 (Cell signaling technology, #9644, 53H11, 1:1000)
22.anti-puromycin (Millipore Sigma, #MABE343, 12D10, 1:3000)
23. anti-GAPDH (Cell signaling technology, #2118, 14C10, 1:3000)

Validation

Manufacturer validation information can be found on the respective company websites: Cell Signaling Technology (www.cellsignal.com), Abcam (www.abcam.com), Boster Bio (www.bosterbio.com), Active Motif (www.activemotif.com), R&D Systems (www.rndsystems.com), and Millipore Sigma (www.emdmillipore.com).
1. anti-Flag rabbit (Cell Signaling Technology, #14793, D6W5B)
Species Reactivity: All Species Expected
Validated Applications: WB-Western Blotting, IP-Immunoprecipitation, IHC-Immunohistochemistry, IF-Immunofluorescence, F-Flow Cytometry, ChIP-Chromatin Immunoprecipitation
2. anti-Flag mouse (Cell Signaling Technology, #8146, 9A3)
Species Reactivity: All Species Expected
Validated Applications: WB, IP, IHC IF, F
3. anti-V5 rabbit (Cell Signaling Technology, #13202, D3H8Q)
Species Reactivity: All Species Expected
Validated Applications: WB, IP, IF, F
4. anti-V5-mouse (Cell Signaling Technology, #80076, E9H8O)
Species Reactivity: All Species Expected
Validated Applications: WB, IP, IF, F
5. anti-poly ADP-ribosylation (Cell Signaling Technology, #89190, D9P7Z)
Species Reactivity: All Species Expected
Validated Applications: WB, IF, F
6. anti-SerRS (Bosterbio, #A00501-1)
Species Reactivity: Human, Mouse, Rat
Validated Applications: ELISA, F, IF, ICC, WB
7. Anti-α-tubulin (Cell Signaling Technology, #3873, DM1A)
Species Reactivity: Human, Mouse, Rat, Monkey
Validated Applications: WB, IHC, IF, F
8. Anti-acetylated-α-tubulin (K40Ac) (Cell Signaling Technology, #5335, D20G3)
Species Reactivity: Human, Mouse, Rat, Monkey, Zebrafish
Validated Applications: WB, IP, IF, F

9. Anti-SIRT2 (Cell Signaling Technology, #12650, D4O5O)
Species Reactivity: Human, Mouse, Rat, Monkey
Validated Applications: WB
10. anti-SIRT2 (Abcam, #ab211033, EPR20411-105)
Species Reactivity: Human, Mouse, Rat, Monkey
Validated Applications: WB, IHC, IP
11. anti-H4 (Active motif, # 91295)
Species Reactivity: Human
Validated Applications: CHIP, ELISA, F, IF, ICC, WB
12. anti-acetylated-H4K16 (Active motif, #39167)
Species Reactivity: Budding Yeast, Drosophila, Human, Mouse, Wide Range Predicted
Validated Applications: CHIP, ELISA, F, IF, ICC, WB
13. anti-puromycin (Abcam, #ab315887, EPR27218-173)
Species Reactivity: Unspecified reactive species
Validated Applications: F, IF, ICC, WB, IP, IHC
14. anti-SIRT2(R&D Systems, MAB4358)
Species Reactivity: Human
Validated Applications: WB, IP
15.anti-p-eIF2α (Cell signaling technology, #9721)
Species Reactivity: Human, Mouse, Rat, Monkey
Validated Applications: WB
16.anti-eIF2α (Cell signaling technology, #9722)
Species Reactivity: Human, Mouse, Rat, Monkey
Validated Applications: WB
17.anti-ATF4 (Cell signaling technology, #11815, D4B8)
Species Reactivity: Human, Mouse, Rat
Validated Applications: WB, IP, IF, CHIP
18.anti-p-p70 S6K (Cell signaling technology, #9234, 108D2)
Species Reactivity: Human, Mouse, Rat, Monkey
Validated Applications: WB
19.anti-p70 S6K (Cell signaling technology, #2708, 49D7)
Species Reactivity: Human
Validated Applications: WB
20.anti-p-4EBP1 (Cell signaling technology, #2855,236B4)
Species Reactivity: Human, Mouse, Rat, Monkey
Validated Applications: WB, IF, F, IHC
21.anti-4EBP1 (Cell signaling technology, #9644, 53H11)
Species Reactivity: Human, Mouse, Rat, Monkey
Validated Applications: WB, IP, IF, F, IHC
22.anti-puromycin (Millipore Sigma, #MABE343, 12D10)
Species Reactivity: Human
Validated Applications: FACS, ICC, IF, IHC, IP, WB
23. anti-GAPDH (Cell signaling technology, #2118, 14C10)
Species Reactivity:Human, Mouse, Rat, Monkey, Bovine, Pig
Validated Applications: WB, IF, F, IHC

Eukaryotic cell lines

Policy information about cell lines and Sex and Gender in Research

Cell line source(s)	Human HEK293 cell line (ATCC, #CRL-1573), human HEK293AD cell line (Agilent, #240085)
Authentication	We authenticate our cells annually through the Short Tandem Repeat (STR) Profiling Cell Authentication Service provided by ATCC. Stably transfected cells are authenticated by sequencing following RT-PCR
Mycoplasma contamination	All cell lines tested negative for mycoplasma containimation
Commonly misidentified lines (See ICLAC register)	Not involved

Plants

Seed stocks	Not involved
Novel plant genotypes	Not involved
Authentication	Not involved
