# Supplementary material for: A metabolite-bridged complex between SerRS and SIRT2 couples NAD⁺ metabolism to translation control
Source: Nat Commun. 2026 Jul 8;17:8427. doi: 10.1038/s41467-026-75266-4 (PMC13478228; doi:10.1038/s41467-026-75266-4)
Supplement: Supplementary file 3 [file 41467_2026_75266_MOESM3_ESM.pdf]

nature portfolio

Peer Review File

A metabolite-bridged complex between SerRS and SIRT2 couples NAD$^+$ metabolism to translation control

Corresponding Author: Professor Xiang-Lei Yang

This file contains all reviewer reports in order by version, followed by all author rebuttals in order by version.

Version 0:

Reviewer comments:

Reviewer #1

(Remarks to the Author)
Zhang and colleagues present a comprehensive analysis of the interaction between SerRS and SirT2 utilising primarily structural and biochemical methodologies. The analysis is well carried out and has the potential to elucidate a new regulatory mechanism for tRNA charging/protein biogenesis. That said the manuscript could be strengthened by providing more evidence for in vivo relevance and mentioned below in detail are evolutionary and experimental suggestions do to so.

Critique
1) The phylogenetic analysis is intriguing but lacks depth. It would be interesting to know whether the described interaction is a feature of the Animalia lineage. For this the phylogenetic analysis should be extended to more basal phyla such as Cnidaria, Porifera and Placozoa and the analysis of SerRS to animal species that have lost SirT2 (so far only C. elegans included). Distribution of SirT2 within different kingdoms/phyla is well documented within the literature and should support this analysis. While the absence of a similar co-evolution in fungi and plants could also be a bit better supported a clear focus on the Animalia kingdom would be more important.
2) In addition to the phylogenetic analysis (see (1)), experimental evidence for an interaction within a distant/basal species would also be desirable.
3) The authors propose that the SirT2:SerRS interaction is stimulated under stress conditions that increase the cellular levels of OAADPR/ADPR. From the presented data it would be follow that the Ser-tRNASer /tRNASer ratio would shift towards the unmodified tRNA. As the authors mentioned induced DNA damage leads to NAD+ consumption by PARP enzymes and the production of ADPR. Therefore, it would be good if the authors could test their hypothesis following induced DNA damage (e.g. Co-IP and/or charge ratio determination).
4) The analysis of SerRS K414 as potential acetylation acceptor site is insufficient. Recombinant proteins expressed in E. coli are not expected to carry relevant modifications due to the different acetyl-transferase/hydrolase enzymes present. Therefore, the analysis should be performed on mammalian cell culture-derived SerRS either endogenous or over-expressed. The analysis could also include potentially relevant culture conditions (e.g. high/low glucose and DNA damage stress) as these can impact acetylation pattern.
5) It would be very informative in the context of the study if the authors could present quantitative binding data for the SerRS:SirT2, SerRS:SirT2:ADPR, and SerRS:tRNA interactions and set these into context with available data of protein/tRNA abundance and in vivo potential of the described interaction.

Reviewer #2

(Remarks to the Author)
In the manuscript by Zhang et al., the authors explore the structural basis of the interaction between the seryl-tRNA synthetase (SerRS) and the NAD+-dependent deacetylase SIRT2. The authors had described previously the interaction (Shi et al., eLife 2014) in the nucleus, where SerRS was shown to recruit SIRT2 to specific genes and deacetylate histones. The authors solve the structure of SerRS bound to SIRT2, which involves an ADPribose molecule as a mediator of the interaction. The authors identify a binding-like motif in SerRS that interacts with the substrate binding domain of SIRT2 and inhibits its catalytic activity. In contrast, the binding of SIRT2 interferes with the ability of SerRs to generate tRNAs, and therefore its role in translation.

The work presents several interesting findings that, if properly developed, could appeal to a broad audience. For example, the involvement of ADPR in SIRT2 binding to proteins or the functional implications of SerRS-mediated regulation of translation by SIRT2. However, the manuscript falls short of fully exploring these aspects and the authors have not provided any evidence to demonstrate the functional relevance of these in vitro studies. In its current form, the study is quite descriptive and primarily focuses on structural data, making it more suitable for a specialized journal.

Some specific Major issues:

1) The involvement of ADPR in the binding is intriguing and potentially very relevant but it raises many unaddressed questions:

a) The importance of ADPR in binding is demonstrated only in vitro using bacterially expressed proteins, with no evidence provided to confirm its relevance in vivo. The authors also present competition data suggesting that neither OAADPR nor NAD+ are involved in this binding. Can the authors rule out the possibility that, in vivo, the detected ADPR is actually OAADPR, the product of the Sirtuin deacetylation reaction, which may undergo deacetylation during the binding process?

b) Linked to this previous point. The authors show that the binding does not require deacetylation of SeRS by SIRT2, but the key question here is whether this ADPR-dependent binding is triggered by SIRT2-mediated deacetylation. For instance, could it be triggered by SIRT2 auto-deacetylation? Can SIRT2 previously incubated with NAD+ bind more efficiently to SeRS?
c) The model proposed by the authors is confusing. Do the authors imply that SerRS binds to ADPR and then bind to SIRT2? Would that mean that SeRS is an ADPR-binding protein? or ADPR is already bound to SIRT2 by the time SeRS interacts with it?

d) The author's claim that SIRT2 inhibits translation through SerRS is an overstatement that needs to be demonstrated. The authors only show a decreased rate of Ser-tRNA vs Tyr-tRNA.

2) The authors' major claim here is that SerRS inhibits SIRT2 activity but in the previous work (Shi et al., eLife 2014), they proposed that SerRS in fact induces SIRT2 activity by recruiting it to specific DNA regions. How do the authors reconcile both models? does this mean that in the first case, the binding mechanism is not the same?

3) What is the primary purpose of the functional interplay between these two factors? The negative regulation of translation? Is SIRT2/SerRS binding enhanced in cells under stress conditions or metabolic alterations (e.g., calorie restriction)? The authors should provide evidence to address this question.

4) Is this ADPR-mediated SIRT2 interaction a SerRS-specific mechanism? Or is a general mechanism involving binding to SIRT2? Is the SerRS motif involved in this interaction conserved in other proteins?

Reviewer #3

(Remarks to the Author)
In this work, Zhang and colleagues aim to structurally and functionally characterize the complex between the human cytosolic seryl-tRNA synthetase (SerRS) and sirtuin-2 (SIRT2), which is a rather vaguely studied NAD+-dependent deacetylase.
The authors initially detected the specific interaction between these two proteins in human cells, which they described in an eLife paper published in 2014. Here, they purify the co-expressed complex and they determine the structure of the complex by single particle cryo-EM at a nominal resolution of 3.2 Å. The structure shows how the two proteins interact with each other and that a co-purified ADPR molecule is needed for complex formation. Of note, they obtain the complex at different stoichiometries and they use mutational analyses (e.g. interface, APPR binding site and catalytic centers) to validate their structural observations in vitro and in human cell lines. Furthermore, they show that the binding to each other inhibits the catalytic activity of each proteins, which indicates a co-regulation that is also linked to the metabolic state of the cell via ADPR.
The manuscript is well written and the illustration nicely highlight the main observations. In my opinion the cryo-EM analyses needs to be carried out with much more care and I could imagine that the actually collected dataset might allow to arrive at a much better reconstruction. The manuscript builds on previous cellular observations (eLife 2014) and at the moment I am very confused that several observations stand in stark contrast to the previous conclusions. In addition, the authors do not address the open question whether the DNA-binding capacity of SerRS in connection with the anticipated histone deacetylation activity of SIRT2 could be mainly responsible for the cellular effects. Here the authors focus on the cytoplasmatic aspects, but as additional structure-guided assays in human cells are missing the conclusions of the two manuscript do often contradict each other. Despite my interest in the regulatory function of this rather unexpected protein complex, I am not convinced that the work is suitable for publication in Nature Communication in its current form.

Major issues
1. In the previous paper the authors state "SerRS interaction promotes SIRT2 deacetylase activity". Here they state on page 8 line 211-213 "Indeed, SerRS inhibits the deacetylase activity of SIRT2 in a concentration dependent manner (Fig. 3b)." The authors do not interpret these obviously contradicting conclusions or discuss potential differences between the function of the complex in the cytoplasm or in the nucleus. The authors analyse the effect of overexpressing/knocking down of SerRS of the acetylation of Lys40 alpha-tubulin, but they do not analyse the effect on histone acetylation. Did the authors perform

celllar fractionation experiments to see whether the complex forms in both cellular compartments or if the availability of ADPR spatially restricts complex formation?
2. Along these lines, the author state that “Conversely, SIRT2 binding disrupts the aminoacylation activity of SerRS by directly competing with and allosterically regulating its interaction with tRNASer,…”. In the previously published work they conclude that the complex formation affects the DNA binding capacity of SerRS. Do the authors think that this is not case anymore, did the authors test the effect of complex formation on DNA binding and would specific mutations that inhibit complex formation bind stronger to DNA in cells?
3. There are several critical issues with the presented cryo-EM data and the performed processing pipeline
a. The presented raw micrograph is not very informative – it needs to magnified, it might be good to also show a denoised version of it and the scale bar should be the size of the expected particles…please also use boxes to highlight the picked/selected particles
b. I missing Table 1 that summarizes all cryo-EM collection and refinement parameters
c. The Global FSC curves do not reach 0 – indicating that the authors did not remove duplicates. Considering that the data was apparently collected from rather overcrowded holes, I would be very curious about the number of duplicates. A large number of duplicated “particles” would explain that the nominal resolution is artificially overstated and that the map quality does not fit the stated resolution limits.
d. Please show orientation distribution plots (after removing duplicates)
e. Could the authors explain the reasoning behind the local refinement step – the mask in this step does not remove/exclude the particles that don’t fulfil the requirements. A real focused 3D classification protocol needs to be used. In general, I would suggest to process the dataset in parallel in Relion, which allows to perform true 3D classification steps.
f. Please highlight the local resolution around the ADPR ligand
g. Could the authors comment on the chosen box size – it appears too small for the size of the particles
h. Did the authors use RBMC implemented in the latest CryoSPARC version to further improve their reconstructions
4. The experiments described in the lower paragraph on page 9 are interesting, but they only indirectly address the question if SIRT2 has an effect on the activity of SerRS. I assume the authors could test which effect increasing amounts of SIRT2 (in presence or absence of ADPR) have on the in vitro aminoacylation activity of SerRS. The authors use the preformed complex to compare the activity, but after they showed that they can reconstitute the complex in vitro in the presence of ADPR (Fig. 2f) this would be the most direct way to answer the raised question.
5. In the SUnSET assays it would important to clarify the levels of ADPR in cells.
6. I don’t see dramatic reduction of SerRS levels in the si-SerRS sample in Figure 4h – could the authors quantify the knock down efficiency and comment on it?
7. Did the authors test the interaction of SIRT1 carrying Phe at the equivalent position as F244 in SIRT2? Would this SIRT1 mutant start interacting with SerRS as well?
Minor
• Please use “KDa” instead of “KD”
• Page 4 line 112 – “had an estimated molecular weight of 213.4 kD” – either provide error estimates or use “approximately 215 KDa” – gel filtration column is that exact. The conclusions of the stoichiometry are still valid.
• Page 5 ine 121/122 – “Both 2:2 and 2:1 complexes were selected for ab initio Non-Uniform (NU)-refinement with C2 symmetry, …” Why would you choose C2 symmetry for the 2:1 complex? Extended data Fig. 1 shows that you did not use symmetry – please clarify.
• The authors use the structure tRNASec bound to SerRS, but due the very specific shape of tRNASec, I would suggest to use either PDB ID 8FFY of the mitochondrial tRNASer or PDB ID 8P7B of SerRS-tRNASer from the recently determined structure of the MTTL6-SerRS complex.

Reviewer #4

(Remarks to the Author)
Zhang et al. demonstrated the physical interaction between dimeric SerRS and SIRT2 by determining the cryo-EM structures of the 2:2 and 2:1 complexes. The structures reveal that ADPR is a key metabolite mediating protein-protein interactions, leading to the inactivation of both SIRT2 and SerRS. This complex provides a compelling model in which the cell's energy status, represented by the accumulation of ADPR, contributes to the regulation of protein synthesis and the homeostasis of serine, a central node in metabolic pathways. The authors have accomplished remarkable work in solving the structure of the SIRT2-SerRS complex. However, my primary concern lies in the weakness of their biophysical analyses. The key finding, namely the ADPR-dependent complex formation between SerRS and SIRT2, requires a more thorough quantitative examination. After addressing the following issues, I would recommend this manuscript for publication in Nature Communications.

Major Points

1. In line 181, the authors present BLI data showing complex formation with ADPR and NAD+. Can BLI measure the dissociation constants (kd) between SerRS and SIRT2 in the presence of ligands such as ADPR or NAD+ at physiological concentrations?

2. In Figure 3c, it appears that SIRT2 activity is significantly reduced (by approximately 50%?) by SerRS, even when [ADPR] = 0. Furthermore, deacetylation activity does not change substantially when [ADPR] is increased up to 400 μM. In Figures 4c and 4d, SIRT2 and SerRS appear to form a complex in the absence of ADPR, with a more favorable interaction (by more than 4-fold?) when ADPR is present, though the specific concentration is not mentioned. It would be beneficial to

demonstrate the correlation between complex formation and deacetylation activity in a more quantitative manner. I suggest completing titration curves for in vitro activity and complex formation at fixed protein concentrations while varying [ADPR].

Minor Points

3. The authors provide a structure-based explanation of the importance of F235 in SIRT2's interaction with SerRS via a cation-π interaction with K414. However, what about F244 and S271 in SIRT2? What insights does the structure offer about their specific molecular interactions with SerRS?

4. Similarly, the key residues of SerRS at the protein interface (e.g., Q412, K414, M416, D282, and E283) should be described in greater detail, including the molecular interactions in which they are involved.

5. Line 350–351: "The cellular level of serine is the lowest among all proteinogenic amino acids" (Reference 44). This is surprising—shouldn't it be Trp or Cys? I encourage the authors to verify this statement with a more recent reference.

Version 1:

Reviewer comments:

Reviewer #1

(Remarks to the Author)
In their revised manuscript Zhang and colleagues have address the concerns I raised regarding the original version. The new data support and clarify their original findings and strengthen the proposed physiological role for the SerRS:SirT2 interaction. While experimental data for the conservation of the interaction would be desirable, I feel that this is not so important as to further delay the publication of this work.

Reviewer #2

(Remarks to the Author)
The authors have addressed the majority of my previous concerns satisfactorily, and the new experiments included in the revised manuscript significantly strengthen the study. In particular, the additional biochemical analyses and the new cellular experiments linking oxidative stress, PARP activation, and the SerRS–SIRT2 interaction improve the biological relevance of the work and clarify several mechanistic aspects of the proposed model. Overall, the revised manuscript represents a substantial improvement over the original version.
However, there are still two remaining points that I believe should be fully addressed or clarified before the manuscript can be considered for publication:

First, the authors attempt to reconcile the conclusions of the present study with their previous work (Shi et al., eLife 2014), where SerRS was reported to activate SIRT2 activity, whereas in the current manuscript SerRS is shown to inhibit SIRT2. The explanation provided by the authors is that the previous study used a GST-tagged SIRT2, whereas the present work uses an untagged protein. This explanation, which is now mentioned in the Discussion section, is reasonable but remains speculative, as it has not been experimentally demonstrated. If this difference indeed explains the apparent discrepancy between the two studies, the authors should demonstrate it experimentally.

Second, the authors present new observations suggesting that oxidative stress promotes the formation of the SerRS–SIRT2 complex through a PARP-dependent pathway, presumably via increased ADPR production. These results are interesting and potentially important, as they link cellular stress signaling with the regulation of SIRT2 activity. However, the manuscript still does not formally demonstrate that SerRS-mediated inhibition of SIRT2 under these conditions results in a direct restraint of translation. While the authors provide data on Ser-tRNA charging and SUnSET assays that are consistent with reduced protein synthesis, the causal relationship between SerRS–SIRT2 complex formation and translational inhibition remains somewhat indirect. The authors should provide at least minimal evidence supporting this mechanistic link.

Reviewer #3

(Remarks to the Author)
I have carefully reviewed the revised version of the manuscript “A metabolite-bridged complex between SerRS and SIRT2 couples NAD^+ metabolism to translation control”.
I am genuinely impressed by the newly presented work and the clarity of the novel results. I also appreciate that authors have carefully revised their cryo-EM work flow, which is now at a state-of-the-art level – I am still not sure how the authors have overlooked numerous weaknesses in the data processing routines shown in the original submission.
I believe that the novelty of results and broad interest justifies publication in Nature Communications after the data in reviewer figures R2 and R3 are included in the actual manuscript and a few minor issues are resolved.
Major issue
1. Please include the very interesting data shown in Reviewer figures R2 and R3 in the manuscript – I believe it is crucial to

show these results to interested reader to comprehend the link between the previous and the current work.
Minor
• I simply don't see enough connection to highlight "translation control" in the title – maybe the new data would rather suggest a link to "oxidative stress response"
• I would suggest to mention the overall resolution in the abstract (3.9 Å) or leave the resolution out at all.
• Table 1 – I think "X" should be replaced by "×"

Reviewer #4

(Remarks to the Author)
1. Although the authors have performed more quantitative analyses of ligand-dependent SIRT2–SerRS complex formation (Figs. 2e and 2h), they did not provide a direct answer to my previous question: why can these proteins still form a complex in the absence of ADPR (Figs. 4c,d), which appears to contradict the results in Fig. 1c and Fig. 2h? These inconsistencies should be addressed.

2. The inhibition of deacetylation at [ADPR] = 0 in Fig. 3c should be described in the text to avoid confusion, particularly regarding whether non-enzymatic conversion of OAADPR to ADPR during the deacetylation reaction may contribute to the observed formation of the protein complex. It is also somewhat surprising that the addition of 200 and 400 μM ADPR has only a limited effect. Would pre-incubation of SIRT2, SerRS, and ADPR prior to substrate addition influence complex formation or enzymatic activity?
3. The intracellular concentration of NAD^+ in E. coli is estimated to be 2–3 mM, whereas that of ADPR is likely to be substantially lower. Given these conditions, why is ADPR co-purified with the protein complex instead of NAD^+, considering that the binding equilibria would be expected to favor NAD^+ at such concentrations?

Version 2:

Reviewer comments:

Reviewer #2

(Remarks to the Author)
The authors have made a substantial effort to address the previous comments, and the revised manuscript has been significantly improved. In particular, the additional experiments strengthen the biological relevance of the study and provide further support for the proposed model.

While I still believe that some mechanistic aspects would benefit from a more formal demonstration — particularly regarding the direct causal link between the SerRS–SIRT2 interaction and translational repression, as well as the explanation for the apparent discrepancy with the authors' previous work, I appreciate that the authors have openly acknowledged these limitations and have provided additional evidence supporting their hypothesis.

Overall, I believe the current data are consistent with the proposed model, even if some aspects will likely require further investigation in future studies. I recognize the considerable effort made by the authors during revision and I have no further concerns.

Reviewer #4

(Remarks to the Author)
The authors have fully addressed my comments. I recommend accepting this revised manuscript for publication in Nature Communications.

Open Access This Peer Review File is licensed under a Creative Commons Attribution 4.0 International License, which permits use, sharing, adaptation, distribution and reproduction in any medium or format, as long as you give appropriate credit to the original author(s) and the source, provide a link to the Creative Commons license, and indicate if changes were made.
In cases where reviewers are anonymous, credit should be given to 'Anonymous Referee' and the source.
The images or other third party material in this Peer Review File are included in the article's Creative Commons license, unless indicated otherwise in a credit line to the material. If material is not included in the article's Creative Commons license and your intended use is not permitted by statutory regulation or exceeds the permitted use, you will need to obtain permission directly from the copyright holder.
To view a copy of this license, visit https://creativecommons.org/licenses/by/4.0/

Response to reviewers

We thank the reviewers for their careful evaluation and constructive comments. In response, we performed extensive new experiments and analyses that address concerns regarding biological relevance, mechanistic clarity, and quantitative rigor. Specifically, we resolved the apparent discrepancy with our prior work by defining context-dependent outcomes of the SerRS–SIRT2 interaction and proposing a unifying model supported by new biochemical, cellular, and structural data. We expanded the phylogenetic analysis to establish the evolutionary emergence of the SerRS–SIRT2 interaction in vertebrates, quantified the ADPR-dependent binding affinity between SerRS and SIRT2 using mass photometry, and demonstrated that oxidative stress enhances complex formation in cells through a PARP1-mediated pathway. In addition, we clarified the role of ADPR versus OAADPR, defined the concentration range relevant to intracellular conditions, and established reciprocal inhibition of SerRS and SIRT2 as a stress-responsive regulatory mechanism. Finally, we substantially improved the cryo-EM data processing and validation in response to technical concerns. Below, we provide a detailed point-by-point response to each reviewer.

Reviewers' comments:

Reviewer #1 (Remarks to the Author):

Zhang and colleagues present a comprehensive analysis of the interaction between SerRS and SirT2 utilising primarily structural and biochemical methodologies. The analysis is well carried out and has the potential to elucidate a new regulatory mechanism for tRNA charging/protein biogenesis. That said the manuscript could be strengthened by providing more evidence for in vivo relevance and mentioned below in detail are evolutionary and experimental suggestions do to so.

Critique
1) The phylogenetic analysis is intriguing but lacks depth. It would be interesting to know whether the described interaction is a feature of the Animalia lineage. For this the phylogenetic analysis should be extended to more basal phyla such as Cnidaria, Porifera and Placozoa and the analysis of SerRS to animal species that have lost SirT2 (so far only C. elegans included). Distribution of SirT2 within different kingdoms/phyla is well documented within the literature and should support this analysis. While the absence of a similar co-evolution in fungi and plants could also be a bit better supported a clear focus on the Animalia kingdom would be more important.

We thank the reviewer for this insightful comment. As suggested, we performed a comprehensive phylogenetic analysis, extending the dataset to include bacteria, archaea, fungi, plants, early-branching metazoans (cnidarians, placozoans, poriferans, and insects), non-vertebrates, and vertebrates. These expanded data strengthen the conclusion that the SerRS–SIRT2 interaction emerges specifically during the transition from non-vertebrates to vertebrates. Accordingly, we have updated Fig. 5g and revised the corresponding text in a new section subtitled “SerRS–SIRT2 interaction is likely conserved in vertebrates”.

2) In addition to the phylogenetic analysis (see (1)), experimental evidence for an interaction within a distant/basal species would also be desirable.

We agree that experimental validation of the SerRS–SIRT2 interaction in a distant or basal species would be of interest. However, based on our expanded phylogenetic analysis, even though the key SerRS residues responsible for interaction are conserved in the animal kingdom,

SIRT2 is either absent or lacks a key interaction residue (i.e., F244 in human SIRT2) in non-vertebrate lineages (new Fig. 5g), arguing against the presence of a comparable interaction in basal species.

3) The authors propose that the SirT2:SerRS interaction is stimulated under stress conditions that increase the cellular levels of OAADPR/ADPR. From the presented data it would be follow that the Ser-tRNASer /tRNASer ratio would shift towards the unmodified tRNA. As the authors mentioned induced DNA damage leads to NAD+ consumption by PARP enzymes and the production of ADPR. Therefore, it would be good if the authors could test their hypothesis following induced DNA damage (e.g. Co-IP and/or charge ratio determination).

Both reviewers #1 and #2 brought up the importance of testing our hypothesis and confirming the biological relevance of the SerRS–SIRT2 interaction in cells. For this purpose, we performed new experiments (new Fig. 6). Specifically, we treated cells with H_2O_2, which activated PARP1 and led to a marked increase in PAR polymer levels, followed by time-dependent PAR clearance, a process expected to elevate intracellular free ADPR (Fig. 6a). In parallel, we observed a significant increase in SerRS–SIRT2 interaction following PAR degradation (Fig. 6b-c). Importantly, pretreatment with the PARP1 inhibitor PJ34 attenuated both PAR accumulation and the stress-induced enhancement of the SerRS–SIRT2 interaction. Therefore, as we hypothesized, oxidative stress enhances the SerRS–SIRT2 complex formation via PARP1-mediated ADPR production. These findings confirm the biological relevance of the SerRS–SIRT2 interaction in cells and establish the SerRS–SIRT2 complex as a stress-sensitive hub that links metabolic and translational control (new Fig. 7). Based on these new results, we added a new section entitled "Oxidative stress promotes SerRS–SIRT2 interaction" and updated the Abstract and the Discussion sections of the manuscript.

4) The analysis of SerRS K414 as potential acetylation acceptor site is insufficient. Recombinant proteins expressed in E. coli are not expected to carry relevant modifications due to the different acetyl-transferase/hydrolase enzymes present. Therefore, the analysis should be performed on mammalian cell culture-derived SerRS either endogenous or over-expressed. The analysis could also include potentially relevant culture conditions (e.g. high/low glucose and DNA damage stress) as these can impact acetylation pattern.

We thank the reviewer for this important point and fully agree that acetylation analysis based on E. coli–expressed proteins has inherent limitations. We indeed attempted to detect SerRS acetylation, including at K414, using mammalian cell–derived SerRS in HEK293AD cells under both basal conditions and following H_2O_2 treatment. Despite multiple independent efforts, we were unable to reliably identify the peptide containing K414 by mass spectrometry in either endogenous or overexpressed SerRS (Figure R1), precluding a definitive assessment of its acetylation status in mammalian cells.

While we cannot exclude the possibility that K414 may be acetylated and deacetylated under specific cellular conditions, several lines of evidence indicate that the SerRS–SIRT2 interaction does not depend on K414 acetylation. First, the acetylation-mimetic mutant SerRS^{K414Q} fails to interact with SIRT2 (Fig. 2b). Second, SerRS expressed in E. coli, which lacks detectable K414 acetylation (Extended Data Fig. 5a–b) and shows no evidence of deacetylation by SIRT2 (Extended Data Fig. 6), nevertheless robustly interacts with SIRT2 (updated Fig. 2e–g) and inhibits its deacetylase activity (Fig. 3b–c). Together, these results support our conclusion that SerRS–SIRT2 interaction is independent of K414 acetylation.

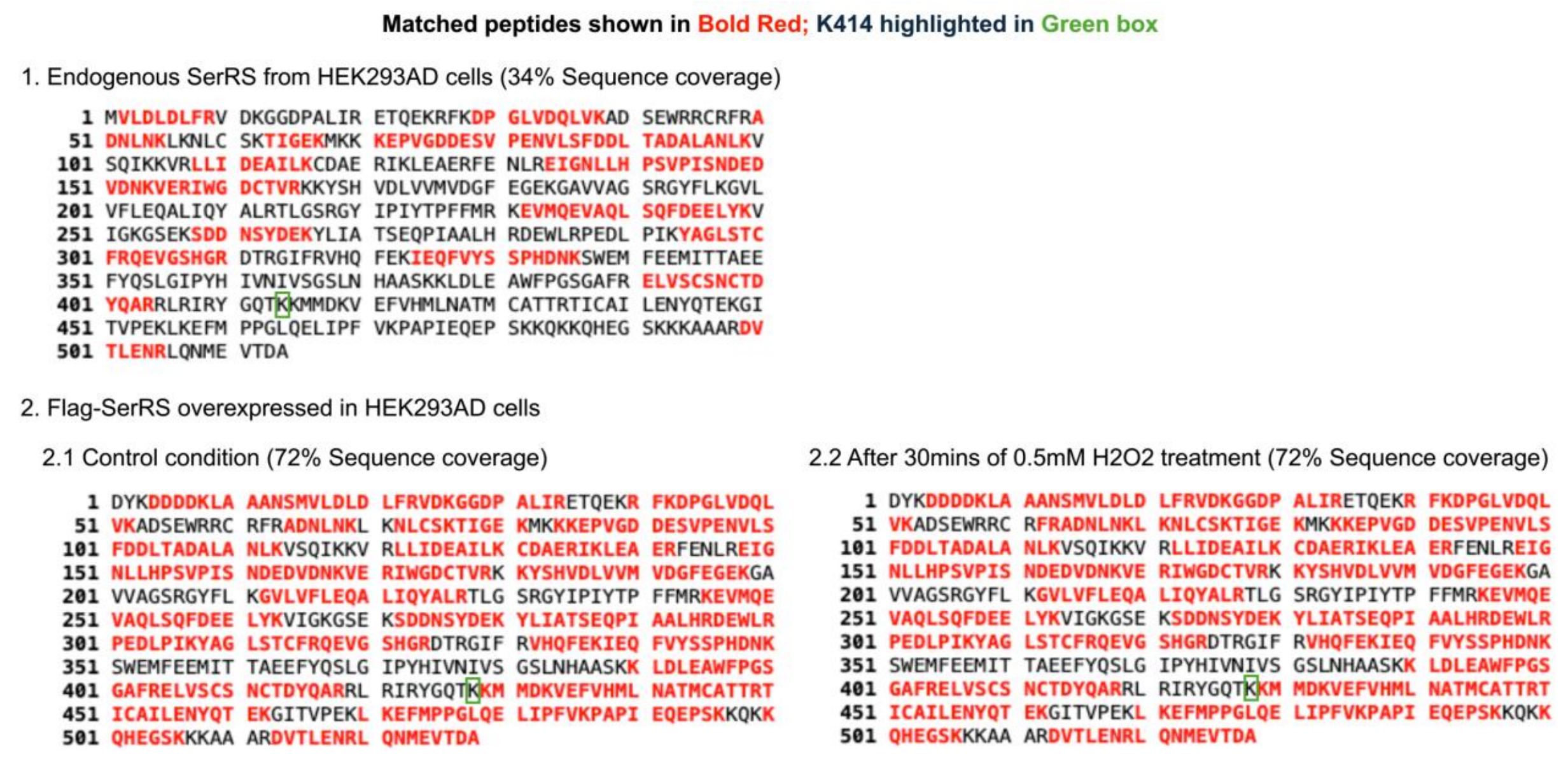

Figure R1. Sequence coverage of mammalian cell–derived SerRS. Top: Sequence coverage of endogenous SerRS derived from HEK293AD cells; Bottom: Sequence coverage of exogenous Flag-SerRS (left: control condition, right: H_2O_2 treated) derived from HEK293AD cells. K414 was highlighted with a green box.

5) It would be very informative in the context of the study if the authors could present quantitative binding data for the SerRS:SirT2, SerRS:SirT2:ADPR, and SerRS:tRNA interactions and set these into context with available data of protein/tRNA abundance and in vivo potential of the described interaction.

As suggested, we now have determined the dissociation constants (K_D) of the SerRS–SIRT2 complex in the absence or presence of NAD^+ or ADPR with mass photometry (new Fig. 2h). And to place our quantitative binding data in a cellular context, we also compared our measurements with reported intracellular concentrations of ADPR, NAD^+, and tRNA, as well as known affinities of SerRS for tRNA.

All measurements were performed with 25 nM SerRS dimer and 200 nM SIRT2. In the absence of ligand, no SerRS–SIRT2 complex was detected, indicating a weak basal interaction with an estimated K_D > 10 µM. Addition of NAD^+ supported detectable complex formation only at high concentrations, with binding observed at 400 µM NAD^+ (K_D = 0.81 ± 0.25 µM). Notably, ADPR enhanced SerRS–SIRT2 complex formation more effectively, with detectable binding observed at ADPR concentrations as low as 16 µM (K_D = 1.06 ± 0.13 µM) and substantially tighter affinity at higher ADPR concentrations (K_D = 0.33 ± 0.02 µM at 400 µM).

Reported intracellular NAD^+ concentrations vary widely depending on measurement methods and subcellular compartments. Genetically encoded biosensors that report free, accessible NAD^+ in live cells typically detect tens to low hundreds of micromolar NAD^+ in the cytosol and nucleus (e.g., ~70–120 µM in U2OS cells and ~40–70 µM in other mammalian cell lines)[1], whereas biochemical assays measuring total cellular NAD^+ often estimate higher, sub-millimolar concentrations (≈0.2–0.5 mM)[2]. Although direct measurements of free intracellular ADP-ribose

(ADPR) are more limited, HPLC-based analyses have reported basal ADPR levels in the tens micromolar range in mammalian lymphoid cells (~40–70 μM)[3]. Together, these comparisons indicate that the ADPR-dependent enhancement of SerRS–SIRT2 interaction observed *in vitro* occurs within a concentration range that is compatible with intracellular ADPR levels.

We were not able to determine, side-by-side with SerRS–SIRT2, the K_D for the SerRS–$tRNA^{Ser}$ interaction by mass photometry, as the low molecular weight of $tRNA^{Ser}$ makes it difficult to distinguish the SerRS–$tRNA^{Ser}$ complex from free SerRS. Although absolute affinities of tRNA–aminoacyl-tRNA synthetase (aaRS) interactions vary depending on the experimental method, and cellular context, classic and subsequent biochemical studies have consistently reported K_D or approximate affinities (often inferred from K_m in aminoacylation assays) for cognate pairs in the high-nanomolar to low-micromolar range (typically 0.1–1 μM for many isolated aaRS–tRNA pairs)[4, 5, 6]. Considering the cellular tRNA is highly abundant and that tRNA can inhibit SerRS–SIRT2 interaction (Fig. 4c.d), SerRS–SIRT2 interaction is perhaps only stabilized in cells under stress conditions that cause ADPR accumulation and tRNA reduction[7].

Together, these quantitative and biological considerations suggest a dynamic shift in SerRS interactions: under basal conditions, SerRS is primarily engaged with $tRNA^{Ser}$, whereas upon oxidative stress, reduced tRNA availability and elevated ADPR concentrations favor SerRS–SIRT2 binding. We have revised the Discussion section accordingly and added a new figure (Fig. 7) to illustrate the analysis.

Reviewer #2 (Remarks to the Author):

In the manuscript by Zhang et al., the authors explore the structural basis of the interaction between the seryl-tRNA synthetase (SerRS) and the NAD+-dependent deacetylase SIRT2. The authors had described previously the interaction (Shi et al., eLife 2014) in the nucleus, where SerRS was shown to recruit SIRT2 to specific genes and deacetylate histones. The authors solve the structure of SerRS bound to SIRT2, which involves an ADPribose molecule as a mediator of the interaction. The authors identify a binding-like motif in SerRS that interacts with the substrate binding domain of SIRT2 and inhibits its catalytic activity. In contrast, the binding of SIRT2 interferes with the ability of SerRs to generate tRNAs, and therefore its role in translation.

The work presents several interesting findings that, if properly developed, could appeal to a broad audience. For example, the involvement of ADPR in SIRT2 binding to proteins or the functional implications of SerRS-mediated regulation of translation by SIRT2. However, the manuscript falls short of fully exploring these aspects and the authors have not provided any evidence to demonstrate the functional relevance of these in vitro studies. In its current form, the study is quite descriptive and primarily focuses on structural data, making it more suitable for a specialized journal.

Some specific Major issues:

1) The involvement of ADPR in the binding is intriguing and potentially very relevant but it raises many unaddressed questions:

a) The importance of ADPR in binding is demonstrated only in vitro using bacterially expressed proteins, with no evidence provided to confirm its relevance in vivo. The authors also present competition data suggesting that neither OAADPR nor NAD+ are involved in this binding. Can the

authors rule out the possibility that, in vivo, the detected ADPR is actually OAADPR, the product of the Sirtuin deacetylation reaction, which may undergo deacetylation during the binding process?

We thank the reviewer for this important comment. We performed new experiments and analysis to address this question and are now confident that the bound molecule is ADPR rather than OAADPR. Biochemically, our new binding analysis demonstrated that OAADPR is much less effective than ADPR in promoting the SerRS–SIRT2 interaction (new Fig. 2e). Structurally, no additional electron density corresponding to an acetyl group was observed (updated Fig. 2a). Moreover, the 2′-OH of ADPR forms a hydrogen bond with K414 of SIRT2 (updated Fig. 2a). Acetylation at this position as in OAADPR (Fig. 1b) would either create steric clashes or disrupt the hydrogen bond, explaining why OAADPR exhibits weaker activity in stabilizing the SerRS–SIRT2 complex. Finally, we now show that oxidative stress enhances SerRS–SIRT2 complex formation through a PARP1-mediated pathway as pretreatment with the PARP1 inhibitor PJ34 abolished the stress-induced enhancement of the SerRS–SIRT2 interaction (new Fig. 6). As PARP activation generates ADPR through PAR (poly(ADP-ribose)) synthesis and degradation, whereas OAADPR is produced by sirtuin-catalyzed deacetylation reactions, the selectivity of ADPR—but not OAADPR—aligns with the PARP1-mediated mechanism. In addition to adding the new analyses, we have expanded the Discussion.

b) Linked to this previous point. The authors show that the binding does not require deacetylation of SeRS by SIRT2, but the key question here is whether this ADPR-dependent binding is triggered by SIRT2-mediated deacetylation. For instance, could it be triggered by SIRT2 auto-deacetylation? Can SIRT2 previously incubated with NAD+ bind more efficiently to SeRS?

For the reasons stated above, we consider it unlikely that the ADPR-dependent SerRS–SIRT2 interaction is triggered by SIRT2-mediated deacetylation. Instead, our data and analyses support a model in which PARP activation promotes SerRS–SIRT2 complex formation via ADPR accumulation, thereby linking PARP activity to SIRT2 inhibition within a shared, stress-responsive NAD^+-dependent pathway.

c) The model proposed by the authors is confusing. Do the authors imply that SerRS binds to ADPR and then bind to SIRT2? Would that mean that SeRS is an ADPR-binding protein? or ADPR is already bound to SIRT2 by the time SeRS interacts with it?

This is an important clarification, and we thank the reviewer for highlighting it. Our model does not suggest that SerRS itself is an ADPR-binding protein. Rather, structural analysis shows that ADPR is bound within the catalytic pocket of SIRT2 and forms hydrogen bonds with a SerRS catalytic-domain loop, particularly involving residue K414 of SerRS. ADPR is not a strong binder to SIRT2. In the absence of SerRS, the addition of ADPR at 5–10× the NAD^+ concentration had no effect on SIRT2 activity (Fig. 3c). However, in the presence of SerRS, ADPR enhanced SerRS-mediated inhibition of SIRT2 in a concentration-dependent manner (Fig. 3c), reflecting ADPR-induced stabilization of the SerRS–SIRT2 complex. Therefore, it is likely that ADPR first associates with SIRT2 and subsequently engages SerRS through the K414-containing catalytic-domain loop.

d) The author's claim that SIRT2 inhibits translation through SerRS is an overstatement that needs to be demonstrated. The authors only show a decreased rate of Ser-tRNA vs Tyr-tRNA.

We agree that the acidic gel northern blot (Fig. 4g, h) only reflects the aminoacylation efficiency of SerRS and does not directly assess translation. Our conclusion that SIRT2 inhibits translation is supported by SUnSET assay results (Fig. 4i, j; previously Fig. 4h,i), which measures global

protein synthesis rates by detecting puromycin incorporation into nascent peptides. In this assay, SIRT2 (but not N168A or H187Y mutant) overexpression led to reduced puromycin incorporation, indicating decreased translational activity.

2) The authors' major claim here is that SerRS inhibits SIRT2 activity but in the previous work (Shi et al., eLife 2014), they proposed that SerRS in fact induces SIRT2 activity by recruiting it to specific DNA regions. How do the authors reconcile both models? does this mean that in the first case, the binding mechanism is not the same?

We appreciate the reviewer for raising this very important controversy, which we now have extensively investigated and gained a clearer understanding.

First, structural, *in vitro* biochemical, and cell-based analyses carried out in the study clearly demonstrate that the SerRS–SIRT2 interaction results in reciprocal inhibition of both enzymatic activities (updated Fig. 3 and 4). However, our previous study reported that SerRS modestly enhanced SIRT2 activity *in vitro*[8], an observation that appears inconsistent with the inhibitory effects described here. One likely explanation for this discrepancy lies in differences in experimental reagents. Whereas the present study used purified, untagged SIRT2, our earlier work relied on a commercially sourced GST-tagged SIRT2 protein, which may have interfered with SerRS binding and/or SIRT2 enzymatic activity, thereby masking the inhibitory interaction revealed here.

Consistent with this interpretation, we now show that SerRS overexpression increases global H4K16 acetylation (new Fig. 3f, g), whereas our prior work reported decreased H4K16 acetylation at the *VEGFA* promoter following SerRS overexpression[8], which we are also able to reproduce (Figure R2a). Together, these findings suggest that SerRS differentially regulates global versus locus-specific H4K16 acetylation. We propose a model in which SerRS restrains SIRT2 activity through complex formation while, at specific genomic loci, SerRS–DNA interactions may weaken this complex and locally release SIRT2 to drive targeted H4K16 deacetylation. This model reconciles the global inhibitory effects observed here with the promoter-specific activation reported previously. Consistent with this model, pull-down assays demonstrate direct competition between SIRT2 and the VEGFA promoter DNA for SerRS binding, while EMSA analysis show diminished capacity of the SerRS–SIRT2 complex for DNA binding (Figure R2b, c).

Clarifying this regulatory principle—particularly its potential extension beyond VEGFA to other SerRS-associated loci—would require substantial additional investigation. Such an analysis is important, yet beyond the scope of the present study, which instead focuses on the structural and mechanistic basis of SerRS-mediated SIRT2 inhibition, directly supported by our biochemical, cellular, and structural data. Within this framework, we conclude that SerRS functions as a bona fide inhibitor of SIRT2 when present in a stable complex. Accordingly, we limit discussion of locus-specific regulatory outcomes to a brief reconciliation with prior observations in the Discussion and defer a systematic analysis of these effects to future studies.

We summarized the current unpublished data below that support our model for reviewers only.

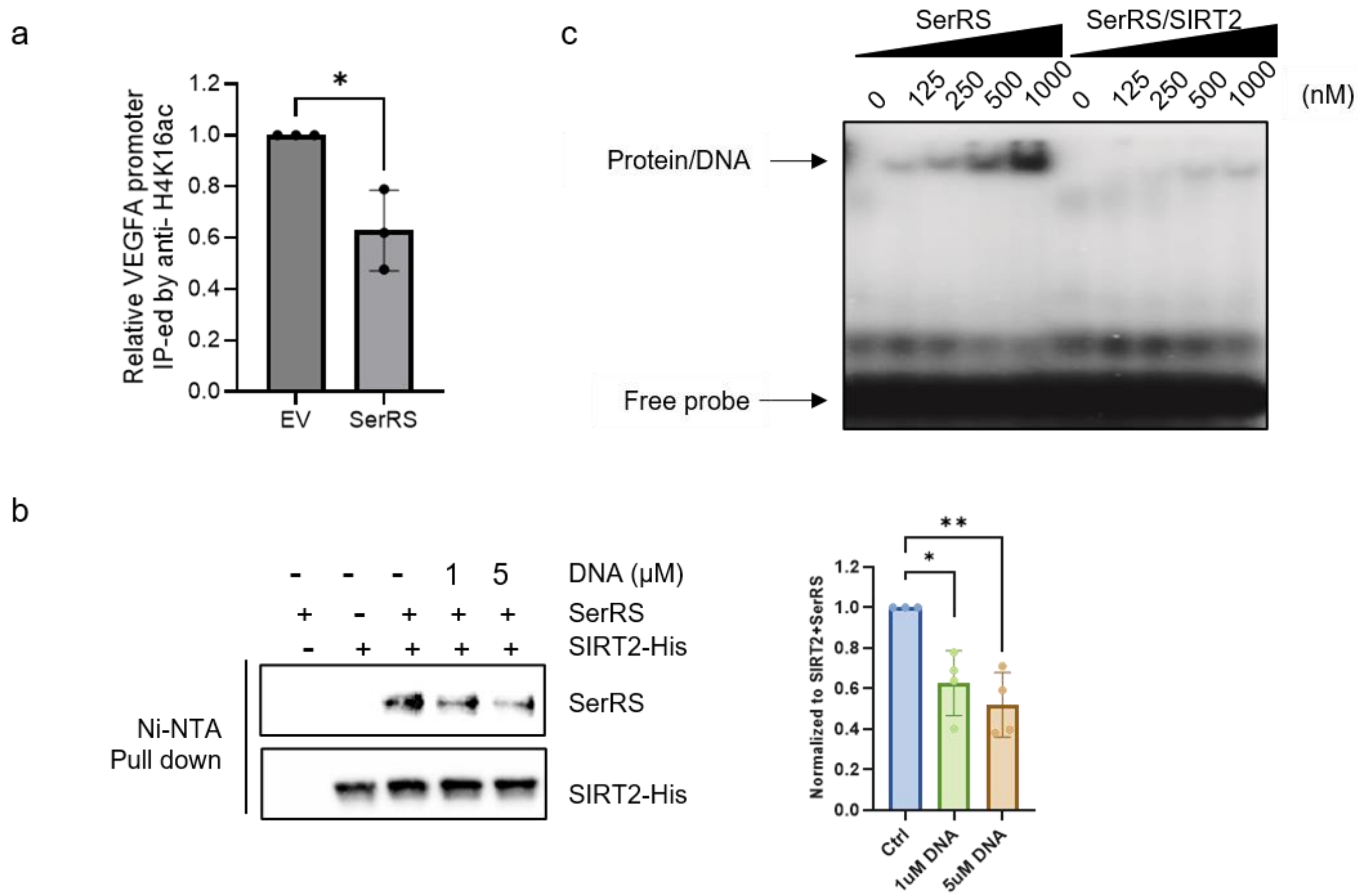

Figure R2. (a) ChIP-qPCR assay showing overexpression of SerRS reduce H4K16 acetylation at the VEGFA promoter. (b) Ni-NTA pull-down assay showing that the SerRS–SIRT2 interaction is suppressed by DNA in a does dependent manner. (c) EMSA showing SerRS–SIRT2 complex exhibit diminished DNA binding capacity.

3) What is the primary purpose of the functional interplay between these two factors? The negative regulation of translation? Is SIRT2/SerRS binding enhanced in cells under stress conditions or metabolic alterations (e.g., calorie restriction)? The authors should provide evidence to address this question.

Both reviewers #1 and #2 brought up the importance of understanding the biological relevance of the SerRS–SIRT2 interaction in cells. We have conducted additional experiments to address this point.

Given that ADPR promotes SerRS–SIRT2 interaction *in vitro,* with dissociation constants decreasing as ADPR concentrations increase into the tens to hundreds of micromolar range of ADPR ($K_D \approx$ 1.1 μM at 16 μM ADPR and ~0.3 μM at 400 μM ADPR; new Fig. 2h), we hypothesized that cellular stress conditions that elevate ADPR level would promotes the SerRS–SIRT2 interaction. In cells, oxidative stress induced PARP1 activation is a well-established pathway for ADPR production.

Therefore, we treated cells with H_2O_2, which activated PARP1 and led to a marked increase in PAR polymer levels, followed by time-dependent PAR clearance, a process expected to elevate

intracellular free ADPR (new Fig. 6a). In parallel, we observed a significant increase in SerRS–SIRT2 interaction following PAR degradation (new Fig. 6b-c). Importantly, pretreatment with the PARP1 inhibitor PJ34 attenuated both PAR accumulation and the stress-induced enhancement of the SerRS–SIRT2 interaction. Therefore, as we hypothesized, oxidative stress enhances the SerRS–SIRT2 complex formation via PARP1-mediated ADPR production.

We speculate that under basal conditions, when ADPR levels are low, the interaction between SerRS and SIRT2 is weak, and the high abundance of cellular tRNA would also block the interaction and shield SerRS from SIRT2-mediated inhibition (updated Fig. 4c-e). In this context, SerRS would predominantly function in its canonical role, charging tRNA in an ATP-dependent manner to sustain protein synthesis, while SIRT2 would continue to utilize NAD^+ to deacetylate α-tubulin at K40, supporting microtubule dynamics associated with cell migration, division, and intracellular transport (new Fig. 7).

During cellular stress, increased ADPR levels could favor the formation of a more stable SerRS–SIRT2 complex, resulting in mutual inhibition of both enzymatic activities. This process may be further facilitated by stress-associated reductions in cytosolic tRNA. Indeed, tRNAs are cleaved by angiogenin under various stress conditions[7], and oxidative stress has also been reported to enhance tRNA retrograde transport from the cytoplasm to the nucleus[9]. Formation of the SerRS–SIRT2 complex is expected to reduce tRNA serylation and dampen global translation (updated Fig. 4e-j), thereby limiting ATP and amino acid consumption. In parallel, inhibition of SIRT2 activity could attenuate α-tubulin deacetylation, potentially promoting microtubule stabilization. Together, these effects suggest a model in which the SerRS–SIRT2 complex may act as a stress-responsive regulatory node that coordinates translational restraint, NAD^+ utilization, and cytoskeletal organization to support cellular adaptation and resilience under adverse conditions (new Fig. 7).

We added a new section entitled "Oxidative stress promotes SerRS–SIRT2 interaction" and also updated the Abstract and the Discussion sections of the manuscript.

4) Is this ADPR-mediated SIRT2 interaction a SerRS-specific mechanism? Or is a general mechanism involving binding to SIRT2? Is the SerRS motif involved in this interaction conserved in other proteins?

This is indeed a very interesting question that warrants further exploration. Previous IP–MS data[10] have shown that several tRNA synthetases co-immunoprecipitated with SIRT2 upon its overexpression (new Extended Data Fig. 12). Interestingly, SerRS is the most enriched and abundant interactor identified among all SIRT2 interactors. GlyRS, the second most abundant tRNA synthetase in the dataset, has also been reported to inhibit SIRT2 deacetylase activity[11]. In addition, interactions between SIRT2 and TyrRS, LysRS, and HisRS were also observed[12], raising the possibility that multiple tRNA synthetases may act as general negative regulators of SIRT2.

However, whether these interactions are also mediated by ADPR remains unknown. Structural comparisons indicate that the K414-containing loop of SerRS and its surrounding architecture are not conserved among other tRNA synthetases identified in the SIRT2 interactomes. Nevertheless, this represents an intriguing direction for future investigation. We have now incorporated this discussion into the revised manuscript.

Reviewer #3 (Remarks to the Author):

In this work, Zhang and colleagues aim to structurally and functionally characterize the complex between the human cytosolic seryl-tRNA synthetase (SerRS) and sirtuin-2 (SIRT2), which is a rather vaguely studied NAD+-dependent deacetylase.

The authors initially detected the specific interaction between these two proteins in human cells, which they described in an eLife paper published in 2014. Here, they purify the co-expressed complex and they determine the structure of the complex by single particle cryo-EM at a nominal resolution of 3.2 Å. The structure shows how the two proteins interact with each other and that a co-purified ADPR molecule is needed for complex formation. Of note, they obtain the complex at different stoichiometries and they use mutational analyses (e.g. interface, APPR binding site and catalytic centers) to validate their structural observations in vitro and in human cell lines. Furthermore, they show that the binding to each other inhibits the catalytic activity of each proteins, which indicates a co-regulation that is also linked to the metabolic state of the cell via ADPR. The manuscript is well written and the illustration nicely highlight the main observations. In my opinion the cryo-EM analyses needs to be carried out with much more care and I could imagine that the actually collected dataset might allow to arrive at a much better reconstruction. The manuscript builds on previous cellular observations (eLife 2014) and at the moment I am very confused that several observations stand in stark contrast to the previous conclusions. In addition, the authors do not address the open question whether the DNA-binding capacity of SerRS in connection with the anticipated histone deacetylation activity of SIRT2 could be mainly responsible for the cellular effects. Here the authors focus on the cytoplasmatic aspects, but as additional structure-guided assays in human cells are missing the conclusions of the two manuscript do often contradict each other. Despite my interest in the regulatory function of this rather unexpected protein complex, I am not convinced that the work is suitable for publication in Nature Communication in its current form.

Major issues

1. In the previous paper the authors state “SerRS interaction promotes SIRT2 deacetylase activity”. Here they state on page 8 line 211-213 “Indeed, SerRS inhibits the deacetylase activity of SIRT2 in a concentration dependent manner (Fig. 3b).” The authors do not interpret these obviously contradicting conclusions or discuss potential differences between the function of the complex in the cytoplasm or in the nucleus. The authors analyse the effect of overexpressing/knocking down of SerRS of the acetylation of Lys40 alpha-tubulin, but they do not analyse the effect on histone acetylation. Did the authors perform celllar fractionation experiments to see whether the complex forms in both cellular compartments or if the availability of ADPR spatially restricts complex formation?

We thank both reviewers (#2 and #3) for raising this very important controversy, which we now have extensively investigated and gained a clearer understanding.

First, we now have also analyzed the impact of SerRS on histone acetylation (H4K16). SerRS overexpression increased global H4K16 acetylation, phenocopying the effect of the SIRT2 inhibitor AGK2 treatment (new Fig. 3f, g). Moreover, co-overexpression of SerRS markedly attenuated the SIRT2-mediated deacetylation of both a-tubulin K40 and H4K16 induced by SIRT2 overexpression alone (updated Fig. 3d-g). Therefore, structural, *in vitro* biochemical, and cell-based analyses carried out in the study clearly demonstrate that the SerRS–SIRT2 interaction results in reciprocal inhibition of both enzymatic activities (updated Fig. 3 and 4).

However, our previous study reported that SerRS modestly enhanced SIRT2 activity *in vitro*[8], an observation that appears inconsistent with the inhibitory effects described here. One likely explanation for this discrepancy lies in differences in experimental reagents. Whereas the present study used purified, untagged SIRT2, our earlier work relied on a commercially sourced GST-tagged SIRT2 protein, which may have interfered with SerRS binding and/or SIRT2 enzymatic activity, thereby masking the inhibitory interaction revealed here.

While SerRS overexpression increases global H4K16 acetylation (new Fig. 3f, g), we were also able to reproduce decreased H4K16 acetylation at the *VEGFA* promoter following SerRS overexpression (Figure R2a), as reported by our prior work[8]. Together, these findings suggest that SerRS differentially regulates global versus locus-specific H4K16 acetylation. We propose a model in which SerRS restrains SIRT2 activity through complex formation while, at specific genomic loci, SerRS–DNA interactions may weaken this complex and locally release SIRT2 to drive targeted H4K16 deacetylation. This model reconciles the global inhibitory effects observed here with the promoter-specific activation reported previously. Consistent with this model, pull-down assays demonstrate direct competition between SIRT2 and the VEGFA promoter DNA for SerRS binding, while EMSA analysis show diminished capacity of the SerRS–SIRT2 complex for DNA binding (Figure R2b, c).

We believe that this regulatory principle likely extends beyond VEGFA to other SerRS-associated loci and represents an important direction for future investigation. In the present study, we focus on the structural and mechanistic basis of SerRS-mediated SIRT2 inhibition; accordingly, locus-specific regulatory outcomes are discussed only briefly in the context of prior observations and will be systematically addressed in future studies.

For reviewers only, we summarized the current unpublished data that support our model in Figure R2 (see above in responses for Reviewer #2).

2. Along these lines, the author state that “Conversely, SIRT2 binding disrupts the aminoacylation activity of SerRS by directly competing with and allosterically regulating its interaction with tRNASer,…”. In the previously published work they conclude that the complex formation affects the DNA binding capacity of SerRS. Do the authors think that this is not case anymore, did the authors test the effect of complex formation on DNA binding and would specific mutations that inhibit complex formation bind stronger to DNA in cells?

Please see above. SerRS–SIRT2 complex indeed has a diminished DNA binding capacity (Figure R2b). Like tRNA (Fig. 4c, d), DNA can also compete with SIRT2 for SerRS binding (Figure R2c). Because of the competition, we expect that mutations in SerRS that inhibit the SIRT2 interaction may have some advantage in DNA binding. Indeed, the $SerRS^{K414R}$ mutant, which lacks SIRT2-binding ability (Fig, 2b), shows a comparable or slightly enhanced DNA-binding by a Chip-qPCR assay (Figure R3).

While our data support a steric and competitive relationship between SIRT2 and DNA for SerRS binding, as explained above, in this study we focus on defining a cross-regulatory mechanism between SerRS and SIRT2 by elucidating the structural basis of ADPR sensing and their reciprocal inhibition. Expanding the manuscript to include additional regulatory layers, while interesting, would extend beyond the primary scope of this work and potentially dilute the central conclusions.

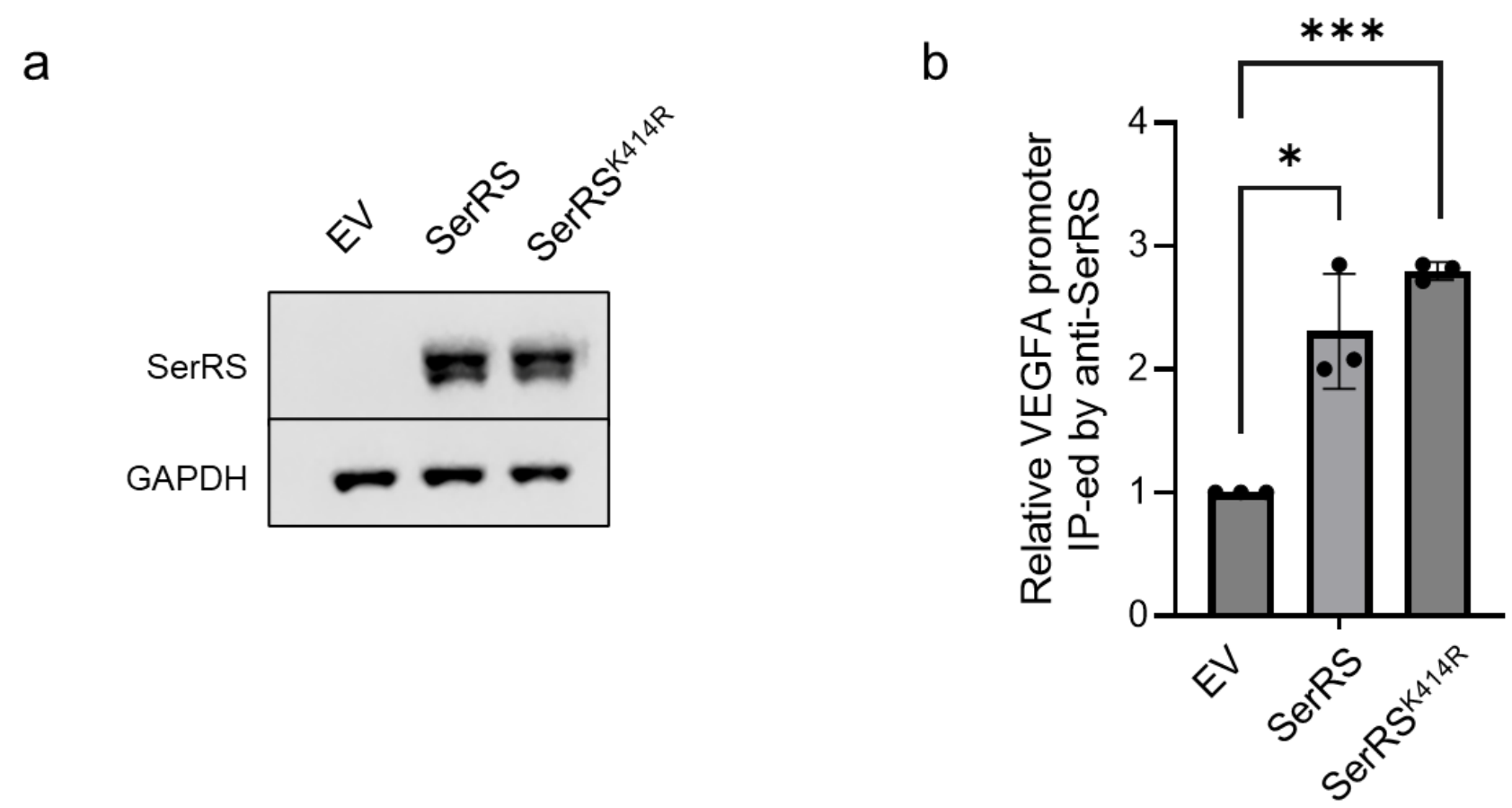

Figure R3. (a) comparable overexpression of SerRS and the SIRT2-binding–deficient $SerRS^{K414R}$ mutant; (b) ChIP-qPCR assay showing that overexpression of $SerRS^{K414R}$ mutant results in comparable or slightly enhanced binding to the VEGFA promoter relative to wild-type SerRS.

3. There are several critical issues with the presented cryo-EM data and the performed processing pipeline

We thank the reviewer for providing constructive suggestions. In response, we have reprocessed the cryo-EM data and updated Extended Data Fig. 1 and relevant main figures accordingly. A detailed point-by-point response to the reviewer is provided below.

a. The presented raw micrograph is not very informative – it needs to magnified, it might be good to also show a denoised version of it and the scale bar should be the size of the expected particles…please also use boxes to highlight the picked/selected particles

For the reviewer's suggestion, we have included a magnified representative micrograph in updated Extended Data Fig. 1a. The scale bar has been adjusted to 10 nm, which approximates the size of the SerRS–SIRT2 complex (~100 Å for the 2:1 complex). We used the "Inspect Particle Picks" job in cryoSPARC to display the particles selected in the representative micrograph used for the final reconstructions. Picked particles are marked with green dots. As shown, the micrograph shows a high degree of particle heterogeneity, likely due to partial denaturation of protein samples at the air–water interface during vitrification[13, 14]. Nevertheless, we have highlighted two clear examples of SerRS–SIRT2 complexes with red elliptical outlines in Extended Data Fig. 1a.

b. I missing Table 1 that summarizes all cryo-EM collection and refinement parameters

We apologize for the earlier mislabeling of Table 1 in the initial submission. The correct and updated version of Table 1 is now provided. This table summarizes the cryo-EM data collection and refinement statistics for the 2:2 and 2:1 reconstructions. All maps have been deposited in the

EMDB, and the structural models have been deposited in the PDB. The validation reports have been submitted with the revised manuscript.

c. The Global FSC curves do not reach 0 – indicating that the authors did not remove duplicates. Considering that the data was apparently collected from rather overcrowded holes, I would be very curious about the number of duplicates. A large number of duplicated “particles” would explain that the nominal resolution is artificially overstated and that the map quality does not fit the stated resolution limits.

We thank the reviewer for the noticing this issue with the FSCs, as we should have noticed this ourselves. We typically pack our particles into holes to reduce motion and improve CTF estimation, and didn’t account for potential duplicates. However, following extensive 2D classification, we selected 591,091 particles that exhibited high-resolution features in the 2D classes. When we map these selected particles back on to micrographs (green spots in the updated Extended Data Fig. 1a) we observe substantial spacing between most of the particles. We now also used the “Remove Duplicate Particles” job in cryoSPARC with a minimum separation distance of 20 Å, resulting in the removal of 5,196 duplicate particles. All downstream processing was done with those duplicate particles removed. The global FSC curves of the updated reconstructions now extend to 0, as shown in Extended Data Fig. 1c. After the reprocessing, the overall resolution is now 3.38 Å for the 2:1 complex and 3.73 Å for the 2:2 complex.

d. Please show orientation distribution plots (after removing duplicates)
The orientation distribution plot now is shown in updated Extended Data Fig. 1d.

e. Could the authors explain the reasoning behind the local refinement step – the mask in this step does not remove/exclude the particles that don’t fulfil the requirements. A real focused 3D classification protocol needs to be used. In general, I would suggest to process the dataset in parallel in Relion, which allows to perform true 3D classification steps.

We appreciate the reviewer’s comments and suggestions. As shown in updated Extended Data Fig. 1, we now used a processing pipeline that begins with extensive 2D classification, followed by reference-free *ab initio* reconstructions, which effectively distinguished the 2:1 and 2:2 SerRS–SIRT2 complexes. Specifically, *ab initio* modeling yielded three classes: the first corresponding to the 2:1 complex, the second to the 2:2 complex, and the third to junk particles (Extended Data Fig. 1b).

Notably, both the first and second *ab initio* classes contained the portion of the 2:1 complex. We also observed that one SIRT2 subunit was consistently better resolved than the other even in the 2:2 complex. To maximize the structural information, we combined particles from both classes and performed non-uniform refinement to align all particles first. To further improve resolution, we performed local refinement with masks encompassing the more stably associated 2:1 SerRS-SIRT2 region. These local refinements yielded improved resolution (from 3.82 Å to 3.38 Å) and map quality, particularly in the SIRT2 region (Figure R4). We attribute these improvements to the intrinsic flexibility of the complex, where the second SIRT2 subunit and the tRNA-binding arm impacted the alignment of the first SIRT2. We recognize that RELION has a better 3D classification algorithm than cryoSPARC, but given that the resolution limitation in this system arises more from misalignment due to flexibility of a portion of the structure than the presence or absence of a subunit, we don’t see how further 3D classification would improve the density.

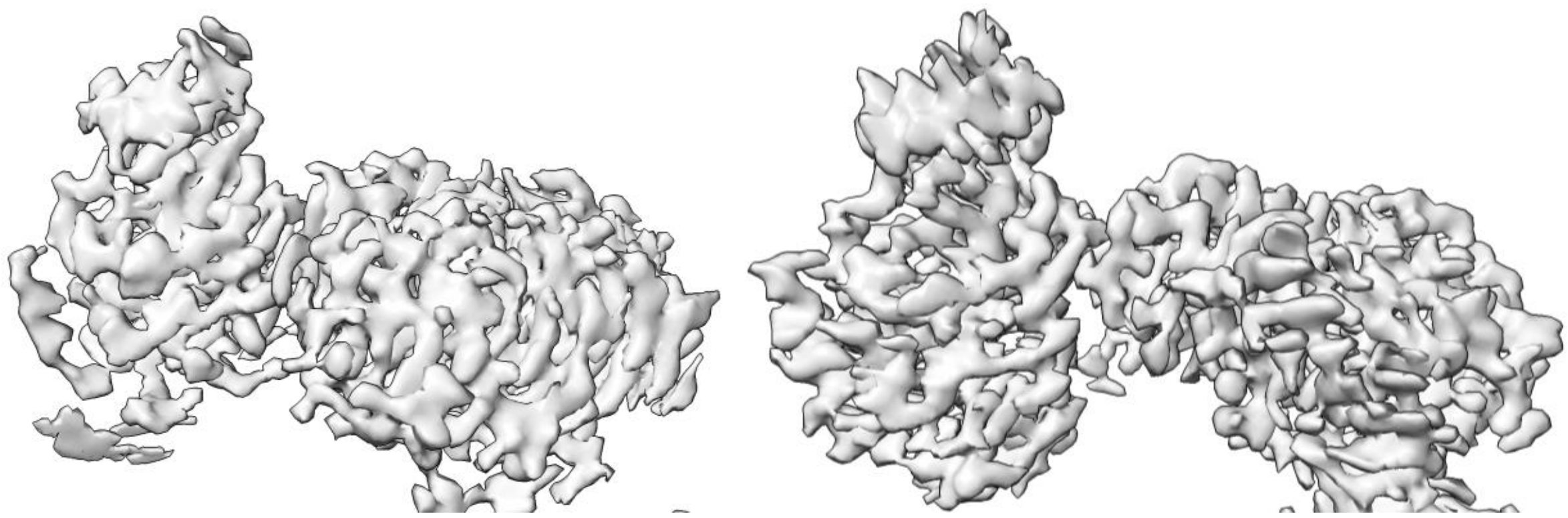

Figure R4. Comparison of the NU-refined and locally refined maps shows that local refinement improves both resolution and map quality, particularly in the SIRT2 region.

f. Please highlight the local resolution around the ADPR ligand

We thank the reviewer for the suggestion. We have now included a local density map surrounding the ADPR-binding region, as shown in the updated Extended Data Fig. 2b. The local resolution in the vicinity of the ADPR ligand is estimated to be approximately 3.2–3.6 Å. Together with Fig. 2a, which shows well-defined cryo-EM density for the ADPR ligand alone, these data support confident modeling of ADPR at the binding site.

g. Could the authors comment on the chosen box size – it appears too small for the size of the particles

We thank the reviewer for pointing this out. As suggested, we have reprocessed the dataset using a larger box size, increasing it from 196 pixels to 256 pixels (1.15 Å/pixel).

h. Did the authors use RBMC implemented in the latest CryoSPARC version to further improve their reconstructions

During data collection, we used Appion to monitor the acquisition process and perform initial motion correction and dose-weighted frame alignment. The aligned micrographs were then imported into cryoSPARC for downstream processing. Some of the original raw.tiff images have since been archived to tape storage, making it non-trivial to retrieve them for reprocessing. As a result, we did not apply cryoSPARC's Reference-Based Motion Correction (RBMC) for this project.

4. The experiments described in the lower paragraph on page 9 are interesting, but they only indirectly address the question if SIRT2 has an effect on the activity of SerRS. I assume the authors could test which effect increasing amounts of SIRT2 (in presence or absence of ADPR) have on the in vitro aminoacylation activity of SerRS. The authors use the preformed complex to compare the activity, but after they showed that they can reconstitute the complex in vitro in the presence of ADPR (Fig. 2f) this would be the most direct way to answer the raised question.

We appreciate reviewer's insightful suggestion. As recommended, we now performed the aminoacylation assay using separately purified SerRS and SIRT2 proteins. We found that addition of SIRT2 alone did not affect SerRS aminoacylation activity, suggesting that SerRS/tRNA binding predominates in the absence of additional ligands (new Fig. 4e). However, inclusion of ADPR in the reaction promoted SerRS/SIRT2 complex formation and led to a clear inhibition of SerRS activity, confirming that ADPR stabilizes the interaction between the two proteins and mediates functional inhibition.

5. In the SUnSET assays it would important to clarify the levels of ADPR in cells.

We appreciate the reviewer's comment. We attempted to quantify intracellular ADPR levels in HEK293AD cells under basal and H_2O_2-treated conditions using ESI triple-quadrupole mass spectrometry (Table R1). Briefly, metabolites were extracted using a MeOH:H_2O (4:1) solvent system and analyzed following the same procedure used for purified standards. The absolute ADPR levels we obtained (see below) were substantially lower than those reported in the literature, likely reflecting suboptimal extraction efficiency and/or rapid degradation of this labile metabolite under the current conditions. Indeed, previous HPLC-based studies reported mean intracellular ADPR concentrations of 44 ± 11 µM in Jurkat cells and 73 ± 11 µM in the murine thymoma cell line BW5147[3], whereas cellular NAD^+ concentrations are typically in the range of 200–500 µM[2]. Therefore, to avoid potential misinterpretation, we have not included these quantitative measurements in the manuscript. Nevertheless, the observed decrease in NAD^+ signals accompanied by an increase in ADPR signals is consistent with enhanced NAD^+ consumption and free ADPR production under oxidative stress.

Although we were unable to precisely determine intracellular ADPR levels in the cells used for the SUnSET assay, we have now defined the range of ADPR concentrations that promote SerRS–SIRT2 interaction in vitro (new Fig. 2h). ADPR at 16 µM is sufficient to induce SerRS–SIRT2 complex formation (K_D = 1.1 µM), and the interaction is further strengthened at 400 µM ADPR (K_D = 0.3 µM). Considering reported intracellular ADPR concentrations (~40–70 µM), these values are well within the range expected to support SerRS–SIRT2 interaction in cells and the resulting reciprocal inhibition of both enzymes, with downstream consequences including suppression of protein synthesis.

Table R1 NAD^+, OAADPR and ADPR level in HEK293AD cells detected by ESI Triple Quad Mass Spectrometry

	NAD^+ (µM)	OAADPR (nM)	ADPR (µM)
Control	375.28	<10	0.29
H_2O_2 treated	253.14	<10	1.23

6. I don't see dramatic reduction of SerRS levels in the si-SerRS sample in Figure 4h – could the authors quantify the knock down efficiency and comment on it?

In response to the reviewer's comment, we quantified SerRS band intensities and normalized them to α-tubulin to assess the knockdown efficiency. This analysis indicates that approximately

67% (0.70/1.06) of SerRS remains in the si-SerRS sample. The relative SerRS and α-tubulin intensities are now included in the revised Fig. 4i (formerly Fig. 4h).

7. Did the authors test the interaction of SIRT1 carrying Phe at the equivalent position as F244 in SIRT2? Would this SIRT1 mutant start interacting with SerRS as well?

We appreciate the reviewer's thoughtful question. Our mutational analysis indicates that SerRS–SIRT2 interaction is mediated by multiple residues, including F235, F244, and S271. Single-point mutation of any one of these residues is sufficient to disrupt the interaction (Fig. 5f). Although F235 is conserved in SIRT1, both F244 and S271 are not conserved (Extended Data Fig. 10). Given that SerRS binding requires the coordinated contribution of multiple SIRT2-specific residues, we do not consider it likely that introducing a single phenylalanine at the position equivalent to F244 in SIRT2 would be sufficient to confer SerRS-binding capability to SIRT1.

Minor

• Please use "KDa" instead of "KD"

We have corrected the unit notation from "kD" to "kDa".

• Page 4 line 112 – "had an estimated molecular weight of 213.4 kD" – either provide error estimates or use "approximately 215 KDa" – gel filtration column is that exact. The conclusions of the stoichiometry are still valid.

We have revised the text to "approximately 215 kDa" to better reflect the accuracy of the gel filtration estimation.

• Page 5 Line 121/122 – "Both 2:2 and 2:1 complexes were selected for ab initio Non-Uniform (NU)-refinement with C2 symmetry, …" Why would you choose C2 symmetry for the 2:1 complex? Extended data Fig. 1 shows that you did not use symmetry – please clarify.

We apologize for the typo in the previous version of the manuscript. We have revised this paragraph on page 5 to clarify that non-uniform refinement of the 2:1 SerRS–SIRT2 complex was performed using C1 symmetry rather than C2 symmetry, consistent with the processing workflow shown in Extended Data Fig. 1.

• The authors use the structure tRNASec bound to SerRS, but due the very specific shape of tRNASec, I would suggest to use either PDB ID 8FFY of the mitochondrial tRNASer or PDB ID 8P7B of SerRS-tRNASer from the recently determined structure of the METTL6-SerRS complex.

We appreciate the reviewer's valuable suggestion. As recommended, we updated the structural comparison by superimposing the SerRS–SIRT2 complex with the recently determined SerRS–$tRNA^{Ser}$ interaction within the METTL6–SerRS–$tRNA^{Ser}$ complex (PDB ID: 8P7B; updated Fig. 4a). The updated model revealed a clear steric clash between SIRT2 and $tRNA^{Ser}$ at the SerRS binding interface, supporting a competition between SIRT2 and $tRNA^{Ser}$ for SerRS binding.

Reviewer #4 (Remarks to the Author):

Zhang et al. demonstrated the physical interaction between dimeric SerRS and SIRT2 by determining the cryo-EM structures of the 2:2 and 2:1 complexes. The structures reveal that ADPR is a key metabolite mediating protein-protein interactions, leading to the inactivation of both

SIRT2 and SerRS. This complex provides a compelling model in which the cell's energy status, represented by the accumulation of ADPR, contributes to the regulation of protein synthesis and the homeostasis of serine, a central node in metabolic pathways. The authors have accomplished remarkable work in solving the structure of the SIRT2-SerRS complex. However, my primary concern lies in the weakness of their biophysical analyses. The key finding, namely the ADPR-dependent complex formation between SerRS and SIRT2, requires a more thorough quantitative examination. After addressing the following issues, I would recommend this manuscript for publication in Nature Communications.

Major Points

1. In line 181, the authors present BLI data showing complex formation with ADPR and NAD+. Can BLI measure the dissociation constants (kd) between SerRS and SIRT2 in the presence of ligands such as ADPR or NAD+ at physiological concentrations?

We greatly appreciate the reviewer's insightful suggestion. As recommended, we measured the dissociation constants (K_D) between SerRS and SIRT2 across a wide concentration range using mass photometry, a label-free technique capable of determining K_D values from individual measurements[15, 16].

Reported metabolomic studies indicate that free ADPR exists at tens of micromolar concentrations under basal conditions (approximately 40–70 μM in mammalian cells)[3] and rises several-fold upon PARP1 activation or SARM1-mediated NAD^+ cleavage during oxidative or genotoxic stress. Reported intracellular NAD^+ concentrations vary widely depending on the measurement method and compartment. Live-cell biosensors detect free NAD^+ in the cytosol and nucleus at tens to low hundreds of micromolar[1], whereas biochemical assays measuring total cellular NAD^+ estimate higher levels, around 0.2-0.5mM[2]. Using mass photometry, we found that ADPR markedly enhances SerRS–SIRT2 complex formation, with detectable binding at 16 μM ADPR (K_D = 1.06 ± 0.13 μM) and a tighter affinity at higher concentrations (K_D = 0.33 ± 0.02 μM at 400 μM). By comparison, NAD^+ required 400 μM to support detectable complex formation (K_D = 0.81 ± 0.25 μM) (new Fig. 2h).

Taken together, these quantitative and biological observations indicate that under physiological conditions, both ADPR (having a stronger capacity to stabilize the complex) and NAD^+ (present at higher intracellular concentrations) might serves as basal modulators of the SerRS–SIRT2 interaction. Given that intracellular NAD^+ levels decline while ADPR levels rise dramatically under oxidative or genotoxic stress, ADPR accumulation likely acts as a molecular switch that promotes and stabilizes SerRS–SIRT2 complex formation in vivo, thereby coupling cellular stress responses to SIRT2 and SerRS regulation.

2. In Figure 3c, it appears that SIRT2 activity is significantly reduced (by approximately 50%?) by SerRS, even when [ADPR] = 0. Furthermore, deacetylation activity does not change substantially when [ADPR] is increased up to 400 μM. In Figures 4c and 4d, SIRT2 and SerRS appear to form a complex in the absence of ADPR, with a more favorable interaction (by more than 4-fold?) when ADPR is present, though the specific concentration is not mentioned. It would be beneficial to demonstrate the correlation between complex formation and deacetylation activity in a more quantitative manner. I suggest completing titration curves for in vitro activity and complex formation at fixed protein concentrations while varying [ADPR].

This is an excellent point, and we appreciate the opportunity to clarify this issue. In Fig. 3c, the apparent inhibition of SIRT2 by SerRS in the absence of exogenously added ADPR likely reflects

two contributing factors. First, NAD^+, which is required for SIRT2 deacetylation activity, can transiently promote SerRS–SIRT2 interaction, resulting in partial inhibition even when [ADPR] = 0. Second, and likely more importantly, during the deacetylation reaction NAD^+ is converted to OAADPR and subsequently hydrolyzed to ADPR, as confirmed by our mass spectrometry analysis (Extended Data Fig. 6). Because the precipitation efficiency of OAADPR and ADPR is low, their true concentrations in the assay are likely substantially higher than detected. Therefore, ADPR is naturally generated during the deacetylation reaction, which explains why the addition of exogenous ADPR produces only a modest further reduction in SIRT2 activity (Fig. 3c). Moreover, because NAD^+ binds SIRT2 with higher affinity than ADPR, it may competitively preclude ADPR binding to SIRT2, thereby limiting formation of the SIRT2–SerRS complex. Together, these factors account for the observed basal inhibition and the limited dynamic range upon increasing ADPR concentration.

As suggested, we performed a new Ni-NTA pull-down assay (new Fig. 2e) using fixed concentrations of SerRS and SIRT2 while titrating NAD^+, ADPR, and OAADPR. The results show that ADPR > NAD^+ > OAADPR in stabilizing the SerRS–SIRT2 complex. Furthermore, our mass photometry analysis provided quantitative K_D measurements for these interactions at varying ligand concentrations, as detailed in our response to the previous comment. Together, these data confirm that ADPR effectively promotes and stabilizes SerRS–SIRT2 complex formation in vitro.

Minor Points

3. The authors provide a structure-based explanation of the importance of F235 in SIRT2's interaction with SerRS via a cation-π interaction with K414. However, what about F244 and S271 in SIRT2? What insights does the structure offer about their specific molecular interactions with SerRS?

We have incorporated additional structure-based details clarifying the roles of F235, F244 and S271 in mediating the SIRT2–SerRS interaction (updated Fig. 5a). Specifically, F235 of SIRT2 forms a cation–π interaction with K414 of SerRS and engages in a hydrophobic interaction with M416. In addition, F244 of SIRT2 forms an amide–π stacking interaction with Q412 of SerRS, while S271 of SIRT2 forms a hydrogen bond with R407 of SerRS.

4. Similarly, the key residues of SerRS at the protein interface (e.g., Q412, K414, M416, D282, and E283) should be described in greater detail, including the molecular interactions in which they are involved.

Key SerRS residues at the SIRT2 interface are primarily located within the major loop, including Q412, K414, and M416, which, as noted above, are described in detail. Residues D282 and E283 are located in the minor loop of SerRS. Although these residues lie at the SerRS–SIRT2 interface, they do not form specific interactions with SIRT2. We have revised Fig. 5a to more clearly illustrate these interactions.

5. Line 350–351: "The cellular level of serine is the lowest among all proteinogenic amino acids" (Reference 44). This is surprising—shouldn't it be Trp or Cys? I encourage the authors to verify this statement with a more recent reference.

We thank the reviewer for this valuable comment. Upon double confirm, we found that this statement is not consistently supported by the literature. The intracellular abundance of amino acids varies by cell type and condition, and recent metabolomic data indicate that tryptophan,

rather than serine, is among the least abundant amino acids in most cell types. To avoid any potential inaccuracy, we have removed this sentence from the revised manuscript.

References

1. Sallin O, Reymond L, Gondrand C, Raith F, Koch B, Johnsson K. Semisynthetic biosensors for mapping cellular concentrations of nicotinamide adenine dinucleotides. *Elife* **7**, (2018).
2. Cantó C, Auwerx J. NAD+ as a signaling molecule modulating metabolism. *Cold Spring Harbor symposia on quantitative biology* **76**, 291-298 (2011).
3. Gasser A, Guse AH. Determination of intracellular concentrations of the TRPM2 agonist ADP-ribose by reversed-phase HPLC. *Journal of chromatography B, Analytical technologies in the biomedical and life sciences* **821**, 181-187 (2005).
4. Hausmann CD, Ibba M. Aminoacyl-tRNA synthetase complexes: molecular multitasking revealed. *FEMS microbiology reviews* **32**, 705-721 (2008).
5. Lin SX, Wang Q, Wang YL. Interactions between Escherichia coli arginyl-tRNA synthetase and its substrates. *Biochemistry* **27**, 6348-6353 (1988).
6. Nomanbhoy TK, Hendrickson TL, Schimmel P. Transfer RNA-dependent translocation of misactivated amino acids to prevent errors in protein synthesis. *Molecular cell* **4**, 519-528 (1999).
7. Thompson DM, Parker R. Stressing out over tRNA cleavage. *Cell* **138**, 215-219 (2009).
8. Shi Y*, et al.* tRNA synthetase counteracts c-Myc to develop functional vasculature. *Elife* **3**, e02349 (2014).
9. Schwenzer H*, et al.* Oxidative Stress Triggers Selective tRNA Retrograde Transport in Human Cells during the Integrated Stress Response. *Cell Rep* **26**, 3416-3428 e3415 (2019).
10. Eldridge MJG, Pereira JM, Impens F, Hamon MA. Active nuclear import of the deacetylase Sirtuin-2 is controlled by its C-terminus and importins. *Scientific reports* **10**, 2034 (2020).
11. Zhao Y*, et al.* SIRT2-knockdown rescues GARS-induced Charcot-Marie-Tooth neuropathy. *Aging cell* **20**, e13391 (2021).
12. Li X*, et al.* Knockdown of SIRT2 Rescues YARS-induced Charcot-Marie-Tooth Neuropathy in Drosophila. *Neuroscience bulletin* **40**, 539-543 (2024).
13. D'Imprima E, Floris D, Joppe M, Sánchez R, Grininger M, Kühlbrandt W. Protein denaturation at the air-water interface and how to prevent it. *Elife* **8**, (2019).
14. Noble AJ*, et al.* Reducing effects of particle adsorption to the air-water interface in cryo-EM. *Nat Methods* **15**, 793-795 (2018).
15. Soltermann F*, et al.* Quantifying Protein-Protein Interactions by Molecular Counting with Mass Photometry. *Angewandte Chemie (International ed in English)* **59**, 10774-10779 (2020).
16. Kofinova Z, Karunanithy G, Ferreira AS, Struwe WB. Measuring Protein-Protein Interactions and Quantifying Their Dissociation Constants with Mass Photometry. *Current protocols* **4**, e962 (2024).

REVIEWER COMMENTS

Reviewer #1 (Remarks to the Author):

In their revised manuscript Zhang and colleagues have address the concerns I raised regarding the original version. The new data support and clarify their original findings and strengthen the proposed physiological role for the SerRS:SirT2 interaction. While experimental data for the conservation of the interaction would be desirable, I feel that this is not so important as to further delay the publication of this work.

We thank the reviewer for the positive evaluation of our revised manuscript and for recognizing that the new data strengthen our conclusions regarding the physiological role of the SerRS–SIRT2 interaction.

Reviewer #2 (Remarks to the Author):

The authors have addressed the majority of my previous concerns satisfactorily, and the new experiments included in the revised manuscript significantly strengthen the study. In particular, the additional biochemical analyses and the new cellular experiments linking oxidative stress, PARP activation, and the SerRS–SIRT2 interaction improve the biological relevance of the work and clarify several mechanistic aspects of the proposed model. Overall, the revised manuscript represents a substantial improvement over the original version.
However, there are still two remaining points that I believe should be fully addressed or clarified before the manuscript can be considered for publication:

We thank the reviewer for the positive evaluation of our revised manuscript and for recognizing that the additional experiments have strengthened the study and improved its biological relevance. We also appreciate the reviewer's constructive comments and suggestions. Below, we address the remaining points in detail.

First, the authors attempt to reconcile the conclusions of the present study with their previous work (Shi et al., eLife 2014), where SerRS was reported to activate SIRT2 activity, whereas in the current manuscript SerRS is shown to inhibit SIRT2. The explanation provided by the authors is that the previous study used a GST-tagged SIRT2, whereas the present work uses an untagged protein. This explanation, which is now mentioned in the Discussion section, is reasonable but remains speculative, as it has not been experimentally demonstrated. If this difference indeed explains the apparent discrepancy between the two studies, the authors should demonstrate it experimentally.

We acknowledge that this explanation remains speculative and has not been directly tested. Because the previous study relied on commercially sourced reagents from over

a decade ago, a direct comparison with our current reagent under matched conditions is difficult to achieve.

Importantly, the conclusions of the present study are supported by multiple independent lines of evidence from well-controlled biochemical and cellular assays. Therefore, while the precise cause of the discrepancy remains to be determined, it does not affect the validity of the findings reported here.

To acknowledge this caveat, we have added the following sentence to the Discussion: “Because these commercially sourced reagents date back over a decade, direct testing is difficult; therefore, the precise cause of the discrepancy remains to be determined.”

Second, the authors present new observations suggesting that oxidative stress promotes the formation of the SerRS–SIRT2 complex through a PARP-dependent pathway, presumably via increased ADPR production. These results are interesting and potentially important, as they link cellular stress signaling with the regulation of SIRT2 activity. However, the manuscript still does not formally demonstrate that SerRS-mediated inhibition of SIRT2 under these conditions results in a direct restraint of translation. While the authors provide data on Ser-tRNA charging and SUnSET assays that are consistent with reduced protein synthesis, the causal relationship between SerRS–SIRT2 complex formation and translational inhibition remains somewhat indirect. The authors should provide at least minimal evidence supporting this mechanistic link.

We thank the reviewer for this insightful comment. We agree that linking SerRS–SIRT2 complex formation to translational regulation under oxidative stress is of particular interest.

To further address this point, we have performed additional SUnSET assays under H_2O_2 treatment conditions. We find that oxidative stress reduces global protein synthesis, and this effect is further enhanced by overexpression of SIRT2. In contrast, overexpressing a SIRT2 mutant (N168A) that is deficient in binding to SerRS has a minimal impact, suggesting that the translation inhibition effect is related to the SerRS–SIRT2 interaction (new Fig. 6d and 6e).

Importantly, this additional translational repression appears to occur independently of canonical ISR (as assessed by p-eIF2α and ATF4) and mTOR (as assessed by p-4EBP1 and p-p70 S6K) pathways (new Fig. 6d). This is consistent with a model in which reduced availability of charged $tRNA^{Ser}$, resulting from SerRS inhibition by SIRT2, may contribute to reduced translation rates at serine codons. This mechanism represents a potentially important direction for future investigation.

Reviewer #3 (Remarks to the Author):

I have carefully reviewed the revised version of the manuscript “A metabolite-bridged complex between SerRS and SIRT2 couples NAD^+ metabolism to translation control”. I am genuinely impressed by the newly presented work and the clarity of the novel results. I also appreciate that authors have carefully revised their cryo-EM work flow, which is now at a state-of-the-art level – I am still not sure how the authors have overlooked numerous weaknesses in the data processing routines shown in the original submission.
I believe that the novelty of results and broad interest justifies publication in Nature Communications after the data in reviewer figures R2 and R3 are included in the actual manuscript and a few minor issues are resolved.

We thank the reviewer for the careful evaluation of our revised manuscript and for the positive assessment of the novelty and clarity of our work. We also appreciate the reviewer’s recognition of the improvements in the cryo-EM data processing workflow.

We have now incorporated the key data requested into the main manuscript, as suggested. In addition, we have addressed the remaining minor issues raised by the reviewer, as detailed below.

Major issue
1. Please include the very interesting data shown in Reviewer figures R2 and R3 in the manuscript – I believe it is crucial to show these results to interested reader to comprehend the link between the previous and the current work.

We agree with the reviewer that these data are important for linking our previous and current work. We have now incorporated the key data (R2b and R2c) into the manuscript as new Extended Data Fig. 12 and have included the corresponding discussion in the revised manuscript.

Minor
• I simply don’t see enough connection to highlight “translation control” in the title – maybe the new data would rather suggest a link to “oxidative stress response”

We have now strengthened the “translational control” aspect of the manuscript with the new experiment linking SerRS–SIRT2 complex formation to translational control under oxidative stress (new Fig. 6d and 6e).

• I would suggest to mention the overall resolution in the abstract (3.9 Å) or leave the resolution out at all.

We have now removed the structural resolution from the Abstract.

• Table 1 – I think “X” should be replaced by “×”
Thanks for point it out. They have been corrected as suggested.

Reviewer #4 (Remarks to the Author):

1. Although the authors have performed more quantitative analyses of ligand-dependent SIRT2–SerRS complex formation (Figs. 2e and 2h), they did not provide a direct answer to my previous question: why can these proteins still form a complex in the absence of ADPR (Figs. 4c,d), which appears to contradict the results in Fig. 1c and Fig. 2h? These inconsistencies should be addressed.

These observations can be reconciled by considering the differences in sensitivity, stringency, and equilibrium conditions across the assays used. Our cryo-EM structures reveal that the SerRS–SIRT2 interface comprises two regions: the major loop (centered on K414), which forms an ADPR-dependent interaction and drives stable complex formation, and the minor loop, which contributes additional ADPR-independent contacts that are insufficient on their own to support a stable complex (Fig. 2a; Fig. 5a–c).

In the absence of ADPR, SerRS and SIRT2 likely exist in a weak or transiently associated state mediated by the minor loop, falling below the detection threshold of methods performed at lower concentrations or under equilibrium dilution conditions. In mass photometry (Fig. 2h), performed at 20 nM SerRS and 80 nM SIRT2, no complex is detected without ADPR. Similarly, SEC (Fig. 1c) involves progressive dilution during chromatography, such that only sufficiently stable complexes co-migrate as a distinct peak.

In contrast, pull-down assays involve surface immobilization, which creates high local concentrations and shifts the equilibrium toward the capture of transient or low-affinity encounters that would otherwise dissociate freely in solution. Under the relatively high protein concentrations used (150 nM SerRS and 175 nM SIRT2; Figs. 2e and 4c, d), a low but detectable level of ADPR-independent interaction is observed. Importantly, this does not reflect stable complex formation, but rather the inherent ability of pull-down assays to enrich weak, transient binding events.

Taken together, the results are not contradictory but reflect a difference of detection sensitivity. ADPR engagement of the major loop is required to stabilize a robust SerRS–SIRT2 complex, while the minor loop mediates a residual weak interaction only detectable under the permissive conditions of pull-down assays.

2. The inhibition of deacetylation at [ADPR] = 0 in Fig. 3c should be described in the text to avoid confusion, particularly regarding whether non-enzymatic conversion of OAADPR to ADPR during the deacetylation reaction may contribute to the observed formation of the protein complex. It is also somewhat surprising that the addition of 200 and 400 μM

ADPR has only a limited effect. Would pre-incubation of SIRT2, SerRS, and ADPR prior to substrate addition influence complex formation or enzymatic activity?

First, as suggested, we now revised this part of the manuscript and added a description stating that SerRS inhibits SIRT2 activity in the absence of added ADPR, along with the following discussion: “First, we performed an in vitro deacetylation assay using the Ac-K40-containing α-tubulin peptide as the substrate in the absence of added ADPR. Considering that NAD^+ can promote SerRS–SIRT2 interaction (Figs. 2e and 2h), which would compete with SIRT2 substrates for binding to SIRT2 (Fig. 3a), and that ADPR is generated during the deacetylation reaction (Extended Data Fig. 6), both factors are likely to contribute to the inhibitory effect of SerRS.”

Second, we would like to clarify that the limited additional effect of exogenous ADPR at 200–400 μM was observed under pre-incubation conditions, where SerRS, SIRT2, and ADPR were incubated together prior to substrate addition. Notably, without pre-incubation the effect was even weaker, suggesting that competition from NAD^+ and endogenously generated ADPR buffers the additional effect of exogenously added ADPR. We now clarified this point in Fig. 3 legend of the revised manuscript.

3. The intracellular concentration of NAD^+ in *E. coli* is estimated to be 2–3 mM, whereas that of ADPR is likely to be substantially lower. Given these conditions, why is ADPR co-purified with the protein complex instead of NAD^+, considering that the binding equilibria would be expected to favor NAD^+ at such concentrations?

We would like to respectfully clarify that the premise that intracellular ADPR is substantially lower than NAD^+ in *E. coli* may not be accurate. While intracellular NAD^+ in *E. coli* is reported at approximately 2.6 mM, free ADPR levels in *E. coli* have been measured in the low mM range, which is substantially higher than in human or yeast cells[1]. Furthermore, we speculate that co-overexpression of SerRS and SIRT2 as recombinant proteins is known to induce cellular stress responses in E. coli, which could further elevate ADPR levels through increased NAD^+ turnover.

Beyond the question of intracellular concentrations, ADPR is markedly more effective than NAD^+ at stabilizing the SerRS–SIRT2 complex under comparable concentrations (Fig. 2h). Additionally, co-purification inherently selects for the most stable molecular complex. Even if NAD^+ transiently engages SIRT2 more frequently due to its abundance, the SerRS–SIRT2–ADPR ternary complex is significantly more stable, as evidenced by our structural and biochemical data, and would therefore be preferentially captured during purification.

References

1. Huang N, *et al.* Structure and function of an ADP-ribose-dependent transcriptional regulator of NAD metabolism. *Structure (London, England : 1993)* **17**, 939-951 (2009).

REVIEWERS' COMMENTS

Reviewer #2 (Remarks to the Author):

The authors have made a substantial effort to address the previous comments, and the revised manuscript has been significantly improved. In particular, the additional experiments strengthen the biological relevance of the study and provide further support for the proposed model.

While I still believe that some mechanistic aspects would benefit from a more formal demonstration — particularly regarding the direct causal link between the SerRS–SIRT2 interaction and translational repression, as well as the explanation for the apparent discrepancy with the authors' previous work, I appreciate that the authors have openly acknowledged these limitations and have provided additional evidence supporting their hypothesis.

Overall, I believe the current data are consistent with the proposed model, even if some aspects will likely require further investigation in future studies. I recognize the considerable effort made by the authors during revision and I have no further concerns.

We thank the reviewer for the thorough and balanced assessment of our revised manuscript. We sincerely appreciate the reviewer's recognition of our efforts during revision and their constructive engagement throughout. The insightful comments have been invaluable in improving this work. We agree that the mechanistic aspects raised deserve further investigation, and we look forward to exploring these questions in future studies.

Reviewer #4 (Remarks to the Author):

The authors have fully addressed my comments. I recommend accepting this revised manuscript for publication in Nature Communications.

We thank the reviewer for their constructive engagement throughout the review process.
