# Supplementary material for: A metabolite-bridged complex between SerRS and SIRT2 couples NAD⁺ metabolism to translation control
Source: Nat Commun. 2026 Jul 8;17:8427. doi: 10.1038/s41467-026-75266-4 (PMC13478228; doi:10.1038/s41467-026-75266-4)
Supplement: Supplementary file 4 [file 41467_2026_75266_MOESM4_ESM.zip › Source_data_Trypsin Digestion Mass Spectrometry_SeRS_SIRT2.pdf]

Mascot Search Results

Protein View

Match to: **AABT7310_SerRS** Score: **3253**
AABT7310_SerRS
Found in search of \\scarletvault.scripps.edu\userdata4\QE_data\2022\Nov 2022\11302022\AABT7571.raw

Nominal mass (M_r): **59253**; Calculated pI value: **6.05**
NCBI BLAST search of AABT7310_SerRS against nr
Unformatted sequence string for pasting into other applications

Fixed modifications: Carbamidomethyl (C)
Variable modifications: Acetylation (K),Myristoylation_K (K),Oxidation (M),Palmitoylation_K (K)
Cleavage by Trypsin: cuts C-term side of KR unless next residue is P
Sequence Coverage: **79%**

Matched peptides shown in **Bold Red**

```
  1 MVLDLDLFRV DKGGDPALIR ETQEKRFKDP GLVDQLVKAD SEWRRCRFRA
 51 DNLNKLKNLC SKTIGEKMKK KEPVGDDESV PENVLSFDDL TADALANLKV
101 SQIKKVRLLI DEAILKCDAE RIKLEAERFE NLREIGNLLH PSVPISNDED
151 VDNKVERIWG DCTVRKKYSH VDLVVMVDGF EGEKGAVVAG SRGYFLKGVL
201 VFLEQALIQY ALRTLGSRGY IPIYTPFFMR KEVMQEVAQL SQFDEELYKV
251 IGKGSEKSDD NSYDEKYLIA TSEQPIAALH RDEWLRPEDL PIKYAGLSTC
301 FRQEVGSHGR DTRGIFRVHQ FEKIEQFVYS SPHDNKSWEM FEEMITTAEE
351 FYQSLGIPYH IVNIVSGSLN HAASKKLDLE AWFPGSGAFR ELVSCSNCTD
401 YQARRLRIRY GQTKKMMDKV EFVHMLNATM CATTRTICAI LENYQTEKGI
451 TVPEKLKEFM PPGLQELIPF VKPAPIEQEP SKKQKKQHEG SKKKAAARDV
501 TLENRLQNME VTDA
```

Show predicted peptides also

Sort Peptides By ◉ Residue Number ○ Increasing Mass ○ Decreasing Mass

Start - End	Observed	Mr(expt)	Mr(calc)	ppm	Miss	Sequence	
1 - 9	561.3057	1120.5968	1120.5951	2	0	-.MVLDLDLFR.V	(Ions score 42)
1 - 9	561.3060	1120.5975	1120.5951	2	0	-.MVLDLDLFR.V	(Ions score 50)
1 - 9	569.3022	1136.5899	1136.5900	-0	0	-.MVLDLDLFR.V	Oxidation (M) (Ions score 33)
1 - 9	569.3031	1136.5917	1136.5900	2	0	-.MVLDLDLFR.V	Oxidation (M) (Ions score 36)
1 - 9	569.3033	1136.5920	1136.5900	2	0	-.MVLDLDLFR.V	Oxidation (M) (Ions score 36)
2 - 9	495.7835	989.5525	989.5546	-2	0	M.VLDLDLFR.V	(Ions score 47)
2 - 9	495.7844	989.5542	989.5546	-0	0	M.VLDLDLFR.V	(Ions score 39)
2 - 9	495.7845	989.5544	989.5546	-0	0	M.VLDLDLFR.V	(Ions score 35)
2 - 9	495.7846	989.5547	989.5546	0	0	M.VLDLDLFR.V	(Ions score 39)
2 - 9	495.7847	989.5549	989.5546	0	0	M.VLDLDLFR.V	(Ions score 40)
2 - 9	495.7847	989.5549	989.5546	0	0	M.VLDLDLFR.V	(Ions score 39)
2 - 9	495.7848	989.5550	989.5546	0	0	M.VLDLDLFR.V	(Ions score 35)
2 - 9	495.7848	989.5550	989.5546	0	0	M.VLDLDLFR.V	(Ions score 36)
2 - 9	495.7848	989.5551	989.5546	1	0	M.VLDLDLFR.V	(Ions score 24)
2 - 9	495.7849	989.5552	989.5546	1	0	M.VLDLDLFR.V	(Ions score 40)
2 - 9	495.7849	989.5552	989.5546	1	0	M.VLDLDLFR.V	(Ions score 40)
2 - 9	495.7850	989.5555	989.5546	1	0	M.VLDLDLFR.V	(Ions score 35)
2 - 9	495.7850	989.5555	989.5546	1	0	M.VLDLDLFR.V	(Ions score 40)
2 - 9	495.7850	989.5555	989.5546	1	0	M.VLDLDLFR.V	(Ions score 42)
2 - 9	495.7851	989.5556	989.5546	1	0	M.VLDLDLFR.V	(Ions score 39)
2 - 9	495.7851	989.5556	989.5546	1	0	M.VLDLDLFR.V	(Ions score 36)
2 - 9	495.7851	989.5556	989.5546	1	0	M.VLDLDLFR.V	(Ions score 42)
2 - 9	495.7851	989.5556	989.5546	1	0	M.VLDLDLFR.V	(Ions score 35)

2 - 9	495.7851	989.5557	989.5546	1	0	M.VLDLDLFR.V	(Ions score 36)
2 - 9	495.7852	989.5558	989.5546	1	0	M.VLDLDLFR.V	(Ions score 36)
2 - 9	495.7852	989.5558	989.5546	1	0	M.VLDLDLFR.V	(Ions score 35)
2 - 9	495.7852	989.5558	989.5546	1	0	M.VLDLDLFR.V	(Ions score 39)
2 - 9	495.7852	989.5558	989.5546	1	0	M.VLDLDLFR.V	(Ions score 35)
2 - 9	495.7852	989.5558	989.5546	1	0	M.VLDLDLFR.V	(Ions score 39)
2 - 9	495.7852	989.5558	989.5546	1	0	M.VLDLDLFR.V	(Ions score 39)
2 - 9	495.7852	989.5558	989.5546	1	0	M.VLDLDLFR.V	(Ions score 33)
2 - 9	495.7852	989.5558	989.5546	1	0	M.VLDLDLFR.V	(Ions score 35)
2 - 9	495.7852	989.5558	989.5546	1	0	M.VLDLDLFR.V	(Ions score 39)
2 - 9	495.7852	989.5559	989.5546	1	0	M.VLDLDLFR.V	(Ions score 40)
2 - 9	495.7852	989.5559	989.5546	1	0	M.VLDLDLFR.V	(Ions score 39)
2 - 9	495.7852	989.5559	989.5546	1	0	M.VLDLDLFR.V	(Ions score 35)
2 - 9	495.7852	989.5559	989.5546	1	0	M.VLDLDLFR.V	(Ions score 35)
2 - 9	495.7852	989.5559	989.5546	1	0	M.VLDLDLFR.V	(Ions score 35)
2 - 9	495.7852	989.5559	989.5546	1	0	M.VLDLDLFR.V	(Ions score 39)
2 - 9	495.7852	989.5559	989.5546	1	0	M.VLDLDLFR.V	(Ions score 35)
2 - 9	495.7852	989.5559	989.5546	1	0	M.VLDLDLFR.V	(Ions score 35)
2 - 9	495.7852	989.5559	989.5546	1	0	M.VLDLDLFR.V	(Ions score 35)
2 - 9	495.7852	989.5559	989.5546	1	0	M.VLDLDLFR.V	(Ions score 34)
2 - 9	495.7852	989.5559	989.5546	1	0	M.VLDLDLFR.V	(Ions score 53)
2 - 9	495.7852	989.5559	989.5546	1	0	M.VLDLDLFR.V	(Ions score 39)
2 - 9	495.7852	989.5559	989.5546	1	0	M.VLDLDLFR.V	(Ions score 40)
2 - 9	495.7853	989.5560	989.5546	1	0	M.VLDLDLFR.V	(Ions score 35)
2 - 9	495.7853	989.5560	989.5546	1	0	M.VLDLDLFR.V	(Ions score 39)
2 - 9	495.7853	989.5560	989.5546	1	0	M.VLDLDLFR.V	(Ions score 40)
2 - 9	495.7853	989.5561	989.5546	1	0	M.VLDLDLFR.V	(Ions score 39)
2 - 9	495.7853	989.5561	989.5546	2	0	M.VLDLDLFR.V	(Ions score 39)
2 - 9	495.7853	989.5561	989.5546	2	0	M.VLDLDLFR.V	(Ions score 39)
2 - 9	495.7853	989.5561	989.5546	2	0	M.VLDLDLFR.V	(Ions score 35)
2 - 9	495.7853	989.5561	989.5546	2	0	M.VLDLDLFR.V	(Ions score 40)
2 - 9	495.7853	989.5561	989.5546	2	0	M.VLDLDLFR.V	(Ions score 40)
2 - 9	495.7853	989.5561	989.5546	2	0	M.VLDLDLFR.V	(Ions score 36)
2 - 9	495.7853	989.5561	989.5546	2	0	M.VLDLDLFR.V	(Ions score 40)
2 - 9	495.7853	989.5561	989.5546	2	0	M.VLDLDLFR.V	(Ions score 39)
2 - 9	495.7853	989.5561	989.5546	2	0	M.VLDLDLFR.V	(Ions score 39)
2 - 9	495.7853	989.5561	989.5546	2	0	M.VLDLDLFR.V	(Ions score 39)
2 - 9	495.7853	989.5561	989.5546	2	0	M.VLDLDLFR.V	(Ions score 35)
2 - 9	495.7853	989.5561	989.5546	2	0	M.VLDLDLFR.V	(Ions score 40)
2 - 9	495.7854	989.5562	989.5546	2	0	M.VLDLDLFR.V	(Ions score 41)
2 - 9	495.7854	989.5562	989.5546	2	0	M.VLDLDLFR.V	(Ions score 39)
2 - 9	495.7854	989.5562	989.5546	2	0	M.VLDLDLFR.V	(Ions score 39)
2 - 9	495.7854	989.5562	989.5546	2	0	M.VLDLDLFR.V	(Ions score 39)
2 - 9	495.7854	989.5562	989.5546	2	0	M.VLDLDLFR.V	(Ions score 40)
2 - 9	495.7854	989.5562	989.5546	2	0	M.VLDLDLFR.V	(Ions score 39)
2 - 9	495.7854	989.5562	989.5546	2	0	M.VLDLDLFR.V	(Ions score 35)
2 - 9	495.7854	989.5562	989.5546	2	0	M.VLDLDLFR.V	(Ions score 39)
2 - 9	495.7854	989.5562	989.5546	2	0	M.VLDLDLFR.V	(Ions score 40)
2 - 9	495.7854	989.5562	989.5546	2	0	M.VLDLDLFR.V	(Ions score 39)
2 - 9	495.7854	989.5562	989.5546	2	0	M.VLDLDLFR.V	(Ions score 40)
2 - 9	495.7854	989.5562	989.5546	2	0	M.VLDLDLFR.V	(Ions score 39)
2 - 9	495.7854	989.5562	989.5546	2	0	M.VLDLDLFR.V	(Ions score 35)
2 - 9	495.7854	989.5562	989.5546	2	0	M.VLDLDLFR.V	(Ions score 39)
2 - 9	495.7854	989.5562	989.5546	2	0	M.VLDLDLFR.V	(Ions score 35)
2 - 9	495.7854	989.5562	989.5546	2	0	M.VLDLDLFR.V	(Ions score 40)
2 - 9	495.7854	989.5562	989.5546	2	0	M.VLDLDLFR.V	(Ions score 39)
2 - 9	495.7854	989.5562	989.5546	2	0	M.VLDLDLFR.V	(Ions score 40)

2 - 9	495.7854	989.5563	989.5546	2	0	M.VLDLDLFR.V	(Ions score 40)
2 - 9	495.7854	989.5563	989.5546	2	0	M.VLDLDLFR.V	(Ions score 40)
2 - 9	495.7854	989.5563	989.5546	2	0	M.VLDLDLFR.V	(Ions score 35)
2 - 9	495.7854	989.5563	989.5546	2	0	M.VLDLDLFR.V	(Ions score 39)
2 - 9	495.7854	989.5563	989.5546	2	0	M.VLDLDLFR.V	(Ions score 39)
2 - 9	495.7854	989.5563	989.5546	2	0	M.VLDLDLFR.V	(Ions score 35)
2 - 9	495.7855	989.5564	989.5546	2	0	M.VLDLDLFR.V	(Ions score 39)
2 - 9	495.7855	989.5564	989.5546	2	0	M.VLDLDLFR.V	(Ions score 36)
2 - 9	495.7855	989.5564	989.5546	2	0	M.VLDLDLFR.V	(Ions score 40)
2 - 9	495.7855	989.5564	989.5546	2	0	M.VLDLDLFR.V	(Ions score 39)
2 - 9	495.7855	989.5564	989.5546	2	0	M.VLDLDLFR.V	(Ions score 36)
2 - 9	495.7855	989.5564	989.5546	2	0	M.VLDLDLFR.V	(Ions score 35)
2 - 9	495.7855	989.5564	989.5546	2	0	M.VLDLDLFR.V	(Ions score 40)
2 - 9	495.7855	989.5564	989.5546	2	0	M.VLDLDLFR.V	(Ions score 36)
2 - 9	495.7855	989.5564	989.5546	2	0	M.VLDLDLFR.V	(Ions score 39)
2 - 9	495.7855	989.5564	989.5546	2	0	M.VLDLDLFR.V	(Ions score 39)
2 - 9	495.7855	989.5565	989.5546	2	0	M.VLDLDLFR.V	(Ions score 40)
2 - 9	495.7855	989.5565	989.5546	2	0	M.VLDLDLFR.V	(Ions score 35)
2 - 9	495.7855	989.5565	989.5546	2	0	M.VLDLDLFR.V	(Ions score 39)
2 - 9	495.7855	989.5565	989.5546	2	0	M.VLDLDLFR.V	(Ions score 40)
2 - 9	495.7856	989.5566	989.5546	2	0	M.VLDLDLFR.V	(Ions score 35)
2 - 9	495.7856	989.5566	989.5546	2	0	M.VLDLDLFR.V	(Ions score 39)
2 - 9	495.7856	989.5566	989.5546	2	0	M.VLDLDLFR.V	(Ions score 39)
2 - 9	495.7856	989.5566	989.5546	2	0	M.VLDLDLFR.V	(Ions score 36)
2 - 9	495.7856	989.5566	989.5546	2	0	M.VLDLDLFR.V	(Ions score 35)
2 - 9	495.7856	989.5566	989.5546	2	0	M.VLDLDLFR.V	(Ions score 35)
2 - 9	495.7856	989.5566	989.5546	2	0	M.VLDLDLFR.V	(Ions score 39)
2 - 9	495.7856	989.5566	989.5546	2	0	M.VLDLDLFR.V	(Ions score 35)
2 - 9	495.7856	989.5566	989.5546	2	0	M.VLDLDLFR.V	(Ions score 35)
2 - 9	495.7856	989.5566	989.5546	2	0	M.VLDLDLFR.V	(Ions score 35)
2 - 9	495.7856	989.5566	989.5546	2	0	M.VLDLDLFR.V	(Ions score 35)
2 - 9	495.7856	989.5566	989.5546	2	0	M.VLDLDLFR.V	(Ions score 40)
2 - 9	495.7856	989.5566	989.5546	2	0	M.VLDLDLFR.V	(Ions score 39)
2 - 9	495.7856	989.5566	989.5546	2	0	M.VLDLDLFR.V	(Ions score 35)
2 - 9	495.7856	989.5566	989.5546	2	0	M.VLDLDLFR.V	(Ions score 35)
2 - 9	495.7856	989.5566	989.5546	2	0	M.VLDLDLFR.V	(Ions score 35)
2 - 9	495.7856	989.5566	989.5546	2	0	M.VLDLDLFR.V	(Ions score 40)
2 - 9	495.7856	989.5567	989.5546	2	0	M.VLDLDLFR.V	(Ions score 22)
2 - 9	495.7856	989.5567	989.5546	2	0	M.VLDLDLFR.V	(Ions score 39)
2 - 9	495.7856	989.5567	989.5546	2	0	M.VLDLDLFR.V	(Ions score 35)
2 - 9	495.7856	989.5567	989.5546	2	0	M.VLDLDLFR.V	(Ions score 39)
2 - 9	495.7856	989.5567	989.5546	2	0	M.VLDLDLFR.V	(Ions score 35)
2 - 9	495.7856	989.5567	989.5546	2	0	M.VLDLDLFR.V	(Ions score 35)
2 - 9	495.7856	989.5567	989.5546	2	0	M.VLDLDLFR.V	(Ions score 39)
2 - 9	495.7856	989.5567	989.5546	2	0	M.VLDLDLFR.V	(Ions score 35)
2 - 9	495.7857	989.5568	989.5546	2	0	M.VLDLDLFR.V	(Ions score 39)
2 - 9	495.7857	989.5568	989.5546	2	0	M.VLDLDLFR.V	(Ions score 35)
2 - 9	495.7857	989.5568	989.5546	2	0	M.VLDLDLFR.V	(Ions score 35)
2 - 9	495.7857	989.5568	989.5546	2	0	M.VLDLDLFR.V	(Ions score 35)
2 - 9	495.7857	989.5569	989.5546	2	0	M.VLDLDLFR.V	(Ions score 39)
2 - 9	495.7857	989.5569	989.5546	2	0	M.VLDLDLFR.V	(Ions score 39)
2 - 9	495.7857	989.5569	989.5546	2	0	M.VLDLDLFR.V	(Ions score 35)
2 - 9	495.7857	989.5569	989.5546	2	0	M.VLDLDLFR.V	(Ions score 39)
2 - 9	495.7857	989.5569	989.5546	2	0	M.VLDLDLFR.V	(Ions score 36)
2 - 9	495.7857	989.5569	989.5546	2	0	M.VLDLDLFR.V	(Ions score 39)
2 - 9	495.7857	989.5569	989.5546	2	0	M.VLDLDLFR.V	(Ions score 35)
2 - 9	495.7858	989.5570	989.5546	2	0	M.VLDLDLFR.V	(Ions score 36)

2 - 9	495.7858	989.5570	989.5546	2	0	M.VLDLDLFR.V	(Ions score 35)
2 - 9	495.7858	989.5570	989.5546	2	0	M.VLDLDLFR.V	(Ions score 35)
2 - 9	495.7858	989.5570	989.5546	2	0	M.VLDLDLFR.V	(Ions score 39)
2 - 9	495.7858	989.5570	989.5546	2	0	M.VLDLDLFR.V	(Ions score 35)
2 - 9	495.7858	989.5570	989.5546	2	0	M.VLDLDLFR.V	(Ions score 40)
2 - 9	495.7858	989.5570	989.5546	2	0	M.VLDLDLFR.V	(Ions score 36)
2 - 9	495.7858	989.5570	989.5546	2	0	M.VLDLDLFR.V	(Ions score 25)
2 - 9	495.7858	989.5570	989.5546	2	0	M.VLDLDLFR.V	(Ions score 40)
2 - 9	495.7858	989.5571	989.5546	3	0	M.VLDLDLFR.V	(Ions score 33)
2 - 9	495.7858	989.5571	989.5546	3	0	M.VLDLDLFR.V	(Ions score 40)
2 - 9	495.7858	989.5571	989.5546	3	0	M.VLDLDLFR.V	(Ions score 40)
2 - 9	495.7858	989.5571	989.5546	3	0	M.VLDLDLFR.V	(Ions score 36)
2 - 9	495.7858	989.5571	989.5546	3	0	M.VLDLDLFR.V	(Ions score 39)
2 - 9	495.7858	989.5571	989.5546	3	0	M.VLDLDLFR.V	(Ions score 35)
2 - 9	495.7858	989.5571	989.5546	3	0	M.VLDLDLFR.V	(Ions score 35)
2 - 9	495.7859	989.5572	989.5546	3	0	M.VLDLDLFR.V	(Ions score 39)
2 - 9	495.7859	989.5572	989.5546	3	0	M.VLDLDLFR.V	(Ions score 39)
2 - 9	495.7859	989.5572	989.5546	3	0	M.VLDLDLFR.V	(Ions score 39)
2 - 9	495.7859	989.5572	989.5546	3	0	M.VLDLDLFR.V	(Ions score 39)
2 - 9	495.7859	989.5572	989.5546	3	0	M.VLDLDLFR.V	(Ions score 35)
2 - 9	495.7859	989.5572	989.5546	3	0	M.VLDLDLFR.V	(Ions score 39)
2 - 9	495.7859	989.5572	989.5546	3	0	M.VLDLDLFR.V	(Ions score 39)
2 - 9	495.7859	989.5572	989.5546	3	0	M.VLDLDLFR.V	(Ions score 33)
2 - 9	495.7859	989.5572	989.5546	3	0	M.VLDLDLFR.V	(Ions score 39)
2 - 9	495.7859	989.5572	989.5546	3	0	M.VLDLDLFR.V	(Ions score 39)
2 - 9	495.7859	989.5572	989.5546	3	0	M.VLDLDLFR.V	(Ions score 39)
2 - 9	495.7859	989.5572	989.5546	3	0	M.VLDLDLFR.V	(Ions score 39)
2 - 9	495.7859	989.5572	989.5546	3	0	M.VLDLDLFR.V	(Ions score 44)
2 - 9	495.7859	989.5573	989.5546	3	0	M.VLDLDLFR.V	(Ions score 40)
2 - 9	495.7859	989.5573	989.5546	3	0	M.VLDLDLFR.V	(Ions score 36)
2 - 9	495.7859	989.5573	989.5546	3	0	M.VLDLDLFR.V	(Ions score 35)
2 - 9	495.7859	989.5573	989.5546	3	0	M.VLDLDLFR.V	(Ions score 39)
2 - 9	495.7859	989.5573	989.5546	3	0	M.VLDLDLFR.V	(Ions score 35)
2 - 9	495.7859	989.5573	989.5546	3	0	M.VLDLDLFR.V	(Ions score 36)
2 - 9	495.7860	989.5573	989.5546	3	0	M.VLDLDLFR.V	(Ions score 40)
2 - 9	495.7860	989.5573	989.5546	3	0	M.VLDLDLFR.V	(Ions score 40)
2 - 9	495.7860	989.5573	989.5546	3	0	M.VLDLDLFR.V	(Ions score 35)
2 - 9	495.7860	989.5573	989.5546	3	0	M.VLDLDLFR.V	(Ions score 39)
2 - 9	495.7860	989.5573	989.5546	3	0	M.VLDLDLFR.V	(Ions score 39)
2 - 9	495.7860	989.5573	989.5546	3	0	M.VLDLDLFR.V	(Ions score 22)
2 - 9	495.7860	989.5574	989.5546	3	0	M.VLDLDLFR.V	(Ions score 40)
2 - 9	495.7860	989.5574	989.5546	3	0	M.VLDLDLFR.V	(Ions score 53)
2 - 9	495.7860	989.5574	989.5546	3	0	M.VLDLDLFR.V	(Ions score 51)
2 - 9	495.7860	989.5574	989.5546	3	0	M.VLDLDLFR.V	(Ions score 40)
2 - 9	495.7860	989.5574	989.5546	3	0	M.VLDLDLFR.V	(Ions score 39)
2 - 9	495.7860	989.5574	989.5546	3	0	M.VLDLDLFR.V	(Ions score 39)
2 - 9	495.7860	989.5574	989.5546	3	0	M.VLDLDLFR.V	(Ions score 36)
2 - 9	495.7860	989.5574	989.5546	3	0	M.VLDLDLFR.V	(Ions score 39)
2 - 9	495.7860	989.5574	989.5546	3	0	M.VLDLDLFR.V	(Ions score 35)
2 - 9	495.7860	989.5574	989.5546	3	0	M.VLDLDLFR.V	(Ions score 49)
2 - 9	495.7860	989.5575	989.5546	3	0	M.VLDLDLFR.V	(Ions score 35)
2 - 9	495.7860	989.5575	989.5546	3	0	M.VLDLDLFR.V	(Ions score 39)
2 - 9	495.7860	989.5575	989.5546	3	0	M.VLDLDLFR.V	(Ions score 35)
2 - 9	495.7860	989.5575	989.5546	3	0	M.VLDLDLFR.V	(Ions score 39)
2 - 9	495.7860	989.5575	989.5546	3	0	M.VLDLDLFR.V	(Ions score 35)
2 - 9	495.7860	989.5575	989.5546	3	0	M.VLDLDLFR.V	(Ions score 41)
2 - 9	495.7860	989.5575	989.5546	3	0	M.VLDLDLFR.V	(Ions score 40)

2 - 9	495.7860	989.5575	989.5546	3	0	M.VLDLDLFR.V	(Ions score 40)
2 - 9	495.7860	989.5575	989.5546	3	0	M.VLDLDLFR.V	(Ions score 40)
2 - 9	495.7860	989.5575	989.5546	3	0	M.VLDLDLFR.V	(Ions score 39)
2 - 9	495.7860	989.5575	989.5546	3	0	M.VLDLDLFR.V	(Ions score 32)
2 - 9	495.7860	989.5575	989.5546	3	0	M.VLDLDLFR.V	(Ions score 35)
2 - 9	495.7860	989.5575	989.5546	3	0	M.VLDLDLFR.V	(Ions score 39)
2 - 9	495.7860	989.5575	989.5546	3	0	M.VLDLDLFR.V	(Ions score 44)
2 - 9	495.7861	989.5576	989.5546	3	0	M.VLDLDLFR.V	(Ions score 41)
2 - 9	495.7861	989.5576	989.5546	3	0	M.VLDLDLFR.V	(Ions score 40)
2 - 9	495.7861	989.5576	989.5546	3	0	M.VLDLDLFR.V	(Ions score 39)
2 - 9	495.7861	989.5576	989.5546	3	0	M.VLDLDLFR.V	(Ions score 35)
2 - 9	495.7861	989.5576	989.5546	3	0	M.VLDLDLFR.V	(Ions score 40)
2 - 9	495.7861	989.5577	989.5546	3	0	M.VLDLDLFR.V	(Ions score 40)
2 - 9	495.7861	989.5577	989.5546	3	0	M.VLDLDLFR.V	(Ions score 36)
2 - 9	495.7861	989.5577	989.5546	3	0	M.VLDLDLFR.V	(Ions score 39)
2 - 9	495.7861	989.5577	989.5546	3	0	M.VLDLDLFR.V	(Ions score 36)
2 - 9	495.7861	989.5577	989.5546	3	0	M.VLDLDLFR.V	(Ions score 39)
2 - 9	495.7861	989.5577	989.5546	3	0	M.VLDLDLFR.V	(Ions score 39)
2 - 9	495.7861	989.5577	989.5546	3	0	M.VLDLDLFR.V	(Ions score 72)
2 - 9	495.7861	989.5577	989.5546	3	0	M.VLDLDLFR.V	(Ions score 39)
2 - 9	495.7861	989.5577	989.5546	3	0	M.VLDLDLFR.V	(Ions score 40)
2 - 9	495.7862	989.5578	989.5546	3	0	M.VLDLDLFR.V	(Ions score 42)
2 - 9	495.7862	989.5578	989.5546	3	0	M.VLDLDLFR.V	(Ions score 36)
2 - 9	495.7862	989.5578	989.5546	3	0	M.VLDLDLFR.V	(Ions score 39)
2 - 9	495.7862	989.5578	989.5546	3	0	M.VLDLDLFR.V	(Ions score 39)
2 - 9	495.7862	989.5578	989.5546	3	0	M.VLDLDLFR.V	(Ions score 40)
2 - 9	495.7862	989.5578	989.5546	3	0	M.VLDLDLFR.V	(Ions score 48)
2 - 9	495.7862	989.5578	989.5546	3	0	M.VLDLDLFR.V	(Ions score 39)
2 - 9	495.7862	989.5579	989.5546	3	0	M.VLDLDLFR.V	(Ions score 42)
2 - 9	495.7862	989.5579	989.5546	3	0	M.VLDLDLFR.V	(Ions score 39)
2 - 9	495.7862	989.5579	989.5546	3	0	M.VLDLDLFR.V	(Ions score 25)
2 - 9	495.7862	989.5579	989.5546	3	0	M.VLDLDLFR.V	(Ions score 39)
2 - 9	495.7862	989.5579	989.5546	3	0	M.VLDLDLFR.V	(Ions score 38)
2 - 9	495.7863	989.5580	989.5546	3	0	M.VLDLDLFR.V	(Ions score 35)
2 - 9	495.7863	989.5580	989.5546	3	0	M.VLDLDLFR.V	(Ions score 40)
2 - 9	495.7863	989.5580	989.5546	3	0	M.VLDLDLFR.V	(Ions score 39)
2 - 9	495.7863	989.5580	989.5546	3	0	M.VLDLDLFR.V	(Ions score 35)
2 - 9	495.7863	989.5581	989.5546	4	0	M.VLDLDLFR.V	(Ions score 52)
2 - 9	495.7863	989.5581	989.5546	4	0	M.VLDLDLFR.V	(Ions score 35)
2 - 9	495.7863	989.5581	989.5546	4	0	M.VLDLDLFR.V	(Ions score 39)
2 - 9	495.7863	989.5581	989.5546	4	0	M.VLDLDLFR.V	(Ions score 40)
2 - 9	495.7863	989.5581	989.5546	4	0	M.VLDLDLFR.V	(Ions score 40)
2 - 9	495.7863	989.5581	989.5546	4	0	M.VLDLDLFR.V	(Ions score 39)
2 - 9	495.7863	989.5581	989.5546	4	0	M.VLDLDLFR.V	(Ions score 39)
2 - 9	495.7863	989.5581	989.5546	4	0	M.VLDLDLFR.V	(Ions score 39)
2 - 9	495.7863	989.5581	989.5546	4	0	M.VLDLDLFR.V	(Ions score 40)
2 - 9	495.7864	989.5581	989.5546	4	0	M.VLDLDLFR.V	(Ions score 40)
2 - 9	495.7864	989.5582	989.5546	4	0	M.VLDLDLFR.V	(Ions score 39)
2 - 9	495.7864	989.5582	989.5546	4	0	M.VLDLDLFR.V	(Ions score 39)
2 - 9	495.7864	989.5582	989.5546	4	0	M.VLDLDLFR.V	(Ions score 35)
2 - 9	495.7864	989.5582	989.5546	4	0	M.VLDLDLFR.V	(Ions score 47)
2 - 9	495.7864	989.5582	989.5546	4	0	M.VLDLDLFR.V	(Ions score 42)
2 - 9	495.7864	989.5582	989.5546	4	0	M.VLDLDLFR.V	(Ions score 41)
2 - 9	495.7864	989.5583	989.5546	4	0	M.VLDLDLFR.V	(Ions score 40)
2 - 9	495.7864	989.5583	989.5546	4	0	M.VLDLDLFR.V	(Ions score 39)
2 - 9	495.7864	989.5583	989.5546	4	0	M.VLDLDLFR.V	(Ions score 36)
2 - 9	495.7864	989.5583	989.5546	4	0	M.VLDLDLFR.V	(Ions score 41)

2 - 9	495.7864	989.5583	989.5546	4	0	M.VLDLDLFR.V		(Ions score 39)
2 - 9	495.7864	989.5583	989.5546	4	0	M.VLDLDLFR.V		(Ions score 40)
2 - 9	495.7864	989.5583	989.5546	4	0	M.VLDLDLFR.V		(Ions score 35)
2 - 9	495.7864	989.5583	989.5546	4	0	M.VLDLDLFR.V		(Ions score 42)
2 - 9	495.7865	989.5584	989.5546	4	0	M.VLDLDLFR.V		(Ions score 39)
2 - 9	495.7865	989.5584	989.5546	4	0	M.VLDLDLFR.V		(Ions score 39)
2 - 9	495.7865	989.5584	989.5546	4	0	M.VLDLDLFR.V		(Ions score 40)
2 - 9	495.7865	989.5585	989.5546	4	0	M.VLDLDLFR.V		(Ions score 41)
2 - 9	495.7865	989.5585	989.5546	4	0	M.VLDLDLFR.V		(Ions score 41)
2 - 9	495.7865	989.5585	989.5546	4	0	M.VLDLDLFR.V		(Ions score 40)
2 - 9	495.7866	989.5586	989.5546	4	0	M.VLDLDLFR.V		(Ions score 40)
2 - 9	495.7867	989.5588	989.5546	4	0	M.VLDLDLFR.V		(Ions score 39)
2 - 9	495.7867	989.5589	989.5546	4	0	M.VLDLDLFR.V		(Ions score 39)
2 - 9	495.7868	989.5591	989.5546	5	0	M.VLDLDLFR.V		(Ions score 35)
2 - 9	495.7868	989.5591	989.5546	5	0	M.VLDLDLFR.V		(Ions score 38)
2 - 9	495.7869	989.5592	989.5546	5	0	M.VLDLDLFR.V		(Ions score 39)
2 - 9	495.7869	989.5592	989.5546	5	0	M.VLDLDLFR.V		(Ions score 43)
2 - 9	495.7869	989.5592	989.5546	5	0	M.VLDLDLFR.V		(Ions score 39)
2 - 9	495.7869	989.5592	989.5546	5	0	M.VLDLDLFR.V		(Ions score 39)
2 - 12	444.9233	1331.7480	1331.7449	2	1	M.VLDLDLFRVDK.G		(Ions score 32)
2 - 12	444.9236	1331.7489	1331.7449	3	1	M.VLDLDLFRVDK.G		(Ions score 26)
10 - 20	1140.6368	1139.6296	1139.6299	-0	1	R.VDKGGDPALIR.E		(Ions score 37)
10 - 20	1140.6377	1139.6304	1139.6299	0	1	R.VDKGGDPALIR.E		(Ions score 57)
10 - 20	1140.6379	1139.6307	1139.6299	1	1	R.VDKGGDPALIR.E		(Ions score 44)
10 - 20	570.8226	1139.6307	1139.6299	1	1	R.VDKGGDPALIR.E		(Ions score 49)
10 - 20	1140.6381	1139.6308	1139.6299	1	1	R.VDKGGDPALIR.E		(Ions score 37)
10 - 20	1140.6385	1139.6313	1139.6299	1	1	R.VDKGGDPALIR.E		(Ions score 37)
10 - 20	570.8230	1139.6315	1139.6299	1	1	R.VDKGGDPALIR.E		(Ions score 47)
10 - 20	570.8230	1139.6315	1139.6299	1	1	R.VDKGGDPALIR.E		(Ions score 61)
10 - 20	1140.6388	1139.6315	1139.6299	1	1	R.VDKGGDPALIR.E		(Ions score 40)
10 - 20	570.8231	1139.6317	1139.6299	2	1	R.VDKGGDPALIR.E		(Ions score 34)
10 - 20	570.8231	1139.6317	1139.6299	2	1	R.VDKGGDPALIR.E		(Ions score 48)
10 - 20	570.8232	1139.6318	1139.6299	2	1	R.VDKGGDPALIR.E		(Ions score 59)
10 - 20	570.8232	1139.6319	1139.6299	2	1	R.VDKGGDPALIR.E		(Ions score 44)
10 - 20	1140.6393	1139.6320	1139.6299	2	1	R.VDKGGDPALIR.E		(Ions score 45)
10 - 20	570.8233	1139.6321	1139.6299	2	1	R.VDKGGDPALIR.E		(Ions score 54)
10 - 20	570.8236	1139.6326	1139.6299	2	1	R.VDKGGDPALIR.E		(Ions score 45)
10 - 20	570.8237	1139.6328	1139.6299	3	1	R.VDKGGDPALIR.E		(Ions score 57)
10 - 20	570.8238	1139.6331	1139.6299	3	1	R.VDKGGDPALIR.E		(Ions score 45)
10 - 20	570.8239	1139.6332	1139.6299	3	1	R.VDKGGDPALIR.E		(Ions score 48)
10 - 20	570.8239	1139.6333	1139.6299	3	1	R.VDKGGDPALIR.E		(Ions score 49)
10 - 20	570.8239	1139.6333	1139.6299	3	1	R.VDKGGDPALIR.E		(Ions score 53)
10 - 20	570.8240	1139.6335	1139.6299	3	1	R.VDKGGDPALIR.E		(Ions score 26)
10 - 20	570.8241	1139.6336	1139.6299	3	1	R.VDKGGDPALIR.E		(Ions score 49)
10 - 20	570.8241	1139.6337	1139.6299	3	1	R.VDKGGDPALIR.E		(Ions score 30)
10 - 20	570.8241	1139.6337	1139.6299	3	1	R.VDKGGDPALIR.E		(Ions score 56)
10 - 20	570.8243	1139.6340	1139.6299	4	1	R.VDKGGDPALIR.E		(Ions score 52)
10 - 20	570.8244	1139.6342	1139.6299	4	1	R.VDKGGDPALIR.E		(Ions score 47)
10 - 20	570.8244	1139.6342	1139.6299	4	1	R.VDKGGDPALIR.E		(Ions score 47)
10 - 20	570.8245	1139.6344	1139.6299	4	1	R.VDKGGDPALIR.E		(Ions score 48)
10 - 20	570.8248	1139.6350	1139.6299	5	1	R.VDKGGDPALIR.E		(Ions score 31)
10 - 20	570.8249	1139.6353	1139.6299	5	1	R.VDKGGDPALIR.E		(Ions score 32)
10 - 20	570.8250	1139.6355	1139.6299	5	1	R.VDKGGDPALIR.E		(Ions score 53)
10 - 20	591.8262	1181.6379	1181.6404	-2	1	R.VDKGGDPALIR.E	Acetylation (K)	(Ions score 30)
10 - 20	591.8280	1181.6415	1181.6404	1	1	R.VDKGGDPALIR.E	Acetylation (K)	(Ions score 32)
10 - 20	591.8282	1181.6419	1181.6404	1	1	R.VDKGGDPALIR.E	Acetylation (K)	(Ions score 24)
10 - 20	591.8283	1181.6420	1181.6404	1	1	R.VDKGGDPALIR.E	Acetylation (K)	(Ions score 33)

10 - 20	591.8283	1181.6420	1181.6404	1	1	R.VDKGGDPALIR.E	Acetylation (K) (Ions score 41)
10 - 20	591.8283	1181.6421	1181.6404	1	1	R.VDKGGDPALIR.E	Acetylation (K) (Ions score 36)
10 - 20	591.8285	1181.6424	1181.6404	2	1	R.VDKGGDPALIR.E	Acetylation (K) (Ions score 35)
10 - 20	591.8285	1181.6424	1181.6404	2	1	R.VDKGGDPALIR.E	Acetylation (K) (Ions score 29)
10 - 20	591.8285	1181.6425	1181.6404	2	1	R.VDKGGDPALIR.E	Acetylation (K) (Ions score 46)
10 - 20	591.8287	1181.6428	1181.6404	2	1	R.VDKGGDPALIR.E	Acetylation (K) (Ions score 41)
10 - 20	591.8288	1181.6430	1181.6404	2	1	R.VDKGGDPALIR.E	Acetylation (K) (Ions score 36)
10 - 20	591.8288	1181.6431	1181.6404	2	1	R.VDKGGDPALIR.E	Acetylation (K) (Ions score 29)
10 - 20	591.8288	1181.6431	1181.6404	2	1	R.VDKGGDPALIR.E	Acetylation (K) (Ions score 46)
10 - 20	591.8296	1181.6446	1181.6404	4	1	R.VDKGGDPALIR.E	Acetylation (K) (Ions score 23)
10 - 20	591.8296	1181.6447	1181.6404	4	1	R.VDKGGDPALIR.E	Acetylation (K) (Ions score 43)
21 - 25	634.3052	633.2979	633.2970	1	0	R.ETQEK.R	(Ions score 23)
27 - 38	679.8859	1357.7573	1357.7606	-2	1	R.FKDPGLVDQLVK.A	(Ions score 27)
27 - 38	679.8868	1357.7591	1357.7606	-1	1	R.FKDPGLVDQLVK.A	(Ions score 27)
27 - 38	453.5938	1357.7597	1357.7606	-1	1	R.FKDPGLVDQLVK.A	(Ions score 45)
27 - 38	453.5938	1357.7597	1357.7606	-1	1	R.FKDPGLVDQLVK.A	(Ions score 51)
27 - 38	453.5939	1357.7599	1357.7606	-0	1	R.FKDPGLVDQLVK.A	(Ions score 46)
27 - 38	679.8873	1357.7600	1357.7606	-0	1	R.FKDPGLVDQLVK.A	(Ions score 25)
27 - 38	453.5940	1357.7603	1357.7606	-0	1	R.FKDPGLVDQLVK.A	(Ions score 35)
27 - 38	453.5941	1357.7603	1357.7606	-0	1	R.FKDPGLVDQLVK.A	(Ions score 50)
27 - 38	453.5941	1357.7604	1357.7606	-0	1	R.FKDPGLVDQLVK.A	(Ions score 43)
27 - 38	453.5941	1357.7604	1357.7606	-0	1	R.FKDPGLVDQLVK.A	(Ions score 38)
27 - 38	453.5941	1357.7606	1357.7606	0	1	R.FKDPGLVDQLVK.A	(Ions score 40)
27 - 38	453.5942	1357.7609	1357.7606	0	1	R.FKDPGLVDQLVK.A	(Ions score 48)
27 - 38	453.5942	1357.7609	1357.7606	0	1	R.FKDPGLVDQLVK.A	(Ions score 53)
27 - 38	453.5943	1357.7610	1357.7606	0	1	R.FKDPGLVDQLVK.A	(Ions score 43)
27 - 38	453.5943	1357.7610	1357.7606	0	1	R.FKDPGLVDQLVK.A	(Ions score 50)
27 - 38	453.5943	1357.7611	1357.7606	0	1	R.FKDPGLVDQLVK.A	(Ions score 34)
27 - 38	453.5943	1357.7611	1357.7606	0	1	R.FKDPGLVDQLVK.A	(Ions score 30)
27 - 38	453.5943	1357.7611	1357.7606	0	1	R.FKDPGLVDQLVK.A	(Ions score 52)
27 - 38	453.5943	1357.7612	1357.7606	0	1	R.FKDPGLVDQLVK.A	(Ions score 41)
27 - 38	679.8879	1357.7612	1357.7606	0	1	R.FKDPGLVDQLVK.A	(Ions score 22)
27 - 38	453.5944	1357.7613	1357.7606	1	1	R.FKDPGLVDQLVK.A	(Ions score 26)
27 - 38	679.8879	1357.7613	1357.7606	1	1	R.FKDPGLVDQLVK.A	(Ions score 30)
27 - 38	453.5944	1357.7613	1357.7606	1	1	R.FKDPGLVDQLVK.A	(Ions score 52)
27 - 38	453.5944	1357.7613	1357.7606	1	1	R.FKDPGLVDQLVK.A	(Ions score 50)
27 - 38	679.8879	1357.7613	1357.7606	1	1	R.FKDPGLVDQLVK.A	(Ions score 41)
27 - 38	453.5944	1357.7613	1357.7606	1	1	R.FKDPGLVDQLVK.A	(Ions score 47)
27 - 38	453.5944	1357.7613	1357.7606	1	1	R.FKDPGLVDQLVK.A	(Ions score 29)
27 - 38	453.5944	1357.7613	1357.7606	1	1	R.FKDPGLVDQLVK.A	(Ions score 48)
27 - 38	453.5944	1357.7613	1357.7606	1	1	R.FKDPGLVDQLVK.A	(Ions score 38)
27 - 38	453.5944	1357.7614	1357.7606	1	1	R.FKDPGLVDQLVK.A	(Ions score 41)
27 - 38	679.8880	1357.7614	1357.7606	1	1	R.FKDPGLVDQLVK.A	(Ions score 21)
27 - 38	679.8880	1357.7615	1357.7606	1	1	R.FKDPGLVDQLVK.A	(Ions score 40)
27 - 38	453.5944	1357.7615	1357.7606	1	1	R.FKDPGLVDQLVK.A	(Ions score 48)
27 - 38	453.5945	1357.7615	1357.7606	1	1	R.FKDPGLVDQLVK.A	(Ions score 35)
27 - 38	453.5945	1357.7615	1357.7606	1	1	R.FKDPGLVDQLVK.A	(Ions score 41)
27 - 38	679.8880	1357.7615	1357.7606	1	1	R.FKDPGLVDQLVK.A	(Ions score 40)
27 - 38	453.5945	1357.7616	1357.7606	1	1	R.FKDPGLVDQLVK.A	(Ions score 36)
27 - 38	453.5945	1357.7616	1357.7606	1	1	R.FKDPGLVDQLVK.A	(Ions score 33)
27 - 38	453.5945	1357.7616	1357.7606	1	1	R.FKDPGLVDQLVK.A	(Ions score 49)
27 - 38	453.5945	1357.7617	1357.7606	1	1	R.FKDPGLVDQLVK.A	(Ions score 46)
27 - 38	453.5945	1357.7617	1357.7606	1	1	R.FKDPGLVDQLVK.A	(Ions score 44)
27 - 38	679.8881	1357.7617	1357.7606	1	1	R.FKDPGLVDQLVK.A	(Ions score 30)
27 - 38	453.5945	1357.7617	1357.7606	1	1	R.FKDPGLVDQLVK.A	(Ions score 35)
27 - 38	453.5945	1357.7617	1357.7606	1	1	R.FKDPGLVDQLVK.A	(Ions score 39)
27 - 38	453.5945	1357.7617	1357.7606	1	1	R.FKDPGLVDQLVK.A	(Ions score 34)

27 - 38	453.5945	1357.7618	1357.7606	1	1	R.FKDPGLVDQLVK.A	(Ions score 29)
27 - 38	453.5945	1357.7618	1357.7606	1	1	R.FKDPGLVDQLVK.A	(Ions score 39)
27 - 38	453.5945	1357.7618	1357.7606	1	1	R.FKDPGLVDQLVK.A	(Ions score 38)
27 - 38	453.5945	1357.7618	1357.7606	1	1	R.FKDPGLVDQLVK.A	(Ions score 55)
27 - 38	679.8882	1357.7618	1357.7606	1	1	R.FKDPGLVDQLVK.A	(Ions score 37)
27 - 38	453.5945	1357.7618	1357.7606	1	1	R.FKDPGLVDQLVK.A	(Ions score 55)
27 - 38	453.5946	1357.7618	1357.7606	1	1	R.FKDPGLVDQLVK.A	(Ions score 46)
27 - 38	453.5946	1357.7618	1357.7606	1	1	R.FKDPGLVDQLVK.A	(Ions score 48)
27 - 38	453.5946	1357.7619	1357.7606	1	1	R.FKDPGLVDQLVK.A	(Ions score 38)
27 - 38	453.5946	1357.7619	1357.7606	1	1	R.FKDPGLVDQLVK.A	(Ions score 31)
27 - 38	453.5946	1357.7619	1357.7606	1	1	R.FKDPGLVDQLVK.A	(Ions score 40)
27 - 38	453.5946	1357.7620	1357.7606	1	1	R.FKDPGLVDQLVK.A	(Ions score 33)
27 - 38	453.5946	1357.7620	1357.7606	1	1	R.FKDPGLVDQLVK.A	(Ions score 47)
27 - 38	453.5946	1357.7620	1357.7606	1	1	R.FKDPGLVDQLVK.A	(Ions score 44)
27 - 38	679.8883	1357.7620	1357.7606	1	1	R.FKDPGLVDQLVK.A	(Ions score 24)
27 - 38	453.5946	1357.7620	1357.7606	1	1	R.FKDPGLVDQLVK.A	(Ions score 46)
27 - 38	679.8883	1357.7620	1357.7606	1	1	R.FKDPGLVDQLVK.A	(Ions score 32)
27 - 38	453.5946	1357.7620	1357.7606	1	1	R.FKDPGLVDQLVK.A	(Ions score 37)
27 - 38	679.8883	1357.7620	1357.7606	1	1	R.FKDPGLVDQLVK.A	(Ions score 30)
27 - 38	453.5946	1357.7620	1357.7606	1	1	R.FKDPGLVDQLVK.A	(Ions score 33)
27 - 38	453.5946	1357.7621	1357.7606	1	1	R.FKDPGLVDQLVK.A	(Ions score 52)
27 - 38	453.5946	1357.7621	1357.7606	1	1	R.FKDPGLVDQLVK.A	(Ions score 50)
27 - 38	453.5946	1357.7621	1357.7606	1	1	R.FKDPGLVDQLVK.A	(Ions score 53)
27 - 38	453.5946	1357.7621	1357.7606	1	1	R.FKDPGLVDQLVK.A	(Ions score 49)
27 - 38	453.5946	1357.7621	1357.7606	1	1	R.FKDPGLVDQLVK.A	(Ions score 46)
27 - 38	453.5946	1357.7621	1357.7606	1	1	R.FKDPGLVDQLVK.A	(Ions score 39)
27 - 38	453.5946	1357.7621	1357.7606	1	1	R.FKDPGLVDQLVK.A	(Ions score 50)
27 - 38	453.5947	1357.7621	1357.7606	1	1	R.FKDPGLVDQLVK.A	(Ions score 44)
27 - 38	453.5947	1357.7621	1357.7606	1	1	R.FKDPGLVDQLVK.A	(Ions score 37)
27 - 38	453.5947	1357.7621	1357.7606	1	1	R.FKDPGLVDQLVK.A	(Ions score 36)
27 - 38	453.5947	1357.7622	1357.7606	1	1	R.FKDPGLVDQLVK.A	(Ions score 41)
27 - 38	453.5947	1357.7622	1357.7606	1	1	R.FKDPGLVDQLVK.A	(Ions score 37)
27 - 38	453.5947	1357.7622	1357.7606	1	1	R.FKDPGLVDQLVK.A	(Ions score 39)
27 - 38	453.5947	1357.7622	1357.7606	1	1	R.FKDPGLVDQLVK.A	(Ions score 48)
27 - 38	453.5947	1357.7622	1357.7606	1	1	R.FKDPGLVDQLVK.A	(Ions score 41)
27 - 38	453.5947	1357.7623	1357.7606	1	1	R.FKDPGLVDQLVK.A	(Ions score 50)
27 - 38	453.5947	1357.7623	1357.7606	1	1	R.FKDPGLVDQLVK.A	(Ions score 39)
27 - 38	453.5947	1357.7623	1357.7606	1	1	R.FKDPGLVDQLVK.A	(Ions score 42)
27 - 38	453.5947	1357.7623	1357.7606	1	1	R.FKDPGLVDQLVK.A	(Ions score 32)
27 - 38	453.5947	1357.7623	1357.7606	1	1	R.FKDPGLVDQLVK.A	(Ions score 26)
27 - 38	453.5947	1357.7623	1357.7606	1	1	R.FKDPGLVDQLVK.A	(Ions score 31)
27 - 38	453.5947	1357.7623	1357.7606	1	1	R.FKDPGLVDQLVK.A	(Ions score 49)
27 - 38	453.5947	1357.7623	1357.7606	1	1	R.FKDPGLVDQLVK.A	(Ions score 41)
27 - 38	453.5947	1357.7623	1357.7606	1	1	R.FKDPGLVDQLVK.A	(Ions score 38)
27 - 38	453.5947	1357.7623	1357.7606	1	1	R.FKDPGLVDQLVK.A	(Ions score 48)
27 - 38	453.5947	1357.7623	1357.7606	1	1	R.FKDPGLVDQLVK.A	(Ions score 40)
27 - 38	453.5947	1357.7624	1357.7606	1	1	R.FKDPGLVDQLVK.A	(Ions score 43)
27 - 38	453.5947	1357.7624	1357.7606	1	1	R.FKDPGLVDQLVK.A	(Ions score 52)
27 - 38	453.5947	1357.7624	1357.7606	1	1	R.FKDPGLVDQLVK.A	(Ions score 52)
27 - 38	453.5947	1357.7624	1357.7606	1	1	R.FKDPGLVDQLVK.A	(Ions score 39)
27 - 38	453.5947	1357.7624	1357.7606	1	1	R.FKDPGLVDQLVK.A	(Ions score 49)
27 - 38	453.5947	1357.7624	1357.7606	1	1	R.FKDPGLVDQLVK.A	(Ions score 37)
27 - 38	453.5948	1357.7624	1357.7606	1	1	R.FKDPGLVDQLVK.A	(Ions score 27)
27 - 38	453.5948	1357.7624	1357.7606	1	1	R.FKDPGLVDQLVK.A	(Ions score 33)
27 - 38	453.5948	1357.7625	1357.7606	1	1	R.FKDPGLVDQLVK.A	(Ions score 36)
27 - 38	453.5948	1357.7625	1357.7606	1	1	R.FKDPGLVDQLVK.A	(Ions score 28)
27 - 38	679.8885	1357.7625	1357.7606	1	1	R.FKDPGLVDQLVK.A	(Ions score 33)

27 - 38	453.5948	1357.7625	1357.7606	1	1	R.FKDPGLVDQLVK.A	(Ions score 49)
27 - 38	453.5948	1357.7625	1357.7606	1	1	R.FKDPGLVDQLVK.A	(Ions score 43)
27 - 38	679.8885	1357.7625	1357.7606	1	1	R.FKDPGLVDQLVK.A	(Ions score 40)
27 - 38	453.5948	1357.7625	1357.7606	1	1	R.FKDPGLVDQLVK.A	(Ions score 42)
27 - 38	453.5948	1357.7625	1357.7606	1	1	R.FKDPGLVDQLVK.A	(Ions score 47)
27 - 38	453.5948	1357.7625	1357.7606	1	1	R.FKDPGLVDQLVK.A	(Ions score 30)
27 - 38	453.5948	1357.7625	1357.7606	1	1	R.FKDPGLVDQLVK.A	(Ions score 46)
27 - 38	453.5948	1357.7625	1357.7606	1	1	R.FKDPGLVDQLVK.A	(Ions score 35)
27 - 38	453.5948	1357.7625	1357.7606	1	1	R.FKDPGLVDQLVK.A	(Ions score 41)
27 - 38	453.5948	1357.7626	1357.7606	1	1	R.FKDPGLVDQLVK.A	(Ions score 41)
27 - 38	453.5948	1357.7626	1357.7606	1	1	R.FKDPGLVDQLVK.A	(Ions score 54)
27 - 38	453.5948	1357.7626	1357.7606	1	1	R.FKDPGLVDQLVK.A	(Ions score 30)
27 - 38	453.5948	1357.7626	1357.7606	1	1	R.FKDPGLVDQLVK.A	(Ions score 40)
27 - 38	453.5948	1357.7626	1357.7606	2	1	R.FKDPGLVDQLVK.A	(Ions score 47)
27 - 38	453.5948	1357.7626	1357.7606	2	1	R.FKDPGLVDQLVK.A	(Ions score 34)
27 - 38	1358.7699	1357.7626	1357.7606	2	1	R.FKDPGLVDQLVK.A	(Ions score 40)
27 - 38	453.5948	1357.7626	1357.7606	2	1	R.FKDPGLVDQLVK.A	(Ions score 36)
27 - 38	453.5948	1357.7627	1357.7606	2	1	R.FKDPGLVDQLVK.A	(Ions score 37)
27 - 38	453.5948	1357.7627	1357.7606	2	1	R.FKDPGLVDQLVK.A	(Ions score 38)
27 - 38	453.5948	1357.7627	1357.7606	2	1	R.FKDPGLVDQLVK.A	(Ions score 39)
27 - 38	453.5948	1357.7627	1357.7606	2	1	R.FKDPGLVDQLVK.A	(Ions score 48)
27 - 38	453.5949	1357.7627	1357.7606	2	1	R.FKDPGLVDQLVK.A	(Ions score 48)
27 - 38	453.5949	1357.7627	1357.7606	2	1	R.FKDPGLVDQLVK.A	(Ions score 51)
27 - 38	453.5949	1357.7627	1357.7606	2	1	R.FKDPGLVDQLVK.A	(Ions score 34)
27 - 38	453.5949	1357.7627	1357.7606	2	1	R.FKDPGLVDQLVK.A	(Ions score 50)
27 - 38	453.5949	1357.7628	1357.7606	2	1	R.FKDPGLVDQLVK.A	(Ions score 43)
27 - 38	453.5949	1357.7628	1357.7606	2	1	R.FKDPGLVDQLVK.A	(Ions score 42)
27 - 38	453.5949	1357.7628	1357.7606	2	1	R.FKDPGLVDQLVK.A	(Ions score 45)
27 - 38	453.5949	1357.7628	1357.7606	2	1	R.FKDPGLVDQLVK.A	(Ions score 48)
27 - 38	453.5949	1357.7628	1357.7606	2	1	R.FKDPGLVDQLVK.A	(Ions score 44)
27 - 38	453.5949	1357.7628	1357.7606	2	1	R.FKDPGLVDQLVK.A	(Ions score 36)
27 - 38	453.5949	1357.7628	1357.7606	2	1	R.FKDPGLVDQLVK.A	(Ions score 39)
27 - 38	453.5949	1357.7628	1357.7606	2	1	R.FKDPGLVDQLVK.A	(Ions score 29)
27 - 38	453.5949	1357.7628	1357.7606	2	1	R.FKDPGLVDQLVK.A	(Ions score 40)
27 - 38	453.5949	1357.7628	1357.7606	2	1	R.FKDPGLVDQLVK.A	(Ions score 41)
27 - 38	679.8887	1357.7628	1357.7606	2	1	R.FKDPGLVDQLVK.A	(Ions score 21)
27 - 38	453.5949	1357.7628	1357.7606	2	1	R.FKDPGLVDQLVK.A	(Ions score 35)
27 - 38	453.5949	1357.7628	1357.7606	2	1	R.FKDPGLVDQLVK.A	(Ions score 40)
27 - 38	453.5949	1357.7629	1357.7606	2	1	R.FKDPGLVDQLVK.A	(Ions score 51)
27 - 38	453.5949	1357.7629	1357.7606	2	1	R.FKDPGLVDQLVK.A	(Ions score 32)
27 - 38	453.5949	1357.7629	1357.7606	2	1	R.FKDPGLVDQLVK.A	(Ions score 39)
27 - 38	453.5949	1357.7629	1357.7606	2	1	R.FKDPGLVDQLVK.A	(Ions score 50)
27 - 38	453.5949	1357.7629	1357.7606	2	1	R.FKDPGLVDQLVK.A	(Ions score 42)
27 - 38	453.5949	1357.7629	1357.7606	2	1	R.FKDPGLVDQLVK.A	(Ions score 49)
27 - 38	453.5949	1357.7629	1357.7606	2	1	R.FKDPGLVDQLVK.A	(Ions score 30)
27 - 38	453.5949	1357.7629	1357.7606	2	1	R.FKDPGLVDQLVK.A	(Ions score 27)
27 - 38	453.5949	1357.7629	1357.7606	2	1	R.FKDPGLVDQLVK.A	(Ions score 55)
27 - 38	453.5949	1357.7629	1357.7606	2	1	R.FKDPGLVDQLVK.A	(Ions score 40)
27 - 38	453.5949	1357.7629	1357.7606	2	1	R.FKDPGLVDQLVK.A	(Ions score 47)
27 - 38	453.5949	1357.7630	1357.7606	2	1	R.FKDPGLVDQLVK.A	(Ions score 34)
27 - 38	1358.7703	1357.7630	1357.7606	2	1	R.FKDPGLVDQLVK.A	(Ions score 38)
27 - 38	453.5949	1357.7630	1357.7606	2	1	R.FKDPGLVDQLVK.A	(Ions score 38)
27 - 38	453.5949	1357.7630	1357.7606	2	1	R.FKDPGLVDQLVK.A	(Ions score 32)
27 - 38	453.5949	1357.7630	1357.7606	2	1	R.FKDPGLVDQLVK.A	(Ions score 47)
27 - 38	453.5950	1357.7630	1357.7606	2	1	R.FKDPGLVDQLVK.A	(Ions score 38)
27 - 38	453.5950	1357.7630	1357.7606	2	1	R.FKDPGLVDQLVK.A	(Ions score 41)
27 - 38	453.5950	1357.7630	1357.7606	2	1	R.FKDPGLVDQLVK.A	(Ions score 40)

27 - 38	453.5950	1357.7630	1357.7606	2	1	R.FKDPGLVDQLVK.A	(Ions score 30)
27 - 38	453.5950	1357.7631	1357.7606	2	1	R.FKDPGLVDQLVK.A	(Ions score 26)
27 - 38	453.5950	1357.7631	1357.7606	2	1	R.FKDPGLVDQLVK.A	(Ions score 49)
27 - 38	453.5950	1357.7631	1357.7606	2	1	R.FKDPGLVDQLVK.A	(Ions score 37)
27 - 38	453.5950	1357.7631	1357.7606	2	1	R.FKDPGLVDQLVK.A	(Ions score 46)
27 - 38	679.8888	1357.7631	1357.7606	2	1	R.FKDPGLVDQLVK.A	(Ions score 37)
27 - 38	453.5950	1357.7631	1357.7606	2	1	R.FKDPGLVDQLVK.A	(Ions score 38)
27 - 38	453.5950	1357.7631	1357.7606	2	1	R.FKDPGLVDQLVK.A	(Ions score 39)
27 - 38	453.5950	1357.7631	1357.7606	2	1	R.FKDPGLVDQLVK.A	(Ions score 51)
27 - 38	453.5950	1357.7631	1357.7606	2	1	R.FKDPGLVDQLVK.A	(Ions score 51)
27 - 38	453.5950	1357.7631	1357.7606	2	1	R.FKDPGLVDQLVK.A	(Ions score 47)
27 - 38	453.5950	1357.7631	1357.7606	2	1	R.FKDPGLVDQLVK.A	(Ions score 45)
27 - 38	679.8888	1357.7631	1357.7606	2	1	R.FKDPGLVDQLVK.A	(Ions score 40)
27 - 38	453.5950	1357.7631	1357.7606	2	1	R.FKDPGLVDQLVK.A	(Ions score 44)
27 - 38	453.5950	1357.7631	1357.7606	2	1	R.FKDPGLVDQLVK.A	(Ions score 50)
27 - 38	453.5950	1357.7631	1357.7606	2	1	R.FKDPGLVDQLVK.A	(Ions score 38)
27 - 38	453.5950	1357.7631	1357.7606	2	1	R.FKDPGLVDQLVK.A	(Ions score 50)
27 - 38	453.5950	1357.7631	1357.7606	2	1	R.FKDPGLVDQLVK.A	(Ions score 48)
27 - 38	453.5950	1357.7631	1357.7606	2	1	R.FKDPGLVDQLVK.A	(Ions score 33)
27 - 38	679.8888	1357.7631	1357.7606	2	1	R.FKDPGLVDQLVK.A	(Ions score 24)
27 - 38	453.5950	1357.7631	1357.7606	2	1	R.FKDPGLVDQLVK.A	(Ions score 40)
27 - 38	453.5950	1357.7631	1357.7606	2	1	R.FKDPGLVDQLVK.A	(Ions score 48)
27 - 38	453.5950	1357.7631	1357.7606	2	1	R.FKDPGLVDQLVK.A	(Ions score 47)
27 - 38	453.5950	1357.7632	1357.7606	2	1	R.FKDPGLVDQLVK.A	(Ions score 34)
27 - 38	453.5950	1357.7632	1357.7606	2	1	R.FKDPGLVDQLVK.A	(Ions score 23)
27 - 38	453.5950	1357.7632	1357.7606	2	1	R.FKDPGLVDQLVK.A	(Ions score 52)
27 - 38	453.5950	1357.7632	1357.7606	2	1	R.FKDPGLVDQLVK.A	(Ions score 30)
27 - 38	453.5950	1357.7632	1357.7606	2	1	R.FKDPGLVDQLVK.A	(Ions score 52)
27 - 38	453.5950	1357.7632	1357.7606	2	1	R.FKDPGLVDQLVK.A	(Ions score 24)
27 - 38	453.5950	1357.7632	1357.7606	2	1	R.FKDPGLVDQLVK.A	(Ions score 38)
27 - 38	453.5950	1357.7632	1357.7606	2	1	R.FKDPGLVDQLVK.A	(Ions score 49)
27 - 38	453.5950	1357.7632	1357.7606	2	1	R.FKDPGLVDQLVK.A	(Ions score 45)
27 - 38	453.5950	1357.7632	1357.7606	2	1	R.FKDPGLVDQLVK.A	(Ions score 35)
27 - 38	453.5950	1357.7632	1357.7606	2	1	R.FKDPGLVDQLVK.A	(Ions score 39)
27 - 38	679.8889	1357.7632	1357.7606	2	1	R.FKDPGLVDQLVK.A	(Ions score 57)
27 - 38	453.5950	1357.7632	1357.7606	2	1	R.FKDPGLVDQLVK.A	(Ions score 45)
27 - 38	453.5950	1357.7632	1357.7606	2	1	R.FKDPGLVDQLVK.A	(Ions score 39)
27 - 38	453.5950	1357.7632	1357.7606	2	1	R.FKDPGLVDQLVK.A	(Ions score 49)
27 - 38	453.5950	1357.7632	1357.7606	2	1	R.FKDPGLVDQLVK.A	(Ions score 45)
27 - 38	453.5950	1357.7632	1357.7606	2	1	R.FKDPGLVDQLVK.A	(Ions score 24)
27 - 38	679.8889	1357.7632	1357.7606	2	1	R.FKDPGLVDQLVK.A	(Ions score 40)
27 - 38	453.5950	1357.7632	1357.7606	2	1	R.FKDPGLVDQLVK.A	(Ions score 42)
27 - 38	453.5950	1357.7632	1357.7606	2	1	R.FKDPGLVDQLVK.A	(Ions score 39)
27 - 38	679.8889	1357.7633	1357.7606	2	1	R.FKDPGLVDQLVK.A	(Ions score 24)
27 - 38	453.5950	1357.7633	1357.7606	2	1	R.FKDPGLVDQLVK.A	(Ions score 38)
27 - 38	453.5950	1357.7633	1357.7606	2	1	R.FKDPGLVDQLVK.A	(Ions score 40)
27 - 38	453.5950	1357.7633	1357.7606	2	1	R.FKDPGLVDQLVK.A	(Ions score 33)
27 - 38	453.5950	1357.7633	1357.7606	2	1	R.FKDPGLVDQLVK.A	(Ions score 28)
27 - 38	453.5950	1357.7633	1357.7606	2	1	R.FKDPGLVDQLVK.A	(Ions score 56)
27 - 38	453.5950	1357.7633	1357.7606	2	1	R.FKDPGLVDQLVK.A	(Ions score 51)
27 - 38	453.5950	1357.7633	1357.7606	2	1	R.FKDPGLVDQLVK.A	(Ions score 31)
27 - 38	453.5950	1357.7633	1357.7606	2	1	R.FKDPGLVDQLVK.A	(Ions score 48)
27 - 38	453.5950	1357.7633	1357.7606	2	1	R.FKDPGLVDQLVK.A	(Ions score 39)
27 - 38	453.5950	1357.7633	1357.7606	2	1	R.FKDPGLVDQLVK.A	(Ions score 40)
27 - 38	453.5950	1357.7633	1357.7606	2	1	R.FKDPGLVDQLVK.A	(Ions score 47)
27 - 38	453.5950	1357.7633	1357.7606	2	1	R.FKDPGLVDQLVK.A	(Ions score 35)
27 - 38	453.5950	1357.7633	1357.7606	2	1	R.FKDPGLVDQLVK.A	(Ions score 49)

27 - 38	453.5951	1357.7633	1357.7606	2	1	R.FKDPGLVDQLVK.A	(Ions score 33)
27 - 38	453.5951	1357.7633	1357.7606	2	1	R.FKDPGLVDQLVK.A	(Ions score 32)
27 - 38	453.5951	1357.7634	1357.7606	2	1	R.FKDPGLVDQLVK.A	(Ions score 41)
27 - 38	679.8890	1357.7634	1357.7606	2	1	R.FKDPGLVDQLVK.A	(Ions score 37)
27 - 38	453.5951	1357.7634	1357.7606	2	1	R.FKDPGLVDQLVK.A	(Ions score 46)
27 - 38	679.8890	1357.7634	1357.7606	2	1	R.FKDPGLVDQLVK.A	(Ions score 24)
27 - 38	453.5951	1357.7634	1357.7606	2	1	R.FKDPGLVDQLVK.A	(Ions score 38)
27 - 38	679.8890	1357.7634	1357.7606	2	1	R.FKDPGLVDQLVK.A	(Ions score 22)
27 - 38	679.8890	1357.7634	1357.7606	2	1	R.FKDPGLVDQLVK.A	(Ions score 24)
27 - 38	453.5951	1357.7634	1357.7606	2	1	R.FKDPGLVDQLVK.A	(Ions score 39)
27 - 38	453.5951	1357.7634	1357.7606	2	1	R.FKDPGLVDQLVK.A	(Ions score 41)
27 - 38	453.5951	1357.7635	1357.7606	2	1	R.FKDPGLVDQLVK.A	(Ions score 37)
27 - 38	679.8890	1357.7635	1357.7606	2	1	R.FKDPGLVDQLVK.A	(Ions score 30)
27 - 38	453.5951	1357.7635	1357.7606	2	1	R.FKDPGLVDQLVK.A	(Ions score 38)
27 - 38	453.5951	1357.7635	1357.7606	2	1	R.FKDPGLVDQLVK.A	(Ions score 51)
27 - 38	453.5951	1357.7635	1357.7606	2	1	R.FKDPGLVDQLVK.A	(Ions score 32)
27 - 38	453.5951	1357.7635	1357.7606	2	1	R.FKDPGLVDQLVK.A	(Ions score 41)
27 - 38	453.5951	1357.7635	1357.7606	2	1	R.FKDPGLVDQLVK.A	(Ions score 42)
27 - 38	453.5951	1357.7635	1357.7606	2	1	R.FKDPGLVDQLVK.A	(Ions score 33)
27 - 38	679.8890	1357.7635	1357.7606	2	1	R.FKDPGLVDQLVK.A	(Ions score 40)
27 - 38	679.8890	1357.7635	1357.7606	2	1	R.FKDPGLVDQLVK.A	(Ions score 29)
27 - 38	453.5951	1357.7635	1357.7606	2	1	R.FKDPGLVDQLVK.A	(Ions score 39)
27 - 38	679.8890	1357.7635	1357.7606	2	1	R.FKDPGLVDQLVK.A	(Ions score 32)
27 - 38	453.5951	1357.7635	1357.7606	2	1	R.FKDPGLVDQLVK.A	(Ions score 31)
27 - 38	453.5951	1357.7635	1357.7606	2	1	R.FKDPGLVDQLVK.A	(Ions score 42)
27 - 38	453.5951	1357.7636	1357.7606	2	1	R.FKDPGLVDQLVK.A	(Ions score 34)
27 - 38	453.5951	1357.7636	1357.7606	2	1	R.FKDPGLVDQLVK.A	(Ions score 45)
27 - 38	453.5951	1357.7636	1357.7606	2	1	R.FKDPGLVDQLVK.A	(Ions score 52)
27 - 38	453.5951	1357.7636	1357.7606	2	1	R.FKDPGLVDQLVK.A	(Ions score 29)
27 - 38	679.8891	1357.7636	1357.7606	2	1	R.FKDPGLVDQLVK.A	(Ions score 39)
27 - 38	453.5951	1357.7636	1357.7606	2	1	R.FKDPGLVDQLVK.A	(Ions score 41)
27 - 38	453.5951	1357.7636	1357.7606	2	1	R.FKDPGLVDQLVK.A	(Ions score 27)
27 - 38	453.5951	1357.7636	1357.7606	2	1	R.FKDPGLVDQLVK.A	(Ions score 40)
27 - 38	453.5951	1357.7636	1357.7606	2	1	R.FKDPGLVDQLVK.A	(Ions score 31)
27 - 38	453.5952	1357.7636	1357.7606	2	1	R.FKDPGLVDQLVK.A	(Ions score 32)
27 - 38	453.5952	1357.7636	1357.7606	2	1	R.FKDPGLVDQLVK.A	(Ions score 36)
27 - 38	453.5952	1357.7636	1357.7606	2	1	R.FKDPGLVDQLVK.A	(Ions score 47)
27 - 38	453.5952	1357.7636	1357.7606	2	1	R.FKDPGLVDQLVK.A	(Ions score 36)
27 - 38	453.5952	1357.7636	1357.7606	2	1	R.FKDPGLVDQLVK.A	(Ions score 45)
27 - 38	453.5952	1357.7637	1357.7606	2	1	R.FKDPGLVDQLVK.A	(Ions score 48)
27 - 38	453.5952	1357.7637	1357.7606	2	1	R.FKDPGLVDQLVK.A	(Ions score 35)
27 - 38	453.5952	1357.7637	1357.7606	2	1	R.FKDPGLVDQLVK.A	(Ions score 47)
27 - 38	679.8891	1357.7637	1357.7606	2	1	R.FKDPGLVDQLVK.A	(Ions score 36)
27 - 38	679.8891	1357.7637	1357.7606	2	1	R.FKDPGLVDQLVK.A	(Ions score 33)
27 - 38	679.8891	1357.7637	1357.7606	2	1	R.FKDPGLVDQLVK.A	(Ions score 25)
27 - 38	453.5952	1357.7637	1357.7606	2	1	R.FKDPGLVDQLVK.A	(Ions score 50)
27 - 38	453.5952	1357.7637	1357.7606	2	1	R.FKDPGLVDQLVK.A	(Ions score 46)
27 - 38	679.8891	1357.7637	1357.7606	2	1	R.FKDPGLVDQLVK.A	(Ions score 36)
27 - 38	453.5952	1357.7637	1357.7606	2	1	R.FKDPGLVDQLVK.A	(Ions score 37)
27 - 38	453.5952	1357.7638	1357.7606	2	1	R.FKDPGLVDQLVK.A	(Ions score 41)
27 - 38	453.5952	1357.7638	1357.7606	2	1	R.FKDPGLVDQLVK.A	(Ions score 47)
27 - 38	453.5952	1357.7638	1357.7606	2	1	R.FKDPGLVDQLVK.A	(Ions score 51)
27 - 38	453.5952	1357.7638	1357.7606	2	1	R.FKDPGLVDQLVK.A	(Ions score 45)
27 - 38	453.5952	1357.7638	1357.7606	2	1	R.FKDPGLVDQLVK.A	(Ions score 52)
27 - 38	453.5952	1357.7638	1357.7606	2	1	R.FKDPGLVDQLVK.A	(Ions score 37)
27 - 38	453.5952	1357.7638	1357.7606	2	1	R.FKDPGLVDQLVK.A	(Ions score 39)
27 - 38	453.5952	1357.7639	1357.7606	2	1	R.FKDPGLVDQLVK.A	(Ions score 49)

27 - 38	453.5952	1357.7639	1357.7606	2	1	R.FKDPGLVDQLVK.A	(Ions score 40)
27 - 38	453.5952	1357.7639	1357.7606	2	1	R.FKDPGLVDQLVK.A	(Ions score 46)
27 - 38	453.5952	1357.7639	1357.7606	2	1	R.FKDPGLVDQLVK.A	(Ions score 48)
27 - 38	453.5952	1357.7639	1357.7606	2	1	R.FKDPGLVDQLVK.A	(Ions score 35)
27 - 38	453.5952	1357.7639	1357.7606	2	1	R.FKDPGLVDQLVK.A	(Ions score 36)
27 - 38	453.5952	1357.7639	1357.7606	2	1	R.FKDPGLVDQLVK.A	(Ions score 49)
27 - 38	453.5953	1357.7639	1357.7606	2	1	R.FKDPGLVDQLVK.A	(Ions score 41)
27 - 38	453.5953	1357.7640	1357.7606	3	1	R.FKDPGLVDQLVK.A	(Ions score 50)
27 - 38	453.5953	1357.7640	1357.7606	3	1	R.FKDPGLVDQLVK.A	(Ions score 41)
27 - 38	453.5953	1357.7640	1357.7606	3	1	R.FKDPGLVDQLVK.A	(Ions score 49)
27 - 38	453.5953	1357.7640	1357.7606	3	1	R.FKDPGLVDQLVK.A	(Ions score 40)
27 - 38	453.5953	1357.7640	1357.7606	3	1	R.FKDPGLVDQLVK.A	(Ions score 32)
27 - 38	453.5953	1357.7640	1357.7606	3	1	R.FKDPGLVDQLVK.A	(Ions score 49)
27 - 38	679.8893	1357.7641	1357.7606	3	1	R.FKDPGLVDQLVK.A	(Ions score 32)
27 - 38	453.5953	1357.7641	1357.7606	3	1	R.FKDPGLVDQLVK.A	(Ions score 32)
27 - 38	453.5953	1357.7641	1357.7606	3	1	R.FKDPGLVDQLVK.A	(Ions score 31)
27 - 38	453.5953	1357.7641	1357.7606	3	1	R.FKDPGLVDQLVK.A	(Ions score 51)
27 - 38	453.5953	1357.7641	1357.7606	3	1	R.FKDPGLVDQLVK.A	(Ions score 41)
27 - 38	453.5953	1357.7641	1357.7606	3	1	R.FKDPGLVDQLVK.A	(Ions score 31)
27 - 38	453.5953	1357.7641	1357.7606	3	1	R.FKDPGLVDQLVK.A	(Ions score 49)
27 - 38	453.5953	1357.7641	1357.7606	3	1	R.FKDPGLVDQLVK.A	(Ions score 52)
27 - 38	453.5953	1357.7641	1357.7606	3	1	R.FKDPGLVDQLVK.A	(Ions score 50)
27 - 38	453.5953	1357.7641	1357.7606	3	1	R.FKDPGLVDQLVK.A	(Ions score 28)
27 - 38	453.5953	1357.7641	1357.7606	3	1	R.FKDPGLVDQLVK.A	(Ions score 45)
27 - 38	679.8893	1357.7641	1357.7606	3	1	R.FKDPGLVDQLVK.A	(Ions score 27)
27 - 38	453.5953	1357.7642	1357.7606	3	1	R.FKDPGLVDQLVK.A	(Ions score 35)
27 - 38	453.5953	1357.7642	1357.7606	3	1	R.FKDPGLVDQLVK.A	(Ions score 50)
27 - 38	453.5953	1357.7642	1357.7606	3	1	R.FKDPGLVDQLVK.A	(Ions score 38)
27 - 38	453.5953	1357.7642	1357.7606	3	1	R.FKDPGLVDQLVK.A	(Ions score 45)
27 - 38	453.5953	1357.7642	1357.7606	3	1	R.FKDPGLVDQLVK.A	(Ions score 51)
27 - 38	453.5953	1357.7642	1357.7606	3	1	R.FKDPGLVDQLVK.A	(Ions score 30)
27 - 38	453.5954	1357.7642	1357.7606	3	1	R.FKDPGLVDQLVK.A	(Ions score 40)
27 - 38	453.5954	1357.7642	1357.7606	3	1	R.FKDPGLVDQLVK.A	(Ions score 38)
27 - 38	453.5954	1357.7642	1357.7606	3	1	R.FKDPGLVDQLVK.A	(Ions score 31)
27 - 38	453.5954	1357.7643	1357.7606	3	1	R.FKDPGLVDQLVK.A	(Ions score 48)
27 - 38	453.5954	1357.7643	1357.7606	3	1	R.FKDPGLVDQLVK.A	(Ions score 43)
27 - 38	453.5954	1357.7643	1357.7606	3	1	R.FKDPGLVDQLVK.A	(Ions score 48)
27 - 38	453.5954	1357.7643	1357.7606	3	1	R.FKDPGLVDQLVK.A	(Ions score 51)
27 - 38	453.5954	1357.7643	1357.7606	3	1	R.FKDPGLVDQLVK.A	(Ions score 39)
27 - 38	453.5954	1357.7643	1357.7606	3	1	R.FKDPGLVDQLVK.A	(Ions score 43)
27 - 38	453.5954	1357.7643	1357.7606	3	1	R.FKDPGLVDQLVK.A	(Ions score 48)
27 - 38	453.5954	1357.7643	1357.7606	3	1	R.FKDPGLVDQLVK.A	(Ions score 40)
27 - 38	453.5954	1357.7643	1357.7606	3	1	R.FKDPGLVDQLVK.A	(Ions score 32)
27 - 38	679.8895	1357.7644	1357.7606	3	1	R.FKDPGLVDQLVK.A	(Ions score 31)
27 - 38	453.5954	1357.7644	1357.7606	3	1	R.FKDPGLVDQLVK.A	(Ions score 31)
27 - 38	453.5954	1357.7644	1357.7606	3	1	R.FKDPGLVDQLVK.A	(Ions score 39)
27 - 38	453.5954	1357.7644	1357.7606	3	1	R.FKDPGLVDQLVK.A	(Ions score 38)
27 - 38	453.5954	1357.7644	1357.7606	3	1	R.FKDPGLVDQLVK.A	(Ions score 49)
27 - 38	453.5954	1357.7644	1357.7606	3	1	R.FKDPGLVDQLVK.A	(Ions score 49)
27 - 38	453.5954	1357.7644	1357.7606	3	1	R.FKDPGLVDQLVK.A	(Ions score 39)
27 - 38	453.5954	1357.7644	1357.7606	3	1	R.FKDPGLVDQLVK.A	(Ions score 40)
27 - 38	453.5954	1357.7645	1357.7606	3	1	R.FKDPGLVDQLVK.A	(Ions score 34)
27 - 38	453.5954	1357.7645	1357.7606	3	1	R.FKDPGLVDQLVK.A	(Ions score 38)
27 - 38	453.5954	1357.7645	1357.7606	3	1	R.FKDPGLVDQLVK.A	(Ions score 40)
27 - 38	453.5954	1357.7645	1357.7606	3	1	R.FKDPGLVDQLVK.A	(Ions score 52)
27 - 38	453.5954	1357.7645	1357.7606	3	1	R.FKDPGLVDQLVK.A	(Ions score 41)
27 - 38	453.5954	1357.7645	1357.7606	3	1	R.FKDPGLVDQLVK.A	(Ions score 38)

27 - 38	453.5954	1357.7645	1357.7606	3	1	R.FKDPGLVDQLVK.A	(Ions score 43)
27 - 38	453.5954	1357.7645	1357.7606	3	1	R.FKDPGLVDQLVK.A	(Ions score 34)
27 - 38	453.5955	1357.7645	1357.7606	3	1	R.FKDPGLVDQLVK.A	(Ions score 34)
27 - 38	453.5955	1357.7645	1357.7606	3	1	R.FKDPGLVDQLVK.A	(Ions score 43)
27 - 38	679.8896	1357.7646	1357.7606	3	1	R.FKDPGLVDQLVK.A	(Ions score 44)
27 - 38	453.5955	1357.7646	1357.7606	3	1	R.FKDPGLVDQLVK.A	(Ions score 34)
27 - 38	679.8896	1357.7646	1357.7606	3	1	R.FKDPGLVDQLVK.A	(Ions score 30)
27 - 38	679.8896	1357.7646	1357.7606	3	1	R.FKDPGLVDQLVK.A	(Ions score 30)
27 - 38	453.5955	1357.7646	1357.7606	3	1	R.FKDPGLVDQLVK.A	(Ions score 33)
27 - 38	453.5955	1357.7647	1357.7606	3	1	R.FKDPGLVDQLVK.A	(Ions score 22)
27 - 38	453.5955	1357.7647	1357.7606	3	1	R.FKDPGLVDQLVK.A	(Ions score 50)
27 - 38	453.5955	1357.7647	1357.7606	3	1	R.FKDPGLVDQLVK.A	(Ions score 40)
27 - 38	453.5955	1357.7647	1357.7606	3	1	R.FKDPGLVDQLVK.A	(Ions score 30)
27 - 38	679.8896	1357.7647	1357.7606	3	1	R.FKDPGLVDQLVK.A	(Ions score 44)
27 - 38	453.5955	1357.7647	1357.7606	3	1	R.FKDPGLVDQLVK.A	(Ions score 31)
27 - 38	453.5955	1357.7647	1357.7606	3	1	R.FKDPGLVDQLVK.A	(Ions score 44)
27 - 38	453.5955	1357.7648	1357.7606	3	1	R.FKDPGLVDQLVK.A	(Ions score 49)
27 - 38	453.5955	1357.7648	1357.7606	3	1	R.FKDPGLVDQLVK.A	(Ions score 32)
27 - 38	453.5955	1357.7648	1357.7606	3	1	R.FKDPGLVDQLVK.A	(Ions score 48)
27 - 38	453.5955	1357.7648	1357.7606	3	1	R.FKDPGLVDQLVK.A	(Ions score 46)
27 - 38	679.8897	1357.7648	1357.7606	3	1	R.FKDPGLVDQLVK.A	(Ions score 38)
27 - 38	453.5955	1357.7648	1357.7606	3	1	R.FKDPGLVDQLVK.A	(Ions score 40)
27 - 38	453.5956	1357.7649	1357.7606	3	1	R.FKDPGLVDQLVK.A	(Ions score 40)
27 - 38	453.5956	1357.7649	1357.7606	3	1	R.FKDPGLVDQLVK.A	(Ions score 27)
27 - 38	453.5956	1357.7649	1357.7606	3	1	R.FKDPGLVDQLVK.A	(Ions score 49)
27 - 38	453.5956	1357.7650	1357.7606	3	1	R.FKDPGLVDQLVK.A	(Ions score 34)
27 - 38	453.5956	1357.7650	1357.7606	3	1	R.FKDPGLVDQLVK.A	(Ions score 29)
27 - 38	679.8898	1357.7650	1357.7606	3	1	R.FKDPGLVDQLVK.A	(Ions score 28)
27 - 38	453.5956	1357.7650	1357.7606	3	1	R.FKDPGLVDQLVK.A	(Ions score 30)
27 - 38	453.5956	1357.7650	1357.7606	3	1	R.FKDPGLVDQLVK.A	(Ions score 34)
27 - 38	453.5956	1357.7650	1357.7606	3	1	R.FKDPGLVDQLVK.A	(Ions score 38)
27 - 38	453.5956	1357.7650	1357.7606	3	1	R.FKDPGLVDQLVK.A	(Ions score 29)
27 - 38	453.5956	1357.7650	1357.7606	3	1	R.FKDPGLVDQLVK.A	(Ions score 50)
27 - 38	453.5956	1357.7651	1357.7606	3	1	R.FKDPGLVDQLVK.A	(Ions score 52)
27 - 38	453.5956	1357.7651	1357.7606	3	1	R.FKDPGLVDQLVK.A	(Ions score 48)
27 - 38	453.5956	1357.7651	1357.7606	3	1	R.FKDPGLVDQLVK.A	(Ions score 43)
27 - 38	453.5957	1357.7651	1357.7606	3	1	R.FKDPGLVDQLVK.A	(Ions score 52)
27 - 38	453.5957	1357.7652	1357.7606	3	1	R.FKDPGLVDQLVK.A	(Ions score 40)
27 - 38	453.5957	1357.7652	1357.7606	3	1	R.FKDPGLVDQLVK.A	(Ions score 43)
27 - 38	679.8899	1357.7652	1357.7606	3	1	R.FKDPGLVDQLVK.A	(Ions score 33)
27 - 38	453.5957	1357.7652	1357.7606	3	1	R.FKDPGLVDQLVK.A	(Ions score 48)
27 - 38	453.5957	1357.7652	1357.7606	3	1	R.FKDPGLVDQLVK.A	(Ions score 36)
27 - 38	453.5957	1357.7653	1357.7606	3	1	R.FKDPGLVDQLVK.A	(Ions score 52)
27 - 38	453.5957	1357.7653	1357.7606	3	1	R.FKDPGLVDQLVK.A	(Ions score 55)
27 - 38	453.5957	1357.7653	1357.7606	3	1	R.FKDPGLVDQLVK.A	(Ions score 47)
27 - 38	679.8899	1357.7653	1357.7606	4	1	R.FKDPGLVDQLVK.A	(Ions score 29)
27 - 38	679.8899	1357.7653	1357.7606	4	1	R.FKDPGLVDQLVK.A	(Ions score 25)
27 - 38	453.5957	1357.7653	1357.7606	4	1	R.FKDPGLVDQLVK.A	(Ions score 48)
27 - 38	453.5957	1357.7654	1357.7606	4	1	R.FKDPGLVDQLVK.A	(Ions score 43)
27 - 38	453.5957	1357.7654	1357.7606	4	1	R.FKDPGLVDQLVK.A	(Ions score 28)
27 - 38	453.5957	1357.7654	1357.7606	4	1	R.FKDPGLVDQLVK.A	(Ions score 25)
27 - 38	453.5958	1357.7655	1357.7606	4	1	R.FKDPGLVDQLVK.A	(Ions score 29)
27 - 38	453.5958	1357.7655	1357.7606	4	1	R.FKDPGLVDQLVK.A	(Ions score 40)
27 - 38	679.8900	1357.7655	1357.7606	4	1	R.FKDPGLVDQLVK.A	(Ions score 32)
27 - 38	453.5958	1357.7655	1357.7606	4	1	R.FKDPGLVDQLVK.A	(Ions score 50)
27 - 38	453.5958	1357.7655	1357.7606	4	1	R.FKDPGLVDQLVK.A	(Ions score 33)
27 - 38	453.5958	1357.7656	1357.7606	4	1	R.FKDPGLVDQLVK.A	(Ions score 46)

27 - 38	453.5958	1357.7656	1357.7606	4	1	R.FKDPGLVDQLVK.A		(Ions score 40)
27 - 38	453.5958	1357.7657	1357.7606	4	1	R.FKDPGLVDQLVK.A		(Ions score 23)
27 - 38	453.5958	1357.7657	1357.7606	4	1	R.FKDPGLVDQLVK.A		(Ions score 34)
27 - 38	679.8901	1357.7657	1357.7606	4	1	R.FKDPGLVDQLVK.A		(Ions score 32)
27 - 38	453.5959	1357.7658	1357.7606	4	1	R.FKDPGLVDQLVK.A		(Ions score 47)
27 - 38	453.5959	1357.7658	1357.7606	4	1	R.FKDPGLVDQLVK.A		(Ions score 39)
27 - 38	453.5959	1357.7658	1357.7606	4	1	R.FKDPGLVDQLVK.A		(Ions score 27)
27 - 38	453.5959	1357.7658	1357.7606	4	1	R.FKDPGLVDQLVK.A		(Ions score 46)
27 - 38	453.5959	1357.7658	1357.7606	4	1	R.FKDPGLVDQLVK.A		(Ions score 44)
27 - 38	453.5959	1357.7658	1357.7606	4	1	R.FKDPGLVDQLVK.A		(Ions score 49)
27 - 38	679.8903	1357.7660	1357.7606	4	1	R.FKDPGLVDQLVK.A		(Ions score 42)
27 - 38	453.5959	1357.7660	1357.7606	4	1	R.FKDPGLVDQLVK.A		(Ions score 50)
27 - 38	679.8903	1357.7660	1357.7606	4	1	R.FKDPGLVDQLVK.A		(Ions score 29)
27 - 38	453.5960	1357.7661	1357.7606	4	1	R.FKDPGLVDQLVK.A		(Ions score 39)
27 - 38	453.5960	1357.7661	1357.7606	4	1	R.FKDPGLVDQLVK.A		(Ions score 47)
27 - 38	453.5960	1357.7661	1357.7606	4	1	R.FKDPGLVDQLVK.A		(Ions score 55)
27 - 38	453.5960	1357.7663	1357.7606	4	1	R.FKDPGLVDQLVK.A		(Ions score 51)
27 - 38	453.5960	1357.7663	1357.7606	4	1	R.FKDPGLVDQLVK.A		(Ions score 36)
27 - 38	453.5960	1357.7663	1357.7606	4	1	R.FKDPGLVDQLVK.A		(Ions score 52)
27 - 38	679.8904	1357.7663	1357.7606	4	1	R.FKDPGLVDQLVK.A		(Ions score 26)
27 - 38	453.5961	1357.7664	1357.7606	4	1	R.FKDPGLVDQLVK.A		(Ions score 33)
27 - 38	679.8905	1357.7664	1357.7606	4	1	R.FKDPGLVDQLVK.A		(Ions score 39)
27 - 38	453.5961	1357.7665	1357.7606	4	1	R.FKDPGLVDQLVK.A		(Ions score 50)
27 - 38	679.8906	1357.7666	1357.7606	4	1	R.FKDPGLVDQLVK.A		(Ions score 23)
27 - 38	679.8906	1357.7667	1357.7606	4	1	R.FKDPGLVDQLVK.A		(Ions score 28)
27 - 38	679.8906	1357.7667	1357.7606	5	1	R.FKDPGLVDQLVK.A		(Ions score 39)
27 - 38	453.5962	1357.7668	1357.7606	5	1	R.FKDPGLVDQLVK.A		(Ions score 36)
27 - 38	453.5962	1357.7669	1357.7606	5	1	R.FKDPGLVDQLVK.A		(Ions score 48)
27 - 38	453.5963	1357.7670	1357.7606	5	1	R.FKDPGLVDQLVK.A		(Ions score 41)
27 - 38	453.5963	1357.7670	1357.7606	5	1	R.FKDPGLVDQLVK.A		(Ions score 37)
27 - 38	453.5963	1357.7670	1357.7606	5	1	R.FKDPGLVDQLVK.A		(Ions score 34)
27 - 38	679.8908	1357.7670	1357.7606	5	1	R.FKDPGLVDQLVK.A		(Ions score 22)
27 - 38	467.5984	1399.7732	1399.7711	2	1	R.FKDPGLVDQLVK.A	Acetylation (K)	(Ions score 36)
27 - 38	700.8943	1399.7740	1399.7711	2	1	R.FKDPGLVDQLVK.A	Acetylation (K)	(Ions score 21)
27 - 38	467.5988	1399.7746	1399.7711	3	1	R.FKDPGLVDQLVK.A	Acetylation (K)	(Ions score 56)
27 - 38	467.5990	1399.7752	1399.7711	3	1	R.FKDPGLVDQLVK.A	Acetylation (K)	(Ions score 32)
27 - 38	467.5990	1399.7753	1399.7711	3	1	R.FKDPGLVDQLVK.A	Acetylation (K)	(Ions score 42)
27 - 38	467.5991	1399.7754	1399.7711	3	1	R.FKDPGLVDQLVK.A	Acetylation (K)	(Ions score 57)
27 - 38	700.8950	1399.7754	1399.7711	3	1	R.FKDPGLVDQLVK.A	Acetylation (K)	(Ions score 43)
27 - 38	467.5991	1399.7756	1399.7711	3	1	R.FKDPGLVDQLVK.A	Acetylation (K)	(Ions score 22)
27 - 38	467.5992	1399.7757	1399.7711	3	1	R.FKDPGLVDQLVK.A	Acetylation (K)	(Ions score 51)
27 - 38	700.8952	1399.7758	1399.7711	3	1	R.FKDPGLVDQLVK.A	Acetylation (K)	(Ions score 40)
27 - 38	700.8952	1399.7759	1399.7711	3	1	R.FKDPGLVDQLVK.A	Acetylation (K)	(Ions score 42)
27 - 38	467.5993	1399.7759	1399.7711	3	1	R.FKDPGLVDQLVK.A	Acetylation (K)	(Ions score 49)
27 - 38	700.8953	1399.7760	1399.7711	3	1	R.FKDPGLVDQLVK.A	Acetylation (K)	(Ions score 41)
27 - 38	467.5993	1399.7761	1399.7711	4	1	R.FKDPGLVDQLVK.A	Acetylation (K)	(Ions score 57)
27 - 38	467.5995	1399.7766	1399.7711	4	1	R.FKDPGLVDQLVK.A	Acetylation (K)	(Ions score 45)
27 - 38	700.8956	1399.7766	1399.7711	4	1	R.FKDPGLVDQLVK.A	Acetylation (K)	(Ions score 22)
27 - 38	467.5996	1399.7768	1399.7711	4	1	R.FKDPGLVDQLVK.A	Acetylation (K)	(Ions score 56)
29 - 38	542.3068	1082.5990	1082.5972	2	0	K.DPGLVDQLVK.A		(Ions score 26)
29 - 38	542.3069	1082.5992	1082.5972	2	0	K.DPGLVDQLVK.A		(Ions score 55)
29 - 38	542.3070	1082.5993	1082.5972	2	0	K.DPGLVDQLVK.A		(Ions score 49)
29 - 38	542.3070	1082.5994	1082.5972	2	0	K.DPGLVDQLVK.A		(Ions score 39)
29 - 38	542.3071	1082.5997	1082.5972	2	0	K.DPGLVDQLVK.A		(Ions score 50)
29 - 38	542.3071	1082.5997	1082.5972	2	0	K.DPGLVDQLVK.A		(Ions score 36)
29 - 38	542.3072	1082.5998	1082.5972	2	0	K.DPGLVDQLVK.A		(Ions score 25)
29 - 38	542.3073	1082.6000	1082.5972	3	0	K.DPGLVDQLVK.A		(Ions score 42)

29 - 38	542.3073	1082.6000	1082.5972	3	0	K.DPGLVDQLVK.A	(Ions score 39)
29 - 38	542.3073	1082.6000	1082.5972	3	0	K.DPGLVDQLVK.A	(Ions score 48)
29 - 38	542.3073	1082.6000	1082.5972	3	0	K.DPGLVDQLVK.A	(Ions score 63)
29 - 38	542.3074	1082.6001	1082.5972	3	0	K.DPGLVDQLVK.A	(Ions score 50)
29 - 38	542.3074	1082.6002	1082.5972	3	0	K.DPGLVDQLVK.A	(Ions score 47)
29 - 38	542.3075	1082.6005	1082.5972	3	0	K.DPGLVDQLVK.A	(Ions score 46)
29 - 38	542.3075	1082.6005	1082.5972	3	0	K.DPGLVDQLVK.A	(Ions score 27)
29 - 38	542.3076	1082.6006	1082.5972	3	0	K.DPGLVDQLVK.A	(Ions score 45)
29 - 38	542.3078	1082.6010	1082.5972	3	0	K.DPGLVDQLVK.A	(Ions score 38)
29 - 38	542.3082	1082.6019	1082.5972	4	0	K.DPGLVDQLVK.A	(Ions score 46)
29 - 38	542.3082	1082.6019	1082.5972	4	0	K.DPGLVDQLVK.A	(Ions score 48)
29 - 44	609.9802	1826.9187	1826.9163	1	1	K.DPGLVDQLVKADSEWR.R	(Ions score 31)
29 - 44	609.9808	1826.9207	1826.9163	2	1	K.DPGLVDQLVKADSEWR.R	(Ions score 36)
39 - 44	763.3375	762.3302	762.3297	1	0	K.ADSEWR.R	(Ions score 25)
39 - 44	763.3384	762.3311	762.3297	2	0	K.ADSEWR.R	(Ions score 37)
39 - 44	763.3386	762.3313	762.3297	2	0	K.ADSEWR.R	(Ions score 22)
39 - 44	763.3391	762.3318	762.3297	3	0	K.ADSEWR.R	(Ions score 33)
39 - 45	919.4390	918.4317	918.4308	1	1	K.ADSEWRR.C	(Ions score 23)
48 - 55	489.2618	976.5090	976.5090	-0	1	R.FRADNLNK.L	(Ions score 32)
48 - 55	977.5164	976.5091	976.5090	0	1	R.FRADNLNK.L	(Ions score 39)
48 - 55	489.2621	976.5096	976.5090	1	1	R.FRADNLNK.L	(Ions score 21)
48 - 55	489.2626	976.5106	976.5090	2	1	R.FRADNLNK.L	(Ions score 26)
48 - 55	489.2626	976.5106	976.5090	2	1	R.FRADNLNK.L	(Ions score 21)
48 - 55	489.2626	976.5107	976.5090	2	1	R.FRADNLNK.L	(Ions score 23)
48 - 55	489.2627	976.5108	976.5090	2	1	R.FRADNLNK.L	(Ions score 30)
48 - 55	489.2627	976.5108	976.5090	2	1	R.FRADNLNK.L	(Ions score 25)
48 - 55	489.2627	976.5109	976.5090	2	1	R.FRADNLNK.L	(Ions score 25)
48 - 55	489.2627	976.5109	976.5090	2	1	R.FRADNLNK.L	(Ions score 28)
48 - 55	489.2631	976.5117	976.5090	3	1	R.FRADNLNK.L	(Ions score 27)
48 - 55	489.2633	976.5121	976.5090	3	1	R.FRADNLNK.L	(Ions score 35)
48 - 55	489.2640	976.5134	976.5090	5	1	R.FRADNLNK.L	(Ions score 21)
50 - 57	458.2672	914.5198	914.5185	1	1	R.ADNLNKLK.N	(Ions score 51)
50 - 57	479.2728	956.5310	956.5291	2	1	R.ADNLNKLK.N	Acetylation (K) (Ions score 31)
50 - 57	479.2733	956.5320	956.5291	3	1	R.ADNLNKLK.N	Acetylation (K) (Ions score 39)
56 - 62	452.7510	903.4874	903.4848	3	1	K.LKNLCSK.T	Acetylation (K) (Ions score 43)
58 - 67	575.3001	1148.5856	1148.5859	-0	1	K.NLCSKTIGEK.M	(Ions score 34)
58 - 67	575.3006	1148.5867	1148.5859	1	1	K.NLCSKTIGEK.M	(Ions score 25)
58 - 67	575.3006	1148.5867	1148.5859	1	1	K.NLCSKTIGEK.M	(Ions score 42)
58 - 67	575.3009	1148.5872	1148.5859	1	1	K.NLCSKTIGEK.M	(Ions score 35)
58 - 67	575.3009	1148.5872	1148.5859	1	1	K.NLCSKTIGEK.M	(Ions score 37)
58 - 67	575.3011	1148.5875	1148.5859	1	1	K.NLCSKTIGEK.M	(Ions score 40)
58 - 67	575.3013	1148.5880	1148.5859	2	1	K.NLCSKTIGEK.M	(Ions score 44)
58 - 67	575.3013	1148.5881	1148.5859	2	1	K.NLCSKTIGEK.M	(Ions score 42)
58 - 67	575.3015	1148.5884	1148.5859	2	1	K.NLCSKTIGEK.M	(Ions score 32)
58 - 67	575.3015	1148.5884	1148.5859	2	1	K.NLCSKTIGEK.M	(Ions score 25)
58 - 67	575.3017	1148.5888	1148.5859	2	1	K.NLCSKTIGEK.M	(Ions score 42)
58 - 67	575.3017	1148.5889	1148.5859	3	1	K.NLCSKTIGEK.M	(Ions score 38)
58 - 67	575.3018	1148.5890	1148.5859	3	1	K.NLCSKTIGEK.M	(Ions score 39)
58 - 67	575.3021	1148.5896	1148.5859	3	1	K.NLCSKTIGEK.M	(Ions score 28)
63 - 67	547.3085	546.3012	546.3013	-0	0	K.TIGEK.M	(Ions score 24)
63 - 67	547.3090	546.3017	546.3013	1	0	K.TIGEK.M	(Ions score 26)
63 - 67	547.3090	546.3017	546.3013	1	0	K.TIGEK.M	(Ions score 26)
63 - 67	547.3090	546.3017	546.3013	1	0	K.TIGEK.M	(Ions score 26)
63 - 67	547.3090	546.3017	546.3013	1	0	K.TIGEK.M	(Ions score 25)
63 - 67	547.3091	546.3019	546.3013	1	0	K.TIGEK.M	(Ions score 26)
63 - 67	547.3092	546.3019	546.3013	1	0	K.TIGEK.M	(Ions score 25)
63 - 67	547.3097	546.3024	546.3013	2	0	K.TIGEK.M	(Ions score 25)

63 - 67	547.3105	546.3033	546.3013	4	0	K.TIGEK.M (Ions score 24)
71 - 99	1034.5149	3100.5229	3100.5088	5	1	K.KEPVGDDESVPENVLSFDDLTADALANLK.V (Ions score 66)
72 - 99	991.8147	2972.4222	2972.4138	3	0	K.EPVGDDESVPENVLSFDDLTADALANLK.V (Ions score 84)
100 - 104	574.3563	573.3490	573.3486	1	0	K.VSQIK.K (Ions score 23)
100 - 104	574.3564	573.3492	573.3486	1	0	K.VSQIK.K (Ions score 23)
100 - 104	574.3566	573.3493	573.3486	1	0	K.VSQIK.K (Ions score 22)
100 - 104	574.3572	573.3499	573.3486	2	0	K.VSQIK.K (Ions score 23)
108 - 116	514.3236	1026.6327	1026.6325	0	0	R.LLIDEAILK.C (Ions score 45)
108 - 116	514.3236	1026.6327	1026.6325	0	0	R.LLIDEAILK.C (Ions score 46)
122 - 128	429.7564	857.4982	857.4970	1	1	R.IKLEAER.F (Ions score 27)
122 - 128	429.7571	857.4996	857.4970	3	1	R.IKLEAER.F (Ions score 31)
124 - 133	638.8368	1275.6590	1275.6571	1	1	K.LEAERFENLR.E (Ions score 32)
124 - 133	638.8376	1275.6607	1275.6571	3	1	K.LEAERFENLR.E (Ions score 34)
124 - 133	638.8379	1275.6613	1275.6571	3	1	K.LEAERFENLR.E (Ions score 47)
124 - 133	638.8382	1275.6618	1275.6571	4	1	K.LEAERFENLR.E (Ions score 32)
129 - 133	678.3590	677.3517	677.3497	3	0	R.FENLR.E (Ions score 28)
129 - 133	678.3596	677.3523	677.3497	4	0	R.FENLR.E (Ions score 23)
129 - 133	678.3596	677.3523	677.3497	4	0	R.FENLR.E (Ions score 29)
129 - 133	678.3597	677.3524	677.3497	4	0	R.FENLR.E (Ions score 27)
129 - 133	678.3597	677.3525	677.3497	4	0	R.FENLR.E (Ions score 23)
129 - 133	678.3597	677.3525	677.3497	4	0	R.FENLR.E (Ions score 28)
129 - 133	678.3600	677.3527	677.3497	5	0	R.FENLR.E (Ions score 21)
134 - 154	769.0479	2304.1219	2304.1234	-1	0	R.EIGNLLHPSVPISNDEDVDNK.V (Ions score 24)
134 - 154	769.0489	2304.1249	2304.1234	1	0	R.EIGNLLHPSVPISNDEDVDNK.V (Ions score 60)
134 - 157	673.0924	2688.3404	2688.3355	2	1	R.EIGNLLHPSVPISNDEDVDNKVER.I (Ions score 40)
134 - 157	673.0925	2688.3411	2688.3355	2	1	R.EIGNLLHPSVPISNDEDVDNKVER.I (Ions score 31)
134 - 157	673.0929	2688.3425	2688.3355	3	1	R.EIGNLLHPSVPISNDEDVDNKVER.I (Ions score 44)
134 - 157	897.1216	2688.3428	2688.3355	3	1	R.EIGNLLHPSVPISNDEDVDNKVER.I (Ions score 88)
134 - 157	673.0931	2688.3432	2688.3355	3	1	R.EIGNLLHPSVPISNDEDVDNKVER.I (Ions score 61)
134 - 157	897.1217	2688.3433	2688.3355	3	1	R.EIGNLLHPSVPISNDEDVDNKVER.I (Ions score 105)
134 - 157	673.0933	2688.3441	2688.3355	3	1	R.EIGNLLHPSVPISNDEDVDNKVER.I (Ions score 28)
134 - 157	897.1228	2688.3466	2688.3355	4	1	R.EIGNLLHPSVPISNDEDVDNKVER.I (Ions score 93)
158 - 165	503.7412	1005.4678	1005.4702	-2	0	R.IWGDCTVR.K (Ions score 36)
158 - 165	503.7412	1005.4679	1005.4702	-2	0	R.IWGDCTVR.K (Ions score 33)
158 - 165	503.7413	1005.4681	1005.4702	-2	0	R.IWGDCTVR.K (Ions score 36)
158 - 165	503.7414	1005.4683	1005.4702	-2	0	R.IWGDCTVR.K (Ions score 33)
158 - 165	503.7416	1005.4687	1005.4702	-2	0	R.IWGDCTVR.K (Ions score 33)
158 - 165	503.7418	1005.4690	1005.4702	-1	0	R.IWGDCTVR.K (Ions score 33)
158 - 165	503.7420	1005.4694	1005.4702	-1	0	R.IWGDCTVR.K (Ions score 33)
158 - 165	503.7420	1005.4694	1005.4702	-1	0	R.IWGDCTVR.K (Ions score 39)
158 - 165	503.7422	1005.4699	1005.4702	-0	0	R.IWGDCTVR.K (Ions score 35)
158 - 165	503.7423	1005.4700	1005.4702	-0	0	R.IWGDCTVR.K (Ions score 37)
158 - 165	503.7423	1005.4701	1005.4702	-0	0	R.IWGDCTVR.K (Ions score 39)
158 - 165	503.7424	1005.4702	1005.4702	0	0	R.IWGDCTVR.K (Ions score 39)
158 - 165	503.7424	1005.4703	1005.4702	0	0	R.IWGDCTVR.K (Ions score 35)
158 - 165	503.7425	1005.4704	1005.4702	0	0	R.IWGDCTVR.K (Ions score 36)
158 - 165	503.7426	1005.4707	1005.4702	0	0	R.IWGDCTVR.K (Ions score 39)
158 - 165	503.7426	1005.4707	1005.4702	0	0	R.IWGDCTVR.K (Ions score 33)
158 - 165	503.7426	1005.4707	1005.4702	1	0	R.IWGDCTVR.K (Ions score 21)
158 - 165	503.7427	1005.4709	1005.4702	1	0	R.IWGDCTVR.K (Ions score 39)
158 - 165	503.7427	1005.4709	1005.4702	1	0	R.IWGDCTVR.K (Ions score 34)
158 - 165	503.7427	1005.4709	1005.4702	1	0	R.IWGDCTVR.K (Ions score 32)
158 - 165	503.7428	1005.4711	1005.4702	1	0	R.IWGDCTVR.K (Ions score 33)
158 - 165	503.7428	1005.4711	1005.4702	1	0	R.IWGDCTVR.K (Ions score 38)
158 - 165	503.7429	1005.4712	1005.4702	1	0	R.IWGDCTVR.K (Ions score 35)
158 - 165	503.7429	1005.4713	1005.4702	1	0	R.IWGDCTVR.K (Ions score 26)
158 - 165	503.7429	1005.4713	1005.4702	1	0	R.IWGDCTVR.K (Ions score 35)

158 - 165	503.7430	1005.4714	1005.4702	1	0	R.IWGDCTVR.K	(Ions score 36)
158 - 165	503.7430	1005.4715	1005.4702	1	0	R.IWGDCTVR.K	(Ions score 25)
158 - 165	503.7430	1005.4715	1005.4702	1	0	R.IWGDCTVR.K	(Ions score 39)
158 - 165	503.7430	1005.4715	1005.4702	1	0	R.IWGDCTVR.K	(Ions score 39)
158 - 165	503.7431	1005.4716	1005.4702	1	0	R.IWGDCTVR.K	(Ions score 38)
158 - 165	503.7431	1005.4716	1005.4702	1	0	R.IWGDCTVR.K	(Ions score 35)
158 - 165	503.7431	1005.4717	1005.4702	1	0	R.IWGDCTVR.K	(Ions score 35)
158 - 165	503.7431	1005.4717	1005.4702	1	0	R.IWGDCTVR.K	(Ions score 35)
158 - 165	503.7431	1005.4717	1005.4702	1	0	R.IWGDCTVR.K	(Ions score 36)
158 - 165	503.7431	1005.4717	1005.4702	1	0	R.IWGDCTVR.K	(Ions score 39)
158 - 165	503.7431	1005.4717	1005.4702	1	0	R.IWGDCTVR.K	(Ions score 38)
158 - 165	503.7431	1005.4717	1005.4702	2	0	R.IWGDCTVR.K	(Ions score 21)
158 - 165	503.7432	1005.4718	1005.4702	2	0	R.IWGDCTVR.K	(Ions score 39)
158 - 165	503.7432	1005.4718	1005.4702	2	0	R.IWGDCTVR.K	(Ions score 35)
158 - 165	503.7432	1005.4719	1005.4702	2	0	R.IWGDCTVR.K	(Ions score 38)
158 - 165	503.7432	1005.4719	1005.4702	2	0	R.IWGDCTVR.K	(Ions score 37)
158 - 165	503.7432	1005.4719	1005.4702	2	0	R.IWGDCTVR.K	(Ions score 35)
158 - 165	503.7432	1005.4719	1005.4702	2	0	R.IWGDCTVR.K	(Ions score 35)
158 - 165	503.7432	1005.4719	1005.4702	2	0	R.IWGDCTVR.K	(Ions score 37)
158 - 165	503.7432	1005.4719	1005.4702	2	0	R.IWGDCTVR.K	(Ions score 36)
158 - 165	503.7432	1005.4719	1005.4702	2	0	R.IWGDCTVR.K	(Ions score 36)
158 - 165	503.7433	1005.4720	1005.4702	2	0	R.IWGDCTVR.K	(Ions score 35)
158 - 165	503.7433	1005.4720	1005.4702	2	0	R.IWGDCTVR.K	(Ions score 40)
158 - 165	503.7433	1005.4720	1005.4702	2	0	R.IWGDCTVR.K	(Ions score 35)
158 - 165	503.7433	1005.4720	1005.4702	2	0	R.IWGDCTVR.K	(Ions score 36)
158 - 165	503.7433	1005.4721	1005.4702	2	0	R.IWGDCTVR.K	(Ions score 36)
158 - 165	503.7433	1005.4721	1005.4702	2	0	R.IWGDCTVR.K	(Ions score 36)
158 - 165	503.7433	1005.4721	1005.4702	2	0	R.IWGDCTVR.K	(Ions score 35)
158 - 165	503.7433	1005.4721	1005.4702	2	0	R.IWGDCTVR.K	(Ions score 35)
158 - 165	503.7433	1005.4721	1005.4702	2	0	R.IWGDCTVR.K	(Ions score 35)
158 - 165	503.7433	1005.4721	1005.4702	2	0	R.IWGDCTVR.K	(Ions score 35)
158 - 165	503.7433	1005.4721	1005.4702	2	0	R.IWGDCTVR.K	(Ions score 39)
158 - 165	503.7434	1005.4722	1005.4702	2	0	R.IWGDCTVR.K	(Ions score 35)
158 - 165	503.7434	1005.4723	1005.4702	2	0	R.IWGDCTVR.K	(Ions score 36)
158 - 165	503.7434	1005.4723	1005.4702	2	0	R.IWGDCTVR.K	(Ions score 39)
158 - 165	503.7435	1005.4724	1005.4702	2	0	R.IWGDCTVR.K	(Ions score 39)
158 - 165	503.7435	1005.4724	1005.4702	2	0	R.IWGDCTVR.K	(Ions score 35)
158 - 165	503.7435	1005.4724	1005.4702	2	0	R.IWGDCTVR.K	(Ions score 35)
158 - 165	503.7435	1005.4724	1005.4702	2	0	R.IWGDCTVR.K	(Ions score 38)
158 - 165	503.7435	1005.4724	1005.4702	2	0	R.IWGDCTVR.K	(Ions score 35)
158 - 165	503.7435	1005.4724	1005.4702	2	0	R.IWGDCTVR.K	(Ions score 36)
158 - 165	503.7435	1005.4725	1005.4702	2	0	R.IWGDCTVR.K	(Ions score 38)
158 - 165	503.7435	1005.4725	1005.4702	2	0	R.IWGDCTVR.K	(Ions score 31)
158 - 165	503.7435	1005.4725	1005.4702	2	0	R.IWGDCTVR.K	(Ions score 35)
158 - 165	503.7435	1005.4725	1005.4702	2	0	R.IWGDCTVR.K	(Ions score 35)
158 - 165	503.7435	1005.4725	1005.4702	2	0	R.IWGDCTVR.K	(Ions score 40)
158 - 165	503.7435	1005.4725	1005.4702	2	0	R.IWGDCTVR.K	(Ions score 35)
158 - 165	503.7436	1005.4726	1005.4702	2	0	R.IWGDCTVR.K	(Ions score 39)
158 - 165	503.7436	1005.4726	1005.4702	2	0	R.IWGDCTVR.K	(Ions score 39)
158 - 165	503.7436	1005.4726	1005.4702	2	0	R.IWGDCTVR.K	(Ions score 23)
158 - 165	503.7436	1005.4726	1005.4702	2	0	R.IWGDCTVR.K	(Ions score 39)
158 - 165	503.7436	1005.4726	1005.4702	2	0	R.IWGDCTVR.K	(Ions score 35)
158 - 165	503.7436	1005.4726	1005.4702	2	0	R.IWGDCTVR.K	(Ions score 38)
158 - 165	503.7436	1005.4727	1005.4702	2	0	R.IWGDCTVR.K	(Ions score 35)
158 - 165	503.7436	1005.4727	1005.4702	2	0	R.IWGDCTVR.K	(Ions score 35)
158 - 165	503.7436	1005.4727	1005.4702	2	0	R.IWGDCTVR.K	(Ions score 35)
158 - 165	503.7436	1005.4727	1005.4702	3	0	R.IWGDCTVR.K	(Ions score 36)

158 - 165	503.7437	1005.4728	1005.4702	3	0	R.IWGDCTVR.K	(Ions score 35)
158 - 165	503.7437	1005.4729	1005.4702	3	0	R.IWGDCTVR.K	(Ions score 39)
158 - 165	503.7437	1005.4729	1005.4702	3	0	R.IWGDCTVR.K	(Ions score 31)
158 - 165	503.7438	1005.4730	1005.4702	3	0	R.IWGDCTVR.K	(Ions score 38)
158 - 165	503.7438	1005.4730	1005.4702	3	0	R.IWGDCTVR.K	(Ions score 35)
158 - 165	503.7438	1005.4730	1005.4702	3	0	R.IWGDCTVR.K	(Ions score 35)
158 - 165	503.7438	1005.4731	1005.4702	3	0	R.IWGDCTVR.K	(Ions score 37)
158 - 165	503.7438	1005.4731	1005.4702	3	0	R.IWGDCTVR.K	(Ions score 34)
158 - 165	503.7438	1005.4731	1005.4702	3	0	R.IWGDCTVR.K	(Ions score 33)
158 - 165	503.7438	1005.4731	1005.4702	3	0	R.IWGDCTVR.K	(Ions score 39)
158 - 165	503.7438	1005.4731	1005.4702	3	0	R.IWGDCTVR.K	(Ions score 38)
158 - 165	503.7439	1005.4732	1005.4702	3	0	R.IWGDCTVR.K	(Ions score 33)
158 - 165	503.7439	1005.4732	1005.4702	3	0	R.IWGDCTVR.K	(Ions score 42)
158 - 165	503.7439	1005.4732	1005.4702	3	0	R.IWGDCTVR.K	(Ions score 40)
158 - 165	503.7439	1005.4732	1005.4702	3	0	R.IWGDCTVR.K	(Ions score 37)
158 - 165	503.7439	1005.4733	1005.4702	3	0	R.IWGDCTVR.K	(Ions score 40)
158 - 165	503.7439	1005.4733	1005.4702	3	0	R.IWGDCTVR.K	(Ions score 32)
158 - 165	503.7439	1005.4733	1005.4702	3	0	R.IWGDCTVR.K	(Ions score 42)
158 - 165	503.7440	1005.4734	1005.4702	3	0	R.IWGDCTVR.K	(Ions score 35)
158 - 165	503.7440	1005.4734	1005.4702	3	0	R.IWGDCTVR.K	(Ions score 38)
158 - 165	503.7440	1005.4734	1005.4702	3	0	R.IWGDCTVR.K	(Ions score 42)
158 - 165	503.7440	1005.4734	1005.4702	3	0	R.IWGDCTVR.K	(Ions score 35)
158 - 165	503.7440	1005.4734	1005.4702	3	0	R.IWGDCTVR.K	(Ions score 35)
158 - 165	503.7440	1005.4734	1005.4702	3	0	R.IWGDCTVR.K	(Ions score 36)
158 - 165	503.7440	1005.4734	1005.4702	3	0	R.IWGDCTVR.K	(Ions score 35)
158 - 165	503.7440	1005.4735	1005.4702	3	0	R.IWGDCTVR.K	(Ions score 40)
158 - 165	503.7440	1005.4735	1005.4702	3	0	R.IWGDCTVR.K	(Ions score 36)
158 - 165	503.7440	1005.4735	1005.4702	3	0	R.IWGDCTVR.K	(Ions score 36)
158 - 165	503.7440	1005.4735	1005.4702	3	0	R.IWGDCTVR.K	(Ions score 41)
158 - 165	503.7441	1005.4736	1005.4702	3	0	R.IWGDCTVR.K	(Ions score 21)
158 - 165	503.7441	1005.4736	1005.4702	3	0	R.IWGDCTVR.K	(Ions score 35)
158 - 165	503.7441	1005.4737	1005.4702	3	0	R.IWGDCTVR.K	(Ions score 36)
158 - 165	503.7442	1005.4738	1005.4702	4	0	R.IWGDCTVR.K	(Ions score 41)
158 - 165	503.7442	1005.4738	1005.4702	4	0	R.IWGDCTVR.K	(Ions score 40)
158 - 165	503.7443	1005.4740	1005.4702	4	0	R.IWGDCTVR.K	(Ions score 35)
158 - 165	503.7443	1005.4740	1005.4702	4	0	R.IWGDCTVR.K	(Ions score 35)
158 - 165	503.7444	1005.4742	1005.4702	4	0	R.IWGDCTVR.K	(Ions score 32)
158 - 165	503.7444	1005.4742	1005.4702	4	0	R.IWGDCTVR.K	(Ions score 38)
158 - 165	503.7444	1005.4742	1005.4702	4	0	R.IWGDCTVR.K	(Ions score 33)
158 - 165	503.7444	1005.4743	1005.4702	4	0	R.IWGDCTVR.K	(Ions score 21)
158 - 165	503.7447	1005.4749	1005.4702	5	0	R.IWGDCTVR.K	(Ions score 36)
158 - 166	567.7917	1133.5688	1133.5652	3	1	R.IWGDCTVRK.K	(Ions score 23)
167 - 184	1026.5044	2050.9943	2051.0034	-4	1	K.KYSHVDLVVMVDGFEGEK.G	(Ions score 22)
167 - 184	1026.5066	2050.9986	2051.0034	-2	1	K.KYSHVDLVVMVDGFEGEK.G	(Ions score 66)
167 - 184	1026.5072	2050.9998	2051.0034	-2	1	K.KYSHVDLVVMVDGFEGEK.G	(Ions score 77)
167 - 184	684.6742	2051.0007	2051.0034	-1	1	K.KYSHVDLVVMVDGFEGEK.G	(Ions score 22)
167 - 184	1026.5078	2051.0011	2051.0034	-1	1	K.KYSHVDLVVMVDGFEGEK.G	(Ions score 72)
167 - 184	513.7577	2051.0017	2051.0034	-1	1	K.KYSHVDLVVMVDGFEGEK.G	(Ions score 26)
167 - 184	684.6745	2051.0018	2051.0034	-1	1	K.KYSHVDLVVMVDGFEGEK.G	(Ions score 33)
167 - 184	513.7578	2051.0021	2051.0034	-1	1	K.KYSHVDLVVMVDGFEGEK.G	(Ions score 29)
167 - 184	1026.5085	2051.0025	2051.0034	-0	1	K.KYSHVDLVVMVDGFEGEK.G	(Ions score 22)
167 - 184	684.6748	2051.0026	2051.0034	-0	1	K.KYSHVDLVVMVDGFEGEK.G	(Ions score 23)
167 - 184	684.6748	2051.0027	2051.0034	-0	1	K.KYSHVDLVVMVDGFEGEK.G	(Ions score 22)
167 - 184	684.6749	2051.0028	2051.0034	-0	1	K.KYSHVDLVVMVDGFEGEK.G	(Ions score 47)
167 - 184	684.6749	2051.0028	2051.0034	-0	1	K.KYSHVDLVVMVDGFEGEK.G	(Ions score 25)
167 - 184	513.7580	2051.0029	2051.0034	-0	1	K.KYSHVDLVVMVDGFEGEK.G	(Ions score 36)
167 - 184	513.7581	2051.0031	2051.0034	-0	1	K.KYSHVDLVVMVDGFEGEK.G	(Ions score 25)

167 - 184	513.7581	2051.0031	2051.0034	-0	1	K.KYSHVDLVVMVDGFEGEK.G	(Ions score 31)
167 - 184	684.6750	2051.0031	2051.0034	-0	1	K.KYSHVDLVVMVDGFEGEK.G	(Ions score 57)
167 - 184	684.6750	2051.0033	2051.0034	-0	1	K.KYSHVDLVVMVDGFEGEK.G	(Ions score 26)
167 - 184	513.7581	2051.0033	2051.0034	-0	1	K.KYSHVDLVVMVDGFEGEK.G	(Ions score 43)
167 - 184	513.7581	2051.0034	2051.0034	-0	1	K.KYSHVDLVVMVDGFEGEK.G	(Ions score 38)
167 - 184	684.6751	2051.0034	2051.0034	0	1	K.KYSHVDLVVMVDGFEGEK.G	(Ions score 37)
167 - 184	684.6751	2051.0036	2051.0034	0	1	K.KYSHVDLVVMVDGFEGEK.G	(Ions score 82)
167 - 184	513.7582	2051.0036	2051.0034	0	1	K.KYSHVDLVVMVDGFEGEK.G	(Ions score 32)
167 - 184	513.7582	2051.0036	2051.0034	0	1	K.KYSHVDLVVMVDGFEGEK.G	(Ions score 23)
167 - 184	513.7582	2051.0039	2051.0034	0	1	K.KYSHVDLVVMVDGFEGEK.G	(Ions score 50)
167 - 184	684.6752	2051.0039	2051.0034	0	1	K.KYSHVDLVVMVDGFEGEK.G	(Ions score 26)
167 - 184	513.7583	2051.0041	2051.0034	0	1	K.KYSHVDLVVMVDGFEGEK.G	(Ions score 36)
167 - 184	684.6754	2051.0043	2051.0034	0	1	K.KYSHVDLVVMVDGFEGEK.G	(Ions score 22)
167 - 184	684.6754	2051.0044	2051.0034	0	1	K.KYSHVDLVVMVDGFEGEK.G	(Ions score 22)
167 - 184	1026.5098	2051.0050	2051.0034	1	1	K.KYSHVDLVVMVDGFEGEK.G	(Ions score 94)
167 - 184	513.7585	2051.0050	2051.0034	1	1	K.KYSHVDLVVMVDGFEGEK.G	(Ions score 44)
167 - 184	513.7585	2051.0051	2051.0034	1	1	K.KYSHVDLVVMVDGFEGEK.G	(Ions score 27)
167 - 184	684.6757	2051.0052	2051.0034	1	1	K.KYSHVDLVVMVDGFEGEK.G	(Ions score 48)
167 - 184	513.7586	2051.0054	2051.0034	1	1	K.KYSHVDLVVMVDGFEGEK.G	(Ions score 27)
167 - 184	684.6758	2051.0056	2051.0034	1	1	K.KYSHVDLVVMVDGFEGEK.G	(Ions score 28)
167 - 184	684.6758	2051.0056	2051.0034	1	1	K.KYSHVDLVVMVDGFEGEK.G	(Ions score 58)
167 - 184	513.7587	2051.0057	2051.0034	1	1	K.KYSHVDLVVMVDGFEGEK.G	(Ions score 53)
167 - 184	684.6758	2051.0057	2051.0034	1	1	K.KYSHVDLVVMVDGFEGEK.G	(Ions score 28)
167 - 184	513.7587	2051.0059	2051.0034	1	1	K.KYSHVDLVVMVDGFEGEK.G	(Ions score 43)
167 - 184	684.6759	2051.0059	2051.0034	1	1	K.KYSHVDLVVMVDGFEGEK.G	(Ions score 47)
167 - 184	684.6759	2051.0059	2051.0034	1	1	K.KYSHVDLVVMVDGFEGEK.G	(Ions score 47)
167 - 184	684.6759	2051.0060	2051.0034	1	1	K.KYSHVDLVVMVDGFEGEK.G	(Ions score 46)
167 - 184	684.6759	2051.0060	2051.0034	1	1	K.KYSHVDLVVMVDGFEGEK.G	(Ions score 24)
167 - 184	513.7588	2051.0061	2051.0034	1	1	K.KYSHVDLVVMVDGFEGEK.G	(Ions score 50)
167 - 184	684.6760	2051.0061	2051.0034	1	1	K.KYSHVDLVVMVDGFEGEK.G	(Ions score 27)
167 - 184	684.6760	2051.0061	2051.0034	1	1	K.KYSHVDLVVMVDGFEGEK.G	(Ions score 37)
167 - 184	513.7588	2051.0061	2051.0034	1	1	K.KYSHVDLVVMVDGFEGEK.G	(Ions score 35)
167 - 184	684.6760	2051.0061	2051.0034	1	1	K.KYSHVDLVVMVDGFEGEK.G	(Ions score 35)
167 - 184	684.6760	2051.0062	2051.0034	1	1	K.KYSHVDLVVMVDGFEGEK.G	(Ions score 28)
167 - 184	513.7588	2051.0062	2051.0034	1	1	K.KYSHVDLVVMVDGFEGEK.G	(Ions score 44)
167 - 184	684.6760	2051.0062	2051.0034	1	1	K.KYSHVDLVVMVDGFEGEK.G	(Ions score 44)
167 - 184	684.6760	2051.0062	2051.0034	1	1	K.KYSHVDLVVMVDGFEGEK.G	(Ions score 22)
167 - 184	684.6760	2051.0062	2051.0034	1	1	K.KYSHVDLVVMVDGFEGEK.G	(Ions score 37)
167 - 184	513.7588	2051.0063	2051.0034	1	1	K.KYSHVDLVVMVDGFEGEK.G	(Ions score 45)
167 - 184	513.7589	2051.0063	2051.0034	1	1	K.KYSHVDLVVMVDGFEGEK.G	(Ions score 34)
167 - 184	684.6760	2051.0063	2051.0034	1	1	K.KYSHVDLVVMVDGFEGEK.G	(Ions score 42)
167 - 184	684.6761	2051.0063	2051.0034	1	1	K.KYSHVDLVVMVDGFEGEK.G	(Ions score 22)
167 - 184	513.7589	2051.0063	2051.0034	1	1	K.KYSHVDLVVMVDGFEGEK.G	(Ions score 45)
167 - 184	684.6761	2051.0064	2051.0034	1	1	K.KYSHVDLVVMVDGFEGEK.G	(Ions score 22)
167 - 184	1026.5105	2051.0064	2051.0034	1	1	K.KYSHVDLVVMVDGFEGEK.G	(Ions score 77)
167 - 184	684.6761	2051.0064	2051.0034	1	1	K.KYSHVDLVVMVDGFEGEK.G	(Ions score 35)
167 - 184	684.6761	2051.0065	2051.0034	2	1	K.KYSHVDLVVMVDGFEGEK.G	(Ions score 56)
167 - 184	513.7589	2051.0065	2051.0034	2	1	K.KYSHVDLVVMVDGFEGEK.G	(Ions score 31)
167 - 184	684.6761	2051.0065	2051.0034	2	1	K.KYSHVDLVVMVDGFEGEK.G	(Ions score 58)
167 - 184	513.7589	2051.0065	2051.0034	2	1	K.KYSHVDLVVMVDGFEGEK.G	(Ions score 37)
167 - 184	684.6762	2051.0066	2051.0034	2	1	K.KYSHVDLVVMVDGFEGEK.G	(Ions score 48)
167 - 184	684.6762	2051.0067	2051.0034	2	1	K.KYSHVDLVVMVDGFEGEK.G	(Ions score 36)
167 - 184	513.7590	2051.0067	2051.0034	2	1	K.KYSHVDLVVMVDGFEGEK.G	(Ions score 41)
167 - 184	684.6762	2051.0067	2051.0034	2	1	K.KYSHVDLVVMVDGFEGEK.G	(Ions score 44)
167 - 184	513.7590	2051.0068	2051.0034	2	1	K.KYSHVDLVVMVDGFEGEK.G	(Ions score 39)
167 - 184	684.6762	2051.0068	2051.0034	2	1	K.KYSHVDLVVMVDGFEGEK.G	(Ions score 52)
167 - 184	684.6762	2051.0068	2051.0034	2	1	K.KYSHVDLVVMVDGFEGEK.G	(Ions score 43)

167 - 184	684.6762	2051.0068	2051.0034	2	1	K.KYSHVDLVVMVDGFEGEK.G	(Ions score 45)
167 - 184	1026.5107	2051.0069	2051.0034	2	1	K.KYSHVDLVVMVDGFEGEK.G	(Ions score 86)
167 - 184	684.6763	2051.0069	2051.0034	2	1	K.KYSHVDLVVMVDGFEGEK.G	(Ions score 54)
167 - 184	684.6763	2051.0070	2051.0034	2	1	K.KYSHVDLVVMVDGFEGEK.G	(Ions score 46)
167 - 184	684.6763	2051.0070	2051.0034	2	1	K.KYSHVDLVVMVDGFEGEK.G	(Ions score 29)
167 - 184	684.6763	2051.0070	2051.0034	2	1	K.KYSHVDLVVMVDGFEGEK.G	(Ions score 47)
167 - 184	513.7590	2051.0070	2051.0034	2	1	K.KYSHVDLVVMVDGFEGEK.G	(Ions score 47)
167 - 184	513.7590	2051.0070	2051.0034	2	1	K.KYSHVDLVVMVDGFEGEK.G	(Ions score 45)
167 - 184	513.7590	2051.0070	2051.0034	2	1	K.KYSHVDLVVMVDGFEGEK.G	(Ions score 35)
167 - 184	684.6763	2051.0071	2051.0034	2	1	K.KYSHVDLVVMVDGFEGEK.G	(Ions score 47)
167 - 184	684.6763	2051.0071	2051.0034	2	1	K.KYSHVDLVVMVDGFEGEK.G	(Ions score 47)
167 - 184	684.6763	2051.0071	2051.0034	2	1	K.KYSHVDLVVMVDGFEGEK.G	(Ions score 52)
167 - 184	513.7591	2051.0071	2051.0034	2	1	K.KYSHVDLVVMVDGFEGEK.G	(Ions score 22)
167 - 184	1026.5108	2051.0071	2051.0034	2	1	K.KYSHVDLVVMVDGFEGEK.G	(Ions score 77)
167 - 184	684.6763	2051.0071	2051.0034	2	1	K.KYSHVDLVVMVDGFEGEK.G	(Ions score 50)
167 - 184	513.7591	2051.0071	2051.0034	2	1	K.KYSHVDLVVMVDGFEGEK.G	(Ions score 37)
167 - 184	513.7591	2051.0071	2051.0034	2	1	K.KYSHVDLVVMVDGFEGEK.G	(Ions score 26)
167 - 184	684.6763	2051.0072	2051.0034	2	1	K.KYSHVDLVVMVDGFEGEK.G	(Ions score 43)
167 - 184	513.7591	2051.0072	2051.0034	2	1	K.KYSHVDLVVMVDGFEGEK.G	(Ions score 41)
167 - 184	513.7591	2051.0072	2051.0034	2	1	K.KYSHVDLVVMVDGFEGEK.G	(Ions score 30)
167 - 184	513.7591	2051.0072	2051.0034	2	1	K.KYSHVDLVVMVDGFEGEK.G	(Ions score 56)
167 - 184	684.6764	2051.0073	2051.0034	2	1	K.KYSHVDLVVMVDGFEGEK.G	(Ions score 23)
167 - 184	684.6764	2051.0073	2051.0034	2	1	K.KYSHVDLVVMVDGFEGEK.G	(Ions score 73)
167 - 184	684.6764	2051.0075	2051.0034	2	1	K.KYSHVDLVVMVDGFEGEK.G	(Ions score 44)
167 - 184	684.6764	2051.0075	2051.0034	2	1	K.KYSHVDLVVMVDGFEGEK.G	(Ions score 47)
167 - 184	684.6764	2051.0075	2051.0034	2	1	K.KYSHVDLVVMVDGFEGEK.G	(Ions score 52)
167 - 184	513.7592	2051.0075	2051.0034	2	1	K.KYSHVDLVVMVDGFEGEK.G	(Ions score 41)
167 - 184	513.7592	2051.0075	2051.0034	2	1	K.KYSHVDLVVMVDGFEGEK.G	(Ions score 56)
167 - 184	684.6765	2051.0075	2051.0034	2	1	K.KYSHVDLVVMVDGFEGEK.G	(Ions score 49)
167 - 184	684.6765	2051.0075	2051.0034	2	1	K.KYSHVDLVVMVDGFEGEK.G	(Ions score 28)
167 - 184	513.7592	2051.0075	2051.0034	2	1	K.KYSHVDLVVMVDGFEGEK.G	(Ions score 44)
167 - 184	684.6765	2051.0076	2051.0034	2	1	K.KYSHVDLVVMVDGFEGEK.G	(Ions score 55)
167 - 184	684.6765	2051.0076	2051.0034	2	1	K.KYSHVDLVVMVDGFEGEK.G	(Ions score 59)
167 - 184	684.6765	2051.0076	2051.0034	2	1	K.KYSHVDLVVMVDGFEGEK.G	(Ions score 53)
167 - 184	684.6765	2051.0076	2051.0034	2	1	K.KYSHVDLVVMVDGFEGEK.G	(Ions score 62)
167 - 184	684.6765	2051.0076	2051.0034	2	1	K.KYSHVDLVVMVDGFEGEK.G	(Ions score 46)
167 - 184	513.7592	2051.0076	2051.0034	2	1	K.KYSHVDLVVMVDGFEGEK.G	(Ions score 42)
167 - 184	513.7592	2051.0076	2051.0034	2	1	K.KYSHVDLVVMVDGFEGEK.G	(Ions score 38)
167 - 184	513.7592	2051.0076	2051.0034	2	1	K.KYSHVDLVVMVDGFEGEK.G	(Ions score 36)
167 - 184	684.6765	2051.0076	2051.0034	2	1	K.KYSHVDLVVMVDGFEGEK.G	(Ions score 28)
167 - 184	513.7592	2051.0077	2051.0034	2	1	K.KYSHVDLVVMVDGFEGEK.G	(Ions score 40)
167 - 184	1026.5111	2051.0077	2051.0034	2	1	K.KYSHVDLVVMVDGFEGEK.G	(Ions score 93)
167 - 184	684.6765	2051.0077	2051.0034	2	1	K.KYSHVDLVVMVDGFEGEK.G	(Ions score 67)
167 - 184	684.6765	2051.0077	2051.0034	2	1	K.KYSHVDLVVMVDGFEGEK.G	(Ions score 49)
167 - 184	513.7592	2051.0077	2051.0034	2	1	K.KYSHVDLVVMVDGFEGEK.G	(Ions score 40)
167 - 184	684.6765	2051.0077	2051.0034	2	1	K.KYSHVDLVVMVDGFEGEK.G	(Ions score 45)
167 - 184	513.7592	2051.0077	2051.0034	2	1	K.KYSHVDLVVMVDGFEGEK.G	(Ions score 22)
167 - 184	513.7592	2051.0078	2051.0034	2	1	K.KYSHVDLVVMVDGFEGEK.G	(Ions score 42)
167 - 184	684.6765	2051.0078	2051.0034	2	1	K.KYSHVDLVVMVDGFEGEK.G	(Ions score 48)
167 - 184	513.7592	2051.0078	2051.0034	2	1	K.KYSHVDLVVMVDGFEGEK.G	(Ions score 46)
167 - 184	684.6766	2051.0078	2051.0034	2	1	K.KYSHVDLVVMVDGFEGEK.G	(Ions score 53)
167 - 184	513.7592	2051.0078	2051.0034	2	1	K.KYSHVDLVVMVDGFEGEK.G	(Ions score 51)
167 - 184	513.7592	2051.0079	2051.0034	2	1	K.KYSHVDLVVMVDGFEGEK.G	(Ions score 43)
167 - 184	513.7592	2051.0079	2051.0034	2	1	K.KYSHVDLVVMVDGFEGEK.G	(Ions score 45)
167 - 184	513.7592	2051.0079	2051.0034	2	1	K.KYSHVDLVVMVDGFEGEK.G	(Ions score 22)
167 - 184	684.6766	2051.0079	2051.0034	2	1	K.KYSHVDLVVMVDGFEGEK.G	(Ions score 50)
167 - 184	684.6766	2051.0079	2051.0034	2	1	K.KYSHVDLVVMVDGFEGEK.G	(Ions score 44)

167 - 184	684.6766	2051.0079	2051.0034	2	1	K.KYSHVDLVVMVDGFEGEK.G	(Ions score 41)
167 - 184	513.7593	2051.0079	2051.0034	2	1	K.KYSHVDLVVMVDGFEGEK.G	(Ions score 40)
167 - 184	513.7593	2051.0079	2051.0034	2	1	K.KYSHVDLVVMVDGFEGEK.G	(Ions score 47)
167 - 184	1026.5113	2051.0080	2051.0034	2	1	K.KYSHVDLVVMVDGFEGEK.G	(Ions score 79)
167 - 184	684.6766	2051.0080	2051.0034	2	1	K.KYSHVDLVVMVDGFEGEK.G	(Ions score 54)
167 - 184	513.7593	2051.0080	2051.0034	2	1	K.KYSHVDLVVMVDGFEGEK.G	(Ions score 43)
167 - 184	684.6766	2051.0080	2051.0034	2	1	K.KYSHVDLVVMVDGFEGEK.G	(Ions score 44)
167 - 184	513.7593	2051.0080	2051.0034	2	1	K.KYSHVDLVVMVDGFEGEK.G	(Ions score 32)
167 - 184	684.6766	2051.0080	2051.0034	2	1	K.KYSHVDLVVMVDGFEGEK.G	(Ions score 60)
167 - 184	684.6766	2051.0080	2051.0034	2	1	K.KYSHVDLVVMVDGFEGEK.G	(Ions score 46)
167 - 184	1026.5113	2051.0081	2051.0034	2	1	K.KYSHVDLVVMVDGFEGEK.G	(Ions score 98)
167 - 184	684.6766	2051.0081	2051.0034	2	1	K.KYSHVDLVVMVDGFEGEK.G	(Ions score 45)
167 - 184	513.7593	2051.0081	2051.0034	2	1	K.KYSHVDLVVMVDGFEGEK.G	(Ions score 38)
167 - 184	1026.5113	2051.0081	2051.0034	2	1	K.KYSHVDLVVMVDGFEGEK.G	(Ions score 78)
167 - 184	684.6766	2051.0081	2051.0034	2	1	K.KYSHVDLVVMVDGFEGEK.G	(Ions score 39)
167 - 184	684.6767	2051.0082	2051.0034	2	1	K.KYSHVDLVVMVDGFEGEK.G	(Ions score 25)
167 - 184	684.6767	2051.0082	2051.0034	2	1	K.KYSHVDLVVMVDGFEGEK.G	(Ions score 46)
167 - 184	684.6767	2051.0082	2051.0034	2	1	K.KYSHVDLVVMVDGFEGEK.G	(Ions score 50)
167 - 184	684.6767	2051.0082	2051.0034	2	1	K.KYSHVDLVVMVDGFEGEK.G	(Ions score 56)
167 - 184	513.7593	2051.0082	2051.0034	2	1	K.KYSHVDLVVMVDGFEGEK.G	(Ions score 49)
167 - 184	513.7593	2051.0082	2051.0034	2	1	K.KYSHVDLVVMVDGFEGEK.G	(Ions score 44)
167 - 184	513.7593	2051.0082	2051.0034	2	1	K.KYSHVDLVVMVDGFEGEK.G	(Ions score 38)
167 - 184	513.7593	2051.0082	2051.0034	2	1	K.KYSHVDLVVMVDGFEGEK.G	(Ions score 31)
167 - 184	684.6767	2051.0083	2051.0034	2	1	K.KYSHVDLVVMVDGFEGEK.G	(Ions score 36)
167 - 184	1026.5115	2051.0084	2051.0034	2	1	K.KYSHVDLVVMVDGFEGEK.G	(Ions score 100)
167 - 184	684.6767	2051.0084	2051.0034	2	1	K.KYSHVDLVVMVDGFEGEK.G	(Ions score 49)
167 - 184	684.6767	2051.0084	2051.0034	2	1	K.KYSHVDLVVMVDGFEGEK.G	(Ions score 54)
167 - 184	513.7594	2051.0084	2051.0034	2	1	K.KYSHVDLVVMVDGFEGEK.G	(Ions score 43)
167 - 184	513.7594	2051.0084	2051.0034	2	1	K.KYSHVDLVVMVDGFEGEK.G	(Ions score 48)
167 - 184	684.6767	2051.0084	2051.0034	2	1	K.KYSHVDLVVMVDGFEGEK.G	(Ions score 46)
167 - 184	684.6768	2051.0084	2051.0034	2	1	K.KYSHVDLVVMVDGFEGEK.G	(Ions score 24)
167 - 184	513.7594	2051.0084	2051.0034	2	1	K.KYSHVDLVVMVDGFEGEK.G	(Ions score 39)
167 - 184	513.7594	2051.0084	2051.0034	2	1	K.KYSHVDLVVMVDGFEGEK.G	(Ions score 42)
167 - 184	513.7594	2051.0085	2051.0034	2	1	K.KYSHVDLVVMVDGFEGEK.G	(Ions score 44)
167 - 184	684.6768	2051.0085	2051.0034	2	1	K.KYSHVDLVVMVDGFEGEK.G	(Ions score 48)
167 - 184	513.7594	2051.0085	2051.0034	2	1	K.KYSHVDLVVMVDGFEGEK.G	(Ions score 44)
167 - 184	513.7594	2051.0085	2051.0034	2	1	K.KYSHVDLVVMVDGFEGEK.G	(Ions score 44)
167 - 184	513.7594	2051.0085	2051.0034	2	1	K.KYSHVDLVVMVDGFEGEK.G	(Ions score 34)
167 - 184	513.7594	2051.0085	2051.0034	2	1	K.KYSHVDLVVMVDGFEGEK.G	(Ions score 40)
167 - 184	513.7594	2051.0085	2051.0034	2	1	K.KYSHVDLVVMVDGFEGEK.G	(Ions score 30)
167 - 184	684.6768	2051.0085	2051.0034	2	1	K.KYSHVDLVVMVDGFEGEK.G	(Ions score 44)
167 - 184	684.6768	2051.0085	2051.0034	2	1	K.KYSHVDLVVMVDGFEGEK.G	(Ions score 56)
167 - 184	1026.5115	2051.0085	2051.0034	3	1	K.KYSHVDLVVMVDGFEGEK.G	(Ions score 73)
167 - 184	684.6768	2051.0085	2051.0034	3	1	K.KYSHVDLVVMVDGFEGEK.G	(Ions score 58)
167 - 184	684.6768	2051.0086	2051.0034	3	1	K.KYSHVDLVVMVDGFEGEK.G	(Ions score 37)
167 - 184	513.7594	2051.0086	2051.0034	3	1	K.KYSHVDLVVMVDGFEGEK.G	(Ions score 43)
167 - 184	684.6768	2051.0086	2051.0034	3	1	K.KYSHVDLVVMVDGFEGEK.G	(Ions score 60)
167 - 184	684.6768	2051.0086	2051.0034	3	1	K.KYSHVDLVVMVDGFEGEK.G	(Ions score 58)
167 - 184	513.7594	2051.0086	2051.0034	3	1	K.KYSHVDLVVMVDGFEGEK.G	(Ions score 49)
167 - 184	684.6768	2051.0086	2051.0034	3	1	K.KYSHVDLVVMVDGFEGEK.G	(Ions score 46)
167 - 184	1026.5116	2051.0086	2051.0034	3	1	K.KYSHVDLVVMVDGFEGEK.G	(Ions score 82)
167 - 184	513.7594	2051.0086	2051.0034	3	1	K.KYSHVDLVVMVDGFEGEK.G	(Ions score 34)
167 - 184	513.7594	2051.0086	2051.0034	3	1	K.KYSHVDLVVMVDGFEGEK.G	(Ions score 45)
167 - 184	684.6768	2051.0087	2051.0034	3	1	K.KYSHVDLVVMVDGFEGEK.G	(Ions score 49)
167 - 184	684.6768	2051.0087	2051.0034	3	1	K.KYSHVDLVVMVDGFEGEK.G	(Ions score 37)
167 - 184	513.7594	2051.0087	2051.0034	3	1	K.KYSHVDLVVMVDGFEGEK.G	(Ions score 43)
167 - 184	684.6768	2051.0087	2051.0034	3	1	K.KYSHVDLVVMVDGFEGEK.G	(Ions score 48)

167 - 184	513.7594	2051.0087	2051.0034	3	1	K.KYSHVDLVVMVDGFEGEK.G	(Ions score 45)
167 - 184	684.6768	2051.0087	2051.0034	3	1	K.KYSHVDLVVMVDGFEGEK.G	(Ions score 39)
167 - 184	513.7594	2051.0087	2051.0034	3	1	K.KYSHVDLVVMVDGFEGEK.G	(Ions score 30)
167 - 184	684.6768	2051.0087	2051.0034	3	1	K.KYSHVDLVVMVDGFEGEK.G	(Ions score 66)
167 - 184	684.6768	2051.0087	2051.0034	3	1	K.KYSHVDLVVMVDGFEGEK.G	(Ions score 39)
167 - 184	684.6768	2051.0087	2051.0034	3	1	K.KYSHVDLVVMVDGFEGEK.G	(Ions score 40)
167 - 184	513.7595	2051.0087	2051.0034	3	1	K.KYSHVDLVVMVDGFEGEK.G	(Ions score 47)
167 - 184	684.6769	2051.0087	2051.0034	3	1	K.KYSHVDLVVMVDGFEGEK.G	(Ions score 54)
167 - 184	684.6769	2051.0087	2051.0034	3	1	K.KYSHVDLVVMVDGFEGEK.G	(Ions score 59)
167 - 184	684.6769	2051.0088	2051.0034	3	1	K.KYSHVDLVVMVDGFEGEK.G	(Ions score 44)
167 - 184	513.7595	2051.0088	2051.0034	3	1	K.KYSHVDLVVMVDGFEGEK.G	(Ions score 36)
167 - 184	513.7595	2051.0088	2051.0034	3	1	K.KYSHVDLVVMVDGFEGEK.G	(Ions score 39)
167 - 184	684.6769	2051.0088	2051.0034	3	1	K.KYSHVDLVVMVDGFEGEK.G	(Ions score 47)
167 - 184	684.6769	2051.0088	2051.0034	3	1	K.KYSHVDLVVMVDGFEGEK.G	(Ions score 50)
167 - 184	1026.5117	2051.0089	2051.0034	3	1	K.KYSHVDLVVMVDGFEGEK.G	(Ions score 94)
167 - 184	684.6769	2051.0089	2051.0034	3	1	K.KYSHVDLVVMVDGFEGEK.G	(Ions score 47)
167 - 184	684.6769	2051.0089	2051.0034	3	1	K.KYSHVDLVVMVDGFEGEK.G	(Ions score 49)
167 - 184	513.7595	2051.0089	2051.0034	3	1	K.KYSHVDLVVMVDGFEGEK.G	(Ions score 40)
167 - 184	684.6769	2051.0089	2051.0034	3	1	K.KYSHVDLVVMVDGFEGEK.G	(Ions score 47)
167 - 184	684.6769	2051.0090	2051.0034	3	1	K.KYSHVDLVVMVDGFEGEK.G	(Ions score 59)
167 - 184	684.6769	2051.0090	2051.0034	3	1	K.KYSHVDLVVMVDGFEGEK.G	(Ions score 52)
167 - 184	513.7595	2051.0090	2051.0034	3	1	K.KYSHVDLVVMVDGFEGEK.G	(Ions score 38)
167 - 184	513.7595	2051.0090	2051.0034	3	1	K.KYSHVDLVVMVDGFEGEK.G	(Ions score 36)
167 - 184	513.7595	2051.0091	2051.0034	3	1	K.KYSHVDLVVMVDGFEGEK.G	(Ions score 44)
167 - 184	684.6770	2051.0091	2051.0034	3	1	K.KYSHVDLVVMVDGFEGEK.G	(Ions score 41)
167 - 184	684.6770	2051.0091	2051.0034	3	1	K.KYSHVDLVVMVDGFEGEK.G	(Ions score 58)
167 - 184	513.7596	2051.0091	2051.0034	3	1	K.KYSHVDLVVMVDGFEGEK.G	(Ions score 29)
167 - 184	684.6770	2051.0091	2051.0034	3	1	K.KYSHVDLVVMVDGFEGEK.G	(Ions score 47)
167 - 184	684.6770	2051.0091	2051.0034	3	1	K.KYSHVDLVVMVDGFEGEK.G	(Ions score 56)
167 - 184	513.7596	2051.0092	2051.0034	3	1	K.KYSHVDLVVMVDGFEGEK.G	(Ions score 43)
167 - 184	684.6770	2051.0092	2051.0034	3	1	K.KYSHVDLVVMVDGFEGEK.G	(Ions score 56)
167 - 184	684.6770	2051.0092	2051.0034	3	1	K.KYSHVDLVVMVDGFEGEK.G	(Ions score 34)
167 - 184	513.7596	2051.0092	2051.0034	3	1	K.KYSHVDLVVMVDGFEGEK.G	(Ions score 37)
167 - 184	684.6770	2051.0092	2051.0034	3	1	K.KYSHVDLVVMVDGFEGEK.G	(Ions score 42)
167 - 184	684.6770	2051.0092	2051.0034	3	1	K.KYSHVDLVVMVDGFEGEK.G	(Ions score 59)
167 - 184	513.7596	2051.0092	2051.0034	3	1	K.KYSHVDLVVMVDGFEGEK.G	(Ions score 47)
167 - 184	684.6770	2051.0092	2051.0034	3	1	K.KYSHVDLVVMVDGFEGEK.G	(Ions score 59)
167 - 184	684.6770	2051.0092	2051.0034	3	1	K.KYSHVDLVVMVDGFEGEK.G	(Ions score 49)
167 - 184	684.6770	2051.0093	2051.0034	3	1	K.KYSHVDLVVMVDGFEGEK.G	(Ions score 56)
167 - 184	513.7596	2051.0093	2051.0034	3	1	K.KYSHVDLVVMVDGFEGEK.G	(Ions score 34)
167 - 184	513.7596	2051.0093	2051.0034	3	1	K.KYSHVDLVVMVDGFEGEK.G	(Ions score 45)
167 - 184	513.7596	2051.0093	2051.0034	3	1	K.KYSHVDLVVMVDGFEGEK.G	(Ions score 29)
167 - 184	513.7596	2051.0094	2051.0034	3	1	K.KYSHVDLVVMVDGFEGEK.G	(Ions score 36)
167 - 184	684.6771	2051.0094	2051.0034	3	1	K.KYSHVDLVVMVDGFEGEK.G	(Ions score 57)
167 - 184	684.6771	2051.0094	2051.0034	3	1	K.KYSHVDLVVMVDGFEGEK.G	(Ions score 54)
167 - 184	684.6771	2051.0094	2051.0034	3	1	K.KYSHVDLVVMVDGFEGEK.G	(Ions score 46)
167 - 184	684.6771	2051.0094	2051.0034	3	1	K.KYSHVDLVVMVDGFEGEK.G	(Ions score 41)
167 - 184	684.6771	2051.0094	2051.0034	3	1	K.KYSHVDLVVMVDGFEGEK.G	(Ions score 43)
167 - 184	513.7596	2051.0094	2051.0034	3	1	K.KYSHVDLVVMVDGFEGEK.G	(Ions score 45)
167 - 184	513.7596	2051.0094	2051.0034	3	1	K.KYSHVDLVVMVDGFEGEK.G	(Ions score 54)
167 - 184	684.6771	2051.0095	2051.0034	3	1	K.KYSHVDLVVMVDGFEGEK.G	(Ions score 46)
167 - 184	513.7596	2051.0095	2051.0034	3	1	K.KYSHVDLVVMVDGFEGEK.G	(Ions score 44)
167 - 184	513.7596	2051.0095	2051.0034	3	1	K.KYSHVDLVVMVDGFEGEK.G	(Ions score 43)
167 - 184	513.7596	2051.0095	2051.0034	3	1	K.KYSHVDLVVMVDGFEGEK.G	(Ions score 44)
167 - 184	513.7596	2051.0095	2051.0034	3	1	K.KYSHVDLVVMVDGFEGEK.G	(Ions score 54)
167 - 184	513.7597	2051.0095	2051.0034	3	1	K.KYSHVDLVVMVDGFEGEK.G	(Ions score 39)
167 - 184	513.7597	2051.0095	2051.0034	3	1	K.KYSHVDLVVMVDGFEGEK.G	(Ions score 34)

167 - 184	513.7597	2051.0096	2051.0034	3	1	K.KYSHVDLVVMVDGFEGEK.G	(Ions score 30)
167 - 184	513.7597	2051.0096	2051.0034	3	1	K.KYSHVDLVVMVDGFEGEK.G	(Ions score 44)
167 - 184	684.6771	2051.0096	2051.0034	3	1	K.KYSHVDLVVMVDGFEGEK.G	(Ions score 60)
167 - 184	684.6771	2051.0096	2051.0034	3	1	K.KYSHVDLVVMVDGFEGEK.G	(Ions score 50)
167 - 184	513.7597	2051.0096	2051.0034	3	1	K.KYSHVDLVVMVDGFEGEK.G	(Ions score 40)
167 - 184	684.6771	2051.0096	2051.0034	3	1	K.KYSHVDLVVMVDGFEGEK.G	(Ions score 54)
167 - 184	513.7597	2051.0097	2051.0034	3	1	K.KYSHVDLVVMVDGFEGEK.G	(Ions score 51)
167 - 184	513.7597	2051.0097	2051.0034	3	1	K.KYSHVDLVVMVDGFEGEK.G	(Ions score 37)
167 - 184	684.6772	2051.0097	2051.0034	3	1	K.KYSHVDLVVMVDGFEGEK.G	(Ions score 58)
167 - 184	513.7597	2051.0097	2051.0034	3	1	K.KYSHVDLVVMVDGFEGEK.G	(Ions score 47)
167 - 184	513.7597	2051.0097	2051.0034	3	1	K.KYSHVDLVVMVDGFEGEK.G	(Ions score 44)
167 - 184	513.7597	2051.0097	2051.0034	3	1	K.KYSHVDLVVMVDGFEGEK.G	(Ions score 43)
167 - 184	1026.5121	2051.0097	2051.0034	3	1	K.KYSHVDLVVMVDGFEGEK.G	(Ions score 102)
167 - 184	513.7597	2051.0097	2051.0034	3	1	K.KYSHVDLVVMVDGFEGEK.G	(Ions score 31)
167 - 184	513.7597	2051.0097	2051.0034	3	1	K.KYSHVDLVVMVDGFEGEK.G	(Ions score 39)
167 - 184	684.6772	2051.0097	2051.0034	3	1	K.KYSHVDLVVMVDGFEGEK.G	(Ions score 47)
167 - 184	684.6772	2051.0098	2051.0034	3	1	K.KYSHVDLVVMVDGFEGEK.G	(Ions score 49)
167 - 184	684.6772	2051.0098	2051.0034	3	1	K.KYSHVDLVVMVDGFEGEK.G	(Ions score 23)
167 - 184	684.6772	2051.0098	2051.0034	3	1	K.KYSHVDLVVMVDGFEGEK.G	(Ions score 54)
167 - 184	513.7597	2051.0098	2051.0034	3	1	K.KYSHVDLVVMVDGFEGEK.G	(Ions score 39)
167 - 184	1026.5122	2051.0098	2051.0034	3	1	K.KYSHVDLVVMVDGFEGEK.G	(Ions score 99)
167 - 184	684.6772	2051.0098	2051.0034	3	1	K.KYSHVDLVVMVDGFEGEK.G	(Ions score 39)
167 - 184	513.7597	2051.0098	2051.0034	3	1	K.KYSHVDLVVMVDGFEGEK.G	(Ions score 41)
167 - 184	513.7597	2051.0098	2051.0034	3	1	K.KYSHVDLVVMVDGFEGEK.G	(Ions score 44)
167 - 184	684.6772	2051.0099	2051.0034	3	1	K.KYSHVDLVVMVDGFEGEK.G	(Ions score 51)
167 - 184	513.7598	2051.0099	2051.0034	3	1	K.KYSHVDLVVMVDGFEGEK.G	(Ions score 41)
167 - 184	513.7598	2051.0099	2051.0034	3	1	K.KYSHVDLVVMVDGFEGEK.G	(Ions score 27)
167 - 184	513.7598	2051.0099	2051.0034	3	1	K.KYSHVDLVVMVDGFEGEK.G	(Ions score 35)
167 - 184	684.6773	2051.0100	2051.0034	3	1	K.KYSHVDLVVMVDGFEGEK.G	(Ions score 33)
167 - 184	513.7598	2051.0100	2051.0034	3	1	K.KYSHVDLVVMVDGFEGEK.G	(Ions score 45)
167 - 184	513.7598	2051.0100	2051.0034	3	1	K.KYSHVDLVVMVDGFEGEK.G	(Ions score 39)
167 - 184	513.7598	2051.0100	2051.0034	3	1	K.KYSHVDLVVMVDGFEGEK.G	(Ions score 49)
167 - 184	684.6773	2051.0100	2051.0034	3	1	K.KYSHVDLVVMVDGFEGEK.G	(Ions score 44)
167 - 184	513.7598	2051.0100	2051.0034	3	1	K.KYSHVDLVVMVDGFEGEK.G	(Ions score 37)
167 - 184	513.7598	2051.0100	2051.0034	3	1	K.KYSHVDLVVMVDGFEGEK.G	(Ions score 44)
167 - 184	513.7598	2051.0100	2051.0034	3	1	K.KYSHVDLVVMVDGFEGEK.G	(Ions score 46)
167 - 184	1026.5123	2051.0101	2051.0034	3	1	K.KYSHVDLVVMVDGFEGEK.G	(Ions score 104)
167 - 184	513.7598	2051.0101	2051.0034	3	1	K.KYSHVDLVVMVDGFEGEK.G	(Ions score 44)
167 - 184	684.6773	2051.0101	2051.0034	3	1	K.KYSHVDLVVMVDGFEGEK.G	(Ions score 46)
167 - 184	684.6773	2051.0101	2051.0034	3	1	K.KYSHVDLVVMVDGFEGEK.G	(Ions score 43)
167 - 184	684.6773	2051.0101	2051.0034	3	1	K.KYSHVDLVVMVDGFEGEK.G	(Ions score 44)
167 - 184	684.6773	2051.0101	2051.0034	3	1	K.KYSHVDLVVMVDGFEGEK.G	(Ions score 51)
167 - 184	513.7598	2051.0101	2051.0034	3	1	K.KYSHVDLVVMVDGFEGEK.G	(Ions score 49)
167 - 184	684.6773	2051.0101	2051.0034	3	1	K.KYSHVDLVVMVDGFEGEK.G	(Ions score 53)
167 - 184	513.7598	2051.0102	2051.0034	3	1	K.KYSHVDLVVMVDGFEGEK.G	(Ions score 33)
167 - 184	684.6773	2051.0102	2051.0034	3	1	K.KYSHVDLVVMVDGFEGEK.G	(Ions score 47)
167 - 184	513.7598	2051.0102	2051.0034	3	1	K.KYSHVDLVVMVDGFEGEK.G	(Ions score 44)
167 - 184	513.7598	2051.0102	2051.0034	3	1	K.KYSHVDLVVMVDGFEGEK.G	(Ions score 43)
167 - 184	513.7598	2051.0102	2051.0034	3	1	K.KYSHVDLVVMVDGFEGEK.G	(Ions score 46)
167 - 184	513.7598	2051.0103	2051.0034	3	1	K.KYSHVDLVVMVDGFEGEK.G	(Ions score 31)
167 - 184	1026.5124	2051.0103	2051.0034	3	1	K.KYSHVDLVVMVDGFEGEK.G	(Ions score 77)
167 - 184	513.7598	2051.0103	2051.0034	3	1	K.KYSHVDLVVMVDGFEGEK.G	(Ions score 49)
167 - 184	513.7599	2051.0103	2051.0034	3	1	K.KYSHVDLVVMVDGFEGEK.G	(Ions score 47)
167 - 184	684.6774	2051.0103	2051.0034	3	1	K.KYSHVDLVVMVDGFEGEK.G	(Ions score 49)
167 - 184	513.7599	2051.0104	2051.0034	3	1	K.KYSHVDLVVMVDGFEGEK.G	(Ions score 41)
167 - 184	684.6774	2051.0104	2051.0034	3	1	K.KYSHVDLVVMVDGFEGEK.G	(Ions score 41)
167 - 184	513.7599	2051.0104	2051.0034	3	1	K.KYSHVDLVVMVDGFEGEK.G	(Ions score 46)

167 - 184	513.7599	2051.0104	2051.0034	3	1	K.KYSHVDLVVMVDGFEGEK.G	(Ions score 36)
167 - 184	513.7599	2051.0104	2051.0034	3	1	K.KYSHVDLVVMVDGFEGEK.G	(Ions score 32)
167 - 184	513.7599	2051.0105	2051.0034	3	1	K.KYSHVDLVVMVDGFEGEK.G	(Ions score 51)
167 - 184	513.7599	2051.0105	2051.0034	3	1	K.KYSHVDLVVMVDGFEGEK.G	(Ions score 52)
167 - 184	684.6774	2051.0105	2051.0034	3	1	K.KYSHVDLVVMVDGFEGEK.G	(Ions score 48)
167 - 184	684.6774	2051.0105	2051.0034	3	1	K.KYSHVDLVVMVDGFEGEK.G	(Ions score 58)
167 - 184	513.7599	2051.0105	2051.0034	3	1	K.KYSHVDLVVMVDGFEGEK.G	(Ions score 41)
167 - 184	684.6774	2051.0105	2051.0034	3	1	K.KYSHVDLVVMVDGFEGEK.G	(Ions score 44)
167 - 184	513.7599	2051.0105	2051.0034	3	1	K.KYSHVDLVVMVDGFEGEK.G	(Ions score 53)
167 - 184	513.7599	2051.0105	2051.0034	3	1	K.KYSHVDLVVMVDGFEGEK.G	(Ions score 40)
167 - 184	513.7599	2051.0106	2051.0034	3	1	K.KYSHVDLVVMVDGFEGEK.G	(Ions score 44)
167 - 184	513.7599	2051.0106	2051.0034	3	1	K.KYSHVDLVVMVDGFEGEK.G	(Ions score 43)
167 - 184	513.7599	2051.0106	2051.0034	4	1	K.KYSHVDLVVMVDGFEGEK.G	(Ions score 32)
167 - 184	513.7599	2051.0106	2051.0034	4	1	K.KYSHVDLVVMVDGFEGEK.G	(Ions score 40)
167 - 184	684.6775	2051.0106	2051.0034	4	1	K.KYSHVDLVVMVDGFEGEK.G	(Ions score 47)
167 - 184	513.7599	2051.0106	2051.0034	4	1	K.KYSHVDLVVMVDGFEGEK.G	(Ions score 39)
167 - 184	513.7599	2051.0106	2051.0034	4	1	K.KYSHVDLVVMVDGFEGEK.G	(Ions score 45)
167 - 184	513.7599	2051.0107	2051.0034	4	1	K.KYSHVDLVVMVDGFEGEK.G	(Ions score 39)
167 - 184	513.7599	2051.0107	2051.0034	4	1	K.KYSHVDLVVMVDGFEGEK.G	(Ions score 36)
167 - 184	513.7600	2051.0107	2051.0034	4	1	K.KYSHVDLVVMVDGFEGEK.G	(Ions score 43)
167 - 184	513.7600	2051.0107	2051.0034	4	1	K.KYSHVDLVVMVDGFEGEK.G	(Ions score 47)
167 - 184	684.6775	2051.0108	2051.0034	4	1	K.KYSHVDLVVMVDGFEGEK.G	(Ions score 70)
167 - 184	513.7600	2051.0108	2051.0034	4	1	K.KYSHVDLVVMVDGFEGEK.G	(Ions score 32)
167 - 184	684.6775	2051.0108	2051.0034	4	1	K.KYSHVDLVVMVDGFEGEK.G	(Ions score 46)
167 - 184	684.6775	2051.0108	2051.0034	4	1	K.KYSHVDLVVMVDGFEGEK.G	(Ions score 43)
167 - 184	513.7600	2051.0108	2051.0034	4	1	K.KYSHVDLVVMVDGFEGEK.G	(Ions score 39)
167 - 184	513.7600	2051.0108	2051.0034	4	1	K.KYSHVDLVVMVDGFEGEK.G	(Ions score 31)
167 - 184	513.7600	2051.0109	2051.0034	4	1	K.KYSHVDLVVMVDGFEGEK.G	(Ions score 33)
167 - 184	513.7600	2051.0109	2051.0034	4	1	K.KYSHVDLVVMVDGFEGEK.G	(Ions score 44)
167 - 184	513.7600	2051.0109	2051.0034	4	1	K.KYSHVDLVVMVDGFEGEK.G	(Ions score 37)
167 - 184	513.7600	2051.0109	2051.0034	4	1	K.KYSHVDLVVMVDGFEGEK.G	(Ions score 48)
167 - 184	513.7600	2051.0109	2051.0034	4	1	K.KYSHVDLVVMVDGFEGEK.G	(Ions score 33)
167 - 184	684.6776	2051.0109	2051.0034	4	1	K.KYSHVDLVVMVDGFEGEK.G	(Ions score 58)
167 - 184	684.6776	2051.0109	2051.0034	4	1	K.KYSHVDLVVMVDGFEGEK.G	(Ions score 42)
167 - 184	513.7600	2051.0109	2051.0034	4	1	K.KYSHVDLVVMVDGFEGEK.G	(Ions score 44)
167 - 184	684.6776	2051.0109	2051.0034	4	1	K.KYSHVDLVVMVDGFEGEK.G	(Ions score 58)
167 - 184	513.7600	2051.0110	2051.0034	4	1	K.KYSHVDLVVMVDGFEGEK.G	(Ions score 50)
167 - 184	684.6776	2051.0110	2051.0034	4	1	K.KYSHVDLVVMVDGFEGEK.G	(Ions score 52)
167 - 184	513.7600	2051.0110	2051.0034	4	1	K.KYSHVDLVVMVDGFEGEK.G	(Ions score 38)
167 - 184	684.6776	2051.0110	2051.0034	4	1	K.KYSHVDLVVMVDGFEGEK.G	(Ions score 44)
167 - 184	513.7600	2051.0110	2051.0034	4	1	K.KYSHVDLVVMVDGFEGEK.G	(Ions score 51)
167 - 184	513.7600	2051.0111	2051.0034	4	1	K.KYSHVDLVVMVDGFEGEK.G	(Ions score 49)
167 - 184	684.6776	2051.0111	2051.0034	4	1	K.KYSHVDLVVMVDGFEGEK.G	(Ions score 23)
167 - 184	513.7600	2051.0111	2051.0034	4	1	K.KYSHVDLVVMVDGFEGEK.G	(Ions score 46)
167 - 184	684.6776	2051.0111	2051.0034	4	1	K.KYSHVDLVVMVDGFEGEK.G	(Ions score 53)
167 - 184	513.7600	2051.0111	2051.0034	4	1	K.KYSHVDLVVMVDGFEGEK.G	(Ions score 45)
167 - 184	513.7600	2051.0111	2051.0034	4	1	K.KYSHVDLVVMVDGFEGEK.G	(Ions score 52)
167 - 184	684.6776	2051.0111	2051.0034	4	1	K.KYSHVDLVVMVDGFEGEK.G	(Ions score 58)
167 - 184	513.7601	2051.0111	2051.0034	4	1	K.KYSHVDLVVMVDGFEGEK.G	(Ions score 36)
167 - 184	513.7601	2051.0111	2051.0034	4	1	K.KYSHVDLVVMVDGFEGEK.G	(Ions score 36)
167 - 184	513.7601	2051.0111	2051.0034	4	1	K.KYSHVDLVVMVDGFEGEK.G	(Ions score 49)
167 - 184	684.6777	2051.0112	2051.0034	4	1	K.KYSHVDLVVMVDGFEGEK.G	(Ions score 64)
167 - 184	513.7601	2051.0112	2051.0034	4	1	K.KYSHVDLVVMVDGFEGEK.G	(Ions score 36)
167 - 184	513.7601	2051.0112	2051.0034	4	1	K.KYSHVDLVVMVDGFEGEK.G	(Ions score 37)
167 - 184	513.7601	2051.0112	2051.0034	4	1	K.KYSHVDLVVMVDGFEGEK.G	(Ions score 40)
167 - 184	684.6777	2051.0113	2051.0034	4	1	K.KYSHVDLVVMVDGFEGEK.G	(Ions score 47)
167 - 184	513.7601	2051.0113	2051.0034	4	1	K.KYSHVDLVVMVDGFEGEK.G	(Ions score 43)

167 - 184	684.6777	2051.0114	2051.0034	4	1	K.KYSHVDLVVMVDGFEGEK.G	(Ions score 66)
167 - 184	513.7601	2051.0114	2051.0034	4	1	K.KYSHVDLVVMVDGFEGEK.G	(Ions score 32)
167 - 184	684.6777	2051.0114	2051.0034	4	1	K.KYSHVDLVVMVDGFEGEK.G	(Ions score 49)
167 - 184	513.7601	2051.0114	2051.0034	4	1	K.KYSHVDLVVMVDGFEGEK.G	(Ions score 33)
167 - 184	684.6777	2051.0114	2051.0034	4	1	K.KYSHVDLVVMVDGFEGEK.G	(Ions score 46)
167 - 184	513.7601	2051.0114	2051.0034	4	1	K.KYSHVDLVVMVDGFEGEK.G	(Ions score 36)
167 - 184	684.6778	2051.0114	2051.0034	4	1	K.KYSHVDLVVMVDGFEGEK.G	(Ions score 49)
167 - 184	684.6778	2051.0114	2051.0034	4	1	K.KYSHVDLVVMVDGFEGEK.G	(Ions score 49)
167 - 184	513.7601	2051.0114	2051.0034	4	1	K.KYSHVDLVVMVDGFEGEK.G	(Ions score 33)
167 - 184	513.7601	2051.0114	2051.0034	4	1	K.KYSHVDLVVMVDGFEGEK.G	(Ions score 35)
167 - 184	513.7601	2051.0114	2051.0034	4	1	K.KYSHVDLVVMVDGFEGEK.G	(Ions score 50)
167 - 184	684.6778	2051.0115	2051.0034	4	1	K.KYSHVDLVVMVDGFEGEK.G	(Ions score 47)
167 - 184	684.6778	2051.0115	2051.0034	4	1	K.KYSHVDLVVMVDGFEGEK.G	(Ions score 37)
167 - 184	513.7601	2051.0115	2051.0034	4	1	K.KYSHVDLVVMVDGFEGEK.G	(Ions score 43)
167 - 184	684.6778	2051.0115	2051.0034	4	1	K.KYSHVDLVVMVDGFEGEK.G	(Ions score 56)
167 - 184	513.7602	2051.0116	2051.0034	4	1	K.KYSHVDLVVMVDGFEGEK.G	(Ions score 44)
167 - 184	513.7602	2051.0116	2051.0034	4	1	K.KYSHVDLVVMVDGFEGEK.G	(Ions score 37)
167 - 184	513.7602	2051.0116	2051.0034	4	1	K.KYSHVDLVVMVDGFEGEK.G	(Ions score 34)
167 - 184	513.7602	2051.0116	2051.0034	4	1	K.KYSHVDLVVMVDGFEGEK.G	(Ions score 44)
167 - 184	513.7602	2051.0116	2051.0034	4	1	K.KYSHVDLVVMVDGFEGEK.G	(Ions score 42)
167 - 184	513.7602	2051.0116	2051.0034	4	1	K.KYSHVDLVVMVDGFEGEK.G	(Ions score 48)
167 - 184	513.7602	2051.0116	2051.0034	4	1	K.KYSHVDLVVMVDGFEGEK.G	(Ions score 45)
167 - 184	513.7602	2051.0116	2051.0034	4	1	K.KYSHVDLVVMVDGFEGEK.G	(Ions score 42)
167 - 184	684.6778	2051.0116	2051.0034	4	1	K.KYSHVDLVVMVDGFEGEK.G	(Ions score 47)
167 - 184	513.7602	2051.0116	2051.0034	4	1	K.KYSHVDLVVMVDGFEGEK.G	(Ions score 43)
167 - 184	513.7602	2051.0117	2051.0034	4	1	K.KYSHVDLVVMVDGFEGEK.G	(Ions score 47)
167 - 184	684.6778	2051.0117	2051.0034	4	1	K.KYSHVDLVVMVDGFEGEK.G	(Ions score 49)
167 - 184	513.7602	2051.0117	2051.0034	4	1	K.KYSHVDLVVMVDGFEGEK.G	(Ions score 37)
167 - 184	513.7602	2051.0118	2051.0034	4	1	K.KYSHVDLVVMVDGFEGEK.G	(Ions score 39)
167 - 184	513.7602	2051.0118	2051.0034	4	1	K.KYSHVDLVVMVDGFEGEK.G	(Ions score 43)
167 - 184	684.6779	2051.0118	2051.0034	4	1	K.KYSHVDLVVMVDGFEGEK.G	(Ions score 58)
167 - 184	684.6779	2051.0118	2051.0034	4	1	K.KYSHVDLVVMVDGFEGEK.G	(Ions score 40)
167 - 184	513.7602	2051.0118	2051.0034	4	1	K.KYSHVDLVVMVDGFEGEK.G	(Ions score 37)
167 - 184	684.6779	2051.0118	2051.0034	4	1	K.KYSHVDLVVMVDGFEGEK.G	(Ions score 53)
167 - 184	684.6779	2051.0119	2051.0034	4	1	K.KYSHVDLVVMVDGFEGEK.G	(Ions score 59)
167 - 184	513.7602	2051.0119	2051.0034	4	1	K.KYSHVDLVVMVDGFEGEK.G	(Ions score 34)
167 - 184	684.6779	2051.0119	2051.0034	4	1	K.KYSHVDLVVMVDGFEGEK.G	(Ions score 49)
167 - 184	513.7602	2051.0119	2051.0034	4	1	K.KYSHVDLVVMVDGFEGEK.G	(Ions score 42)
167 - 184	684.6779	2051.0119	2051.0034	4	1	K.KYSHVDLVVMVDGFEGEK.G	(Ions score 33)
167 - 184	513.7603	2051.0119	2051.0034	4	1	K.KYSHVDLVVMVDGFEGEK.G	(Ions score 46)
167 - 184	684.6779	2051.0119	2051.0034	4	1	K.KYSHVDLVVMVDGFEGEK.G	(Ions score 58)
167 - 184	684.6779	2051.0119	2051.0034	4	1	K.KYSHVDLVVMVDGFEGEK.G	(Ions score 46)
167 - 184	513.7603	2051.0119	2051.0034	4	1	K.KYSHVDLVVMVDGFEGEK.G	(Ions score 40)
167 - 184	684.6779	2051.0120	2051.0034	4	1	K.KYSHVDLVVMVDGFEGEK.G	(Ions score 54)
167 - 184	684.6779	2051.0120	2051.0034	4	1	K.KYSHVDLVVMVDGFEGEK.G	(Ions score 47)
167 - 184	684.6779	2051.0120	2051.0034	4	1	K.KYSHVDLVVMVDGFEGEK.G	(Ions score 70)
167 - 184	684.6779	2051.0120	2051.0034	4	1	K.KYSHVDLVVMVDGFEGEK.G	(Ions score 47)
167 - 184	513.7603	2051.0120	2051.0034	4	1	K.KYSHVDLVVMVDGFEGEK.G	(Ions score 46)
167 - 184	513.7603	2051.0120	2051.0034	4	1	K.KYSHVDLVVMVDGFEGEK.G	(Ions score 41)
167 - 184	684.6779	2051.0120	2051.0034	4	1	K.KYSHVDLVVMVDGFEGEK.G	(Ions score 69)
167 - 184	513.7603	2051.0120	2051.0034	4	1	K.KYSHVDLVVMVDGFEGEK.G	(Ions score 34)
167 - 184	684.6780	2051.0120	2051.0034	4	1	K.KYSHVDLVVMVDGFEGEK.G	(Ions score 54)
167 - 184	684.6780	2051.0121	2051.0034	4	1	K.KYSHVDLVVMVDGFEGEK.G	(Ions score 58)
167 - 184	684.6780	2051.0121	2051.0034	4	1	K.KYSHVDLVVMVDGFEGEK.G	(Ions score 59)
167 - 184	684.6780	2051.0121	2051.0034	4	1	K.KYSHVDLVVMVDGFEGEK.G	(Ions score 43)
167 - 184	684.6780	2051.0122	2051.0034	4	1	K.KYSHVDLVVMVDGFEGEK.G	(Ions score 52)
167 - 184	513.7603	2051.0122	2051.0034	4	1	K.KYSHVDLVVMVDGFEGEK.G	(Ions score 44)

167 - 184	513.7603	2051.0122	2051.0034	4	1	K.KYSHVDLVVMVDGFEGEK.G		(Ions score 36)
167 - 184	513.7603	2051.0122	2051.0034	4	1	K.KYSHVDLVVMVDGFEGEK.G		(Ions score 47)
167 - 184	684.6780	2051.0122	2051.0034	4	1	K.KYSHVDLVVMVDGFEGEK.G		(Ions score 45)
167 - 184	513.7603	2051.0122	2051.0034	4	1	K.KYSHVDLVVMVDGFEGEK.G		(Ions score 38)
167 - 184	513.7603	2051.0122	2051.0034	4	1	K.KYSHVDLVVMVDGFEGEK.G		(Ions score 45)
167 - 184	513.7603	2051.0122	2051.0034	4	1	K.KYSHVDLVVMVDGFEGEK.G		(Ions score 34)
167 - 184	513.7603	2051.0122	2051.0034	4	1	K.KYSHVDLVVMVDGFEGEK.G		(Ions score 38)
167 - 184	684.6780	2051.0122	2051.0034	4	1	K.KYSHVDLVVMVDGFEGEK.G		(Ions score 49)
167 - 184	684.6780	2051.0123	2051.0034	4	1	K.KYSHVDLVVMVDGFEGEK.G		(Ions score 31)
167 - 184	513.7604	2051.0123	2051.0034	4	1	K.KYSHVDLVVMVDGFEGEK.G		(Ions score 45)
167 - 184	684.6781	2051.0123	2051.0034	4	1	K.KYSHVDLVVMVDGFEGEK.G		(Ions score 42)
167 - 184	684.6781	2051.0123	2051.0034	4	1	K.KYSHVDLVVMVDGFEGEK.G		(Ions score 49)
167 - 184	684.6781	2051.0124	2051.0034	4	1	K.KYSHVDLVVMVDGFEGEK.G		(Ions score 58)
167 - 184	1026.5135	2051.0124	2051.0034	4	1	K.KYSHVDLVVMVDGFEGEK.G		(Ions score 81)
167 - 184	513.7604	2051.0125	2051.0034	4	1	K.KYSHVDLVVMVDGFEGEK.G		(Ions score 41)
167 - 184	684.6781	2051.0125	2051.0034	4	1	K.KYSHVDLVVMVDGFEGEK.G		(Ions score 43)
167 - 184	684.6781	2051.0125	2051.0034	4	1	K.KYSHVDLVVMVDGFEGEK.G		(Ions score 31)
167 - 184	513.7604	2051.0125	2051.0034	4	1	K.KYSHVDLVVMVDGFEGEK.G		(Ions score 46)
167 - 184	513.7604	2051.0126	2051.0034	4	1	K.KYSHVDLVVMVDGFEGEK.G		(Ions score 43)
167 - 184	513.7604	2051.0126	2051.0034	5	1	K.KYSHVDLVVMVDGFEGEK.G		(Ions score 35)
167 - 184	513.7604	2051.0126	2051.0034	5	1	K.KYSHVDLVVMVDGFEGEK.G		(Ions score 42)
167 - 184	513.7604	2051.0126	2051.0034	5	1	K.KYSHVDLVVMVDGFEGEK.G		(Ions score 35)
167 - 184	513.7604	2051.0127	2051.0034	5	1	K.KYSHVDLVVMVDGFEGEK.G		(Ions score 47)
167 - 184	684.6782	2051.0127	2051.0034	5	1	K.KYSHVDLVVMVDGFEGEK.G		(Ions score 49)
167 - 184	684.6782	2051.0127	2051.0034	5	1	K.KYSHVDLVVMVDGFEGEK.G		(Ions score 56)
167 - 184	684.6782	2051.0127	2051.0034	5	1	K.KYSHVDLVVMVDGFEGEK.G		(Ions score 47)
167 - 184	684.6782	2051.0127	2051.0034	5	1	K.KYSHVDLVVMVDGFEGEK.G		(Ions score 56)
167 - 184	684.6782	2051.0128	2051.0034	5	1	K.KYSHVDLVVMVDGFEGEK.G		(Ions score 54)
167 - 184	684.6782	2051.0129	2051.0034	5	1	K.KYSHVDLVVMVDGFEGEK.G		(Ions score 58)
167 - 184	513.7605	2051.0129	2051.0034	5	1	K.KYSHVDLVVMVDGFEGEK.G		(Ions score 37)
167 - 184	513.7605	2051.0129	2051.0034	5	1	K.KYSHVDLVVMVDGFEGEK.G		(Ions score 47)
167 - 184	513.7605	2051.0130	2051.0034	5	1	K.KYSHVDLVVMVDGFEGEK.G		(Ions score 38)
167 - 184	684.6783	2051.0130	2051.0034	5	1	K.KYSHVDLVVMVDGFEGEK.G		(Ions score 49)
167 - 184	513.7605	2051.0130	2051.0034	5	1	K.KYSHVDLVVMVDGFEGEK.G		(Ions score 33)
167 - 184	513.7606	2051.0132	2051.0034	5	1	K.KYSHVDLVVMVDGFEGEK.G		(Ions score 34)
167 - 184	1026.5139	2051.0132	2051.0034	5	1	K.KYSHVDLVVMVDGFEGEK.G		(Ions score 78)
167 - 184	684.6784	2051.0132	2051.0034	5	1	K.KYSHVDLVVMVDGFEGEK.G		(Ions score 35)
167 - 184	513.7606	2051.0133	2051.0034	5	1	K.KYSHVDLVVMVDGFEGEK.G		(Ions score 44)
167 - 184	513.7606	2051.0134	2051.0034	5	1	K.KYSHVDLVVMVDGFEGEK.G		(Ions score 36)
167 - 184	684.6784	2051.0134	2051.0034	5	1	K.KYSHVDLVVMVDGFEGEK.G		(Ions score 47)
167 - 184	684.6784	2051.0134	2051.0034	5	1	K.KYSHVDLVVMVDGFEGEK.G		(Ions score 38)
167 - 184	684.6784	2051.0135	2051.0034	5	1	K.KYSHVDLVVMVDGFEGEK.G		(Ions score 49)
167 - 184	684.6784	2051.0135	2051.0034	5	1	K.KYSHVDLVVMVDGFEGEK.G		(Ions score 45)
167 - 184	513.7607	2051.0135	2051.0034	5	1	K.KYSHVDLVVMVDGFEGEK.G		(Ions score 38)
167 - 184	513.7607	2051.0136	2051.0034	5	1	K.KYSHVDLVVMVDGFEGEK.G		(Ions score 36)
167 - 184	684.6785	2051.0136	2051.0034	5	1	K.KYSHVDLVVMVDGFEGEK.G		(Ions score 46)
167 - 184	513.7607	2051.0136	2051.0034	5	1	K.KYSHVDLVVMVDGFEGEK.G		(Ions score 39)
167 - 184	690.0054	2066.9945	2066.9983	-2	1	K.KYSHVDLVVMVDGFEGEK.G	Oxidation (M)	(Ions score 42)
167 - 184	690.0060	2066.9961	2066.9983	-1	1	K.KYSHVDLVVMVDGFEGEK.G	Oxidation (M)	(Ions score 25)
167 - 184	517.7563	2066.9961	2066.9983	-1	1	K.KYSHVDLVVMVDGFEGEK.G	Oxidation (M)	(Ions score 46)
167 - 184	517.7565	2066.9968	2066.9983	-1	1	K.KYSHVDLVVMVDGFEGEK.G	Oxidation (M)	(Ions score 22)
167 - 184	517.7565	2066.9969	2066.9983	-1	1	K.KYSHVDLVVMVDGFEGEK.G	Oxidation (M)	(Ions score 30)
167 - 184	690.0063	2066.9970	2066.9983	-1	1	K.KYSHVDLVVMVDGFEGEK.G	Oxidation (M)	(Ions score 30)
167 - 184	690.0063	2066.9971	2066.9983	-1	1	K.KYSHVDLVVMVDGFEGEK.G	Oxidation (M)	(Ions score 44)
167 - 184	517.7567	2066.9978	2066.9983	-0	1	K.KYSHVDLVVMVDGFEGEK.G	Oxidation (M)	(Ions score 29)
167 - 184	690.0065	2066.9978	2066.9983	-0	1	K.KYSHVDLVVMVDGFEGEK.G	Oxidation (M)	(Ions score 26)
167 - 184	690.0066	2066.9981	2066.9983	-0	1	K.KYSHVDLVVMVDGFEGEK.G	Oxidation (M)	(Ions score 40)

167 - 184	690.0067	2066.9984	2066.9983	0	1	K.KYSHVDLVVMVDGFEGEK.G	Oxidation (M)	(Ions score 32)
167 - 184	690.0068	2066.9985	2066.9983	0	1	K.KYSHVDLVVMVDGFEGEK.G	Oxidation (M)	(Ions score 44)
167 - 184	690.0068	2066.9987	2066.9983	0	1	K.KYSHVDLVVMVDGFEGEK.G	Oxidation (M)	(Ions score 29)
167 - 184	517.7570	2066.9987	2066.9983	0	1	K.KYSHVDLVVMVDGFEGEK.G	Oxidation (M)	(Ions score 36)
167 - 184	517.7570	2066.9987	2066.9983	0	1	K.KYSHVDLVVMVDGFEGEK.G	Oxidation (M)	(Ions score 38)
167 - 184	690.0069	2066.9989	2066.9983	0	1	K.KYSHVDLVVMVDGFEGEK.G	Oxidation (M)	(Ions score 52)
167 - 184	690.0069	2066.9990	2066.9983	0	1	K.KYSHVDLVVMVDGFEGEK.G	Oxidation (M)	(Ions score 67)
167 - 184	690.0070	2066.9992	2066.9983	0	1	K.KYSHVDLVVMVDGFEGEK.G	Oxidation (M)	(Ions score 25)
167 - 184	690.0070	2066.9992	2066.9983	0	1	K.KYSHVDLVVMVDGFEGEK.G	Oxidation (M)	(Ions score 30)
167 - 184	690.0071	2066.9994	2066.9983	1	1	K.KYSHVDLVVMVDGFEGEK.G	Oxidation (M)	(Ions score 66)
167 - 184	517.7571	2066.9995	2066.9983	1	1	K.KYSHVDLVVMVDGFEGEK.G	Oxidation (M)	(Ions score 32)
167 - 184	517.7572	2066.9995	2066.9983	1	1	K.KYSHVDLVVMVDGFEGEK.G	Oxidation (M)	(Ions score 35)
167 - 184	517.7572	2066.9996	2066.9983	1	1	K.KYSHVDLVVMVDGFEGEK.G	Oxidation (M)	(Ions score 32)
167 - 184	690.0072	2066.9997	2066.9983	1	1	K.KYSHVDLVVMVDGFEGEK.G	Oxidation (M)	(Ions score 26)
167 - 184	690.0072	2066.9997	2066.9983	1	1	K.KYSHVDLVVMVDGFEGEK.G	Oxidation (M)	(Ions score 33)
167 - 184	690.0072	2066.9999	2066.9983	1	1	K.KYSHVDLVVMVDGFEGEK.G	Oxidation (M)	(Ions score 24)
167 - 184	690.0072	2066.9999	2066.9983	1	1	K.KYSHVDLVVMVDGFEGEK.G	Oxidation (M)	(Ions score 22)
167 - 184	517.7573	2067.0000	2066.9983	1	1	K.KYSHVDLVVMVDGFEGEK.G	Oxidation (M)	(Ions score 41)
167 - 184	690.0073	2067.0000	2066.9983	1	1	K.KYSHVDLVVMVDGFEGEK.G	Oxidation (M)	(Ions score 52)
167 - 184	517.7573	2067.0000	2066.9983	1	1	K.KYSHVDLVVMVDGFEGEK.G	Oxidation (M)	(Ions score 35)
167 - 184	690.0073	2067.0001	2066.9983	1	1	K.KYSHVDLVVMVDGFEGEK.G	Oxidation (M)	(Ions score 33)
167 - 184	517.7573	2067.0002	2066.9983	1	1	K.KYSHVDLVVMVDGFEGEK.G	Oxidation (M)	(Ions score 41)
167 - 184	690.0074	2067.0003	2066.9983	1	1	K.KYSHVDLVVMVDGFEGEK.G	Oxidation (M)	(Ions score 30)
167 - 184	517.7573	2067.0003	2066.9983	1	1	K.KYSHVDLVVMVDGFEGEK.G	Oxidation (M)	(Ions score 26)
167 - 184	690.0074	2067.0003	2066.9983	1	1	K.KYSHVDLVVMVDGFEGEK.G	Oxidation (M)	(Ions score 45)
167 - 184	690.0074	2067.0003	2066.9983	1	1	K.KYSHVDLVVMVDGFEGEK.G	Oxidation (M)	(Ions score 30)
167 - 184	517.7574	2067.0003	2066.9983	1	1	K.KYSHVDLVVMVDGFEGEK.G	Oxidation (M)	(Ions score 34)
167 - 184	690.0074	2067.0004	2066.9983	1	1	K.KYSHVDLVVMVDGFEGEK.G	Oxidation (M)	(Ions score 25)
167 - 184	690.0074	2067.0004	2066.9983	1	1	K.KYSHVDLVVMVDGFEGEK.G	Oxidation (M)	(Ions score 41)
167 - 184	517.7574	2067.0004	2066.9983	1	1	K.KYSHVDLVVMVDGFEGEK.G	Oxidation (M)	(Ions score 36)
167 - 184	517.7574	2067.0004	2066.9983	1	1	K.KYSHVDLVVMVDGFEGEK.G	Oxidation (M)	(Ions score 36)
167 - 184	690.0074	2067.0005	2066.9983	1	1	K.KYSHVDLVVMVDGFEGEK.G	Oxidation (M)	(Ions score 54)
167 - 184	690.0075	2067.0006	2066.9983	1	1	K.KYSHVDLVVMVDGFEGEK.G	Oxidation (M)	(Ions score 32)
167 - 184	690.0075	2067.0006	2066.9983	1	1	K.KYSHVDLVVMVDGFEGEK.G	Oxidation (M)	(Ions score 35)
167 - 184	517.7574	2067.0006	2066.9983	1	1	K.KYSHVDLVVMVDGFEGEK.G	Oxidation (M)	(Ions score 28)
167 - 184	517.7574	2067.0006	2066.9983	1	1	K.KYSHVDLVVMVDGFEGEK.G	Oxidation (M)	(Ions score 33)
167 - 184	690.0075	2067.0007	2066.9983	1	1	K.KYSHVDLVVMVDGFEGEK.G	Oxidation (M)	(Ions score 42)
167 - 184	1034.5076	2067.0007	2066.9983	1	1	K.KYSHVDLVVMVDGFEGEK.G	Oxidation (M)	(Ions score 49)
167 - 184	517.7575	2067.0008	2066.9983	1	1	K.KYSHVDLVVMVDGFEGEK.G	Oxidation (M)	(Ions score 36)
167 - 184	690.0076	2067.0009	2066.9983	1	1	K.KYSHVDLVVMVDGFEGEK.G	Oxidation (M)	(Ions score 37)
167 - 184	690.0076	2067.0009	2066.9983	1	1	K.KYSHVDLVVMVDGFEGEK.G	Oxidation (M)	(Ions score 28)
167 - 184	690.0076	2067.0009	2066.9983	1	1	K.KYSHVDLVVMVDGFEGEK.G	Oxidation (M)	(Ions score 38)
167 - 184	517.7575	2067.0009	2066.9983	1	1	K.KYSHVDLVVMVDGFEGEK.G	Oxidation (M)	(Ions score 41)
167 - 184	690.0076	2067.0009	2066.9983	1	1	K.KYSHVDLVVMVDGFEGEK.G	Oxidation (M)	(Ions score 46)
167 - 184	517.7575	2067.0009	2066.9983	1	1	K.KYSHVDLVVMVDGFEGEK.G	Oxidation (M)	(Ions score 27)
167 - 184	517.7575	2067.0010	2066.9983	1	1	K.KYSHVDLVVMVDGFEGEK.G	Oxidation (M)	(Ions score 33)
167 - 184	517.7575	2067.0011	2066.9983	1	1	K.KYSHVDLVVMVDGFEGEK.G	Oxidation (M)	(Ions score 31)
167 - 184	690.0076	2067.0011	2066.9983	1	1	K.KYSHVDLVVMVDGFEGEK.G	Oxidation (M)	(Ions score 34)
167 - 184	690.0077	2067.0012	2066.9983	1	1	K.KYSHVDLVVMVDGFEGEK.G	Oxidation (M)	(Ions score 45)
167 - 184	690.0077	2067.0012	2066.9983	1	1	K.KYSHVDLVVMVDGFEGEK.G	Oxidation (M)	(Ions score 49)
167 - 184	690.0077	2067.0012	2066.9983	1	1	K.KYSHVDLVVMVDGFEGEK.G	Oxidation (M)	(Ions score 21)
167 - 184	690.0077	2067.0013	2066.9983	1	1	K.KYSHVDLVVMVDGFEGEK.G	Oxidation (M)	(Ions score 47)
167 - 184	517.7576	2067.0014	2066.9983	1	1	K.KYSHVDLVVMVDGFEGEK.G	Oxidation (M)	(Ions score 33)
167 - 184	517.7576	2067.0014	2066.9983	2	1	K.KYSHVDLVVMVDGFEGEK.G	Oxidation (M)	(Ions score 33)
167 - 184	690.0078	2067.0015	2066.9983	2	1	K.KYSHVDLVVMVDGFEGEK.G	Oxidation (M)	(Ions score 47)
167 - 184	517.7577	2067.0015	2066.9983	2	1	K.KYSHVDLVVMVDGFEGEK.G	Oxidation (M)	(Ions score 37)
167 - 184	517.7577	2067.0016	2066.9983	2	1	K.KYSHVDLVVMVDGFEGEK.G	Oxidation (M)	(Ions score 34)

167 - 184	517.7577	2067.0017	2066.9983	2	1	K.KYSHVDLVVMVDGFEGEK.G	Oxidation (M)	(Ions score 34)
167 - 184	517.7577	2067.0017	2066.9983	2	1	K.KYSHVDLVVMVDGFEGEK.G	Oxidation (M)	(Ions score 25)
167 - 184	690.0078	2067.0017	2066.9983	2	1	K.KYSHVDLVVMVDGFEGEK.G	Oxidation (M)	(Ions score 79)
167 - 184	690.0079	2067.0017	2066.9983	2	1	K.KYSHVDLVVMVDGFEGEK.G	Oxidation (M)	(Ions score 54)
167 - 184	690.0079	2067.0018	2066.9983	2	1	K.KYSHVDLVVMVDGFEGEK.G	Oxidation (M)	(Ions score 54)
167 - 184	690.0079	2067.0018	2066.9983	2	1	K.KYSHVDLVVMVDGFEGEK.G	Oxidation (M)	(Ions score 27)
167 - 184	690.0079	2067.0018	2066.9983	2	1	K.KYSHVDLVVMVDGFEGEK.G	Oxidation (M)	(Ions score 32)
167 - 184	690.0079	2067.0019	2066.9983	2	1	K.KYSHVDLVVMVDGFEGEK.G	Oxidation (M)	(Ions score 54)
167 - 184	517.7578	2067.0019	2066.9983	2	1	K.KYSHVDLVVMVDGFEGEK.G	Oxidation (M)	(Ions score 25)
167 - 184	690.0079	2067.0020	2066.9983	2	1	K.KYSHVDLVVMVDGFEGEK.G	Oxidation (M)	(Ions score 24)
167 - 184	517.7578	2067.0020	2066.9983	2	1	K.KYSHVDLVVMVDGFEGEK.G	Oxidation (M)	(Ions score 37)
167 - 184	690.0080	2067.0020	2066.9983	2	1	K.KYSHVDLVVMVDGFEGEK.G	Oxidation (M)	(Ions score 44)
167 - 184	690.0080	2067.0021	2066.9983	2	1	K.KYSHVDLVVMVDGFEGEK.G	Oxidation (M)	(Ions score 35)
167 - 184	690.0080	2067.0021	2066.9983	2	1	K.KYSHVDLVVMVDGFEGEK.G	Oxidation (M)	(Ions score 39)
167 - 184	517.7578	2067.0021	2066.9983	2	1	K.KYSHVDLVVMVDGFEGEK.G	Oxidation (M)	(Ions score 32)
167 - 184	690.0080	2067.0021	2066.9983	2	1	K.KYSHVDLVVMVDGFEGEK.G	Oxidation (M)	(Ions score 35)
167 - 184	517.7578	2067.0021	2066.9983	2	1	K.KYSHVDLVVMVDGFEGEK.G	Oxidation (M)	(Ions score 23)
167 - 184	517.7578	2067.0022	2066.9983	2	1	K.KYSHVDLVVMVDGFEGEK.G	Oxidation (M)	(Ions score 35)
167 - 184	517.7578	2067.0022	2066.9983	2	1	K.KYSHVDLVVMVDGFEGEK.G	Oxidation (M)	(Ions score 32)
167 - 184	517.7579	2067.0023	2066.9983	2	1	K.KYSHVDLVVMVDGFEGEK.G	Oxidation (M)	(Ions score 24)
167 - 184	690.0081	2067.0023	2066.9983	2	1	K.KYSHVDLVVMVDGFEGEK.G	Oxidation (M)	(Ions score 45)
167 - 184	690.0081	2067.0023	2066.9983	2	1	K.KYSHVDLVVMVDGFEGEK.G	Oxidation (M)	(Ions score 55)
167 - 184	690.0081	2067.0024	2066.9983	2	1	K.KYSHVDLVVMVDGFEGEK.G	Oxidation (M)	(Ions score 42)
167 - 184	517.7579	2067.0024	2066.9983	2	1	K.KYSHVDLVVMVDGFEGEK.G	Oxidation (M)	(Ions score 30)
167 - 184	517.7579	2067.0024	2066.9983	2	1	K.KYSHVDLVVMVDGFEGEK.G	Oxidation (M)	(Ions score 23)
167 - 184	690.0081	2067.0025	2066.9983	2	1	K.KYSHVDLVVMVDGFEGEK.G	Oxidation (M)	(Ions score 49)
167 - 184	517.7579	2067.0025	2066.9983	2	1	K.KYSHVDLVVMVDGFEGEK.G	Oxidation (M)	(Ions score 24)
167 - 184	690.0081	2067.0025	2066.9983	2	1	K.KYSHVDLVVMVDGFEGEK.G	Oxidation (M)	(Ions score 52)
167 - 184	690.0081	2067.0025	2066.9983	2	1	K.KYSHVDLVVMVDGFEGEK.G	Oxidation (M)	(Ions score 33)
167 - 184	517.7579	2067.0026	2066.9983	2	1	K.KYSHVDLVVMVDGFEGEK.G	Oxidation (M)	(Ions score 32)
167 - 184	517.7579	2067.0026	2066.9983	2	1	K.KYSHVDLVVMVDGFEGEK.G	Oxidation (M)	(Ions score 31)
167 - 184	690.0081	2067.0026	2066.9983	2	1	K.KYSHVDLVVMVDGFEGEK.G	Oxidation (M)	(Ions score 54)
167 - 184	517.7579	2067.0026	2066.9983	2	1	K.KYSHVDLVVMVDGFEGEK.G	Oxidation (M)	(Ions score 34)
167 - 184	517.7579	2067.0027	2066.9983	2	1	K.KYSHVDLVVMVDGFEGEK.G	Oxidation (M)	(Ions score 25)
167 - 184	517.7579	2067.0027	2066.9983	2	1	K.KYSHVDLVVMVDGFEGEK.G	Oxidation (M)	(Ions score 31)
167 - 184	690.0082	2067.0027	2066.9983	2	1	K.KYSHVDLVVMVDGFEGEK.G	Oxidation (M)	(Ions score 62)
167 - 184	690.0082	2067.0028	2066.9983	2	1	K.KYSHVDLVVMVDGFEGEK.G	Oxidation (M)	(Ions score 35)
167 - 184	1034.5087	2067.0028	2066.9983	2	1	K.KYSHVDLVVMVDGFEGEK.G	Oxidation (M)	(Ions score 44)
167 - 184	690.0082	2067.0029	2066.9983	2	1	K.KYSHVDLVVMVDGFEGEK.G	Oxidation (M)	(Ions score 41)
167 - 184	690.0083	2067.0030	2066.9983	2	1	K.KYSHVDLVVMVDGFEGEK.G	Oxidation (M)	(Ions score 29)
167 - 184	690.0083	2067.0030	2066.9983	2	1	K.KYSHVDLVVMVDGFEGEK.G	Oxidation (M)	(Ions score 36)
167 - 184	517.7580	2067.0030	2066.9983	2	1	K.KYSHVDLVVMVDGFEGEK.G	Oxidation (M)	(Ions score 45)
167 - 184	517.7580	2067.0030	2066.9983	2	1	K.KYSHVDLVVMVDGFEGEK.G	Oxidation (M)	(Ions score 32)
167 - 184	690.0083	2067.0030	2066.9983	2	1	K.KYSHVDLVVMVDGFEGEK.G	Oxidation (M)	(Ions score 51)
167 - 184	690.0083	2067.0030	2066.9983	2	1	K.KYSHVDLVVMVDGFEGEK.G	Oxidation (M)	(Ions score 26)
167 - 184	690.0083	2067.0030	2066.9983	2	1	K.KYSHVDLVVMVDGFEGEK.G	Oxidation (M)	(Ions score 25)
167 - 184	690.0083	2067.0031	2066.9983	2	1	K.KYSHVDLVVMVDGFEGEK.G	Oxidation (M)	(Ions score 47)
167 - 184	690.0083	2067.0031	2066.9983	2	1	K.KYSHVDLVVMVDGFEGEK.G	Oxidation (M)	(Ions score 51)
167 - 184	690.0083	2067.0031	2066.9983	2	1	K.KYSHVDLVVMVDGFEGEK.G	Oxidation (M)	(Ions score 31)
167 - 184	690.0083	2067.0032	2066.9983	2	1	K.KYSHVDLVVMVDGFEGEK.G	Oxidation (M)	(Ions score 38)
167 - 184	690.0083	2067.0032	2066.9983	2	1	K.KYSHVDLVVMVDGFEGEK.G	Oxidation (M)	(Ions score 42)
167 - 184	690.0083	2067.0032	2066.9983	2	1	K.KYSHVDLVVMVDGFEGEK.G	Oxidation (M)	(Ions score 58)
167 - 184	690.0083	2067.0032	2066.9983	2	1	K.KYSHVDLVVMVDGFEGEK.G	Oxidation (M)	(Ions score 76)
167 - 184	1034.5089	2067.0032	2066.9983	2	1	K.KYSHVDLVVMVDGFEGEK.G	Oxidation (M)	(Ions score 34)
167 - 184	690.0084	2067.0033	2066.9983	2	1	K.KYSHVDLVVMVDGFEGEK.G	Oxidation (M)	(Ions score 38)
167 - 184	517.7581	2067.0033	2066.9983	2	1	K.KYSHVDLVVMVDGFEGEK.G	Oxidation (M)	(Ions score 33)
167 - 184	517.7581	2067.0034	2066.9983	2	1	K.KYSHVDLVVMVDGFEGEK.G	Oxidation (M)	(Ions score 35)

167 - 184	690.0084	2067.0034	2066.9983	2	1	K.KYSHVDLVVMVDGFEGEK.G	Oxidation (M)	(Ions score 46)
167 - 184	517.7581	2067.0034	2066.9983	2	1	K.KYSHVDLVVMVDGFEGEK.G	Oxidation (M)	(Ions score 29)
167 - 184	690.0084	2067.0034	2066.9983	2	1	K.KYSHVDLVVMVDGFEGEK.G	Oxidation (M)	(Ions score 44)
167 - 184	690.0084	2067.0034	2066.9983	2	1	K.KYSHVDLVVMVDGFEGEK.G	Oxidation (M)	(Ions score 52)
167 - 184	517.7581	2067.0035	2066.9983	2	1	K.KYSHVDLVVMVDGFEGEK.G	Oxidation (M)	(Ions score 34)
167 - 184	517.7581	2067.0035	2066.9983	3	1	K.KYSHVDLVVMVDGFEGEK.G	Oxidation (M)	(Ions score 33)
167 - 184	517.7582	2067.0035	2066.9983	3	1	K.KYSHVDLVVMVDGFEGEK.G	Oxidation (M)	(Ions score 36)
167 - 184	690.0084	2067.0035	2066.9983	3	1	K.KYSHVDLVVMVDGFEGEK.G	Oxidation (M)	(Ions score 44)
167 - 184	690.0085	2067.0035	2066.9983	3	1	K.KYSHVDLVVMVDGFEGEK.G	Oxidation (M)	(Ions score 30)
167 - 184	517.7582	2067.0036	2066.9983	3	1	K.KYSHVDLVVMVDGFEGEK.G	Oxidation (M)	(Ions score 38)
167 - 184	690.0085	2067.0036	2066.9983	3	1	K.KYSHVDLVVMVDGFEGEK.G	Oxidation (M)	(Ions score 51)
167 - 184	1034.5091	2067.0036	2066.9983	3	1	K.KYSHVDLVVMVDGFEGEK.G	Oxidation (M)	(Ions score 32)
167 - 184	690.0085	2067.0036	2066.9983	3	1	K.KYSHVDLVVMVDGFEGEK.G	Oxidation (M)	(Ions score 47)
167 - 184	517.7582	2067.0036	2066.9983	3	1	K.KYSHVDLVVMVDGFEGEK.G	Oxidation (M)	(Ions score 32)
167 - 184	517.7582	2067.0036	2066.9983	3	1	K.KYSHVDLVVMVDGFEGEK.G	Oxidation (M)	(Ions score 37)
167 - 184	690.0085	2067.0036	2066.9983	3	1	K.KYSHVDLVVMVDGFEGEK.G	Oxidation (M)	(Ions score 42)
167 - 184	517.7582	2067.0036	2066.9983	3	1	K.KYSHVDLVVMVDGFEGEK.G	Oxidation (M)	(Ions score 30)
167 - 184	517.7582	2067.0036	2066.9983	3	1	K.KYSHVDLVVMVDGFEGEK.G	Oxidation (M)	(Ions score 23)
167 - 184	690.0085	2067.0036	2066.9983	3	1	K.KYSHVDLVVMVDGFEGEK.G	Oxidation (M)	(Ions score 38)
167 - 184	517.7582	2067.0037	2066.9983	3	1	K.KYSHVDLVVMVDGFEGEK.G	Oxidation (M)	(Ions score 32)
167 - 184	517.7582	2067.0037	2066.9983	3	1	K.KYSHVDLVVMVDGFEGEK.G	Oxidation (M)	(Ions score 34)
167 - 184	517.7582	2067.0037	2066.9983	3	1	K.KYSHVDLVVMVDGFEGEK.G	Oxidation (M)	(Ions score 34)
167 - 184	517.7582	2067.0038	2066.9983	3	1	K.KYSHVDLVVMVDGFEGEK.G	Oxidation (M)	(Ions score 34)
167 - 184	517.7582	2067.0039	2066.9983	3	1	K.KYSHVDLVVMVDGFEGEK.G	Oxidation (M)	(Ions score 29)
167 - 184	690.0086	2067.0039	2066.9983	3	1	K.KYSHVDLVVMVDGFEGEK.G	Oxidation (M)	(Ions score 59)
167 - 184	517.7583	2067.0039	2066.9983	3	1	K.KYSHVDLVVMVDGFEGEK.G	Oxidation (M)	(Ions score 29)
167 - 184	517.7583	2067.0039	2066.9983	3	1	K.KYSHVDLVVMVDGFEGEK.G	Oxidation (M)	(Ions score 37)
167 - 184	690.0086	2067.0039	2066.9983	3	1	K.KYSHVDLVVMVDGFEGEK.G	Oxidation (M)	(Ions score 52)
167 - 184	690.0086	2067.0040	2066.9983	3	1	K.KYSHVDLVVMVDGFEGEK.G	Oxidation (M)	(Ions score 47)
167 - 184	690.0086	2067.0040	2066.9983	3	1	K.KYSHVDLVVMVDGFEGEK.G	Oxidation (M)	(Ions score 54)
167 - 184	690.0086	2067.0040	2066.9983	3	1	K.KYSHVDLVVMVDGFEGEK.G	Oxidation (M)	(Ions score 46)
167 - 184	690.0086	2067.0040	2066.9983	3	1	K.KYSHVDLVVMVDGFEGEK.G	Oxidation (M)	(Ions score 45)
167 - 184	517.7583	2067.0040	2066.9983	3	1	K.KYSHVDLVVMVDGFEGEK.G	Oxidation (M)	(Ions score 36)
167 - 184	690.0086	2067.0040	2066.9983	3	1	K.KYSHVDLVVMVDGFEGEK.G	Oxidation (M)	(Ions score 43)
167 - 184	517.7583	2067.0041	2066.9983	3	1	K.KYSHVDLVVMVDGFEGEK.G	Oxidation (M)	(Ions score 24)
167 - 184	517.7583	2067.0041	2066.9983	3	1	K.KYSHVDLVVMVDGFEGEK.G	Oxidation (M)	(Ions score 26)
167 - 184	517.7583	2067.0041	2066.9983	3	1	K.KYSHVDLVVMVDGFEGEK.G	Oxidation (M)	(Ions score 43)
167 - 184	517.7583	2067.0041	2066.9983	3	1	K.KYSHVDLVVMVDGFEGEK.G	Oxidation (M)	(Ions score 33)
167 - 184	517.7583	2067.0042	2066.9983	3	1	K.KYSHVDLVVMVDGFEGEK.G	Oxidation (M)	(Ions score 36)
167 - 184	517.7583	2067.0042	2066.9983	3	1	K.KYSHVDLVVMVDGFEGEK.G	Oxidation (M)	(Ions score 32)
167 - 184	690.0087	2067.0043	2066.9983	3	1	K.KYSHVDLVVMVDGFEGEK.G	Oxidation (M)	(Ions score 54)
167 - 184	690.0087	2067.0043	2066.9983	3	1	K.KYSHVDLVVMVDGFEGEK.G	Oxidation (M)	(Ions score 51)
167 - 184	517.7584	2067.0043	2066.9983	3	1	K.KYSHVDLVVMVDGFEGEK.G	Oxidation (M)	(Ions score 34)
167 - 184	690.0087	2067.0043	2066.9983	3	1	K.KYSHVDLVVMVDGFEGEK.G	Oxidation (M)	(Ions score 37)
167 - 184	517.7584	2067.0043	2066.9983	3	1	K.KYSHVDLVVMVDGFEGEK.G	Oxidation (M)	(Ions score 25)
167 - 184	690.0087	2067.0044	2066.9983	3	1	K.KYSHVDLVVMVDGFEGEK.G	Oxidation (M)	(Ions score 39)
167 - 184	690.0087	2067.0044	2066.9983	3	1	K.KYSHVDLVVMVDGFEGEK.G	Oxidation (M)	(Ions score 55)
167 - 184	690.0087	2067.0044	2066.9983	3	1	K.KYSHVDLVVMVDGFEGEK.G	Oxidation (M)	(Ions score 41)
167 - 184	690.0087	2067.0044	2066.9983	3	1	K.KYSHVDLVVMVDGFEGEK.G	Oxidation (M)	(Ions score 43)
167 - 184	517.7584	2067.0044	2066.9983	3	1	K.KYSHVDLVVMVDGFEGEK.G	Oxidation (M)	(Ions score 29)
167 - 184	690.0087	2067.0044	2066.9983	3	1	K.KYSHVDLVVMVDGFEGEK.G	Oxidation (M)	(Ions score 37)
167 - 184	517.7584	2067.0044	2066.9983	3	1	K.KYSHVDLVVMVDGFEGEK.G	Oxidation (M)	(Ions score 33)
167 - 184	1034.5095	2067.0044	2066.9983	3	1	K.KYSHVDLVVMVDGFEGEK.G	Oxidation (M)	(Ions score 60)
167 - 184	690.0087	2067.0044	2066.9983	3	1	K.KYSHVDLVVMVDGFEGEK.G	Oxidation (M)	(Ions score 53)
167 - 184	517.7584	2067.0044	2066.9983	3	1	K.KYSHVDLVVMVDGFEGEK.G	Oxidation (M)	(Ions score 34)
167 - 184	690.0088	2067.0045	2066.9983	3	1	K.KYSHVDLVVMVDGFEGEK.G	Oxidation (M)	(Ions score 22)
167 - 184	690.0088	2067.0045	2066.9983	3	1	K.KYSHVDLVVMVDGFEGEK.G	Oxidation (M)	(Ions score 36)

167 - 184	517.7584	2067.0045	2066.9983	3	1	K.KYSHVDLVVMVDGFEGEK.G	Oxidation (M)	(Ions score 32)
167 - 184	517.7584	2067.0045	2066.9983	3	1	K.KYSHVDLVVMVDGFEGEK.G	Oxidation (M)	(Ions score 29)
167 - 184	517.7584	2067.0045	2066.9983	3	1	K.KYSHVDLVVMVDGFEGEK.G	Oxidation (M)	(Ions score 34)
167 - 184	690.0088	2067.0045	2066.9983	3	1	K.KYSHVDLVVMVDGFEGEK.G	Oxidation (M)	(Ions score 46)
167 - 184	690.0088	2067.0045	2066.9983	3	1	K.KYSHVDLVVMVDGFEGEK.G	Oxidation (M)	(Ions score 44)
167 - 184	517.7584	2067.0045	2066.9983	3	1	K.KYSHVDLVVMVDGFEGEK.G	Oxidation (M)	(Ions score 33)
167 - 184	690.0088	2067.0046	2066.9983	3	1	K.KYSHVDLVVMVDGFEGEK.G	Oxidation (M)	(Ions score 40)
167 - 184	690.0088	2067.0046	2066.9983	3	1	K.KYSHVDLVVMVDGFEGEK.G	Oxidation (M)	(Ions score 65)
167 - 184	690.0088	2067.0046	2066.9983	3	1	K.KYSHVDLVVMVDGFEGEK.G	Oxidation (M)	(Ions score 43)
167 - 184	690.0088	2067.0046	2066.9983	3	1	K.KYSHVDLVVMVDGFEGEK.G	Oxidation (M)	(Ions score 41)
167 - 184	517.7584	2067.0046	2066.9983	3	1	K.KYSHVDLVVMVDGFEGEK.G	Oxidation (M)	(Ions score 33)
167 - 184	517.7584	2067.0046	2066.9983	3	1	K.KYSHVDLVVMVDGFEGEK.G	Oxidation (M)	(Ions score 47)
167 - 184	517.7584	2067.0047	2066.9983	3	1	K.KYSHVDLVVMVDGFEGEK.G	Oxidation (M)	(Ions score 36)
167 - 184	517.7584	2067.0047	2066.9983	3	1	K.KYSHVDLVVMVDGFEGEK.G	Oxidation (M)	(Ions score 24)
167 - 184	690.0088	2067.0047	2066.9983	3	1	K.KYSHVDLVVMVDGFEGEK.G	Oxidation (M)	(Ions score 44)
167 - 184	690.0088	2067.0047	2066.9983	3	1	K.KYSHVDLVVMVDGFEGEK.G	Oxidation (M)	(Ions score 29)
167 - 184	517.7585	2067.0047	2066.9983	3	1	K.KYSHVDLVVMVDGFEGEK.G	Oxidation (M)	(Ions score 40)
167 - 184	517.7585	2067.0048	2066.9983	3	1	K.KYSHVDLVVMVDGFEGEK.G	Oxidation (M)	(Ions score 33)
167 - 184	517.7585	2067.0048	2066.9983	3	1	K.KYSHVDLVVMVDGFEGEK.G	Oxidation (M)	(Ions score 30)
167 - 184	517.7585	2067.0048	2066.9983	3	1	K.KYSHVDLVVMVDGFEGEK.G	Oxidation (M)	(Ions score 36)
167 - 184	517.7585	2067.0048	2066.9983	3	1	K.KYSHVDLVVMVDGFEGEK.G	Oxidation (M)	(Ions score 33)
167 - 184	517.7585	2067.0049	2066.9983	3	1	K.KYSHVDLVVMVDGFEGEK.G	Oxidation (M)	(Ions score 34)
167 - 184	517.7585	2067.0049	2066.9983	3	1	K.KYSHVDLVVMVDGFEGEK.G	Oxidation (M)	(Ions score 40)
167 - 184	517.7585	2067.0049	2066.9983	3	1	K.KYSHVDLVVMVDGFEGEK.G	Oxidation (M)	(Ions score 34)
167 - 184	517.7585	2067.0049	2066.9983	3	1	K.KYSHVDLVVMVDGFEGEK.G	Oxidation (M)	(Ions score 36)
167 - 184	690.0089	2067.0050	2066.9983	3	1	K.KYSHVDLVVMVDGFEGEK.G	Oxidation (M)	(Ions score 45)
167 - 184	517.7585	2067.0050	2066.9983	3	1	K.KYSHVDLVVMVDGFEGEK.G	Oxidation (M)	(Ions score 32)
167 - 184	517.7585	2067.0050	2066.9983	3	1	K.KYSHVDLVVMVDGFEGEK.G	Oxidation (M)	(Ions score 23)
167 - 184	517.7585	2067.0050	2066.9983	3	1	K.KYSHVDLVVMVDGFEGEK.G	Oxidation (M)	(Ions score 23)
167 - 184	517.7585	2067.0050	2066.9983	3	1	K.KYSHVDLVVMVDGFEGEK.G	Oxidation (M)	(Ions score 29)
167 - 184	517.7585	2067.0051	2066.9983	3	1	K.KYSHVDLVVMVDGFEGEK.G	Oxidation (M)	(Ions score 32)
167 - 184	690.0090	2067.0051	2066.9983	3	1	K.KYSHVDLVVMVDGFEGEK.G	Oxidation (M)	(Ions score 54)
167 - 184	690.0090	2067.0051	2066.9983	3	1	K.KYSHVDLVVMVDGFEGEK.G	Oxidation (M)	(Ions score 50)
167 - 184	690.0090	2067.0051	2066.9983	3	1	K.KYSHVDLVVMVDGFEGEK.G	Oxidation (M)	(Ions score 44)
167 - 184	690.0090	2067.0051	2066.9983	3	1	K.KYSHVDLVVMVDGFEGEK.G	Oxidation (M)	(Ions score 46)
167 - 184	517.7586	2067.0051	2066.9983	3	1	K.KYSHVDLVVMVDGFEGEK.G	Oxidation (M)	(Ions score 26)
167 - 184	690.0090	2067.0051	2066.9983	3	1	K.KYSHVDLVVMVDGFEGEK.G	Oxidation (M)	(Ions score 27)
167 - 184	517.7586	2067.0051	2066.9983	3	1	K.KYSHVDLVVMVDGFEGEK.G	Oxidation (M)	(Ions score 32)
167 - 184	517.7586	2067.0052	2066.9983	3	1	K.KYSHVDLVVMVDGFEGEK.G	Oxidation (M)	(Ions score 38)
167 - 184	517.7586	2067.0052	2066.9983	3	1	K.KYSHVDLVVMVDGFEGEK.G	Oxidation (M)	(Ions score 36)
167 - 184	517.7586	2067.0052	2066.9983	3	1	K.KYSHVDLVVMVDGFEGEK.G	Oxidation (M)	(Ions score 36)
167 - 184	517.7586	2067.0052	2066.9983	3	1	K.KYSHVDLVVMVDGFEGEK.G	Oxidation (M)	(Ions score 36)
167 - 184	690.0090	2067.0052	2066.9983	3	1	K.KYSHVDLVVMVDGFEGEK.G	Oxidation (M)	(Ions score 23)
167 - 184	517.7586	2067.0053	2066.9983	3	1	K.KYSHVDLVVMVDGFEGEK.G	Oxidation (M)	(Ions score 41)
167 - 184	690.0090	2067.0053	2066.9983	3	1	K.KYSHVDLVVMVDGFEGEK.G	Oxidation (M)	(Ions score 45)
167 - 184	517.7586	2067.0053	2066.9983	3	1	K.KYSHVDLVVMVDGFEGEK.G	Oxidation (M)	(Ions score 39)
167 - 184	517.7586	2067.0053	2066.9983	3	1	K.KYSHVDLVVMVDGFEGEK.G	Oxidation (M)	(Ions score 35)
167 - 184	690.0091	2067.0053	2066.9983	3	1	K.KYSHVDLVVMVDGFEGEK.G	Oxidation (M)	(Ions score 55)
167 - 184	517.7586	2067.0053	2066.9983	3	1	K.KYSHVDLVVMVDGFEGEK.G	Oxidation (M)	(Ions score 31)
167 - 184	517.7586	2067.0054	2066.9983	3	1	K.KYSHVDLVVMVDGFEGEK.G	Oxidation (M)	(Ions score 39)
167 - 184	690.0091	2067.0054	2066.9983	3	1	K.KYSHVDLVVMVDGFEGEK.G	Oxidation (M)	(Ions score 44)
167 - 184	690.0091	2067.0054	2066.9983	3	1	K.KYSHVDLVVMVDGFEGEK.G	Oxidation (M)	(Ions score 38)
167 - 184	517.7586	2067.0054	2066.9983	3	1	K.KYSHVDLVVMVDGFEGEK.G	Oxidation (M)	(Ions score 33)
167 - 184	690.0091	2067.0054	2066.9983	3	1	K.KYSHVDLVVMVDGFEGEK.G	Oxidation (M)	(Ions score 46)
167 - 184	517.7586	2067.0054	2066.9983	3	1	K.KYSHVDLVVMVDGFEGEK.G	Oxidation (M)	(Ions score 29)
167 - 184	517.7586	2067.0054	2066.9983	3	1	K.KYSHVDLVVMVDGFEGEK.G	Oxidation (M)	(Ions score 40)
167 - 184	690.0091	2067.0055	2066.9983	3	1	K.KYSHVDLVVMVDGFEGEK.G	Oxidation (M)	(Ions score 51)

167 - 184	517.7587	2067.0055	2066.9983	3	1	K.KYSHVDLVVMVDGFEGEK.G	Oxidation (M)	(Ions score 30)
167 - 184	517.7587	2067.0055	2066.9983	3	1	K.KYSHVDLVVMVDGFEGEK.G	Oxidation (M)	(Ions score 39)
167 - 184	690.0091	2067.0055	2066.9983	3	1	K.KYSHVDLVVMVDGFEGEK.G	Oxidation (M)	(Ions score 50)
167 - 184	517.7587	2067.0055	2066.9983	3	1	K.KYSHVDLVVMVDGFEGEK.G	Oxidation (M)	(Ions score 25)
167 - 184	517.7587	2067.0056	2066.9983	4	1	K.KYSHVDLVVMVDGFEGEK.G	Oxidation (M)	(Ions score 29)
167 - 184	517.7587	2067.0057	2066.9983	4	1	K.KYSHVDLVVMVDGFEGEK.G	Oxidation (M)	(Ions score 39)
167 - 184	690.0092	2067.0057	2066.9983	4	1	K.KYSHVDLVVMVDGFEGEK.G	Oxidation (M)	(Ions score 35)
167 - 184	517.7587	2067.0057	2066.9983	4	1	K.KYSHVDLVVMVDGFEGEK.G	Oxidation (M)	(Ions score 34)
167 - 184	690.0092	2067.0057	2066.9983	4	1	K.KYSHVDLVVMVDGFEGEK.G	Oxidation (M)	(Ions score 36)
167 - 184	517.7587	2067.0058	2066.9983	4	1	K.KYSHVDLVVMVDGFEGEK.G	Oxidation (M)	(Ions score 29)
167 - 184	690.0092	2067.0058	2066.9983	4	1	K.KYSHVDLVVMVDGFEGEK.G	Oxidation (M)	(Ions score 44)
167 - 184	517.7587	2067.0058	2066.9983	4	1	K.KYSHVDLVVMVDGFEGEK.G	Oxidation (M)	(Ions score 39)
167 - 184	517.7587	2067.0058	2066.9983	4	1	K.KYSHVDLVVMVDGFEGEK.G	Oxidation (M)	(Ions score 35)
167 - 184	517.7587	2067.0058	2066.9983	4	1	K.KYSHVDLVVMVDGFEGEK.G	Oxidation (M)	(Ions score 34)
167 - 184	690.0092	2067.0058	2066.9983	4	1	K.KYSHVDLVVMVDGFEGEK.G	Oxidation (M)	(Ions score 35)
167 - 184	517.7587	2067.0059	2066.9983	4	1	K.KYSHVDLVVMVDGFEGEK.G	Oxidation (M)	(Ions score 28)
167 - 184	517.7587	2067.0059	2066.9983	4	1	K.KYSHVDLVVMVDGFEGEK.G	Oxidation (M)	(Ions score 42)
167 - 184	517.7587	2067.0059	2066.9983	4	1	K.KYSHVDLVVMVDGFEGEK.G	Oxidation (M)	(Ions score 35)
167 - 184	517.7587	2067.0059	2066.9983	4	1	K.KYSHVDLVVMVDGFEGEK.G	Oxidation (M)	(Ions score 30)
167 - 184	517.7587	2067.0059	2066.9983	4	1	K.KYSHVDLVVMVDGFEGEK.G	Oxidation (M)	(Ions score 33)
167 - 184	690.0092	2067.0059	2066.9983	4	1	K.KYSHVDLVVMVDGFEGEK.G	Oxidation (M)	(Ions score 38)
167 - 184	690.0093	2067.0059	2066.9983	4	1	K.KYSHVDLVVMVDGFEGEK.G	Oxidation (M)	(Ions score 53)
167 - 184	517.7588	2067.0060	2066.9983	4	1	K.KYSHVDLVVMVDGFEGEK.G	Oxidation (M)	(Ions score 34)
167 - 184	517.7588	2067.0060	2066.9983	4	1	K.KYSHVDLVVMVDGFEGEK.G	Oxidation (M)	(Ions score 38)
167 - 184	690.0093	2067.0060	2066.9983	4	1	K.KYSHVDLVVMVDGFEGEK.G	Oxidation (M)	(Ions score 45)
167 - 184	517.7588	2067.0060	2066.9983	4	1	K.KYSHVDLVVMVDGFEGEK.G	Oxidation (M)	(Ions score 27)
167 - 184	690.0093	2067.0060	2066.9983	4	1	K.KYSHVDLVVMVDGFEGEK.G	Oxidation (M)	(Ions score 41)
167 - 184	690.0093	2067.0061	2066.9983	4	1	K.KYSHVDLVVMVDGFEGEK.G	Oxidation (M)	(Ions score 35)
167 - 184	690.0093	2067.0061	2066.9983	4	1	K.KYSHVDLVVMVDGFEGEK.G	Oxidation (M)	(Ions score 43)
167 - 184	517.7588	2067.0061	2066.9983	4	1	K.KYSHVDLVVMVDGFEGEK.G	Oxidation (M)	(Ions score 31)
167 - 184	517.7588	2067.0061	2066.9983	4	1	K.KYSHVDLVVMVDGFEGEK.G	Oxidation (M)	(Ions score 34)
167 - 184	690.0093	2067.0061	2066.9983	4	1	K.KYSHVDLVVMVDGFEGEK.G	Oxidation (M)	(Ions score 51)
167 - 184	517.7588	2067.0061	2066.9983	4	1	K.KYSHVDLVVMVDGFEGEK.G	Oxidation (M)	(Ions score 35)
167 - 184	690.0093	2067.0061	2066.9983	4	1	K.KYSHVDLVVMVDGFEGEK.G	Oxidation (M)	(Ions score 70)
167 - 184	517.7588	2067.0062	2066.9983	4	1	K.KYSHVDLVVMVDGFEGEK.G	Oxidation (M)	(Ions score 34)
167 - 184	690.0093	2067.0062	2066.9983	4	1	K.KYSHVDLVVMVDGFEGEK.G	Oxidation (M)	(Ions score 43)
167 - 184	517.7588	2067.0062	2066.9983	4	1	K.KYSHVDLVVMVDGFEGEK.G	Oxidation (M)	(Ions score 33)
167 - 184	517.7588	2067.0062	2066.9983	4	1	K.KYSHVDLVVMVDGFEGEK.G	Oxidation (M)	(Ions score 31)
167 - 184	517.7588	2067.0062	2066.9983	4	1	K.KYSHVDLVVMVDGFEGEK.G	Oxidation (M)	(Ions score 38)
167 - 184	517.7588	2067.0062	2066.9983	4	1	K.KYSHVDLVVMVDGFEGEK.G	Oxidation (M)	(Ions score 35)
167 - 184	517.7588	2067.0062	2066.9983	4	1	K.KYSHVDLVVMVDGFEGEK.G	Oxidation (M)	(Ions score 36)
167 - 184	517.7588	2067.0063	2066.9983	4	1	K.KYSHVDLVVMVDGFEGEK.G	Oxidation (M)	(Ions score 34)
167 - 184	517.7588	2067.0063	2066.9983	4	1	K.KYSHVDLVVMVDGFEGEK.G	Oxidation (M)	(Ions score 33)
167 - 184	517.7589	2067.0063	2066.9983	4	1	K.KYSHVDLVVMVDGFEGEK.G	Oxidation (M)	(Ions score 34)
167 - 184	517.7589	2067.0063	2066.9983	4	1	K.KYSHVDLVVMVDGFEGEK.G	Oxidation (M)	(Ions score 37)
167 - 184	517.7589	2067.0063	2066.9983	4	1	K.KYSHVDLVVMVDGFEGEK.G	Oxidation (M)	(Ions score 41)
167 - 184	517.7589	2067.0063	2066.9983	4	1	K.KYSHVDLVVMVDGFEGEK.G	Oxidation (M)	(Ions score 27)
167 - 184	690.0094	2067.0063	2066.9983	4	1	K.KYSHVDLVVMVDGFEGEK.G	Oxidation (M)	(Ions score 47)
167 - 184	517.7589	2067.0063	2066.9983	4	1	K.KYSHVDLVVMVDGFEGEK.G	Oxidation (M)	(Ions score 36)
167 - 184	690.0094	2067.0064	2066.9983	4	1	K.KYSHVDLVVMVDGFEGEK.G	Oxidation (M)	(Ions score 45)
167 - 184	690.0094	2067.0064	2066.9983	4	1	K.KYSHVDLVVMVDGFEGEK.G	Oxidation (M)	(Ions score 44)
167 - 184	517.7589	2067.0064	2066.9983	4	1	K.KYSHVDLVVMVDGFEGEK.G	Oxidation (M)	(Ions score 36)
167 - 184	690.0094	2067.0064	2066.9983	4	1	K.KYSHVDLVVMVDGFEGEK.G	Oxidation (M)	(Ions score 70)
167 - 184	517.7589	2067.0064	2066.9983	4	1	K.KYSHVDLVVMVDGFEGEK.G	Oxidation (M)	(Ions score 39)
167 - 184	690.0094	2067.0065	2066.9983	4	1	K.KYSHVDLVVMVDGFEGEK.G	Oxidation (M)	(Ions score 27)
167 - 184	517.7589	2067.0065	2066.9983	4	1	K.KYSHVDLVVMVDGFEGEK.G	Oxidation (M)	(Ions score 33)
167 - 184	517.7589	2067.0065	2066.9983	4	1	K.KYSHVDLVVMVDGFEGEK.G	Oxidation (M)	(Ions score 31)

167 - 184	690.0094	2067.0065	2066.9983	4	1	K.KYSHVDLVVMVDGFEGEK.G	Oxidation (M)	(Ions score 41)
167 - 184	690.0094	2067.0065	2066.9983	4	1	K.KYSHVDLVVMVDGFEGEK.G	Oxidation (M)	(Ions score 51)
167 - 184	690.0094	2067.0065	2066.9983	4	1	K.KYSHVDLVVMVDGFEGEK.G	Oxidation (M)	(Ions score 66)
167 - 184	517.7589	2067.0065	2066.9983	4	1	K.KYSHVDLVVMVDGFEGEK.G	Oxidation (M)	(Ions score 27)
167 - 184	517.7589	2067.0065	2066.9983	4	1	K.KYSHVDLVVMVDGFEGEK.G	Oxidation (M)	(Ions score 33)
167 - 184	690.0095	2067.0065	2066.9983	4	1	K.KYSHVDLVVMVDGFEGEK.G	Oxidation (M)	(Ions score 52)
167 - 184	690.0095	2067.0065	2066.9983	4	1	K.KYSHVDLVVMVDGFEGEK.G	Oxidation (M)	(Ions score 57)
167 - 184	517.7589	2067.0065	2066.9983	4	1	K.KYSHVDLVVMVDGFEGEK.G	Oxidation (M)	(Ions score 33)
167 - 184	690.0095	2067.0066	2066.9983	4	1	K.KYSHVDLVVMVDGFEGEK.G	Oxidation (M)	(Ions score 47)
167 - 184	517.7589	2067.0066	2066.9983	4	1	K.KYSHVDLVVMVDGFEGEK.G	Oxidation (M)	(Ions score 23)
167 - 184	690.0095	2067.0066	2066.9983	4	1	K.KYSHVDLVVMVDGFEGEK.G	Oxidation (M)	(Ions score 74)
167 - 184	517.7590	2067.0067	2066.9983	4	1	K.KYSHVDLVVMVDGFEGEK.G	Oxidation (M)	(Ions score 43)
167 - 184	517.7590	2067.0068	2066.9983	4	1	K.KYSHVDLVVMVDGFEGEK.G	Oxidation (M)	(Ions score 43)
167 - 184	517.7590	2067.0068	2066.9983	4	1	K.KYSHVDLVVMVDGFEGEK.G	Oxidation (M)	(Ions score 35)
167 - 184	1034.5107	2067.0068	2066.9983	4	1	K.KYSHVDLVVMVDGFEGEK.G	Oxidation (M)	(Ions score 67)
167 - 184	517.7590	2067.0069	2066.9983	4	1	K.KYSHVDLVVMVDGFEGEK.G	Oxidation (M)	(Ions score 53)
167 - 184	517.7590	2067.0069	2066.9983	4	1	K.KYSHVDLVVMVDGFEGEK.G	Oxidation (M)	(Ions score 33)
167 - 184	517.7590	2067.0070	2066.9983	4	1	K.KYSHVDLVVMVDGFEGEK.G	Oxidation (M)	(Ions score 31)
167 - 184	690.0096	2067.0070	2066.9983	4	1	K.KYSHVDLVVMVDGFEGEK.G	Oxidation (M)	(Ions score 43)
167 - 184	690.0096	2067.0070	2066.9983	4	1	K.KYSHVDLVVMVDGFEGEK.G	Oxidation (M)	(Ions score 32)
167 - 184	517.7590	2067.0070	2066.9983	4	1	K.KYSHVDLVVMVDGFEGEK.G	Oxidation (M)	(Ions score 26)
167 - 184	517.7590	2067.0070	2066.9983	4	1	K.KYSHVDLVVMVDGFEGEK.G	Oxidation (M)	(Ions score 28)
167 - 184	690.0096	2067.0071	2066.9983	4	1	K.KYSHVDLVVMVDGFEGEK.G	Oxidation (M)	(Ions score 34)
167 - 184	517.7590	2067.0071	2066.9983	4	1	K.KYSHVDLVVMVDGFEGEK.G	Oxidation (M)	(Ions score 36)
167 - 184	690.0096	2067.0071	2066.9983	4	1	K.KYSHVDLVVMVDGFEGEK.G	Oxidation (M)	(Ions score 44)
167 - 184	517.7591	2067.0071	2066.9983	4	1	K.KYSHVDLVVMVDGFEGEK.G	Oxidation (M)	(Ions score 39)
167 - 184	690.0097	2067.0072	2066.9983	4	1	K.KYSHVDLVVMVDGFEGEK.G	Oxidation (M)	(Ions score 47)
167 - 184	690.0097	2067.0072	2066.9983	4	1	K.KYSHVDLVVMVDGFEGEK.G	Oxidation (M)	(Ions score 43)
167 - 184	517.7591	2067.0073	2066.9983	4	1	K.KYSHVDLVVMVDGFEGEK.G	Oxidation (M)	(Ions score 33)
167 - 184	690.0097	2067.0073	2066.9983	4	1	K.KYSHVDLVVMVDGFEGEK.G	Oxidation (M)	(Ions score 33)
167 - 184	690.0097	2067.0073	2066.9983	4	1	K.KYSHVDLVVMVDGFEGEK.G	Oxidation (M)	(Ions score 46)
167 - 184	1034.5109	2067.0073	2066.9983	4	1	K.KYSHVDLVVMVDGFEGEK.G	Oxidation (M)	(Ions score 49)
167 - 184	690.0097	2067.0073	2066.9983	4	1	K.KYSHVDLVVMVDGFEGEK.G	Oxidation (M)	(Ions score 36)
167 - 184	690.0097	2067.0074	2066.9983	4	1	K.KYSHVDLVVMVDGFEGEK.G	Oxidation (M)	(Ions score 46)
167 - 184	517.7591	2067.0074	2066.9983	4	1	K.KYSHVDLVVMVDGFEGEK.G	Oxidation (M)	(Ions score 37)
167 - 184	517.7591	2067.0075	2066.9983	4	1	K.KYSHVDLVVMVDGFEGEK.G	Oxidation (M)	(Ions score 39)
167 - 184	517.7592	2067.0075	2066.9983	4	1	K.KYSHVDLVVMVDGFEGEK.G	Oxidation (M)	(Ions score 29)
167 - 184	690.0098	2067.0076	2066.9983	5	1	K.KYSHVDLVVMVDGFEGEK.G	Oxidation (M)	(Ions score 37)
167 - 184	690.0098	2067.0076	2066.9983	5	1	K.KYSHVDLVVMVDGFEGEK.G	Oxidation (M)	(Ions score 55)
167 - 184	517.7592	2067.0077	2066.9983	5	1	K.KYSHVDLVVMVDGFEGEK.G	Oxidation (M)	(Ions score 31)
167 - 184	517.7592	2067.0077	2066.9983	5	1	K.KYSHVDLVVMVDGFEGEK.G	Oxidation (M)	(Ions score 31)
167 - 184	517.7592	2067.0077	2066.9983	5	1	K.KYSHVDLVVMVDGFEGEK.G	Oxidation (M)	(Ions score 34)
167 - 184	517.7592	2067.0078	2066.9983	5	1	K.KYSHVDLVVMVDGFEGEK.G	Oxidation (M)	(Ions score 22)
167 - 184	517.7592	2067.0078	2066.9983	5	1	K.KYSHVDLVVMVDGFEGEK.G	Oxidation (M)	(Ions score 33)
167 - 184	690.0099	2067.0078	2066.9983	5	1	K.KYSHVDLVVMVDGFEGEK.G	Oxidation (M)	(Ions score 42)
167 - 184	690.0099	2067.0078	2066.9983	5	1	K.KYSHVDLVVMVDGFEGEK.G	Oxidation (M)	(Ions score 35)
167 - 184	517.7592	2067.0078	2066.9983	5	1	K.KYSHVDLVVMVDGFEGEK.G	Oxidation (M)	(Ions score 32)
167 - 184	690.0099	2067.0079	2066.9983	5	1	K.KYSHVDLVVMVDGFEGEK.G	Oxidation (M)	(Ions score 36)
167 - 184	517.7592	2067.0079	2066.9983	5	1	K.KYSHVDLVVMVDGFEGEK.G	Oxidation (M)	(Ions score 22)
167 - 184	517.7593	2067.0080	2066.9983	5	1	K.KYSHVDLVVMVDGFEGEK.G	Oxidation (M)	(Ions score 32)
167 - 184	690.0100	2067.0080	2066.9983	5	1	K.KYSHVDLVVMVDGFEGEK.G	Oxidation (M)	(Ions score 60)
167 - 184	690.0100	2067.0081	2066.9983	5	1	K.KYSHVDLVVMVDGFEGEK.G	Oxidation (M)	(Ions score 55)
167 - 184	690.0100	2067.0081	2066.9983	5	1	K.KYSHVDLVVMVDGFEGEK.G	Oxidation (M)	(Ions score 44)
167 - 184	517.7593	2067.0081	2066.9983	5	1	K.KYSHVDLVVMVDGFEGEK.G	Oxidation (M)	(Ions score 37)
167 - 184	690.0100	2067.0082	2066.9983	5	1	K.KYSHVDLVVMVDGFEGEK.G	Oxidation (M)	(Ions score 43)
167 - 184	517.7593	2067.0083	2066.9983	5	1	K.KYSHVDLVVMVDGFEGEK.G	Oxidation (M)	(Ions score 34)
167 - 184	690.0101	2067.0084	2066.9983	5	1	K.KYSHVDLVVMVDGFEGEK.G	Oxidation (M)	(Ions score 36)

167 - 184	517.7594	2067.0084	2066.9983	5	1	K.KYSHVDLVVMVDGFEGEK.G	Oxidation (M) (Ions score 39)
167 - 184	690.0101	2067.0084	2066.9983	5	1	K.KYSHVDLVVMVDGFEGEK.G	Oxidation (M) (Ions score 32)
167 - 184	690.0101	2067.0085	2066.9983	5	1	K.KYSHVDLVVMVDGFEGEK.G	Oxidation (M) (Ions score 41)
167 - 184	690.0101	2067.0086	2066.9983	5	1	K.KYSHVDLVVMVDGFEGEK.G	Oxidation (M) (Ions score 45)
167 - 184	698.6781	2093.0124	2093.0139	-1	1	K.KYSHVDLVVMVDGFEGEK.G	Acetylation (K) (Ions score 31)
167 - 184	698.6792	2093.0157	2093.0139	1	1	K.KYSHVDLVVMVDGFEGEK.G	Acetylation (K) (Ions score 38)
167 - 184	698.6795	2093.0165	2093.0139	1	1	K.KYSHVDLVVMVDGFEGEK.G	Acetylation (K) (Ions score 44)
167 - 184	1047.5158	2093.0170	2093.0139	1	1	K.KYSHVDLVVMVDGFEGEK.G	Acetylation (K) (Ions score 81)
167 - 184	698.6798	2093.0176	2093.0139	2	1	K.KYSHVDLVVMVDGFEGEK.G	Acetylation (K) (Ions score 33)
167 - 184	524.2618	2093.0181	2093.0139	2	1	K.KYSHVDLVVMVDGFEGEK.G	Acetylation (K) (Ions score 43)
167 - 184	698.6801	2093.0183	2093.0139	2	1	K.KYSHVDLVVMVDGFEGEK.G	Acetylation (K) (Ions score 56)
167 - 184	524.2619	2093.0185	2093.0139	2	1	K.KYSHVDLVVMVDGFEGEK.G	Acetylation (K) (Ions score 44)
167 - 184	698.6802	2093.0189	2093.0139	2	1	K.KYSHVDLVVMVDGFEGEK.G	Acetylation (K) (Ions score 60)
167 - 184	698.6803	2093.0191	2093.0139	2	1	K.KYSHVDLVVMVDGFEGEK.G	Acetylation (K) (Ions score 28)
167 - 184	698.6807	2093.0203	2093.0139	3	1	K.KYSHVDLVVMVDGFEGEK.G	Acetylation (K) (Ions score 41)
167 - 184	698.6808	2093.0207	2093.0139	3	1	K.KYSHVDLVVMVDGFEGEK.G	Acetylation (K) (Ions score 30)
167 - 184	698.6810	2093.0212	2093.0139	3	1	K.KYSHVDLVVMVDGFEGEK.G	Acetylation (K) (Ions score 42)
167 - 184	698.6811	2093.0214	2093.0139	4	1	K.KYSHVDLVVMVDGFEGEK.G	Acetylation (K) (Ions score 48)
167 - 184	698.6811	2093.0216	2093.0139	4	1	K.KYSHVDLVVMVDGFEGEK.G	Acetylation (K) (Ions score 32)
167 - 184	698.6817	2093.0233	2093.0139	4	1	K.KYSHVDLVVMVDGFEGEK.G	Acetylation (K) (Ions score 33)
167 - 184	704.0127	2109.0163	2109.0089	4	1	K.KYSHVDLVVMVDGFEGEK.G	Acetylation (K); Oxidation (M) (Ions score 52)
168 - 184	962.4578	1922.9011	1922.9084	-4	0	K.YSHVDLVVMVDGFEGEK.G	(Ions score 55)
168 - 184	962.4587	1922.9028	1922.9084	-3	0	K.YSHVDLVVMVDGFEGEK.G	(Ions score 103)
168 - 184	962.4589	1922.9032	1922.9084	-3	0	K.YSHVDLVVMVDGFEGEK.G	(Ions score 75)
168 - 184	641.9751	1922.9035	1922.9084	-3	0	K.YSHVDLVVMVDGFEGEK.G	(Ions score 41)
168 - 184	962.4592	1922.9039	1922.9084	-2	0	K.YSHVDLVVMVDGFEGEK.G	(Ions score 74)
168 - 184	962.4594	1922.9041	1922.9084	-2	0	K.YSHVDLVVMVDGFEGEK.G	(Ions score 72)
168 - 184	641.9755	1922.9046	1922.9084	-2	0	K.YSHVDLVVMVDGFEGEK.G	(Ions score 50)
168 - 184	641.9756	1922.9051	1922.9084	-2	0	K.YSHVDLVVMVDGFEGEK.G	(Ions score 26)
168 - 184	641.9757	1922.9053	1922.9084	-2	0	K.YSHVDLVVMVDGFEGEK.G	(Ions score 50)
168 - 184	962.4600	1922.9054	1922.9084	-2	0	K.YSHVDLVVMVDGFEGEK.G	(Ions score 77)
168 - 184	962.4602	1922.9058	1922.9084	-1	0	K.YSHVDLVVMVDGFEGEK.G	(Ions score 69)
168 - 184	962.4604	1922.9063	1922.9084	-1	0	K.YSHVDLVVMVDGFEGEK.G	(Ions score 92)
168 - 184	641.9761	1922.9064	1922.9084	-1	0	K.YSHVDLVVMVDGFEGEK.G	(Ions score 37)
168 - 184	962.4606	1922.9067	1922.9084	-1	0	K.YSHVDLVVMVDGFEGEK.G	(Ions score 86)
168 - 184	962.4607	1922.9068	1922.9084	-1	0	K.YSHVDLVVMVDGFEGEK.G	(Ions score 97)
168 - 184	641.9763	1922.9071	1922.9084	-1	0	K.YSHVDLVVMVDGFEGEK.G	(Ions score 43)
168 - 184	641.9763	1922.9071	1922.9084	-1	0	K.YSHVDLVVMVDGFEGEK.G	(Ions score 52)
168 - 184	962.4610	1922.9074	1922.9084	-1	0	K.YSHVDLVVMVDGFEGEK.G	(Ions score 84)
168 - 184	962.4611	1922.9076	1922.9084	-0	0	K.YSHVDLVVMVDGFEGEK.G	(Ions score 69)
168 - 184	962.4611	1922.9077	1922.9084	-0	0	K.YSHVDLVVMVDGFEGEK.G	(Ions score 83)
168 - 184	641.9766	1922.9078	1922.9084	-0	0	K.YSHVDLVVMVDGFEGEK.G	(Ions score 24)
168 - 184	962.4614	1922.9082	1922.9084	-0	0	K.YSHVDLVVMVDGFEGEK.G	(Ions score 75)
168 - 184	641.9767	1922.9082	1922.9084	-0	0	K.YSHVDLVVMVDGFEGEK.G	(Ions score 36)
168 - 184	962.4615	1922.9085	1922.9084	0	0	K.YSHVDLVVMVDGFEGEK.G	(Ions score 109)
168 - 184	962.4615	1922.9085	1922.9084	0	0	K.YSHVDLVVMVDGFEGEK.G	(Ions score 26)
168 - 184	641.9768	1922.9086	1922.9084	0	0	K.YSHVDLVVMVDGFEGEK.G	(Ions score 50)
168 - 184	962.4617	1922.9089	1922.9084	0	0	K.YSHVDLVVMVDGFEGEK.G	(Ions score 75)
168 - 184	962.4618	1922.9091	1922.9084	0	0	K.YSHVDLVVMVDGFEGEK.G	(Ions score 98)
168 - 184	641.9770	1922.9091	1922.9084	0	0	K.YSHVDLVVMVDGFEGEK.G	(Ions score 42)
168 - 184	962.4619	1922.9092	1922.9084	0	0	K.YSHVDLVVMVDGFEGEK.G	(Ions score 92)
168 - 184	641.9770	1922.9092	1922.9084	0	0	K.YSHVDLVVMVDGFEGEK.G	(Ions score 50)
168 - 184	481.7346	1922.9093	1922.9084	0	0	K.YSHVDLVVMVDGFEGEK.G	(Ions score 60)
168 - 184	962.4620	1922.9094	1922.9084	1	0	K.YSHVDLVVMVDGFEGEK.G	(Ions score 87)
168 - 184	641.9772	1922.9097	1922.9084	1	0	K.YSHVDLVVMVDGFEGEK.G	(Ions score 73)
168 - 184	641.9772	1922.9097	1922.9084	1	0	K.YSHVDLVVMVDGFEGEK.G	(Ions score 49)
168 - 184	481.7347	1922.9097	1922.9084	1	0	K.YSHVDLVVMVDGFEGEK.G	(Ions score 66)

168 - 184	641.9772	1922.9098	1922.9084	1	0	K.YSHVDLVVMVDGFEGEK.G	(Ions score 51)
168 - 184	641.9772	1922.9098	1922.9084	1	0	K.YSHVDLVVMVDGFEGEK.G	(Ions score 60)
168 - 184	481.7348	1922.9099	1922.9084	1	0	K.YSHVDLVVMVDGFEGEK.G	(Ions score 63)
168 - 184	641.9773	1922.9100	1922.9084	1	0	K.YSHVDLVVMVDGFEGEK.G	(Ions score 37)
168 - 184	641.9773	1922.9101	1922.9084	1	0	K.YSHVDLVVMVDGFEGEK.G	(Ions score 53)
168 - 184	962.4623	1922.9101	1922.9084	1	0	K.YSHVDLVVMVDGFEGEK.G	(Ions score 87)
168 - 184	641.9774	1922.9102	1922.9084	1	0	K.YSHVDLVVMVDGFEGEK.G	(Ions score 32)
168 - 184	641.9774	1922.9102	1922.9084	1	0	K.YSHVDLVVMVDGFEGEK.G	(Ions score 49)
168 - 184	481.7348	1922.9103	1922.9084	1	0	K.YSHVDLVVMVDGFEGEK.G	(Ions score 50)
168 - 184	962.4624	1922.9103	1922.9084	1	0	K.YSHVDLVVMVDGFEGEK.G	(Ions score 74)
168 - 184	962.4625	1922.9104	1922.9084	1	0	K.YSHVDLVVMVDGFEGEK.G	(Ions score 80)
168 - 184	641.9774	1922.9105	1922.9084	1	0	K.YSHVDLVVMVDGFEGEK.G	(Ions score 40)
168 - 184	962.4625	1922.9105	1922.9084	1	0	K.YSHVDLVVMVDGFEGEK.G	(Ions score 100)
168 - 184	962.4625	1922.9105	1922.9084	1	0	K.YSHVDLVVMVDGFEGEK.G	(Ions score 77)
168 - 184	962.4626	1922.9106	1922.9084	1	0	K.YSHVDLVVMVDGFEGEK.G	(Ions score 60)
168 - 184	481.7349	1922.9106	1922.9084	1	0	K.YSHVDLVVMVDGFEGEK.G	(Ions score 65)
168 - 184	481.7349	1922.9106	1922.9084	1	0	K.YSHVDLVVMVDGFEGEK.G	(Ions score 66)
168 - 184	962.4626	1922.9106	1922.9084	1	0	K.YSHVDLVVMVDGFEGEK.G	(Ions score 87)
168 - 184	641.9775	1922.9107	1922.9084	1	0	K.YSHVDLVVMVDGFEGEK.G	(Ions score 73)
168 - 184	962.4626	1922.9107	1922.9084	1	0	K.YSHVDLVVMVDGFEGEK.G	(Ions score 56)
168 - 184	962.4626	1922.9107	1922.9084	1	0	K.YSHVDLVVMVDGFEGEK.G	(Ions score 72)
168 - 184	962.4626	1922.9107	1922.9084	1	0	K.YSHVDLVVMVDGFEGEK.G	(Ions score 99)
168 - 184	641.9775	1922.9107	1922.9084	1	0	K.YSHVDLVVMVDGFEGEK.G	(Ions score 67)
168 - 184	962.4627	1922.9108	1922.9084	1	0	K.YSHVDLVVMVDGFEGEK.G	(Ions score 92)
168 - 184	962.4627	1922.9108	1922.9084	1	0	K.YSHVDLVVMVDGFEGEK.G	(Ions score 74)
168 - 184	641.9775	1922.9108	1922.9084	1	0	K.YSHVDLVVMVDGFEGEK.G	(Ions score 61)
168 - 184	641.9775	1922.9108	1922.9084	1	0	K.YSHVDLVVMVDGFEGEK.G	(Ions score 67)
168 - 184	641.9776	1922.9108	1922.9084	1	0	K.YSHVDLVVMVDGFEGEK.G	(Ions score 76)
168 - 184	641.9776	1922.9109	1922.9084	1	0	K.YSHVDLVVMVDGFEGEK.G	(Ions score 76)
168 - 184	641.9776	1922.9110	1922.9084	1	0	K.YSHVDLVVMVDGFEGEK.G	(Ions score 40)
168 - 184	962.4628	1922.9110	1922.9084	1	0	K.YSHVDLVVMVDGFEGEK.G	(Ions score 92)
168 - 184	641.9776	1922.9110	1922.9084	1	0	K.YSHVDLVVMVDGFEGEK.G	(Ions score 67)
168 - 184	962.4628	1922.9110	1922.9084	1	0	K.YSHVDLVVMVDGFEGEK.G	(Ions score 76)
168 - 184	962.4628	1922.9110	1922.9084	1	0	K.YSHVDLVVMVDGFEGEK.G	(Ions score 72)
168 - 184	962.4628	1922.9111	1922.9084	1	0	K.YSHVDLVVMVDGFEGEK.G	(Ions score 61)
168 - 184	641.9776	1922.9111	1922.9084	1	0	K.YSHVDLVVMVDGFEGEK.G	(Ions score 65)
168 - 184	962.4628	1922.9111	1922.9084	1	0	K.YSHVDLVVMVDGFEGEK.G	(Ions score 79)
168 - 184	962.4629	1922.9112	1922.9084	1	0	K.YSHVDLVVMVDGFEGEK.G	(Ions score 80)
168 - 184	641.9777	1922.9112	1922.9084	1	0	K.YSHVDLVVMVDGFEGEK.G	(Ions score 76)
168 - 184	962.4629	1922.9112	1922.9084	1	0	K.YSHVDLVVMVDGFEGEK.G	(Ions score 92)
168 - 184	641.9777	1922.9112	1922.9084	1	0	K.YSHVDLVVMVDGFEGEK.G	(Ions score 36)
168 - 184	641.9777	1922.9113	1922.9084	1	0	K.YSHVDLVVMVDGFEGEK.G	(Ions score 49)
168 - 184	641.9777	1922.9113	1922.9084	1	0	K.YSHVDLVVMVDGFEGEK.G	(Ions score 76)
168 - 184	962.4629	1922.9113	1922.9084	1	0	K.YSHVDLVVMVDGFEGEK.G	(Ions score 105)
168 - 184	962.4629	1922.9113	1922.9084	1	0	K.YSHVDLVVMVDGFEGEK.G	(Ions score 102)
168 - 184	641.9777	1922.9113	1922.9084	1	0	K.YSHVDLVVMVDGFEGEK.G	(Ions score 70)
168 - 184	962.4629	1922.9113	1922.9084	1	0	K.YSHVDLVVMVDGFEGEK.G	(Ions score 92)
168 - 184	962.4629	1922.9113	1922.9084	2	0	K.YSHVDLVVMVDGFEGEK.G	(Ions score 88)
168 - 184	641.9777	1922.9113	1922.9084	2	0	K.YSHVDLVVMVDGFEGEK.G	(Ions score 68)
168 - 184	962.4629	1922.9113	1922.9084	2	0	K.YSHVDLVVMVDGFEGEK.G	(Ions score 78)
168 - 184	641.9777	1922.9114	1922.9084	2	0	K.YSHVDLVVMVDGFEGEK.G	(Ions score 69)
168 - 184	962.4630	1922.9114	1922.9084	2	0	K.YSHVDLVVMVDGFEGEK.G	(Ions score 92)
168 - 184	641.9777	1922.9114	1922.9084	2	0	K.YSHVDLVVMVDGFEGEK.G	(Ions score 61)
168 - 184	641.9778	1922.9115	1922.9084	2	0	K.YSHVDLVVMVDGFEGEK.G	(Ions score 89)
168 - 184	641.9778	1922.9115	1922.9084	2	0	K.YSHVDLVVMVDGFEGEK.G	(Ions score 49)
168 - 184	641.9778	1922.9115	1922.9084	2	0	K.YSHVDLVVMVDGFEGEK.G	(Ions score 76)
168 - 184	641.9778	1922.9115	1922.9084	2	0	K.YSHVDLVVMVDGFEGEK.G	(Ions score 76)

168 - 184	962.4631	1922.9116	1922.9084	2	0	K.YSHVDLVVMVDGFEGEK.G	(Ions score 76)
168 - 184	962.4631	1922.9116	1922.9084	2	0	K.YSHVDLVVMVDGFEGEK.G	(Ions score 75)
168 - 184	962.4631	1922.9117	1922.9084	2	0	K.YSHVDLVVMVDGFEGEK.G	(Ions score 97)
168 - 184	962.4631	1922.9117	1922.9084	2	0	K.YSHVDLVVMVDGFEGEK.G	(Ions score 72)
168 - 184	962.4631	1922.9117	1922.9084	2	0	K.YSHVDLVVMVDGFEGEK.G	(Ions score 80)
168 - 184	641.9778	1922.9117	1922.9084	2	0	K.YSHVDLVVMVDGFEGEK.G	(Ions score 30)
168 - 184	962.4631	1922.9117	1922.9084	2	0	K.YSHVDLVVMVDGFEGEK.G	(Ions score 87)
168 - 184	962.4631	1922.9117	1922.9084	2	0	K.YSHVDLVVMVDGFEGEK.G	(Ions score 90)
168 - 184	962.4632	1922.9117	1922.9084	2	0	K.YSHVDLVVMVDGFEGEK.G	(Ions score 97)
168 - 184	962.4632	1922.9118	1922.9084	2	0	K.YSHVDLVVMVDGFEGEK.G	(Ions score 76)
168 - 184	962.4632	1922.9118	1922.9084	2	0	K.YSHVDLVVMVDGFEGEK.G	(Ions score 92)
168 - 184	641.9779	1922.9118	1922.9084	2	0	K.YSHVDLVVMVDGFEGEK.G	(Ions score 70)
168 - 184	641.9779	1922.9118	1922.9084	2	0	K.YSHVDLVVMVDGFEGEK.G	(Ions score 76)
168 - 184	962.4632	1922.9118	1922.9084	2	0	K.YSHVDLVVMVDGFEGEK.G	(Ions score 62)
168 - 184	962.4632	1922.9118	1922.9084	2	0	K.YSHVDLVVMVDGFEGEK.G	(Ions score 61)
168 - 184	962.4632	1922.9118	1922.9084	2	0	K.YSHVDLVVMVDGFEGEK.G	(Ions score 83)
168 - 184	641.9779	1922.9118	1922.9084	2	0	K.YSHVDLVVMVDGFEGEK.G	(Ions score 65)
168 - 184	641.9779	1922.9119	1922.9084	2	0	K.YSHVDLVVMVDGFEGEK.G	(Ions score 76)
168 - 184	962.4632	1922.9119	1922.9084	2	0	K.YSHVDLVVMVDGFEGEK.G	(Ions score 97)
168 - 184	962.4632	1922.9119	1922.9084	2	0	K.YSHVDLVVMVDGFEGEK.G	(Ions score 65)
168 - 184	962.4632	1922.9119	1922.9084	2	0	K.YSHVDLVVMVDGFEGEK.G	(Ions score 76)
168 - 184	641.9779	1922.9119	1922.9084	2	0	K.YSHVDLVVMVDGFEGEK.G	(Ions score 76)
168 - 184	641.9779	1922.9119	1922.9084	2	0	K.YSHVDLVVMVDGFEGEK.G	(Ions score 76)
168 - 184	641.9779	1922.9119	1922.9084	2	0	K.YSHVDLVVMVDGFEGEK.G	(Ions score 40)
168 - 184	641.9779	1922.9119	1922.9084	2	0	K.YSHVDLVVMVDGFEGEK.G	(Ions score 76)
168 - 184	962.4633	1922.9119	1922.9084	2	0	K.YSHVDLVVMVDGFEGEK.G	(Ions score 84)
168 - 184	641.9779	1922.9120	1922.9084	2	0	K.YSHVDLVVMVDGFEGEK.G	(Ions score 89)
168 - 184	641.9779	1922.9120	1922.9084	2	0	K.YSHVDLVVMVDGFEGEK.G	(Ions score 50)
168 - 184	641.9779	1922.9120	1922.9084	2	0	K.YSHVDLVVMVDGFEGEK.G	(Ions score 59)
168 - 184	641.9779	1922.9120	1922.9084	2	0	K.YSHVDLVVMVDGFEGEK.G	(Ions score 73)
168 - 184	641.9780	1922.9120	1922.9084	2	0	K.YSHVDLVVMVDGFEGEK.G	(Ions score 64)
168 - 184	641.9780	1922.9121	1922.9084	2	0	K.YSHVDLVVMVDGFEGEK.G	(Ions score 85)
168 - 184	641.9780	1922.9121	1922.9084	2	0	K.YSHVDLVVMVDGFEGEK.G	(Ions score 73)
168 - 184	641.9780	1922.9121	1922.9084	2	0	K.YSHVDLVVMVDGFEGEK.G	(Ions score 76)
168 - 184	641.9780	1922.9121	1922.9084	2	0	K.YSHVDLVVMVDGFEGEK.G	(Ions score 76)
168 - 184	641.9780	1922.9121	1922.9084	2	0	K.YSHVDLVVMVDGFEGEK.G	(Ions score 60)
168 - 184	962.4633	1922.9121	1922.9084	2	0	K.YSHVDLVVMVDGFEGEK.G	(Ions score 70)
168 - 184	641.9780	1922.9121	1922.9084	2	0	K.YSHVDLVVMVDGFEGEK.G	(Ions score 61)
168 - 184	962.4633	1922.9121	1922.9084	2	0	K.YSHVDLVVMVDGFEGEK.G	(Ions score 70)
168 - 184	641.9780	1922.9122	1922.9084	2	0	K.YSHVDLVVMVDGFEGEK.G	(Ions score 70)
168 - 184	962.4634	1922.9122	1922.9084	2	0	K.YSHVDLVVMVDGFEGEK.G	(Ions score 92)
168 - 184	962.4634	1922.9122	1922.9084	2	0	K.YSHVDLVVMVDGFEGEK.G	(Ions score 87)
168 - 184	962.4634	1922.9122	1922.9084	2	0	K.YSHVDLVVMVDGFEGEK.G	(Ions score 92)
168 - 184	962.4634	1922.9122	1922.9084	2	0	K.YSHVDLVVMVDGFEGEK.G	(Ions score 84)
168 - 184	962.4634	1922.9122	1922.9084	2	0	K.YSHVDLVVMVDGFEGEK.G	(Ions score 76)
168 - 184	962.4634	1922.9122	1922.9084	2	0	K.YSHVDLVVMVDGFEGEK.G	(Ions score 92)
168 - 184	641.9780	1922.9122	1922.9084	2	0	K.YSHVDLVVMVDGFEGEK.G	(Ions score 76)
168 - 184	641.9780	1922.9122	1922.9084	2	0	K.YSHVDLVVMVDGFEGEK.G	(Ions score 77)
168 - 184	641.9780	1922.9122	1922.9084	2	0	K.YSHVDLVVMVDGFEGEK.G	(Ions score 64)
168 - 184	962.4634	1922.9123	1922.9084	2	0	K.YSHVDLVVMVDGFEGEK.G	(Ions score 77)
168 - 184	641.9780	1922.9123	1922.9084	2	0	K.YSHVDLVVMVDGFEGEK.G	(Ions score 73)
168 - 184	962.4634	1922.9123	1922.9084	2	0	K.YSHVDLVVMVDGFEGEK.G	(Ions score 71)
168 - 184	641.9780	1922.9123	1922.9084	2	0	K.YSHVDLVVMVDGFEGEK.G	(Ions score 72)
168 - 184	641.9780	1922.9123	1922.9084	2	0	K.YSHVDLVVMVDGFEGEK.G	(Ions score 73)
168 - 184	641.9780	1922.9123	1922.9084	2	0	K.YSHVDLVVMVDGFEGEK.G	(Ions score 89)
168 - 184	641.9780	1922.9123	1922.9084	2	0	K.YSHVDLVVMVDGFEGEK.G	(Ions score 63)
168 - 184	641.9780	1922.9123	1922.9084	2	0	K.YSHVDLVVMVDGFEGEK.G	(Ions score 69)

168 - 184	962.4634	1922.9123	1922.9084	2	0	K.YSHVDLVVMVDGFEGEK.G	(Ions score 97)
168 - 184	962.4635	1922.9124	1922.9084	2	0	K.YSHVDLVVMVDGFEGEK.G	(Ions score 76)
168 - 184	962.4635	1922.9124	1922.9084	2	0	K.YSHVDLVVMVDGFEGEK.G	(Ions score 88)
168 - 184	962.4635	1922.9124	1922.9084	2	0	K.YSHVDLVVMVDGFEGEK.G	(Ions score 86)
168 - 184	641.9781	1922.9124	1922.9084	2	0	K.YSHVDLVVMVDGFEGEK.G	(Ions score 64)
168 - 184	641.9781	1922.9124	1922.9084	2	0	K.YSHVDLVVMVDGFEGEK.G	(Ions score 73)
168 - 184	641.9781	1922.9124	1922.9084	2	0	K.YSHVDLVVMVDGFEGEK.G	(Ions score 76)
168 - 184	641.9781	1922.9124	1922.9084	2	0	K.YSHVDLVVMVDGFEGEK.G	(Ions score 76)
168 - 184	641.9781	1922.9124	1922.9084	2	0	K.YSHVDLVVMVDGFEGEK.G	(Ions score 76)
168 - 184	962.4635	1922.9124	1922.9084	2	0	K.YSHVDLVVMVDGFEGEK.G	(Ions score 75)
168 - 184	641.9781	1922.9124	1922.9084	2	0	K.YSHVDLVVMVDGFEGEK.G	(Ions score 68)
168 - 184	962.4635	1922.9124	1922.9084	2	0	K.YSHVDLVVMVDGFEGEK.G	(Ions score 81)
168 - 184	962.4635	1922.9124	1922.9084	2	0	K.YSHVDLVVMVDGFEGEK.G	(Ions score 86)
168 - 184	962.4635	1922.9125	1922.9084	2	0	K.YSHVDLVVMVDGFEGEK.G	(Ions score 88)
168 - 184	962.4635	1922.9125	1922.9084	2	0	K.YSHVDLVVMVDGFEGEK.G	(Ions score 88)
168 - 184	641.9781	1922.9125	1922.9084	2	0	K.YSHVDLVVMVDGFEGEK.G	(Ions score 78)
168 - 184	962.4635	1922.9125	1922.9084	2	0	K.YSHVDLVVMVDGFEGEK.G	(Ions score 79)
168 - 184	962.4635	1922.9125	1922.9084	2	0	K.YSHVDLVVMVDGFEGEK.G	(Ions score 72)
168 - 184	641.9781	1922.9125	1922.9084	2	0	K.YSHVDLVVMVDGFEGEK.G	(Ions score 73)
168 - 184	641.9781	1922.9125	1922.9084	2	0	K.YSHVDLVVMVDGFEGEK.G	(Ions score 76)
168 - 184	481.7354	1922.9125	1922.9084	2	0	K.YSHVDLVVMVDGFEGEK.G	(Ions score 58)
168 - 184	641.9781	1922.9126	1922.9084	2	0	K.YSHVDLVVMVDGFEGEK.G	(Ions score 73)
168 - 184	641.9781	1922.9126	1922.9084	2	0	K.YSHVDLVVMVDGFEGEK.G	(Ions score 62)
168 - 184	962.4636	1922.9126	1922.9084	2	0	K.YSHVDLVVMVDGFEGEK.G	(Ions score 97)
168 - 184	641.9781	1922.9126	1922.9084	2	0	K.YSHVDLVVMVDGFEGEK.G	(Ions score 55)
168 - 184	962.4636	1922.9126	1922.9084	2	0	K.YSHVDLVVMVDGFEGEK.G	(Ions score 75)
168 - 184	641.9781	1922.9126	1922.9084	2	0	K.YSHVDLVVMVDGFEGEK.G	(Ions score 76)
168 - 184	641.9781	1922.9126	1922.9084	2	0	K.YSHVDLVVMVDGFEGEK.G	(Ions score 69)
168 - 184	641.9782	1922.9126	1922.9084	2	0	K.YSHVDLVVMVDGFEGEK.G	(Ions score 67)
168 - 184	962.4636	1922.9126	1922.9084	2	0	K.YSHVDLVVMVDGFEGEK.G	(Ions score 75)
168 - 184	962.4636	1922.9126	1922.9084	2	0	K.YSHVDLVVMVDGFEGEK.G	(Ions score 87)
168 - 184	641.9782	1922.9126	1922.9084	2	0	K.YSHVDLVVMVDGFEGEK.G	(Ions score 73)
168 - 184	641.9782	1922.9127	1922.9084	2	0	K.YSHVDLVVMVDGFEGEK.G	(Ions score 76)
168 - 184	962.4636	1922.9127	1922.9084	2	0	K.YSHVDLVVMVDGFEGEK.G	(Ions score 88)
168 - 184	962.4636	1922.9127	1922.9084	2	0	K.YSHVDLVVMVDGFEGEK.G	(Ions score 72)
168 - 184	962.4636	1922.9127	1922.9084	2	0	K.YSHVDLVVMVDGFEGEK.G	(Ions score 87)
168 - 184	962.4636	1922.9127	1922.9084	2	0	K.YSHVDLVVMVDGFEGEK.G	(Ions score 88)
168 - 184	962.4636	1922.9127	1922.9084	2	0	K.YSHVDLVVMVDGFEGEK.G	(Ions score 80)
168 - 184	641.9782	1922.9127	1922.9084	2	0	K.YSHVDLVVMVDGFEGEK.G	(Ions score 70)
168 - 184	641.9782	1922.9127	1922.9084	2	0	K.YSHVDLVVMVDGFEGEK.G	(Ions score 76)
168 - 184	641.9782	1922.9127	1922.9084	2	0	K.YSHVDLVVMVDGFEGEK.G	(Ions score 76)
168 - 184	641.9782	1922.9128	1922.9084	2	0	K.YSHVDLVVMVDGFEGEK.G	(Ions score 88)
168 - 184	962.4637	1922.9128	1922.9084	2	0	K.YSHVDLVVMVDGFEGEK.G	(Ions score 75)
168 - 184	641.9782	1922.9128	1922.9084	2	0	K.YSHVDLVVMVDGFEGEK.G	(Ions score 80)
168 - 184	641.9782	1922.9128	1922.9084	2	0	K.YSHVDLVVMVDGFEGEK.G	(Ions score 76)
168 - 184	641.9782	1922.9128	1922.9084	2	0	K.YSHVDLVVMVDGFEGEK.G	(Ions score 76)
168 - 184	641.9782	1922.9128	1922.9084	2	0	K.YSHVDLVVMVDGFEGEK.G	(Ions score 73)
168 - 184	962.4637	1922.9128	1922.9084	2	0	K.YSHVDLVVMVDGFEGEK.G	(Ions score 85)
168 - 184	962.4637	1922.9128	1922.9084	2	0	K.YSHVDLVVMVDGFEGEK.G	(Ions score 88)
168 - 184	641.9782	1922.9129	1922.9084	2	0	K.YSHVDLVVMVDGFEGEK.G	(Ions score 56)
168 - 184	641.9782	1922.9129	1922.9084	2	0	K.YSHVDLVVMVDGFEGEK.G	(Ions score 43)
168 - 184	962.4637	1922.9129	1922.9084	2	0	K.YSHVDLVVMVDGFEGEK.G	(Ions score 75)
168 - 184	641.9782	1922.9129	1922.9084	2	0	K.YSHVDLVVMVDGFEGEK.G	(Ions score 73)
168 - 184	962.4637	1922.9129	1922.9084	2	0	K.YSHVDLVVMVDGFEGEK.G	(Ions score 90)
168 - 184	962.4637	1922.9129	1922.9084	2	0	K.YSHVDLVVMVDGFEGEK.G	(Ions score 72)
168 - 184	641.9782	1922.9129	1922.9084	2	0	K.YSHVDLVVMVDGFEGEK.G	(Ions score 73)
168 - 184	962.4637	1922.9129	1922.9084	2	0	K.YSHVDLVVMVDGFEGEK.G	(Ions score 90)

168 - 184	962.4637	1922.9129	1922.9084	2	0	K.YSHVDLVVMVDGFEGEK.G	(Ions score 85)
168 - 184	641.9782	1922.9129	1922.9084	2	0	K.YSHVDLVVMVDGFEGEK.G	(Ions score 64)
168 - 184	641.9783	1922.9129	1922.9084	2	0	K.YSHVDLVVMVDGFEGEK.G	(Ions score 71)
168 - 184	962.4637	1922.9129	1922.9084	2	0	K.YSHVDLVVMVDGFEGEK.G	(Ions score 91)
168 - 184	962.4638	1922.9130	1922.9084	2	0	K.YSHVDLVVMVDGFEGEK.G	(Ions score 87)
168 - 184	641.9783	1922.9130	1922.9084	2	0	K.YSHVDLVVMVDGFEGEK.G	(Ions score 76)
168 - 184	641.9783	1922.9130	1922.9084	2	0	K.YSHVDLVVMVDGFEGEK.G	(Ions score 93)
168 - 184	962.4638	1922.9130	1922.9084	2	0	K.YSHVDLVVMVDGFEGEK.G	(Ions score 99)
168 - 184	641.9783	1922.9130	1922.9084	2	0	K.YSHVDLVVMVDGFEGEK.G	(Ions score 76)
168 - 184	641.9783	1922.9130	1922.9084	2	0	K.YSHVDLVVMVDGFEGEK.G	(Ions score 68)
168 - 184	641.9783	1922.9130	1922.9084	2	0	K.YSHVDLVVMVDGFEGEK.G	(Ions score 73)
168 - 184	641.9783	1922.9130	1922.9084	2	0	K.YSHVDLVVMVDGFEGEK.G	(Ions score 76)
168 - 184	641.9783	1922.9130	1922.9084	2	0	K.YSHVDLVVMVDGFEGEK.G	(Ions score 73)
168 - 184	641.9783	1922.9130	1922.9084	2	0	K.YSHVDLVVMVDGFEGEK.G	(Ions score 76)
168 - 184	962.4638	1922.9130	1922.9084	2	0	K.YSHVDLVVMVDGFEGEK.G	(Ions score 71)
168 - 184	641.9783	1922.9131	1922.9084	2	0	K.YSHVDLVVMVDGFEGEK.G	(Ions score 73)
168 - 184	962.4638	1922.9131	1922.9084	2	0	K.YSHVDLVVMVDGFEGEK.G	(Ions score 75)
168 - 184	962.4638	1922.9131	1922.9084	2	0	K.YSHVDLVVMVDGFEGEK.G	(Ions score 88)
168 - 184	641.9783	1922.9131	1922.9084	2	0	K.YSHVDLVVMVDGFEGEK.G	(Ions score 70)
168 - 184	962.4638	1922.9131	1922.9084	2	0	K.YSHVDLVVMVDGFEGEK.G	(Ions score 91)
168 - 184	641.9783	1922.9131	1922.9084	2	0	K.YSHVDLVVMVDGFEGEK.G	(Ions score 69)
168 - 184	641.9783	1922.9131	1922.9084	2	0	K.YSHVDLVVMVDGFEGEK.G	(Ions score 76)
168 - 184	641.9783	1922.9131	1922.9084	2	0	K.YSHVDLVVMVDGFEGEK.G	(Ions score 76)
168 - 184	962.4638	1922.9131	1922.9084	2	0	K.YSHVDLVVMVDGFEGEK.G	(Ions score 79)
168 - 184	641.9783	1922.9131	1922.9084	2	0	K.YSHVDLVVMVDGFEGEK.G	(Ions score 76)
168 - 184	641.9783	1922.9131	1922.9084	2	0	K.YSHVDLVVMVDGFEGEK.G	(Ions score 76)
168 - 184	962.4638	1922.9131	1922.9084	2	0	K.YSHVDLVVMVDGFEGEK.G	(Ions score 71)
168 - 184	641.9783	1922.9131	1922.9084	2	0	K.YSHVDLVVMVDGFEGEK.G	(Ions score 76)
168 - 184	962.4639	1922.9132	1922.9084	2	0	K.YSHVDLVVMVDGFEGEK.G	(Ions score 90)
168 - 184	641.9783	1922.9132	1922.9084	2	0	K.YSHVDLVVMVDGFEGEK.G	(Ions score 70)
168 - 184	641.9783	1922.9132	1922.9084	2	0	K.YSHVDLVVMVDGFEGEK.G	(Ions score 73)
168 - 184	641.9783	1922.9132	1922.9084	2	0	K.YSHVDLVVMVDGFEGEK.G	(Ions score 73)
168 - 184	641.9783	1922.9132	1922.9084	2	0	K.YSHVDLVVMVDGFEGEK.G	(Ions score 76)
168 - 184	641.9783	1922.9132	1922.9084	2	0	K.YSHVDLVVMVDGFEGEK.G	(Ions score 76)
168 - 184	641.9783	1922.9132	1922.9084	2	0	K.YSHVDLVVMVDGFEGEK.G	(Ions score 61)
168 - 184	641.9784	1922.9132	1922.9084	2	0	K.YSHVDLVVMVDGFEGEK.G	(Ions score 76)
168 - 184	962.4639	1922.9132	1922.9084	3	0	K.YSHVDLVVMVDGFEGEK.G	(Ions score 80)
168 - 184	962.4639	1922.9133	1922.9084	3	0	K.YSHVDLVVMVDGFEGEK.G	(Ions score 79)
168 - 184	641.9784	1922.9133	1922.9084	3	0	K.YSHVDLVVMVDGFEGEK.G	(Ions score 35)
168 - 184	962.4639	1922.9133	1922.9084	3	0	K.YSHVDLVVMVDGFEGEK.G	(Ions score 92)
168 - 184	962.4639	1922.9133	1922.9084	3	0	K.YSHVDLVVMVDGFEGEK.G	(Ions score 73)
168 - 184	962.4639	1922.9133	1922.9084	3	0	K.YSHVDLVVMVDGFEGEK.G	(Ions score 83)
168 - 184	641.9784	1922.9133	1922.9084	3	0	K.YSHVDLVVMVDGFEGEK.G	(Ions score 49)
168 - 184	641.9784	1922.9134	1922.9084	3	0	K.YSHVDLVVMVDGFEGEK.G	(Ions score 45)
168 - 184	641.9784	1922.9134	1922.9084	3	0	K.YSHVDLVVMVDGFEGEK.G	(Ions score 76)
168 - 184	481.7356	1922.9134	1922.9084	3	0	K.YSHVDLVVMVDGFEGEK.G	(Ions score 43)
168 - 184	641.9784	1922.9134	1922.9084	3	0	K.YSHVDLVVMVDGFEGEK.G	(Ions score 66)
168 - 184	962.4640	1922.9134	1922.9084	3	0	K.YSHVDLVVMVDGFEGEK.G	(Ions score 38)
168 - 184	962.4640	1922.9134	1922.9084	3	0	K.YSHVDLVVMVDGFEGEK.G	(Ions score 85)
168 - 184	641.9784	1922.9134	1922.9084	3	0	K.YSHVDLVVMVDGFEGEK.G	(Ions score 76)
168 - 184	962.4640	1922.9134	1922.9084	3	0	K.YSHVDLVVMVDGFEGEK.G	(Ions score 75)
168 - 184	641.9784	1922.9134	1922.9084	3	0	K.YSHVDLVVMVDGFEGEK.G	(Ions score 76)
168 - 184	641.9784	1922.9134	1922.9084	3	0	K.YSHVDLVVMVDGFEGEK.G	(Ions score 66)
168 - 184	641.9784	1922.9134	1922.9084	3	0	K.YSHVDLVVMVDGFEGEK.G	(Ions score 73)
168 - 184	962.4640	1922.9135	1922.9084	3	0	K.YSHVDLVVMVDGFEGEK.G	(Ions score 71)
168 - 184	641.9784	1922.9135	1922.9084	3	0	K.YSHVDLVVMVDGFEGEK.G	(Ions score 76)
168 - 184	641.9784	1922.9135	1922.9084	3	0	K.YSHVDLVVMVDGFEGEK.G	(Ions score 66)

168 - 184	641.9784	1922.9135	1922.9084	3	0	K.YSHVDLVVMVDGFEGEK.G	(Ions score 76)
168 - 184	641.9784	1922.9135	1922.9084	3	0	K.YSHVDLVVMVDGFEGEK.G	(Ions score 66)
168 - 184	641.9784	1922.9135	1922.9084	3	0	K.YSHVDLVVMVDGFEGEK.G	(Ions score 70)
168 - 184	962.4640	1922.9135	1922.9084	3	0	K.YSHVDLVVMVDGFEGEK.G	(Ions score 71)
168 - 184	962.4640	1922.9135	1922.9084	3	0	K.YSHVDLVVMVDGFEGEK.G	(Ions score 79)
168 - 184	481.7357	1922.9135	1922.9084	3	0	K.YSHVDLVVMVDGFEGEK.G	(Ions score 66)
168 - 184	962.4640	1922.9135	1922.9084	3	0	K.YSHVDLVVMVDGFEGEK.G	(Ions score 108)
168 - 184	641.9785	1922.9136	1922.9084	3	0	K.YSHVDLVVMVDGFEGEK.G	(Ions score 53)
168 - 184	962.4641	1922.9136	1922.9084	3	0	K.YSHVDLVVMVDGFEGEK.G	(Ions score 92)
168 - 184	962.4641	1922.9136	1922.9084	3	0	K.YSHVDLVVMVDGFEGEK.G	(Ions score 87)
168 - 184	641.9785	1922.9136	1922.9084	3	0	K.YSHVDLVVMVDGFEGEK.G	(Ions score 76)
168 - 184	641.9785	1922.9136	1922.9084	3	0	K.YSHVDLVVMVDGFEGEK.G	(Ions score 76)
168 - 184	962.4641	1922.9136	1922.9084	3	0	K.YSHVDLVVMVDGFEGEK.G	(Ions score 79)
168 - 184	962.4641	1922.9136	1922.9084	3	0	K.YSHVDLVVMVDGFEGEK.G	(Ions score 92)
168 - 184	641.9785	1922.9136	1922.9084	3	0	K.YSHVDLVVMVDGFEGEK.G	(Ions score 66)
168 - 184	641.9785	1922.9136	1922.9084	3	0	K.YSHVDLVVMVDGFEGEK.G	(Ions score 60)
168 - 184	962.4641	1922.9136	1922.9084	3	0	K.YSHVDLVVMVDGFEGEK.G	(Ions score 97)
168 - 184	962.4641	1922.9136	1922.9084	3	0	K.YSHVDLVVMVDGFEGEK.G	(Ions score 92)
168 - 184	962.4641	1922.9136	1922.9084	3	0	K.YSHVDLVVMVDGFEGEK.G	(Ions score 87)
168 - 184	641.9785	1922.9136	1922.9084	3	0	K.YSHVDLVVMVDGFEGEK.G	(Ions score 76)
168 - 184	962.4641	1922.9136	1922.9084	3	0	K.YSHVDLVVMVDGFEGEK.G	(Ions score 75)
168 - 184	641.9785	1922.9137	1922.9084	3	0	K.YSHVDLVVMVDGFEGEK.G	(Ions score 76)
168 - 184	962.4641	1922.9137	1922.9084	3	0	K.YSHVDLVVMVDGFEGEK.G	(Ions score 76)
168 - 184	962.4641	1922.9137	1922.9084	3	0	K.YSHVDLVVMVDGFEGEK.G	(Ions score 74)
168 - 184	641.9785	1922.9137	1922.9084	3	0	K.YSHVDLVVMVDGFEGEK.G	(Ions score 76)
168 - 184	962.4641	1922.9137	1922.9084	3	0	K.YSHVDLVVMVDGFEGEK.G	(Ions score 72)
168 - 184	641.9785	1922.9137	1922.9084	3	0	K.YSHVDLVVMVDGFEGEK.G	(Ions score 73)
168 - 184	641.9785	1922.9137	1922.9084	3	0	K.YSHVDLVVMVDGFEGEK.G	(Ions score 73)
168 - 184	962.4641	1922.9137	1922.9084	3	0	K.YSHVDLVVMVDGFEGEK.G	(Ions score 75)
168 - 184	641.9785	1922.9137	1922.9084	3	0	K.YSHVDLVVMVDGFEGEK.G	(Ions score 68)
168 - 184	641.9785	1922.9137	1922.9084	3	0	K.YSHVDLVVMVDGFEGEK.G	(Ions score 70)
168 - 184	641.9785	1922.9137	1922.9084	3	0	K.YSHVDLVVMVDGFEGEK.G	(Ions score 73)
168 - 184	962.4641	1922.9137	1922.9084	3	0	K.YSHVDLVVMVDGFEGEK.G	(Ions score 64)
168 - 184	641.9785	1922.9138	1922.9084	3	0	K.YSHVDLVVMVDGFEGEK.G	(Ions score 73)
168 - 184	962.4642	1922.9138	1922.9084	3	0	K.YSHVDLVVMVDGFEGEK.G	(Ions score 92)
168 - 184	962.4642	1922.9138	1922.9084	3	0	K.YSHVDLVVMVDGFEGEK.G	(Ions score 75)
168 - 184	641.9785	1922.9138	1922.9084	3	0	K.YSHVDLVVMVDGFEGEK.G	(Ions score 76)
168 - 184	962.4642	1922.9138	1922.9084	3	0	K.YSHVDLVVMVDGFEGEK.G	(Ions score 72)
168 - 184	641.9785	1922.9138	1922.9084	3	0	K.YSHVDLVVMVDGFEGEK.G	(Ions score 69)
168 - 184	641.9785	1922.9138	1922.9084	3	0	K.YSHVDLVVMVDGFEGEK.G	(Ions score 76)
168 - 184	641.9785	1922.9138	1922.9084	3	0	K.YSHVDLVVMVDGFEGEK.G	(Ions score 62)
168 - 184	641.9786	1922.9138	1922.9084	3	0	K.YSHVDLVVMVDGFEGEK.G	(Ions score 76)
168 - 184	962.4642	1922.9138	1922.9084	3	0	K.YSHVDLVVMVDGFEGEK.G	(Ions score 80)
168 - 184	641.9786	1922.9138	1922.9084	3	0	K.YSHVDLVVMVDGFEGEK.G	(Ions score 76)
168 - 184	962.4642	1922.9138	1922.9084	3	0	K.YSHVDLVVMVDGFEGEK.G	(Ions score 79)
168 - 184	641.9786	1922.9138	1922.9084	3	0	K.YSHVDLVVMVDGFEGEK.G	(Ions score 53)
168 - 184	962.4642	1922.9138	1922.9084	3	0	K.YSHVDLVVMVDGFEGEK.G	(Ions score 92)
168 - 184	962.4642	1922.9139	1922.9084	3	0	K.YSHVDLVVMVDGFEGEK.G	(Ions score 75)
168 - 184	641.9786	1922.9139	1922.9084	3	0	K.YSHVDLVVMVDGFEGEK.G	(Ions score 73)
168 - 184	641.9786	1922.9139	1922.9084	3	0	K.YSHVDLVVMVDGFEGEK.G	(Ions score 76)
168 - 184	641.9786	1922.9139	1922.9084	3	0	K.YSHVDLVVMVDGFEGEK.G	(Ions score 73)
168 - 184	641.9786	1922.9139	1922.9084	3	0	K.YSHVDLVVMVDGFEGEK.G	(Ions score 65)
168 - 184	962.4642	1922.9139	1922.9084	3	0	K.YSHVDLVVMVDGFEGEK.G	(Ions score 92)
168 - 184	641.9786	1922.9139	1922.9084	3	0	K.YSHVDLVVMVDGFEGEK.G	(Ions score 76)
168 - 184	641.9786	1922.9139	1922.9084	3	0	K.YSHVDLVVMVDGFEGEK.G	(Ions score 76)
168 - 184	962.4643	1922.9140	1922.9084	3	0	K.YSHVDLVVMVDGFEGEK.G	(Ions score 76)
168 - 184	962.4643	1922.9140	1922.9084	3	0	K.YSHVDLVVMVDGFEGEK.G	(Ions score 87)

168 - 184	962.4643	1922.9140	1922.9084	3	0	K.YSHVDLVVMVDGFEGEK.G	(Ions score 97)
168 - 184	962.4643	1922.9140	1922.9084	3	0	K.YSHVDLVVMVDGFEGEK.G	(Ions score 105)
168 - 184	641.9786	1922.9140	1922.9084	3	0	K.YSHVDLVVMVDGFEGEK.G	(Ions score 76)
168 - 184	641.9786	1922.9140	1922.9084	3	0	K.YSHVDLVVMVDGFEGEK.G	(Ions score 73)
168 - 184	641.9786	1922.9140	1922.9084	3	0	K.YSHVDLVVMVDGFEGEK.G	(Ions score 73)
168 - 184	962.4643	1922.9140	1922.9084	3	0	K.YSHVDLVVMVDGFEGEK.G	(Ions score 89)
168 - 184	641.9786	1922.9140	1922.9084	3	0	K.YSHVDLVVMVDGFEGEK.G	(Ions score 70)
168 - 184	962.4643	1922.9141	1922.9084	3	0	K.YSHVDLVVMVDGFEGEK.G	(Ions score 93)
168 - 184	962.4643	1922.9141	1922.9084	3	0	K.YSHVDLVVMVDGFEGEK.G	(Ions score 76)
168 - 184	962.4643	1922.9141	1922.9084	3	0	K.YSHVDLVVMVDGFEGEK.G	(Ions score 75)
168 - 184	641.9786	1922.9141	1922.9084	3	0	K.YSHVDLVVMVDGFEGEK.G	(Ions score 63)
168 - 184	641.9786	1922.9141	1922.9084	3	0	K.YSHVDLVVMVDGFEGEK.G	(Ions score 43)
168 - 184	641.9786	1922.9141	1922.9084	3	0	K.YSHVDLVVMVDGFEGEK.G	(Ions score 76)
168 - 184	962.4643	1922.9141	1922.9084	3	0	K.YSHVDLVVMVDGFEGEK.G	(Ions score 76)
168 - 184	962.4643	1922.9141	1922.9084	3	0	K.YSHVDLVVMVDGFEGEK.G	(Ions score 88)
168 - 184	641.9786	1922.9141	1922.9084	3	0	K.YSHVDLVVMVDGFEGEK.G	(Ions score 75)
168 - 184	962.4643	1922.9141	1922.9084	3	0	K.YSHVDLVVMVDGFEGEK.G	(Ions score 90)
168 - 184	962.4643	1922.9141	1922.9084	3	0	K.YSHVDLVVMVDGFEGEK.G	(Ions score 75)
168 - 184	641.9787	1922.9142	1922.9084	3	0	K.YSHVDLVVMVDGFEGEK.G	(Ions score 70)
168 - 184	641.9787	1922.9142	1922.9084	3	0	K.YSHVDLVVMVDGFEGEK.G	(Ions score 79)
168 - 184	962.4644	1922.9142	1922.9084	3	0	K.YSHVDLVVMVDGFEGEK.G	(Ions score 75)
168 - 184	641.9787	1922.9142	1922.9084	3	0	K.YSHVDLVVMVDGFEGEK.G	(Ions score 76)
168 - 184	962.4644	1922.9142	1922.9084	3	0	K.YSHVDLVVMVDGFEGEK.G	(Ions score 92)
168 - 184	641.9787	1922.9142	1922.9084	3	0	K.YSHVDLVVMVDGFEGEK.G	(Ions score 76)
168 - 184	962.4644	1922.9142	1922.9084	3	0	K.YSHVDLVVMVDGFEGEK.G	(Ions score 88)
168 - 184	962.4644	1922.9142	1922.9084	3	0	K.YSHVDLVVMVDGFEGEK.G	(Ions score 76)
168 - 184	641.9787	1922.9142	1922.9084	3	0	K.YSHVDLVVMVDGFEGEK.G	(Ions score 77)
168 - 184	962.4644	1922.9142	1922.9084	3	0	K.YSHVDLVVMVDGFEGEK.G	(Ions score 75)
168 - 184	641.9787	1922.9142	1922.9084	3	0	K.YSHVDLVVMVDGFEGEK.G	(Ions score 68)
168 - 184	962.4644	1922.9142	1922.9084	3	0	K.YSHVDLVVMVDGFEGEK.G	(Ions score 80)
168 - 184	962.4644	1922.9143	1922.9084	3	0	K.YSHVDLVVMVDGFEGEK.G	(Ions score 76)
168 - 184	641.9787	1922.9143	1922.9084	3	0	K.YSHVDLVVMVDGFEGEK.G	(Ions score 75)
168 - 184	962.4644	1922.9143	1922.9084	3	0	K.YSHVDLVVMVDGFEGEK.G	(Ions score 80)
168 - 184	641.9787	1922.9143	1922.9084	3	0	K.YSHVDLVVMVDGFEGEK.G	(Ions score 76)
168 - 184	641.9787	1922.9143	1922.9084	3	0	K.YSHVDLVVMVDGFEGEK.G	(Ions score 73)
168 - 184	962.4644	1922.9143	1922.9084	3	0	K.YSHVDLVVMVDGFEGEK.G	(Ions score 79)
168 - 184	962.4644	1922.9143	1922.9084	3	0	K.YSHVDLVVMVDGFEGEK.G	(Ions score 79)
168 - 184	641.9787	1922.9143	1922.9084	3	0	K.YSHVDLVVMVDGFEGEK.G	(Ions score 80)
168 - 184	641.9787	1922.9143	1922.9084	3	0	K.YSHVDLVVMVDGFEGEK.G	(Ions score 76)
168 - 184	962.4644	1922.9143	1922.9084	3	0	K.YSHVDLVVMVDGFEGEK.G	(Ions score 92)
168 - 184	962.4645	1922.9144	1922.9084	3	0	K.YSHVDLVVMVDGFEGEK.G	(Ions score 90)
168 - 184	962.4645	1922.9144	1922.9084	3	0	K.YSHVDLVVMVDGFEGEK.G	(Ions score 61)
168 - 184	962.4645	1922.9144	1922.9084	3	0	K.YSHVDLVVMVDGFEGEK.G	(Ions score 76)
168 - 184	962.4645	1922.9144	1922.9084	3	0	K.YSHVDLVVMVDGFEGEK.G	(Ions score 64)
168 - 184	641.9787	1922.9144	1922.9084	3	0	K.YSHVDLVVMVDGFEGEK.G	(Ions score 76)
168 - 184	641.9788	1922.9144	1922.9084	3	0	K.YSHVDLVVMVDGFEGEK.G	(Ions score 66)
168 - 184	641.9788	1922.9145	1922.9084	3	0	K.YSHVDLVVMVDGFEGEK.G	(Ions score 76)
168 - 184	962.4645	1922.9145	1922.9084	3	0	K.YSHVDLVVMVDGFEGEK.G	(Ions score 92)
168 - 184	641.9788	1922.9145	1922.9084	3	0	K.YSHVDLVVMVDGFEGEK.G	(Ions score 57)
168 - 184	641.9788	1922.9145	1922.9084	3	0	K.YSHVDLVVMVDGFEGEK.G	(Ions score 76)
168 - 184	641.9788	1922.9145	1922.9084	3	0	K.YSHVDLVVMVDGFEGEK.G	(Ions score 65)
168 - 184	641.9788	1922.9145	1922.9084	3	0	K.YSHVDLVVMVDGFEGEK.G	(Ions score 73)
168 - 184	962.4645	1922.9145	1922.9084	3	0	K.YSHVDLVVMVDGFEGEK.G	(Ions score 92)
168 - 184	962.4645	1922.9145	1922.9084	3	0	K.YSHVDLVVMVDGFEGEK.G	(Ions score 92)
168 - 184	962.4646	1922.9145	1922.9084	3	0	K.YSHVDLVVMVDGFEGEK.G	(Ions score 87)
168 - 184	641.9788	1922.9146	1922.9084	3	0	K.YSHVDLVVMVDGFEGEK.G	(Ions score 69)
168 - 184	641.9788	1922.9146	1922.9084	3	0	K.YSHVDLVVMVDGFEGEK.G	(Ions score 73)

168 - 184	641.9788	1922.9146	1922.9084	3	0	K.YSHVDLVVMVDGFEGEK.G	(Ions score 89)
168 - 184	641.9788	1922.9146	1922.9084	3	0	K.YSHVDLVVMVDGFEGEK.G	(Ions score 62)
168 - 184	641.9788	1922.9146	1922.9084	3	0	K.YSHVDLVVMVDGFEGEK.G	(Ions score 64)
168 - 184	641.9788	1922.9146	1922.9084	3	0	K.YSHVDLVVMVDGFEGEK.G	(Ions score 70)
168 - 184	962.4646	1922.9146	1922.9084	3	0	K.YSHVDLVVMVDGFEGEK.G	(Ions score 79)
168 - 184	641.9788	1922.9146	1922.9084	3	0	K.YSHVDLVVMVDGFEGEK.G	(Ions score 73)
168 - 184	641.9788	1922.9146	1922.9084	3	0	K.YSHVDLVVMVDGFEGEK.G	(Ions score 64)
168 - 184	962.4646	1922.9147	1922.9084	3	0	K.YSHVDLVVMVDGFEGEK.G	(Ions score 87)
168 - 184	962.4646	1922.9147	1922.9084	3	0	K.YSHVDLVVMVDGFEGEK.G	(Ions score 77)
168 - 184	641.9788	1922.9147	1922.9084	3	0	K.YSHVDLVVMVDGFEGEK.G	(Ions score 89)
168 - 184	962.4646	1922.9147	1922.9084	3	0	K.YSHVDLVVMVDGFEGEK.G	(Ions score 88)
168 - 184	962.4646	1922.9147	1922.9084	3	0	K.YSHVDLVVMVDGFEGEK.G	(Ions score 92)
168 - 184	641.9788	1922.9147	1922.9084	3	0	K.YSHVDLVVMVDGFEGEK.G	(Ions score 76)
168 - 184	962.4646	1922.9147	1922.9084	3	0	K.YSHVDLVVMVDGFEGEK.G	(Ions score 74)
168 - 184	962.4646	1922.9147	1922.9084	3	0	K.YSHVDLVVMVDGFEGEK.G	(Ions score 79)
168 - 184	641.9788	1922.9147	1922.9084	3	0	K.YSHVDLVVMVDGFEGEK.G	(Ions score 63)
168 - 184	962.4646	1922.9147	1922.9084	3	0	K.YSHVDLVVMVDGFEGEK.G	(Ions score 85)
168 - 184	641.9789	1922.9147	1922.9084	3	0	K.YSHVDLVVMVDGFEGEK.G	(Ions score 76)
168 - 184	641.9789	1922.9147	1922.9084	3	0	K.YSHVDLVVMVDGFEGEK.G	(Ions score 70)
168 - 184	962.4646	1922.9147	1922.9084	3	0	K.YSHVDLVVMVDGFEGEK.G	(Ions score 74)
168 - 184	641.9789	1922.9147	1922.9084	3	0	K.YSHVDLVVMVDGFEGEK.G	(Ions score 76)
168 - 184	641.9789	1922.9148	1922.9084	3	0	K.YSHVDLVVMVDGFEGEK.G	(Ions score 73)
168 - 184	962.4647	1922.9148	1922.9084	3	0	K.YSHVDLVVMVDGFEGEK.G	(Ions score 75)
168 - 184	962.4647	1922.9148	1922.9084	3	0	K.YSHVDLVVMVDGFEGEK.G	(Ions score 79)
168 - 184	641.9789	1922.9148	1922.9084	3	0	K.YSHVDLVVMVDGFEGEK.G	(Ions score 73)
168 - 184	641.9789	1922.9148	1922.9084	3	0	K.YSHVDLVVMVDGFEGEK.G	(Ions score 76)
168 - 184	641.9789	1922.9148	1922.9084	3	0	K.YSHVDLVVMVDGFEGEK.G	(Ions score 73)
168 - 184	962.4647	1922.9148	1922.9084	3	0	K.YSHVDLVVMVDGFEGEK.G	(Ions score 56)
168 - 184	962.4647	1922.9148	1922.9084	3	0	K.YSHVDLVVMVDGFEGEK.G	(Ions score 97)
168 - 184	962.4647	1922.9148	1922.9084	3	0	K.YSHVDLVVMVDGFEGEK.G	(Ions score 100)
168 - 184	962.4647	1922.9148	1922.9084	3	0	K.YSHVDLVVMVDGFEGEK.G	(Ions score 71)
168 - 184	962.4647	1922.9148	1922.9084	3	0	K.YSHVDLVVMVDGFEGEK.G	(Ions score 76)
168 - 184	962.4647	1922.9149	1922.9084	3	0	K.YSHVDLVVMVDGFEGEK.G	(Ions score 74)
168 - 184	641.9789	1922.9149	1922.9084	3	0	K.YSHVDLVVMVDGFEGEK.G	(Ions score 76)
168 - 184	641.9789	1922.9149	1922.9084	3	0	K.YSHVDLVVMVDGFEGEK.G	(Ions score 76)
168 - 184	962.4647	1922.9149	1922.9084	3	0	K.YSHVDLVVMVDGFEGEK.G	(Ions score 105)
168 - 184	962.4647	1922.9149	1922.9084	3	0	K.YSHVDLVVMVDGFEGEK.G	(Ions score 71)
168 - 184	962.4647	1922.9149	1922.9084	3	0	K.YSHVDLVVMVDGFEGEK.G	(Ions score 100)
168 - 184	962.4647	1922.9149	1922.9084	3	0	K.YSHVDLVVMVDGFEGEK.G	(Ions score 84)
168 - 184	641.9789	1922.9149	1922.9084	3	0	K.YSHVDLVVMVDGFEGEK.G	(Ions score 76)
168 - 184	641.9789	1922.9149	1922.9084	3	0	K.YSHVDLVVMVDGFEGEK.G	(Ions score 73)
168 - 184	641.9789	1922.9149	1922.9084	3	0	K.YSHVDLVVMVDGFEGEK.G	(Ions score 66)
168 - 184	962.4648	1922.9149	1922.9084	3	0	K.YSHVDLVVMVDGFEGEK.G	(Ions score 79)
168 - 184	641.9789	1922.9150	1922.9084	3	0	K.YSHVDLVVMVDGFEGEK.G	(Ions score 73)
168 - 184	641.9789	1922.9150	1922.9084	3	0	K.YSHVDLVVMVDGFEGEK.G	(Ions score 69)
168 - 184	641.9789	1922.9150	1922.9084	3	0	K.YSHVDLVVMVDGFEGEK.G	(Ions score 73)
168 - 184	641.9789	1922.9150	1922.9084	3	0	K.YSHVDLVVMVDGFEGEK.G	(Ions score 73)
168 - 184	962.4648	1922.9150	1922.9084	3	0	K.YSHVDLVVMVDGFEGEK.G	(Ions score 65)
168 - 184	641.9789	1922.9150	1922.9084	3	0	K.YSHVDLVVMVDGFEGEK.G	(Ions score 66)
168 - 184	962.4648	1922.9150	1922.9084	3	0	K.YSHVDLVVMVDGFEGEK.G	(Ions score 79)
168 - 184	641.9790	1922.9150	1922.9084	3	0	K.YSHVDLVVMVDGFEGEK.G	(Ions score 43)
168 - 184	641.9790	1922.9151	1922.9084	3	0	K.YSHVDLVVMVDGFEGEK.G	(Ions score 73)
168 - 184	962.4648	1922.9151	1922.9084	3	0	K.YSHVDLVVMVDGFEGEK.G	(Ions score 87)
168 - 184	962.4648	1922.9151	1922.9084	3	0	K.YSHVDLVVMVDGFEGEK.G	(Ions score 92)
168 - 184	962.4648	1922.9151	1922.9084	3	0	K.YSHVDLVVMVDGFEGEK.G	(Ions score 71)
168 - 184	962.4648	1922.9151	1922.9084	3	0	K.YSHVDLVVMVDGFEGEK.G	(Ions score 114)
168 - 184	962.4648	1922.9151	1922.9084	3	0	K.YSHVDLVVMVDGFEGEK.G	(Ions score 73)

168 - 184	962.4648	1922.9151	1922.9084	3	0	K.YSHVDLVVMVDGFEGEK.G	(Ions score 92)
168 - 184	962.4648	1922.9151	1922.9084	3	0	K.YSHVDLVVMVDGFEGEK.G	(Ions score 75)
168 - 184	641.9790	1922.9152	1922.9084	3	0	K.YSHVDLVVMVDGFEGEK.G	(Ions score 67)
168 - 184	641.9790	1922.9152	1922.9084	3	0	K.YSHVDLVVMVDGFEGEK.G	(Ions score 73)
168 - 184	641.9790	1922.9152	1922.9084	4	0	K.YSHVDLVVMVDGFEGEK.G	(Ions score 70)
168 - 184	641.9790	1922.9152	1922.9084	4	0	K.YSHVDLVVMVDGFEGEK.G	(Ions score 53)
168 - 184	962.4649	1922.9152	1922.9084	4	0	K.YSHVDLVVMVDGFEGEK.G	(Ions score 80)
168 - 184	641.9790	1922.9152	1922.9084	4	0	K.YSHVDLVVMVDGFEGEK.G	(Ions score 65)
168 - 184	641.9790	1922.9152	1922.9084	4	0	K.YSHVDLVVMVDGFEGEK.G	(Ions score 76)
168 - 184	641.9790	1922.9152	1922.9084	4	0	K.YSHVDLVVMVDGFEGEK.G	(Ions score 73)
168 - 184	641.9790	1922.9153	1922.9084	4	0	K.YSHVDLVVMVDGFEGEK.G	(Ions score 63)
168 - 184	641.9790	1922.9153	1922.9084	4	0	K.YSHVDLVVMVDGFEGEK.G	(Ions score 76)
168 - 184	641.9790	1922.9153	1922.9084	4	0	K.YSHVDLVVMVDGFEGEK.G	(Ions score 76)
168 - 184	641.9790	1922.9153	1922.9084	4	0	K.YSHVDLVVMVDGFEGEK.G	(Ions score 70)
168 - 184	962.4649	1922.9153	1922.9084	4	0	K.YSHVDLVVMVDGFEGEK.G	(Ions score 71)
168 - 184	641.9790	1922.9153	1922.9084	4	0	K.YSHVDLVVMVDGFEGEK.G	(Ions score 73)
168 - 184	962.4649	1922.9153	1922.9084	4	0	K.YSHVDLVVMVDGFEGEK.G	(Ions score 86)
168 - 184	962.4650	1922.9154	1922.9084	4	0	K.YSHVDLVVMVDGFEGEK.G	(Ions score 67)
168 - 184	962.4650	1922.9154	1922.9084	4	0	K.YSHVDLVVMVDGFEGEK.G	(Ions score 103)
168 - 184	641.9791	1922.9154	1922.9084	4	0	K.YSHVDLVVMVDGFEGEK.G	(Ions score 64)
168 - 184	641.9791	1922.9154	1922.9084	4	0	K.YSHVDLVVMVDGFEGEK.G	(Ions score 70)
168 - 184	641.9791	1922.9154	1922.9084	4	0	K.YSHVDLVVMVDGFEGEK.G	(Ions score 69)
168 - 184	962.4650	1922.9154	1922.9084	4	0	K.YSHVDLVVMVDGFEGEK.G	(Ions score 79)
168 - 184	962.4650	1922.9154	1922.9084	4	0	K.YSHVDLVVMVDGFEGEK.G	(Ions score 76)
168 - 184	962.4650	1922.9154	1922.9084	4	0	K.YSHVDLVVMVDGFEGEK.G	(Ions score 64)
168 - 184	641.9791	1922.9155	1922.9084	4	0	K.YSHVDLVVMVDGFEGEK.G	(Ions score 73)
168 - 184	641.9791	1922.9155	1922.9084	4	0	K.YSHVDLVVMVDGFEGEK.G	(Ions score 73)
168 - 184	962.4650	1922.9155	1922.9084	4	0	K.YSHVDLVVMVDGFEGEK.G	(Ions score 78)
168 - 184	641.9791	1922.9155	1922.9084	4	0	K.YSHVDLVVMVDGFEGEK.G	(Ions score 68)
168 - 184	962.4651	1922.9156	1922.9084	4	0	K.YSHVDLVVMVDGFEGEK.G	(Ions score 80)
168 - 184	641.9791	1922.9156	1922.9084	4	0	K.YSHVDLVVMVDGFEGEK.G	(Ions score 65)
168 - 184	641.9791	1922.9156	1922.9084	4	0	K.YSHVDLVVMVDGFEGEK.G	(Ions score 62)
168 - 184	962.4651	1922.9156	1922.9084	4	0	K.YSHVDLVVMVDGFEGEK.G	(Ions score 92)
168 - 184	641.9791	1922.9156	1922.9084	4	0	K.YSHVDLVVMVDGFEGEK.G	(Ions score 76)
168 - 184	641.9791	1922.9156	1922.9084	4	0	K.YSHVDLVVMVDGFEGEK.G	(Ions score 65)
168 - 184	641.9792	1922.9157	1922.9084	4	0	K.YSHVDLVVMVDGFEGEK.G	(Ions score 73)
168 - 184	962.4651	1922.9157	1922.9084	4	0	K.YSHVDLVVMVDGFEGEK.G	(Ions score 80)
168 - 184	641.9792	1922.9157	1922.9084	4	0	K.YSHVDLVVMVDGFEGEK.G	(Ions score 76)
168 - 184	962.4651	1922.9157	1922.9084	4	0	K.YSHVDLVVMVDGFEGEK.G	(Ions score 84)
168 - 184	962.4651	1922.9157	1922.9084	4	0	K.YSHVDLVVMVDGFEGEK.G	(Ions score 85)
168 - 184	962.4651	1922.9157	1922.9084	4	0	K.YSHVDLVVMVDGFEGEK.G	(Ions score 75)
168 - 184	962.4651	1922.9157	1922.9084	4	0	K.YSHVDLVVMVDGFEGEK.G	(Ions score 100)
168 - 184	641.9792	1922.9157	1922.9084	4	0	K.YSHVDLVVMVDGFEGEK.G	(Ions score 73)
168 - 184	962.4651	1922.9157	1922.9084	4	0	K.YSHVDLVVMVDGFEGEK.G	(Ions score 87)
168 - 184	962.4651	1922.9157	1922.9084	4	0	K.YSHVDLVVMVDGFEGEK.G	(Ions score 79)
168 - 184	962.4651	1922.9157	1922.9084	4	0	K.YSHVDLVVMVDGFEGEK.G	(Ions score 92)
168 - 184	641.9792	1922.9158	1922.9084	4	0	K.YSHVDLVVMVDGFEGEK.G	(Ions score 65)
168 - 184	641.9792	1922.9158	1922.9084	4	0	K.YSHVDLVVMVDGFEGEK.G	(Ions score 76)
168 - 184	641.9792	1922.9158	1922.9084	4	0	K.YSHVDLVVMVDGFEGEK.G	(Ions score 76)
168 - 184	641.9792	1922.9158	1922.9084	4	0	K.YSHVDLVVMVDGFEGEK.G	(Ions score 73)
168 - 184	641.9792	1922.9158	1922.9084	4	0	K.YSHVDLVVMVDGFEGEK.G	(Ions score 76)
168 - 184	641.9792	1922.9159	1922.9084	4	0	K.YSHVDLVVMVDGFEGEK.G	(Ions score 76)
168 - 184	641.9792	1922.9159	1922.9084	4	0	K.YSHVDLVVMVDGFEGEK.G	(Ions score 65)
168 - 184	962.4652	1922.9159	1922.9084	4	0	K.YSHVDLVVMVDGFEGEK.G	(Ions score 72)
168 - 184	641.9792	1922.9159	1922.9084	4	0	K.YSHVDLVVMVDGFEGEK.G	(Ions score 65)
168 - 184	962.4652	1922.9159	1922.9084	4	0	K.YSHVDLVVMVDGFEGEK.G	(Ions score 92)
168 - 184	962.4652	1922.9159	1922.9084	4	0	K.YSHVDLVVMVDGFEGEK.G	(Ions score 86)

168 - 184	962.4652	1922.9159	1922.9084	4	0	K.YSHVDLVVMVDGFEGEK.G	(Ions score 87)
168 - 184	962.4652	1922.9159	1922.9084	4	0	K.YSHVDLVVMVDGFEGEK.G	(Ions score 91)
168 - 184	641.9793	1922.9160	1922.9084	4	0	K.YSHVDLVVMVDGFEGEK.G	(Ions score 76)
168 - 184	641.9793	1922.9160	1922.9084	4	0	K.YSHVDLVVMVDGFEGEK.G	(Ions score 60)
168 - 184	641.9793	1922.9160	1922.9084	4	0	K.YSHVDLVVMVDGFEGEK.G	(Ions score 62)
168 - 184	962.4653	1922.9161	1922.9084	4	0	K.YSHVDLVVMVDGFEGEK.G	(Ions score 72)
168 - 184	962.4653	1922.9161	1922.9084	4	0	K.YSHVDLVVMVDGFEGEK.G	(Ions score 88)
168 - 184	641.9793	1922.9161	1922.9084	4	0	K.YSHVDLVVMVDGFEGEK.G	(Ions score 68)
168 - 184	641.9793	1922.9161	1922.9084	4	0	K.YSHVDLVVMVDGFEGEK.G	(Ions score 64)
168 - 184	641.9793	1922.9161	1922.9084	4	0	K.YSHVDLVVMVDGFEGEK.G	(Ions score 73)
168 - 184	962.4653	1922.9161	1922.9084	4	0	K.YSHVDLVVMVDGFEGEK.G	(Ions score 63)
168 - 184	641.9793	1922.9162	1922.9084	4	0	K.YSHVDLVVMVDGFEGEK.G	(Ions score 65)
168 - 184	641.9793	1922.9162	1922.9084	4	0	K.YSHVDLVVMVDGFEGEK.G	(Ions score 76)
168 - 184	962.4654	1922.9162	1922.9084	4	0	K.YSHVDLVVMVDGFEGEK.G	(Ions score 108)
168 - 184	962.4654	1922.9162	1922.9084	4	0	K.YSHVDLVVMVDGFEGEK.G	(Ions score 97)
168 - 184	641.9793	1922.9162	1922.9084	4	0	K.YSHVDLVVMVDGFEGEK.G	(Ions score 73)
168 - 184	962.4654	1922.9162	1922.9084	4	0	K.YSHVDLVVMVDGFEGEK.G	(Ions score 72)
168 - 184	641.9794	1922.9162	1922.9084	4	0	K.YSHVDLVVMVDGFEGEK.G	(Ions score 76)
168 - 184	641.9794	1922.9163	1922.9084	4	0	K.YSHVDLVVMVDGFEGEK.G	(Ions score 73)
168 - 184	962.4654	1922.9163	1922.9084	4	0	K.YSHVDLVVMVDGFEGEK.G	(Ions score 76)
168 - 184	641.9794	1922.9163	1922.9084	4	0	K.YSHVDLVVMVDGFEGEK.G	(Ions score 76)
168 - 184	641.9794	1922.9163	1922.9084	4	0	K.YSHVDLVVMVDGFEGEK.G	(Ions score 76)
168 - 184	641.9794	1922.9164	1922.9084	4	0	K.YSHVDLVVMVDGFEGEK.G	(Ions score 48)
168 - 184	641.9794	1922.9164	1922.9084	4	0	K.YSHVDLVVMVDGFEGEK.G	(Ions score 65)
168 - 184	962.4655	1922.9164	1922.9084	4	0	K.YSHVDLVVMVDGFEGEK.G	(Ions score 97)
168 - 184	641.9794	1922.9164	1922.9084	4	0	K.YSHVDLVVMVDGFEGEK.G	(Ions score 76)
168 - 184	641.9794	1922.9164	1922.9084	4	0	K.YSHVDLVVMVDGFEGEK.G	(Ions score 70)
168 - 184	641.9794	1922.9165	1922.9084	4	0	K.YSHVDLVVMVDGFEGEK.G	(Ions score 73)
168 - 184	641.9794	1922.9165	1922.9084	4	0	K.YSHVDLVVMVDGFEGEK.G	(Ions score 41)
168 - 184	641.9795	1922.9166	1922.9084	4	0	K.YSHVDLVVMVDGFEGEK.G	(Ions score 76)
168 - 184	641.9795	1922.9166	1922.9084	4	0	K.YSHVDLVVMVDGFEGEK.G	(Ions score 66)
168 - 184	962.4656	1922.9166	1922.9084	4	0	K.YSHVDLVVMVDGFEGEK.G	(Ions score 75)
168 - 184	962.4656	1922.9166	1922.9084	4	0	K.YSHVDLVVMVDGFEGEK.G	(Ions score 103)
168 - 184	641.9795	1922.9166	1922.9084	4	0	K.YSHVDLVVMVDGFEGEK.G	(Ions score 76)
168 - 184	641.9795	1922.9167	1922.9084	4	0	K.YSHVDLVVMVDGFEGEK.G	(Ions score 76)
168 - 184	641.9795	1922.9167	1922.9084	4	0	K.YSHVDLVVMVDGFEGEK.G	(Ions score 76)
168 - 184	962.4656	1922.9167	1922.9084	4	0	K.YSHVDLVVMVDGFEGEK.G	(Ions score 76)
168 - 184	962.4656	1922.9167	1922.9084	4	0	K.YSHVDLVVMVDGFEGEK.G	(Ions score 100)
168 - 184	641.9795	1922.9167	1922.9084	4	0	K.YSHVDLVVMVDGFEGEK.G	(Ions score 73)
168 - 184	641.9795	1922.9168	1922.9084	4	0	K.YSHVDLVVMVDGFEGEK.G	(Ions score 71)
168 - 184	962.4657	1922.9168	1922.9084	4	0	K.YSHVDLVVMVDGFEGEK.G	(Ions score 65)
168 - 184	641.9796	1922.9168	1922.9084	4	0	K.YSHVDLVVMVDGFEGEK.G	(Ions score 73)
168 - 184	962.4657	1922.9169	1922.9084	4	0	K.YSHVDLVVMVDGFEGEK.G	(Ions score 76)
168 - 184	641.9796	1922.9169	1922.9084	4	0	K.YSHVDLVVMVDGFEGEK.G	(Ions score 70)
168 - 184	641.9796	1922.9170	1922.9084	4	0	K.YSHVDLVVMVDGFEGEK.G	(Ions score 51)
168 - 184	641.9796	1922.9170	1922.9084	4	0	K.YSHVDLVVMVDGFEGEK.G	(Ions score 77)
168 - 184	641.9796	1922.9170	1922.9084	4	0	K.YSHVDLVVMVDGFEGEK.G	(Ions score 76)
168 - 184	962.4658	1922.9170	1922.9084	4	0	K.YSHVDLVVMVDGFEGEK.G	(Ions score 69)
168 - 184	641.9796	1922.9171	1922.9084	4	0	K.YSHVDLVVMVDGFEGEK.G	(Ions score 76)
168 - 184	641.9796	1922.9171	1922.9084	4	0	K.YSHVDLVVMVDGFEGEK.G	(Ions score 68)
168 - 184	962.4658	1922.9171	1922.9084	5	0	K.YSHVDLVVMVDGFEGEK.G	(Ions score 77)
168 - 184	641.9796	1922.9171	1922.9084	5	0	K.YSHVDLVVMVDGFEGEK.G	(Ions score 76)
168 - 184	962.4658	1922.9171	1922.9084	5	0	K.YSHVDLVVMVDGFEGEK.G	(Ions score 72)
168 - 184	641.9797	1922.9171	1922.9084	5	0	K.YSHVDLVVMVDGFEGEK.G	(Ions score 70)
168 - 184	962.4659	1922.9172	1922.9084	5	0	K.YSHVDLVVMVDGFEGEK.G	(Ions score 79)
168 - 184	641.9797	1922.9172	1922.9084	5	0	K.YSHVDLVVMVDGFEGEK.G	(Ions score 76)
168 - 184	641.9797	1922.9173	1922.9084	5	0	K.YSHVDLVVMVDGFEGEK.G	(Ions score 76)

168 - 184	641.9797	1922.9173	1922.9084	5	0	K.YSHVDLVVMVDGFEGEK.G	(Ions score 61)
168 - 184	641.9797	1922.9173	1922.9084	5	0	K.YSHVDLVVMVDGFEGEK.G	(Ions score 70)
168 - 184	641.9797	1922.9173	1922.9084	5	0	K.YSHVDLVVMVDGFEGEK.G	(Ions score 76)
168 - 184	641.9797	1922.9174	1922.9084	5	0	K.YSHVDLVVMVDGFEGEK.G	(Ions score 77)
168 - 184	962.4660	1922.9174	1922.9084	5	0	K.YSHVDLVVMVDGFEGEK.G	(Ions score 85)
168 - 184	641.9797	1922.9174	1922.9084	5	0	K.YSHVDLVVMVDGFEGEK.G	(Ions score 73)
168 - 184	962.4660	1922.9174	1922.9084	5	0	K.YSHVDLVVMVDGFEGEK.G	(Ions score 90)
168 - 184	641.9798	1922.9174	1922.9084	5	0	K.YSHVDLVVMVDGFEGEK.G	(Ions score 70)
168 - 184	962.4660	1922.9175	1922.9084	5	0	K.YSHVDLVVMVDGFEGEK.G	(Ions score 66)
168 - 184	962.4660	1922.9175	1922.9084	5	0	K.YSHVDLVVMVDGFEGEK.G	(Ions score 76)
168 - 184	641.9798	1922.9175	1922.9084	5	0	K.YSHVDLVVMVDGFEGEK.G	(Ions score 76)
168 - 184	962.4660	1922.9175	1922.9084	5	0	K.YSHVDLVVMVDGFEGEK.G	(Ions score 91)
168 - 184	641.9798	1922.9175	1922.9084	5	0	K.YSHVDLVVMVDGFEGEK.G	(Ions score 73)
168 - 184	641.9798	1922.9175	1922.9084	5	0	K.YSHVDLVVMVDGFEGEK.G	(Ions score 73)
168 - 184	641.9798	1922.9175	1922.9084	5	0	K.YSHVDLVVMVDGFEGEK.G	(Ions score 62)
168 - 184	962.4660	1922.9175	1922.9084	5	0	K.YSHVDLVVMVDGFEGEK.G	(Ions score 74)
168 - 184	962.4660	1922.9175	1922.9084	5	0	K.YSHVDLVVMVDGFEGEK.G	(Ions score 59)
168 - 184	962.4660	1922.9175	1922.9084	5	0	K.YSHVDLVVMVDGFEGEK.G	(Ions score 88)
168 - 184	641.9798	1922.9176	1922.9084	5	0	K.YSHVDLVVMVDGFEGEK.G	(Ions score 69)
168 - 184	641.9798	1922.9176	1922.9084	5	0	K.YSHVDLVVMVDGFEGEK.G	(Ions score 73)
168 - 184	641.9798	1922.9176	1922.9084	5	0	K.YSHVDLVVMVDGFEGEK.G	(Ions score 70)
168 - 184	962.4661	1922.9176	1922.9084	5	0	K.YSHVDLVVMVDGFEGEK.G	(Ions score 105)
168 - 184	962.4661	1922.9176	1922.9084	5	0	K.YSHVDLVVMVDGFEGEK.G	(Ions score 87)
168 - 184	641.9798	1922.9177	1922.9084	5	0	K.YSHVDLVVMVDGFEGEK.G	(Ions score 70)
168 - 184	641.9798	1922.9177	1922.9084	5	0	K.YSHVDLVVMVDGFEGEK.G	(Ions score 76)
168 - 184	962.4661	1922.9177	1922.9084	5	0	K.YSHVDLVVMVDGFEGEK.G	(Ions score 54)
168 - 184	962.4662	1922.9178	1922.9084	5	0	K.YSHVDLVVMVDGFEGEK.G	(Ions score 63)
168 - 184	962.4662	1922.9179	1922.9084	5	0	K.YSHVDLVVMVDGFEGEK.G	(Ions score 69)
168 - 184	641.9800	1922.9180	1922.9084	5	0	K.YSHVDLVVMVDGFEGEK.G	(Ions score 73)
168 - 184	970.4568	1938.8990	1938.9034	-2	0	K.YSHVDLVVMVDGFEGEK.G	Oxidation (M) (Ions score 49)
168 - 184	970.4568	1938.8991	1938.9034	-2	0	K.YSHVDLVVMVDGFEGEK.G	Oxidation (M) (Ions score 47)
168 - 184	970.4573	1938.9000	1938.9034	-2	0	K.YSHVDLVVMVDGFEGEK.G	Oxidation (M) (Ions score 38)
168 - 184	647.3074	1938.9005	1938.9034	-1	0	K.YSHVDLVVMVDGFEGEK.G	Oxidation (M) (Ions score 41)
168 - 184	970.4578	1938.9010	1938.9034	-1	0	K.YSHVDLVVMVDGFEGEK.G	Oxidation (M) (Ions score 40)
168 - 184	647.3077	1938.9011	1938.9034	-1	0	K.YSHVDLVVMVDGFEGEK.G	Oxidation (M) (Ions score 41)
168 - 184	970.4579	1938.9013	1938.9034	-1	0	K.YSHVDLVVMVDGFEGEK.G	Oxidation (M) (Ions score 38)
168 - 184	647.3082	1938.9029	1938.9034	-0	0	K.YSHVDLVVMVDGFEGEK.G	Oxidation (M) (Ions score 33)
168 - 184	970.4588	1938.9030	1938.9034	-0	0	K.YSHVDLVVMVDGFEGEK.G	Oxidation (M) (Ions score 40)
168 - 184	970.4590	1938.9035	1938.9034	0	0	K.YSHVDLVVMVDGFEGEK.G	Oxidation (M) (Ions score 44)
168 - 184	970.4590	1938.9035	1938.9034	0	0	K.YSHVDLVVMVDGFEGEK.G	Oxidation (M) (Ions score 49)
168 - 184	647.3085	1938.9038	1938.9034	0	0	K.YSHVDLVVMVDGFEGEK.G	Oxidation (M) (Ions score 61)
168 - 184	970.4592	1938.9038	1938.9034	0	0	K.YSHVDLVVMVDGFEGEK.G	Oxidation (M) (Ions score 48)
168 - 184	970.4593	1938.9040	1938.9034	0	0	K.YSHVDLVVMVDGFEGEK.G	Oxidation (M) (Ions score 70)
168 - 184	970.4593	1938.9041	1938.9034	0	0	K.YSHVDLVVMVDGFEGEK.G	Oxidation (M) (Ions score 51)
168 - 184	970.4594	1938.9043	1938.9034	0	0	K.YSHVDLVVMVDGFEGEK.G	Oxidation (M) (Ions score 55)
168 - 184	647.3087	1938.9043	1938.9034	0	0	K.YSHVDLVVMVDGFEGEK.G	Oxidation (M) (Ions score 58)
168 - 184	647.3087	1938.9044	1938.9034	1	0	K.YSHVDLVVMVDGFEGEK.G	Oxidation (M) (Ions score 62)
168 - 184	647.3087	1938.9044	1938.9034	1	0	K.YSHVDLVVMVDGFEGEK.G	Oxidation (M) (Ions score 67)
168 - 184	647.3087	1938.9044	1938.9034	1	0	K.YSHVDLVVMVDGFEGEK.G	Oxidation (M) (Ions score 59)
168 - 184	970.4595	1938.9044	1938.9034	1	0	K.YSHVDLVVMVDGFEGEK.G	Oxidation (M) (Ions score 58)
168 - 184	970.4595	1938.9044	1938.9034	1	0	K.YSHVDLVVMVDGFEGEK.G	Oxidation (M) (Ions score 58)
168 - 184	970.4596	1938.9046	1938.9034	1	0	K.YSHVDLVVMVDGFEGEK.G	Oxidation (M) (Ions score 75)
168 - 184	970.4596	1938.9046	1938.9034	1	0	K.YSHVDLVVMVDGFEGEK.G	Oxidation (M) (Ions score 57)
168 - 184	970.4596	1938.9046	1938.9034	1	0	K.YSHVDLVVMVDGFEGEK.G	Oxidation (M) (Ions score 49)
168 - 184	970.4596	1938.9046	1938.9034	1	0	K.YSHVDLVVMVDGFEGEK.G	Oxidation (M) (Ions score 52)
168 - 184	647.3088	1938.9047	1938.9034	1	0	K.YSHVDLVVMVDGFEGEK.G	Oxidation (M) (Ions score 61)
168 - 184	647.3088	1938.9047	1938.9034	1	0	K.YSHVDLVVMVDGFEGEK.G	Oxidation (M) (Ions score 59)

168 - 184	970.4596	1938.9047	1938.9034	1	0	K.YSHVDLVVMVDGFEGEK.G	Oxidation (M)	(Ions score 50)
168 - 184	970.4597	1938.9047	1938.9034	1	0	K.YSHVDLVVMVDGFEGEK.G	Oxidation (M)	(Ions score 41)
168 - 184	647.3089	1938.9048	1938.9034	1	0	K.YSHVDLVVMVDGFEGEK.G	Oxidation (M)	(Ions score 34)
168 - 184	970.4597	1938.9049	1938.9034	1	0	K.YSHVDLVVMVDGFEGEK.G	Oxidation (M)	(Ions score 43)
168 - 184	647.3089	1938.9049	1938.9034	1	0	K.YSHVDLVVMVDGFEGEK.G	Oxidation (M)	(Ions score 33)
168 - 184	970.4598	1938.9050	1938.9034	1	0	K.YSHVDLVVMVDGFEGEK.G	Oxidation (M)	(Ions score 39)
168 - 184	970.4598	1938.9050	1938.9034	1	0	K.YSHVDLVVMVDGFEGEK.G	Oxidation (M)	(Ions score 39)
168 - 184	970.4598	1938.9050	1938.9034	1	0	K.YSHVDLVVMVDGFEGEK.G	Oxidation (M)	(Ions score 45)
168 - 184	970.4598	1938.9050	1938.9034	1	0	K.YSHVDLVVMVDGFEGEK.G	Oxidation (M)	(Ions score 57)
168 - 184	970.4598	1938.9051	1938.9034	1	0	K.YSHVDLVVMVDGFEGEK.G	Oxidation (M)	(Ions score 56)
168 - 184	970.4598	1938.9051	1938.9034	1	0	K.YSHVDLVVMVDGFEGEK.G	Oxidation (M)	(Ions score 53)
168 - 184	970.4598	1938.9051	1938.9034	1	0	K.YSHVDLVVMVDGFEGEK.G	Oxidation (M)	(Ions score 42)
168 - 184	970.4598	1938.9051	1938.9034	1	0	K.YSHVDLVVMVDGFEGEK.G	Oxidation (M)	(Ions score 55)
168 - 184	647.3090	1938.9051	1938.9034	1	0	K.YSHVDLVVMVDGFEGEK.G	Oxidation (M)	(Ions score 61)
168 - 184	970.4598	1938.9051	1938.9034	1	0	K.YSHVDLVVMVDGFEGEK.G	Oxidation (M)	(Ions score 74)
168 - 184	647.3090	1938.9053	1938.9034	1	0	K.YSHVDLVVMVDGFEGEK.G	Oxidation (M)	(Ions score 64)
168 - 184	970.4599	1938.9053	1938.9034	1	0	K.YSHVDLVVMVDGFEGEK.G	Oxidation (M)	(Ions score 67)
168 - 184	970.4599	1938.9053	1938.9034	1	0	K.YSHVDLVVMVDGFEGEK.G	Oxidation (M)	(Ions score 55)
168 - 184	647.3090	1938.9053	1938.9034	1	0	K.YSHVDLVVMVDGFEGEK.G	Oxidation (M)	(Ions score 56)
168 - 184	970.4600	1938.9054	1938.9034	1	0	K.YSHVDLVVMVDGFEGEK.G	Oxidation (M)	(Ions score 40)
168 - 184	647.3091	1938.9054	1938.9034	1	0	K.YSHVDLVVMVDGFEGEK.G	Oxidation (M)	(Ions score 50)
168 - 184	970.4600	1938.9054	1938.9034	1	0	K.YSHVDLVVMVDGFEGEK.G	Oxidation (M)	(Ions score 55)
168 - 184	647.3091	1938.9054	1938.9034	1	0	K.YSHVDLVVMVDGFEGEK.G	Oxidation (M)	(Ions score 58)
168 - 184	647.3091	1938.9054	1938.9034	1	0	K.YSHVDLVVMVDGFEGEK.G	Oxidation (M)	(Ions score 62)
168 - 184	970.4600	1938.9054	1938.9034	1	0	K.YSHVDLVVMVDGFEGEK.G	Oxidation (M)	(Ions score 48)
168 - 184	970.4600	1938.9054	1938.9034	1	0	K.YSHVDLVVMVDGFEGEK.G	Oxidation (M)	(Ions score 60)
168 - 184	970.4600	1938.9054	1938.9034	1	0	K.YSHVDLVVMVDGFEGEK.G	Oxidation (M)	(Ions score 57)
168 - 184	970.4600	1938.9055	1938.9034	1	0	K.YSHVDLVVMVDGFEGEK.G	Oxidation (M)	(Ions score 31)
168 - 184	970.4600	1938.9055	1938.9034	1	0	K.YSHVDLVVMVDGFEGEK.G	Oxidation (M)	(Ions score 62)
168 - 184	970.4601	1938.9056	1938.9034	1	0	K.YSHVDLVVMVDGFEGEK.G	Oxidation (M)	(Ions score 39)
168 - 184	647.3091	1938.9056	1938.9034	1	0	K.YSHVDLVVMVDGFEGEK.G	Oxidation (M)	(Ions score 35)
168 - 184	647.3092	1938.9056	1938.9034	1	0	K.YSHVDLVVMVDGFEGEK.G	Oxidation (M)	(Ions score 56)
168 - 184	647.3092	1938.9056	1938.9034	1	0	K.YSHVDLVVMVDGFEGEK.G	Oxidation (M)	(Ions score 56)
168 - 184	647.3092	1938.9057	1938.9034	1	0	K.YSHVDLVVMVDGFEGEK.G	Oxidation (M)	(Ions score 72)
168 - 184	970.4601	1938.9057	1938.9034	1	0	K.YSHVDLVVMVDGFEGEK.G	Oxidation (M)	(Ions score 63)
168 - 184	647.3092	1938.9057	1938.9034	1	0	K.YSHVDLVVMVDGFEGEK.G	Oxidation (M)	(Ions score 63)
168 - 184	647.3092	1938.9057	1938.9034	1	0	K.YSHVDLVVMVDGFEGEK.G	Oxidation (M)	(Ions score 56)
168 - 184	647.3092	1938.9057	1938.9034	1	0	K.YSHVDLVVMVDGFEGEK.G	Oxidation (M)	(Ions score 64)
168 - 184	647.3092	1938.9057	1938.9034	1	0	K.YSHVDLVVMVDGFEGEK.G	Oxidation (M)	(Ions score 56)
168 - 184	647.3092	1938.9058	1938.9034	1	0	K.YSHVDLVVMVDGFEGEK.G	Oxidation (M)	(Ions score 59)
168 - 184	647.3092	1938.9058	1938.9034	1	0	K.YSHVDLVVMVDGFEGEK.G	Oxidation (M)	(Ions score 53)
168 - 184	647.3092	1938.9058	1938.9034	1	0	K.YSHVDLVVMVDGFEGEK.G	Oxidation (M)	(Ions score 55)
168 - 184	647.3092	1938.9058	1938.9034	1	0	K.YSHVDLVVMVDGFEGEK.G	Oxidation (M)	(Ions score 52)
168 - 184	647.3092	1938.9058	1938.9034	1	0	K.YSHVDLVVMVDGFEGEK.G	Oxidation (M)	(Ions score 44)
168 - 184	970.4602	1938.9058	1938.9034	1	0	K.YSHVDLVVMVDGFEGEK.G	Oxidation (M)	(Ions score 61)
168 - 184	970.4602	1938.9058	1938.9034	1	0	K.YSHVDLVVMVDGFEGEK.G	Oxidation (M)	(Ions score 56)
168 - 184	647.3092	1938.9059	1938.9034	1	0	K.YSHVDLVVMVDGFEGEK.G	Oxidation (M)	(Ions score 66)
168 - 184	970.4602	1938.9059	1938.9034	1	0	K.YSHVDLVVMVDGFEGEK.G	Oxidation (M)	(Ions score 58)
168 - 184	970.4602	1938.9059	1938.9034	1	0	K.YSHVDLVVMVDGFEGEK.G	Oxidation (M)	(Ions score 42)
168 - 184	647.3093	1938.9060	1938.9034	1	0	K.YSHVDLVVMVDGFEGEK.G	Oxidation (M)	(Ions score 42)
168 - 184	970.4603	1938.9060	1938.9034	1	0	K.YSHVDLVVMVDGFEGEK.G	Oxidation (M)	(Ions score 51)
168 - 184	647.3093	1938.9060	1938.9034	1	0	K.YSHVDLVVMVDGFEGEK.G	Oxidation (M)	(Ions score 69)
168 - 184	647.3093	1938.9060	1938.9034	1	0	K.YSHVDLVVMVDGFEGEK.G	Oxidation (M)	(Ions score 63)
168 - 184	970.4603	1938.9060	1938.9034	1	0	K.YSHVDLVVMVDGFEGEK.G	Oxidation (M)	(Ions score 62)
168 - 184	647.3093	1938.9061	1938.9034	1	0	K.YSHVDLVVMVDGFEGEK.G	Oxidation (M)	(Ions score 61)
168 - 184	647.3093	1938.9061	1938.9034	1	0	K.YSHVDLVVMVDGFEGEK.G	Oxidation (M)	(Ions score 66)
168 - 184	647.3093	1938.9061	1938.9034	1	0	K.YSHVDLVVMVDGFEGEK.G	Oxidation (M)	(Ions score 41)

168 - 184	970.4603	1938.9061	1938.9034	1	0	K.YSHVDLVVMVDGFEGEK.G	Oxidation (M)	(Ions score 51)
168 - 184	647.3093	1938.9062	1938.9034	1	0	K.YSHVDLVVMVDGFEGEK.G	Oxidation (M)	(Ions score 51)
168 - 184	647.3093	1938.9062	1938.9034	1	0	K.YSHVDLVVMVDGFEGEK.G	Oxidation (M)	(Ions score 59)
168 - 184	970.4604	1938.9062	1938.9034	1	0	K.YSHVDLVVMVDGFEGEK.G	Oxidation (M)	(Ions score 55)
168 - 184	647.3094	1938.9062	1938.9034	1	0	K.YSHVDLVVMVDGFEGEK.G	Oxidation (M)	(Ions score 51)
168 - 184	647.3094	1938.9062	1938.9034	1	0	K.YSHVDLVVMVDGFEGEK.G	Oxidation (M)	(Ions score 69)
168 - 184	647.3094	1938.9062	1938.9034	1	0	K.YSHVDLVVMVDGFEGEK.G	Oxidation (M)	(Ions score 54)
168 - 184	970.4604	1938.9062	1938.9034	1	0	K.YSHVDLVVMVDGFEGEK.G	Oxidation (M)	(Ions score 51)
168 - 184	970.4604	1938.9063	1938.9034	1	0	K.YSHVDLVVMVDGFEGEK.G	Oxidation (M)	(Ions score 61)
168 - 184	970.4604	1938.9063	1938.9034	1	0	K.YSHVDLVVMVDGFEGEK.G	Oxidation (M)	(Ions score 56)
168 - 184	647.3094	1938.9063	1938.9034	2	0	K.YSHVDLVVMVDGFEGEK.G	Oxidation (M)	(Ions score 69)
168 - 184	647.3094	1938.9063	1938.9034	2	0	K.YSHVDLVVMVDGFEGEK.G	Oxidation (M)	(Ions score 69)
168 - 184	647.3094	1938.9063	1938.9034	2	0	K.YSHVDLVVMVDGFEGEK.G	Oxidation (M)	(Ions score 61)
168 - 184	647.3094	1938.9064	1938.9034	2	0	K.YSHVDLVVMVDGFEGEK.G	Oxidation (M)	(Ions score 61)
168 - 184	647.3094	1938.9064	1938.9034	2	0	K.YSHVDLVVMVDGFEGEK.G	Oxidation (M)	(Ions score 46)
168 - 184	970.4605	1938.9064	1938.9034	2	0	K.YSHVDLVVMVDGFEGEK.G	Oxidation (M)	(Ions score 59)
168 - 184	970.4605	1938.9064	1938.9034	2	0	K.YSHVDLVVMVDGFEGEK.G	Oxidation (M)	(Ions score 50)
168 - 184	970.4605	1938.9064	1938.9034	2	0	K.YSHVDLVVMVDGFEGEK.G	Oxidation (M)	(Ions score 47)
168 - 184	970.4605	1938.9064	1938.9034	2	0	K.YSHVDLVVMVDGFEGEK.G	Oxidation (M)	(Ions score 57)
168 - 184	970.4605	1938.9064	1938.9034	2	0	K.YSHVDLVVMVDGFEGEK.G	Oxidation (M)	(Ions score 62)
168 - 184	970.4605	1938.9064	1938.9034	2	0	K.YSHVDLVVMVDGFEGEK.G	Oxidation (M)	(Ions score 55)
168 - 184	647.3094	1938.9065	1938.9034	2	0	K.YSHVDLVVMVDGFEGEK.G	Oxidation (M)	(Ions score 61)
168 - 184	647.3094	1938.9065	1938.9034	2	0	K.YSHVDLVVMVDGFEGEK.G	Oxidation (M)	(Ions score 59)
168 - 184	970.4605	1938.9065	1938.9034	2	0	K.YSHVDLVVMVDGFEGEK.G	Oxidation (M)	(Ions score 44)
168 - 184	647.3094	1938.9065	1938.9034	2	0	K.YSHVDLVVMVDGFEGEK.G	Oxidation (M)	(Ions score 58)
168 - 184	647.3094	1938.9065	1938.9034	2	0	K.YSHVDLVVMVDGFEGEK.G	Oxidation (M)	(Ions score 57)
168 - 184	647.3094	1938.9065	1938.9034	2	0	K.YSHVDLVVMVDGFEGEK.G	Oxidation (M)	(Ions score 62)
168 - 184	647.3094	1938.9065	1938.9034	2	0	K.YSHVDLVVMVDGFEGEK.G	Oxidation (M)	(Ions score 62)
168 - 184	970.4605	1938.9065	1938.9034	2	0	K.YSHVDLVVMVDGFEGEK.G	Oxidation (M)	(Ions score 54)
168 - 184	647.3095	1938.9065	1938.9034	2	0	K.YSHVDLVVMVDGFEGEK.G	Oxidation (M)	(Ions score 64)
168 - 184	970.4606	1938.9066	1938.9034	2	0	K.YSHVDLVVMVDGFEGEK.G	Oxidation (M)	(Ions score 41)
168 - 184	647.3095	1938.9066	1938.9034	2	0	K.YSHVDLVVMVDGFEGEK.G	Oxidation (M)	(Ions score 61)
168 - 184	647.3095	1938.9066	1938.9034	2	0	K.YSHVDLVVMVDGFEGEK.G	Oxidation (M)	(Ions score 63)
168 - 184	647.3095	1938.9066	1938.9034	2	0	K.YSHVDLVVMVDGFEGEK.G	Oxidation (M)	(Ions score 64)
168 - 184	647.3095	1938.9066	1938.9034	2	0	K.YSHVDLVVMVDGFEGEK.G	Oxidation (M)	(Ions score 54)
168 - 184	647.3095	1938.9066	1938.9034	2	0	K.YSHVDLVVMVDGFEGEK.G	Oxidation (M)	(Ions score 69)
168 - 184	647.3095	1938.9066	1938.9034	2	0	K.YSHVDLVVMVDGFEGEK.G	Oxidation (M)	(Ions score 63)
168 - 184	647.3095	1938.9066	1938.9034	2	0	K.YSHVDLVVMVDGFEGEK.G	Oxidation (M)	(Ions score 49)
168 - 184	647.3095	1938.9066	1938.9034	2	0	K.YSHVDLVVMVDGFEGEK.G	Oxidation (M)	(Ions score 72)
168 - 184	970.4606	1938.9066	1938.9034	2	0	K.YSHVDLVVMVDGFEGEK.G	Oxidation (M)	(Ions score 66)
168 - 184	647.3095	1938.9066	1938.9034	2	0	K.YSHVDLVVMVDGFEGEK.G	Oxidation (M)	(Ions score 81)
168 - 184	647.3095	1938.9066	1938.9034	2	0	K.YSHVDLVVMVDGFEGEK.G	Oxidation (M)	(Ions score 46)
168 - 184	970.4606	1938.9067	1938.9034	2	0	K.YSHVDLVVMVDGFEGEK.G	Oxidation (M)	(Ions score 62)
168 - 184	970.4606	1938.9067	1938.9034	2	0	K.YSHVDLVVMVDGFEGEK.G	Oxidation (M)	(Ions score 57)
168 - 184	647.3095	1938.9067	1938.9034	2	0	K.YSHVDLVVMVDGFEGEK.G	Oxidation (M)	(Ions score 64)
168 - 184	970.4606	1938.9067	1938.9034	2	0	K.YSHVDLVVMVDGFEGEK.G	Oxidation (M)	(Ions score 69)
168 - 184	647.3095	1938.9067	1938.9034	2	0	K.YSHVDLVVMVDGFEGEK.G	Oxidation (M)	(Ions score 40)
168 - 184	647.3095	1938.9067	1938.9034	2	0	K.YSHVDLVVMVDGFEGEK.G	Oxidation (M)	(Ions score 38)
168 - 184	647.3095	1938.9067	1938.9034	2	0	K.YSHVDLVVMVDGFEGEK.G	Oxidation (M)	(Ions score 62)
168 - 184	647.3095	1938.9067	1938.9034	2	0	K.YSHVDLVVMVDGFEGEK.G	Oxidation (M)	(Ions score 42)
168 - 184	647.3095	1938.9067	1938.9034	2	0	K.YSHVDLVVMVDGFEGEK.G	Oxidation (M)	(Ions score 70)
168 - 184	970.4606	1938.9067	1938.9034	2	0	K.YSHVDLVVMVDGFEGEK.G	Oxidation (M)	(Ions score 58)
168 - 184	970.4606	1938.9067	1938.9034	2	0	K.YSHVDLVVMVDGFEGEK.G	Oxidation (M)	(Ions score 63)
168 - 184	970.4606	1938.9067	1938.9034	2	0	K.YSHVDLVVMVDGFEGEK.G	Oxidation (M)	(Ions score 55)
168 - 184	970.4606	1938.9067	1938.9034	2	0	K.YSHVDLVVMVDGFEGEK.G	Oxidation (M)	(Ions score 49)
168 - 184	647.3095	1938.9067	1938.9034	2	0	K.YSHVDLVVMVDGFEGEK.G	Oxidation (M)	(Ions score 51)
168 - 184	647.3095	1938.9067	1938.9034	2	0	K.YSHVDLVVMVDGFEGEK.G	Oxidation (M)	(Ions score 45)

168 - 184	647.3095	1938.9067	1938.9034	2	0	K.YSHVDLVVMVDGFEGEK.G	Oxidation (M)	(Ions score 49)
168 - 184	970.4607	1938.9068	1938.9034	2	0	K.YSHVDLVVMVDGFEGEK.G	Oxidation (M)	(Ions score 41)
168 - 184	647.3095	1938.9068	1938.9034	2	0	K.YSHVDLVVMVDGFEGEK.G	Oxidation (M)	(Ions score 56)
168 - 184	647.3095	1938.9068	1938.9034	2	0	K.YSHVDLVVMVDGFEGEK.G	Oxidation (M)	(Ions score 56)
168 - 184	970.4607	1938.9068	1938.9034	2	0	K.YSHVDLVVMVDGFEGEK.G	Oxidation (M)	(Ions score 70)
168 - 184	647.3095	1938.9068	1938.9034	2	0	K.YSHVDLVVMVDGFEGEK.G	Oxidation (M)	(Ions score 49)
168 - 184	647.3095	1938.9068	1938.9034	2	0	K.YSHVDLVVMVDGFEGEK.G	Oxidation (M)	(Ions score 55)
168 - 184	970.4607	1938.9068	1938.9034	2	0	K.YSHVDLVVMVDGFEGEK.G	Oxidation (M)	(Ions score 58)
168 - 184	647.3095	1938.9068	1938.9034	2	0	K.YSHVDLVVMVDGFEGEK.G	Oxidation (M)	(Ions score 59)
168 - 184	647.3095	1938.9068	1938.9034	2	0	K.YSHVDLVVMVDGFEGEK.G	Oxidation (M)	(Ions score 66)
168 - 184	647.3096	1938.9068	1938.9034	2	0	K.YSHVDLVVMVDGFEGEK.G	Oxidation (M)	(Ions score 58)
168 - 184	970.4607	1938.9068	1938.9034	2	0	K.YSHVDLVVMVDGFEGEK.G	Oxidation (M)	(Ions score 55)
168 - 184	970.4607	1938.9068	1938.9034	2	0	K.YSHVDLVVMVDGFEGEK.G	Oxidation (M)	(Ions score 41)
168 - 184	647.3096	1938.9068	1938.9034	2	0	K.YSHVDLVVMVDGFEGEK.G	Oxidation (M)	(Ions score 48)
168 - 184	970.4607	1938.9068	1938.9034	2	0	K.YSHVDLVVMVDGFEGEK.G	Oxidation (M)	(Ions score 56)
168 - 184	970.4607	1938.9068	1938.9034	2	0	K.YSHVDLVVMVDGFEGEK.G	Oxidation (M)	(Ions score 53)
168 - 184	970.4607	1938.9068	1938.9034	2	0	K.YSHVDLVVMVDGFEGEK.G	Oxidation (M)	(Ions score 63)
168 - 184	970.4607	1938.9068	1938.9034	2	0	K.YSHVDLVVMVDGFEGEK.G	Oxidation (M)	(Ions score 69)
168 - 184	970.4607	1938.9069	1938.9034	2	0	K.YSHVDLVVMVDGFEGEK.G	Oxidation (M)	(Ions score 59)
168 - 184	647.3096	1938.9069	1938.9034	2	0	K.YSHVDLVVMVDGFEGEK.G	Oxidation (M)	(Ions score 60)
168 - 184	647.3096	1938.9069	1938.9034	2	0	K.YSHVDLVVMVDGFEGEK.G	Oxidation (M)	(Ions score 61)
168 - 184	970.4607	1938.9069	1938.9034	2	0	K.YSHVDLVVMVDGFEGEK.G	Oxidation (M)	(Ions score 54)
168 - 184	647.3096	1938.9069	1938.9034	2	0	K.YSHVDLVVMVDGFEGEK.G	Oxidation (M)	(Ions score 58)
168 - 184	647.3096	1938.9069	1938.9034	2	0	K.YSHVDLVVMVDGFEGEK.G	Oxidation (M)	(Ions score 58)
168 - 184	647.3096	1938.9069	1938.9034	2	0	K.YSHVDLVVMVDGFEGEK.G	Oxidation (M)	(Ions score 58)
168 - 184	647.3096	1938.9069	1938.9034	2	0	K.YSHVDLVVMVDGFEGEK.G	Oxidation (M)	(Ions score 58)
168 - 184	647.3096	1938.9069	1938.9034	2	0	K.YSHVDLVVMVDGFEGEK.G	Oxidation (M)	(Ions score 69)
168 - 184	647.3096	1938.9069	1938.9034	2	0	K.YSHVDLVVMVDGFEGEK.G	Oxidation (M)	(Ions score 52)
168 - 184	647.3096	1938.9069	1938.9034	2	0	K.YSHVDLVVMVDGFEGEK.G	Oxidation (M)	(Ions score 63)
168 - 184	647.3096	1938.9070	1938.9034	2	0	K.YSHVDLVVMVDGFEGEK.G	Oxidation (M)	(Ions score 59)
168 - 184	647.3096	1938.9070	1938.9034	2	0	K.YSHVDLVVMVDGFEGEK.G	Oxidation (M)	(Ions score 61)
168 - 184	647.3096	1938.9070	1938.9034	2	0	K.YSHVDLVVMVDGFEGEK.G	Oxidation (M)	(Ions score 62)
168 - 184	647.3096	1938.9070	1938.9034	2	0	K.YSHVDLVVMVDGFEGEK.G	Oxidation (M)	(Ions score 54)
168 - 184	647.3096	1938.9070	1938.9034	2	0	K.YSHVDLVVMVDGFEGEK.G	Oxidation (M)	(Ions score 69)
168 - 184	647.3096	1938.9070	1938.9034	2	0	K.YSHVDLVVMVDGFEGEK.G	Oxidation (M)	(Ions score 64)
168 - 184	647.3096	1938.9070	1938.9034	2	0	K.YSHVDLVVMVDGFEGEK.G	Oxidation (M)	(Ions score 61)
168 - 184	647.3096	1938.9070	1938.9034	2	0	K.YSHVDLVVMVDGFEGEK.G	Oxidation (M)	(Ions score 55)
168 - 184	647.3096	1938.9070	1938.9034	2	0	K.YSHVDLVVMVDGFEGEK.G	Oxidation (M)	(Ions score 62)
168 - 184	970.4608	1938.9070	1938.9034	2	0	K.YSHVDLVVMVDGFEGEK.G	Oxidation (M)	(Ions score 49)
168 - 184	647.3096	1938.9070	1938.9034	2	0	K.YSHVDLVVMVDGFEGEK.G	Oxidation (M)	(Ions score 69)
168 - 184	970.4608	1938.9070	1938.9034	2	0	K.YSHVDLVVMVDGFEGEK.G	Oxidation (M)	(Ions score 58)
168 - 184	647.3096	1938.9070	1938.9034	2	0	K.YSHVDLVVMVDGFEGEK.G	Oxidation (M)	(Ions score 42)
168 - 184	970.4608	1938.9070	1938.9034	2	0	K.YSHVDLVVMVDGFEGEK.G	Oxidation (M)	(Ions score 68)
168 - 184	647.3096	1938.9071	1938.9034	2	0	K.YSHVDLVVMVDGFEGEK.G	Oxidation (M)	(Ions score 44)
168 - 184	970.4608	1938.9071	1938.9034	2	0	K.YSHVDLVVMVDGFEGEK.G	Oxidation (M)	(Ions score 50)
168 - 184	647.3096	1938.9071	1938.9034	2	0	K.YSHVDLVVMVDGFEGEK.G	Oxidation (M)	(Ions score 36)
168 - 184	970.4608	1938.9071	1938.9034	2	0	K.YSHVDLVVMVDGFEGEK.G	Oxidation (M)	(Ions score 57)
168 - 184	970.4608	1938.9071	1938.9034	2	0	K.YSHVDLVVMVDGFEGEK.G	Oxidation (M)	(Ions score 63)
168 - 184	970.4608	1938.9071	1938.9034	2	0	K.YSHVDLVVMVDGFEGEK.G	Oxidation (M)	(Ions score 54)
168 - 184	970.4608	1938.9071	1938.9034	2	0	K.YSHVDLVVMVDGFEGEK.G	Oxidation (M)	(Ions score 51)
168 - 184	647.3096	1938.9071	1938.9034	2	0	K.YSHVDLVVMVDGFEGEK.G	Oxidation (M)	(Ions score 69)
168 - 184	647.3096	1938.9071	1938.9034	2	0	K.YSHVDLVVMVDGFEGEK.G	Oxidation (M)	(Ions score 58)
168 - 184	647.3096	1938.9071	1938.9034	2	0	K.YSHVDLVVMVDGFEGEK.G	Oxidation (M)	(Ions score 69)
168 - 184	970.4608	1938.9071	1938.9034	2	0	K.YSHVDLVVMVDGFEGEK.G	Oxidation (M)	(Ions score 63)
168 - 184	647.3096	1938.9071	1938.9034	2	0	K.YSHVDLVVMVDGFEGEK.G	Oxidation (M)	(Ions score 72)
168 - 184	647.3096	1938.9071	1938.9034	2	0	K.YSHVDLVVMVDGFEGEK.G	Oxidation (M)	(Ions score 70)
168 - 184	647.3097	1938.9071	1938.9034	2	0	K.YSHVDLVVMVDGFEGEK.G	Oxidation (M)	(Ions score 63)

168 - 184	647.3097	1938.9071	1938.9034	2	0	K.YSHVDLVVMVDGFEGEK.G	Oxidation (M)	(Ions score 64)
168 - 184	970.4608	1938.9071	1938.9034	2	0	K.YSHVDLVVMVDGFEGEK.G	Oxidation (M)	(Ions score 72)
168 - 184	647.3097	1938.9071	1938.9034	2	0	K.YSHVDLVVMVDGFEGEK.G	Oxidation (M)	(Ions score 73)
168 - 184	970.4609	1938.9072	1938.9034	2	0	K.YSHVDLVVMVDGFEGEK.G	Oxidation (M)	(Ions score 62)
168 - 184	647.3097	1938.9072	1938.9034	2	0	K.YSHVDLVVMVDGFEGEK.G	Oxidation (M)	(Ions score 35)
168 - 184	970.4609	1938.9072	1938.9034	2	0	K.YSHVDLVVMVDGFEGEK.G	Oxidation (M)	(Ions score 78)
168 - 184	647.3097	1938.9072	1938.9034	2	0	K.YSHVDLVVMVDGFEGEK.G	Oxidation (M)	(Ions score 69)
168 - 184	647.3097	1938.9072	1938.9034	2	0	K.YSHVDLVVMVDGFEGEK.G	Oxidation (M)	(Ions score 62)
168 - 184	970.4609	1938.9072	1938.9034	2	0	K.YSHVDLVVMVDGFEGEK.G	Oxidation (M)	(Ions score 49)
168 - 184	647.3097	1938.9072	1938.9034	2	0	K.YSHVDLVVMVDGFEGEK.G	Oxidation (M)	(Ions score 59)
168 - 184	970.4609	1938.9072	1938.9034	2	0	K.YSHVDLVVMVDGFEGEK.G	Oxidation (M)	(Ions score 58)
168 - 184	647.3097	1938.9072	1938.9034	2	0	K.YSHVDLVVMVDGFEGEK.G	Oxidation (M)	(Ions score 57)
168 - 184	970.4609	1938.9072	1938.9034	2	0	K.YSHVDLVVMVDGFEGEK.G	Oxidation (M)	(Ions score 55)
168 - 184	647.3097	1938.9072	1938.9034	2	0	K.YSHVDLVVMVDGFEGEK.G	Oxidation (M)	(Ions score 66)
168 - 184	970.4609	1938.9072	1938.9034	2	0	K.YSHVDLVVMVDGFEGEK.G	Oxidation (M)	(Ions score 59)
168 - 184	647.3097	1938.9072	1938.9034	2	0	K.YSHVDLVVMVDGFEGEK.G	Oxidation (M)	(Ions score 58)
168 - 184	647.3097	1938.9072	1938.9034	2	0	K.YSHVDLVVMVDGFEGEK.G	Oxidation (M)	(Ions score 61)
168 - 184	647.3097	1938.9072	1938.9034	2	0	K.YSHVDLVVMVDGFEGEK.G	Oxidation (M)	(Ions score 58)
168 - 184	647.3097	1938.9072	1938.9034	2	0	K.YSHVDLVVMVDGFEGEK.G	Oxidation (M)	(Ions score 61)
168 - 184	647.3097	1938.9072	1938.9034	2	0	K.YSHVDLVVMVDGFEGEK.G	Oxidation (M)	(Ions score 69)
168 - 184	647.3097	1938.9072	1938.9034	2	0	K.YSHVDLVVMVDGFEGEK.G	Oxidation (M)	(Ions score 51)
168 - 184	647.3097	1938.9073	1938.9034	2	0	K.YSHVDLVVMVDGFEGEK.G	Oxidation (M)	(Ions score 69)
168 - 184	970.4609	1938.9073	1938.9034	2	0	K.YSHVDLVVMVDGFEGEK.G	Oxidation (M)	(Ions score 57)
168 - 184	970.4609	1938.9073	1938.9034	2	0	K.YSHVDLVVMVDGFEGEK.G	Oxidation (M)	(Ions score 50)
168 - 184	647.3097	1938.9073	1938.9034	2	0	K.YSHVDLVVMVDGFEGEK.G	Oxidation (M)	(Ions score 58)
168 - 184	647.3097	1938.9073	1938.9034	2	0	K.YSHVDLVVMVDGFEGEK.G	Oxidation (M)	(Ions score 58)
168 - 184	647.3097	1938.9073	1938.9034	2	0	K.YSHVDLVVMVDGFEGEK.G	Oxidation (M)	(Ions score 63)
168 - 184	970.4609	1938.9073	1938.9034	2	0	K.YSHVDLVVMVDGFEGEK.G	Oxidation (M)	(Ions score 72)
168 - 184	647.3097	1938.9073	1938.9034	2	0	K.YSHVDLVVMVDGFEGEK.G	Oxidation (M)	(Ions score 52)
168 - 184	970.4609	1938.9073	1938.9034	2	0	K.YSHVDLVVMVDGFEGEK.G	Oxidation (M)	(Ions score 58)
168 - 184	647.3097	1938.9073	1938.9034	2	0	K.YSHVDLVVMVDGFEGEK.G	Oxidation (M)	(Ions score 66)
168 - 184	647.3097	1938.9073	1938.9034	2	0	K.YSHVDLVVMVDGFEGEK.G	Oxidation (M)	(Ions score 59)
168 - 184	647.3097	1938.9073	1938.9034	2	0	K.YSHVDLVVMVDGFEGEK.G	Oxidation (M)	(Ions score 62)
168 - 184	647.3097	1938.9073	1938.9034	2	0	K.YSHVDLVVMVDGFEGEK.G	Oxidation (M)	(Ions score 56)
168 - 184	647.3097	1938.9073	1938.9034	2	0	K.YSHVDLVVMVDGFEGEK.G	Oxidation (M)	(Ions score 49)
168 - 184	647.3097	1938.9073	1938.9034	2	0	K.YSHVDLVVMVDGFEGEK.G	Oxidation (M)	(Ions score 58)
168 - 184	647.3097	1938.9073	1938.9034	2	0	K.YSHVDLVVMVDGFEGEK.G	Oxidation (M)	(Ions score 58)
168 - 184	970.4609	1938.9073	1938.9034	2	0	K.YSHVDLVVMVDGFEGEK.G	Oxidation (M)	(Ions score 57)
168 - 184	647.3097	1938.9073	1938.9034	2	0	K.YSHVDLVVMVDGFEGEK.G	Oxidation (M)	(Ions score 69)
168 - 184	647.3097	1938.9073	1938.9034	2	0	K.YSHVDLVVMVDGFEGEK.G	Oxidation (M)	(Ions score 52)
168 - 184	647.3097	1938.9073	1938.9034	2	0	K.YSHVDLVVMVDGFEGEK.G	Oxidation (M)	(Ions score 66)
168 - 184	647.3097	1938.9074	1938.9034	2	0	K.YSHVDLVVMVDGFEGEK.G	Oxidation (M)	(Ions score 54)
168 - 184	647.3097	1938.9074	1938.9034	2	0	K.YSHVDLVVMVDGFEGEK.G	Oxidation (M)	(Ions score 58)
168 - 184	647.3097	1938.9074	1938.9034	2	0	K.YSHVDLVVMVDGFEGEK.G	Oxidation (M)	(Ions score 52)
168 - 184	647.3097	1938.9074	1938.9034	2	0	K.YSHVDLVVMVDGFEGEK.G	Oxidation (M)	(Ions score 56)
168 - 184	647.3097	1938.9074	1938.9034	2	0	K.YSHVDLVVMVDGFEGEK.G	Oxidation (M)	(Ions score 63)
168 - 184	647.3097	1938.9074	1938.9034	2	0	K.YSHVDLVVMVDGFEGEK.G	Oxidation (M)	(Ions score 69)
168 - 184	647.3097	1938.9074	1938.9034	2	0	K.YSHVDLVVMVDGFEGEK.G	Oxidation (M)	(Ions score 34)
168 - 184	970.4610	1938.9074	1938.9034	2	0	K.YSHVDLVVMVDGFEGEK.G	Oxidation (M)	(Ions score 59)
168 - 184	647.3097	1938.9074	1938.9034	2	0	K.YSHVDLVVMVDGFEGEK.G	Oxidation (M)	(Ions score 81)
168 - 184	970.4610	1938.9074	1938.9034	2	0	K.YSHVDLVVMVDGFEGEK.G	Oxidation (M)	(Ions score 56)
168 - 184	647.3098	1938.9074	1938.9034	2	0	K.YSHVDLVVMVDGFEGEK.G	Oxidation (M)	(Ions score 62)
168 - 184	647.3098	1938.9074	1938.9034	2	0	K.YSHVDLVVMVDGFEGEK.G	Oxidation (M)	(Ions score 61)
168 - 184	970.4610	1938.9074	1938.9034	2	0	K.YSHVDLVVMVDGFEGEK.G	Oxidation (M)	(Ions score 72)
168 - 184	647.3098	1938.9074	1938.9034	2	0	K.YSHVDLVVMVDGFEGEK.G	Oxidation (M)	(Ions score 61)
168 - 184	647.3098	1938.9074	1938.9034	2	0	K.YSHVDLVVMVDGFEGEK.G	Oxidation (M)	(Ions score 69)
168 - 184	647.3098	1938.9074	1938.9034	2	0	K.YSHVDLVVMVDGFEGEK.G	Oxidation (M)	(Ions score 59)

168 - 184	647.3098	1938.9075	1938.9034	2	0	K.YSHVDLVVMVDGFEGEK.G	Oxidation (M)	(Ions score 58)
168 - 184	647.3098	1938.9075	1938.9034	2	0	K.YSHVDLVVMVDGFEGEK.G	Oxidation (M)	(Ions score 59)
168 - 184	647.3098	1938.9075	1938.9034	2	0	K.YSHVDLVVMVDGFEGEK.G	Oxidation (M)	(Ions score 58)
168 - 184	647.3098	1938.9075	1938.9034	2	0	K.YSHVDLVVMVDGFEGEK.G	Oxidation (M)	(Ions score 56)
168 - 184	647.3098	1938.9075	1938.9034	2	0	K.YSHVDLVVMVDGFEGEK.G	Oxidation (M)	(Ions score 72)
168 - 184	647.3098	1938.9075	1938.9034	2	0	K.YSHVDLVVMVDGFEGEK.G	Oxidation (M)	(Ions score 60)
168 - 184	647.3098	1938.9075	1938.9034	2	0	K.YSHVDLVVMVDGFEGEK.G	Oxidation (M)	(Ions score 65)
168 - 184	647.3098	1938.9075	1938.9034	2	0	K.YSHVDLVVMVDGFEGEK.G	Oxidation (M)	(Ions score 54)
168 - 184	647.3098	1938.9075	1938.9034	2	0	K.YSHVDLVVMVDGFEGEK.G	Oxidation (M)	(Ions score 64)
168 - 184	970.4610	1938.9075	1938.9034	2	0	K.YSHVDLVVMVDGFEGEK.G	Oxidation (M)	(Ions score 64)
168 - 184	647.3098	1938.9075	1938.9034	2	0	K.YSHVDLVVMVDGFEGEK.G	Oxidation (M)	(Ions score 69)
168 - 184	647.3098	1938.9075	1938.9034	2	0	K.YSHVDLVVMVDGFEGEK.G	Oxidation (M)	(Ions score 69)
168 - 184	970.4611	1938.9075	1938.9034	2	0	K.YSHVDLVVMVDGFEGEK.G	Oxidation (M)	(Ions score 55)
168 - 184	647.3098	1938.9076	1938.9034	2	0	K.YSHVDLVVMVDGFEGEK.G	Oxidation (M)	(Ions score 44)
168 - 184	647.3098	1938.9076	1938.9034	2	0	K.YSHVDLVVMVDGFEGEK.G	Oxidation (M)	(Ions score 55)
168 - 184	647.3098	1938.9076	1938.9034	2	0	K.YSHVDLVVMVDGFEGEK.G	Oxidation (M)	(Ions score 61)
168 - 184	647.3098	1938.9076	1938.9034	2	0	K.YSHVDLVVMVDGFEGEK.G	Oxidation (M)	(Ions score 61)
168 - 184	647.3098	1938.9076	1938.9034	2	0	K.YSHVDLVVMVDGFEGEK.G	Oxidation (M)	(Ions score 50)
168 - 184	647.3098	1938.9076	1938.9034	2	0	K.YSHVDLVVMVDGFEGEK.G	Oxidation (M)	(Ions score 34)
168 - 184	970.4611	1938.9076	1938.9034	2	0	K.YSHVDLVVMVDGFEGEK.G	Oxidation (M)	(Ions score 57)
168 - 184	647.3098	1938.9076	1938.9034	2	0	K.YSHVDLVVMVDGFEGEK.G	Oxidation (M)	(Ions score 52)
168 - 184	970.4611	1938.9076	1938.9034	2	0	K.YSHVDLVVMVDGFEGEK.G	Oxidation (M)	(Ions score 47)
168 - 184	970.4611	1938.9076	1938.9034	2	0	K.YSHVDLVVMVDGFEGEK.G	Oxidation (M)	(Ions score 72)
168 - 184	970.4611	1938.9076	1938.9034	2	0	K.YSHVDLVVMVDGFEGEK.G	Oxidation (M)	(Ions score 42)
168 - 184	647.3098	1938.9076	1938.9034	2	0	K.YSHVDLVVMVDGFEGEK.G	Oxidation (M)	(Ions score 44)
168 - 184	647.3098	1938.9076	1938.9034	2	0	K.YSHVDLVVMVDGFEGEK.G	Oxidation (M)	(Ions score 61)
168 - 184	647.3098	1938.9077	1938.9034	2	0	K.YSHVDLVVMVDGFEGEK.G	Oxidation (M)	(Ions score 59)
168 - 184	970.4611	1938.9077	1938.9034	2	0	K.YSHVDLVVMVDGFEGEK.G	Oxidation (M)	(Ions score 59)
168 - 184	647.3098	1938.9077	1938.9034	2	0	K.YSHVDLVVMVDGFEGEK.G	Oxidation (M)	(Ions score 61)
168 - 184	647.3098	1938.9077	1938.9034	2	0	K.YSHVDLVVMVDGFEGEK.G	Oxidation (M)	(Ions score 54)
168 - 184	970.4611	1938.9077	1938.9034	2	0	K.YSHVDLVVMVDGFEGEK.G	Oxidation (M)	(Ions score 56)
168 - 184	647.3099	1938.9077	1938.9034	2	0	K.YSHVDLVVMVDGFEGEK.G	Oxidation (M)	(Ions score 64)
168 - 184	647.3099	1938.9077	1938.9034	2	0	K.YSHVDLVVMVDGFEGEK.G	Oxidation (M)	(Ions score 69)
168 - 184	647.3099	1938.9077	1938.9034	2	0	K.YSHVDLVVMVDGFEGEK.G	Oxidation (M)	(Ions score 56)
168 - 184	647.3099	1938.9077	1938.9034	2	0	K.YSHVDLVVMVDGFEGEK.G	Oxidation (M)	(Ions score 58)
168 - 184	970.4611	1938.9077	1938.9034	2	0	K.YSHVDLVVMVDGFEGEK.G	Oxidation (M)	(Ions score 58)
168 - 184	970.4611	1938.9077	1938.9034	2	0	K.YSHVDLVVMVDGFEGEK.G	Oxidation (M)	(Ions score 53)
168 - 184	647.3099	1938.9078	1938.9034	2	0	K.YSHVDLVVMVDGFEGEK.G	Oxidation (M)	(Ions score 59)
168 - 184	647.3099	1938.9078	1938.9034	2	0	K.YSHVDLVVMVDGFEGEK.G	Oxidation (M)	(Ions score 61)
168 - 184	970.4612	1938.9078	1938.9034	2	0	K.YSHVDLVVMVDGFEGEK.G	Oxidation (M)	(Ions score 68)
168 - 184	647.3099	1938.9078	1938.9034	2	0	K.YSHVDLVVMVDGFEGEK.G	Oxidation (M)	(Ions score 43)
168 - 184	970.4612	1938.9078	1938.9034	2	0	K.YSHVDLVVMVDGFEGEK.G	Oxidation (M)	(Ions score 42)
168 - 184	647.3099	1938.9078	1938.9034	2	0	K.YSHVDLVVMVDGFEGEK.G	Oxidation (M)	(Ions score 60)
168 - 184	647.3099	1938.9078	1938.9034	2	0	K.YSHVDLVVMVDGFEGEK.G	Oxidation (M)	(Ions score 61)
168 - 184	647.3099	1938.9078	1938.9034	2	0	K.YSHVDLVVMVDGFEGEK.G	Oxidation (M)	(Ions score 57)
168 - 184	647.3099	1938.9078	1938.9034	2	0	K.YSHVDLVVMVDGFEGEK.G	Oxidation (M)	(Ions score 61)
168 - 184	970.4612	1938.9078	1938.9034	2	0	K.YSHVDLVVMVDGFEGEK.G	Oxidation (M)	(Ions score 61)
168 - 184	970.4612	1938.9078	1938.9034	2	0	K.YSHVDLVVMVDGFEGEK.G	Oxidation (M)	(Ions score 68)
168 - 184	647.3099	1938.9078	1938.9034	2	0	K.YSHVDLVVMVDGFEGEK.G	Oxidation (M)	(Ions score 40)
168 - 184	647.3099	1938.9078	1938.9034	2	0	K.YSHVDLVVMVDGFEGEK.G	Oxidation (M)	(Ions score 53)
168 - 184	970.4612	1938.9078	1938.9034	2	0	K.YSHVDLVVMVDGFEGEK.G	Oxidation (M)	(Ions score 56)
168 - 184	647.3099	1938.9078	1938.9034	2	0	K.YSHVDLVVMVDGFEGEK.G	Oxidation (M)	(Ions score 56)
168 - 184	647.3099	1938.9078	1938.9034	2	0	K.YSHVDLVVMVDGFEGEK.G	Oxidation (M)	(Ions score 53)
168 - 184	647.3099	1938.9078	1938.9034	2	0	K.YSHVDLVVMVDGFEGEK.G	Oxidation (M)	(Ions score 69)
168 - 184	970.4612	1938.9078	1938.9034	2	0	K.YSHVDLVVMVDGFEGEK.G	Oxidation (M)	(Ions score 50)
168 - 184	647.3099	1938.9078	1938.9034	2	0	K.YSHVDLVVMVDGFEGEK.G	Oxidation (M)	(Ions score 54)
168 - 184	970.4612	1938.9078	1938.9034	2	0	K.YSHVDLVVMVDGFEGEK.G	Oxidation (M)	(Ions score 65)

168 - 184	970.4612	1938.9079	1938.9034	2	0	K.YSHVDLVVMVDGFEGEK.G	Oxidation (M)	(Ions score 60)
168 - 184	647.3099	1938.9079	1938.9034	2	0	K.YSHVDLVVMVDGFEGEK.G	Oxidation (M)	(Ions score 58)
168 - 184	970.4612	1938.9079	1938.9034	2	0	K.YSHVDLVVMVDGFEGEK.G	Oxidation (M)	(Ions score 56)
168 - 184	647.3099	1938.9079	1938.9034	2	0	K.YSHVDLVVMVDGFEGEK.G	Oxidation (M)	(Ions score 66)
168 - 184	970.4612	1938.9079	1938.9034	2	0	K.YSHVDLVVMVDGFEGEK.G	Oxidation (M)	(Ions score 54)
168 - 184	970.4612	1938.9079	1938.9034	2	0	K.YSHVDLVVMVDGFEGEK.G	Oxidation (M)	(Ions score 56)
168 - 184	970.4612	1938.9079	1938.9034	2	0	K.YSHVDLVVMVDGFEGEK.G	Oxidation (M)	(Ions score 57)
168 - 184	970.4612	1938.9079	1938.9034	2	0	K.YSHVDLVVMVDGFEGEK.G	Oxidation (M)	(Ions score 65)
168 - 184	647.3099	1938.9079	1938.9034	2	0	K.YSHVDLVVMVDGFEGEK.G	Oxidation (M)	(Ions score 59)
168 - 184	970.4612	1938.9079	1938.9034	2	0	K.YSHVDLVVMVDGFEGEK.G	Oxidation (M)	(Ions score 68)
168 - 184	647.3099	1938.9079	1938.9034	2	0	K.YSHVDLVVMVDGFEGEK.G	Oxidation (M)	(Ions score 61)
168 - 184	647.3099	1938.9079	1938.9034	2	0	K.YSHVDLVVMVDGFEGEK.G	Oxidation (M)	(Ions score 69)
168 - 184	647.3099	1938.9079	1938.9034	2	0	K.YSHVDLVVMVDGFEGEK.G	Oxidation (M)	(Ions score 63)
168 - 184	970.4613	1938.9080	1938.9034	2	0	K.YSHVDLVVMVDGFEGEK.G	Oxidation (M)	(Ions score 52)
168 - 184	970.4613	1938.9080	1938.9034	2	0	K.YSHVDLVVMVDGFEGEK.G	Oxidation (M)	(Ions score 53)
168 - 184	970.4613	1938.9080	1938.9034	2	0	K.YSHVDLVVMVDGFEGEK.G	Oxidation (M)	(Ions score 56)
168 - 184	970.4613	1938.9080	1938.9034	2	0	K.YSHVDLVVMVDGFEGEK.G	Oxidation (M)	(Ions score 58)
168 - 184	970.4613	1938.9080	1938.9034	2	0	K.YSHVDLVVMVDGFEGEK.G	Oxidation (M)	(Ions score 58)
168 - 184	970.4613	1938.9080	1938.9034	2	0	K.YSHVDLVVMVDGFEGEK.G	Oxidation (M)	(Ions score 41)
168 - 184	647.3100	1938.9080	1938.9034	2	0	K.YSHVDLVVMVDGFEGEK.G	Oxidation (M)	(Ions score 54)
168 - 184	647.3100	1938.9080	1938.9034	2	0	K.YSHVDLVVMVDGFEGEK.G	Oxidation (M)	(Ions score 66)
168 - 184	647.3100	1938.9080	1938.9034	2	0	K.YSHVDLVVMVDGFEGEK.G	Oxidation (M)	(Ions score 64)
168 - 184	970.4613	1938.9081	1938.9034	2	0	K.YSHVDLVVMVDGFEGEK.G	Oxidation (M)	(Ions score 52)
168 - 184	647.3100	1938.9081	1938.9034	2	0	K.YSHVDLVVMVDGFEGEK.G	Oxidation (M)	(Ions score 58)
168 - 184	970.4613	1938.9081	1938.9034	2	0	K.YSHVDLVVMVDGFEGEK.G	Oxidation (M)	(Ions score 54)
168 - 184	647.3100	1938.9081	1938.9034	2	0	K.YSHVDLVVMVDGFEGEK.G	Oxidation (M)	(Ions score 55)
168 - 184	647.3100	1938.9081	1938.9034	2	0	K.YSHVDLVVMVDGFEGEK.G	Oxidation (M)	(Ions score 56)
168 - 184	970.4613	1938.9081	1938.9034	2	0	K.YSHVDLVVMVDGFEGEK.G	Oxidation (M)	(Ions score 60)
168 - 184	647.3100	1938.9081	1938.9034	2	0	K.YSHVDLVVMVDGFEGEK.G	Oxidation (M)	(Ions score 47)
168 - 184	647.3100	1938.9081	1938.9034	2	0	K.YSHVDLVVMVDGFEGEK.G	Oxidation (M)	(Ions score 64)
168 - 184	970.4613	1938.9081	1938.9034	2	0	K.YSHVDLVVMVDGFEGEK.G	Oxidation (M)	(Ions score 74)
168 - 184	970.4613	1938.9081	1938.9034	2	0	K.YSHVDLVVMVDGFEGEK.G	Oxidation (M)	(Ions score 67)
168 - 184	970.4613	1938.9081	1938.9034	2	0	K.YSHVDLVVMVDGFEGEK.G	Oxidation (M)	(Ions score 50)
168 - 184	647.3100	1938.9081	1938.9034	2	0	K.YSHVDLVVMVDGFEGEK.G	Oxidation (M)	(Ions score 72)
168 - 184	647.3100	1938.9081	1938.9034	2	0	K.YSHVDLVVMVDGFEGEK.G	Oxidation (M)	(Ions score 48)
168 - 184	970.4613	1938.9081	1938.9034	2	0	K.YSHVDLVVMVDGFEGEK.G	Oxidation (M)	(Ions score 55)
168 - 184	647.3100	1938.9082	1938.9034	2	0	K.YSHVDLVVMVDGFEGEK.G	Oxidation (M)	(Ions score 69)
168 - 184	647.3100	1938.9082	1938.9034	2	0	K.YSHVDLVVMVDGFEGEK.G	Oxidation (M)	(Ions score 58)
168 - 184	647.3100	1938.9082	1938.9034	2	0	K.YSHVDLVVMVDGFEGEK.G	Oxidation (M)	(Ions score 34)
168 - 184	647.3100	1938.9082	1938.9034	2	0	K.YSHVDLVVMVDGFEGEK.G	Oxidation (M)	(Ions score 54)
168 - 184	647.3100	1938.9082	1938.9034	2	0	K.YSHVDLVVMVDGFEGEK.G	Oxidation (M)	(Ions score 66)
168 - 184	647.3100	1938.9082	1938.9034	2	0	K.YSHVDLVVMVDGFEGEK.G	Oxidation (M)	(Ions score 61)
168 - 184	647.3100	1938.9082	1938.9034	2	0	K.YSHVDLVVMVDGFEGEK.G	Oxidation (M)	(Ions score 42)
168 - 184	970.4614	1938.9082	1938.9034	2	0	K.YSHVDLVVMVDGFEGEK.G	Oxidation (M)	(Ions score 58)
168 - 184	970.4614	1938.9082	1938.9034	2	0	K.YSHVDLVVMVDGFEGEK.G	Oxidation (M)	(Ions score 47)
168 - 184	970.4614	1938.9082	1938.9034	2	0	K.YSHVDLVVMVDGFEGEK.G	Oxidation (M)	(Ions score 60)
168 - 184	970.4614	1938.9082	1938.9034	2	0	K.YSHVDLVVMVDGFEGEK.G	Oxidation (M)	(Ions score 50)
168 - 184	647.3100	1938.9082	1938.9034	3	0	K.YSHVDLVVMVDGFEGEK.G	Oxidation (M)	(Ions score 47)
168 - 184	970.4614	1938.9082	1938.9034	3	0	K.YSHVDLVVMVDGFEGEK.G	Oxidation (M)	(Ions score 58)
168 - 184	970.4614	1938.9082	1938.9034	3	0	K.YSHVDLVVMVDGFEGEK.G	Oxidation (M)	(Ions score 53)
168 - 184	647.3100	1938.9082	1938.9034	3	0	K.YSHVDLVVMVDGFEGEK.G	Oxidation (M)	(Ions score 65)
168 - 184	970.4614	1938.9082	1938.9034	3	0	K.YSHVDLVVMVDGFEGEK.G	Oxidation (M)	(Ions score 63)
168 - 184	647.3100	1938.9083	1938.9034	3	0	K.YSHVDLVVMVDGFEGEK.G	Oxidation (M)	(Ions score 50)
168 - 184	970.4614	1938.9083	1938.9034	3	0	K.YSHVDLVVMVDGFEGEK.G	Oxidation (M)	(Ions score 51)
168 - 184	647.3100	1938.9083	1938.9034	3	0	K.YSHVDLVVMVDGFEGEK.G	Oxidation (M)	(Ions score 61)
168 - 184	647.3100	1938.9083	1938.9034	3	0	K.YSHVDLVVMVDGFEGEK.G	Oxidation (M)	(Ions score 49)
168 - 184	970.4614	1938.9083	1938.9034	3	0	K.YSHVDLVVMVDGFEGEK.G	Oxidation (M)	(Ions score 56)

168 - 184	647.3101	1938.9083	1938.9034	3	0	K.YSHVDLVVMVDGFEGEK.G	Oxidation (M)	(Ions score 53)
168 - 184	647.3101	1938.9083	1938.9034	3	0	K.YSHVDLVVMVDGFEGEK.G	Oxidation (M)	(Ions score 39)
168 - 184	647.3101	1938.9084	1938.9034	3	0	K.YSHVDLVVMVDGFEGEK.G	Oxidation (M)	(Ions score 58)
168 - 184	647.3101	1938.9084	1938.9034	3	0	K.YSHVDLVVMVDGFEGEK.G	Oxidation (M)	(Ions score 66)
168 - 184	647.3101	1938.9084	1938.9034	3	0	K.YSHVDLVVMVDGFEGEK.G	Oxidation (M)	(Ions score 61)
168 - 184	647.3101	1938.9084	1938.9034	3	0	K.YSHVDLVVMVDGFEGEK.G	Oxidation (M)	(Ions score 64)
168 - 184	647.3101	1938.9084	1938.9034	3	0	K.YSHVDLVVMVDGFEGEK.G	Oxidation (M)	(Ions score 58)
168 - 184	647.3101	1938.9084	1938.9034	3	0	K.YSHVDLVVMVDGFEGEK.G	Oxidation (M)	(Ions score 59)
168 - 184	647.3101	1938.9084	1938.9034	3	0	K.YSHVDLVVMVDGFEGEK.G	Oxidation (M)	(Ions score 56)
168 - 184	647.3101	1938.9084	1938.9034	3	0	K.YSHVDLVVMVDGFEGEK.G	Oxidation (M)	(Ions score 68)
168 - 184	647.3101	1938.9084	1938.9034	3	0	K.YSHVDLVVMVDGFEGEK.G	Oxidation (M)	(Ions score 58)
168 - 184	647.3101	1938.9085	1938.9034	3	0	K.YSHVDLVVMVDGFEGEK.G	Oxidation (M)	(Ions score 63)
168 - 184	647.3101	1938.9085	1938.9034	3	0	K.YSHVDLVVMVDGFEGEK.G	Oxidation (M)	(Ions score 53)
168 - 184	647.3101	1938.9085	1938.9034	3	0	K.YSHVDLVVMVDGFEGEK.G	Oxidation (M)	(Ions score 66)
168 - 184	647.3101	1938.9085	1938.9034	3	0	K.YSHVDLVVMVDGFEGEK.G	Oxidation (M)	(Ions score 59)
168 - 184	647.3101	1938.9085	1938.9034	3	0	K.YSHVDLVVMVDGFEGEK.G	Oxidation (M)	(Ions score 53)
168 - 184	647.3101	1938.9085	1938.9034	3	0	K.YSHVDLVVMVDGFEGEK.G	Oxidation (M)	(Ions score 53)
168 - 184	647.3101	1938.9085	1938.9034	3	0	K.YSHVDLVVMVDGFEGEK.G	Oxidation (M)	(Ions score 69)
168 - 184	970.4615	1938.9085	1938.9034	3	0	K.YSHVDLVVMVDGFEGEK.G	Oxidation (M)	(Ions score 60)
168 - 184	970.4615	1938.9085	1938.9034	3	0	K.YSHVDLVVMVDGFEGEK.G	Oxidation (M)	(Ions score 48)
168 - 184	970.4616	1938.9086	1938.9034	3	0	K.YSHVDLVVMVDGFEGEK.G	Oxidation (M)	(Ions score 60)
168 - 184	647.3101	1938.9086	1938.9034	3	0	K.YSHVDLVVMVDGFEGEK.G	Oxidation (M)	(Ions score 58)
168 - 184	970.4616	1938.9086	1938.9034	3	0	K.YSHVDLVVMVDGFEGEK.G	Oxidation (M)	(Ions score 57)
168 - 184	970.4616	1938.9086	1938.9034	3	0	K.YSHVDLVVMVDGFEGEK.G	Oxidation (M)	(Ions score 72)
168 - 184	647.3101	1938.9086	1938.9034	3	0	K.YSHVDLVVMVDGFEGEK.G	Oxidation (M)	(Ions score 58)
168 - 184	647.3101	1938.9086	1938.9034	3	0	K.YSHVDLVVMVDGFEGEK.G	Oxidation (M)	(Ions score 69)
168 - 184	647.3101	1938.9086	1938.9034	3	0	K.YSHVDLVVMVDGFEGEK.G	Oxidation (M)	(Ions score 61)
168 - 184	970.4616	1938.9086	1938.9034	3	0	K.YSHVDLVVMVDGFEGEK.G	Oxidation (M)	(Ions score 58)
168 - 184	970.4616	1938.9086	1938.9034	3	0	K.YSHVDLVVMVDGFEGEK.G	Oxidation (M)	(Ions score 51)
168 - 184	647.3101	1938.9086	1938.9034	3	0	K.YSHVDLVVMVDGFEGEK.G	Oxidation (M)	(Ions score 61)
168 - 184	647.3101	1938.9086	1938.9034	3	0	K.YSHVDLVVMVDGFEGEK.G	Oxidation (M)	(Ions score 61)
168 - 184	647.3101	1938.9086	1938.9034	3	0	K.YSHVDLVVMVDGFEGEK.G	Oxidation (M)	(Ions score 64)
168 - 184	647.3102	1938.9086	1938.9034	3	0	K.YSHVDLVVMVDGFEGEK.G	Oxidation (M)	(Ions score 69)
168 - 184	647.3102	1938.9086	1938.9034	3	0	K.YSHVDLVVMVDGFEGEK.G	Oxidation (M)	(Ions score 50)
168 - 184	970.4616	1938.9086	1938.9034	3	0	K.YSHVDLVVMVDGFEGEK.G	Oxidation (M)	(Ions score 63)
168 - 184	647.3102	1938.9086	1938.9034	3	0	K.YSHVDLVVMVDGFEGEK.G	Oxidation (M)	(Ions score 61)
168 - 184	970.4616	1938.9086	1938.9034	3	0	K.YSHVDLVVMVDGFEGEK.G	Oxidation (M)	(Ions score 56)
168 - 184	970.4616	1938.9086	1938.9034	3	0	K.YSHVDLVVMVDGFEGEK.G	Oxidation (M)	(Ions score 61)
168 - 184	970.4616	1938.9086	1938.9034	3	0	K.YSHVDLVVMVDGFEGEK.G	Oxidation (M)	(Ions score 75)
168 - 184	647.3102	1938.9087	1938.9034	3	0	K.YSHVDLVVMVDGFEGEK.G	Oxidation (M)	(Ions score 69)
168 - 184	647.3102	1938.9087	1938.9034	3	0	K.YSHVDLVVMVDGFEGEK.G	Oxidation (M)	(Ions score 59)
168 - 184	970.4616	1938.9087	1938.9034	3	0	K.YSHVDLVVMVDGFEGEK.G	Oxidation (M)	(Ions score 56)
168 - 184	970.4616	1938.9087	1938.9034	3	0	K.YSHVDLVVMVDGFEGEK.G	Oxidation (M)	(Ions score 55)
168 - 184	970.4616	1938.9087	1938.9034	3	0	K.YSHVDLVVMVDGFEGEK.G	Oxidation (M)	(Ions score 47)
168 - 184	647.3102	1938.9087	1938.9034	3	0	K.YSHVDLVVMVDGFEGEK.G	Oxidation (M)	(Ions score 62)
168 - 184	970.4616	1938.9087	1938.9034	3	0	K.YSHVDLVVMVDGFEGEK.G	Oxidation (M)	(Ions score 59)
168 - 184	970.4616	1938.9087	1938.9034	3	0	K.YSHVDLVVMVDGFEGEK.G	Oxidation (M)	(Ions score 65)
168 - 184	647.3102	1938.9087	1938.9034	3	0	K.YSHVDLVVMVDGFEGEK.G	Oxidation (M)	(Ions score 42)
168 - 184	970.4616	1938.9087	1938.9034	3	0	K.YSHVDLVVMVDGFEGEK.G	Oxidation (M)	(Ions score 58)
168 - 184	647.3102	1938.9087	1938.9034	3	0	K.YSHVDLVVMVDGFEGEK.G	Oxidation (M)	(Ions score 66)
168 - 184	647.3102	1938.9087	1938.9034	3	0	K.YSHVDLVVMVDGFEGEK.G	Oxidation (M)	(Ions score 58)
168 - 184	970.4616	1938.9087	1938.9034	3	0	K.YSHVDLVVMVDGFEGEK.G	Oxidation (M)	(Ions score 60)
168 - 184	647.3102	1938.9087	1938.9034	3	0	K.YSHVDLVVMVDGFEGEK.G	Oxidation (M)	(Ions score 66)
168 - 184	970.4616	1938.9087	1938.9034	3	0	K.YSHVDLVVMVDGFEGEK.G	Oxidation (M)	(Ions score 72)
168 - 184	647.3102	1938.9088	1938.9034	3	0	K.YSHVDLVVMVDGFEGEK.G	Oxidation (M)	(Ions score 58)
168 - 184	647.3102	1938.9088	1938.9034	3	0	K.YSHVDLVVMVDGFEGEK.G	Oxidation (M)	(Ions score 58)
168 - 184	647.3102	1938.9088	1938.9034	3	0	K.YSHVDLVVMVDGFEGEK.G	Oxidation (M)	(Ions score 63)

168 - 184	970.4617	1938.9088	1938.9034	3	0	K.YSHVDLVVMVDGFEGEK.G	Oxidation (M)	(Ions score 63)
168 - 184	647.3102	1938.9088	1938.9034	3	0	K.YSHVDLVVMVDGFEGEK.G	Oxidation (M)	(Ions score 73)
168 - 184	970.4617	1938.9088	1938.9034	3	0	K.YSHVDLVVMVDGFEGEK.G	Oxidation (M)	(Ions score 49)
168 - 184	970.4617	1938.9088	1938.9034	3	0	K.YSHVDLVVMVDGFEGEK.G	Oxidation (M)	(Ions score 63)
168 - 184	647.3102	1938.9088	1938.9034	3	0	K.YSHVDLVVMVDGFEGEK.G	Oxidation (M)	(Ions score 58)
168 - 184	970.4617	1938.9088	1938.9034	3	0	K.YSHVDLVVMVDGFEGEK.G	Oxidation (M)	(Ions score 57)
168 - 184	647.3102	1938.9088	1938.9034	3	0	K.YSHVDLVVMVDGFEGEK.G	Oxidation (M)	(Ions score 61)
168 - 184	970.4617	1938.9088	1938.9034	3	0	K.YSHVDLVVMVDGFEGEK.G	Oxidation (M)	(Ions score 51)
168 - 184	647.3102	1938.9088	1938.9034	3	0	K.YSHVDLVVMVDGFEGEK.G	Oxidation (M)	(Ions score 52)
168 - 184	970.4617	1938.9089	1938.9034	3	0	K.YSHVDLVVMVDGFEGEK.G	Oxidation (M)	(Ions score 50)
168 - 184	647.3102	1938.9089	1938.9034	3	0	K.YSHVDLVVMVDGFEGEK.G	Oxidation (M)	(Ions score 59)
168 - 184	647.3102	1938.9089	1938.9034	3	0	K.YSHVDLVVMVDGFEGEK.G	Oxidation (M)	(Ions score 59)
168 - 184	647.3102	1938.9089	1938.9034	3	0	K.YSHVDLVVMVDGFEGEK.G	Oxidation (M)	(Ions score 51)
168 - 184	647.3102	1938.9089	1938.9034	3	0	K.YSHVDLVVMVDGFEGEK.G	Oxidation (M)	(Ions score 61)
168 - 184	970.4617	1938.9089	1938.9034	3	0	K.YSHVDLVVMVDGFEGEK.G	Oxidation (M)	(Ions score 73)
168 - 184	647.3103	1938.9089	1938.9034	3	0	K.YSHVDLVVMVDGFEGEK.G	Oxidation (M)	(Ions score 50)
168 - 184	647.3103	1938.9089	1938.9034	3	0	K.YSHVDLVVMVDGFEGEK.G	Oxidation (M)	(Ions score 54)
168 - 184	647.3103	1938.9089	1938.9034	3	0	K.YSHVDLVVMVDGFEGEK.G	Oxidation (M)	(Ions score 58)
168 - 184	970.4617	1938.9089	1938.9034	3	0	K.YSHVDLVVMVDGFEGEK.G	Oxidation (M)	(Ions score 59)
168 - 184	970.4618	1938.9090	1938.9034	3	0	K.YSHVDLVVMVDGFEGEK.G	Oxidation (M)	(Ions score 54)
168 - 184	970.4618	1938.9090	1938.9034	3	0	K.YSHVDLVVMVDGFEGEK.G	Oxidation (M)	(Ions score 51)
168 - 184	647.3103	1938.9090	1938.9034	3	0	K.YSHVDLVVMVDGFEGEK.G	Oxidation (M)	(Ions score 61)
168 - 184	970.4618	1938.9090	1938.9034	3	0	K.YSHVDLVVMVDGFEGEK.G	Oxidation (M)	(Ions score 62)
168 - 184	647.3103	1938.9090	1938.9034	3	0	K.YSHVDLVVMVDGFEGEK.G	Oxidation (M)	(Ions score 58)
168 - 184	647.3103	1938.9090	1938.9034	3	0	K.YSHVDLVVMVDGFEGEK.G	Oxidation (M)	(Ions score 58)
168 - 184	970.4618	1938.9090	1938.9034	3	0	K.YSHVDLVVMVDGFEGEK.G	Oxidation (M)	(Ions score 55)
168 - 184	970.4618	1938.9090	1938.9034	3	0	K.YSHVDLVVMVDGFEGEK.G	Oxidation (M)	(Ions score 58)
168 - 184	970.4618	1938.9090	1938.9034	3	0	K.YSHVDLVVMVDGFEGEK.G	Oxidation (M)	(Ions score 43)
168 - 184	647.3103	1938.9091	1938.9034	3	0	K.YSHVDLVVMVDGFEGEK.G	Oxidation (M)	(Ions score 58)
168 - 184	970.4618	1938.9091	1938.9034	3	0	K.YSHVDLVVMVDGFEGEK.G	Oxidation (M)	(Ions score 55)
168 - 184	647.3103	1938.9091	1938.9034	3	0	K.YSHVDLVVMVDGFEGEK.G	Oxidation (M)	(Ions score 58)
168 - 184	647.3103	1938.9091	1938.9034	3	0	K.YSHVDLVVMVDGFEGEK.G	Oxidation (M)	(Ions score 56)
168 - 184	647.3103	1938.9091	1938.9034	3	0	K.YSHVDLVVMVDGFEGEK.G	Oxidation (M)	(Ions score 60)
168 - 184	647.3103	1938.9091	1938.9034	3	0	K.YSHVDLVVMVDGFEGEK.G	Oxidation (M)	(Ions score 69)
168 - 184	970.4618	1938.9091	1938.9034	3	0	K.YSHVDLVVMVDGFEGEK.G	Oxidation (M)	(Ions score 56)
168 - 184	647.3103	1938.9091	1938.9034	3	0	K.YSHVDLVVMVDGFEGEK.G	Oxidation (M)	(Ions score 61)
168 - 184	970.4618	1938.9091	1938.9034	3	0	K.YSHVDLVVMVDGFEGEK.G	Oxidation (M)	(Ions score 71)
168 - 184	647.3103	1938.9091	1938.9034	3	0	K.YSHVDLVVMVDGFEGEK.G	Oxidation (M)	(Ions score 37)
168 - 184	970.4618	1938.9091	1938.9034	3	0	K.YSHVDLVVMVDGFEGEK.G	Oxidation (M)	(Ions score 70)
168 - 184	647.3103	1938.9091	1938.9034	3	0	K.YSHVDLVVMVDGFEGEK.G	Oxidation (M)	(Ions score 58)
168 - 184	970.4619	1938.9092	1938.9034	3	0	K.YSHVDLVVMVDGFEGEK.G	Oxidation (M)	(Ions score 58)
168 - 184	970.4619	1938.9092	1938.9034	3	0	K.YSHVDLVVMVDGFEGEK.G	Oxidation (M)	(Ions score 50)
168 - 184	970.4619	1938.9092	1938.9034	3	0	K.YSHVDLVVMVDGFEGEK.G	Oxidation (M)	(Ions score 49)
168 - 184	970.4619	1938.9092	1938.9034	3	0	K.YSHVDLVVMVDGFEGEK.G	Oxidation (M)	(Ions score 52)
168 - 184	647.3103	1938.9092	1938.9034	3	0	K.YSHVDLVVMVDGFEGEK.G	Oxidation (M)	(Ions score 53)
168 - 184	970.4619	1938.9092	1938.9034	3	0	K.YSHVDLVVMVDGFEGEK.G	Oxidation (M)	(Ions score 68)
168 - 184	647.3103	1938.9092	1938.9034	3	0	K.YSHVDLVVMVDGFEGEK.G	Oxidation (M)	(Ions score 63)
168 - 184	970.4619	1938.9092	1938.9034	3	0	K.YSHVDLVVMVDGFEGEK.G	Oxidation (M)	(Ions score 52)
168 - 184	970.4619	1938.9092	1938.9034	3	0	K.YSHVDLVVMVDGFEGEK.G	Oxidation (M)	(Ions score 56)
168 - 184	647.3103	1938.9092	1938.9034	3	0	K.YSHVDLVVMVDGFEGEK.G	Oxidation (M)	(Ions score 61)
168 - 184	647.3103	1938.9092	1938.9034	3	0	K.YSHVDLVVMVDGFEGEK.G	Oxidation (M)	(Ions score 58)
168 - 184	647.3103	1938.9092	1938.9034	3	0	K.YSHVDLVVMVDGFEGEK.G	Oxidation (M)	(Ions score 54)
168 - 184	647.3103	1938.9092	1938.9034	3	0	K.YSHVDLVVMVDGFEGEK.G	Oxidation (M)	(Ions score 58)
168 - 184	647.3103	1938.9092	1938.9034	3	0	K.YSHVDLVVMVDGFEGEK.G	Oxidation (M)	(Ions score 69)
168 - 184	647.3104	1938.9092	1938.9034	3	0	K.YSHVDLVVMVDGFEGEK.G	Oxidation (M)	(Ions score 58)
168 - 184	647.3104	1938.9092	1938.9034	3	0	K.YSHVDLVVMVDGFEGEK.G	Oxidation (M)	(Ions score 58)
168 - 184	647.3104	1938.9093	1938.9034	3	0	K.YSHVDLVVMVDGFEGEK.G	Oxidation (M)	(Ions score 58)

168 - 184	647.3104	1938.9093	1938.9034	3	0	K.YSHVDLVVMVDGFEGEK.G	Oxidation (M)	(Ions score 58)
168 - 184	647.3104	1938.9093	1938.9034	3	0	K.YSHVDLVVMVDGFEGEK.G	Oxidation (M)	(Ions score 56)
168 - 184	647.3104	1938.9093	1938.9034	3	0	K.YSHVDLVVMVDGFEGEK.G	Oxidation (M)	(Ions score 48)
168 - 184	970.4619	1938.9093	1938.9034	3	0	K.YSHVDLVVMVDGFEGEK.G	Oxidation (M)	(Ions score 49)
168 - 184	970.4619	1938.9093	1938.9034	3	0	K.YSHVDLVVMVDGFEGEK.G	Oxidation (M)	(Ions score 57)
168 - 184	970.4619	1938.9093	1938.9034	3	0	K.YSHVDLVVMVDGFEGEK.G	Oxidation (M)	(Ions score 51)
168 - 184	970.4619	1938.9093	1938.9034	3	0	K.YSHVDLVVMVDGFEGEK.G	Oxidation (M)	(Ions score 55)
168 - 184	647.3104	1938.9093	1938.9034	3	0	K.YSHVDLVVMVDGFEGEK.G	Oxidation (M)	(Ions score 59)
168 - 184	647.3104	1938.9094	1938.9034	3	0	K.YSHVDLVVMVDGFEGEK.G	Oxidation (M)	(Ions score 61)
168 - 184	647.3104	1938.9094	1938.9034	3	0	K.YSHVDLVVMVDGFEGEK.G	Oxidation (M)	(Ions score 58)
168 - 184	647.3104	1938.9094	1938.9034	3	0	K.YSHVDLVVMVDGFEGEK.G	Oxidation (M)	(Ions score 56)
168 - 184	647.3104	1938.9094	1938.9034	3	0	K.YSHVDLVVMVDGFEGEK.G	Oxidation (M)	(Ions score 51)
168 - 184	647.3104	1938.9094	1938.9034	3	0	K.YSHVDLVVMVDGFEGEK.G	Oxidation (M)	(Ions score 48)
168 - 184	970.4620	1938.9094	1938.9034	3	0	K.YSHVDLVVMVDGFEGEK.G	Oxidation (M)	(Ions score 72)
168 - 184	970.4620	1938.9094	1938.9034	3	0	K.YSHVDLVVMVDGFEGEK.G	Oxidation (M)	(Ions score 57)
168 - 184	647.3104	1938.9094	1938.9034	3	0	K.YSHVDLVVMVDGFEGEK.G	Oxidation (M)	(Ions score 69)
168 - 184	970.4620	1938.9094	1938.9034	3	0	K.YSHVDLVVMVDGFEGEK.G	Oxidation (M)	(Ions score 53)
168 - 184	647.3104	1938.9094	1938.9034	3	0	K.YSHVDLVVMVDGFEGEK.G	Oxidation (M)	(Ions score 61)
168 - 184	970.4620	1938.9094	1938.9034	3	0	K.YSHVDLVVMVDGFEGEK.G	Oxidation (M)	(Ions score 58)
168 - 184	970.4620	1938.9094	1938.9034	3	0	K.YSHVDLVVMVDGFEGEK.G	Oxidation (M)	(Ions score 55)
168 - 184	970.4620	1938.9095	1938.9034	3	0	K.YSHVDLVVMVDGFEGEK.G	Oxidation (M)	(Ions score 56)
168 - 184	647.3104	1938.9095	1938.9034	3	0	K.YSHVDLVVMVDGFEGEK.G	Oxidation (M)	(Ions score 56)
168 - 184	647.3104	1938.9095	1938.9034	3	0	K.YSHVDLVVMVDGFEGEK.G	Oxidation (M)	(Ions score 52)
168 - 184	970.4620	1938.9095	1938.9034	3	0	K.YSHVDLVVMVDGFEGEK.G	Oxidation (M)	(Ions score 56)
168 - 184	647.3105	1938.9095	1938.9034	3	0	K.YSHVDLVVMVDGFEGEK.G	Oxidation (M)	(Ions score 69)
168 - 184	970.4620	1938.9095	1938.9034	3	0	K.YSHVDLVVMVDGFEGEK.G	Oxidation (M)	(Ions score 57)
168 - 184	647.3105	1938.9095	1938.9034	3	0	K.YSHVDLVVMVDGFEGEK.G	Oxidation (M)	(Ions score 61)
168 - 184	970.4621	1938.9096	1938.9034	3	0	K.YSHVDLVVMVDGFEGEK.G	Oxidation (M)	(Ions score 65)
168 - 184	970.4621	1938.9096	1938.9034	3	0	K.YSHVDLVVMVDGFEGEK.G	Oxidation (M)	(Ions score 50)
168 - 184	970.4621	1938.9096	1938.9034	3	0	K.YSHVDLVVMVDGFEGEK.G	Oxidation (M)	(Ions score 68)
168 - 184	647.3105	1938.9096	1938.9034	3	0	K.YSHVDLVVMVDGFEGEK.G	Oxidation (M)	(Ions score 61)
168 - 184	970.4621	1938.9096	1938.9034	3	0	K.YSHVDLVVMVDGFEGEK.G	Oxidation (M)	(Ions score 64)
168 - 184	647.3105	1938.9097	1938.9034	3	0	K.YSHVDLVVMVDGFEGEK.G	Oxidation (M)	(Ions score 58)
168 - 184	647.3105	1938.9097	1938.9034	3	0	K.YSHVDLVVMVDGFEGEK.G	Oxidation (M)	(Ions score 63)
168 - 184	647.3105	1938.9097	1938.9034	3	0	K.YSHVDLVVMVDGFEGEK.G	Oxidation (M)	(Ions score 56)
168 - 184	970.4621	1938.9097	1938.9034	3	0	K.YSHVDLVVMVDGFEGEK.G	Oxidation (M)	(Ions score 70)
168 - 184	970.4621	1938.9097	1938.9034	3	0	K.YSHVDLVVMVDGFEGEK.G	Oxidation (M)	(Ions score 52)
168 - 184	647.3105	1938.9097	1938.9034	3	0	K.YSHVDLVVMVDGFEGEK.G	Oxidation (M)	(Ions score 66)
168 - 184	647.3105	1938.9097	1938.9034	3	0	K.YSHVDLVVMVDGFEGEK.G	Oxidation (M)	(Ions score 54)
168 - 184	647.3105	1938.9097	1938.9034	3	0	K.YSHVDLVVMVDGFEGEK.G	Oxidation (M)	(Ions score 57)
168 - 184	970.4622	1938.9098	1938.9034	3	0	K.YSHVDLVVMVDGFEGEK.G	Oxidation (M)	(Ions score 67)
168 - 184	970.4622	1938.9098	1938.9034	3	0	K.YSHVDLVVMVDGFEGEK.G	Oxidation (M)	(Ions score 62)
168 - 184	970.4622	1938.9098	1938.9034	3	0	K.YSHVDLVVMVDGFEGEK.G	Oxidation (M)	(Ions score 72)
168 - 184	970.4622	1938.9098	1938.9034	3	0	K.YSHVDLVVMVDGFEGEK.G	Oxidation (M)	(Ions score 68)
168 - 184	647.3105	1938.9098	1938.9034	3	0	K.YSHVDLVVMVDGFEGEK.G	Oxidation (M)	(Ions score 61)
168 - 184	970.4622	1938.9098	1938.9034	3	0	K.YSHVDLVVMVDGFEGEK.G	Oxidation (M)	(Ions score 53)
168 - 184	647.3105	1938.9098	1938.9034	3	0	K.YSHVDLVVMVDGFEGEK.G	Oxidation (M)	(Ions score 56)
168 - 184	970.4622	1938.9098	1938.9034	3	0	K.YSHVDLVVMVDGFEGEK.G	Oxidation (M)	(Ions score 76)
168 - 184	647.3105	1938.9098	1938.9034	3	0	K.YSHVDLVVMVDGFEGEK.G	Oxidation (M)	(Ions score 60)
168 - 184	970.4622	1938.9098	1938.9034	3	0	K.YSHVDLVVMVDGFEGEK.G	Oxidation (M)	(Ions score 50)
168 - 184	647.3106	1938.9098	1938.9034	3	0	K.YSHVDLVVMVDGFEGEK.G	Oxidation (M)	(Ions score 53)
168 - 184	647.3106	1938.9098	1938.9034	3	0	K.YSHVDLVVMVDGFEGEK.G	Oxidation (M)	(Ions score 58)
168 - 184	647.3106	1938.9098	1938.9034	3	0	K.YSHVDLVVMVDGFEGEK.G	Oxidation (M)	(Ions score 49)
168 - 184	970.4622	1938.9098	1938.9034	3	0	K.YSHVDLVVMVDGFEGEK.G	Oxidation (M)	(Ions score 62)
168 - 184	647.3106	1938.9098	1938.9034	3	0	K.YSHVDLVVMVDGFEGEK.G	Oxidation (M)	(Ions score 58)
168 - 184	970.4622	1938.9099	1938.9034	3	0	K.YSHVDLVVMVDGFEGEK.G	Oxidation (M)	(Ions score 59)
168 - 184	970.4622	1938.9099	1938.9034	3	0	K.YSHVDLVVMVDGFEGEK.G	Oxidation (M)	(Ions score 43)

168 - 184	647.3106	1938.9099	1938.9034	3	0	K.YSHVDLVVMVDGFEGEK.G	Oxidation (M)	(Ions score 66)
168 - 184	647.3106	1938.9099	1938.9034	3	0	K.YSHVDLVVMVDGFEGEK.G	Oxidation (M)	(Ions score 64)
168 - 184	970.4623	1938.9099	1938.9034	3	0	K.YSHVDLVVMVDGFEGEK.G	Oxidation (M)	(Ions score 67)
168 - 184	970.4623	1938.9099	1938.9034	3	0	K.YSHVDLVVMVDGFEGEK.G	Oxidation (M)	(Ions score 68)
168 - 184	970.4623	1938.9100	1938.9034	3	0	K.YSHVDLVVMVDGFEGEK.G	Oxidation (M)	(Ions score 56)
168 - 184	647.3106	1938.9100	1938.9034	3	0	K.YSHVDLVVMVDGFEGEK.G	Oxidation (M)	(Ions score 56)
168 - 184	970.4623	1938.9100	1938.9034	3	0	K.YSHVDLVVMVDGFEGEK.G	Oxidation (M)	(Ions score 54)
168 - 184	647.3106	1938.9100	1938.9034	3	0	K.YSHVDLVVMVDGFEGEK.G	Oxidation (M)	(Ions score 58)
168 - 184	970.4623	1938.9100	1938.9034	3	0	K.YSHVDLVVMVDGFEGEK.G	Oxidation (M)	(Ions score 61)
168 - 184	970.4623	1938.9100	1938.9034	3	0	K.YSHVDLVVMVDGFEGEK.G	Oxidation (M)	(Ions score 59)
168 - 184	970.4623	1938.9100	1938.9034	3	0	K.YSHVDLVVMVDGFEGEK.G	Oxidation (M)	(Ions score 54)
168 - 184	647.3106	1938.9100	1938.9034	3	0	K.YSHVDLVVMVDGFEGEK.G	Oxidation (M)	(Ions score 53)
168 - 184	647.3106	1938.9101	1938.9034	3	0	K.YSHVDLVVMVDGFEGEK.G	Oxidation (M)	(Ions score 58)
168 - 184	647.3106	1938.9101	1938.9034	3	0	K.YSHVDLVVMVDGFEGEK.G	Oxidation (M)	(Ions score 53)
168 - 184	970.4623	1938.9101	1938.9034	3	0	K.YSHVDLVVMVDGFEGEK.G	Oxidation (M)	(Ions score 63)
168 - 184	970.4623	1938.9101	1938.9034	3	0	K.YSHVDLVVMVDGFEGEK.G	Oxidation (M)	(Ions score 49)
168 - 184	970.4623	1938.9101	1938.9034	3	0	K.YSHVDLVVMVDGFEGEK.G	Oxidation (M)	(Ions score 55)
168 - 184	970.4623	1938.9101	1938.9034	3	0	K.YSHVDLVVMVDGFEGEK.G	Oxidation (M)	(Ions score 57)
168 - 184	970.4623	1938.9101	1938.9034	3	0	K.YSHVDLVVMVDGFEGEK.G	Oxidation (M)	(Ions score 48)
168 - 184	647.3107	1938.9102	1938.9034	4	0	K.YSHVDLVVMVDGFEGEK.G	Oxidation (M)	(Ions score 66)
168 - 184	647.3107	1938.9102	1938.9034	4	0	K.YSHVDLVVMVDGFEGEK.G	Oxidation (M)	(Ions score 30)
168 - 184	970.4624	1938.9102	1938.9034	4	0	K.YSHVDLVVMVDGFEGEK.G	Oxidation (M)	(Ions score 65)
168 - 184	970.4624	1938.9102	1938.9034	4	0	K.YSHVDLVVMVDGFEGEK.G	Oxidation (M)	(Ions score 61)
168 - 184	970.4624	1938.9102	1938.9034	4	0	K.YSHVDLVVMVDGFEGEK.G	Oxidation (M)	(Ions score 61)
168 - 184	970.4624	1938.9103	1938.9034	4	0	K.YSHVDLVVMVDGFEGEK.G	Oxidation (M)	(Ions score 63)
168 - 184	647.3107	1938.9103	1938.9034	4	0	K.YSHVDLVVMVDGFEGEK.G	Oxidation (M)	(Ions score 51)
168 - 184	647.3107	1938.9103	1938.9034	4	0	K.YSHVDLVVMVDGFEGEK.G	Oxidation (M)	(Ions score 48)
168 - 184	970.4624	1938.9103	1938.9034	4	0	K.YSHVDLVVMVDGFEGEK.G	Oxidation (M)	(Ions score 58)
168 - 184	647.3107	1938.9103	1938.9034	4	0	K.YSHVDLVVMVDGFEGEK.G	Oxidation (M)	(Ions score 58)
168 - 184	647.3107	1938.9104	1938.9034	4	0	K.YSHVDLVVMVDGFEGEK.G	Oxidation (M)	(Ions score 52)
168 - 184	647.3107	1938.9104	1938.9034	4	0	K.YSHVDLVVMVDGFEGEK.G	Oxidation (M)	(Ions score 58)
168 - 184	970.4625	1938.9104	1938.9034	4	0	K.YSHVDLVVMVDGFEGEK.G	Oxidation (M)	(Ions score 58)
168 - 184	970.4625	1938.9105	1938.9034	4	0	K.YSHVDLVVMVDGFEGEK.G	Oxidation (M)	(Ions score 55)
168 - 184	970.4625	1938.9105	1938.9034	4	0	K.YSHVDLVVMVDGFEGEK.G	Oxidation (M)	(Ions score 72)
168 - 184	647.3108	1938.9105	1938.9034	4	0	K.YSHVDLVVMVDGFEGEK.G	Oxidation (M)	(Ions score 61)
168 - 184	970.4625	1938.9105	1938.9034	4	0	K.YSHVDLVVMVDGFEGEK.G	Oxidation (M)	(Ions score 64)
168 - 184	647.3108	1938.9105	1938.9034	4	0	K.YSHVDLVVMVDGFEGEK.G	Oxidation (M)	(Ions score 58)
168 - 184	970.4625	1938.9105	1938.9034	4	0	K.YSHVDLVVMVDGFEGEK.G	Oxidation (M)	(Ions score 56)
168 - 184	647.3108	1938.9105	1938.9034	4	0	K.YSHVDLVVMVDGFEGEK.G	Oxidation (M)	(Ions score 59)
168 - 184	970.4625	1938.9105	1938.9034	4	0	K.YSHVDLVVMVDGFEGEK.G	Oxidation (M)	(Ions score 53)
168 - 184	647.3108	1938.9105	1938.9034	4	0	K.YSHVDLVVMVDGFEGEK.G	Oxidation (M)	(Ions score 81)
168 - 184	970.4625	1938.9105	1938.9034	4	0	K.YSHVDLVVMVDGFEGEK.G	Oxidation (M)	(Ions score 70)
168 - 184	970.4625	1938.9105	1938.9034	4	0	K.YSHVDLVVMVDGFEGEK.G	Oxidation (M)	(Ions score 62)
168 - 184	970.4625	1938.9105	1938.9034	4	0	K.YSHVDLVVMVDGFEGEK.G	Oxidation (M)	(Ions score 60)
168 - 184	970.4626	1938.9106	1938.9034	4	0	K.YSHVDLVVMVDGFEGEK.G	Oxidation (M)	(Ions score 69)
168 - 184	970.4626	1938.9106	1938.9034	4	0	K.YSHVDLVVMVDGFEGEK.G	Oxidation (M)	(Ions score 57)
168 - 184	970.4626	1938.9106	1938.9034	4	0	K.YSHVDLVVMVDGFEGEK.G	Oxidation (M)	(Ions score 71)
168 - 184	970.4626	1938.9106	1938.9034	4	0	K.YSHVDLVVMVDGFEGEK.G	Oxidation (M)	(Ions score 58)
168 - 184	970.4626	1938.9107	1938.9034	4	0	K.YSHVDLVVMVDGFEGEK.G	Oxidation (M)	(Ions score 50)
168 - 184	647.3108	1938.9107	1938.9034	4	0	K.YSHVDLVVMVDGFEGEK.G	Oxidation (M)	(Ions score 58)
168 - 184	970.4626	1938.9107	1938.9034	4	0	K.YSHVDLVVMVDGFEGEK.G	Oxidation (M)	(Ions score 37)
168 - 184	970.4626	1938.9107	1938.9034	4	0	K.YSHVDLVVMVDGFEGEK.G	Oxidation (M)	(Ions score 55)
168 - 184	647.3108	1938.9107	1938.9034	4	0	K.YSHVDLVVMVDGFEGEK.G	Oxidation (M)	(Ions score 64)
168 - 184	647.3108	1938.9107	1938.9034	4	0	K.YSHVDLVVMVDGFEGEK.G	Oxidation (M)	(Ions score 53)
168 - 184	647.3109	1938.9107	1938.9034	4	0	K.YSHVDLVVMVDGFEGEK.G	Oxidation (M)	(Ions score 61)
168 - 184	970.4626	1938.9107	1938.9034	4	0	K.YSHVDLVVMVDGFEGEK.G	Oxidation (M)	(Ions score 60)
168 - 184	647.3109	1938.9107	1938.9034	4	0	K.YSHVDLVVMVDGFEGEK.G	Oxidation (M)	(Ions score 58)

168 - 184	970.4627	1938.9108	1938.9034	4	0	K.YSHVDLVVMVDGFEGEK.G	Oxidation (M)	(Ions score 70)
168 - 184	970.4627	1938.9108	1938.9034	4	0	K.YSHVDLVVMVDGFEGEK.G	Oxidation (M)	(Ions score 57)
168 - 184	647.3109	1938.9108	1938.9034	4	0	K.YSHVDLVVMVDGFEGEK.G	Oxidation (M)	(Ions score 34)
168 - 184	970.4627	1938.9109	1938.9034	4	0	K.YSHVDLVVMVDGFEGEK.G	Oxidation (M)	(Ions score 63)
168 - 184	647.3109	1938.9109	1938.9034	4	0	K.YSHVDLVVMVDGFEGEK.G	Oxidation (M)	(Ions score 58)
168 - 184	970.4628	1938.9110	1938.9034	4	0	K.YSHVDLVVMVDGFEGEK.G	Oxidation (M)	(Ions score 56)
168 - 184	970.4628	1938.9110	1938.9034	4	0	K.YSHVDLVVMVDGFEGEK.G	Oxidation (M)	(Ions score 55)
168 - 184	970.4628	1938.9110	1938.9034	4	0	K.YSHVDLVVMVDGFEGEK.G	Oxidation (M)	(Ions score 70)
168 - 184	970.4628	1938.9110	1938.9034	4	0	K.YSHVDLVVMVDGFEGEK.G	Oxidation (M)	(Ions score 58)
168 - 184	970.4628	1938.9111	1938.9034	4	0	K.YSHVDLVVMVDGFEGEK.G	Oxidation (M)	(Ions score 75)
168 - 184	970.4628	1938.9111	1938.9034	4	0	K.YSHVDLVVMVDGFEGEK.G	Oxidation (M)	(Ions score 65)
168 - 184	647.3110	1938.9111	1938.9034	4	0	K.YSHVDLVVMVDGFEGEK.G	Oxidation (M)	(Ions score 52)
168 - 184	970.4628	1938.9111	1938.9034	4	0	K.YSHVDLVVMVDGFEGEK.G	Oxidation (M)	(Ions score 59)
168 - 184	647.3110	1938.9111	1938.9034	4	0	K.YSHVDLVVMVDGFEGEK.G	Oxidation (M)	(Ions score 66)
168 - 184	647.3110	1938.9111	1938.9034	4	0	K.YSHVDLVVMVDGFEGEK.G	Oxidation (M)	(Ions score 56)
168 - 184	647.3110	1938.9111	1938.9034	4	0	K.YSHVDLVVMVDGFEGEK.G	Oxidation (M)	(Ions score 61)
168 - 184	970.4628	1938.9111	1938.9034	4	0	K.YSHVDLVVMVDGFEGEK.G	Oxidation (M)	(Ions score 62)
168 - 184	647.3110	1938.9112	1938.9034	4	0	K.YSHVDLVVMVDGFEGEK.G	Oxidation (M)	(Ions score 51)
168 - 184	970.4629	1938.9112	1938.9034	4	0	K.YSHVDLVVMVDGFEGEK.G	Oxidation (M)	(Ions score 56)
168 - 184	970.4629	1938.9112	1938.9034	4	0	K.YSHVDLVVMVDGFEGEK.G	Oxidation (M)	(Ions score 70)
168 - 184	970.4629	1938.9113	1938.9034	4	0	K.YSHVDLVVMVDGFEGEK.G	Oxidation (M)	(Ions score 73)
168 - 184	970.4629	1938.9113	1938.9034	4	0	K.YSHVDLVVMVDGFEGEK.G	Oxidation (M)	(Ions score 57)
168 - 184	970.4629	1938.9113	1938.9034	4	0	K.YSHVDLVVMVDGFEGEK.G	Oxidation (M)	(Ions score 56)
168 - 184	970.4629	1938.9113	1938.9034	4	0	K.YSHVDLVVMVDGFEGEK.G	Oxidation (M)	(Ions score 67)
168 - 184	647.3110	1938.9113	1938.9034	4	0	K.YSHVDLVVMVDGFEGEK.G	Oxidation (M)	(Ions score 56)
168 - 184	647.3110	1938.9113	1938.9034	4	0	K.YSHVDLVVMVDGFEGEK.G	Oxidation (M)	(Ions score 66)
168 - 184	970.4629	1938.9113	1938.9034	4	0	K.YSHVDLVVMVDGFEGEK.G	Oxidation (M)	(Ions score 61)
168 - 184	970.4629	1938.9113	1938.9034	4	0	K.YSHVDLVVMVDGFEGEK.G	Oxidation (M)	(Ions score 67)
168 - 184	647.3110	1938.9113	1938.9034	4	0	K.YSHVDLVVMVDGFEGEK.G	Oxidation (M)	(Ions score 63)
168 - 184	970.4629	1938.9113	1938.9034	4	0	K.YSHVDLVVMVDGFEGEK.G	Oxidation (M)	(Ions score 50)
168 - 184	647.3111	1938.9115	1938.9034	4	0	K.YSHVDLVVMVDGFEGEK.G	Oxidation (M)	(Ions score 69)
168 - 184	970.4630	1938.9115	1938.9034	4	0	K.YSHVDLVVMVDGFEGEK.G	Oxidation (M)	(Ions score 58)
168 - 184	970.4630	1938.9115	1938.9034	4	0	K.YSHVDLVVMVDGFEGEK.G	Oxidation (M)	(Ions score 58)
168 - 184	647.3111	1938.9116	1938.9034	4	0	K.YSHVDLVVMVDGFEGEK.G	Oxidation (M)	(Ions score 69)
168 - 184	970.4631	1938.9116	1938.9034	4	0	K.YSHVDLVVMVDGFEGEK.G	Oxidation (M)	(Ions score 48)
168 - 184	970.4631	1938.9116	1938.9034	4	0	K.YSHVDLVVMVDGFEGEK.G	Oxidation (M)	(Ions score 54)
168 - 184	970.4631	1938.9117	1938.9034	4	0	K.YSHVDLVVMVDGFEGEK.G	Oxidation (M)	(Ions score 55)
168 - 184	647.3112	1938.9117	1938.9034	4	0	K.YSHVDLVVMVDGFEGEK.G	Oxidation (M)	(Ions score 60)
168 - 184	970.4631	1938.9117	1938.9034	4	0	K.YSHVDLVVMVDGFEGEK.G	Oxidation (M)	(Ions score 60)
168 - 184	970.4631	1938.9117	1938.9034	4	0	K.YSHVDLVVMVDGFEGEK.G	Oxidation (M)	(Ions score 53)
168 - 184	647.3112	1938.9118	1938.9034	4	0	K.YSHVDLVVMVDGFEGEK.G	Oxidation (M)	(Ions score 52)
168 - 184	970.4632	1938.9119	1938.9034	4	0	K.YSHVDLVVMVDGFEGEK.G	Oxidation (M)	(Ions score 57)
168 - 184	970.4632	1938.9119	1938.9034	4	0	K.YSHVDLVVMVDGFEGEK.G	Oxidation (M)	(Ions score 56)
168 - 184	647.3113	1938.9119	1938.9034	4	0	K.YSHVDLVVMVDGFEGEK.G	Oxidation (M)	(Ions score 53)
168 - 184	970.4632	1938.9119	1938.9034	4	0	K.YSHVDLVVMVDGFEGEK.G	Oxidation (M)	(Ions score 75)
168 - 184	647.3113	1938.9120	1938.9034	4	0	K.YSHVDLVVMVDGFEGEK.G	Oxidation (M)	(Ions score 58)
168 - 184	970.4633	1938.9120	1938.9034	4	0	K.YSHVDLVVMVDGFEGEK.G	Oxidation (M)	(Ions score 67)
168 - 184	647.3113	1938.9120	1938.9034	4	0	K.YSHVDLVVMVDGFEGEK.G	Oxidation (M)	(Ions score 53)
168 - 184	970.4633	1938.9120	1938.9034	4	0	K.YSHVDLVVMVDGFEGEK.G	Oxidation (M)	(Ions score 72)
168 - 184	970.4633	1938.9121	1938.9034	5	0	K.YSHVDLVVMVDGFEGEK.G	Oxidation (M)	(Ions score 64)
168 - 184	647.3113	1938.9122	1938.9034	5	0	K.YSHVDLVVMVDGFEGEK.G	Oxidation (M)	(Ions score 64)
168 - 184	970.4634	1938.9122	1938.9034	5	0	K.YSHVDLVVMVDGFEGEK.G	Oxidation (M)	(Ions score 56)
168 - 184	647.3113	1938.9122	1938.9034	5	0	K.YSHVDLVVMVDGFEGEK.G	Oxidation (M)	(Ions score 58)
168 - 184	970.4634	1938.9122	1938.9034	5	0	K.YSHVDLVVMVDGFEGEK.G	Oxidation (M)	(Ions score 58)
168 - 184	970.4634	1938.9122	1938.9034	5	0	K.YSHVDLVVMVDGFEGEK.G	Oxidation (M)	(Ions score 67)
168 - 184	970.4634	1938.9122	1938.9034	5	0	K.YSHVDLVVMVDGFEGEK.G	Oxidation (M)	(Ions score 56)
168 - 184	970.4634	1938.9123	1938.9034	5	0	K.YSHVDLVVMVDGFEGEK.G	Oxidation (M)	(Ions score 58)

168 - 184	647.3114	1938.9124	1938.9034	5	0	K.YSHVDLVVMVDGFEGEK.G	Oxidation (M)	(Ions score 66)
168 - 184	970.4635	1938.9124	1938.9034	5	0	K.YSHVDLVVMVDGFEGEK.G	Oxidation (M)	(Ions score 62)
168 - 184	970.4635	1938.9124	1938.9034	5	0	K.YSHVDLVVMVDGFEGEK.G	Oxidation (M)	(Ions score 55)
168 - 184	970.4635	1938.9124	1938.9034	5	0	K.YSHVDLVVMVDGFEGEK.G	Oxidation (M)	(Ions score 57)
168 - 184	647.3114	1938.9124	1938.9034	5	0	K.YSHVDLVVMVDGFEGEK.G	Oxidation (M)	(Ions score 41)
168 - 184	970.4635	1938.9124	1938.9034	5	0	K.YSHVDLVVMVDGFEGEK.G	Oxidation (M)	(Ions score 55)
168 - 184	970.4635	1938.9125	1938.9034	5	0	K.YSHVDLVVMVDGFEGEK.G	Oxidation (M)	(Ions score 57)
168 - 184	647.3115	1938.9125	1938.9034	5	0	K.YSHVDLVVMVDGFEGEK.G	Oxidation (M)	(Ions score 41)
168 - 184	970.4635	1938.9125	1938.9034	5	0	K.YSHVDLVVMVDGFEGEK.G	Oxidation (M)	(Ions score 59)
168 - 184	970.4637	1938.9128	1938.9034	5	0	K.YSHVDLVVMVDGFEGEK.G	Oxidation (M)	(Ions score 29)
168 - 184	970.4638	1938.9130	1938.9034	5	0	K.YSHVDLVVMVDGFEGEK.G	Oxidation (M)	(Ions score 50)
185 - 192	716.4056	715.3983	715.3977	1	0	K.GAVVAGSR.G		(Ions score 27)
193 - 197	627.3507	626.3434	626.3428	1	0	R.GYFLK.G		(Ions score 31)
193 - 197	627.3512	626.3439	626.3428	2	0	R.GYFLK.G		(Ions score 24)
198 - 213	611.6904	1832.0495	1832.0560	-4	0	K.GVLVFLEQALIQYALR.T		(Ions score 23)
198 - 213	917.0361	1832.0576	1832.0560	1	0	K.GVLVFLEQALIQYALR.T		(Ions score 101)
219 - 230	752.8869	1503.7593	1503.7584	1	0	R.GYIPIYTPFFMR.K		(Ions score 26)
219 - 230	752.8877	1503.7608	1503.7584	2	0	R.GYIPIYTPFFMR.K		(Ions score 39)
231 - 249	772.0438	2313.1096	2313.1198	-4	1	R.KEVMQEVAQLSQFDEELYK.V		(Ions score 29)
231 - 249	772.0439	2313.1098	2313.1198	-4	1	R.KEVMQEVAQLSQFDEELYK.V		(Ions score 25)
231 - 249	772.0446	2313.1120	2313.1198	-3	1	R.KEVMQEVAQLSQFDEELYK.V		(Ions score 21)
231 - 249	772.0447	2313.1124	2313.1198	-3	1	R.KEVMQEVAQLSQFDEELYK.V		(Ions score 38)
231 - 249	772.0449	2313.1128	2313.1198	-3	1	R.KEVMQEVAQLSQFDEELYK.V		(Ions score 24)
231 - 249	772.0449	2313.1128	2313.1198	-3	1	R.KEVMQEVAQLSQFDEELYK.V		(Ions score 42)
231 - 249	772.0449	2313.1129	2313.1198	-3	1	R.KEVMQEVAQLSQFDEELYK.V		(Ions score 29)
231 - 249	772.0450	2313.1133	2313.1198	-3	1	R.KEVMQEVAQLSQFDEELYK.V		(Ions score 41)
231 - 249	772.0457	2313.1153	2313.1198	-2	1	R.KEVMQEVAQLSQFDEELYK.V		(Ions score 27)
231 - 249	772.0458	2313.1155	2313.1198	-2	1	R.KEVMQEVAQLSQFDEELYK.V		(Ions score 30)
231 - 249	772.0461	2313.1164	2313.1198	-1	1	R.KEVMQEVAQLSQFDEELYK.V		(Ions score 38)
231 - 249	772.0463	2313.1171	2313.1198	-1	1	R.KEVMQEVAQLSQFDEELYK.V		(Ions score 24)
231 - 249	772.0464	2313.1175	2313.1198	-1	1	R.KEVMQEVAQLSQFDEELYK.V		(Ions score 34)
231 - 249	772.0466	2313.1181	2313.1198	-1	1	R.KEVMQEVAQLSQFDEELYK.V		(Ions score 33)
231 - 249	772.0470	2313.1193	2313.1198	-0	1	R.KEVMQEVAQLSQFDEELYK.V		(Ions score 31)
231 - 249	772.0471	2313.1194	2313.1198	-0	1	R.KEVMQEVAQLSQFDEELYK.V		(Ions score 49)
231 - 249	772.0471	2313.1195	2313.1198	-0	1	R.KEVMQEVAQLSQFDEELYK.V		(Ions score 32)
231 - 249	772.0473	2313.1201	2313.1198	0	1	R.KEVMQEVAQLSQFDEELYK.V		(Ions score 34)
231 - 249	772.0473	2313.1202	2313.1198	0	1	R.KEVMQEVAQLSQFDEELYK.V		(Ions score 69)
231 - 249	1157.5676	2313.1206	2313.1198	0	1	R.KEVMQEVAQLSQFDEELYK.V		(Ions score 57)
231 - 249	772.0475	2313.1208	2313.1198	0	1	R.KEVMQEVAQLSQFDEELYK.V		(Ions score 61)
231 - 249	772.0475	2313.1208	2313.1198	0	1	R.KEVMQEVAQLSQFDEELYK.V		(Ions score 52)
231 - 249	772.0476	2313.1209	2313.1198	0	1	R.KEVMQEVAQLSQFDEELYK.V		(Ions score 37)
231 - 249	772.0476	2313.1210	2313.1198	1	1	R.KEVMQEVAQLSQFDEELYK.V		(Ions score 47)
231 - 249	1157.5681	2313.1216	2313.1198	1	1	R.KEVMQEVAQLSQFDEELYK.V		(Ions score 77)
231 - 249	772.0480	2313.1221	2313.1198	1	1	R.KEVMQEVAQLSQFDEELYK.V		(Ions score 30)
231 - 249	772.0480	2313.1223	2313.1198	1	1	R.KEVMQEVAQLSQFDEELYK.V		(Ions score 22)
231 - 249	1157.5685	2313.1224	2313.1198	1	1	R.KEVMQEVAQLSQFDEELYK.V		(Ions score 86)
231 - 249	772.0481	2313.1225	2313.1198	1	1	R.KEVMQEVAQLSQFDEELYK.V		(Ions score 74)
231 - 249	772.0482	2313.1228	2313.1198	1	1	R.KEVMQEVAQLSQFDEELYK.V		(Ions score 55)
231 - 249	772.0483	2313.1230	2313.1198	1	1	R.KEVMQEVAQLSQFDEELYK.V		(Ions score 31)
231 - 249	772.0483	2313.1230	2313.1198	1	1	R.KEVMQEVAQLSQFDEELYK.V		(Ions score 52)
231 - 249	772.0483	2313.1231	2313.1198	1	1	R.KEVMQEVAQLSQFDEELYK.V		(Ions score 44)
231 - 249	772.0484	2313.1233	2313.1198	2	1	R.KEVMQEVAQLSQFDEELYK.V		(Ions score 72)
231 - 249	772.0485	2313.1238	2313.1198	2	1	R.KEVMQEVAQLSQFDEELYK.V		(Ions score 36)
231 - 249	772.0485	2313.1238	2313.1198	2	1	R.KEVMQEVAQLSQFDEELYK.V		(Ions score 31)
231 - 249	579.2882	2313.1238	2313.1198	2	1	R.KEVMQEVAQLSQFDEELYK.V		(Ions score 45)
231 - 249	772.0485	2313.1238	2313.1198	2	1	R.KEVMQEVAQLSQFDEELYK.V		(Ions score 49)
231 - 249	1157.5692	2313.1239	2313.1198	2	1	R.KEVMQEVAQLSQFDEELYK.V		(Ions score 79)

231 - 249	772.0486	2313.1240	2313.1198	2	1	R.KEVMQEVAQLSQFDEELYK.V	(Ions score 48)
231 - 249	772.0487	2313.1243	2313.1198	2	1	R.KEVMQEVAQLSQFDEELYK.V	(Ions score 59)
231 - 249	772.0487	2313.1244	2313.1198	2	1	R.KEVMQEVAQLSQFDEELYK.V	(Ions score 49)
231 - 249	772.0488	2313.1244	2313.1198	2	1	R.KEVMQEVAQLSQFDEELYK.V	(Ions score 75)
231 - 249	772.0488	2313.1245	2313.1198	2	1	R.KEVMQEVAQLSQFDEELYK.V	(Ions score 80)
231 - 249	772.0489	2313.1248	2313.1198	2	1	R.KEVMQEVAQLSQFDEELYK.V	(Ions score 34)
231 - 249	772.0489	2313.1248	2313.1198	2	1	R.KEVMQEVAQLSQFDEELYK.V	(Ions score 77)
231 - 249	772.0489	2313.1249	2313.1198	2	1	R.KEVMQEVAQLSQFDEELYK.V	(Ions score 57)
231 - 249	1157.5698	2313.1250	2313.1198	2	1	R.KEVMQEVAQLSQFDEELYK.V	(Ions score 88)
231 - 249	772.0490	2313.1250	2313.1198	2	1	R.KEVMQEVAQLSQFDEELYK.V	(Ions score 62)
231 - 249	772.0490	2313.1250	2313.1198	2	1	R.KEVMQEVAQLSQFDEELYK.V	(Ions score 56)
231 - 249	772.0490	2313.1251	2313.1198	2	1	R.KEVMQEVAQLSQFDEELYK.V	(Ions score 81)
231 - 249	772.0490	2313.1251	2313.1198	2	1	R.KEVMQEVAQLSQFDEELYK.V	(Ions score 51)
231 - 249	772.0490	2313.1252	2313.1198	2	1	R.KEVMQEVAQLSQFDEELYK.V	(Ions score 57)
231 - 249	772.0490	2313.1253	2313.1198	2	1	R.KEVMQEVAQLSQFDEELYK.V	(Ions score 62)
231 - 249	772.0490	2313.1253	2313.1198	2	1	R.KEVMQEVAQLSQFDEELYK.V	(Ions score 72)
231 - 249	772.0491	2313.1254	2313.1198	2	1	R.KEVMQEVAQLSQFDEELYK.V	(Ions score 68)
231 - 249	1157.5700	2313.1254	2313.1198	2	1	R.KEVMQEVAQLSQFDEELYK.V	(Ions score 77)
231 - 249	772.0491	2313.1254	2313.1198	2	1	R.KEVMQEVAQLSQFDEELYK.V	(Ions score 52)
231 - 249	772.0492	2313.1256	2313.1198	3	1	R.KEVMQEVAQLSQFDEELYK.V	(Ions score 68)
231 - 249	772.0492	2313.1258	2313.1198	3	1	R.KEVMQEVAQLSQFDEELYK.V	(Ions score 78)
231 - 249	772.0492	2313.1258	2313.1198	3	1	R.KEVMQEVAQLSQFDEELYK.V	(Ions score 70)
231 - 249	1157.5702	2313.1258	2313.1198	3	1	R.KEVMQEVAQLSQFDEELYK.V	(Ions score 70)
231 - 249	1157.5702	2313.1259	2313.1198	3	1	R.KEVMQEVAQLSQFDEELYK.V	(Ions score 78)
231 - 249	772.0493	2313.1260	2313.1198	3	1	R.KEVMQEVAQLSQFDEELYK.V	(Ions score 55)
231 - 249	1157.5703	2313.1260	2313.1198	3	1	R.KEVMQEVAQLSQFDEELYK.V	(Ions score 74)
231 - 249	772.0493	2313.1261	2313.1198	3	1	R.KEVMQEVAQLSQFDEELYK.V	(Ions score 46)
231 - 249	1157.5704	2313.1262	2313.1198	3	1	R.KEVMQEVAQLSQFDEELYK.V	(Ions score 55)
231 - 249	772.0494	2313.1263	2313.1198	3	1	R.KEVMQEVAQLSQFDEELYK.V	(Ions score 34)
231 - 249	772.0494	2313.1264	2313.1198	3	1	R.KEVMQEVAQLSQFDEELYK.V	(Ions score 60)
231 - 249	772.0495	2313.1265	2313.1198	3	1	R.KEVMQEVAQLSQFDEELYK.V	(Ions score 51)
231 - 249	1157.5706	2313.1266	2313.1198	3	1	R.KEVMQEVAQLSQFDEELYK.V	(Ions score 74)
231 - 249	1157.5706	2313.1267	2313.1198	3	1	R.KEVMQEVAQLSQFDEELYK.V	(Ions score 62)
231 - 249	1157.5707	2313.1268	2313.1198	3	1	R.KEVMQEVAQLSQFDEELYK.V	(Ions score 85)
231 - 249	1157.5707	2313.1268	2313.1198	3	1	R.KEVMQEVAQLSQFDEELYK.V	(Ions score 101)
231 - 249	1157.5707	2313.1269	2313.1198	3	1	R.KEVMQEVAQLSQFDEELYK.V	(Ions score 82)
231 - 249	772.0496	2313.1269	2313.1198	3	1	R.KEVMQEVAQLSQFDEELYK.V	(Ions score 60)
231 - 249	772.0496	2313.1269	2313.1198	3	1	R.KEVMQEVAQLSQFDEELYK.V	(Ions score 64)
231 - 249	772.0496	2313.1270	2313.1198	3	1	R.KEVMQEVAQLSQFDEELYK.V	(Ions score 61)
231 - 249	772.0496	2313.1270	2313.1198	3	1	R.KEVMQEVAQLSQFDEELYK.V	(Ions score 42)
231 - 249	772.0496	2313.1270	2313.1198	3	1	R.KEVMQEVAQLSQFDEELYK.V	(Ions score 66)
231 - 249	1157.5708	2313.1271	2313.1198	3	1	R.KEVMQEVAQLSQFDEELYK.V	(Ions score 84)
231 - 249	772.0497	2313.1271	2313.1198	3	1	R.KEVMQEVAQLSQFDEELYK.V	(Ions score 34)
231 - 249	772.0497	2313.1272	2313.1198	3	1	R.KEVMQEVAQLSQFDEELYK.V	(Ions score 43)
231 - 249	772.0497	2313.1273	2313.1198	3	1	R.KEVMQEVAQLSQFDEELYK.V	(Ions score 57)
231 - 249	772.0497	2313.1273	2313.1198	3	1	R.KEVMQEVAQLSQFDEELYK.V	(Ions score 54)
231 - 249	772.0498	2313.1275	2313.1198	3	1	R.KEVMQEVAQLSQFDEELYK.V	(Ions score 59)
231 - 249	1157.5710	2313.1275	2313.1198	3	1	R.KEVMQEVAQLSQFDEELYK.V	(Ions score 72)
231 - 249	772.0498	2313.1276	2313.1198	3	1	R.KEVMQEVAQLSQFDEELYK.V	(Ions score 72)
231 - 249	772.0498	2313.1277	2313.1198	3	1	R.KEVMQEVAQLSQFDEELYK.V	(Ions score 33)
231 - 249	772.0499	2313.1278	2313.1198	3	1	R.KEVMQEVAQLSQFDEELYK.V	(Ions score 29)
231 - 249	772.0499	2313.1278	2313.1198	3	1	R.KEVMQEVAQLSQFDEELYK.V	(Ions score 23)
231 - 249	772.0499	2313.1279	2313.1198	3	1	R.KEVMQEVAQLSQFDEELYK.V	(Ions score 60)
231 - 249	772.0499	2313.1279	2313.1198	3	1	R.KEVMQEVAQLSQFDEELYK.V	(Ions score 57)
231 - 249	772.0499	2313.1279	2313.1198	3	1	R.KEVMQEVAQLSQFDEELYK.V	(Ions score 26)
231 - 249	1157.5712	2313.1279	2313.1198	3	1	R.KEVMQEVAQLSQFDEELYK.V	(Ions score 109)
231 - 249	772.0499	2313.1280	2313.1198	4	1	R.KEVMQEVAQLSQFDEELYK.V	(Ions score 72)

231 - 249	772.0500	2313.1282	2313.1198	4	1	R.KEVMQEVAQLSQFDEELYK.V		(Ions score 58)
231 - 249	772.0500	2313.1282	2313.1198	4	1	R.KEVMQEVAQLSQFDEELYK.V		(Ions score 60)
231 - 249	772.0500	2313.1283	2313.1198	4	1	R.KEVMQEVAQLSQFDEELYK.V		(Ions score 59)
231 - 249	772.0501	2313.1284	2313.1198	4	1	R.KEVMQEVAQLSQFDEELYK.V		(Ions score 69)
231 - 249	772.0501	2313.1285	2313.1198	4	1	R.KEVMQEVAQLSQFDEELYK.V		(Ions score 34)
231 - 249	772.0501	2313.1285	2313.1198	4	1	R.KEVMQEVAQLSQFDEELYK.V		(Ions score 53)
231 - 249	772.0501	2313.1286	2313.1198	4	1	R.KEVMQEVAQLSQFDEELYK.V		(Ions score 73)
231 - 249	772.0501	2313.1286	2313.1198	4	1	R.KEVMQEVAQLSQFDEELYK.V		(Ions score 71)
231 - 249	772.0502	2313.1287	2313.1198	4	1	R.KEVMQEVAQLSQFDEELYK.V		(Ions score 47)
231 - 249	772.0502	2313.1287	2313.1198	4	1	R.KEVMQEVAQLSQFDEELYK.V		(Ions score 48)
231 - 249	772.0502	2313.1288	2313.1198	4	1	R.KEVMQEVAQLSQFDEELYK.V		(Ions score 59)
231 - 249	772.0503	2313.1290	2313.1198	4	1	R.KEVMQEVAQLSQFDEELYK.V		(Ions score 54)
231 - 249	1157.5718	2313.1291	2313.1198	4	1	R.KEVMQEVAQLSQFDEELYK.V		(Ions score 102)
231 - 249	772.0504	2313.1293	2313.1198	4	1	R.KEVMQEVAQLSQFDEELYK.V		(Ions score 60)
231 - 249	772.0504	2313.1293	2313.1198	4	1	R.KEVMQEVAQLSQFDEELYK.V		(Ions score 70)
231 - 249	772.0505	2313.1295	2313.1198	4	1	R.KEVMQEVAQLSQFDEELYK.V		(Ions score 62)
231 - 249	772.0505	2313.1298	2313.1198	4	1	R.KEVMQEVAQLSQFDEELYK.V		(Ions score 50)
231 - 249	772.0505	2313.1298	2313.1198	4	1	R.KEVMQEVAQLSQFDEELYK.V		(Ions score 55)
231 - 249	1157.5722	2313.1299	2313.1198	4	1	R.KEVMQEVAQLSQFDEELYK.V		(Ions score 51)
231 - 249	772.0506	2313.1299	2313.1198	4	1	R.KEVMQEVAQLSQFDEELYK.V		(Ions score 69)
231 - 249	772.0506	2313.1300	2313.1198	4	1	R.KEVMQEVAQLSQFDEELYK.V		(Ions score 70)
231 - 249	772.0506	2313.1300	2313.1198	4	1	R.KEVMQEVAQLSQFDEELYK.V		(Ions score 41)
231 - 249	772.0506	2313.1301	2313.1198	4	1	R.KEVMQEVAQLSQFDEELYK.V		(Ions score 72)
231 - 249	772.0507	2313.1302	2313.1198	4	1	R.KEVMQEVAQLSQFDEELYK.V		(Ions score 59)
231 - 249	772.0507	2313.1302	2313.1198	4	1	R.KEVMQEVAQLSQFDEELYK.V		(Ions score 60)
231 - 249	772.0507	2313.1302	2313.1198	4	1	R.KEVMQEVAQLSQFDEELYK.V		(Ions score 57)
231 - 249	1157.5724	2313.1303	2313.1198	5	1	R.KEVMQEVAQLSQFDEELYK.V		(Ions score 49)
231 - 249	772.0507	2313.1303	2313.1198	5	1	R.KEVMQEVAQLSQFDEELYK.V		(Ions score 67)
231 - 249	772.0508	2313.1305	2313.1198	5	1	R.KEVMQEVAQLSQFDEELYK.V		(Ions score 75)
231 - 249	772.0508	2313.1306	2313.1198	5	1	R.KEVMQEVAQLSQFDEELYK.V		(Ions score 56)
231 - 249	772.0510	2313.1311	2313.1198	5	1	R.KEVMQEVAQLSQFDEELYK.V		(Ions score 61)
231 - 249	1157.5729	2313.1312	2313.1198	5	1	R.KEVMQEVAQLSQFDEELYK.V		(Ions score 82)
231 - 249	1165.5657	2329.1169	2329.1147	1	1	R.KEVMQEVAQLSQFDEELYK.V	Oxidation (M)	(Ions score 72)
231 - 249	1165.5663	2329.1180	2329.1147	1	1	R.KEVMQEVAQLSQFDEELYK.V	Oxidation (M)	(Ions score 68)
231 - 249	1165.5664	2329.1182	2329.1147	1	1	R.KEVMQEVAQLSQFDEELYK.V	Oxidation (M)	(Ions score 68)
231 - 249	1165.5666	2329.1187	2329.1147	2	1	R.KEVMQEVAQLSQFDEELYK.V	Oxidation (M)	(Ions score 55)
231 - 249	1165.5671	2329.1196	2329.1147	2	1	R.KEVMQEVAQLSQFDEELYK.V	Oxidation (M)	(Ions score 68)
231 - 249	1165.5673	2329.1201	2329.1147	2	1	R.KEVMQEVAQLSQFDEELYK.V	Oxidation (M)	(Ions score 50)
231 - 249	1165.5673	2329.1201	2329.1147	2	1	R.KEVMQEVAQLSQFDEELYK.V	Oxidation (M)	(Ions score 83)
231 - 249	1165.5674	2329.1202	2329.1147	2	1	R.KEVMQEVAQLSQFDEELYK.V	Oxidation (M)	(Ions score 72)
231 - 249	1165.5674	2329.1203	2329.1147	2	1	R.KEVMQEVAQLSQFDEELYK.V	Oxidation (M)	(Ions score 58)
231 - 249	1165.5674	2329.1203	2329.1147	2	1	R.KEVMQEVAQLSQFDEELYK.V	Oxidation (M)	(Ions score 55)
231 - 249	1165.5676	2329.1207	2329.1147	3	1	R.KEVMQEVAQLSQFDEELYK.V	Oxidation (M)	(Ions score 55)
231 - 249	1165.5678	2329.1210	2329.1147	3	1	R.KEVMQEVAQLSQFDEELYK.V	Oxidation (M)	(Ions score 68)
231 - 249	777.3812	2329.1217	2329.1147	3	1	R.KEVMQEVAQLSQFDEELYK.V	Oxidation (M)	(Ions score 48)
231 - 249	1165.5683	2329.1220	2329.1147	3	1	R.KEVMQEVAQLSQFDEELYK.V	Oxidation (M)	(Ions score 61)
231 - 249	583.2878	2329.1220	2329.1147	3	1	R.KEVMQEVAQLSQFDEELYK.V	Oxidation (M)	(Ions score 25)
231 - 249	777.3813	2329.1221	2329.1147	3	1	R.KEVMQEVAQLSQFDEELYK.V	Oxidation (M)	(Ions score 49)
231 - 249	1165.5685	2329.1225	2329.1147	3	1	R.KEVMQEVAQLSQFDEELYK.V	Oxidation (M)	(Ions score 55)
231 - 249	777.3815	2329.1227	2329.1147	3	1	R.KEVMQEVAQLSQFDEELYK.V	Oxidation (M)	(Ions score 37)
231 - 249	777.3815	2329.1228	2329.1147	3	1	R.KEVMQEVAQLSQFDEELYK.V	Oxidation (M)	(Ions score 40)
231 - 249	1165.5687	2329.1228	2329.1147	3	1	R.KEVMQEVAQLSQFDEELYK.V	Oxidation (M)	(Ions score 93)
231 - 249	777.3817	2329.1233	2329.1147	4	1	R.KEVMQEVAQLSQFDEELYK.V	Oxidation (M)	(Ions score 38)
231 - 249	777.3817	2329.1233	2329.1147	4	1	R.KEVMQEVAQLSQFDEELYK.V	Oxidation (M)	(Ions score 44)
231 - 249	777.3817	2329.1234	2329.1147	4	1	R.KEVMQEVAQLSQFDEELYK.V	Oxidation (M)	(Ions score 41)
231 - 249	777.3819	2329.1240	2329.1147	4	1	R.KEVMQEVAQLSQFDEELYK.V	Oxidation (M)	(Ions score 49)
231 - 249	777.3821	2329.1245	2329.1147	4	1	R.KEVMQEVAQLSQFDEELYK.V	Oxidation (M)	(Ions score 32)

231 - 249	1178.5729	2355.1312	2355.1304	0	1	R.KEVMQEVAQLSQFDEELYK.V	Acetylation (K)	(Ions score 77)
231 - 249	786.0514	2355.1322	2355.1304	1	1	R.KEVMQEVAQLSQFDEELYK.V	Acetylation (K)	(Ions score 26)
231 - 249	786.0516	2355.1331	2355.1304	1	1	R.KEVMQEVAQLSQFDEELYK.V	Acetylation (K)	(Ions score 54)
231 - 249	786.0521	2355.1344	2355.1304	2	1	R.KEVMQEVAQLSQFDEELYK.V	Acetylation (K)	(Ions score 41)
231 - 249	1178.5753	2355.1361	2355.1304	2	1	R.KEVMQEVAQLSQFDEELYK.V	Acetylation (K)	(Ions score 92)
231 - 249	786.0527	2355.1362	2355.1304	2	1	R.KEVMQEVAQLSQFDEELYK.V	Acetylation (K)	(Ions score 57)
231 - 249	786.0528	2355.1365	2355.1304	3	1	R.KEVMQEVAQLSQFDEELYK.V	Acetylation (K)	(Ions score 48)
231 - 249	786.0528	2355.1366	2355.1304	3	1	R.KEVMQEVAQLSQFDEELYK.V	Acetylation (K)	(Ions score 33)
231 - 249	786.0528	2355.1367	2355.1304	3	1	R.KEVMQEVAQLSQFDEELYK.V	Acetylation (K)	(Ions score 59)
231 - 249	786.0529	2355.1367	2355.1304	3	1	R.KEVMQEVAQLSQFDEELYK.V	Acetylation (K)	(Ions score 57)
231 - 249	1178.5758	2355.1370	2355.1304	3	1	R.KEVMQEVAQLSQFDEELYK.V	Acetylation (K)	(Ions score 52)
231 - 249	786.0531	2355.1375	2355.1304	3	1	R.KEVMQEVAQLSQFDEELYK.V	Acetylation (K)	(Ions score 66)
231 - 249	1178.5761	2355.1377	2355.1304	3	1	R.KEVMQEVAQLSQFDEELYK.V	Acetylation (K)	(Ions score 60)
231 - 249	786.0536	2355.1389	2355.1304	4	1	R.KEVMQEVAQLSQFDEELYK.V	Acetylation (K)	(Ions score 45)
231 - 249	1178.5768	2355.1391	2355.1304	4	1	R.KEVMQEVAQLSQFDEELYK.V	Acetylation (K)	(Ions score 94)
231 - 249	1178.5771	2355.1396	2355.1304	4	1	R.KEVMQEVAQLSQFDEELYK.V	Acetylation (K)	(Ions score 59)
231 - 249	1178.5779	2355.1412	2355.1304	5	1	R.KEVMQEVAQLSQFDEELYK.V	Acetylation (K)	(Ions score 50)
231 - 249	786.0543	2355.1412	2355.1304	5	1	R.KEVMQEVAQLSQFDEELYK.V	Acetylation (K)	(Ions score 53)
231 - 249	786.0546	2355.1418	2355.1304	5	1	R.KEVMQEVAQLSQFDEELYK.V	Acetylation (K)	(Ions score 46)
231 - 249	791.3840	2371.1301	2371.1253	2	1	R.KEVMQEVAQLSQFDEELYK.V	Acetylation (K); Oxidation (M)	(Ions score 49)
231 - 249	791.3840	2371.1303	2371.1253	2	1	R.KEVMQEVAQLSQFDEELYK.V	Acetylation (K); Oxidation (M)	(Ions score 23)
231 - 249	1186.5736	2371.1326	2371.1253	3	1	R.KEVMQEVAQLSQFDEELYK.V	Acetylation (K); Oxidation (M)	(Ions score 90)
231 - 249	1186.5744	2371.1342	2371.1253	4	1	R.KEVMQEVAQLSQFDEELYK.V	Acetylation (K); Oxidation (M)	(Ions score 85)
231 - 249	791.3855	2371.1346	2371.1253	4	1	R.KEVMQEVAQLSQFDEELYK.V	Acetylation (K); Oxidation (M)	(Ions score 36)
231 - 249	791.3858	2371.1355	2371.1253	4	1	R.KEVMQEVAQLSQFDEELYK.V	Acetylation (K); Oxidation (M)	(Ions score 29)
231 - 249	1186.5753	2371.1361	2371.1253	5	1	R.KEVMQEVAQLSQFDEELYK.V	Acetylation (K); Oxidation (M)	(Ions score 45)
231 - 249	1186.5754	2371.1362	2371.1253	5	1	R.KEVMQEVAQLSQFDEELYK.V	Acetylation (K); Oxidation (M)	(Ions score 63)
232 - 249	1093.5164	2185.0182	2185.0249	-3	0	K.EVMQEVAQLSQFDEELYK.V		(Ions score 49)
232 - 249	1093.5174	2185.0203	2185.0249	-2	0	K.EVMQEVAQLSQFDEELYK.V		(Ions score 54)
232 - 249	1093.5193	2185.0240	2185.0249	-0	0	K.EVMQEVAQLSQFDEELYK.V		(Ions score 55)
232 - 249	1093.5195	2185.0245	2185.0249	-0	0	K.EVMQEVAQLSQFDEELYK.V		(Ions score 63)
232 - 249	729.3492	2185.0257	2185.0249	0	0	K.EVMQEVAQLSQFDEELYK.V		(Ions score 78)
232 - 249	1093.5202	2185.0259	2185.0249	0	0	K.EVMQEVAQLSQFDEELYK.V		(Ions score 91)
232 - 249	1093.5203	2185.0261	2185.0249	1	0	K.EVMQEVAQLSQFDEELYK.V		(Ions score 86)
232 - 249	1093.5203	2185.0261	2185.0249	1	0	K.EVMQEVAQLSQFDEELYK.V		(Ions score 80)
232 - 249	729.3494	2185.0264	2185.0249	1	0	K.EVMQEVAQLSQFDEELYK.V		(Ions score 66)
232 - 249	1093.5206	2185.0267	2185.0249	1	0	K.EVMQEVAQLSQFDEELYK.V		(Ions score 66)
232 - 249	1093.5206	2185.0267	2185.0249	1	0	K.EVMQEVAQLSQFDEELYK.V		(Ions score 73)
232 - 249	729.3496	2185.0269	2185.0249	1	0	K.EVMQEVAQLSQFDEELYK.V		(Ions score 91)
232 - 249	1093.5207	2185.0269	2185.0249	1	0	K.EVMQEVAQLSQFDEELYK.V		(Ions score 87)
232 - 249	1093.5207	2185.0269	2185.0249	1	0	K.EVMQEVAQLSQFDEELYK.V		(Ions score 114)
232 - 249	1093.5209	2185.0272	2185.0249	1	0	K.EVMQEVAQLSQFDEELYK.V		(Ions score 56)
232 - 249	1093.5210	2185.0274	2185.0249	1	0	K.EVMQEVAQLSQFDEELYK.V		(Ions score 73)
232 - 249	1093.5212	2185.0279	2185.0249	1	0	K.EVMQEVAQLSQFDEELYK.V		(Ions score 88)
232 - 249	729.3499	2185.0279	2185.0249	1	0	K.EVMQEVAQLSQFDEELYK.V		(Ions score 51)
232 - 249	1093.5212	2185.0279	2185.0249	1	0	K.EVMQEVAQLSQFDEELYK.V		(Ions score 58)
232 - 249	1093.5213	2185.0280	2185.0249	1	0	K.EVMQEVAQLSQFDEELYK.V		(Ions score 96)
232 - 249	1093.5214	2185.0283	2185.0249	2	0	K.EVMQEVAQLSQFDEELYK.V		(Ions score 81)
232 - 249	729.3501	2185.0284	2185.0249	2	0	K.EVMQEVAQLSQFDEELYK.V		(Ions score 61)
232 - 249	729.3501	2185.0284	2185.0249	2	0	K.EVMQEVAQLSQFDEELYK.V		(Ions score 74)
232 - 249	729.3501	2185.0285	2185.0249	2	0	K.EVMQEVAQLSQFDEELYK.V		(Ions score 67)
232 - 249	1093.5215	2185.0285	2185.0249	2	0	K.EVMQEVAQLSQFDEELYK.V		(Ions score 69)
232 - 249	1093.5216	2185.0286	2185.0249	2	0	K.EVMQEVAQLSQFDEELYK.V		(Ions score 86)
232 - 249	1093.5216	2185.0287	2185.0249	2	0	K.EVMQEVAQLSQFDEELYK.V		(Ions score 87)
232 - 249	1093.5217	2185.0289	2185.0249	2	0	K.EVMQEVAQLSQFDEELYK.V		(Ions score 101)
232 - 249	1093.5218	2185.0289	2185.0249	2	0	K.EVMQEVAQLSQFDEELYK.V		(Ions score 80)
232 - 249	1093.5218	2185.0290	2185.0249	2	0	K.EVMQEVAQLSQFDEELYK.V		(Ions score 74)

232 - 249	729.3503	2185.0290	2185.0249	2	0	K.EVMQEVAQLSQFDEELYK.V	(Ions score 71)
232 - 249	1093.5218	2185.0291	2185.0249	2	0	K.EVMQEVAQLSQFDEELYK.V	(Ions score 96)
232 - 249	729.3504	2185.0292	2185.0249	2	0	K.EVMQEVAQLSQFDEELYK.V	(Ions score 72)
232 - 249	1093.5219	2185.0293	2185.0249	2	0	K.EVMQEVAQLSQFDEELYK.V	(Ions score 62)
232 - 249	729.3504	2185.0293	2185.0249	2	0	K.EVMQEVAQLSQFDEELYK.V	(Ions score 85)
232 - 249	1093.5220	2185.0294	2185.0249	2	0	K.EVMQEVAQLSQFDEELYK.V	(Ions score 105)
232 - 249	1093.5220	2185.0294	2185.0249	2	0	K.EVMQEVAQLSQFDEELYK.V	(Ions score 84)
232 - 249	1093.5220	2185.0294	2185.0249	2	0	K.EVMQEVAQLSQFDEELYK.V	(Ions score 79)
232 - 249	1093.5220	2185.0294	2185.0249	2	0	K.EVMQEVAQLSQFDEELYK.V	(Ions score 87)
232 - 249	1093.5220	2185.0295	2185.0249	2	0	K.EVMQEVAQLSQFDEELYK.V	(Ions score 80)
232 - 249	729.3504	2185.0295	2185.0249	2	0	K.EVMQEVAQLSQFDEELYK.V	(Ions score 66)
232 - 249	729.3504	2185.0295	2185.0249	2	0	K.EVMQEVAQLSQFDEELYK.V	(Ions score 59)
232 - 249	1093.5220	2185.0295	2185.0249	2	0	K.EVMQEVAQLSQFDEELYK.V	(Ions score 96)
232 - 249	729.3505	2185.0296	2185.0249	2	0	K.EVMQEVAQLSQFDEELYK.V	(Ions score 64)
232 - 249	1093.5221	2185.0296	2185.0249	2	0	K.EVMQEVAQLSQFDEELYK.V	(Ions score 85)
232 - 249	729.3505	2185.0296	2185.0249	2	0	K.EVMQEVAQLSQFDEELYK.V	(Ions score 77)
232 - 249	1093.5221	2185.0297	2185.0249	2	0	K.EVMQEVAQLSQFDEELYK.V	(Ions score 65)
232 - 249	729.3505	2185.0297	2185.0249	2	0	K.EVMQEVAQLSQFDEELYK.V	(Ions score 77)
232 - 249	1093.5221	2185.0297	2185.0249	2	0	K.EVMQEVAQLSQFDEELYK.V	(Ions score 96)
232 - 249	1093.5221	2185.0297	2185.0249	2	0	K.EVMQEVAQLSQFDEELYK.V	(Ions score 102)
232 - 249	1093.5221	2185.0297	2185.0249	2	0	K.EVMQEVAQLSQFDEELYK.V	(Ions score 99)
232 - 249	729.3505	2185.0297	2185.0249	2	0	K.EVMQEVAQLSQFDEELYK.V	(Ions score 50)
232 - 249	729.3505	2185.0298	2185.0249	2	0	K.EVMQEVAQLSQFDEELYK.V	(Ions score 66)
232 - 249	1093.5222	2185.0298	2185.0249	2	0	K.EVMQEVAQLSQFDEELYK.V	(Ions score 100)
232 - 249	1093.5222	2185.0298	2185.0249	2	0	K.EVMQEVAQLSQFDEELYK.V	(Ions score 100)
232 - 249	729.3505	2185.0298	2185.0249	2	0	K.EVMQEVAQLSQFDEELYK.V	(Ions score 75)
232 - 249	1093.5222	2185.0298	2185.0249	2	0	K.EVMQEVAQLSQFDEELYK.V	(Ions score 81)
232 - 249	729.3506	2185.0299	2185.0249	2	0	K.EVMQEVAQLSQFDEELYK.V	(Ions score 64)
232 - 249	1093.5223	2185.0300	2185.0249	2	0	K.EVMQEVAQLSQFDEELYK.V	(Ions score 86)
232 - 249	1093.5223	2185.0300	2185.0249	2	0	K.EVMQEVAQLSQFDEELYK.V	(Ions score 96)
232 - 249	1093.5223	2185.0300	2185.0249	2	0	K.EVMQEVAQLSQFDEELYK.V	(Ions score 98)
232 - 249	729.3506	2185.0300	2185.0249	2	0	K.EVMQEVAQLSQFDEELYK.V	(Ions score 69)
232 - 249	1093.5223	2185.0301	2185.0249	2	0	K.EVMQEVAQLSQFDEELYK.V	(Ions score 99)
232 - 249	729.3506	2185.0301	2185.0249	2	0	K.EVMQEVAQLSQFDEELYK.V	(Ions score 56)
232 - 249	1093.5223	2185.0301	2185.0249	2	0	K.EVMQEVAQLSQFDEELYK.V	(Ions score 96)
232 - 249	729.3506	2185.0301	2185.0249	2	0	K.EVMQEVAQLSQFDEELYK.V	(Ions score 44)
232 - 249	1093.5223	2185.0301	2185.0249	2	0	K.EVMQEVAQLSQFDEELYK.V	(Ions score 96)
232 - 249	1093.5223	2185.0301	2185.0249	2	0	K.EVMQEVAQLSQFDEELYK.V	(Ions score 96)
232 - 249	729.3507	2185.0302	2185.0249	2	0	K.EVMQEVAQLSQFDEELYK.V	(Ions score 53)
232 - 249	729.3507	2185.0302	2185.0249	2	0	K.EVMQEVAQLSQFDEELYK.V	(Ions score 52)
232 - 249	729.3507	2185.0302	2185.0249	2	0	K.EVMQEVAQLSQFDEELYK.V	(Ions score 66)
232 - 249	1093.5224	2185.0302	2185.0249	2	0	K.EVMQEVAQLSQFDEELYK.V	(Ions score 86)
232 - 249	729.3507	2185.0302	2185.0249	2	0	K.EVMQEVAQLSQFDEELYK.V	(Ions score 77)
232 - 249	1093.5224	2185.0302	2185.0249	2	0	K.EVMQEVAQLSQFDEELYK.V	(Ions score 87)
232 - 249	1093.5224	2185.0303	2185.0249	2	0	K.EVMQEVAQLSQFDEELYK.V	(Ions score 80)
232 - 249	1093.5224	2185.0303	2185.0249	2	0	K.EVMQEVAQLSQFDEELYK.V	(Ions score 64)
232 - 249	1093.5224	2185.0303	2185.0249	2	0	K.EVMQEVAQLSQFDEELYK.V	(Ions score 100)
232 - 249	1093.5224	2185.0303	2185.0249	2	0	K.EVMQEVAQLSQFDEELYK.V	(Ions score 97)
232 - 249	1093.5224	2185.0303	2185.0249	2	0	K.EVMQEVAQLSQFDEELYK.V	(Ions score 89)
232 - 249	1093.5225	2185.0304	2185.0249	3	0	K.EVMQEVAQLSQFDEELYK.V	(Ions score 93)
232 - 249	1093.5225	2185.0304	2185.0249	3	0	K.EVMQEVAQLSQFDEELYK.V	(Ions score 94)
232 - 249	1093.5225	2185.0304	2185.0249	3	0	K.EVMQEVAQLSQFDEELYK.V	(Ions score 80)
232 - 249	729.3507	2185.0304	2185.0249	3	0	K.EVMQEVAQLSQFDEELYK.V	(Ions score 71)
232 - 249	1093.5225	2185.0304	2185.0249	3	0	K.EVMQEVAQLSQFDEELYK.V	(Ions score 85)
232 - 249	1093.5225	2185.0304	2185.0249	3	0	K.EVMQEVAQLSQFDEELYK.V	(Ions score 76)
232 - 249	1093.5225	2185.0304	2185.0249	3	0	K.EVMQEVAQLSQFDEELYK.V	(Ions score 88)
232 - 249	1093.5225	2185.0304	2185.0249	3	0	K.EVMQEVAQLSQFDEELYK.V	(Ions score 80)

232 - 249	729.3508	2185.0305	2185.0249	3	0	K.EVMQEVAQLSQFDEELYK.V	(Ions score 71)
232 - 249	1093.5225	2185.0305	2185.0249	3	0	K.EVMQEVAQLSQFDEELYK.V	(Ions score 80)
232 - 249	1093.5225	2185.0305	2185.0249	3	0	K.EVMQEVAQLSQFDEELYK.V	(Ions score 99)
232 - 249	1093.5226	2185.0306	2185.0249	3	0	K.EVMQEVAQLSQFDEELYK.V	(Ions score 101)
232 - 249	1093.5226	2185.0306	2185.0249	3	0	K.EVMQEVAQLSQFDEELYK.V	(Ions score 100)
232 - 249	1093.5226	2185.0306	2185.0249	3	0	K.EVMQEVAQLSQFDEELYK.V	(Ions score 99)
232 - 249	729.3508	2185.0306	2185.0249	3	0	K.EVMQEVAQLSQFDEELYK.V	(Ions score 72)
232 - 249	1093.5226	2185.0307	2185.0249	3	0	K.EVMQEVAQLSQFDEELYK.V	(Ions score 85)
232 - 249	1093.5226	2185.0307	2185.0249	3	0	K.EVMQEVAQLSQFDEELYK.V	(Ions score 87)
232 - 249	729.3509	2185.0307	2185.0249	3	0	K.EVMQEVAQLSQFDEELYK.V	(Ions score 72)
232 - 249	1093.5226	2185.0307	2185.0249	3	0	K.EVMQEVAQLSQFDEELYK.V	(Ions score 68)
232 - 249	729.3509	2185.0307	2185.0249	3	0	K.EVMQEVAQLSQFDEELYK.V	(Ions score 52)
232 - 249	1093.5227	2185.0308	2185.0249	3	0	K.EVMQEVAQLSQFDEELYK.V	(Ions score 85)
232 - 249	1093.5227	2185.0308	2185.0249	3	0	K.EVMQEVAQLSQFDEELYK.V	(Ions score 86)
232 - 249	729.3509	2185.0308	2185.0249	3	0	K.EVMQEVAQLSQFDEELYK.V	(Ions score 55)
232 - 249	1093.5227	2185.0308	2185.0249	3	0	K.EVMQEVAQLSQFDEELYK.V	(Ions score 84)
232 - 249	729.3509	2185.0309	2185.0249	3	0	K.EVMQEVAQLSQFDEELYK.V	(Ions score 94)
232 - 249	1093.5227	2185.0309	2185.0249	3	0	K.EVMQEVAQLSQFDEELYK.V	(Ions score 87)
232 - 249	729.3509	2185.0309	2185.0249	3	0	K.EVMQEVAQLSQFDEELYK.V	(Ions score 57)
232 - 249	1093.5228	2185.0310	2185.0249	3	0	K.EVMQEVAQLSQFDEELYK.V	(Ions score 84)
232 - 249	729.3509	2185.0310	2185.0249	3	0	K.EVMQEVAQLSQFDEELYK.V	(Ions score 62)
232 - 249	729.3510	2185.0310	2185.0249	3	0	K.EVMQEVAQLSQFDEELYK.V	(Ions score 64)
232 - 249	729.3510	2185.0311	2185.0249	3	0	K.EVMQEVAQLSQFDEELYK.V	(Ions score 61)
232 - 249	1093.5228	2185.0311	2185.0249	3	0	K.EVMQEVAQLSQFDEELYK.V	(Ions score 86)
232 - 249	1093.5228	2185.0311	2185.0249	3	0	K.EVMQEVAQLSQFDEELYK.V	(Ions score 88)
232 - 249	1093.5228	2185.0311	2185.0249	3	0	K.EVMQEVAQLSQFDEELYK.V	(Ions score 89)
232 - 249	1093.5228	2185.0311	2185.0249	3	0	K.EVMQEVAQLSQFDEELYK.V	(Ions score 96)
232 - 249	729.3510	2185.0312	2185.0249	3	0	K.EVMQEVAQLSQFDEELYK.V	(Ions score 66)
232 - 249	1093.5229	2185.0312	2185.0249	3	0	K.EVMQEVAQLSQFDEELYK.V	(Ions score 83)
232 - 249	1093.5229	2185.0312	2185.0249	3	0	K.EVMQEVAQLSQFDEELYK.V	(Ions score 107)
232 - 249	729.3510	2185.0312	2185.0249	3	0	K.EVMQEVAQLSQFDEELYK.V	(Ions score 42)
232 - 249	729.3510	2185.0312	2185.0249	3	0	K.EVMQEVAQLSQFDEELYK.V	(Ions score 50)
232 - 249	729.3510	2185.0312	2185.0249	3	0	K.EVMQEVAQLSQFDEELYK.V	(Ions score 64)
232 - 249	1093.5229	2185.0312	2185.0249	3	0	K.EVMQEVAQLSQFDEELYK.V	(Ions score 100)
232 - 249	1093.5229	2185.0313	2185.0249	3	0	K.EVMQEVAQLSQFDEELYK.V	(Ions score 83)
232 - 249	1093.5229	2185.0313	2185.0249	3	0	K.EVMQEVAQLSQFDEELYK.V	(Ions score 98)
232 - 249	1093.5230	2185.0314	2185.0249	3	0	K.EVMQEVAQLSQFDEELYK.V	(Ions score 96)
232 - 249	729.3511	2185.0314	2185.0249	3	0	K.EVMQEVAQLSQFDEELYK.V	(Ions score 61)
232 - 249	729.3511	2185.0314	2185.0249	3	0	K.EVMQEVAQLSQFDEELYK.V	(Ions score 64)
232 - 249	1093.5230	2185.0314	2185.0249	3	0	K.EVMQEVAQLSQFDEELYK.V	(Ions score 76)
232 - 249	1093.5230	2185.0314	2185.0249	3	0	K.EVMQEVAQLSQFDEELYK.V	(Ions score 81)
232 - 249	1093.5230	2185.0315	2185.0249	3	0	K.EVMQEVAQLSQFDEELYK.V	(Ions score 85)
232 - 249	729.3511	2185.0315	2185.0249	3	0	K.EVMQEVAQLSQFDEELYK.V	(Ions score 66)
232 - 249	1093.5230	2185.0315	2185.0249	3	0	K.EVMQEVAQLSQFDEELYK.V	(Ions score 86)
232 - 249	1093.5231	2185.0316	2185.0249	3	0	K.EVMQEVAQLSQFDEELYK.V	(Ions score 87)
232 - 249	729.3511	2185.0316	2185.0249	3	0	K.EVMQEVAQLSQFDEELYK.V	(Ions score 77)
232 - 249	729.3511	2185.0316	2185.0249	3	0	K.EVMQEVAQLSQFDEELYK.V	(Ions score 75)
232 - 249	1093.5231	2185.0316	2185.0249	3	0	K.EVMQEVAQLSQFDEELYK.V	(Ions score 86)
232 - 249	1093.5231	2185.0316	2185.0249	3	0	K.EVMQEVAQLSQFDEELYK.V	(Ions score 84)
232 - 249	729.3511	2185.0316	2185.0249	3	0	K.EVMQEVAQLSQFDEELYK.V	(Ions score 91)
232 - 249	1093.5231	2185.0317	2185.0249	3	0	K.EVMQEVAQLSQFDEELYK.V	(Ions score 86)
232 - 249	729.3512	2185.0317	2185.0249	3	0	K.EVMQEVAQLSQFDEELYK.V	(Ions score 58)
232 - 249	729.3512	2185.0317	2185.0249	3	0	K.EVMQEVAQLSQFDEELYK.V	(Ions score 71)
232 - 249	1093.5231	2185.0317	2185.0249	3	0	K.EVMQEVAQLSQFDEELYK.V	(Ions score 86)
232 - 249	1093.5231	2185.0317	2185.0249	3	0	K.EVMQEVAQLSQFDEELYK.V	(Ions score 100)
232 - 249	1093.5231	2185.0317	2185.0249	3	0	K.EVMQEVAQLSQFDEELYK.V	(Ions score 90)
232 - 249	1093.5232	2185.0318	2185.0249	3	0	K.EVMQEVAQLSQFDEELYK.V	(Ions score 99)

232 - 249	729.3512	2185.0318	2185.0249	3	0	K.EVMQEVAQLSQFDEELYK.V	(Ions score 69)
232 - 249	1093.5232	2185.0318	2185.0249	3	0	K.EVMQEVAQLSQFDEELYK.V	(Ions score 98)
232 - 249	1093.5232	2185.0318	2185.0249	3	0	K.EVMQEVAQLSQFDEELYK.V	(Ions score 80)
232 - 249	1093.5232	2185.0318	2185.0249	3	0	K.EVMQEVAQLSQFDEELYK.V	(Ions score 96)
232 - 249	1093.5232	2185.0318	2185.0249	3	0	K.EVMQEVAQLSQFDEELYK.V	(Ions score 38)
232 - 249	1093.5232	2185.0318	2185.0249	3	0	K.EVMQEVAQLSQFDEELYK.V	(Ions score 96)
232 - 249	729.3512	2185.0318	2185.0249	3	0	K.EVMQEVAQLSQFDEELYK.V	(Ions score 77)
232 - 249	1093.5232	2185.0318	2185.0249	3	0	K.EVMQEVAQLSQFDEELYK.V	(Ions score 76)
232 - 249	729.3512	2185.0319	2185.0249	3	0	K.EVMQEVAQLSQFDEELYK.V	(Ions score 66)
232 - 249	729.3512	2185.0319	2185.0249	3	0	K.EVMQEVAQLSQFDEELYK.V	(Ions score 62)
232 - 249	1093.5232	2185.0319	2185.0249	3	0	K.EVMQEVAQLSQFDEELYK.V	(Ions score 87)
232 - 249	729.3513	2185.0319	2185.0249	3	0	K.EVMQEVAQLSQFDEELYK.V	(Ions score 64)
232 - 249	729.3513	2185.0319	2185.0249	3	0	K.EVMQEVAQLSQFDEELYK.V	(Ions score 72)
232 - 249	1093.5233	2185.0320	2185.0249	3	0	K.EVMQEVAQLSQFDEELYK.V	(Ions score 80)
232 - 249	729.3513	2185.0320	2185.0249	3	0	K.EVMQEVAQLSQFDEELYK.V	(Ions score 54)
232 - 249	729.3513	2185.0321	2185.0249	3	0	K.EVMQEVAQLSQFDEELYK.V	(Ions score 52)
232 - 249	729.3513	2185.0321	2185.0249	3	0	K.EVMQEVAQLSQFDEELYK.V	(Ions score 56)
232 - 249	729.3513	2185.0321	2185.0249	3	0	K.EVMQEVAQLSQFDEELYK.V	(Ions score 72)
232 - 249	729.3513	2185.0321	2185.0249	3	0	K.EVMQEVAQLSQFDEELYK.V	(Ions score 67)
232 - 249	729.3513	2185.0321	2185.0249	3	0	K.EVMQEVAQLSQFDEELYK.V	(Ions score 78)
232 - 249	729.3513	2185.0321	2185.0249	3	0	K.EVMQEVAQLSQFDEELYK.V	(Ions score 39)
232 - 249	1093.5234	2185.0322	2185.0249	3	0	K.EVMQEVAQLSQFDEELYK.V	(Ions score 83)
232 - 249	1093.5234	2185.0323	2185.0249	3	0	K.EVMQEVAQLSQFDEELYK.V	(Ions score 84)
232 - 249	729.3514	2185.0323	2185.0249	3	0	K.EVMQEVAQLSQFDEELYK.V	(Ions score 55)
232 - 249	729.3514	2185.0323	2185.0249	3	0	K.EVMQEVAQLSQFDEELYK.V	(Ions score 63)
232 - 249	729.3514	2185.0323	2185.0249	3	0	K.EVMQEVAQLSQFDEELYK.V	(Ions score 30)
232 - 249	729.3514	2185.0323	2185.0249	3	0	K.EVMQEVAQLSQFDEELYK.V	(Ions score 64)
232 - 249	1093.5234	2185.0323	2185.0249	3	0	K.EVMQEVAQLSQFDEELYK.V	(Ions score 100)
232 - 249	729.3514	2185.0323	2185.0249	3	0	K.EVMQEVAQLSQFDEELYK.V	(Ions score 80)
232 - 249	729.3514	2185.0324	2185.0249	3	0	K.EVMQEVAQLSQFDEELYK.V	(Ions score 82)
232 - 249	729.3514	2185.0324	2185.0249	3	0	K.EVMQEVAQLSQFDEELYK.V	(Ions score 64)
232 - 249	729.3514	2185.0324	2185.0249	3	0	K.EVMQEVAQLSQFDEELYK.V	(Ions score 64)
232 - 249	729.3514	2185.0324	2185.0249	3	0	K.EVMQEVAQLSQFDEELYK.V	(Ions score 66)
232 - 249	729.3514	2185.0324	2185.0249	3	0	K.EVMQEVAQLSQFDEELYK.V	(Ions score 65)
232 - 249	1093.5235	2185.0324	2185.0249	3	0	K.EVMQEVAQLSQFDEELYK.V	(Ions score 74)
232 - 249	729.3514	2185.0324	2185.0249	3	0	K.EVMQEVAQLSQFDEELYK.V	(Ions score 61)
232 - 249	729.3514	2185.0324	2185.0249	3	0	K.EVMQEVAQLSQFDEELYK.V	(Ions score 64)
232 - 249	729.3514	2185.0325	2185.0249	3	0	K.EVMQEVAQLSQFDEELYK.V	(Ions score 64)
232 - 249	729.3514	2185.0325	2185.0249	3	0	K.EVMQEVAQLSQFDEELYK.V	(Ions score 80)
232 - 249	729.3515	2185.0325	2185.0249	4	0	K.EVMQEVAQLSQFDEELYK.V	(Ions score 68)
232 - 249	729.3515	2185.0326	2185.0249	4	0	K.EVMQEVAQLSQFDEELYK.V	(Ions score 55)
232 - 249	729.3515	2185.0326	2185.0249	4	0	K.EVMQEVAQLSQFDEELYK.V	(Ions score 70)
232 - 249	729.3515	2185.0326	2185.0249	4	0	K.EVMQEVAQLSQFDEELYK.V	(Ions score 69)
232 - 249	1093.5236	2185.0326	2185.0249	4	0	K.EVMQEVAQLSQFDEELYK.V	(Ions score 96)
232 - 249	729.3515	2185.0327	2185.0249	4	0	K.EVMQEVAQLSQFDEELYK.V	(Ions score 74)
232 - 249	1093.5236	2185.0327	2185.0249	4	0	K.EVMQEVAQLSQFDEELYK.V	(Ions score 79)
232 - 249	729.3515	2185.0328	2185.0249	4	0	K.EVMQEVAQLSQFDEELYK.V	(Ions score 66)
232 - 249	729.3515	2185.0328	2185.0249	4	0	K.EVMQEVAQLSQFDEELYK.V	(Ions score 64)
232 - 249	729.3515	2185.0328	2185.0249	4	0	K.EVMQEVAQLSQFDEELYK.V	(Ions score 92)
232 - 249	1093.5237	2185.0328	2185.0249	4	0	K.EVMQEVAQLSQFDEELYK.V	(Ions score 79)
232 - 249	729.3516	2185.0328	2185.0249	4	0	K.EVMQEVAQLSQFDEELYK.V	(Ions score 66)
232 - 249	729.3516	2185.0329	2185.0249	4	0	K.EVMQEVAQLSQFDEELYK.V	(Ions score 62)
232 - 249	729.3516	2185.0329	2185.0249	4	0	K.EVMQEVAQLSQFDEELYK.V	(Ions score 57)
232 - 249	729.3516	2185.0329	2185.0249	4	0	K.EVMQEVAQLSQFDEELYK.V	(Ions score 69)
232 - 249	729.3516	2185.0329	2185.0249	4	0	K.EVMQEVAQLSQFDEELYK.V	(Ions score 79)
232 - 249	729.3516	2185.0330	2185.0249	4	0	K.EVMQEVAQLSQFDEELYK.V	(Ions score 57)
232 - 249	729.3516	2185.0330	2185.0249	4	0	K.EVMQEVAQLSQFDEELYK.V	(Ions score 66)

232 - 249	1093.5238	2185.0330	2185.0249	4	0	K.EVMQEVAQLSQFDEELYK.V	(Ions score 98)
232 - 249	1093.5238	2185.0330	2185.0249	4	0	K.EVMQEVAQLSQFDEELYK.V	(Ions score 84)
232 - 249	729.3516	2185.0331	2185.0249	4	0	K.EVMQEVAQLSQFDEELYK.V	(Ions score 58)
232 - 249	729.3516	2185.0331	2185.0249	4	0	K.EVMQEVAQLSQFDEELYK.V	(Ions score 77)
232 - 249	729.3516	2185.0331	2185.0249	4	0	K.EVMQEVAQLSQFDEELYK.V	(Ions score 58)
232 - 249	729.3516	2185.0331	2185.0249	4	0	K.EVMQEVAQLSQFDEELYK.V	(Ions score 77)
232 - 249	729.3516	2185.0331	2185.0249	4	0	K.EVMQEVAQLSQFDEELYK.V	(Ions score 66)
232 - 249	1093.5238	2185.0331	2185.0249	4	0	K.EVMQEVAQLSQFDEELYK.V	(Ions score 101)
232 - 249	1093.5238	2185.0331	2185.0249	4	0	K.EVMQEVAQLSQFDEELYK.V	(Ions score 107)
232 - 249	729.3517	2185.0332	2185.0249	4	0	K.EVMQEVAQLSQFDEELYK.V	(Ions score 61)
232 - 249	729.3517	2185.0332	2185.0249	4	0	K.EVMQEVAQLSQFDEELYK.V	(Ions score 51)
232 - 249	729.3517	2185.0332	2185.0249	4	0	K.EVMQEVAQLSQFDEELYK.V	(Ions score 69)
232 - 249	729.3517	2185.0333	2185.0249	4	0	K.EVMQEVAQLSQFDEELYK.V	(Ions score 70)
232 - 249	729.3517	2185.0333	2185.0249	4	0	K.EVMQEVAQLSQFDEELYK.V	(Ions score 66)
232 - 249	1093.5239	2185.0333	2185.0249	4	0	K.EVMQEVAQLSQFDEELYK.V	(Ions score 82)
232 - 249	729.3517	2185.0334	2185.0249	4	0	K.EVMQEVAQLSQFDEELYK.V	(Ions score 62)
232 - 249	1093.5240	2185.0334	2185.0249	4	0	K.EVMQEVAQLSQFDEELYK.V	(Ions score 87)
232 - 249	729.3517	2185.0334	2185.0249	4	0	K.EVMQEVAQLSQFDEELYK.V	(Ions score 77)
232 - 249	729.3517	2185.0334	2185.0249	4	0	K.EVMQEVAQLSQFDEELYK.V	(Ions score 71)
232 - 249	729.3518	2185.0334	2185.0249	4	0	K.EVMQEVAQLSQFDEELYK.V	(Ions score 60)
232 - 249	1093.5240	2185.0334	2185.0249	4	0	K.EVMQEVAQLSQFDEELYK.V	(Ions score 96)
232 - 249	729.3518	2185.0335	2185.0249	4	0	K.EVMQEVAQLSQFDEELYK.V	(Ions score 80)
232 - 249	729.3518	2185.0335	2185.0249	4	0	K.EVMQEVAQLSQFDEELYK.V	(Ions score 64)
232 - 249	729.3518	2185.0335	2185.0249	4	0	K.EVMQEVAQLSQFDEELYK.V	(Ions score 56)
232 - 249	729.3518	2185.0336	2185.0249	4	0	K.EVMQEVAQLSQFDEELYK.V	(Ions score 64)
232 - 249	729.3518	2185.0336	2185.0249	4	0	K.EVMQEVAQLSQFDEELYK.V	(Ions score 69)
232 - 249	1093.5241	2185.0337	2185.0249	4	0	K.EVMQEVAQLSQFDEELYK.V	(Ions score 84)
232 - 249	729.3518	2185.0337	2185.0249	4	0	K.EVMQEVAQLSQFDEELYK.V	(Ions score 79)
232 - 249	729.3518	2185.0337	2185.0249	4	0	K.EVMQEVAQLSQFDEELYK.V	(Ions score 60)
232 - 249	729.3518	2185.0337	2185.0249	4	0	K.EVMQEVAQLSQFDEELYK.V	(Ions score 65)
232 - 249	1093.5241	2185.0337	2185.0249	4	0	K.EVMQEVAQLSQFDEELYK.V	(Ions score 100)
232 - 249	1093.5242	2185.0338	2185.0249	4	0	K.EVMQEVAQLSQFDEELYK.V	(Ions score 84)
232 - 249	1093.5242	2185.0338	2185.0249	4	0	K.EVMQEVAQLSQFDEELYK.V	(Ions score 75)
232 - 249	729.3519	2185.0339	2185.0249	4	0	K.EVMQEVAQLSQFDEELYK.V	(Ions score 69)
232 - 249	729.3519	2185.0339	2185.0249	4	0	K.EVMQEVAQLSQFDEELYK.V	(Ions score 73)
232 - 249	1093.5242	2185.0339	2185.0249	4	0	K.EVMQEVAQLSQFDEELYK.V	(Ions score 85)
232 - 249	729.3519	2185.0339	2185.0249	4	0	K.EVMQEVAQLSQFDEELYK.V	(Ions score 60)
232 - 249	729.3519	2185.0339	2185.0249	4	0	K.EVMQEVAQLSQFDEELYK.V	(Ions score 66)
232 - 249	729.3520	2185.0340	2185.0249	4	0	K.EVMQEVAQLSQFDEELYK.V	(Ions score 77)
232 - 249	729.3520	2185.0340	2185.0249	4	0	K.EVMQEVAQLSQFDEELYK.V	(Ions score 69)
232 - 249	1093.5243	2185.0341	2185.0249	4	0	K.EVMQEVAQLSQFDEELYK.V	(Ions score 96)
232 - 249	729.3520	2185.0341	2185.0249	4	0	K.EVMQEVAQLSQFDEELYK.V	(Ions score 72)
232 - 249	729.3520	2185.0341	2185.0249	4	0	K.EVMQEVAQLSQFDEELYK.V	(Ions score 61)
232 - 249	1093.5243	2185.0341	2185.0249	4	0	K.EVMQEVAQLSQFDEELYK.V	(Ions score 80)
232 - 249	729.3520	2185.0342	2185.0249	4	0	K.EVMQEVAQLSQFDEELYK.V	(Ions score 66)
232 - 249	729.3520	2185.0342	2185.0249	4	0	K.EVMQEVAQLSQFDEELYK.V	(Ions score 66)
232 - 249	729.3520	2185.0342	2185.0249	4	0	K.EVMQEVAQLSQFDEELYK.V	(Ions score 71)
232 - 249	729.3520	2185.0343	2185.0249	4	0	K.EVMQEVAQLSQFDEELYK.V	(Ions score 65)
232 - 249	729.3521	2185.0343	2185.0249	4	0	K.EVMQEVAQLSQFDEELYK.V	(Ions score 98)
232 - 249	1093.5245	2185.0344	2185.0249	4	0	K.EVMQEVAQLSQFDEELYK.V	(Ions score 92)
232 - 249	729.3521	2185.0345	2185.0249	4	0	K.EVMQEVAQLSQFDEELYK.V	(Ions score 74)
232 - 249	729.3521	2185.0345	2185.0249	4	0	K.EVMQEVAQLSQFDEELYK.V	(Ions score 53)
232 - 249	1093.5245	2185.0345	2185.0249	4	0	K.EVMQEVAQLSQFDEELYK.V	(Ions score 80)
232 - 249	1093.5246	2185.0345	2185.0249	4	0	K.EVMQEVAQLSQFDEELYK.V	(Ions score 92)
232 - 249	729.3522	2185.0346	2185.0249	4	0	K.EVMQEVAQLSQFDEELYK.V	(Ions score 60)
232 - 249	729.3522	2185.0347	2185.0249	4	0	K.EVMQEVAQLSQFDEELYK.V	(Ions score 62)
232 - 249	729.3522	2185.0347	2185.0249	4	0	K.EVMQEVAQLSQFDEELYK.V	(Ions score 75)

232 - 249	729.3522	2185.0349	2185.0249	5	0	K.EVMQEVAQLSQFDEELYK.V		(Ions score 63)
232 - 249	729.3522	2185.0349	2185.0249	5	0	K.EVMQEVAQLSQFDEELYK.V		(Ions score 55)
232 - 249	729.3523	2185.0349	2185.0249	5	0	K.EVMQEVAQLSQFDEELYK.V		(Ions score 77)
232 - 249	1093.5247	2185.0349	2185.0249	5	0	K.EVMQEVAQLSQFDEELYK.V		(Ions score 84)
232 - 249	729.3523	2185.0350	2185.0249	5	0	K.EVMQEVAQLSQFDEELYK.V		(Ions score 69)
232 - 249	729.3523	2185.0351	2185.0249	5	0	K.EVMQEVAQLSQFDEELYK.V		(Ions score 68)
232 - 249	1093.5248	2185.0351	2185.0249	5	0	K.EVMQEVAQLSQFDEELYK.V		(Ions score 79)
232 - 249	729.3523	2185.0351	2185.0249	5	0	K.EVMQEVAQLSQFDEELYK.V		(Ions score 53)
232 - 249	729.3523	2185.0351	2185.0249	5	0	K.EVMQEVAQLSQFDEELYK.V		(Ions score 64)
232 - 249	1093.5249	2185.0352	2185.0249	5	0	K.EVMQEVAQLSQFDEELYK.V		(Ions score 72)
232 - 249	729.3524	2185.0352	2185.0249	5	0	K.EVMQEVAQLSQFDEELYK.V		(Ions score 64)
232 - 249	729.3524	2185.0353	2185.0249	5	0	K.EVMQEVAQLSQFDEELYK.V		(Ions score 65)
232 - 249	729.3524	2185.0354	2185.0249	5	0	K.EVMQEVAQLSQFDEELYK.V		(Ions score 80)
232 - 249	1093.5250	2185.0354	2185.0249	5	0	K.EVMQEVAQLSQFDEELYK.V		(Ions score 85)
232 - 249	729.3524	2185.0354	2185.0249	5	0	K.EVMQEVAQLSQFDEELYK.V		(Ions score 69)
232 - 249	1093.5250	2185.0355	2185.0249	5	0	K.EVMQEVAQLSQFDEELYK.V		(Ions score 69)
232 - 249	729.3525	2185.0356	2185.0249	5	0	K.EVMQEVAQLSQFDEELYK.V		(Ions score 76)
232 - 249	729.3525	2185.0356	2185.0249	5	0	K.EVMQEVAQLSQFDEELYK.V		(Ions score 63)
232 - 249	729.3525	2185.0356	2185.0249	5	0	K.EVMQEVAQLSQFDEELYK.V		(Ions score 66)
232 - 249	729.3525	2185.0357	2185.0249	5	0	K.EVMQEVAQLSQFDEELYK.V		(Ions score 44)
232 - 249	1093.5251	2185.0357	2185.0249	5	0	K.EVMQEVAQLSQFDEELYK.V		(Ions score 76)
232 - 249	1093.5252	2185.0358	2185.0249	5	0	K.EVMQEVAQLSQFDEELYK.V		(Ions score 87)
232 - 249	729.3525	2185.0358	2185.0249	5	0	K.EVMQEVAQLSQFDEELYK.V		(Ions score 79)
232 - 249	1093.5252	2185.0358	2185.0249	5	0	K.EVMQEVAQLSQFDEELYK.V		(Ions score 96)
232 - 249	1101.5133	2201.0121	2201.0198	-4	0	K.EVMQEVAQLSQFDEELYK.V	Oxidation (M)	(Ions score 50)
232 - 249	1101.5164	2201.0183	2201.0198	-1	0	K.EVMQEVAQLSQFDEELYK.V	Oxidation (M)	(Ions score 79)
232 - 249	734.6801	2201.0185	2201.0198	-1	0	K.EVMQEVAQLSQFDEELYK.V	Oxidation (M)	(Ions score 21)
232 - 249	734.6802	2201.0187	2201.0198	-0	0	K.EVMQEVAQLSQFDEELYK.V	Oxidation (M)	(Ions score 36)
232 - 249	1101.5167	2201.0188	2201.0198	-0	0	K.EVMQEVAQLSQFDEELYK.V	Oxidation (M)	(Ions score 70)
232 - 249	734.6804	2201.0192	2201.0198	-0	0	K.EVMQEVAQLSQFDEELYK.V	Oxidation (M)	(Ions score 38)
232 - 249	734.6804	2201.0192	2201.0198	-0	0	K.EVMQEVAQLSQFDEELYK.V	Oxidation (M)	(Ions score 65)
232 - 249	734.6804	2201.0195	2201.0198	-0	0	K.EVMQEVAQLSQFDEELYK.V	Oxidation (M)	(Ions score 37)
232 - 249	734.6805	2201.0196	2201.0198	-0	0	K.EVMQEVAQLSQFDEELYK.V	Oxidation (M)	(Ions score 47)
232 - 249	1101.5171	2201.0196	2201.0198	-0	0	K.EVMQEVAQLSQFDEELYK.V	Oxidation (M)	(Ions score 64)
232 - 249	1101.5172	2201.0198	2201.0198	-0	0	K.EVMQEVAQLSQFDEELYK.V	Oxidation (M)	(Ions score 80)
232 - 249	734.6805	2201.0198	2201.0198	0	0	K.EVMQEVAQLSQFDEELYK.V	Oxidation (M)	(Ions score 35)
232 - 249	734.6807	2201.0202	2201.0198	0	0	K.EVMQEVAQLSQFDEELYK.V	Oxidation (M)	(Ions score 51)
232 - 249	734.6807	2201.0203	2201.0198	0	0	K.EVMQEVAQLSQFDEELYK.V	Oxidation (M)	(Ions score 51)
232 - 249	734.6808	2201.0206	2201.0198	0	0	K.EVMQEVAQLSQFDEELYK.V	Oxidation (M)	(Ions score 29)
232 - 249	1101.5178	2201.0210	2201.0198	1	0	K.EVMQEVAQLSQFDEELYK.V	Oxidation (M)	(Ions score 60)
232 - 249	734.6810	2201.0211	2201.0198	1	0	K.EVMQEVAQLSQFDEELYK.V	Oxidation (M)	(Ions score 39)
232 - 249	734.6810	2201.0213	2201.0198	1	0	K.EVMQEVAQLSQFDEELYK.V	Oxidation (M)	(Ions score 39)
232 - 249	734.6810	2201.0213	2201.0198	1	0	K.EVMQEVAQLSQFDEELYK.V	Oxidation (M)	(Ions score 47)
232 - 249	1101.5181	2201.0216	2201.0198	1	0	K.EVMQEVAQLSQFDEELYK.V	Oxidation (M)	(Ions score 61)
232 - 249	734.6812	2201.0217	2201.0198	1	0	K.EVMQEVAQLSQFDEELYK.V	Oxidation (M)	(Ions score 32)
232 - 249	1101.5181	2201.0217	2201.0198	1	0	K.EVMQEVAQLSQFDEELYK.V	Oxidation (M)	(Ions score 55)
232 - 249	1101.5181	2201.0217	2201.0198	1	0	K.EVMQEVAQLSQFDEELYK.V	Oxidation (M)	(Ions score 49)
232 - 249	1101.5182	2201.0218	2201.0198	1	0	K.EVMQEVAQLSQFDEELYK.V	Oxidation (M)	(Ions score 63)
232 - 249	1101.5182	2201.0219	2201.0198	1	0	K.EVMQEVAQLSQFDEELYK.V	Oxidation (M)	(Ions score 78)
232 - 249	1101.5183	2201.0219	2201.0198	1	0	K.EVMQEVAQLSQFDEELYK.V	Oxidation (M)	(Ions score 68)
232 - 249	1101.5183	2201.0221	2201.0198	1	0	K.EVMQEVAQLSQFDEELYK.V	Oxidation (M)	(Ions score 58)
232 - 249	1101.5183	2201.0221	2201.0198	1	0	K.EVMQEVAQLSQFDEELYK.V	Oxidation (M)	(Ions score 59)
232 - 249	734.6813	2201.0222	2201.0198	1	0	K.EVMQEVAQLSQFDEELYK.V	Oxidation (M)	(Ions score 27)
232 - 249	1101.5184	2201.0222	2201.0198	1	0	K.EVMQEVAQLSQFDEELYK.V	Oxidation (M)	(Ions score 74)
232 - 249	1101.5184	2201.0223	2201.0198	1	0	K.EVMQEVAQLSQFDEELYK.V	Oxidation (M)	(Ions score 48)
232 - 249	1101.5184	2201.0223	2201.0198	1	0	K.EVMQEVAQLSQFDEELYK.V	Oxidation (M)	(Ions score 44)
232 - 249	734.6814	2201.0223	2201.0198	1	0	K.EVMQEVAQLSQFDEELYK.V	Oxidation (M)	(Ions score 27)

232 - 249	1101.5184	2201.0223	2201.0198	1	0	K.EVMQEVAQLSQFDEELYK.V	Oxidation (M)	(Ions score 66)
232 - 249	1101.5185	2201.0224	2201.0198	1	0	K.EVMQEVAQLSQFDEELYK.V	Oxidation (M)	(Ions score 60)
232 - 249	1101.5185	2201.0225	2201.0198	1	0	K.EVMQEVAQLSQFDEELYK.V	Oxidation (M)	(Ions score 52)
232 - 249	1101.5185	2201.0225	2201.0198	1	0	K.EVMQEVAQLSQFDEELYK.V	Oxidation (M)	(Ions score 50)
232 - 249	1101.5185	2201.0225	2201.0198	1	0	K.EVMQEVAQLSQFDEELYK.V	Oxidation (M)	(Ions score 87)
232 - 249	734.6815	2201.0225	2201.0198	1	0	K.EVMQEVAQLSQFDEELYK.V	Oxidation (M)	(Ions score 27)
232 - 249	1101.5186	2201.0226	2201.0198	1	0	K.EVMQEVAQLSQFDEELYK.V	Oxidation (M)	(Ions score 60)
232 - 249	1101.5186	2201.0226	2201.0198	1	0	K.EVMQEVAQLSQFDEELYK.V	Oxidation (M)	(Ions score 85)
232 - 249	734.6815	2201.0226	2201.0198	1	0	K.EVMQEVAQLSQFDEELYK.V	Oxidation (M)	(Ions score 37)
232 - 249	1101.5186	2201.0227	2201.0198	1	0	K.EVMQEVAQLSQFDEELYK.V	Oxidation (M)	(Ions score 81)
232 - 249	734.6815	2201.0227	2201.0198	1	0	K.EVMQEVAQLSQFDEELYK.V	Oxidation (M)	(Ions score 24)
232 - 249	734.6815	2201.0227	2201.0198	1	0	K.EVMQEVAQLSQFDEELYK.V	Oxidation (M)	(Ions score 38)
232 - 249	734.6815	2201.0227	2201.0198	1	0	K.EVMQEVAQLSQFDEELYK.V	Oxidation (M)	(Ions score 32)
232 - 249	1101.5187	2201.0228	2201.0198	1	0	K.EVMQEVAQLSQFDEELYK.V	Oxidation (M)	(Ions score 70)
232 - 249	1101.5187	2201.0228	2201.0198	1	0	K.EVMQEVAQLSQFDEELYK.V	Oxidation (M)	(Ions score 57)
232 - 249	734.6815	2201.0228	2201.0198	1	0	K.EVMQEVAQLSQFDEELYK.V	Oxidation (M)	(Ions score 27)
232 - 249	1101.5187	2201.0228	2201.0198	1	0	K.EVMQEVAQLSQFDEELYK.V	Oxidation (M)	(Ions score 60)
232 - 249	1101.5187	2201.0228	2201.0198	1	0	K.EVMQEVAQLSQFDEELYK.V	Oxidation (M)	(Ions score 52)
232 - 249	734.6816	2201.0228	2201.0198	1	0	K.EVMQEVAQLSQFDEELYK.V	Oxidation (M)	(Ions score 27)
232 - 249	1101.5187	2201.0229	2201.0198	1	0	K.EVMQEVAQLSQFDEELYK.V	Oxidation (M)	(Ions score 86)
232 - 249	734.6816	2201.0229	2201.0198	1	0	K.EVMQEVAQLSQFDEELYK.V	Oxidation (M)	(Ions score 31)
232 - 249	1101.5187	2201.0229	2201.0198	1	0	K.EVMQEVAQLSQFDEELYK.V	Oxidation (M)	(Ions score 70)
232 - 249	734.6816	2201.0229	2201.0198	1	0	K.EVMQEVAQLSQFDEELYK.V	Oxidation (M)	(Ions score 44)
232 - 249	1101.5188	2201.0230	2201.0198	1	0	K.EVMQEVAQLSQFDEELYK.V	Oxidation (M)	(Ions score 68)
232 - 249	1101.5188	2201.0231	2201.0198	1	0	K.EVMQEVAQLSQFDEELYK.V	Oxidation (M)	(Ions score 57)
232 - 249	734.6816	2201.0231	2201.0198	2	0	K.EVMQEVAQLSQFDEELYK.V	Oxidation (M)	(Ions score 39)
232 - 249	1101.5189	2201.0232	2201.0198	2	0	K.EVMQEVAQLSQFDEELYK.V	Oxidation (M)	(Ions score 85)
232 - 249	734.6817	2201.0232	2201.0198	2	0	K.EVMQEVAQLSQFDEELYK.V	Oxidation (M)	(Ions score 30)
232 - 249	1101.5189	2201.0232	2201.0198	2	0	K.EVMQEVAQLSQFDEELYK.V	Oxidation (M)	(Ions score 58)
232 - 249	1101.5189	2201.0232	2201.0198	2	0	K.EVMQEVAQLSQFDEELYK.V	Oxidation (M)	(Ions score 67)
232 - 249	734.6817	2201.0232	2201.0198	2	0	K.EVMQEVAQLSQFDEELYK.V	Oxidation (M)	(Ions score 42)
232 - 249	734.6817	2201.0234	2201.0198	2	0	K.EVMQEVAQLSQFDEELYK.V	Oxidation (M)	(Ions score 34)
232 - 249	734.6817	2201.0234	2201.0198	2	0	K.EVMQEVAQLSQFDEELYK.V	Oxidation (M)	(Ions score 29)
232 - 249	1101.5190	2201.0234	2201.0198	2	0	K.EVMQEVAQLSQFDEELYK.V	Oxidation (M)	(Ions score 52)
232 - 249	1101.5190	2201.0234	2201.0198	2	0	K.EVMQEVAQLSQFDEELYK.V	Oxidation (M)	(Ions score 70)
232 - 249	1101.5190	2201.0235	2201.0198	2	0	K.EVMQEVAQLSQFDEELYK.V	Oxidation (M)	(Ions score 70)
232 - 249	1101.5191	2201.0235	2201.0198	2	0	K.EVMQEVAQLSQFDEELYK.V	Oxidation (M)	(Ions score 52)
232 - 249	734.6818	2201.0236	2201.0198	2	0	K.EVMQEVAQLSQFDEELYK.V	Oxidation (M)	(Ions score 29)
232 - 249	734.6818	2201.0236	2201.0198	2	0	K.EVMQEVAQLSQFDEELYK.V	Oxidation (M)	(Ions score 40)
232 - 249	1101.5191	2201.0236	2201.0198	2	0	K.EVMQEVAQLSQFDEELYK.V	Oxidation (M)	(Ions score 70)
232 - 249	734.6818	2201.0236	2201.0198	2	0	K.EVMQEVAQLSQFDEELYK.V	Oxidation (M)	(Ions score 38)
232 - 249	734.6818	2201.0236	2201.0198	2	0	K.EVMQEVAQLSQFDEELYK.V	Oxidation (M)	(Ions score 27)
232 - 249	734.6819	2201.0237	2201.0198	2	0	K.EVMQEVAQLSQFDEELYK.V	Oxidation (M)	(Ions score 43)
232 - 249	1101.5192	2201.0238	2201.0198	2	0	K.EVMQEVAQLSQFDEELYK.V	Oxidation (M)	(Ions score 80)
232 - 249	734.6819	2201.0238	2201.0198	2	0	K.EVMQEVAQLSQFDEELYK.V	Oxidation (M)	(Ions score 26)
232 - 249	1101.5192	2201.0238	2201.0198	2	0	K.EVMQEVAQLSQFDEELYK.V	Oxidation (M)	(Ions score 57)
232 - 249	1101.5192	2201.0238	2201.0198	2	0	K.EVMQEVAQLSQFDEELYK.V	Oxidation (M)	(Ions score 58)
232 - 249	1101.5192	2201.0239	2201.0198	2	0	K.EVMQEVAQLSQFDEELYK.V	Oxidation (M)	(Ions score 64)
232 - 249	734.6819	2201.0239	2201.0198	2	0	K.EVMQEVAQLSQFDEELYK.V	Oxidation (M)	(Ions score 45)
232 - 249	734.6819	2201.0240	2201.0198	2	0	K.EVMQEVAQLSQFDEELYK.V	Oxidation (M)	(Ions score 39)
232 - 249	1101.5193	2201.0240	2201.0198	2	0	K.EVMQEVAQLSQFDEELYK.V	Oxidation (M)	(Ions score 66)
232 - 249	1101.5193	2201.0240	2201.0198	2	0	K.EVMQEVAQLSQFDEELYK.V	Oxidation (M)	(Ions score 58)
232 - 249	1101.5193	2201.0240	2201.0198	2	0	K.EVMQEVAQLSQFDEELYK.V	Oxidation (M)	(Ions score 86)
232 - 249	734.6820	2201.0240	2201.0198	2	0	K.EVMQEVAQLSQFDEELYK.V	Oxidation (M)	(Ions score 31)
232 - 249	1101.5193	2201.0241	2201.0198	2	0	K.EVMQEVAQLSQFDEELYK.V	Oxidation (M)	(Ions score 66)
232 - 249	1101.5193	2201.0241	2201.0198	2	0	K.EVMQEVAQLSQFDEELYK.V	Oxidation (M)	(Ions score 48)
232 - 249	1101.5193	2201.0241	2201.0198	2	0	K.EVMQEVAQLSQFDEELYK.V	Oxidation (M)	(Ions score 70)

232 - 249	734.6820	2201.0242	2201.0198	2	0	K.EVMQEVAQLSQFDEELYK.V	Oxidation	(M)	(Ions score 27)
232 - 249	1101.5194	2201.0242	2201.0198	2	0	K.EVMQEVAQLSQFDEELYK.V	Oxidation	(M)	(Ions score 55)
232 - 249	1101.5194	2201.0242	2201.0198	2	0	K.EVMQEVAQLSQFDEELYK.V	Oxidation	(M)	(Ions score 71)
232 - 249	1101.5194	2201.0242	2201.0198	2	0	K.EVMQEVAQLSQFDEELYK.V	Oxidation	(M)	(Ions score 67)
232 - 249	1101.5194	2201.0243	2201.0198	2	0	K.EVMQEVAQLSQFDEELYK.V	Oxidation	(M)	(Ions score 48)
232 - 249	734.6820	2201.0243	2201.0198	2	0	K.EVMQEVAQLSQFDEELYK.V	Oxidation	(M)	(Ions score 29)
232 - 249	1101.5194	2201.0243	2201.0198	2	0	K.EVMQEVAQLSQFDEELYK.V	Oxidation	(M)	(Ions score 56)
232 - 249	1101.5194	2201.0243	2201.0198	2	0	K.EVMQEVAQLSQFDEELYK.V	Oxidation	(M)	(Ions score 52)
232 - 249	1101.5195	2201.0244	2201.0198	2	0	K.EVMQEVAQLSQFDEELYK.V	Oxidation	(M)	(Ions score 86)
232 - 249	1101.5195	2201.0244	2201.0198	2	0	K.EVMQEVAQLSQFDEELYK.V	Oxidation	(M)	(Ions score 71)
232 - 249	1101.5195	2201.0244	2201.0198	2	0	K.EVMQEVAQLSQFDEELYK.V	Oxidation	(M)	(Ions score 57)
232 - 249	734.6821	2201.0244	2201.0198	2	0	K.EVMQEVAQLSQFDEELYK.V	Oxidation	(M)	(Ions score 38)
232 - 249	734.6821	2201.0244	2201.0198	2	0	K.EVMQEVAQLSQFDEELYK.V	Oxidation	(M)	(Ions score 24)
232 - 249	734.6821	2201.0244	2201.0198	2	0	K.EVMQEVAQLSQFDEELYK.V	Oxidation	(M)	(Ions score 38)
232 - 249	734.6821	2201.0245	2201.0198	2	0	K.EVMQEVAQLSQFDEELYK.V	Oxidation	(M)	(Ions score 24)
232 - 249	734.6821	2201.0245	2201.0198	2	0	K.EVMQEVAQLSQFDEELYK.V	Oxidation	(M)	(Ions score 26)
232 - 249	1101.5195	2201.0245	2201.0198	2	0	K.EVMQEVAQLSQFDEELYK.V	Oxidation	(M)	(Ions score 56)
232 - 249	1101.5195	2201.0245	2201.0198	2	0	K.EVMQEVAQLSQFDEELYK.V	Oxidation	(M)	(Ions score 60)
232 - 249	1101.5195	2201.0245	2201.0198	2	0	K.EVMQEVAQLSQFDEELYK.V	Oxidation	(M)	(Ions score 74)
232 - 249	734.6821	2201.0246	2201.0198	2	0	K.EVMQEVAQLSQFDEELYK.V	Oxidation	(M)	(Ions score 34)
232 - 249	734.6821	2201.0246	2201.0198	2	0	K.EVMQEVAQLSQFDEELYK.V	Oxidation	(M)	(Ions score 71)
232 - 249	1101.5196	2201.0246	2201.0198	2	0	K.EVMQEVAQLSQFDEELYK.V	Oxidation	(M)	(Ions score 42)
232 - 249	1101.5196	2201.0246	2201.0198	2	0	K.EVMQEVAQLSQFDEELYK.V	Oxidation	(M)	(Ions score 58)
232 - 249	1101.5196	2201.0246	2201.0198	2	0	K.EVMQEVAQLSQFDEELYK.V	Oxidation	(M)	(Ions score 55)
232 - 249	734.6821	2201.0246	2201.0198	2	0	K.EVMQEVAQLSQFDEELYK.V	Oxidation	(M)	(Ions score 41)
232 - 249	734.6822	2201.0246	2201.0198	2	0	K.EVMQEVAQLSQFDEELYK.V	Oxidation	(M)	(Ions score 44)
232 - 249	734.6822	2201.0247	2201.0198	2	0	K.EVMQEVAQLSQFDEELYK.V	Oxidation	(M)	(Ions score 50)
232 - 249	1101.5197	2201.0248	2201.0198	2	0	K.EVMQEVAQLSQFDEELYK.V	Oxidation	(M)	(Ions score 66)
232 - 249	1101.5197	2201.0248	2201.0198	2	0	K.EVMQEVAQLSQFDEELYK.V	Oxidation	(M)	(Ions score 64)
232 - 249	734.6822	2201.0248	2201.0198	2	0	K.EVMQEVAQLSQFDEELYK.V	Oxidation	(M)	(Ions score 41)
232 - 249	1101.5197	2201.0248	2201.0198	2	0	K.EVMQEVAQLSQFDEELYK.V	Oxidation	(M)	(Ions score 56)
232 - 249	734.6822	2201.0248	2201.0198	2	0	K.EVMQEVAQLSQFDEELYK.V	Oxidation	(M)	(Ions score 24)
232 - 249	1101.5197	2201.0248	2201.0198	2	0	K.EVMQEVAQLSQFDEELYK.V	Oxidation	(M)	(Ions score 70)
232 - 249	1101.5197	2201.0248	2201.0198	2	0	K.EVMQEVAQLSQFDEELYK.V	Oxidation	(M)	(Ions score 64)
232 - 249	1101.5197	2201.0248	2201.0198	2	0	K.EVMQEVAQLSQFDEELYK.V	Oxidation	(M)	(Ions score 82)
232 - 249	734.6822	2201.0249	2201.0198	2	0	K.EVMQEVAQLSQFDEELYK.V	Oxidation	(M)	(Ions score 63)
232 - 249	734.6822	2201.0249	2201.0198	2	0	K.EVMQEVAQLSQFDEELYK.V	Oxidation	(M)	(Ions score 35)
232 - 249	1101.5197	2201.0249	2201.0198	2	0	K.EVMQEVAQLSQFDEELYK.V	Oxidation	(M)	(Ions score 57)
232 - 249	734.6823	2201.0249	2201.0198	2	0	K.EVMQEVAQLSQFDEELYK.V	Oxidation	(M)	(Ions score 34)
232 - 249	1101.5197	2201.0249	2201.0198	2	0	K.EVMQEVAQLSQFDEELYK.V	Oxidation	(M)	(Ions score 66)
232 - 249	734.6823	2201.0250	2201.0198	2	0	K.EVMQEVAQLSQFDEELYK.V	Oxidation	(M)	(Ions score 38)
232 - 249	1101.5198	2201.0250	2201.0198	2	0	K.EVMQEVAQLSQFDEELYK.V	Oxidation	(M)	(Ions score 71)
232 - 249	1101.5198	2201.0250	2201.0198	2	0	K.EVMQEVAQLSQFDEELYK.V	Oxidation	(M)	(Ions score 70)
232 - 249	1101.5198	2201.0250	2201.0198	2	0	K.EVMQEVAQLSQFDEELYK.V	Oxidation	(M)	(Ions score 85)
232 - 249	734.6823	2201.0251	2201.0198	2	0	K.EVMQEVAQLSQFDEELYK.V	Oxidation	(M)	(Ions score 44)
232 - 249	1101.5198	2201.0251	2201.0198	2	0	K.EVMQEVAQLSQFDEELYK.V	Oxidation	(M)	(Ions score 74)
232 - 249	1101.5198	2201.0251	2201.0198	2	0	K.EVMQEVAQLSQFDEELYK.V	Oxidation	(M)	(Ions score 75)
232 - 249	1101.5198	2201.0251	2201.0198	2	0	K.EVMQEVAQLSQFDEELYK.V	Oxidation	(M)	(Ions score 78)
232 - 249	1101.5198	2201.0251	2201.0198	2	0	K.EVMQEVAQLSQFDEELYK.V	Oxidation	(M)	(Ions score 70)
232 - 249	1101.5198	2201.0251	2201.0198	2	0	K.EVMQEVAQLSQFDEELYK.V	Oxidation	(M)	(Ions score 86)
232 - 249	734.6823	2201.0251	2201.0198	2	0	K.EVMQEVAQLSQFDEELYK.V	Oxidation	(M)	(Ions score 35)
232 - 249	1101.5198	2201.0251	2201.0198	2	0	K.EVMQEVAQLSQFDEELYK.V	Oxidation	(M)	(Ions score 67)
232 - 249	734.6823	2201.0251	2201.0198	2	0	K.EVMQEVAQLSQFDEELYK.V	Oxidation	(M)	(Ions score 32)
232 - 249	1101.5198	2201.0251	2201.0198	2	0	K.EVMQEVAQLSQFDEELYK.V	Oxidation	(M)	(Ions score 64)
232 - 249	734.6823	2201.0251	2201.0198	2	0	K.EVMQEVAQLSQFDEELYK.V	Oxidation	(M)	(Ions score 45)
232 - 249	1101.5198	2201.0251	2201.0198	2	0	K.EVMQEVAQLSQFDEELYK.V	Oxidation	(M)	(Ions score 79)
232 - 249	734.6823	2201.0251	2201.0198	2	0	K.EVMQEVAQLSQFDEELYK.V	Oxidation	(M)	(Ions score 37)

232 - 249	1101.5198	2201.0251	2201.0198	2	0	K.EVMQEVAQLSQFDEELYK.V	Oxidation (M)	(Ions score 58)
232 - 249	734.6823	2201.0251	2201.0198	2	0	K.EVMQEVAQLSQFDEELYK.V	Oxidation (M)	(Ions score 34)
232 - 249	734.6823	2201.0251	2201.0198	2	0	K.EVMQEVAQLSQFDEELYK.V	Oxidation (M)	(Ions score 44)
232 - 249	1101.5199	2201.0252	2201.0198	2	0	K.EVMQEVAQLSQFDEELYK.V	Oxidation (M)	(Ions score 70)
232 - 249	1101.5199	2201.0252	2201.0198	2	0	K.EVMQEVAQLSQFDEELYK.V	Oxidation (M)	(Ions score 86)
232 - 249	734.6823	2201.0252	2201.0198	2	0	K.EVMQEVAQLSQFDEELYK.V	Oxidation (M)	(Ions score 62)
232 - 249	1101.5199	2201.0252	2201.0198	2	0	K.EVMQEVAQLSQFDEELYK.V	Oxidation (M)	(Ions score 66)
232 - 249	1101.5199	2201.0252	2201.0198	2	0	K.EVMQEVAQLSQFDEELYK.V	Oxidation (M)	(Ions score 67)
232 - 249	1101.5199	2201.0252	2201.0198	2	0	K.EVMQEVAQLSQFDEELYK.V	Oxidation (M)	(Ions score 71)
232 - 249	1101.5199	2201.0252	2201.0198	2	0	K.EVMQEVAQLSQFDEELYK.V	Oxidation (M)	(Ions score 70)
232 - 249	734.6824	2201.0253	2201.0198	2	0	K.EVMQEVAQLSQFDEELYK.V	Oxidation (M)	(Ions score 47)
232 - 249	734.6824	2201.0253	2201.0198	2	0	K.EVMQEVAQLSQFDEELYK.V	Oxidation (M)	(Ions score 26)
232 - 249	1101.5199	2201.0253	2201.0198	2	0	K.EVMQEVAQLSQFDEELYK.V	Oxidation (M)	(Ions score 67)
232 - 249	734.6824	2201.0253	2201.0198	2	0	K.EVMQEVAQLSQFDEELYK.V	Oxidation (M)	(Ions score 36)
232 - 249	1101.5199	2201.0253	2201.0198	3	0	K.EVMQEVAQLSQFDEELYK.V	Oxidation (M)	(Ions score 82)
232 - 249	1101.5199	2201.0253	2201.0198	3	0	K.EVMQEVAQLSQFDEELYK.V	Oxidation (M)	(Ions score 66)
232 - 249	1101.5200	2201.0254	2201.0198	3	0	K.EVMQEVAQLSQFDEELYK.V	Oxidation (M)	(Ions score 70)
232 - 249	1101.5200	2201.0254	2201.0198	3	0	K.EVMQEVAQLSQFDEELYK.V	Oxidation (M)	(Ions score 71)
232 - 249	1101.5200	2201.0254	2201.0198	3	0	K.EVMQEVAQLSQFDEELYK.V	Oxidation (M)	(Ions score 74)
232 - 249	734.6824	2201.0254	2201.0198	3	0	K.EVMQEVAQLSQFDEELYK.V	Oxidation (M)	(Ions score 49)
232 - 249	1101.5200	2201.0254	2201.0198	3	0	K.EVMQEVAQLSQFDEELYK.V	Oxidation (M)	(Ions score 67)
232 - 249	1101.5200	2201.0254	2201.0198	3	0	K.EVMQEVAQLSQFDEELYK.V	Oxidation (M)	(Ions score 70)
232 - 249	1101.5200	2201.0254	2201.0198	3	0	K.EVMQEVAQLSQFDEELYK.V	Oxidation (M)	(Ions score 56)
232 - 249	1101.5200	2201.0254	2201.0198	3	0	K.EVMQEVAQLSQFDEELYK.V	Oxidation (M)	(Ions score 81)
232 - 249	734.6824	2201.0254	2201.0198	3	0	K.EVMQEVAQLSQFDEELYK.V	Oxidation (M)	(Ions score 41)
232 - 249	1101.5200	2201.0255	2201.0198	3	0	K.EVMQEVAQLSQFDEELYK.V	Oxidation (M)	(Ions score 81)
232 - 249	1101.5200	2201.0255	2201.0198	3	0	K.EVMQEVAQLSQFDEELYK.V	Oxidation (M)	(Ions score 66)
232 - 249	1101.5200	2201.0255	2201.0198	3	0	K.EVMQEVAQLSQFDEELYK.V	Oxidation (M)	(Ions score 71)
232 - 249	734.6824	2201.0255	2201.0198	3	0	K.EVMQEVAQLSQFDEELYK.V	Oxidation (M)	(Ions score 36)
232 - 249	734.6824	2201.0255	2201.0198	3	0	K.EVMQEVAQLSQFDEELYK.V	Oxidation (M)	(Ions score 34)
232 - 249	1101.5200	2201.0255	2201.0198	3	0	K.EVMQEVAQLSQFDEELYK.V	Oxidation (M)	(Ions score 82)
232 - 249	734.6824	2201.0255	2201.0198	3	0	K.EVMQEVAQLSQFDEELYK.V	Oxidation (M)	(Ions score 38)
232 - 249	734.6824	2201.0255	2201.0198	3	0	K.EVMQEVAQLSQFDEELYK.V	Oxidation (M)	(Ions score 43)
232 - 249	734.6825	2201.0255	2201.0198	3	0	K.EVMQEVAQLSQFDEELYK.V	Oxidation (M)	(Ions score 38)
232 - 249	734.6825	2201.0256	2201.0198	3	0	K.EVMQEVAQLSQFDEELYK.V	Oxidation (M)	(Ions score 44)
232 - 249	1101.5201	2201.0256	2201.0198	3	0	K.EVMQEVAQLSQFDEELYK.V	Oxidation (M)	(Ions score 65)
232 - 249	734.6825	2201.0256	2201.0198	3	0	K.EVMQEVAQLSQFDEELYK.V	Oxidation (M)	(Ions score 41)
232 - 249	734.6825	2201.0256	2201.0198	3	0	K.EVMQEVAQLSQFDEELYK.V	Oxidation (M)	(Ions score 29)
232 - 249	1101.5201	2201.0256	2201.0198	3	0	K.EVMQEVAQLSQFDEELYK.V	Oxidation (M)	(Ions score 64)
232 - 249	1101.5201	2201.0256	2201.0198	3	0	K.EVMQEVAQLSQFDEELYK.V	Oxidation (M)	(Ions score 75)
232 - 249	1101.5201	2201.0256	2201.0198	3	0	K.EVMQEVAQLSQFDEELYK.V	Oxidation (M)	(Ions score 66)
232 - 249	734.6825	2201.0256	2201.0198	3	0	K.EVMQEVAQLSQFDEELYK.V	Oxidation (M)	(Ions score 22)
232 - 249	734.6825	2201.0257	2201.0198	3	0	K.EVMQEVAQLSQFDEELYK.V	Oxidation (M)	(Ions score 40)
232 - 249	1101.5201	2201.0257	2201.0198	3	0	K.EVMQEVAQLSQFDEELYK.V	Oxidation (M)	(Ions score 70)
232 - 249	1101.5201	2201.0257	2201.0198	3	0	K.EVMQEVAQLSQFDEELYK.V	Oxidation (M)	(Ions score 70)
232 - 249	734.6825	2201.0257	2201.0198	3	0	K.EVMQEVAQLSQFDEELYK.V	Oxidation (M)	(Ions score 41)
232 - 249	734.6825	2201.0257	2201.0198	3	0	K.EVMQEVAQLSQFDEELYK.V	Oxidation (M)	(Ions score 50)
232 - 249	734.6825	2201.0257	2201.0198	3	0	K.EVMQEVAQLSQFDEELYK.V	Oxidation (M)	(Ions score 37)
232 - 249	1101.5202	2201.0258	2201.0198	3	0	K.EVMQEVAQLSQFDEELYK.V	Oxidation (M)	(Ions score 86)
232 - 249	1101.5202	2201.0258	2201.0198	3	0	K.EVMQEVAQLSQFDEELYK.V	Oxidation (M)	(Ions score 70)
232 - 249	1101.5202	2201.0258	2201.0198	3	0	K.EVMQEVAQLSQFDEELYK.V	Oxidation (M)	(Ions score 48)
232 - 249	734.6825	2201.0258	2201.0198	3	0	K.EVMQEVAQLSQFDEELYK.V	Oxidation (M)	(Ions score 26)
232 - 249	734.6826	2201.0258	2201.0198	3	0	K.EVMQEVAQLSQFDEELYK.V	Oxidation (M)	(Ions score 26)
232 - 249	734.6826	2201.0258	2201.0198	3	0	K.EVMQEVAQLSQFDEELYK.V	Oxidation (M)	(Ions score 25)
232 - 249	1101.5202	2201.0258	2201.0198	3	0	K.EVMQEVAQLSQFDEELYK.V	Oxidation (M)	(Ions score 56)
232 - 249	1101.5202	2201.0259	2201.0198	3	0	K.EVMQEVAQLSQFDEELYK.V	Oxidation (M)	(Ions score 69)
232 - 249	1101.5202	2201.0259	2201.0198	3	0	K.EVMQEVAQLSQFDEELYK.V	Oxidation (M)	(Ions score 63)

232 - 249	734.6826	2201.0259	2201.0198	3	0	K.EVMQEVAQLSQFDEELYK.V	Oxidation (M)	(Ions score 35)
232 - 249	1101.5202	2201.0259	2201.0198	3	0	K.EVMQEVAQLSQFDEELYK.V	Oxidation (M)	(Ions score 71)
232 - 249	734.6826	2201.0259	2201.0198	3	0	K.EVMQEVAQLSQFDEELYK.V	Oxidation (M)	(Ions score 30)
232 - 249	734.6826	2201.0259	2201.0198	3	0	K.EVMQEVAQLSQFDEELYK.V	Oxidation (M)	(Ions score 25)
232 - 249	1101.5202	2201.0259	2201.0198	3	0	K.EVMQEVAQLSQFDEELYK.V	Oxidation (M)	(Ions score 78)
232 - 249	734.6826	2201.0259	2201.0198	3	0	K.EVMQEVAQLSQFDEELYK.V	Oxidation (M)	(Ions score 37)
232 - 249	1101.5202	2201.0259	2201.0198	3	0	K.EVMQEVAQLSQFDEELYK.V	Oxidation (M)	(Ions score 70)
232 - 249	734.6826	2201.0260	2201.0198	3	0	K.EVMQEVAQLSQFDEELYK.V	Oxidation (M)	(Ions score 43)
232 - 249	1101.5203	2201.0260	2201.0198	3	0	K.EVMQEVAQLSQFDEELYK.V	Oxidation (M)	(Ions score 70)
232 - 249	734.6826	2201.0260	2201.0198	3	0	K.EVMQEVAQLSQFDEELYK.V	Oxidation (M)	(Ions score 26)
232 - 249	1101.5203	2201.0260	2201.0198	3	0	K.EVMQEVAQLSQFDEELYK.V	Oxidation (M)	(Ions score 74)
232 - 249	1101.5203	2201.0260	2201.0198	3	0	K.EVMQEVAQLSQFDEELYK.V	Oxidation (M)	(Ions score 81)
232 - 249	1101.5203	2201.0260	2201.0198	3	0	K.EVMQEVAQLSQFDEELYK.V	Oxidation (M)	(Ions score 67)
232 - 249	1101.5203	2201.0260	2201.0198	3	0	K.EVMQEVAQLSQFDEELYK.V	Oxidation (M)	(Ions score 70)
232 - 249	734.6826	2201.0260	2201.0198	3	0	K.EVMQEVAQLSQFDEELYK.V	Oxidation (M)	(Ions score 29)
232 - 249	734.6826	2201.0260	2201.0198	3	0	K.EVMQEVAQLSQFDEELYK.V	Oxidation (M)	(Ions score 37)
232 - 249	734.6826	2201.0260	2201.0198	3	0	K.EVMQEVAQLSQFDEELYK.V	Oxidation (M)	(Ions score 45)
232 - 249	734.6826	2201.0260	2201.0198	3	0	K.EVMQEVAQLSQFDEELYK.V	Oxidation (M)	(Ions score 39)
232 - 249	1101.5203	2201.0260	2201.0198	3	0	K.EVMQEVAQLSQFDEELYK.V	Oxidation (M)	(Ions score 70)
232 - 249	734.6826	2201.0261	2201.0198	3	0	K.EVMQEVAQLSQFDEELYK.V	Oxidation (M)	(Ions score 32)
232 - 249	734.6826	2201.0261	2201.0198	3	0	K.EVMQEVAQLSQFDEELYK.V	Oxidation (M)	(Ions score 30)
232 - 249	734.6827	2201.0261	2201.0198	3	0	K.EVMQEVAQLSQFDEELYK.V	Oxidation (M)	(Ions score 22)
232 - 249	1101.5204	2201.0262	2201.0198	3	0	K.EVMQEVAQLSQFDEELYK.V	Oxidation (M)	(Ions score 48)
232 - 249	734.6827	2201.0262	2201.0198	3	0	K.EVMQEVAQLSQFDEELYK.V	Oxidation (M)	(Ions score 33)
232 - 249	734.6827	2201.0262	2201.0198	3	0	K.EVMQEVAQLSQFDEELYK.V	Oxidation (M)	(Ions score 65)
232 - 249	1101.5204	2201.0262	2201.0198	3	0	K.EVMQEVAQLSQFDEELYK.V	Oxidation (M)	(Ions score 74)
232 - 249	734.6827	2201.0262	2201.0198	3	0	K.EVMQEVAQLSQFDEELYK.V	Oxidation (M)	(Ions score 36)
232 - 249	734.6827	2201.0262	2201.0198	3	0	K.EVMQEVAQLSQFDEELYK.V	Oxidation (M)	(Ions score 75)
232 - 249	1101.5204	2201.0262	2201.0198	3	0	K.EVMQEVAQLSQFDEELYK.V	Oxidation (M)	(Ions score 64)
232 - 249	1101.5204	2201.0263	2201.0198	3	0	K.EVMQEVAQLSQFDEELYK.V	Oxidation (M)	(Ions score 67)
232 - 249	734.6827	2201.0263	2201.0198	3	0	K.EVMQEVAQLSQFDEELYK.V	Oxidation (M)	(Ions score 37)
232 - 249	734.6827	2201.0263	2201.0198	3	0	K.EVMQEVAQLSQFDEELYK.V	Oxidation (M)	(Ions score 34)
232 - 249	734.6827	2201.0263	2201.0198	3	0	K.EVMQEVAQLSQFDEELYK.V	Oxidation (M)	(Ions score 34)
232 - 249	734.6827	2201.0264	2201.0198	3	0	K.EVMQEVAQLSQFDEELYK.V	Oxidation (M)	(Ions score 44)
232 - 249	1101.5205	2201.0264	2201.0198	3	0	K.EVMQEVAQLSQFDEELYK.V	Oxidation (M)	(Ions score 74)
232 - 249	734.6827	2201.0264	2201.0198	3	0	K.EVMQEVAQLSQFDEELYK.V	Oxidation (M)	(Ions score 36)
232 - 249	734.6827	2201.0264	2201.0198	3	0	K.EVMQEVAQLSQFDEELYK.V	Oxidation (M)	(Ions score 23)
232 - 249	734.6827	2201.0264	2201.0198	3	0	K.EVMQEVAQLSQFDEELYK.V	Oxidation (M)	(Ions score 35)
232 - 249	1101.5205	2201.0264	2201.0198	3	0	K.EVMQEVAQLSQFDEELYK.V	Oxidation (M)	(Ions score 66)
232 - 249	1101.5205	2201.0264	2201.0198	3	0	K.EVMQEVAQLSQFDEELYK.V	Oxidation (M)	(Ions score 66)
232 - 249	734.6828	2201.0264	2201.0198	3	0	K.EVMQEVAQLSQFDEELYK.V	Oxidation (M)	(Ions score 42)
232 - 249	734.6828	2201.0265	2201.0198	3	0	K.EVMQEVAQLSQFDEELYK.V	Oxidation (M)	(Ions score 44)
232 - 249	734.6828	2201.0265	2201.0198	3	0	K.EVMQEVAQLSQFDEELYK.V	Oxidation (M)	(Ions score 26)
232 - 249	1101.5206	2201.0266	2201.0198	3	0	K.EVMQEVAQLSQFDEELYK.V	Oxidation (M)	(Ions score 86)
232 - 249	734.6828	2201.0266	2201.0198	3	0	K.EVMQEVAQLSQFDEELYK.V	Oxidation (M)	(Ions score 42)
232 - 249	734.6828	2201.0266	2201.0198	3	0	K.EVMQEVAQLSQFDEELYK.V	Oxidation (M)	(Ions score 27)
232 - 249	734.6828	2201.0266	2201.0198	3	0	K.EVMQEVAQLSQFDEELYK.V	Oxidation (M)	(Ions score 36)
232 - 249	734.6828	2201.0266	2201.0198	3	0	K.EVMQEVAQLSQFDEELYK.V	Oxidation (M)	(Ions score 35)
232 - 249	734.6828	2201.0267	2201.0198	3	0	K.EVMQEVAQLSQFDEELYK.V	Oxidation (M)	(Ions score 35)
232 - 249	734.6828	2201.0267	2201.0198	3	0	K.EVMQEVAQLSQFDEELYK.V	Oxidation (M)	(Ions score 25)
232 - 249	734.6828	2201.0267	2201.0198	3	0	K.EVMQEVAQLSQFDEELYK.V	Oxidation (M)	(Ions score 27)
232 - 249	1101.5206	2201.0267	2201.0198	3	0	K.EVMQEVAQLSQFDEELYK.V	Oxidation (M)	(Ions score 75)
232 - 249	1101.5206	2201.0267	2201.0198	3	0	K.EVMQEVAQLSQFDEELYK.V	Oxidation (M)	(Ions score 74)
232 - 249	734.6829	2201.0268	2201.0198	3	0	K.EVMQEVAQLSQFDEELYK.V	Oxidation (M)	(Ions score 38)
232 - 249	1101.5207	2201.0268	2201.0198	3	0	K.EVMQEVAQLSQFDEELYK.V	Oxidation (M)	(Ions score 81)
232 - 249	734.6829	2201.0268	2201.0198	3	0	K.EVMQEVAQLSQFDEELYK.V	Oxidation (M)	(Ions score 45)
232 - 249	1101.5207	2201.0268	2201.0198	3	0	K.EVMQEVAQLSQFDEELYK.V	Oxidation (M)	(Ions score 57)

232 - 249	734.6829	2201.0269	2201.0198	3	0	K.EVMQEVAQLSQFDEELYK.V	Oxidation (M)	(Ions score 44)
232 - 249	734.6829	2201.0269	2201.0198	3	0	K.EVMQEVAQLSQFDEELYK.V	Oxidation (M)	(Ions score 41)
232 - 249	734.6829	2201.0269	2201.0198	3	0	K.EVMQEVAQLSQFDEELYK.V	Oxidation (M)	(Ions score 23)
232 - 249	1101.5207	2201.0269	2201.0198	3	0	K.EVMQEVAQLSQFDEELYK.V	Oxidation (M)	(Ions score 67)
232 - 249	734.6829	2201.0269	2201.0198	3	0	K.EVMQEVAQLSQFDEELYK.V	Oxidation (M)	(Ions score 26)
232 - 249	1101.5208	2201.0270	2201.0198	3	0	K.EVMQEVAQLSQFDEELYK.V	Oxidation (M)	(Ions score 64)
232 - 249	1101.5208	2201.0270	2201.0198	3	0	K.EVMQEVAQLSQFDEELYK.V	Oxidation (M)	(Ions score 53)
232 - 249	1101.5208	2201.0270	2201.0198	3	0	K.EVMQEVAQLSQFDEELYK.V	Oxidation (M)	(Ions score 90)
232 - 249	734.6829	2201.0270	2201.0198	3	0	K.EVMQEVAQLSQFDEELYK.V	Oxidation (M)	(Ions score 25)
232 - 249	1101.5208	2201.0270	2201.0198	3	0	K.EVMQEVAQLSQFDEELYK.V	Oxidation (M)	(Ions score 70)
232 - 249	1101.5208	2201.0270	2201.0198	3	0	K.EVMQEVAQLSQFDEELYK.V	Oxidation (M)	(Ions score 67)
232 - 249	734.6830	2201.0270	2201.0198	3	0	K.EVMQEVAQLSQFDEELYK.V	Oxidation (M)	(Ions score 36)
232 - 249	1101.5208	2201.0271	2201.0198	3	0	K.EVMQEVAQLSQFDEELYK.V	Oxidation (M)	(Ions score 66)
232 - 249	1101.5208	2201.0271	2201.0198	3	0	K.EVMQEVAQLSQFDEELYK.V	Oxidation (M)	(Ions score 82)
232 - 249	1101.5208	2201.0271	2201.0198	3	0	K.EVMQEVAQLSQFDEELYK.V	Oxidation (M)	(Ions score 74)
232 - 249	734.6830	2201.0271	2201.0198	3	0	K.EVMQEVAQLSQFDEELYK.V	Oxidation (M)	(Ions score 38)
232 - 249	1101.5208	2201.0271	2201.0198	3	0	K.EVMQEVAQLSQFDEELYK.V	Oxidation (M)	(Ions score 81)
232 - 249	1101.5208	2201.0271	2201.0198	3	0	K.EVMQEVAQLSQFDEELYK.V	Oxidation (M)	(Ions score 66)
232 - 249	734.6830	2201.0271	2201.0198	3	0	K.EVMQEVAQLSQFDEELYK.V	Oxidation (M)	(Ions score 49)
232 - 249	734.6830	2201.0271	2201.0198	3	0	K.EVMQEVAQLSQFDEELYK.V	Oxidation (M)	(Ions score 27)
232 - 249	734.6830	2201.0271	2201.0198	3	0	K.EVMQEVAQLSQFDEELYK.V	Oxidation (M)	(Ions score 24)
232 - 249	734.6830	2201.0271	2201.0198	3	0	K.EVMQEVAQLSQFDEELYK.V	Oxidation (M)	(Ions score 40)
232 - 249	1101.5208	2201.0271	2201.0198	3	0	K.EVMQEVAQLSQFDEELYK.V	Oxidation (M)	(Ions score 58)
232 - 249	1101.5209	2201.0272	2201.0198	3	0	K.EVMQEVAQLSQFDEELYK.V	Oxidation (M)	(Ions score 86)
232 - 249	1101.5209	2201.0272	2201.0198	3	0	K.EVMQEVAQLSQFDEELYK.V	Oxidation (M)	(Ions score 55)
232 - 249	1101.5209	2201.0272	2201.0198	3	0	K.EVMQEVAQLSQFDEELYK.V	Oxidation (M)	(Ions score 67)
232 - 249	1101.5209	2201.0272	2201.0198	3	0	K.EVMQEVAQLSQFDEELYK.V	Oxidation (M)	(Ions score 66)
232 - 249	1101.5209	2201.0272	2201.0198	3	0	K.EVMQEVAQLSQFDEELYK.V	Oxidation (M)	(Ions score 70)
232 - 249	1101.5209	2201.0272	2201.0198	3	0	K.EVMQEVAQLSQFDEELYK.V	Oxidation (M)	(Ions score 67)
232 - 249	734.6830	2201.0272	2201.0198	3	0	K.EVMQEVAQLSQFDEELYK.V	Oxidation (M)	(Ions score 49)
232 - 249	1101.5209	2201.0272	2201.0198	3	0	K.EVMQEVAQLSQFDEELYK.V	Oxidation (M)	(Ions score 70)
232 - 249	734.6830	2201.0272	2201.0198	3	0	K.EVMQEVAQLSQFDEELYK.V	Oxidation (M)	(Ions score 30)
232 - 249	734.6830	2201.0272	2201.0198	3	0	K.EVMQEVAQLSQFDEELYK.V	Oxidation (M)	(Ions score 41)
232 - 249	734.6830	2201.0272	2201.0198	3	0	K.EVMQEVAQLSQFDEELYK.V	Oxidation (M)	(Ions score 29)
232 - 249	734.6830	2201.0273	2201.0198	3	0	K.EVMQEVAQLSQFDEELYK.V	Oxidation (M)	(Ions score 37)
232 - 249	1101.5209	2201.0273	2201.0198	3	0	K.EVMQEVAQLSQFDEELYK.V	Oxidation (M)	(Ions score 57)
232 - 249	734.6830	2201.0273	2201.0198	3	0	K.EVMQEVAQLSQFDEELYK.V	Oxidation (M)	(Ions score 26)
232 - 249	1101.5209	2201.0273	2201.0198	3	0	K.EVMQEVAQLSQFDEELYK.V	Oxidation (M)	(Ions score 70)
232 - 249	1101.5209	2201.0273	2201.0198	3	0	K.EVMQEVAQLSQFDEELYK.V	Oxidation (M)	(Ions score 56)
232 - 249	1101.5209	2201.0273	2201.0198	3	0	K.EVMQEVAQLSQFDEELYK.V	Oxidation (M)	(Ions score 56)
232 - 249	734.6831	2201.0274	2201.0198	3	0	K.EVMQEVAQLSQFDEELYK.V	Oxidation (M)	(Ions score 47)
232 - 249	734.6831	2201.0274	2201.0198	3	0	K.EVMQEVAQLSQFDEELYK.V	Oxidation (M)	(Ions score 45)
232 - 249	734.6831	2201.0274	2201.0198	3	0	K.EVMQEVAQLSQFDEELYK.V	Oxidation (M)	(Ions score 28)
232 - 249	734.6831	2201.0274	2201.0198	3	0	K.EVMQEVAQLSQFDEELYK.V	Oxidation (M)	(Ions score 48)
232 - 249	1101.5210	2201.0274	2201.0198	3	0	K.EVMQEVAQLSQFDEELYK.V	Oxidation (M)	(Ions score 60)
232 - 249	1101.5210	2201.0274	2201.0198	3	0	K.EVMQEVAQLSQFDEELYK.V	Oxidation (M)	(Ions score 66)
232 - 249	734.6831	2201.0274	2201.0198	3	0	K.EVMQEVAQLSQFDEELYK.V	Oxidation (M)	(Ions score 39)
232 - 249	1101.5210	2201.0274	2201.0198	3	0	K.EVMQEVAQLSQFDEELYK.V	Oxidation (M)	(Ions score 86)
232 - 249	734.6831	2201.0274	2201.0198	3	0	K.EVMQEVAQLSQFDEELYK.V	Oxidation (M)	(Ions score 28)
232 - 249	734.6831	2201.0275	2201.0198	3	0	K.EVMQEVAQLSQFDEELYK.V	Oxidation (M)	(Ions score 41)
232 - 249	734.6831	2201.0275	2201.0198	3	0	K.EVMQEVAQLSQFDEELYK.V	Oxidation (M)	(Ions score 38)
232 - 249	734.6831	2201.0275	2201.0198	3	0	K.EVMQEVAQLSQFDEELYK.V	Oxidation (M)	(Ions score 38)
232 - 249	734.6831	2201.0275	2201.0198	4	0	K.EVMQEVAQLSQFDEELYK.V	Oxidation (M)	(Ions score 43)
232 - 249	1101.5210	2201.0275	2201.0198	4	0	K.EVMQEVAQLSQFDEELYK.V	Oxidation (M)	(Ions score 75)
232 - 249	1101.5210	2201.0275	2201.0198	4	0	K.EVMQEVAQLSQFDEELYK.V	Oxidation (M)	(Ions score 66)
232 - 249	734.6831	2201.0275	2201.0198	4	0	K.EVMQEVAQLSQFDEELYK.V	Oxidation (M)	(Ions score 41)
232 - 249	1101.5210	2201.0275	2201.0198	4	0	K.EVMQEVAQLSQFDEELYK.V	Oxidation (M)	(Ions score 81)

232 - 249	734.6831	2201.0276	2201.0198	4	0	K.EVMQEVAQLSQFDEELYK.V	Oxidation (M)	(Ions score 38)
232 - 249	1101.5211	2201.0276	2201.0198	4	0	K.EVMQEVAQLSQFDEELYK.V	Oxidation (M)	(Ions score 45)
232 - 249	1101.5211	2201.0276	2201.0198	4	0	K.EVMQEVAQLSQFDEELYK.V	Oxidation (M)	(Ions score 70)
232 - 249	734.6831	2201.0276	2201.0198	4	0	K.EVMQEVAQLSQFDEELYK.V	Oxidation (M)	(Ions score 38)
232 - 249	1101.5211	2201.0276	2201.0198	4	0	K.EVMQEVAQLSQFDEELYK.V	Oxidation (M)	(Ions score 67)
232 - 249	734.6831	2201.0276	2201.0198	4	0	K.EVMQEVAQLSQFDEELYK.V	Oxidation (M)	(Ions score 31)
232 - 249	1101.5211	2201.0276	2201.0198	4	0	K.EVMQEVAQLSQFDEELYK.V	Oxidation (M)	(Ions score 70)
232 - 249	1101.5211	2201.0276	2201.0198	4	0	K.EVMQEVAQLSQFDEELYK.V	Oxidation (M)	(Ions score 56)
232 - 249	1101.5211	2201.0277	2201.0198	4	0	K.EVMQEVAQLSQFDEELYK.V	Oxidation (M)	(Ions score 53)
232 - 249	734.6832	2201.0277	2201.0198	4	0	K.EVMQEVAQLSQFDEELYK.V	Oxidation (M)	(Ions score 27)
232 - 249	734.6832	2201.0278	2201.0198	4	0	K.EVMQEVAQLSQFDEELYK.V	Oxidation (M)	(Ions score 38)
232 - 249	734.6832	2201.0278	2201.0198	4	0	K.EVMQEVAQLSQFDEELYK.V	Oxidation (M)	(Ions score 39)
232 - 249	1101.5212	2201.0278	2201.0198	4	0	K.EVMQEVAQLSQFDEELYK.V	Oxidation (M)	(Ions score 71)
232 - 249	1101.5212	2201.0278	2201.0198	4	0	K.EVMQEVAQLSQFDEELYK.V	Oxidation (M)	(Ions score 58)
232 - 249	1101.5212	2201.0278	2201.0198	4	0	K.EVMQEVAQLSQFDEELYK.V	Oxidation (M)	(Ions score 70)
232 - 249	1101.5212	2201.0279	2201.0198	4	0	K.EVMQEVAQLSQFDEELYK.V	Oxidation (M)	(Ions score 71)
232 - 249	734.6832	2201.0279	2201.0198	4	0	K.EVMQEVAQLSQFDEELYK.V	Oxidation (M)	(Ions score 41)
232 - 249	734.6832	2201.0279	2201.0198	4	0	K.EVMQEVAQLSQFDEELYK.V	Oxidation (M)	(Ions score 31)
232 - 249	734.6832	2201.0279	2201.0198	4	0	K.EVMQEVAQLSQFDEELYK.V	Oxidation (M)	(Ions score 41)
232 - 249	734.6832	2201.0279	2201.0198	4	0	K.EVMQEVAQLSQFDEELYK.V	Oxidation (M)	(Ions score 24)
232 - 249	734.6833	2201.0280	2201.0198	4	0	K.EVMQEVAQLSQFDEELYK.V	Oxidation (M)	(Ions score 26)
232 - 249	1101.5213	2201.0280	2201.0198	4	0	K.EVMQEVAQLSQFDEELYK.V	Oxidation (M)	(Ions score 81)
232 - 249	734.6833	2201.0280	2201.0198	4	0	K.EVMQEVAQLSQFDEELYK.V	Oxidation (M)	(Ions score 35)
232 - 249	1101.5213	2201.0280	2201.0198	4	0	K.EVMQEVAQLSQFDEELYK.V	Oxidation (M)	(Ions score 63)
232 - 249	734.6833	2201.0280	2201.0198	4	0	K.EVMQEVAQLSQFDEELYK.V	Oxidation (M)	(Ions score 42)
232 - 249	734.6833	2201.0280	2201.0198	4	0	K.EVMQEVAQLSQFDEELYK.V	Oxidation (M)	(Ions score 46)
232 - 249	1101.5213	2201.0281	2201.0198	4	0	K.EVMQEVAQLSQFDEELYK.V	Oxidation (M)	(Ions score 63)
232 - 249	734.6833	2201.0281	2201.0198	4	0	K.EVMQEVAQLSQFDEELYK.V	Oxidation (M)	(Ions score 43)
232 - 249	1101.5213	2201.0281	2201.0198	4	0	K.EVMQEVAQLSQFDEELYK.V	Oxidation (M)	(Ions score 66)
232 - 249	734.6833	2201.0281	2201.0198	4	0	K.EVMQEVAQLSQFDEELYK.V	Oxidation (M)	(Ions score 50)
232 - 249	1101.5213	2201.0281	2201.0198	4	0	K.EVMQEVAQLSQFDEELYK.V	Oxidation (M)	(Ions score 57)
232 - 249	1101.5214	2201.0282	2201.0198	4	0	K.EVMQEVAQLSQFDEELYK.V	Oxidation (M)	(Ions score 70)
232 - 249	734.6833	2201.0282	2201.0198	4	0	K.EVMQEVAQLSQFDEELYK.V	Oxidation (M)	(Ions score 23)
232 - 249	734.6834	2201.0283	2201.0198	4	0	K.EVMQEVAQLSQFDEELYK.V	Oxidation (M)	(Ions score 38)
232 - 249	1101.5214	2201.0283	2201.0198	4	0	K.EVMQEVAQLSQFDEELYK.V	Oxidation (M)	(Ions score 78)
232 - 249	734.6834	2201.0283	2201.0198	4	0	K.EVMQEVAQLSQFDEELYK.V	Oxidation (M)	(Ions score 43)
232 - 249	734.6834	2201.0283	2201.0198	4	0	K.EVMQEVAQLSQFDEELYK.V	Oxidation (M)	(Ions score 38)
232 - 249	1101.5215	2201.0284	2201.0198	4	0	K.EVMQEVAQLSQFDEELYK.V	Oxidation (M)	(Ions score 64)
232 - 249	1101.5215	2201.0284	2201.0198	4	0	K.EVMQEVAQLSQFDEELYK.V	Oxidation (M)	(Ions score 86)
232 - 249	734.6834	2201.0284	2201.0198	4	0	K.EVMQEVAQLSQFDEELYK.V	Oxidation (M)	(Ions score 28)
232 - 249	1101.5215	2201.0284	2201.0198	4	0	K.EVMQEVAQLSQFDEELYK.V	Oxidation (M)	(Ions score 86)
232 - 249	734.6834	2201.0285	2201.0198	4	0	K.EVMQEVAQLSQFDEELYK.V	Oxidation (M)	(Ions score 31)
232 - 249	734.6835	2201.0286	2201.0198	4	0	K.EVMQEVAQLSQFDEELYK.V	Oxidation (M)	(Ions score 25)
232 - 249	734.6835	2201.0286	2201.0198	4	0	K.EVMQEVAQLSQFDEELYK.V	Oxidation (M)	(Ions score 42)
232 - 249	734.6835	2201.0286	2201.0198	4	0	K.EVMQEVAQLSQFDEELYK.V	Oxidation (M)	(Ions score 38)
232 - 249	1101.5216	2201.0286	2201.0198	4	0	K.EVMQEVAQLSQFDEELYK.V	Oxidation (M)	(Ions score 86)
232 - 249	734.6835	2201.0287	2201.0198	4	0	K.EVMQEVAQLSQFDEELYK.V	Oxidation (M)	(Ions score 27)
232 - 249	734.6835	2201.0287	2201.0198	4	0	K.EVMQEVAQLSQFDEELYK.V	Oxidation (M)	(Ions score 36)
232 - 249	734.6835	2201.0288	2201.0198	4	0	K.EVMQEVAQLSQFDEELYK.V	Oxidation (M)	(Ions score 44)
232 - 249	734.6836	2201.0289	2201.0198	4	0	K.EVMQEVAQLSQFDEELYK.V	Oxidation (M)	(Ions score 47)
232 - 249	734.6836	2201.0289	2201.0198	4	0	K.EVMQEVAQLSQFDEELYK.V	Oxidation (M)	(Ions score 26)
232 - 249	734.6836	2201.0289	2201.0198	4	0	K.EVMQEVAQLSQFDEELYK.V	Oxidation (M)	(Ions score 24)
232 - 249	734.6836	2201.0289	2201.0198	4	0	K.EVMQEVAQLSQFDEELYK.V	Oxidation (M)	(Ions score 47)
232 - 249	734.6836	2201.0289	2201.0198	4	0	K.EVMQEVAQLSQFDEELYK.V	Oxidation (M)	(Ions score 30)
232 - 249	1101.5218	2201.0289	2201.0198	4	0	K.EVMQEVAQLSQFDEELYK.V	Oxidation (M)	(Ions score 49)
232 - 249	734.6836	2201.0290	2201.0198	4	0	K.EVMQEVAQLSQFDEELYK.V	Oxidation (M)	(Ions score 32)
232 - 249	734.6836	2201.0290	2201.0198	4	0	K.EVMQEVAQLSQFDEELYK.V	Oxidation (M)	(Ions score 42)

232 - 249	734.6836	2201.0290	2201.0198	4	0	K.EVMQEVAQLSQFDEELYK.V	Oxidation (M)	(Ions score 41)
232 - 249	1101.5218	2201.0290	2201.0198	4	0	K.EVMQEVAQLSQFDEELYK.V	Oxidation (M)	(Ions score 63)
232 - 249	1101.5218	2201.0291	2201.0198	4	0	K.EVMQEVAQLSQFDEELYK.V	Oxidation (M)	(Ions score 53)
232 - 249	1101.5218	2201.0291	2201.0198	4	0	K.EVMQEVAQLSQFDEELYK.V	Oxidation (M)	(Ions score 71)
232 - 249	734.6837	2201.0292	2201.0198	4	0	K.EVMQEVAQLSQFDEELYK.V	Oxidation (M)	(Ions score 41)
232 - 249	1101.5219	2201.0292	2201.0198	4	0	K.EVMQEVAQLSQFDEELYK.V	Oxidation (M)	(Ions score 50)
232 - 249	734.6837	2201.0292	2201.0198	4	0	K.EVMQEVAQLSQFDEELYK.V	Oxidation (M)	(Ions score 41)
232 - 249	734.6837	2201.0293	2201.0198	4	0	K.EVMQEVAQLSQFDEELYK.V	Oxidation (M)	(Ions score 36)
232 - 249	734.6837	2201.0294	2201.0198	4	0	K.EVMQEVAQLSQFDEELYK.V	Oxidation (M)	(Ions score 37)
232 - 249	1101.5220	2201.0295	2201.0198	4	0	K.EVMQEVAQLSQFDEELYK.V	Oxidation (M)	(Ions score 63)
232 - 249	1101.5220	2201.0295	2201.0198	4	0	K.EVMQEVAQLSQFDEELYK.V	Oxidation (M)	(Ions score 86)
232 - 249	734.6838	2201.0295	2201.0198	4	0	K.EVMQEVAQLSQFDEELYK.V	Oxidation (M)	(Ions score 38)
232 - 249	1101.5220	2201.0295	2201.0198	4	0	K.EVMQEVAQLSQFDEELYK.V	Oxidation (M)	(Ions score 86)
232 - 249	734.6838	2201.0295	2201.0198	4	0	K.EVMQEVAQLSQFDEELYK.V	Oxidation (M)	(Ions score 38)
232 - 249	1101.5220	2201.0295	2201.0198	4	0	K.EVMQEVAQLSQFDEELYK.V	Oxidation (M)	(Ions score 82)
232 - 249	734.6838	2201.0296	2201.0198	4	0	K.EVMQEVAQLSQFDEELYK.V	Oxidation (M)	(Ions score 45)
232 - 249	1101.5221	2201.0296	2201.0198	4	0	K.EVMQEVAQLSQFDEELYK.V	Oxidation (M)	(Ions score 56)
232 - 249	734.6838	2201.0296	2201.0198	4	0	K.EVMQEVAQLSQFDEELYK.V	Oxidation (M)	(Ions score 37)
232 - 249	1101.5221	2201.0296	2201.0198	4	0	K.EVMQEVAQLSQFDEELYK.V	Oxidation (M)	(Ions score 81)
232 - 249	734.6839	2201.0298	2201.0198	5	0	K.EVMQEVAQLSQFDEELYK.V	Oxidation (M)	(Ions score 44)
232 - 249	734.6839	2201.0298	2201.0198	5	0	K.EVMQEVAQLSQFDEELYK.V	Oxidation (M)	(Ions score 44)
232 - 249	734.6839	2201.0299	2201.0198	5	0	K.EVMQEVAQLSQFDEELYK.V	Oxidation (M)	(Ions score 38)
232 - 249	1101.5223	2201.0300	2201.0198	5	0	K.EVMQEVAQLSQFDEELYK.V	Oxidation (M)	(Ions score 86)
232 - 249	734.6840	2201.0300	2201.0198	5	0	K.EVMQEVAQLSQFDEELYK.V	Oxidation (M)	(Ions score 30)
232 - 249	734.6840	2201.0301	2201.0198	5	0	K.EVMQEVAQLSQFDEELYK.V	Oxidation (M)	(Ions score 49)
232 - 249	734.6840	2201.0301	2201.0198	5	0	K.EVMQEVAQLSQFDEELYK.V	Oxidation (M)	(Ions score 26)
232 - 249	734.6840	2201.0301	2201.0198	5	0	K.EVMQEVAQLSQFDEELYK.V	Oxidation (M)	(Ions score 38)
232 - 249	1101.5223	2201.0301	2201.0198	5	0	K.EVMQEVAQLSQFDEELYK.V	Oxidation (M)	(Ions score 52)
232 - 249	734.6840	2201.0302	2201.0198	5	0	K.EVMQEVAQLSQFDEELYK.V	Oxidation (M)	(Ions score 46)
232 - 249	734.6840	2201.0302	2201.0198	5	0	K.EVMQEVAQLSQFDEELYK.V	Oxidation (M)	(Ions score 47)
232 - 249	734.6840	2201.0303	2201.0198	5	0	K.EVMQEVAQLSQFDEELYK.V	Oxidation (M)	(Ions score 35)
232 - 249	734.6841	2201.0304	2201.0198	5	0	K.EVMQEVAQLSQFDEELYK.V	Oxidation (M)	(Ions score 29)
232 - 249	1101.5226	2201.0306	2201.0198	5	0	K.EVMQEVAQLSQFDEELYK.V	Oxidation (M)	(Ions score 74)
232 - 249	1101.5226	2201.0306	2201.0198	5	0	K.EVMQEVAQLSQFDEELYK.V	Oxidation (M)	(Ions score 63)
232 - 249	1101.5226	2201.0306	2201.0198	5	0	K.EVMQEVAQLSQFDEELYK.V	Oxidation (M)	(Ions score 67)
232 - 249	734.6842	2201.0306	2201.0198	5	0	K.EVMQEVAQLSQFDEELYK.V	Oxidation (M)	(Ions score 39)
232 - 249	734.6842	2201.0307	2201.0198	5	0	K.EVMQEVAQLSQFDEELYK.V	Oxidation (M)	(Ions score 35)
232 - 249	1101.5227	2201.0308	2201.0198	5	0	K.EVMQEVAQLSQFDEELYK.V	Oxidation (M)	(Ions score 66)
232 - 249	734.6842	2201.0308	2201.0198	5	0	K.EVMQEVAQLSQFDEELYK.V	Oxidation (M)	(Ions score 43)
232 - 249	1122.5279	2243.0412	2243.0303	5	0	K.EVMQEVAQLSQFDEELYK.V	Acetylation (K); Oxidation (M)	(Ions score 32)
232 - 253	1292.1599	2582.3053	2582.2938	4	1	K.EVMQEVAQLSQFDEELYKVIGK.G		(Ions score 71)
232 - 253	861.7758	2582.3055	2582.2938	5	1	K.EVMQEVAQLSQFDEELYKVIGK.G		(Ions score 52)
232 - 253	1300.1568	2598.2991	2598.2887	4	1	K.EVMQEVAQLSQFDEELYKVIGK.G	Oxidation (M)	(Ions score 23)
232 - 253	867.1071	2598.2993	2598.2887	4	1	K.EVMQEVAQLSQFDEELYKVIGK.G	Oxidation (M)	(Ions score 55)
232 - 253	867.1071	2598.2996	2598.2887	4	1	K.EVMQEVAQLSQFDEELYKVIGK.G	Oxidation (M)	(Ions score 61)
254 - 266	737.3015	1472.5885	1472.5903	-1	1	K.GSEKSDDNSYDEK.Y		(Ions score 30)
254 - 266	737.3035	1472.5925	1472.5903	1	1	K.GSEKSDDNSYDEK.Y		(Ions score 51)
254 - 266	491.8716	1472.5931	1472.5903	2	1	K.GSEKSDDNSYDEK.Y		(Ions score 25)
254 - 266	737.3038	1472.5931	1472.5903	2	1	K.GSEKSDDNSYDEK.Y		(Ions score 49)
254 - 266	491.8718	1472.5937	1472.5903	2	1	K.GSEKSDDNSYDEK.Y		(Ions score 27)
254 - 266	737.3043	1472.5940	1472.5903	3	1	K.GSEKSDDNSYDEK.Y		(Ions score 37)
254 - 266	737.3044	1472.5942	1472.5903	3	1	K.GSEKSDDNSYDEK.Y		(Ions score 69)
254 - 266	737.3044	1472.5943	1472.5903	3	1	K.GSEKSDDNSYDEK.Y		(Ions score 68)
254 - 266	491.8722	1472.5948	1472.5903	3	1	K.GSEKSDDNSYDEK.Y		(Ions score 29)
254 - 266	758.3083	1514.6020	1514.6008	1	1	K.GSEKSDDNSYDEK.Y	Acetylation (K)	(Ions score 91)
254 - 266	758.3086	1514.6027	1514.6008	1	1	K.GSEKSDDNSYDEK.Y	Acetylation (K)	(Ions score 70)
258 - 266	536.7065	1071.3985	1071.3992	-1	0	K.SDDNSYDEK.Y		(Ions score 26)

258 - 266	536.7070	1071.3994	1071.3992	0	0	K.SDDNSYDEK.Y	(Ions score 38)
258 - 266	536.7070	1071.3995	1071.3992	0	0	K.SDDNSYDEK.Y	(Ions score 37)
258 - 266	536.7070	1071.3995	1071.3992	0	0	K.SDDNSYDEK.Y	(Ions score 47)
258 - 266	536.7071	1071.3996	1071.3992	0	0	K.SDDNSYDEK.Y	(Ions score 57)
258 - 266	536.7071	1071.3996	1071.3992	0	0	K.SDDNSYDEK.Y	(Ions score 28)
258 - 266	536.7073	1071.4000	1071.3992	1	0	K.SDDNSYDEK.Y	(Ions score 56)
258 - 266	536.7073	1071.4001	1071.3992	1	0	K.SDDNSYDEK.Y	(Ions score 50)
258 - 266	536.7073	1071.4001	1071.3992	1	0	K.SDDNSYDEK.Y	(Ions score 25)
258 - 266	536.7073	1071.4001	1071.3992	1	0	K.SDDNSYDEK.Y	(Ions score 35)
258 - 266	536.7074	1071.4002	1071.3992	1	0	K.SDDNSYDEK.Y	(Ions score 57)
258 - 266	536.7075	1071.4004	1071.3992	1	0	K.SDDNSYDEK.Y	(Ions score 43)
258 - 266	536.7075	1071.4005	1071.3992	1	0	K.SDDNSYDEK.Y	(Ions score 41)
258 - 266	536.7076	1071.4006	1071.3992	1	0	K.SDDNSYDEK.Y	(Ions score 57)
258 - 266	536.7076	1071.4006	1071.3992	1	0	K.SDDNSYDEK.Y	(Ions score 41)
258 - 266	536.7076	1071.4007	1071.3992	1	0	K.SDDNSYDEK.Y	(Ions score 24)
258 - 266	536.7076	1071.4007	1071.3992	1	0	K.SDDNSYDEK.Y	(Ions score 35)
258 - 266	536.7077	1071.4009	1071.3992	2	0	K.SDDNSYDEK.Y	(Ions score 26)
258 - 266	536.7078	1071.4010	1071.3992	2	0	K.SDDNSYDEK.Y	(Ions score 33)
258 - 266	536.7078	1071.4010	1071.3992	2	0	K.SDDNSYDEK.Y	(Ions score 55)
258 - 266	536.7078	1071.4011	1071.3992	2	0	K.SDDNSYDEK.Y	(Ions score 35)
258 - 266	536.7078	1071.4011	1071.3992	2	0	K.SDDNSYDEK.Y	(Ions score 47)
258 - 266	536.7078	1071.4011	1071.3992	2	0	K.SDDNSYDEK.Y	(Ions score 47)
258 - 266	536.7078	1071.4011	1071.3992	2	0	K.SDDNSYDEK.Y	(Ions score 44)
258 - 266	536.7078	1071.4011	1071.3992	2	0	K.SDDNSYDEK.Y	(Ions score 41)
258 - 266	536.7078	1071.4012	1071.3992	2	0	K.SDDNSYDEK.Y	(Ions score 61)
258 - 266	536.7079	1071.4012	1071.3992	2	0	K.SDDNSYDEK.Y	(Ions score 49)
258 - 266	536.7079	1071.4013	1071.3992	2	0	K.SDDNSYDEK.Y	(Ions score 56)
258 - 266	536.7079	1071.4013	1071.3992	2	0	K.SDDNSYDEK.Y	(Ions score 53)
258 - 266	536.7079	1071.4013	1071.3992	2	0	K.SDDNSYDEK.Y	(Ions score 49)
258 - 266	536.7080	1071.4014	1071.3992	2	0	K.SDDNSYDEK.Y	(Ions score 34)
258 - 266	536.7080	1071.4014	1071.3992	2	0	K.SDDNSYDEK.Y	(Ions score 42)
258 - 266	536.7080	1071.4014	1071.3992	2	0	K.SDDNSYDEK.Y	(Ions score 42)
258 - 266	536.7080	1071.4014	1071.3992	2	0	K.SDDNSYDEK.Y	(Ions score 54)
258 - 266	536.7080	1071.4015	1071.3992	2	0	K.SDDNSYDEK.Y	(Ions score 50)
258 - 266	536.7080	1071.4015	1071.3992	2	0	K.SDDNSYDEK.Y	(Ions score 46)
258 - 266	536.7080	1071.4015	1071.3992	2	0	K.SDDNSYDEK.Y	(Ions score 29)
258 - 266	536.7080	1071.4015	1071.3992	2	0	K.SDDNSYDEK.Y	(Ions score 21)
258 - 266	536.7080	1071.4015	1071.3992	2	0	K.SDDNSYDEK.Y	(Ions score 50)
258 - 266	536.7080	1071.4015	1071.3992	2	0	K.SDDNSYDEK.Y	(Ions score 56)
258 - 266	536.7080	1071.4015	1071.3992	2	0	K.SDDNSYDEK.Y	(Ions score 56)
258 - 266	536.7080	1071.4015	1071.3992	2	0	K.SDDNSYDEK.Y	(Ions score 60)
258 - 266	536.7080	1071.4015	1071.3992	2	0	K.SDDNSYDEK.Y	(Ions score 55)
258 - 266	536.7080	1071.4015	1071.3992	2	0	K.SDDNSYDEK.Y	(Ions score 41)
258 - 266	536.7080	1071.4015	1071.3992	2	0	K.SDDNSYDEK.Y	(Ions score 50)
258 - 266	536.7081	1071.4016	1071.3992	2	0	K.SDDNSYDEK.Y	(Ions score 35)
258 - 266	536.7081	1071.4016	1071.3992	2	0	K.SDDNSYDEK.Y	(Ions score 55)
258 - 266	536.7081	1071.4016	1071.3992	2	0	K.SDDNSYDEK.Y	(Ions score 57)
258 - 266	536.7081	1071.4016	1071.3992	2	0	K.SDDNSYDEK.Y	(Ions score 57)
258 - 266	536.7081	1071.4016	1071.3992	2	0	K.SDDNSYDEK.Y	(Ions score 27)
258 - 266	536.7081	1071.4016	1071.3992	2	0	K.SDDNSYDEK.Y	(Ions score 57)
258 - 266	536.7081	1071.4017	1071.3992	2	0	K.SDDNSYDEK.Y	(Ions score 55)
258 - 266	536.7081	1071.4017	1071.3992	2	0	K.SDDNSYDEK.Y	(Ions score 49)
258 - 266	536.7081	1071.4017	1071.3992	2	0	K.SDDNSYDEK.Y	(Ions score 34)
258 - 266	536.7081	1071.4017	1071.3992	2	0	K.SDDNSYDEK.Y	(Ions score 30)
258 - 266	536.7081	1071.4017	1071.3992	2	0	K.SDDNSYDEK.Y	(Ions score 58)
258 - 266	536.7081	1071.4017	1071.3992	2	0	K.SDDNSYDEK.Y	(Ions score 56)
258 - 266	536.7081	1071.4017	1071.3992	2	0	K.SDDNSYDEK.Y	(Ions score 46)

258 - 266	536.7081	1071.4017	1071.3992	2	0	K.SDDNSYDEK.Y	(Ions score 60)
258 - 266	536.7081	1071.4017	1071.3992	2	0	K.SDDNSYDEK.Y	(Ions score 31)
258 - 266	536.7082	1071.4018	1071.3992	2	0	K.SDDNSYDEK.Y	(Ions score 57)
258 - 266	536.7082	1071.4018	1071.3992	2	0	K.SDDNSYDEK.Y	(Ions score 43)
258 - 266	536.7082	1071.4018	1071.3992	2	0	K.SDDNSYDEK.Y	(Ions score 47)
258 - 266	536.7082	1071.4018	1071.3992	2	0	K.SDDNSYDEK.Y	(Ions score 58)
258 - 266	536.7082	1071.4018	1071.3992	2	0	K.SDDNSYDEK.Y	(Ions score 56)
258 - 266	536.7082	1071.4018	1071.3992	2	0	K.SDDNSYDEK.Y	(Ions score 44)
258 - 266	536.7082	1071.4018	1071.3992	2	0	K.SDDNSYDEK.Y	(Ions score 44)
258 - 266	536.7082	1071.4019	1071.3992	2	0	K.SDDNSYDEK.Y	(Ions score 31)
258 - 266	536.7082	1071.4019	1071.3992	2	0	K.SDDNSYDEK.Y	(Ions score 40)
258 - 266	536.7082	1071.4019	1071.3992	2	0	K.SDDNSYDEK.Y	(Ions score 31)
258 - 266	536.7082	1071.4019	1071.3992	2	0	K.SDDNSYDEK.Y	(Ions score 35)
258 - 266	536.7082	1071.4019	1071.3992	2	0	K.SDDNSYDEK.Y	(Ions score 69)
258 - 266	536.7082	1071.4019	1071.3992	3	0	K.SDDNSYDEK.Y	(Ions score 40)
258 - 266	536.7083	1071.4020	1071.3992	3	0	K.SDDNSYDEK.Y	(Ions score 25)
258 - 266	536.7083	1071.4020	1071.3992	3	0	K.SDDNSYDEK.Y	(Ions score 44)
258 - 266	536.7083	1071.4020	1071.3992	3	0	K.SDDNSYDEK.Y	(Ions score 57)
258 - 266	536.7083	1071.4020	1071.3992	3	0	K.SDDNSYDEK.Y	(Ions score 43)
258 - 266	536.7083	1071.4020	1071.3992	3	0	K.SDDNSYDEK.Y	(Ions score 42)
258 - 266	536.7083	1071.4020	1071.3992	3	0	K.SDDNSYDEK.Y	(Ions score 26)
258 - 266	536.7083	1071.4020	1071.3992	3	0	K.SDDNSYDEK.Y	(Ions score 56)
258 - 266	536.7083	1071.4021	1071.3992	3	0	K.SDDNSYDEK.Y	(Ions score 28)
258 - 266	536.7083	1071.4021	1071.3992	3	0	K.SDDNSYDEK.Y	(Ions score 36)
258 - 266	536.7083	1071.4021	1071.3992	3	0	K.SDDNSYDEK.Y	(Ions score 56)
258 - 266	536.7083	1071.4021	1071.3992	3	0	K.SDDNSYDEK.Y	(Ions score 29)
258 - 266	536.7083	1071.4021	1071.3992	3	0	K.SDDNSYDEK.Y	(Ions score 41)
258 - 266	536.7084	1071.4023	1071.3992	3	0	K.SDDNSYDEK.Y	(Ions score 35)
258 - 266	536.7084	1071.4023	1071.3992	3	0	K.SDDNSYDEK.Y	(Ions score 23)
258 - 266	536.7085	1071.4024	1071.3992	3	0	K.SDDNSYDEK.Y	(Ions score 27)
258 - 266	536.7085	1071.4025	1071.3992	3	0	K.SDDNSYDEK.Y	(Ions score 50)
258 - 266	536.7086	1071.4026	1071.3992	3	0	K.SDDNSYDEK.Y	(Ions score 32)
258 - 266	536.7086	1071.4027	1071.3992	3	0	K.SDDNSYDEK.Y	(Ions score 50)
258 - 266	536.7086	1071.4027	1071.3992	3	0	K.SDDNSYDEK.Y	(Ions score 41)
258 - 266	536.7087	1071.4028	1071.3992	3	0	K.SDDNSYDEK.Y	(Ions score 29)
258 - 266	536.7087	1071.4029	1071.3992	3	0	K.SDDNSYDEK.Y	(Ions score 25)
258 - 266	536.7087	1071.4029	1071.3992	3	0	K.SDDNSYDEK.Y	(Ions score 37)
258 - 266	536.7088	1071.4030	1071.3992	4	0	K.SDDNSYDEK.Y	(Ions score 37)
258 - 266	536.7088	1071.4031	1071.3992	4	0	K.SDDNSYDEK.Y	(Ions score 26)
258 - 266	536.7088	1071.4031	1071.3992	4	0	K.SDDNSYDEK.Y	(Ions score 26)
258 - 266	536.7089	1071.4032	1071.3992	4	0	K.SDDNSYDEK.Y	(Ions score 31)
258 - 266	536.7090	1071.4034	1071.3992	4	0	K.SDDNSYDEK.Y	(Ions score 49)
258 - 266	536.7090	1071.4034	1071.3992	4	0	K.SDDNSYDEK.Y	(Ions score 34)
258 - 266	536.7090	1071.4035	1071.3992	4	0	K.SDDNSYDEK.Y	(Ions score 36)
258 - 266	536.7090	1071.4035	1071.3992	4	0	K.SDDNSYDEK.Y	(Ions score 35)
258 - 266	536.7091	1071.4036	1071.3992	4	0	K.SDDNSYDEK.Y	(Ions score 37)
258 - 266	536.7091	1071.4037	1071.3992	4	0	K.SDDNSYDEK.Y	(Ions score 26)
258 - 266	536.7091	1071.4037	1071.3992	4	0	K.SDDNSYDEK.Y	(Ions score 30)
258 - 266	536.7091	1071.4037	1071.3992	4	0	K.SDDNSYDEK.Y	(Ions score 32)
258 - 266	536.7091	1071.4037	1071.3992	4	0	K.SDDNSYDEK.Y	(Ions score 36)
258 - 266	536.7092	1071.4038	1071.3992	4	0	K.SDDNSYDEK.Y	(Ions score 41)
258 - 266	536.7092	1071.4039	1071.3992	4	0	K.SDDNSYDEK.Y	(Ions score 36)
258 - 266	536.7092	1071.4039	1071.3992	4	0	K.SDDNSYDEK.Y	(Ions score 37)
258 - 266	536.7092	1071.4039	1071.3992	4	0	K.SDDNSYDEK.Y	(Ions score 37)
258 - 266	536.7093	1071.4040	1071.3992	4	0	K.SDDNSYDEK.Y	(Ions score 29)
258 - 266	536.7093	1071.4041	1071.3992	4	0	K.SDDNSYDEK.Y	(Ions score 35)
258 - 266	536.7094	1071.4041	1071.3992	5	0	K.SDDNSYDEK.Y	(Ions score 41)

258 - 266	536.7094	1071.4042	1071.3992	5	0	K.SDDNSYDEK.Y		(Ions score 34)
258 - 266	536.7094	1071.4043	1071.3992	5	0	K.SDDNSYDEK.Y		(Ions score 35)
258 - 266	536.7095	1071.4044	1071.3992	5	0	K.SDDNSYDEK.Y		(Ions score 36)
258 - 266	536.7095	1071.4044	1071.3992	5	0	K.SDDNSYDEK.Y		(Ions score 36)
258 - 266	536.7095	1071.4044	1071.3992	5	0	K.SDDNSYDEK.Y		(Ions score 26)
258 - 266	536.7095	1071.4045	1071.3992	5	0	K.SDDNSYDEK.Y		(Ions score 30)
258 - 266	536.7095	1071.4045	1071.3992	5	0	K.SDDNSYDEK.Y		(Ions score 42)
258 - 266	536.7096	1071.4046	1071.3992	5	0	K.SDDNSYDEK.Y		(Ions score 41)
258 - 281	912.7763	2735.3070	2735.3038	1	1	K.SDDNSYDEKYLIATSEQPIAALHR.D		(Ions score 59)
258 - 281	548.0692	2735.3097	2735.3038	2	1	K.SDDNSYDEKYLIATSEQPIAALHR.D		(Ions score 40)
258 - 281	684.8348	2735.3101	2735.3038	2	1	K.SDDNSYDEKYLIATSEQPIAALHR.D		(Ions score 25)
258 - 281	684.8352	2735.3119	2735.3038	3	1	K.SDDNSYDEKYLIATSEQPIAALHR.D		(Ions score 38)
258 - 281	912.7781	2735.3126	2735.3038	3	1	K.SDDNSYDEKYLIATSEQPIAALHR.D		(Ions score 40)
258 - 281	684.8355	2735.3127	2735.3038	3	1	K.SDDNSYDEKYLIATSEQPIAALHR.D		(Ions score 46)
258 - 281	548.0699	2735.3133	2735.3038	3	1	K.SDDNSYDEKYLIATSEQPIAALHR.D		(Ions score 21)
258 - 281	695.3376	2777.3211	2777.3144	2	1	K.SDDNSYDEKYLIATSEQPIAALHR.D	Acetylation (K)	(Ions score 35)
258 - 281	695.3391	2777.3274	2777.3144	5	1	K.SDDNSYDEKYLIATSEQPIAALHR.D	Acetylation (K)	(Ions score 64)
267 - 281	561.6447	1681.9123	1681.9151	-2	0	K.YLIATSEQPIAALHR.D		(Ions score 70)
267 - 281	561.6452	1681.9138	1681.9151	-1	0	K.YLIATSEQPIAALHR.D		(Ions score 83)
267 - 281	561.6453	1681.9140	1681.9151	-1	0	K.YLIATSEQPIAALHR.D		(Ions score 88)
267 - 281	561.6453	1681.9141	1681.9151	-1	0	K.YLIATSEQPIAALHR.D		(Ions score 66)
267 - 281	561.6454	1681.9142	1681.9151	-1	0	K.YLIATSEQPIAALHR.D		(Ions score 66)
267 - 281	561.6454	1681.9143	1681.9151	-0	0	K.YLIATSEQPIAALHR.D		(Ions score 56)
267 - 281	561.6454	1681.9145	1681.9151	-0	0	K.YLIATSEQPIAALHR.D		(Ions score 81)
267 - 281	561.6454	1681.9145	1681.9151	-0	0	K.YLIATSEQPIAALHR.D		(Ions score 90)
267 - 281	561.6455	1681.9145	1681.9151	-0	0	K.YLIATSEQPIAALHR.D		(Ions score 32)
267 - 281	561.6455	1681.9146	1681.9151	-0	0	K.YLIATSEQPIAALHR.D		(Ions score 72)
267 - 281	561.6455	1681.9147	1681.9151	-0	0	K.YLIATSEQPIAALHR.D		(Ions score 72)
267 - 281	841.9646	1681.9147	1681.9151	-0	0	K.YLIATSEQPIAALHR.D		(Ions score 70)
267 - 281	561.6456	1681.9149	1681.9151	-0	0	K.YLIATSEQPIAALHR.D		(Ions score 35)
267 - 281	561.6457	1681.9152	1681.9151	0	0	K.YLIATSEQPIAALHR.D		(Ions score 61)
267 - 281	561.6457	1681.9153	1681.9151	0	0	K.YLIATSEQPIAALHR.D		(Ions score 77)
267 - 281	561.6457	1681.9154	1681.9151	0	0	K.YLIATSEQPIAALHR.D		(Ions score 71)
267 - 281	561.6458	1681.9154	1681.9151	0	0	K.YLIATSEQPIAALHR.D		(Ions score 75)
267 - 281	421.4862	1681.9156	1681.9151	0	0	K.YLIATSEQPIAALHR.D		(Ions score 21)
267 - 281	561.6458	1681.9156	1681.9151	0	0	K.YLIATSEQPIAALHR.D		(Ions score 72)
267 - 281	561.6458	1681.9156	1681.9151	0	0	K.YLIATSEQPIAALHR.D		(Ions score 81)
267 - 281	561.6458	1681.9157	1681.9151	0	0	K.YLIATSEQPIAALHR.D		(Ions score 73)
267 - 281	561.6459	1681.9158	1681.9151	0	0	K.YLIATSEQPIAALHR.D		(Ions score 68)
267 - 281	561.6459	1681.9158	1681.9151	0	0	K.YLIATSEQPIAALHR.D		(Ions score 61)
267 - 281	561.6459	1681.9158	1681.9151	0	0	K.YLIATSEQPIAALHR.D		(Ions score 66)
267 - 281	561.6459	1681.9158	1681.9151	0	0	K.YLIATSEQPIAALHR.D		(Ions score 73)
267 - 281	841.9652	1681.9159	1681.9151	0	0	K.YLIATSEQPIAALHR.D		(Ions score 64)
267 - 281	841.9652	1681.9159	1681.9151	0	0	K.YLIATSEQPIAALHR.D		(Ions score 91)
267 - 281	421.4863	1681.9160	1681.9151	1	0	K.YLIATSEQPIAALHR.D		(Ions score 43)
267 - 281	561.6459	1681.9160	1681.9151	1	0	K.YLIATSEQPIAALHR.D		(Ions score 73)
267 - 281	561.6459	1681.9160	1681.9151	1	0	K.YLIATSEQPIAALHR.D		(Ions score 86)
267 - 281	561.6459	1681.9160	1681.9151	1	0	K.YLIATSEQPIAALHR.D		(Ions score 66)
267 - 281	561.6460	1681.9162	1681.9151	1	0	K.YLIATSEQPIAALHR.D		(Ions score 72)
267 - 281	561.6460	1681.9163	1681.9151	1	0	K.YLIATSEQPIAALHR.D		(Ions score 42)
267 - 281	561.6460	1681.9163	1681.9151	1	0	K.YLIATSEQPIAALHR.D		(Ions score 29)
267 - 281	421.4864	1681.9163	1681.9151	1	0	K.YLIATSEQPIAALHR.D		(Ions score 49)
267 - 281	561.6461	1681.9163	1681.9151	1	0	K.YLIATSEQPIAALHR.D		(Ions score 34)
267 - 281	561.6461	1681.9164	1681.9151	1	0	K.YLIATSEQPIAALHR.D		(Ions score 78)
267 - 281	561.6461	1681.9164	1681.9151	1	0	K.YLIATSEQPIAALHR.D		(Ions score 53)
267 - 281	561.6461	1681.9164	1681.9151	1	0	K.YLIATSEQPIAALHR.D		(Ions score 60)
267 - 281	561.6461	1681.9164	1681.9151	1	0	K.YLIATSEQPIAALHR.D		(Ions score 81)

267 - 281	561.6461	1681.9165	1681.9151	1	0	K.YLIATSEQPIAALHR.D	(Ions score 23)
267 - 281	561.6461	1681.9165	1681.9151	1	0	K.YLIATSEQPIAALHR.D	(Ions score 62)
267 - 281	561.6461	1681.9165	1681.9151	1	0	K.YLIATSEQPIAALHR.D	(Ions score 69)
267 - 281	561.6461	1681.9165	1681.9151	1	0	K.YLIATSEQPIAALHR.D	(Ions score 66)
267 - 281	561.6461	1681.9166	1681.9151	1	0	K.YLIATSEQPIAALHR.D	(Ions score 44)
267 - 281	421.4864	1681.9167	1681.9151	1	0	K.YLIATSEQPIAALHR.D	(Ions score 47)
267 - 281	561.6462	1681.9167	1681.9151	1	0	K.YLIATSEQPIAALHR.D	(Ions score 56)
267 - 281	561.6462	1681.9167	1681.9151	1	0	K.YLIATSEQPIAALHR.D	(Ions score 38)
267 - 281	561.6462	1681.9167	1681.9151	1	0	K.YLIATSEQPIAALHR.D	(Ions score 71)
267 - 281	561.6462	1681.9168	1681.9151	1	0	K.YLIATSEQPIAALHR.D	(Ions score 79)
267 - 281	561.6462	1681.9168	1681.9151	1	0	K.YLIATSEQPIAALHR.D	(Ions score 59)
267 - 281	561.6463	1681.9169	1681.9151	1	0	K.YLIATSEQPIAALHR.D	(Ions score 72)
267 - 281	561.6463	1681.9169	1681.9151	1	0	K.YLIATSEQPIAALHR.D	(Ions score 69)
267 - 281	561.6463	1681.9169	1681.9151	1	0	K.YLIATSEQPIAALHR.D	(Ions score 73)
267 - 281	421.4865	1681.9170	1681.9151	1	0	K.YLIATSEQPIAALHR.D	(Ions score 58)
267 - 281	561.6463	1681.9170	1681.9151	1	0	K.YLIATSEQPIAALHR.D	(Ions score 73)
267 - 281	561.6463	1681.9170	1681.9151	1	0	K.YLIATSEQPIAALHR.D	(Ions score 49)
267 - 281	841.9658	1681.9170	1681.9151	1	0	K.YLIATSEQPIAALHR.D	(Ions score 67)
267 - 281	561.6463	1681.9171	1681.9151	1	0	K.YLIATSEQPIAALHR.D	(Ions score 30)
267 - 281	561.6463	1681.9171	1681.9151	1	0	K.YLIATSEQPIAALHR.D	(Ions score 29)
267 - 281	561.6463	1681.9171	1681.9151	1	0	K.YLIATSEQPIAALHR.D	(Ions score 75)
267 - 281	561.6463	1681.9171	1681.9151	1	0	K.YLIATSEQPIAALHR.D	(Ions score 73)
267 - 281	421.4865	1681.9171	1681.9151	1	0	K.YLIATSEQPIAALHR.D	(Ions score 49)
267 - 281	421.4866	1681.9171	1681.9151	1	0	K.YLIATSEQPIAALHR.D	(Ions score 47)
267 - 281	561.6464	1681.9173	1681.9151	1	0	K.YLIATSEQPIAALHR.D	(Ions score 55)
267 - 281	561.6464	1681.9173	1681.9151	1	0	K.YLIATSEQPIAALHR.D	(Ions score 63)
267 - 281	561.6464	1681.9173	1681.9151	1	0	K.YLIATSEQPIAALHR.D	(Ions score 61)
267 - 281	1682.9246	1681.9173	1681.9151	1	0	K.YLIATSEQPIAALHR.D	(Ions score 27)
267 - 281	421.4866	1681.9173	1681.9151	1	0	K.YLIATSEQPIAALHR.D	(Ions score 62)
267 - 281	841.9660	1681.9174	1681.9151	1	0	K.YLIATSEQPIAALHR.D	(Ions score 78)
267 - 281	561.6464	1681.9174	1681.9151	1	0	K.YLIATSEQPIAALHR.D	(Ions score 69)
267 - 281	561.6464	1681.9174	1681.9151	1	0	K.YLIATSEQPIAALHR.D	(Ions score 54)
267 - 281	561.6464	1681.9174	1681.9151	1	0	K.YLIATSEQPIAALHR.D	(Ions score 54)
267 - 281	561.6464	1681.9174	1681.9151	1	0	K.YLIATSEQPIAALHR.D	(Ions score 58)
267 - 281	561.6464	1681.9174	1681.9151	1	0	K.YLIATSEQPIAALHR.D	(Ions score 63)
267 - 281	561.6464	1681.9174	1681.9151	1	0	K.YLIATSEQPIAALHR.D	(Ions score 66)
267 - 281	421.4867	1681.9175	1681.9151	1	0	K.YLIATSEQPIAALHR.D	(Ions score 46)
267 - 281	561.6465	1681.9175	1681.9151	1	0	K.YLIATSEQPIAALHR.D	(Ions score 60)
267 - 281	561.6465	1681.9176	1681.9151	1	0	K.YLIATSEQPIAALHR.D	(Ions score 24)
267 - 281	421.4867	1681.9177	1681.9151	2	0	K.YLIATSEQPIAALHR.D	(Ions score 48)
267 - 281	561.6465	1681.9177	1681.9151	2	0	K.YLIATSEQPIAALHR.D	(Ions score 70)
267 - 281	1682.9250	1681.9178	1681.9151	2	0	K.YLIATSEQPIAALHR.D	(Ions score 45)
267 - 281	1682.9250	1681.9178	1681.9151	2	0	K.YLIATSEQPIAALHR.D	(Ions score 58)
267 - 281	561.6465	1681.9178	1681.9151	2	0	K.YLIATSEQPIAALHR.D	(Ions score 40)
267 - 281	561.6465	1681.9178	1681.9151	2	0	K.YLIATSEQPIAALHR.D	(Ions score 69)
267 - 281	561.6465	1681.9178	1681.9151	2	0	K.YLIATSEQPIAALHR.D	(Ions score 62)
267 - 281	561.6465	1681.9178	1681.9151	2	0	K.YLIATSEQPIAALHR.D	(Ions score 67)
267 - 281	561.6466	1681.9179	1681.9151	2	0	K.YLIATSEQPIAALHR.D	(Ions score 53)
267 - 281	561.6466	1681.9179	1681.9151	2	0	K.YLIATSEQPIAALHR.D	(Ions score 83)
267 - 281	561.6466	1681.9179	1681.9151	2	0	K.YLIATSEQPIAALHR.D	(Ions score 41)
267 - 281	421.4868	1681.9179	1681.9151	2	0	K.YLIATSEQPIAALHR.D	(Ions score 47)
267 - 281	421.4868	1681.9179	1681.9151	2	0	K.YLIATSEQPIAALHR.D	(Ions score 47)
267 - 281	561.6466	1681.9179	1681.9151	2	0	K.YLIATSEQPIAALHR.D	(Ions score 69)
267 - 281	561.6466	1681.9179	1681.9151	2	0	K.YLIATSEQPIAALHR.D	(Ions score 69)
267 - 281	841.9662	1681.9179	1681.9151	2	0	K.YLIATSEQPIAALHR.D	(Ions score 96)
267 - 281	561.6466	1681.9180	1681.9151	2	0	K.YLIATSEQPIAALHR.D	(Ions score 61)
267 - 281	841.9663	1681.9180	1681.9151	2	0	K.YLIATSEQPIAALHR.D	(Ions score 85)

267 - 281	561.6466	1681.9180	1681.9151	2	0	K.YLIATSEQPIAALHR.D	(Ions score 40)
267 - 281	561.6466	1681.9180	1681.9151	2	0	K.YLIATSEQPIAALHR.D	(Ions score 38)
267 - 281	561.6466	1681.9180	1681.9151	2	0	K.YLIATSEQPIAALHR.D	(Ions score 73)
267 - 281	561.6466	1681.9180	1681.9151	2	0	K.YLIATSEQPIAALHR.D	(Ions score 58)
267 - 281	561.6466	1681.9180	1681.9151	2	0	K.YLIATSEQPIAALHR.D	(Ions score 59)
267 - 281	561.6466	1681.9180	1681.9151	2	0	K.YLIATSEQPIAALHR.D	(Ions score 65)
267 - 281	561.6466	1681.9180	1681.9151	2	0	K.YLIATSEQPIAALHR.D	(Ions score 49)
267 - 281	841.9663	1681.9180	1681.9151	2	0	K.YLIATSEQPIAALHR.D	(Ions score 80)
267 - 281	561.6466	1681.9181	1681.9151	2	0	K.YLIATSEQPIAALHR.D	(Ions score 75)
267 - 281	421.4868	1681.9181	1681.9151	2	0	K.YLIATSEQPIAALHR.D	(Ions score 41)
267 - 281	841.9663	1681.9181	1681.9151	2	0	K.YLIATSEQPIAALHR.D	(Ions score 101)
267 - 281	841.9663	1681.9181	1681.9151	2	0	K.YLIATSEQPIAALHR.D	(Ions score 105)
267 - 281	421.4868	1681.9181	1681.9151	2	0	K.YLIATSEQPIAALHR.D	(Ions score 42)
267 - 281	841.9663	1681.9181	1681.9151	2	0	K.YLIATSEQPIAALHR.D	(Ions score 100)
267 - 281	561.6467	1681.9181	1681.9151	2	0	K.YLIATSEQPIAALHR.D	(Ions score 58)
267 - 281	561.6467	1681.9182	1681.9151	2	0	K.YLIATSEQPIAALHR.D	(Ions score 41)
267 - 281	561.6467	1681.9182	1681.9151	2	0	K.YLIATSEQPIAALHR.D	(Ions score 74)
267 - 281	561.6467	1681.9182	1681.9151	2	0	K.YLIATSEQPIAALHR.D	(Ions score 70)
267 - 281	561.6467	1681.9183	1681.9151	2	0	K.YLIATSEQPIAALHR.D	(Ions score 83)
267 - 281	421.4868	1681.9183	1681.9151	2	0	K.YLIATSEQPIAALHR.D	(Ions score 33)
267 - 281	561.6467	1681.9183	1681.9151	2	0	K.YLIATSEQPIAALHR.D	(Ions score 62)
267 - 281	561.6467	1681.9183	1681.9151	2	0	K.YLIATSEQPIAALHR.D	(Ions score 65)
267 - 281	561.6467	1681.9183	1681.9151	2	0	K.YLIATSEQPIAALHR.D	(Ions score 69)
267 - 281	841.9664	1681.9183	1681.9151	2	0	K.YLIATSEQPIAALHR.D	(Ions score 88)
267 - 281	841.9664	1681.9183	1681.9151	2	0	K.YLIATSEQPIAALHR.D	(Ions score 83)
267 - 281	561.6467	1681.9183	1681.9151	2	0	K.YLIATSEQPIAALHR.D	(Ions score 64)
267 - 281	561.6467	1681.9184	1681.9151	2	0	K.YLIATSEQPIAALHR.D	(Ions score 65)
267 - 281	561.6467	1681.9184	1681.9151	2	0	K.YLIATSEQPIAALHR.D	(Ions score 71)
267 - 281	561.6467	1681.9184	1681.9151	2	0	K.YLIATSEQPIAALHR.D	(Ions score 57)
267 - 281	561.6467	1681.9184	1681.9151	2	0	K.YLIATSEQPIAALHR.D	(Ions score 60)
267 - 281	561.6467	1681.9184	1681.9151	2	0	K.YLIATSEQPIAALHR.D	(Ions score 69)
267 - 281	841.9665	1681.9184	1681.9151	2	0	K.YLIATSEQPIAALHR.D	(Ions score 78)
267 - 281	841.9665	1681.9184	1681.9151	2	0	K.YLIATSEQPIAALHR.D	(Ions score 88)
267 - 281	561.6467	1681.9184	1681.9151	2	0	K.YLIATSEQPIAALHR.D	(Ions score 77)
267 - 281	841.9665	1681.9184	1681.9151	2	0	K.YLIATSEQPIAALHR.D	(Ions score 85)
267 - 281	561.6467	1681.9184	1681.9151	2	0	K.YLIATSEQPIAALHR.D	(Ions score 59)
267 - 281	841.9665	1681.9184	1681.9151	2	0	K.YLIATSEQPIAALHR.D	(Ions score 98)
267 - 281	561.6468	1681.9185	1681.9151	2	0	K.YLIATSEQPIAALHR.D	(Ions score 75)
267 - 281	841.9665	1681.9185	1681.9151	2	0	K.YLIATSEQPIAALHR.D	(Ions score 72)
267 - 281	561.6468	1681.9185	1681.9151	2	0	K.YLIATSEQPIAALHR.D	(Ions score 74)
267 - 281	561.6468	1681.9185	1681.9151	2	0	K.YLIATSEQPIAALHR.D	(Ions score 79)
267 - 281	841.9665	1681.9185	1681.9151	2	0	K.YLIATSEQPIAALHR.D	(Ions score 99)
267 - 281	561.6468	1681.9185	1681.9151	2	0	K.YLIATSEQPIAALHR.D	(Ions score 25)
267 - 281	561.6468	1681.9185	1681.9151	2	0	K.YLIATSEQPIAALHR.D	(Ions score 54)
267 - 281	561.6468	1681.9185	1681.9151	2	0	K.YLIATSEQPIAALHR.D	(Ions score 37)
267 - 281	561.6468	1681.9185	1681.9151	2	0	K.YLIATSEQPIAALHR.D	(Ions score 38)
267 - 281	561.6468	1681.9185	1681.9151	2	0	K.YLIATSEQPIAALHR.D	(Ions score 73)
267 - 281	561.6468	1681.9185	1681.9151	2	0	K.YLIATSEQPIAALHR.D	(Ions score 45)
267 - 281	561.6468	1681.9186	1681.9151	2	0	K.YLIATSEQPIAALHR.D	(Ions score 62)
267 - 281	561.6468	1681.9186	1681.9151	2	0	K.YLIATSEQPIAALHR.D	(Ions score 43)
267 - 281	561.6468	1681.9186	1681.9151	2	0	K.YLIATSEQPIAALHR.D	(Ions score 65)
267 - 281	561.6468	1681.9186	1681.9151	2	0	K.YLIATSEQPIAALHR.D	(Ions score 62)
267 - 281	841.9666	1681.9186	1681.9151	2	0	K.YLIATSEQPIAALHR.D	(Ions score 85)
267 - 281	561.6468	1681.9186	1681.9151	2	0	K.YLIATSEQPIAALHR.D	(Ions score 59)
267 - 281	561.6468	1681.9187	1681.9151	2	0	K.YLIATSEQPIAALHR.D	(Ions score 53)
267 - 281	561.6468	1681.9187	1681.9151	2	0	K.YLIATSEQPIAALHR.D	(Ions score 62)
267 - 281	561.6469	1681.9187	1681.9151	2	0	K.YLIATSEQPIAALHR.D	(Ions score 27)

267 - 281	561.6469	1681.9187	1681.9151	2	0	K.YLIATSEQPIAALHR.D	(Ions score 28)
267 - 281	561.6469	1681.9187	1681.9151	2	0	K.YLIATSEQPIAALHR.D	(Ions score 75)
267 - 281	561.6469	1681.9187	1681.9151	2	0	K.YLIATSEQPIAALHR.D	(Ions score 45)
267 - 281	841.9666	1681.9187	1681.9151	2	0	K.YLIATSEQPIAALHR.D	(Ions score 97)
267 - 281	561.6469	1681.9188	1681.9151	2	0	K.YLIATSEQPIAALHR.D	(Ions score 64)
267 - 281	561.6469	1681.9188	1681.9151	2	0	K.YLIATSEQPIAALHR.D	(Ions score 35)
267 - 281	561.6469	1681.9188	1681.9151	2	0	K.YLIATSEQPIAALHR.D	(Ions score 64)
267 - 281	561.6469	1681.9188	1681.9151	2	0	K.YLIATSEQPIAALHR.D	(Ions score 67)
267 - 281	561.6469	1681.9189	1681.9151	2	0	K.YLIATSEQPIAALHR.D	(Ions score 23)
267 - 281	841.9667	1681.9189	1681.9151	2	0	K.YLIATSEQPIAALHR.D	(Ions score 100)
267 - 281	841.9667	1681.9189	1681.9151	2	0	K.YLIATSEQPIAALHR.D	(Ions score 62)
267 - 281	561.6469	1681.9189	1681.9151	2	0	K.YLIATSEQPIAALHR.D	(Ions score 56)
267 - 281	561.6469	1681.9189	1681.9151	2	0	K.YLIATSEQPIAALHR.D	(Ions score 58)
267 - 281	561.6469	1681.9189	1681.9151	2	0	K.YLIATSEQPIAALHR.D	(Ions score 40)
267 - 281	561.6469	1681.9189	1681.9151	2	0	K.YLIATSEQPIAALHR.D	(Ions score 69)
267 - 281	561.6469	1681.9189	1681.9151	2	0	K.YLIATSEQPIAALHR.D	(Ions score 58)
267 - 281	561.6469	1681.9189	1681.9151	2	0	K.YLIATSEQPIAALHR.D	(Ions score 44)
267 - 281	561.6469	1681.9189	1681.9151	2	0	K.YLIATSEQPIAALHR.D	(Ions score 81)
267 - 281	561.6469	1681.9189	1681.9151	2	0	K.YLIATSEQPIAALHR.D	(Ions score 84)
267 - 281	561.6469	1681.9190	1681.9151	2	0	K.YLIATSEQPIAALHR.D	(Ions score 75)
267 - 281	561.6469	1681.9190	1681.9151	2	0	K.YLIATSEQPIAALHR.D	(Ions score 68)
267 - 281	841.9668	1681.9190	1681.9151	2	0	K.YLIATSEQPIAALHR.D	(Ions score 81)
267 - 281	561.6469	1681.9190	1681.9151	2	0	K.YLIATSEQPIAALHR.D	(Ions score 53)
267 - 281	561.6469	1681.9190	1681.9151	2	0	K.YLIATSEQPIAALHR.D	(Ions score 69)
267 - 281	841.9668	1681.9190	1681.9151	2	0	K.YLIATSEQPIAALHR.D	(Ions score 98)
267 - 281	421.4870	1681.9190	1681.9151	2	0	K.YLIATSEQPIAALHR.D	(Ions score 34)
267 - 281	841.9668	1681.9190	1681.9151	2	0	K.YLIATSEQPIAALHR.D	(Ions score 105)
267 - 281	841.9668	1681.9190	1681.9151	2	0	K.YLIATSEQPIAALHR.D	(Ions score 78)
267 - 281	561.6469	1681.9190	1681.9151	2	0	K.YLIATSEQPIAALHR.D	(Ions score 48)
267 - 281	561.6469	1681.9190	1681.9151	2	0	K.YLIATSEQPIAALHR.D	(Ions score 74)
267 - 281	561.6470	1681.9190	1681.9151	2	0	K.YLIATSEQPIAALHR.D	(Ions score 81)
267 - 281	561.6470	1681.9190	1681.9151	2	0	K.YLIATSEQPIAALHR.D	(Ions score 72)
267 - 281	561.6470	1681.9190	1681.9151	2	0	K.YLIATSEQPIAALHR.D	(Ions score 62)
267 - 281	561.6470	1681.9191	1681.9151	2	0	K.YLIATSEQPIAALHR.D	(Ions score 67)
267 - 281	561.6470	1681.9191	1681.9151	2	0	K.YLIATSEQPIAALHR.D	(Ions score 63)
267 - 281	561.6470	1681.9191	1681.9151	2	0	K.YLIATSEQPIAALHR.D	(Ions score 62)
267 - 281	561.6470	1681.9191	1681.9151	2	0	K.YLIATSEQPIAALHR.D	(Ions score 64)
267 - 281	561.6470	1681.9191	1681.9151	2	0	K.YLIATSEQPIAALHR.D	(Ions score 31)
267 - 281	421.4871	1681.9191	1681.9151	2	0	K.YLIATSEQPIAALHR.D	(Ions score 45)
267 - 281	561.6470	1681.9191	1681.9151	2	0	K.YLIATSEQPIAALHR.D	(Ions score 65)
267 - 281	561.6470	1681.9192	1681.9151	2	0	K.YLIATSEQPIAALHR.D	(Ions score 64)
267 - 281	561.6470	1681.9192	1681.9151	2	0	K.YLIATSEQPIAALHR.D	(Ions score 58)
267 - 281	841.9669	1681.9192	1681.9151	2	0	K.YLIATSEQPIAALHR.D	(Ions score 100)
267 - 281	561.6470	1681.9192	1681.9151	2	0	K.YLIATSEQPIAALHR.D	(Ions score 47)
267 - 281	841.9669	1681.9192	1681.9151	2	0	K.YLIATSEQPIAALHR.D	(Ions score 53)
267 - 281	561.6470	1681.9192	1681.9151	2	0	K.YLIATSEQPIAALHR.D	(Ions score 73)
267 - 281	561.6470	1681.9192	1681.9151	2	0	K.YLIATSEQPIAALHR.D	(Ions score 85)
267 - 281	561.6470	1681.9192	1681.9151	2	0	K.YLIATSEQPIAALHR.D	(Ions score 28)
267 - 281	561.6470	1681.9192	1681.9151	2	0	K.YLIATSEQPIAALHR.D	(Ions score 69)
267 - 281	561.6470	1681.9193	1681.9151	2	0	K.YLIATSEQPIAALHR.D	(Ions score 78)
267 - 281	561.6470	1681.9193	1681.9151	2	0	K.YLIATSEQPIAALHR.D	(Ions score 67)
267 - 281	561.6470	1681.9193	1681.9151	2	0	K.YLIATSEQPIAALHR.D	(Ions score 38)
267 - 281	561.6470	1681.9193	1681.9151	2	0	K.YLIATSEQPIAALHR.D	(Ions score 52)
267 - 281	561.6470	1681.9193	1681.9151	2	0	K.YLIATSEQPIAALHR.D	(Ions score 68)
267 - 281	561.6470	1681.9193	1681.9151	2	0	K.YLIATSEQPIAALHR.D	(Ions score 58)
267 - 281	561.6470	1681.9193	1681.9151	2	0	K.YLIATSEQPIAALHR.D	(Ions score 59)
267 - 281	561.6470	1681.9193	1681.9151	2	0	K.YLIATSEQPIAALHR.D	(Ions score 65)

267 - 281	561.6471	1681.9193	1681.9151	3	0	K.YLIATSEQPIAALHR.D	(Ions score 81)
267 - 281	841.9670	1681.9194	1681.9151	3	0	K.YLIATSEQPIAALHR.D	(Ions score 75)
267 - 281	561.6471	1681.9194	1681.9151	3	0	K.YLIATSEQPIAALHR.D	(Ions score 73)
267 - 281	561.6471	1681.9194	1681.9151	3	0	K.YLIATSEQPIAALHR.D	(Ions score 69)
267 - 281	561.6471	1681.9194	1681.9151	3	0	K.YLIATSEQPIAALHR.D	(Ions score 66)
267 - 281	561.6471	1681.9194	1681.9151	3	0	K.YLIATSEQPIAALHR.D	(Ions score 55)
267 - 281	561.6471	1681.9194	1681.9151	3	0	K.YLIATSEQPIAALHR.D	(Ions score 66)
267 - 281	421.4871	1681.9194	1681.9151	3	0	K.YLIATSEQPIAALHR.D	(Ions score 47)
267 - 281	561.6471	1681.9194	1681.9151	3	0	K.YLIATSEQPIAALHR.D	(Ions score 79)
267 - 281	561.6471	1681.9195	1681.9151	3	0	K.YLIATSEQPIAALHR.D	(Ions score 31)
267 - 281	561.6471	1681.9195	1681.9151	3	0	K.YLIATSEQPIAALHR.D	(Ions score 40)
267 - 281	561.6471	1681.9195	1681.9151	3	0	K.YLIATSEQPIAALHR.D	(Ions score 62)
267 - 281	561.6471	1681.9195	1681.9151	3	0	K.YLIATSEQPIAALHR.D	(Ions score 69)
267 - 281	561.6471	1681.9195	1681.9151	3	0	K.YLIATSEQPIAALHR.D	(Ions score 49)
267 - 281	561.6471	1681.9195	1681.9151	3	0	K.YLIATSEQPIAALHR.D	(Ions score 74)
267 - 281	561.6471	1681.9195	1681.9151	3	0	K.YLIATSEQPIAALHR.D	(Ions score 73)
267 - 281	561.6471	1681.9195	1681.9151	3	0	K.YLIATSEQPIAALHR.D	(Ions score 72)
267 - 281	561.6471	1681.9195	1681.9151	3	0	K.YLIATSEQPIAALHR.D	(Ions score 76)
267 - 281	561.6471	1681.9195	1681.9151	3	0	K.YLIATSEQPIAALHR.D	(Ions score 72)
267 - 281	561.6471	1681.9195	1681.9151	3	0	K.YLIATSEQPIAALHR.D	(Ions score 84)
267 - 281	561.6471	1681.9196	1681.9151	3	0	K.YLIATSEQPIAALHR.D	(Ions score 76)
267 - 281	561.6471	1681.9196	1681.9151	3	0	K.YLIATSEQPIAALHR.D	(Ions score 69)
267 - 281	841.9671	1681.9196	1681.9151	3	0	K.YLIATSEQPIAALHR.D	(Ions score 64)
267 - 281	841.9671	1681.9196	1681.9151	3	0	K.YLIATSEQPIAALHR.D	(Ions score 83)
267 - 281	561.6471	1681.9196	1681.9151	3	0	K.YLIATSEQPIAALHR.D	(Ions score 75)
267 - 281	561.6471	1681.9196	1681.9151	3	0	K.YLIATSEQPIAALHR.D	(Ions score 70)
267 - 281	561.6471	1681.9196	1681.9151	3	0	K.YLIATSEQPIAALHR.D	(Ions score 76)
267 - 281	561.6472	1681.9196	1681.9151	3	0	K.YLIATSEQPIAALHR.D	(Ions score 66)
267 - 281	561.6472	1681.9196	1681.9151	3	0	K.YLIATSEQPIAALHR.D	(Ions score 22)
267 - 281	561.6472	1681.9196	1681.9151	3	0	K.YLIATSEQPIAALHR.D	(Ions score 81)
267 - 281	561.6472	1681.9196	1681.9151	3	0	K.YLIATSEQPIAALHR.D	(Ions score 62)
267 - 281	561.6472	1681.9197	1681.9151	3	0	K.YLIATSEQPIAALHR.D	(Ions score 55)
267 - 281	561.6472	1681.9197	1681.9151	3	0	K.YLIATSEQPIAALHR.D	(Ions score 69)
267 - 281	561.6472	1681.9197	1681.9151	3	0	K.YLIATSEQPIAALHR.D	(Ions score 71)
267 - 281	561.6472	1681.9197	1681.9151	3	0	K.YLIATSEQPIAALHR.D	(Ions score 32)
267 - 281	561.6472	1681.9197	1681.9151	3	0	K.YLIATSEQPIAALHR.D	(Ions score 61)
267 - 281	561.6472	1681.9197	1681.9151	3	0	K.YLIATSEQPIAALHR.D	(Ions score 73)
267 - 281	561.6472	1681.9198	1681.9151	3	0	K.YLIATSEQPIAALHR.D	(Ions score 75)
267 - 281	561.6472	1681.9198	1681.9151	3	0	K.YLIATSEQPIAALHR.D	(Ions score 74)
267 - 281	561.6472	1681.9198	1681.9151	3	0	K.YLIATSEQPIAALHR.D	(Ions score 78)
267 - 281	561.6472	1681.9198	1681.9151	3	0	K.YLIATSEQPIAALHR.D	(Ions score 22)
267 - 281	561.6472	1681.9198	1681.9151	3	0	K.YLIATSEQPIAALHR.D	(Ions score 55)
267 - 281	561.6472	1681.9198	1681.9151	3	0	K.YLIATSEQPIAALHR.D	(Ions score 35)
267 - 281	561.6472	1681.9199	1681.9151	3	0	K.YLIATSEQPIAALHR.D	(Ions score 66)
267 - 281	561.6472	1681.9199	1681.9151	3	0	K.YLIATSEQPIAALHR.D	(Ions score 75)
267 - 281	841.9672	1681.9199	1681.9151	3	0	K.YLIATSEQPIAALHR.D	(Ions score 99)
267 - 281	561.6472	1681.9199	1681.9151	3	0	K.YLIATSEQPIAALHR.D	(Ions score 57)
267 - 281	561.6472	1681.9199	1681.9151	3	0	K.YLIATSEQPIAALHR.D	(Ions score 73)
267 - 281	561.6472	1681.9199	1681.9151	3	0	K.YLIATSEQPIAALHR.D	(Ions score 78)
267 - 281	561.6472	1681.9199	1681.9151	3	0	K.YLIATSEQPIAALHR.D	(Ions score 68)
267 - 281	561.6472	1681.9199	1681.9151	3	0	K.YLIATSEQPIAALHR.D	(Ions score 70)
267 - 281	561.6472	1681.9199	1681.9151	3	0	K.YLIATSEQPIAALHR.D	(Ions score 65)
267 - 281	561.6472	1681.9199	1681.9151	3	0	K.YLIATSEQPIAALHR.D	(Ions score 80)
267 - 281	561.6473	1681.9199	1681.9151	3	0	K.YLIATSEQPIAALHR.D	(Ions score 73)
267 - 281	561.6473	1681.9199	1681.9151	3	0	K.YLIATSEQPIAALHR.D	(Ions score 70)
267 - 281	561.6473	1681.9200	1681.9151	3	0	K.YLIATSEQPIAALHR.D	(Ions score 67)
267 - 281	561.6473	1681.9200	1681.9151	3	0	K.YLIATSEQPIAALHR.D	(Ions score 84)

267 - 281	561.6473	1681.9200	1681.9151	3	0	K.YLIATSEQPIAALHR.D	(Ions score 69)
267 - 281	561.6473	1681.9200	1681.9151	3	0	K.YLIATSEQPIAALHR.D	(Ions score 81)
267 - 281	561.6473	1681.9200	1681.9151	3	0	K.YLIATSEQPIAALHR.D	(Ions score 72)
267 - 281	561.6473	1681.9200	1681.9151	3	0	K.YLIATSEQPIAALHR.D	(Ions score 73)
267 - 281	561.6473	1681.9200	1681.9151	3	0	K.YLIATSEQPIAALHR.D	(Ions score 64)
267 - 281	561.6473	1681.9200	1681.9151	3	0	K.YLIATSEQPIAALHR.D	(Ions score 43)
267 - 281	561.6473	1681.9201	1681.9151	3	0	K.YLIATSEQPIAALHR.D	(Ions score 43)
267 - 281	561.6473	1681.9201	1681.9151	3	0	K.YLIATSEQPIAALHR.D	(Ions score 91)
267 - 281	561.6473	1681.9201	1681.9151	3	0	K.YLIATSEQPIAALHR.D	(Ions score 46)
267 - 281	561.6473	1681.9201	1681.9151	3	0	K.YLIATSEQPIAALHR.D	(Ions score 50)
267 - 281	561.6473	1681.9201	1681.9151	3	0	K.YLIATSEQPIAALHR.D	(Ions score 47)
267 - 281	561.6473	1681.9201	1681.9151	3	0	K.YLIATSEQPIAALHR.D	(Ions score 65)
267 - 281	561.6473	1681.9202	1681.9151	3	0	K.YLIATSEQPIAALHR.D	(Ions score 65)
267 - 281	561.6473	1681.9202	1681.9151	3	0	K.YLIATSEQPIAALHR.D	(Ions score 67)
267 - 281	561.6473	1681.9202	1681.9151	3	0	K.YLIATSEQPIAALHR.D	(Ions score 54)
267 - 281	561.6474	1681.9203	1681.9151	3	0	K.YLIATSEQPIAALHR.D	(Ions score 77)
267 - 281	561.6474	1681.9203	1681.9151	3	0	K.YLIATSEQPIAALHR.D	(Ions score 64)
267 - 281	561.6474	1681.9204	1681.9151	3	0	K.YLIATSEQPIAALHR.D	(Ions score 45)
267 - 281	561.6474	1681.9204	1681.9151	3	0	K.YLIATSEQPIAALHR.D	(Ions score 72)
267 - 281	561.6474	1681.9204	1681.9151	3	0	K.YLIATSEQPIAALHR.D	(Ions score 64)
267 - 281	561.6474	1681.9204	1681.9151	3	0	K.YLIATSEQPIAALHR.D	(Ions score 67)
267 - 281	561.6474	1681.9204	1681.9151	3	0	K.YLIATSEQPIAALHR.D	(Ions score 74)
267 - 281	561.6474	1681.9204	1681.9151	3	0	K.YLIATSEQPIAALHR.D	(Ions score 65)
267 - 281	561.6474	1681.9204	1681.9151	3	0	K.YLIATSEQPIAALHR.D	(Ions score 71)
267 - 281	561.6474	1681.9205	1681.9151	3	0	K.YLIATSEQPIAALHR.D	(Ions score 70)
267 - 281	561.6474	1681.9205	1681.9151	3	0	K.YLIATSEQPIAALHR.D	(Ions score 61)
267 - 281	561.6474	1681.9205	1681.9151	3	0	K.YLIATSEQPIAALHR.D	(Ions score 64)
267 - 281	561.6475	1681.9205	1681.9151	3	0	K.YLIATSEQPIAALHR.D	(Ions score 71)
267 - 281	561.6475	1681.9206	1681.9151	3	0	K.YLIATSEQPIAALHR.D	(Ions score 84)
267 - 281	561.6475	1681.9206	1681.9151	3	0	K.YLIATSEQPIAALHR.D	(Ions score 77)
267 - 281	561.6475	1681.9206	1681.9151	3	0	K.YLIATSEQPIAALHR.D	(Ions score 79)
267 - 281	561.6475	1681.9207	1681.9151	3	0	K.YLIATSEQPIAALHR.D	(Ions score 86)
267 - 281	561.6475	1681.9207	1681.9151	3	0	K.YLIATSEQPIAALHR.D	(Ions score 67)
267 - 281	561.6475	1681.9207	1681.9151	3	0	K.YLIATSEQPIAALHR.D	(Ions score 58)
267 - 281	561.6475	1681.9207	1681.9151	3	0	K.YLIATSEQPIAALHR.D	(Ions score 75)
267 - 281	561.6475	1681.9207	1681.9151	3	0	K.YLIATSEQPIAALHR.D	(Ions score 81)
267 - 281	841.9676	1681.9207	1681.9151	3	0	K.YLIATSEQPIAALHR.D	(Ions score 67)
267 - 281	561.6475	1681.9207	1681.9151	3	0	K.YLIATSEQPIAALHR.D	(Ions score 64)
267 - 281	561.6475	1681.9208	1681.9151	3	0	K.YLIATSEQPIAALHR.D	(Ions score 72)
267 - 281	561.6476	1681.9208	1681.9151	3	0	K.YLIATSEQPIAALHR.D	(Ions score 82)
267 - 281	561.6476	1681.9209	1681.9151	3	0	K.YLIATSEQPIAALHR.D	(Ions score 77)
267 - 281	561.6476	1681.9209	1681.9151	3	0	K.YLIATSEQPIAALHR.D	(Ions score 65)
267 - 281	561.6476	1681.9209	1681.9151	3	0	K.YLIATSEQPIAALHR.D	(Ions score 69)
267 - 281	561.6476	1681.9209	1681.9151	3	0	K.YLIATSEQPIAALHR.D	(Ions score 72)
267 - 281	561.6476	1681.9209	1681.9151	3	0	K.YLIATSEQPIAALHR.D	(Ions score 75)
267 - 281	561.6476	1681.9209	1681.9151	3	0	K.YLIATSEQPIAALHR.D	(Ions score 67)
267 - 281	561.6476	1681.9209	1681.9151	3	0	K.YLIATSEQPIAALHR.D	(Ions score 69)
267 - 281	561.6476	1681.9209	1681.9151	3	0	K.YLIATSEQPIAALHR.D	(Ions score 71)
267 - 281	561.6476	1681.9209	1681.9151	3	0	K.YLIATSEQPIAALHR.D	(Ions score 79)
267 - 281	561.6476	1681.9209	1681.9151	3	0	K.YLIATSEQPIAALHR.D	(Ions score 69)
267 - 281	561.6476	1681.9209	1681.9151	3	0	K.YLIATSEQPIAALHR.D	(Ions score 81)
267 - 281	561.6476	1681.9209	1681.9151	3	0	K.YLIATSEQPIAALHR.D	(Ions score 49)
267 - 281	561.6476	1681.9209	1681.9151	3	0	K.YLIATSEQPIAALHR.D	(Ions score 58)
267 - 281	561.6476	1681.9210	1681.9151	3	0	K.YLIATSEQPIAALHR.D	(Ions score 62)
267 - 281	561.6476	1681.9210	1681.9151	3	0	K.YLIATSEQPIAALHR.D	(Ions score 61)
267 - 281	561.6476	1681.9210	1681.9151	3	0	K.YLIATSEQPIAALHR.D	(Ions score 60)
267 - 281	561.6476	1681.9210	1681.9151	4	0	K.YLIATSEQPIAALHR.D	(Ions score 66)

267 - 281	561.6476	1681.9210	1681.9151	4	0	K.YLIATSEQPIAALHR.D	(Ions score 76)
267 - 281	561.6476	1681.9210	1681.9151	4	0	K.YLIATSEQPIAALHR.D	(Ions score 65)
267 - 281	561.6476	1681.9211	1681.9151	4	0	K.YLIATSEQPIAALHR.D	(Ions score 75)
267 - 281	561.6476	1681.9211	1681.9151	4	0	K.YLIATSEQPIAALHR.D	(Ions score 75)
267 - 281	561.6476	1681.9211	1681.9151	4	0	K.YLIATSEQPIAALHR.D	(Ions score 73)
267 - 281	561.6476	1681.9211	1681.9151	4	0	K.YLIATSEQPIAALHR.D	(Ions score 69)
267 - 281	561.6477	1681.9212	1681.9151	4	0	K.YLIATSEQPIAALHR.D	(Ions score 62)
267 - 281	561.6477	1681.9212	1681.9151	4	0	K.YLIATSEQPIAALHR.D	(Ions score 57)
267 - 281	841.9679	1681.9212	1681.9151	4	0	K.YLIATSEQPIAALHR.D	(Ions score 77)
267 - 281	561.6477	1681.9212	1681.9151	4	0	K.YLIATSEQPIAALHR.D	(Ions score 65)
267 - 281	561.6477	1681.9213	1681.9151	4	0	K.YLIATSEQPIAALHR.D	(Ions score 73)
267 - 281	561.6477	1681.9213	1681.9151	4	0	K.YLIATSEQPIAALHR.D	(Ions score 42)
267 - 281	561.6477	1681.9213	1681.9151	4	0	K.YLIATSEQPIAALHR.D	(Ions score 74)
267 - 281	561.6477	1681.9213	1681.9151	4	0	K.YLIATSEQPIAALHR.D	(Ions score 81)
267 - 281	561.6477	1681.9214	1681.9151	4	0	K.YLIATSEQPIAALHR.D	(Ions score 58)
267 - 281	561.6477	1681.9214	1681.9151	4	0	K.YLIATSEQPIAALHR.D	(Ions score 69)
267 - 281	561.6477	1681.9214	1681.9151	4	0	K.YLIATSEQPIAALHR.D	(Ions score 69)
267 - 281	561.6478	1681.9214	1681.9151	4	0	K.YLIATSEQPIAALHR.D	(Ions score 79)
267 - 281	561.6478	1681.9214	1681.9151	4	0	K.YLIATSEQPIAALHR.D	(Ions score 75)
267 - 281	561.6478	1681.9214	1681.9151	4	0	K.YLIATSEQPIAALHR.D	(Ions score 64)
267 - 281	561.6478	1681.9214	1681.9151	4	0	K.YLIATSEQPIAALHR.D	(Ions score 65)
267 - 281	561.6478	1681.9214	1681.9151	4	0	K.YLIATSEQPIAALHR.D	(Ions score 55)
267 - 281	561.6478	1681.9215	1681.9151	4	0	K.YLIATSEQPIAALHR.D	(Ions score 54)
267 - 281	561.6478	1681.9215	1681.9151	4	0	K.YLIATSEQPIAALHR.D	(Ions score 79)
267 - 281	561.6478	1681.9215	1681.9151	4	0	K.YLIATSEQPIAALHR.D	(Ions score 73)
267 - 281	561.6478	1681.9215	1681.9151	4	0	K.YLIATSEQPIAALHR.D	(Ions score 44)
267 - 281	561.6478	1681.9216	1681.9151	4	0	K.YLIATSEQPIAALHR.D	(Ions score 77)
267 - 281	561.6478	1681.9216	1681.9151	4	0	K.YLIATSEQPIAALHR.D	(Ions score 72)
267 - 281	561.6478	1681.9216	1681.9151	4	0	K.YLIATSEQPIAALHR.D	(Ions score 67)
267 - 281	561.6478	1681.9216	1681.9151	4	0	K.YLIATSEQPIAALHR.D	(Ions score 74)
267 - 281	561.6478	1681.9216	1681.9151	4	0	K.YLIATSEQPIAALHR.D	(Ions score 65)
267 - 281	561.6478	1681.9216	1681.9151	4	0	K.YLIATSEQPIAALHR.D	(Ions score 43)
267 - 281	561.6478	1681.9217	1681.9151	4	0	K.YLIATSEQPIAALHR.D	(Ions score 73)
267 - 281	561.6478	1681.9217	1681.9151	4	0	K.YLIATSEQPIAALHR.D	(Ions score 67)
267 - 281	561.6479	1681.9217	1681.9151	4	0	K.YLIATSEQPIAALHR.D	(Ions score 63)
267 - 281	561.6479	1681.9218	1681.9151	4	0	K.YLIATSEQPIAALHR.D	(Ions score 68)
267 - 281	561.6479	1681.9218	1681.9151	4	0	K.YLIATSEQPIAALHR.D	(Ions score 77)
267 - 281	561.6479	1681.9218	1681.9151	4	0	K.YLIATSEQPIAALHR.D	(Ions score 70)
267 - 281	561.6479	1681.9218	1681.9151	4	0	K.YLIATSEQPIAALHR.D	(Ions score 70)
267 - 281	561.6479	1681.9219	1681.9151	4	0	K.YLIATSEQPIAALHR.D	(Ions score 40)
267 - 281	561.6479	1681.9219	1681.9151	4	0	K.YLIATSEQPIAALHR.D	(Ions score 64)
267 - 281	561.6479	1681.9220	1681.9151	4	0	K.YLIATSEQPIAALHR.D	(Ions score 75)
267 - 281	561.6479	1681.9220	1681.9151	4	0	K.YLIATSEQPIAALHR.D	(Ions score 80)
267 - 281	561.6480	1681.9220	1681.9151	4	0	K.YLIATSEQPIAALHR.D	(Ions score 79)
267 - 281	561.6480	1681.9221	1681.9151	4	0	K.YLIATSEQPIAALHR.D	(Ions score 75)
267 - 281	841.9683	1681.9221	1681.9151	4	0	K.YLIATSEQPIAALHR.D	(Ions score 42)
267 - 281	561.6480	1681.9221	1681.9151	4	0	K.YLIATSEQPIAALHR.D	(Ions score 77)
267 - 281	561.6480	1681.9221	1681.9151	4	0	K.YLIATSEQPIAALHR.D	(Ions score 69)
267 - 281	841.9683	1681.9221	1681.9151	4	0	K.YLIATSEQPIAALHR.D	(Ions score 69)
267 - 281	561.6480	1681.9222	1681.9151	4	0	K.YLIATSEQPIAALHR.D	(Ions score 49)
267 - 281	561.6480	1681.9222	1681.9151	4	0	K.YLIATSEQPIAALHR.D	(Ions score 79)
267 - 281	561.6480	1681.9222	1681.9151	4	0	K.YLIATSEQPIAALHR.D	(Ions score 66)
267 - 281	561.6480	1681.9222	1681.9151	4	0	K.YLIATSEQPIAALHR.D	(Ions score 57)
267 - 281	561.6480	1681.9223	1681.9151	4	0	K.YLIATSEQPIAALHR.D	(Ions score 69)
267 - 281	561.6480	1681.9223	1681.9151	4	0	K.YLIATSEQPIAALHR.D	(Ions score 74)
267 - 281	561.6480	1681.9223	1681.9151	4	0	K.YLIATSEQPIAALHR.D	(Ions score 81)
267 - 281	561.6481	1681.9223	1681.9151	4	0	K.YLIATSEQPIAALHR.D	(Ions score 79)

267 - 281	561.6481	1681.9224	1681.9151	4	0	K.YLIATSEQPIAALHR.D	(Ions score 72)
267 - 281	561.6481	1681.9224	1681.9151	4	0	K.YLIATSEQPIAALHR.D	(Ions score 67)
267 - 281	561.6481	1681.9224	1681.9151	4	0	K.YLIATSEQPIAALHR.D	(Ions score 72)
267 - 281	561.6481	1681.9224	1681.9151	4	0	K.YLIATSEQPIAALHR.D	(Ions score 71)
267 - 281	561.6481	1681.9225	1681.9151	4	0	K.YLIATSEQPIAALHR.D	(Ions score 72)
267 - 281	561.6481	1681.9225	1681.9151	4	0	K.YLIATSEQPIAALHR.D	(Ions score 73)
267 - 281	561.6481	1681.9225	1681.9151	4	0	K.YLIATSEQPIAALHR.D	(Ions score 72)
267 - 281	561.6481	1681.9226	1681.9151	4	0	K.YLIATSEQPIAALHR.D	(Ions score 79)
267 - 281	561.6482	1681.9226	1681.9151	4	0	K.YLIATSEQPIAALHR.D	(Ions score 83)
267 - 281	561.6482	1681.9226	1681.9151	4	0	K.YLIATSEQPIAALHR.D	(Ions score 70)
267 - 281	561.6482	1681.9227	1681.9151	4	0	K.YLIATSEQPIAALHR.D	(Ions score 67)
267 - 281	561.6482	1681.9227	1681.9151	4	0	K.YLIATSEQPIAALHR.D	(Ions score 69)
267 - 281	561.6482	1681.9228	1681.9151	5	0	K.YLIATSEQPIAALHR.D	(Ions score 73)
267 - 281	561.6482	1681.9228	1681.9151	5	0	K.YLIATSEQPIAALHR.D	(Ions score 69)
267 - 281	561.6482	1681.9229	1681.9151	5	0	K.YLIATSEQPIAALHR.D	(Ions score 77)
267 - 281	561.6483	1681.9230	1681.9151	5	0	K.YLIATSEQPIAALHR.D	(Ions score 74)
267 - 281	841.9688	1681.9231	1681.9151	5	0	K.YLIATSEQPIAALHR.D	(Ions score 60)
267 - 281	841.9688	1681.9231	1681.9151	5	0	K.YLIATSEQPIAALHR.D	(Ions score 60)
267 - 281	561.6483	1681.9232	1681.9151	5	0	K.YLIATSEQPIAALHR.D	(Ions score 44)
267 - 281	561.6484	1681.9232	1681.9151	5	0	K.YLIATSEQPIAALHR.D	(Ions score 65)
267 - 281	561.6484	1681.9233	1681.9151	5	0	K.YLIATSEQPIAALHR.D	(Ions score 67)
267 - 281	561.6484	1681.9233	1681.9151	5	0	K.YLIATSEQPIAALHR.D	(Ions score 70)
267 - 281	561.6484	1681.9233	1681.9151	5	0	K.YLIATSEQPIAALHR.D	(Ions score 66)
267 - 281	561.6484	1681.9233	1681.9151	5	0	K.YLIATSEQPIAALHR.D	(Ions score 67)
267 - 281	561.6484	1681.9235	1681.9151	5	0	K.YLIATSEQPIAALHR.D	(Ions score 73)
267 - 281	561.6484	1681.9235	1681.9151	5	0	K.YLIATSEQPIAALHR.D	(Ions score 75)
282 - 293	755.8995	1509.7844	1509.7827	1	0	R.DEWLRPEDLPIK.Y	(Ions score 26)
282 - 293	755.8997	1509.7848	1509.7827	1	0	R.DEWLRPEDLPIK.Y	(Ions score 28)
282 - 293	755.8998	1509.7851	1509.7827	2	0	R.DEWLRPEDLPIK.Y	(Ions score 54)
282 - 293	755.9000	1509.7854	1509.7827	2	0	R.DEWLRPEDLPIK.Y	(Ions score 54)
282 - 293	1510.7928	1509.7856	1509.7827	2	0	R.DEWLRPEDLPIK.Y	(Ions score 60)
282 - 293	755.9001	1509.7857	1509.7827	2	0	R.DEWLRPEDLPIK.Y	(Ions score 48)
282 - 293	755.9001	1509.7857	1509.7827	2	0	R.DEWLRPEDLPIK.Y	(Ions score 54)
282 - 293	755.9002	1509.7858	1509.7827	2	0	R.DEWLRPEDLPIK.Y	(Ions score 45)
282 - 293	755.9003	1509.7860	1509.7827	2	0	R.DEWLRPEDLPIK.Y	(Ions score 24)
282 - 293	755.9003	1509.7860	1509.7827	2	0	R.DEWLRPEDLPIK.Y	(Ions score 56)
282 - 293	755.9003	1509.7860	1509.7827	2	0	R.DEWLRPEDLPIK.Y	(Ions score 54)
282 - 293	755.9003	1509.7860	1509.7827	2	0	R.DEWLRPEDLPIK.Y	(Ions score 45)
282 - 293	755.9003	1509.7861	1509.7827	2	0	R.DEWLRPEDLPIK.Y	(Ions score 45)
282 - 293	755.9003	1509.7861	1509.7827	2	0	R.DEWLRPEDLPIK.Y	(Ions score 41)
282 - 293	755.9003	1509.7861	1509.7827	2	0	R.DEWLRPEDLPIK.Y	(Ions score 56)
282 - 293	755.9004	1509.7862	1509.7827	2	0	R.DEWLRPEDLPIK.Y	(Ions score 43)
282 - 293	755.9004	1509.7862	1509.7827	2	0	R.DEWLRPEDLPIK.Y	(Ions score 44)
282 - 293	755.9004	1509.7862	1509.7827	2	0	R.DEWLRPEDLPIK.Y	(Ions score 54)
282 - 293	755.9004	1509.7863	1509.7827	2	0	R.DEWLRPEDLPIK.Y	(Ions score 53)
282 - 293	755.9004	1509.7863	1509.7827	2	0	R.DEWLRPEDLPIK.Y	(Ions score 59)
282 - 293	755.9004	1509.7863	1509.7827	2	0	R.DEWLRPEDLPIK.Y	(Ions score 53)
282 - 293	755.9004	1509.7863	1509.7827	2	0	R.DEWLRPEDLPIK.Y	(Ions score 42)
282 - 293	755.9004	1509.7863	1509.7827	2	0	R.DEWLRPEDLPIK.Y	(Ions score 45)
282 - 293	755.9005	1509.7864	1509.7827	2	0	R.DEWLRPEDLPIK.Y	(Ions score 45)
282 - 293	755.9005	1509.7864	1509.7827	2	0	R.DEWLRPEDLPIK.Y	(Ions score 61)
282 - 293	755.9005	1509.7864	1509.7827	2	0	R.DEWLRPEDLPIK.Y	(Ions score 53)
282 - 293	1510.7938	1509.7865	1509.7827	3	0	R.DEWLRPEDLPIK.Y	(Ions score 55)
282 - 293	755.9005	1509.7865	1509.7827	3	0	R.DEWLRPEDLPIK.Y	(Ions score 42)
282 - 293	755.9006	1509.7866	1509.7827	3	0	R.DEWLRPEDLPIK.Y	(Ions score 42)
282 - 293	755.9006	1509.7866	1509.7827	3	0	R.DEWLRPEDLPIK.Y	(Ions score 45)
282 - 293	755.9006	1509.7867	1509.7827	3	0	R.DEWLRPEDLPIK.Y	(Ions score 44)

282 - 293	755.9006	1509.7867	1509.7827	3	0	R.DEWLRPEDLPIK.Y	(Ions score 42)
282 - 293	755.9006	1509.7867	1509.7827	3	0	R.DEWLRPEDLPIK.Y	(Ions score 53)
282 - 293	755.9007	1509.7869	1509.7827	3	0	R.DEWLRPEDLPIK.Y	(Ions score 41)
282 - 293	755.9007	1509.7869	1509.7827	3	0	R.DEWLRPEDLPIK.Y	(Ions score 44)
282 - 293	755.9008	1509.7870	1509.7827	3	0	R.DEWLRPEDLPIK.Y	(Ions score 55)
282 - 293	755.9008	1509.7870	1509.7827	3	0	R.DEWLRPEDLPIK.Y	(Ions score 52)
282 - 293	755.9009	1509.7873	1509.7827	3	0	R.DEWLRPEDLPIK.Y	(Ions score 24)
282 - 293	755.9011	1509.7876	1509.7827	3	0	R.DEWLRPEDLPIK.Y	(Ions score 41)
282 - 293	755.9012	1509.7877	1509.7827	3	0	R.DEWLRPEDLPIK.Y	(Ions score 40)
282 - 293	755.9013	1509.7880	1509.7827	3	0	R.DEWLRPEDLPIK.Y	(Ions score 57)
282 - 293	755.9013	1509.7880	1509.7827	4	0	R.DEWLRPEDLPIK.Y	(Ions score 39)
282 - 293	755.9014	1509.7882	1509.7827	4	0	R.DEWLRPEDLPIK.Y	(Ions score 44)
282 - 293	504.2700	1509.7882	1509.7827	4	0	R.DEWLRPEDLPIK.Y	(Ions score 23)
282 - 293	755.9014	1509.7883	1509.7827	4	0	R.DEWLRPEDLPIK.Y	(Ions score 21)
282 - 293	755.9014	1509.7883	1509.7827	4	0	R.DEWLRPEDLPIK.Y	(Ions score 41)
282 - 293	755.9015	1509.7885	1509.7827	4	0	R.DEWLRPEDLPIK.Y	(Ions score 41)
282 - 293	755.9016	1509.7886	1509.7827	4	0	R.DEWLRPEDLPIK.Y	(Ions score 25)
282 - 293	755.9016	1509.7886	1509.7827	4	0	R.DEWLRPEDLPIK.Y	(Ions score 22)
282 - 293	755.9016	1509.7887	1509.7827	4	0	R.DEWLRPEDLPIK.Y	(Ions score 28)
282 - 293	755.9018	1509.7890	1509.7827	4	0	R.DEWLRPEDLPIK.Y	(Ions score 56)
282 - 293	755.9020	1509.7894	1509.7827	4	0	R.DEWLRPEDLPIK.Y	(Ions score 54)
282 - 293	755.9021	1509.7897	1509.7827	5	0	R.DEWLRPEDLPIK.Y	(Ions score 42)
282 - 293	755.9022	1509.7898	1509.7827	5	0	R.DEWLRPEDLPIK.Y	(Ions score 39)
282 - 293	755.9022	1509.7899	1509.7827	5	0	R.DEWLRPEDLPIK.Y	(Ions score 22)
282 - 293	504.2706	1509.7901	1509.7827	5	0	R.DEWLRPEDLPIK.Y	(Ions score 24)
282 - 302	856.0984	2565.2734	2565.2686	2	1	R.DEWLRPEDLPIKYAGLSTCFR.Q	(Ions score 33)
282 - 302	856.0994	2565.2763	2565.2686	3	1	R.DEWLRPEDLPIKYAGLSTCFR.Q	(Ions score 41)
282 - 302	856.0999	2565.2778	2565.2686	4	1	R.DEWLRPEDLPIKYAGLSTCFR.Q	(Ions score 43)
282 - 302	856.1001	2565.2784	2565.2686	4	1	R.DEWLRPEDLPIKYAGLSTCFR.Q	(Ions score 34)
282 - 302	870.1034	2607.2883	2607.2791	4	1	R.DEWLRPEDLPIKYAGLSTCFR.Q	Acetylation (K) (Ions score 36)
294 - 302	537.7529	1073.4912	1073.4964	-5	0	K.YAGLSTCFR.Q	(Ions score 40)
294 - 302	537.7531	1073.4916	1073.4964	-5	0	K.YAGLSTCFR.Q	(Ions score 40)
294 - 302	537.7535	1073.4925	1073.4964	-4	0	K.YAGLSTCFR.Q	(Ions score 42)
294 - 302	537.7538	1073.4930	1073.4964	-3	0	K.YAGLSTCFR.Q	(Ions score 38)
294 - 302	537.7541	1073.4937	1073.4964	-2	0	K.YAGLSTCFR.Q	(Ions score 38)
294 - 302	537.7543	1073.4940	1073.4964	-2	0	K.YAGLSTCFR.Q	(Ions score 36)
294 - 302	537.7543	1073.4941	1073.4964	-2	0	K.YAGLSTCFR.Q	(Ions score 34)
294 - 302	537.7544	1073.4943	1073.4964	-2	0	K.YAGLSTCFR.Q	(Ions score 24)
294 - 302	537.7545	1073.4944	1073.4964	-2	0	K.YAGLSTCFR.Q	(Ions score 25)
294 - 302	537.7546	1073.4947	1073.4964	-2	0	K.YAGLSTCFR.Q	(Ions score 35)
294 - 302	537.7546	1073.4947	1073.4964	-2	0	K.YAGLSTCFR.Q	(Ions score 37)
294 - 302	537.7546	1073.4947	1073.4964	-2	0	K.YAGLSTCFR.Q	(Ions score 23)
294 - 302	537.7547	1073.4948	1073.4964	-1	0	K.YAGLSTCFR.Q	(Ions score 35)
294 - 302	537.7548	1073.4950	1073.4964	-1	0	K.YAGLSTCFR.Q	(Ions score 35)
294 - 302	537.7548	1073.4951	1073.4964	-1	0	K.YAGLSTCFR.Q	(Ions score 32)
294 - 302	537.7549	1073.4952	1073.4964	-1	0	K.YAGLSTCFR.Q	(Ions score 25)
294 - 302	537.7549	1073.4953	1073.4964	-1	0	K.YAGLSTCFR.Q	(Ions score 37)
294 - 302	537.7549	1073.4953	1073.4964	-1	0	K.YAGLSTCFR.Q	(Ions score 24)
294 - 302	537.7549	1073.4953	1073.4964	-1	0	K.YAGLSTCFR.Q	(Ions score 30)
294 - 302	537.7550	1073.4954	1073.4964	-1	0	K.YAGLSTCFR.Q	(Ions score 28)
294 - 302	537.7550	1073.4955	1073.4964	-1	0	K.YAGLSTCFR.Q	(Ions score 23)
294 - 302	537.7551	1073.4956	1073.4964	-1	0	K.YAGLSTCFR.Q	(Ions score 29)
294 - 302	537.7551	1073.4956	1073.4964	-1	0	K.YAGLSTCFR.Q	(Ions score 36)
294 - 302	537.7551	1073.4957	1073.4964	-1	0	K.YAGLSTCFR.Q	(Ions score 28)
294 - 302	537.7551	1073.4957	1073.4964	-1	0	K.YAGLSTCFR.Q	(Ions score 40)
294 - 302	537.7551	1073.4957	1073.4964	-1	0	K.YAGLSTCFR.Q	(Ions score 32)
294 - 302	537.7552	1073.4958	1073.4964	-1	0	K.YAGLSTCFR.Q	(Ions score 28)

294 - 302	537.7552	1073.4959	1073.4964	-0	0	K.YAGLSTCFR.Q	(Ions score 28)
294 - 302	537.7552	1073.4959	1073.4964	-0	0	K.YAGLSTCFR.Q	(Ions score 24)
294 - 302	537.7552	1073.4959	1073.4964	-0	0	K.YAGLSTCFR.Q	(Ions score 36)
294 - 302	537.7552	1073.4959	1073.4964	-0	0	K.YAGLSTCFR.Q	(Ions score 21)
294 - 302	537.7553	1073.4960	1073.4964	-0	0	K.YAGLSTCFR.Q	(Ions score 33)
294 - 302	537.7553	1073.4960	1073.4964	-0	0	K.YAGLSTCFR.Q	(Ions score 34)
294 - 302	537.7554	1073.4962	1073.4964	-0	0	K.YAGLSTCFR.Q	(Ions score 29)
294 - 302	537.7554	1073.4963	1073.4964	-0	0	K.YAGLSTCFR.Q	(Ions score 28)
294 - 302	537.7555	1073.4964	1073.4964	-0	0	K.YAGLSTCFR.Q	(Ions score 36)
294 - 302	537.7555	1073.4964	1073.4964	0	0	K.YAGLSTCFR.Q	(Ions score 38)
294 - 302	537.7555	1073.4964	1073.4964	0	0	K.YAGLSTCFR.Q	(Ions score 35)
294 - 302	537.7556	1073.4966	1073.4964	0	0	K.YAGLSTCFR.Q	(Ions score 27)
294 - 302	537.7556	1073.4966	1073.4964	0	0	K.YAGLSTCFR.Q	(Ions score 24)
294 - 302	537.7556	1073.4966	1073.4964	0	0	K.YAGLSTCFR.Q	(Ions score 25)
294 - 302	537.7556	1073.4967	1073.4964	0	0	K.YAGLSTCFR.Q	(Ions score 33)
294 - 302	537.7556	1073.4967	1073.4964	0	0	K.YAGLSTCFR.Q	(Ions score 36)
294 - 302	537.7557	1073.4969	1073.4964	0	0	K.YAGLSTCFR.Q	(Ions score 31)
294 - 302	537.7558	1073.4970	1073.4964	1	0	K.YAGLSTCFR.Q	(Ions score 23)
294 - 302	537.7558	1073.4971	1073.4964	1	0	K.YAGLSTCFR.Q	(Ions score 40)
294 - 302	537.7558	1073.4971	1073.4964	1	0	K.YAGLSTCFR.Q	(Ions score 33)
294 - 302	537.7558	1073.4971	1073.4964	1	0	K.YAGLSTCFR.Q	(Ions score 24)
294 - 302	537.7559	1073.4972	1073.4964	1	0	K.YAGLSTCFR.Q	(Ions score 31)
294 - 302	537.7559	1073.4972	1073.4964	1	0	K.YAGLSTCFR.Q	(Ions score 40)
294 - 302	537.7559	1073.4973	1073.4964	1	0	K.YAGLSTCFR.Q	(Ions score 41)
294 - 302	537.7559	1073.4973	1073.4964	1	0	K.YAGLSTCFR.Q	(Ions score 26)
294 - 302	537.7560	1073.4974	1073.4964	1	0	K.YAGLSTCFR.Q	(Ions score 24)
294 - 302	537.7560	1073.4974	1073.4964	1	0	K.YAGLSTCFR.Q	(Ions score 27)
294 - 302	537.7560	1073.4975	1073.4964	1	0	K.YAGLSTCFR.Q	(Ions score 27)
294 - 302	537.7560	1073.4975	1073.4964	1	0	K.YAGLSTCFR.Q	(Ions score 27)
294 - 302	537.7560	1073.4975	1073.4964	1	0	K.YAGLSTCFR.Q	(Ions score 23)
294 - 302	537.7561	1073.4976	1073.4964	1	0	K.YAGLSTCFR.Q	(Ions score 31)
294 - 302	537.7561	1073.4976	1073.4964	1	0	K.YAGLSTCFR.Q	(Ions score 26)
294 - 302	537.7561	1073.4976	1073.4964	1	0	K.YAGLSTCFR.Q	(Ions score 30)
294 - 302	537.7561	1073.4976	1073.4964	1	0	K.YAGLSTCFR.Q	(Ions score 33)
294 - 302	537.7561	1073.4977	1073.4964	1	0	K.YAGLSTCFR.Q	(Ions score 42)
294 - 302	537.7561	1073.4977	1073.4964	1	0	K.YAGLSTCFR.Q	(Ions score 23)
294 - 302	537.7561	1073.4977	1073.4964	1	0	K.YAGLSTCFR.Q	(Ions score 30)
294 - 302	537.7561	1073.4977	1073.4964	1	0	K.YAGLSTCFR.Q	(Ions score 36)
294 - 302	537.7562	1073.4978	1073.4964	1	0	K.YAGLSTCFR.Q	(Ions score 26)
294 - 302	537.7562	1073.4978	1073.4964	1	0	K.YAGLSTCFR.Q	(Ions score 26)
294 - 302	537.7562	1073.4978	1073.4964	1	0	K.YAGLSTCFR.Q	(Ions score 27)
294 - 302	537.7562	1073.4979	1073.4964	1	0	K.YAGLSTCFR.Q	(Ions score 24)
294 - 302	537.7562	1073.4979	1073.4964	1	0	K.YAGLSTCFR.Q	(Ions score 24)
294 - 302	537.7562	1073.4979	1073.4964	1	0	K.YAGLSTCFR.Q	(Ions score 26)
294 - 302	537.7563	1073.4980	1073.4964	1	0	K.YAGLSTCFR.Q	(Ions score 46)
294 - 302	537.7563	1073.4980	1073.4964	1	0	K.YAGLSTCFR.Q	(Ions score 50)
294 - 302	537.7563	1073.4980	1073.4964	1	0	K.YAGLSTCFR.Q	(Ions score 41)
294 - 302	537.7563	1073.4980	1073.4964	2	0	K.YAGLSTCFR.Q	(Ions score 28)
294 - 302	537.7563	1073.4980	1073.4964	2	0	K.YAGLSTCFR.Q	(Ions score 36)
294 - 302	537.7563	1073.4981	1073.4964	2	0	K.YAGLSTCFR.Q	(Ions score 31)
294 - 302	537.7563	1073.4981	1073.4964	2	0	K.YAGLSTCFR.Q	(Ions score 27)
294 - 302	537.7563	1073.4981	1073.4964	2	0	K.YAGLSTCFR.Q	(Ions score 38)
294 - 302	537.7563	1073.4981	1073.4964	2	0	K.YAGLSTCFR.Q	(Ions score 34)
294 - 302	537.7563	1073.4981	1073.4964	2	0	K.YAGLSTCFR.Q	(Ions score 24)
294 - 302	537.7563	1073.4981	1073.4964	2	0	K.YAGLSTCFR.Q	(Ions score 27)
294 - 302	537.7563	1073.4981	1073.4964	2	0	K.YAGLSTCFR.Q	(Ions score 24)
294 - 302	537.7563	1073.4981	1073.4964	2	0	K.YAGLSTCFR.Q	(Ions score 28)

294 - 302	537.7563	1073.4981	1073.4964	2	0	K.YAGLSTCFR.Q	(Ions score 24)
294 - 302	537.7563	1073.4981	1073.4964	2	0	K.YAGLSTCFR.Q	(Ions score 26)
294 - 302	537.7563	1073.4981	1073.4964	2	0	K.YAGLSTCFR.Q	(Ions score 31)
294 - 302	537.7564	1073.4982	1073.4964	2	0	K.YAGLSTCFR.Q	(Ions score 25)
294 - 302	537.7564	1073.4982	1073.4964	2	0	K.YAGLSTCFR.Q	(Ions score 40)
294 - 302	537.7564	1073.4983	1073.4964	2	0	K.YAGLSTCFR.Q	(Ions score 21)
294 - 302	537.7564	1073.4983	1073.4964	2	0	K.YAGLSTCFR.Q	(Ions score 47)
294 - 302	537.7564	1073.4983	1073.4964	2	0	K.YAGLSTCFR.Q	(Ions score 35)
294 - 302	537.7565	1073.4984	1073.4964	2	0	K.YAGLSTCFR.Q	(Ions score 35)
294 - 302	537.7565	1073.4984	1073.4964	2	0	K.YAGLSTCFR.Q	(Ions score 29)
294 - 302	537.7565	1073.4984	1073.4964	2	0	K.YAGLSTCFR.Q	(Ions score 28)
294 - 302	537.7565	1073.4984	1073.4964	2	0	K.YAGLSTCFR.Q	(Ions score 23)
294 - 302	537.7565	1073.4984	1073.4964	2	0	K.YAGLSTCFR.Q	(Ions score 27)
294 - 302	537.7565	1073.4985	1073.4964	2	0	K.YAGLSTCFR.Q	(Ions score 33)
294 - 302	537.7565	1073.4985	1073.4964	2	0	K.YAGLSTCFR.Q	(Ions score 28)
294 - 302	537.7565	1073.4985	1073.4964	2	0	K.YAGLSTCFR.Q	(Ions score 35)
294 - 302	537.7565	1073.4985	1073.4964	2	0	K.YAGLSTCFR.Q	(Ions score 34)
294 - 302	537.7565	1073.4985	1073.4964	2	0	K.YAGLSTCFR.Q	(Ions score 30)
294 - 302	537.7565	1073.4985	1073.4964	2	0	K.YAGLSTCFR.Q	(Ions score 34)
294 - 302	537.7565	1073.4985	1073.4964	2	0	K.YAGLSTCFR.Q	(Ions score 36)
294 - 302	537.7566	1073.4986	1073.4964	2	0	K.YAGLSTCFR.Q	(Ions score 24)
294 - 302	537.7566	1073.4986	1073.4964	2	0	K.YAGLSTCFR.Q	(Ions score 29)
294 - 302	537.7566	1073.4986	1073.4964	2	0	K.YAGLSTCFR.Q	(Ions score 36)
294 - 302	537.7566	1073.4986	1073.4964	2	0	K.YAGLSTCFR.Q	(Ions score 29)
294 - 302	537.7566	1073.4986	1073.4964	2	0	K.YAGLSTCFR.Q	(Ions score 25)
294 - 302	537.7566	1073.4986	1073.4964	2	0	K.YAGLSTCFR.Q	(Ions score 29)
294 - 302	537.7566	1073.4986	1073.4964	2	0	K.YAGLSTCFR.Q	(Ions score 36)
294 - 302	537.7566	1073.4986	1073.4964	2	0	K.YAGLSTCFR.Q	(Ions score 33)
294 - 302	537.7566	1073.4987	1073.4964	2	0	K.YAGLSTCFR.Q	(Ions score 27)
294 - 302	537.7566	1073.4987	1073.4964	2	0	K.YAGLSTCFR.Q	(Ions score 35)
294 - 302	537.7566	1073.4987	1073.4964	2	0	K.YAGLSTCFR.Q	(Ions score 33)
294 - 302	537.7566	1073.4987	1073.4964	2	0	K.YAGLSTCFR.Q	(Ions score 24)
294 - 302	537.7566	1073.4987	1073.4964	2	0	K.YAGLSTCFR.Q	(Ions score 36)
294 - 302	537.7567	1073.4987	1073.4964	2	0	K.YAGLSTCFR.Q	(Ions score 36)
294 - 302	537.7567	1073.4988	1073.4964	2	0	K.YAGLSTCFR.Q	(Ions score 29)
294 - 302	537.7567	1073.4988	1073.4964	2	0	K.YAGLSTCFR.Q	(Ions score 22)
294 - 302	537.7567	1073.4988	1073.4964	2	0	K.YAGLSTCFR.Q	(Ions score 53)
294 - 302	537.7567	1073.4988	1073.4964	2	0	K.YAGLSTCFR.Q	(Ions score 39)
294 - 302	537.7567	1073.4988	1073.4964	2	0	K.YAGLSTCFR.Q	(Ions score 36)
294 - 302	537.7567	1073.4988	1073.4964	2	0	K.YAGLSTCFR.Q	(Ions score 36)
294 - 302	537.7567	1073.4989	1073.4964	2	0	K.YAGLSTCFR.Q	(Ions score 36)
294 - 302	537.7567	1073.4989	1073.4964	2	0	K.YAGLSTCFR.Q	(Ions score 24)
294 - 302	537.7567	1073.4989	1073.4964	2	0	K.YAGLSTCFR.Q	(Ions score 34)
294 - 302	537.7567	1073.4989	1073.4964	2	0	K.YAGLSTCFR.Q	(Ions score 25)
294 - 302	537.7567	1073.4989	1073.4964	2	0	K.YAGLSTCFR.Q	(Ions score 42)
294 - 302	537.7567	1073.4989	1073.4964	2	0	K.YAGLSTCFR.Q	(Ions score 36)
294 - 302	537.7567	1073.4989	1073.4964	2	0	K.YAGLSTCFR.Q	(Ions score 39)
294 - 302	537.7567	1073.4989	1073.4964	2	0	K.YAGLSTCFR.Q	(Ions score 30)
294 - 302	537.7567	1073.4989	1073.4964	2	0	K.YAGLSTCFR.Q	(Ions score 36)
294 - 302	537.7568	1073.4989	1073.4964	2	0	K.YAGLSTCFR.Q	(Ions score 25)
294 - 302	537.7568	1073.4990	1073.4964	2	0	K.YAGLSTCFR.Q	(Ions score 35)
294 - 302	537.7568	1073.4990	1073.4964	2	0	K.YAGLSTCFR.Q	(Ions score 27)
294 - 302	537.7568	1073.4990	1073.4964	2	0	K.YAGLSTCFR.Q	(Ions score 28)
294 - 302	537.7568	1073.4990	1073.4964	2	0	K.YAGLSTCFR.Q	(Ions score 49)
294 - 302	537.7568	1073.4990	1073.4964	2	0	K.YAGLSTCFR.Q	(Ions score 28)
294 - 302	537.7568	1073.4991	1073.4964	2	0	K.YAGLSTCFR.Q	(Ions score 31)
294 - 302	537.7568	1073.4991	1073.4964	2	0	K.YAGLSTCFR.Q	(Ions score 36)

294 - 302	537.7568	1073.4991	1073.4964	2	0	K.YAGLSTCFR.Q	(Ions score 29)
294 - 302	537.7568	1073.4991	1073.4964	2	0	K.YAGLSTCFR.Q	(Ions score 36)
294 - 302	537.7568	1073.4991	1073.4964	2	0	K.YAGLSTCFR.Q	(Ions score 29)
294 - 302	537.7568	1073.4991	1073.4964	2	0	K.YAGLSTCFR.Q	(Ions score 37)
294 - 302	537.7568	1073.4991	1073.4964	3	0	K.YAGLSTCFR.Q	(Ions score 34)
294 - 302	537.7568	1073.4991	1073.4964	3	0	K.YAGLSTCFR.Q	(Ions score 36)
294 - 302	537.7568	1073.4991	1073.4964	3	0	K.YAGLSTCFR.Q	(Ions score 27)
294 - 302	537.7568	1073.4991	1073.4964	3	0	K.YAGLSTCFR.Q	(Ions score 25)
294 - 302	537.7569	1073.4992	1073.4964	3	0	K.YAGLSTCFR.Q	(Ions score 38)
294 - 302	537.7569	1073.4992	1073.4964	3	0	K.YAGLSTCFR.Q	(Ions score 24)
294 - 302	537.7569	1073.4992	1073.4964	3	0	K.YAGLSTCFR.Q	(Ions score 35)
294 - 302	537.7569	1073.4992	1073.4964	3	0	K.YAGLSTCFR.Q	(Ions score 36)
294 - 302	537.7569	1073.4992	1073.4964	3	0	K.YAGLSTCFR.Q	(Ions score 25)
294 - 302	537.7569	1073.4992	1073.4964	3	0	K.YAGLSTCFR.Q	(Ions score 28)
294 - 302	537.7569	1073.4992	1073.4964	3	0	K.YAGLSTCFR.Q	(Ions score 36)
294 - 302	537.7569	1073.4993	1073.4964	3	0	K.YAGLSTCFR.Q	(Ions score 36)
294 - 302	537.7569	1073.4993	1073.4964	3	0	K.YAGLSTCFR.Q	(Ions score 27)
294 - 302	537.7569	1073.4993	1073.4964	3	0	K.YAGLSTCFR.Q	(Ions score 29)
294 - 302	537.7569	1073.4993	1073.4964	3	0	K.YAGLSTCFR.Q	(Ions score 30)
294 - 302	537.7569	1073.4993	1073.4964	3	0	K.YAGLSTCFR.Q	(Ions score 38)
294 - 302	537.7569	1073.4993	1073.4964	3	0	K.YAGLSTCFR.Q	(Ions score 34)
294 - 302	537.7569	1073.4993	1073.4964	3	0	K.YAGLSTCFR.Q	(Ions score 36)
294 - 302	537.7569	1073.4993	1073.4964	3	0	K.YAGLSTCFR.Q	(Ions score 36)
294 - 302	537.7569	1073.4993	1073.4964	3	0	K.YAGLSTCFR.Q	(Ions score 40)
294 - 302	537.7569	1073.4993	1073.4964	3	0	K.YAGLSTCFR.Q	(Ions score 27)
294 - 302	537.7569	1073.4993	1073.4964	3	0	K.YAGLSTCFR.Q	(Ions score 30)
294 - 302	537.7569	1073.4993	1073.4964	3	0	K.YAGLSTCFR.Q	(Ions score 26)
294 - 302	537.7570	1073.4994	1073.4964	3	0	K.YAGLSTCFR.Q	(Ions score 28)
294 - 302	537.7570	1073.4994	1073.4964	3	0	K.YAGLSTCFR.Q	(Ions score 23)
294 - 302	537.7570	1073.4994	1073.4964	3	0	K.YAGLSTCFR.Q	(Ions score 24)
294 - 302	537.7570	1073.4994	1073.4964	3	0	K.YAGLSTCFR.Q	(Ions score 24)
294 - 302	537.7570	1073.4994	1073.4964	3	0	K.YAGLSTCFR.Q	(Ions score 30)
294 - 302	537.7570	1073.4994	1073.4964	3	0	K.YAGLSTCFR.Q	(Ions score 36)
294 - 302	537.7570	1073.4994	1073.4964	3	0	K.YAGLSTCFR.Q	(Ions score 33)
294 - 302	537.7570	1073.4994	1073.4964	3	0	K.YAGLSTCFR.Q	(Ions score 30)
294 - 302	537.7570	1073.4994	1073.4964	3	0	K.YAGLSTCFR.Q	(Ions score 26)
294 - 302	537.7570	1073.4994	1073.4964	3	0	K.YAGLSTCFR.Q	(Ions score 28)
294 - 302	537.7570	1073.4994	1073.4964	3	0	K.YAGLSTCFR.Q	(Ions score 25)
294 - 302	537.7570	1073.4995	1073.4964	3	0	K.YAGLSTCFR.Q	(Ions score 35)
294 - 302	537.7570	1073.4995	1073.4964	3	0	K.YAGLSTCFR.Q	(Ions score 36)
294 - 302	537.7570	1073.4995	1073.4964	3	0	K.YAGLSTCFR.Q	(Ions score 36)
294 - 302	537.7570	1073.4995	1073.4964	3	0	K.YAGLSTCFR.Q	(Ions score 27)
294 - 302	537.7570	1073.4995	1073.4964	3	0	K.YAGLSTCFR.Q	(Ions score 29)
294 - 302	537.7570	1073.4995	1073.4964	3	0	K.YAGLSTCFR.Q	(Ions score 24)
294 - 302	537.7570	1073.4995	1073.4964	3	0	K.YAGLSTCFR.Q	(Ions score 41)
294 - 302	537.7570	1073.4995	1073.4964	3	0	K.YAGLSTCFR.Q	(Ions score 25)
294 - 302	537.7570	1073.4995	1073.4964	3	0	K.YAGLSTCFR.Q	(Ions score 34)
294 - 302	537.7571	1073.4996	1073.4964	3	0	K.YAGLSTCFR.Q	(Ions score 36)
294 - 302	537.7571	1073.4996	1073.4964	3	0	K.YAGLSTCFR.Q	(Ions score 32)
294 - 302	537.7571	1073.4996	1073.4964	3	0	K.YAGLSTCFR.Q	(Ions score 38)
294 - 302	537.7571	1073.4996	1073.4964	3	0	K.YAGLSTCFR.Q	(Ions score 43)
294 - 302	537.7571	1073.4996	1073.4964	3	0	K.YAGLSTCFR.Q	(Ions score 21)
294 - 302	537.7571	1073.4996	1073.4964	3	0	K.YAGLSTCFR.Q	(Ions score 22)
294 - 302	537.7571	1073.4996	1073.4964	3	0	K.YAGLSTCFR.Q	(Ions score 29)
294 - 302	537.7571	1073.4996	1073.4964	3	0	K.YAGLSTCFR.Q	(Ions score 37)
294 - 302	537.7571	1073.4997	1073.4964	3	0	K.YAGLSTCFR.Q	(Ions score 36)
294 - 302	537.7571	1073.4997	1073.4964	3	0	K.YAGLSTCFR.Q	(Ions score 46)

294 - 302	537.7571	1073.4997	1073.4964	3	0	K.YAGLSTCFR.Q	(Ions score 35)
294 - 302	537.7571	1073.4997	1073.4964	3	0	K.YAGLSTCFR.Q	(Ions score 35)
294 - 302	537.7571	1073.4997	1073.4964	3	0	K.YAGLSTCFR.Q	(Ions score 31)
294 - 302	537.7571	1073.4997	1073.4964	3	0	K.YAGLSTCFR.Q	(Ions score 25)
294 - 302	537.7571	1073.4997	1073.4964	3	0	K.YAGLSTCFR.Q	(Ions score 32)
294 - 302	537.7571	1073.4997	1073.4964	3	0	K.YAGLSTCFR.Q	(Ions score 29)
294 - 302	537.7571	1073.4997	1073.4964	3	0	K.YAGLSTCFR.Q	(Ions score 29)
294 - 302	537.7572	1073.4998	1073.4964	3	0	K.YAGLSTCFR.Q	(Ions score 29)
294 - 302	537.7572	1073.4998	1073.4964	3	0	K.YAGLSTCFR.Q	(Ions score 26)
294 - 302	537.7572	1073.4998	1073.4964	3	0	K.YAGLSTCFR.Q	(Ions score 36)
294 - 302	537.7572	1073.4998	1073.4964	3	0	K.YAGLSTCFR.Q	(Ions score 26)
294 - 302	537.7572	1073.4998	1073.4964	3	0	K.YAGLSTCFR.Q	(Ions score 23)
294 - 302	537.7572	1073.4998	1073.4964	3	0	K.YAGLSTCFR.Q	(Ions score 28)
294 - 302	537.7572	1073.4998	1073.4964	3	0	K.YAGLSTCFR.Q	(Ions score 58)
294 - 302	537.7572	1073.4998	1073.4964	3	0	K.YAGLSTCFR.Q	(Ions score 56)
294 - 302	537.7572	1073.4999	1073.4964	3	0	K.YAGLSTCFR.Q	(Ions score 35)
294 - 302	537.7572	1073.4999	1073.4964	3	0	K.YAGLSTCFR.Q	(Ions score 58)
294 - 302	537.7572	1073.4999	1073.4964	3	0	K.YAGLSTCFR.Q	(Ions score 34)
294 - 302	537.7572	1073.4999	1073.4964	3	0	K.YAGLSTCFR.Q	(Ions score 27)
294 - 302	537.7572	1073.4999	1073.4964	3	0	K.YAGLSTCFR.Q	(Ions score 51)
294 - 302	537.7573	1073.5000	1073.4964	3	0	K.YAGLSTCFR.Q	(Ions score 56)
294 - 302	537.7573	1073.5000	1073.4964	3	0	K.YAGLSTCFR.Q	(Ions score 56)
294 - 302	537.7573	1073.5000	1073.4964	3	0	K.YAGLSTCFR.Q	(Ions score 37)
294 - 302	537.7573	1073.5000	1073.4964	3	0	K.YAGLSTCFR.Q	(Ions score 38)
294 - 302	537.7573	1073.5000	1073.4964	3	0	K.YAGLSTCFR.Q	(Ions score 52)
294 - 302	537.7573	1073.5000	1073.4964	3	0	K.YAGLSTCFR.Q	(Ions score 34)
294 - 302	537.7573	1073.5000	1073.4964	3	0	K.YAGLSTCFR.Q	(Ions score 28)
294 - 302	537.7573	1073.5000	1073.4964	3	0	K.YAGLSTCFR.Q	(Ions score 36)
294 - 302	537.7573	1073.5000	1073.4964	3	0	K.YAGLSTCFR.Q	(Ions score 26)
294 - 302	537.7573	1073.5001	1073.4964	3	0	K.YAGLSTCFR.Q	(Ions score 36)
294 - 302	537.7573	1073.5001	1073.4964	3	0	K.YAGLSTCFR.Q	(Ions score 36)
294 - 302	537.7573	1073.5001	1073.4964	3	0	K.YAGLSTCFR.Q	(Ions score 34)
294 - 302	537.7573	1073.5001	1073.4964	3	0	K.YAGLSTCFR.Q	(Ions score 28)
294 - 302	537.7573	1073.5001	1073.4964	3	0	K.YAGLSTCFR.Q	(Ions score 25)
294 - 302	537.7573	1073.5001	1073.4964	3	0	K.YAGLSTCFR.Q	(Ions score 60)
294 - 302	537.7573	1073.5001	1073.4964	3	0	K.YAGLSTCFR.Q	(Ions score 35)
294 - 302	537.7573	1073.5001	1073.4964	3	0	K.YAGLSTCFR.Q	(Ions score 27)
294 - 302	537.7574	1073.5001	1073.4964	3	0	K.YAGLSTCFR.Q	(Ions score 36)
294 - 302	537.7574	1073.5002	1073.4964	3	0	K.YAGLSTCFR.Q	(Ions score 28)
294 - 302	537.7574	1073.5002	1073.4964	4	0	K.YAGLSTCFR.Q	(Ions score 51)
294 - 302	537.7574	1073.5002	1073.4964	4	0	K.YAGLSTCFR.Q	(Ions score 58)
294 - 302	537.7574	1073.5002	1073.4964	4	0	K.YAGLSTCFR.Q	(Ions score 24)
294 - 302	537.7574	1073.5002	1073.4964	4	0	K.YAGLSTCFR.Q	(Ions score 35)
294 - 302	537.7574	1073.5002	1073.4964	4	0	K.YAGLSTCFR.Q	(Ions score 53)
294 - 302	537.7574	1073.5002	1073.4964	4	0	K.YAGLSTCFR.Q	(Ions score 25)
294 - 302	537.7574	1073.5002	1073.4964	4	0	K.YAGLSTCFR.Q	(Ions score 56)
294 - 302	537.7574	1073.5003	1073.4964	4	0	K.YAGLSTCFR.Q	(Ions score 37)
294 - 302	537.7574	1073.5003	1073.4964	4	0	K.YAGLSTCFR.Q	(Ions score 26)
294 - 302	537.7574	1073.5003	1073.4964	4	0	K.YAGLSTCFR.Q	(Ions score 25)
294 - 302	537.7574	1073.5003	1073.4964	4	0	K.YAGLSTCFR.Q	(Ions score 41)
294 - 302	537.7574	1073.5003	1073.4964	4	0	K.YAGLSTCFR.Q	(Ions score 48)
294 - 302	537.7574	1073.5003	1073.4964	4	0	K.YAGLSTCFR.Q	(Ions score 35)
294 - 302	537.7574	1073.5003	1073.4964	4	0	K.YAGLSTCFR.Q	(Ions score 25)
294 - 302	537.7575	1073.5004	1073.4964	4	0	K.YAGLSTCFR.Q	(Ions score 24)
294 - 302	537.7575	1073.5004	1073.4964	4	0	K.YAGLSTCFR.Q	(Ions score 24)
294 - 302	537.7575	1073.5004	1073.4964	4	0	K.YAGLSTCFR.Q	(Ions score 60)
294 - 302	537.7575	1073.5004	1073.4964	4	0	K.YAGLSTCFR.Q	(Ions score 36)

294 - 302	537.7575	1073.5004	1073.4964	4	0	K.YAGLSTCFR.Q	(Ions score 36)
294 - 302	537.7575	1073.5004	1073.4964	4	0	K.YAGLSTCFR.Q	(Ions score 29)
294 - 302	537.7575	1073.5004	1073.4964	4	0	K.YAGLSTCFR.Q	(Ions score 24)
294 - 302	537.7575	1073.5004	1073.4964	4	0	K.YAGLSTCFR.Q	(Ions score 53)
294 - 302	537.7575	1073.5005	1073.4964	4	0	K.YAGLSTCFR.Q	(Ions score 26)
294 - 302	537.7575	1073.5005	1073.4964	4	0	K.YAGLSTCFR.Q	(Ions score 47)
294 - 302	537.7575	1073.5005	1073.4964	4	0	K.YAGLSTCFR.Q	(Ions score 21)
294 - 302	537.7575	1073.5005	1073.4964	4	0	K.YAGLSTCFR.Q	(Ions score 27)
294 - 302	537.7575	1073.5005	1073.4964	4	0	K.YAGLSTCFR.Q	(Ions score 26)
294 - 302	537.7576	1073.5005	1073.4964	4	0	K.YAGLSTCFR.Q	(Ions score 57)
294 - 302	537.7576	1073.5006	1073.4964	4	0	K.YAGLSTCFR.Q	(Ions score 28)
294 - 302	537.7576	1073.5006	1073.4964	4	0	K.YAGLSTCFR.Q	(Ions score 53)
294 - 302	537.7576	1073.5006	1073.4964	4	0	K.YAGLSTCFR.Q	(Ions score 32)
294 - 302	537.7576	1073.5007	1073.4964	4	0	K.YAGLSTCFR.Q	(Ions score 36)
294 - 302	537.7576	1073.5007	1073.4964	4	0	K.YAGLSTCFR.Q	(Ions score 36)
294 - 302	537.7576	1073.5007	1073.4964	4	0	K.YAGLSTCFR.Q	(Ions score 51)
294 - 302	537.7577	1073.5007	1073.4964	4	0	K.YAGLSTCFR.Q	(Ions score 28)
294 - 302	537.7577	1073.5008	1073.4964	4	0	K.YAGLSTCFR.Q	(Ions score 35)
294 - 302	537.7577	1073.5008	1073.4964	4	0	K.YAGLSTCFR.Q	(Ions score 36)
294 - 302	537.7577	1073.5008	1073.4964	4	0	K.YAGLSTCFR.Q	(Ions score 22)
294 - 302	537.7577	1073.5009	1073.4964	4	0	K.YAGLSTCFR.Q	(Ions score 23)
294 - 302	537.7577	1073.5009	1073.4964	4	0	K.YAGLSTCFR.Q	(Ions score 31)
294 - 302	537.7577	1073.5009	1073.4964	4	0	K.YAGLSTCFR.Q	(Ions score 26)
294 - 302	537.7578	1073.5009	1073.4964	4	0	K.YAGLSTCFR.Q	(Ions score 35)
294 - 302	537.7578	1073.5010	1073.4964	4	0	K.YAGLSTCFR.Q	(Ions score 37)
294 - 302	537.7578	1073.5010	1073.4964	4	0	K.YAGLSTCFR.Q	(Ions score 22)
294 - 302	537.7578	1073.5010	1073.4964	4	0	K.YAGLSTCFR.Q	(Ions score 50)
294 - 302	537.7578	1073.5010	1073.4964	4	0	K.YAGLSTCFR.Q	(Ions score 52)
294 - 302	537.7578	1073.5011	1073.4964	4	0	K.YAGLSTCFR.Q	(Ions score 28)
294 - 302	537.7578	1073.5011	1073.4964	4	0	K.YAGLSTCFR.Q	(Ions score 47)
294 - 302	537.7578	1073.5011	1073.4964	4	0	K.YAGLSTCFR.Q	(Ions score 37)
294 - 302	537.7579	1073.5012	1073.4964	4	0	K.YAGLSTCFR.Q	(Ions score 36)
294 - 302	537.7579	1073.5012	1073.4964	4	0	K.YAGLSTCFR.Q	(Ions score 25)
294 - 302	537.7579	1073.5012	1073.4964	4	0	K.YAGLSTCFR.Q	(Ions score 47)
294 - 302	537.7579	1073.5013	1073.4964	5	0	K.YAGLSTCFR.Q	(Ions score 51)
294 - 302	537.7579	1073.5013	1073.4964	5	0	K.YAGLSTCFR.Q	(Ions score 43)
294 - 302	537.7579	1073.5013	1073.4964	5	0	K.YAGLSTCFR.Q	(Ions score 25)
294 - 302	537.7579	1073.5013	1073.4964	5	0	K.YAGLSTCFR.Q	(Ions score 29)
294 - 302	537.7579	1073.5013	1073.4964	5	0	K.YAGLSTCFR.Q	(Ions score 36)
294 - 302	537.7579	1073.5013	1073.4964	5	0	K.YAGLSTCFR.Q	(Ions score 36)
294 - 302	537.7580	1073.5014	1073.4964	5	0	K.YAGLSTCFR.Q	(Ions score 61)
294 - 302	537.7580	1073.5014	1073.4964	5	0	K.YAGLSTCFR.Q	(Ions score 36)
294 - 302	537.7580	1073.5014	1073.4964	5	0	K.YAGLSTCFR.Q	(Ions score 29)
294 - 302	537.7582	1073.5018	1073.4964	5	0	K.YAGLSTCFR.Q	(Ions score 36)
303 - 310	435.2137	868.4128	868.4151	-3	0	R.QEVGSHGR.D	(Ions score 30)
303 - 310	435.2144	868.4142	868.4151	-1	0	R.QEVGSHGR.D	(Ions score 44)
303 - 310	435.2145	868.4144	868.4151	-1	0	R.QEVGSHGR.D	(Ions score 42)
303 - 310	435.2147	868.4148	868.4151	-0	0	R.QEVGSHGR.D	(Ions score 36)
303 - 310	435.2148	868.4150	868.4151	-0	0	R.QEVGSHGR.D	(Ions score 30)
303 - 310	435.2148	868.4151	868.4151	-0	0	R.QEVGSHGR.D	(Ions score 47)
303 - 310	435.2151	868.4156	868.4151	1	0	R.QEVGSHGR.D	(Ions score 31)
303 - 310	435.2152	868.4158	868.4151	1	0	R.QEVGSHGR.D	(Ions score 45)
303 - 310	435.2152	868.4159	868.4151	1	0	R.QEVGSHGR.D	(Ions score 39)
303 - 310	435.2152	868.4159	868.4151	1	0	R.QEVGSHGR.D	(Ions score 42)
303 - 310	435.2152	868.4159	868.4151	1	0	R.QEVGSHGR.D	(Ions score 43)
303 - 310	435.2154	868.4162	868.4151	1	0	R.QEVGSHGR.D	(Ions score 31)
303 - 310	435.2154	868.4163	868.4151	1	0	R.QEVGSHGR.D	(Ions score 43)

303 - 310	435.2154	868.4163	868.4151	1	0	R.QEVGSHGR.D	(Ions score 41)		
303 - 310	435.2154	868.4163	868.4151	1	0	R.QEVGSHGR.D	(Ions score 46)		
303 - 310	435.2154	868.4163	868.4151	1	0	R.QEVGSHGR.D	(Ions score 40)		
303 - 310	435.2154	868.4163	868.4151	1	0	R.QEVGSHGR.D	(Ions score 39)		
303 - 310	435.2155	868.4164	868.4151	1	0	R.QEVGSHGR.D	(Ions score 37)		
303 - 310	435.2155	868.4164	868.4151	1	0	R.QEVGSHGR.D	(Ions score 44)		
303 - 310	435.2155	868.4164	868.4151	1	0	R.QEVGSHGR.D	(Ions score 34)		
303 - 310	435.2155	868.4164	868.4151	1	0	R.QEVGSHGR.D	(Ions score 40)		
303 - 310	435.2155	868.4165	868.4151	2	0	R.QEVGSHGR.D	(Ions score 40)		
303 - 310	435.2155	868.4165	868.4151	2	0	R.QEVGSHGR.D	(Ions score 42)		
303 - 310	435.2155	868.4165	868.4151	2	0	R.QEVGSHGR.D	(Ions score 47)		
303 - 310	435.2155	868.4165	868.4151	2	0	R.QEVGSHGR.D	(Ions score 47)		
303 - 310	435.2156	868.4166	868.4151	2	0	R.QEVGSHGR.D	(Ions score 28)		
303 - 310	435.2156	868.4167	868.4151	2	0	R.QEVGSHGR.D	(Ions score 34)		
303 - 310	435.2156	868.4167	868.4151	2	0	R.QEVGSHGR.D	(Ions score 31)		
303 - 310	435.2157	868.4168	868.4151	2	0	R.QEVGSHGR.D	(Ions score 46)		
303 - 310	435.2157	868.4168	868.4151	2	0	R.QEVGSHGR.D	(Ions score 24)		
303 - 310	435.2159	868.4172	868.4151	2	0	R.QEVGSHGR.D	(Ions score 34)		
303 - 310	435.2161	868.4176	868.4151	3	0	R.QEVGSHGR.D	(Ions score 26)		
303 - 310	435.2162	868.4178	868.4151	3	0	R.QEVGSHGR.D	(Ions score 40)		
303 - 310	435.2164	868.4183	868.4151	4	0	R.QEVGSHGR.D	(Ions score 36)		
303 - 310	435.2168	868.4191	868.4151	5	0	R.QEVGSHGR.D	(Ions score 22)		
303 - 310	435.2169	868.4193	868.4151	5	0	R.QEVGSHGR.D	(Ions score 30)		
318 - 336	583.7903	2331.1321	2331.1284	2	1	R.VHQFEKIEQFVYSSPHDNK.S	(Ions score 40)		
318 - 336	583.7903	2331.1321	2331.1284	2	1	R.VHQFEKIEQFVYSSPHDNK.S	(Ions score 47)		
318 - 336	778.0516	2331.1331	2331.1284	2	1	R.VHQFEKIEQFVYSSPHDNK.S	(Ions score 50)		
318 - 336	778.0517	2331.1332	2331.1284	2	1	R.VHQFEKIEQFVYSSPHDNK.S	(Ions score 54)		
318 - 336	467.2344	2331.1355	2331.1284	3	1	R.VHQFEKIEQFVYSSPHDNK.S	(Ions score 50)		
318 - 336	583.7913	2331.1362	2331.1284	3	1	R.VHQFEKIEQFVYSSPHDNK.S	(Ions score 28)		
318 - 336	467.2350	2331.1387	2331.1284	4	1	R.VHQFEKIEQFVYSSPHDNK.S	(Ions score 35)		
318 - 336	594.2931	2373.1433	2373.1389	2	1	R.VHQFEKIEQFVYSSPHDNK.S	Acetylation (K)	(Ions score 57)	
318 - 336	792.0555	2373.1446	2373.1389	2	1	R.VHQFEKIEQFVYSSPHDNK.S	Acetylation (K)	(Ions score 72)	
318 - 336	792.0555	2373.1446	2373.1389	2	1	R.VHQFEKIEQFVYSSPHDNK.S	Acetylation (K)	(Ions score 79)	
318 - 336	594.2936	2373.1453	2373.1389	3	1	R.VHQFEKIEQFVYSSPHDNK.S	Acetylation (K)	(Ions score 56)	
318 - 336	594.2937	2373.1456	2373.1389	3	1	R.VHQFEKIEQFVYSSPHDNK.S	Acetylation (K)	(Ions score 69)	
318 - 336	792.0574	2373.1503	2373.1389	5	1	R.VHQFEKIEQFVYSSPHDNK.S	Acetylation (K)	(Ions score 22)	
324 - 336	782.3738	1562.7330	1562.7365	-2	0	K.IEQFVYSSPHDNK.S	(Ions score 40)		
324 - 336	782.3738	1562.7331	1562.7365	-2	0	K.IEQFVYSSPHDNK.S	(Ions score 41)		
324 - 336	782.3745	1562.7345	1562.7365	-1	0	K.IEQFVYSSPHDNK.S	(Ions score 56)		
324 - 336	782.3747	1562.7348	1562.7365	-1	0	K.IEQFVYSSPHDNK.S	(Ions score 46)		
324 - 336	782.3748	1562.7350	1562.7365	-1	0	K.IEQFVYSSPHDNK.S	(Ions score 46)		
324 - 336	521.9190	1562.7351	1562.7365	-1	0	K.IEQFVYSSPHDNK.S	(Ions score 37)		
324 - 336	782.3752	1562.7359	1562.7365	-0	0	K.IEQFVYSSPHDNK.S	(Ions score 50)		
324 - 336	782.3753	1562.7361	1562.7365	-0	0	K.IEQFVYSSPHDNK.S	(Ions score 54)		
324 - 336	782.3753	1562.7361	1562.7365	-0	0	K.IEQFVYSSPHDNK.S	(Ions score 67)		
324 - 336	782.3754	1562.7362	1562.7365	-0	0	K.IEQFVYSSPHDNK.S	(Ions score 72)		
324 - 336	521.9194	1562.7363	1562.7365	-0	0	K.IEQFVYSSPHDNK.S	(Ions score 48)		
324 - 336	782.3755	1562.7364	1562.7365	-0	0	K.IEQFVYSSPHDNK.S	(Ions score 42)		
324 - 336	782.3755	1562.7364	1562.7365	-0	0	K.IEQFVYSSPHDNK.S	(Ions score 66)		
324 - 336	782.3755	1562.7365	1562.7365	-0	0	K.IEQFVYSSPHDNK.S	(Ions score 70)		
324 - 336	782.3757	1562.7368	1562.7365	0	0	K.IEQFVYSSPHDNK.S	(Ions score 66)		
324 - 336	782.3758	1562.7371	1562.7365	0	0	K.IEQFVYSSPHDNK.S	(Ions score 39)		
324 - 336	782.3761	1562.7376	1562.7365	1	0	K.IEQFVYSSPHDNK.S	(Ions score 55)		
324 - 336	782.3761	1562.7377	1562.7365	1	0	K.IEQFVYSSPHDNK.S	(Ions score 46)		
324 - 336	782.3762	1562.7378	1562.7365	1	0	K.IEQFVYSSPHDNK.S	(Ions score 65)		
324 - 336	521.9199	1562.7379	1562.7365	1	0	K.IEQFVYSSPHDNK.S	(Ions score 36)		
324 - 336	521.9199	1562.7380	1562.7365	1	0	K.IEQFVYSSPHDNK.S	(Ions score 46)		

324 - 336	782.3763	1562.7380	1562.7365	1	0	K.IEQFVYSSPHDNK.S	(Ions score 71)
324 - 336	782.3763	1562.7380	1562.7365	1	0	K.IEQFVYSSPHDNK.S	(Ions score 51)
324 - 336	521.9200	1562.7380	1562.7365	1	0	K.IEQFVYSSPHDNK.S	(Ions score 39)
324 - 336	782.3763	1562.7381	1562.7365	1	0	K.IEQFVYSSPHDNK.S	(Ions score 54)
324 - 336	521.9200	1562.7381	1562.7365	1	0	K.IEQFVYSSPHDNK.S	(Ions score 34)
324 - 336	1563.7454	1562.7381	1562.7365	1	0	K.IEQFVYSSPHDNK.S	(Ions score 84)
324 - 336	1563.7454	1562.7381	1562.7365	1	0	K.IEQFVYSSPHDNK.S	(Ions score 72)
324 - 336	1563.7455	1562.7382	1562.7365	1	0	K.IEQFVYSSPHDNK.S	(Ions score 82)
324 - 336	782.3764	1562.7382	1562.7365	1	0	K.IEQFVYSSPHDNK.S	(Ions score 59)
324 - 336	782.3764	1562.7383	1562.7365	1	0	K.IEQFVYSSPHDNK.S	(Ions score 39)
324 - 336	782.3764	1562.7383	1562.7365	1	0	K.IEQFVYSSPHDNK.S	(Ions score 59)
324 - 336	521.9201	1562.7384	1562.7365	1	0	K.IEQFVYSSPHDNK.S	(Ions score 39)
324 - 336	521.9201	1562.7384	1562.7365	1	0	K.IEQFVYSSPHDNK.S	(Ions score 43)
324 - 336	782.3765	1562.7385	1562.7365	1	0	K.IEQFVYSSPHDNK.S	(Ions score 62)
324 - 336	521.9201	1562.7385	1562.7365	1	0	K.IEQFVYSSPHDNK.S	(Ions score 40)
324 - 336	521.9201	1562.7385	1562.7365	1	0	K.IEQFVYSSPHDNK.S	(Ions score 22)
324 - 336	782.3766	1562.7386	1562.7365	1	0	K.IEQFVYSSPHDNK.S	(Ions score 67)
324 - 336	782.3766	1562.7387	1562.7365	1	0	K.IEQFVYSSPHDNK.S	(Ions score 64)
324 - 336	521.9202	1562.7388	1562.7365	1	0	K.IEQFVYSSPHDNK.S	(Ions score 38)
324 - 336	782.3767	1562.7389	1562.7365	2	0	K.IEQFVYSSPHDNK.S	(Ions score 67)
324 - 336	521.9202	1562.7389	1562.7365	2	0	K.IEQFVYSSPHDNK.S	(Ions score 46)
324 - 336	782.3767	1562.7389	1562.7365	2	0	K.IEQFVYSSPHDNK.S	(Ions score 49)
324 - 336	521.9202	1562.7389	1562.7365	2	0	K.IEQFVYSSPHDNK.S	(Ions score 50)
324 - 336	782.3767	1562.7389	1562.7365	2	0	K.IEQFVYSSPHDNK.S	(Ions score 58)
324 - 336	1563.7462	1562.7389	1562.7365	2	0	K.IEQFVYSSPHDNK.S	(Ions score 82)
324 - 336	782.3768	1562.7390	1562.7365	2	0	K.IEQFVYSSPHDNK.S	(Ions score 66)
324 - 336	521.9203	1562.7390	1562.7365	2	0	K.IEQFVYSSPHDNK.S	(Ions score 40)
324 - 336	782.3768	1562.7390	1562.7365	2	0	K.IEQFVYSSPHDNK.S	(Ions score 73)
324 - 336	782.3768	1562.7390	1562.7365	2	0	K.IEQFVYSSPHDNK.S	(Ions score 70)
324 - 336	521.9203	1562.7391	1562.7365	2	0	K.IEQFVYSSPHDNK.S	(Ions score 39)
324 - 336	782.3768	1562.7391	1562.7365	2	0	K.IEQFVYSSPHDNK.S	(Ions score 62)
324 - 336	782.3768	1562.7391	1562.7365	2	0	K.IEQFVYSSPHDNK.S	(Ions score 60)
324 - 336	782.3768	1562.7391	1562.7365	2	0	K.IEQFVYSSPHDNK.S	(Ions score 39)
324 - 336	521.9203	1562.7391	1562.7365	2	0	K.IEQFVYSSPHDNK.S	(Ions score 46)
324 - 336	782.3768	1562.7391	1562.7365	2	0	K.IEQFVYSSPHDNK.S	(Ions score 68)
324 - 336	782.3768	1562.7391	1562.7365	2	0	K.IEQFVYSSPHDNK.S	(Ions score 70)
324 - 336	521.9203	1562.7391	1562.7365	2	0	K.IEQFVYSSPHDNK.S	(Ions score 30)
324 - 336	782.3769	1562.7392	1562.7365	2	0	K.IEQFVYSSPHDNK.S	(Ions score 70)
324 - 336	521.9203	1562.7392	1562.7365	2	0	K.IEQFVYSSPHDNK.S	(Ions score 46)
324 - 336	521.9204	1562.7392	1562.7365	2	0	K.IEQFVYSSPHDNK.S	(Ions score 45)
324 - 336	782.3769	1562.7392	1562.7365	2	0	K.IEQFVYSSPHDNK.S	(Ions score 51)
324 - 336	782.3769	1562.7393	1562.7365	2	0	K.IEQFVYSSPHDNK.S	(Ions score 55)
324 - 336	521.9204	1562.7393	1562.7365	2	0	K.IEQFVYSSPHDNK.S	(Ions score 44)
324 - 336	782.3769	1562.7393	1562.7365	2	0	K.IEQFVYSSPHDNK.S	(Ions score 70)
324 - 336	782.3769	1562.7393	1562.7365	2	0	K.IEQFVYSSPHDNK.S	(Ions score 53)
324 - 336	782.3769	1562.7393	1562.7365	2	0	K.IEQFVYSSPHDNK.S	(Ions score 56)
324 - 336	782.3769	1562.7393	1562.7365	2	0	K.IEQFVYSSPHDNK.S	(Ions score 67)
324 - 336	782.3770	1562.7394	1562.7365	2	0	K.IEQFVYSSPHDNK.S	(Ions score 74)
324 - 336	521.9204	1562.7394	1562.7365	2	0	K.IEQFVYSSPHDNK.S	(Ions score 49)
324 - 336	782.3770	1562.7394	1562.7365	2	0	K.IEQFVYSSPHDNK.S	(Ions score 59)
324 - 336	782.3770	1562.7394	1562.7365	2	0	K.IEQFVYSSPHDNK.S	(Ions score 69)
324 - 336	1563.7467	1562.7394	1562.7365	2	0	K.IEQFVYSSPHDNK.S	(Ions score 79)
324 - 336	521.9204	1562.7395	1562.7365	2	0	K.IEQFVYSSPHDNK.S	(Ions score 53)
324 - 336	782.3770	1562.7395	1562.7365	2	0	K.IEQFVYSSPHDNK.S	(Ions score 69)
324 - 336	782.3770	1562.7395	1562.7365	2	0	K.IEQFVYSSPHDNK.S	(Ions score 51)
324 - 336	782.3771	1562.7396	1562.7365	2	0	K.IEQFVYSSPHDNK.S	(Ions score 31)
324 - 336	521.9205	1562.7396	1562.7365	2	0	K.IEQFVYSSPHDNK.S	(Ions score 43)

324 - 336	782.3771	1562.7396	1562.7365	2	0	K.IEQFVYSSPHDNK.S	(Ions score 37)
324 - 336	782.3771	1562.7397	1562.7365	2	0	K.IEQFVYSSPHDNK.S	(Ions score 63)
324 - 336	1563.7469	1562.7397	1562.7365	2	0	K.IEQFVYSSPHDNK.S	(Ions score 72)
324 - 336	782.3771	1562.7397	1562.7365	2	0	K.IEQFVYSSPHDNK.S	(Ions score 65)
324 - 336	521.9205	1562.7397	1562.7365	2	0	K.IEQFVYSSPHDNK.S	(Ions score 40)
324 - 336	521.9205	1562.7398	1562.7365	2	0	K.IEQFVYSSPHDNK.S	(Ions score 22)
324 - 336	521.9205	1562.7398	1562.7365	2	0	K.IEQFVYSSPHDNK.S	(Ions score 33)
324 - 336	782.3772	1562.7399	1562.7365	2	0	K.IEQFVYSSPHDNK.S	(Ions score 68)
324 - 336	782.3772	1562.7399	1562.7365	2	0	K.IEQFVYSSPHDNK.S	(Ions score 74)
324 - 336	782.3772	1562.7399	1562.7365	2	0	K.IEQFVYSSPHDNK.S	(Ions score 73)
324 - 336	521.9206	1562.7399	1562.7365	2	0	K.IEQFVYSSPHDNK.S	(Ions score 37)
324 - 336	521.9206	1562.7399	1562.7365	2	0	K.IEQFVYSSPHDNK.S	(Ions score 48)
324 - 336	782.3772	1562.7399	1562.7365	2	0	K.IEQFVYSSPHDNK.S	(Ions score 60)
324 - 336	782.3772	1562.7399	1562.7365	2	0	K.IEQFVYSSPHDNK.S	(Ions score 56)
324 - 336	782.3773	1562.7399	1562.7365	2	0	K.IEQFVYSSPHDNK.S	(Ions score 75)
324 - 336	782.3773	1562.7400	1562.7365	2	0	K.IEQFVYSSPHDNK.S	(Ions score 45)
324 - 336	521.9206	1562.7400	1562.7365	2	0	K.IEQFVYSSPHDNK.S	(Ions score 56)
324 - 336	1563.7473	1562.7400	1562.7365	2	0	K.IEQFVYSSPHDNK.S	(Ions score 82)
324 - 336	782.3773	1562.7401	1562.7365	2	0	K.IEQFVYSSPHDNK.S	(Ions score 64)
324 - 336	521.9207	1562.7401	1562.7365	2	0	K.IEQFVYSSPHDNK.S	(Ions score 46)
324 - 336	521.9207	1562.7402	1562.7365	2	0	K.IEQFVYSSPHDNK.S	(Ions score 46)
324 - 336	521.9207	1562.7402	1562.7365	2	0	K.IEQFVYSSPHDNK.S	(Ions score 41)
324 - 336	521.9207	1562.7402	1562.7365	2	0	K.IEQFVYSSPHDNK.S	(Ions score 51)
324 - 336	521.9207	1562.7402	1562.7365	2	0	K.IEQFVYSSPHDNK.S	(Ions score 30)
324 - 336	782.3774	1562.7403	1562.7365	2	0	K.IEQFVYSSPHDNK.S	(Ions score 70)
324 - 336	782.3774	1562.7403	1562.7365	2	0	K.IEQFVYSSPHDNK.S	(Ions score 58)
324 - 336	521.9207	1562.7404	1562.7365	2	0	K.IEQFVYSSPHDNK.S	(Ions score 26)
324 - 336	521.9208	1562.7405	1562.7365	3	0	K.IEQFVYSSPHDNK.S	(Ions score 51)
324 - 336	521.9208	1562.7405	1562.7365	3	0	K.IEQFVYSSPHDNK.S	(Ions score 44)
324 - 336	521.9208	1562.7405	1562.7365	3	0	K.IEQFVYSSPHDNK.S	(Ions score 52)
324 - 336	521.9208	1562.7405	1562.7365	3	0	K.IEQFVYSSPHDNK.S	(Ions score 39)
324 - 336	782.3775	1562.7405	1562.7365	3	0	K.IEQFVYSSPHDNK.S	(Ions score 45)
324 - 336	521.9208	1562.7406	1562.7365	3	0	K.IEQFVYSSPHDNK.S	(Ions score 30)
324 - 336	521.9208	1562.7406	1562.7365	3	0	K.IEQFVYSSPHDNK.S	(Ions score 34)
324 - 336	521.9208	1562.7406	1562.7365	3	0	K.IEQFVYSSPHDNK.S	(Ions score 44)
324 - 336	521.9208	1562.7406	1562.7365	3	0	K.IEQFVYSSPHDNK.S	(Ions score 38)
324 - 336	521.9208	1562.7407	1562.7365	3	0	K.IEQFVYSSPHDNK.S	(Ions score 44)
324 - 336	521.9209	1562.7408	1562.7365	3	0	K.IEQFVYSSPHDNK.S	(Ions score 30)
324 - 336	521.9209	1562.7408	1562.7365	3	0	K.IEQFVYSSPHDNK.S	(Ions score 35)
324 - 336	521.9209	1562.7409	1562.7365	3	0	K.IEQFVYSSPHDNK.S	(Ions score 47)
324 - 336	521.9210	1562.7411	1562.7365	3	0	K.IEQFVYSSPHDNK.S	(Ions score 43)
324 - 336	521.9210	1562.7411	1562.7365	3	0	K.IEQFVYSSPHDNK.S	(Ions score 41)
324 - 336	782.3779	1562.7412	1562.7365	3	0	K.IEQFVYSSPHDNK.S	(Ions score 28)
324 - 336	521.9210	1562.7413	1562.7365	3	0	K.IEQFVYSSPHDNK.S	(Ions score 43)
324 - 336	782.3779	1562.7413	1562.7365	3	0	K.IEQFVYSSPHDNK.S	(Ions score 67)
324 - 336	521.9211	1562.7414	1562.7365	3	0	K.IEQFVYSSPHDNK.S	(Ions score 48)
324 - 336	782.3780	1562.7414	1562.7365	3	0	K.IEQFVYSSPHDNK.S	(Ions score 63)
324 - 336	521.9211	1562.7415	1562.7365	3	0	K.IEQFVYSSPHDNK.S	(Ions score 41)
324 - 336	782.3780	1562.7415	1562.7365	3	0	K.IEQFVYSSPHDNK.S	(Ions score 62)
324 - 336	521.9211	1562.7415	1562.7365	3	0	K.IEQFVYSSPHDNK.S	(Ions score 45)
324 - 336	782.3781	1562.7416	1562.7365	3	0	K.IEQFVYSSPHDNK.S	(Ions score 54)
324 - 336	521.9212	1562.7417	1562.7365	3	0	K.IEQFVYSSPHDNK.S	(Ions score 42)
324 - 336	521.9212	1562.7418	1562.7365	3	0	K.IEQFVYSSPHDNK.S	(Ions score 31)
324 - 336	521.9212	1562.7418	1562.7365	3	0	K.IEQFVYSSPHDNK.S	(Ions score 31)
324 - 336	521.9212	1562.7418	1562.7365	3	0	K.IEQFVYSSPHDNK.S	(Ions score 47)
324 - 336	521.9212	1562.7418	1562.7365	3	0	K.IEQFVYSSPHDNK.S	(Ions score 39)
324 - 336	521.9212	1562.7419	1562.7365	3	0	K.IEQFVYSSPHDNK.S	(Ions score 46)

324 - 336	782.3783	1562.7420	1562.7365	3	0	K.IEQFVYSSPHDNK.S	(Ions score 43)
324 - 336	521.9213	1562.7420	1562.7365	4	0	K.IEQFVYSSPHDNK.S	(Ions score 48)
324 - 336	521.9213	1562.7422	1562.7365	4	0	K.IEQFVYSSPHDNK.S	(Ions score 37)
324 - 336	782.3785	1562.7424	1562.7365	4	0	K.IEQFVYSSPHDNK.S	(Ions score 57)
324 - 336	521.9214	1562.7424	1562.7365	4	0	K.IEQFVYSSPHDNK.S	(Ions score 43)
324 - 336	782.3785	1562.7424	1562.7365	4	0	K.IEQFVYSSPHDNK.S	(Ions score 60)
324 - 336	782.3786	1562.7426	1562.7365	4	0	K.IEQFVYSSPHDNK.S	(Ions score 54)
324 - 336	521.9215	1562.7428	1562.7365	4	0	K.IEQFVYSSPHDNK.S	(Ions score 27)
324 - 336	521.9215	1562.7428	1562.7365	4	0	K.IEQFVYSSPHDNK.S	(Ions score 28)
324 - 336	521.9215	1562.7428	1562.7365	4	0	K.IEQFVYSSPHDNK.S	(Ions score 39)
324 - 336	782.3787	1562.7428	1562.7365	4	0	K.IEQFVYSSPHDNK.S	(Ions score 23)
324 - 336	521.9216	1562.7429	1562.7365	4	0	K.IEQFVYSSPHDNK.S	(Ions score 29)
324 - 336	521.9216	1562.7429	1562.7365	4	0	K.IEQFVYSSPHDNK.S	(Ions score 32)
324 - 336	782.3788	1562.7431	1562.7365	4	0	K.IEQFVYSSPHDNK.S	(Ions score 58)
324 - 336	521.9217	1562.7432	1562.7365	4	0	K.IEQFVYSSPHDNK.S	(Ions score 44)
324 - 336	521.9217	1562.7433	1562.7365	4	0	K.IEQFVYSSPHDNK.S	(Ions score 33)
324 - 336	521.9218	1562.7436	1562.7365	5	0	K.IEQFVYSSPHDNK.S	(Ions score 38)
324 - 336	521.9218	1562.7436	1562.7365	5	0	K.IEQFVYSSPHDNK.S	(Ions score 43)
324 - 336	521.9218	1562.7436	1562.7365	5	0	K.IEQFVYSSPHDNK.S	(Ions score 47)
324 - 336	521.9219	1562.7437	1562.7365	5	0	K.IEQFVYSSPHDNK.S	(Ions score 40)
324 - 336	521.9219	1562.7439	1562.7365	5	0	K.IEQFVYSSPHDNK.S	(Ions score 42)
324 - 336	521.9219	1562.7440	1562.7365	5	0	K.IEQFVYSSPHDNK.S	(Ions score 33)
324 - 336	521.9220	1562.7441	1562.7365	5	0	K.IEQFVYSSPHDNK.S	(Ions score 33)
324 - 336	521.9220	1562.7442	1562.7365	5	0	K.IEQFVYSSPHDNK.S	(Ions score 25)
324 - 336	521.9220	1562.7442	1562.7365	5	0	K.IEQFVYSSPHDNK.S	(Ions score 30)
324 - 336	521.9220	1562.7442	1562.7365	5	0	K.IEQFVYSSPHDNK.S	(Ions score 44)
376 - 390	847.4362	1692.8579	1692.8624	-3	1	K.KLDLEAWFPGSGAFR.E	(Ions score 48)
376 - 390	847.4369	1692.8593	1692.8624	-2	1	K.KLDLEAWFPGSGAFR.E	(Ions score 55)
376 - 390	847.4374	1692.8602	1692.8624	-1	1	K.KLDLEAWFPGSGAFR.E	(Ions score 63)
376 - 390	847.4374	1692.8603	1692.8624	-1	1	K.KLDLEAWFPGSGAFR.E	(Ions score 59)
376 - 390	565.2948	1692.8626	1692.8624	0	1	K.KLDLEAWFPGSGAFR.E	(Ions score 23)
376 - 390	847.4386	1692.8626	1692.8624	0	1	K.KLDLEAWFPGSGAFR.E	(Ions score 57)
376 - 390	565.2949	1692.8628	1692.8624	0	1	K.KLDLEAWFPGSGAFR.E	(Ions score 23)
376 - 390	847.4387	1692.8629	1692.8624	0	1	K.KLDLEAWFPGSGAFR.E	(Ions score 31)
376 - 390	847.4388	1692.8630	1692.8624	0	1	K.KLDLEAWFPGSGAFR.E	(Ions score 62)
376 - 390	565.2950	1692.8633	1692.8624	1	1	K.KLDLEAWFPGSGAFR.E	(Ions score 31)
376 - 390	565.2950	1692.8633	1692.8624	1	1	K.KLDLEAWFPGSGAFR.E	(Ions score 31)
376 - 390	424.2231	1692.8634	1692.8624	1	1	K.KLDLEAWFPGSGAFR.E	(Ions score 55)
376 - 390	847.4390	1692.8635	1692.8624	1	1	K.KLDLEAWFPGSGAFR.E	(Ions score 67)
376 - 390	565.2951	1692.8635	1692.8624	1	1	K.KLDLEAWFPGSGAFR.E	(Ions score 25)
376 - 390	424.2232	1692.8635	1692.8624	1	1	K.KLDLEAWFPGSGAFR.E	(Ions score 43)
376 - 390	424.2232	1692.8636	1692.8624	1	1	K.KLDLEAWFPGSGAFR.E	(Ions score 56)
376 - 390	424.2232	1692.8636	1692.8624	1	1	K.KLDLEAWFPGSGAFR.E	(Ions score 50)
376 - 390	565.2952	1692.8637	1692.8624	1	1	K.KLDLEAWFPGSGAFR.E	(Ions score 25)
376 - 390	847.4391	1692.8637	1692.8624	1	1	K.KLDLEAWFPGSGAFR.E	(Ions score 58)
376 - 390	847.4392	1692.8638	1692.8624	1	1	K.KLDLEAWFPGSGAFR.E	(Ions score 42)
376 - 390	847.4392	1692.8638	1692.8624	1	1	K.KLDLEAWFPGSGAFR.E	(Ions score 65)
376 - 390	424.2232	1692.8638	1692.8624	1	1	K.KLDLEAWFPGSGAFR.E	(Ions score 57)
376 - 390	424.2233	1692.8639	1692.8624	1	1	K.KLDLEAWFPGSGAFR.E	(Ions score 53)
376 - 390	424.2233	1692.8640	1692.8624	1	1	K.KLDLEAWFPGSGAFR.E	(Ions score 52)
376 - 390	847.4393	1692.8640	1692.8624	1	1	K.KLDLEAWFPGSGAFR.E	(Ions score 31)
376 - 390	847.4393	1692.8640	1692.8624	1	1	K.KLDLEAWFPGSGAFR.E	(Ions score 54)
376 - 390	1693.8713	1692.8641	1692.8624	1	1	K.KLDLEAWFPGSGAFR.E	(Ions score 90)
376 - 390	424.2234	1692.8643	1692.8624	1	1	K.KLDLEAWFPGSGAFR.E	(Ions score 55)
376 - 390	847.4394	1692.8643	1692.8624	1	1	K.KLDLEAWFPGSGAFR.E	(Ions score 29)
376 - 390	847.4394	1692.8643	1692.8624	1	1	K.KLDLEAWFPGSGAFR.E	(Ions score 46)
376 - 390	424.2234	1692.8644	1692.8624	1	1	K.KLDLEAWFPGSGAFR.E	(Ions score 58)

376 - 390	424.2234	1692.8645	1692.8624	1	1	K.KLDLEAWFPGSGAFR.E	(Ions score 53)
376 - 390	424.2234	1692.8645	1692.8624	1	1	K.KLDLEAWFPGSGAFR.E	(Ions score 52)
376 - 390	565.2954	1692.8645	1692.8624	1	1	K.KLDLEAWFPGSGAFR.E	(Ions score 27)
376 - 390	565.2955	1692.8646	1692.8624	1	1	K.KLDLEAWFPGSGAFR.E	(Ions score 24)
376 - 390	424.2234	1692.8646	1692.8624	1	1	K.KLDLEAWFPGSGAFR.E	(Ions score 53)
376 - 390	424.2234	1692.8647	1692.8624	1	1	K.KLDLEAWFPGSGAFR.E	(Ions score 55)
376 - 390	424.2234	1692.8647	1692.8624	1	1	K.KLDLEAWFPGSGAFR.E	(Ions score 60)
376 - 390	565.2955	1692.8647	1692.8624	1	1	K.KLDLEAWFPGSGAFR.E	(Ions score 26)
376 - 390	424.2235	1692.8648	1692.8624	1	1	K.KLDLEAWFPGSGAFR.E	(Ions score 56)
376 - 390	424.2235	1692.8648	1692.8624	1	1	K.KLDLEAWFPGSGAFR.E	(Ions score 56)
376 - 390	565.2955	1692.8648	1692.8624	1	1	K.KLDLEAWFPGSGAFR.E	(Ions score 29)
376 - 390	565.2955	1692.8648	1692.8624	1	1	K.KLDLEAWFPGSGAFR.E	(Ions score 28)
376 - 390	565.2956	1692.8649	1692.8624	1	1	K.KLDLEAWFPGSGAFR.E	(Ions score 24)
376 - 390	424.2235	1692.8649	1692.8624	1	1	K.KLDLEAWFPGSGAFR.E	(Ions score 56)
376 - 390	424.2235	1692.8649	1692.8624	2	1	K.KLDLEAWFPGSGAFR.E	(Ions score 60)
376 - 390	847.4398	1692.8651	1692.8624	2	1	K.KLDLEAWFPGSGAFR.E	(Ions score 62)
376 - 390	565.2956	1692.8651	1692.8624	2	1	K.KLDLEAWFPGSGAFR.E	(Ions score 30)
376 - 390	847.4398	1692.8651	1692.8624	2	1	K.KLDLEAWFPGSGAFR.E	(Ions score 32)
376 - 390	1693.8724	1692.8652	1692.8624	2	1	K.KLDLEAWFPGSGAFR.E	(Ions score 76)
376 - 390	424.2236	1692.8652	1692.8624	2	1	K.KLDLEAWFPGSGAFR.E	(Ions score 51)
376 - 390	847.4399	1692.8652	1692.8624	2	1	K.KLDLEAWFPGSGAFR.E	(Ions score 65)
376 - 390	565.2957	1692.8652	1692.8624	2	1	K.KLDLEAWFPGSGAFR.E	(Ions score 25)
376 - 390	847.4400	1692.8654	1692.8624	2	1	K.KLDLEAWFPGSGAFR.E	(Ions score 65)
376 - 390	565.2957	1692.8654	1692.8624	2	1	K.KLDLEAWFPGSGAFR.E	(Ions score 24)
376 - 390	565.2957	1692.8654	1692.8624	2	1	K.KLDLEAWFPGSGAFR.E	(Ions score 27)
376 - 390	847.4400	1692.8654	1692.8624	2	1	K.KLDLEAWFPGSGAFR.E	(Ions score 65)
376 - 390	847.4400	1692.8655	1692.8624	2	1	K.KLDLEAWFPGSGAFR.E	(Ions score 58)
376 - 390	565.2958	1692.8655	1692.8624	2	1	K.KLDLEAWFPGSGAFR.E	(Ions score 30)
376 - 390	847.4400	1692.8655	1692.8624	2	1	K.KLDLEAWFPGSGAFR.E	(Ions score 49)
376 - 390	565.2958	1692.8655	1692.8624	2	1	K.KLDLEAWFPGSGAFR.E	(Ions score 31)
376 - 390	424.2237	1692.8655	1692.8624	2	1	K.KLDLEAWFPGSGAFR.E	(Ions score 53)
376 - 390	565.2958	1692.8656	1692.8624	2	1	K.KLDLEAWFPGSGAFR.E	(Ions score 27)
376 - 390	565.2958	1692.8656	1692.8624	2	1	K.KLDLEAWFPGSGAFR.E	(Ions score 32)
376 - 390	565.2958	1692.8657	1692.8624	2	1	K.KLDLEAWFPGSGAFR.E	(Ions score 29)
376 - 390	565.2958	1692.8657	1692.8624	2	1	K.KLDLEAWFPGSGAFR.E	(Ions score 26)
376 - 390	847.4401	1692.8657	1692.8624	2	1	K.KLDLEAWFPGSGAFR.E	(Ions score 73)
376 - 390	565.2959	1692.8658	1692.8624	2	1	K.KLDLEAWFPGSGAFR.E	(Ions score 25)
376 - 390	847.4402	1692.8658	1692.8624	2	1	K.KLDLEAWFPGSGAFR.E	(Ions score 62)
376 - 390	565.2959	1692.8658	1692.8624	2	1	K.KLDLEAWFPGSGAFR.E	(Ions score 26)
376 - 390	847.4402	1692.8659	1692.8624	2	1	K.KLDLEAWFPGSGAFR.E	(Ions score 64)
376 - 390	847.4402	1692.8659	1692.8624	2	1	K.KLDLEAWFPGSGAFR.E	(Ions score 52)
376 - 390	565.2959	1692.8659	1692.8624	2	1	K.KLDLEAWFPGSGAFR.E	(Ions score 36)
376 - 390	565.2959	1692.8659	1692.8624	2	1	K.KLDLEAWFPGSGAFR.E	(Ions score 30)
376 - 390	847.4402	1692.8659	1692.8624	2	1	K.KLDLEAWFPGSGAFR.E	(Ions score 61)
376 - 390	565.2959	1692.8659	1692.8624	2	1	K.KLDLEAWFPGSGAFR.E	(Ions score 29)
376 - 390	847.4402	1692.8659	1692.8624	2	1	K.KLDLEAWFPGSGAFR.E	(Ions score 54)
376 - 390	847.4402	1692.8659	1692.8624	2	1	K.KLDLEAWFPGSGAFR.E	(Ions score 57)
376 - 390	424.2238	1692.8660	1692.8624	2	1	K.KLDLEAWFPGSGAFR.E	(Ions score 52)
376 - 390	847.4403	1692.8660	1692.8624	2	1	K.KLDLEAWFPGSGAFR.E	(Ions score 62)
376 - 390	424.2238	1692.8660	1692.8624	2	1	K.KLDLEAWFPGSGAFR.E	(Ions score 52)
376 - 390	565.2960	1692.8660	1692.8624	2	1	K.KLDLEAWFPGSGAFR.E	(Ions score 29)
376 - 390	565.2960	1692.8660	1692.8624	2	1	K.KLDLEAWFPGSGAFR.E	(Ions score 27)
376 - 390	847.4403	1692.8661	1692.8624	2	1	K.KLDLEAWFPGSGAFR.E	(Ions score 75)
376 - 390	847.4403	1692.8661	1692.8624	2	1	K.KLDLEAWFPGSGAFR.E	(Ions score 53)
376 - 390	565.2960	1692.8661	1692.8624	2	1	K.KLDLEAWFPGSGAFR.E	(Ions score 38)
376 - 390	565.2960	1692.8661	1692.8624	2	1	K.KLDLEAWFPGSGAFR.E	(Ions score 28)
376 - 390	424.2238	1692.8661	1692.8624	2	1	K.KLDLEAWFPGSGAFR.E	(Ions score 54)

376 - 390	565.2960	1692.8661	1692.8624	2	1	K.KLDLEAWFPGSGAFR.E	(Ions score 28)
376 - 390	847.4403	1692.8661	1692.8624	2	1	K.KLDLEAWFPGSGAFR.E	(Ions score 46)
376 - 390	847.4403	1692.8661	1692.8624	2	1	K.KLDLEAWFPGSGAFR.E	(Ions score 63)
376 - 390	565.2960	1692.8661	1692.8624	2	1	K.KLDLEAWFPGSGAFR.E	(Ions score 29)
376 - 390	424.2238	1692.8661	1692.8624	2	1	K.KLDLEAWFPGSGAFR.E	(Ions score 55)
376 - 390	847.4404	1692.8662	1692.8624	2	1	K.KLDLEAWFPGSGAFR.E	(Ions score 73)
376 - 390	847.4404	1692.8662	1692.8624	2	1	K.KLDLEAWFPGSGAFR.E	(Ions score 61)
376 - 390	565.2960	1692.8662	1692.8624	2	1	K.KLDLEAWFPGSGAFR.E	(Ions score 30)
376 - 390	847.4404	1692.8662	1692.8624	2	1	K.KLDLEAWFPGSGAFR.E	(Ions score 60)
376 - 390	847.4404	1692.8662	1692.8624	2	1	K.KLDLEAWFPGSGAFR.E	(Ions score 52)
376 - 390	847.4404	1692.8662	1692.8624	2	1	K.KLDLEAWFPGSGAFR.E	(Ions score 62)
376 - 390	565.2960	1692.8662	1692.8624	2	1	K.KLDLEAWFPGSGAFR.E	(Ions score 28)
376 - 390	847.4404	1692.8662	1692.8624	2	1	K.KLDLEAWFPGSGAFR.E	(Ions score 66)
376 - 390	847.4404	1692.8663	1692.8624	2	1	K.KLDLEAWFPGSGAFR.E	(Ions score 68)
376 - 390	565.2960	1692.8663	1692.8624	2	1	K.KLDLEAWFPGSGAFR.E	(Ions score 24)
376 - 390	565.2960	1692.8663	1692.8624	2	1	K.KLDLEAWFPGSGAFR.E	(Ions score 30)
376 - 390	565.2960	1692.8663	1692.8624	2	1	K.KLDLEAWFPGSGAFR.E	(Ions score 33)
376 - 390	847.4404	1692.8663	1692.8624	2	1	K.KLDLEAWFPGSGAFR.E	(Ions score 62)
376 - 390	847.4404	1692.8663	1692.8624	2	1	K.KLDLEAWFPGSGAFR.E	(Ions score 66)
376 - 390	847.4404	1692.8663	1692.8624	2	1	K.KLDLEAWFPGSGAFR.E	(Ions score 27)
376 - 390	565.2961	1692.8663	1692.8624	2	1	K.KLDLEAWFPGSGAFR.E	(Ions score 33)
376 - 390	847.4404	1692.8663	1692.8624	2	1	K.KLDLEAWFPGSGAFR.E	(Ions score 63)
376 - 390	424.2239	1692.8663	1692.8624	2	1	K.KLDLEAWFPGSGAFR.E	(Ions score 49)
376 - 390	565.2961	1692.8663	1692.8624	2	1	K.KLDLEAWFPGSGAFR.E	(Ions score 25)
376 - 390	847.4405	1692.8664	1692.8624	2	1	K.KLDLEAWFPGSGAFR.E	(Ions score 69)
376 - 390	565.2961	1692.8664	1692.8624	2	1	K.KLDLEAWFPGSGAFR.E	(Ions score 29)
376 - 390	847.4405	1692.8664	1692.8624	2	1	K.KLDLEAWFPGSGAFR.E	(Ions score 71)
376 - 390	847.4405	1692.8664	1692.8624	2	1	K.KLDLEAWFPGSGAFR.E	(Ions score 57)
376 - 390	847.4405	1692.8664	1692.8624	2	1	K.KLDLEAWFPGSGAFR.E	(Ions score 71)
376 - 390	565.2961	1692.8664	1692.8624	2	1	K.KLDLEAWFPGSGAFR.E	(Ions score 28)
376 - 390	565.2961	1692.8664	1692.8624	2	1	K.KLDLEAWFPGSGAFR.E	(Ions score 21)
376 - 390	565.2961	1692.8664	1692.8624	2	1	K.KLDLEAWFPGSGAFR.E	(Ions score 21)
376 - 390	847.4405	1692.8664	1692.8624	2	1	K.KLDLEAWFPGSGAFR.E	(Ions score 70)
376 - 390	565.2961	1692.8664	1692.8624	2	1	K.KLDLEAWFPGSGAFR.E	(Ions score 26)
376 - 390	847.4405	1692.8664	1692.8624	2	1	K.KLDLEAWFPGSGAFR.E	(Ions score 28)
376 - 390	847.4405	1692.8665	1692.8624	2	1	K.KLDLEAWFPGSGAFR.E	(Ions score 65)
376 - 390	847.4405	1692.8665	1692.8624	2	1	K.KLDLEAWFPGSGAFR.E	(Ions score 82)
376 - 390	565.2961	1692.8665	1692.8624	2	1	K.KLDLEAWFPGSGAFR.E	(Ions score 27)
376 - 390	847.4405	1692.8665	1692.8624	2	1	K.KLDLEAWFPGSGAFR.E	(Ions score 61)
376 - 390	565.2961	1692.8665	1692.8624	2	1	K.KLDLEAWFPGSGAFR.E	(Ions score 29)
376 - 390	847.4405	1692.8665	1692.8624	2	1	K.KLDLEAWFPGSGAFR.E	(Ions score 54)
376 - 390	847.4405	1692.8665	1692.8624	2	1	K.KLDLEAWFPGSGAFR.E	(Ions score 67)
376 - 390	565.2961	1692.8665	1692.8624	2	1	K.KLDLEAWFPGSGAFR.E	(Ions score 26)
376 - 390	565.2961	1692.8666	1692.8624	2	1	K.KLDLEAWFPGSGAFR.E	(Ions score 27)
376 - 390	847.4406	1692.8666	1692.8624	2	1	K.KLDLEAWFPGSGAFR.E	(Ions score 71)
376 - 390	847.4406	1692.8666	1692.8624	2	1	K.KLDLEAWFPGSGAFR.E	(Ions score 57)
376 - 390	847.4406	1692.8666	1692.8624	2	1	K.KLDLEAWFPGSGAFR.E	(Ions score 68)
376 - 390	847.4406	1692.8666	1692.8624	3	1	K.KLDLEAWFPGSGAFR.E	(Ions score 61)
376 - 390	847.4406	1692.8666	1692.8624	3	1	K.KLDLEAWFPGSGAFR.E	(Ions score 50)
376 - 390	565.2961	1692.8666	1692.8624	3	1	K.KLDLEAWFPGSGAFR.E	(Ions score 32)
376 - 390	565.2962	1692.8666	1692.8624	3	1	K.KLDLEAWFPGSGAFR.E	(Ions score 24)
376 - 390	565.2962	1692.8666	1692.8624	3	1	K.KLDLEAWFPGSGAFR.E	(Ions score 29)
376 - 390	424.2239	1692.8667	1692.8624	3	1	K.KLDLEAWFPGSGAFR.E	(Ions score 56)
376 - 390	565.2962	1692.8667	1692.8624	3	1	K.KLDLEAWFPGSGAFR.E	(Ions score 29)
376 - 390	847.4406	1692.8667	1692.8624	3	1	K.KLDLEAWFPGSGAFR.E	(Ions score 68)
376 - 390	565.2962	1692.8667	1692.8624	3	1	K.KLDLEAWFPGSGAFR.E	(Ions score 29)
376 - 390	847.4406	1692.8667	1692.8624	3	1	K.KLDLEAWFPGSGAFR.E	(Ions score 58)

376 - 390	847.4406	1692.8667	1692.8624	3	1	K.KLDLEAWFPGSGAFR.E	(Ions score 66)
376 - 390	565.2962	1692.8667	1692.8624	3	1	K.KLDLEAWFPGSGAFR.E	(Ions score 23)
376 - 390	565.2962	1692.8667	1692.8624	3	1	K.KLDLEAWFPGSGAFR.E	(Ions score 25)
376 - 390	847.4406	1692.8667	1692.8624	3	1	K.KLDLEAWFPGSGAFR.E	(Ions score 59)
376 - 390	847.4407	1692.8667	1692.8624	3	1	K.KLDLEAWFPGSGAFR.E	(Ions score 70)
376 - 390	565.2962	1692.8668	1692.8624	3	1	K.KLDLEAWFPGSGAFR.E	(Ions score 27)
376 - 390	565.2962	1692.8668	1692.8624	3	1	K.KLDLEAWFPGSGAFR.E	(Ions score 28)
376 - 390	565.2962	1692.8668	1692.8624	3	1	K.KLDLEAWFPGSGAFR.E	(Ions score 27)
376 - 390	565.2962	1692.8668	1692.8624	3	1	K.KLDLEAWFPGSGAFR.E	(Ions score 29)
376 - 390	565.2962	1692.8668	1692.8624	3	1	K.KLDLEAWFPGSGAFR.E	(Ions score 24)
376 - 390	847.4407	1692.8668	1692.8624	3	1	K.KLDLEAWFPGSGAFR.E	(Ions score 50)
376 - 390	565.2962	1692.8668	1692.8624	3	1	K.KLDLEAWFPGSGAFR.E	(Ions score 27)
376 - 390	847.4407	1692.8668	1692.8624	3	1	K.KLDLEAWFPGSGAFR.E	(Ions score 64)
376 - 390	565.2962	1692.8669	1692.8624	3	1	K.KLDLEAWFPGSGAFR.E	(Ions score 33)
376 - 390	565.2962	1692.8669	1692.8624	3	1	K.KLDLEAWFPGSGAFR.E	(Ions score 22)
376 - 390	847.4407	1692.8669	1692.8624	3	1	K.KLDLEAWFPGSGAFR.E	(Ions score 47)
376 - 390	565.2962	1692.8669	1692.8624	3	1	K.KLDLEAWFPGSGAFR.E	(Ions score 26)
376 - 390	565.2963	1692.8669	1692.8624	3	1	K.KLDLEAWFPGSGAFR.E	(Ions score 24)
376 - 390	565.2963	1692.8669	1692.8624	3	1	K.KLDLEAWFPGSGAFR.E	(Ions score 27)
376 - 390	565.2963	1692.8669	1692.8624	3	1	K.KLDLEAWFPGSGAFR.E	(Ions score 28)
376 - 390	565.2963	1692.8670	1692.8624	3	1	K.KLDLEAWFPGSGAFR.E	(Ions score 29)
376 - 390	847.4408	1692.8670	1692.8624	3	1	K.KLDLEAWFPGSGAFR.E	(Ions score 67)
376 - 390	565.2963	1692.8670	1692.8624	3	1	K.KLDLEAWFPGSGAFR.E	(Ions score 30)
376 - 390	565.2963	1692.8670	1692.8624	3	1	K.KLDLEAWFPGSGAFR.E	(Ions score 24)
376 - 390	424.2240	1692.8670	1692.8624	3	1	K.KLDLEAWFPGSGAFR.E	(Ions score 54)
376 - 390	565.2963	1692.8670	1692.8624	3	1	K.KLDLEAWFPGSGAFR.E	(Ions score 28)
376 - 390	565.2963	1692.8671	1692.8624	3	1	K.KLDLEAWFPGSGAFR.E	(Ions score 29)
376 - 390	565.2963	1692.8671	1692.8624	3	1	K.KLDLEAWFPGSGAFR.E	(Ions score 27)
376 - 390	565.2963	1692.8671	1692.8624	3	1	K.KLDLEAWFPGSGAFR.E	(Ions score 29)
376 - 390	847.4408	1692.8671	1692.8624	3	1	K.KLDLEAWFPGSGAFR.E	(Ions score 62)
376 - 390	847.4408	1692.8671	1692.8624	3	1	K.KLDLEAWFPGSGAFR.E	(Ions score 70)
376 - 390	565.2963	1692.8671	1692.8624	3	1	K.KLDLEAWFPGSGAFR.E	(Ions score 30)
376 - 390	565.2963	1692.8672	1692.8624	3	1	K.KLDLEAWFPGSGAFR.E	(Ions score 25)
376 - 390	565.2963	1692.8672	1692.8624	3	1	K.KLDLEAWFPGSGAFR.E	(Ions score 25)
376 - 390	565.2964	1692.8672	1692.8624	3	1	K.KLDLEAWFPGSGAFR.E	(Ions score 29)
376 - 390	565.2964	1692.8673	1692.8624	3	1	K.KLDLEAWFPGSGAFR.E	(Ions score 31)
376 - 390	565.2964	1692.8673	1692.8624	3	1	K.KLDLEAWFPGSGAFR.E	(Ions score 27)
376 - 390	565.2964	1692.8673	1692.8624	3	1	K.KLDLEAWFPGSGAFR.E	(Ions score 22)
376 - 390	565.2964	1692.8673	1692.8624	3	1	K.KLDLEAWFPGSGAFR.E	(Ions score 27)
376 - 390	565.2964	1692.8674	1692.8624	3	1	K.KLDLEAWFPGSGAFR.E	(Ions score 29)
376 - 390	565.2964	1692.8674	1692.8624	3	1	K.KLDLEAWFPGSGAFR.E	(Ions score 24)
376 - 390	847.4410	1692.8674	1692.8624	3	1	K.KLDLEAWFPGSGAFR.E	(Ions score 29)
376 - 390	565.2964	1692.8674	1692.8624	3	1	K.KLDLEAWFPGSGAFR.E	(Ions score 32)
376 - 390	565.2964	1692.8675	1692.8624	3	1	K.KLDLEAWFPGSGAFR.E	(Ions score 29)
376 - 390	565.2964	1692.8675	1692.8624	3	1	K.KLDLEAWFPGSGAFR.E	(Ions score 26)
376 - 390	565.2964	1692.8675	1692.8624	3	1	K.KLDLEAWFPGSGAFR.E	(Ions score 25)
376 - 390	565.2964	1692.8675	1692.8624	3	1	K.KLDLEAWFPGSGAFR.E	(Ions score 34)
376 - 390	565.2965	1692.8675	1692.8624	3	1	K.KLDLEAWFPGSGAFR.E	(Ions score 28)
376 - 390	565.2965	1692.8675	1692.8624	3	1	K.KLDLEAWFPGSGAFR.E	(Ions score 24)
376 - 390	565.2965	1692.8676	1692.8624	3	1	K.KLDLEAWFPGSGAFR.E	(Ions score 29)
376 - 390	565.2965	1692.8676	1692.8624	3	1	K.KLDLEAWFPGSGAFR.E	(Ions score 25)
376 - 390	565.2965	1692.8676	1692.8624	3	1	K.KLDLEAWFPGSGAFR.E	(Ions score 31)
376 - 390	565.2965	1692.8676	1692.8624	3	1	K.KLDLEAWFPGSGAFR.E	(Ions score 27)
376 - 390	565.2965	1692.8676	1692.8624	3	1	K.KLDLEAWFPGSGAFR.E	(Ions score 30)
376 - 390	565.2965	1692.8676	1692.8624	3	1	K.KLDLEAWFPGSGAFR.E	(Ions score 29)
376 - 390	565.2965	1692.8676	1692.8624	3	1	K.KLDLEAWFPGSGAFR.E	(Ions score 24)
376 - 390	565.2965	1692.8677	1692.8624	3	1	K.KLDLEAWFPGSGAFR.E	(Ions score 28)

376 - 390	565.2965	1692.8677	1692.8624	3	1	K.KLDLEAWFPGSGAFR.E	(Ions score 26)
376 - 390	565.2965	1692.8677	1692.8624	3	1	K.KLDLEAWFPGSGAFR.E	(Ions score 27)
376 - 390	847.4411	1692.8677	1692.8624	3	1	K.KLDLEAWFPGSGAFR.E	(Ions score 60)
376 - 390	847.4411	1692.8677	1692.8624	3	1	K.KLDLEAWFPGSGAFR.E	(Ions score 50)
376 - 390	565.2965	1692.8677	1692.8624	3	1	K.KLDLEAWFPGSGAFR.E	(Ions score 29)
376 - 390	565.2965	1692.8677	1692.8624	3	1	K.KLDLEAWFPGSGAFR.E	(Ions score 25)
376 - 390	565.2965	1692.8678	1692.8624	3	1	K.KLDLEAWFPGSGAFR.E	(Ions score 29)
376 - 390	847.4412	1692.8678	1692.8624	3	1	K.KLDLEAWFPGSGAFR.E	(Ions score 35)
376 - 390	565.2965	1692.8678	1692.8624	3	1	K.KLDLEAWFPGSGAFR.E	(Ions score 26)
376 - 390	565.2965	1692.8678	1692.8624	3	1	K.KLDLEAWFPGSGAFR.E	(Ions score 30)
376 - 390	847.4412	1692.8678	1692.8624	3	1	K.KLDLEAWFPGSGAFR.E	(Ions score 70)
376 - 390	565.2966	1692.8678	1692.8624	3	1	K.KLDLEAWFPGSGAFR.E	(Ions score 24)
376 - 390	847.4412	1692.8678	1692.8624	3	1	K.KLDLEAWFPGSGAFR.E	(Ions score 64)
376 - 390	565.2966	1692.8678	1692.8624	3	1	K.KLDLEAWFPGSGAFR.E	(Ions score 29)
376 - 390	847.4412	1692.8679	1692.8624	3	1	K.KLDLEAWFPGSGAFR.E	(Ions score 65)
376 - 390	847.4412	1692.8679	1692.8624	3	1	K.KLDLEAWFPGSGAFR.E	(Ions score 44)
376 - 390	847.4412	1692.8679	1692.8624	3	1	K.KLDLEAWFPGSGAFR.E	(Ions score 65)
376 - 390	565.2966	1692.8679	1692.8624	3	1	K.KLDLEAWFPGSGAFR.E	(Ions score 27)
376 - 390	565.2966	1692.8679	1692.8624	3	1	K.KLDLEAWFPGSGAFR.E	(Ions score 27)
376 - 390	847.4412	1692.8679	1692.8624	3	1	K.KLDLEAWFPGSGAFR.E	(Ions score 58)
376 - 390	424.2243	1692.8680	1692.8624	3	1	K.KLDLEAWFPGSGAFR.E	(Ions score 50)
376 - 390	565.2966	1692.8680	1692.8624	3	1	K.KLDLEAWFPGSGAFR.E	(Ions score 28)
376 - 390	565.2966	1692.8680	1692.8624	3	1	K.KLDLEAWFPGSGAFR.E	(Ions score 30)
376 - 390	565.2966	1692.8681	1692.8624	3	1	K.KLDLEAWFPGSGAFR.E	(Ions score 29)
376 - 390	565.2966	1692.8681	1692.8624	3	1	K.KLDLEAWFPGSGAFR.E	(Ions score 28)
376 - 390	565.2966	1692.8681	1692.8624	3	1	K.KLDLEAWFPGSGAFR.E	(Ions score 27)
376 - 390	565.2966	1692.8681	1692.8624	3	1	K.KLDLEAWFPGSGAFR.E	(Ions score 28)
376 - 390	565.2967	1692.8682	1692.8624	3	1	K.KLDLEAWFPGSGAFR.E	(Ions score 26)
376 - 390	565.2967	1692.8682	1692.8624	3	1	K.KLDLEAWFPGSGAFR.E	(Ions score 28)
376 - 390	565.2967	1692.8682	1692.8624	3	1	K.KLDLEAWFPGSGAFR.E	(Ions score 28)
376 - 390	565.2967	1692.8682	1692.8624	3	1	K.KLDLEAWFPGSGAFR.E	(Ions score 25)
376 - 390	565.2967	1692.8682	1692.8624	3	1	K.KLDLEAWFPGSGAFR.E	(Ions score 26)
376 - 390	565.2967	1692.8683	1692.8624	3	1	K.KLDLEAWFPGSGAFR.E	(Ions score 27)
376 - 390	565.2967	1692.8683	1692.8624	3	1	K.KLDLEAWFPGSGAFR.E	(Ions score 24)
376 - 390	565.2967	1692.8683	1692.8624	3	1	K.KLDLEAWFPGSGAFR.E	(Ions score 30)
376 - 390	565.2967	1692.8683	1692.8624	3	1	K.KLDLEAWFPGSGAFR.E	(Ions score 30)
376 - 390	565.2967	1692.8683	1692.8624	4	1	K.KLDLEAWFPGSGAFR.E	(Ions score 25)
376 - 390	565.2967	1692.8683	1692.8624	4	1	K.KLDLEAWFPGSGAFR.E	(Ions score 27)
376 - 390	565.2967	1692.8683	1692.8624	4	1	K.KLDLEAWFPGSGAFR.E	(Ions score 29)
376 - 390	847.4415	1692.8684	1692.8624	4	1	K.KLDLEAWFPGSGAFR.E	(Ions score 54)
376 - 390	565.2967	1692.8684	1692.8624	4	1	K.KLDLEAWFPGSGAFR.E	(Ions score 25)
376 - 390	565.2967	1692.8684	1692.8624	4	1	K.KLDLEAWFPGSGAFR.E	(Ions score 28)
376 - 390	565.2967	1692.8684	1692.8624	4	1	K.KLDLEAWFPGSGAFR.E	(Ions score 28)
376 - 390	565.2967	1692.8684	1692.8624	4	1	K.KLDLEAWFPGSGAFR.E	(Ions score 27)
376 - 390	565.2968	1692.8684	1692.8624	4	1	K.KLDLEAWFPGSGAFR.E	(Ions score 28)
376 - 390	565.2968	1692.8685	1692.8624	4	1	K.KLDLEAWFPGSGAFR.E	(Ions score 29)
376 - 390	565.2968	1692.8685	1692.8624	4	1	K.KLDLEAWFPGSGAFR.E	(Ions score 32)
376 - 390	565.2968	1692.8686	1692.8624	4	1	K.KLDLEAWFPGSGAFR.E	(Ions score 24)
376 - 390	565.2968	1692.8686	1692.8624	4	1	K.KLDLEAWFPGSGAFR.E	(Ions score 21)
376 - 390	565.2968	1692.8686	1692.8624	4	1	K.KLDLEAWFPGSGAFR.E	(Ions score 27)
376 - 390	847.4416	1692.8687	1692.8624	4	1	K.KLDLEAWFPGSGAFR.E	(Ions score 44)
376 - 390	565.2968	1692.8687	1692.8624	4	1	K.KLDLEAWFPGSGAFR.E	(Ions score 28)
376 - 390	565.2969	1692.8687	1692.8624	4	1	K.KLDLEAWFPGSGAFR.E	(Ions score 29)
376 - 390	565.2969	1692.8687	1692.8624	4	1	K.KLDLEAWFPGSGAFR.E	(Ions score 26)
376 - 390	565.2969	1692.8688	1692.8624	4	1	K.KLDLEAWFPGSGAFR.E	(Ions score 29)
376 - 390	565.2969	1692.8688	1692.8624	4	1	K.KLDLEAWFPGSGAFR.E	(Ions score 22)
376 - 390	565.2969	1692.8688	1692.8624	4	1	K.KLDLEAWFPGSGAFR.E	(Ions score 24)

376 - 390	847.4417	1692.8688	1692.8624	4	1	K.KLDLEAWFPGSGAFR.E	(Ions score 46)
376 - 390	847.4417	1692.8688	1692.8624	4	1	K.KLDLEAWFPGSGAFR.E	(Ions score 52)
376 - 390	565.2969	1692.8688	1692.8624	4	1	K.KLDLEAWFPGSGAFR.E	(Ions score 27)
376 - 390	565.2969	1692.8688	1692.8624	4	1	K.KLDLEAWFPGSGAFR.E	(Ions score 25)
376 - 390	847.4417	1692.8688	1692.8624	4	1	K.KLDLEAWFPGSGAFR.E	(Ions score 32)
376 - 390	565.2969	1692.8689	1692.8624	4	1	K.KLDLEAWFPGSGAFR.E	(Ions score 27)
376 - 390	565.2969	1692.8689	1692.8624	4	1	K.KLDLEAWFPGSGAFR.E	(Ions score 33)
376 - 390	565.2969	1692.8690	1692.8624	4	1	K.KLDLEAWFPGSGAFR.E	(Ions score 26)
376 - 390	565.2969	1692.8690	1692.8624	4	1	K.KLDLEAWFPGSGAFR.E	(Ions score 28)
376 - 390	565.2970	1692.8691	1692.8624	4	1	K.KLDLEAWFPGSGAFR.E	(Ions score 28)
376 - 390	565.2970	1692.8691	1692.8624	4	1	K.KLDLEAWFPGSGAFR.E	(Ions score 29)
376 - 390	847.4418	1692.8691	1692.8624	4	1	K.KLDLEAWFPGSGAFR.E	(Ions score 65)
376 - 390	565.2970	1692.8691	1692.8624	4	1	K.KLDLEAWFPGSGAFR.E	(Ions score 29)
376 - 390	847.4418	1692.8691	1692.8624	4	1	K.KLDLEAWFPGSGAFR.E	(Ions score 54)
376 - 390	847.4419	1692.8692	1692.8624	4	1	K.KLDLEAWFPGSGAFR.E	(Ions score 51)
376 - 390	847.4419	1692.8693	1692.8624	4	1	K.KLDLEAWFPGSGAFR.E	(Ions score 51)
376 - 390	565.2970	1692.8693	1692.8624	4	1	K.KLDLEAWFPGSGAFR.E	(Ions score 30)
376 - 390	565.2970	1692.8693	1692.8624	4	1	K.KLDLEAWFPGSGAFR.E	(Ions score 24)
376 - 390	565.2970	1692.8693	1692.8624	4	1	K.KLDLEAWFPGSGAFR.E	(Ions score 27)
376 - 390	847.4419	1692.8693	1692.8624	4	1	K.KLDLEAWFPGSGAFR.E	(Ions score 62)
376 - 390	565.2971	1692.8694	1692.8624	4	1	K.KLDLEAWFPGSGAFR.E	(Ions score 29)
376 - 390	565.2971	1692.8694	1692.8624	4	1	K.KLDLEAWFPGSGAFR.E	(Ions score 28)
376 - 390	847.4420	1692.8694	1692.8624	4	1	K.KLDLEAWFPGSGAFR.E	(Ions score 51)
376 - 390	847.4420	1692.8694	1692.8624	4	1	K.KLDLEAWFPGSGAFR.E	(Ions score 62)
376 - 390	847.4420	1692.8694	1692.8624	4	1	K.KLDLEAWFPGSGAFR.E	(Ions score 58)
376 - 390	847.4420	1692.8694	1692.8624	4	1	K.KLDLEAWFPGSGAFR.E	(Ions score 35)
376 - 390	847.4420	1692.8695	1692.8624	4	1	K.KLDLEAWFPGSGAFR.E	(Ions score 58)
376 - 390	847.4420	1692.8695	1692.8624	4	1	K.KLDLEAWFPGSGAFR.E	(Ions score 54)
376 - 390	565.2971	1692.8695	1692.8624	4	1	K.KLDLEAWFPGSGAFR.E	(Ions score 26)
376 - 390	847.4420	1692.8695	1692.8624	4	1	K.KLDLEAWFPGSGAFR.E	(Ions score 65)
376 - 390	565.2971	1692.8696	1692.8624	4	1	K.KLDLEAWFPGSGAFR.E	(Ions score 29)
376 - 390	565.2971	1692.8696	1692.8624	4	1	K.KLDLEAWFPGSGAFR.E	(Ions score 27)
376 - 390	847.4421	1692.8696	1692.8624	4	1	K.KLDLEAWFPGSGAFR.E	(Ions score 32)
376 - 390	565.2971	1692.8696	1692.8624	4	1	K.KLDLEAWFPGSGAFR.E	(Ions score 27)
376 - 390	565.2972	1692.8696	1692.8624	4	1	K.KLDLEAWFPGSGAFR.E	(Ions score 29)
376 - 390	565.2972	1692.8697	1692.8624	4	1	K.KLDLEAWFPGSGAFR.E	(Ions score 27)
376 - 390	565.2972	1692.8697	1692.8624	4	1	K.KLDLEAWFPGSGAFR.E	(Ions score 28)
376 - 390	565.2972	1692.8697	1692.8624	4	1	K.KLDLEAWFPGSGAFR.E	(Ions score 27)
376 - 390	565.2972	1692.8698	1692.8624	4	1	K.KLDLEAWFPGSGAFR.E	(Ions score 26)
376 - 390	847.4422	1692.8698	1692.8624	4	1	K.KLDLEAWFPGSGAFR.E	(Ions score 49)
376 - 390	565.2972	1692.8698	1692.8624	4	1	K.KLDLEAWFPGSGAFR.E	(Ions score 28)
376 - 390	847.4422	1692.8698	1692.8624	4	1	K.KLDLEAWFPGSGAFR.E	(Ions score 39)
376 - 390	847.4422	1692.8698	1692.8624	4	1	K.KLDLEAWFPGSGAFR.E	(Ions score 40)
376 - 390	847.4422	1692.8699	1692.8624	4	1	K.KLDLEAWFPGSGAFR.E	(Ions score 65)
376 - 390	847.4422	1692.8699	1692.8624	4	1	K.KLDLEAWFPGSGAFR.E	(Ions score 47)
376 - 390	565.2973	1692.8699	1692.8624	4	1	K.KLDLEAWFPGSGAFR.E	(Ions score 26)
376 - 390	847.4422	1692.8699	1692.8624	4	1	K.KLDLEAWFPGSGAFR.E	(Ions score 47)
376 - 390	565.2973	1692.8699	1692.8624	4	1	K.KLDLEAWFPGSGAFR.E	(Ions score 25)
376 - 390	847.4423	1692.8700	1692.8624	4	1	K.KLDLEAWFPGSGAFR.E	(Ions score 62)
376 - 390	847.4423	1692.8700	1692.8624	5	1	K.KLDLEAWFPGSGAFR.E	(Ions score 67)
376 - 390	565.2973	1692.8701	1692.8624	5	1	K.KLDLEAWFPGSGAFR.E	(Ions score 23)
376 - 390	565.2973	1692.8701	1692.8624	5	1	K.KLDLEAWFPGSGAFR.E	(Ions score 24)
376 - 390	847.4424	1692.8702	1692.8624	5	1	K.KLDLEAWFPGSGAFR.E	(Ions score 64)
376 - 390	565.2973	1692.8702	1692.8624	5	1	K.KLDLEAWFPGSGAFR.E	(Ions score 27)
376 - 390	847.4424	1692.8702	1692.8624	5	1	K.KLDLEAWFPGSGAFR.E	(Ions score 60)
376 - 390	847.4424	1692.8703	1692.8624	5	1	K.KLDLEAWFPGSGAFR.E	(Ions score 62)
376 - 390	565.2974	1692.8703	1692.8624	5	1	K.KLDLEAWFPGSGAFR.E	(Ions score 27)

376 - 390	565.2974	1692.8703	1692.8624	5	1	K.KLDLEAWFPGSGAFR.E		(Ions score 33)
376 - 390	565.2974	1692.8704	1692.8624	5	1	K.KLDLEAWFPGSGAFR.E		(Ions score 26)
376 - 390	565.2974	1692.8705	1692.8624	5	1	K.KLDLEAWFPGSGAFR.E		(Ions score 26)
376 - 390	847.4426	1692.8706	1692.8624	5	1	K.KLDLEAWFPGSGAFR.E		(Ions score 45)
376 - 390	847.4426	1692.8707	1692.8624	5	1	K.KLDLEAWFPGSGAFR.E		(Ions score 60)
376 - 390	565.2975	1692.8707	1692.8624	5	1	K.KLDLEAWFPGSGAFR.E		(Ions score 30)
376 - 390	565.2975	1692.8707	1692.8624	5	1	K.KLDLEAWFPGSGAFR.E		(Ions score 29)
376 - 390	847.4426	1692.8707	1692.8624	5	1	K.KLDLEAWFPGSGAFR.E		(Ions score 59)
376 - 390	847.4427	1692.8708	1692.8624	5	1	K.KLDLEAWFPGSGAFR.E		(Ions score 40)
376 - 390	847.4427	1692.8708	1692.8624	5	1	K.KLDLEAWFPGSGAFR.E		(Ions score 38)
376 - 390	565.2976	1692.8708	1692.8624	5	1	K.KLDLEAWFPGSGAFR.E		(Ions score 29)
376 - 390	579.2974	1734.8703	1734.8729	-2	1	K.KLDLEAWFPGSGAFR.E	Acetylation (K)	(Ions score 29)
376 - 390	868.4430	1734.8714	1734.8729	-1	1	K.KLDLEAWFPGSGAFR.E	Acetylation (K)	(Ions score 38)
376 - 390	868.4449	1734.8753	1734.8729	1	1	K.KLDLEAWFPGSGAFR.E	Acetylation (K)	(Ions score 64)
376 - 390	868.4454	1734.8762	1734.8729	2	1	K.KLDLEAWFPGSGAFR.E	Acetylation (K)	(Ions score 48)
376 - 390	579.2995	1734.8767	1734.8729	2	1	K.KLDLEAWFPGSGAFR.E	Acetylation (K)	(Ions score 24)
376 - 390	868.4457	1734.8768	1734.8729	2	1	K.KLDLEAWFPGSGAFR.E	Acetylation (K)	(Ions score 66)
376 - 390	868.4458	1734.8771	1734.8729	2	1	K.KLDLEAWFPGSGAFR.E	Acetylation (K)	(Ions score 50)
376 - 390	579.2999	1734.8778	1734.8729	3	1	K.KLDLEAWFPGSGAFR.E	Acetylation (K)	(Ions score 29)
376 - 390	868.4462	1734.8779	1734.8729	3	1	K.KLDLEAWFPGSGAFR.E	Acetylation (K)	(Ions score 55)
376 - 390	868.4466	1734.8786	1734.8729	3	1	K.KLDLEAWFPGSGAFR.E	Acetylation (K)	(Ions score 63)
376 - 390	868.4468	1734.8791	1734.8729	4	1	K.KLDLEAWFPGSGAFR.E	Acetylation (K)	(Ions score 65)
376 - 390	868.4468	1734.8791	1734.8729	4	1	K.KLDLEAWFPGSGAFR.E	Acetylation (K)	(Ions score 51)
376 - 390	579.3003	1734.8792	1734.8729	4	1	K.KLDLEAWFPGSGAFR.E	Acetylation (K)	(Ions score 29)
376 - 390	868.4469	1734.8792	1734.8729	4	1	K.KLDLEAWFPGSGAFR.E	Acetylation (K)	(Ions score 61)
376 - 390	579.3004	1734.8792	1734.8729	4	1	K.KLDLEAWFPGSGAFR.E	Acetylation (K)	(Ions score 23)
376 - 390	579.3004	1734.8793	1734.8729	4	1	K.KLDLEAWFPGSGAFR.E	Acetylation (K)	(Ions score 27)
376 - 390	579.3004	1734.8793	1734.8729	4	1	K.KLDLEAWFPGSGAFR.E	Acetylation (K)	(Ions score 28)
376 - 390	579.3006	1734.8799	1734.8729	4	1	K.KLDLEAWFPGSGAFR.E	Acetylation (K)	(Ions score 31)
376 - 390	579.3007	1734.8802	1734.8729	4	1	K.KLDLEAWFPGSGAFR.E	Acetylation (K)	(Ions score 26)
376 - 390	868.4474	1734.8803	1734.8729	4	1	K.KLDLEAWFPGSGAFR.E	Acetylation (K)	(Ions score 59)
376 - 390	579.3010	1734.8811	1734.8729	5	1	K.KLDLEAWFPGSGAFR.E	Acetylation (K)	(Ions score 28)
376 - 390	868.4480	1734.8815	1734.8729	5	1	K.KLDLEAWFPGSGAFR.E	Acetylation (K)	(Ions score 45)
377 - 390	783.3908	1564.7671	1564.7674	-0	0	K.LDLEAWFPGSGAFR.E		(Ions score 40)
377 - 390	783.3915	1564.7685	1564.7674	1	0	K.LDLEAWFPGSGAFR.E		(Ions score 25)
377 - 390	783.3919	1564.7693	1564.7674	1	0	K.LDLEAWFPGSGAFR.E		(Ions score 27)
377 - 390	783.3922	1564.7698	1564.7674	2	0	K.LDLEAWFPGSGAFR.E		(Ions score 42)
377 - 390	783.3922	1564.7698	1564.7674	2	0	K.LDLEAWFPGSGAFR.E		(Ions score 48)
377 - 390	783.3926	1564.7706	1564.7674	2	0	K.LDLEAWFPGSGAFR.E		(Ions score 39)
377 - 390	783.3926	1564.7706	1564.7674	2	0	K.LDLEAWFPGSGAFR.E		(Ions score 45)
377 - 390	522.5975	1564.7707	1564.7674	2	0	K.LDLEAWFPGSGAFR.E		(Ions score 37)
377 - 390	522.5975	1564.7707	1564.7674	2	0	K.LDLEAWFPGSGAFR.E		(Ions score 38)
377 - 390	522.5975	1564.7708	1564.7674	2	0	K.LDLEAWFPGSGAFR.E		(Ions score 42)
377 - 390	522.5976	1564.7709	1564.7674	2	0	K.LDLEAWFPGSGAFR.E		(Ions score 38)
377 - 390	783.3927	1564.7709	1564.7674	2	0	K.LDLEAWFPGSGAFR.E		(Ions score 40)
377 - 390	783.3928	1564.7710	1564.7674	2	0	K.LDLEAWFPGSGAFR.E		(Ions score 32)
377 - 390	783.3928	1564.7710	1564.7674	2	0	K.LDLEAWFPGSGAFR.E		(Ions score 51)
377 - 390	783.3928	1564.7711	1564.7674	2	0	K.LDLEAWFPGSGAFR.E		(Ions score 55)
377 - 390	783.3929	1564.7712	1564.7674	2	0	K.LDLEAWFPGSGAFR.E		(Ions score 43)
377 - 390	783.3929	1564.7712	1564.7674	2	0	K.LDLEAWFPGSGAFR.E		(Ions score 40)
377 - 390	522.5977	1564.7712	1564.7674	2	0	K.LDLEAWFPGSGAFR.E		(Ions score 40)
377 - 390	783.3930	1564.7715	1564.7674	3	0	K.LDLEAWFPGSGAFR.E		(Ions score 32)
377 - 390	783.3931	1564.7716	1564.7674	3	0	K.LDLEAWFPGSGAFR.E		(Ions score 30)
377 - 390	783.3931	1564.7717	1564.7674	3	0	K.LDLEAWFPGSGAFR.E		(Ions score 42)
377 - 390	783.3931	1564.7717	1564.7674	3	0	K.LDLEAWFPGSGAFR.E		(Ions score 27)
377 - 390	783.3931	1564.7717	1564.7674	3	0	K.LDLEAWFPGSGAFR.E		(Ions score 59)
377 - 390	783.3932	1564.7718	1564.7674	3	0	K.LDLEAWFPGSGAFR.E		(Ions score 42)

377 - 390	522.5979	1564.7719	1564.7674	3	0	K.LDLEAWFPGSGAFR.E	(Ions score 38)
377 - 390	783.3932	1564.7719	1564.7674	3	0	K.LDLEAWFPGSGAFR.E	(Ions score 59)
377 - 390	522.5979	1564.7719	1564.7674	3	0	K.LDLEAWFPGSGAFR.E	(Ions score 26)
377 - 390	783.3932	1564.7719	1564.7674	3	0	K.LDLEAWFPGSGAFR.E	(Ions score 30)
377 - 390	783.3933	1564.7720	1564.7674	3	0	K.LDLEAWFPGSGAFR.E	(Ions score 42)
377 - 390	783.3934	1564.7722	1564.7674	3	0	K.LDLEAWFPGSGAFR.E	(Ions score 44)
377 - 390	783.3934	1564.7722	1564.7674	3	0	K.LDLEAWFPGSGAFR.E	(Ions score 31)
377 - 390	522.5980	1564.7723	1564.7674	3	0	K.LDLEAWFPGSGAFR.E	(Ions score 26)
377 - 390	783.3935	1564.7724	1564.7674	3	0	K.LDLEAWFPGSGAFR.E	(Ions score 47)
377 - 390	522.5982	1564.7727	1564.7674	3	0	K.LDLEAWFPGSGAFR.E	(Ions score 25)
377 - 390	783.3936	1564.7727	1564.7674	3	0	K.LDLEAWFPGSGAFR.E	(Ions score 33)
377 - 390	783.3937	1564.7728	1564.7674	3	0	K.LDLEAWFPGSGAFR.E	(Ions score 43)
377 - 390	783.3937	1564.7728	1564.7674	3	0	K.LDLEAWFPGSGAFR.E	(Ions score 32)
377 - 390	783.3937	1564.7729	1564.7674	3	0	K.LDLEAWFPGSGAFR.E	(Ions score 43)
377 - 390	522.5983	1564.7732	1564.7674	4	0	K.LDLEAWFPGSGAFR.E	(Ions score 28)
377 - 390	783.3939	1564.7733	1564.7674	4	0	K.LDLEAWFPGSGAFR.E	(Ions score 64)
377 - 390	783.3941	1564.7736	1564.7674	4	0	K.LDLEAWFPGSGAFR.E	(Ions score 27)
377 - 390	783.3945	1564.7745	1564.7674	4	0	K.LDLEAWFPGSGAFR.E	(Ions score 28)
377 - 390	783.3945	1564.7745	1564.7674	5	0	K.LDLEAWFPGSGAFR.E	(Ions score 28)
377 - 390	783.3946	1564.7747	1564.7674	5	0	K.LDLEAWFPGSGAFR.E	(Ions score 52)
377 - 390	783.3946	1564.7747	1564.7674	5	0	K.LDLEAWFPGSGAFR.E	(Ions score 44)
377 - 390	783.3948	1564.7750	1564.7674	5	0	K.LDLEAWFPGSGAFR.E	(Ions score 40)
377 - 390	783.3949	1564.7752	1564.7674	5	0	K.LDLEAWFPGSGAFR.E	(Ions score 45)
391 - 404	568.2420	1701.7042	1701.7087	-3	0	R.ELVSCSNCTDYQAR.R	(Ions score 36)
391 - 404	851.8603	1701.7060	1701.7087	-2	0	R.ELVSCSNCTDYQAR.R	(Ions score 92)
391 - 404	851.8607	1701.7068	1701.7087	-1	0	R.ELVSCSNCTDYQAR.R	(Ions score 59)
391 - 404	851.8611	1701.7076	1701.7087	-1	0	R.ELVSCSNCTDYQAR.R	(Ions score 80)
391 - 404	851.8614	1701.7082	1701.7087	-0	0	R.ELVSCSNCTDYQAR.R	(Ions score 94)
391 - 404	851.8614	1701.7083	1701.7087	-0	0	R.ELVSCSNCTDYQAR.R	(Ions score 74)
391 - 404	851.8615	1701.7085	1701.7087	-0	0	R.ELVSCSNCTDYQAR.R	(Ions score 95)
391 - 404	851.8616	1701.7086	1701.7087	-0	0	R.ELVSCSNCTDYQAR.R	(Ions score 75)
391 - 404	851.8616	1701.7086	1701.7087	-0	0	R.ELVSCSNCTDYQAR.R	(Ions score 92)
391 - 404	851.8616	1701.7087	1701.7087	0	0	R.ELVSCSNCTDYQAR.R	(Ions score 95)
391 - 404	851.8617	1701.7088	1701.7087	0	0	R.ELVSCSNCTDYQAR.R	(Ions score 81)
391 - 404	851.8618	1701.7090	1701.7087	0	0	R.ELVSCSNCTDYQAR.R	(Ions score 79)
391 - 404	1702.7166	1701.7093	1701.7087	0	0	R.ELVSCSNCTDYQAR.R	(Ions score 76)
391 - 404	851.8621	1701.7096	1701.7087	1	0	R.ELVSCSNCTDYQAR.R	(Ions score 79)
391 - 404	1702.7172	1701.7099	1701.7087	1	0	R.ELVSCSNCTDYQAR.R	(Ions score 93)
391 - 404	851.8622	1701.7099	1701.7087	1	0	R.ELVSCSNCTDYQAR.R	(Ions score 92)
391 - 404	851.8623	1701.7100	1701.7087	1	0	R.ELVSCSNCTDYQAR.R	(Ions score 52)
391 - 404	851.8623	1701.7101	1701.7087	1	0	R.ELVSCSNCTDYQAR.R	(Ions score 89)
391 - 404	851.8624	1701.7102	1701.7087	1	0	R.ELVSCSNCTDYQAR.R	(Ions score 81)
391 - 404	851.8624	1701.7103	1701.7087	1	0	R.ELVSCSNCTDYQAR.R	(Ions score 72)
391 - 404	851.8624	1701.7103	1701.7087	1	0	R.ELVSCSNCTDYQAR.R	(Ions score 94)
391 - 404	851.8625	1701.7104	1701.7087	1	0	R.ELVSCSNCTDYQAR.R	(Ions score 69)
391 - 404	851.8625	1701.7104	1701.7087	1	0	R.ELVSCSNCTDYQAR.R	(Ions score 85)
391 - 404	1702.7177	1701.7104	1701.7087	1	0	R.ELVSCSNCTDYQAR.R	(Ions score 93)
391 - 404	851.8625	1701.7104	1701.7087	1	0	R.ELVSCSNCTDYQAR.R	(Ions score 92)
391 - 404	851.8625	1701.7104	1701.7087	1	0	R.ELVSCSNCTDYQAR.R	(Ions score 82)
391 - 404	851.8625	1701.7105	1701.7087	1	0	R.ELVSCSNCTDYQAR.R	(Ions score 92)
391 - 404	851.8626	1701.7106	1701.7087	1	0	R.ELVSCSNCTDYQAR.R	(Ions score 91)
391 - 404	851.8626	1701.7106	1701.7087	1	0	R.ELVSCSNCTDYQAR.R	(Ions score 92)
391 - 404	568.2442	1701.7107	1701.7087	1	0	R.ELVSCSNCTDYQAR.R	(Ions score 41)
391 - 404	568.2442	1701.7107	1701.7087	1	0	R.ELVSCSNCTDYQAR.R	(Ions score 44)
391 - 404	851.8626	1701.7107	1701.7087	1	0	R.ELVSCSNCTDYQAR.R	(Ions score 68)
391 - 404	568.2442	1701.7107	1701.7087	1	0	R.ELVSCSNCTDYQAR.R	(Ions score 61)
391 - 404	851.8626	1701.7107	1701.7087	1	0	R.ELVSCSNCTDYQAR.R	(Ions score 54)

391 - 404	851.8627	1701.7108	1701.7087	1	0	R.ELVSCSNCTDYQAR.R	(Ions score 86)
391 - 404	851.8627	1701.7108	1701.7087	1	0	R.ELVSCSNCTDYQAR.R	(Ions score 38)
391 - 404	851.8627	1701.7108	1701.7087	1	0	R.ELVSCSNCTDYQAR.R	(Ions score 89)
391 - 404	851.8627	1701.7108	1701.7087	1	0	R.ELVSCSNCTDYQAR.R	(Ions score 51)
391 - 404	568.2442	1701.7109	1701.7087	1	0	R.ELVSCSNCTDYQAR.R	(Ions score 47)
391 - 404	851.8627	1701.7109	1701.7087	1	0	R.ELVSCSNCTDYQAR.R	(Ions score 40)
391 - 404	851.8628	1701.7110	1701.7087	1	0	R.ELVSCSNCTDYQAR.R	(Ions score 94)
391 - 404	851.8628	1701.7110	1701.7087	1	0	R.ELVSCSNCTDYQAR.R	(Ions score 89)
391 - 404	1702.7184	1701.7111	1701.7087	1	0	R.ELVSCSNCTDYQAR.R	(Ions score 99)
391 - 404	568.2443	1701.7111	1701.7087	1	0	R.ELVSCSNCTDYQAR.R	(Ions score 39)
391 - 404	851.8628	1701.7111	1701.7087	1	0	R.ELVSCSNCTDYQAR.R	(Ions score 95)
391 - 404	851.8629	1701.7112	1701.7087	1	0	R.ELVSCSNCTDYQAR.R	(Ions score 70)
391 - 404	851.8629	1701.7112	1701.7087	1	0	R.ELVSCSNCTDYQAR.R	(Ions score 78)
391 - 404	568.2443	1701.7112	1701.7087	1	0	R.ELVSCSNCTDYQAR.R	(Ions score 37)
391 - 404	1702.7185	1701.7112	1701.7087	2	0	R.ELVSCSNCTDYQAR.R	(Ions score 88)
391 - 404	851.8629	1701.7113	1701.7087	2	0	R.ELVSCSNCTDYQAR.R	(Ions score 101)
391 - 404	568.2444	1701.7113	1701.7087	2	0	R.ELVSCSNCTDYQAR.R	(Ions score 57)
391 - 404	568.2444	1701.7113	1701.7087	2	0	R.ELVSCSNCTDYQAR.R	(Ions score 56)
391 - 404	568.2444	1701.7114	1701.7087	2	0	R.ELVSCSNCTDYQAR.R	(Ions score 41)
391 - 404	568.2444	1701.7114	1701.7087	2	0	R.ELVSCSNCTDYQAR.R	(Ions score 46)
391 - 404	851.8630	1701.7115	1701.7087	2	0	R.ELVSCSNCTDYQAR.R	(Ions score 101)
391 - 404	851.8630	1701.7115	1701.7087	2	0	R.ELVSCSNCTDYQAR.R	(Ions score 72)
391 - 404	1702.7188	1701.7115	1701.7087	2	0	R.ELVSCSNCTDYQAR.R	(Ions score 81)
391 - 404	1702.7188	1701.7115	1701.7087	2	0	R.ELVSCSNCTDYQAR.R	(Ions score 93)
391 - 404	568.2444	1701.7115	1701.7087	2	0	R.ELVSCSNCTDYQAR.R	(Ions score 43)
391 - 404	568.2445	1701.7116	1701.7087	2	0	R.ELVSCSNCTDYQAR.R	(Ions score 48)
391 - 404	851.8631	1701.7116	1701.7087	2	0	R.ELVSCSNCTDYQAR.R	(Ions score 94)
391 - 404	851.8631	1701.7117	1701.7087	2	0	R.ELVSCSNCTDYQAR.R	(Ions score 69)
391 - 404	851.8631	1701.7117	1701.7087	2	0	R.ELVSCSNCTDYQAR.R	(Ions score 94)
391 - 404	851.8632	1701.7117	1701.7087	2	0	R.ELVSCSNCTDYQAR.R	(Ions score 48)
391 - 404	851.8632	1701.7118	1701.7087	2	0	R.ELVSCSNCTDYQAR.R	(Ions score 95)
391 - 404	568.2445	1701.7118	1701.7087	2	0	R.ELVSCSNCTDYQAR.R	(Ions score 57)
391 - 404	568.2446	1701.7118	1701.7087	2	0	R.ELVSCSNCTDYQAR.R	(Ions score 36)
391 - 404	568.2446	1701.7118	1701.7087	2	0	R.ELVSCSNCTDYQAR.R	(Ions score 49)
391 - 404	851.8632	1701.7119	1701.7087	2	0	R.ELVSCSNCTDYQAR.R	(Ions score 78)
391 - 404	568.2446	1701.7119	1701.7087	2	0	R.ELVSCSNCTDYQAR.R	(Ions score 45)
391 - 404	568.2446	1701.7119	1701.7087	2	0	R.ELVSCSNCTDYQAR.R	(Ions score 46)
391 - 404	568.2446	1701.7119	1701.7087	2	0	R.ELVSCSNCTDYQAR.R	(Ions score 54)
391 - 404	851.8632	1701.7119	1701.7087	2	0	R.ELVSCSNCTDYQAR.R	(Ions score 67)
391 - 404	851.8633	1701.7120	1701.7087	2	0	R.ELVSCSNCTDYQAR.R	(Ions score 76)
391 - 404	851.8633	1701.7120	1701.7087	2	0	R.ELVSCSNCTDYQAR.R	(Ions score 95)
391 - 404	1702.7192	1701.7120	1701.7087	2	0	R.ELVSCSNCTDYQAR.R	(Ions score 74)
391 - 404	568.2446	1701.7120	1701.7087	2	0	R.ELVSCSNCTDYQAR.R	(Ions score 45)
391 - 404	851.8633	1701.7120	1701.7087	2	0	R.ELVSCSNCTDYQAR.R	(Ions score 72)
391 - 404	851.8633	1701.7120	1701.7087	2	0	R.ELVSCSNCTDYQAR.R	(Ions score 61)
391 - 404	851.8634	1701.7123	1701.7087	2	0	R.ELVSCSNCTDYQAR.R	(Ions score 81)
391 - 404	851.8634	1701.7123	1701.7087	2	0	R.ELVSCSNCTDYQAR.R	(Ions score 91)
391 - 404	1702.7196	1701.7123	1701.7087	2	0	R.ELVSCSNCTDYQAR.R	(Ions score 62)
391 - 404	851.8635	1701.7124	1701.7087	2	0	R.ELVSCSNCTDYQAR.R	(Ions score 72)
391 - 404	1702.7197	1701.7125	1701.7087	2	0	R.ELVSCSNCTDYQAR.R	(Ions score 76)
391 - 404	851.8635	1701.7125	1701.7087	2	0	R.ELVSCSNCTDYQAR.R	(Ions score 90)
391 - 404	851.8636	1701.7125	1701.7087	2	0	R.ELVSCSNCTDYQAR.R	(Ions score 59)
391 - 404	851.8636	1701.7126	1701.7087	2	0	R.ELVSCSNCTDYQAR.R	(Ions score 82)
391 - 404	851.8636	1701.7126	1701.7087	2	0	R.ELVSCSNCTDYQAR.R	(Ions score 82)
391 - 404	851.8636	1701.7126	1701.7087	2	0	R.ELVSCSNCTDYQAR.R	(Ions score 77)
391 - 404	851.8636	1701.7127	1701.7087	2	0	R.ELVSCSNCTDYQAR.R	(Ions score 95)
391 - 404	851.8636	1701.7127	1701.7087	2	0	R.ELVSCSNCTDYQAR.R	(Ions score 96)

391 - 404	568.2448	1701.7127	1701.7087	2	0	R.ELVSCSNCTDYQAR.R	(Ions score 51)
391 - 404	1702.7201	1701.7128	1701.7087	2	0	R.ELVSCSNCTDYQAR.R	(Ions score 90)
391 - 404	851.8637	1701.7129	1701.7087	2	0	R.ELVSCSNCTDYQAR.R	(Ions score 95)
391 - 404	851.8637	1701.7129	1701.7087	3	0	R.ELVSCSNCTDYQAR.R	(Ions score 55)
391 - 404	568.2449	1701.7130	1701.7087	3	0	R.ELVSCSNCTDYQAR.R	(Ions score 45)
391 - 404	1702.7203	1701.7131	1701.7087	3	0	R.ELVSCSNCTDYQAR.R	(Ions score 71)
391 - 404	851.8638	1701.7131	1701.7087	3	0	R.ELVSCSNCTDYQAR.R	(Ions score 77)
391 - 404	851.8639	1701.7132	1701.7087	3	0	R.ELVSCSNCTDYQAR.R	(Ions score 81)
391 - 404	851.8639	1701.7133	1701.7087	3	0	R.ELVSCSNCTDYQAR.R	(Ions score 92)
391 - 404	851.8639	1701.7133	1701.7087	3	0	R.ELVSCSNCTDYQAR.R	(Ions score 80)
391 - 404	851.8640	1701.7134	1701.7087	3	0	R.ELVSCSNCTDYQAR.R	(Ions score 95)
391 - 404	851.8640	1701.7134	1701.7087	3	0	R.ELVSCSNCTDYQAR.R	(Ions score 76)
391 - 404	851.8640	1701.7134	1701.7087	3	0	R.ELVSCSNCTDYQAR.R	(Ions score 56)
391 - 404	851.8640	1701.7135	1701.7087	3	0	R.ELVSCSNCTDYQAR.R	(Ions score 24)
391 - 404	851.8640	1701.7135	1701.7087	3	0	R.ELVSCSNCTDYQAR.R	(Ions score 89)
391 - 404	851.8641	1701.7136	1701.7087	3	0	R.ELVSCSNCTDYQAR.R	(Ions score 95)
391 - 404	851.8641	1701.7136	1701.7087	3	0	R.ELVSCSNCTDYQAR.R	(Ions score 79)
391 - 404	851.8641	1701.7136	1701.7087	3	0	R.ELVSCSNCTDYQAR.R	(Ions score 82)
391 - 404	851.8641	1701.7136	1701.7087	3	0	R.ELVSCSNCTDYQAR.R	(Ions score 84)
391 - 404	851.8642	1701.7138	1701.7087	3	0	R.ELVSCSNCTDYQAR.R	(Ions score 95)
391 - 404	851.8642	1701.7138	1701.7087	3	0	R.ELVSCSNCTDYQAR.R	(Ions score 81)
391 - 404	851.8642	1701.7139	1701.7087	3	0	R.ELVSCSNCTDYQAR.R	(Ions score 91)
391 - 404	851.8644	1701.7142	1701.7087	3	0	R.ELVSCSNCTDYQAR.R	(Ions score 95)
391 - 404	851.8644	1701.7142	1701.7087	3	0	R.ELVSCSNCTDYQAR.R	(Ions score 52)
391 - 404	851.8644	1701.7143	1701.7087	3	0	R.ELVSCSNCTDYQAR.R	(Ions score 84)
391 - 404	851.8644	1701.7143	1701.7087	3	0	R.ELVSCSNCTDYQAR.R	(Ions score 60)
391 - 404	851.8645	1701.7144	1701.7087	3	0	R.ELVSCSNCTDYQAR.R	(Ions score 95)
391 - 404	851.8645	1701.7144	1701.7087	3	0	R.ELVSCSNCTDYQAR.R	(Ions score 82)
391 - 404	851.8645	1701.7145	1701.7087	3	0	R.ELVSCSNCTDYQAR.R	(Ions score 80)
391 - 404	851.8645	1701.7145	1701.7087	3	0	R.ELVSCSNCTDYQAR.R	(Ions score 77)
391 - 404	851.8645	1701.7145	1701.7087	3	0	R.ELVSCSNCTDYQAR.R	(Ions score 29)
391 - 404	851.8646	1701.7146	1701.7087	3	0	R.ELVSCSNCTDYQAR.R	(Ions score 72)
391 - 404	851.8646	1701.7146	1701.7087	3	0	R.ELVSCSNCTDYQAR.R	(Ions score 92)
391 - 404	851.8646	1701.7147	1701.7087	4	0	R.ELVSCSNCTDYQAR.R	(Ions score 77)
391 - 404	851.8647	1701.7148	1701.7087	4	0	R.ELVSCSNCTDYQAR.R	(Ions score 98)
391 - 404	851.8647	1701.7148	1701.7087	4	0	R.ELVSCSNCTDYQAR.R	(Ions score 84)
391 - 404	851.8647	1701.7148	1701.7087	4	0	R.ELVSCSNCTDYQAR.R	(Ions score 93)
391 - 404	851.8647	1701.7148	1701.7087	4	0	R.ELVSCSNCTDYQAR.R	(Ions score 72)
391 - 404	851.8647	1701.7149	1701.7087	4	0	R.ELVSCSNCTDYQAR.R	(Ions score 77)
391 - 404	851.8647	1701.7149	1701.7087	4	0	R.ELVSCSNCTDYQAR.R	(Ions score 87)
391 - 404	851.8648	1701.7150	1701.7087	4	0	R.ELVSCSNCTDYQAR.R	(Ions score 95)
391 - 404	851.8648	1701.7150	1701.7087	4	0	R.ELVSCSNCTDYQAR.R	(Ions score 76)
391 - 404	851.8648	1701.7150	1701.7087	4	0	R.ELVSCSNCTDYQAR.R	(Ions score 94)
391 - 404	851.8648	1701.7151	1701.7087	4	0	R.ELVSCSNCTDYQAR.R	(Ions score 30)
391 - 404	851.8648	1701.7151	1701.7087	4	0	R.ELVSCSNCTDYQAR.R	(Ions score 89)
391 - 404	851.8648	1701.7151	1701.7087	4	0	R.ELVSCSNCTDYQAR.R	(Ions score 55)
391 - 404	851.8649	1701.7152	1701.7087	4	0	R.ELVSCSNCTDYQAR.R	(Ions score 42)
391 - 404	851.8649	1701.7152	1701.7087	4	0	R.ELVSCSNCTDYQAR.R	(Ions score 91)
391 - 404	851.8649	1701.7152	1701.7087	4	0	R.ELVSCSNCTDYQAR.R	(Ions score 88)
391 - 404	851.8649	1701.7153	1701.7087	4	0	R.ELVSCSNCTDYQAR.R	(Ions score 82)
391 - 404	851.8649	1701.7153	1701.7087	4	0	R.ELVSCSNCTDYQAR.R	(Ions score 73)
391 - 404	851.8649	1701.7153	1701.7087	4	0	R.ELVSCSNCTDYQAR.R	(Ions score 73)
391 - 404	851.8651	1701.7157	1701.7087	4	0	R.ELVSCSNCTDYQAR.R	(Ions score 63)
391 - 404	568.2459	1701.7157	1701.7087	4	0	R.ELVSCSNCTDYQAR.R	(Ions score 37)
391 - 404	851.8652	1701.7158	1701.7087	4	0	R.ELVSCSNCTDYQAR.R	(Ions score 95)
391 - 404	851.8652	1701.7158	1701.7087	4	0	R.ELVSCSNCTDYQAR.R	(Ions score 56)
391 - 404	851.8652	1701.7159	1701.7087	4	0	R.ELVSCSNCTDYQAR.R	(Ions score 83)

391 - 404	851.8652	1701.7159	1701.7087	4	0	R.ELVSCSNCTDYQAR.R	(Ions score 77)
391 - 404	851.8652	1701.7159	1701.7087	4	0	R.ELVSCSNCTDYQAR.R	(Ions score 85)
391 - 404	851.8652	1701.7159	1701.7087	4	0	R.ELVSCSNCTDYQAR.R	(Ions score 55)
391 - 404	851.8653	1701.7161	1701.7087	4	0	R.ELVSCSNCTDYQAR.R	(Ions score 64)
391 - 404	851.8653	1701.7161	1701.7087	4	0	R.ELVSCSNCTDYQAR.R	(Ions score 73)
391 - 404	851.8653	1701.7161	1701.7087	4	0	R.ELVSCSNCTDYQAR.R	(Ions score 77)
391 - 404	851.8654	1701.7162	1701.7087	4	0	R.ELVSCSNCTDYQAR.R	(Ions score 80)
391 - 404	851.8654	1701.7163	1701.7087	5	0	R.ELVSCSNCTDYQAR.R	(Ions score 98)
391 - 404	851.8654	1701.7163	1701.7087	5	0	R.ELVSCSNCTDYQAR.R	(Ions score 56)
391 - 404	851.8655	1701.7164	1701.7087	5	0	R.ELVSCSNCTDYQAR.R	(Ions score 27)
391 - 404	851.8655	1701.7164	1701.7087	5	0	R.ELVSCSNCTDYQAR.R	(Ions score 82)
391 - 404	851.8655	1701.7165	1701.7087	5	0	R.ELVSCSNCTDYQAR.R	(Ions score 77)
391 - 404	851.8655	1701.7165	1701.7087	5	0	R.ELVSCSNCTDYQAR.R	(Ions score 22)
391 - 404	851.8655	1701.7165	1701.7087	5	0	R.ELVSCSNCTDYQAR.R	(Ions score 86)
391 - 404	851.8656	1701.7166	1701.7087	5	0	R.ELVSCSNCTDYQAR.R	(Ions score 56)
391 - 404	851.8656	1701.7166	1701.7087	5	0	R.ELVSCSNCTDYQAR.R	(Ions score 98)
391 - 404	851.8656	1701.7167	1701.7087	5	0	R.ELVSCSNCTDYQAR.R	(Ions score 39)
391 - 404	851.8656	1701.7167	1701.7087	5	0	R.ELVSCSNCTDYQAR.R	(Ions score 90)
391 - 404	851.8656	1701.7167	1701.7087	5	0	R.ELVSCSNCTDYQAR.R	(Ions score 95)
391 - 404	851.8656	1701.7167	1701.7087	5	0	R.ELVSCSNCTDYQAR.R	(Ions score 83)
391 - 404	568.2462	1701.7168	1701.7087	5	0	R.ELVSCSNCTDYQAR.R	(Ions score 45)
391 - 404	851.8657	1701.7168	1701.7087	5	0	R.ELVSCSNCTDYQAR.R	(Ions score 88)
391 - 404	851.8657	1701.7168	1701.7087	5	0	R.ELVSCSNCTDYQAR.R	(Ions score 83)
391 - 404	851.8657	1701.7168	1701.7087	5	0	R.ELVSCSNCTDYQAR.R	(Ions score 56)
391 - 404	851.8657	1701.7168	1701.7087	5	0	R.ELVSCSNCTDYQAR.R	(Ions score 95)
391 - 404	851.8657	1701.7169	1701.7087	5	0	R.ELVSCSNCTDYQAR.R	(Ions score 76)
391 - 404	851.8657	1701.7169	1701.7087	5	0	R.ELVSCSNCTDYQAR.R	(Ions score 92)
391 - 404	851.8658	1701.7170	1701.7087	5	0	R.ELVSCSNCTDYQAR.R	(Ions score 77)
391 - 404	851.8658	1701.7171	1701.7087	5	0	R.ELVSCSNCTDYQAR.R	(Ions score 95)
391 - 404	851.8659	1701.7172	1701.7087	5	0	R.ELVSCSNCTDYQAR.R	(Ions score 42)
410 - 414	596.3039	595.2966	595.2966	0	0	R.YGQTK.K	(Ions score 21)
410 - 414	596.3039	595.2966	595.2966	0	0	R.YGQTK.K	(Ions score 23)
410 - 414	596.3039	595.2966	595.2966	0	0	R.YGQTK.K	(Ions score 22)
410 - 414	596.3039	595.2966	595.2966	0	0	R.YGQTK.K	(Ions score 21)
410 - 414	596.3039	595.2966	595.2966	0	0	R.YGQTK.K	(Ions score 22)
410 - 414	596.3039	595.2966	595.2966	0	0	R.YGQTK.K	(Ions score 21)
410 - 414	596.3039	595.2966	595.2966	0	0	R.YGQTK.K	(Ions score 21)
410 - 414	596.3055	595.2983	595.2966	3	0	R.YGQTK.K	(Ions score 23)
416 - 435	796.0300	2385.0683	2385.0772	-4	1	K.MMDKVEFVHMLNATMCATTR.T	(Ions score 25)
416 - 435	597.2745	2385.0688	2385.0772	-4	1	K.MMDKVEFVHMLNATMCATTR.T	(Ions score 62)
416 - 435	796.0306	2385.0699	2385.0772	-3	1	K.MMDKVEFVHMLNATMCATTR.T	(Ions score 38)
416 - 435	796.0309	2385.0708	2385.0772	-3	1	K.MMDKVEFVHMLNATMCATTR.T	(Ions score 30)
416 - 435	796.0313	2385.0720	2385.0772	-2	1	K.MMDKVEFVHMLNATMCATTR.T	(Ions score 48)
416 - 435	796.0313	2385.0721	2385.0772	-2	1	K.MMDKVEFVHMLNATMCATTR.T	(Ions score 56)
416 - 435	796.0314	2385.0725	2385.0772	-2	1	K.MMDKVEFVHMLNATMCATTR.T	(Ions score 47)
416 - 435	796.0319	2385.0740	2385.0772	-1	1	K.MMDKVEFVHMLNATMCATTR.T	(Ions score 22)
416 - 435	597.2758	2385.0741	2385.0772	-1	1	K.MMDKVEFVHMLNATMCATTR.T	(Ions score 25)
416 - 435	796.0321	2385.0745	2385.0772	-1	1	K.MMDKVEFVHMLNATMCATTR.T	(Ions score 45)
416 - 435	796.0322	2385.0749	2385.0772	-1	1	K.MMDKVEFVHMLNATMCATTR.T	(Ions score 29)
416 - 435	796.0324	2385.0753	2385.0772	-1	1	K.MMDKVEFVHMLNATMCATTR.T	(Ions score 30)
416 - 435	597.2762	2385.0757	2385.0772	-1	1	K.MMDKVEFVHMLNATMCATTR.T	(Ions score 57)
416 - 435	796.0325	2385.0758	2385.0772	-1	1	K.MMDKVEFVHMLNATMCATTR.T	(Ions score 54)
416 - 435	796.0328	2385.0765	2385.0772	-0	1	K.MMDKVEFVHMLNATMCATTR.T	(Ions score 21)
416 - 435	597.2764	2385.0766	2385.0772	-0	1	K.MMDKVEFVHMLNATMCATTR.T	(Ions score 45)
416 - 435	796.0329	2385.0768	2385.0772	-0	1	K.MMDKVEFVHMLNATMCATTR.T	(Ions score 60)
416 - 435	597.2765	2385.0769	2385.0772	-0	1	K.MMDKVEFVHMLNATMCATTR.T	(Ions score 53)
416 - 435	597.2767	2385.0776	2385.0772	0	1	K.MMDKVEFVHMLNATMCATTR.T	(Ions score 30)

416 - 435	796.0332	2385.0777	2385.0772	0	1	K.MMDKVEFVHMLNATMCATTR.T	(Ions score 45)
416 - 435	597.2767	2385.0777	2385.0772	0	1	K.MMDKVEFVHMLNATMCATTR.T	(Ions score 38)
416 - 435	796.0333	2385.0780	2385.0772	0	1	K.MMDKVEFVHMLNATMCATTR.T	(Ions score 44)
416 - 435	796.0333	2385.0781	2385.0772	0	1	K.MMDKVEFVHMLNATMCATTR.T	(Ions score 34)
416 - 435	796.0336	2385.0791	2385.0772	1	1	K.MMDKVEFVHMLNATMCATTR.T	(Ions score 26)
416 - 435	597.2771	2385.0791	2385.0772	1	1	K.MMDKVEFVHMLNATMCATTR.T	(Ions score 42)
416 - 435	796.0337	2385.0792	2385.0772	1	1	K.MMDKVEFVHMLNATMCATTR.T	(Ions score 38)
416 - 435	796.0337	2385.0792	2385.0772	1	1	K.MMDKVEFVHMLNATMCATTR.T	(Ions score 38)
416 - 435	796.0338	2385.0795	2385.0772	1	1	K.MMDKVEFVHMLNATMCATTR.T	(Ions score 28)
416 - 435	796.0338	2385.0796	2385.0772	1	1	K.MMDKVEFVHMLNATMCATTR.T	(Ions score 29)
416 - 435	597.2772	2385.0796	2385.0772	1	1	K.MMDKVEFVHMLNATMCATTR.T	(Ions score 35)
416 - 435	597.2773	2385.0799	2385.0772	1	1	K.MMDKVEFVHMLNATMCATTR.T	(Ions score 36)
416 - 435	796.0339	2385.0799	2385.0772	1	1	K.MMDKVEFVHMLNATMCATTR.T	(Ions score 41)
416 - 435	796.0340	2385.0801	2385.0772	1	1	K.MMDKVEFVHMLNATMCATTR.T	(Ions score 40)
416 - 435	597.2773	2385.0802	2385.0772	1	1	K.MMDKVEFVHMLNATMCATTR.T	(Ions score 24)
416 - 435	796.0341	2385.0805	2385.0772	1	1	K.MMDKVEFVHMLNATMCATTR.T	(Ions score 43)
416 - 435	796.0342	2385.0807	2385.0772	1	1	K.MMDKVEFVHMLNATMCATTR.T	(Ions score 25)
416 - 435	597.2774	2385.0807	2385.0772	1	1	K.MMDKVEFVHMLNATMCATTR.T	(Ions score 44)
416 - 435	796.0342	2385.0809	2385.0772	2	1	K.MMDKVEFVHMLNATMCATTR.T	(Ions score 39)
416 - 435	597.2775	2385.0811	2385.0772	2	1	K.MMDKVEFVHMLNATMCATTR.T	(Ions score 57)
416 - 435	597.2776	2385.0813	2385.0772	2	1	K.MMDKVEFVHMLNATMCATTR.T	(Ions score 45)
416 - 435	796.0344	2385.0813	2385.0772	2	1	K.MMDKVEFVHMLNATMCATTR.T	(Ions score 26)
416 - 435	597.2776	2385.0815	2385.0772	2	1	K.MMDKVEFVHMLNATMCATTR.T	(Ions score 58)
416 - 435	597.2777	2385.0815	2385.0772	2	1	K.MMDKVEFVHMLNATMCATTR.T	(Ions score 31)
416 - 435	597.2777	2385.0815	2385.0772	2	1	K.MMDKVEFVHMLNATMCATTR.T	(Ions score 43)
416 - 435	796.0345	2385.0816	2385.0772	2	1	K.MMDKVEFVHMLNATMCATTR.T	(Ions score 39)
416 - 435	597.2777	2385.0817	2385.0772	2	1	K.MMDKVEFVHMLNATMCATTR.T	(Ions score 45)
416 - 435	597.2777	2385.0817	2385.0772	2	1	K.MMDKVEFVHMLNATMCATTR.T	(Ions score 32)
416 - 435	597.2777	2385.0818	2385.0772	2	1	K.MMDKVEFVHMLNATMCATTR.T	(Ions score 42)
416 - 435	796.0345	2385.0818	2385.0772	2	1	K.MMDKVEFVHMLNATMCATTR.T	(Ions score 40)
416 - 435	597.2777	2385.0818	2385.0772	2	1	K.MMDKVEFVHMLNATMCATTR.T	(Ions score 53)
416 - 435	597.2778	2385.0819	2385.0772	2	1	K.MMDKVEFVHMLNATMCATTR.T	(Ions score 53)
416 - 435	796.0346	2385.0819	2385.0772	2	1	K.MMDKVEFVHMLNATMCATTR.T	(Ions score 77)
416 - 435	597.2778	2385.0819	2385.0772	2	1	K.MMDKVEFVHMLNATMCATTR.T	(Ions score 29)
416 - 435	597.2778	2385.0820	2385.0772	2	1	K.MMDKVEFVHMLNATMCATTR.T	(Ions score 43)
416 - 435	597.2778	2385.0820	2385.0772	2	1	K.MMDKVEFVHMLNATMCATTR.T	(Ions score 32)
416 - 435	597.2778	2385.0821	2385.0772	2	1	K.MMDKVEFVHMLNATMCATTR.T	(Ions score 35)
416 - 435	796.0347	2385.0822	2385.0772	2	1	K.MMDKVEFVHMLNATMCATTR.T	(Ions score 39)
416 - 435	796.0347	2385.0824	2385.0772	2	1	K.MMDKVEFVHMLNATMCATTR.T	(Ions score 92)
416 - 435	796.0348	2385.0824	2385.0772	2	1	K.MMDKVEFVHMLNATMCATTR.T	(Ions score 61)
416 - 435	597.2779	2385.0825	2385.0772	2	1	K.MMDKVEFVHMLNATMCATTR.T	(Ions score 41)
416 - 435	1193.5485	2385.0825	2385.0772	2	1	K.MMDKVEFVHMLNATMCATTR.T	(Ions score 82)
416 - 435	597.2779	2385.0825	2385.0772	2	1	K.MMDKVEFVHMLNATMCATTR.T	(Ions score 45)
416 - 435	796.0348	2385.0827	2385.0772	2	1	K.MMDKVEFVHMLNATMCATTR.T	(Ions score 57)
416 - 435	597.2780	2385.0827	2385.0772	2	1	K.MMDKVEFVHMLNATMCATTR.T	(Ions score 58)
416 - 435	796.0349	2385.0828	2385.0772	2	1	K.MMDKVEFVHMLNATMCATTR.T	(Ions score 49)
416 - 435	597.2780	2385.0828	2385.0772	2	1	K.MMDKVEFVHMLNATMCATTR.T	(Ions score 32)
416 - 435	597.2780	2385.0828	2385.0772	2	1	K.MMDKVEFVHMLNATMCATTR.T	(Ions score 45)
416 - 435	597.2780	2385.0829	2385.0772	2	1	K.MMDKVEFVHMLNATMCATTR.T	(Ions score 41)
416 - 435	597.2780	2385.0829	2385.0772	2	1	K.MMDKVEFVHMLNATMCATTR.T	(Ions score 45)
416 - 435	597.2781	2385.0831	2385.0772	3	1	K.MMDKVEFVHMLNATMCATTR.T	(Ions score 35)
416 - 435	597.2781	2385.0833	2385.0772	3	1	K.MMDKVEFVHMLNATMCATTR.T	(Ions score 25)
416 - 435	597.2781	2385.0834	2385.0772	3	1	K.MMDKVEFVHMLNATMCATTR.T	(Ions score 53)
416 - 435	597.2782	2385.0835	2385.0772	3	1	K.MMDKVEFVHMLNATMCATTR.T	(Ions score 46)
416 - 435	597.2782	2385.0835	2385.0772	3	1	K.MMDKVEFVHMLNATMCATTR.T	(Ions score 32)
416 - 435	597.2782	2385.0835	2385.0772	3	1	K.MMDKVEFVHMLNATMCATTR.T	(Ions score 35)
416 - 435	796.0352	2385.0836	2385.0772	3	1	K.MMDKVEFVHMLNATMCATTR.T	(Ions score 72)

416 - 435	597.2782	2385.0838	2385.0772	3	1	K.MMDKVEFVHMLNATMCATTR.T		(Ions score 58)
416 - 435	597.2782	2385.0839	2385.0772	3	1	K.MMDKVEFVHMLNATMCATTR.T		(Ions score 40)
416 - 435	597.2783	2385.0839	2385.0772	3	1	K.MMDKVEFVHMLNATMCATTR.T		(Ions score 42)
416 - 435	597.2783	2385.0840	2385.0772	3	1	K.MMDKVEFVHMLNATMCATTR.T		(Ions score 45)
416 - 435	796.0353	2385.0840	2385.0772	3	1	K.MMDKVEFVHMLNATMCATTR.T		(Ions score 46)
416 - 435	597.2783	2385.0841	2385.0772	3	1	K.MMDKVEFVHMLNATMCATTR.T		(Ions score 30)
416 - 435	597.2783	2385.0841	2385.0772	3	1	K.MMDKVEFVHMLNATMCATTR.T		(Ions score 34)
416 - 435	796.0353	2385.0842	2385.0772	3	1	K.MMDKVEFVHMLNATMCATTR.T		(Ions score 81)
416 - 435	597.2783	2385.0842	2385.0772	3	1	K.MMDKVEFVHMLNATMCATTR.T		(Ions score 23)
416 - 435	796.0354	2385.0842	2385.0772	3	1	K.MMDKVEFVHMLNATMCATTR.T		(Ions score 39)
416 - 435	796.0354	2385.0844	2385.0772	3	1	K.MMDKVEFVHMLNATMCATTR.T		(Ions score 50)
416 - 435	597.2784	2385.0844	2385.0772	3	1	K.MMDKVEFVHMLNATMCATTR.T		(Ions score 44)
416 - 435	597.2784	2385.0844	2385.0772	3	1	K.MMDKVEFVHMLNATMCATTR.T		(Ions score 46)
416 - 435	597.2784	2385.0846	2385.0772	3	1	K.MMDKVEFVHMLNATMCATTR.T		(Ions score 36)
416 - 435	796.0355	2385.0848	2385.0772	3	1	K.MMDKVEFVHMLNATMCATTR.T		(Ions score 72)
416 - 435	597.2785	2385.0849	2385.0772	3	1	K.MMDKVEFVHMLNATMCATTR.T		(Ions score 45)
416 - 435	597.2785	2385.0849	2385.0772	3	1	K.MMDKVEFVHMLNATMCATTR.T		(Ions score 38)
416 - 435	597.2785	2385.0850	2385.0772	3	1	K.MMDKVEFVHMLNATMCATTR.T		(Ions score 38)
416 - 435	597.2785	2385.0850	2385.0772	3	1	K.MMDKVEFVHMLNATMCATTR.T		(Ions score 58)
416 - 435	597.2786	2385.0852	2385.0772	3	1	K.MMDKVEFVHMLNATMCATTR.T		(Ions score 35)
416 - 435	597.2786	2385.0852	2385.0772	3	1	K.MMDKVEFVHMLNATMCATTR.T		(Ions score 32)
416 - 435	597.2786	2385.0854	2385.0772	3	1	K.MMDKVEFVHMLNATMCATTR.T		(Ions score 42)
416 - 435	796.0358	2385.0857	2385.0772	4	1	K.MMDKVEFVHMLNATMCATTR.T		(Ions score 57)
416 - 435	597.2787	2385.0857	2385.0772	4	1	K.MMDKVEFVHMLNATMCATTR.T		(Ions score 40)
416 - 435	597.2787	2385.0857	2385.0772	4	1	K.MMDKVEFVHMLNATMCATTR.T		(Ions score 42)
416 - 435	597.2787	2385.0859	2385.0772	4	1	K.MMDKVEFVHMLNATMCATTR.T		(Ions score 43)
416 - 435	597.2787	2385.0859	2385.0772	4	1	K.MMDKVEFVHMLNATMCATTR.T		(Ions score 42)
416 - 435	796.0359	2385.0860	2385.0772	4	1	K.MMDKVEFVHMLNATMCATTR.T		(Ions score 35)
416 - 435	597.2788	2385.0862	2385.0772	4	1	K.MMDKVEFVHMLNATMCATTR.T		(Ions score 42)
416 - 435	796.0360	2385.0862	2385.0772	4	1	K.MMDKVEFVHMLNATMCATTR.T		(Ions score 43)
416 - 435	597.2788	2385.0862	2385.0772	4	1	K.MMDKVEFVHMLNATMCATTR.T		(Ions score 45)
416 - 435	597.2789	2385.0863	2385.0772	4	1	K.MMDKVEFVHMLNATMCATTR.T		(Ions score 41)
416 - 435	796.0361	2385.0864	2385.0772	4	1	K.MMDKVEFVHMLNATMCATTR.T		(Ions score 30)
416 - 435	597.2789	2385.0865	2385.0772	4	1	K.MMDKVEFVHMLNATMCATTR.T		(Ions score 45)
416 - 435	597.2789	2385.0865	2385.0772	4	1	K.MMDKVEFVHMLNATMCATTR.T		(Ions score 43)
416 - 435	597.2789	2385.0865	2385.0772	4	1	K.MMDKVEFVHMLNATMCATTR.T		(Ions score 52)
416 - 435	796.0361	2385.0866	2385.0772	4	1	K.MMDKVEFVHMLNATMCATTR.T		(Ions score 45)
416 - 435	796.0362	2385.0868	2385.0772	4	1	K.MMDKVEFVHMLNATMCATTR.T		(Ions score 30)
416 - 435	597.2790	2385.0869	2385.0772	4	1	K.MMDKVEFVHMLNATMCATTR.T		(Ions score 50)
416 - 435	597.2792	2385.0875	2385.0772	4	1	K.MMDKVEFVHMLNATMCATTR.T		(Ions score 42)
416 - 435	597.2792	2385.0877	2385.0772	4	1	K.MMDKVEFVHMLNATMCATTR.T		(Ions score 26)
416 - 435	796.0365	2385.0877	2385.0772	4	1	K.MMDKVEFVHMLNATMCATTR.T		(Ions score 45)
416 - 435	597.2792	2385.0878	2385.0772	4	1	K.MMDKVEFVHMLNATMCATTR.T		(Ions score 53)
416 - 435	597.2793	2385.0880	2385.0772	5	1	K.MMDKVEFVHMLNATMCATTR.T		(Ions score 37)
416 - 435	597.2793	2385.0882	2385.0772	5	1	K.MMDKVEFVHMLNATMCATTR.T		(Ions score 53)
416 - 435	597.2794	2385.0886	2385.0772	5	1	K.MMDKVEFVHMLNATMCATTR.T		(Ions score 25)
416 - 435	801.3618	2401.0634	2401.0721	-4	1	K.MMDKVEFVHMLNATMCATTR.T	Oxidation (M)	(Ions score 46)
416 - 435	801.3619	2401.0638	2401.0721	-3	1	K.MMDKVEFVHMLNATMCATTR.T	Oxidation (M)	(Ions score 40)
416 - 435	601.2734	2401.0644	2401.0721	-3	1	K.MMDKVEFVHMLNATMCATTR.T	Oxidation (M)	(Ions score 54)
416 - 435	801.3621	2401.0645	2401.0721	-3	1	K.MMDKVEFVHMLNATMCATTR.T	Oxidation (M)	(Ions score 41)
416 - 435	601.2735	2401.0649	2401.0721	-3	1	K.MMDKVEFVHMLNATMCATTR.T	Oxidation (M)	(Ions score 22)
416 - 435	801.3627	2401.0664	2401.0721	-2	1	K.MMDKVEFVHMLNATMCATTR.T	Oxidation (M)	(Ions score 48)
416 - 435	801.3629	2401.0667	2401.0721	-2	1	K.MMDKVEFVHMLNATMCATTR.T	Oxidation (M)	(Ions score 75)
416 - 435	801.3633	2401.0680	2401.0721	-2	1	K.MMDKVEFVHMLNATMCATTR.T	Oxidation (M)	(Ions score 59)
416 - 435	801.3633	2401.0680	2401.0721	-2	1	K.MMDKVEFVHMLNATMCATTR.T	Oxidation (M)	(Ions score 59)
416 - 435	801.3634	2401.0684	2401.0721	-2	1	K.MMDKVEFVHMLNATMCATTR.T	Oxidation (M)	(Ions score 59)
416 - 435	801.3635	2401.0687	2401.0721	-1	1	K.MMDKVEFVHMLNATMCATTR.T	Oxidation (M)	(Ions score 47)

416 - 435	801.3636	2401.0691	2401.0721	-1	1	K.MMDKVEFVHMLNATMCATTR.T	Oxidation (M)	(Ions score 60)
416 - 435	801.3637	2401.0693	2401.0721	-1	1	K.MMDKVEFVHMLNATMCATTR.T	Oxidation (M)	(Ions score 39)
416 - 435	801.3638	2401.0695	2401.0721	-1	1	K.MMDKVEFVHMLNATMCATTR.T	Oxidation (M)	(Ions score 32)
416 - 435	801.3639	2401.0699	2401.0721	-1	1	K.MMDKVEFVHMLNATMCATTR.T	Oxidation (M)	(Ions score 56)
416 - 435	801.3641	2401.0704	2401.0721	-1	1	K.MMDKVEFVHMLNATMCATTR.T	Oxidation (M)	(Ions score 34)
416 - 435	601.2749	2401.0705	2401.0721	-1	1	K.MMDKVEFVHMLNATMCATTR.T	Oxidation (M)	(Ions score 51)
416 - 435	801.3641	2401.0705	2401.0721	-1	1	K.MMDKVEFVHMLNATMCATTR.T	Oxidation (M)	(Ions score 63)
416 - 435	801.3642	2401.0706	2401.0721	-1	1	K.MMDKVEFVHMLNATMCATTR.T	Oxidation (M)	(Ions score 63)
416 - 435	801.3642	2401.0708	2401.0721	-1	1	K.MMDKVEFVHMLNATMCATTR.T	Oxidation (M)	(Ions score 71)
416 - 435	801.3642	2401.0709	2401.0721	-0	1	K.MMDKVEFVHMLNATMCATTR.T	Oxidation (M)	(Ions score 46)
416 - 435	801.3643	2401.0709	2401.0721	-0	1	K.MMDKVEFVHMLNATMCATTR.T	Oxidation (M)	(Ions score 54)
416 - 435	801.3643	2401.0710	2401.0721	-0	1	K.MMDKVEFVHMLNATMCATTR.T	Oxidation (M)	(Ions score 60)
416 - 435	801.3643	2401.0711	2401.0721	-0	1	K.MMDKVEFVHMLNATMCATTR.T	Oxidation (M)	(Ions score 43)
416 - 435	801.3643	2401.0712	2401.0721	-0	1	K.MMDKVEFVHMLNATMCATTR.T	Oxidation (M)	(Ions score 59)
416 - 435	601.2751	2401.0715	2401.0721	-0	1	K.MMDKVEFVHMLNATMCATTR.T	Oxidation (M)	(Ions score 50)
416 - 435	801.3644	2401.0715	2401.0721	-0	1	K.MMDKVEFVHMLNATMCATTR.T	Oxidation (M)	(Ions score 59)
416 - 435	801.3645	2401.0716	2401.0721	-0	1	K.MMDKVEFVHMLNATMCATTR.T	Oxidation (M)	(Ions score 64)
416 - 435	801.3647	2401.0722	2401.0721	0	1	K.MMDKVEFVHMLNATMCATTR.T	Oxidation (M)	(Ions score 46)
416 - 435	601.2753	2401.0722	2401.0721	0	1	K.MMDKVEFVHMLNATMCATTR.T	Oxidation (M)	(Ions score 30)
416 - 435	801.3647	2401.0724	2401.0721	0	1	K.MMDKVEFVHMLNATMCATTR.T	Oxidation (M)	(Ions score 72)
416 - 435	1201.5435	2401.0725	2401.0721	0	1	K.MMDKVEFVHMLNATMCATTR.T	Oxidation (M)	(Ions score 81)
416 - 435	801.3648	2401.0726	2401.0721	0	1	K.MMDKVEFVHMLNATMCATTR.T	Oxidation (M)	(Ions score 52)
416 - 435	801.3649	2401.0728	2401.0721	0	1	K.MMDKVEFVHMLNATMCATTR.T	Oxidation (M)	(Ions score 60)
416 - 435	801.3650	2401.0730	2401.0721	0	1	K.MMDKVEFVHMLNATMCATTR.T	Oxidation (M)	(Ions score 64)
416 - 435	801.3650	2401.0731	2401.0721	0	1	K.MMDKVEFVHMLNATMCATTR.T	Oxidation (M)	(Ions score 57)
416 - 435	801.3650	2401.0731	2401.0721	0	1	K.MMDKVEFVHMLNATMCATTR.T	Oxidation (M)	(Ions score 60)
416 - 435	601.2756	2401.0732	2401.0721	0	1	K.MMDKVEFVHMLNATMCATTR.T	Oxidation (M)	(Ions score 21)
416 - 435	601.2756	2401.0732	2401.0721	0	1	K.MMDKVEFVHMLNATMCATTR.T	Oxidation (M)	(Ions score 24)
416 - 435	801.3650	2401.0732	2401.0721	0	1	K.MMDKVEFVHMLNATMCATTR.T	Oxidation (M)	(Ions score 63)
416 - 435	801.3650	2401.0733	2401.0721	0	1	K.MMDKVEFVHMLNATMCATTR.T	Oxidation (M)	(Ions score 46)
416 - 435	801.3650	2401.0733	2401.0721	1	1	K.MMDKVEFVHMLNATMCATTR.T	Oxidation (M)	(Ions score 76)
416 - 435	801.3651	2401.0734	2401.0721	1	1	K.MMDKVEFVHMLNATMCATTR.T	Oxidation (M)	(Ions score 32)
416 - 435	801.3651	2401.0735	2401.0721	1	1	K.MMDKVEFVHMLNATMCATTR.T	Oxidation (M)	(Ions score 49)
416 - 435	801.3651	2401.0735	2401.0721	1	1	K.MMDKVEFVHMLNATMCATTR.T	Oxidation (M)	(Ions score 63)
416 - 435	801.3652	2401.0736	2401.0721	1	1	K.MMDKVEFVHMLNATMCATTR.T	Oxidation (M)	(Ions score 60)
416 - 435	801.3652	2401.0738	2401.0721	1	1	K.MMDKVEFVHMLNATMCATTR.T	Oxidation (M)	(Ions score 33)
416 - 435	801.3652	2401.0739	2401.0721	1	1	K.MMDKVEFVHMLNATMCATTR.T	Oxidation (M)	(Ions score 31)
416 - 435	601.2758	2401.0739	2401.0721	1	1	K.MMDKVEFVHMLNATMCATTR.T	Oxidation (M)	(Ions score 34)
416 - 435	801.3653	2401.0740	2401.0721	1	1	K.MMDKVEFVHMLNATMCATTR.T	Oxidation (M)	(Ions score 46)
416 - 435	801.3653	2401.0741	2401.0721	1	1	K.MMDKVEFVHMLNATMCATTR.T	Oxidation (M)	(Ions score 44)
416 - 435	801.3653	2401.0741	2401.0721	1	1	K.MMDKVEFVHMLNATMCATTR.T	Oxidation (M)	(Ions score 60)
416 - 435	801.3653	2401.0741	2401.0721	1	1	K.MMDKVEFVHMLNATMCATTR.T	Oxidation (M)	(Ions score 64)
416 - 435	801.3653	2401.0741	2401.0721	1	1	K.MMDKVEFVHMLNATMCATTR.T	Oxidation (M)	(Ions score 64)
416 - 435	601.2758	2401.0742	2401.0721	1	1	K.MMDKVEFVHMLNATMCATTR.T	Oxidation (M)	(Ions score 45)
416 - 435	601.2758	2401.0742	2401.0721	1	1	K.MMDKVEFVHMLNATMCATTR.T	Oxidation (M)	(Ions score 22)
416 - 435	801.3653	2401.0742	2401.0721	1	1	K.MMDKVEFVHMLNATMCATTR.T	Oxidation (M)	(Ions score 46)
416 - 435	801.3654	2401.0742	2401.0721	1	1	K.MMDKVEFVHMLNATMCATTR.T	Oxidation (M)	(Ions score 33)
416 - 435	801.3654	2401.0742	2401.0721	1	1	K.MMDKVEFVHMLNATMCATTR.T	Oxidation (M)	(Ions score 32)
416 - 435	601.2759	2401.0743	2401.0721	1	1	K.MMDKVEFVHMLNATMCATTR.T	Oxidation (M)	(Ions score 24)
416 - 435	801.3654	2401.0744	2401.0721	1	1	K.MMDKVEFVHMLNATMCATTR.T	Oxidation (M)	(Ions score 50)
416 - 435	801.3654	2401.0745	2401.0721	1	1	K.MMDKVEFVHMLNATMCATTR.T	Oxidation (M)	(Ions score 55)
416 - 435	801.3654	2401.0745	2401.0721	1	1	K.MMDKVEFVHMLNATMCATTR.T	Oxidation (M)	(Ions score 49)
416 - 435	1201.5446	2401.0746	2401.0721	1	1	K.MMDKVEFVHMLNATMCATTR.T	Oxidation (M)	(Ions score 64)
416 - 435	801.3655	2401.0746	2401.0721	1	1	K.MMDKVEFVHMLNATMCATTR.T	Oxidation (M)	(Ions score 47)
416 - 435	801.3655	2401.0748	2401.0721	1	1	K.MMDKVEFVHMLNATMCATTR.T	Oxidation (M)	(Ions score 48)
416 - 435	801.3655	2401.0748	2401.0721	1	1	K.MMDKVEFVHMLNATMCATTR.T	Oxidation (M)	(Ions score 45)
416 - 435	801.3656	2401.0748	2401.0721	1	1	K.MMDKVEFVHMLNATMCATTR.T	Oxidation (M)	(Ions score 48)

416 - 435	801.3656	2401.0749	2401.0721	1	1	K.MMDKVEFVHMLNATMCATTR.T	Oxidation (M)	(Ions score 61)
416 - 435	801.3656	2401.0750	2401.0721	1	1	K.MMDKVEFVHMLNATMCATTR.T	Oxidation (M)	(Ions score 51)
416 - 435	801.3656	2401.0750	2401.0721	1	1	K.MMDKVEFVHMLNATMCATTR.T	Oxidation (M)	(Ions score 65)
416 - 435	801.3656	2401.0750	2401.0721	1	1	K.MMDKVEFVHMLNATMCATTR.T	Oxidation (M)	(Ions score 64)
416 - 435	801.3656	2401.0750	2401.0721	1	1	K.MMDKVEFVHMLNATMCATTR.T	Oxidation (M)	(Ions score 61)
416 - 435	801.3656	2401.0751	2401.0721	1	1	K.MMDKVEFVHMLNATMCATTR.T	Oxidation (M)	(Ions score 72)
416 - 435	801.3657	2401.0751	2401.0721	1	1	K.MMDKVEFVHMLNATMCATTR.T	Oxidation (M)	(Ions score 65)
416 - 435	1201.5449	2401.0752	2401.0721	1	1	K.MMDKVEFVHMLNATMCATTR.T	Oxidation (M)	(Ions score 75)
416 - 435	801.3657	2401.0753	2401.0721	1	1	K.MMDKVEFVHMLNATMCATTR.T	Oxidation (M)	(Ions score 72)
416 - 435	801.3657	2401.0753	2401.0721	1	1	K.MMDKVEFVHMLNATMCATTR.T	Oxidation (M)	(Ions score 58)
416 - 435	801.3657	2401.0754	2401.0721	1	1	K.MMDKVEFVHMLNATMCATTR.T	Oxidation (M)	(Ions score 63)
416 - 435	801.3657	2401.0754	2401.0721	1	1	K.MMDKVEFVHMLNATMCATTR.T	Oxidation (M)	(Ions score 50)
416 - 435	801.3657	2401.0754	2401.0721	1	1	K.MMDKVEFVHMLNATMCATTR.T	Oxidation (M)	(Ions score 37)
416 - 435	801.3658	2401.0755	2401.0721	1	1	K.MMDKVEFVHMLNATMCATTR.T	Oxidation (M)	(Ions score 50)
416 - 435	801.3658	2401.0755	2401.0721	1	1	K.MMDKVEFVHMLNATMCATTR.T	Oxidation (M)	(Ions score 45)
416 - 435	801.3658	2401.0755	2401.0721	1	1	K.MMDKVEFVHMLNATMCATTR.T	Oxidation (M)	(Ions score 38)
416 - 435	801.3658	2401.0755	2401.0721	1	1	K.MMDKVEFVHMLNATMCATTR.T	Oxidation (M)	(Ions score 45)
416 - 435	601.2762	2401.0756	2401.0721	1	1	K.MMDKVEFVHMLNATMCATTR.T	Oxidation (M)	(Ions score 59)
416 - 435	801.3658	2401.0756	2401.0721	1	1	K.MMDKVEFVHMLNATMCATTR.T	Oxidation (M)	(Ions score 55)
416 - 435	801.3658	2401.0757	2401.0721	2	1	K.MMDKVEFVHMLNATMCATTR.T	Oxidation (M)	(Ions score 58)
416 - 435	801.3659	2401.0758	2401.0721	2	1	K.MMDKVEFVHMLNATMCATTR.T	Oxidation (M)	(Ions score 46)
416 - 435	801.3659	2401.0759	2401.0721	2	1	K.MMDKVEFVHMLNATMCATTR.T	Oxidation (M)	(Ions score 64)
416 - 435	801.3660	2401.0761	2401.0721	2	1	K.MMDKVEFVHMLNATMCATTR.T	Oxidation (M)	(Ions score 57)
416 - 435	801.3660	2401.0761	2401.0721	2	1	K.MMDKVEFVHMLNATMCATTR.T	Oxidation (M)	(Ions score 87)
416 - 435	801.3660	2401.0761	2401.0721	2	1	K.MMDKVEFVHMLNATMCATTR.T	Oxidation (M)	(Ions score 60)
416 - 435	601.2763	2401.0761	2401.0721	2	1	K.MMDKVEFVHMLNATMCATTR.T	Oxidation (M)	(Ions score 42)
416 - 435	801.3660	2401.0762	2401.0721	2	1	K.MMDKVEFVHMLNATMCATTR.T	Oxidation (M)	(Ions score 31)
416 - 435	801.3660	2401.0762	2401.0721	2	1	K.MMDKVEFVHMLNATMCATTR.T	Oxidation (M)	(Ions score 60)
416 - 435	601.2763	2401.0762	2401.0721	2	1	K.MMDKVEFVHMLNATMCATTR.T	Oxidation (M)	(Ions score 21)
416 - 435	801.3660	2401.0762	2401.0721	2	1	K.MMDKVEFVHMLNATMCATTR.T	Oxidation (M)	(Ions score 47)
416 - 435	801.3660	2401.0762	2401.0721	2	1	K.MMDKVEFVHMLNATMCATTR.T	Oxidation (M)	(Ions score 55)
416 - 435	801.3660	2401.0763	2401.0721	2	1	K.MMDKVEFVHMLNATMCATTR.T	Oxidation (M)	(Ions score 51)
416 - 435	801.3661	2401.0763	2401.0721	2	1	K.MMDKVEFVHMLNATMCATTR.T	Oxidation (M)	(Ions score 57)
416 - 435	801.3661	2401.0766	2401.0721	2	1	K.MMDKVEFVHMLNATMCATTR.T	Oxidation (M)	(Ions score 57)
416 - 435	801.3661	2401.0766	2401.0721	2	1	K.MMDKVEFVHMLNATMCATTR.T	Oxidation (M)	(Ions score 64)
416 - 435	801.3662	2401.0767	2401.0721	2	1	K.MMDKVEFVHMLNATMCATTR.T	Oxidation (M)	(Ions score 48)
416 - 435	801.3662	2401.0768	2401.0721	2	1	K.MMDKVEFVHMLNATMCATTR.T	Oxidation (M)	(Ions score 60)
416 - 435	801.3662	2401.0768	2401.0721	2	1	K.MMDKVEFVHMLNATMCATTR.T	Oxidation (M)	(Ions score 72)
416 - 435	801.3662	2401.0768	2401.0721	2	1	K.MMDKVEFVHMLNATMCATTR.T	Oxidation (M)	(Ions score 52)
416 - 435	801.3662	2401.0768	2401.0721	2	1	K.MMDKVEFVHMLNATMCATTR.T	Oxidation (M)	(Ions score 24)
416 - 435	601.2765	2401.0769	2401.0721	2	1	K.MMDKVEFVHMLNATMCATTR.T	Oxidation (M)	(Ions score 23)
416 - 435	801.3662	2401.0769	2401.0721	2	1	K.MMDKVEFVHMLNATMCATTR.T	Oxidation (M)	(Ions score 43)
416 - 435	801.3663	2401.0770	2401.0721	2	1	K.MMDKVEFVHMLNATMCATTR.T	Oxidation (M)	(Ions score 47)
416 - 435	601.2765	2401.0770	2401.0721	2	1	K.MMDKVEFVHMLNATMCATTR.T	Oxidation (M)	(Ions score 21)
416 - 435	801.3663	2401.0770	2401.0721	2	1	K.MMDKVEFVHMLNATMCATTR.T	Oxidation (M)	(Ions score 58)
416 - 435	801.3663	2401.0771	2401.0721	2	1	K.MMDKVEFVHMLNATMCATTR.T	Oxidation (M)	(Ions score 76)
416 - 435	601.2766	2401.0771	2401.0721	2	1	K.MMDKVEFVHMLNATMCATTR.T	Oxidation (M)	(Ions score 22)
416 - 435	801.3663	2401.0771	2401.0721	2	1	K.MMDKVEFVHMLNATMCATTR.T	Oxidation (M)	(Ions score 47)
416 - 435	801.3663	2401.0771	2401.0721	2	1	K.MMDKVEFVHMLNATMCATTR.T	Oxidation (M)	(Ions score 60)
416 - 435	1201.5459	2401.0772	2401.0721	2	1	K.MMDKVEFVHMLNATMCATTR.T	Oxidation (M)	(Ions score 83)
416 - 435	801.3663	2401.0772	2401.0721	2	1	K.MMDKVEFVHMLNATMCATTR.T	Oxidation (M)	(Ions score 44)
416 - 435	801.3664	2401.0773	2401.0721	2	1	K.MMDKVEFVHMLNATMCATTR.T	Oxidation (M)	(Ions score 38)
416 - 435	801.3664	2401.0773	2401.0721	2	1	K.MMDKVEFVHMLNATMCATTR.T	Oxidation (M)	(Ions score 64)
416 - 435	601.2766	2401.0774	2401.0721	2	1	K.MMDKVEFVHMLNATMCATTR.T	Oxidation (M)	(Ions score 22)
416 - 435	801.3665	2401.0775	2401.0721	2	1	K.MMDKVEFVHMLNATMCATTR.T	Oxidation (M)	(Ions score 63)
416 - 435	1201.5461	2401.0776	2401.0721	2	1	K.MMDKVEFVHMLNATMCATTR.T	Oxidation (M)	(Ions score 81)
416 - 435	601.2767	2401.0776	2401.0721	2	1	K.MMDKVEFVHMLNATMCATTR.T	Oxidation (M)	(Ions score 27)

416 - 435	801.3665	2401.0777	2401.0721	2	1	K.MMDKVEFVHMLNATMCATTR.T	Oxidation (M)	(Ions score 57)
416 - 435	801.3665	2401.0777	2401.0721	2	1	K.MMDKVEFVHMLNATMCATTR.T	Oxidation (M)	(Ions score 49)
416 - 435	801.3665	2401.0778	2401.0721	2	1	K.MMDKVEFVHMLNATMCATTR.T	Oxidation (M)	(Ions score 61)
416 - 435	601.2767	2401.0778	2401.0721	2	1	K.MMDKVEFVHMLNATMCATTR.T	Oxidation (M)	(Ions score 22)
416 - 435	801.3665	2401.0778	2401.0721	2	1	K.MMDKVEFVHMLNATMCATTR.T	Oxidation (M)	(Ions score 46)
416 - 435	601.2767	2401.0778	2401.0721	2	1	K.MMDKVEFVHMLNATMCATTR.T	Oxidation (M)	(Ions score 21)
416 - 435	601.2767	2401.0779	2401.0721	2	1	K.MMDKVEFVHMLNATMCATTR.T	Oxidation (M)	(Ions score 22)
416 - 435	601.2767	2401.0779	2401.0721	2	1	K.MMDKVEFVHMLNATMCATTR.T	Oxidation (M)	(Ions score 23)
416 - 435	801.3666	2401.0779	2401.0721	2	1	K.MMDKVEFVHMLNATMCATTR.T	Oxidation (M)	(Ions score 35)
416 - 435	1201.5462	2401.0779	2401.0721	2	1	K.MMDKVEFVHMLNATMCATTR.T	Oxidation (M)	(Ions score 110)
416 - 435	801.3666	2401.0779	2401.0721	2	1	K.MMDKVEFVHMLNATMCATTR.T	Oxidation (M)	(Ions score 60)
416 - 435	601.2768	2401.0779	2401.0721	2	1	K.MMDKVEFVHMLNATMCATTR.T	Oxidation (M)	(Ions score 21)
416 - 435	801.3666	2401.0780	2401.0721	2	1	K.MMDKVEFVHMLNATMCATTR.T	Oxidation (M)	(Ions score 67)
416 - 435	1201.5463	2401.0781	2401.0721	2	1	K.MMDKVEFVHMLNATMCATTR.T	Oxidation (M)	(Ions score 97)
416 - 435	801.3666	2401.0781	2401.0721	3	1	K.MMDKVEFVHMLNATMCATTR.T	Oxidation (M)	(Ions score 21)
416 - 435	801.3666	2401.0781	2401.0721	3	1	K.MMDKVEFVHMLNATMCATTR.T	Oxidation (M)	(Ions score 48)
416 - 435	601.2768	2401.0781	2401.0721	3	1	K.MMDKVEFVHMLNATMCATTR.T	Oxidation (M)	(Ions score 28)
416 - 435	801.3667	2401.0782	2401.0721	3	1	K.MMDKVEFVHMLNATMCATTR.T	Oxidation (M)	(Ions score 58)
416 - 435	1201.5464	2401.0782	2401.0721	3	1	K.MMDKVEFVHMLNATMCATTR.T	Oxidation (M)	(Ions score 63)
416 - 435	601.2769	2401.0783	2401.0721	3	1	K.MMDKVEFVHMLNATMCATTR.T	Oxidation (M)	(Ions score 22)
416 - 435	801.3667	2401.0783	2401.0721	3	1	K.MMDKVEFVHMLNATMCATTR.T	Oxidation (M)	(Ions score 49)
416 - 435	801.3667	2401.0783	2401.0721	3	1	K.MMDKVEFVHMLNATMCATTR.T	Oxidation (M)	(Ions score 49)
416 - 435	801.3667	2401.0783	2401.0721	3	1	K.MMDKVEFVHMLNATMCATTR.T	Oxidation (M)	(Ions score 76)
416 - 435	601.2769	2401.0783	2401.0721	3	1	K.MMDKVEFVHMLNATMCATTR.T	Oxidation (M)	(Ions score 22)
416 - 435	801.3667	2401.0784	2401.0721	3	1	K.MMDKVEFVHMLNATMCATTR.T	Oxidation (M)	(Ions score 76)
416 - 435	1201.5465	2401.0784	2401.0721	3	1	K.MMDKVEFVHMLNATMCATTR.T	Oxidation (M)	(Ions score 94)
416 - 435	801.3668	2401.0785	2401.0721	3	1	K.MMDKVEFVHMLNATMCATTR.T	Oxidation (M)	(Ions score 24)
416 - 435	801.3668	2401.0785	2401.0721	3	1	K.MMDKVEFVHMLNATMCATTR.T	Oxidation (M)	(Ions score 50)
416 - 435	801.3668	2401.0785	2401.0721	3	1	K.MMDKVEFVHMLNATMCATTR.T	Oxidation (M)	(Ions score 40)
416 - 435	601.2769	2401.0785	2401.0721	3	1	K.MMDKVEFVHMLNATMCATTR.T	Oxidation (M)	(Ions score 22)
416 - 435	1201.5466	2401.0786	2401.0721	3	1	K.MMDKVEFVHMLNATMCATTR.T	Oxidation (M)	(Ions score 65)
416 - 435	801.3668	2401.0786	2401.0721	3	1	K.MMDKVEFVHMLNATMCATTR.T	Oxidation (M)	(Ions score 64)
416 - 435	801.3668	2401.0786	2401.0721	3	1	K.MMDKVEFVHMLNATMCATTR.T	Oxidation (M)	(Ions score 64)
416 - 435	801.3668	2401.0786	2401.0721	3	1	K.MMDKVEFVHMLNATMCATTR.T	Oxidation (M)	(Ions score 73)
416 - 435	801.3668	2401.0786	2401.0721	3	1	K.MMDKVEFVHMLNATMCATTR.T	Oxidation (M)	(Ions score 64)
416 - 435	801.3668	2401.0787	2401.0721	3	1	K.MMDKVEFVHMLNATMCATTR.T	Oxidation (M)	(Ions score 47)
416 - 435	801.3668	2401.0787	2401.0721	3	1	K.MMDKVEFVHMLNATMCATTR.T	Oxidation (M)	(Ions score 40)
416 - 435	601.2770	2401.0787	2401.0721	3	1	K.MMDKVEFVHMLNATMCATTR.T	Oxidation (M)	(Ions score 22)
416 - 435	801.3669	2401.0787	2401.0721	3	1	K.MMDKVEFVHMLNATMCATTR.T	Oxidation (M)	(Ions score 57)
416 - 435	1201.5467	2401.0788	2401.0721	3	1	K.MMDKVEFVHMLNATMCATTR.T	Oxidation (M)	(Ions score 56)
416 - 435	801.3669	2401.0788	2401.0721	3	1	K.MMDKVEFVHMLNATMCATTR.T	Oxidation (M)	(Ions score 61)
416 - 435	801.3669	2401.0788	2401.0721	3	1	K.MMDKVEFVHMLNATMCATTR.T	Oxidation (M)	(Ions score 35)
416 - 435	801.3669	2401.0790	2401.0721	3	1	K.MMDKVEFVHMLNATMCATTR.T	Oxidation (M)	(Ions score 61)
416 - 435	1201.5468	2401.0791	2401.0721	3	1	K.MMDKVEFVHMLNATMCATTR.T	Oxidation (M)	(Ions score 80)
416 - 435	601.2770	2401.0791	2401.0721	3	1	K.MMDKVEFVHMLNATMCATTR.T	Oxidation (M)	(Ions score 29)
416 - 435	601.2770	2401.0791	2401.0721	3	1	K.MMDKVEFVHMLNATMCATTR.T	Oxidation (M)	(Ions score 28)
416 - 435	1201.5469	2401.0792	2401.0721	3	1	K.MMDKVEFVHMLNATMCATTR.T	Oxidation (M)	(Ions score 121)
416 - 435	1201.5469	2401.0792	2401.0721	3	1	K.MMDKVEFVHMLNATMCATTR.T	Oxidation (M)	(Ions score 83)
416 - 435	801.3670	2401.0792	2401.0721	3	1	K.MMDKVEFVHMLNATMCATTR.T	Oxidation (M)	(Ions score 64)
416 - 435	801.3670	2401.0792	2401.0721	3	1	K.MMDKVEFVHMLNATMCATTR.T	Oxidation (M)	(Ions score 53)
416 - 435	801.3670	2401.0793	2401.0721	3	1	K.MMDKVEFVHMLNATMCATTR.T	Oxidation (M)	(Ions score 37)
416 - 435	801.3671	2401.0794	2401.0721	3	1	K.MMDKVEFVHMLNATMCATTR.T	Oxidation (M)	(Ions score 76)
416 - 435	801.3671	2401.0794	2401.0721	3	1	K.MMDKVEFVHMLNATMCATTR.T	Oxidation (M)	(Ions score 30)
416 - 435	801.3671	2401.0794	2401.0721	3	1	K.MMDKVEFVHMLNATMCATTR.T	Oxidation (M)	(Ions score 71)
416 - 435	801.3671	2401.0794	2401.0721	3	1	K.MMDKVEFVHMLNATMCATTR.T	Oxidation (M)	(Ions score 48)
416 - 435	801.3671	2401.0795	2401.0721	3	1	K.MMDKVEFVHMLNATMCATTR.T	Oxidation (M)	(Ions score 50)
416 - 435	801.3671	2401.0795	2401.0721	3	1	K.MMDKVEFVHMLNATMCATTR.T	Oxidation (M)	(Ions score 55)

416 - 435	1201.5471	2401.0796	2401.0721	3	1	K.MMDKVEFVHMLNATMCATTR.T	Oxidation (M) (Ions score 98)
416 - 435	601.2772	2401.0796	2401.0721	3	1	K.MMDKVEFVHMLNATMCATTR.T	Oxidation (M) (Ions score 23)
416 - 435	801.3672	2401.0797	2401.0721	3	1	K.MMDKVEFVHMLNATMCATTR.T	Oxidation (M) (Ions score 45)
416 - 435	801.3672	2401.0798	2401.0721	3	1	K.MMDKVEFVHMLNATMCATTR.T	Oxidation (M) (Ions score 48)
416 - 435	801.3672	2401.0798	2401.0721	3	1	K.MMDKVEFVHMLNATMCATTR.T	Oxidation (M) (Ions score 69)
416 - 435	601.2772	2401.0799	2401.0721	3	1	K.MMDKVEFVHMLNATMCATTR.T	Oxidation (M) (Ions score 35)
416 - 435	801.3672	2401.0799	2401.0721	3	1	K.MMDKVEFVHMLNATMCATTR.T	Oxidation (M) (Ions score 48)
416 - 435	801.3672	2401.0799	2401.0721	3	1	K.MMDKVEFVHMLNATMCATTR.T	Oxidation (M) (Ions score 71)
416 - 435	801.3673	2401.0799	2401.0721	3	1	K.MMDKVEFVHMLNATMCATTR.T	Oxidation (M) (Ions score 62)
416 - 435	601.2773	2401.0800	2401.0721	3	1	K.MMDKVEFVHMLNATMCATTR.T	Oxidation (M) (Ions score 22)
416 - 435	801.3673	2401.0802	2401.0721	3	1	K.MMDKVEFVHMLNATMCATTR.T	Oxidation (M) (Ions score 36)
416 - 435	801.3674	2401.0803	2401.0721	3	1	K.MMDKVEFVHMLNATMCATTR.T	Oxidation (M) (Ions score 45)
416 - 435	1201.5474	2401.0803	2401.0721	3	1	K.MMDKVEFVHMLNATMCATTR.T	Oxidation (M) (Ions score 75)
416 - 435	801.3674	2401.0804	2401.0721	3	1	K.MMDKVEFVHMLNATMCATTR.T	Oxidation (M) (Ions score 50)
416 - 435	801.3675	2401.0805	2401.0721	4	1	K.MMDKVEFVHMLNATMCATTR.T	Oxidation (M) (Ions score 48)
416 - 435	801.3675	2401.0806	2401.0721	4	1	K.MMDKVEFVHMLNATMCATTR.T	Oxidation (M) (Ions score 50)
416 - 435	801.3675	2401.0806	2401.0721	4	1	K.MMDKVEFVHMLNATMCATTR.T	Oxidation (M) (Ions score 45)
416 - 435	801.3675	2401.0807	2401.0721	4	1	K.MMDKVEFVHMLNATMCATTR.T	Oxidation (M) (Ions score 47)
416 - 435	801.3675	2401.0808	2401.0721	4	1	K.MMDKVEFVHMLNATMCATTR.T	Oxidation (M) (Ions score 54)
416 - 435	801.3675	2401.0808	2401.0721	4	1	K.MMDKVEFVHMLNATMCATTR.T	Oxidation (M) (Ions score 58)
416 - 435	801.3675	2401.0808	2401.0721	4	1	K.MMDKVEFVHMLNATMCATTR.T	Oxidation (M) (Ions score 50)
416 - 435	601.2775	2401.0811	2401.0721	4	1	K.MMDKVEFVHMLNATMCATTR.T	Oxidation (M) (Ions score 23)
416 - 435	801.3676	2401.0811	2401.0721	4	1	K.MMDKVEFVHMLNATMCATTR.T	Oxidation (M) (Ions score 53)
416 - 435	801.3676	2401.0811	2401.0721	4	1	K.MMDKVEFVHMLNATMCATTR.T	Oxidation (M) (Ions score 45)
416 - 435	801.3677	2401.0812	2401.0721	4	1	K.MMDKVEFVHMLNATMCATTR.T	Oxidation (M) (Ions score 52)
416 - 435	801.3677	2401.0812	2401.0721	4	1	K.MMDKVEFVHMLNATMCATTR.T	Oxidation (M) (Ions score 62)
416 - 435	801.3678	2401.0816	2401.0721	4	1	K.MMDKVEFVHMLNATMCATTR.T	Oxidation (M) (Ions score 48)
416 - 435	801.3678	2401.0816	2401.0721	4	1	K.MMDKVEFVHMLNATMCATTR.T	Oxidation (M) (Ions score 58)
416 - 435	601.2777	2401.0816	2401.0721	4	1	K.MMDKVEFVHMLNATMCATTR.T	Oxidation (M) (Ions score 37)
416 - 435	801.3679	2401.0818	2401.0721	4	1	K.MMDKVEFVHMLNATMCATTR.T	Oxidation (M) (Ions score 43)
416 - 435	801.3680	2401.0822	2401.0721	4	1	K.MMDKVEFVHMLNATMCATTR.T	Oxidation (M) (Ions score 38)
416 - 435	801.3680	2401.0823	2401.0721	4	1	K.MMDKVEFVHMLNATMCATTR.T	Oxidation (M) (Ions score 59)
416 - 435	801.3681	2401.0825	2401.0721	4	1	K.MMDKVEFVHMLNATMCATTR.T	Oxidation (M) (Ions score 46)
416 - 435	801.3681	2401.0826	2401.0721	4	1	K.MMDKVEFVHMLNATMCATTR.T	Oxidation (M) (Ions score 71)
416 - 435	801.3681	2401.0826	2401.0721	4	1	K.MMDKVEFVHMLNATMCATTR.T	Oxidation (M) (Ions score 54)
416 - 435	1201.5489	2401.0832	2401.0721	5	1	K.MMDKVEFVHMLNATMCATTR.T	Oxidation (M) (Ions score 72)
416 - 435	1201.5491	2401.0836	2401.0721	5	1	K.MMDKVEFVHMLNATMCATTR.T	Oxidation (M) (Ions score 92)
416 - 435	801.3686	2401.0840	2401.0721	5	1	K.MMDKVEFVHMLNATMCATTR.T	Oxidation (M) (Ions score 53)
416 - 435	801.3686	2401.0840	2401.0721	5	1	K.MMDKVEFVHMLNATMCATTR.T	Oxidation (M) (Ions score 59)
416 - 435	801.3686	2401.0840	2401.0721	5	1	K.MMDKVEFVHMLNATMCATTR.T	Oxidation (M) (Ions score 45)
416 - 435	806.6927	2417.0564	2417.0670	-4	1	K.MMDKVEFVHMLNATMCATTR.T	2 Oxidation (M) (Ions score 39)
416 - 435	806.6943	2417.0612	2417.0670	-2	1	K.MMDKVEFVHMLNATMCATTR.T	2 Oxidation (M) (Ions score 26)
416 - 435	605.2727	2417.0617	2417.0670	-2	1	K.MMDKVEFVHMLNATMCATTR.T	2 Oxidation (M) (Ions score 43)
416 - 435	806.6947	2417.0624	2417.0670	-2	1	K.MMDKVEFVHMLNATMCATTR.T	2 Oxidation (M) (Ions score 49)
416 - 435	806.6952	2417.0637	2417.0670	-1	1	K.MMDKVEFVHMLNATMCATTR.T	2 Oxidation (M) (Ions score 27)
416 - 435	806.6954	2417.0644	2417.0670	-1	1	K.MMDKVEFVHMLNATMCATTR.T	2 Oxidation (M) (Ions score 25)
416 - 435	1209.5396	2417.0646	2417.0670	-1	1	K.MMDKVEFVHMLNATMCATTR.T	2 Oxidation (M) (Ions score 90)
416 - 435	605.2737	2417.0656	2417.0670	-1	1	K.MMDKVEFVHMLNATMCATTR.T	2 Oxidation (M) (Ions score 23)
416 - 435	806.6960	2417.0662	2417.0670	-0	1	K.MMDKVEFVHMLNATMCATTR.T	2 Oxidation (M) (Ions score 49)
416 - 435	806.6962	2417.0668	2417.0670	-0	1	K.MMDKVEFVHMLNATMCATTR.T	2 Oxidation (M) (Ions score 42)
416 - 435	1209.5408	2417.0670	2417.0670	0	1	K.MMDKVEFVHMLNATMCATTR.T	2 Oxidation (M) (Ions score 93)
416 - 435	806.6963	2417.0671	2417.0670	0	1	K.MMDKVEFVHMLNATMCATTR.T	2 Oxidation (M) (Ions score 33)
416 - 435	806.6963	2417.0671	2417.0670	0	1	K.MMDKVEFVHMLNATMCATTR.T	2 Oxidation (M) (Ions score 49)
416 - 435	806.6963	2417.0671	2417.0670	0	1	K.MMDKVEFVHMLNATMCATTR.T	2 Oxidation (M) (Ions score 47)
416 - 435	1209.5408	2417.0671	2417.0670	0	1	K.MMDKVEFVHMLNATMCATTR.T	2 Oxidation (M) (Ions score 62)
416 - 435	806.6964	2417.0672	2417.0670	0	1	K.MMDKVEFVHMLNATMCATTR.T	2 Oxidation (M) (Ions score 45)
416 - 435	806.6964	2417.0674	2417.0670	0	1	K.MMDKVEFVHMLNATMCATTR.T	2 Oxidation (M) (Ions score 49)

416 - 435	806.6964	2417.0674	2417.0670	0	1	K.MMDKVEFVHMLNATMCATTR.T	2 Oxidation (M)	(Ions score 36)
416 - 435	806.6964	2417.0674	2417.0670	0	1	K.MMDKVEFVHMLNATMCATTR.T	2 Oxidation (M)	(Ions score 45)
416 - 435	605.2742	2417.0676	2417.0670	0	1	K.MMDKVEFVHMLNATMCATTR.T	2 Oxidation (M)	(Ions score 45)
416 - 435	806.6965	2417.0676	2417.0670	0	1	K.MMDKVEFVHMLNATMCATTR.T	2 Oxidation (M)	(Ions score 46)
416 - 435	1209.5411	2417.0677	2417.0670	0	1	K.MMDKVEFVHMLNATMCATTR.T	2 Oxidation (M)	(Ions score 72)
416 - 435	806.6965	2417.0677	2417.0670	0	1	K.MMDKVEFVHMLNATMCATTR.T	2 Oxidation (M)	(Ions score 69)
416 - 435	806.6965	2417.0678	2417.0670	0	1	K.MMDKVEFVHMLNATMCATTR.T	2 Oxidation (M)	(Ions score 23)
416 - 435	605.2742	2417.0678	2417.0670	0	1	K.MMDKVEFVHMLNATMCATTR.T	2 Oxidation (M)	(Ions score 54)
416 - 435	605.2742	2417.0678	2417.0670	0	1	K.MMDKVEFVHMLNATMCATTR.T	2 Oxidation (M)	(Ions score 49)
416 - 435	806.6966	2417.0679	2417.0670	0	1	K.MMDKVEFVHMLNATMCATTR.T	2 Oxidation (M)	(Ions score 24)
416 - 435	806.6966	2417.0680	2417.0670	0	1	K.MMDKVEFVHMLNATMCATTR.T	2 Oxidation (M)	(Ions score 57)
416 - 435	806.6966	2417.0680	2417.0670	0	1	K.MMDKVEFVHMLNATMCATTR.T	2 Oxidation (M)	(Ions score 43)
416 - 435	806.6967	2417.0682	2417.0670	0	1	K.MMDKVEFVHMLNATMCATTR.T	2 Oxidation (M)	(Ions score 60)
416 - 435	806.6967	2417.0682	2417.0670	0	1	K.MMDKVEFVHMLNATMCATTR.T	2 Oxidation (M)	(Ions score 31)
416 - 435	806.6967	2417.0684	2417.0670	1	1	K.MMDKVEFVHMLNATMCATTR.T	2 Oxidation (M)	(Ions score 46)
416 - 435	806.6968	2417.0685	2417.0670	1	1	K.MMDKVEFVHMLNATMCATTR.T	2 Oxidation (M)	(Ions score 60)
416 - 435	806.6968	2417.0685	2417.0670	1	1	K.MMDKVEFVHMLNATMCATTR.T	2 Oxidation (M)	(Ions score 49)
416 - 435	806.6968	2417.0685	2417.0670	1	1	K.MMDKVEFVHMLNATMCATTR.T	2 Oxidation (M)	(Ions score 42)
416 - 435	806.6968	2417.0686	2417.0670	1	1	K.MMDKVEFVHMLNATMCATTR.T	2 Oxidation (M)	(Ions score 49)
416 - 435	806.6968	2417.0687	2417.0670	1	1	K.MMDKVEFVHMLNATMCATTR.T	2 Oxidation (M)	(Ions score 23)
416 - 435	806.6968	2417.0687	2417.0670	1	1	K.MMDKVEFVHMLNATMCATTR.T	2 Oxidation (M)	(Ions score 32)
416 - 435	605.2745	2417.0687	2417.0670	1	1	K.MMDKVEFVHMLNATMCATTR.T	2 Oxidation (M)	(Ions score 23)
416 - 435	806.6969	2417.0689	2417.0670	1	1	K.MMDKVEFVHMLNATMCATTR.T	2 Oxidation (M)	(Ions score 38)
416 - 435	806.6969	2417.0689	2417.0670	1	1	K.MMDKVEFVHMLNATMCATTR.T	2 Oxidation (M)	(Ions score 46)
416 - 435	806.6969	2417.0689	2417.0670	1	1	K.MMDKVEFVHMLNATMCATTR.T	2 Oxidation (M)	(Ions score 47)
416 - 435	806.6969	2417.0689	2417.0670	1	1	K.MMDKVEFVHMLNATMCATTR.T	2 Oxidation (M)	(Ions score 75)
416 - 435	806.6969	2417.0690	2417.0670	1	1	K.MMDKVEFVHMLNATMCATTR.T	2 Oxidation (M)	(Ions score 35)
416 - 435	806.6970	2417.0691	2417.0670	1	1	K.MMDKVEFVHMLNATMCATTR.T	2 Oxidation (M)	(Ions score 33)
416 - 435	806.6970	2417.0691	2417.0670	1	1	K.MMDKVEFVHMLNATMCATTR.T	2 Oxidation (M)	(Ions score 29)
416 - 435	806.6970	2417.0692	2417.0670	1	1	K.MMDKVEFVHMLNATMCATTR.T	2 Oxidation (M)	(Ions score 43)
416 - 435	806.6970	2417.0693	2417.0670	1	1	K.MMDKVEFVHMLNATMCATTR.T	2 Oxidation (M)	(Ions score 57)
416 - 435	806.6971	2417.0694	2417.0670	1	1	K.MMDKVEFVHMLNATMCATTR.T	2 Oxidation (M)	(Ions score 24)
416 - 435	605.2746	2417.0694	2417.0670	1	1	K.MMDKVEFVHMLNATMCATTR.T	2 Oxidation (M)	(Ions score 30)
416 - 435	806.6971	2417.0695	2417.0670	1	1	K.MMDKVEFVHMLNATMCATTR.T	2 Oxidation (M)	(Ions score 46)
416 - 435	806.6971	2417.0695	2417.0670	1	1	K.MMDKVEFVHMLNATMCATTR.T	2 Oxidation (M)	(Ions score 38)
416 - 435	806.6971	2417.0695	2417.0670	1	1	K.MMDKVEFVHMLNATMCATTR.T	2 Oxidation (M)	(Ions score 45)
416 - 435	605.2747	2417.0695	2417.0670	1	1	K.MMDKVEFVHMLNATMCATTR.T	2 Oxidation (M)	(Ions score 49)
416 - 435	806.6971	2417.0696	2417.0670	1	1	K.MMDKVEFVHMLNATMCATTR.T	2 Oxidation (M)	(Ions score 40)
416 - 435	605.2747	2417.0696	2417.0670	1	1	K.MMDKVEFVHMLNATMCATTR.T	2 Oxidation (M)	(Ions score 23)
416 - 435	806.6971	2417.0696	2417.0670	1	1	K.MMDKVEFVHMLNATMCATTR.T	2 Oxidation (M)	(Ions score 33)
416 - 435	1209.5421	2417.0697	2417.0670	1	1	K.MMDKVEFVHMLNATMCATTR.T	2 Oxidation (M)	(Ions score 60)
416 - 435	806.6972	2417.0698	2417.0670	1	1	K.MMDKVEFVHMLNATMCATTR.T	2 Oxidation (M)	(Ions score 37)
416 - 435	806.6972	2417.0698	2417.0670	1	1	K.MMDKVEFVHMLNATMCATTR.T	2 Oxidation (M)	(Ions score 50)
416 - 435	806.6972	2417.0698	2417.0670	1	1	K.MMDKVEFVHMLNATMCATTR.T	2 Oxidation (M)	(Ions score 51)
416 - 435	806.6972	2417.0698	2417.0670	1	1	K.MMDKVEFVHMLNATMCATTR.T	2 Oxidation (M)	(Ions score 75)
416 - 435	806.6972	2417.0698	2417.0670	1	1	K.MMDKVEFVHMLNATMCATTR.T	2 Oxidation (M)	(Ions score 52)
416 - 435	806.6972	2417.0698	2417.0670	1	1	K.MMDKVEFVHMLNATMCATTR.T	2 Oxidation (M)	(Ions score 60)
416 - 435	806.6972	2417.0698	2417.0670	1	1	K.MMDKVEFVHMLNATMCATTR.T	2 Oxidation (M)	(Ions score 49)
416 - 435	806.6972	2417.0699	2417.0670	1	1	K.MMDKVEFVHMLNATMCATTR.T	2 Oxidation (M)	(Ions score 54)
416 - 435	806.6972	2417.0699	2417.0670	1	1	K.MMDKVEFVHMLNATMCATTR.T	2 Oxidation (M)	(Ions score 46)
416 - 435	806.6973	2417.0699	2417.0670	1	1	K.MMDKVEFVHMLNATMCATTR.T	2 Oxidation (M)	(Ions score 60)
416 - 435	806.6973	2417.0699	2417.0670	1	1	K.MMDKVEFVHMLNATMCATTR.T	2 Oxidation (M)	(Ions score 29)
416 - 435	806.6973	2417.0700	2417.0670	1	1	K.MMDKVEFVHMLNATMCATTR.T	2 Oxidation (M)	(Ions score 52)
416 - 435	806.6973	2417.0700	2417.0670	1	1	K.MMDKVEFVHMLNATMCATTR.T	2 Oxidation (M)	(Ions score 53)
416 - 435	605.2748	2417.0700	2417.0670	1	1	K.MMDKVEFVHMLNATMCATTR.T	2 Oxidation (M)	(Ions score 54)
416 - 435	806.6973	2417.0700	2417.0670	1	1	K.MMDKVEFVHMLNATMCATTR.T	2 Oxidation (M)	(Ions score 49)
416 - 435	806.6973	2417.0701	2417.0670	1	1	K.MMDKVEFVHMLNATMCATTR.T	2 Oxidation (M)	(Ions score 49)

416 - 435	806.6973	2417.0701	2417.0670	1	1	K.MMDKVEFVHMLNATMCATTR.T	2 Oxidation (M)	(Ions score 56)
416 - 435	806.6973	2417.0702	2417.0670	1	1	K.MMDKVEFVHMLNATMCATTR.T	2 Oxidation (M)	(Ions score 48)
416 - 435	806.6973	2417.0702	2417.0670	1	1	K.MMDKVEFVHMLNATMCATTR.T	2 Oxidation (M)	(Ions score 45)
416 - 435	806.6974	2417.0704	2417.0670	1	1	K.MMDKVEFVHMLNATMCATTR.T	2 Oxidation (M)	(Ions score 75)
416 - 435	806.6974	2417.0704	2417.0670	1	1	K.MMDKVEFVHMLNATMCATTR.T	2 Oxidation (M)	(Ions score 46)
416 - 435	806.6974	2417.0704	2417.0670	1	1	K.MMDKVEFVHMLNATMCATTR.T	2 Oxidation (M)	(Ions score 71)
416 - 435	806.6974	2417.0704	2417.0670	1	1	K.MMDKVEFVHMLNATMCATTR.T	2 Oxidation (M)	(Ions score 45)
416 - 435	806.6974	2417.0705	2417.0670	1	1	K.MMDKVEFVHMLNATMCATTR.T	2 Oxidation (M)	(Ions score 25)
416 - 435	605.2749	2417.0705	2417.0670	1	1	K.MMDKVEFVHMLNATMCATTR.T	2 Oxidation (M)	(Ions score 23)
416 - 435	806.6974	2417.0705	2417.0670	1	1	K.MMDKVEFVHMLNATMCATTR.T	2 Oxidation (M)	(Ions score 33)
416 - 435	806.6975	2417.0705	2417.0670	1	1	K.MMDKVEFVHMLNATMCATTR.T	2 Oxidation (M)	(Ions score 23)
416 - 435	806.6975	2417.0705	2417.0670	1	1	K.MMDKVEFVHMLNATMCATTR.T	2 Oxidation (M)	(Ions score 48)
416 - 435	1209.5426	2417.0706	2417.0670	1	1	K.MMDKVEFVHMLNATMCATTR.T	2 Oxidation (M)	(Ions score 60)
416 - 435	806.6975	2417.0706	2417.0670	2	1	K.MMDKVEFVHMLNATMCATTR.T	2 Oxidation (M)	(Ions score 60)
416 - 435	806.6975	2417.0706	2417.0670	2	1	K.MMDKVEFVHMLNATMCATTR.T	2 Oxidation (M)	(Ions score 40)
416 - 435	806.6975	2417.0707	2417.0670	2	1	K.MMDKVEFVHMLNATMCATTR.T	2 Oxidation (M)	(Ions score 59)
416 - 435	605.2749	2417.0707	2417.0670	2	1	K.MMDKVEFVHMLNATMCATTR.T	2 Oxidation (M)	(Ions score 22)
416 - 435	806.6975	2417.0707	2417.0670	2	1	K.MMDKVEFVHMLNATMCATTR.T	2 Oxidation (M)	(Ions score 36)
416 - 435	806.6975	2417.0707	2417.0670	2	1	K.MMDKVEFVHMLNATMCATTR.T	2 Oxidation (M)	(Ions score 50)
416 - 435	806.6975	2417.0707	2417.0670	2	1	K.MMDKVEFVHMLNATMCATTR.T	2 Oxidation (M)	(Ions score 21)
416 - 435	605.2750	2417.0707	2417.0670	2	1	K.MMDKVEFVHMLNATMCATTR.T	2 Oxidation (M)	(Ions score 51)
416 - 435	806.6976	2417.0709	2417.0670	2	1	K.MMDKVEFVHMLNATMCATTR.T	2 Oxidation (M)	(Ions score 59)
416 - 435	806.6976	2417.0709	2417.0670	2	1	K.MMDKVEFVHMLNATMCATTR.T	2 Oxidation (M)	(Ions score 49)
416 - 435	806.6976	2417.0709	2417.0670	2	1	K.MMDKVEFVHMLNATMCATTR.T	2 Oxidation (M)	(Ions score 60)
416 - 435	806.6976	2417.0709	2417.0670	2	1	K.MMDKVEFVHMLNATMCATTR.T	2 Oxidation (M)	(Ions score 46)
416 - 435	1209.5428	2417.0710	2417.0670	2	1	K.MMDKVEFVHMLNATMCATTR.T	2 Oxidation (M)	(Ions score 95)
416 - 435	806.6976	2417.0711	2417.0670	2	1	K.MMDKVEFVHMLNATMCATTR.T	2 Oxidation (M)	(Ions score 49)
416 - 435	806.6976	2417.0711	2417.0670	2	1	K.MMDKVEFVHMLNATMCATTR.T	2 Oxidation (M)	(Ions score 40)
416 - 435	806.6977	2417.0711	2417.0670	2	1	K.MMDKVEFVHMLNATMCATTR.T	2 Oxidation (M)	(Ions score 46)
416 - 435	806.6977	2417.0712	2417.0670	2	1	K.MMDKVEFVHMLNATMCATTR.T	2 Oxidation (M)	(Ions score 46)
416 - 435	605.2751	2417.0712	2417.0670	2	1	K.MMDKVEFVHMLNATMCATTR.T	2 Oxidation (M)	(Ions score 23)
416 - 435	806.6977	2417.0713	2417.0670	2	1	K.MMDKVEFVHMLNATMCATTR.T	2 Oxidation (M)	(Ions score 50)
416 - 435	806.6977	2417.0713	2417.0670	2	1	K.MMDKVEFVHMLNATMCATTR.T	2 Oxidation (M)	(Ions score 36)
416 - 435	605.2751	2417.0713	2417.0670	2	1	K.MMDKVEFVHMLNATMCATTR.T	2 Oxidation (M)	(Ions score 23)
416 - 435	806.6977	2417.0713	2417.0670	2	1	K.MMDKVEFVHMLNATMCATTR.T	2 Oxidation (M)	(Ions score 38)
416 - 435	806.6977	2417.0713	2417.0670	2	1	K.MMDKVEFVHMLNATMCATTR.T	2 Oxidation (M)	(Ions score 35)
416 - 435	806.6977	2417.0714	2417.0670	2	1	K.MMDKVEFVHMLNATMCATTR.T	2 Oxidation (M)	(Ions score 57)
416 - 435	806.6977	2417.0714	2417.0670	2	1	K.MMDKVEFVHMLNATMCATTR.T	2 Oxidation (M)	(Ions score 60)
416 - 435	806.6978	2417.0714	2417.0670	2	1	K.MMDKVEFVHMLNATMCATTR.T	2 Oxidation (M)	(Ions score 33)
416 - 435	806.6978	2417.0714	2417.0670	2	1	K.MMDKVEFVHMLNATMCATTR.T	2 Oxidation (M)	(Ions score 36)
416 - 435	806.6978	2417.0714	2417.0670	2	1	K.MMDKVEFVHMLNATMCATTR.T	2 Oxidation (M)	(Ions score 44)
416 - 435	806.6978	2417.0714	2417.0670	2	1	K.MMDKVEFVHMLNATMCATTR.T	2 Oxidation (M)	(Ions score 54)
416 - 435	605.2751	2417.0715	2417.0670	2	1	K.MMDKVEFVHMLNATMCATTR.T	2 Oxidation (M)	(Ions score 23)
416 - 435	806.6978	2417.0715	2417.0670	2	1	K.MMDKVEFVHMLNATMCATTR.T	2 Oxidation (M)	(Ions score 41)
416 - 435	806.6978	2417.0715	2417.0670	2	1	K.MMDKVEFVHMLNATMCATTR.T	2 Oxidation (M)	(Ions score 68)
416 - 435	605.2752	2417.0715	2417.0670	2	1	K.MMDKVEFVHMLNATMCATTR.T	2 Oxidation (M)	(Ions score 23)
416 - 435	806.6978	2417.0716	2417.0670	2	1	K.MMDKVEFVHMLNATMCATTR.T	2 Oxidation (M)	(Ions score 61)
416 - 435	605.2752	2417.0716	2417.0670	2	1	K.MMDKVEFVHMLNATMCATTR.T	2 Oxidation (M)	(Ions score 22)
416 - 435	806.6978	2417.0716	2417.0670	2	1	K.MMDKVEFVHMLNATMCATTR.T	2 Oxidation (M)	(Ions score 60)
416 - 435	806.6978	2417.0717	2417.0670	2	1	K.MMDKVEFVHMLNATMCATTR.T	2 Oxidation (M)	(Ions score 60)
416 - 435	806.6979	2417.0717	2417.0670	2	1	K.MMDKVEFVHMLNATMCATTR.T	2 Oxidation (M)	(Ions score 34)
416 - 435	806.6979	2417.0718	2417.0670	2	1	K.MMDKVEFVHMLNATMCATTR.T	2 Oxidation (M)	(Ions score 23)
416 - 435	806.6979	2417.0718	2417.0670	2	1	K.MMDKVEFVHMLNATMCATTR.T	2 Oxidation (M)	(Ions score 53)
416 - 435	806.6979	2417.0718	2417.0670	2	1	K.MMDKVEFVHMLNATMCATTR.T	2 Oxidation (M)	(Ions score 36)
416 - 435	806.6979	2417.0718	2417.0670	2	1	K.MMDKVEFVHMLNATMCATTR.T	2 Oxidation (M)	(Ions score 46)
416 - 435	806.6979	2417.0718	2417.0670	2	1	K.MMDKVEFVHMLNATMCATTR.T	2 Oxidation (M)	(Ions score 58)
416 - 435	806.6979	2417.0718	2417.0670	2	1	K.MMDKVEFVHMLNATMCATTR.T	2 Oxidation (M)	(Ions score 57)

416 - 435	806.6979	2417.0718	2417.0670	2	1	K.MMDKVEFVHMLNATMCATTR.T	2 Oxidation (M)	(Ions score 54)
416 - 435	806.6979	2417.0718	2417.0670	2	1	K.MMDKVEFVHMLNATMCATTR.T	2 Oxidation (M)	(Ions score 27)
416 - 435	1209.5432	2417.0719	2417.0670	2	1	K.MMDKVEFVHMLNATMCATTR.T	2 Oxidation (M)	(Ions score 99)
416 - 435	806.6979	2417.0719	2417.0670	2	1	K.MMDKVEFVHMLNATMCATTR.T	2 Oxidation (M)	(Ions score 37)
416 - 435	806.6979	2417.0719	2417.0670	2	1	K.MMDKVEFVHMLNATMCATTR.T	2 Oxidation (M)	(Ions score 51)
416 - 435	806.6979	2417.0719	2417.0670	2	1	K.MMDKVEFVHMLNATMCATTR.T	2 Oxidation (M)	(Ions score 36)
416 - 435	806.6979	2417.0719	2417.0670	2	1	K.MMDKVEFVHMLNATMCATTR.T	2 Oxidation (M)	(Ions score 38)
416 - 435	605.2753	2417.0720	2417.0670	2	1	K.MMDKVEFVHMLNATMCATTR.T	2 Oxidation (M)	(Ions score 23)
416 - 435	806.6979	2417.0720	2417.0670	2	1	K.MMDKVEFVHMLNATMCATTR.T	2 Oxidation (M)	(Ions score 33)
416 - 435	806.6979	2417.0720	2417.0670	2	1	K.MMDKVEFVHMLNATMCATTR.T	2 Oxidation (M)	(Ions score 60)
416 - 435	806.6979	2417.0720	2417.0670	2	1	K.MMDKVEFVHMLNATMCATTR.T	2 Oxidation (M)	(Ions score 57)
416 - 435	806.6979	2417.0720	2417.0670	2	1	K.MMDKVEFVHMLNATMCATTR.T	2 Oxidation (M)	(Ions score 36)
416 - 435	806.6980	2417.0720	2417.0670	2	1	K.MMDKVEFVHMLNATMCATTR.T	2 Oxidation (M)	(Ions score 58)
416 - 435	605.2753	2417.0721	2417.0670	2	1	K.MMDKVEFVHMLNATMCATTR.T	2 Oxidation (M)	(Ions score 28)
416 - 435	806.6980	2417.0721	2417.0670	2	1	K.MMDKVEFVHMLNATMCATTR.T	2 Oxidation (M)	(Ions score 29)
416 - 435	605.2753	2417.0721	2417.0670	2	1	K.MMDKVEFVHMLNATMCATTR.T	2 Oxidation (M)	(Ions score 22)
416 - 435	806.6980	2417.0721	2417.0670	2	1	K.MMDKVEFVHMLNATMCATTR.T	2 Oxidation (M)	(Ions score 60)
416 - 435	806.6980	2417.0722	2417.0670	2	1	K.MMDKVEFVHMLNATMCATTR.T	2 Oxidation (M)	(Ions score 24)
416 - 435	806.6980	2417.0722	2417.0670	2	1	K.MMDKVEFVHMLNATMCATTR.T	2 Oxidation (M)	(Ions score 60)
416 - 435	806.6980	2417.0722	2417.0670	2	1	K.MMDKVEFVHMLNATMCATTR.T	2 Oxidation (M)	(Ions score 26)
416 - 435	605.2753	2417.0722	2417.0670	2	1	K.MMDKVEFVHMLNATMCATTR.T	2 Oxidation (M)	(Ions score 30)
416 - 435	605.2753	2417.0722	2417.0670	2	1	K.MMDKVEFVHMLNATMCATTR.T	2 Oxidation (M)	(Ions score 22)
416 - 435	806.6980	2417.0722	2417.0670	2	1	K.MMDKVEFVHMLNATMCATTR.T	2 Oxidation (M)	(Ions score 28)
416 - 435	605.2753	2417.0723	2417.0670	2	1	K.MMDKVEFVHMLNATMCATTR.T	2 Oxidation (M)	(Ions score 23)
416 - 435	605.2753	2417.0723	2417.0670	2	1	K.MMDKVEFVHMLNATMCATTR.T	2 Oxidation (M)	(Ions score 23)
416 - 435	806.6980	2417.0723	2417.0670	2	1	K.MMDKVEFVHMLNATMCATTR.T	2 Oxidation (M)	(Ions score 60)
416 - 435	605.2754	2417.0723	2417.0670	2	1	K.MMDKVEFVHMLNATMCATTR.T	2 Oxidation (M)	(Ions score 31)
416 - 435	806.6981	2417.0723	2417.0670	2	1	K.MMDKVEFVHMLNATMCATTR.T	2 Oxidation (M)	(Ions score 32)
416 - 435	806.6981	2417.0724	2417.0670	2	1	K.MMDKVEFVHMLNATMCATTR.T	2 Oxidation (M)	(Ions score 48)
416 - 435	806.6981	2417.0724	2417.0670	2	1	K.MMDKVEFVHMLNATMCATTR.T	2 Oxidation (M)	(Ions score 54)
416 - 435	806.6981	2417.0724	2417.0670	2	1	K.MMDKVEFVHMLNATMCATTR.T	2 Oxidation (M)	(Ions score 56)
416 - 435	806.6981	2417.0725	2417.0670	2	1	K.MMDKVEFVHMLNATMCATTR.T	2 Oxidation (M)	(Ions score 60)
416 - 435	806.6981	2417.0725	2417.0670	2	1	K.MMDKVEFVHMLNATMCATTR.T	2 Oxidation (M)	(Ions score 46)
416 - 435	806.6981	2417.0725	2417.0670	2	1	K.MMDKVEFVHMLNATMCATTR.T	2 Oxidation (M)	(Ions score 46)
416 - 435	806.6981	2417.0725	2417.0670	2	1	K.MMDKVEFVHMLNATMCATTR.T	2 Oxidation (M)	(Ions score 49)
416 - 435	806.6981	2417.0725	2417.0670	2	1	K.MMDKVEFVHMLNATMCATTR.T	2 Oxidation (M)	(Ions score 34)
416 - 435	806.6981	2417.0726	2417.0670	2	1	K.MMDKVEFVHMLNATMCATTR.T	2 Oxidation (M)	(Ions score 46)
416 - 435	806.6981	2417.0726	2417.0670	2	1	K.MMDKVEFVHMLNATMCATTR.T	2 Oxidation (M)	(Ions score 60)
416 - 435	605.2754	2417.0726	2417.0670	2	1	K.MMDKVEFVHMLNATMCATTR.T	2 Oxidation (M)	(Ions score 28)
416 - 435	1209.5436	2417.0727	2417.0670	2	1	K.MMDKVEFVHMLNATMCATTR.T	2 Oxidation (M)	(Ions score 73)
416 - 435	806.6982	2417.0727	2417.0670	2	1	K.MMDKVEFVHMLNATMCATTR.T	2 Oxidation (M)	(Ions score 28)
416 - 435	806.6982	2417.0727	2417.0670	2	1	K.MMDKVEFVHMLNATMCATTR.T	2 Oxidation (M)	(Ions score 42)
416 - 435	806.6982	2417.0727	2417.0670	2	1	K.MMDKVEFVHMLNATMCATTR.T	2 Oxidation (M)	(Ions score 46)
416 - 435	605.2755	2417.0728	2417.0670	2	1	K.MMDKVEFVHMLNATMCATTR.T	2 Oxidation (M)	(Ions score 23)
416 - 435	806.6982	2417.0728	2417.0670	2	1	K.MMDKVEFVHMLNATMCATTR.T	2 Oxidation (M)	(Ions score 60)
416 - 435	1209.5437	2417.0728	2417.0670	2	1	K.MMDKVEFVHMLNATMCATTR.T	2 Oxidation (M)	(Ions score 68)
416 - 435	605.2755	2417.0729	2417.0670	2	1	K.MMDKVEFVHMLNATMCATTR.T	2 Oxidation (M)	(Ions score 22)
416 - 435	1209.5437	2417.0729	2417.0670	2	1	K.MMDKVEFVHMLNATMCATTR.T	2 Oxidation (M)	(Ions score 71)
416 - 435	806.6982	2417.0729	2417.0670	2	1	K.MMDKVEFVHMLNATMCATTR.T	2 Oxidation (M)	(Ions score 68)
416 - 435	806.6983	2417.0729	2417.0670	2	1	K.MMDKVEFVHMLNATMCATTR.T	2 Oxidation (M)	(Ions score 64)
416 - 435	806.6983	2417.0730	2417.0670	2	1	K.MMDKVEFVHMLNATMCATTR.T	2 Oxidation (M)	(Ions score 46)
416 - 435	1209.5438	2417.0730	2417.0670	2	1	K.MMDKVEFVHMLNATMCATTR.T	2 Oxidation (M)	(Ions score 61)
416 - 435	806.6983	2417.0730	2417.0670	2	1	K.MMDKVEFVHMLNATMCATTR.T	2 Oxidation (M)	(Ions score 54)
416 - 435	806.6983	2417.0731	2417.0670	3	1	K.MMDKVEFVHMLNATMCATTR.T	2 Oxidation (M)	(Ions score 63)
416 - 435	806.6983	2417.0731	2417.0670	3	1	K.MMDKVEFVHMLNATMCATTR.T	2 Oxidation (M)	(Ions score 46)
416 - 435	605.2756	2417.0732	2417.0670	3	1	K.MMDKVEFVHMLNATMCATTR.T	2 Oxidation (M)	(Ions score 24)
416 - 435	806.6983	2417.0732	2417.0670	3	1	K.MMDKVEFVHMLNATMCATTR.T	2 Oxidation (M)	(Ions score 30)

416 - 435	605.2756	2417.0732	2417.0670	3	1	K.MMDKVEFVHMLNATMCATTR.T	2 Oxidation (M)	(Ions score 23)
416 - 435	806.6984	2417.0732	2417.0670	3	1	K.MMDKVEFVHMLNATMCATTR.T	2 Oxidation (M)	(Ions score 75)
416 - 435	605.2756	2417.0733	2417.0670	3	1	K.MMDKVEFVHMLNATMCATTR.T	2 Oxidation (M)	(Ions score 22)
416 - 435	806.6984	2417.0733	2417.0670	3	1	K.MMDKVEFVHMLNATMCATTR.T	2 Oxidation (M)	(Ions score 40)
416 - 435	806.6984	2417.0733	2417.0670	3	1	K.MMDKVEFVHMLNATMCATTR.T	2 Oxidation (M)	(Ions score 48)
416 - 435	806.6984	2417.0733	2417.0670	3	1	K.MMDKVEFVHMLNATMCATTR.T	2 Oxidation (M)	(Ions score 22)
416 - 435	1209.5439	2417.0733	2417.0670	3	1	K.MMDKVEFVHMLNATMCATTR.T	2 Oxidation (M)	(Ions score 100)
416 - 435	806.6984	2417.0733	2417.0670	3	1	K.MMDKVEFVHMLNATMCATTR.T	2 Oxidation (M)	(Ions score 56)
416 - 435	806.6984	2417.0734	2417.0670	3	1	K.MMDKVEFVHMLNATMCATTR.T	2 Oxidation (M)	(Ions score 30)
416 - 435	806.6984	2417.0734	2417.0670	3	1	K.MMDKVEFVHMLNATMCATTR.T	2 Oxidation (M)	(Ions score 36)
416 - 435	806.6984	2417.0735	2417.0670	3	1	K.MMDKVEFVHMLNATMCATTR.T	2 Oxidation (M)	(Ions score 33)
416 - 435	806.6984	2417.0735	2417.0670	3	1	K.MMDKVEFVHMLNATMCATTR.T	2 Oxidation (M)	(Ions score 57)
416 - 435	806.6984	2417.0735	2417.0670	3	1	K.MMDKVEFVHMLNATMCATTR.T	2 Oxidation (M)	(Ions score 60)
416 - 435	806.6984	2417.0735	2417.0670	3	1	K.MMDKVEFVHMLNATMCATTR.T	2 Oxidation (M)	(Ions score 49)
416 - 435	806.6985	2417.0735	2417.0670	3	1	K.MMDKVEFVHMLNATMCATTR.T	2 Oxidation (M)	(Ions score 59)
416 - 435	806.6985	2417.0736	2417.0670	3	1	K.MMDKVEFVHMLNATMCATTR.T	2 Oxidation (M)	(Ions score 57)
416 - 435	605.2757	2417.0736	2417.0670	3	1	K.MMDKVEFVHMLNATMCATTR.T	2 Oxidation (M)	(Ions score 22)
416 - 435	806.6985	2417.0736	2417.0670	3	1	K.MMDKVEFVHMLNATMCATTR.T	2 Oxidation (M)	(Ions score 46)
416 - 435	605.2757	2417.0736	2417.0670	3	1	K.MMDKVEFVHMLNATMCATTR.T	2 Oxidation (M)	(Ions score 23)
416 - 435	806.6985	2417.0736	2417.0670	3	1	K.MMDKVEFVHMLNATMCATTR.T	2 Oxidation (M)	(Ions score 57)
416 - 435	806.6985	2417.0737	2417.0670	3	1	K.MMDKVEFVHMLNATMCATTR.T	2 Oxidation (M)	(Ions score 37)
416 - 435	806.6985	2417.0738	2417.0670	3	1	K.MMDKVEFVHMLNATMCATTR.T	2 Oxidation (M)	(Ions score 75)
416 - 435	806.6985	2417.0738	2417.0670	3	1	K.MMDKVEFVHMLNATMCATTR.T	2 Oxidation (M)	(Ions score 59)
416 - 435	806.6985	2417.0738	2417.0670	3	1	K.MMDKVEFVHMLNATMCATTR.T	2 Oxidation (M)	(Ions score 41)
416 - 435	1209.5442	2417.0738	2417.0670	3	1	K.MMDKVEFVHMLNATMCATTR.T	2 Oxidation (M)	(Ions score 58)
416 - 435	806.6986	2417.0738	2417.0670	3	1	K.MMDKVEFVHMLNATMCATTR.T	2 Oxidation (M)	(Ions score 40)
416 - 435	806.6986	2417.0738	2417.0670	3	1	K.MMDKVEFVHMLNATMCATTR.T	2 Oxidation (M)	(Ions score 37)
416 - 435	806.6986	2417.0738	2417.0670	3	1	K.MMDKVEFVHMLNATMCATTR.T	2 Oxidation (M)	(Ions score 67)
416 - 435	806.6986	2417.0739	2417.0670	3	1	K.MMDKVEFVHMLNATMCATTR.T	2 Oxidation (M)	(Ions score 46)
416 - 435	806.6986	2417.0739	2417.0670	3	1	K.MMDKVEFVHMLNATMCATTR.T	2 Oxidation (M)	(Ions score 49)
416 - 435	1209.5442	2417.0739	2417.0670	3	1	K.MMDKVEFVHMLNATMCATTR.T	2 Oxidation (M)	(Ions score 44)
416 - 435	806.6986	2417.0740	2417.0670	3	1	K.MMDKVEFVHMLNATMCATTR.T	2 Oxidation (M)	(Ions score 60)
416 - 435	806.6986	2417.0740	2417.0670	3	1	K.MMDKVEFVHMLNATMCATTR.T	2 Oxidation (M)	(Ions score 47)
416 - 435	806.6986	2417.0740	2417.0670	3	1	K.MMDKVEFVHMLNATMCATTR.T	2 Oxidation (M)	(Ions score 60)
416 - 435	605.2758	2417.0741	2417.0670	3	1	K.MMDKVEFVHMLNATMCATTR.T	2 Oxidation (M)	(Ions score 21)
416 - 435	806.6987	2417.0741	2417.0670	3	1	K.MMDKVEFVHMLNATMCATTR.T	2 Oxidation (M)	(Ions score 46)
416 - 435	806.6987	2417.0742	2417.0670	3	1	K.MMDKVEFVHMLNATMCATTR.T	2 Oxidation (M)	(Ions score 47)
416 - 435	806.6987	2417.0742	2417.0670	3	1	K.MMDKVEFVHMLNATMCATTR.T	2 Oxidation (M)	(Ions score 33)
416 - 435	806.6987	2417.0742	2417.0670	3	1	K.MMDKVEFVHMLNATMCATTR.T	2 Oxidation (M)	(Ions score 55)
416 - 435	806.6987	2417.0743	2417.0670	3	1	K.MMDKVEFVHMLNATMCATTR.T	2 Oxidation (M)	(Ions score 57)
416 - 435	806.6987	2417.0743	2417.0670	3	1	K.MMDKVEFVHMLNATMCATTR.T	2 Oxidation (M)	(Ions score 47)
416 - 435	806.6987	2417.0744	2417.0670	3	1	K.MMDKVEFVHMLNATMCATTR.T	2 Oxidation (M)	(Ions score 35)
416 - 435	605.2759	2417.0744	2417.0670	3	1	K.MMDKVEFVHMLNATMCATTR.T	2 Oxidation (M)	(Ions score 22)
416 - 435	806.6987	2417.0744	2417.0670	3	1	K.MMDKVEFVHMLNATMCATTR.T	2 Oxidation (M)	(Ions score 47)
416 - 435	806.6987	2417.0744	2417.0670	3	1	K.MMDKVEFVHMLNATMCATTR.T	2 Oxidation (M)	(Ions score 60)
416 - 435	806.6987	2417.0744	2417.0670	3	1	K.MMDKVEFVHMLNATMCATTR.T	2 Oxidation (M)	(Ions score 46)
416 - 435	1209.5445	2417.0745	2417.0670	3	1	K.MMDKVEFVHMLNATMCATTR.T	2 Oxidation (M)	(Ions score 53)
416 - 435	806.6988	2417.0745	2417.0670	3	1	K.MMDKVEFVHMLNATMCATTR.T	2 Oxidation (M)	(Ions score 49)
416 - 435	806.6988	2417.0745	2417.0670	3	1	K.MMDKVEFVHMLNATMCATTR.T	2 Oxidation (M)	(Ions score 22)
416 - 435	806.6988	2417.0745	2417.0670	3	1	K.MMDKVEFVHMLNATMCATTR.T	2 Oxidation (M)	(Ions score 63)
416 - 435	806.6988	2417.0745	2417.0670	3	1	K.MMDKVEFVHMLNATMCATTR.T	2 Oxidation (M)	(Ions score 25)
416 - 435	806.6988	2417.0746	2417.0670	3	1	K.MMDKVEFVHMLNATMCATTR.T	2 Oxidation (M)	(Ions score 50)
416 - 435	806.6988	2417.0746	2417.0670	3	1	K.MMDKVEFVHMLNATMCATTR.T	2 Oxidation (M)	(Ions score 58)
416 - 435	806.6989	2417.0748	2417.0670	3	1	K.MMDKVEFVHMLNATMCATTR.T	2 Oxidation (M)	(Ions score 35)
416 - 435	605.2760	2417.0749	2417.0670	3	1	K.MMDKVEFVHMLNATMCATTR.T	2 Oxidation (M)	(Ions score 22)
416 - 435	806.6989	2417.0749	2417.0670	3	1	K.MMDKVEFVHMLNATMCATTR.T	2 Oxidation (M)	(Ions score 51)
416 - 435	806.6989	2417.0750	2417.0670	3	1	K.MMDKVEFVHMLNATMCATTR.T	2 Oxidation (M)	(Ions score 50)

416 - 435	806.6989	2417.0750	2417.0670	3	1	K.MMDKVEFVHMLNATMCATTR.T	2 Oxidation (M)	(Ions score 39)
416 - 435	1209.5448	2417.0751	2417.0670	3	1	K.MMDKVEFVHMLNATMCATTR.T	2 Oxidation (M)	(Ions score 52)
416 - 435	806.6990	2417.0751	2417.0670	3	1	K.MMDKVEFVHMLNATMCATTR.T	2 Oxidation (M)	(Ions score 40)
416 - 435	806.6990	2417.0751	2417.0670	3	1	K.MMDKVEFVHMLNATMCATTR.T	2 Oxidation (M)	(Ions score 36)
416 - 435	806.6990	2417.0751	2417.0670	3	1	K.MMDKVEFVHMLNATMCATTR.T	2 Oxidation (M)	(Ions score 36)
416 - 435	806.6990	2417.0751	2417.0670	3	1	K.MMDKVEFVHMLNATMCATTR.T	2 Oxidation (M)	(Ions score 49)
416 - 435	806.6990	2417.0752	2417.0670	3	1	K.MMDKVEFVHMLNATMCATTR.T	2 Oxidation (M)	(Ions score 35)
416 - 435	605.2761	2417.0753	2417.0670	3	1	K.MMDKVEFVHMLNATMCATTR.T	2 Oxidation (M)	(Ions score 24)
416 - 435	806.6990	2417.0753	2417.0670	3	1	K.MMDKVEFVHMLNATMCATTR.T	2 Oxidation (M)	(Ions score 58)
416 - 435	806.6991	2417.0753	2417.0670	3	1	K.MMDKVEFVHMLNATMCATTR.T	2 Oxidation (M)	(Ions score 31)
416 - 435	605.2761	2417.0753	2417.0670	3	1	K.MMDKVEFVHMLNATMCATTR.T	2 Oxidation (M)	(Ions score 21)
416 - 435	605.2762	2417.0756	2417.0670	4	1	K.MMDKVEFVHMLNATMCATTR.T	2 Oxidation (M)	(Ions score 21)
416 - 435	806.6991	2417.0756	2417.0670	4	1	K.MMDKVEFVHMLNATMCATTR.T	2 Oxidation (M)	(Ions score 57)
416 - 435	605.2762	2417.0756	2417.0670	4	1	K.MMDKVEFVHMLNATMCATTR.T	2 Oxidation (M)	(Ions score 43)
416 - 435	806.6992	2417.0756	2417.0670	4	1	K.MMDKVEFVHMLNATMCATTR.T	2 Oxidation (M)	(Ions score 45)
416 - 435	806.6992	2417.0756	2417.0670	4	1	K.MMDKVEFVHMLNATMCATTR.T	2 Oxidation (M)	(Ions score 63)
416 - 435	806.6992	2417.0757	2417.0670	4	1	K.MMDKVEFVHMLNATMCATTR.T	2 Oxidation (M)	(Ions score 36)
416 - 435	806.6992	2417.0757	2417.0670	4	1	K.MMDKVEFVHMLNATMCATTR.T	2 Oxidation (M)	(Ions score 45)
416 - 435	806.6992	2417.0758	2417.0670	4	1	K.MMDKVEFVHMLNATMCATTR.T	2 Oxidation (M)	(Ions score 48)
416 - 435	806.6992	2417.0758	2417.0670	4	1	K.MMDKVEFVHMLNATMCATTR.T	2 Oxidation (M)	(Ions score 48)
416 - 435	806.6992	2417.0759	2417.0670	4	1	K.MMDKVEFVHMLNATMCATTR.T	2 Oxidation (M)	(Ions score 57)
416 - 435	806.6992	2417.0759	2417.0670	4	1	K.MMDKVEFVHMLNATMCATTR.T	2 Oxidation (M)	(Ions score 37)
416 - 435	605.2763	2417.0760	2417.0670	4	1	K.MMDKVEFVHMLNATMCATTR.T	2 Oxidation (M)	(Ions score 34)
416 - 435	605.2763	2417.0760	2417.0670	4	1	K.MMDKVEFVHMLNATMCATTR.T	2 Oxidation (M)	(Ions score 21)
416 - 435	806.6993	2417.0761	2417.0670	4	1	K.MMDKVEFVHMLNATMCATTR.T	2 Oxidation (M)	(Ions score 36)
416 - 435	806.6994	2417.0763	2417.0670	4	1	K.MMDKVEFVHMLNATMCATTR.T	2 Oxidation (M)	(Ions score 33)
416 - 435	806.6994	2417.0763	2417.0670	4	1	K.MMDKVEFVHMLNATMCATTR.T	2 Oxidation (M)	(Ions score 36)
416 - 435	1209.5455	2417.0764	2417.0670	4	1	K.MMDKVEFVHMLNATMCATTR.T	2 Oxidation (M)	(Ions score 64)
416 - 435	806.6994	2417.0764	2417.0670	4	1	K.MMDKVEFVHMLNATMCATTR.T	2 Oxidation (M)	(Ions score 49)
416 - 435	806.6994	2417.0764	2417.0670	4	1	K.MMDKVEFVHMLNATMCATTR.T	2 Oxidation (M)	(Ions score 71)
416 - 435	806.6995	2417.0765	2417.0670	4	1	K.MMDKVEFVHMLNATMCATTR.T	2 Oxidation (M)	(Ions score 71)
416 - 435	806.6995	2417.0766	2417.0670	4	1	K.MMDKVEFVHMLNATMCATTR.T	2 Oxidation (M)	(Ions score 22)
416 - 435	806.6995	2417.0766	2417.0670	4	1	K.MMDKVEFVHMLNATMCATTR.T	2 Oxidation (M)	(Ions score 22)
416 - 435	605.2764	2417.0766	2417.0670	4	1	K.MMDKVEFVHMLNATMCATTR.T	2 Oxidation (M)	(Ions score 22)
416 - 435	806.6995	2417.0766	2417.0670	4	1	K.MMDKVEFVHMLNATMCATTR.T	2 Oxidation (M)	(Ions score 41)
416 - 435	806.6995	2417.0767	2417.0670	4	1	K.MMDKVEFVHMLNATMCATTR.T	2 Oxidation (M)	(Ions score 36)
416 - 435	605.2765	2417.0767	2417.0670	4	1	K.MMDKVEFVHMLNATMCATTR.T	2 Oxidation (M)	(Ions score 22)
416 - 435	1209.5457	2417.0768	2417.0670	4	1	K.MMDKVEFVHMLNATMCATTR.T	2 Oxidation (M)	(Ions score 64)
416 - 435	806.6996	2417.0771	2417.0670	4	1	K.MMDKVEFVHMLNATMCATTR.T	2 Oxidation (M)	(Ions score 40)
416 - 435	1209.5458	2417.0771	2417.0670	4	1	K.MMDKVEFVHMLNATMCATTR.T	2 Oxidation (M)	(Ions score 75)
416 - 435	806.6997	2417.0771	2417.0670	4	1	K.MMDKVEFVHMLNATMCATTR.T	2 Oxidation (M)	(Ions score 49)
416 - 435	806.6997	2417.0772	2417.0670	4	1	K.MMDKVEFVHMLNATMCATTR.T	2 Oxidation (M)	(Ions score 36)
416 - 435	806.6997	2417.0772	2417.0670	4	1	K.MMDKVEFVHMLNATMCATTR.T	2 Oxidation (M)	(Ions score 42)
416 - 435	806.6997	2417.0772	2417.0670	4	1	K.MMDKVEFVHMLNATMCATTR.T	2 Oxidation (M)	(Ions score 46)
416 - 435	806.6997	2417.0774	2417.0670	4	1	K.MMDKVEFVHMLNATMCATTR.T	2 Oxidation (M)	(Ions score 36)
416 - 435	1209.5460	2417.0775	2417.0670	4	1	K.MMDKVEFVHMLNATMCATTR.T	2 Oxidation (M)	(Ions score 83)
416 - 435	806.6998	2417.0777	2417.0670	4	1	K.MMDKVEFVHMLNATMCATTR.T	2 Oxidation (M)	(Ions score 42)
416 - 435	806.6998	2417.0777	2417.0670	4	1	K.MMDKVEFVHMLNATMCATTR.T	2 Oxidation (M)	(Ions score 45)
416 - 435	605.2767	2417.0777	2417.0670	4	1	K.MMDKVEFVHMLNATMCATTR.T	2 Oxidation (M)	(Ions score 22)
416 - 435	605.2767	2417.0777	2417.0670	4	1	K.MMDKVEFVHMLNATMCATTR.T	2 Oxidation (M)	(Ions score 22)
416 - 435	806.6999	2417.0778	2417.0670	4	1	K.MMDKVEFVHMLNATMCATTR.T	2 Oxidation (M)	(Ions score 49)
416 - 435	605.2767	2417.0778	2417.0670	4	1	K.MMDKVEFVHMLNATMCATTR.T	2 Oxidation (M)	(Ions score 21)
416 - 435	806.6999	2417.0779	2417.0670	4	1	K.MMDKVEFVHMLNATMCATTR.T	2 Oxidation (M)	(Ions score 39)
416 - 435	605.2767	2417.0779	2417.0670	4	1	K.MMDKVEFVHMLNATMCATTR.T	2 Oxidation (M)	(Ions score 21)
416 - 435	1209.5462	2417.0779	2417.0670	4	1	K.MMDKVEFVHMLNATMCATTR.T	2 Oxidation (M)	(Ions score 97)
416 - 435	806.7000	2417.0782	2417.0670	5	1	K.MMDKVEFVHMLNATMCATTR.T	2 Oxidation (M)	(Ions score 22)
416 - 435	806.7000	2417.0782	2417.0670	5	1	K.MMDKVEFVHMLNATMCATTR.T	2 Oxidation (M)	(Ions score 51)

416 - 435	806.7001	2417.0784	2417.0670	5	1	K.MMDKVEFVHMLNATMCATTR.T	2 Oxidation (M)	(Ions score 54)
416 - 435	605.2769	2417.0784	2417.0670	5	1	K.MMDKVEFVHMLNATMCATTR.T	2 Oxidation (M)	(Ions score 22)
416 - 435	605.2769	2417.0784	2417.0670	5	1	K.MMDKVEFVHMLNATMCATTR.T	2 Oxidation (M)	(Ions score 21)
416 - 435	806.7001	2417.0785	2417.0670	5	1	K.MMDKVEFVHMLNATMCATTR.T	2 Oxidation (M)	(Ions score 56)
416 - 435	605.2769	2417.0787	2417.0670	5	1	K.MMDKVEFVHMLNATMCATTR.T	2 Oxidation (M)	(Ions score 22)
416 - 435	806.7003	2417.0789	2417.0670	5	1	K.MMDKVEFVHMLNATMCATTR.T	2 Oxidation (M)	(Ions score 46)
416 - 435	810.0384	2427.0934	2427.0877	2	1	K.MMDKVEFVHMLNATMCATTR.T	Acetylation (K)	(Ions score 54)
416 - 435	810.0386	2427.0941	2427.0877	3	1	K.MMDKVEFVHMLNATMCATTR.T	Acetylation (K)	(Ions score 52)
416 - 435	609.2701	2433.0515	2433.0619	-4	1	K.MMDKVEFVHMLNATMCATTR.T	3 Oxidation (M)	(Ions score 26)
416 - 435	609.2708	2433.0541	2433.0619	-3	1	K.MMDKVEFVHMLNATMCATTR.T	3 Oxidation (M)	(Ions score 38)
416 - 435	812.0256	2433.0549	2433.0619	-3	1	K.MMDKVEFVHMLNATMCATTR.T	3 Oxidation (M)	(Ions score 35)
416 - 435	812.0261	2433.0564	2433.0619	-2	1	K.MMDKVEFVHMLNATMCATTR.T	3 Oxidation (M)	(Ions score 54)
416 - 435	812.0263	2433.0571	2433.0619	-2	1	K.MMDKVEFVHMLNATMCATTR.T	3 Oxidation (M)	(Ions score 39)
416 - 435	609.2719	2433.0586	2433.0619	-1	1	K.MMDKVEFVHMLNATMCATTR.T	3 Oxidation (M)	(Ions score 29)
416 - 435	812.0273	2433.0600	2433.0619	-1	1	K.MMDKVEFVHMLNATMCATTR.T	3 Oxidation (M)	(Ions score 44)
416 - 435	812.0276	2433.0608	2433.0619	-0	1	K.MMDKVEFVHMLNATMCATTR.T	3 Oxidation (M)	(Ions score 39)
416 - 435	812.0276	2433.0609	2433.0619	-0	1	K.MMDKVEFVHMLNATMCATTR.T	3 Oxidation (M)	(Ions score 32)
416 - 435	812.0276	2433.0611	2433.0619	-0	1	K.MMDKVEFVHMLNATMCATTR.T	3 Oxidation (M)	(Ions score 32)
416 - 435	609.2726	2433.0612	2433.0619	-0	1	K.MMDKVEFVHMLNATMCATTR.T	3 Oxidation (M)	(Ions score 27)
416 - 435	812.0278	2433.0615	2433.0619	-0	1	K.MMDKVEFVHMLNATMCATTR.T	3 Oxidation (M)	(Ions score 56)
416 - 435	812.0278	2433.0615	2433.0619	-0	1	K.MMDKVEFVHMLNATMCATTR.T	3 Oxidation (M)	(Ions score 40)
416 - 435	812.0279	2433.0618	2433.0619	-0	1	K.MMDKVEFVHMLNATMCATTR.T	3 Oxidation (M)	(Ions score 44)
416 - 435	812.0280	2433.0620	2433.0619	0	1	K.MMDKVEFVHMLNATMCATTR.T	3 Oxidation (M)	(Ions score 35)
416 - 435	609.2729	2433.0623	2433.0619	0	1	K.MMDKVEFVHMLNATMCATTR.T	3 Oxidation (M)	(Ions score 25)
416 - 435	812.0281	2433.0624	2433.0619	0	1	K.MMDKVEFVHMLNATMCATTR.T	3 Oxidation (M)	(Ions score 32)
416 - 435	812.0281	2433.0624	2433.0619	0	1	K.MMDKVEFVHMLNATMCATTR.T	3 Oxidation (M)	(Ions score 42)
416 - 435	609.2729	2433.0624	2433.0619	0	1	K.MMDKVEFVHMLNATMCATTR.T	3 Oxidation (M)	(Ions score 27)
416 - 435	609.2729	2433.0625	2433.0619	0	1	K.MMDKVEFVHMLNATMCATTR.T	3 Oxidation (M)	(Ions score 29)
416 - 435	609.2729	2433.0627	2433.0619	0	1	K.MMDKVEFVHMLNATMCATTR.T	3 Oxidation (M)	(Ions score 27)
416 - 435	609.2729	2433.0627	2433.0619	0	1	K.MMDKVEFVHMLNATMCATTR.T	3 Oxidation (M)	(Ions score 27)
416 - 435	812.0282	2433.0628	2433.0619	0	1	K.MMDKVEFVHMLNATMCATTR.T	3 Oxidation (M)	(Ions score 33)
416 - 435	609.2730	2433.0629	2433.0619	0	1	K.MMDKVEFVHMLNATMCATTR.T	3 Oxidation (M)	(Ions score 23)
416 - 435	812.0283	2433.0629	2433.0619	0	1	K.MMDKVEFVHMLNATMCATTR.T	3 Oxidation (M)	(Ions score 34)
416 - 435	812.0283	2433.0631	2433.0619	0	1	K.MMDKVEFVHMLNATMCATTR.T	3 Oxidation (M)	(Ions score 44)
416 - 435	812.0283	2433.0632	2433.0619	1	1	K.MMDKVEFVHMLNATMCATTR.T	3 Oxidation (M)	(Ions score 34)
416 - 435	812.0283	2433.0632	2433.0619	1	1	K.MMDKVEFVHMLNATMCATTR.T	3 Oxidation (M)	(Ions score 40)
416 - 435	812.0284	2433.0633	2433.0619	1	1	K.MMDKVEFVHMLNATMCATTR.T	3 Oxidation (M)	(Ions score 31)
416 - 435	609.2731	2433.0634	2433.0619	1	1	K.MMDKVEFVHMLNATMCATTR.T	3 Oxidation (M)	(Ions score 31)
416 - 435	812.0284	2433.0634	2433.0619	1	1	K.MMDKVEFVHMLNATMCATTR.T	3 Oxidation (M)	(Ions score 39)
416 - 435	812.0285	2433.0636	2433.0619	1	1	K.MMDKVEFVHMLNATMCATTR.T	3 Oxidation (M)	(Ions score 35)
416 - 435	609.2732	2433.0637	2433.0619	1	1	K.MMDKVEFVHMLNATMCATTR.T	3 Oxidation (M)	(Ions score 27)
416 - 435	812.0285	2433.0637	2433.0619	1	1	K.MMDKVEFVHMLNATMCATTR.T	3 Oxidation (M)	(Ions score 50)
416 - 435	812.0285	2433.0638	2433.0619	1	1	K.MMDKVEFVHMLNATMCATTR.T	3 Oxidation (M)	(Ions score 24)
416 - 435	812.0285	2433.0638	2433.0619	1	1	K.MMDKVEFVHMLNATMCATTR.T	3 Oxidation (M)	(Ions score 32)
416 - 435	812.0286	2433.0639	2433.0619	1	1	K.MMDKVEFVHMLNATMCATTR.T	3 Oxidation (M)	(Ions score 45)
416 - 435	609.2733	2433.0639	2433.0619	1	1	K.MMDKVEFVHMLNATMCATTR.T	3 Oxidation (M)	(Ions score 25)
416 - 435	812.0286	2433.0640	2433.0619	1	1	K.MMDKVEFVHMLNATMCATTR.T	3 Oxidation (M)	(Ions score 31)
416 - 435	812.0286	2433.0641	2433.0619	1	1	K.MMDKVEFVHMLNATMCATTR.T	3 Oxidation (M)	(Ions score 34)
416 - 435	812.0287	2433.0643	2433.0619	1	1	K.MMDKVEFVHMLNATMCATTR.T	3 Oxidation (M)	(Ions score 24)
416 - 435	812.0287	2433.0643	2433.0619	1	1	K.MMDKVEFVHMLNATMCATTR.T	3 Oxidation (M)	(Ions score 27)
416 - 435	812.0288	2433.0645	2433.0619	1	1	K.MMDKVEFVHMLNATMCATTR.T	3 Oxidation (M)	(Ions score 31)
416 - 435	1217.5396	2433.0646	2433.0619	1	1	K.MMDKVEFVHMLNATMCATTR.T	3 Oxidation (M)	(Ions score 48)
416 - 435	812.0288	2433.0646	2433.0619	1	1	K.MMDKVEFVHMLNATMCATTR.T	3 Oxidation (M)	(Ions score 21)
416 - 435	812.0288	2433.0646	2433.0619	1	1	K.MMDKVEFVHMLNATMCATTR.T	3 Oxidation (M)	(Ions score 44)
416 - 435	609.2734	2433.0646	2433.0619	1	1	K.MMDKVEFVHMLNATMCATTR.T	3 Oxidation (M)	(Ions score 29)
416 - 435	812.0288	2433.0647	2433.0619	1	1	K.MMDKVEFVHMLNATMCATTR.T	3 Oxidation (M)	(Ions score 40)
416 - 435	812.0289	2433.0648	2433.0619	1	1	K.MMDKVEFVHMLNATMCATTR.T	3 Oxidation (M)	(Ions score 57)

416 - 435	1217.5397	2433.0649	2433.0619	1	1	K.MMDKVEFVHMLNATMCATTR.T	3 Oxidation (M)	(Ions score 59)
416 - 435	609.2735	2433.0649	2433.0619	1	1	K.MMDKVEFVHMLNATMCATTR.T	3 Oxidation (M)	(Ions score 21)
416 - 435	812.0289	2433.0649	2433.0619	1	1	K.MMDKVEFVHMLNATMCATTR.T	3 Oxidation (M)	(Ions score 35)
416 - 435	812.0289	2433.0649	2433.0619	1	1	K.MMDKVEFVHMLNATMCATTR.T	3 Oxidation (M)	(Ions score 40)
416 - 435	812.0289	2433.0650	2433.0619	1	1	K.MMDKVEFVHMLNATMCATTR.T	3 Oxidation (M)	(Ions score 30)
416 - 435	812.0289	2433.0650	2433.0619	1	1	K.MMDKVEFVHMLNATMCATTR.T	3 Oxidation (M)	(Ions score 21)
416 - 435	812.0290	2433.0650	2433.0619	1	1	K.MMDKVEFVHMLNATMCATTR.T	3 Oxidation (M)	(Ions score 28)
416 - 435	609.2736	2433.0651	2433.0619	1	1	K.MMDKVEFVHMLNATMCATTR.T	3 Oxidation (M)	(Ions score 23)
416 - 435	812.0290	2433.0651	2433.0619	1	1	K.MMDKVEFVHMLNATMCATTR.T	3 Oxidation (M)	(Ions score 50)
416 - 435	812.0290	2433.0651	2433.0619	1	1	K.MMDKVEFVHMLNATMCATTR.T	3 Oxidation (M)	(Ions score 56)
416 - 435	812.0290	2433.0651	2433.0619	1	1	K.MMDKVEFVHMLNATMCATTR.T	3 Oxidation (M)	(Ions score 34)
416 - 435	812.0290	2433.0652	2433.0619	1	1	K.MMDKVEFVHMLNATMCATTR.T	3 Oxidation (M)	(Ions score 45)
416 - 435	812.0290	2433.0653	2433.0619	1	1	K.MMDKVEFVHMLNATMCATTR.T	3 Oxidation (M)	(Ions score 37)
416 - 435	812.0291	2433.0656	2433.0619	2	1	K.MMDKVEFVHMLNATMCATTR.T	3 Oxidation (M)	(Ions score 44)
416 - 435	812.0292	2433.0657	2433.0619	2	1	K.MMDKVEFVHMLNATMCATTR.T	3 Oxidation (M)	(Ions score 31)
416 - 435	812.0292	2433.0657	2433.0619	2	1	K.MMDKVEFVHMLNATMCATTR.T	3 Oxidation (M)	(Ions score 39)
416 - 435	609.2737	2433.0657	2433.0619	2	1	K.MMDKVEFVHMLNATMCATTR.T	3 Oxidation (M)	(Ions score 22)
416 - 435	1217.5402	2433.0658	2433.0619	2	1	K.MMDKVEFVHMLNATMCATTR.T	3 Oxidation (M)	(Ions score 36)
416 - 435	812.0292	2433.0658	2433.0619	2	1	K.MMDKVEFVHMLNATMCATTR.T	3 Oxidation (M)	(Ions score 45)
416 - 435	609.2737	2433.0659	2433.0619	2	1	K.MMDKVEFVHMLNATMCATTR.T	3 Oxidation (M)	(Ions score 21)
416 - 435	812.0292	2433.0659	2433.0619	2	1	K.MMDKVEFVHMLNATMCATTR.T	3 Oxidation (M)	(Ions score 26)
416 - 435	812.0293	2433.0661	2433.0619	2	1	K.MMDKVEFVHMLNATMCATTR.T	3 Oxidation (M)	(Ions score 34)
416 - 435	812.0293	2433.0661	2433.0619	2	1	K.MMDKVEFVHMLNATMCATTR.T	3 Oxidation (M)	(Ions score 24)
416 - 435	812.0293	2433.0661	2433.0619	2	1	K.MMDKVEFVHMLNATMCATTR.T	3 Oxidation (M)	(Ions score 32)
416 - 435	812.0293	2433.0661	2433.0619	2	1	K.MMDKVEFVHMLNATMCATTR.T	3 Oxidation (M)	(Ions score 32)
416 - 435	609.2738	2433.0661	2433.0619	2	1	K.MMDKVEFVHMLNATMCATTR.T	3 Oxidation (M)	(Ions score 24)
416 - 435	609.2738	2433.0662	2433.0619	2	1	K.MMDKVEFVHMLNATMCATTR.T	3 Oxidation (M)	(Ions score 33)
416 - 435	609.2738	2433.0662	2433.0619	2	1	K.MMDKVEFVHMLNATMCATTR.T	3 Oxidation (M)	(Ions score 31)
416 - 435	812.0294	2433.0662	2433.0619	2	1	K.MMDKVEFVHMLNATMCATTR.T	3 Oxidation (M)	(Ions score 31)
416 - 435	609.2738	2433.0663	2433.0619	2	1	K.MMDKVEFVHMLNATMCATTR.T	3 Oxidation (M)	(Ions score 31)
416 - 435	609.2738	2433.0663	2433.0619	2	1	K.MMDKVEFVHMLNATMCATTR.T	3 Oxidation (M)	(Ions score 34)
416 - 435	609.2738	2433.0663	2433.0619	2	1	K.MMDKVEFVHMLNATMCATTR.T	3 Oxidation (M)	(Ions score 25)
416 - 435	812.0294	2433.0663	2433.0619	2	1	K.MMDKVEFVHMLNATMCATTR.T	3 Oxidation (M)	(Ions score 23)
416 - 435	812.0294	2433.0663	2433.0619	2	1	K.MMDKVEFVHMLNATMCATTR.T	3 Oxidation (M)	(Ions score 43)
416 - 435	609.2739	2433.0664	2433.0619	2	1	K.MMDKVEFVHMLNATMCATTR.T	3 Oxidation (M)	(Ions score 29)
416 - 435	609.2739	2433.0664	2433.0619	2	1	K.MMDKVEFVHMLNATMCATTR.T	3 Oxidation (M)	(Ions score 29)
416 - 435	1217.5405	2433.0664	2433.0619	2	1	K.MMDKVEFVHMLNATMCATTR.T	3 Oxidation (M)	(Ions score 43)
416 - 435	812.0294	2433.0664	2433.0619	2	1	K.MMDKVEFVHMLNATMCATTR.T	3 Oxidation (M)	(Ions score 45)
416 - 435	812.0294	2433.0664	2433.0619	2	1	K.MMDKVEFVHMLNATMCATTR.T	3 Oxidation (M)	(Ions score 40)
416 - 435	812.0294	2433.0664	2433.0619	2	1	K.MMDKVEFVHMLNATMCATTR.T	3 Oxidation (M)	(Ions score 37)
416 - 435	812.0294	2433.0664	2433.0619	2	1	K.MMDKVEFVHMLNATMCATTR.T	3 Oxidation (M)	(Ions score 55)
416 - 435	609.2739	2433.0666	2433.0619	2	1	K.MMDKVEFVHMLNATMCATTR.T	3 Oxidation (M)	(Ions score 35)
416 - 435	609.2739	2433.0666	2433.0619	2	1	K.MMDKVEFVHMLNATMCATTR.T	3 Oxidation (M)	(Ions score 22)
416 - 435	609.2739	2433.0666	2433.0619	2	1	K.MMDKVEFVHMLNATMCATTR.T	3 Oxidation (M)	(Ions score 21)
416 - 435	812.0295	2433.0666	2433.0619	2	1	K.MMDKVEFVHMLNATMCATTR.T	3 Oxidation (M)	(Ions score 41)
416 - 435	812.0295	2433.0667	2433.0619	2	1	K.MMDKVEFVHMLNATMCATTR.T	3 Oxidation (M)	(Ions score 34)
416 - 435	812.0295	2433.0668	2433.0619	2	1	K.MMDKVEFVHMLNATMCATTR.T	3 Oxidation (M)	(Ions score 35)
416 - 435	812.0295	2433.0668	2433.0619	2	1	K.MMDKVEFVHMLNATMCATTR.T	3 Oxidation (M)	(Ions score 50)
416 - 435	812.0295	2433.0668	2433.0619	2	1	K.MMDKVEFVHMLNATMCATTR.T	3 Oxidation (M)	(Ions score 56)
416 - 435	812.0296	2433.0669	2433.0619	2	1	K.MMDKVEFVHMLNATMCATTR.T	3 Oxidation (M)	(Ions score 39)
416 - 435	812.0296	2433.0669	2433.0619	2	1	K.MMDKVEFVHMLNATMCATTR.T	3 Oxidation (M)	(Ions score 43)
416 - 435	812.0296	2433.0669	2433.0619	2	1	K.MMDKVEFVHMLNATMCATTR.T	3 Oxidation (M)	(Ions score 47)
416 - 435	812.0296	2433.0669	2433.0619	2	1	K.MMDKVEFVHMLNATMCATTR.T	3 Oxidation (M)	(Ions score 43)
416 - 435	812.0296	2433.0670	2433.0619	2	1	K.MMDKVEFVHMLNATMCATTR.T	3 Oxidation (M)	(Ions score 37)
416 - 435	812.0296	2433.0670	2433.0619	2	1	K.MMDKVEFVHMLNATMCATTR.T	3 Oxidation (M)	(Ions score 32)
416 - 435	812.0296	2433.0670	2433.0619	2	1	K.MMDKVEFVHMLNATMCATTR.T	3 Oxidation (M)	(Ions score 23)
416 - 435	1217.5408	2433.0670	2433.0619	2	1	K.MMDKVEFVHMLNATMCATTR.T	3 Oxidation (M)	(Ions score 52)

416 - 435	609.2740	2433.0671	2433.0619	2	1	K.MMDKVEFVHMLNATMCATTR.T	3 Oxidation (M)	(Ions score 32)
416 - 435	609.2741	2433.0671	2433.0619	2	1	K.MMDKVEFVHMLNATMCATTR.T	3 Oxidation (M)	(Ions score 26)
416 - 435	812.0297	2433.0672	2433.0619	2	1	K.MMDKVEFVHMLNATMCATTR.T	3 Oxidation (M)	(Ions score 31)
416 - 435	812.0297	2433.0672	2433.0619	2	1	K.MMDKVEFVHMLNATMCATTR.T	3 Oxidation (M)	(Ions score 39)
416 - 435	812.0297	2433.0673	2433.0619	2	1	K.MMDKVEFVHMLNATMCATTR.T	3 Oxidation (M)	(Ions score 44)
416 - 435	609.2741	2433.0674	2433.0619	2	1	K.MMDKVEFVHMLNATMCATTR.T	3 Oxidation (M)	(Ions score 24)
416 - 435	609.2741	2433.0674	2433.0619	2	1	K.MMDKVEFVHMLNATMCATTR.T	3 Oxidation (M)	(Ions score 27)
416 - 435	812.0298	2433.0675	2433.0619	2	1	K.MMDKVEFVHMLNATMCATTR.T	3 Oxidation (M)	(Ions score 24)
416 - 435	812.0298	2433.0675	2433.0619	2	1	K.MMDKVEFVHMLNATMCATTR.T	3 Oxidation (M)	(Ions score 56)
416 - 435	609.2742	2433.0676	2433.0619	2	1	K.MMDKVEFVHMLNATMCATTR.T	3 Oxidation (M)	(Ions score 36)
416 - 435	812.0298	2433.0676	2433.0619	2	1	K.MMDKVEFVHMLNATMCATTR.T	3 Oxidation (M)	(Ions score 53)
416 - 435	609.2742	2433.0677	2433.0619	2	1	K.MMDKVEFVHMLNATMCATTR.T	3 Oxidation (M)	(Ions score 32)
416 - 435	812.0299	2433.0678	2433.0619	2	1	K.MMDKVEFVHMLNATMCATTR.T	3 Oxidation (M)	(Ions score 42)
416 - 435	609.2742	2433.0678	2433.0619	2	1	K.MMDKVEFVHMLNATMCATTR.T	3 Oxidation (M)	(Ions score 22)
416 - 435	812.0299	2433.0679	2433.0619	2	1	K.MMDKVEFVHMLNATMCATTR.T	3 Oxidation (M)	(Ions score 41)
416 - 435	812.0299	2433.0679	2433.0619	2	1	K.MMDKVEFVHMLNATMCATTR.T	3 Oxidation (M)	(Ions score 40)
416 - 435	609.2742	2433.0679	2433.0619	2	1	K.MMDKVEFVHMLNATMCATTR.T	3 Oxidation (M)	(Ions score 31)
416 - 435	812.0299	2433.0679	2433.0619	2	1	K.MMDKVEFVHMLNATMCATTR.T	3 Oxidation (M)	(Ions score 34)
416 - 435	812.0299	2433.0679	2433.0619	2	1	K.MMDKVEFVHMLNATMCATTR.T	3 Oxidation (M)	(Ions score 40)
416 - 435	609.2743	2433.0679	2433.0619	2	1	K.MMDKVEFVHMLNATMCATTR.T	3 Oxidation (M)	(Ions score 29)
416 - 435	609.2743	2433.0680	2433.0619	2	1	K.MMDKVEFVHMLNATMCATTR.T	3 Oxidation (M)	(Ions score 29)
416 - 435	812.0299	2433.0680	2433.0619	2	1	K.MMDKVEFVHMLNATMCATTR.T	3 Oxidation (M)	(Ions score 43)
416 - 435	812.0299	2433.0680	2433.0619	2	1	K.MMDKVEFVHMLNATMCATTR.T	3 Oxidation (M)	(Ions score 43)
416 - 435	609.2743	2433.0680	2433.0619	3	1	K.MMDKVEFVHMLNATMCATTR.T	3 Oxidation (M)	(Ions score 29)
416 - 435	812.0300	2433.0680	2433.0619	3	1	K.MMDKVEFVHMLNATMCATTR.T	3 Oxidation (M)	(Ions score 41)
416 - 435	812.0300	2433.0680	2433.0619	3	1	K.MMDKVEFVHMLNATMCATTR.T	3 Oxidation (M)	(Ions score 44)
416 - 435	812.0300	2433.0681	2433.0619	3	1	K.MMDKVEFVHMLNATMCATTR.T	3 Oxidation (M)	(Ions score 31)
416 - 435	812.0300	2433.0681	2433.0619	3	1	K.MMDKVEFVHMLNATMCATTR.T	3 Oxidation (M)	(Ions score 42)
416 - 435	1217.5413	2433.0681	2433.0619	3	1	K.MMDKVEFVHMLNATMCATTR.T	3 Oxidation (M)	(Ions score 61)
416 - 435	812.0300	2433.0681	2433.0619	3	1	K.MMDKVEFVHMLNATMCATTR.T	3 Oxidation (M)	(Ions score 48)
416 - 435	812.0300	2433.0681	2433.0619	3	1	K.MMDKVEFVHMLNATMCATTR.T	3 Oxidation (M)	(Ions score 24)
416 - 435	812.0300	2433.0682	2433.0619	3	1	K.MMDKVEFVHMLNATMCATTR.T	3 Oxidation (M)	(Ions score 24)
416 - 435	609.2743	2433.0682	2433.0619	3	1	K.MMDKVEFVHMLNATMCATTR.T	3 Oxidation (M)	(Ions score 28)
416 - 435	812.0300	2433.0682	2433.0619	3	1	K.MMDKVEFVHMLNATMCATTR.T	3 Oxidation (M)	(Ions score 53)
416 - 435	812.0300	2433.0682	2433.0619	3	1	K.MMDKVEFVHMLNATMCATTR.T	3 Oxidation (M)	(Ions score 35)
416 - 435	609.2743	2433.0683	2433.0619	3	1	K.MMDKVEFVHMLNATMCATTR.T	3 Oxidation (M)	(Ions score 24)
416 - 435	812.0300	2433.0683	2433.0619	3	1	K.MMDKVEFVHMLNATMCATTR.T	3 Oxidation (M)	(Ions score 47)
416 - 435	609.2744	2433.0683	2433.0619	3	1	K.MMDKVEFVHMLNATMCATTR.T	3 Oxidation (M)	(Ions score 29)
416 - 435	609.2744	2433.0683	2433.0619	3	1	K.MMDKVEFVHMLNATMCATTR.T	3 Oxidation (M)	(Ions score 25)
416 - 435	812.0301	2433.0685	2433.0619	3	1	K.MMDKVEFVHMLNATMCATTR.T	3 Oxidation (M)	(Ions score 31)
416 - 435	609.2744	2433.0685	2433.0619	3	1	K.MMDKVEFVHMLNATMCATTR.T	3 Oxidation (M)	(Ions score 29)
416 - 435	609.2744	2433.0685	2433.0619	3	1	K.MMDKVEFVHMLNATMCATTR.T	3 Oxidation (M)	(Ions score 23)
416 - 435	812.0301	2433.0685	2433.0619	3	1	K.MMDKVEFVHMLNATMCATTR.T	3 Oxidation (M)	(Ions score 37)
416 - 435	812.0301	2433.0685	2433.0619	3	1	K.MMDKVEFVHMLNATMCATTR.T	3 Oxidation (M)	(Ions score 51)
416 - 435	609.2744	2433.0685	2433.0619	3	1	K.MMDKVEFVHMLNATMCATTR.T	3 Oxidation (M)	(Ions score 26)
416 - 435	609.2744	2433.0686	2433.0619	3	1	K.MMDKVEFVHMLNATMCATTR.T	3 Oxidation (M)	(Ions score 29)
416 - 435	609.2744	2433.0686	2433.0619	3	1	K.MMDKVEFVHMLNATMCATTR.T	3 Oxidation (M)	(Ions score 29)
416 - 435	609.2744	2433.0686	2433.0619	3	1	K.MMDKVEFVHMLNATMCATTR.T	3 Oxidation (M)	(Ions score 26)
416 - 435	812.0302	2433.0686	2433.0619	3	1	K.MMDKVEFVHMLNATMCATTR.T	3 Oxidation (M)	(Ions score 41)
416 - 435	812.0302	2433.0687	2433.0619	3	1	K.MMDKVEFVHMLNATMCATTR.T	3 Oxidation (M)	(Ions score 49)
416 - 435	812.0302	2433.0687	2433.0619	3	1	K.MMDKVEFVHMLNATMCATTR.T	3 Oxidation (M)	(Ions score 38)
416 - 435	812.0302	2433.0688	2433.0619	3	1	K.MMDKVEFVHMLNATMCATTR.T	3 Oxidation (M)	(Ions score 43)
416 - 435	812.0302	2433.0688	2433.0619	3	1	K.MMDKVEFVHMLNATMCATTR.T	3 Oxidation (M)	(Ions score 48)
416 - 435	609.2745	2433.0688	2433.0619	3	1	K.MMDKVEFVHMLNATMCATTR.T	3 Oxidation (M)	(Ions score 35)
416 - 435	812.0302	2433.0689	2433.0619	3	1	K.MMDKVEFVHMLNATMCATTR.T	3 Oxidation (M)	(Ions score 43)
416 - 435	609.2745	2433.0689	2433.0619	3	1	K.MMDKVEFVHMLNATMCATTR.T	3 Oxidation (M)	(Ions score 34)
416 - 435	609.2745	2433.0689	2433.0619	3	1	K.MMDKVEFVHMLNATMCATTR.T	3 Oxidation (M)	(Ions score 26)

416 - 435	812.0302	2433.0689	2433.0619	3	1	K.MMDKVEFVHMLNATMCATTR.T	3 Oxidation (M)	(Ions score 56)
416 - 435	812.0303	2433.0689	2433.0619	3	1	K.MMDKVEFVHMLNATMCATTR.T	3 Oxidation (M)	(Ions score 42)
416 - 435	609.2745	2433.0689	2433.0619	3	1	K.MMDKVEFVHMLNATMCATTR.T	3 Oxidation (M)	(Ions score 34)
416 - 435	812.0303	2433.0689	2433.0619	3	1	K.MMDKVEFVHMLNATMCATTR.T	3 Oxidation (M)	(Ions score 49)
416 - 435	609.2745	2433.0689	2433.0619	3	1	K.MMDKVEFVHMLNATMCATTR.T	3 Oxidation (M)	(Ions score 34)
416 - 435	609.2745	2433.0689	2433.0619	3	1	K.MMDKVEFVHMLNATMCATTR.T	3 Oxidation (M)	(Ions score 31)
416 - 435	609.2745	2433.0690	2433.0619	3	1	K.MMDKVEFVHMLNATMCATTR.T	3 Oxidation (M)	(Ions score 30)
416 - 435	609.2745	2433.0690	2433.0619	3	1	K.MMDKVEFVHMLNATMCATTR.T	3 Oxidation (M)	(Ions score 25)
416 - 435	609.2745	2433.0690	2433.0619	3	1	K.MMDKVEFVHMLNATMCATTR.T	3 Oxidation (M)	(Ions score 32)
416 - 435	609.2745	2433.0691	2433.0619	3	1	K.MMDKVEFVHMLNATMCATTR.T	3 Oxidation (M)	(Ions score 22)
416 - 435	609.2745	2433.0691	2433.0619	3	1	K.MMDKVEFVHMLNATMCATTR.T	3 Oxidation (M)	(Ions score 23)
416 - 435	812.0303	2433.0692	2433.0619	3	1	K.MMDKVEFVHMLNATMCATTR.T	3 Oxidation (M)	(Ions score 30)
416 - 435	609.2746	2433.0692	2433.0619	3	1	K.MMDKVEFVHMLNATMCATTR.T	3 Oxidation (M)	(Ions score 26)
416 - 435	609.2746	2433.0692	2433.0619	3	1	K.MMDKVEFVHMLNATMCATTR.T	3 Oxidation (M)	(Ions score 26)
416 - 435	812.0303	2433.0692	2433.0619	3	1	K.MMDKVEFVHMLNATMCATTR.T	3 Oxidation (M)	(Ions score 43)
416 - 435	609.2746	2433.0692	2433.0619	3	1	K.MMDKVEFVHMLNATMCATTR.T	3 Oxidation (M)	(Ions score 34)
416 - 435	812.0304	2433.0693	2433.0619	3	1	K.MMDKVEFVHMLNATMCATTR.T	3 Oxidation (M)	(Ions score 38)
416 - 435	812.0304	2433.0694	2433.0619	3	1	K.MMDKVEFVHMLNATMCATTR.T	3 Oxidation (M)	(Ions score 31)
416 - 435	812.0304	2433.0694	2433.0619	3	1	K.MMDKVEFVHMLNATMCATTR.T	3 Oxidation (M)	(Ions score 31)
416 - 435	609.2746	2433.0694	2433.0619	3	1	K.MMDKVEFVHMLNATMCATTR.T	3 Oxidation (M)	(Ions score 22)
416 - 435	609.2746	2433.0695	2433.0619	3	1	K.MMDKVEFVHMLNATMCATTR.T	3 Oxidation (M)	(Ions score 34)
416 - 435	609.2747	2433.0695	2433.0619	3	1	K.MMDKVEFVHMLNATMCATTR.T	3 Oxidation (M)	(Ions score 29)
416 - 435	812.0305	2433.0695	2433.0619	3	1	K.MMDKVEFVHMLNATMCATTR.T	3 Oxidation (M)	(Ions score 39)
416 - 435	609.2747	2433.0695	2433.0619	3	1	K.MMDKVEFVHMLNATMCATTR.T	3 Oxidation (M)	(Ions score 27)
416 - 435	812.0305	2433.0695	2433.0619	3	1	K.MMDKVEFVHMLNATMCATTR.T	3 Oxidation (M)	(Ions score 37)
416 - 435	609.2747	2433.0696	2433.0619	3	1	K.MMDKVEFVHMLNATMCATTR.T	3 Oxidation (M)	(Ions score 22)
416 - 435	609.2747	2433.0696	2433.0619	3	1	K.MMDKVEFVHMLNATMCATTR.T	3 Oxidation (M)	(Ions score 30)
416 - 435	812.0305	2433.0697	2433.0619	3	1	K.MMDKVEFVHMLNATMCATTR.T	3 Oxidation (M)	(Ions score 48)
416 - 435	812.0305	2433.0698	2433.0619	3	1	K.MMDKVEFVHMLNATMCATTR.T	3 Oxidation (M)	(Ions score 25)
416 - 435	812.0305	2433.0698	2433.0619	3	1	K.MMDKVEFVHMLNATMCATTR.T	3 Oxidation (M)	(Ions score 41)
416 - 435	609.2747	2433.0698	2433.0619	3	1	K.MMDKVEFVHMLNATMCATTR.T	3 Oxidation (M)	(Ions score 34)
416 - 435	609.2747	2433.0698	2433.0619	3	1	K.MMDKVEFVHMLNATMCATTR.T	3 Oxidation (M)	(Ions score 31)
416 - 435	609.2747	2433.0699	2433.0619	3	1	K.MMDKVEFVHMLNATMCATTR.T	3 Oxidation (M)	(Ions score 22)
416 - 435	812.0306	2433.0699	2433.0619	3	1	K.MMDKVEFVHMLNATMCATTR.T	3 Oxidation (M)	(Ions score 37)
416 - 435	812.0306	2433.0699	2433.0619	3	1	K.MMDKVEFVHMLNATMCATTR.T	3 Oxidation (M)	(Ions score 33)
416 - 435	609.2747	2433.0699	2433.0619	3	1	K.MMDKVEFVHMLNATMCATTR.T	3 Oxidation (M)	(Ions score 27)
416 - 435	609.2748	2433.0699	2433.0619	3	1	K.MMDKVEFVHMLNATMCATTR.T	3 Oxidation (M)	(Ions score 28)
416 - 435	812.0306	2433.0699	2433.0619	3	1	K.MMDKVEFVHMLNATMCATTR.T	3 Oxidation (M)	(Ions score 35)
416 - 435	609.2748	2433.0700	2433.0619	3	1	K.MMDKVEFVHMLNATMCATTR.T	3 Oxidation (M)	(Ions score 26)
416 - 435	609.2748	2433.0701	2433.0619	3	1	K.MMDKVEFVHMLNATMCATTR.T	3 Oxidation (M)	(Ions score 34)
416 - 435	812.0306	2433.0701	2433.0619	3	1	K.MMDKVEFVHMLNATMCATTR.T	3 Oxidation (M)	(Ions score 47)
416 - 435	812.0307	2433.0701	2433.0619	3	1	K.MMDKVEFVHMLNATMCATTR.T	3 Oxidation (M)	(Ions score 50)
416 - 435	609.2748	2433.0703	2433.0619	3	1	K.MMDKVEFVHMLNATMCATTR.T	3 Oxidation (M)	(Ions score 21)
416 - 435	812.0307	2433.0703	2433.0619	3	1	K.MMDKVEFVHMLNATMCATTR.T	3 Oxidation (M)	(Ions score 42)
416 - 435	812.0307	2433.0703	2433.0619	3	1	K.MMDKVEFVHMLNATMCATTR.T	3 Oxidation (M)	(Ions score 35)
416 - 435	609.2749	2433.0703	2433.0619	3	1	K.MMDKVEFVHMLNATMCATTR.T	3 Oxidation (M)	(Ions score 22)
416 - 435	609.2749	2433.0705	2433.0619	4	1	K.MMDKVEFVHMLNATMCATTR.T	3 Oxidation (M)	(Ions score 31)
416 - 435	812.0308	2433.0705	2433.0619	4	1	K.MMDKVEFVHMLNATMCATTR.T	3 Oxidation (M)	(Ions score 33)
416 - 435	812.0308	2433.0706	2433.0619	4	1	K.MMDKVEFVHMLNATMCATTR.T	3 Oxidation (M)	(Ions score 44)
416 - 435	609.2749	2433.0706	2433.0619	4	1	K.MMDKVEFVHMLNATMCATTR.T	3 Oxidation (M)	(Ions score 31)
416 - 435	812.0308	2433.0707	2433.0619	4	1	K.MMDKVEFVHMLNATMCATTR.T	3 Oxidation (M)	(Ions score 41)
416 - 435	609.2749	2433.0707	2433.0619	4	1	K.MMDKVEFVHMLNATMCATTR.T	3 Oxidation (M)	(Ions score 26)
416 - 435	609.2750	2433.0707	2433.0619	4	1	K.MMDKVEFVHMLNATMCATTR.T	3 Oxidation (M)	(Ions score 23)
416 - 435	609.2750	2433.0708	2433.0619	4	1	K.MMDKVEFVHMLNATMCATTR.T	3 Oxidation (M)	(Ions score 25)
416 - 435	812.0309	2433.0708	2433.0619	4	1	K.MMDKVEFVHMLNATMCATTR.T	3 Oxidation (M)	(Ions score 41)
416 - 435	609.2750	2433.0709	2433.0619	4	1	K.MMDKVEFVHMLNATMCATTR.T	3 Oxidation (M)	(Ions score 27)
416 - 435	812.0309	2433.0709	2433.0619	4	1	K.MMDKVEFVHMLNATMCATTR.T	3 Oxidation (M)	(Ions score 31)

416 - 435	609.2750	2433.0709	2433.0619	4	1	K.MMDKVEFVHMLNATMCATTR.T	3 Oxidation (M)	(Ions score 22)
416 - 435	609.2750	2433.0709	2433.0619	4	1	K.MMDKVEFVHMLNATMCATTR.T	3 Oxidation (M)	(Ions score 34)
416 - 435	609.2750	2433.0710	2433.0619	4	1	K.MMDKVEFVHMLNATMCATTR.T	3 Oxidation (M)	(Ions score 25)
416 - 435	812.0309	2433.0710	2433.0619	4	1	K.MMDKVEFVHMLNATMCATTR.T	3 Oxidation (M)	(Ions score 48)
416 - 435	609.2750	2433.0711	2433.0619	4	1	K.MMDKVEFVHMLNATMCATTR.T	3 Oxidation (M)	(Ions score 29)
416 - 435	812.0310	2433.0711	2433.0619	4	1	K.MMDKVEFVHMLNATMCATTR.T	3 Oxidation (M)	(Ions score 47)
416 - 435	812.0310	2433.0711	2433.0619	4	1	K.MMDKVEFVHMLNATMCATTR.T	3 Oxidation (M)	(Ions score 40)
416 - 435	812.0310	2433.0712	2433.0619	4	1	K.MMDKVEFVHMLNATMCATTR.T	3 Oxidation (M)	(Ions score 29)
416 - 435	609.2751	2433.0712	2433.0619	4	1	K.MMDKVEFVHMLNATMCATTR.T	3 Oxidation (M)	(Ions score 22)
416 - 435	609.2751	2433.0712	2433.0619	4	1	K.MMDKVEFVHMLNATMCATTR.T	3 Oxidation (M)	(Ions score 29)
416 - 435	609.2751	2433.0712	2433.0619	4	1	K.MMDKVEFVHMLNATMCATTR.T	3 Oxidation (M)	(Ions score 26)
416 - 435	812.0310	2433.0713	2433.0619	4	1	K.MMDKVEFVHMLNATMCATTR.T	3 Oxidation (M)	(Ions score 36)
416 - 435	609.2751	2433.0713	2433.0619	4	1	K.MMDKVEFVHMLNATMCATTR.T	3 Oxidation (M)	(Ions score 21)
416 - 435	812.0310	2433.0713	2433.0619	4	1	K.MMDKVEFVHMLNATMCATTR.T	3 Oxidation (M)	(Ions score 37)
416 - 435	812.0311	2433.0714	2433.0619	4	1	K.MMDKVEFVHMLNATMCATTR.T	3 Oxidation (M)	(Ions score 57)
416 - 435	812.0311	2433.0716	2433.0619	4	1	K.MMDKVEFVHMLNATMCATTR.T	3 Oxidation (M)	(Ions score 51)
416 - 435	812.0311	2433.0716	2433.0619	4	1	K.MMDKVEFVHMLNATMCATTR.T	3 Oxidation (M)	(Ions score 35)
416 - 435	812.0311	2433.0716	2433.0619	4	1	K.MMDKVEFVHMLNATMCATTR.T	3 Oxidation (M)	(Ions score 35)
416 - 435	609.2752	2433.0716	2433.0619	4	1	K.MMDKVEFVHMLNATMCATTR.T	3 Oxidation (M)	(Ions score 34)
416 - 435	609.2752	2433.0717	2433.0619	4	1	K.MMDKVEFVHMLNATMCATTR.T	3 Oxidation (M)	(Ions score 29)
416 - 435	812.0312	2433.0717	2433.0619	4	1	K.MMDKVEFVHMLNATMCATTR.T	3 Oxidation (M)	(Ions score 54)
416 - 435	609.2752	2433.0717	2433.0619	4	1	K.MMDKVEFVHMLNATMCATTR.T	3 Oxidation (M)	(Ions score 22)
416 - 435	812.0312	2433.0717	2433.0619	4	1	K.MMDKVEFVHMLNATMCATTR.T	3 Oxidation (M)	(Ions score 22)
416 - 435	812.0312	2433.0717	2433.0619	4	1	K.MMDKVEFVHMLNATMCATTR.T	3 Oxidation (M)	(Ions score 44)
416 - 435	609.2752	2433.0717	2433.0619	4	1	K.MMDKVEFVHMLNATMCATTR.T	3 Oxidation (M)	(Ions score 32)
416 - 435	812.0312	2433.0718	2433.0619	4	1	K.MMDKVEFVHMLNATMCATTR.T	3 Oxidation (M)	(Ions score 43)
416 - 435	812.0312	2433.0718	2433.0619	4	1	K.MMDKVEFVHMLNATMCATTR.T	3 Oxidation (M)	(Ions score 41)
416 - 435	812.0312	2433.0719	2433.0619	4	1	K.MMDKVEFVHMLNATMCATTR.T	3 Oxidation (M)	(Ions score 31)
416 - 435	609.2753	2433.0719	2433.0619	4	1	K.MMDKVEFVHMLNATMCATTR.T	3 Oxidation (M)	(Ions score 34)
416 - 435	812.0313	2433.0720	2433.0619	4	1	K.MMDKVEFVHMLNATMCATTR.T	3 Oxidation (M)	(Ions score 34)
416 - 435	812.0313	2433.0720	2433.0619	4	1	K.MMDKVEFVHMLNATMCATTR.T	3 Oxidation (M)	(Ions score 33)
416 - 435	609.2753	2433.0720	2433.0619	4	1	K.MMDKVEFVHMLNATMCATTR.T	3 Oxidation (M)	(Ions score 22)
416 - 435	609.2753	2433.0721	2433.0619	4	1	K.MMDKVEFVHMLNATMCATTR.T	3 Oxidation (M)	(Ions score 31)
416 - 435	812.0313	2433.0721	2433.0619	4	1	K.MMDKVEFVHMLNATMCATTR.T	3 Oxidation (M)	(Ions score 45)
416 - 435	812.0313	2433.0721	2433.0619	4	1	K.MMDKVEFVHMLNATMCATTR.T	3 Oxidation (M)	(Ions score 44)
416 - 435	609.2753	2433.0721	2433.0619	4	1	K.MMDKVEFVHMLNATMCATTR.T	3 Oxidation (M)	(Ions score 31)
416 - 435	812.0313	2433.0722	2433.0619	4	1	K.MMDKVEFVHMLNATMCATTR.T	3 Oxidation (M)	(Ions score 33)
416 - 435	609.2753	2433.0722	2433.0619	4	1	K.MMDKVEFVHMLNATMCATTR.T	3 Oxidation (M)	(Ions score 27)
416 - 435	812.0313	2433.0722	2433.0619	4	1	K.MMDKVEFVHMLNATMCATTR.T	3 Oxidation (M)	(Ions score 44)
416 - 435	609.2753	2433.0723	2433.0619	4	1	K.MMDKVEFVHMLNATMCATTR.T	3 Oxidation (M)	(Ions score 29)
416 - 435	1217.5435	2433.0724	2433.0619	4	1	K.MMDKVEFVHMLNATMCATTR.T	3 Oxidation (M)	(Ions score 46)
416 - 435	812.0314	2433.0725	2433.0619	4	1	K.MMDKVEFVHMLNATMCATTR.T	3 Oxidation (M)	(Ions score 33)
416 - 435	609.2754	2433.0726	2433.0619	4	1	K.MMDKVEFVHMLNATMCATTR.T	3 Oxidation (M)	(Ions score 26)
416 - 435	812.0315	2433.0727	2433.0619	4	1	K.MMDKVEFVHMLNATMCATTR.T	3 Oxidation (M)	(Ions score 27)
416 - 435	812.0316	2433.0729	2433.0619	5	1	K.MMDKVEFVHMLNATMCATTR.T	3 Oxidation (M)	(Ions score 33)
416 - 435	609.2755	2433.0729	2433.0619	5	1	K.MMDKVEFVHMLNATMCATTR.T	3 Oxidation (M)	(Ions score 23)
416 - 435	609.2755	2433.0729	2433.0619	5	1	K.MMDKVEFVHMLNATMCATTR.T	3 Oxidation (M)	(Ions score 21)
416 - 435	609.2755	2433.0729	2433.0619	5	1	K.MMDKVEFVHMLNATMCATTR.T	3 Oxidation (M)	(Ions score 34)
416 - 435	812.0316	2433.0731	2433.0619	5	1	K.MMDKVEFVHMLNATMCATTR.T	3 Oxidation (M)	(Ions score 43)
416 - 435	812.0317	2433.0732	2433.0619	5	1	K.MMDKVEFVHMLNATMCATTR.T	3 Oxidation (M)	(Ions score 38)
416 - 435	609.2756	2433.0735	2433.0619	5	1	K.MMDKVEFVHMLNATMCATTR.T	3 Oxidation (M)	(Ions score 34)
416 - 435	812.0318	2433.0736	2433.0619	5	1	K.MMDKVEFVHMLNATMCATTR.T	3 Oxidation (M)	(Ions score 35)
416 - 435	609.2757	2433.0736	2433.0619	5	1	K.MMDKVEFVHMLNATMCATTR.T	3 Oxidation (M)	(Ions score 29)
416 - 435	812.0318	2433.0736	2433.0619	5	1	K.MMDKVEFVHMLNATMCATTR.T	3 Oxidation (M)	(Ions score 44)
416 - 435	609.2757	2433.0737	2433.0619	5	1	K.MMDKVEFVHMLNATMCATTR.T	3 Oxidation (M)	(Ions score 29)
416 - 435	812.0319	2433.0739	2433.0619	5	1	K.MMDKVEFVHMLNATMCATTR.T	3 Oxidation (M)	(Ions score 44)
416 - 435	812.0319	2433.0740	2433.0619	5	1	K.MMDKVEFVHMLNATMCATTR.T	3 Oxidation (M)	(Ions score 48)

416 - 435	812.0320	2433.0740	2433.0619	5	1	K.MMDKVEFVHMLNATMCATTR.T	3 Oxidation (M) (Ions score 28)
416 - 435	812.0320	2433.0741	2433.0619	5	1	K.MMDKVEFVHMLNATMCATTR.T	3 Oxidation (M) (Ions score 45)
416 - 435	611.7753	2443.0720	2443.0826	-4	1	K.MMDKVEFVHMLNATMCATTR.T	Acetylation (K); Oxidation (M) (Ions score 32)
416 - 435	611.7758	2443.0742	2443.0826	-3	1	K.MMDKVEFVHMLNATMCATTR.T	Acetylation (K); Oxidation (M) (Ions score 33)
416 - 435	611.7766	2443.0771	2443.0826	-2	1	K.MMDKVEFVHMLNATMCATTR.T	Acetylation (K); Oxidation (M) (Ions score 52)
416 - 435	611.7766	2443.0771	2443.0826	-2	1	K.MMDKVEFVHMLNATMCATTR.T	Acetylation (K); Oxidation (M) (Ions score 48)
416 - 435	815.3669	2443.0790	2443.0826	-2	1	K.MMDKVEFVHMLNATMCATTR.T	Acetylation (K); Oxidation (M) (Ions score 68)
416 - 435	611.7772	2443.0795	2443.0826	-1	1	K.MMDKVEFVHMLNATMCATTR.T	Acetylation (K); Oxidation (M) (Ions score 21)
416 - 435	611.7772	2443.0798	2443.0826	-1	1	K.MMDKVEFVHMLNATMCATTR.T	Acetylation (K); Oxidation (M) (Ions score 23)
416 - 435	611.7774	2443.0805	2443.0826	-1	1	K.MMDKVEFVHMLNATMCATTR.T	Acetylation (K); Oxidation (M) (Ions score 26)
416 - 435	815.3680	2443.0822	2443.0826	-0	1	K.MMDKVEFVHMLNATMCATTR.T	Acetylation (K); Oxidation (M) (Ions score 56)
416 - 435	815.3682	2443.0827	2443.0826	0	1	K.MMDKVEFVHMLNATMCATTR.T	Acetylation (K); Oxidation (M) (Ions score 57)
416 - 435	815.3685	2443.0837	2443.0826	0	1	K.MMDKVEFVHMLNATMCATTR.T	Acetylation (K); Oxidation (M) (Ions score 48)
416 - 435	611.7785	2443.0847	2443.0826	1	1	K.MMDKVEFVHMLNATMCATTR.T	Acetylation (K); Oxidation (M) (Ions score 36)
416 - 435	815.3693	2443.0861	2443.0826	1	1	K.MMDKVEFVHMLNATMCATTR.T	Acetylation (K); Oxidation (M) (Ions score 55)
416 - 435	815.3699	2443.0878	2443.0826	2	1	K.MMDKVEFVHMLNATMCATTR.T	Acetylation (K); Oxidation (M) (Ions score 54)
416 - 435	815.3699	2443.0879	2443.0826	2	1	K.MMDKVEFVHMLNATMCATTR.T	Acetylation (K); Oxidation (M) (Ions score 40)
416 - 435	815.3699	2443.0880	2443.0826	2	1	K.MMDKVEFVHMLNATMCATTR.T	Acetylation (K); Oxidation (M) (Ions score 47)
416 - 435	815.3700	2443.0881	2443.0826	2	1	K.MMDKVEFVHMLNATMCATTR.T	Acetylation (K); Oxidation (M) (Ions score 40)
416 - 435	815.3702	2443.0888	2443.0826	3	1	K.MMDKVEFVHMLNATMCATTR.T	Acetylation (K); Oxidation (M) (Ions score 67)
416 - 435	815.3702	2443.0889	2443.0826	3	1	K.MMDKVEFVHMLNATMCATTR.T	Acetylation (K); Oxidation (M) (Ions score 67)
416 - 435	815.3707	2443.0904	2443.0826	3	1	K.MMDKVEFVHMLNATMCATTR.T	Acetylation (K); Oxidation (M) (Ions score 68)
416 - 435	611.7803	2443.0919	2443.0826	4	1	K.MMDKVEFVHMLNATMCATTR.T	Acetylation (K); Oxidation (M) (Ions score 25)
416 - 435	815.3716	2443.0929	2443.0826	4	1	K.MMDKVEFVHMLNATMCATTR.T	Acetylation (K); Oxidation (M) (Ions score 47)
416 - 435	611.7808	2443.0942	2443.0826	5	1	K.MMDKVEFVHMLNATMCATTR.T	Acetylation (K); Oxidation (M) (Ions score 61)
416 - 435	1225.5343	2449.0541	2449.0568	-1	1	K.MMDKVEFVHMLNATMCATTR.T	4 Oxidation (M) (Ions score 46)
416 - 435	613.2724	2449.0606	2449.0568	2	1	K.MMDKVEFVHMLNATMCATTR.T	4 Oxidation (M) (Ions score 21)
416 - 435	613.2724	2449.0606	2449.0568	2	1	K.MMDKVEFVHMLNATMCATTR.T	4 Oxidation (M) (Ions score 21)
416 - 435	1225.5376	2449.0606	2449.0568	2	1	K.MMDKVEFVHMLNATMCATTR.T	4 Oxidation (M) (Ions score 35)
416 - 435	817.3609	2449.0609	2449.0568	2	1	K.MMDKVEFVHMLNATMCATTR.T	4 Oxidation (M) (Ions score 27)
416 - 435	1225.5380	2449.0614	2449.0568	2	1	K.MMDKVEFVHMLNATMCATTR.T	4 Oxidation (M) (Ions score 35)
416 - 435	817.3611	2449.0614	2449.0568	2	1	K.MMDKVEFVHMLNATMCATTR.T	4 Oxidation (M) (Ions score 22)
416 - 435	817.3614	2449.0623	2449.0568	2	1	K.MMDKVEFVHMLNATMCATTR.T	4 Oxidation (M) (Ions score 23)
416 - 435	1225.5387	2449.0628	2449.0568	2	1	K.MMDKVEFVHMLNATMCATTR.T	4 Oxidation (M) (Ions score 29)
416 - 435	613.2731	2449.0634	2449.0568	3	1	K.MMDKVEFVHMLNATMCATTR.T	4 Oxidation (M) (Ions score 21)
416 - 435	613.2734	2449.0646	2449.0568	3	1	K.MMDKVEFVHMLNATMCATTR.T	4 Oxidation (M) (Ions score 21)
416 - 435	613.2736	2449.0651	2449.0568	3	1	K.MMDKVEFVHMLNATMCATTR.T	4 Oxidation (M) (Ions score 21)
416 - 435	615.7740	2459.0668	2459.0776	-4	1	K.MMDKVEFVHMLNATMCATTR.T	Acetylation (K); 2 Oxidation (M) (Ions score 38)
416 - 435	820.6999	2459.0778	2459.0776	0	1	K.MMDKVEFVHMLNATMCATTR.T	Acetylation (K); 2 Oxidation (M) (Ions score 86)
416 - 435	820.7008	2459.0807	2459.0776	1	1	K.MMDKVEFVHMLNATMCATTR.T	Acetylation (K); 2 Oxidation (M) (Ions score 55)
416 - 435	820.7010	2459.0813	2459.0776	2	1	K.MMDKVEFVHMLNATMCATTR.T	Acetylation (K); 2 Oxidation (M) (Ions score 121)
416 - 435	820.7013	2459.0820	2459.0776	2	1	K.MMDKVEFVHMLNATMCATTR.T	Acetylation (K); 2 Oxidation (M) (Ions score 45)
416 - 435	820.7014	2459.0824	2459.0776	2	1	K.MMDKVEFVHMLNATMCATTR.T	Acetylation (K); 2 Oxidation (M) (Ions score 43)
416 - 435	820.7017	2459.0832	2459.0776	2	1	K.MMDKVEFVHMLNATMCATTR.T	Acetylation (K); 2 Oxidation (M) (Ions score 41)
416 - 435	820.7018	2459.0835	2459.0776	2	1	K.MMDKVEFVHMLNATMCATTR.T	Acetylation (K); 2 Oxidation (M) (Ions score 62)
416 - 435	820.7019	2459.0839	2459.0776	3	1	K.MMDKVEFVHMLNATMCATTR.T	Acetylation (K); 2 Oxidation (M) (Ions score 59)
416 - 435	820.7025	2459.0857	2459.0776	3	1	K.MMDKVEFVHMLNATMCATTR.T	Acetylation (K); 2 Oxidation (M) (Ions score 34)
416 - 435	615.7788	2459.0860	2459.0776	3	1	K.MMDKVEFVHMLNATMCATTR.T	Acetylation (K); 2 Oxidation (M) (Ions score 25)
416 - 435	615.7789	2459.0864	2459.0776	4	1	K.MMDKVEFVHMLNATMCATTR.T	Acetylation (K); 2 Oxidation (M) (Ions score 25)
416 - 435	820.7031	2459.0874	2459.0776	4	1	K.MMDKVEFVHMLNATMCATTR.T	Acetylation (K); 2 Oxidation (M) (Ions score 39)
416 - 435	820.7033	2459.0880	2459.0776	4	1	K.MMDKVEFVHMLNATMCATTR.T	Acetylation (K); 2 Oxidation (M) (Ions score 34)
416 - 435	615.7794	2459.0883	2459.0776	4	1	K.MMDKVEFVHMLNATMCATTR.T	Acetylation (K); 2 Oxidation (M) (Ions score 33)
416 - 435	826.0312	2475.0718	2475.0725	-0	1	K.MMDKVEFVHMLNATMCATTR.T	Acetylation (K); 3 Oxidation (M) (Ions score 30)
416 - 435	619.7754	2475.0725	2475.0725	-0	1	K.MMDKVEFVHMLNATMCATTR.T	Acetylation (K); 3 Oxidation (M) (Ions score 34)
416 - 435	826.0333	2475.0782	2475.0725	2	1	K.MMDKVEFVHMLNATMCATTR.T	Acetylation (K); 3 Oxidation (M) (Ions score 26)
416 - 435	826.0337	2475.0793	2475.0725	3	1	K.MMDKVEFVHMLNATMCATTR.T	Acetylation (K); 3 Oxidation (M) (Ions score 23)
416 - 435	826.0338	2475.0796	2475.0725	3	1	K.MMDKVEFVHMLNATMCATTR.T	Acetylation (K); 3 Oxidation (M) (Ions score 52)

416 - 435	619.7775	2475.0808	2475.0725	3	1	K.MMDKVEFVHMLNATMCATTR.T	Acetylation (K); 3 Oxidation (M) (Ions score 23)
416 - 435	826.0346	2475.0819	2475.0725	4	1	K.MMDKVEFVHMLNATMCATTR.T	Acetylation (K); 3 Oxidation (M) (Ions score 26)
416 - 435	619.7781	2475.0833	2475.0725	4	1	K.MMDKVEFVHMLNATMCATTR.T	Acetylation (K); 3 Oxidation (M) (Ions score 25)
416 - 435	826.0351	2475.0834	2475.0725	4	1	K.MMDKVEFVHMLNATMCATTR.T	Acetylation (K); 3 Oxidation (M) (Ions score 42)
420 - 435	627.6302	1879.8688	1879.8743	-3	0	K.VEFVHMLNATMCATTR.T	(Ions score 50)
420 - 435	940.9419	1879.8692	1879.8743	-3	0	K.VEFVHMLNATMCATTR.T	(Ions score 45)
420 - 435	940.9427	1879.8709	1879.8743	-2	0	K.VEFVHMLNATMCATTR.T	(Ions score 46)
420 - 435	940.9429	1879.8712	1879.8743	-2	0	K.VEFVHMLNATMCATTR.T	(Ions score 69)
420 - 435	940.9431	1879.8717	1879.8743	-1	0	K.VEFVHMLNATMCATTR.T	(Ions score 63)
420 - 435	940.9436	1879.8727	1879.8743	-1	0	K.VEFVHMLNATMCATTR.T	(Ions score 59)
420 - 435	940.9437	1879.8729	1879.8743	-1	0	K.VEFVHMLNATMCATTR.T	(Ions score 72)
420 - 435	940.9437	1879.8729	1879.8743	-1	0	K.VEFVHMLNATMCATTR.T	(Ions score 58)
420 - 435	940.9438	1879.8730	1879.8743	-1	0	K.VEFVHMLNATMCATTR.T	(Ions score 73)
420 - 435	940.9438	1879.8731	1879.8743	-1	0	K.VEFVHMLNATMCATTR.T	(Ions score 54)
420 - 435	627.6317	1879.8731	1879.8743	-1	0	K.VEFVHMLNATMCATTR.T	(Ions score 60)
420 - 435	940.9440	1879.8735	1879.8743	-0	0	K.VEFVHMLNATMCATTR.T	(Ions score 44)
420 - 435	940.9443	1879.8740	1879.8743	-0	0	K.VEFVHMLNATMCATTR.T	(Ions score 73)
420 - 435	940.9443	1879.8741	1879.8743	-0	0	K.VEFVHMLNATMCATTR.T	(Ions score 58)
420 - 435	940.9443	1879.8741	1879.8743	-0	0	K.VEFVHMLNATMCATTR.T	(Ions score 55)
420 - 435	940.9444	1879.8742	1879.8743	-0	0	K.VEFVHMLNATMCATTR.T	(Ions score 64)
420 - 435	940.9445	1879.8744	1879.8743	0	0	K.VEFVHMLNATMCATTR.T	(Ions score 63)
420 - 435	940.9445	1879.8744	1879.8743	0	0	K.VEFVHMLNATMCATTR.T	(Ions score 54)
420 - 435	627.6322	1879.8748	1879.8743	0	0	K.VEFVHMLNATMCATTR.T	(Ions score 59)
420 - 435	940.9447	1879.8748	1879.8743	0	0	K.VEFVHMLNATMCATTR.T	(Ions score 62)
420 - 435	940.9447	1879.8749	1879.8743	0	0	K.VEFVHMLNATMCATTR.T	(Ions score 61)
420 - 435	940.9449	1879.8752	1879.8743	0	0	K.VEFVHMLNATMCATTR.T	(Ions score 52)
420 - 435	940.9449	1879.8752	1879.8743	0	0	K.VEFVHMLNATMCATTR.T	(Ions score 76)
420 - 435	940.9449	1879.8753	1879.8743	1	0	K.VEFVHMLNATMCATTR.T	(Ions score 60)
420 - 435	627.6324	1879.8753	1879.8743	1	0	K.VEFVHMLNATMCATTR.T	(Ions score 31)
420 - 435	627.6324	1879.8753	1879.8743	1	0	K.VEFVHMLNATMCATTR.T	(Ions score 58)
420 - 435	627.6324	1879.8753	1879.8743	1	0	K.VEFVHMLNATMCATTR.T	(Ions score 66)
420 - 435	627.6324	1879.8754	1879.8743	1	0	K.VEFVHMLNATMCATTR.T	(Ions score 79)
420 - 435	627.6324	1879.8754	1879.8743	1	0	K.VEFVHMLNATMCATTR.T	(Ions score 61)
420 - 435	940.9450	1879.8754	1879.8743	1	0	K.VEFVHMLNATMCATTR.T	(Ions score 49)
420 - 435	627.6324	1879.8754	1879.8743	1	0	K.VEFVHMLNATMCATTR.T	(Ions score 71)
420 - 435	940.9451	1879.8756	1879.8743	1	0	K.VEFVHMLNATMCATTR.T	(Ions score 50)
420 - 435	627.6325	1879.8758	1879.8743	1	0	K.VEFVHMLNATMCATTR.T	(Ions score 78)
420 - 435	940.9452	1879.8759	1879.8743	1	0	K.VEFVHMLNATMCATTR.T	(Ions score 45)
420 - 435	627.6326	1879.8759	1879.8743	1	0	K.VEFVHMLNATMCATTR.T	(Ions score 65)
420 - 435	940.9452	1879.8759	1879.8743	1	0	K.VEFVHMLNATMCATTR.T	(Ions score 53)
420 - 435	940.9452	1879.8759	1879.8743	1	0	K.VEFVHMLNATMCATTR.T	(Ions score 48)
420 - 435	940.9453	1879.8760	1879.8743	1	0	K.VEFVHMLNATMCATTR.T	(Ions score 71)
420 - 435	627.6326	1879.8761	1879.8743	1	0	K.VEFVHMLNATMCATTR.T	(Ions score 90)
420 - 435	940.9453	1879.8761	1879.8743	1	0	K.VEFVHMLNATMCATTR.T	(Ions score 52)
420 - 435	940.9454	1879.8762	1879.8743	1	0	K.VEFVHMLNATMCATTR.T	(Ions score 60)
420 - 435	627.6327	1879.8762	1879.8743	1	0	K.VEFVHMLNATMCATTR.T	(Ions score 34)
420 - 435	627.6327	1879.8762	1879.8743	1	0	K.VEFVHMLNATMCATTR.T	(Ions score 81)
420 - 435	940.9454	1879.8762	1879.8743	1	0	K.VEFVHMLNATMCATTR.T	(Ions score 49)
420 - 435	940.9454	1879.8763	1879.8743	1	0	K.VEFVHMLNATMCATTR.T	(Ions score 67)
420 - 435	940.9454	1879.8763	1879.8743	1	0	K.VEFVHMLNATMCATTR.T	(Ions score 53)
420 - 435	940.9455	1879.8764	1879.8743	1	0	K.VEFVHMLNATMCATTR.T	(Ions score 71)
420 - 435	627.6327	1879.8764	1879.8743	1	0	K.VEFVHMLNATMCATTR.T	(Ions score 68)
420 - 435	627.6327	1879.8764	1879.8743	1	0	K.VEFVHMLNATMCATTR.T	(Ions score 63)
420 - 435	940.9455	1879.8764	1879.8743	1	0	K.VEFVHMLNATMCATTR.T	(Ions score 65)
420 - 435	940.9455	1879.8764	1879.8743	1	0	K.VEFVHMLNATMCATTR.T	(Ions score 63)
420 - 435	627.6328	1879.8765	1879.8743	1	0	K.VEFVHMLNATMCATTR.T	(Ions score 45)
420 - 435	627.6328	1879.8766	1879.8743	1	0	K.VEFVHMLNATMCATTR.T	(Ions score 64)

420 - 435	940.9456	1879.8766	1879.8743	1	0	K.VEFVHMLNATMCATTR.T	(Ions score 73)
420 - 435	940.9456	1879.8767	1879.8743	1	0	K.VEFVHMLNATMCATTR.T	(Ions score 50)
420 - 435	627.6328	1879.8767	1879.8743	1	0	K.VEFVHMLNATMCATTR.T	(Ions score 38)
420 - 435	940.9456	1879.8767	1879.8743	1	0	K.VEFVHMLNATMCATTR.T	(Ions score 66)
420 - 435	940.9456	1879.8767	1879.8743	1	0	K.VEFVHMLNATMCATTR.T	(Ions score 58)
420 - 435	627.6328	1879.8767	1879.8743	1	0	K.VEFVHMLNATMCATTR.T	(Ions score 59)
420 - 435	627.6328	1879.8767	1879.8743	1	0	K.VEFVHMLNATMCATTR.T	(Ions score 83)
420 - 435	627.6329	1879.8767	1879.8743	1	0	K.VEFVHMLNATMCATTR.T	(Ions score 80)
420 - 435	940.9457	1879.8768	1879.8743	1	0	K.VEFVHMLNATMCATTR.T	(Ions score 50)
420 - 435	940.9457	1879.8768	1879.8743	1	0	K.VEFVHMLNATMCATTR.T	(Ions score 70)
420 - 435	940.9457	1879.8768	1879.8743	1	0	K.VEFVHMLNATMCATTR.T	(Ions score 47)
420 - 435	940.9457	1879.8768	1879.8743	1	0	K.VEFVHMLNATMCATTR.T	(Ions score 59)
420 - 435	940.9457	1879.8768	1879.8743	1	0	K.VEFVHMLNATMCATTR.T	(Ions score 76)
420 - 435	940.9457	1879.8768	1879.8743	1	0	K.VEFVHMLNATMCATTR.T	(Ions score 72)
420 - 435	940.9457	1879.8768	1879.8743	1	0	K.VEFVHMLNATMCATTR.T	(Ions score 72)
420 - 435	940.9457	1879.8768	1879.8743	1	0	K.VEFVHMLNATMCATTR.T	(Ions score 49)
420 - 435	627.6329	1879.8768	1879.8743	1	0	K.VEFVHMLNATMCATTR.T	(Ions score 60)
420 - 435	627.6329	1879.8768	1879.8743	1	0	K.VEFVHMLNATMCATTR.T	(Ions score 89)
420 - 435	940.9457	1879.8769	1879.8743	1	0	K.VEFVHMLNATMCATTR.T	(Ions score 49)
420 - 435	627.6329	1879.8769	1879.8743	1	0	K.VEFVHMLNATMCATTR.T	(Ions score 90)
420 - 435	627.6329	1879.8770	1879.8743	1	0	K.VEFVHMLNATMCATTR.T	(Ions score 69)
420 - 435	940.9458	1879.8770	1879.8743	1	0	K.VEFVHMLNATMCATTR.T	(Ions score 54)
420 - 435	627.6329	1879.8770	1879.8743	1	0	K.VEFVHMLNATMCATTR.T	(Ions score 61)
420 - 435	940.9458	1879.8770	1879.8743	1	0	K.VEFVHMLNATMCATTR.T	(Ions score 66)
420 - 435	627.6330	1879.8770	1879.8743	1	0	K.VEFVHMLNATMCATTR.T	(Ions score 92)
420 - 435	940.9458	1879.8771	1879.8743	1	0	K.VEFVHMLNATMCATTR.T	(Ions score 53)
420 - 435	627.6330	1879.8771	1879.8743	1	0	K.VEFVHMLNATMCATTR.T	(Ions score 53)
420 - 435	940.9458	1879.8771	1879.8743	1	0	K.VEFVHMLNATMCATTR.T	(Ions score 50)
420 - 435	940.9458	1879.8771	1879.8743	2	0	K.VEFVHMLNATMCATTR.T	(Ions score 68)
420 - 435	627.6330	1879.8771	1879.8743	2	0	K.VEFVHMLNATMCATTR.T	(Ions score 75)
420 - 435	627.6330	1879.8771	1879.8743	2	0	K.VEFVHMLNATMCATTR.T	(Ions score 70)
420 - 435	940.9459	1879.8772	1879.8743	2	0	K.VEFVHMLNATMCATTR.T	(Ions score 74)
420 - 435	627.6330	1879.8772	1879.8743	2	0	K.VEFVHMLNATMCATTR.T	(Ions score 80)
420 - 435	627.6330	1879.8772	1879.8743	2	0	K.VEFVHMLNATMCATTR.T	(Ions score 92)
420 - 435	627.6330	1879.8772	1879.8743	2	0	K.VEFVHMLNATMCATTR.T	(Ions score 92)
420 - 435	627.6330	1879.8772	1879.8743	2	0	K.VEFVHMLNATMCATTR.T	(Ions score 64)
420 - 435	627.6330	1879.8773	1879.8743	2	0	K.VEFVHMLNATMCATTR.T	(Ions score 79)
420 - 435	940.9459	1879.8773	1879.8743	2	0	K.VEFVHMLNATMCATTR.T	(Ions score 51)
420 - 435	627.6330	1879.8773	1879.8743	2	0	K.VEFVHMLNATMCATTR.T	(Ions score 79)
420 - 435	627.6331	1879.8773	1879.8743	2	0	K.VEFVHMLNATMCATTR.T	(Ions score 75)
420 - 435	940.9459	1879.8773	1879.8743	2	0	K.VEFVHMLNATMCATTR.T	(Ions score 63)
420 - 435	627.6331	1879.8773	1879.8743	2	0	K.VEFVHMLNATMCATTR.T	(Ions score 55)
420 - 435	627.6331	1879.8773	1879.8743	2	0	K.VEFVHMLNATMCATTR.T	(Ions score 57)
420 - 435	627.6331	1879.8773	1879.8743	2	0	K.VEFVHMLNATMCATTR.T	(Ions score 75)
420 - 435	940.9460	1879.8773	1879.8743	2	0	K.VEFVHMLNATMCATTR.T	(Ions score 54)
420 - 435	627.6331	1879.8774	1879.8743	2	0	K.VEFVHMLNATMCATTR.T	(Ions score 77)
420 - 435	627.6331	1879.8774	1879.8743	2	0	K.VEFVHMLNATMCATTR.T	(Ions score 56)
420 - 435	940.9460	1879.8774	1879.8743	2	0	K.VEFVHMLNATMCATTR.T	(Ions score 78)
420 - 435	940.9460	1879.8774	1879.8743	2	0	K.VEFVHMLNATMCATTR.T	(Ions score 88)
420 - 435	627.6331	1879.8774	1879.8743	2	0	K.VEFVHMLNATMCATTR.T	(Ions score 58)
420 - 435	940.9460	1879.8774	1879.8743	2	0	K.VEFVHMLNATMCATTR.T	(Ions score 47)
420 - 435	940.9460	1879.8774	1879.8743	2	0	K.VEFVHMLNATMCATTR.T	(Ions score 62)
420 - 435	940.9460	1879.8774	1879.8743	2	0	K.VEFVHMLNATMCATTR.T	(Ions score 58)
420 - 435	627.6331	1879.8775	1879.8743	2	0	K.VEFVHMLNATMCATTR.T	(Ions score 58)
420 - 435	940.9460	1879.8775	1879.8743	2	0	K.VEFVHMLNATMCATTR.T	(Ions score 39)
420 - 435	940.9460	1879.8775	1879.8743	2	0	K.VEFVHMLNATMCATTR.T	(Ions score 59)
420 - 435	940.9460	1879.8775	1879.8743	2	0	K.VEFVHMLNATMCATTR.T	(Ions score 57)

420 - 435	627.6331	1879.8775	1879.8743	2	0	K.VEFVHMLNATMCATTR.T	(Ions score 83)
420 - 435	627.6331	1879.8775	1879.8743	2	0	K.VEFVHMLNATMCATTR.T	(Ions score 76)
420 - 435	627.6331	1879.8775	1879.8743	2	0	K.VEFVHMLNATMCATTR.T	(Ions score 87)
420 - 435	627.6331	1879.8775	1879.8743	2	0	K.VEFVHMLNATMCATTR.T	(Ions score 89)
420 - 435	627.6331	1879.8775	1879.8743	2	0	K.VEFVHMLNATMCATTR.T	(Ions score 75)
420 - 435	627.6331	1879.8775	1879.8743	2	0	K.VEFVHMLNATMCATTR.T	(Ions score 73)
420 - 435	940.9460	1879.8775	1879.8743	2	0	K.VEFVHMLNATMCATTR.T	(Ions score 66)
420 - 435	940.9461	1879.8776	1879.8743	2	0	K.VEFVHMLNATMCATTR.T	(Ions score 62)
420 - 435	627.6331	1879.8776	1879.8743	2	0	K.VEFVHMLNATMCATTR.T	(Ions score 87)
420 - 435	627.6331	1879.8776	1879.8743	2	0	K.VEFVHMLNATMCATTR.T	(Ions score 87)
420 - 435	627.6331	1879.8776	1879.8743	2	0	K.VEFVHMLNATMCATTR.T	(Ions score 77)
420 - 435	940.9461	1879.8776	1879.8743	2	0	K.VEFVHMLNATMCATTR.T	(Ions score 52)
420 - 435	627.6332	1879.8776	1879.8743	2	0	K.VEFVHMLNATMCATTR.T	(Ions score 83)
420 - 435	627.6332	1879.8776	1879.8743	2	0	K.VEFVHMLNATMCATTR.T	(Ions score 90)
420 - 435	627.6332	1879.8776	1879.8743	2	0	K.VEFVHMLNATMCATTR.T	(Ions score 79)
420 - 435	940.9461	1879.8776	1879.8743	2	0	K.VEFVHMLNATMCATTR.T	(Ions score 59)
420 - 435	627.6332	1879.8776	1879.8743	2	0	K.VEFVHMLNATMCATTR.T	(Ions score 68)
420 - 435	940.9461	1879.8776	1879.8743	2	0	K.VEFVHMLNATMCATTR.T	(Ions score 67)
420 - 435	940.9461	1879.8777	1879.8743	2	0	K.VEFVHMLNATMCATTR.T	(Ions score 64)
420 - 435	627.6332	1879.8777	1879.8743	2	0	K.VEFVHMLNATMCATTR.T	(Ions score 34)
420 - 435	627.6332	1879.8777	1879.8743	2	0	K.VEFVHMLNATMCATTR.T	(Ions score 69)
420 - 435	940.9461	1879.8777	1879.8743	2	0	K.VEFVHMLNATMCATTR.T	(Ions score 45)
420 - 435	627.6332	1879.8777	1879.8743	2	0	K.VEFVHMLNATMCATTR.T	(Ions score 75)
420 - 435	627.6332	1879.8778	1879.8743	2	0	K.VEFVHMLNATMCATTR.T	(Ions score 74)
420 - 435	627.6332	1879.8778	1879.8743	2	0	K.VEFVHMLNATMCATTR.T	(Ions score 69)
420 - 435	627.6332	1879.8778	1879.8743	2	0	K.VEFVHMLNATMCATTR.T	(Ions score 82)
420 - 435	940.9462	1879.8778	1879.8743	2	0	K.VEFVHMLNATMCATTR.T	(Ions score 40)
420 - 435	627.6332	1879.8778	1879.8743	2	0	K.VEFVHMLNATMCATTR.T	(Ions score 38)
420 - 435	627.6332	1879.8778	1879.8743	2	0	K.VEFVHMLNATMCATTR.T	(Ions score 64)
420 - 435	627.6332	1879.8778	1879.8743	2	0	K.VEFVHMLNATMCATTR.T	(Ions score 75)
420 - 435	940.9462	1879.8778	1879.8743	2	0	K.VEFVHMLNATMCATTR.T	(Ions score 54)
420 - 435	940.9462	1879.8779	1879.8743	2	0	K.VEFVHMLNATMCATTR.T	(Ions score 59)
420 - 435	940.9462	1879.8779	1879.8743	2	0	K.VEFVHMLNATMCATTR.T	(Ions score 76)
420 - 435	940.9462	1879.8779	1879.8743	2	0	K.VEFVHMLNATMCATTR.T	(Ions score 51)
420 - 435	627.6333	1879.8779	1879.8743	2	0	K.VEFVHMLNATMCATTR.T	(Ions score 76)
420 - 435	627.6333	1879.8779	1879.8743	2	0	K.VEFVHMLNATMCATTR.T	(Ions score 82)
420 - 435	940.9462	1879.8779	1879.8743	2	0	K.VEFVHMLNATMCATTR.T	(Ions score 67)
420 - 435	627.6333	1879.8779	1879.8743	2	0	K.VEFVHMLNATMCATTR.T	(Ions score 77)
420 - 435	627.6333	1879.8779	1879.8743	2	0	K.VEFVHMLNATMCATTR.T	(Ions score 71)
420 - 435	627.6333	1879.8780	1879.8743	2	0	K.VEFVHMLNATMCATTR.T	(Ions score 48)
420 - 435	940.9463	1879.8780	1879.8743	2	0	K.VEFVHMLNATMCATTR.T	(Ions score 54)
420 - 435	940.9463	1879.8780	1879.8743	2	0	K.VEFVHMLNATMCATTR.T	(Ions score 84)
420 - 435	627.6333	1879.8780	1879.8743	2	0	K.VEFVHMLNATMCATTR.T	(Ions score 74)
420 - 435	627.6333	1879.8780	1879.8743	2	0	K.VEFVHMLNATMCATTR.T	(Ions score 73)
420 - 435	627.6333	1879.8781	1879.8743	2	0	K.VEFVHMLNATMCATTR.T	(Ions score 53)
420 - 435	627.6333	1879.8781	1879.8743	2	0	K.VEFVHMLNATMCATTR.T	(Ions score 85)
420 - 435	940.9463	1879.8781	1879.8743	2	0	K.VEFVHMLNATMCATTR.T	(Ions score 62)
420 - 435	627.6333	1879.8781	1879.8743	2	0	K.VEFVHMLNATMCATTR.T	(Ions score 78)
420 - 435	627.6333	1879.8781	1879.8743	2	0	K.VEFVHMLNATMCATTR.T	(Ions score 87)
420 - 435	627.6333	1879.8781	1879.8743	2	0	K.VEFVHMLNATMCATTR.T	(Ions score 64)
420 - 435	940.9463	1879.8781	1879.8743	2	0	K.VEFVHMLNATMCATTR.T	(Ions score 64)
420 - 435	940.9463	1879.8781	1879.8743	2	0	K.VEFVHMLNATMCATTR.T	(Ions score 38)
420 - 435	627.6333	1879.8781	1879.8743	2	0	K.VEFVHMLNATMCATTR.T	(Ions score 76)
420 - 435	627.6333	1879.8781	1879.8743	2	0	K.VEFVHMLNATMCATTR.T	(Ions score 77)
420 - 435	627.6333	1879.8781	1879.8743	2	0	K.VEFVHMLNATMCATTR.T	(Ions score 75)
420 - 435	627.6333	1879.8781	1879.8743	2	0	K.VEFVHMLNATMCATTR.T	(Ions score 67)
420 - 435	940.9464	1879.8781	1879.8743	2	0	K.VEFVHMLNATMCATTR.T	(Ions score 56)

420 - 435	940.9464	1879.8782	1879.8743	2	0	K.VEFVHMLNATMCATTR.T	(Ions score 75)
420 - 435	627.6334	1879.8782	1879.8743	2	0	K.VEFVHMLNATMCATTR.T	(Ions score 77)
420 - 435	627.6334	1879.8782	1879.8743	2	0	K.VEFVHMLNATMCATTR.T	(Ions score 74)
420 - 435	627.6334	1879.8783	1879.8743	2	0	K.VEFVHMLNATMCATTR.T	(Ions score 90)
420 - 435	940.9464	1879.8783	1879.8743	2	0	K.VEFVHMLNATMCATTR.T	(Ions score 74)
420 - 435	627.6334	1879.8783	1879.8743	2	0	K.VEFVHMLNATMCATTR.T	(Ions score 90)
420 - 435	940.9464	1879.8783	1879.8743	2	0	K.VEFVHMLNATMCATTR.T	(Ions score 54)
420 - 435	627.6334	1879.8783	1879.8743	2	0	K.VEFVHMLNATMCATTR.T	(Ions score 75)
420 - 435	940.9464	1879.8783	1879.8743	2	0	K.VEFVHMLNATMCATTR.T	(Ions score 45)
420 - 435	940.9464	1879.8783	1879.8743	2	0	K.VEFVHMLNATMCATTR.T	(Ions score 56)
420 - 435	627.6334	1879.8783	1879.8743	2	0	K.VEFVHMLNATMCATTR.T	(Ions score 79)
420 - 435	627.6334	1879.8783	1879.8743	2	0	K.VEFVHMLNATMCATTR.T	(Ions score 55)
420 - 435	940.9465	1879.8784	1879.8743	2	0	K.VEFVHMLNATMCATTR.T	(Ions score 50)
420 - 435	627.6334	1879.8784	1879.8743	2	0	K.VEFVHMLNATMCATTR.T	(Ions score 78)
420 - 435	627.6334	1879.8784	1879.8743	2	0	K.VEFVHMLNATMCATTR.T	(Ions score 81)
420 - 435	627.6334	1879.8784	1879.8743	2	0	K.VEFVHMLNATMCATTR.T	(Ions score 69)
420 - 435	627.6334	1879.8784	1879.8743	2	0	K.VEFVHMLNATMCATTR.T	(Ions score 72)
420 - 435	627.6334	1879.8784	1879.8743	2	0	K.VEFVHMLNATMCATTR.T	(Ions score 65)
420 - 435	627.6334	1879.8784	1879.8743	2	0	K.VEFVHMLNATMCATTR.T	(Ions score 82)
420 - 435	627.6334	1879.8784	1879.8743	2	0	K.VEFVHMLNATMCATTR.T	(Ions score 82)
420 - 435	940.9465	1879.8785	1879.8743	2	0	K.VEFVHMLNATMCATTR.T	(Ions score 67)
420 - 435	940.9465	1879.8785	1879.8743	2	0	K.VEFVHMLNATMCATTR.T	(Ions score 69)
420 - 435	940.9465	1879.8785	1879.8743	2	0	K.VEFVHMLNATMCATTR.T	(Ions score 57)
420 - 435	940.9465	1879.8785	1879.8743	2	0	K.VEFVHMLNATMCATTR.T	(Ions score 54)
420 - 435	627.6334	1879.8785	1879.8743	2	0	K.VEFVHMLNATMCATTR.T	(Ions score 73)
420 - 435	940.9465	1879.8785	1879.8743	2	0	K.VEFVHMLNATMCATTR.T	(Ions score 47)
420 - 435	627.6334	1879.8785	1879.8743	2	0	K.VEFVHMLNATMCATTR.T	(Ions score 67)
420 - 435	627.6334	1879.8785	1879.8743	2	0	K.VEFVHMLNATMCATTR.T	(Ions score 72)
420 - 435	627.6335	1879.8785	1879.8743	2	0	K.VEFVHMLNATMCATTR.T	(Ions score 89)
420 - 435	627.6335	1879.8786	1879.8743	2	0	K.VEFVHMLNATMCATTR.T	(Ions score 77)
420 - 435	940.9466	1879.8786	1879.8743	2	0	K.VEFVHMLNATMCATTR.T	(Ions score 59)
420 - 435	627.6335	1879.8786	1879.8743	2	0	K.VEFVHMLNATMCATTR.T	(Ions score 74)
420 - 435	940.9466	1879.8786	1879.8743	2	0	K.VEFVHMLNATMCATTR.T	(Ions score 74)
420 - 435	627.6335	1879.8786	1879.8743	2	0	K.VEFVHMLNATMCATTR.T	(Ions score 80)
420 - 435	627.6335	1879.8786	1879.8743	2	0	K.VEFVHMLNATMCATTR.T	(Ions score 92)
420 - 435	940.9466	1879.8786	1879.8743	2	0	K.VEFVHMLNATMCATTR.T	(Ions score 63)
420 - 435	940.9466	1879.8786	1879.8743	2	0	K.VEFVHMLNATMCATTR.T	(Ions score 49)
420 - 435	627.6335	1879.8786	1879.8743	2	0	K.VEFVHMLNATMCATTR.T	(Ions score 91)
420 - 435	627.6335	1879.8786	1879.8743	2	0	K.VEFVHMLNATMCATTR.T	(Ions score 89)
420 - 435	627.6335	1879.8787	1879.8743	2	0	K.VEFVHMLNATMCATTR.T	(Ions score 74)
420 - 435	627.6335	1879.8787	1879.8743	2	0	K.VEFVHMLNATMCATTR.T	(Ions score 89)
420 - 435	940.9466	1879.8787	1879.8743	2	0	K.VEFVHMLNATMCATTR.T	(Ions score 53)
420 - 435	627.6335	1879.8787	1879.8743	2	0	K.VEFVHMLNATMCATTR.T	(Ions score 63)
420 - 435	627.6335	1879.8787	1879.8743	2	0	K.VEFVHMLNATMCATTR.T	(Ions score 76)
420 - 435	940.9466	1879.8787	1879.8743	2	0	K.VEFVHMLNATMCATTR.T	(Ions score 54)
420 - 435	940.9466	1879.8787	1879.8743	2	0	K.VEFVHMLNATMCATTR.T	(Ions score 76)
420 - 435	627.6335	1879.8787	1879.8743	2	0	K.VEFVHMLNATMCATTR.T	(Ions score 80)
420 - 435	940.9467	1879.8788	1879.8743	2	0	K.VEFVHMLNATMCATTR.T	(Ions score 35)
420 - 435	627.6335	1879.8788	1879.8743	2	0	K.VEFVHMLNATMCATTR.T	(Ions score 85)
420 - 435	627.6335	1879.8788	1879.8743	2	0	K.VEFVHMLNATMCATTR.T	(Ions score 73)
420 - 435	627.6335	1879.8788	1879.8743	2	0	K.VEFVHMLNATMCATTR.T	(Ions score 58)
420 - 435	627.6335	1879.8788	1879.8743	2	0	K.VEFVHMLNATMCATTR.T	(Ions score 67)
420 - 435	627.6336	1879.8788	1879.8743	2	0	K.VEFVHMLNATMCATTR.T	(Ions score 78)
420 - 435	627.6336	1879.8788	1879.8743	2	0	K.VEFVHMLNATMCATTR.T	(Ions score 71)
420 - 435	627.6336	1879.8788	1879.8743	2	0	K.VEFVHMLNATMCATTR.T	(Ions score 80)
420 - 435	627.6336	1879.8788	1879.8743	2	0	K.VEFVHMLNATMCATTR.T	(Ions score 70)
420 - 435	940.9467	1879.8789	1879.8743	2	0	K.VEFVHMLNATMCATTR.T	(Ions score 39)

420 - 435	940.9467	1879.8789	1879.8743	2	0	K.VEFVHMLNATMCATTR.T	(Ions score 54)
420 - 435	627.6336	1879.8789	1879.8743	2	0	K.VEFVHMLNATMCATTR.T	(Ions score 78)
420 - 435	627.6336	1879.8789	1879.8743	2	0	K.VEFVHMLNATMCATTR.T	(Ions score 80)
420 - 435	940.9467	1879.8789	1879.8743	2	0	K.VEFVHMLNATMCATTR.T	(Ions score 45)
420 - 435	627.6336	1879.8789	1879.8743	2	0	K.VEFVHMLNATMCATTR.T	(Ions score 87)
420 - 435	627.6336	1879.8789	1879.8743	2	0	K.VEFVHMLNATMCATTR.T	(Ions score 72)
420 - 435	627.6336	1879.8789	1879.8743	2	0	K.VEFVHMLNATMCATTR.T	(Ions score 64)
420 - 435	940.9467	1879.8789	1879.8743	2	0	K.VEFVHMLNATMCATTR.T	(Ions score 62)
420 - 435	627.6336	1879.8789	1879.8743	2	0	K.VEFVHMLNATMCATTR.T	(Ions score 76)
420 - 435	627.6336	1879.8790	1879.8743	2	0	K.VEFVHMLNATMCATTR.T	(Ions score 90)
420 - 435	940.9468	1879.8790	1879.8743	2	0	K.VEFVHMLNATMCATTR.T	(Ions score 66)
420 - 435	627.6336	1879.8790	1879.8743	2	0	K.VEFVHMLNATMCATTR.T	(Ions score 76)
420 - 435	940.9468	1879.8790	1879.8743	2	0	K.VEFVHMLNATMCATTR.T	(Ions score 50)
420 - 435	627.6336	1879.8790	1879.8743	2	0	K.VEFVHMLNATMCATTR.T	(Ions score 72)
420 - 435	627.6336	1879.8790	1879.8743	2	0	K.VEFVHMLNATMCATTR.T	(Ions score 72)
420 - 435	940.9468	1879.8790	1879.8743	2	0	K.VEFVHMLNATMCATTR.T	(Ions score 62)
420 - 435	627.6336	1879.8790	1879.8743	2	0	K.VEFVHMLNATMCATTR.T	(Ions score 50)
420 - 435	627.6336	1879.8790	1879.8743	2	0	K.VEFVHMLNATMCATTR.T	(Ions score 50)
420 - 435	627.6336	1879.8790	1879.8743	2	0	K.VEFVHMLNATMCATTR.T	(Ions score 90)
420 - 435	940.9468	1879.8790	1879.8743	3	0	K.VEFVHMLNATMCATTR.T	(Ions score 62)
420 - 435	940.9468	1879.8790	1879.8743	3	0	K.VEFVHMLNATMCATTR.T	(Ions score 87)
420 - 435	940.9468	1879.8790	1879.8743	3	0	K.VEFVHMLNATMCATTR.T	(Ions score 65)
420 - 435	627.6336	1879.8790	1879.8743	3	0	K.VEFVHMLNATMCATTR.T	(Ions score 72)
420 - 435	627.6336	1879.8790	1879.8743	3	0	K.VEFVHMLNATMCATTR.T	(Ions score 91)
420 - 435	940.9468	1879.8791	1879.8743	3	0	K.VEFVHMLNATMCATTR.T	(Ions score 66)
420 - 435	627.6336	1879.8791	1879.8743	3	0	K.VEFVHMLNATMCATTR.T	(Ions score 86)
420 - 435	627.6336	1879.8791	1879.8743	3	0	K.VEFVHMLNATMCATTR.T	(Ions score 85)
420 - 435	627.6336	1879.8791	1879.8743	3	0	K.VEFVHMLNATMCATTR.T	(Ions score 79)
420 - 435	940.9468	1879.8791	1879.8743	3	0	K.VEFVHMLNATMCATTR.T	(Ions score 54)
420 - 435	940.9468	1879.8791	1879.8743	3	0	K.VEFVHMLNATMCATTR.T	(Ions score 50)
420 - 435	627.6337	1879.8791	1879.8743	3	0	K.VEFVHMLNATMCATTR.T	(Ions score 67)
420 - 435	627.6337	1879.8792	1879.8743	3	0	K.VEFVHMLNATMCATTR.T	(Ions score 69)
420 - 435	627.6337	1879.8792	1879.8743	3	0	K.VEFVHMLNATMCATTR.T	(Ions score 78)
420 - 435	627.6337	1879.8792	1879.8743	3	0	K.VEFVHMLNATMCATTR.T	(Ions score 74)
420 - 435	627.6337	1879.8792	1879.8743	3	0	K.VEFVHMLNATMCATTR.T	(Ions score 79)
420 - 435	940.9469	1879.8792	1879.8743	3	0	K.VEFVHMLNATMCATTR.T	(Ions score 51)
420 - 435	940.9469	1879.8792	1879.8743	3	0	K.VEFVHMLNATMCATTR.T	(Ions score 62)
420 - 435	627.6337	1879.8792	1879.8743	3	0	K.VEFVHMLNATMCATTR.T	(Ions score 72)
420 - 435	627.6337	1879.8792	1879.8743	3	0	K.VEFVHMLNATMCATTR.T	(Ions score 76)
420 - 435	627.6337	1879.8792	1879.8743	3	0	K.VEFVHMLNATMCATTR.T	(Ions score 80)
420 - 435	627.6337	1879.8793	1879.8743	3	0	K.VEFVHMLNATMCATTR.T	(Ions score 92)
420 - 435	627.6337	1879.8793	1879.8743	3	0	K.VEFVHMLNATMCATTR.T	(Ions score 78)
420 - 435	940.9469	1879.8793	1879.8743	3	0	K.VEFVHMLNATMCATTR.T	(Ions score 60)
420 - 435	940.9469	1879.8793	1879.8743	3	0	K.VEFVHMLNATMCATTR.T	(Ions score 81)
420 - 435	940.9469	1879.8793	1879.8743	3	0	K.VEFVHMLNATMCATTR.T	(Ions score 67)
420 - 435	627.6337	1879.8793	1879.8743	3	0	K.VEFVHMLNATMCATTR.T	(Ions score 88)
420 - 435	627.6337	1879.8793	1879.8743	3	0	K.VEFVHMLNATMCATTR.T	(Ions score 66)
420 - 435	627.6337	1879.8793	1879.8743	3	0	K.VEFVHMLNATMCATTR.T	(Ions score 92)
420 - 435	627.6337	1879.8793	1879.8743	3	0	K.VEFVHMLNATMCATTR.T	(Ions score 74)
420 - 435	627.6337	1879.8793	1879.8743	3	0	K.VEFVHMLNATMCATTR.T	(Ions score 86)
420 - 435	627.6337	1879.8794	1879.8743	3	0	K.VEFVHMLNATMCATTR.T	(Ions score 79)
420 - 435	627.6337	1879.8794	1879.8743	3	0	K.VEFVHMLNATMCATTR.T	(Ions score 87)
420 - 435	627.6337	1879.8794	1879.8743	3	0	K.VEFVHMLNATMCATTR.T	(Ions score 67)
420 - 435	940.9470	1879.8794	1879.8743	3	0	K.VEFVHMLNATMCATTR.T	(Ions score 54)
420 - 435	627.6337	1879.8794	1879.8743	3	0	K.VEFVHMLNATMCATTR.T	(Ions score 75)
420 - 435	627.6338	1879.8794	1879.8743	3	0	K.VEFVHMLNATMCATTR.T	(Ions score 65)
420 - 435	627.6338	1879.8794	1879.8743	3	0	K.VEFVHMLNATMCATTR.T	(Ions score 90)

420 - 435	627.6338	1879.8794	1879.8743	3	0	K.VEFVHMLNATMCATTR.T	(Ions score 86)
420 - 435	627.6338	1879.8795	1879.8743	3	0	K.VEFVHMLNATMCATTR.T	(Ions score 63)
420 - 435	940.9470	1879.8795	1879.8743	3	0	K.VEFVHMLNATMCATTR.T	(Ions score 44)
420 - 435	627.6338	1879.8795	1879.8743	3	0	K.VEFVHMLNATMCATTR.T	(Ions score 68)
420 - 435	627.6338	1879.8795	1879.8743	3	0	K.VEFVHMLNATMCATTR.T	(Ions score 80)
420 - 435	940.9470	1879.8795	1879.8743	3	0	K.VEFVHMLNATMCATTR.T	(Ions score 79)
420 - 435	940.9470	1879.8795	1879.8743	3	0	K.VEFVHMLNATMCATTR.T	(Ions score 72)
420 - 435	940.9470	1879.8795	1879.8743	3	0	K.VEFVHMLNATMCATTR.T	(Ions score 36)
420 - 435	627.6338	1879.8795	1879.8743	3	0	K.VEFVHMLNATMCATTR.T	(Ions score 80)
420 - 435	940.9471	1879.8796	1879.8743	3	0	K.VEFVHMLNATMCATTR.T	(Ions score 57)
420 - 435	627.6338	1879.8796	1879.8743	3	0	K.VEFVHMLNATMCATTR.T	(Ions score 87)
420 - 435	627.6338	1879.8796	1879.8743	3	0	K.VEFVHMLNATMCATTR.T	(Ions score 79)
420 - 435	627.6338	1879.8796	1879.8743	3	0	K.VEFVHMLNATMCATTR.T	(Ions score 72)
420 - 435	940.9471	1879.8796	1879.8743	3	0	K.VEFVHMLNATMCATTR.T	(Ions score 67)
420 - 435	627.6338	1879.8796	1879.8743	3	0	K.VEFVHMLNATMCATTR.T	(Ions score 63)
420 - 435	627.6338	1879.8796	1879.8743	3	0	K.VEFVHMLNATMCATTR.T	(Ions score 80)
420 - 435	627.6338	1879.8796	1879.8743	3	0	K.VEFVHMLNATMCATTR.T	(Ions score 66)
420 - 435	940.9471	1879.8796	1879.8743	3	0	K.VEFVHMLNATMCATTR.T	(Ions score 64)
420 - 435	627.6338	1879.8796	1879.8743	3	0	K.VEFVHMLNATMCATTR.T	(Ions score 79)
420 - 435	940.9471	1879.8797	1879.8743	3	0	K.VEFVHMLNATMCATTR.T	(Ions score 68)
420 - 435	627.6338	1879.8797	1879.8743	3	0	K.VEFVHMLNATMCATTR.T	(Ions score 63)
420 - 435	627.6338	1879.8797	1879.8743	3	0	K.VEFVHMLNATMCATTR.T	(Ions score 45)
420 - 435	627.6338	1879.8797	1879.8743	3	0	K.VEFVHMLNATMCATTR.T	(Ions score 63)
420 - 435	940.9471	1879.8797	1879.8743	3	0	K.VEFVHMLNATMCATTR.T	(Ions score 49)
420 - 435	627.6338	1879.8797	1879.8743	3	0	K.VEFVHMLNATMCATTR.T	(Ions score 47)
420 - 435	627.6338	1879.8797	1879.8743	3	0	K.VEFVHMLNATMCATTR.T	(Ions score 71)
420 - 435	627.6338	1879.8797	1879.8743	3	0	K.VEFVHMLNATMCATTR.T	(Ions score 72)
420 - 435	627.6339	1879.8797	1879.8743	3	0	K.VEFVHMLNATMCATTR.T	(Ions score 76)
420 - 435	940.9471	1879.8797	1879.8743	3	0	K.VEFVHMLNATMCATTR.T	(Ions score 74)
420 - 435	627.6339	1879.8798	1879.8743	3	0	K.VEFVHMLNATMCATTR.T	(Ions score 70)
420 - 435	940.9472	1879.8798	1879.8743	3	0	K.VEFVHMLNATMCATTR.T	(Ions score 54)
420 - 435	627.6339	1879.8798	1879.8743	3	0	K.VEFVHMLNATMCATTR.T	(Ions score 85)
420 - 435	627.6339	1879.8798	1879.8743	3	0	K.VEFVHMLNATMCATTR.T	(Ions score 34)
420 - 435	627.6339	1879.8798	1879.8743	3	0	K.VEFVHMLNATMCATTR.T	(Ions score 50)
420 - 435	627.6339	1879.8798	1879.8743	3	0	K.VEFVHMLNATMCATTR.T	(Ions score 75)
420 - 435	627.6339	1879.8798	1879.8743	3	0	K.VEFVHMLNATMCATTR.T	(Ions score 76)
420 - 435	940.9472	1879.8798	1879.8743	3	0	K.VEFVHMLNATMCATTR.T	(Ions score 51)
420 - 435	627.6339	1879.8798	1879.8743	3	0	K.VEFVHMLNATMCATTR.T	(Ions score 87)
420 - 435	940.9472	1879.8798	1879.8743	3	0	K.VEFVHMLNATMCATTR.T	(Ions score 58)
420 - 435	627.6339	1879.8798	1879.8743	3	0	K.VEFVHMLNATMCATTR.T	(Ions score 87)
420 - 435	940.9472	1879.8798	1879.8743	3	0	K.VEFVHMLNATMCATTR.T	(Ions score 37)
420 - 435	627.6339	1879.8799	1879.8743	3	0	K.VEFVHMLNATMCATTR.T	(Ions score 87)
420 - 435	627.6339	1879.8799	1879.8743	3	0	K.VEFVHMLNATMCATTR.T	(Ions score 72)
420 - 435	627.6339	1879.8799	1879.8743	3	0	K.VEFVHMLNATMCATTR.T	(Ions score 90)
420 - 435	627.6339	1879.8799	1879.8743	3	0	K.VEFVHMLNATMCATTR.T	(Ions score 80)
420 - 435	627.6339	1879.8799	1879.8743	3	0	K.VEFVHMLNATMCATTR.T	(Ions score 92)
420 - 435	940.9472	1879.8799	1879.8743	3	0	K.VEFVHMLNATMCATTR.T	(Ions score 62)
420 - 435	940.9472	1879.8799	1879.8743	3	0	K.VEFVHMLNATMCATTR.T	(Ions score 58)
420 - 435	627.6339	1879.8799	1879.8743	3	0	K.VEFVHMLNATMCATTR.T	(Ions score 63)
420 - 435	627.6339	1879.8799	1879.8743	3	0	K.VEFVHMLNATMCATTR.T	(Ions score 77)
420 - 435	940.9472	1879.8799	1879.8743	3	0	K.VEFVHMLNATMCATTR.T	(Ions score 61)
420 - 435	940.9473	1879.8800	1879.8743	3	0	K.VEFVHMLNATMCATTR.T	(Ions score 48)
420 - 435	940.9473	1879.8800	1879.8743	3	0	K.VEFVHMLNATMCATTR.T	(Ions score 76)
420 - 435	940.9473	1879.8800	1879.8743	3	0	K.VEFVHMLNATMCATTR.T	(Ions score 50)
420 - 435	627.6339	1879.8800	1879.8743	3	0	K.VEFVHMLNATMCATTR.T	(Ions score 81)
420 - 435	940.9473	1879.8800	1879.8743	3	0	K.VEFVHMLNATMCATTR.T	(Ions score 54)
420 - 435	627.6339	1879.8800	1879.8743	3	0	K.VEFVHMLNATMCATTR.T	(Ions score 76)

420 - 435	940.9473	1879.8800	1879.8743	3	0	K.VEFVHMLNATMCATTR.T	(Ions score 67)
420 - 435	940.9473	1879.8800	1879.8743	3	0	K.VEFVHMLNATMCATTR.T	(Ions score 54)
420 - 435	940.9473	1879.8800	1879.8743	3	0	K.VEFVHMLNATMCATTR.T	(Ions score 57)
420 - 435	627.6340	1879.8800	1879.8743	3	0	K.VEFVHMLNATMCATTR.T	(Ions score 87)
420 - 435	627.6340	1879.8801	1879.8743	3	0	K.VEFVHMLNATMCATTR.T	(Ions score 78)
420 - 435	627.6340	1879.8801	1879.8743	3	0	K.VEFVHMLNATMCATTR.T	(Ions score 72)
420 - 435	627.6340	1879.8801	1879.8743	3	0	K.VEFVHMLNATMCATTR.T	(Ions score 87)
420 - 435	627.6340	1879.8801	1879.8743	3	0	K.VEFVHMLNATMCATTR.T	(Ions score 71)
420 - 435	627.6340	1879.8802	1879.8743	3	0	K.VEFVHMLNATMCATTR.T	(Ions score 87)
420 - 435	627.6340	1879.8802	1879.8743	3	0	K.VEFVHMLNATMCATTR.T	(Ions score 70)
420 - 435	627.6340	1879.8802	1879.8743	3	0	K.VEFVHMLNATMCATTR.T	(Ions score 80)
420 - 435	627.6340	1879.8802	1879.8743	3	0	K.VEFVHMLNATMCATTR.T	(Ions score 68)
420 - 435	940.9474	1879.8802	1879.8743	3	0	K.VEFVHMLNATMCATTR.T	(Ions score 47)
420 - 435	627.6340	1879.8802	1879.8743	3	0	K.VEFVHMLNATMCATTR.T	(Ions score 74)
420 - 435	940.9474	1879.8803	1879.8743	3	0	K.VEFVHMLNATMCATTR.T	(Ions score 61)
420 - 435	627.6341	1879.8803	1879.8743	3	0	K.VEFVHMLNATMCATTR.T	(Ions score 81)
420 - 435	627.6341	1879.8803	1879.8743	3	0	K.VEFVHMLNATMCATTR.T	(Ions score 77)
420 - 435	940.9474	1879.8803	1879.8743	3	0	K.VEFVHMLNATMCATTR.T	(Ions score 66)
420 - 435	940.9474	1879.8803	1879.8743	3	0	K.VEFVHMLNATMCATTR.T	(Ions score 46)
420 - 435	627.6341	1879.8803	1879.8743	3	0	K.VEFVHMLNATMCATTR.T	(Ions score 63)
420 - 435	940.9475	1879.8804	1879.8743	3	0	K.VEFVHMLNATMCATTR.T	(Ions score 61)
420 - 435	627.6341	1879.8804	1879.8743	3	0	K.VEFVHMLNATMCATTR.T	(Ions score 62)
420 - 435	627.6341	1879.8804	1879.8743	3	0	K.VEFVHMLNATMCATTR.T	(Ions score 78)
420 - 435	627.6341	1879.8804	1879.8743	3	0	K.VEFVHMLNATMCATTR.T	(Ions score 68)
420 - 435	627.6341	1879.8804	1879.8743	3	0	K.VEFVHMLNATMCATTR.T	(Ions score 75)
420 - 435	627.6341	1879.8805	1879.8743	3	0	K.VEFVHMLNATMCATTR.T	(Ions score 61)
420 - 435	940.9475	1879.8805	1879.8743	3	0	K.VEFVHMLNATMCATTR.T	(Ions score 65)
420 - 435	940.9475	1879.8805	1879.8743	3	0	K.VEFVHMLNATMCATTR.T	(Ions score 63)
420 - 435	627.6341	1879.8805	1879.8743	3	0	K.VEFVHMLNATMCATTR.T	(Ions score 91)
420 - 435	627.6341	1879.8806	1879.8743	3	0	K.VEFVHMLNATMCATTR.T	(Ions score 78)
420 - 435	627.6341	1879.8806	1879.8743	3	0	K.VEFVHMLNATMCATTR.T	(Ions score 70)
420 - 435	627.6342	1879.8806	1879.8743	3	0	K.VEFVHMLNATMCATTR.T	(Ions score 66)
420 - 435	627.6342	1879.8806	1879.8743	3	0	K.VEFVHMLNATMCATTR.T	(Ions score 79)
420 - 435	627.6342	1879.8807	1879.8743	3	0	K.VEFVHMLNATMCATTR.T	(Ions score 29)
420 - 435	627.6342	1879.8807	1879.8743	3	0	K.VEFVHMLNATMCATTR.T	(Ions score 80)
420 - 435	627.6342	1879.8807	1879.8743	3	0	K.VEFVHMLNATMCATTR.T	(Ions score 76)
420 - 435	627.6342	1879.8807	1879.8743	3	0	K.VEFVHMLNATMCATTR.T	(Ions score 80)
420 - 435	627.6342	1879.8807	1879.8743	3	0	K.VEFVHMLNATMCATTR.T	(Ions score 68)
420 - 435	627.6342	1879.8807	1879.8743	3	0	K.VEFVHMLNATMCATTR.T	(Ions score 94)
420 - 435	940.9476	1879.8807	1879.8743	3	0	K.VEFVHMLNATMCATTR.T	(Ions score 67)
420 - 435	627.6342	1879.8807	1879.8743	3	0	K.VEFVHMLNATMCATTR.T	(Ions score 82)
420 - 435	940.9476	1879.8807	1879.8743	3	0	K.VEFVHMLNATMCATTR.T	(Ions score 52)
420 - 435	627.6342	1879.8807	1879.8743	3	0	K.VEFVHMLNATMCATTR.T	(Ions score 61)
420 - 435	627.6342	1879.8808	1879.8743	3	0	K.VEFVHMLNATMCATTR.T	(Ions score 44)
420 - 435	940.9477	1879.8808	1879.8743	3	0	K.VEFVHMLNATMCATTR.T	(Ions score 47)
420 - 435	627.6342	1879.8808	1879.8743	3	0	K.VEFVHMLNATMCATTR.T	(Ions score 79)
420 - 435	940.9477	1879.8808	1879.8743	3	0	K.VEFVHMLNATMCATTR.T	(Ions score 71)
420 - 435	940.9477	1879.8809	1879.8743	3	0	K.VEFVHMLNATMCATTR.T	(Ions score 62)
420 - 435	627.6342	1879.8809	1879.8743	3	0	K.VEFVHMLNATMCATTR.T	(Ions score 79)
420 - 435	627.6342	1879.8809	1879.8743	4	0	K.VEFVHMLNATMCATTR.T	(Ions score 80)
420 - 435	627.6342	1879.8809	1879.8743	4	0	K.VEFVHMLNATMCATTR.T	(Ions score 76)
420 - 435	940.9478	1879.8810	1879.8743	4	0	K.VEFVHMLNATMCATTR.T	(Ions score 58)
420 - 435	627.6343	1879.8810	1879.8743	4	0	K.VEFVHMLNATMCATTR.T	(Ions score 70)
420 - 435	627.6343	1879.8810	1879.8743	4	0	K.VEFVHMLNATMCATTR.T	(Ions score 81)
420 - 435	627.6343	1879.8811	1879.8743	4	0	K.VEFVHMLNATMCATTR.T	(Ions score 63)
420 - 435	627.6343	1879.8811	1879.8743	4	0	K.VEFVHMLNATMCATTR.T	(Ions score 71)
420 - 435	627.6343	1879.8811	1879.8743	4	0	K.VEFVHMLNATMCATTR.T	(Ions score 87)

420 - 435	627.6343	1879.8811	1879.8743	4	0	K.VEFVHMLNATMCATTR.T	(Ions score 74)
420 - 435	627.6343	1879.8811	1879.8743	4	0	K.VEFVHMLNATMCATTR.T	(Ions score 79)
420 - 435	627.6343	1879.8811	1879.8743	4	0	K.VEFVHMLNATMCATTR.T	(Ions score 73)
420 - 435	627.6343	1879.8811	1879.8743	4	0	K.VEFVHMLNATMCATTR.T	(Ions score 78)
420 - 435	627.6343	1879.8811	1879.8743	4	0	K.VEFVHMLNATMCATTR.T	(Ions score 87)
420 - 435	627.6343	1879.8812	1879.8743	4	0	K.VEFVHMLNATMCATTR.T	(Ions score 79)
420 - 435	627.6343	1879.8812	1879.8743	4	0	K.VEFVHMLNATMCATTR.T	(Ions score 85)
420 - 435	627.6343	1879.8812	1879.8743	4	0	K.VEFVHMLNATMCATTR.T	(Ions score 71)
420 - 435	627.6344	1879.8812	1879.8743	4	0	K.VEFVHMLNATMCATTR.T	(Ions score 78)
420 - 435	627.6344	1879.8813	1879.8743	4	0	K.VEFVHMLNATMCATTR.T	(Ions score 87)
420 - 435	627.6344	1879.8813	1879.8743	4	0	K.VEFVHMLNATMCATTR.T	(Ions score 46)
420 - 435	627.6344	1879.8813	1879.8743	4	0	K.VEFVHMLNATMCATTR.T	(Ions score 78)
420 - 435	940.9479	1879.8813	1879.8743	4	0	K.VEFVHMLNATMCATTR.T	(Ions score 41)
420 - 435	627.6344	1879.8813	1879.8743	4	0	K.VEFVHMLNATMCATTR.T	(Ions score 79)
420 - 435	627.6344	1879.8814	1879.8743	4	0	K.VEFVHMLNATMCATTR.T	(Ions score 69)
420 - 435	627.6344	1879.8814	1879.8743	4	0	K.VEFVHMLNATMCATTR.T	(Ions score 82)
420 - 435	940.9480	1879.8814	1879.8743	4	0	K.VEFVHMLNATMCATTR.T	(Ions score 53)
420 - 435	940.9480	1879.8814	1879.8743	4	0	K.VEFVHMLNATMCATTR.T	(Ions score 55)
420 - 435	627.6344	1879.8814	1879.8743	4	0	K.VEFVHMLNATMCATTR.T	(Ions score 85)
420 - 435	940.9480	1879.8814	1879.8743	4	0	K.VEFVHMLNATMCATTR.T	(Ions score 54)
420 - 435	627.6344	1879.8815	1879.8743	4	0	K.VEFVHMLNATMCATTR.T	(Ions score 87)
420 - 435	940.9480	1879.8815	1879.8743	4	0	K.VEFVHMLNATMCATTR.T	(Ions score 46)
420 - 435	627.6345	1879.8815	1879.8743	4	0	K.VEFVHMLNATMCATTR.T	(Ions score 63)
420 - 435	627.6345	1879.8815	1879.8743	4	0	K.VEFVHMLNATMCATTR.T	(Ions score 89)
420 - 435	627.6345	1879.8815	1879.8743	4	0	K.VEFVHMLNATMCATTR.T	(Ions score 80)
420 - 435	627.6345	1879.8816	1879.8743	4	0	K.VEFVHMLNATMCATTR.T	(Ions score 66)
420 - 435	627.6345	1879.8816	1879.8743	4	0	K.VEFVHMLNATMCATTR.T	(Ions score 86)
420 - 435	627.6345	1879.8816	1879.8743	4	0	K.VEFVHMLNATMCATTR.T	(Ions score 64)
420 - 435	940.9481	1879.8816	1879.8743	4	0	K.VEFVHMLNATMCATTR.T	(Ions score 54)
420 - 435	627.6345	1879.8817	1879.8743	4	0	K.VEFVHMLNATMCATTR.T	(Ions score 76)
420 - 435	940.9481	1879.8817	1879.8743	4	0	K.VEFVHMLNATMCATTR.T	(Ions score 50)
420 - 435	627.6345	1879.8817	1879.8743	4	0	K.VEFVHMLNATMCATTR.T	(Ions score 80)
420 - 435	627.6345	1879.8817	1879.8743	4	0	K.VEFVHMLNATMCATTR.T	(Ions score 60)
420 - 435	627.6345	1879.8817	1879.8743	4	0	K.VEFVHMLNATMCATTR.T	(Ions score 75)
420 - 435	627.6345	1879.8817	1879.8743	4	0	K.VEFVHMLNATMCATTR.T	(Ions score 64)
420 - 435	627.6345	1879.8818	1879.8743	4	0	K.VEFVHMLNATMCATTR.T	(Ions score 86)
420 - 435	940.9482	1879.8818	1879.8743	4	0	K.VEFVHMLNATMCATTR.T	(Ions score 60)
420 - 435	940.9482	1879.8819	1879.8743	4	0	K.VEFVHMLNATMCATTR.T	(Ions score 50)
420 - 435	627.6346	1879.8819	1879.8743	4	0	K.VEFVHMLNATMCATTR.T	(Ions score 85)
420 - 435	940.9482	1879.8819	1879.8743	4	0	K.VEFVHMLNATMCATTR.T	(Ions score 55)
420 - 435	940.9482	1879.8819	1879.8743	4	0	K.VEFVHMLNATMCATTR.T	(Ions score 65)
420 - 435	627.6346	1879.8819	1879.8743	4	0	K.VEFVHMLNATMCATTR.T	(Ions score 79)
420 - 435	627.6346	1879.8819	1879.8743	4	0	K.VEFVHMLNATMCATTR.T	(Ions score 58)
420 - 435	940.9483	1879.8821	1879.8743	4	0	K.VEFVHMLNATMCATTR.T	(Ions score 53)
420 - 435	627.6346	1879.8821	1879.8743	4	0	K.VEFVHMLNATMCATTR.T	(Ions score 60)
420 - 435	627.6347	1879.8821	1879.8743	4	0	K.VEFVHMLNATMCATTR.T	(Ions score 80)
420 - 435	940.9483	1879.8821	1879.8743	4	0	K.VEFVHMLNATMCATTR.T	(Ions score 29)
420 - 435	940.9483	1879.8821	1879.8743	4	0	K.VEFVHMLNATMCATTR.T	(Ions score 47)
420 - 435	627.6347	1879.8821	1879.8743	4	0	K.VEFVHMLNATMCATTR.T	(Ions score 90)
420 - 435	940.9484	1879.8821	1879.8743	4	0	K.VEFVHMLNATMCATTR.T	(Ions score 64)
420 - 435	940.9484	1879.8822	1879.8743	4	0	K.VEFVHMLNATMCATTR.T	(Ions score 46)
420 - 435	627.6347	1879.8822	1879.8743	4	0	K.VEFVHMLNATMCATTR.T	(Ions score 72)
420 - 435	627.6347	1879.8822	1879.8743	4	0	K.VEFVHMLNATMCATTR.T	(Ions score 79)
420 - 435	940.9484	1879.8822	1879.8743	4	0	K.VEFVHMLNATMCATTR.T	(Ions score 62)
420 - 435	627.6347	1879.8823	1879.8743	4	0	K.VEFVHMLNATMCATTR.T	(Ions score 73)
420 - 435	627.6347	1879.8823	1879.8743	4	0	K.VEFVHMLNATMCATTR.T	(Ions score 78)
420 - 435	627.6347	1879.8823	1879.8743	4	0	K.VEFVHMLNATMCATTR.T	(Ions score 74)

420 - 435	627.6347	1879.8824	1879.8743	4	0	K.VEFVHMLNATMCATTR.T		(Ions score 68)
420 - 435	627.6347	1879.8824	1879.8743	4	0	K.VEFVHMLNATMCATTR.T		(Ions score 79)
420 - 435	627.6348	1879.8824	1879.8743	4	0	K.VEFVHMLNATMCATTR.T		(Ions score 79)
420 - 435	627.6348	1879.8824	1879.8743	4	0	K.VEFVHMLNATMCATTR.T		(Ions score 82)
420 - 435	627.6348	1879.8825	1879.8743	4	0	K.VEFVHMLNATMCATTR.T		(Ions score 69)
420 - 435	627.6348	1879.8825	1879.8743	4	0	K.VEFVHMLNATMCATTR.T		(Ions score 63)
420 - 435	940.9485	1879.8825	1879.8743	4	0	K.VEFVHMLNATMCATTR.T		(Ions score 71)
420 - 435	627.6348	1879.8827	1879.8743	4	0	K.VEFVHMLNATMCATTR.T		(Ions score 65)
420 - 435	627.6348	1879.8827	1879.8743	4	0	K.VEFVHMLNATMCATTR.T		(Ions score 73)
420 - 435	627.6348	1879.8827	1879.8743	4	0	K.VEFVHMLNATMCATTR.T		(Ions score 91)
420 - 435	627.6349	1879.8827	1879.8743	4	0	K.VEFVHMLNATMCATTR.T		(Ions score 78)
420 - 435	940.9486	1879.8827	1879.8743	4	0	K.VEFVHMLNATMCATTR.T		(Ions score 58)
420 - 435	940.9487	1879.8828	1879.8743	5	0	K.VEFVHMLNATMCATTR.T		(Ions score 51)
420 - 435	627.6349	1879.8828	1879.8743	5	0	K.VEFVHMLNATMCATTR.T		(Ions score 86)
420 - 435	627.6349	1879.8828	1879.8743	5	0	K.VEFVHMLNATMCATTR.T		(Ions score 53)
420 - 435	627.6349	1879.8828	1879.8743	5	0	K.VEFVHMLNATMCATTR.T		(Ions score 75)
420 - 435	627.6349	1879.8829	1879.8743	5	0	K.VEFVHMLNATMCATTR.T		(Ions score 88)
420 - 435	940.9487	1879.8829	1879.8743	5	0	K.VEFVHMLNATMCATTR.T		(Ions score 73)
420 - 435	627.6349	1879.8830	1879.8743	5	0	K.VEFVHMLNATMCATTR.T		(Ions score 85)
420 - 435	627.6349	1879.8830	1879.8743	5	0	K.VEFVHMLNATMCATTR.T		(Ions score 67)
420 - 435	940.9488	1879.8830	1879.8743	5	0	K.VEFVHMLNATMCATTR.T		(Ions score 76)
420 - 435	627.6350	1879.8831	1879.8743	5	0	K.VEFVHMLNATMCATTR.T		(Ions score 74)
420 - 435	940.9488	1879.8831	1879.8743	5	0	K.VEFVHMLNATMCATTR.T		(Ions score 65)
420 - 435	627.6350	1879.8831	1879.8743	5	0	K.VEFVHMLNATMCATTR.T		(Ions score 60)
420 - 435	940.9488	1879.8831	1879.8743	5	0	K.VEFVHMLNATMCATTR.T		(Ions score 41)
420 - 435	627.6350	1879.8832	1879.8743	5	0	K.VEFVHMLNATMCATTR.T		(Ions score 77)
420 - 435	627.6350	1879.8832	1879.8743	5	0	K.VEFVHMLNATMCATTR.T		(Ions score 89)
420 - 435	627.6350	1879.8833	1879.8743	5	0	K.VEFVHMLNATMCATTR.T		(Ions score 86)
420 - 435	627.6350	1879.8833	1879.8743	5	0	K.VEFVHMLNATMCATTR.T		(Ions score 80)
420 - 435	627.6351	1879.8833	1879.8743	5	0	K.VEFVHMLNATMCATTR.T		(Ions score 91)
420 - 435	627.6351	1879.8835	1879.8743	5	0	K.VEFVHMLNATMCATTR.T		(Ions score 78)
420 - 435	627.6351	1879.8835	1879.8743	5	0	K.VEFVHMLNATMCATTR.T		(Ions score 79)
420 - 435	627.6351	1879.8835	1879.8743	5	0	K.VEFVHMLNATMCATTR.T		(Ions score 75)
420 - 435	627.6351	1879.8835	1879.8743	5	0	K.VEFVHMLNATMCATTR.T		(Ions score 74)
420 - 435	627.6351	1879.8835	1879.8743	5	0	K.VEFVHMLNATMCATTR.T		(Ions score 85)
420 - 435	627.6351	1879.8836	1879.8743	5	0	K.VEFVHMLNATMCATTR.T		(Ions score 67)
420 - 435	940.9491	1879.8836	1879.8743	5	0	K.VEFVHMLNATMCATTR.T		(Ions score 62)
420 - 435	627.6352	1879.8837	1879.8743	5	0	K.VEFVHMLNATMCATTR.T		(Ions score 72)
420 - 435	948.9374	1895.8602	1895.8692	-5	0	K.VEFVHMLNATMCATTR.T	Oxidation (M)	(Ions score 53)
420 - 435	948.9377	1895.8608	1895.8692	-4	0	K.VEFVHMLNATMCATTR.T	Oxidation (M)	(Ions score 33)
420 - 435	948.9384	1895.8622	1895.8692	-4	0	K.VEFVHMLNATMCATTR.T	Oxidation (M)	(Ions score 42)
420 - 435	948.9385	1895.8624	1895.8692	-4	0	K.VEFVHMLNATMCATTR.T	Oxidation (M)	(Ions score 32)
420 - 435	632.9616	1895.8630	1895.8692	-3	0	K.VEFVHMLNATMCATTR.T	Oxidation (M)	(Ions score 69)
420 - 435	948.9388	1895.8630	1895.8692	-3	0	K.VEFVHMLNATMCATTR.T	Oxidation (M)	(Ions score 28)
420 - 435	948.9396	1895.8646	1895.8692	-2	0	K.VEFVHMLNATMCATTR.T	Oxidation (M)	(Ions score 38)
420 - 435	948.9396	1895.8646	1895.8692	-2	0	K.VEFVHMLNATMCATTR.T	Oxidation (M)	(Ions score 25)
420 - 435	948.9396	1895.8646	1895.8692	-2	0	K.VEFVHMLNATMCATTR.T	Oxidation (M)	(Ions score 41)
420 - 435	948.9398	1895.8650	1895.8692	-2	0	K.VEFVHMLNATMCATTR.T	Oxidation (M)	(Ions score 33)
420 - 435	948.9400	1895.8655	1895.8692	-2	0	K.VEFVHMLNATMCATTR.T	Oxidation (M)	(Ions score 43)
420 - 435	948.9401	1895.8657	1895.8692	-2	0	K.VEFVHMLNATMCATTR.T	Oxidation (M)	(Ions score 53)
420 - 435	948.9404	1895.8663	1895.8692	-2	0	K.VEFVHMLNATMCATTR.T	Oxidation (M)	(Ions score 26)
420 - 435	948.9405	1895.8665	1895.8692	-1	0	K.VEFVHMLNATMCATTR.T	Oxidation (M)	(Ions score 40)
420 - 435	948.9405	1895.8665	1895.8692	-1	0	K.VEFVHMLNATMCATTR.T	Oxidation (M)	(Ions score 31)
420 - 435	948.9406	1895.8667	1895.8692	-1	0	K.VEFVHMLNATMCATTR.T	Oxidation (M)	(Ions score 51)
420 - 435	948.9408	1895.8670	1895.8692	-1	0	K.VEFVHMLNATMCATTR.T	Oxidation (M)	(Ions score 41)
420 - 435	948.9408	1895.8670	1895.8692	-1	0	K.VEFVHMLNATMCATTR.T	Oxidation (M)	(Ions score 33)
420 - 435	948.9408	1895.8671	1895.8692	-1	0	K.VEFVHMLNATMCATTR.T	Oxidation (M)	(Ions score 30)

420 - 435	948.9408	1895.8671	1895.8692	-1	0	K.VEFVHMLNATMCATTR.T	Oxidation (M)	(Ions score 30)
420 - 435	632.9630	1895.8671	1895.8692	-1	0	K.VEFVHMLNATMCATTR.T	Oxidation (M)	(Ions score 59)
420 - 435	632.9630	1895.8672	1895.8692	-1	0	K.VEFVHMLNATMCATTR.T	Oxidation (M)	(Ions score 48)
420 - 435	632.9631	1895.8674	1895.8692	-1	0	K.VEFVHMLNATMCATTR.T	Oxidation (M)	(Ions score 58)
420 - 435	948.9410	1895.8675	1895.8692	-1	0	K.VEFVHMLNATMCATTR.T	Oxidation (M)	(Ions score 32)
420 - 435	948.9411	1895.8676	1895.8692	-1	0	K.VEFVHMLNATMCATTR.T	Oxidation (M)	(Ions score 41)
420 - 435	632.9632	1895.8676	1895.8692	-1	0	K.VEFVHMLNATMCATTR.T	Oxidation (M)	(Ions score 73)
420 - 435	948.9412	1895.8679	1895.8692	-1	0	K.VEFVHMLNATMCATTR.T	Oxidation (M)	(Ions score 32)
420 - 435	948.9413	1895.8681	1895.8692	-1	0	K.VEFVHMLNATMCATTR.T	Oxidation (M)	(Ions score 43)
420 - 435	948.9415	1895.8685	1895.8692	-0	0	K.VEFVHMLNATMCATTR.T	Oxidation (M)	(Ions score 42)
420 - 435	948.9415	1895.8685	1895.8692	-0	0	K.VEFVHMLNATMCATTR.T	Oxidation (M)	(Ions score 31)
420 - 435	632.9635	1895.8686	1895.8692	-0	0	K.VEFVHMLNATMCATTR.T	Oxidation (M)	(Ions score 68)
420 - 435	948.9416	1895.8686	1895.8692	-0	0	K.VEFVHMLNATMCATTR.T	Oxidation (M)	(Ions score 54)
420 - 435	948.9416	1895.8687	1895.8692	-0	0	K.VEFVHMLNATMCATTR.T	Oxidation (M)	(Ions score 23)
420 - 435	632.9636	1895.8688	1895.8692	-0	0	K.VEFVHMLNATMCATTR.T	Oxidation (M)	(Ions score 56)
420 - 435	948.9417	1895.8689	1895.8692	-0	0	K.VEFVHMLNATMCATTR.T	Oxidation (M)	(Ions score 56)
420 - 435	948.9417	1895.8689	1895.8692	-0	0	K.VEFVHMLNATMCATTR.T	Oxidation (M)	(Ions score 77)
420 - 435	948.9418	1895.8691	1895.8692	-0	0	K.VEFVHMLNATMCATTR.T	Oxidation (M)	(Ions score 44)
420 - 435	948.9419	1895.8692	1895.8692	-0	0	K.VEFVHMLNATMCATTR.T	Oxidation (M)	(Ions score 62)
420 - 435	948.9419	1895.8692	1895.8692	0	0	K.VEFVHMLNATMCATTR.T	Oxidation (M)	(Ions score 24)
420 - 435	948.9420	1895.8695	1895.8692	0	0	K.VEFVHMLNATMCATTR.T	Oxidation (M)	(Ions score 43)
420 - 435	948.9421	1895.8697	1895.8692	0	0	K.VEFVHMLNATMCATTR.T	Oxidation (M)	(Ions score 44)
420 - 435	632.9639	1895.8699	1895.8692	0	0	K.VEFVHMLNATMCATTR.T	Oxidation (M)	(Ions score 73)
420 - 435	948.9423	1895.8700	1895.8692	0	0	K.VEFVHMLNATMCATTR.T	Oxidation (M)	(Ions score 44)
420 - 435	632.9640	1895.8700	1895.8692	0	0	K.VEFVHMLNATMCATTR.T	Oxidation (M)	(Ions score 52)
420 - 435	948.9423	1895.8701	1895.8692	0	0	K.VEFVHMLNATMCATTR.T	Oxidation (M)	(Ions score 39)
420 - 435	948.9423	1895.8701	1895.8692	0	0	K.VEFVHMLNATMCATTR.T	Oxidation (M)	(Ions score 47)
420 - 435	632.9640	1895.8701	1895.8692	0	0	K.VEFVHMLNATMCATTR.T	Oxidation (M)	(Ions score 73)
420 - 435	948.9424	1895.8703	1895.8692	1	0	K.VEFVHMLNATMCATTR.T	Oxidation (M)	(Ions score 36)
420 - 435	948.9424	1895.8703	1895.8692	1	0	K.VEFVHMLNATMCATTR.T	Oxidation (M)	(Ions score 34)
420 - 435	632.9641	1895.8704	1895.8692	1	0	K.VEFVHMLNATMCATTR.T	Oxidation (M)	(Ions score 73)
420 - 435	948.9425	1895.8704	1895.8692	1	0	K.VEFVHMLNATMCATTR.T	Oxidation (M)	(Ions score 38)
420 - 435	632.9641	1895.8704	1895.8692	1	0	K.VEFVHMLNATMCATTR.T	Oxidation (M)	(Ions score 73)
420 - 435	948.9425	1895.8705	1895.8692	1	0	K.VEFVHMLNATMCATTR.T	Oxidation (M)	(Ions score 65)
420 - 435	948.9425	1895.8705	1895.8692	1	0	K.VEFVHMLNATMCATTR.T	Oxidation (M)	(Ions score 50)
420 - 435	632.9641	1895.8706	1895.8692	1	0	K.VEFVHMLNATMCATTR.T	Oxidation (M)	(Ions score 52)
420 - 435	948.9426	1895.8706	1895.8692	1	0	K.VEFVHMLNATMCATTR.T	Oxidation (M)	(Ions score 44)
420 - 435	948.9426	1895.8706	1895.8692	1	0	K.VEFVHMLNATMCATTR.T	Oxidation (M)	(Ions score 44)
420 - 435	632.9642	1895.8707	1895.8692	1	0	K.VEFVHMLNATMCATTR.T	Oxidation (M)	(Ions score 30)
420 - 435	948.9426	1895.8707	1895.8692	1	0	K.VEFVHMLNATMCATTR.T	Oxidation (M)	(Ions score 54)
420 - 435	948.9426	1895.8707	1895.8692	1	0	K.VEFVHMLNATMCATTR.T	Oxidation (M)	(Ions score 45)
420 - 435	632.9642	1895.8708	1895.8692	1	0	K.VEFVHMLNATMCATTR.T	Oxidation (M)	(Ions score 69)
420 - 435	632.9642	1895.8708	1895.8692	1	0	K.VEFVHMLNATMCATTR.T	Oxidation (M)	(Ions score 47)
420 - 435	948.9427	1895.8708	1895.8692	1	0	K.VEFVHMLNATMCATTR.T	Oxidation (M)	(Ions score 24)
420 - 435	632.9642	1895.8708	1895.8692	1	0	K.VEFVHMLNATMCATTR.T	Oxidation (M)	(Ions score 54)
420 - 435	948.9427	1895.8708	1895.8692	1	0	K.VEFVHMLNATMCATTR.T	Oxidation (M)	(Ions score 30)
420 - 435	632.9642	1895.8709	1895.8692	1	0	K.VEFVHMLNATMCATTR.T	Oxidation (M)	(Ions score 41)
420 - 435	948.9427	1895.8709	1895.8692	1	0	K.VEFVHMLNATMCATTR.T	Oxidation (M)	(Ions score 45)
420 - 435	948.9427	1895.8709	1895.8692	1	0	K.VEFVHMLNATMCATTR.T	Oxidation (M)	(Ions score 31)
420 - 435	632.9643	1895.8709	1895.8692	1	0	K.VEFVHMLNATMCATTR.T	Oxidation (M)	(Ions score 73)
420 - 435	632.9643	1895.8710	1895.8692	1	0	K.VEFVHMLNATMCATTR.T	Oxidation (M)	(Ions score 61)
420 - 435	948.9428	1895.8710	1895.8692	1	0	K.VEFVHMLNATMCATTR.T	Oxidation (M)	(Ions score 49)
420 - 435	948.9428	1895.8710	1895.8692	1	0	K.VEFVHMLNATMCATTR.T	Oxidation (M)	(Ions score 41)
420 - 435	632.9643	1895.8710	1895.8692	1	0	K.VEFVHMLNATMCATTR.T	Oxidation (M)	(Ions score 36)
420 - 435	948.9428	1895.8710	1895.8692	1	0	K.VEFVHMLNATMCATTR.T	Oxidation (M)	(Ions score 52)
420 - 435	948.9428	1895.8711	1895.8692	1	0	K.VEFVHMLNATMCATTR.T	Oxidation (M)	(Ions score 28)
420 - 435	948.9428	1895.8711	1895.8692	1	0	K.VEFVHMLNATMCATTR.T	Oxidation (M)	(Ions score 26)

420 - 435	948.9428	1895.8711	1895.8692	1	0	K.VEFVHMLNATMCATTR.T	Oxidation (M)	(Ions score 42)
420 - 435	948.9428	1895.8711	1895.8692	1	0	K.VEFVHMLNATMCATTR.T	Oxidation (M)	(Ions score 55)
420 - 435	948.9428	1895.8711	1895.8692	1	0	K.VEFVHMLNATMCATTR.T	Oxidation (M)	(Ions score 32)
420 - 435	948.9428	1895.8711	1895.8692	1	0	K.VEFVHMLNATMCATTR.T	Oxidation (M)	(Ions score 45)
420 - 435	632.9643	1895.8712	1895.8692	1	0	K.VEFVHMLNATMCATTR.T	Oxidation (M)	(Ions score 73)
420 - 435	948.9429	1895.8712	1895.8692	1	0	K.VEFVHMLNATMCATTR.T	Oxidation (M)	(Ions score 40)
420 - 435	948.9429	1895.8712	1895.8692	1	0	K.VEFVHMLNATMCATTR.T	Oxidation (M)	(Ions score 64)
420 - 435	632.9644	1895.8713	1895.8692	1	0	K.VEFVHMLNATMCATTR.T	Oxidation (M)	(Ions score 61)
420 - 435	632.9644	1895.8713	1895.8692	1	0	K.VEFVHMLNATMCATTR.T	Oxidation (M)	(Ions score 40)
420 - 435	632.9644	1895.8713	1895.8692	1	0	K.VEFVHMLNATMCATTR.T	Oxidation (M)	(Ions score 50)
420 - 435	948.9429	1895.8713	1895.8692	1	0	K.VEFVHMLNATMCATTR.T	Oxidation (M)	(Ions score 42)
420 - 435	632.9644	1895.8714	1895.8692	1	0	K.VEFVHMLNATMCATTR.T	Oxidation (M)	(Ions score 73)
420 - 435	948.9430	1895.8714	1895.8692	1	0	K.VEFVHMLNATMCATTR.T	Oxidation (M)	(Ions score 51)
420 - 435	948.9430	1895.8714	1895.8692	1	0	K.VEFVHMLNATMCATTR.T	Oxidation (M)	(Ions score 45)
420 - 435	632.9644	1895.8714	1895.8692	1	0	K.VEFVHMLNATMCATTR.T	Oxidation (M)	(Ions score 44)
420 - 435	632.9644	1895.8714	1895.8692	1	0	K.VEFVHMLNATMCATTR.T	Oxidation (M)	(Ions score 44)
420 - 435	948.9430	1895.8714	1895.8692	1	0	K.VEFVHMLNATMCATTR.T	Oxidation (M)	(Ions score 31)
420 - 435	948.9430	1895.8714	1895.8692	1	0	K.VEFVHMLNATMCATTR.T	Oxidation (M)	(Ions score 43)
420 - 435	632.9644	1895.8714	1895.8692	1	0	K.VEFVHMLNATMCATTR.T	Oxidation (M)	(Ions score 73)
420 - 435	632.9644	1895.8714	1895.8692	1	0	K.VEFVHMLNATMCATTR.T	Oxidation (M)	(Ions score 34)
420 - 435	948.9430	1895.8715	1895.8692	1	0	K.VEFVHMLNATMCATTR.T	Oxidation (M)	(Ions score 45)
420 - 435	632.9644	1895.8715	1895.8692	1	0	K.VEFVHMLNATMCATTR.T	Oxidation (M)	(Ions score 76)
420 - 435	948.9430	1895.8715	1895.8692	1	0	K.VEFVHMLNATMCATTR.T	Oxidation (M)	(Ions score 53)
420 - 435	948.9430	1895.8715	1895.8692	1	0	K.VEFVHMLNATMCATTR.T	Oxidation (M)	(Ions score 36)
420 - 435	948.9430	1895.8715	1895.8692	1	0	K.VEFVHMLNATMCATTR.T	Oxidation (M)	(Ions score 36)
420 - 435	632.9645	1895.8715	1895.8692	1	0	K.VEFVHMLNATMCATTR.T	Oxidation (M)	(Ions score 50)
420 - 435	632.9645	1895.8715	1895.8692	1	0	K.VEFVHMLNATMCATTR.T	Oxidation (M)	(Ions score 70)
420 - 435	948.9431	1895.8715	1895.8692	1	0	K.VEFVHMLNATMCATTR.T	Oxidation (M)	(Ions score 28)
420 - 435	632.9645	1895.8716	1895.8692	1	0	K.VEFVHMLNATMCATTR.T	Oxidation (M)	(Ions score 58)
420 - 435	632.9645	1895.8716	1895.8692	1	0	K.VEFVHMLNATMCATTR.T	Oxidation (M)	(Ions score 47)
420 - 435	632.9645	1895.8716	1895.8692	1	0	K.VEFVHMLNATMCATTR.T	Oxidation (M)	(Ions score 65)
420 - 435	948.9431	1895.8716	1895.8692	1	0	K.VEFVHMLNATMCATTR.T	Oxidation (M)	(Ions score 39)
420 - 435	632.9645	1895.8716	1895.8692	1	0	K.VEFVHMLNATMCATTR.T	Oxidation (M)	(Ions score 55)
420 - 435	632.9645	1895.8717	1895.8692	1	0	K.VEFVHMLNATMCATTR.T	Oxidation (M)	(Ions score 57)
420 - 435	948.9431	1895.8717	1895.8692	1	0	K.VEFVHMLNATMCATTR.T	Oxidation (M)	(Ions score 28)
420 - 435	948.9431	1895.8717	1895.8692	1	0	K.VEFVHMLNATMCATTR.T	Oxidation (M)	(Ions score 24)
420 - 435	632.9645	1895.8717	1895.8692	1	0	K.VEFVHMLNATMCATTR.T	Oxidation (M)	(Ions score 23)
420 - 435	948.9431	1895.8717	1895.8692	1	0	K.VEFVHMLNATMCATTR.T	Oxidation (M)	(Ions score 51)
420 - 435	632.9645	1895.8717	1895.8692	1	0	K.VEFVHMLNATMCATTR.T	Oxidation (M)	(Ions score 46)
420 - 435	948.9431	1895.8717	1895.8692	1	0	K.VEFVHMLNATMCATTR.T	Oxidation (M)	(Ions score 23)
420 - 435	632.9645	1895.8718	1895.8692	1	0	K.VEFVHMLNATMCATTR.T	Oxidation (M)	(Ions score 57)
420 - 435	632.9645	1895.8718	1895.8692	1	0	K.VEFVHMLNATMCATTR.T	Oxidation (M)	(Ions score 59)
420 - 435	632.9645	1895.8718	1895.8692	1	0	K.VEFVHMLNATMCATTR.T	Oxidation (M)	(Ions score 77)
420 - 435	632.9645	1895.8718	1895.8692	1	0	K.VEFVHMLNATMCATTR.T	Oxidation (M)	(Ions score 74)
420 - 435	948.9432	1895.8718	1895.8692	1	0	K.VEFVHMLNATMCATTR.T	Oxidation (M)	(Ions score 55)
420 - 435	632.9646	1895.8718	1895.8692	1	0	K.VEFVHMLNATMCATTR.T	Oxidation (M)	(Ions score 59)
420 - 435	632.9646	1895.8718	1895.8692	1	0	K.VEFVHMLNATMCATTR.T	Oxidation (M)	(Ions score 50)
420 - 435	632.9646	1895.8719	1895.8692	1	0	K.VEFVHMLNATMCATTR.T	Oxidation (M)	(Ions score 61)
420 - 435	632.9646	1895.8719	1895.8692	1	0	K.VEFVHMLNATMCATTR.T	Oxidation (M)	(Ions score 71)
420 - 435	948.9432	1895.8719	1895.8692	1	0	K.VEFVHMLNATMCATTR.T	Oxidation (M)	(Ions score 29)
420 - 435	632.9646	1895.8719	1895.8692	1	0	K.VEFVHMLNATMCATTR.T	Oxidation (M)	(Ions score 53)
420 - 435	632.9646	1895.8719	1895.8692	1	0	K.VEFVHMLNATMCATTR.T	Oxidation (M)	(Ions score 65)
420 - 435	632.9646	1895.8719	1895.8692	1	0	K.VEFVHMLNATMCATTR.T	Oxidation (M)	(Ions score 59)
420 - 435	948.9432	1895.8719	1895.8692	1	0	K.VEFVHMLNATMCATTR.T	Oxidation (M)	(Ions score 56)
420 - 435	948.9432	1895.8719	1895.8692	1	0	K.VEFVHMLNATMCATTR.T	Oxidation (M)	(Ions score 37)
420 - 435	948.9433	1895.8720	1895.8692	1	0	K.VEFVHMLNATMCATTR.T	Oxidation (M)	(Ions score 46)
420 - 435	632.9646	1895.8720	1895.8692	1	0	K.VEFVHMLNATMCATTR.T	Oxidation (M)	(Ions score 49)

420 - 435	632.9646	1895.8720	1895.8692	1	0	K.VEFVHMLNATMCATTR.T	Oxidation (M)	(Ions score 65)
420 - 435	632.9646	1895.8720	1895.8692	1	0	K.VEFVHMLNATMCATTR.T	Oxidation (M)	(Ions score 58)
420 - 435	632.9646	1895.8720	1895.8692	1	0	K.VEFVHMLNATMCATTR.T	Oxidation (M)	(Ions score 60)
420 - 435	948.9433	1895.8720	1895.8692	1	0	K.VEFVHMLNATMCATTR.T	Oxidation (M)	(Ions score 42)
420 - 435	632.9646	1895.8720	1895.8692	1	0	K.VEFVHMLNATMCATTR.T	Oxidation (M)	(Ions score 73)
420 - 435	632.9646	1895.8720	1895.8692	1	0	K.VEFVHMLNATMCATTR.T	Oxidation (M)	(Ions score 57)
420 - 435	632.9646	1895.8720	1895.8692	1	0	K.VEFVHMLNATMCATTR.T	Oxidation (M)	(Ions score 74)
420 - 435	632.9646	1895.8721	1895.8692	2	0	K.VEFVHMLNATMCATTR.T	Oxidation (M)	(Ions score 74)
420 - 435	632.9646	1895.8721	1895.8692	2	0	K.VEFVHMLNATMCATTR.T	Oxidation (M)	(Ions score 56)
420 - 435	632.9646	1895.8721	1895.8692	2	0	K.VEFVHMLNATMCATTR.T	Oxidation (M)	(Ions score 23)
420 - 435	632.9646	1895.8721	1895.8692	2	0	K.VEFVHMLNATMCATTR.T	Oxidation (M)	(Ions score 74)
420 - 435	632.9647	1895.8721	1895.8692	2	0	K.VEFVHMLNATMCATTR.T	Oxidation (M)	(Ions score 71)
420 - 435	632.9647	1895.8721	1895.8692	2	0	K.VEFVHMLNATMCATTR.T	Oxidation (M)	(Ions score 59)
420 - 435	632.9647	1895.8722	1895.8692	2	0	K.VEFVHMLNATMCATTR.T	Oxidation (M)	(Ions score 60)
420 - 435	632.9647	1895.8722	1895.8692	2	0	K.VEFVHMLNATMCATTR.T	Oxidation (M)	(Ions score 71)
420 - 435	632.9647	1895.8722	1895.8692	2	0	K.VEFVHMLNATMCATTR.T	Oxidation (M)	(Ions score 75)
420 - 435	632.9647	1895.8722	1895.8692	2	0	K.VEFVHMLNATMCATTR.T	Oxidation (M)	(Ions score 61)
420 - 435	948.9434	1895.8722	1895.8692	2	0	K.VEFVHMLNATMCATTR.T	Oxidation (M)	(Ions score 37)
420 - 435	632.9647	1895.8722	1895.8692	2	0	K.VEFVHMLNATMCATTR.T	Oxidation (M)	(Ions score 71)
420 - 435	632.9647	1895.8722	1895.8692	2	0	K.VEFVHMLNATMCATTR.T	Oxidation (M)	(Ions score 75)
420 - 435	948.9434	1895.8722	1895.8692	2	0	K.VEFVHMLNATMCATTR.T	Oxidation (M)	(Ions score 52)
420 - 435	632.9647	1895.8722	1895.8692	2	0	K.VEFVHMLNATMCATTR.T	Oxidation (M)	(Ions score 60)
420 - 435	632.9647	1895.8723	1895.8692	2	0	K.VEFVHMLNATMCATTR.T	Oxidation (M)	(Ions score 64)
420 - 435	632.9647	1895.8723	1895.8692	2	0	K.VEFVHMLNATMCATTR.T	Oxidation (M)	(Ions score 71)
420 - 435	948.9434	1895.8723	1895.8692	2	0	K.VEFVHMLNATMCATTR.T	Oxidation (M)	(Ions score 32)
420 - 435	632.9647	1895.8723	1895.8692	2	0	K.VEFVHMLNATMCATTR.T	Oxidation (M)	(Ions score 62)
420 - 435	632.9647	1895.8723	1895.8692	2	0	K.VEFVHMLNATMCATTR.T	Oxidation (M)	(Ions score 22)
420 - 435	632.9647	1895.8723	1895.8692	2	0	K.VEFVHMLNATMCATTR.T	Oxidation (M)	(Ions score 45)
420 - 435	632.9647	1895.8723	1895.8692	2	0	K.VEFVHMLNATMCATTR.T	Oxidation (M)	(Ions score 57)
420 - 435	632.9647	1895.8724	1895.8692	2	0	K.VEFVHMLNATMCATTR.T	Oxidation (M)	(Ions score 58)
420 - 435	632.9647	1895.8724	1895.8692	2	0	K.VEFVHMLNATMCATTR.T	Oxidation (M)	(Ions score 50)
420 - 435	632.9647	1895.8724	1895.8692	2	0	K.VEFVHMLNATMCATTR.T	Oxidation (M)	(Ions score 67)
420 - 435	632.9647	1895.8724	1895.8692	2	0	K.VEFVHMLNATMCATTR.T	Oxidation (M)	(Ions score 60)
420 - 435	948.9435	1895.8724	1895.8692	2	0	K.VEFVHMLNATMCATTR.T	Oxidation (M)	(Ions score 42)
420 - 435	948.9435	1895.8725	1895.8692	2	0	K.VEFVHMLNATMCATTR.T	Oxidation (M)	(Ions score 50)
420 - 435	948.9435	1895.8725	1895.8692	2	0	K.VEFVHMLNATMCATTR.T	Oxidation (M)	(Ions score 45)
420 - 435	632.9648	1895.8725	1895.8692	2	0	K.VEFVHMLNATMCATTR.T	Oxidation (M)	(Ions score 55)
420 - 435	632.9648	1895.8725	1895.8692	2	0	K.VEFVHMLNATMCATTR.T	Oxidation (M)	(Ions score 44)
420 - 435	632.9648	1895.8725	1895.8692	2	0	K.VEFVHMLNATMCATTR.T	Oxidation (M)	(Ions score 66)
420 - 435	632.9648	1895.8726	1895.8692	2	0	K.VEFVHMLNATMCATTR.T	Oxidation (M)	(Ions score 57)
420 - 435	632.9648	1895.8726	1895.8692	2	0	K.VEFVHMLNATMCATTR.T	Oxidation (M)	(Ions score 74)
420 - 435	632.9648	1895.8726	1895.8692	2	0	K.VEFVHMLNATMCATTR.T	Oxidation (M)	(Ions score 51)
420 - 435	948.9436	1895.8726	1895.8692	2	0	K.VEFVHMLNATMCATTR.T	Oxidation (M)	(Ions score 56)
420 - 435	632.9648	1895.8726	1895.8692	2	0	K.VEFVHMLNATMCATTR.T	Oxidation (M)	(Ions score 60)
420 - 435	948.9436	1895.8727	1895.8692	2	0	K.VEFVHMLNATMCATTR.T	Oxidation (M)	(Ions score 55)
420 - 435	948.9436	1895.8727	1895.8692	2	0	K.VEFVHMLNATMCATTR.T	Oxidation (M)	(Ions score 30)
420 - 435	632.9648	1895.8727	1895.8692	2	0	K.VEFVHMLNATMCATTR.T	Oxidation (M)	(Ions score 45)
420 - 435	632.9648	1895.8727	1895.8692	2	0	K.VEFVHMLNATMCATTR.T	Oxidation (M)	(Ions score 60)
420 - 435	632.9648	1895.8727	1895.8692	2	0	K.VEFVHMLNATMCATTR.T	Oxidation (M)	(Ions score 41)
420 - 435	632.9648	1895.8727	1895.8692	2	0	K.VEFVHMLNATMCATTR.T	Oxidation (M)	(Ions score 61)
420 - 435	632.9649	1895.8727	1895.8692	2	0	K.VEFVHMLNATMCATTR.T	Oxidation (M)	(Ions score 72)
420 - 435	632.9649	1895.8727	1895.8692	2	0	K.VEFVHMLNATMCATTR.T	Oxidation (M)	(Ions score 54)
420 - 435	632.9649	1895.8727	1895.8692	2	0	K.VEFVHMLNATMCATTR.T	Oxidation (M)	(Ions score 52)
420 - 435	632.9649	1895.8727	1895.8692	2	0	K.VEFVHMLNATMCATTR.T	Oxidation (M)	(Ions score 76)
420 - 435	632.9649	1895.8728	1895.8692	2	0	K.VEFVHMLNATMCATTR.T	Oxidation (M)	(Ions score 40)
420 - 435	632.9649	1895.8728	1895.8692	2	0	K.VEFVHMLNATMCATTR.T	Oxidation (M)	(Ions score 75)
420 - 435	632.9649	1895.8728	1895.8692	2	0	K.VEFVHMLNATMCATTR.T	Oxidation (M)	(Ions score 60)

420 - 435	948.9437	1895.8728	1895.8692	2	0	K.VEFVHMLNATMCATTR.T	Oxidation (M)	(Ions score 52)
420 - 435	632.9649	1895.8728	1895.8692	2	0	K.VEFVHMLNATMCATTR.T	Oxidation (M)	(Ions score 68)
420 - 435	632.9649	1895.8728	1895.8692	2	0	K.VEFVHMLNATMCATTR.T	Oxidation (M)	(Ions score 59)
420 - 435	632.9649	1895.8728	1895.8692	2	0	K.VEFVHMLNATMCATTR.T	Oxidation (M)	(Ions score 48)
420 - 435	632.9649	1895.8728	1895.8692	2	0	K.VEFVHMLNATMCATTR.T	Oxidation (M)	(Ions score 65)
420 - 435	632.9649	1895.8729	1895.8692	2	0	K.VEFVHMLNATMCATTR.T	Oxidation (M)	(Ions score 73)
420 - 435	632.9649	1895.8729	1895.8692	2	0	K.VEFVHMLNATMCATTR.T	Oxidation (M)	(Ions score 61)
420 - 435	632.9649	1895.8729	1895.8692	2	0	K.VEFVHMLNATMCATTR.T	Oxidation (M)	(Ions score 70)
420 - 435	632.9649	1895.8729	1895.8692	2	0	K.VEFVHMLNATMCATTR.T	Oxidation (M)	(Ions score 46)
420 - 435	948.9437	1895.8729	1895.8692	2	0	K.VEFVHMLNATMCATTR.T	Oxidation (M)	(Ions score 45)
420 - 435	632.9649	1895.8729	1895.8692	2	0	K.VEFVHMLNATMCATTR.T	Oxidation (M)	(Ions score 40)
420 - 435	948.9437	1895.8729	1895.8692	2	0	K.VEFVHMLNATMCATTR.T	Oxidation (M)	(Ions score 53)
420 - 435	632.9649	1895.8729	1895.8692	2	0	K.VEFVHMLNATMCATTR.T	Oxidation (M)	(Ions score 65)
420 - 435	632.9649	1895.8730	1895.8692	2	0	K.VEFVHMLNATMCATTR.T	Oxidation (M)	(Ions score 59)
420 - 435	632.9649	1895.8730	1895.8692	2	0	K.VEFVHMLNATMCATTR.T	Oxidation (M)	(Ions score 42)
420 - 435	632.9649	1895.8730	1895.8692	2	0	K.VEFVHMLNATMCATTR.T	Oxidation (M)	(Ions score 53)
420 - 435	632.9649	1895.8730	1895.8692	2	0	K.VEFVHMLNATMCATTR.T	Oxidation (M)	(Ions score 61)
420 - 435	632.9649	1895.8730	1895.8692	2	0	K.VEFVHMLNATMCATTR.T	Oxidation (M)	(Ions score 45)
420 - 435	632.9649	1895.8730	1895.8692	2	0	K.VEFVHMLNATMCATTR.T	Oxidation (M)	(Ions score 59)
420 - 435	948.9438	1895.8730	1895.8692	2	0	K.VEFVHMLNATMCATTR.T	Oxidation (M)	(Ions score 46)
420 - 435	632.9650	1895.8730	1895.8692	2	0	K.VEFVHMLNATMCATTR.T	Oxidation (M)	(Ions score 73)
420 - 435	632.9650	1895.8730	1895.8692	2	0	K.VEFVHMLNATMCATTR.T	Oxidation (M)	(Ions score 53)
420 - 435	632.9650	1895.8731	1895.8692	2	0	K.VEFVHMLNATMCATTR.T	Oxidation (M)	(Ions score 74)
420 - 435	632.9650	1895.8731	1895.8692	2	0	K.VEFVHMLNATMCATTR.T	Oxidation (M)	(Ions score 73)
420 - 435	632.9650	1895.8731	1895.8692	2	0	K.VEFVHMLNATMCATTR.T	Oxidation (M)	(Ions score 42)
420 - 435	632.9650	1895.8731	1895.8692	2	0	K.VEFVHMLNATMCATTR.T	Oxidation (M)	(Ions score 65)
420 - 435	632.9650	1895.8731	1895.8692	2	0	K.VEFVHMLNATMCATTR.T	Oxidation (M)	(Ions score 56)
420 - 435	632.9650	1895.8731	1895.8692	2	0	K.VEFVHMLNATMCATTR.T	Oxidation (M)	(Ions score 63)
420 - 435	948.9438	1895.8731	1895.8692	2	0	K.VEFVHMLNATMCATTR.T	Oxidation (M)	(Ions score 42)
420 - 435	632.9650	1895.8731	1895.8692	2	0	K.VEFVHMLNATMCATTR.T	Oxidation (M)	(Ions score 43)
420 - 435	632.9650	1895.8731	1895.8692	2	0	K.VEFVHMLNATMCATTR.T	Oxidation (M)	(Ions score 59)
420 - 435	632.9650	1895.8731	1895.8692	2	0	K.VEFVHMLNATMCATTR.T	Oxidation (M)	(Ions score 24)
420 - 435	632.9650	1895.8731	1895.8692	2	0	K.VEFVHMLNATMCATTR.T	Oxidation (M)	(Ions score 22)
420 - 435	948.9439	1895.8731	1895.8692	2	0	K.VEFVHMLNATMCATTR.T	Oxidation (M)	(Ions score 46)
420 - 435	948.9439	1895.8732	1895.8692	2	0	K.VEFVHMLNATMCATTR.T	Oxidation (M)	(Ions score 54)
420 - 435	632.9650	1895.8732	1895.8692	2	0	K.VEFVHMLNATMCATTR.T	Oxidation (M)	(Ions score 50)
420 - 435	948.9439	1895.8732	1895.8692	2	0	K.VEFVHMLNATMCATTR.T	Oxidation (M)	(Ions score 51)
420 - 435	632.9650	1895.8732	1895.8692	2	0	K.VEFVHMLNATMCATTR.T	Oxidation (M)	(Ions score 62)
420 - 435	632.9650	1895.8732	1895.8692	2	0	K.VEFVHMLNATMCATTR.T	Oxidation (M)	(Ions score 73)
420 - 435	632.9650	1895.8732	1895.8692	2	0	K.VEFVHMLNATMCATTR.T	Oxidation (M)	(Ions score 59)
420 - 435	948.9439	1895.8732	1895.8692	2	0	K.VEFVHMLNATMCATTR.T	Oxidation (M)	(Ions score 42)
420 - 435	632.9650	1895.8732	1895.8692	2	0	K.VEFVHMLNATMCATTR.T	Oxidation (M)	(Ions score 64)
420 - 435	632.9650	1895.8732	1895.8692	2	0	K.VEFVHMLNATMCATTR.T	Oxidation (M)	(Ions score 71)
420 - 435	948.9439	1895.8732	1895.8692	2	0	K.VEFVHMLNATMCATTR.T	Oxidation (M)	(Ions score 32)
420 - 435	948.9439	1895.8732	1895.8692	2	0	K.VEFVHMLNATMCATTR.T	Oxidation (M)	(Ions score 44)
420 - 435	948.9439	1895.8732	1895.8692	2	0	K.VEFVHMLNATMCATTR.T	Oxidation (M)	(Ions score 43)
420 - 435	632.9650	1895.8732	1895.8692	2	0	K.VEFVHMLNATMCATTR.T	Oxidation (M)	(Ions score 76)
420 - 435	632.9650	1895.8733	1895.8692	2	0	K.VEFVHMLNATMCATTR.T	Oxidation (M)	(Ions score 74)
420 - 435	632.9650	1895.8733	1895.8692	2	0	K.VEFVHMLNATMCATTR.T	Oxidation (M)	(Ions score 58)
420 - 435	632.9650	1895.8733	1895.8692	2	0	K.VEFVHMLNATMCATTR.T	Oxidation (M)	(Ions score 58)
420 - 435	632.9650	1895.8733	1895.8692	2	0	K.VEFVHMLNATMCATTR.T	Oxidation (M)	(Ions score 58)
420 - 435	632.9650	1895.8733	1895.8692	2	0	K.VEFVHMLNATMCATTR.T	Oxidation (M)	(Ions score 58)
420 - 435	632.9650	1895.8733	1895.8692	2	0	K.VEFVHMLNATMCATTR.T	Oxidation (M)	(Ions score 63)
420 - 435	632.9650	1895.8733	1895.8692	2	0	K.VEFVHMLNATMCATTR.T	Oxidation (M)	(Ions score 66)
420 - 435	632.9650	1895.8733	1895.8692	2	0	K.VEFVHMLNATMCATTR.T	Oxidation (M)	(Ions score 56)
420 - 435	632.9650	1895.8733	1895.8692	2	0	K.VEFVHMLNATMCATTR.T	Oxidation (M)	(Ions score 69)
420 - 435	948.9439	1895.8733	1895.8692	2	0	K.VEFVHMLNATMCATTR.T	Oxidation (M)	(Ions score 23)

420 - 435	632.9651	1895.8733	1895.8692	2	0	K.VEFVHMLNATMCATTR.T	Oxidation (M)	(Ions score 75)
420 - 435	632.9651	1895.8733	1895.8692	2	0	K.VEFVHMLNATMCATTR.T	Oxidation (M)	(Ions score 65)
420 - 435	632.9651	1895.8733	1895.8692	2	0	K.VEFVHMLNATMCATTR.T	Oxidation (M)	(Ions score 71)
420 - 435	632.9651	1895.8733	1895.8692	2	0	K.VEFVHMLNATMCATTR.T	Oxidation (M)	(Ions score 48)
420 - 435	948.9440	1895.8733	1895.8692	2	0	K.VEFVHMLNATMCATTR.T	Oxidation (M)	(Ions score 54)
420 - 435	948.9440	1895.8734	1895.8692	2	0	K.VEFVHMLNATMCATTR.T	Oxidation (M)	(Ions score 54)
420 - 435	948.9440	1895.8734	1895.8692	2	0	K.VEFVHMLNATMCATTR.T	Oxidation (M)	(Ions score 57)
420 - 435	948.9440	1895.8734	1895.8692	2	0	K.VEFVHMLNATMCATTR.T	Oxidation (M)	(Ions score 52)
420 - 435	948.9440	1895.8734	1895.8692	2	0	K.VEFVHMLNATMCATTR.T	Oxidation (M)	(Ions score 41)
420 - 435	948.9440	1895.8734	1895.8692	2	0	K.VEFVHMLNATMCATTR.T	Oxidation (M)	(Ions score 49)
420 - 435	632.9651	1895.8734	1895.8692	2	0	K.VEFVHMLNATMCATTR.T	Oxidation (M)	(Ions score 72)
420 - 435	948.9440	1895.8734	1895.8692	2	0	K.VEFVHMLNATMCATTR.T	Oxidation (M)	(Ions score 50)
420 - 435	948.9440	1895.8734	1895.8692	2	0	K.VEFVHMLNATMCATTR.T	Oxidation (M)	(Ions score 41)
420 - 435	948.9440	1895.8734	1895.8692	2	0	K.VEFVHMLNATMCATTR.T	Oxidation (M)	(Ions score 51)
420 - 435	632.9651	1895.8734	1895.8692	2	0	K.VEFVHMLNATMCATTR.T	Oxidation (M)	(Ions score 62)
420 - 435	632.9651	1895.8735	1895.8692	2	0	K.VEFVHMLNATMCATTR.T	Oxidation (M)	(Ions score 58)
420 - 435	632.9651	1895.8735	1895.8692	2	0	K.VEFVHMLNATMCATTR.T	Oxidation (M)	(Ions score 60)
420 - 435	632.9651	1895.8735	1895.8692	2	0	K.VEFVHMLNATMCATTR.T	Oxidation (M)	(Ions score 70)
420 - 435	948.9440	1895.8735	1895.8692	2	0	K.VEFVHMLNATMCATTR.T	Oxidation (M)	(Ions score 41)
420 - 435	632.9651	1895.8735	1895.8692	2	0	K.VEFVHMLNATMCATTR.T	Oxidation (M)	(Ions score 43)
420 - 435	632.9651	1895.8735	1895.8692	2	0	K.VEFVHMLNATMCATTR.T	Oxidation (M)	(Ions score 73)
420 - 435	632.9651	1895.8735	1895.8692	2	0	K.VEFVHMLNATMCATTR.T	Oxidation (M)	(Ions score 73)
420 - 435	632.9651	1895.8735	1895.8692	2	0	K.VEFVHMLNATMCATTR.T	Oxidation (M)	(Ions score 59)
420 - 435	632.9651	1895.8735	1895.8692	2	0	K.VEFVHMLNATMCATTR.T	Oxidation (M)	(Ions score 76)
420 - 435	632.9651	1895.8735	1895.8692	2	0	K.VEFVHMLNATMCATTR.T	Oxidation (M)	(Ions score 63)
420 - 435	632.9651	1895.8735	1895.8692	2	0	K.VEFVHMLNATMCATTR.T	Oxidation (M)	(Ions score 59)
420 - 435	632.9651	1895.8735	1895.8692	2	0	K.VEFVHMLNATMCATTR.T	Oxidation (M)	(Ions score 58)
420 - 435	632.9651	1895.8735	1895.8692	2	0	K.VEFVHMLNATMCATTR.T	Oxidation (M)	(Ions score 32)
420 - 435	632.9651	1895.8736	1895.8692	2	0	K.VEFVHMLNATMCATTR.T	Oxidation (M)	(Ions score 34)
420 - 435	948.9441	1895.8736	1895.8692	2	0	K.VEFVHMLNATMCATTR.T	Oxidation (M)	(Ions score 53)
420 - 435	632.9651	1895.8736	1895.8692	2	0	K.VEFVHMLNATMCATTR.T	Oxidation (M)	(Ions score 29)
420 - 435	632.9651	1895.8736	1895.8692	2	0	K.VEFVHMLNATMCATTR.T	Oxidation (M)	(Ions score 71)
420 - 435	632.9651	1895.8736	1895.8692	2	0	K.VEFVHMLNATMCATTR.T	Oxidation (M)	(Ions score 76)
420 - 435	632.9651	1895.8736	1895.8692	2	0	K.VEFVHMLNATMCATTR.T	Oxidation (M)	(Ions score 73)
420 - 435	948.9441	1895.8736	1895.8692	2	0	K.VEFVHMLNATMCATTR.T	Oxidation (M)	(Ions score 29)
420 - 435	632.9651	1895.8736	1895.8692	2	0	K.VEFVHMLNATMCATTR.T	Oxidation (M)	(Ions score 65)
420 - 435	948.9441	1895.8736	1895.8692	2	0	K.VEFVHMLNATMCATTR.T	Oxidation (M)	(Ions score 45)
420 - 435	948.9441	1895.8736	1895.8692	2	0	K.VEFVHMLNATMCATTR.T	Oxidation (M)	(Ions score 46)
420 - 435	632.9652	1895.8737	1895.8692	2	0	K.VEFVHMLNATMCATTR.T	Oxidation (M)	(Ions score 60)
420 - 435	948.9442	1895.8737	1895.8692	2	0	K.VEFVHMLNATMCATTR.T	Oxidation (M)	(Ions score 43)
420 - 435	948.9442	1895.8738	1895.8692	2	0	K.VEFVHMLNATMCATTR.T	Oxidation (M)	(Ions score 44)
420 - 435	632.9652	1895.8738	1895.8692	2	0	K.VEFVHMLNATMCATTR.T	Oxidation (M)	(Ions score 62)
420 - 435	948.9442	1895.8738	1895.8692	2	0	K.VEFVHMLNATMCATTR.T	Oxidation (M)	(Ions score 41)
420 - 435	632.9652	1895.8738	1895.8692	2	0	K.VEFVHMLNATMCATTR.T	Oxidation (M)	(Ions score 57)
420 - 435	632.9652	1895.8738	1895.8692	2	0	K.VEFVHMLNATMCATTR.T	Oxidation (M)	(Ions score 62)
420 - 435	632.9652	1895.8738	1895.8692	2	0	K.VEFVHMLNATMCATTR.T	Oxidation (M)	(Ions score 52)
420 - 435	632.9652	1895.8738	1895.8692	2	0	K.VEFVHMLNATMCATTR.T	Oxidation (M)	(Ions score 54)
420 - 435	632.9652	1895.8738	1895.8692	2	0	K.VEFVHMLNATMCATTR.T	Oxidation (M)	(Ions score 67)
420 - 435	948.9442	1895.8738	1895.8692	2	0	K.VEFVHMLNATMCATTR.T	Oxidation (M)	(Ions score 53)
420 - 435	948.9442	1895.8738	1895.8692	2	0	K.VEFVHMLNATMCATTR.T	Oxidation (M)	(Ions score 45)
420 - 435	632.9652	1895.8739	1895.8692	2	0	K.VEFVHMLNATMCATTR.T	Oxidation (M)	(Ions score 63)
420 - 435	948.9442	1895.8739	1895.8692	2	0	K.VEFVHMLNATMCATTR.T	Oxidation (M)	(Ions score 22)
420 - 435	948.9442	1895.8739	1895.8692	2	0	K.VEFVHMLNATMCATTR.T	Oxidation (M)	(Ions score 53)
420 - 435	632.9652	1895.8739	1895.8692	2	0	K.VEFVHMLNATMCATTR.T	Oxidation (M)	(Ions score 58)
420 - 435	632.9652	1895.8739	1895.8692	2	0	K.VEFVHMLNATMCATTR.T	Oxidation (M)	(Ions score 74)
420 - 435	632.9652	1895.8739	1895.8692	2	0	K.VEFVHMLNATMCATTR.T	Oxidation (M)	(Ions score 57)
420 - 435	632.9652	1895.8739	1895.8692	2	0	K.VEFVHMLNATMCATTR.T	Oxidation (M)	(Ions score 74)

420 - 435	948.9442	1895.8739	1895.8692	2	0	K.VEFVHMLNATMCATTR.T	Oxidation (M)	(Ions score 53)
420 - 435	948.9442	1895.8739	1895.8692	2	0	K.VEFVHMLNATMCATTR.T	Oxidation (M)	(Ions score 42)
420 - 435	948.9443	1895.8739	1895.8692	2	0	K.VEFVHMLNATMCATTR.T	Oxidation (M)	(Ions score 44)
420 - 435	632.9653	1895.8740	1895.8692	2	0	K.VEFVHMLNATMCATTR.T	Oxidation (M)	(Ions score 70)
420 - 435	948.9443	1895.8740	1895.8692	3	0	K.VEFVHMLNATMCATTR.T	Oxidation (M)	(Ions score 56)
420 - 435	632.9653	1895.8740	1895.8692	3	0	K.VEFVHMLNATMCATTR.T	Oxidation (M)	(Ions score 28)
420 - 435	632.9653	1895.8740	1895.8692	3	0	K.VEFVHMLNATMCATTR.T	Oxidation (M)	(Ions score 72)
420 - 435	632.9653	1895.8740	1895.8692	3	0	K.VEFVHMLNATMCATTR.T	Oxidation (M)	(Ions score 68)
420 - 435	632.9653	1895.8740	1895.8692	3	0	K.VEFVHMLNATMCATTR.T	Oxidation (M)	(Ions score 55)
420 - 435	632.9653	1895.8740	1895.8692	3	0	K.VEFVHMLNATMCATTR.T	Oxidation (M)	(Ions score 61)
420 - 435	948.9443	1895.8740	1895.8692	3	0	K.VEFVHMLNATMCATTR.T	Oxidation (M)	(Ions score 29)
420 - 435	632.9653	1895.8740	1895.8692	3	0	K.VEFVHMLNATMCATTR.T	Oxidation (M)	(Ions score 63)
420 - 435	948.9443	1895.8740	1895.8692	3	0	K.VEFVHMLNATMCATTR.T	Oxidation (M)	(Ions score 55)
420 - 435	948.9443	1895.8741	1895.8692	3	0	K.VEFVHMLNATMCATTR.T	Oxidation (M)	(Ions score 24)
420 - 435	632.9653	1895.8742	1895.8692	3	0	K.VEFVHMLNATMCATTR.T	Oxidation (M)	(Ions score 60)
420 - 435	632.9653	1895.8742	1895.8692	3	0	K.VEFVHMLNATMCATTR.T	Oxidation (M)	(Ions score 58)
420 - 435	632.9653	1895.8742	1895.8692	3	0	K.VEFVHMLNATMCATTR.T	Oxidation (M)	(Ions score 61)
420 - 435	632.9653	1895.8742	1895.8692	3	0	K.VEFVHMLNATMCATTR.T	Oxidation (M)	(Ions score 58)
420 - 435	632.9653	1895.8742	1895.8692	3	0	K.VEFVHMLNATMCATTR.T	Oxidation (M)	(Ions score 60)
420 - 435	948.9444	1895.8742	1895.8692	3	0	K.VEFVHMLNATMCATTR.T	Oxidation (M)	(Ions score 42)
420 - 435	948.9444	1895.8742	1895.8692	3	0	K.VEFVHMLNATMCATTR.T	Oxidation (M)	(Ions score 35)
420 - 435	632.9653	1895.8742	1895.8692	3	0	K.VEFVHMLNATMCATTR.T	Oxidation (M)	(Ions score 59)
420 - 435	632.9654	1895.8742	1895.8692	3	0	K.VEFVHMLNATMCATTR.T	Oxidation (M)	(Ions score 47)
420 - 435	632.9654	1895.8742	1895.8692	3	0	K.VEFVHMLNATMCATTR.T	Oxidation (M)	(Ions score 55)
420 - 435	632.9654	1895.8742	1895.8692	3	0	K.VEFVHMLNATMCATTR.T	Oxidation (M)	(Ions score 46)
420 - 435	632.9654	1895.8742	1895.8692	3	0	K.VEFVHMLNATMCATTR.T	Oxidation (M)	(Ions score 60)
420 - 435	632.9654	1895.8743	1895.8692	3	0	K.VEFVHMLNATMCATTR.T	Oxidation (M)	(Ions score 73)
420 - 435	632.9654	1895.8743	1895.8692	3	0	K.VEFVHMLNATMCATTR.T	Oxidation (M)	(Ions score 76)
420 - 435	632.9654	1895.8743	1895.8692	3	0	K.VEFVHMLNATMCATTR.T	Oxidation (M)	(Ions score 47)
420 - 435	632.9654	1895.8743	1895.8692	3	0	K.VEFVHMLNATMCATTR.T	Oxidation (M)	(Ions score 75)
420 - 435	632.9654	1895.8743	1895.8692	3	0	K.VEFVHMLNATMCATTR.T	Oxidation (M)	(Ions score 60)
420 - 435	948.9444	1895.8743	1895.8692	3	0	K.VEFVHMLNATMCATTR.T	Oxidation (M)	(Ions score 26)
420 - 435	632.9654	1895.8743	1895.8692	3	0	K.VEFVHMLNATMCATTR.T	Oxidation (M)	(Ions score 61)
420 - 435	632.9654	1895.8744	1895.8692	3	0	K.VEFVHMLNATMCATTR.T	Oxidation (M)	(Ions score 61)
420 - 435	632.9654	1895.8744	1895.8692	3	0	K.VEFVHMLNATMCATTR.T	Oxidation (M)	(Ions score 53)
420 - 435	948.9445	1895.8744	1895.8692	3	0	K.VEFVHMLNATMCATTR.T	Oxidation (M)	(Ions score 28)
420 - 435	632.9654	1895.8744	1895.8692	3	0	K.VEFVHMLNATMCATTR.T	Oxidation (M)	(Ions score 61)
420 - 435	948.9445	1895.8744	1895.8692	3	0	K.VEFVHMLNATMCATTR.T	Oxidation (M)	(Ions score 40)
420 - 435	948.9445	1895.8744	1895.8692	3	0	K.VEFVHMLNATMCATTR.T	Oxidation (M)	(Ions score 50)
420 - 435	632.9654	1895.8744	1895.8692	3	0	K.VEFVHMLNATMCATTR.T	Oxidation (M)	(Ions score 64)
420 - 435	632.9654	1895.8744	1895.8692	3	0	K.VEFVHMLNATMCATTR.T	Oxidation (M)	(Ions score 53)
420 - 435	632.9654	1895.8744	1895.8692	3	0	K.VEFVHMLNATMCATTR.T	Oxidation (M)	(Ions score 42)
420 - 435	948.9445	1895.8744	1895.8692	3	0	K.VEFVHMLNATMCATTR.T	Oxidation (M)	(Ions score 43)
420 - 435	632.9654	1895.8744	1895.8692	3	0	K.VEFVHMLNATMCATTR.T	Oxidation (M)	(Ions score 70)
420 - 435	948.9445	1895.8744	1895.8692	3	0	K.VEFVHMLNATMCATTR.T	Oxidation (M)	(Ions score 24)
420 - 435	632.9654	1895.8745	1895.8692	3	0	K.VEFVHMLNATMCATTR.T	Oxidation (M)	(Ions score 73)
420 - 435	632.9654	1895.8745	1895.8692	3	0	K.VEFVHMLNATMCATTR.T	Oxidation (M)	(Ions score 60)
420 - 435	948.9445	1895.8745	1895.8692	3	0	K.VEFVHMLNATMCATTR.T	Oxidation (M)	(Ions score 30)
420 - 435	632.9655	1895.8745	1895.8692	3	0	K.VEFVHMLNATMCATTR.T	Oxidation (M)	(Ions score 49)
420 - 435	632.9655	1895.8745	1895.8692	3	0	K.VEFVHMLNATMCATTR.T	Oxidation (M)	(Ions score 74)
420 - 435	632.9655	1895.8745	1895.8692	3	0	K.VEFVHMLNATMCATTR.T	Oxidation (M)	(Ions score 39)
420 - 435	632.9655	1895.8745	1895.8692	3	0	K.VEFVHMLNATMCATTR.T	Oxidation (M)	(Ions score 62)
420 - 435	632.9655	1895.8746	1895.8692	3	0	K.VEFVHMLNATMCATTR.T	Oxidation (M)	(Ions score 58)
420 - 435	632.9655	1895.8746	1895.8692	3	0	K.VEFVHMLNATMCATTR.T	Oxidation (M)	(Ions score 66)
420 - 435	632.9655	1895.8746	1895.8692	3	0	K.VEFVHMLNATMCATTR.T	Oxidation (M)	(Ions score 69)
420 - 435	632.9655	1895.8746	1895.8692	3	0	K.VEFVHMLNATMCATTR.T	Oxidation (M)	(Ions score 47)
420 - 435	948.9446	1895.8746	1895.8692	3	0	K.VEFVHMLNATMCATTR.T	Oxidation (M)	(Ions score 43)

420 - 435	632.9655	1895.8746	1895.8692	3	0	K.VEFVHMLNATMCATTR.T	Oxidation (M)	(Ions score 24)
420 - 435	632.9655	1895.8746	1895.8692	3	0	K.VEFVHMLNATMCATTR.T	Oxidation (M)	(Ions score 58)
420 - 435	632.9655	1895.8746	1895.8692	3	0	K.VEFVHMLNATMCATTR.T	Oxidation (M)	(Ions score 62)
420 - 435	632.9655	1895.8746	1895.8692	3	0	K.VEFVHMLNATMCATTR.T	Oxidation (M)	(Ions score 45)
420 - 435	948.9446	1895.8746	1895.8692	3	0	K.VEFVHMLNATMCATTR.T	Oxidation (M)	(Ions score 24)
420 - 435	632.9655	1895.8747	1895.8692	3	0	K.VEFVHMLNATMCATTR.T	Oxidation (M)	(Ions score 64)
420 - 435	948.9446	1895.8747	1895.8692	3	0	K.VEFVHMLNATMCATTR.T	Oxidation (M)	(Ions score 53)
420 - 435	632.9655	1895.8747	1895.8692	3	0	K.VEFVHMLNATMCATTR.T	Oxidation (M)	(Ions score 62)
420 - 435	632.9655	1895.8747	1895.8692	3	0	K.VEFVHMLNATMCATTR.T	Oxidation (M)	(Ions score 60)
420 - 435	948.9446	1895.8747	1895.8692	3	0	K.VEFVHMLNATMCATTR.T	Oxidation (M)	(Ions score 45)
420 - 435	632.9655	1895.8747	1895.8692	3	0	K.VEFVHMLNATMCATTR.T	Oxidation (M)	(Ions score 61)
420 - 435	632.9655	1895.8747	1895.8692	3	0	K.VEFVHMLNATMCATTR.T	Oxidation (M)	(Ions score 70)
420 - 435	632.9655	1895.8747	1895.8692	3	0	K.VEFVHMLNATMCATTR.T	Oxidation (M)	(Ions score 61)
420 - 435	632.9655	1895.8747	1895.8692	3	0	K.VEFVHMLNATMCATTR.T	Oxidation (M)	(Ions score 72)
420 - 435	632.9655	1895.8747	1895.8692	3	0	K.VEFVHMLNATMCATTR.T	Oxidation (M)	(Ions score 76)
420 - 435	632.9655	1895.8747	1895.8692	3	0	K.VEFVHMLNATMCATTR.T	Oxidation (M)	(Ions score 50)
420 - 435	632.9655	1895.8748	1895.8692	3	0	K.VEFVHMLNATMCATTR.T	Oxidation (M)	(Ions score 62)
420 - 435	948.9447	1895.8748	1895.8692	3	0	K.VEFVHMLNATMCATTR.T	Oxidation (M)	(Ions score 49)
420 - 435	948.9447	1895.8748	1895.8692	3	0	K.VEFVHMLNATMCATTR.T	Oxidation (M)	(Ions score 38)
420 - 435	632.9656	1895.8748	1895.8692	3	0	K.VEFVHMLNATMCATTR.T	Oxidation (M)	(Ions score 62)
420 - 435	632.9656	1895.8748	1895.8692	3	0	K.VEFVHMLNATMCATTR.T	Oxidation (M)	(Ions score 43)
420 - 435	948.9447	1895.8749	1895.8692	3	0	K.VEFVHMLNATMCATTR.T	Oxidation (M)	(Ions score 40)
420 - 435	632.9656	1895.8749	1895.8692	3	0	K.VEFVHMLNATMCATTR.T	Oxidation (M)	(Ions score 59)
420 - 435	632.9656	1895.8749	1895.8692	3	0	K.VEFVHMLNATMCATTR.T	Oxidation (M)	(Ions score 73)
420 - 435	632.9656	1895.8749	1895.8692	3	0	K.VEFVHMLNATMCATTR.T	Oxidation (M)	(Ions score 55)
420 - 435	948.9447	1895.8749	1895.8692	3	0	K.VEFVHMLNATMCATTR.T	Oxidation (M)	(Ions score 31)
420 - 435	632.9656	1895.8749	1895.8692	3	0	K.VEFVHMLNATMCATTR.T	Oxidation (M)	(Ions score 57)
420 - 435	632.9656	1895.8749	1895.8692	3	0	K.VEFVHMLNATMCATTR.T	Oxidation (M)	(Ions score 52)
420 - 435	948.9447	1895.8749	1895.8692	3	0	K.VEFVHMLNATMCATTR.T	Oxidation (M)	(Ions score 50)
420 - 435	632.9656	1895.8749	1895.8692	3	0	K.VEFVHMLNATMCATTR.T	Oxidation (M)	(Ions score 55)
420 - 435	632.9656	1895.8749	1895.8692	3	0	K.VEFVHMLNATMCATTR.T	Oxidation (M)	(Ions score 73)
420 - 435	948.9447	1895.8749	1895.8692	3	0	K.VEFVHMLNATMCATTR.T	Oxidation (M)	(Ions score 56)
420 - 435	632.9656	1895.8749	1895.8692	3	0	K.VEFVHMLNATMCATTR.T	Oxidation (M)	(Ions score 64)
420 - 435	632.9656	1895.8749	1895.8692	3	0	K.VEFVHMLNATMCATTR.T	Oxidation (M)	(Ions score 61)
420 - 435	632.9656	1895.8749	1895.8692	3	0	K.VEFVHMLNATMCATTR.T	Oxidation (M)	(Ions score 59)
420 - 435	948.9447	1895.8749	1895.8692	3	0	K.VEFVHMLNATMCATTR.T	Oxidation (M)	(Ions score 49)
420 - 435	632.9656	1895.8749	1895.8692	3	0	K.VEFVHMLNATMCATTR.T	Oxidation (M)	(Ions score 59)
420 - 435	632.9656	1895.8749	1895.8692	3	0	K.VEFVHMLNATMCATTR.T	Oxidation (M)	(Ions score 58)
420 - 435	632.9656	1895.8750	1895.8692	3	0	K.VEFVHMLNATMCATTR.T	Oxidation (M)	(Ions score 55)
420 - 435	632.9656	1895.8750	1895.8692	3	0	K.VEFVHMLNATMCATTR.T	Oxidation (M)	(Ions score 61)
420 - 435	948.9448	1895.8750	1895.8692	3	0	K.VEFVHMLNATMCATTR.T	Oxidation (M)	(Ions score 54)
420 - 435	948.9448	1895.8750	1895.8692	3	0	K.VEFVHMLNATMCATTR.T	Oxidation (M)	(Ions score 38)
420 - 435	948.9448	1895.8750	1895.8692	3	0	K.VEFVHMLNATMCATTR.T	Oxidation (M)	(Ions score 42)
420 - 435	632.9656	1895.8750	1895.8692	3	0	K.VEFVHMLNATMCATTR.T	Oxidation (M)	(Ions score 60)
420 - 435	948.9448	1895.8750	1895.8692	3	0	K.VEFVHMLNATMCATTR.T	Oxidation (M)	(Ions score 62)
420 - 435	632.9656	1895.8750	1895.8692	3	0	K.VEFVHMLNATMCATTR.T	Oxidation (M)	(Ions score 69)
420 - 435	948.9448	1895.8751	1895.8692	3	0	K.VEFVHMLNATMCATTR.T	Oxidation (M)	(Ions score 55)
420 - 435	632.9656	1895.8751	1895.8692	3	0	K.VEFVHMLNATMCATTR.T	Oxidation (M)	(Ions score 44)
420 - 435	632.9656	1895.8751	1895.8692	3	0	K.VEFVHMLNATMCATTR.T	Oxidation (M)	(Ions score 58)
420 - 435	632.9656	1895.8751	1895.8692	3	0	K.VEFVHMLNATMCATTR.T	Oxidation (M)	(Ions score 65)
420 - 435	948.9448	1895.8751	1895.8692	3	0	K.VEFVHMLNATMCATTR.T	Oxidation (M)	(Ions score 50)
420 - 435	632.9656	1895.8751	1895.8692	3	0	K.VEFVHMLNATMCATTR.T	Oxidation (M)	(Ions score 75)
420 - 435	948.9448	1895.8751	1895.8692	3	0	K.VEFVHMLNATMCATTR.T	Oxidation (M)	(Ions score 24)
420 - 435	632.9657	1895.8751	1895.8692	3	0	K.VEFVHMLNATMCATTR.T	Oxidation (M)	(Ions score 58)
420 - 435	632.9657	1895.8752	1895.8692	3	0	K.VEFVHMLNATMCATTR.T	Oxidation (M)	(Ions score 75)
420 - 435	632.9657	1895.8752	1895.8692	3	0	K.VEFVHMLNATMCATTR.T	Oxidation (M)	(Ions score 59)
420 - 435	632.9657	1895.8752	1895.8692	3	0	K.VEFVHMLNATMCATTR.T	Oxidation (M)	(Ions score 61)

420 - 435	632.9657	1895.8752	1895.8692	3	0	K.VEFVHMLNATMCATTR.T	Oxidation (M)	(Ions score 74)
420 - 435	632.9657	1895.8753	1895.8692	3	0	K.VEFVHMLNATMCATTR.T	Oxidation (M)	(Ions score 57)
420 - 435	948.9449	1895.8753	1895.8692	3	0	K.VEFVHMLNATMCATTR.T	Oxidation (M)	(Ions score 65)
420 - 435	632.9657	1895.8753	1895.8692	3	0	K.VEFVHMLNATMCATTR.T	Oxidation (M)	(Ions score 47)
420 - 435	632.9657	1895.8753	1895.8692	3	0	K.VEFVHMLNATMCATTR.T	Oxidation (M)	(Ions score 70)
420 - 435	632.9657	1895.8753	1895.8692	3	0	K.VEFVHMLNATMCATTR.T	Oxidation (M)	(Ions score 64)
420 - 435	632.9657	1895.8753	1895.8692	3	0	K.VEFVHMLNATMCATTR.T	Oxidation (M)	(Ions score 77)
420 - 435	948.9449	1895.8753	1895.8692	3	0	K.VEFVHMLNATMCATTR.T	Oxidation (M)	(Ions score 45)
420 - 435	632.9657	1895.8753	1895.8692	3	0	K.VEFVHMLNATMCATTR.T	Oxidation (M)	(Ions score 65)
420 - 435	948.9449	1895.8753	1895.8692	3	0	K.VEFVHMLNATMCATTR.T	Oxidation (M)	(Ions score 39)
420 - 435	632.9657	1895.8753	1895.8692	3	0	K.VEFVHMLNATMCATTR.T	Oxidation (M)	(Ions score 74)
420 - 435	948.9450	1895.8754	1895.8692	3	0	K.VEFVHMLNATMCATTR.T	Oxidation (M)	(Ions score 36)
420 - 435	948.9450	1895.8754	1895.8692	3	0	K.VEFVHMLNATMCATTR.T	Oxidation (M)	(Ions score 24)
420 - 435	632.9657	1895.8754	1895.8692	3	0	K.VEFVHMLNATMCATTR.T	Oxidation (M)	(Ions score 52)
420 - 435	632.9657	1895.8754	1895.8692	3	0	K.VEFVHMLNATMCATTR.T	Oxidation (M)	(Ions score 76)
420 - 435	632.9657	1895.8754	1895.8692	3	0	K.VEFVHMLNATMCATTR.T	Oxidation (M)	(Ions score 71)
420 - 435	632.9658	1895.8754	1895.8692	3	0	K.VEFVHMLNATMCATTR.T	Oxidation (M)	(Ions score 59)
420 - 435	632.9658	1895.8755	1895.8692	3	0	K.VEFVHMLNATMCATTR.T	Oxidation (M)	(Ions score 65)
420 - 435	948.9450	1895.8755	1895.8692	3	0	K.VEFVHMLNATMCATTR.T	Oxidation (M)	(Ions score 54)
420 - 435	632.9658	1895.8755	1895.8692	3	0	K.VEFVHMLNATMCATTR.T	Oxidation (M)	(Ions score 61)
420 - 435	948.9450	1895.8755	1895.8692	3	0	K.VEFVHMLNATMCATTR.T	Oxidation (M)	(Ions score 45)
420 - 435	948.9450	1895.8755	1895.8692	3	0	K.VEFVHMLNATMCATTR.T	Oxidation (M)	(Ions score 43)
420 - 435	948.9451	1895.8755	1895.8692	3	0	K.VEFVHMLNATMCATTR.T	Oxidation (M)	(Ions score 36)
420 - 435	948.9451	1895.8756	1895.8692	3	0	K.VEFVHMLNATMCATTR.T	Oxidation (M)	(Ions score 43)
420 - 435	632.9658	1895.8756	1895.8692	3	0	K.VEFVHMLNATMCATTR.T	Oxidation (M)	(Ions score 58)
420 - 435	948.9451	1895.8756	1895.8692	3	0	K.VEFVHMLNATMCATTR.T	Oxidation (M)	(Ions score 50)
420 - 435	632.9658	1895.8756	1895.8692	3	0	K.VEFVHMLNATMCATTR.T	Oxidation (M)	(Ions score 60)
420 - 435	632.9658	1895.8756	1895.8692	3	0	K.VEFVHMLNATMCATTR.T	Oxidation (M)	(Ions score 71)
420 - 435	632.9658	1895.8756	1895.8692	3	0	K.VEFVHMLNATMCATTR.T	Oxidation (M)	(Ions score 74)
420 - 435	632.9658	1895.8756	1895.8692	3	0	K.VEFVHMLNATMCATTR.T	Oxidation (M)	(Ions score 60)
420 - 435	632.9658	1895.8756	1895.8692	3	0	K.VEFVHMLNATMCATTR.T	Oxidation (M)	(Ions score 63)
420 - 435	632.9658	1895.8757	1895.8692	3	0	K.VEFVHMLNATMCATTR.T	Oxidation (M)	(Ions score 62)
420 - 435	632.9658	1895.8757	1895.8692	3	0	K.VEFVHMLNATMCATTR.T	Oxidation (M)	(Ions score 59)
420 - 435	632.9658	1895.8757	1895.8692	3	0	K.VEFVHMLNATMCATTR.T	Oxidation (M)	(Ions score 61)
420 - 435	948.9451	1895.8757	1895.8692	3	0	K.VEFVHMLNATMCATTR.T	Oxidation (M)	(Ions score 44)
420 - 435	632.9658	1895.8757	1895.8692	3	0	K.VEFVHMLNATMCATTR.T	Oxidation (M)	(Ions score 62)
420 - 435	948.9451	1895.8757	1895.8692	3	0	K.VEFVHMLNATMCATTR.T	Oxidation (M)	(Ions score 42)
420 - 435	948.9451	1895.8757	1895.8692	3	0	K.VEFVHMLNATMCATTR.T	Oxidation (M)	(Ions score 51)
420 - 435	632.9659	1895.8757	1895.8692	3	0	K.VEFVHMLNATMCATTR.T	Oxidation (M)	(Ions score 59)
420 - 435	632.9659	1895.8757	1895.8692	3	0	K.VEFVHMLNATMCATTR.T	Oxidation (M)	(Ions score 56)
420 - 435	632.9659	1895.8757	1895.8692	3	0	K.VEFVHMLNATMCATTR.T	Oxidation (M)	(Ions score 71)
420 - 435	632.9659	1895.8758	1895.8692	3	0	K.VEFVHMLNATMCATTR.T	Oxidation (M)	(Ions score 73)
420 - 435	632.9659	1895.8758	1895.8692	3	0	K.VEFVHMLNATMCATTR.T	Oxidation (M)	(Ions score 57)
420 - 435	948.9452	1895.8758	1895.8692	3	0	K.VEFVHMLNATMCATTR.T	Oxidation (M)	(Ions score 56)
420 - 435	948.9452	1895.8758	1895.8692	3	0	K.VEFVHMLNATMCATTR.T	Oxidation (M)	(Ions score 54)
420 - 435	632.9659	1895.8758	1895.8692	3	0	K.VEFVHMLNATMCATTR.T	Oxidation (M)	(Ions score 75)
420 - 435	948.9452	1895.8758	1895.8692	3	0	K.VEFVHMLNATMCATTR.T	Oxidation (M)	(Ions score 51)
420 - 435	632.9659	1895.8758	1895.8692	3	0	K.VEFVHMLNATMCATTR.T	Oxidation (M)	(Ions score 73)
420 - 435	948.9452	1895.8758	1895.8692	3	0	K.VEFVHMLNATMCATTR.T	Oxidation (M)	(Ions score 67)
420 - 435	948.9452	1895.8758	1895.8692	3	0	K.VEFVHMLNATMCATTR.T	Oxidation (M)	(Ions score 34)
420 - 435	632.9659	1895.8759	1895.8692	4	0	K.VEFVHMLNATMCATTR.T	Oxidation (M)	(Ions score 56)
420 - 435	632.9659	1895.8759	1895.8692	4	0	K.VEFVHMLNATMCATTR.T	Oxidation (M)	(Ions score 61)
420 - 435	948.9452	1895.8759	1895.8692	4	0	K.VEFVHMLNATMCATTR.T	Oxidation (M)	(Ions score 52)
420 - 435	632.9659	1895.8759	1895.8692	4	0	K.VEFVHMLNATMCATTR.T	Oxidation (M)	(Ions score 66)
420 - 435	632.9659	1895.8759	1895.8692	4	0	K.VEFVHMLNATMCATTR.T	Oxidation (M)	(Ions score 54)
420 - 435	632.9659	1895.8759	1895.8692	4	0	K.VEFVHMLNATMCATTR.T	Oxidation (M)	(Ions score 63)

420 - 435	632.9659	1895.8759	1895.8692	4	0	K.VEFVHMLNATMCATTR.T	Oxidation (M)	(Ions score 65)
420 - 435	632.9659	1895.8760	1895.8692	4	0	K.VEFVHMLNATMCATTR.T	Oxidation (M)	(Ions score 60)
420 - 435	632.9659	1895.8760	1895.8692	4	0	K.VEFVHMLNATMCATTR.T	Oxidation (M)	(Ions score 63)
420 - 435	632.9659	1895.8760	1895.8692	4	0	K.VEFVHMLNATMCATTR.T	Oxidation (M)	(Ions score 73)
420 - 435	632.9659	1895.8760	1895.8692	4	0	K.VEFVHMLNATMCATTR.T	Oxidation (M)	(Ions score 65)
420 - 435	948.9453	1895.8760	1895.8692	4	0	K.VEFVHMLNATMCATTR.T	Oxidation (M)	(Ions score 51)
420 - 435	632.9660	1895.8761	1895.8692	4	0	K.VEFVHMLNATMCATTR.T	Oxidation (M)	(Ions score 72)
420 - 435	632.9660	1895.8761	1895.8692	4	0	K.VEFVHMLNATMCATTR.T	Oxidation (M)	(Ions score 59)
420 - 435	948.9453	1895.8761	1895.8692	4	0	K.VEFVHMLNATMCATTR.T	Oxidation (M)	(Ions score 34)
420 - 435	632.9660	1895.8761	1895.8692	4	0	K.VEFVHMLNATMCATTR.T	Oxidation (M)	(Ions score 70)
420 - 435	948.9453	1895.8761	1895.8692	4	0	K.VEFVHMLNATMCATTR.T	Oxidation (M)	(Ions score 23)
420 - 435	632.9660	1895.8761	1895.8692	4	0	K.VEFVHMLNATMCATTR.T	Oxidation (M)	(Ions score 63)
420 - 435	632.9660	1895.8761	1895.8692	4	0	K.VEFVHMLNATMCATTR.T	Oxidation (M)	(Ions score 61)
420 - 435	948.9454	1895.8762	1895.8692	4	0	K.VEFVHMLNATMCATTR.T	Oxidation (M)	(Ions score 36)
420 - 435	632.9660	1895.8762	1895.8692	4	0	K.VEFVHMLNATMCATTR.T	Oxidation (M)	(Ions score 71)
420 - 435	632.9660	1895.8763	1895.8692	4	0	K.VEFVHMLNATMCATTR.T	Oxidation (M)	(Ions score 64)
420 - 435	632.9660	1895.8763	1895.8692	4	0	K.VEFVHMLNATMCATTR.T	Oxidation (M)	(Ions score 74)
420 - 435	948.9454	1895.8763	1895.8692	4	0	K.VEFVHMLNATMCATTR.T	Oxidation (M)	(Ions score 27)
420 - 435	948.9454	1895.8763	1895.8692	4	0	K.VEFVHMLNATMCATTR.T	Oxidation (M)	(Ions score 36)
420 - 435	632.9661	1895.8763	1895.8692	4	0	K.VEFVHMLNATMCATTR.T	Oxidation (M)	(Ions score 64)
420 - 435	632.9661	1895.8763	1895.8692	4	0	K.VEFVHMLNATMCATTR.T	Oxidation (M)	(Ions score 62)
420 - 435	632.9661	1895.8763	1895.8692	4	0	K.VEFVHMLNATMCATTR.T	Oxidation (M)	(Ions score 71)
420 - 435	632.9661	1895.8764	1895.8692	4	0	K.VEFVHMLNATMCATTR.T	Oxidation (M)	(Ions score 64)
420 - 435	948.9455	1895.8764	1895.8692	4	0	K.VEFVHMLNATMCATTR.T	Oxidation (M)	(Ions score 52)
420 - 435	632.9661	1895.8764	1895.8692	4	0	K.VEFVHMLNATMCATTR.T	Oxidation (M)	(Ions score 62)
420 - 435	632.9661	1895.8764	1895.8692	4	0	K.VEFVHMLNATMCATTR.T	Oxidation (M)	(Ions score 63)
420 - 435	948.9455	1895.8764	1895.8692	4	0	K.VEFVHMLNATMCATTR.T	Oxidation (M)	(Ions score 43)
420 - 435	632.9661	1895.8764	1895.8692	4	0	K.VEFVHMLNATMCATTR.T	Oxidation (M)	(Ions score 72)
420 - 435	948.9455	1895.8765	1895.8692	4	0	K.VEFVHMLNATMCATTR.T	Oxidation (M)	(Ions score 45)
420 - 435	632.9661	1895.8765	1895.8692	4	0	K.VEFVHMLNATMCATTR.T	Oxidation (M)	(Ions score 60)
420 - 435	632.9661	1895.8765	1895.8692	4	0	K.VEFVHMLNATMCATTR.T	Oxidation (M)	(Ions score 71)
420 - 435	948.9455	1895.8765	1895.8692	4	0	K.VEFVHMLNATMCATTR.T	Oxidation (M)	(Ions score 49)
420 - 435	948.9455	1895.8765	1895.8692	4	0	K.VEFVHMLNATMCATTR.T	Oxidation (M)	(Ions score 46)
420 - 435	632.9661	1895.8766	1895.8692	4	0	K.VEFVHMLNATMCATTR.T	Oxidation (M)	(Ions score 76)
420 - 435	632.9661	1895.8766	1895.8692	4	0	K.VEFVHMLNATMCATTR.T	Oxidation (M)	(Ions score 60)
420 - 435	948.9456	1895.8766	1895.8692	4	0	K.VEFVHMLNATMCATTR.T	Oxidation (M)	(Ions score 47)
420 - 435	948.9456	1895.8766	1895.8692	4	0	K.VEFVHMLNATMCATTR.T	Oxidation (M)	(Ions score 36)
420 - 435	632.9662	1895.8766	1895.8692	4	0	K.VEFVHMLNATMCATTR.T	Oxidation (M)	(Ions score 58)
420 - 435	632.9662	1895.8767	1895.8692	4	0	K.VEFVHMLNATMCATTR.T	Oxidation (M)	(Ions score 63)
420 - 435	948.9456	1895.8767	1895.8692	4	0	K.VEFVHMLNATMCATTR.T	Oxidation (M)	(Ions score 40)
420 - 435	948.9456	1895.8767	1895.8692	4	0	K.VEFVHMLNATMCATTR.T	Oxidation (M)	(Ions score 53)
420 - 435	632.9662	1895.8768	1895.8692	4	0	K.VEFVHMLNATMCATTR.T	Oxidation (M)	(Ions score 53)
420 - 435	948.9457	1895.8768	1895.8692	4	0	K.VEFVHMLNATMCATTR.T	Oxidation (M)	(Ions score 46)
420 - 435	948.9457	1895.8768	1895.8692	4	0	K.VEFVHMLNATMCATTR.T	Oxidation (M)	(Ions score 35)
420 - 435	948.9457	1895.8768	1895.8692	4	0	K.VEFVHMLNATMCATTR.T	Oxidation (M)	(Ions score 54)
420 - 435	632.9662	1895.8768	1895.8692	4	0	K.VEFVHMLNATMCATTR.T	Oxidation (M)	(Ions score 42)
420 - 435	632.9662	1895.8768	1895.8692	4	0	K.VEFVHMLNATMCATTR.T	Oxidation (M)	(Ions score 67)
420 - 435	948.9457	1895.8768	1895.8692	4	0	K.VEFVHMLNATMCATTR.T	Oxidation (M)	(Ions score 46)
420 - 435	948.9457	1895.8769	1895.8692	4	0	K.VEFVHMLNATMCATTR.T	Oxidation (M)	(Ions score 39)
420 - 435	632.9662	1895.8769	1895.8692	4	0	K.VEFVHMLNATMCATTR.T	Oxidation (M)	(Ions score 59)
420 - 435	632.9662	1895.8769	1895.8692	4	0	K.VEFVHMLNATMCATTR.T	Oxidation (M)	(Ions score 63)
420 - 435	948.9458	1895.8769	1895.8692	4	0	K.VEFVHMLNATMCATTR.T	Oxidation (M)	(Ions score 57)
420 - 435	948.9458	1895.8770	1895.8692	4	0	K.VEFVHMLNATMCATTR.T	Oxidation (M)	(Ions score 54)
420 - 435	632.9663	1895.8770	1895.8692	4	0	K.VEFVHMLNATMCATTR.T	Oxidation (M)	(Ions score 62)
420 - 435	948.9458	1895.8770	1895.8692	4	0	K.VEFVHMLNATMCATTR.T	Oxidation (M)	(Ions score 43)
420 - 435	632.9663	1895.8770	1895.8692	4	0	K.VEFVHMLNATMCATTR.T	Oxidation (M)	(Ions score 62)
420 - 435	632.9663	1895.8770	1895.8692	4	0	K.VEFVHMLNATMCATTR.T	Oxidation (M)	(Ions score 56)

420 - 435	948.9458	1895.8770	1895.8692	4	0	K.VEFVHMLNATMCATTR.T	Oxidation (M)	(Ions score 51)
420 - 435	948.9458	1895.8771	1895.8692	4	0	K.VEFVHMLNATMCATTR.T	Oxidation (M)	(Ions score 52)
420 - 435	632.9663	1895.8771	1895.8692	4	0	K.VEFVHMLNATMCATTR.T	Oxidation (M)	(Ions score 51)
420 - 435	948.9458	1895.8771	1895.8692	4	0	K.VEFVHMLNATMCATTR.T	Oxidation (M)	(Ions score 32)
420 - 435	632.9663	1895.8771	1895.8692	4	0	K.VEFVHMLNATMCATTR.T	Oxidation (M)	(Ions score 64)
420 - 435	948.9459	1895.8772	1895.8692	4	0	K.VEFVHMLNATMCATTR.T	Oxidation (M)	(Ions score 43)
420 - 435	632.9664	1895.8773	1895.8692	4	0	K.VEFVHMLNATMCATTR.T	Oxidation (M)	(Ions score 60)
420 - 435	632.9664	1895.8773	1895.8692	4	0	K.VEFVHMLNATMCATTR.T	Oxidation (M)	(Ions score 72)
420 - 435	632.9664	1895.8773	1895.8692	4	0	K.VEFVHMLNATMCATTR.T	Oxidation (M)	(Ions score 77)
420 - 435	632.9664	1895.8773	1895.8692	4	0	K.VEFVHMLNATMCATTR.T	Oxidation (M)	(Ions score 76)
420 - 435	632.9664	1895.8774	1895.8692	4	0	K.VEFVHMLNATMCATTR.T	Oxidation (M)	(Ions score 76)
420 - 435	632.9664	1895.8774	1895.8692	4	0	K.VEFVHMLNATMCATTR.T	Oxidation (M)	(Ions score 46)
420 - 435	948.9460	1895.8774	1895.8692	4	0	K.VEFVHMLNATMCATTR.T	Oxidation (M)	(Ions score 51)
420 - 435	948.9460	1895.8774	1895.8692	4	0	K.VEFVHMLNATMCATTR.T	Oxidation (M)	(Ions score 40)
420 - 435	632.9664	1895.8774	1895.8692	4	0	K.VEFVHMLNATMCATTR.T	Oxidation (M)	(Ions score 62)
420 - 435	948.9460	1895.8774	1895.8692	4	0	K.VEFVHMLNATMCATTR.T	Oxidation (M)	(Ions score 32)
420 - 435	948.9460	1895.8774	1895.8692	4	0	K.VEFVHMLNATMCATTR.T	Oxidation (M)	(Ions score 57)
420 - 435	632.9664	1895.8775	1895.8692	4	0	K.VEFVHMLNATMCATTR.T	Oxidation (M)	(Ions score 74)
420 - 435	948.9460	1895.8775	1895.8692	4	0	K.VEFVHMLNATMCATTR.T	Oxidation (M)	(Ions score 53)
420 - 435	948.9460	1895.8775	1895.8692	4	0	K.VEFVHMLNATMCATTR.T	Oxidation (M)	(Ions score 50)
420 - 435	632.9665	1895.8775	1895.8692	4	0	K.VEFVHMLNATMCATTR.T	Oxidation (M)	(Ions score 66)
420 - 435	948.9460	1895.8775	1895.8692	4	0	K.VEFVHMLNATMCATTR.T	Oxidation (M)	(Ions score 54)
420 - 435	632.9665	1895.8776	1895.8692	4	0	K.VEFVHMLNATMCATTR.T	Oxidation (M)	(Ions score 64)
420 - 435	632.9665	1895.8776	1895.8692	4	0	K.VEFVHMLNATMCATTR.T	Oxidation (M)	(Ions score 60)
420 - 435	948.9461	1895.8777	1895.8692	4	0	K.VEFVHMLNATMCATTR.T	Oxidation (M)	(Ions score 55)
420 - 435	948.9461	1895.8777	1895.8692	4	0	K.VEFVHMLNATMCATTR.T	Oxidation (M)	(Ions score 54)
420 - 435	632.9665	1895.8777	1895.8692	5	0	K.VEFVHMLNATMCATTR.T	Oxidation (M)	(Ions score 63)
420 - 435	948.9462	1895.8777	1895.8692	5	0	K.VEFVHMLNATMCATTR.T	Oxidation (M)	(Ions score 38)
420 - 435	632.9665	1895.8778	1895.8692	5	0	K.VEFVHMLNATMCATTR.T	Oxidation (M)	(Ions score 74)
420 - 435	948.9462	1895.8778	1895.8692	5	0	K.VEFVHMLNATMCATTR.T	Oxidation (M)	(Ions score 55)
420 - 435	632.9665	1895.8778	1895.8692	5	0	K.VEFVHMLNATMCATTR.T	Oxidation (M)	(Ions score 60)
420 - 435	632.9665	1895.8778	1895.8692	5	0	K.VEFVHMLNATMCATTR.T	Oxidation (M)	(Ions score 71)
420 - 435	632.9665	1895.8778	1895.8692	5	0	K.VEFVHMLNATMCATTR.T	Oxidation (M)	(Ions score 50)
420 - 435	948.9462	1895.8778	1895.8692	5	0	K.VEFVHMLNATMCATTR.T	Oxidation (M)	(Ions score 54)
420 - 435	948.9462	1895.8778	1895.8692	5	0	K.VEFVHMLNATMCATTR.T	Oxidation (M)	(Ions score 37)
420 - 435	632.9666	1895.8779	1895.8692	5	0	K.VEFVHMLNATMCATTR.T	Oxidation (M)	(Ions score 63)
420 - 435	948.9463	1895.8779	1895.8692	5	0	K.VEFVHMLNATMCATTR.T	Oxidation (M)	(Ions score 42)
420 - 435	948.9463	1895.8781	1895.8692	5	0	K.VEFVHMLNATMCATTR.T	Oxidation (M)	(Ions score 42)
420 - 435	948.9463	1895.8781	1895.8692	5	0	K.VEFVHMLNATMCATTR.T	Oxidation (M)	(Ions score 23)
420 - 435	632.9666	1895.8781	1895.8692	5	0	K.VEFVHMLNATMCATTR.T	Oxidation (M)	(Ions score 77)
420 - 435	632.9667	1895.8782	1895.8692	5	0	K.VEFVHMLNATMCATTR.T	Oxidation (M)	(Ions score 59)
420 - 435	948.9464	1895.8782	1895.8692	5	0	K.VEFVHMLNATMCATTR.T	Oxidation (M)	(Ions score 48)
420 - 435	948.9464	1895.8782	1895.8692	5	0	K.VEFVHMLNATMCATTR.T	Oxidation (M)	(Ions score 36)
420 - 435	632.9667	1895.8783	1895.8692	5	0	K.VEFVHMLNATMCATTR.T	Oxidation (M)	(Ions score 65)
420 - 435	632.9667	1895.8784	1895.8692	5	0	K.VEFVHMLNATMCATTR.T	Oxidation (M)	(Ions score 58)
420 - 435	948.9465	1895.8784	1895.8692	5	0	K.VEFVHMLNATMCATTR.T	Oxidation (M)	(Ions score 38)
420 - 435	632.9667	1895.8784	1895.8692	5	0	K.VEFVHMLNATMCATTR.T	Oxidation (M)	(Ions score 61)
420 - 435	632.9667	1895.8784	1895.8692	5	0	K.VEFVHMLNATMCATTR.T	Oxidation (M)	(Ions score 69)
420 - 435	632.9667	1895.8784	1895.8692	5	0	K.VEFVHMLNATMCATTR.T	Oxidation (M)	(Ions score 74)
420 - 435	632.9668	1895.8785	1895.8692	5	0	K.VEFVHMLNATMCATTR.T	Oxidation (M)	(Ions score 49)
420 - 435	632.9668	1895.8785	1895.8692	5	0	K.VEFVHMLNATMCATTR.T	Oxidation (M)	(Ions score 64)
420 - 435	632.9668	1895.8785	1895.8692	5	0	K.VEFVHMLNATMCATTR.T	Oxidation (M)	(Ions score 63)
420 - 435	632.9668	1895.8786	1895.8692	5	0	K.VEFVHMLNATMCATTR.T	Oxidation (M)	(Ions score 50)
420 - 435	948.9466	1895.8786	1895.8692	5	0	K.VEFVHMLNATMCATTR.T	Oxidation (M)	(Ions score 46)
420 - 435	632.9668	1895.8786	1895.8692	5	0	K.VEFVHMLNATMCATTR.T	Oxidation (M)	(Ions score 62)
420 - 435	632.9668	1895.8786	1895.8692	5	0	K.VEFVHMLNATMCATTR.T	Oxidation (M)	(Ions score 63)
420 - 435	632.9668	1895.8786	1895.8692	5	0	K.VEFVHMLNATMCATTR.T	Oxidation (M)	(Ions score 64)

420 - 435	948.9466	1895.8786	1895.8692	5	0	K.VEFVHMLNATMCATTR.T	Oxidation (M) (Ions score 39)
420 - 435	632.9668	1895.8787	1895.8692	5	0	K.VEFVHMLNATMCATTR.T	Oxidation (M) (Ions score 59)
420 - 435	638.2935	1911.8587	1911.8641	-3	0	K.VEFVHMLNATMCATTR.T	2 Oxidation (M) (Ions score 45)
420 - 435	956.9372	1911.8599	1911.8641	-2	0	K.VEFVHMLNATMCATTR.T	2 Oxidation (M) (Ions score 31)
420 - 435	638.2942	1911.8607	1911.8641	-2	0	K.VEFVHMLNATMCATTR.T	2 Oxidation (M) (Ions score 54)
420 - 435	638.2944	1911.8613	1911.8641	-1	0	K.VEFVHMLNATMCATTR.T	2 Oxidation (M) (Ions score 41)
420 - 435	638.2951	1911.8635	1911.8641	-0	0	K.VEFVHMLNATMCATTR.T	2 Oxidation (M) (Ions score 22)
420 - 435	638.2954	1911.8644	1911.8641	0	0	K.VEFVHMLNATMCATTR.T	2 Oxidation (M) (Ions score 56)
420 - 435	638.2955	1911.8647	1911.8641	0	0	K.VEFVHMLNATMCATTR.T	2 Oxidation (M) (Ions score 32)
420 - 435	638.2956	1911.8649	1911.8641	0	0	K.VEFVHMLNATMCATTR.T	2 Oxidation (M) (Ions score 45)
420 - 435	956.9404	1911.8662	1911.8641	1	0	K.VEFVHMLNATMCATTR.T	2 Oxidation (M) (Ions score 37)
420 - 435	956.9406	1911.8666	1911.8641	1	0	K.VEFVHMLNATMCATTR.T	2 Oxidation (M) (Ions score 42)
420 - 435	956.9406	1911.8666	1911.8641	1	0	K.VEFVHMLNATMCATTR.T	2 Oxidation (M) (Ions score 34)
420 - 435	956.9406	1911.8666	1911.8641	1	0	K.VEFVHMLNATMCATTR.T	2 Oxidation (M) (Ions score 24)
420 - 435	956.9406	1911.8666	1911.8641	1	0	K.VEFVHMLNATMCATTR.T	2 Oxidation (M) (Ions score 50)
420 - 435	956.9407	1911.8668	1911.8641	1	0	K.VEFVHMLNATMCATTR.T	2 Oxidation (M) (Ions score 39)
420 - 435	956.9407	1911.8669	1911.8641	1	0	K.VEFVHMLNATMCATTR.T	2 Oxidation (M) (Ions score 31)
420 - 435	956.9407	1911.8669	1911.8641	1	0	K.VEFVHMLNATMCATTR.T	2 Oxidation (M) (Ions score 29)
420 - 435	638.2963	1911.8671	1911.8641	2	0	K.VEFVHMLNATMCATTR.T	2 Oxidation (M) (Ions score 56)
420 - 435	956.9409	1911.8672	1911.8641	2	0	K.VEFVHMLNATMCATTR.T	2 Oxidation (M) (Ions score 30)
420 - 435	956.9409	1911.8673	1911.8641	2	0	K.VEFVHMLNATMCATTR.T	2 Oxidation (M) (Ions score 23)
420 - 435	638.2964	1911.8674	1911.8641	2	0	K.VEFVHMLNATMCATTR.T	2 Oxidation (M) (Ions score 43)
420 - 435	638.2964	1911.8674	1911.8641	2	0	K.VEFVHMLNATMCATTR.T	2 Oxidation (M) (Ions score 59)
420 - 435	638.2964	1911.8674	1911.8641	2	0	K.VEFVHMLNATMCATTR.T	2 Oxidation (M) (Ions score 57)
420 - 435	638.2964	1911.8675	1911.8641	2	0	K.VEFVHMLNATMCATTR.T	2 Oxidation (M) (Ions score 28)
420 - 435	638.2965	1911.8676	1911.8641	2	0	K.VEFVHMLNATMCATTR.T	2 Oxidation (M) (Ions score 43)
420 - 435	638.2965	1911.8677	1911.8641	2	0	K.VEFVHMLNATMCATTR.T	2 Oxidation (M) (Ions score 46)
420 - 435	638.2965	1911.8677	1911.8641	2	0	K.VEFVHMLNATMCATTR.T	2 Oxidation (M) (Ions score 37)
420 - 435	956.9411	1911.8677	1911.8641	2	0	K.VEFVHMLNATMCATTR.T	2 Oxidation (M) (Ions score 22)
420 - 435	638.2966	1911.8679	1911.8641	2	0	K.VEFVHMLNATMCATTR.T	2 Oxidation (M) (Ions score 46)
420 - 435	638.2966	1911.8680	1911.8641	2	0	K.VEFVHMLNATMCATTR.T	2 Oxidation (M) (Ions score 42)
420 - 435	638.2966	1911.8681	1911.8641	2	0	K.VEFVHMLNATMCATTR.T	2 Oxidation (M) (Ions score 57)
420 - 435	638.2967	1911.8683	1911.8641	2	0	K.VEFVHMLNATMCATTR.T	2 Oxidation (M) (Ions score 56)
420 - 435	638.2967	1911.8684	1911.8641	2	0	K.VEFVHMLNATMCATTR.T	2 Oxidation (M) (Ions score 31)
420 - 435	638.2968	1911.8685	1911.8641	2	0	K.VEFVHMLNATMCATTR.T	2 Oxidation (M) (Ions score 59)
420 - 435	956.9416	1911.8687	1911.8641	2	0	K.VEFVHMLNATMCATTR.T	2 Oxidation (M) (Ions score 29)
420 - 435	638.2969	1911.8688	1911.8641	2	0	K.VEFVHMLNATMCATTR.T	2 Oxidation (M) (Ions score 56)
420 - 435	638.2969	1911.8688	1911.8641	2	0	K.VEFVHMLNATMCATTR.T	2 Oxidation (M) (Ions score 55)
420 - 435	638.2971	1911.8695	1911.8641	3	0	K.VEFVHMLNATMCATTR.T	2 Oxidation (M) (Ions score 23)
420 - 435	638.2971	1911.8696	1911.8641	3	0	K.VEFVHMLNATMCATTR.T	2 Oxidation (M) (Ions score 36)
420 - 435	638.2971	1911.8696	1911.8641	3	0	K.VEFVHMLNATMCATTR.T	2 Oxidation (M) (Ions score 49)
420 - 435	638.2972	1911.8699	1911.8641	3	0	K.VEFVHMLNATMCATTR.T	2 Oxidation (M) (Ions score 54)
420 - 435	638.2974	1911.8703	1911.8641	3	0	K.VEFVHMLNATMCATTR.T	2 Oxidation (M) (Ions score 35)
420 - 435	638.2976	1911.8710	1911.8641	4	0	K.VEFVHMLNATMCATTR.T	2 Oxidation (M) (Ions score 26)
420 - 435	956.9430	1911.8715	1911.8641	4	0	K.VEFVHMLNATMCATTR.T	2 Oxidation (M) (Ions score 34)
420 - 435	638.2980	1911.8723	1911.8641	4	0	K.VEFVHMLNATMCATTR.T	2 Oxidation (M) (Ions score 29)
420 - 435	638.2981	1911.8723	1911.8641	4	0	K.VEFVHMLNATMCATTR.T	2 Oxidation (M) (Ions score 22)
420 - 435	638.2982	1911.8726	1911.8641	4	0	K.VEFVHMLNATMCATTR.T	2 Oxidation (M) (Ions score 56)
420 - 435	638.2983	1911.8729	1911.8641	5	0	K.VEFVHMLNATMCATTR.T	2 Oxidation (M) (Ions score 31)
436 - 448	791.8931	1581.7717	1581.7708	1	0	R.TICAILENYQTEK.G	(Ions score 49)
436 - 448	791.8955	1581.7765	1581.7708	4	0	R.TICAILENYQTEK.G	(Ions score 56)
456 - 482	766.4248	3061.6702	3061.6562	5	1	K.LKEFMPPGLQELIPFVKPAPIEQEPSK.K	(Ions score 23)
458 - 482	946.5006	2836.4798	2836.4721	3	0	K.EFMPPGLQELIPFVKPAPIEQEPSK.K	Oxidation (M) (Ions score 22)
499 - 505	423.7204	845.4262	845.4243	2	0	R.DVTLENR.L	(Ions score 28)
499 - 505	423.7209	845.4273	845.4243	4	0	R.DVTLENR.L	(Ions score 23)

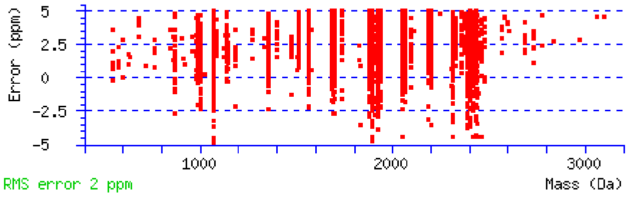

Mascot: http://www.matrixscience.com/

MATRIX SCIENCE Mascot Search Results

```
User                    : Mascot Daemon
Email                   : daemon@localhost
Search title            : 7571 7572 Human (D:\Method_Mascot\NCBI_DB_human-decoy.par), submitted from Daemon on MS-BRUKER-WORK
MS data file            : \\scarletvault.scripps.edu\userdata4\QE_data\2022\Nov 2022\11302022\AABT7571.raw
Database                : NCBInr3  (57851050 sequences; 20740007484 residues)
Taxonomy                : Homo sapiens (human) (273087 sequences)
Timestamp               : 16 Dec 2022 at 19:30:14 GMT
Enzyme                  : Trypsin
Fixed modifications     : Carbamidomethyl (C)
Variable modifications  : Oxidation (M)
Mass values             : Monoisotopic
Protein Mass            : Unrestricted
Peptide Mass Tolerance  : ± 10 ppm
Fragment Mass Tolerance : ± 0.01 Da
Max Missed Cleavages    : 1
Instrument type         : ESI-TRAP
Number of queries       : 70134
Protein hits            : gi|378792521 Chain A, Crystal Structure Of Seryl-Trna Synthetase From Human At 2.9 Angstroms
                          gi|7331218   keratin 1 [Homo sapiens]
                          gi|28317     unnamed protein product [Homo sapiens]
                          gi|1195531   type I keratin 16 [Homo sapiens]
                          gi|435476    cytokeratin 9 [Homo sapiens]
                          gi|12803709  Keratin 14 [Homo sapiens]
                          gi|386849    keratin type II, partial [Homo sapiens]
                          gi|5031839   keratin, type II cytoskeletal 6A [Homo sapiens]
                          gi|181402    epidermal cytokeratin 2 [Homo sapiens]
                          gi|4557701   keratin, type I cytoskeletal 17 [Homo sapiens]
                          gi|386850    keratin K5, partial [Homo sapiens]
                          gi|374977971 Chain A, Crystal Structure Of Branched-chain Alpha-ketoacid Dehydrogenase Phosphatase With Mg (ii) Ions At The Active Site
                          gi|37460     unnamed protein product [Homo sapiens]
                          gi|51873887  DNAJC14 protein, partial [Homo sapiens]
                          gi|1552516   trypsinogen C [Homo sapiens]
                          gi|34533832  unnamed protein product [Homo sapiens]
                          gi|119575127 hCG1987696, isoform CRA_a [Homo sapiens]
                          gi|58330892  interferon-induced protein with tetratricopeptide repeats 1B [Homo sapiens]
                          gi|106258    glycoprotein-fucosylgalactoside alpha-N-acetylgalactosaminyltransferase (EC 2.4.1.40) A1 allele [validated] - human
                          gi|3406800   taxol resistant associated protein [Homo sapiens]
```

	NCBInr3	Decoy	False discovery rate
Peptide matches above identity threshold	7448	75	1.01 %
Peptide matches above homology or identity threshold	7572	160	2.11 %

Select Summary Report

Format As [Select Summary (protein hits)] Help

Significance threshold p< 0.05 Max. number of hits AUTO Show Percolator scores ☐

Standard scoring ◉ MudPIT scoring ○ Ions score or expect cut-off 36 Show sub-sets 1

Show pop-ups ◉ Suppress pop-ups ○ Require bold red ☑

Re-Search ◉ All queries ○ Unassigned ○ Below homology threshold ○ Below identity threshold

1. gi|378792521 **Mass:** 60318 **Score:** 2824 **Matches:** 6102(6102) **Sequences:** 40(40) **emPAI:** 398.62
Chain A, Crystal Structure Of Seryl-Trna Synthetase From Human At 2.9 Angstroms

Query	Observed	Mr(expt)	Mr(calc)	ppm	Miss	Score	Expect	Rank	Unique	Peptide
21978	763.3384	762.3311	762.3297	1.90	0	37	0.035	1	U	K.ADSEWR.R
3171	435.2155	868.4165	868.4151	1.62	0	47	0.0027	1	U	R.QEVGSHGR.D 25660 25661 3151 3154 25662 3158 25663 25664 3164 3165 3166 25665 25666 3167 3168 3170 25667 25668 3172 3176 25671
26870	458.2672	914.5198	914.5185	1.41	1	51	0.00067	1	U	R.ADNLNKLK.N
28247	977.5164	976.5091	976.5090	0.14	1	39	0.016	1	U	R.FRADNLNK.L
7471	495.7861	989.5577	989.5546	3.17	0	72	2.9e-06	1	U	M.VLDLDLFR.V 7296 7297 7299 7300 28497 7302 28498 28499 7304 28501 7305 28503 7307 7309 7311 7312 7315 7316 7317 7321 7326 7327 285

28887	503.7439	1005.4733	1005.4702	3.12	0	42	0.0032	1	U	R.IWGDCTVR.K	28797 8599 28799 28800 28802 28806 28809 28814 28815 28816 28821 28822 28824 28826 28827 28829 28830 28831 28833 288
29430	514.3236	1026.6327	1026.6325	0.24	0	46	0.00024	1	U	R.LLIDEAILK.C	29429
30766	536.7082	1071.4019	1071.3992	2.48	0	69	1.7e-07	1	U	K.SDDNSYDEK.Y	30695 30696 30697 30698 30700 30701 30704 30705 30706 30707 30708 30712 30714 30715 30716 30717 30720 30721 30723 3
31161	537.7586	1073.5027	1073.4964	5.86	0	65	1.1e-05	1	U	K.YAGLSTCFR.Q	11657 11659 30839 11660 30840 30841 30847 30853 30859 30862 30867 30873 30882 30886 30888 30899 30910 30911 30912 3
31311	542.3073	1082.6000	1082.5972	2.65	0	63	2e-05	1	U	K.DPGLVDQLVK.A	31303 31305 31306 31307 12005 12006 31310 31312 31313 31314 31316 31317 31318 31319
12913	561.3060	1120.5975	1120.5951	2.18	0	50	0.0019	1	U	-.MVLDLDLFR.V	12911
32523	569.3033	1136.5920	1136.5900	1.79	0	(36)	0.017	1	U	-.MVLDLDLFR.V	32522
32582	1140.6377	1139.6304	1139.6299	0.48	1	(57)	0.00012	1	U	R.VDKGGDPALIR.E	32580 32583 32585 32586 32589 32594
32588	570.8230	1139.6315	1139.6299	1.40	1	61	2.8e-05	1	U	R.VDKGGDPALIR.E	32584 32587 32591 32592 32593 32595 32596 32597 32599 32601 32602 32603 32605 32607 32608 32609 32610 32611 32615
32708	575.3013	1148.5880	1148.5859	1.77	1	44	0.003	1	U	K.NLCSKTIGEK.M	32704 32706 32707 32709 32712 32713 32714
34262	638.8379	1275.6613	1275.6571	3.29	1	47	0.0015	1	U	K.LEAERFENLR.E	
35739	1358.7699	1357.7626	1357.7606	1.52	1	(40)	0.0038	1	U	R.FKDPGLVDQLVK.A	35776
35820	679.8889	1357.7632	1357.7606	1.96	1	57	6.7e-05	1	U	R.FKDPGLVDQLVK.A	35636 35644 35648 19789 35724 35789 35796 35826 35846 35863 35873 35887 35892 35970 35980 35987 36044 36057 3606
35834	453.5950	1357.7633	1357.7606	2.00	1	(56)	0.0001	1	U	R.FKDPGLVDQLVK.A	35613 35614 35615 35618 35619 35620 35621 35623 35624 35626 35627 35630 35631 35634 35635 35637 35639 35640 3564
38197	737.3044	1472.5942	1472.5903	2.63	1	69	1.2e-06	1	U	K.GSEKSDDNSYDEK.Y	38191 38194 38196 38198
38823	752.8877	1503.7608	1503.7584	1.58	0	39	0.0088	1	U	R.GYIPIYTPFFMR.K	
39131	1510.7928	1509.7856	1509.7827	1.89	0	(60)	7.3e-05	1	U	R.DEWLRPEDLPIK.Y	39228
39217	755.9005	1509.7864	1509.7827	2.42	0	61	4.5e-05	1	U	R.DEWLRPEDLPIK.Y	21817 39118 39145 39149 39152 39172 39177 39178 39183 39184 39186 39193 39194 39199 39206 39208 39212 39213 3921
40969	1563.7454	1562.7381	1562.7365	1.01	0	84	3.1e-07	1	U	K.IEQFVYSSPHDNK.S	40970 40971 40989 41015 41023 41037
41035	782.3773	1562.7399	1562.7365	2.20	0	(75)	1.8e-06	1	U	K.IEQFVYSSPHDNK.S	40942 40943 40945 40946 40947 40949 22921 40950 40951 40953 40954 40955 40956 40957 40959 40960 40961 40964 409
41036	521.9206	1562.7400	1562.7365	2.26	0	(56)	0.00014	1	U	K.IEQFVYSSPHDNK.S	40948 40952 40962 40963 40966 40975 40976 40978 40983 40985 40987 40991 40994 40997 41002 41003 41006 41012 410
41242	522.5975	1564.7708	1564.7674	2.13	0	(42)	0.0071	1	U	K.LDLEAWFPGSGAFR.E	41240 41241 41243 41250 41257
41273	783.3939	1564.7733	1564.7674	3.76	0	64	4.8e-05	1	U	K.LDLEAWFPGSGAFR.E	41229 41234 41235 41238 41239 41244 41246 41247 41248 41249 41253 41255 41256 41258 41261 41262 41265 41268 41
41766	791.8955	1581.7765	1581.7708	3.60	0	56	0.00014	1	U	R.TICAILENYQTEK.G	41761
43458	421.4866	1681.9173	1681.9151	1.31	0	(62)	1.9e-05	1	U	K.YLIATSEQPIAALHR.D	43423 43430 43439 43447 43453 43454 43466 43468 43478 43479 43492 43495 43574 43598
43471	1682.9250	1681.9178	1681.9151	1.58	0	(58)	5.6e-05	1	U	K.YLIATSEQPIAALHR.D	43470 43808
43494	841.9663	1681.9181	1681.9151	1.78	0	105	8.4e-10	1	U	K.YLIATSEQPIAALHR.D	43408 43421 43422 43450 43459 43482 43484 43490 43493 43496 43506 43507 43514 43515 43517 43519 43521 43525 4
43652	561.6473	1681.9201	1681.9151	2.95	0	(91)	2.5e-08	1	U	K.YLIATSEQPIAALHR.D	43398 43399 43400 43401 43402 12942 43403 43404 43406 43407 43409 43410 43411 43412 43414 43415 43416 43417 4
44033	1693.8713	1692.8641	1692.8624	0.99	1	90	7.7e-08	1	U	K.KLDLEAWFPGSGAFR.E	44007 44055
44044	424.2234	1692.8647	1692.8624	1.36	1	(60)	4.8e-05	1	U	K.KLDLEAWFPGSGAFR.E	44019 44022 44023 44024 44029 44030 44031 44034 44037 44038 44039 44042 44043 44046 44047 44051 44052 44056 4
44097	565.2960	1692.8661	1692.8624	2.17	1	(38)	0.0073	1	U	K.KLDLEAWFPGSGAFR.E	
44133	847.4405	1692.8665	1692.8624	2.41	1	(82)	2.8e-07	1	U	K.KLDLEAWFPGSGAFR.E	44008 44009 44010 44011 44014 44016 44020 44026 44027 44028 44032 44036 44053 44057 44060 44063 44064 44066 4
44545	568.2442	1701.7107	1701.7087	1.18	0	(61)	6.8e-06	1	U	R.ELVSCSNCTDYQAR.R	44542 44543 44549 44554 44558 44561 44562 44563 44564 44569 44570 44575 44576 44577 44579 44580 44581 44586 44
44553	1702.7184	1701.7111	1701.7087	1.44	0	(99)	1.8e-09	1	U	R.ELVSCSNCTDYQAR.R	44525 44527 44536 44559 44567 44568 44585 44591 44593 44601 44605
44560	851.8629	1701.7113	1701.7087	1.53	0	(101)	7.1e-10	1	U	R.ELVSCSNCTDYQAR.R	25185 44514 44515 44516 44517 44518 44519 44520 44521 44522 44523 44526 44528 44529 44530 44531 44532 44533 44
48025	917.0361	1832.0576	1832.0560	0.88	0	101	4.7e-10	1	U	K.GVLVFLEQALIQYALR.T	
49119	940.9460	1879.8774	1879.8743	1.65	0	(88)	7.5e-08	1	U	K.VEFVHMLNATMCATTR.T	49022 49023 49024 49025 49026 49027 49028 49029 49030 49031 49032 49033 49034 49035 49036 49037 49038 49039
49391	627.6342	1879.8807	1879.8743	3.40	0	94	2.3e-08	1	U	K.VEFVHMLNATMCATTR.T	49021 16864 16865 49044 49045 49046 49047 49049 49051 49053 49057 49061 49066 49067 49070 49071 49074 49077
49844	948.9417	1895.8689	1895.8692	-0.16	0	(77)	8.7e-07	1	U	K.VEFVHMLNATMCATTR.T	49807 49808 49810 49814 49816 49818 49819 49821 49823 49824 49833 49836 49837 49840 49843 49845 49846 49848
49929	632.9645	1895.8718	1895.8692	1.36	0	(77)	8e-07	1	U	K.VEFVHMLNATMCATTR.T	49812 49828 49830 49831 49834 49839 49842 49850 49852 49855 49858 49860 49864 49870 49871 49873 17095 49877
49978	948.9435	1895.8725	1895.8692	1.71	0	(50)	0.0004	1	U	K.VEFVHMLNATMCATTR.T	49853 49905 50097
49982	632.9648	1895.8725	1895.8692	1.73	0	(66)	9.5e-06	1	U	K.VEFVHMLNATMCATTR.T	49946 50013 50019
50698	956.9406	1911.8666	1911.8641	1.30	0	(50)	0.00034	1	U	K.VEFVHMLNATMCATTR.T	50694 50695 50699
50725	638.2968	1911.8685	1911.8641	2.30	0	(59)	4.9e-05	1	U	K.VEFVHMLNATMCATTR.T	17441 50687 50688 50691 50693 50704 50709 50710 50711 50713 50714 50715 50717 50720 50721 50722 50728 50729
51112	641.9783	1922.9130	1922.9084	2.36	0	(93)	2.4e-08	1	U	K.YSHVDLVVMVDGFEGEK.G	50907 50910 50911 50915 50918 50919 50928 17582 50932 50935 50936 50938 50939 50941 50942 50945 50949 5095
51167	481.7357	1922.9135	1922.9084	2.66	0	(66)	1.2e-05	1	U	K.YSHVDLVVMVDGFEGEK.G	50933 50937 50940 50946 50953 50954 51069 51150
51329	962.4648	1922.9151	1922.9084	3.47	0	114	2.2e-10	1	U	K.YSHVDLVVMVDGFEGEK.G	50903 50905 50906 50908 50909 50912 50913 50914 50916 50917 50920 50921 50922 50924 50926 50929 50930 5093
51933	970.4609	1938.9072	1938.9034	1.97	0	(78)	6.8e-07	1	U	K.YSHVDLVVMVDGFEGEK.G	51734 28094 51736 51738 51740 51742 51743 51744 51746 51747 51748 51749 51754 51755 51756 51757 51758 5175
52304	647.3108	1938.9105	1938.9034	3.69	0	(81)	3.8e-07	1	U	K.YSHVDLVVMVDGFEGEK.G	51737 51739 51745 51750 51751 51752 51753 51760 51761 51775 51777 51780 51782 51784 51785 51792 51793 5179
54302	684.6751	2051.0036	2051.0034	0.09	1	(82)	3.1e-07	1	U	K.KYSHVDLVVMVDGFEGEK.G	54292 54297 54301 54314 54318 54322 54323 54324 54328 54333 54335 54338 54344 54346 54348 54351 54353 543
54382	513.7592	2051.0075	2051.0034	2.01	1	(56)	0.00011	1	U	K.KYSHVDLVVMVDGFEGEK.G	54294 54299 54300 54305 54308 54312 54319 54321 54326 54332 54336 54340 54347 54350 54352 54361 54362 543
54570	1026.5123	2051.0101	2051.0034	3.25	1	104	1.7e-09	1	U	K.KYSHVDLVVMVDGFEGEK.G	54280 54282 54285 54311 54342 54356 54368 54397 54417 54424 54427 54438 54457 54465 54487 54546 54554 545
54992	690.0078	2067.0017	2066.9983	1.64	1	(79)	5.2e-07	1	U	K.KYSHVDLVVMVDGFEGEK.G	54915 54921 54923 54926 54932 54933 54935 54947 54953 54956 54959 54964 54967 54969 54971 54976 54978 549
55247	1034.5107	2067.0068	2066.9983	4.13	1	(67)	1e-05	1	U	K.KYSHVDLVVMVDGFEGEK.G	54965 55030 55107 55265
55248	517.7590	2067.0069	2066.9983	4.15	1	(53)	0.00025	1	U	K.KYSHVDLVVMVDGFEGEK.G	54916 54930 54946 54950 54966 54970 54987 55000 55034 55060 55065 55080 55090 55092 55123 55126 55130 551
55591	1042.9794	2083.9443	2083.9395	2.29	0	63	1.3e-05	1	U	R.LQNMEVTDALEHHHHHH.-	
55592	521.9936	2083.9451	2083.9395	2.71	0	(36)	0.007	1	U	R.LQNMEVTDALEHHHHHH.-	
55596	695.6561	2083.9465	2083.9395	3.37	0	(57)	6.1e-05	1	U	R.LQNMEVTDALEHHHHHH.-	55594
55688	700.9863	2099.9370	2099.9344	1.22	0	(53)	0.00011	1	U	R.LQNMEVTDALEHHHHHH.-	
56190	1093.5207	2185.0269	2185.0249	0.93	0	114	1.8e-10	1	U	K.EVMQEVAQLSQFDEELYK.V	31545 56178 56179 56180 56182 56183 56184 56186 56187 56189 56191 56192 56193 56195 56196 56197 56201 562
56427	729.3521	2185.0343	2185.0249	4.33	0	(98)	7.5e-09	1	U	K.EVMQEVAQLSQFDEELYK.V	56181 56185 56188 56194 56198 56199 56200 56207 56209 56211 56217 56218 56220 56222 56224 56228 56229 562

56777	734.6827	2201.0262	2201.0198	2.92	0	(75)	1.1e-06	1	U	K.EVMQEVAQLSQFDEELYK.V	20908 20909 20910 56557 20911 20913 20914 20915 20917 20918 56584 56587 56598 56601 56606 56615 56617 566
56814	1101.5208	2201.0270	2201.0198	3.28	0	(90)	3.2e-08	1	U	K.EVMQEVAQLSQFDEELYK.V	56553 56554 56556 56558 56559 56561 56562 56564 56565 56566 56567 56568 56569 56570 56572 56573 56574 565
58039	769.0489	2304.1249	2304.1234	0.66	0	60	7.1e-05	1	U	R.EIGNLLHPSVPISNDEDVDNK.V	
58118	579.2882	2313.1238	2313.1198	1.72	1	(45)	0.0016	1	U	R.KEVMQEVAQLSQFDEELYK.V	
58132	772.0490	2313.1251	2313.1198	2.27	1	(81)	4e-07	1	U	R.KEVMQEVAQLSQFDEELYK.V	22199 58091 22201 22203 58098 58100 58102 58103 58104 58105 58110 58111 58113 58114 58115 58116 58119 58
58176	1157.5712	2313.1279	2313.1198	3.47	1	109	6.2e-10	1	U	R.KEVMQEVAQLSQFDEELYK.V	58101 58106 58109 58120 58129 58138 58143 58144 58146 58148 58152 58153 58154 58155 58156 58162 58168 58
58355	777.3813	2329.1221	2329.1147	3.17	1	(49)	0.00049	1	U	R.KEVMQEVAQLSQFDEELYK.V	58352 58358 58359 58363 58364 58365 58366
58360	1165.5687	2329.1228	2329.1147	3.47	1	(93)	1.8e-08	1	U	R.KEVMQEVAQLSQFDEELYK.V	58335 58337 58338 58339 58341 58342 58343 58344 58345 58346 58348 58349 58353 58357
58387	583.7903	2331.1321	2331.1284	1.60	1	(47)	0.00077	1	U	R.VHQFEKIEQFVYSSPHDNK.S	58386
58389	778.0517	2331.1332	2331.1284	2.05	1	54	0.00015	1	U	R.VHQFEKIEQFVYSSPHDNK.S	58388
58391	467.2344	2331.1355	2331.1284	3.06	1	(50)	0.00043	1	U	R.VHQFEKIEQFVYSSPHDNK.S	
58845	597.2745	2385.0688	2385.0772	-3.51	1	(62)	1.5e-05	1	U	K.MMDKVEFVHMLNATMCATTR.T	58858 58861 58863 58866 58870 58877 58883 58885 58886 58888 58890 58892 58894 58896 58897 58900 58907 5
58905	796.0347	2385.0824	2385.0772	2.18	1	(92)	1.9e-08	1	U	K.MMDKVEFVHMLNATMCATTR.T	58846 58849 58850 58851 58855 58859 58862 58865 58867 58871 58872 58878 58879 58881 58884 58891 58895 5
58909	1193.5485	2385.0825	2385.0772	2.25	1	(82)	1.9e-07	1	U	K.MMDKVEFVHMLNATMCATTR.T	
59312	601.2762	2401.0756	2401.0721	1.48	1	(59)	3e-05	1	U	K.MMDKVEFVHMLNATMCATTR.T	59198 59213 59227 59265 59327 59556 59585
59322	801.3660	2401.0761	2401.0721	1.65	1	(87)	4.5e-08	1	U	K.MMDKVEFVHMLNATMCATTR.T	59197 59203 23358 59204 23359 59206 59208 59209 59211 59214 59215 59216 59217 59221 59222 59229 59233 5
59367	801.3663	2401.0771	2401.0721	2.08	1	(76)	6.7e-07	1	U	K.MMDKVEFVHMLNATMCATTR.T	59199 59202 59218 59219 59228 59262 59276 59301 59309 59356 59431 59475 59489 59522 59528
59424	1201.5464	2401.0782	2401.0721	2.56	1	(63)	1.3e-05	1	U	K.MMDKVEFVHMLNATMCATTR.T	
59469	1201.5469	2401.0792	2401.0721	2.95	1	121	2e-11	1	U	K.MMDKVEFVHMLNATMCATTR.T	59238 59296 59374 59393 59411 59419 59433 59442 59455 59465 59472 59491 59519 59572
59495	801.3672	2401.0798	2401.0721	3.20	1	(69)	3.6e-06	1	U	K.MMDKVEFVHMLNATMCATTR.T	59196
59575	1201.5491	2401.0836	2401.0721	4.79	1	(92)	1.6e-08	1	U	K.MMDKVEFVHMLNATMCATTR.T	59277
59864	605.2742	2417.0678	2417.0670	0.34	1	(54)	7.6e-05	1	U	K.MMDKVEFVHMLNATMCATTR.T	59835 59858 59865 59908 59928 59961 60249
60100	1209.5437	2417.0728	2417.0670	2.40	1	(68)	3.8e-06	1	U	K.MMDKVEFVHMLNATMCATTR.T	60111 60166 60273
60103	1209.5437	2417.0729	2417.0670	2.44	1	(71)	1.7e-06	1	U	K.MMDKVEFVHMLNATMCATTR.T	60158 60202 60287
60105	806.6982	2417.0729	2417.0670	2.44	1	(68)	3.7e-06	1	U	K.MMDKVEFVHMLNATMCATTR.T	59833 59849 59855 23607 59879 59907 59915 59920 59927 59992 60039 60131 60153 60161 60186 60212 60223 2
60124	806.6984	2417.0732	2417.0670	2.58	1	(75)	6.8e-07	1	U	K.MMDKVEFVHMLNATMCATTR.T	59836 59846 59852 59853 59856 59857 59859 59862 59867 59868 59869 59875 59876 59878 59881 59888 59889 5
60130	1209.5439	2417.0733	2417.0670	2.62	1	(100)	2.3e-09	1	U	K.MMDKVEFVHMLNATMCATTR.T	59840 59850 59854 59861 59912 59951 59977 60034 60089 60296 60304 60316 60338
60170	806.6986	2417.0740	2417.0670	2.88	1	(47)	0.0004	1	U	K.MMDKVEFVHMLNATMCATTR.T	
60226	1209.5448	2417.0751	2417.0670	3.33	1	(52)	0.00013	1	U	K.MMDKVEFVHMLNATMCATTR.T	
60650	609.2708	2433.0541	2433.0619	-3.19	1	(38)	0.0018	1	U	K.MMDKVEFVHMLNATMCATTR.T	
23845	812.0263	2433.0571	2433.0619	-1.99	1	(39)	0.0021	1	U	K.MMDKVEFVHMLNATMCATTR.T	
60700	1217.5396	2433.0646	2433.0619	1.08	1	(48)	0.00025	1	U	K.MMDKVEFVHMLNATMCATTR.T	60728 60749 60982
60705	812.0289	2433.0648	2433.0619	1.20	1	(57)	3.8e-05	1	U	K.MMDKVEFVHMLNATMCATTR.T	60652 60658 60659 60664 60665 60668 60672 60680 60683 60686 60690 23848 60701 60704 60709 60717 60718 6
60773	1217.5408	2433.0670	2433.0619	2.08	1	(52)	0.00012	1	U	K.MMDKVEFVHMLNATMCATTR.T	
60810	1217.5413	2433.0681	2433.0619	2.55	1	(61)	1.7e-05	1	U	K.MMDKVEFVHMLNATMCATTR.T	60706
61492	1225.5343	2449.0541	2449.0568	-1.14	1	(46)	0.00041	1	U	K.MMDKVEFVHMLNATMCATTR.T	
62572	856.0999	2565.2778	2565.2686	3.61	1	43	0.0024	1	U	R.DEWLRPEDLPIKYAGLSTCFR.Q	62571
62658	1292.1599	2582.3053	2582.2938	4.46	1	71	2.5e-06	1	U	K.EVMQEVAQLSQFDEELYKVIGK.G	
62659	861.7758	2582.3055	2582.2938	4.53	1	(52)	0.0002	1	U	K.EVMQEVAQLSQFDEELYKVIGK.G	62660
62712	867.1071	2598.2996	2598.2887	4.18	1	(61)	3.3e-05	1	U	K.EVMQEVAQLSQFDEELYKVIGK.G	62711
63840	673.0931	2688.3432	2688.3355	2.86	1	(61)	2.6e-05	1	U	R.EIGNLLHPSVPISNDEDVDNKVER.I	63836 63838
63841	897.1217	2688.3433	2688.3355	2.90	1	105	9.8e-10	1	U	R.EIGNLLHPSVPISNDEDVDNKVER.I	63839 63843
64255	912.7763	2735.3070	2735.3038	1.16	1	59	4.3e-05	1	U	K.SDDNSYDEKYLIATSEQPIAALHR.D	26758
64257	548.0692	2735.3097	2735.3038	2.15	1	(40)	0.0037	1	U	K.SDDNSYDEKYLIATSEQPIAALHR.D	
64260	684.8355	2735.3127	2735.3038	3.26	1	(46)	0.00087	1	U	K.SDDNSYDEKYLIATSEQPIAALHR.D	64259
65725	991.8147	2972.4222	2972.4138	2.80	0	84	1.4e-07	1	U	K.EPVGDDESVPENVLSFDDLTADALANLK.V	
66650	1034.5149	3100.5229	3100.5088	4.55	1	66	9e-06	1	U	K.KEPVGDDESVPENVLSFDDLTADALANLK.V	
66652	1551.2727	3100.5309	3100.5088	7.12	1	(61)	3e-05	1	U	K.KEPVGDDESVPENVLSFDDLTADALANLK.V	

Proteins matching a subset of these peptides:

gi|16306548 **Mass:** 59253 **Score:** 2760 **Matches:** 6097(6097) **Sequences:** 39(39)
serine--tRNA ligase, cytoplasmic [Homo sapiens]

gi|62896913 **Mass:** 59166 **Score:** 2760 **Matches:** 6097(6097) **Sequences:** 39(39)
seryl-tRNA synthetase variant [Homo sapiens]

gi|111494145 **Mass:** 58883 **Score:** 2760 **Matches:** 6097(6097) **Sequences:** 39(39)
SARS protein [Homo sapiens]

gi|119576775 **Mass:** 59986 **Score:** 2760 **Matches:** 6097(6097) **Sequences:** 39(39)
seryl-tRNA synthetase, isoform CRA_g [Homo sapiens]

gi|550545083 **Mass:** 54920 **Score:** 2710 **Matches:** 6093(6093) **Sequences:** 38(38)
Chain A, Crystal Structure Of The Human Seryl-trna Synthetase In Complex With Ser-sa At 2.9 Angstrom Resolution

gi|1050527 **Mass:** 59226 **Score:** 2670 **Matches:** 6095(6095) **Sequences:** 37(37)
seryl-tRNA synthetase [Homo sapiens]
gi|119576772 **Mass:** 62172 **Score:** 2545 **Matches:** 4407(4407) **Sequences:** 37(37)
seryl-tRNA synthetase, isoform CRA_d [Homo sapiens]
gi|14424755 **Mass:** 59257 **Score:** 2489 **Matches:** 4405(4405) **Sequences:** 36(36)
Seryl-tRNA synthetase [Homo sapiens]
gi|119576771 **Mass:** 51371 **Score:** 2285 **Matches:** 5572(5572) **Sequences:** 30(30)
seryl-tRNA synthetase, isoform CRA_c [Homo sapiens]
gi|119576769 **Mass:** 53844 **Score:** 2234 **Matches:** 4203(4203) **Sequences:** 33(33)
seryl-tRNA synthetase, isoform CRA_a [Homo sapiens]
gi|119576774 **Mass:** 48467 **Score:** 1901 **Matches:** 3410(3410) **Sequences:** 29(29)
seryl-tRNA synthetase, isoform CRA_f [Homo sapiens]
gi|119576770 **Mass:** 23967 **Score:** 717 **Matches:** 531(531) **Sequences:** 13(13)
seryl-tRNA synthetase, isoform CRA_b [Homo sapiens]

---

2. gi|7331218 **Mass:** 66149 **Score:** 935 **Matches:** 29(29) **Sequences:** 13(13) **emPAI:** 1.27
keratin 1 [Homo sapiens]

Query	Observed	Mr(expt)	Mr(calc)	ppm	Miss	Score	Expect	Rank	Unique	Peptide
28191	487.2712	972.5278	972.5240	3.92	0	49	0.001	1		K.IEISELNR.V
32352	563.2782	1124.5418	1124.5349	6.08	0	52	0.00037	1	U	K.AEAESLYQSK.Y 32351
32390	564.2760	1126.5374	1126.5329	4.05	1	47	0.00094	1	U	K.KDVDGAYMTK.V
34142	633.3233	1264.6320	1264.6299	1.68	0	64	2.7e-05	1	U	R.TNAENEFVTIK.K
34570	651.8624	1301.7103	1301.7078	1.89	0	55	0.00019	1	U	R.SLDLDSIIAEVK.A 34571 18200
35188	666.7645	1331.5145	1331.5122	1.76	0	54	1.3e-05	1	U	K.NMQDMVEDYR.N 35187 35189
35360	670.8397	1339.6649	1339.6619	2.23	1	58	0.0001	1	U	K.SKAEAESLYQSK.Y 35357 35359
38254	738.3823	1474.7501	1474.7416	5.73	0	63	2.9e-05	1	U	K.WELLQQVDTSTR.T 38253
38257	738.3972	1474.7799	1474.7780	1.31	0	64	2.3e-05	1		R.FLEQQNQVLQTK.W 38256 38258
45230	858.9300	1715.8454	1715.8438	0.95	0	102	4.2e-09	1	U	K.QISNLQQSISDAEQR.G 45231
13472	572.9594	1715.8564	1715.8438	7.34	0	(54)	0.00023	1	U	K.QISNLQQSISDAEQR.G
46591	883.3727	1764.7309	1764.7275	1.95	0	111	6e-11	1	U	R.FSSCGGGGGSFGAGGGFGSR.S
19083	665.3322	1992.9746	1992.9693	2.65	0	53	0.00076	1	U	R.THNLEPYFESFINNLR.R 19080 19081 19084
58783	1192.4832	2382.9519	2382.9447	3.04	0	162	2.3e-16	1	U	R.GGGGGGYGSGGSSYGSGGGSYGSGGGGGGGR.G
58784	795.3246	2382.9521	2382.9447	3.12	0	(75)	1.3e-07	1	U	R.GGGGGGYGSGGSSYGSGGGSYGSGGGGGGGR.G

Proteins matching the same set of peptides:
gi|11935049 **Mass:** 66198 **Score:** 935 **Matches:** 29(29) **Sequences:** 13(13)
keratin 1 [Homo sapiens]
gi|119395750 **Mass:** 66170 **Score:** 935 **Matches:** 29(29) **Sequences:** 13(13)
keratin, type II cytoskeletal 1 [Homo sapiens]
gi|375314771 **Mass:** 66184 **Score:** 935 **Matches:** 29(29) **Sequences:** 13(13)
keratin 1 [Homo sapiens]
gi|375314775 **Mass:** 66101 **Score:** 935 **Matches:** 29(29) **Sequences:** 13(13)
keratin 1 [Homo sapiens]
gi|375314777 **Mass:** 66257 **Score:** 935 **Matches:** 29(29) **Sequences:** 13(13)
keratin 1 [Homo sapiens]
gi|375314781 **Mass:** 66242 **Score:** 935 **Matches:** 29(29) **Sequences:** 13(13)
keratin 1 [Homo sapiens]

Proteins matching a subset of these peptides:
gi|189054178 **Mass:** 66151 **Score:** 885 **Matches:** 28(28) **Sequences:** 12(12)
unnamed protein product [Homo sapiens]
gi|375314779 **Mass:** 66197 **Score:** 880 **Matches:** 26(26) **Sequences:** 12(12)
keratin 1 [Homo sapiens]
gi|375314773 **Mass:** 66200 **Score:** 823 **Matches:** 28(28) **Sequences:** 12(12)
keratin 1 [Homo sapiens]
gi|386854 **Mass:** 52928 **Score:** 490 **Matches:** 16(16) **Sequences:** 8(8)
type II keratin subunit protein, partial [Homo sapiens]
gi|31074631 **Mass:** 62049 **Score:** 64 **Matches:** 3(3) **Sequences:** 1(1)
keratin 1b [Homo sapiens]

gi|113414871 **Mass:** 62050 **Score:** 64 **Matches:** 3(3) **Sequences:** 1(1)
Keratin 77 [Homo sapiens]
gi|119617049 **Mass:** 62183 **Score:** 64 **Matches:** 3(3) **Sequences:** 1(1)
keratin 1B [Homo sapiens]
gi|194018511 **Mass:** 62149 **Score:** 64 **Matches:** 3(3) **Sequences:** 1(1)
keratin, type II cytoskeletal 1b [Homo sapiens]

---

3. gi|28317 **Mass:** 59720 **Score:** 905 **Matches:** 23(23) **Sequences:** 13(13) **emPAI:** 1.48
unnamed protein product [Homo sapiens]

Query	Observed	Mr(expt)	Mr(calc)	ppm	Miss	Score	Expect	Rank	Unique	Peptide
23685	404.2048	806.3951	806.3923	3.51	0	41	0.0059	1		R.LAADDFR.L
8236	497.2547	992.4949	992.4927	2.25	0	41	0.012	1	U	K.YENEVALR.Q
31487	545.7697	1089.5249	1089.5237	1.10	0	(51)	0.00054	1		K.VTMQNLNDR.L
31946	553.7676	1105.5206	1105.5186	1.81	0	57	0.0001	1		K.VTMQNLNDR.L 31943 31944 31945
33829	412.2318	1233.6737	1233.6717	1.62	1	53	0.00022	1	U	R.LKYENEVALR.Q 33830
34119	631.8050	1261.5955	1261.5899	4.49	0	70	5.6e-06	1	U	R.SLLEGEGSSGGGGR.G 34120
36273	683.3253	1364.6360	1364.6320	2.92	0	61	3.9e-05	1	U	R.SQYEQLAEQNR.K
36486	691.3293	1380.6440	1380.6408	2.29	0	67	1.7e-05	1	U	R.ALEESNYELEGK.I 36487
36628	695.8455	1389.6765	1389.6736	2.11	0	90	5.1e-08	1	U	K.QSLEASLAETEGR.Y 36629
38631	747.3744	1492.7343	1492.7270	4.88	1	52	0.00037	1	U	R.SQYEQLAEQNRK.D 38630
40436	775.3449	1548.6753	1548.6700	3.41	0	65	6.6e-06	1	U	R.SGGGGGGGGCGGGGGVSSLR.I
53853	998.9910	1995.9674	1995.9636	1.88	0	112	3.4e-10	1	U	K.ELTTEIDNNIEQISSYK.S
55541	1041.9915	2081.9685	2081.9575	5.26	0	107	8.9e-10	1	U	R.AETECQNTEYQQLLDIK.I
55810	704.6879	2111.0419	2111.0343	3.57	0	(82)	9.7e-07	1	U	K.ADLEMQIESLTEELAYLK.K
55811	1056.5288	2111.0431	2111.0343	4.14	0	89	1.7e-07	1	U	K.ADLEMQIESLTEELAYLK.K

Proteins matching the same set of peptides:
gi|21961605 **Mass:** 59020 **Score:** 905 **Matches:** 23(23) **Sequences:** 13(13)
Keratin 10 [Homo sapiens]
gi|119581085 **Mass:** 63536 **Score:** 905 **Matches:** 23(23) **Sequences:** 13(13)
keratin 10 (epidermolytic hyperkeratosis; keratosis palmaris et plantaris), isoform CRA_b [Homo sapiens]
gi|195972866 **Mass:** 58994 **Score:** 905 **Matches:** 23(23) **Sequences:** 13(13)
keratin, type I cytoskeletal 10 [Homo sapiens]
gi|269849769 **Mass:** 59020 **Score:** 905 **Matches:** 23(23) **Sequences:** 13(13)
RecName: Full=Keratin, type I cytoskeletal 10; AltName: Full=Cytokeratin-10; Short=CK-10; AltName: Full=Keratin-10; Short=K10 [Homo sapiens]
gi|530412176 **Mass:** 63536 **Score:** 905 **Matches:** 23(23) **Sequences:** 13(13)
PREDICTED: keratin, type I cytoskeletal 10 isoform X1 [Homo sapiens]

Proteins matching a subset of these peptides:
gi|186629 **Mass:** 39832 **Score:** 646 **Matches:** 13(13) **Sequences:** 9(9)
keratin 10, partial [Homo sapiens]
gi|307086 **Mass:** 46473 **Score:** 623 **Matches:** 13(13) **Sequences:** 9(9)
keratin-10 [Homo sapiens]
gi|623409 **Mass:** 57384 **Score:** 531 **Matches:** 16(16) **Sequences:** 8(8)
keratin 10 [Homo sapiens]
gi|31074643 **Mass:** 51191 **Score:** 98 **Matches:** 6(6) **Sequences:** 2(2)
type I inner root sheath specific keratin 25 irs4 [Homo sapiens]
gi|34526448 **Mass:** 53733 **Score:** 98 **Matches:** 6(6) **Sequences:** 2(2)
unnamed protein product [Homo sapiens]
gi|114431246 **Mass:** 51163 **Score:** 98 **Matches:** 6(6) **Sequences:** 2(2)
keratin, type I cytoskeletal 28 [Homo sapiens]
gi|1000377 **Mass:** 1992 **Score:** 57 **Matches:** 5(5) **Sequences:** 1(1)
type I keratin K16, partial [Homo sapiens]
gi|1332731 **Mass:** 1832 **Score:** 57 **Matches:** 5(5) **Sequences:** 1(1)
keratin 14, K14 {Y129D, rod domain} [human, Dowling-Meara epidermolysis bullosa simplex patient, blood, Peptide Partial Mutant, 16 aa]
gi|31074639 **Mass:** 52578 **Score:** 57 **Matches:** 5(5) **Sequences:** 1(1)
type I inner root sheath specific keratin 25 irs2 [Homo sapiens]
gi|31074641 **Mass:** 50420 **Score:** 57 **Matches:** 5(5) **Sequences:** 1(1)
type I inner root sheath specific keratin 25 irs3 [Homo sapiens]
gi|31559829 **Mass:** 49858 **Score:** 57 **Matches:** 5(5) **Sequences:** 1(1)

keratin, type I cytoskeletal 25 [Homo sapiens]
gi|34526053 **Mass:** 41924 **Score:** 57 **Matches:** 5(5) **Sequences:** 1(1)
unnamed protein product [Homo sapiens]
gi|119581082 **Mass:** 50421 **Score:** 57 **Matches:** 5(5) **Sequences:** 1(1)
keratin 25C [Homo sapiens]
gi|153945736 **Mass:** 50419 **Score:** 57 **Matches:** 5(5) **Sequences:** 1(1)
keratin, type I cytoskeletal 27 [Homo sapiens]
gi|254675185 **Mass:** 52620 **Score:** 57 **Matches:** 5(5) **Sequences:** 1(1)
keratin, type I cytoskeletal 26 [Homo sapiens]
gi|30311 **Mass:** 47305 **Score:** 41 **Matches:** 1(1) **Sequences:** 1(1)
cytokeratin 18 (424 AA) [Homo sapiens]
gi|1181994 **Mass:** 46883 **Score:** 41 **Matches:** 1(1) **Sequences:** 1(1)
hair type I acidic keratin [Homo sapiens]
gi|1668744 **Mass:** 48795 **Score:** 41 **Matches:** 1(1) **Sequences:** 1(1)
HHa5 hair keratin type I intermediate filament [Homo sapiens]
gi|2950168 **Mass:** 48628 **Score:** 41 **Matches:** 1(1) **Sequences:** 1(1)
type I keratin [Homo sapiens]
gi|2950208 **Mass:** 42453 **Score:** 41 **Matches:** 1(1) **Sequences:** 1(1)
hair keratin acidic 3-II [Homo sapiens]
gi|3724107 **Mass:** 48811 **Score:** 41 **Matches:** 1(1) **Sequences:** 1(1)
type I hair keratin 5 [Homo sapiens]
gi|3757666 **Mass:** 51769 **Score:** 41 **Matches:** 1(1) **Sequences:** 1(1)
HHa2 hair keratin type I intermediate filament [Homo sapiens]
gi|4504927 **Mass:** 53354 **Score:** 41 **Matches:** 1(1) **Sequences:** 1(1)
keratin, type I cuticular Ha6 [Homo sapiens]
gi|4557888 **Mass:** 48029 **Score:** 41 **Matches:** 1(1) **Sequences:** 1(1)
keratin, type I cytoskeletal 18 [Homo sapiens]
gi|7020236 **Mass:** 55625 **Score:** 41 **Matches:** 1(1) **Sequences:** 1(1)
unnamed protein product [Homo sapiens]
gi|7594732 **Mass:** 23331 **Score:** 41 **Matches:** 1(1) **Sequences:** 1(1)
keratin 19 [Homo sapiens]
gi|7594734 **Mass:** 11786 **Score:** 41 **Matches:** 1(1) **Sequences:** 1(1)
keratin 19 [Homo sapiens]
gi|10337581 **Mass:** 47325 **Score:** 41 **Matches:** 1(1) **Sequences:** 1(1)
keratin, type I cuticular Ha3-II [Homo sapiens]
gi|12311759 **Mass:** 52054 **Score:** 41 **Matches:** 1(1) **Sequences:** 1(1)
type I hair keratin 8 [Homo sapiens]
gi|12653819 **Mass:** 48003 **Score:** 41 **Matches:** 1(1) **Sequences:** 1(1)
Keratin 18 [Homo sapiens]
gi|14917115 **Mass:** 48633 **Score:** 41 **Matches:** 1(1) **Sequences:** 1(1)
keratin, type I cuticular Ha1 [Homo sapiens]
gi|15431318 **Mass:** 52044 **Score:** 41 **Matches:** 1(1) **Sequences:** 1(1)
keratin, type I cuticular Ha8 [Homo sapiens]
gi|50949256 **Mass:** 46938 **Score:** 41 **Matches:** 1(1) **Sequences:** 1(1)
hHa7 protein [Homo sapiens]
gi|50949258 **Mass:** 50188 **Score:** 41 **Matches:** 1(1) **Sequences:** 1(1)
hHa8 protein [Homo sapiens]
gi|55770882 **Mass:** 51084 **Score:** 41 **Matches:** 1(1) **Sequences:** 1(1)
keratin, type I cuticular Ha7 [Homo sapiens]
gi|62897747 **Mass:** 47957 **Score:** 41 **Matches:** 1(1) **Sequences:** 1(1)
keratin 18 variant [Homo sapiens]
gi|94538345 **Mass:** 51640 **Score:** 41 **Matches:** 1(1) **Sequences:** 1(1)
keratin, type I cuticular Ha5 [Homo sapiens]
gi|116488398 **Mass:** 51793 **Score:** 41 **Matches:** 1(1) **Sequences:** 1(1)
keratin, type I cuticular Ha2 [Homo sapiens]
gi|119581079 **Mass:** 55550 **Score:** 41 **Matches:** 1(1) **Sequences:** 1(1)
keratin 24 [Homo sapiens]
gi|119581136 **Mass:** 55308 **Score:** 41 **Matches:** 1(1) **Sequences:** 1(1)
keratin, hair, acidic, 7, isoform CRA_b [Homo sapiens]
gi|119581137 **Mass:** 54284 **Score:** 41 **Matches:** 1(1) **Sequences:** 1(1)

keratin, hair, acidic, 7, isoform CRA_c [Homo sapiens]
gi|119581139 **Mass:** 51800 **Score:** 41 **Matches:** 1(1) **Sequences:** 1(1)
keratin, hair, acidic, 2 [Homo sapiens]
gi|119581140 **Mass:** 51650 **Score:** 41 **Matches:** 1(1) **Sequences:** 1(1)
keratin, hair, acidic, 5, isoform CRA_a [Homo sapiens]
gi|119588030 **Mass:** 16549 **Score:** 41 **Matches:** 1(1) **Sequences:** 1(1)
hCG1640580, isoform CRA_a, partial [Homo sapiens]
gi|189054180 **Mass:** 48000 **Score:** 41 **Matches:** 1(1) **Sequences:** 1(1)
unnamed protein product [Homo sapiens]
gi|192807292 **Mass:** 55567 **Score:** 41 **Matches:** 1(1) **Sequences:** 1(1)
keratin, type I cytoskeletal 24 [Homo sapiens]
gi|194384386 **Mass:** 24004 **Score:** 41 **Matches:** 1(1) **Sequences:** 1(1)
unnamed protein product [Homo sapiens]
gi|194385560 **Mass:** 21684 **Score:** 41 **Matches:** 1(1) **Sequences:** 1(1)
unnamed protein product [Homo sapiens]
gi|211830808 **Mass:** 42148 **Score:** 41 **Matches:** 1(1) **Sequences:** 1(1)
KRT18 protein, partial [Homo sapiens]
gi|530413043 **Mass:** 48424 **Score:** 41 **Matches:** 1(1) **Sequences:** 1(1)
PREDICTED: keratin, type I cuticular Ha6 isoform X1 [Homo sapiens]
gi|578830527 **Mass:** 46458 **Score:** 41 **Matches:** 1(1) **Sequences:** 1(1)
PREDICTED: keratin, type I cytoskeletal 24 isoform X1 [Homo sapiens]

---

4. gi|1195531 **Mass:** 51548 **Score:** 756 **Matches:** 28(28) **Sequences:** 13(13) **emPAI:** 1.66
type I keratin 16 [Homo sapiens]

Query	Observed	Mr(expt)	Mr(calc)	ppm	Miss	Score	Expect	Rank	Unique	Peptide
23685	404.2048	806.3951	806.3923	3.51	0	41	0.0059	1		R.LAADDFR.T
31487	545.7697	1089.5249	1089.5237	1.10	0	(51)	0.00054	1		K.VTMQNLNDR.L
31946	553.7676	1105.5206	1105.5186	1.81	0	57	0.0001	1		K.VTMQNLNDR.L 31943 31944 31945
31952	553.7857	1105.5568	1105.5550	1.60	0	60	6.3e-05	1		**R.ISSVLAGGSCR.A** 31950 31951
12989	561.7945	1121.5744	1121.5717	2.48	0	49	0.00094	1		**R.LEQEIATYR.R** 12988
15057	601.3138	1200.6131	1200.6099	2.72	0	44	0.0059	1	U	**R.QTVEADVNGLR.R**
33623	610.8025	1219.5905	1219.5932	-2.17	0	45	0.0021	1		**K.ASLENSLEETK.G** 33625 33627 33628
34107	630.7911	1259.5676	1259.5630	3.68	0	53	0.00016	1	U	**R.EVFTSSSSSSSR.Q** 34106
34294	639.7984	1277.5822	1277.5783	3.10	0	69	5.5e-06	1		**K.GSCGIGGGIGGGSSR.I** 34295 34296
35339	669.8370	1337.6595	1337.6575	1.46	0	73	2.8e-06	1	U	**R.APSTYGGGLSVSSR.F** 35340 19440
36450	690.3689	1378.7233	1378.7204	2.10	1	38	0.0077	1		**K.TRLEQEIATYR.R**
36850	703.3529	1404.6913	1404.6845	4.84	0	76	1.5e-06	1	U	**K.EVASNSELVQSSR.S**
56107	723.0371	2166.0895	2166.0878	0.80	1	70	4.3e-06	1	U	**R.TDLEMQIEGLKEELAYLR.K**
58496	784.0361	2349.0866	2349.0833	1.41	0	80	3.6e-07	1	U	**R.LLEGEDAHLSSQQASGQSYSSR.E**

Proteins matching the same set of peptides:
gi|24430192 **Mass:** 51578 **Score:** 756 **Matches:** 28(28) **Sequences:** 13(13)
keratin, type I cytoskeletal 16 [Homo sapiens]

Proteins matching a subset of these peptides:
gi|186685 **Mass:** 51010 **Score:** 500 **Matches:** 17(17) **Sequences:** 9(9)
keratin type 16 [Homo sapiens]
gi|7717238 **Mass:** 27757 **Score:** 324 **Matches:** 9(9) **Sequences:** 5(5)
keratin [Homo sapiens]
gi|34071 **Mass:** 49365 **Score:** 128 **Matches:** 4(4) **Sequences:** 3(3)
unnamed protein product [Homo sapiens]
gi|24430190 **Mass:** 49395 **Score:** 128 **Matches:** 4(4) **Sequences:** 3(3)
keratin, type I cytoskeletal 15 [Homo sapiens]
gi|60814531 **Mass:** 49423 **Score:** 128 **Matches:** 4(4) **Sequences:** 3(3)
keratin 15 [synthetic construct]
gi|131412225 **Mass:** 49870 **Score:** 128 **Matches:** 4(4) **Sequences:** 3(3)
keratin, type I cytoskeletal 13 isoform a [Homo sapiens]
gi|131412228 **Mass:** 46151 **Score:** 128 **Matches:** 4(4) **Sequences:** 3(3)
keratin, type I cytoskeletal 13 isoform b [Homo sapiens]

gi|193783785 **Mass:** 49307 **Score:** 128 **Matches:** 4(4) **Sequences:** 3(3)
unnamed protein product [Homo sapiens]
gi|269849755 **Mass:** 49900 **Score:** 128 **Matches:** 4(4) **Sequences:** 3(3)
RecName: Full=Keratin, type I cytoskeletal 13; AltName: Full=Cytokeratin-13; Short=CK-13; AltName: Full=Keratin-13; Short=K13 [Homo sapiens]
gi|530412180 **Mass:** 49409 **Score:** 128 **Matches:** 4(4) **Sequences:** 3(3)
PREDICTED: keratin, type I cytoskeletal 15 isoform X1 [Homo sapiens]
gi|194377182 **Mass:** 15771 **Score:** 110 **Matches:** 2(2) **Sequences:** 2(2)
unnamed protein product [Homo sapiens]
gi|34039 **Mass:** 44079 **Score:** 90 **Matches:** 3(3) **Sequences:** 2(2)
unnamed protein product [Homo sapiens]
gi|386803 **Mass:** 44065 **Score:** 90 **Matches:** 3(3) **Sequences:** 2(2)
40-kDa keratin protein, partial [Homo sapiens]
gi|24234699 **Mass:** 44079 **Score:** 90 **Matches:** 3(3) **Sequences:** 2(2)
keratin, type I cytoskeletal 19 [Homo sapiens]
gi|194373749 **Mass:** 62862 **Score:** 90 **Matches:** 3(3) **Sequences:** 2(2)
unnamed protein product [Homo sapiens]
gi|30377 **Mass:** 46179 **Score:** 88 **Matches:** 3(3) **Sequences:** 2(2)
cytokeratin 13 [Homo sapiens]
gi|34033 **Mass:** 49956 **Score:** 88 **Matches:** 3(3) **Sequences:** 2(2)
keratin 13 [Homo sapiens]
gi|406854 **Mass:** 39327 **Score:** 88 **Matches:** 3(3) **Sequences:** 2(2)
cytokeratin 20 [Homo sapiens]
gi|27894337 **Mass:** 48514 **Score:** 88 **Matches:** 3(3) **Sequences:** 2(2)
keratin, type I cytoskeletal 20 [Homo sapiens]
gi|62897663 **Mass:** 49997 **Score:** 88 **Matches:** 3(3) **Sequences:** 2(2)
keratin 13 isoform a variant [Homo sapiens]
gi|118835468 **Mass:** 49898 **Score:** 88 **Matches:** 3(3) **Sequences:** 2(2)
Keratin 13 [Homo sapiens]
gi|158261511 **Mass:** 49828 **Score:** 88 **Matches:** 3(3) **Sequences:** 2(2)
unnamed protein product [Homo sapiens]
gi|189053448 **Mass:** 48541 **Score:** 88 **Matches:** 3(3) **Sequences:** 2(2)
unnamed protein product [Homo sapiens]
gi|193787108 **Mass:** 28698 **Score:** 88 **Matches:** 3(3) **Sequences:** 2(2)
unnamed protein product [Homo sapiens]
gi|194384438 **Mass:** 35892 **Score:** 88 **Matches:** 3(3) **Sequences:** 2(2)
unnamed protein product [Homo sapiens]
gi|22748757 **Mass:** 34308 **Score:** 49 **Matches:** 2(2) **Sequences:** 1(1)
keratin-like protein KRT222 [Homo sapiens]
gi|31874218 **Mass:** 29567 **Score:** 49 **Matches:** 2(2) **Sequences:** 1(1)
hypothetical protein [Homo sapiens]

---

5. gi|435476 **Mass:** 62320 **Score:** 687 **Matches:** 17(17) **Sequences:** 10(10) **emPAI:** 1.01
cytokeratin 9 [Homo sapiens]

Query	Observed	Mr(expt)	Mr(calc)	ppm	Miss	Score	Expect	Rank	Unique	Peptide
4960	457.2082	912.4019	912.4011	0.84	0	52	0.00043	1	U	R.MTLDDFR.I 4961
30568	533.2556	1064.4966	1064.4920	4.33	0	38	0.0086	1	U	K.STMQELNSR.L
32291	561.2965	1120.5785	1120.5764	1.89	0	44	0.0025	1	U	R.QEYEQLIAK.N
32823	579.3013	1156.5881	1156.5836	3.89	0	68	1e-05	1	U	R.QGVDADINGLR.Q 32822
33766	616.8036	1231.5926	1231.5906	1.65	0	86	1.6e-07	1	U	R.SGGGGGGGLGSGGSIR.S 33765 33767
41828	529.5956	1585.7650	1585.7583	4.20	0	(50)	0.00049	1	U	K.VQALEEANNDLENK.I
41829	793.8903	1585.7660	1585.7583	4.82	0	83	2.7e-07	1	U	K.VQALEEANNDLENK.I
48195	919.4888	1836.9631	1836.9581	2.69	0	125	1.3e-11	1	U	R.HGVQELEIELQSQLSK.K
48196	613.3292	1836.9658	1836.9581	4.17	0	(54)	0.00016	1	U	R.HGVQELEIELQSQLSK.K
62246	837.3842	2509.1308	2509.1245	2.53	0	91	1.8e-08	1	U	K.EIETYHNLLEGGQEDFESSGAGK.I 62247
64952	968.1444	2901.4115	2901.4032	2.85	1	56	0.00011	1	U	K.NYSPYYNTIDDLKDQIVDLTVGNNK.T
67144	1088.8452	3263.5137	3263.5066	2.19	0	44	0.0011	1	U	K.DIENQYETQITQIEHEVSSSGQEVQSSAK.E

Proteins matching the same set of peptides:
gi|55956899 **Mass:** 62255 **Score:** 687 **Matches:** 17(17) **Sequences:** 10(10)

keratin, type I cytoskeletal 9 [Homo sapiens]
gi|119581148 **Mass:** 57754 **Score:** 687 **Matches:** 17(17) **Sequences:** 10(10)
keratin 9 (epidermolytic palmoplantar keratoderma) [Homo sapiens]

Proteins matching a subset of these peptides:
gi|453155 **Mass:** 62178 **Score:** 601 **Matches:** 14(14) **Sequences:** 9(9)
keratin 9 [Homo sapiens]
gi|113197968 **Mass:** 48171 **Score:** 481 **Matches:** 11(11) **Sequences:** 7(7)
KRT9 protein, partial [Homo sapiens]
gi|1890020 **Mass:** 25913 **Score:** 347 **Matches:** 9(9) **Sequences:** 6(6)
mutant keratin 9 [Homo sapiens]

---

6. gi|12803709 **Mass:** 51905 **Score:** 647 **Matches:** 29(29) **Sequences:** 11(11) **emPAI:** 1.30
Keratin 14 [Homo sapiens]

Query	Observed	Mr(expt)	Mr(calc)	ppm	Miss	Score	Expect	Rank	Unique	Peptide
23685	404.2048	806.3951	806.3923	3.51	0	41	0.0059	1		R.LAADDFR.T
31487	545.7697	1089.5249	1089.5237	1.10	0	(51)	0.00054	1		K.VTMQNLNDR.L
31946	553.7676	1105.5206	1105.5186	1.81	0	57	0.0001	1		K.VTMQNLNDR.L 31943 31944 31945
31952	553.7857	1105.5568	1105.5550	1.60	0	60	6.3e-05	1		R.ISSVLAGGSCR.A 31950 31951
12989	561.7945	1121.5744	1121.5717	2.48	0	49	0.00094	1		R.LEQEIATYR.R 12988
33623	610.8025	1219.5905	1219.5932	-2.17	0	45	0.0021	1		K.ASLENSLEETK.G 33625 33627 33628
34294	639.7984	1277.5822	1277.5783	3.10	0	69	5.5e-06	1		K.GSCGIGGGIGGGSSR.I 34295 34296
36190	**681.3510**	**1360.6875**	**1360.6834**	**3.02**	**0**	**67**	**1.5e-05**	**1**		**R.EVATNSELVQSGK.S** 36189
36450	690.3689	1378.7233	1378.7204	2.10	1	38	0.0077	1		K.TRLEQEIATYR.R
37156	**713.3528**	**1424.6910**	**1424.6896**	**1.02**	**0**	**74**	**2.9e-06**	**1**	**U**	**R.APSTYGGGLSVSSSR.F** 37153 37154 37155
38796	**751.7987**	**1501.5829**	**1501.5813**	**1.06**	**0**	**61**	**6.9e-06**	**1**		**R.CEMEQQNQEYK.I** 38795
58056	**770.3614**	**2308.0625**	**2308.0567**	**2.48**	**0**	**87**	**7.3e-08**	**1**	**U**	**R.LLEGEDAHLSSSQFSSGSQSSR.D** 58055

Proteins matching the same set of peptides:
gi|15431310 **Mass:** 51875 **Score:** 647 **Matches:** 29(29) **Sequences:** 11(11)
keratin, type I cytoskeletal 14 [Homo sapiens]
gi|229463044 **Mass:** 51872 **Score:** 647 **Matches:** 29(29) **Sequences:** 11(11)
RecName: Full=Keratin, type I cytoskeletal 14; AltName: Full=Cytokeratin-14; Short=CK-14; AltName: Full=Keratin-14; Short=K14 [Homo sapiens]

Proteins matching a subset of these peptides:
gi|386848 **Mass:** 51916 **Score:** 504 **Matches:** 22(22) **Sequences:** 9(9)
keratin [Homo sapiens]
gi|119581150 **Mass:** 44865 **Score:** 444 **Matches:** 19(19) **Sequences:** 8(8)
keratin 14 (epidermolysis bullosa simplex, Dowling-Meara, Koebner), isoform CRA_b [Homo sapiens]
gi|119581149 **Mass:** 30075 **Score:** 346 **Matches:** 13(13) **Sequences:** 6(6)
keratin 14 (epidermolysis bullosa simplex, Dowling-Meara, Koebner), isoform CRA_a [Homo sapiens]
gi|177139 **Mass:** 17892 **Score:** 280 **Matches:** 11(11) **Sequences:** 5(5)
epidermal type I keratin [Homo sapiens]
gi|392311615 **Mass:** 15414 **Score:** 260 **Matches:** 11(11) **Sequences:** 5(5)
Chain A, Heterocomplex Of Coil 2b Domains Of Human Intermediate Filament Proteins, Keratin 5 (Krt5) And Keratin 14 (Krt14)
gi|6118241 **Mass:** 8673 **Score:** 193 **Matches:** 9(9) **Sequences:** 4(4)
mutant cytokeratin 14 [Homo sapiens]
gi|6118240 **Mass:** 8703 **Score:** 106 **Matches:** 6(6) **Sequences:** 2(2)
mutant cytokeratin 14 [Homo sapiens]

---

7. gi|386849 **Mass:** 60258 **Score:** 567 **Matches:** 16(16) **Sequences:** 9(9) **emPAI:** 0.82
keratin type II, partial [Homo sapiens]

Query	Observed	Mr(expt)	Mr(calc)	ppm	Miss	Score	Expect	Rank	Unique	Peptide
28163	**486.7612**	**971.5078**	**971.5036**	**4.37**	**0**	**49**	**0.00076**	**1**		**K.QEIAEINR.M** 28162
29089	**508.7731**	**1015.5316**	**1015.5298**	**1.80**	**0**	**51**	**0.00082**	**1**		**R.QLDSIVGER.G** 29090 29091 29092
29391	**513.7649**	**1025.5153**	**1025.5142**	**1.12**	**0**	**40**	**0.0079**	**1**		**R.SGFSSISVSR.S**
32485	**567.2890**	**1132.5634**	**1132.5546**	**7.70**	**1**	**56**	**0.00019**	**1**		**R.KLLEGEECR.L**
32892	**583.2969**	**1164.5792**	**1164.5775**	**1.44**	**0**	**51**	**0.0005**	**1**		**K.YEELQVTAGR.H** 32891
35599	**679.3718**	**1356.7290**	**1356.7249**	**3.05**	**1**	**53**	**0.00027**	**1**		**K.NKLEGLEDALQK.A**
35600	**453.2508**	**1356.7306**	**1356.7249**	**4.24**	**1**	**(41)**	**0.0054**	**1**		**K.NKLEGLEDALQK.A**

37320	718.3751	1434.7357	1434.7315	2.97	0	85	1.9e-07	1	U	R.ATGGGLSSVGGGSSTIK.Y
42089	799.8880	1597.7615	1597.7519	6.02	0	112	3.1e-10	1		R.ISIGGGSCAISGGYGSR.A
49756	945.9924	1889.9703	1889.9635	3.61	0	70	5.9e-06	1		R.QNLEPLFEQYINNLR.R 49755

Proteins matching the same set of peptides:
gi|908790 **Mass:** 60247 **Score:** 567 **Matches:** 16(16) **Sequences:** 9(9)
keratin type II [Homo sapiens]
gi|194383916 **Mass:** 55983 **Score:** 567 **Matches:** 16(16) **Sequences:** 9(9)
unnamed protein product [Homo sapiens]

Proteins matching a subset of these peptides:
gi|119617032 **Mass:** 60159 **Score:** 465 **Matches:** 10(10) **Sequences:** 7(7)
keratin 6B, isoform CRA_a [Homo sapiens]
gi|119617033 **Mass:** 60288 **Score:** 465 **Matches:** 10(10) **Sequences:** 7(7)
keratin 6B, isoform CRA_b [Homo sapiens]
gi|119703753 **Mass:** 60315 **Score:** 465 **Matches:** 10(10) **Sequences:** 7(7)
keratin, type II cytoskeletal 6B [Homo sapiens]
gi|34733245 **Mass:** 8516 **Score:** 121 **Matches:** 6(6) **Sequences:** 2(2)
keratin 5 [Homo sapiens]
gi|119617038 **Mass:** 38781 **Score:** 121 **Matches:** 6(6) **Sequences:** 2(2)
keratin 5 (epidermolysis bullosa simplex, Dowling-Meara/Kobner/Weber-Cockayne types), isoform CRA_a [Homo sapiens]
gi|3901030 **Mass:** 59753 **Score:** 106 **Matches:** 3(3) **Sequences:** 2(2)
cytokeratin type II [Homo sapiens]
gi|7161776 **Mass:** 59779 **Score:** 106 **Matches:** 3(3) **Sequences:** 2(2)
cytokeratin [Homo sapiens]
gi|153791158 **Mass:** 59809 **Score:** 106 **Matches:** 3(3) **Sequences:** 2(2)
keratin, type II cytoskeletal 75 [Homo sapiens]
gi|194386492 **Mass:** 65720 **Score:** 106 **Matches:** 3(3) **Sequences:** 2(2)
unnamed protein product [Homo sapiens]
gi|181390 **Mass:** 66400 **Score:** 56 **Matches:** 1(1) **Sequences:** 1(1)
cytokeratin 2 [Homo sapiens]
gi|1841430 **Mass:** 111941 **Score:** 56 **Matches:** 1(1) **Sequences:** 1(1)
heavy neurofilament subunit [Homo sapiens]
gi|28173564 **Mass:** 59457 **Score:** 56 **Matches:** 1(1) **Sequences:** 1(1)
keratin, type II cytoskeletal 73 [Homo sapiens]
gi|32483416 **Mass:** 111999 **Score:** 56 **Matches:** 1(1) **Sequences:** 1(1)
neurofilament heavy polypeptide [Homo sapiens]
gi|119580210 **Mass:** 64630 **Score:** 56 **Matches:** 1(1) **Sequences:** 1(1)
neurofilament, heavy polypeptide 200kDa, isoform CRA_c [Homo sapiens]
gi|119617050 **Mass:** 66430 **Score:** 56 **Matches:** 1(1) **Sequences:** 1(1)
cytokeratin 2 [Homo sapiens]
gi|153791670 **Mass:** 66370 **Score:** 56 **Matches:** 1(1) **Sequences:** 1(1)
keratin, type II cytoskeletal 2 oral [Homo sapiens]
gi|194380258 **Mass:** 112508 **Score:** 56 **Matches:** 1(1) **Sequences:** 1(1)
unnamed protein product [Homo sapiens]
gi|194387924 **Mass:** 35621 **Score:** 56 **Matches:** 1(1) **Sequences:** 1(1)
unnamed protein product [Homo sapiens]
gi|226726294 **Mass:** 112639 **Score:** 56 **Matches:** 1(1) **Sequences:** 1(1)
RecName: Full=Neurofilament heavy polypeptide; Short=NF-H; AltName: Full=200 kDa neurofilament protein; AltName: Full=Neurofilament triplet H protein [Homo sapiens]
gi|27764565 **Mass:** 59847 **Score:** 55 **Matches:** 1(1) **Sequences:** 1(1)
TPA_exp: keratin 5c [Homo sapiens]
gi|27901524 **Mass:** 58215 **Score:** 55 **Matches:** 1(1) **Sequences:** 1(1)
keratin 6 irs4 [Homo sapiens]
gi|148612803 **Mass:** 58229 **Score:** 55 **Matches:** 1(1) **Sequences:** 1(1)
keratin, type II cytoskeletal 74 [Homo sapiens]

---

8. gi|5031839 **Mass:** 60293 **Score:** 565 **Matches:** 16(16) **Sequences:** 9(9) **emPAI:** 0.82
keratin, type II cytoskeletal 6A [Homo sapiens]

Query	Observed	Mr(expt)	Mr(calc)	ppm	Miss	Score	Expect	Rank	Unique	Peptide

Query	Observed	Mr(expt)	Mr(calc)	ppm	Miss	Score	Expect	Rank	Unique	Peptide
28163	486.7612	971.5078	971.5036	4.37	0	49	0.00076	1		K.QEIAEINR.M 28162
28995	**506.7586**	**1011.5026**	**1011.4985**	**3.98**	**0**	**63**	**2.1e-05**	**1**	**U**	**R.SGFSSVSVSR.S**
29089	508.7731	1015.5316	1015.5298	1.80	0	51	0.00082	1		R.QLDSIVGER.G 29090 29091 29092
32485	567.2890	1132.5634	1132.5546	7.70	1	56	0.00019	1		R.KLLEGEECR.L
32892	583.2969	1164.5792	1164.5775	1.44	0	51	0.0005	1		K.YEELQVTAGR.H 32891
35599	679.3718	1356.7290	1356.7249	3.05	1	53	0.00027	1		K.NKLEGLEDALQK.A
35600	453.2508	1356.7306	1356.7249	4.24	1	(41)	0.0054	1		K.NKLEGLEDALQK.A
37675	**724.3929**	**1446.7712**	**1446.7678**	**2.30**	**0**	**59**	**6.1e-05**	**1**		**R.AIGGGLSSVGGGSSTIK.Y**
42089	799.8880	1597.7615	1597.7519	6.02	0	112	3.1e-10	1		R.ISIGGGSCAISGGYGSR.A
49756	945.9924	1889.9703	1889.9635	3.61	0	70	5.9e-06	1		R.QNLEPLFEQYINNLR.R 49755

Proteins matching the same set of peptides:

gi|46812692 **Mass:** 60323 **Score:** 565 **Matches:** 16(16) **Sequences:** 9(9)
Keratin 6A [Homo sapiens]

gi|114644568 **Mass:** 60266 **Score:** 565 **Matches:** 16(16) **Sequences:** 9(9)
PREDICTED: keratin, type II cytoskeletal 6A [Pan troglodytes]

gi|119617036 **Mass:** 60164 **Score:** 565 **Matches:** 16(16) **Sequences:** 9(9)
hCG2039812, isoform CRA_a [Homo sapiens]

gi|158261513 **Mass:** 60279 **Score:** 565 **Matches:** 16(16) **Sequences:** 9(9)
unnamed protein product [Homo sapiens]

gi|194387918 **Mass:** 58088 **Score:** 565 **Matches:** 16(16) **Sequences:** 9(9)
unnamed protein product [Homo sapiens]

gi|194387990 **Mass:** 58786 **Score:** 565 **Matches:** 16(16) **Sequences:** 9(9)
unnamed protein product [Homo sapiens]

Proteins matching a subset of these peptides:

gi|194377194 **Mass:** 55993 **Score:** 446 **Matches:** 14(14) **Sequences:** 7(7)
unnamed protein product [Homo sapiens]

gi|34069 **Mass:** 39309 **Score:** 390 **Matches:** 14(14) **Sequences:** 7(7)
unnamed protein product [Homo sapiens]

gi|914833 **Mass:** 42557 **Score:** 390 **Matches:** 14(14) **Sequences:** 7(7)
keratin type II [Homo sapiens]

---

9. gi|181402 **Mass:** 66110 **Score:** 498 **Matches:** 14(14) **Sequences:** 9(9) **emPAI:** 0.73
epidermal cytokeratin 2 [Homo sapiens]

Query	Observed	Mr(expt)	Mr(calc)	ppm	Miss	Score	Expect	Rank	Unique	Peptide
28191	487.2712	972.5278	972.5240	3.92	0	49	0.001	1		K.IEISELNR.V
32464	**566.2581**	**1130.5017**	**1130.5026**	**-0.80**	**0**	**43**	**0.00096**	**1**	**U**	**R.STSSFSCLSR.H**
32485	567.2890	1132.5634	1132.5546	7.70	1	56	0.00019	1		R.KLLEGEECR.M
33256	**597.3137**	**1192.6128**	**1192.6088**	**3.33**	**0**	**60**	**7.2e-05**	**1**	**U**	**K.YEELQVTVGR.H** 33255 33257
34033	**627.8094**	**1253.6043**	**1253.6001**	**3.36**	**0**	**45**	**0.0017**	**1**	**U**	**R.GFSSGSAVVSGGSR.R**
35031	**665.3248**	**1328.6350**	**1328.6320**	**2.27**	**0**	**51**	**0.0006**	**1**	**U**	**K.NVQDAIADAEQR.G**
36665	**696.3442**	**1390.6738**	**1390.6728**	**0.68**	**1**	**61**	**5.7e-05**	**1**	**U**	**R.SKEEAEALYHSK.Y**
36666	**464.5657**	**1390.6753**	**1390.6728**	**1.76**	**1**	**(40)**	**0.008**	**1**	**U**	**R.SKEEAEALYHSK.Y**
38257	738.3972	1474.7799	1474.7780	1.31	0	64	2.3e-05	1		R.FLEQQNQVLQTK.W 38256 38258
57728	**752.6886**	**2255.0439**	**2255.0376**	**2.83**	**1**	**70**	**3.6e-06**	**1**	**U**	**R.TSQNSELNNMQDLVEDYKK.K**

Proteins matching the same set of peptides:

gi|47132620 **Mass:** 65678 **Score:** 498 **Matches:** 14(14) **Sequences:** 9(9)
keratin, type II cytoskeletal 2 epidermal [Homo sapiens]

---

10. gi|4557701 **Mass:** 48361 **Score:** 406 **Matches:** 10(10) **Sequences:** 7(7) **emPAI:** 0.68
keratin, type I cytoskeletal 17 [Homo sapiens]

Query	Observed	Mr(expt)	Mr(calc)	ppm	Miss	Score	Expect	Rank	Unique	Peptide
23685	404.2048	806.3951	806.3923	3.51	0	41	0.0059	1		R.LAADDFR.T
12989	561.7945	1121.5744	1121.5717	2.48	0	49	0.00094	1		R.LEQEIATYR.R 12988
35436	**673.3484**	**1344.6823**	**1344.6772**	**3.77**	**0**	**49**	**0.00083**	**1**	**U**	**R.ALEEANTELEVK.I**
36190	681.3510	1360.6875	1360.6834	3.02	0	67	1.5e-05	1		R.EVATNSELVQSGK.S 36189
36450	690.3689	1378.7233	1378.7204	2.10	1	38	0.0077	1		K.TRLEQEIATYR.R

**36840**	**702.3445**	**1402.6745**	**1402.6688**	**4.04**	**0**	**102**	**3.7e-09**	**1**	**U**	**K.ASLEGNLAETENR.Y**
38796	751.7987	1501.5829	1501.5813	1.06	0	61	6.9e-06	1		R.CEMEQQNQEYK.I 38795

Proteins matching the same set of peptides:
gi|21754583 **Mass:** 47859 **Score:** 406 **Matches:** 10(10) **Sequences:** 7(7)
unnamed protein product [Homo sapiens]
gi|119581154 **Mass:** 48232 **Score:** 406 **Matches:** 10(10) **Sequences:** 7(7)
keratin 17, isoform CRA_a [Homo sapiens]
gi|119581155 **Mass:** 48337 **Score:** 406 **Matches:** 10(10) **Sequences:** 7(7)
keratin 17, isoform CRA_b [Homo sapiens]

Proteins matching a subset of these peptides:
gi|47939651 **Mass:** 40520 **Score:** 357 **Matches:** 9(9) **Sequences:** 6(6)
KRT17 protein [Homo sapiens]
gi|34081 **Mass:** 30933 **Score:** 317 **Matches:** 8(8) **Sequences:** 5(5)
unnamed protein product [Homo sapiens]
gi|119581156 **Mass:** 26021 **Score:** 89 **Matches:** 2(2) **Sequences:** 2(2)
keratin 17, isoform CRA_c [Homo sapiens]
gi|194385672 **Mass:** 18209 **Score:** 89 **Matches:** 2(2) **Sequences:** 2(2)
unnamed protein product [Homo sapiens]
gi|119571624 **Mass:** 19610 **Score:** 49 **Matches:** 1(1) **Sequences:** 1(1)
hCG27965, isoform CRA_a [Homo sapiens]
gi|119571625 **Mass:** 19481 **Score:** 49 **Matches:** 1(1) **Sequences:** 1(1)
hCG27965, isoform CRA_b [Homo sapiens]
gi|148615514 **Mass:** 1997 **Score:** 49 **Matches:** 1(1) **Sequences:** 1(1)
keratin 17 epitope S1 [Homo sapiens]

---

11. gi|386850 **Mass:** 54649 **Score:** 257 **Matches:** 9(9) **Sequences:** 5(5) **emPAI:** 0.39
keratin K5, partial [Homo sapiens]

Query	Observed	Mr(expt)	Mr(calc)	ppm	Miss	Score	Expect	Rank	Unique	Peptide
29089	508.7731	1015.5316	1015.5298	1.80	0	51	0.00082	1		R.QLDSIVGER.G 29090 29091 29092
**31550**	**547.2696**	**1092.5247**	**1092.5199**	**4.39**	**0**	**41**	**0.0052**	**1**	**U**	**K.AQYEEIANR.S**
32485	567.2890	1132.5634	1132.5546	7.70	1	56	0.00019	1		R.KLLEGEECR.L
**33264**	**597.7960**	**1193.5774**	**1193.5676**	**8.19**	**0**	**40**	**0.0056**	**1**	**U**	**K.YEELQQTAGR.H**
49756	945.9924	1889.9703	1889.9635	3.61	0	70	5.9e-06	1		R.QNLEPLFEQYINNLR.R 49755

Proteins matching the same set of peptides:
gi|9739163 **Mass:** 62651 **Score:** 257 **Matches:** 9(9) **Sequences:** 5(5)
keratin 5 [Homo sapiens]
gi|18999435 **Mass:** 62568 **Score:** 257 **Matches:** 9(9) **Sequences:** 5(5)
Keratin 5 [Homo sapiens]
gi|119395754 **Mass:** 62568 **Score:** 257 **Matches:** 9(9) **Sequences:** 5(5)
keratin, type II cytoskeletal 5 [Homo sapiens]
gi|119617040 **Mass:** 60317 **Score:** 257 **Matches:** 9(9) **Sequences:** 5(5)
keratin 5 (epidermolysis bullosa simplex, Dowling-Meara/Kobner/Weber-Cockayne types), isoform CRA_c [Homo sapiens]
gi|119617041 **Mass:** 59853 **Score:** 257 **Matches:** 9(9) **Sequences:** 5(5)
keratin 5 (epidermolysis bullosa simplex, Dowling-Meara/Kobner/Weber-Cockayne types), isoform CRA_d [Homo sapiens]
gi|194384240 **Mass:** 59042 **Score:** 257 **Matches:** 9(9) **Sequences:** 5(5)
unnamed protein product [Homo sapiens]

Proteins matching a subset of these peptides:
gi|194384468 **Mass:** 27409 **Score:** 136 **Matches:** 3(3) **Sequences:** 3(3)
unnamed protein product [Homo sapiens]
gi|392311616 **Mass:** 15097 **Score:** 40 **Matches:** 1(1) **Sequences:** 1(1)
Chain B, Heterocomplex Of Coil 2b Domains Of Human Intermediate Filament Proteins, Keratin 5 (Krt5) And Keratin 14 (Krt14)

---

12. gi|374977971 **Mass:** 43647 **Score:** 77 **Matches:** 1(1) **Sequences:** 1(1) **emPAI:** 0.09
Chain A, Crystal Structure Of Branched-chain Alpha-ketoacid Dehydrogenase Phosphatase With Mg (ii) Ions At The Active Site

Query	Observed	Mr(expt)	Mr(calc)	ppm	Miss	Score	Expect	Rank	Unique	Peptide

33584 608.2987 1214.5829 1214.5819 0.79 0 77 8.5e-07 1 U K.VSDGSSEIFFK.I

---

13. gi|37460 **Mass:** 27329 **Score:** 75 **Matches:** 2(2) **Sequences:** 1(1) **emPAI:** 0.14
unnamed protein product [Homo sapiens]

Query	Observed	Mr(expt)	Mr(calc)	ppm	Miss	Score	Expect	Rank	Unique	Peptide
37275	716.8778	1431.7410	1431.7358	3.68	0	75	2e-06	1	U	K.VLEGNEQFINAAK.I 37274

Proteins matching the same set of peptides:
gi|2136330 **Mass:** 28593 **Score:** 75 **Matches:** 2(2) **Sequences:** 1(1)
trypsin (EC 3.4.21.4) IV form b precursor - human (fragment)
gi|3980129 **Mass:** 28611 **Score:** 75 **Matches:** 2(2) **Sequences:** 1(1)
trypsinogen IV b-form [Homo sapiens]
gi|6066378 **Mass:** 28779 **Score:** 75 **Matches:** 2(2) **Sequences:** 1(1)
trypsinogen IV a-form [Homo sapiens]
gi|20988417 **Mass:** 27702 **Score:** 75 **Matches:** 2(2) **Sequences:** 1(1)
PRSS3 protein, partial [Homo sapiens]
gi|21536452 **Mass:** 27250 **Score:** 75 **Matches:** 2(2) **Sequences:** 1(1)
trypsin-3 isoform 2 preproprotein [Homo sapiens]
gi|29649713 **Mass:** 9218 **Score:** 75 **Matches:** 2(2) **Sequences:** 1(1)
protease serine 1 [Homo sapiens]
gi|33126535 **Mass:** 28751 **Score:** 75 **Matches:** 2(2) **Sequences:** 1(1)
protease serine 4 isoform B [Homo sapiens]
gi|118763987 **Mass:** 27040 **Score:** 75 **Matches:** 2(2) **Sequences:** 1(1)
PRSS3 protein [Homo sapiens]
gi|162330095 **Mass:** 24788 **Score:** 75 **Matches:** 2(2) **Sequences:** 1(1)
Chain A, Human Mesotrypsin Complexed With Bovine Pancreatic Trypsin Inhibitor(Bpti)
gi|170296790 **Mass:** 33276 **Score:** 75 **Matches:** 2(2) **Sequences:** 1(1)
trypsin-3 isoform 1 preproprotein [Homo sapiens]
gi|209572698 **Mass:** 33306 **Score:** 75 **Matches:** 2(2) **Sequences:** 1(1)
RecName: Full=Trypsin-3; AltName: Full=Brain trypsinogen; AltName: Full=Mesotrypsinogen; AltName: Full=Serine protease 3; AltName: Full=Serine protease 4; AltName: Full=Trypsin III; AltName: Full=Try
gi|308193325 **Mass:** 26448 **Score:** 75 **Matches:** 2(2) **Sequences:** 1(1)
trypsin-3 isoform 4 preproprotein [Homo sapiens]
gi|342672030 **Mass:** 28924 **Score:** 75 **Matches:** 2(2) **Sequences:** 1(1)
trypsin-3 isoform 3 preproprotein [Homo sapiens]
gi|404312861 **Mass:** 24864 **Score:** 75 **Matches:** 2(2) **Sequences:** 1(1)
Chain A, Human Mesotrypsin-s39y Complexed With Bovine Pancreatic Trypsin Inhibitor (bpti)

---

14. gi|51873887 **Mass:** 48402 **Score:** 47 **Matches:** 12(12) **Sequences:** 1(1) **emPAI:** 0.08
DNAJC14 protein, partial [Homo sapiens]

Query	Observed	Mr(expt)	Mr(calc)	ppm	Miss	Score	Expect	Rank	Unique	Peptide
22864	778.4104	777.4031	777.4021	1.32	0	47	0.003	1	U	-.DLDLFR.V 22866 22867 22868 22870 22871 22873 22874 22875 22876 22879 22883

---

15. gi|1552516 **Mass:** 27092 **Score:** 45 **Matches:** 2(2) **Sequences:** 1(1) **emPAI:** 0.14
trypsinogen C [Homo sapiens]

Query	Observed	Mr(expt)	Mr(calc)	ppm	Miss	Score	Expect	Rank	Unique	Peptide
57337	742.3804	2224.1193	2224.1124	3.13	0	45	0.0014	1	U	R.LGEHNIEVLEGNEQFINAAK.I 57330

Proteins matching the same set of peptides:
gi|1616766 **Mass:** 9200 **Score:** 45 **Matches:** 2(2) **Sequences:** 1(1)
cationic trypsinogen, partial [Homo sapiens]
gi|4506145 **Mass:** 27111 **Score:** 45 **Matches:** 2(2) **Sequences:** 1(1)
trypsin-1 preproprotein [Homo sapiens]
gi|4506147 **Mass:** 26927 **Score:** 45 **Matches:** 2(2) **Sequences:** 1(1)
trypsin-2 isoform 2 preproprotein [Homo sapiens]
gi|11120624 **Mass:** 9203 **Score:** 45 **Matches:** 2(2) **Sequences:** 1(1)
cationic trypsinogen [Homo sapiens]
gi|11120626 **Mass:** 9223 **Score:** 45 **Matches:** 2(2) **Sequences:** 1(1)

cationic trypsinogen [Homo sapiens]
gi|11120628 **Mass:** 9206 **Score:** 45 **Matches:** 2(2) **Sequences:** 1(1)
cationic trypsinogen [Homo sapiens]
gi|29649776 **Mass:** 9223 **Score:** 45 **Matches:** 2(2) **Sequences:** 1(1)
protease serine 1 [Homo sapiens]
gi|29649786 **Mass:** 9251 **Score:** 45 **Matches:** 2(2) **Sequences:** 1(1)
protease serine 1 [Homo sapiens]
gi|33126583 **Mass:** 28575 **Score:** 45 **Matches:** 2(2) **Sequences:** 1(1)
protease serine 2 isoform B [Homo sapiens]
gi|39644739 **Mass:** 20434 **Score:** 45 **Matches:** 2(2) **Sequences:** 1(1)
PRSS2 protein, partial [Homo sapiens]
gi|71738264 **Mass:** 9219 **Score:** 45 **Matches:** 2(2) **Sequences:** 1(1)
trypsin I [Homo sapiens]
gi|74353564 **Mass:** 26911 **Score:** 45 **Matches:** 2(2) **Sequences:** 1(1)
Protease, serine, 2 (trypsin 2) [Homo sapiens]
gi|74355190 **Mass:** 26995 **Score:** 45 **Matches:** 2(2) **Sequences:** 1(1)
PRSS1 protein [Homo sapiens]
gi|118764023 **Mass:** 27145 **Score:** 45 **Matches:** 2(2) **Sequences:** 1(1)
Protease, serine, 1 (trypsin 1) [Homo sapiens]
gi|157880238 **Mass:** 24617 **Score:** 45 **Matches:** 2(2) **Sequences:** 1(1)
Chain A, Crystal Structure Of Human Trypsin 1: Unexpected Phosphorylation Of Tyrosine 151
gi|162330103 **Mass:** 24634 **Score:** 45 **Matches:** 2(2) **Sequences:** 1(1)
Chain A, Human Cationic Trypsin Complexed With Bovine Pancreatic Trypsin Inhibitor (bpti)
gi|530431211 **Mass:** 27166 **Score:** 45 **Matches:** 2(2) **Sequences:** 1(1)
PREDICTED: trypsin-2 isoform X3 [Homo sapiens]
gi|578798106 **Mass:** 27092 **Score:** 45 **Matches:** 2(2) **Sequences:** 1(1)
PREDICTED: putative trypsin-6-like [Homo sapiens]
gi|586830503 **Mass:** 27090 **Score:** 45 **Matches:** 2(2) **Sequences:** 1(1)
RecName: Full=Putative trypsin-6; AltName: Full=Serine protease 3 pseudogene 2; AltName: Full=Trypsinogen C; Flags: Precursor [Homo sapiens]
gi|744066875 **Mass:** 28500 **Score:** 45 **Matches:** 2(2) **Sequences:** 1(1)
trypsin-2 isoform 1 preproprotein [Homo sapiens]

---

16. gi|34533832 **Mass:** 15522 **Score:** 41 **Matches:** 1(1) **Sequences:** 1(1) **emPAI:** 0.25
unnamed protein product [Homo sapiens]

Query	Observed	Mr(expt)	Mr(calc)	ppm	Miss	Score	Expect	Rank	Unique	Peptide
19522	672.4041	671.3968	671.3966	0.32	0	41	0.0055	1	U	K.TVSIPR.S

---

17. gi|119575127 **Mass:** 49672 **Score:** 40 **Matches:** 9(9) **Sequences:** 1(1) **emPAI:** 0.08
hCG1987696, isoform CRA_a [Homo sapiens]

Query	Observed	Mr(expt)	Mr(calc)	ppm	Miss	Score	Expect	Rank	Unique	Peptide
20396	703.3656	702.3583	702.3548	5.02	0	40	0.017	1	U	R.DEAIQK.L 20389 20390 20391 20392 20394 20395 20397 20398

Proteins matching the same set of peptides:
gi|119575129 **Mass:** 56221 **Score:** 40 **Matches:** 9(9) **Sequences:** 1(1)
hCG2045904, isoform CRA_a [Homo sapiens]
gi|119575130 **Mass:** 57386 **Score:** 40 **Matches:** 9(9) **Sequences:** 1(1)
hCG2045904, isoform CRA_b [Homo sapiens]
gi|476007894 **Mass:** 46857 **Score:** 40 **Matches:** 9(9) **Sequences:** 1(1)
T-complex protein 11 homolog [Homo sapiens]
gi|550540902 **Mass:** 34941 **Score:** 40 **Matches:** 9(9) **Sequences:** 1(1)
RecName: Full=T-complex protein 11 X-linked protein 1 [Homo sapiens]

---

18. gi|58330892 **Mass:** 55528 **Score:** 40 **Matches:** 2(2) **Sequences:** 1(1) **emPAI:** 0.07
interferon-induced protein with tetratricopeptide repeats 1B [Homo sapiens]

Query	Observed	Mr(expt)	Mr(calc)	ppm	Miss	Score	Expect	Rank	Unique	Peptide
24077	816.4469	815.4396	815.4389	0.94	0	(40)	0.011	1	U	K.AEDLIQK.E 24079

---

19. gi|106258 **Score:** 39 **Matches:** 1(1) **Sequences:** 1(1) **emPAI:** 0.10

glycoprotein-fucosylgalactoside alpha-N-acetylgalactosaminyltransferase (EC 2.4.1.40) A1 allele [validated] - human

Query	Observed	Mr(expt)	Mr(calc)	ppm	Miss	Score	Expect	Rank	Unique	Peptide
13419	571.3570	570.3497	570.3489	1.37	0	39	0.0044	1	U	R.VSLPR.M

Proteins matching the same set of peptides:

gi|4590452 Score: 39 Matches: 1(1) Sequences: 1(1)
gi|4590454 Score: 39 Matches: 1(1) Sequences: 1(1)
gi|23200402 Score: 39 Matches: 1(1) Sequences: 1(1)
gi|23200403 Score: 39 Matches: 1(1) Sequences: 1(1)
gi|40643612 Score: 39 Matches: 1(1) Sequences: 1(1)
gi|40643614 Score: 39 Matches: 1(1) Sequences: 1(1)
gi|40643620 Score: 39 Matches: 1(1) Sequences: 1(1)
gi|40643622 Score: 39 Matches: 1(1) Sequences: 1(1)
gi|40643628 Score: 39 Matches: 1(1) Sequences: 1(1)
gi|40643630 Score: 39 Matches: 1(1) Sequences: 1(1)
gi|40643632 Score: 39 Matches: 1(1) Sequences: 1(1)
gi|40643636 Score: 39 Matches: 1(1) Sequences: 1(1)
gi|40643638 Score: 39 Matches: 1(1) Sequences: 1(1)
gi|40643642 Score: 39 Matches: 1(1) Sequences: 1(1)
gi|40643644 Score: 39 Matches: 1(1) Sequences: 1(1)
gi|40643646 Score: 39 Matches: 1(1) Sequences: 1(1)
gi|40643648 Score: 39 Matches: 1(1) Sequences: 1(1)
gi|40643672 Score: 39 Matches: 1(1) Sequences: 1(1)
gi|46015440 Score: 39 Matches: 1(1) Sequences: 1(1)
gi|46015441 Score: 39 Matches: 1(1) Sequences: 1(1)
gi|47481204 Score: 39 Matches: 1(1) Sequences: 1(1)
gi|58177213 Score: 39 Matches: 1(1) Sequences: 1(1)
gi|58177214 Score: 39 Matches: 1(1) Sequences: 1(1)
gi|58177215 Score: 39 Matches: 1(1) Sequences: 1(1)
gi|58331216 Score: 39 Matches: 1(1) Sequences: 1(1)
gi|61139582 Score: 39 Matches: 1(1) Sequences: 1(1)
gi|61139584 Score: 39 Matches: 1(1) Sequences: 1(1)
gi|72256530 Score: 39 Matches: 1(1) Sequences: 1(1)
gi|119569500 Score: 39 Matches: 1(1) Sequences: 1(1)
gi|119608460 Score: 39 Matches: 1(1) Sequences: 1(1)
gi|149242126 Score: 39 Matches: 1(1) Sequences: 1(1)
gi|157835678 Score: 39 Matches: 1(1) Sequences: 1(1)
gi|167465947 Score: 39 Matches: 1(1) Sequences: 1(1)
gi|300393858 Score: 39 Matches: 1(1) Sequences: 1(1)
gi|307343246 Score: 39 Matches: 1(1) Sequences: 1(1)
gi|307343248 Score: 39 Matches: 1(1) Sequences: 1(1)
gi|310722951 Score: 39 Matches: 1(1) Sequences: 1(1)
gi|323695272 Score: 39 Matches: 1(1) Sequences: 1(1)
gi|325197080 Score: 39 Matches: 1(1) Sequences: 1(1)
gi|325197088 Score: 39 Matches: 1(1) Sequences: 1(1)
gi|325197094 Score: 39 Matches: 1(1) Sequences: 1(1)
gi|325197098 Score: 39 Matches: 1(1) Sequences: 1(1)
gi|325197100 Score: 39 Matches: 1(1) Sequences: 1(1)
gi|326787485 Score: 39 Matches: 1(1) Sequences: 1(1)
gi|341572387 Score: 39 Matches: 1(1) Sequences: 1(1)
gi|345650982 Score: 39 Matches: 1(1) Sequences: 1(1)
gi|354806304 Score: 39 Matches: 1(1) Sequences: 1(1)
gi|377657173 Score: 39 Matches: 1(1) Sequences: 1(1)
gi|406042101 Score: 39 Matches: 1(1) Sequences: 1(1)
gi|406042103 Score: 39 Matches: 1(1) Sequences: 1(1)
gi|406042107 Score: 39 Matches: 1(1) Sequences: 1(1)
gi|409191781 Score: 39 Matches: 1(1) Sequences: 1(1)
gi|409191783 Score: 39 Matches: 1(1) Sequences: 1(1)
gi|529158559 Score: 39 Matches: 1(1) Sequences: 1(1)
gi|529158561 Score: 39 Matches: 1(1) Sequences: 1(1)
gi|541881490 Score: 39 Matches: 1(1) Sequences: 1(1)
gi|568786489 Score: 39 Matches: 1(1) Sequences: 1(1)
gi|568786493 Score: 39 Matches: 1(1) Sequences: 1(1)
gi|568786495 Score: 39 Matches: 1(1) Sequences: 1(1)

gi|568786499 **Score:** 39 **Matches:** 1(1) **Sequences:** 1(1)
gi|600971539 **Score:** 39 **Matches:** 1(1) **Sequences:** 1(1)
gi|641689262 **Score:** 39 **Matches:** 1(1) **Sequences:** 1(1)
gi|641689264 **Score:** 39 **Matches:** 1(1) **Sequences:** 1(1)
gi|641689266 **Score:** 39 **Matches:** 1(1) **Sequences:** 1(1)
gi|651209619 **Score:** 39 **Matches:** 1(1) **Sequences:** 1(1)
gi|728797230 **Score:** 39 **Matches:** 1(1) **Sequences:** 1(1)
gi|728797232 **Score:** 39 **Matches:** 1(1) **Sequences:** 1(1)
gi|743405510 **Score:** 39 **Matches:** 1(1) **Sequences:** 1(1)
gi|406042105 **Score:** 39 **Matches:** 1(1) **Sequences:** 1(1)

---

20. gi|3406800 **Mass:** 12760 **Score:** 38 **Matches:** 4(4) **Sequences:** 1(1) **emPAI:** 0.31
taxol resistant associated protein [Homo sapiens]

Query	Observed	Mr(expt)	Mr(calc)	ppm	Miss	Score	Expect	Rank	Unique	Peptide
15192	602.3525	601.3452	601.3435	2.80	0	38	0.023	1	U	R.DAGLVK.M 15184 15187 15194

Proteins matching the same set of peptides:
gi|5019774 **Mass:** 14513 **Score:** 38 **Matches:** 4(4) **Sequences:** 1(1)
taxol resistant associated protein [Homo sapiens]
gi|5174695 **Mass:** 37695 **Score:** 38 **Matches:** 4(4) **Sequences:** 1(1)
3-oxo-5-beta-steroid 4-dehydrogenase isoform 1 [Homo sapiens]
gi|16554192 **Mass:** 16911 **Score:** 38 **Matches:** 4(4) **Sequences:** 1(1)
unnamed protein product [Homo sapiens]
gi|119604288 **Mass:** 28624 **Score:** 38 **Matches:** 4(4) **Sequences:** 1(1)
aldo-keto reductase family 1, member D1 (delta 4-3-ketosteroid-5-beta-reductase), isoform CRA_b, partial [Homo sapiens]
gi|119611199 **Mass:** 10113 **Score:** 38 **Matches:** 4(4) **Sequences:** 1(1)
hCG1795084 [Homo sapiens]
gi|158259873 **Mass:** 37679 **Score:** 38 **Matches:** 4(4) **Sequences:** 1(1)
unnamed protein product [Homo sapiens]
gi|178847589 **Mass:** 39857 **Score:** 38 **Matches:** 4(4) **Sequences:** 1(1)
Chain A, Crystal Structure Of Human Liver 5beta-Reductase (Akr1d1) In Complex With Nadp And Cortisone. Resolution 1.90 A.
gi|193788718 **Mass:** 14535 **Score:** 38 **Matches:** 4(4) **Sequences:** 1(1)
chondrosarcoma-associated gene 2/3 protein isoform a [Homo sapiens]
gi|193788726 **Mass:** 12781 **Score:** 38 **Matches:** 4(4) **Sequences:** 1(1)
chondrosarcoma-associated gene 2/3 protein isoform b [Homo sapiens]
gi|237768376 **Mass:** 19103 **Score:** 38 **Matches:** 4(4) **Sequences:** 1(1)
aldo-keto reductase family 1, member D1 [Homo sapiens]
gi|237768378 **Mass:** 20116 **Score:** 38 **Matches:** 4(4) **Sequences:** 1(1)
aldo-keto reductase family 1, member D1 [Homo sapiens]
gi|237768380 **Mass:** 1664 **Score:** 38 **Matches:** 4(4) **Sequences:** 1(1)
aldo-keto reductase family 1, member D1 [Homo sapiens]
gi|300116273 **Mass:** 33069 **Score:** 38 **Matches:** 4(4) **Sequences:** 1(1)
3-oxo-5-beta-steroid 4-dehydrogenase isoform 3 [Homo sapiens]
gi|380764909 **Mass:** 39865 **Score:** 38 **Matches:** 4(4) **Sequences:** 1(1)
Chain A, Crystal Structure Of 5beta-Reductase (Akr1d1) E120h Mutant In Complex With Nadp+

Mascot: http://www.matrixscience.com/
