# Supplementary material for: A metabolite-bridged complex between SerRS and SIRT2 couples NAD⁺ metabolism to translation control
Source: Nat Commun. 2026 Jul 8;17:8427. doi: 10.1038/s41467-026-75266-4 (PMC13478228; doi:10.1038/s41467-026-75266-4)
Supplement: Supplementary file 4 [file 41467_2026_75266_MOESM4_ESM.zip › Source_Data_Trypsin Digestion Mass Spectrometry_SeRS.pdf]

Mascot Search Results

Protein View

Match to: **AABT7310_SerRS** Score: **3669**
AABT7310_SerRS
Found in search of \\scarletvault.scripps.edu\userdata4\QE_data\2022\Nov 2022\11302022\AABT7572.raw

Nominal mass (M_r): **59253**; Calculated pI value: **6.05**
NCBI BLAST search of AABT7310_SerRS against nr
Unformatted sequence string for pasting into other applications

Fixed modifications: Carbamidomethyl (C)
Variable modifications: Acetylation (K),Myristoylation_K (K),Oxidation (M),Palmitoylation_K (K)
Cleavage by Trypsin: cuts C-term side of KR unless next residue is P
Sequence Coverage: **90%**

Matched peptides shown in **Bold Red**

```
  1 MVLDLDLFRV DKGGDPALIR ETQEKRFKDP GLVDQLVKAD SEWRRCRFRA
 51 DNLNKLKNLC SKTIGEKMKK KEPVGDDESV PENVLSFDDL TADALANLKV
101 SQIKKVRLLI DEAILKCDAE RIKLEAERFE NLREIGNLLH PSVPISNDED
151 VDNKVERIWG DCTVRKKYSH VDLVVMVDGF EGEKGAVVAG SRGYFLKGVL
201 VFLEQALIQY ALRTLGSRGY IPIYTPFFMR KEVMQEVAQL SQFDEELYKV
251 IGKGSEKSDD NSYDEKYLIA TSEQPIAALH RDEWLRPEDL PIKYAGLSTC
301 FRQEVGSHGR DTRGIFRVHQ FEKIEQFVYS SPHDNKSWEM FEEMITTAEE
351 FYQSLGIPYH IVNIVSGSLN HAASKKLDLE AWFPGSGAFR ELVSCSNCTD
401 YQARRLRIRY GQTKKMMDKV EFVHMLNATM CATTRTICAI LENYQTEKGI
451 TVPEKLKEFM PPGLQELIPF VKPAPIEQEP SKKQKKQHEG SKKKAAARDV
501 TLENRLQNME VTDA
```

Show predicted peptides also

Sort Peptides By ◉ Residue Number ○ Increasing Mass ○ Decreasing Mass

Start - End	Observed	Mr(expt)	Mr(calc)	ppm	Miss	Sequence		
1 - 9	561.3056	1120.5967	1120.5951	1	0	-.MVLDLDLFR.V		(Ions score 27)
1 - 9	561.3056	1120.5967	1120.5951	1	0	-.MVLDLDLFR.V		(Ions score 51)
1 - 9	561.3057	1120.5969	1120.5951	2	0	-.MVLDLDLFR.V		(Ions score 47)
1 - 9	561.3058	1120.5970	1120.5951	2	0	-.MVLDLDLFR.V		(Ions score 47)
1 - 9	561.3061	1120.5976	1120.5951	2	0	-.MVLDLDLFR.V		(Ions score 46)
1 - 9	561.3061	1120.5976	1120.5951	2	0	-.MVLDLDLFR.V		(Ions score 21)
1 - 9	561.3062	1120.5979	1120.5951	3	0	-.MVLDLDLFR.V		(Ions score 51)
1 - 9	561.3069	1120.5993	1120.5951	4	0	-.MVLDLDLFR.V		(Ions score 21)
1 - 9	569.3008	1136.5870	1136.5900	-3	0	-.MVLDLDLFR.V	Oxidation (M)	(Ions score 42)
1 - 9	569.3027	1136.5909	1136.5900	1	0	-.MVLDLDLFR.V	Oxidation (M)	(Ions score 34)
1 - 9	569.3029	1136.5912	1136.5900	1	0	-.MVLDLDLFR.V	Oxidation (M)	(Ions score 24)
1 - 9	569.3029	1136.5913	1136.5900	1	0	-.MVLDLDLFR.V	Oxidation (M)	(Ions score 32)
1 - 9	569.3032	1136.5919	1136.5900	2	0	-.MVLDLDLFR.V	Oxidation (M)	(Ions score 37)
1 - 9	569.3035	1136.5924	1136.5900	2	0	-.MVLDLDLFR.V	Oxidation (M)	(Ions score 39)
1 - 9	569.3036	1136.5927	1136.5900	2	0	-.MVLDLDLFR.V	Oxidation (M)	(Ions score 23)
2 - 9	495.7841	989.5537	989.5546	-1	0	M.VLDLDLFR.V		(Ions score 37)
2 - 9	495.7842	989.5539	989.5546	-1	0	M.VLDLDLFR.V		(Ions score 39)
2 - 9	495.7842	989.5539	989.5546	-1	0	M.VLDLDLFR.V		(Ions score 24)
2 - 9	495.7843	989.5541	989.5546	-1	0	M.VLDLDLFR.V		(Ions score 52)
2 - 9	495.7843	989.5541	989.5546	-0	0	M.VLDLDLFR.V		(Ions score 36)
2 - 9	495.7844	989.5542	989.5546	-0	0	M.VLDLDLFR.V		(Ions score 35)
2 - 9	495.7844	989.5543	989.5546	-0	0	M.VLDLDLFR.V		(Ions score 39)
2 - 9	495.7845	989.5545	989.5546	-0	0	M.VLDLDLFR.V		(Ions score 40)
2 - 9	495.7845	989.5545	989.5546	-0	0	M.VLDLDLFR.V		(Ions score 26)

2 - 9	495.7846	989.5546	989.5546	0	0	M.VLDLDLFR.V	(Ions score 40)
2 - 9	495.7846	989.5546	989.5546	0	0	M.VLDLDLFR.V	(Ions score 40)
2 - 9	495.7847	989.5548	989.5546	0	0	M.VLDLDLFR.V	(Ions score 36)
2 - 9	495.7847	989.5548	989.5546	0	0	M.VLDLDLFR.V	(Ions score 40)
2 - 9	495.7847	989.5549	989.5546	0	0	M.VLDLDLFR.V	(Ions score 24)
2 - 9	495.7847	989.5549	989.5546	0	0	M.VLDLDLFR.V	(Ions score 40)
2 - 9	495.7848	989.5550	989.5546	0	0	M.VLDLDLFR.V	(Ions score 25)
2 - 9	495.7848	989.5550	989.5546	0	0	M.VLDLDLFR.V	(Ions score 36)
2 - 9	495.7848	989.5550	989.5546	0	0	M.VLDLDLFR.V	(Ions score 39)
2 - 9	495.7848	989.5550	989.5546	0	0	M.VLDLDLFR.V	(Ions score 39)
2 - 9	495.7848	989.5550	989.5546	0	0	M.VLDLDLFR.V	(Ions score 40)
2 - 9	495.7848	989.5550	989.5546	0	0	M.VLDLDLFR.V	(Ions score 40)
2 - 9	495.7848	989.5550	989.5546	0	0	M.VLDLDLFR.V	(Ions score 39)
2 - 9	495.7848	989.5550	989.5546	0	0	M.VLDLDLFR.V	(Ions score 36)
2 - 9	495.7848	989.5550	989.5546	0	0	M.VLDLDLFR.V	(Ions score 35)
2 - 9	495.7848	989.5550	989.5546	0	0	M.VLDLDLFR.V	(Ions score 39)
2 - 9	495.7848	989.5551	989.5546	1	0	M.VLDLDLFR.V	(Ions score 40)
2 - 9	495.7848	989.5551	989.5546	1	0	M.VLDLDLFR.V	(Ions score 36)
2 - 9	495.7848	989.5551	989.5546	1	0	M.VLDLDLFR.V	(Ions score 36)
2 - 9	495.7848	989.5551	989.5546	1	0	M.VLDLDLFR.V	(Ions score 40)
2 - 9	495.7849	989.5552	989.5546	1	0	M.VLDLDLFR.V	(Ions score 39)
2 - 9	495.7849	989.5552	989.5546	1	0	M.VLDLDLFR.V	(Ions score 36)
2 - 9	495.7849	989.5552	989.5546	1	0	M.VLDLDLFR.V	(Ions score 39)
2 - 9	495.7849	989.5552	989.5546	1	0	M.VLDLDLFR.V	(Ions score 22)
2 - 9	495.7849	989.5552	989.5546	1	0	M.VLDLDLFR.V	(Ions score 40)
2 - 9	495.7849	989.5552	989.5546	1	0	M.VLDLDLFR.V	(Ions score 39)
2 - 9	495.7849	989.5552	989.5546	1	0	M.VLDLDLFR.V	(Ions score 40)
2 - 9	495.7849	989.5552	989.5546	1	0	M.VLDLDLFR.V	(Ions score 39)
2 - 9	495.7849	989.5552	989.5546	1	0	M.VLDLDLFR.V	(Ions score 36)
2 - 9	495.7849	989.5552	989.5546	1	0	M.VLDLDLFR.V	(Ions score 25)
2 - 9	495.7849	989.5552	989.5546	1	0	M.VLDLDLFR.V	(Ions score 40)
2 - 9	495.7849	989.5552	989.5546	1	0	M.VLDLDLFR.V	(Ions score 39)
2 - 9	495.7849	989.5553	989.5546	1	0	M.VLDLDLFR.V	(Ions score 40)
2 - 9	495.7849	989.5553	989.5546	1	0	M.VLDLDLFR.V	(Ions score 40)
2 - 9	495.7849	989.5553	989.5546	1	0	M.VLDLDLFR.V	(Ions score 39)
2 - 9	495.7849	989.5553	989.5546	1	0	M.VLDLDLFR.V	(Ions score 39)
2 - 9	495.7849	989.5553	989.5546	1	0	M.VLDLDLFR.V	(Ions score 41)
2 - 9	495.7849	989.5553	989.5546	1	0	M.VLDLDLFR.V	(Ions score 39)
2 - 9	495.7849	989.5553	989.5546	1	0	M.VLDLDLFR.V	(Ions score 24)
2 - 9	495.7850	989.5554	989.5546	1	0	M.VLDLDLFR.V	(Ions score 39)
2 - 9	495.7850	989.5554	989.5546	1	0	M.VLDLDLFR.V	(Ions score 39)
2 - 9	495.7850	989.5554	989.5546	1	0	M.VLDLDLFR.V	(Ions score 36)
2 - 9	495.7850	989.5554	989.5546	1	0	M.VLDLDLFR.V	(Ions score 33)
2 - 9	495.7850	989.5555	989.5546	1	0	M.VLDLDLFR.V	(Ions score 72)
2 - 9	495.7850	989.5555	989.5546	1	0	M.VLDLDLFR.V	(Ions score 40)
2 - 9	495.7850	989.5555	989.5546	1	0	M.VLDLDLFR.V	(Ions score 40)
2 - 9	495.7850	989.5555	989.5546	1	0	M.VLDLDLFR.V	(Ions score 39)
2 - 9	495.7850	989.5555	989.5546	1	0	M.VLDLDLFR.V	(Ions score 36)
2 - 9	495.7850	989.5555	989.5546	1	0	M.VLDLDLFR.V	(Ions score 40)
2 - 9	495.7850	989.5555	989.5546	1	0	M.VLDLDLFR.V	(Ions score 40)
2 - 9	495.7850	989.5555	989.5546	1	0	M.VLDLDLFR.V	(Ions score 39)
2 - 9	495.7850	989.5555	989.5546	1	0	M.VLDLDLFR.V	(Ions score 40)
2 - 9	495.7850	989.5555	989.5546	1	0	M.VLDLDLFR.V	(Ions score 40)
2 - 9	495.7850	989.5555	989.5546	1	0	M.VLDLDLFR.V	(Ions score 39)
2 - 9	495.7851	989.5556	989.5546	1	0	M.VLDLDLFR.V	(Ions score 39)
2 - 9	495.7851	989.5556	989.5546	1	0	M.VLDLDLFR.V	(Ions score 39)
2 - 9	495.7851	989.5556	989.5546	1	0	M.VLDLDLFR.V	(Ions score 40)
2 - 9	495.7851	989.5556	989.5546	1	0	M.VLDLDLFR.V	(Ions score 39)

2 - 9	495.7851	989.5556	989.5546	1	0	M.VLDLDLFR.V	(Ions score 40)
2 - 9	495.7851	989.5556	989.5546	1	0	M.VLDLDLFR.V	(Ions score 25)
2 - 9	495.7851	989.5556	989.5546	1	0	M.VLDLDLFR.V	(Ions score 39)
2 - 9	495.7851	989.5556	989.5546	1	0	M.VLDLDLFR.V	(Ions score 40)
2 - 9	495.7851	989.5556	989.5546	1	0	M.VLDLDLFR.V	(Ions score 40)
2 - 9	495.7851	989.5556	989.5546	1	0	M.VLDLDLFR.V	(Ions score 40)
2 - 9	495.7851	989.5556	989.5546	1	0	M.VLDLDLFR.V	(Ions score 40)
2 - 9	495.7851	989.5556	989.5546	1	0	M.VLDLDLFR.V	(Ions score 39)
2 - 9	495.7851	989.5556	989.5546	1	0	M.VLDLDLFR.V	(Ions score 40)
2 - 9	495.7851	989.5557	989.5546	1	0	M.VLDLDLFR.V	(Ions score 40)
2 - 9	495.7851	989.5557	989.5546	1	0	M.VLDLDLFR.V	(Ions score 39)
2 - 9	495.7851	989.5557	989.5546	1	0	M.VLDLDLFR.V	(Ions score 40)
2 - 9	495.7851	989.5557	989.5546	1	0	M.VLDLDLFR.V	(Ions score 36)
2 - 9	495.7851	989.5557	989.5546	1	0	M.VLDLDLFR.V	(Ions score 40)
2 - 9	495.7851	989.5557	989.5546	1	0	M.VLDLDLFR.V	(Ions score 40)
2 - 9	495.7851	989.5557	989.5546	1	0	M.VLDLDLFR.V	(Ions score 35)
2 - 9	495.7851	989.5557	989.5546	1	0	M.VLDLDLFR.V	(Ions score 52)
2 - 9	495.7852	989.5558	989.5546	1	0	M.VLDLDLFR.V	(Ions score 39)
2 - 9	495.7852	989.5558	989.5546	1	0	M.VLDLDLFR.V	(Ions score 39)
2 - 9	495.7852	989.5558	989.5546	1	0	M.VLDLDLFR.V	(Ions score 40)
2 - 9	495.7852	989.5558	989.5546	1	0	M.VLDLDLFR.V	(Ions score 39)
2 - 9	495.7852	989.5558	989.5546	1	0	M.VLDLDLFR.V	(Ions score 39)
2 - 9	495.7852	989.5559	989.5546	1	0	M.VLDLDLFR.V	(Ions score 40)
2 - 9	495.7852	989.5559	989.5546	1	0	M.VLDLDLFR.V	(Ions score 40)
2 - 9	495.7852	989.5559	989.5546	1	0	M.VLDLDLFR.V	(Ions score 39)
2 - 9	495.7852	989.5559	989.5546	1	0	M.VLDLDLFR.V	(Ions score 40)
2 - 9	495.7852	989.5559	989.5546	1	0	M.VLDLDLFR.V	(Ions score 33)
2 - 9	495.7852	989.5559	989.5546	1	0	M.VLDLDLFR.V	(Ions score 39)
2 - 9	495.7852	989.5559	989.5546	1	0	M.VLDLDLFR.V	(Ions score 36)
2 - 9	495.7852	989.5559	989.5546	1	0	M.VLDLDLFR.V	(Ions score 39)
2 - 9	495.7852	989.5559	989.5546	1	0	M.VLDLDLFR.V	(Ions score 34)
2 - 9	495.7852	989.5559	989.5546	1	0	M.VLDLDLFR.V	(Ions score 36)
2 - 9	495.7852	989.5559	989.5546	1	0	M.VLDLDLFR.V	(Ions score 40)
2 - 9	495.7852	989.5559	989.5546	1	0	M.VLDLDLFR.V	(Ions score 40)
2 - 9	495.7852	989.5559	989.5546	1	0	M.VLDLDLFR.V	(Ions score 40)
2 - 9	495.7852	989.5559	989.5546	1	0	M.VLDLDLFR.V	(Ions score 33)
2 - 9	495.7852	989.5559	989.5546	1	0	M.VLDLDLFR.V	(Ions score 35)
2 - 9	495.7853	989.5560	989.5546	1	0	M.VLDLDLFR.V	(Ions score 40)
2 - 9	495.7853	989.5560	989.5546	1	0	M.VLDLDLFR.V	(Ions score 35)
2 - 9	495.7853	989.5560	989.5546	1	0	M.VLDLDLFR.V	(Ions score 40)
2 - 9	495.7853	989.5560	989.5546	1	0	M.VLDLDLFR.V	(Ions score 50)
2 - 9	495.7853	989.5561	989.5546	2	0	M.VLDLDLFR.V	(Ions score 40)
2 - 9	495.7853	989.5561	989.5546	2	0	M.VLDLDLFR.V	(Ions score 40)
2 - 9	495.7853	989.5561	989.5546	2	0	M.VLDLDLFR.V	(Ions score 40)
2 - 9	495.7853	989.5561	989.5546	2	0	M.VLDLDLFR.V	(Ions score 39)
2 - 9	495.7853	989.5561	989.5546	2	0	M.VLDLDLFR.V	(Ions score 39)
2 - 9	495.7853	989.5561	989.5546	2	0	M.VLDLDLFR.V	(Ions score 35)
2 - 9	495.7853	989.5561	989.5546	2	0	M.VLDLDLFR.V	(Ions score 40)
2 - 9	495.7854	989.5562	989.5546	2	0	M.VLDLDLFR.V	(Ions score 36)
2 - 9	495.7854	989.5562	989.5546	2	0	M.VLDLDLFR.V	(Ions score 40)
2 - 9	495.7854	989.5562	989.5546	2	0	M.VLDLDLFR.V	(Ions score 39)
2 - 9	495.7854	989.5562	989.5546	2	0	M.VLDLDLFR.V	(Ions score 40)
2 - 9	495.7854	989.5562	989.5546	2	0	M.VLDLDLFR.V	(Ions score 39)
2 - 9	495.7854	989.5562	989.5546	2	0	M.VLDLDLFR.V	(Ions score 36)
2 - 9	495.7854	989.5562	989.5546	2	0	M.VLDLDLFR.V	(Ions score 36)
2 - 9	495.7854	989.5562	989.5546	2	0	M.VLDLDLFR.V	(Ions score 40)
2 - 9	495.7854	989.5562	989.5546	2	0	M.VLDLDLFR.V	(Ions score 39)
2 - 9	495.7854	989.5563	989.5546	2	0	M.VLDLDLFR.V	(Ions score 36)

2 - 9	495.7854	989.5563	989.5546	2	0	M.VLDLDLFR.V	(Ions score 40)
2 - 9	495.7854	989.5563	989.5546	2	0	M.VLDLDLFR.V	(Ions score 40)
2 - 9	495.7854	989.5563	989.5546	2	0	M.VLDLDLFR.V	(Ions score 39)
2 - 9	495.7854	989.5563	989.5546	2	0	M.VLDLDLFR.V	(Ions score 40)
2 - 9	495.7854	989.5563	989.5546	2	0	M.VLDLDLFR.V	(Ions score 36)
2 - 9	495.7854	989.5563	989.5546	2	0	M.VLDLDLFR.V	(Ions score 35)
2 - 9	495.7855	989.5564	989.5546	2	0	M.VLDLDLFR.V	(Ions score 39)
2 - 9	495.7855	989.5564	989.5546	2	0	M.VLDLDLFR.V	(Ions score 40)
2 - 9	495.7855	989.5564	989.5546	2	0	M.VLDLDLFR.V	(Ions score 25)
2 - 9	495.7855	989.5564	989.5546	2	0	M.VLDLDLFR.V	(Ions score 36)
2 - 9	495.7855	989.5564	989.5546	2	0	M.VLDLDLFR.V	(Ions score 40)
2 - 9	495.7855	989.5564	989.5546	2	0	M.VLDLDLFR.V	(Ions score 36)
2 - 9	495.7855	989.5565	989.5546	2	0	M.VLDLDLFR.V	(Ions score 39)
2 - 9	495.7855	989.5565	989.5546	2	0	M.VLDLDLFR.V	(Ions score 39)
2 - 9	495.7855	989.5565	989.5546	2	0	M.VLDLDLFR.V	(Ions score 46)
2 - 9	495.7856	989.5566	989.5546	2	0	M.VLDLDLFR.V	(Ions score 40)
2 - 9	495.7856	989.5566	989.5546	2	0	M.VLDLDLFR.V	(Ions score 40)
2 - 9	495.7856	989.5566	989.5546	2	0	M.VLDLDLFR.V	(Ions score 39)
2 - 9	495.7856	989.5566	989.5546	2	0	M.VLDLDLFR.V	(Ions score 40)
2 - 9	495.7856	989.5566	989.5546	2	0	M.VLDLDLFR.V	(Ions score 40)
2 - 9	495.7856	989.5566	989.5546	2	0	M.VLDLDLFR.V	(Ions score 35)
2 - 9	495.7856	989.5566	989.5546	2	0	M.VLDLDLFR.V	(Ions score 39)
2 - 9	495.7856	989.5566	989.5546	2	0	M.VLDLDLFR.V	(Ions score 36)
2 - 9	495.7856	989.5566	989.5546	2	0	M.VLDLDLFR.V	(Ions score 36)
2 - 9	495.7856	989.5567	989.5546	2	0	M.VLDLDLFR.V	(Ions score 41)
2 - 9	495.7856	989.5567	989.5546	2	0	M.VLDLDLFR.V	(Ions score 25)
2 - 9	495.7856	989.5567	989.5546	2	0	M.VLDLDLFR.V	(Ions score 39)
2 - 9	495.7856	989.5567	989.5546	2	0	M.VLDLDLFR.V	(Ions score 39)
2 - 9	495.7856	989.5567	989.5546	2	0	M.VLDLDLFR.V	(Ions score 42)
2 - 9	495.7856	989.5567	989.5546	2	0	M.VLDLDLFR.V	(Ions score 44)
2 - 9	495.7856	989.5567	989.5546	2	0	M.VLDLDLFR.V	(Ions score 39)
2 - 9	495.7856	989.5567	989.5546	2	0	M.VLDLDLFR.V	(Ions score 40)
2 - 9	495.7856	989.5567	989.5546	2	0	M.VLDLDLFR.V	(Ions score 39)
2 - 9	495.7857	989.5568	989.5546	2	0	M.VLDLDLFR.V	(Ions score 44)
2 - 9	495.7857	989.5568	989.5546	2	0	M.VLDLDLFR.V	(Ions score 41)
2 - 9	495.7857	989.5568	989.5546	2	0	M.VLDLDLFR.V	(Ions score 39)
2 - 9	495.7857	989.5568	989.5546	2	0	M.VLDLDLFR.V	(Ions score 36)
2 - 9	495.7857	989.5568	989.5546	2	0	M.VLDLDLFR.V	(Ions score 40)
2 - 9	495.7857	989.5568	989.5546	2	0	M.VLDLDLFR.V	(Ions score 35)
2 - 9	495.7857	989.5568	989.5546	2	0	M.VLDLDLFR.V	(Ions score 42)
2 - 9	495.7857	989.5568	989.5546	2	0	M.VLDLDLFR.V	(Ions score 40)
2 - 9	495.7857	989.5569	989.5546	2	0	M.VLDLDLFR.V	(Ions score 41)
2 - 9	495.7857	989.5569	989.5546	2	0	M.VLDLDLFR.V	(Ions score 40)
2 - 9	495.7857	989.5569	989.5546	2	0	M.VLDLDLFR.V	(Ions score 39)
2 - 9	495.7857	989.5569	989.5546	2	0	M.VLDLDLFR.V	(Ions score 40)
2 - 9	495.7857	989.5569	989.5546	2	0	M.VLDLDLFR.V	(Ions score 36)
2 - 9	495.7857	989.5569	989.5546	2	0	M.VLDLDLFR.V	(Ions score 40)
2 - 9	495.7857	989.5569	989.5546	2	0	M.VLDLDLFR.V	(Ions score 40)
2 - 9	495.7857	989.5569	989.5546	2	0	M.VLDLDLFR.V	(Ions score 39)
2 - 9	495.7857	989.5569	989.5546	2	0	M.VLDLDLFR.V	(Ions score 40)
2 - 9	495.7857	989.5569	989.5546	2	0	M.VLDLDLFR.V	(Ions score 39)
2 - 9	495.7857	989.5569	989.5546	2	0	M.VLDLDLFR.V	(Ions score 39)
2 - 9	495.7858	989.5570	989.5546	2	0	M.VLDLDLFR.V	(Ions score 41)
2 - 9	495.7858	989.5570	989.5546	2	0	M.VLDLDLFR.V	(Ions score 40)
2 - 9	495.7858	989.5570	989.5546	2	0	M.VLDLDLFR.V	(Ions score 42)
2 - 9	495.7858	989.5570	989.5546	2	0	M.VLDLDLFR.V	(Ions score 41)
2 - 9	495.7858	989.5570	989.5546	2	0	M.VLDLDLFR.V	(Ions score 40)
2 - 9	495.7858	989.5570	989.5546	2	0	M.VLDLDLFR.V	(Ions score 40)

2 - 9	495.7858	989.5570	989.5546	2	0	M.VLDLDLFR.V	(Ions score 38)
2 - 9	495.7858	989.5571	989.5546	3	0	M.VLDLDLFR.V	(Ions score 22)
2 - 9	495.7858	989.5571	989.5546	3	0	M.VLDLDLFR.V	(Ions score 40)
2 - 9	495.7858	989.5571	989.5546	3	0	M.VLDLDLFR.V	(Ions score 41)
2 - 9	495.7858	989.5571	989.5546	3	0	M.VLDLDLFR.V	(Ions score 40)
2 - 9	495.7858	989.5571	989.5546	3	0	M.VLDLDLFR.V	(Ions score 41)
2 - 9	495.7859	989.5572	989.5546	3	0	M.VLDLDLFR.V	(Ions score 40)
2 - 9	495.7859	989.5572	989.5546	3	0	M.VLDLDLFR.V	(Ions score 36)
2 - 9	495.7859	989.5572	989.5546	3	0	M.VLDLDLFR.V	(Ions score 40)
2 - 9	495.7859	989.5572	989.5546	3	0	M.VLDLDLFR.V	(Ions score 39)
2 - 9	495.7859	989.5572	989.5546	3	0	M.VLDLDLFR.V	(Ions score 42)
2 - 9	495.7859	989.5572	989.5546	3	0	M.VLDLDLFR.V	(Ions score 39)
2 - 9	495.7859	989.5572	989.5546	3	0	M.VLDLDLFR.V	(Ions score 40)
2 - 9	495.7859	989.5572	989.5546	3	0	M.VLDLDLFR.V	(Ions score 39)
2 - 9	495.7859	989.5573	989.5546	3	0	M.VLDLDLFR.V	(Ions score 44)
2 - 9	495.7859	989.5573	989.5546	3	0	M.VLDLDLFR.V	(Ions score 39)
2 - 9	495.7859	989.5573	989.5546	3	0	M.VLDLDLFR.V	(Ions score 36)
2 - 9	495.7860	989.5573	989.5546	3	0	M.VLDLDLFR.V	(Ions score 39)
2 - 9	495.7860	989.5574	989.5546	3	0	M.VLDLDLFR.V	(Ions score 42)
2 - 9	495.7860	989.5574	989.5546	3	0	M.VLDLDLFR.V	(Ions score 40)
2 - 9	495.7860	989.5574	989.5546	3	0	M.VLDLDLFR.V	(Ions score 39)
2 - 9	495.7860	989.5574	989.5546	3	0	M.VLDLDLFR.V	(Ions score 40)
2 - 9	495.7860	989.5574	989.5546	3	0	M.VLDLDLFR.V	(Ions score 25)
2 - 9	495.7860	989.5574	989.5546	3	0	M.VLDLDLFR.V	(Ions score 39)
2 - 9	495.7860	989.5574	989.5546	3	0	M.VLDLDLFR.V	(Ions score 36)
2 - 9	495.7860	989.5575	989.5546	3	0	M.VLDLDLFR.V	(Ions score 39)
2 - 9	495.7860	989.5575	989.5546	3	0	M.VLDLDLFR.V	(Ions score 35)
2 - 9	495.7860	989.5575	989.5546	3	0	M.VLDLDLFR.V	(Ions score 39)
2 - 9	495.7860	989.5575	989.5546	3	0	M.VLDLDLFR.V	(Ions score 21)
2 - 9	495.7860	989.5575	989.5546	3	0	M.VLDLDLFR.V	(Ions score 35)
2 - 9	495.7861	989.5576	989.5546	3	0	M.VLDLDLFR.V	(Ions score 42)
2 - 9	495.7861	989.5577	989.5546	3	0	M.VLDLDLFR.V	(Ions score 40)
2 - 9	495.7861	989.5577	989.5546	3	0	M.VLDLDLFR.V	(Ions score 39)
2 - 9	495.7861	989.5577	989.5546	3	0	M.VLDLDLFR.V	(Ions score 38)
2 - 9	495.7861	989.5577	989.5546	3	0	M.VLDLDLFR.V	(Ions score 37)
2 - 9	495.7861	989.5577	989.5546	3	0	M.VLDLDLFR.V	(Ions score 40)
2 - 9	495.7861	989.5577	989.5546	3	0	M.VLDLDLFR.V	(Ions score 39)
2 - 9	495.7861	989.5577	989.5546	3	0	M.VLDLDLFR.V	(Ions score 39)
2 - 9	495.7861	989.5577	989.5546	3	0	M.VLDLDLFR.V	(Ions score 43)
2 - 9	495.7861	989.5577	989.5546	3	0	M.VLDLDLFR.V	(Ions score 41)
2 - 9	495.7862	989.5578	989.5546	3	0	M.VLDLDLFR.V	(Ions score 37)
2 - 9	495.7862	989.5578	989.5546	3	0	M.VLDLDLFR.V	(Ions score 45)
2 - 9	495.7862	989.5579	989.5546	3	0	M.VLDLDLFR.V	(Ions score 40)
2 - 9	495.7863	989.5580	989.5546	3	0	M.VLDLDLFR.V	(Ions score 39)
2 - 9	495.7863	989.5580	989.5546	3	0	M.VLDLDLFR.V	(Ions score 40)
2 - 9	495.7863	989.5580	989.5546	3	0	M.VLDLDLFR.V	(Ions score 39)
2 - 9	495.7863	989.5580	989.5546	3	0	M.VLDLDLFR.V	(Ions score 39)
2 - 9	495.7863	989.5581	989.5546	4	0	M.VLDLDLFR.V	(Ions score 40)
2 - 9	495.7863	989.5581	989.5546	4	0	M.VLDLDLFR.V	(Ions score 36)
2 - 9	495.7864	989.5582	989.5546	4	0	M.VLDLDLFR.V	(Ions score 41)
2 - 9	495.7864	989.5582	989.5546	4	0	M.VLDLDLFR.V	(Ions score 37)
2 - 9	495.7864	989.5583	989.5546	4	0	M.VLDLDLFR.V	(Ions score 36)
2 - 9	495.7864	989.5583	989.5546	4	0	M.VLDLDLFR.V	(Ions score 51)
2 - 9	495.7864	989.5583	989.5546	4	0	M.VLDLDLFR.V	(Ions score 40)
2 - 9	495.7865	989.5584	989.5546	4	0	M.VLDLDLFR.V	(Ions score 40)
2 - 9	495.7865	989.5584	989.5546	4	0	M.VLDLDLFR.V	(Ions score 43)
2 - 9	495.7865	989.5584	989.5546	4	0	M.VLDLDLFR.V	(Ions score 40)
2 - 9	495.7865	989.5584	989.5546	4	0	M.VLDLDLFR.V	(Ions score 40)

2 - 9	495.7865	989.5585	989.5546	4	0	M.VLDLDLFR.V	(Ions score 40)
2 - 9	495.7865	989.5585	989.5546	4	0	M.VLDLDLFR.V	(Ions score 36)
2 - 9	495.7865	989.5585	989.5546	4	0	M.VLDLDLFR.V	(Ions score 40)
2 - 9	495.7865	989.5585	989.5546	4	0	M.VLDLDLFR.V	(Ions score 40)
2 - 9	495.7865	989.5585	989.5546	4	0	M.VLDLDLFR.V	(Ions score 40)
2 - 9	495.7866	989.5586	989.5546	4	0	M.VLDLDLFR.V	(Ions score 24)
2 - 9	495.7866	989.5586	989.5546	4	0	M.VLDLDLFR.V	(Ions score 25)
2 - 9	495.7866	989.5586	989.5546	4	0	M.VLDLDLFR.V	(Ions score 39)
2 - 9	495.7866	989.5587	989.5546	4	0	M.VLDLDLFR.V	(Ions score 41)
2 - 9	495.7866	989.5587	989.5546	4	0	M.VLDLDLFR.V	(Ions score 41)
2 - 9	495.7867	989.5588	989.5546	4	0	M.VLDLDLFR.V	(Ions score 40)
2 - 9	495.7867	989.5588	989.5546	4	0	M.VLDLDLFR.V	(Ions score 40)
2 - 9	495.7867	989.5588	989.5546	4	0	M.VLDLDLFR.V	(Ions score 40)
2 - 9	495.7867	989.5589	989.5546	4	0	M.VLDLDLFR.V	(Ions score 35)
2 - 9	495.7868	989.5590	989.5546	4	0	M.VLDLDLFR.V	(Ions score 52)
2 - 9	495.7868	989.5591	989.5546	5	0	M.VLDLDLFR.V	(Ions score 40)
2 - 12	666.8803	1331.7461	1331.7449	1	1	M.VLDLDLFRVDK.G	(Ions score 28)
2 - 12	444.9230	1331.7473	1331.7449	2	1	M.VLDLDLFRVDK.G	(Ions score 30)
2 - 12	444.9231	1331.7475	1331.7449	2	1	M.VLDLDLFRVDK.G	(Ions score 44)
2 - 12	444.9234	1331.7485	1331.7449	3	1	M.VLDLDLFRVDK.G	(Ions score 40)
10 - 20	570.8225	1139.6305	1139.6299	1	1	R.VDKGGDPALIR.E	(Ions score 22)
10 - 20	570.8230	1139.6315	1139.6299	1	1	R.VDKGGDPALIR.E	(Ions score 45)
10 - 20	570.8231	1139.6317	1139.6299	2	1	R.VDKGGDPALIR.E	(Ions score 40)
10 - 20	570.8233	1139.6320	1139.6299	2	1	R.VDKGGDPALIR.E	(Ions score 35)
10 - 20	570.8235	1139.6325	1139.6299	2	1	R.VDKGGDPALIR.E	(Ions score 33)
10 - 20	570.8235	1139.6325	1139.6299	2	1	R.VDKGGDPALIR.E	(Ions score 50)
10 - 20	570.8236	1139.6326	1139.6299	2	1	R.VDKGGDPALIR.E	(Ions score 40)
10 - 20	570.8236	1139.6327	1139.6299	2	1	R.VDKGGDPALIR.E	(Ions score 25)
10 - 20	570.8238	1139.6331	1139.6299	3	1	R.VDKGGDPALIR.E	(Ions score 50)
10 - 20	570.8241	1139.6337	1139.6299	3	1	R.VDKGGDPALIR.E	(Ions score 44)
10 - 20	570.8244	1139.6343	1139.6299	4	1	R.VDKGGDPALIR.E	(Ions score 34)
10 - 20	591.8303	1181.6460	1181.6404	5	1	R.VDKGGDPALIR.E Acetylation (K)	(Ions score 36)
13 - 25	707.3721	1412.7296	1412.7259	3	1	K.GGDPALIRETQEK.R	(Ions score 28)
27 - 38	453.5933	1357.7581	1357.7606	-2	1	R.FKDPGLVDQLVK.A	(Ions score 30)
27 - 38	679.8868	1357.7590	1357.7606	-1	1	R.FKDPGLVDQLVK.A	(Ions score 22)
27 - 38	453.5936	1357.7590	1357.7606	-1	1	R.FKDPGLVDQLVK.A	(Ions score 46)
27 - 38	453.5936	1357.7591	1357.7606	-1	1	R.FKDPGLVDQLVK.A	(Ions score 49)
27 - 38	679.8868	1357.7591	1357.7606	-1	1	R.FKDPGLVDQLVK.A	(Ions score 32)
27 - 38	453.5937	1357.7593	1357.7606	-1	1	R.FKDPGLVDQLVK.A	(Ions score 48)
27 - 38	453.5937	1357.7593	1357.7606	-1	1	R.FKDPGLVDQLVK.A	(Ions score 36)
27 - 38	453.5937	1357.7593	1357.7606	-1	1	R.FKDPGLVDQLVK.A	(Ions score 47)
27 - 38	679.8869	1357.7593	1357.7606	-1	1	R.FKDPGLVDQLVK.A	(Ions score 50)
27 - 38	679.8870	1357.7594	1357.7606	-1	1	R.FKDPGLVDQLVK.A	(Ions score 28)
27 - 38	453.5938	1357.7594	1357.7606	-1	1	R.FKDPGLVDQLVK.A	(Ions score 42)
27 - 38	453.5938	1357.7595	1357.7606	-1	1	R.FKDPGLVDQLVK.A	(Ions score 48)
27 - 38	679.8871	1357.7597	1357.7606	-1	1	R.FKDPGLVDQLVK.A	(Ions score 40)
27 - 38	453.5939	1357.7598	1357.7606	-1	1	R.FKDPGLVDQLVK.A	(Ions score 50)
27 - 38	453.5939	1357.7598	1357.7606	-1	1	R.FKDPGLVDQLVK.A	(Ions score 39)
27 - 38	453.5939	1357.7598	1357.7606	-1	1	R.FKDPGLVDQLVK.A	(Ions score 41)
27 - 38	453.5939	1357.7598	1357.7606	-1	1	R.FKDPGLVDQLVK.A	(Ions score 39)
27 - 38	453.5939	1357.7599	1357.7606	-0	1	R.FKDPGLVDQLVK.A	(Ions score 34)
27 - 38	453.5939	1357.7599	1357.7606	-0	1	R.FKDPGLVDQLVK.A	(Ions score 39)
27 - 38	453.5939	1357.7600	1357.7606	-0	1	R.FKDPGLVDQLVK.A	(Ions score 46)
27 - 38	453.5940	1357.7601	1357.7606	-0	1	R.FKDPGLVDQLVK.A	(Ions score 38)
27 - 38	453.5940	1357.7601	1357.7606	-0	1	R.FKDPGLVDQLVK.A	(Ions score 38)
27 - 38	453.5940	1357.7601	1357.7606	-0	1	R.FKDPGLVDQLVK.A	(Ions score 42)
27 - 38	453.5940	1357.7602	1357.7606	-0	1	R.FKDPGLVDQLVK.A	(Ions score 48)
27 - 38	679.8874	1357.7602	1357.7606	-0	1	R.FKDPGLVDQLVK.A	(Ions score 23)

27 - 38	453.5940	1357.7602	1357.7606	-0	1	R.FKDPGLVDQLVK.A	(Ions score 39)
27 - 38	453.5940	1357.7602	1357.7606	-0	1	R.FKDPGLVDQLVK.A	(Ions score 41)
27 - 38	453.5940	1357.7603	1357.7606	-0	1	R.FKDPGLVDQLVK.A	(Ions score 53)
27 - 38	453.5940	1357.7603	1357.7606	-0	1	R.FKDPGLVDQLVK.A	(Ions score 49)
27 - 38	453.5940	1357.7603	1357.7606	-0	1	R.FKDPGLVDQLVK.A	(Ions score 21)
27 - 38	453.5941	1357.7603	1357.7606	-0	1	R.FKDPGLVDQLVK.A	(Ions score 42)
27 - 38	453.5941	1357.7603	1357.7606	-0	1	R.FKDPGLVDQLVK.A	(Ions score 45)
27 - 38	453.5941	1357.7604	1357.7606	-0	1	R.FKDPGLVDQLVK.A	(Ions score 33)
27 - 38	453.5941	1357.7604	1357.7606	-0	1	R.FKDPGLVDQLVK.A	(Ions score 41)
27 - 38	679.8875	1357.7604	1357.7606	-0	1	R.FKDPGLVDQLVK.A	(Ions score 40)
27 - 38	453.5941	1357.7604	1357.7606	-0	1	R.FKDPGLVDQLVK.A	(Ions score 31)
27 - 38	453.5941	1357.7604	1357.7606	-0	1	R.FKDPGLVDQLVK.A	(Ions score 33)
27 - 38	453.5941	1357.7604	1357.7606	-0	1	R.FKDPGLVDQLVK.A	(Ions score 49)
27 - 38	453.5941	1357.7604	1357.7606	-0	1	R.FKDPGLVDQLVK.A	(Ions score 49)
27 - 38	453.5941	1357.7605	1357.7606	-0	1	R.FKDPGLVDQLVK.A	(Ions score 48)
27 - 38	453.5941	1357.7605	1357.7606	-0	1	R.FKDPGLVDQLVK.A	(Ions score 37)
27 - 38	453.5941	1357.7606	1357.7606	-0	1	R.FKDPGLVDQLVK.A	(Ions score 48)
27 - 38	453.5941	1357.7606	1357.7606	0	1	R.FKDPGLVDQLVK.A	(Ions score 41)
27 - 38	453.5941	1357.7606	1357.7606	0	1	R.FKDPGLVDQLVK.A	(Ions score 34)
27 - 38	453.5942	1357.7607	1357.7606	0	1	R.FKDPGLVDQLVK.A	(Ions score 26)
27 - 38	453.5942	1357.7607	1357.7606	0	1	R.FKDPGLVDQLVK.A	(Ions score 36)
27 - 38	453.5942	1357.7607	1357.7606	0	1	R.FKDPGLVDQLVK.A	(Ions score 42)
27 - 38	453.5942	1357.7607	1357.7606	0	1	R.FKDPGLVDQLVK.A	(Ions score 45)
27 - 38	453.5942	1357.7608	1357.7606	0	1	R.FKDPGLVDQLVK.A	(Ions score 46)
27 - 38	453.5942	1357.7608	1357.7606	0	1	R.FKDPGLVDQLVK.A	(Ions score 49)
27 - 38	453.5942	1357.7608	1357.7606	0	1	R.FKDPGLVDQLVK.A	(Ions score 39)
27 - 38	453.5942	1357.7609	1357.7606	0	1	R.FKDPGLVDQLVK.A	(Ions score 38)
27 - 38	453.5942	1357.7609	1357.7606	0	1	R.FKDPGLVDQLVK.A	(Ions score 49)
27 - 38	679.8877	1357.7609	1357.7606	0	1	R.FKDPGLVDQLVK.A	(Ions score 32)
27 - 38	453.5942	1357.7609	1357.7606	0	1	R.FKDPGLVDQLVK.A	(Ions score 27)
27 - 38	453.5942	1357.7609	1357.7606	0	1	R.FKDPGLVDQLVK.A	(Ions score 41)
27 - 38	453.5943	1357.7609	1357.7606	0	1	R.FKDPGLVDQLVK.A	(Ions score 51)
27 - 38	453.5943	1357.7610	1357.7606	0	1	R.FKDPGLVDQLVK.A	(Ions score 44)
27 - 38	453.5943	1357.7610	1357.7606	0	1	R.FKDPGLVDQLVK.A	(Ions score 49)
27 - 38	453.5943	1357.7610	1357.7606	0	1	R.FKDPGLVDQLVK.A	(Ions score 34)
27 - 38	453.5943	1357.7610	1357.7606	0	1	R.FKDPGLVDQLVK.A	(Ions score 40)
27 - 38	679.8878	1357.7611	1357.7606	0	1	R.FKDPGLVDQLVK.A	(Ions score 29)
27 - 38	453.5943	1357.7611	1357.7606	0	1	R.FKDPGLVDQLVK.A	(Ions score 51)
27 - 38	453.5943	1357.7611	1357.7606	0	1	R.FKDPGLVDQLVK.A	(Ions score 52)
27 - 38	453.5943	1357.7611	1357.7606	0	1	R.FKDPGLVDQLVK.A	(Ions score 22)
27 - 38	453.5943	1357.7611	1357.7606	0	1	R.FKDPGLVDQLVK.A	(Ions score 41)
27 - 38	453.5943	1357.7611	1357.7606	0	1	R.FKDPGLVDQLVK.A	(Ions score 36)
27 - 38	453.5943	1357.7612	1357.7606	0	1	R.FKDPGLVDQLVK.A	(Ions score 50)
27 - 38	679.8879	1357.7612	1357.7606	0	1	R.FKDPGLVDQLVK.A	(Ions score 30)
27 - 38	453.5943	1357.7612	1357.7606	0	1	R.FKDPGLVDQLVK.A	(Ions score 35)
27 - 38	453.5943	1357.7612	1357.7606	0	1	R.FKDPGLVDQLVK.A	(Ions score 50)
27 - 38	679.8879	1357.7612	1357.7606	0	1	R.FKDPGLVDQLVK.A	(Ions score 29)
27 - 38	453.5943	1357.7612	1357.7606	0	1	R.FKDPGLVDQLVK.A	(Ions score 49)
27 - 38	453.5943	1357.7612	1357.7606	0	1	R.FKDPGLVDQLVK.A	(Ions score 29)
27 - 38	453.5944	1357.7612	1357.7606	1	1	R.FKDPGLVDQLVK.A	(Ions score 40)
27 - 38	453.5944	1357.7613	1357.7606	1	1	R.FKDPGLVDQLVK.A	(Ions score 35)
27 - 38	453.5944	1357.7613	1357.7606	1	1	R.FKDPGLVDQLVK.A	(Ions score 29)
27 - 38	679.8879	1357.7613	1357.7606	1	1	R.FKDPGLVDQLVK.A	(Ions score 23)
27 - 38	453.5944	1357.7613	1357.7606	1	1	R.FKDPGLVDQLVK.A	(Ions score 39)
27 - 38	453.5944	1357.7613	1357.7606	1	1	R.FKDPGLVDQLVK.A	(Ions score 35)
27 - 38	453.5944	1357.7613	1357.7606	1	1	R.FKDPGLVDQLVK.A	(Ions score 40)
27 - 38	453.5944	1357.7614	1357.7606	1	1	R.FKDPGLVDQLVK.A	(Ions score 47)
27 - 38	453.5944	1357.7614	1357.7606	1	1	R.FKDPGLVDQLVK.A	(Ions score 51)

27 - 38	453.5944	1357.7614	1357.7606	1	1	R.FKDPGLVDQLVK.A	(Ions score 45)
27 - 38	679.8880	1357.7614	1357.7606	1	1	R.FKDPGLVDQLVK.A	(Ions score 28)
27 - 38	453.5944	1357.7614	1357.7606	1	1	R.FKDPGLVDQLVK.A	(Ions score 48)
27 - 38	453.5944	1357.7614	1357.7606	1	1	R.FKDPGLVDQLVK.A	(Ions score 44)
27 - 38	453.5944	1357.7615	1357.7606	1	1	R.FKDPGLVDQLVK.A	(Ions score 52)
27 - 38	453.5944	1357.7615	1357.7606	1	1	R.FKDPGLVDQLVK.A	(Ions score 39)
27 - 38	453.5944	1357.7615	1357.7606	1	1	R.FKDPGLVDQLVK.A	(Ions score 32)
27 - 38	679.8880	1357.7615	1357.7606	1	1	R.FKDPGLVDQLVK.A	(Ions score 34)
27 - 38	453.5944	1357.7615	1357.7606	1	1	R.FKDPGLVDQLVK.A	(Ions score 39)
27 - 38	453.5944	1357.7615	1357.7606	1	1	R.FKDPGLVDQLVK.A	(Ions score 35)
27 - 38	453.5945	1357.7615	1357.7606	1	1	R.FKDPGLVDQLVK.A	(Ions score 52)
27 - 38	453.5945	1357.7615	1357.7606	1	1	R.FKDPGLVDQLVK.A	(Ions score 48)
27 - 38	453.5945	1357.7616	1357.7606	1	1	R.FKDPGLVDQLVK.A	(Ions score 38)
27 - 38	453.5945	1357.7616	1357.7606	1	1	R.FKDPGLVDQLVK.A	(Ions score 50)
27 - 38	453.5945	1357.7616	1357.7606	1	1	R.FKDPGLVDQLVK.A	(Ions score 34)
27 - 38	453.5945	1357.7616	1357.7606	1	1	R.FKDPGLVDQLVK.A	(Ions score 29)
27 - 38	453.5945	1357.7616	1357.7606	1	1	R.FKDPGLVDQLVK.A	(Ions score 51)
27 - 38	679.8881	1357.7616	1357.7606	1	1	R.FKDPGLVDQLVK.A	(Ions score 39)
27 - 38	453.5945	1357.7616	1357.7606	1	1	R.FKDPGLVDQLVK.A	(Ions score 40)
27 - 38	453.5945	1357.7616	1357.7606	1	1	R.FKDPGLVDQLVK.A	(Ions score 25)
27 - 38	453.5945	1357.7616	1357.7606	1	1	R.FKDPGLVDQLVK.A	(Ions score 48)
27 - 38	1358.7689	1357.7616	1357.7606	1	1	R.FKDPGLVDQLVK.A	(Ions score 42)
27 - 38	453.5945	1357.7617	1357.7606	1	1	R.FKDPGLVDQLVK.A	(Ions score 42)
27 - 38	679.8881	1357.7617	1357.7606	1	1	R.FKDPGLVDQLVK.A	(Ions score 40)
27 - 38	453.5945	1357.7617	1357.7606	1	1	R.FKDPGLVDQLVK.A	(Ions score 48)
27 - 38	453.5945	1357.7617	1357.7606	1	1	R.FKDPGLVDQLVK.A	(Ions score 40)
27 - 38	453.5945	1357.7618	1357.7606	1	1	R.FKDPGLVDQLVK.A	(Ions score 40)
27 - 38	453.5945	1357.7618	1357.7606	1	1	R.FKDPGLVDQLVK.A	(Ions score 43)
27 - 38	679.8882	1357.7618	1357.7606	1	1	R.FKDPGLVDQLVK.A	(Ions score 28)
27 - 38	679.8882	1357.7618	1357.7606	1	1	R.FKDPGLVDQLVK.A	(Ions score 29)
27 - 38	453.5945	1357.7618	1357.7606	1	1	R.FKDPGLVDQLVK.A	(Ions score 41)
27 - 38	679.8882	1357.7618	1357.7606	1	1	R.FKDPGLVDQLVK.A	(Ions score 29)
27 - 38	453.5945	1357.7618	1357.7606	1	1	R.FKDPGLVDQLVK.A	(Ions score 33)
27 - 38	453.5945	1357.7618	1357.7606	1	1	R.FKDPGLVDQLVK.A	(Ions score 46)
27 - 38	453.5945	1357.7618	1357.7606	1	1	R.FKDPGLVDQLVK.A	(Ions score 47)
27 - 38	453.5945	1357.7618	1357.7606	1	1	R.FKDPGLVDQLVK.A	(Ions score 47)
27 - 38	679.8882	1357.7618	1357.7606	1	1	R.FKDPGLVDQLVK.A	(Ions score 34)
27 - 38	453.5945	1357.7618	1357.7606	1	1	R.FKDPGLVDQLVK.A	(Ions score 49)
27 - 38	453.5945	1357.7618	1357.7606	1	1	R.FKDPGLVDQLVK.A	(Ions score 38)
27 - 38	453.5946	1357.7618	1357.7606	1	1	R.FKDPGLVDQLVK.A	(Ions score 50)
27 - 38	453.5946	1357.7619	1357.7606	1	1	R.FKDPGLVDQLVK.A	(Ions score 40)
27 - 38	453.5946	1357.7619	1357.7606	1	1	R.FKDPGLVDQLVK.A	(Ions score 33)
27 - 38	453.5946	1357.7619	1357.7606	1	1	R.FKDPGLVDQLVK.A	(Ions score 37)
27 - 38	679.8882	1357.7619	1357.7606	1	1	R.FKDPGLVDQLVK.A	(Ions score 29)
27 - 38	453.5946	1357.7619	1357.7606	1	1	R.FKDPGLVDQLVK.A	(Ions score 35)
27 - 38	453.5946	1357.7620	1357.7606	1	1	R.FKDPGLVDQLVK.A	(Ions score 52)
27 - 38	453.5946	1357.7620	1357.7606	1	1	R.FKDPGLVDQLVK.A	(Ions score 36)
27 - 38	453.5946	1357.7620	1357.7606	1	1	R.FKDPGLVDQLVK.A	(Ions score 49)
27 - 38	453.5946	1357.7620	1357.7606	1	1	R.FKDPGLVDQLVK.A	(Ions score 23)
27 - 38	453.5946	1357.7620	1357.7606	1	1	R.FKDPGLVDQLVK.A	(Ions score 44)
27 - 38	453.5946	1357.7620	1357.7606	1	1	R.FKDPGLVDQLVK.A	(Ions score 40)
27 - 38	453.5946	1357.7620	1357.7606	1	1	R.FKDPGLVDQLVK.A	(Ions score 38)
27 - 38	453.5946	1357.7620	1357.7606	1	1	R.FKDPGLVDQLVK.A	(Ions score 32)
27 - 38	453.5946	1357.7620	1357.7606	1	1	R.FKDPGLVDQLVK.A	(Ions score 40)
27 - 38	453.5946	1357.7620	1357.7606	1	1	R.FKDPGLVDQLVK.A	(Ions score 40)
27 - 38	453.5946	1357.7620	1357.7606	1	1	R.FKDPGLVDQLVK.A	(Ions score 32)
27 - 38	679.8883	1357.7620	1357.7606	1	1	R.FKDPGLVDQLVK.A	(Ions score 31)
27 - 38	453.5946	1357.7620	1357.7606	1	1	R.FKDPGLVDQLVK.A	(Ions score 37)

27 - 38	679.8883	1357.7620	1357.7606	1	1	R.FKDPGLVDQLVK.A	(Ions score 36)
27 - 38	453.5946	1357.7621	1357.7606	1	1	R.FKDPGLVDQLVK.A	(Ions score 33)
27 - 38	453.5946	1357.7621	1357.7606	1	1	R.FKDPGLVDQLVK.A	(Ions score 48)
27 - 38	453.5946	1357.7621	1357.7606	1	1	R.FKDPGLVDQLVK.A	(Ions score 40)
27 - 38	453.5946	1357.7621	1357.7606	1	1	R.FKDPGLVDQLVK.A	(Ions score 32)
27 - 38	453.5947	1357.7621	1357.7606	1	1	R.FKDPGLVDQLVK.A	(Ions score 30)
27 - 38	679.8883	1357.7621	1357.7606	1	1	R.FKDPGLVDQLVK.A	(Ions score 34)
27 - 38	453.5947	1357.7622	1357.7606	1	1	R.FKDPGLVDQLVK.A	(Ions score 32)
27 - 38	453.5947	1357.7622	1357.7606	1	1	R.FKDPGLVDQLVK.A	(Ions score 39)
27 - 38	453.5947	1357.7622	1357.7606	1	1	R.FKDPGLVDQLVK.A	(Ions score 43)
27 - 38	453.5947	1357.7622	1357.7606	1	1	R.FKDPGLVDQLVK.A	(Ions score 46)
27 - 38	453.5947	1357.7622	1357.7606	1	1	R.FKDPGLVDQLVK.A	(Ions score 39)
27 - 38	453.5947	1357.7622	1357.7606	1	1	R.FKDPGLVDQLVK.A	(Ions score 47)
27 - 38	453.5947	1357.7622	1357.7606	1	1	R.FKDPGLVDQLVK.A	(Ions score 30)
27 - 38	453.5947	1357.7623	1357.7606	1	1	R.FKDPGLVDQLVK.A	(Ions score 40)
27 - 38	453.5947	1357.7623	1357.7606	1	1	R.FKDPGLVDQLVK.A	(Ions score 33)
27 - 38	453.5947	1357.7623	1357.7606	1	1	R.FKDPGLVDQLVK.A	(Ions score 39)
27 - 38	453.5947	1357.7623	1357.7606	1	1	R.FKDPGLVDQLVK.A	(Ions score 40)
27 - 38	453.5947	1357.7624	1357.7606	1	1	R.FKDPGLVDQLVK.A	(Ions score 36)
27 - 38	453.5947	1357.7624	1357.7606	1	1	R.FKDPGLVDQLVK.A	(Ions score 46)
27 - 38	453.5947	1357.7624	1357.7606	1	1	R.FKDPGLVDQLVK.A	(Ions score 41)
27 - 38	453.5947	1357.7624	1357.7606	1	1	R.FKDPGLVDQLVK.A	(Ions score 57)
27 - 38	453.5947	1357.7624	1357.7606	1	1	R.FKDPGLVDQLVK.A	(Ions score 29)
27 - 38	453.5948	1357.7624	1357.7606	1	1	R.FKDPGLVDQLVK.A	(Ions score 28)
27 - 38	679.8885	1357.7625	1357.7606	1	1	R.FKDPGLVDQLVK.A	(Ions score 32)
27 - 38	453.5948	1357.7625	1357.7606	1	1	R.FKDPGLVDQLVK.A	(Ions score 40)
27 - 38	453.5948	1357.7625	1357.7606	1	1	R.FKDPGLVDQLVK.A	(Ions score 39)
27 - 38	453.5948	1357.7625	1357.7606	1	1	R.FKDPGLVDQLVK.A	(Ions score 53)
27 - 38	679.8886	1357.7626	1357.7606	1	1	R.FKDPGLVDQLVK.A	(Ions score 33)
27 - 38	453.5948	1357.7626	1357.7606	1	1	R.FKDPGLVDQLVK.A	(Ions score 31)
27 - 38	453.5948	1357.7626	1357.7606	1	1	R.FKDPGLVDQLVK.A	(Ions score 38)
27 - 38	453.5948	1357.7626	1357.7606	2	1	R.FKDPGLVDQLVK.A	(Ions score 40)
27 - 38	679.8886	1357.7626	1357.7606	2	1	R.FKDPGLVDQLVK.A	(Ions score 31)
27 - 38	453.5948	1357.7626	1357.7606	2	1	R.FKDPGLVDQLVK.A	(Ions score 37)
27 - 38	453.5948	1357.7627	1357.7606	2	1	R.FKDPGLVDQLVK.A	(Ions score 33)
27 - 38	453.5948	1357.7627	1357.7606	2	1	R.FKDPGLVDQLVK.A	(Ions score 48)
27 - 38	453.5948	1357.7627	1357.7606	2	1	R.FKDPGLVDQLVK.A	(Ions score 32)
27 - 38	679.8886	1357.7627	1357.7606	2	1	R.FKDPGLVDQLVK.A	(Ions score 40)
27 - 38	453.5949	1357.7628	1357.7606	2	1	R.FKDPGLVDQLVK.A	(Ions score 48)
27 - 38	453.5949	1357.7628	1357.7606	2	1	R.FKDPGLVDQLVK.A	(Ions score 50)
27 - 38	453.5949	1357.7629	1357.7606	2	1	R.FKDPGLVDQLVK.A	(Ions score 47)
27 - 38	679.8887	1357.7629	1357.7606	2	1	R.FKDPGLVDQLVK.A	(Ions score 34)
27 - 38	453.5949	1357.7630	1357.7606	2	1	R.FKDPGLVDQLVK.A	(Ions score 50)
27 - 38	453.5949	1357.7630	1357.7606	2	1	R.FKDPGLVDQLVK.A	(Ions score 48)
27 - 38	679.8888	1357.7630	1357.7606	2	1	R.FKDPGLVDQLVK.A	(Ions score 39)
27 - 38	453.5949	1357.7630	1357.7606	2	1	R.FKDPGLVDQLVK.A	(Ions score 36)
27 - 38	453.5949	1357.7630	1357.7606	2	1	R.FKDPGLVDQLVK.A	(Ions score 49)
27 - 38	679.8888	1357.7630	1357.7606	2	1	R.FKDPGLVDQLVK.A	(Ions score 35)
27 - 38	453.5950	1357.7630	1357.7606	2	1	R.FKDPGLVDQLVK.A	(Ions score 37)
27 - 38	453.5950	1357.7630	1357.7606	2	1	R.FKDPGLVDQLVK.A	(Ions score 41)
27 - 38	453.5950	1357.7631	1357.7606	2	1	R.FKDPGLVDQLVK.A	(Ions score 41)
27 - 38	453.5950	1357.7631	1357.7606	2	1	R.FKDPGLVDQLVK.A	(Ions score 27)
27 - 38	453.5950	1357.7631	1357.7606	2	1	R.FKDPGLVDQLVK.A	(Ions score 32)
27 - 38	453.5950	1357.7631	1357.7606	2	1	R.FKDPGLVDQLVK.A	(Ions score 52)
27 - 38	453.5950	1357.7631	1357.7606	2	1	R.FKDPGLVDQLVK.A	(Ions score 31)
27 - 38	679.8888	1357.7631	1357.7606	2	1	R.FKDPGLVDQLVK.A	(Ions score 25)
27 - 38	453.5950	1357.7631	1357.7606	2	1	R.FKDPGLVDQLVK.A	(Ions score 42)
27 - 38	679.8888	1357.7631	1357.7606	2	1	R.FKDPGLVDQLVK.A	(Ions score 23)

27 - 38	679.8889	1357.7632	1357.7606	2	1	R.FKDPGLVDQLVK.A	(Ions score 24)
27 - 38	453.5950	1357.7632	1357.7606	2	1	R.FKDPGLVDQLVK.A	(Ions score 50)
27 - 38	453.5950	1357.7632	1357.7606	2	1	R.FKDPGLVDQLVK.A	(Ions score 50)
27 - 38	453.5950	1357.7632	1357.7606	2	1	R.FKDPGLVDQLVK.A	(Ions score 36)
27 - 38	453.5950	1357.7632	1357.7606	2	1	R.FKDPGLVDQLVK.A	(Ions score 50)
27 - 38	453.5950	1357.7632	1357.7606	2	1	R.FKDPGLVDQLVK.A	(Ions score 29)
27 - 38	453.5950	1357.7632	1357.7606	2	1	R.FKDPGLVDQLVK.A	(Ions score 45)
27 - 38	453.5950	1357.7632	1357.7606	2	1	R.FKDPGLVDQLVK.A	(Ions score 38)
27 - 38	679.8889	1357.7632	1357.7606	2	1	R.FKDPGLVDQLVK.A	(Ions score 21)
27 - 38	453.5950	1357.7632	1357.7606	2	1	R.FKDPGLVDQLVK.A	(Ions score 48)
27 - 38	453.5950	1357.7632	1357.7606	2	1	R.FKDPGLVDQLVK.A	(Ions score 29)
27 - 38	453.5950	1357.7632	1357.7606	2	1	R.FKDPGLVDQLVK.A	(Ions score 48)
27 - 38	453.5950	1357.7633	1357.7606	2	1	R.FKDPGLVDQLVK.A	(Ions score 37)
27 - 38	453.5950	1357.7633	1357.7606	2	1	R.FKDPGLVDQLVK.A	(Ions score 36)
27 - 38	453.5950	1357.7633	1357.7606	2	1	R.FKDPGLVDQLVK.A	(Ions score 40)
27 - 38	453.5950	1357.7633	1357.7606	2	1	R.FKDPGLVDQLVK.A	(Ions score 38)
27 - 38	453.5950	1357.7633	1357.7606	2	1	R.FKDPGLVDQLVK.A	(Ions score 36)
27 - 38	453.5950	1357.7633	1357.7606	2	1	R.FKDPGLVDQLVK.A	(Ions score 56)
27 - 38	453.5950	1357.7633	1357.7606	2	1	R.FKDPGLVDQLVK.A	(Ions score 51)
27 - 38	453.5951	1357.7633	1357.7606	2	1	R.FKDPGLVDQLVK.A	(Ions score 38)
27 - 38	453.5951	1357.7634	1357.7606	2	1	R.FKDPGLVDQLVK.A	(Ions score 29)
27 - 38	453.5951	1357.7634	1357.7606	2	1	R.FKDPGLVDQLVK.A	(Ions score 33)
27 - 38	453.5951	1357.7634	1357.7606	2	1	R.FKDPGLVDQLVK.A	(Ions score 30)
27 - 38	453.5951	1357.7634	1357.7606	2	1	R.FKDPGLVDQLVK.A	(Ions score 30)
27 - 38	453.5951	1357.7634	1357.7606	2	1	R.FKDPGLVDQLVK.A	(Ions score 41)
27 - 38	453.5951	1357.7634	1357.7606	2	1	R.FKDPGLVDQLVK.A	(Ions score 39)
27 - 38	453.5951	1357.7634	1357.7606	2	1	R.FKDPGLVDQLVK.A	(Ions score 41)
27 - 38	453.5951	1357.7634	1357.7606	2	1	R.FKDPGLVDQLVK.A	(Ions score 29)
27 - 38	453.5951	1357.7634	1357.7606	2	1	R.FKDPGLVDQLVK.A	(Ions score 46)
27 - 38	679.8890	1357.7634	1357.7606	2	1	R.FKDPGLVDQLVK.A	(Ions score 29)
27 - 38	453.5951	1357.7634	1357.7606	2	1	R.FKDPGLVDQLVK.A	(Ions score 41)
27 - 38	453.5951	1357.7634	1357.7606	2	1	R.FKDPGLVDQLVK.A	(Ions score 29)
27 - 38	453.5951	1357.7635	1357.7606	2	1	R.FKDPGLVDQLVK.A	(Ions score 26)
27 - 38	453.5951	1357.7635	1357.7606	2	1	R.FKDPGLVDQLVK.A	(Ions score 35)
27 - 38	453.5951	1357.7635	1357.7606	2	1	R.FKDPGLVDQLVK.A	(Ions score 34)
27 - 38	453.5951	1357.7635	1357.7606	2	1	R.FKDPGLVDQLVK.A	(Ions score 35)
27 - 38	453.5951	1357.7635	1357.7606	2	1	R.FKDPGLVDQLVK.A	(Ions score 30)
27 - 38	679.8890	1357.7635	1357.7606	2	1	R.FKDPGLVDQLVK.A	(Ions score 29)
27 - 38	453.5951	1357.7635	1357.7606	2	1	R.FKDPGLVDQLVK.A	(Ions score 40)
27 - 38	453.5951	1357.7635	1357.7606	2	1	R.FKDPGLVDQLVK.A	(Ions score 51)
27 - 38	453.5951	1357.7636	1357.7606	2	1	R.FKDPGLVDQLVK.A	(Ions score 29)
27 - 38	453.5951	1357.7636	1357.7606	2	1	R.FKDPGLVDQLVK.A	(Ions score 39)
27 - 38	453.5951	1357.7636	1357.7606	2	1	R.FKDPGLVDQLVK.A	(Ions score 25)
27 - 38	453.5951	1357.7636	1357.7606	2	1	R.FKDPGLVDQLVK.A	(Ions score 50)
27 - 38	453.5951	1357.7636	1357.7606	2	1	R.FKDPGLVDQLVK.A	(Ions score 29)
27 - 38	453.5951	1357.7636	1357.7606	2	1	R.FKDPGLVDQLVK.A	(Ions score 40)
27 - 38	453.5952	1357.7636	1357.7606	2	1	R.FKDPGLVDQLVK.A	(Ions score 40)
27 - 38	453.5952	1357.7636	1357.7606	2	1	R.FKDPGLVDQLVK.A	(Ions score 38)
27 - 38	453.5952	1357.7636	1357.7606	2	1	R.FKDPGLVDQLVK.A	(Ions score 50)
27 - 38	453.5952	1357.7637	1357.7606	2	1	R.FKDPGLVDQLVK.A	(Ions score 36)
27 - 38	453.5952	1357.7637	1357.7606	2	1	R.FKDPGLVDQLVK.A	(Ions score 33)
27 - 38	679.8891	1357.7637	1357.7606	2	1	R.FKDPGLVDQLVK.A	(Ions score 36)
27 - 38	453.5952	1357.7637	1357.7606	2	1	R.FKDPGLVDQLVK.A	(Ions score 38)
27 - 38	453.5952	1357.7637	1357.7606	2	1	R.FKDPGLVDQLVK.A	(Ions score 52)
27 - 38	453.5952	1357.7637	1357.7606	2	1	R.FKDPGLVDQLVK.A	(Ions score 28)
27 - 38	453.5952	1357.7637	1357.7606	2	1	R.FKDPGLVDQLVK.A	(Ions score 29)
27 - 38	679.8891	1357.7637	1357.7606	2	1	R.FKDPGLVDQLVK.A	(Ions score 39)
27 - 38	679.8891	1357.7637	1357.7606	2	1	R.FKDPGLVDQLVK.A	(Ions score 22)

27 - 38	453.5952	1357.7638	1357.7606	2	1	R.FKDPGLVDQLVK.A	(Ions score 39)
27 - 38	453.5952	1357.7638	1357.7606	2	1	R.FKDPGLVDQLVK.A	(Ions score 28)
27 - 38	453.5952	1357.7638	1357.7606	2	1	R.FKDPGLVDQLVK.A	(Ions score 28)
27 - 38	453.5952	1357.7638	1357.7606	2	1	R.FKDPGLVDQLVK.A	(Ions score 38)
27 - 38	679.8892	1357.7638	1357.7606	2	1	R.FKDPGLVDQLVK.A	(Ions score 29)
27 - 38	453.5952	1357.7638	1357.7606	2	1	R.FKDPGLVDQLVK.A	(Ions score 41)
27 - 38	453.5952	1357.7638	1357.7606	2	1	R.FKDPGLVDQLVK.A	(Ions score 32)
27 - 38	453.5952	1357.7639	1357.7606	2	1	R.FKDPGLVDQLVK.A	(Ions score 29)
27 - 38	453.5952	1357.7639	1357.7606	2	1	R.FKDPGLVDQLVK.A	(Ions score 32)
27 - 38	453.5952	1357.7639	1357.7606	2	1	R.FKDPGLVDQLVK.A	(Ions score 38)
27 - 38	453.5952	1357.7639	1357.7606	2	1	R.FKDPGLVDQLVK.A	(Ions score 44)
27 - 38	453.5953	1357.7639	1357.7606	2	1	R.FKDPGLVDQLVK.A	(Ions score 35)
27 - 38	453.5953	1357.7640	1357.7606	3	1	R.FKDPGLVDQLVK.A	(Ions score 36)
27 - 38	453.5953	1357.7640	1357.7606	3	1	R.FKDPGLVDQLVK.A	(Ions score 40)
27 - 38	679.8893	1357.7640	1357.7606	3	1	R.FKDPGLVDQLVK.A	(Ions score 56)
27 - 38	453.5953	1357.7640	1357.7606	3	1	R.FKDPGLVDQLVK.A	(Ions score 49)
27 - 38	453.5953	1357.7640	1357.7606	3	1	R.FKDPGLVDQLVK.A	(Ions score 39)
27 - 38	453.5953	1357.7641	1357.7606	3	1	R.FKDPGLVDQLVK.A	(Ions score 51)
27 - 38	679.8893	1357.7641	1357.7606	3	1	R.FKDPGLVDQLVK.A	(Ions score 27)
27 - 38	453.5953	1357.7641	1357.7606	3	1	R.FKDPGLVDQLVK.A	(Ions score 34)
27 - 38	453.5953	1357.7641	1357.7606	3	1	R.FKDPGLVDQLVK.A	(Ions score 51)
27 - 38	453.5953	1357.7641	1357.7606	3	1	R.FKDPGLVDQLVK.A	(Ions score 31)
27 - 38	453.5953	1357.7641	1357.7606	3	1	R.FKDPGLVDQLVK.A	(Ions score 32)
27 - 38	453.5953	1357.7641	1357.7606	3	1	R.FKDPGLVDQLVK.A	(Ions score 26)
27 - 38	453.5953	1357.7641	1357.7606	3	1	R.FKDPGLVDQLVK.A	(Ions score 30)
27 - 38	453.5953	1357.7641	1357.7606	3	1	R.FKDPGLVDQLVK.A	(Ions score 34)
27 - 38	679.8894	1357.7642	1357.7606	3	1	R.FKDPGLVDQLVK.A	(Ions score 27)
27 - 38	453.5953	1357.7642	1357.7606	3	1	R.FKDPGLVDQLVK.A	(Ions score 50)
27 - 38	453.5953	1357.7642	1357.7606	3	1	R.FKDPGLVDQLVK.A	(Ions score 29)
27 - 38	679.8894	1357.7642	1357.7606	3	1	R.FKDPGLVDQLVK.A	(Ions score 28)
27 - 38	453.5954	1357.7643	1357.7606	3	1	R.FKDPGLVDQLVK.A	(Ions score 40)
27 - 38	453.5954	1357.7643	1357.7606	3	1	R.FKDPGLVDQLVK.A	(Ions score 50)
27 - 38	453.5954	1357.7643	1357.7606	3	1	R.FKDPGLVDQLVK.A	(Ions score 42)
27 - 38	453.5954	1357.7643	1357.7606	3	1	R.FKDPGLVDQLVK.A	(Ions score 32)
27 - 38	679.8894	1357.7643	1357.7606	3	1	R.FKDPGLVDQLVK.A	(Ions score 29)
27 - 38	453.5954	1357.7643	1357.7606	3	1	R.FKDPGLVDQLVK.A	(Ions score 32)
27 - 38	453.5954	1357.7643	1357.7606	3	1	R.FKDPGLVDQLVK.A	(Ions score 38)
27 - 38	453.5954	1357.7643	1357.7606	3	1	R.FKDPGLVDQLVK.A	(Ions score 39)
27 - 38	679.8895	1357.7644	1357.7606	3	1	R.FKDPGLVDQLVK.A	(Ions score 26)
27 - 38	453.5954	1357.7644	1357.7606	3	1	R.FKDPGLVDQLVK.A	(Ions score 51)
27 - 38	453.5954	1357.7645	1357.7606	3	1	R.FKDPGLVDQLVK.A	(Ions score 37)
27 - 38	453.5954	1357.7645	1357.7606	3	1	R.FKDPGLVDQLVK.A	(Ions score 36)
27 - 38	679.8895	1357.7645	1357.7606	3	1	R.FKDPGLVDQLVK.A	(Ions score 39)
27 - 38	453.5954	1357.7645	1357.7606	3	1	R.FKDPGLVDQLVK.A	(Ions score 32)
27 - 38	679.8895	1357.7645	1357.7606	3	1	R.FKDPGLVDQLVK.A	(Ions score 35)
27 - 38	679.8895	1357.7645	1357.7606	3	1	R.FKDPGLVDQLVK.A	(Ions score 44)
27 - 38	453.5955	1357.7646	1357.7606	3	1	R.FKDPGLVDQLVK.A	(Ions score 43)
27 - 38	679.8896	1357.7646	1357.7606	3	1	R.FKDPGLVDQLVK.A	(Ions score 44)
27 - 38	453.5955	1357.7646	1357.7606	3	1	R.FKDPGLVDQLVK.A	(Ions score 47)
27 - 38	453.5955	1357.7647	1357.7606	3	1	R.FKDPGLVDQLVK.A	(Ions score 36)
27 - 38	679.8896	1357.7647	1357.7606	3	1	R.FKDPGLVDQLVK.A	(Ions score 30)
27 - 38	453.5955	1357.7647	1357.7606	3	1	R.FKDPGLVDQLVK.A	(Ions score 30)
27 - 38	679.8896	1357.7647	1357.7606	3	1	R.FKDPGLVDQLVK.A	(Ions score 31)
27 - 38	679.8896	1357.7647	1357.7606	3	1	R.FKDPGLVDQLVK.A	(Ions score 32)
27 - 38	453.5955	1357.7647	1357.7606	3	1	R.FKDPGLVDQLVK.A	(Ions score 50)
27 - 38	679.8896	1357.7647	1357.7606	3	1	R.FKDPGLVDQLVK.A	(Ions score 30)
27 - 38	453.5955	1357.7648	1357.7606	3	1	R.FKDPGLVDQLVK.A	(Ions score 41)
27 - 38	453.5955	1357.7648	1357.7606	3	1	R.FKDPGLVDQLVK.A	(Ions score 50)

27 - 38	679.8897	1357.7648	1357.7606	3	1	R.FKDPGLVDQLVK.A	(Ions score 36)
27 - 38	453.5956	1357.7648	1357.7606	3	1	R.FKDPGLVDQLVK.A	(Ions score 40)
27 - 38	453.5956	1357.7648	1357.7606	3	1	R.FKDPGLVDQLVK.A	(Ions score 27)
27 - 38	679.8897	1357.7649	1357.7606	3	1	R.FKDPGLVDQLVK.A	(Ions score 32)
27 - 38	453.5956	1357.7649	1357.7606	3	1	R.FKDPGLVDQLVK.A	(Ions score 23)
27 - 38	453.5956	1357.7649	1357.7606	3	1	R.FKDPGLVDQLVK.A	(Ions score 26)
27 - 38	679.8897	1357.7649	1357.7606	3	1	R.FKDPGLVDQLVK.A	(Ions score 25)
27 - 38	679.8898	1357.7649	1357.7606	3	1	R.FKDPGLVDQLVK.A	(Ions score 34)
27 - 38	453.5956	1357.7650	1357.7606	3	1	R.FKDPGLVDQLVK.A	(Ions score 32)
27 - 38	679.8898	1357.7650	1357.7606	3	1	R.FKDPGLVDQLVK.A	(Ions score 21)
27 - 38	679.8898	1357.7651	1357.7606	3	1	R.FKDPGLVDQLVK.A	(Ions score 25)
27 - 38	453.5956	1357.7651	1357.7606	3	1	R.FKDPGLVDQLVK.A	(Ions score 37)
27 - 38	453.5956	1357.7651	1357.7606	3	1	R.FKDPGLVDQLVK.A	(Ions score 39)
27 - 38	453.5957	1357.7651	1357.7606	3	1	R.FKDPGLVDQLVK.A	(Ions score 36)
27 - 38	453.5957	1357.7651	1357.7606	3	1	R.FKDPGLVDQLVK.A	(Ions score 35)
27 - 38	453.5957	1357.7652	1357.7606	3	1	R.FKDPGLVDQLVK.A	(Ions score 40)
27 - 38	453.5957	1357.7652	1357.7606	3	1	R.FKDPGLVDQLVK.A	(Ions score 34)
27 - 38	453.5957	1357.7652	1357.7606	3	1	R.FKDPGLVDQLVK.A	(Ions score 39)
27 - 38	679.8899	1357.7652	1357.7606	3	1	R.FKDPGLVDQLVK.A	(Ions score 45)
27 - 38	453.5957	1357.7652	1357.7606	3	1	R.FKDPGLVDQLVK.A	(Ions score 38)
27 - 38	453.5957	1357.7652	1357.7606	3	1	R.FKDPGLVDQLVK.A	(Ions score 48)
27 - 38	453.5957	1357.7652	1357.7606	3	1	R.FKDPGLVDQLVK.A	(Ions score 43)
27 - 38	679.8899	1357.7652	1357.7606	3	1	R.FKDPGLVDQLVK.A	(Ions score 53)
27 - 38	453.5957	1357.7652	1357.7606	3	1	R.FKDPGLVDQLVK.A	(Ions score 23)
27 - 38	453.5957	1357.7652	1357.7606	3	1	R.FKDPGLVDQLVK.A	(Ions score 29)
27 - 38	679.8899	1357.7652	1357.7606	3	1	R.FKDPGLVDQLVK.A	(Ions score 37)
27 - 38	679.8899	1357.7653	1357.7606	3	1	R.FKDPGLVDQLVK.A	(Ions score 23)
27 - 38	679.8899	1357.7653	1357.7606	3	1	R.FKDPGLVDQLVK.A	(Ions score 57)
27 - 38	453.5957	1357.7653	1357.7606	3	1	R.FKDPGLVDQLVK.A	(Ions score 32)
27 - 38	453.5957	1357.7653	1357.7606	3	1	R.FKDPGLVDQLVK.A	(Ions score 35)
27 - 38	453.5957	1357.7653	1357.7606	4	1	R.FKDPGLVDQLVK.A	(Ions score 23)
27 - 38	679.8899	1357.7653	1357.7606	4	1	R.FKDPGLVDQLVK.A	(Ions score 34)
27 - 38	679.8899	1357.7653	1357.7606	4	1	R.FKDPGLVDQLVK.A	(Ions score 28)
27 - 38	679.8900	1357.7654	1357.7606	4	1	R.FKDPGLVDQLVK.A	(Ions score 30)
27 - 38	453.5957	1357.7654	1357.7606	4	1	R.FKDPGLVDQLVK.A	(Ions score 43)
27 - 38	453.5958	1357.7654	1357.7606	4	1	R.FKDPGLVDQLVK.A	(Ions score 38)
27 - 38	453.5958	1357.7654	1357.7606	4	1	R.FKDPGLVDQLVK.A	(Ions score 44)
27 - 38	453.5958	1357.7655	1357.7606	4	1	R.FKDPGLVDQLVK.A	(Ions score 44)
27 - 38	453.5958	1357.7655	1357.7606	4	1	R.FKDPGLVDQLVK.A	(Ions score 38)
27 - 38	453.5958	1357.7655	1357.7606	4	1	R.FKDPGLVDQLVK.A	(Ions score 26)
27 - 38	453.5958	1357.7655	1357.7606	4	1	R.FKDPGLVDQLVK.A	(Ions score 23)
27 - 38	453.5958	1357.7655	1357.7606	4	1	R.FKDPGLVDQLVK.A	(Ions score 40)
27 - 38	453.5958	1357.7656	1357.7606	4	1	R.FKDPGLVDQLVK.A	(Ions score 28)
27 - 38	679.8901	1357.7656	1357.7606	4	1	R.FKDPGLVDQLVK.A	(Ions score 27)
27 - 38	453.5958	1357.7657	1357.7606	4	1	R.FKDPGLVDQLVK.A	(Ions score 30)
27 - 38	679.8901	1357.7657	1357.7606	4	1	R.FKDPGLVDQLVK.A	(Ions score 52)
27 - 38	679.8901	1357.7657	1357.7606	4	1	R.FKDPGLVDQLVK.A	(Ions score 22)
27 - 38	453.5959	1357.7658	1357.7606	4	1	R.FKDPGLVDQLVK.A	(Ions score 31)
27 - 38	453.5959	1357.7658	1357.7606	4	1	R.FKDPGLVDQLVK.A	(Ions score 35)
27 - 38	679.8902	1357.7658	1357.7606	4	1	R.FKDPGLVDQLVK.A	(Ions score 39)
27 - 38	453.5959	1357.7658	1357.7606	4	1	R.FKDPGLVDQLVK.A	(Ions score 26)
27 - 38	453.5959	1357.7659	1357.7606	4	1	R.FKDPGLVDQLVK.A	(Ions score 34)
27 - 38	679.8902	1357.7659	1357.7606	4	1	R.FKDPGLVDQLVK.A	(Ions score 25)
27 - 38	679.8902	1357.7659	1357.7606	4	1	R.FKDPGLVDQLVK.A	(Ions score 23)
27 - 38	453.5959	1357.7660	1357.7606	4	1	R.FKDPGLVDQLVK.A	(Ions score 37)
27 - 38	679.8903	1357.7661	1357.7606	4	1	R.FKDPGLVDQLVK.A	(Ions score 27)
27 - 38	679.8903	1357.7661	1357.7606	4	1	R.FKDPGLVDQLVK.A	(Ions score 31)
27 - 38	453.5960	1357.7661	1357.7606	4	1	R.FKDPGLVDQLVK.A	(Ions score 25)

27 - 38	453.5960	1357.7662	1357.7606	4	1	R.FKDPGLVDQLVK.A		(Ions score 24)
27 - 38	453.5960	1357.7662	1357.7606	4	1	R.FKDPGLVDQLVK.A		(Ions score 26)
27 - 38	679.8904	1357.7663	1357.7606	4	1	R.FKDPGLVDQLVK.A		(Ions score 27)
27 - 38	453.5960	1357.7663	1357.7606	4	1	R.FKDPGLVDQLVK.A		(Ions score 54)
27 - 38	679.8905	1357.7664	1357.7606	4	1	R.FKDPGLVDQLVK.A		(Ions score 53)
27 - 38	453.5961	1357.7664	1357.7606	4	1	R.FKDPGLVDQLVK.A		(Ions score 26)
27 - 38	453.5961	1357.7664	1357.7606	4	1	R.FKDPGLVDQLVK.A		(Ions score 51)
27 - 38	453.5962	1357.7666	1357.7606	4	1	R.FKDPGLVDQLVK.A		(Ions score 30)
27 - 38	453.5962	1357.7667	1357.7606	5	1	R.FKDPGLVDQLVK.A		(Ions score 41)
27 - 38	679.8906	1357.7667	1357.7606	5	1	R.FKDPGLVDQLVK.A		(Ions score 27)
27 - 38	453.5962	1357.7668	1357.7606	5	1	R.FKDPGLVDQLVK.A		(Ions score 38)
27 - 38	453.5962	1357.7668	1357.7606	5	1	R.FKDPGLVDQLVK.A		(Ions score 32)
27 - 38	453.5963	1357.7669	1357.7606	5	1	R.FKDPGLVDQLVK.A		(Ions score 30)
27 - 38	453.5963	1357.7670	1357.7606	5	1	R.FKDPGLVDQLVK.A		(Ions score 36)
27 - 38	679.8909	1357.7672	1357.7606	5	1	R.FKDPGLVDQLVK.A		(Ions score 33)
27 - 38	453.5964	1357.7672	1357.7606	5	1	R.FKDPGLVDQLVK.A		(Ions score 40)
27 - 38	700.8929	1399.7713	1399.7711	0	1	R.FKDPGLVDQLVK.A	Acetylation (K)	(Ions score 33)
27 - 38	467.5981	1399.7724	1399.7711	1	1	R.FKDPGLVDQLVK.A	Acetylation (K)	(Ions score 53)
27 - 38	700.8935	1399.7724	1399.7711	1	1	R.FKDPGLVDQLVK.A	Acetylation (K)	(Ions score 36)
27 - 38	467.5983	1399.7731	1399.7711	1	1	R.FKDPGLVDQLVK.A	Acetylation (K)	(Ions score 40)
27 - 38	700.8940	1399.7734	1399.7711	2	1	R.FKDPGLVDQLVK.A	Acetylation (K)	(Ions score 41)
27 - 38	700.8941	1399.7736	1399.7711	2	1	R.FKDPGLVDQLVK.A	Acetylation (K)	(Ions score 39)
27 - 38	467.5985	1399.7737	1399.7711	2	1	R.FKDPGLVDQLVK.A	Acetylation (K)	(Ions score 42)
27 - 38	467.5985	1399.7738	1399.7711	2	1	R.FKDPGLVDQLVK.A	Acetylation (K)	(Ions score 45)
27 - 38	467.5985	1399.7738	1399.7711	2	1	R.FKDPGLVDQLVK.A	Acetylation (K)	(Ions score 30)
27 - 38	467.5986	1399.7739	1399.7711	2	1	R.FKDPGLVDQLVK.A	Acetylation (K)	(Ions score 23)
27 - 38	467.5986	1399.7740	1399.7711	2	1	R.FKDPGLVDQLVK.A	Acetylation (K)	(Ions score 42)
27 - 38	700.8943	1399.7740	1399.7711	2	1	R.FKDPGLVDQLVK.A	Acetylation (K)	(Ions score 45)
27 - 38	700.8943	1399.7740	1399.7711	2	1	R.FKDPGLVDQLVK.A	Acetylation (K)	(Ions score 46)
27 - 38	700.8944	1399.7741	1399.7711	2	1	R.FKDPGLVDQLVK.A	Acetylation (K)	(Ions score 28)
27 - 38	467.5987	1399.7742	1399.7711	2	1	R.FKDPGLVDQLVK.A	Acetylation (K)	(Ions score 33)
27 - 38	467.5987	1399.7743	1399.7711	2	1	R.FKDPGLVDQLVK.A	Acetylation (K)	(Ions score 56)
27 - 38	467.5987	1399.7744	1399.7711	2	1	R.FKDPGLVDQLVK.A	Acetylation (K)	(Ions score 54)
27 - 38	467.5988	1399.7745	1399.7711	2	1	R.FKDPGLVDQLVK.A	Acetylation (K)	(Ions score 47)
27 - 38	467.5988	1399.7746	1399.7711	2	1	R.FKDPGLVDQLVK.A	Acetylation (K)	(Ions score 33)
27 - 38	467.5989	1399.7747	1399.7711	3	1	R.FKDPGLVDQLVK.A	Acetylation (K)	(Ions score 35)
27 - 38	467.5989	1399.7750	1399.7711	3	1	R.FKDPGLVDQLVK.A	Acetylation (K)	(Ions score 52)
27 - 38	467.5990	1399.7751	1399.7711	3	1	R.FKDPGLVDQLVK.A	Acetylation (K)	(Ions score 31)
27 - 38	467.5990	1399.7752	1399.7711	3	1	R.FKDPGLVDQLVK.A	Acetylation (K)	(Ions score 44)
27 - 38	700.8949	1399.7752	1399.7711	3	1	R.FKDPGLVDQLVK.A	Acetylation (K)	(Ions score 27)
27 - 38	467.5994	1399.7765	1399.7711	4	1	R.FKDPGLVDQLVK.A	Acetylation (K)	(Ions score 28)
29 - 38	542.3056	1082.5966	1082.5972	-0	0	K.DPGLVDQLVK.A		(Ions score 33)
29 - 38	542.3058	1082.5971	1082.5972	-0	0	K.DPGLVDQLVK.A		(Ions score 41)
29 - 38	542.3066	1082.5987	1082.5972	1	0	K.DPGLVDQLVK.A		(Ions score 50)
29 - 38	542.3070	1082.5995	1082.5972	2	0	K.DPGLVDQLVK.A		(Ions score 32)
29 - 38	542.3073	1082.6000	1082.5972	3	0	K.DPGLVDQLVK.A		(Ions score 24)
29 - 38	542.3074	1082.6002	1082.5972	3	0	K.DPGLVDQLVK.A		(Ions score 54)
29 - 38	542.3079	1082.6012	1082.5972	4	0	K.DPGLVDQLVK.A		(Ions score 25)
29 - 38	542.3079	1082.6012	1082.5972	4	0	K.DPGLVDQLVK.A		(Ions score 50)
29 - 38	542.3081	1082.6016	1082.5972	4	0	K.DPGLVDQLVK.A		(Ions score 50)
29 - 44	609.9802	1826.9187	1826.9163	1	1	K.DPGLVDQLVKADSEWR.R		(Ions score 24)
29 - 44	609.9809	1826.9209	1826.9163	3	1	K.DPGLVDQLVKADSEWR.R		(Ions score 26)
39 - 44	763.3375	762.3302	762.3297	1	0	K.ADSEWR.R		(Ions score 33)
39 - 44	763.3382	762.3309	762.3297	2	0	K.ADSEWR.R		(Ions score 26)
48 - 55	489.2618	976.5090	976.5090	0	1	R.FRADNLNK.L		(Ions score 22)
48 - 55	489.2619	976.5093	976.5090	0	1	R.FRADNLNK.L		(Ions score 26)
48 - 55	489.2620	976.5095	976.5090	1	1	R.FRADNLNK.L		(Ions score 28)
48 - 55	489.2622	976.5099	976.5090	1	1	R.FRADNLNK.L		(Ions score 27)

48 - 55	489.2622	976.5099	976.5090	1	1	R.FRADNLNK.L	(Ions score 27)
48 - 55	977.5173	976.5100	976.5090	1	1	R.FRADNLNK.L	(Ions score 28)
48 - 55	977.5173	976.5101	976.5090	1	1	R.FRADNLNK.L	(Ions score 35)
48 - 55	977.5173	976.5101	976.5090	1	1	R.FRADNLNK.L	(Ions score 35)
50 - 55	674.3475	673.3403	673.3395	1	0	R.ADNLNK.L	(Ions score 24)
50 - 55	674.3476	673.3403	673.3395	1	0	R.ADNLNK.L	(Ions score 22)
50 - 55	674.3476	673.3403	673.3395	1	0	R.ADNLNK.L	(Ions score 24)
50 - 55	674.3481	673.3408	673.3395	2	0	R.ADNLNK.L	(Ions score 21)
50 - 55	674.3495	673.3423	673.3395	4	0	R.ADNLNK.L	(Ions score 28)
50 - 57	458.2675	914.5205	914.5185	2	1	R.ADNLNKLK.N	(Ions score 43)
50 - 57	479.2719	956.5292	956.5291	0	1	R.ADNLNKLK.N Acetylation (K)	(Ions score 27)
50 - 57	479.2732	956.5319	956.5291	3	1	R.ADNLNKLK.N Acetylation (K)	(Ions score 38)
63 - 67	547.3088	546.3015	546.3013	0	0	K.TIGEK.M	(Ions score 28)
63 - 67	547.3089	546.3016	546.3013	1	0	K.TIGEK.M	(Ions score 27)
63 - 67	547.3089	546.3016	546.3013	1	0	K.TIGEK.M	(Ions score 29)
63 - 67	547.3090	546.3017	546.3013	1	0	K.TIGEK.M	(Ions score 29)
63 - 67	547.3090	546.3017	546.3013	1	0	K.TIGEK.M	(Ions score 24)
63 - 67	547.3091	546.3019	546.3013	1	0	K.TIGEK.M	(Ions score 25)
63 - 67	547.3096	546.3023	546.3013	2	0	K.TIGEK.M	(Ions score 28)
71 - 99	1034.5101	3100.5084	3100.5088	-0	1	K.KEPVGDDESVPENVLSFDDLTADALANLK.V	(Ions score 63)
71 - 99	1551.2617	3100.5089	3100.5088	0	1	K.KEPVGDDESVPENVLSFDDLTADALANLK.V	(Ions score 98)
71 - 99	621.1091	3100.5089	3100.5088	0	1	K.KEPVGDDESVPENVLSFDDLTADALANLK.V	(Ions score 60)
71 - 99	1034.5105	3100.5098	3100.5088	0	1	K.KEPVGDDESVPENVLSFDDLTADALANLK.V	(Ions score 58)
71 - 99	1551.2622	3100.5099	3100.5088	0	1	K.KEPVGDDESVPENVLSFDDLTADALANLK.V	(Ions score 88)
71 - 99	1034.5107	3100.5104	3100.5088	1	1	K.KEPVGDDESVPENVLSFDDLTADALANLK.V	(Ions score 77)
71 - 99	1034.5113	3100.5120	3100.5088	1	1	K.KEPVGDDESVPENVLSFDDLTADALANLK.V	(Ions score 53)
71 - 99	776.1353	3100.5122	3100.5088	1	1	K.KEPVGDDESVPENVLSFDDLTADALANLK.V	(Ions score 96)
71 - 99	1034.5114	3100.5123	3100.5088	1	1	K.KEPVGDDESVPENVLSFDDLTADALANLK.V	(Ions score 49)
71 - 99	776.1354	3100.5124	3100.5088	1	1	K.KEPVGDDESVPENVLSFDDLTADALANLK.V	(Ions score 66)
71 - 99	1034.5114	3100.5125	3100.5088	1	1	K.KEPVGDDESVPENVLSFDDLTADALANLK.V	(Ions score 63)
71 - 99	1551.2635	3100.5125	3100.5088	1	1	K.KEPVGDDESVPENVLSFDDLTADALANLK.V	(Ions score 91)
71 - 99	1551.2638	3100.5130	3100.5088	1	1	K.KEPVGDDESVPENVLSFDDLTADALANLK.V	(Ions score 78)
71 - 99	1551.2638	3100.5130	3100.5088	1	1	K.KEPVGDDESVPENVLSFDDLTADALANLK.V	(Ions score 84)
71 - 99	1034.5118	3100.5134	3100.5088	1	1	K.KEPVGDDESVPENVLSFDDLTADALANLK.V	(Ions score 59)
71 - 99	1551.2640	3100.5135	3100.5088	2	1	K.KEPVGDDESVPENVLSFDDLTADALANLK.V	(Ions score 45)
71 - 99	1034.5118	3100.5135	3100.5088	2	1	K.KEPVGDDESVPENVLSFDDLTADALANLK.V	(Ions score 53)
71 - 99	1034.5118	3100.5135	3100.5088	2	1	K.KEPVGDDESVPENVLSFDDLTADALANLK.V	(Ions score 86)
71 - 99	1034.5119	3100.5138	3100.5088	2	1	K.KEPVGDDESVPENVLSFDDLTADALANLK.V	(Ions score 55)
71 - 99	1551.2642	3100.5139	3100.5088	2	1	K.KEPVGDDESVPENVLSFDDLTADALANLK.V	(Ions score 58)
71 - 99	1551.2643	3100.5141	3100.5088	2	1	K.KEPVGDDESVPENVLSFDDLTADALANLK.V	(Ions score 72)
71 - 99	1034.5121	3100.5145	3100.5088	2	1	K.KEPVGDDESVPENVLSFDDLTADALANLK.V	(Ions score 57)
71 - 99	776.1360	3100.5149	3100.5088	2	1	K.KEPVGDDESVPENVLSFDDLTADALANLK.V	(Ions score 96)
71 - 99	776.1360	3100.5150	3100.5088	2	1	K.KEPVGDDESVPENVLSFDDLTADALANLK.V	(Ions score 81)
71 - 99	1034.5123	3100.5152	3100.5088	2	1	K.KEPVGDDESVPENVLSFDDLTADALANLK.V	(Ions score 81)
71 - 99	776.1361	3100.5153	3100.5088	2	1	K.KEPVGDDESVPENVLSFDDLTADALANLK.V	(Ions score 92)
71 - 99	776.1361	3100.5155	3100.5088	2	1	K.KEPVGDDESVPENVLSFDDLTADALANLK.V	(Ions score 85)
71 - 99	776.1361	3100.5155	3100.5088	2	1	K.KEPVGDDESVPENVLSFDDLTADALANLK.V	(Ions score 81)
71 - 99	1551.2651	3100.5156	3100.5088	2	1	K.KEPVGDDESVPENVLSFDDLTADALANLK.V	(Ions score 65)
71 - 99	1034.5126	3100.5160	3100.5088	2	1	K.KEPVGDDESVPENVLSFDDLTADALANLK.V	(Ions score 63)
71 - 99	621.1105	3100.5160	3100.5088	2	1	K.KEPVGDDESVPENVLSFDDLTADALANLK.V	(Ions score 53)
71 - 99	1551.2653	3100.5161	3100.5088	2	1	K.KEPVGDDESVPENVLSFDDLTADALANLK.V	(Ions score 60)
71 - 99	1034.5127	3100.5163	3100.5088	2	1	K.KEPVGDDESVPENVLSFDDLTADALANLK.V	(Ions score 67)
71 - 99	621.1106	3100.5164	3100.5088	2	1	K.KEPVGDDESVPENVLSFDDLTADALANLK.V	(Ions score 47)
71 - 99	1034.5128	3100.5166	3100.5088	3	1	K.KEPVGDDESVPENVLSFDDLTADALANLK.V	(Ions score 67)
71 - 99	1034.5129	3100.5168	3100.5088	3	1	K.KEPVGDDESVPENVLSFDDLTADALANLK.V	(Ions score 96)
71 - 99	1034.5130	3100.5171	3100.5088	3	1	K.KEPVGDDESVPENVLSFDDLTADALANLK.V	(Ions score 71)
71 - 99	1034.5131	3100.5173	3100.5088	3	1	K.KEPVGDDESVPENVLSFDDLTADALANLK.V	(Ions score 77)
71 - 99	1034.5132	3100.5179	3100.5088	3	1	K.KEPVGDDESVPENVLSFDDLTADALANLK.V	(Ions score 80)

71 - 99	776.1368	3100.5179	3100.5088	3	1	K.KEPVGDDESVPENVLSFDDLTADALANLK.V	(Ions score 56)
71 - 99	776.1368	3100.5180	3100.5088	3	1	K.KEPVGDDESVPENVLSFDDLTADALANLK.V	(Ions score 74)
71 - 99	1034.5133	3100.5180	3100.5088	3	1	K.KEPVGDDESVPENVLSFDDLTADALANLK.V	(Ions score 92)
71 - 99	1551.2663	3100.5181	3100.5088	3	1	K.KEPVGDDESVPENVLSFDDLTADALANLK.V	(Ions score 47)
71 - 99	1551.2663	3100.5181	3100.5088	3	1	K.KEPVGDDESVPENVLSFDDLTADALANLK.V	(Ions score 52)
71 - 99	1034.5133	3100.5181	3100.5088	3	1	K.KEPVGDDESVPENVLSFDDLTADALANLK.V	(Ions score 80)
71 - 99	1034.5133	3100.5182	3100.5088	3	1	K.KEPVGDDESVPENVLSFDDLTADALANLK.V	(Ions score 63)
71 - 99	1034.5134	3100.5182	3100.5088	3	1	K.KEPVGDDESVPENVLSFDDLTADALANLK.V	(Ions score 73)
71 - 99	1034.5134	3100.5185	3100.5088	3	1	K.KEPVGDDESVPENVLSFDDLTADALANLK.V	(Ions score 63)
71 - 99	776.1369	3100.5185	3100.5088	3	1	K.KEPVGDDESVPENVLSFDDLTADALANLK.V	(Ions score 65)
71 - 99	1034.5134	3100.5185	3100.5088	3	1	K.KEPVGDDESVPENVLSFDDLTADALANLK.V	(Ions score 71)
71 - 99	621.1110	3100.5185	3100.5088	3	1	K.KEPVGDDESVPENVLSFDDLTADALANLK.V	(Ions score 60)
71 - 99	1034.5135	3100.5186	3100.5088	3	1	K.KEPVGDDESVPENVLSFDDLTADALANLK.V	(Ions score 62)
71 - 99	1034.5135	3100.5187	3100.5088	3	1	K.KEPVGDDESVPENVLSFDDLTADALANLK.V	(Ions score 80)
71 - 99	1034.5135	3100.5187	3100.5088	3	1	K.KEPVGDDESVPENVLSFDDLTADALANLK.V	(Ions score 47)
71 - 99	1034.5137	3100.5192	3100.5088	3	1	K.KEPVGDDESVPENVLSFDDLTADALANLK.V	(Ions score 63)
71 - 99	1034.5137	3100.5192	3100.5088	3	1	K.KEPVGDDESVPENVLSFDDLTADALANLK.V	(Ions score 64)
71 - 99	1034.5137	3100.5194	3100.5088	3	1	K.KEPVGDDESVPENVLSFDDLTADALANLK.V	(Ions score 82)
71 - 99	1034.5138	3100.5194	3100.5088	3	1	K.KEPVGDDESVPENVLSFDDLTADALANLK.V	(Ions score 63)
71 - 99	1034.5138	3100.5195	3100.5088	3	1	K.KEPVGDDESVPENVLSFDDLTADALANLK.V	(Ions score 83)
71 - 99	1034.5138	3100.5195	3100.5088	3	1	K.KEPVGDDESVPENVLSFDDLTADALANLK.V	(Ions score 77)
71 - 99	1034.5138	3100.5196	3100.5088	3	1	K.KEPVGDDESVPENVLSFDDLTADALANLK.V	(Ions score 85)
71 - 99	1034.5138	3100.5197	3100.5088	4	1	K.KEPVGDDESVPENVLSFDDLTADALANLK.V	(Ions score 70)
71 - 99	1034.5139	3100.5198	3100.5088	4	1	K.KEPVGDDESVPENVLSFDDLTADALANLK.V	(Ions score 72)
71 - 99	1034.5139	3100.5199	3100.5088	4	1	K.KEPVGDDESVPENVLSFDDLTADALANLK.V	(Ions score 69)
71 - 99	1034.5139	3100.5199	3100.5088	4	1	K.KEPVGDDESVPENVLSFDDLTADALANLK.V	(Ions score 67)
71 - 99	1034.5139	3100.5199	3100.5088	4	1	K.KEPVGDDESVPENVLSFDDLTADALANLK.V	(Ions score 72)
71 - 99	1034.5140	3100.5200	3100.5088	4	1	K.KEPVGDDESVPENVLSFDDLTADALANLK.V	(Ions score 66)
71 - 99	1034.5140	3100.5202	3100.5088	4	1	K.KEPVGDDESVPENVLSFDDLTADALANLK.V	(Ions score 92)
71 - 99	1034.5141	3100.5204	3100.5088	4	1	K.KEPVGDDESVPENVLSFDDLTADALANLK.V	(Ions score 68)
71 - 99	1034.5141	3100.5204	3100.5088	4	1	K.KEPVGDDESVPENVLSFDDLTADALANLK.V	(Ions score 77)
71 - 99	1551.2676	3100.5206	3100.5088	4	1	K.KEPVGDDESVPENVLSFDDLTADALANLK.V	(Ions score 54)
71 - 99	1034.5142	3100.5207	3100.5088	4	1	K.KEPVGDDESVPENVLSFDDLTADALANLK.V	(Ions score 85)
71 - 99	1034.5142	3100.5208	3100.5088	4	1	K.KEPVGDDESVPENVLSFDDLTADALANLK.V	(Ions score 83)
71 - 99	1034.5142	3100.5208	3100.5088	4	1	K.KEPVGDDESVPENVLSFDDLTADALANLK.V	(Ions score 73)
71 - 99	1034.5142	3100.5209	3100.5088	4	1	K.KEPVGDDESVPENVLSFDDLTADALANLK.V	(Ions score 92)
71 - 99	1034.5143	3100.5209	3100.5088	4	1	K.KEPVGDDESVPENVLSFDDLTADALANLK.V	(Ions score 70)
71 - 99	1034.5143	3100.5209	3100.5088	4	1	K.KEPVGDDESVPENVLSFDDLTADALANLK.V	(Ions score 108)
71 - 99	1034.5144	3100.5214	3100.5088	4	1	K.KEPVGDDESVPENVLSFDDLTADALANLK.V	(Ions score 85)
71 - 99	1034.5144	3100.5214	3100.5088	4	1	K.KEPVGDDESVPENVLSFDDLTADALANLK.V	(Ions score 63)
71 - 99	1034.5145	3100.5216	3100.5088	4	1	K.KEPVGDDESVPENVLSFDDLTADALANLK.V	(Ions score 67)
71 - 99	1034.5145	3100.5218	3100.5088	4	1	K.KEPVGDDESVPENVLSFDDLTADALANLK.V	(Ions score 85)
71 - 99	1034.5146	3100.5219	3100.5088	4	1	K.KEPVGDDESVPENVLSFDDLTADALANLK.V	(Ions score 77)
71 - 99	1034.5146	3100.5220	3100.5088	4	1	K.KEPVGDDESVPENVLSFDDLTADALANLK.V	(Ions score 68)
71 - 99	1034.5146	3100.5220	3100.5088	4	1	K.KEPVGDDESVPENVLSFDDLTADALANLK.V	(Ions score 66)
71 - 99	1034.5146	3100.5220	3100.5088	4	1	K.KEPVGDDESVPENVLSFDDLTADALANLK.V	(Ions score 82)
71 - 99	1034.5147	3100.5223	3100.5088	4	1	K.KEPVGDDESVPENVLSFDDLTADALANLK.V	(Ions score 71)
71 - 99	1034.5147	3100.5223	3100.5088	4	1	K.KEPVGDDESVPENVLSFDDLTADALANLK.V	(Ions score 67)
71 - 99	1034.5147	3100.5223	3100.5088	4	1	K.KEPVGDDESVPENVLSFDDLTADALANLK.V	(Ions score 92)
71 - 99	1034.5147	3100.5223	3100.5088	4	1	K.KEPVGDDESVPENVLSFDDLTADALANLK.V	(Ions score 96)
71 - 99	1034.5147	3100.5224	3100.5088	4	1	K.KEPVGDDESVPENVLSFDDLTADALANLK.V	(Ions score 69)
71 - 99	1034.5147	3100.5224	3100.5088	4	1	K.KEPVGDDESVPENVLSFDDLTADALANLK.V	(Ions score 84)
71 - 99	1034.5148	3100.5224	3100.5088	4	1	K.KEPVGDDESVPENVLSFDDLTADALANLK.V	(Ions score 77)
71 - 99	1034.5149	3100.5229	3100.5088	5	1	K.KEPVGDDESVPENVLSFDDLTADALANLK.V	(Ions score 60)
71 - 99	1034.5152	3100.5237	3100.5088	5	1	K.KEPVGDDESVPENVLSFDDLTADALANLK.V	(Ions score 83)
71 - 99	1034.5152	3100.5237	3100.5088	5	1	K.KEPVGDDESVPENVLSFDDLTADALANLK.V	(Ions score 71)
71 - 99	776.1382	3100.5238	3100.5088	5	1	K.KEPVGDDESVPENVLSFDDLTADALANLK.V	(Ions score 45)
71 - 99	1034.5152	3100.5238	3100.5088	5	1	K.KEPVGDDESVPENVLSFDDLTADALANLK.V	(Ions score 77)

71 - 99	1034.5152	3100.5238	3100.5088	5	1	K.KEPVGDDESVPENVLSFDDLTADALANLK.V		(Ions score 88)
71 - 99	1572.2725	3142.5304	3142.5194	4	1	K.KEPVGDDESVPENVLSFDDLTADALANLK.V	Acetylation (K)	(Ions score 56)
71 - 99	1048.5181	3142.5325	3142.5194	4	1	K.KEPVGDDESVPENVLSFDDLTADALANLK.V	Acetylation (K)	(Ions score 66)
71 - 99	786.6408	3142.5341	3142.5194	5	1	K.KEPVGDDESVPENVLSFDDLTADALANLK.V	Acetylation (K)	(Ions score 48)
72 - 99	991.8108	2972.4106	2972.4138	-1	0	K.EPVGDDESVPENVLSFDDLTADALANLK.V		(Ions score 71)
72 - 99	1487.2133	2972.4120	2972.4138	-1	0	K.EPVGDDESVPENVLSFDDLTADALANLK.V		(Ions score 69)
72 - 99	1487.2137	2972.4129	2972.4138	-0	0	K.EPVGDDESVPENVLSFDDLTADALANLK.V		(Ions score 71)
72 - 99	991.8121	2972.4145	2972.4138	0	0	K.EPVGDDESVPENVLSFDDLTADALANLK.V		(Ions score 85)
72 - 99	744.1111	2972.4153	2972.4138	1	0	K.EPVGDDESVPENVLSFDDLTADALANLK.V		(Ions score 87)
72 - 99	991.8128	2972.4164	2972.4138	1	0	K.EPVGDDESVPENVLSFDDLTADALANLK.V		(Ions score 106)
72 - 99	1487.2156	2972.4167	2972.4138	1	0	K.EPVGDDESVPENVLSFDDLTADALANLK.V		(Ions score 80)
72 - 99	1487.2158	2972.4170	2972.4138	1	0	K.EPVGDDESVPENVLSFDDLTADALANLK.V		(Ions score 68)
72 - 99	1487.2158	2972.4171	2972.4138	1	0	K.EPVGDDESVPENVLSFDDLTADALANLK.V		(Ions score 58)
72 - 99	744.1120	2972.4187	2972.4138	2	0	K.EPVGDDESVPENVLSFDDLTADALANLK.V		(Ions score 77)
72 - 99	1487.2167	2972.4188	2972.4138	2	0	K.EPVGDDESVPENVLSFDDLTADALANLK.V		(Ions score 64)
72 - 99	1487.2167	2972.4189	2972.4138	2	0	K.EPVGDDESVPENVLSFDDLTADALANLK.V		(Ions score 39)
72 - 99	1487.2168	2972.4191	2972.4138	2	0	K.EPVGDDESVPENVLSFDDLTADALANLK.V		(Ions score 91)
72 - 99	1487.2169	2972.4192	2972.4138	2	0	K.EPVGDDESVPENVLSFDDLTADALANLK.V		(Ions score 89)
72 - 99	1487.2169	2972.4193	2972.4138	2	0	K.EPVGDDESVPENVLSFDDLTADALANLK.V		(Ions score 116)
72 - 99	991.8138	2972.4195	2972.4138	2	0	K.EPVGDDESVPENVLSFDDLTADALANLK.V		(Ions score 59)
72 - 99	1487.2170	2972.4195	2972.4138	2	0	K.EPVGDDESVPENVLSFDDLTADALANLK.V		(Ions score 30)
72 - 99	991.8138	2972.4196	2972.4138	2	0	K.EPVGDDESVPENVLSFDDLTADALANLK.V		(Ions score 97)
72 - 99	991.8138	2972.4197	2972.4138	2	0	K.EPVGDDESVPENVLSFDDLTADALANLK.V		(Ions score 57)
72 - 99	991.8139	2972.4198	2972.4138	2	0	K.EPVGDDESVPENVLSFDDLTADALANLK.V		(Ions score 82)
72 - 99	991.8139	2972.4198	2972.4138	2	0	K.EPVGDDESVPENVLSFDDLTADALANLK.V		(Ions score 95)
72 - 99	991.8139	2972.4200	2972.4138	2	0	K.EPVGDDESVPENVLSFDDLTADALANLK.V		(Ions score 70)
72 - 99	744.1123	2972.4200	2972.4138	2	0	K.EPVGDDESVPENVLSFDDLTADALANLK.V		(Ions score 84)
72 - 99	991.8140	2972.4201	2972.4138	2	0	K.EPVGDDESVPENVLSFDDLTADALANLK.V		(Ions score 98)
72 - 99	991.8141	2972.4204	2972.4138	2	0	K.EPVGDDESVPENVLSFDDLTADALANLK.V		(Ions score 81)
72 - 99	991.8141	2972.4205	2972.4138	2	0	K.EPVGDDESVPENVLSFDDLTADALANLK.V		(Ions score 75)
72 - 99	991.8143	2972.4210	2972.4138	2	0	K.EPVGDDESVPENVLSFDDLTADALANLK.V		(Ions score 102)
72 - 99	991.8143	2972.4210	2972.4138	2	0	K.EPVGDDESVPENVLSFDDLTADALANLK.V		(Ions score 71)
72 - 99	991.8143	2972.4211	2972.4138	2	0	K.EPVGDDESVPENVLSFDDLTADALANLK.V		(Ions score 87)
72 - 99	991.8146	2972.4219	2972.4138	3	0	K.EPVGDDESVPENVLSFDDLTADALANLK.V		(Ions score 77)
72 - 99	991.8147	2972.4221	2972.4138	3	0	K.EPVGDDESVPENVLSFDDLTADALANLK.V		(Ions score 95)
72 - 99	991.8147	2972.4223	2972.4138	3	0	K.EPVGDDESVPENVLSFDDLTADALANLK.V		(Ions score 52)
72 - 99	1487.2184	2972.4223	2972.4138	3	0	K.EPVGDDESVPENVLSFDDLTADALANLK.V		(Ions score 53)
72 - 99	991.8147	2972.4223	2972.4138	3	0	K.EPVGDDESVPENVLSFDDLTADALANLK.V		(Ions score 108)
72 - 99	991.8147	2972.4224	2972.4138	3	0	K.EPVGDDESVPENVLSFDDLTADALANLK.V		(Ions score 86)
72 - 99	991.8148	2972.4224	2972.4138	3	0	K.EPVGDDESVPENVLSFDDLTADALANLK.V		(Ions score 99)
72 - 99	991.8148	2972.4225	2972.4138	3	0	K.EPVGDDESVPENVLSFDDLTADALANLK.V		(Ions score 96)
72 - 99	991.8148	2972.4226	2972.4138	3	0	K.EPVGDDESVPENVLSFDDLTADALANLK.V		(Ions score 81)
72 - 99	991.8148	2972.4226	2972.4138	3	0	K.EPVGDDESVPENVLSFDDLTADALANLK.V		(Ions score 52)
72 - 99	991.8150	2972.4231	2972.4138	3	0	K.EPVGDDESVPENVLSFDDLTADALANLK.V		(Ions score 77)
72 - 99	991.8151	2972.4234	2972.4138	3	0	K.EPVGDDESVPENVLSFDDLTADALANLK.V		(Ions score 80)
72 - 99	991.8151	2972.4234	2972.4138	3	0	K.EPVGDDESVPENVLSFDDLTADALANLK.V		(Ions score 72)
72 - 99	991.8151	2972.4235	2972.4138	3	0	K.EPVGDDESVPENVLSFDDLTADALANLK.V		(Ions score 99)
72 - 99	991.8152	2972.4236	2972.4138	3	0	K.EPVGDDESVPENVLSFDDLTADALANLK.V		(Ions score 85)
72 - 99	991.8153	2972.4239	2972.4138	3	0	K.EPVGDDESVPENVLSFDDLTADALANLK.V		(Ions score 96)
72 - 99	991.8153	2972.4239	2972.4138	3	0	K.EPVGDDESVPENVLSFDDLTADALANLK.V		(Ions score 51)
72 - 99	1487.2193	2972.4240	2972.4138	3	0	K.EPVGDDESVPENVLSFDDLTADALANLK.V		(Ions score 80)
72 - 99	991.8153	2972.4241	2972.4138	3	0	K.EPVGDDESVPENVLSFDDLTADALANLK.V		(Ions score 103)
72 - 99	991.8154	2972.4244	2972.4138	4	0	K.EPVGDDESVPENVLSFDDLTADALANLK.V		(Ions score 85)
72 - 99	1487.2195	2972.4245	2972.4138	4	0	K.EPVGDDESVPENVLSFDDLTADALANLK.V		(Ions score 41)
72 - 99	991.8155	2972.4248	2972.4138	4	0	K.EPVGDDESVPENVLSFDDLTADALANLK.V		(Ions score 78)
72 - 99	1487.2199	2972.4253	2972.4138	4	0	K.EPVGDDESVPENVLSFDDLTADALANLK.V		(Ions score 56)
72 - 99	991.8157	2972.4253	2972.4138	4	0	K.EPVGDDESVPENVLSFDDLTADALANLK.V		(Ions score 63)
72 - 99	991.8157	2972.4254	2972.4138	4	0	K.EPVGDDESVPENVLSFDDLTADALANLK.V		(Ions score 88)

72 - 99	1487.2201	2972.4256	2972.4138	4	0	K.EPVGDDESVPENVLSFDDLTADALANLK.V	(Ions score 38)
72 - 99	991.8158	2972.4256	2972.4138	4	0	K.EPVGDDESVPENVLSFDDLTADALANLK.V	(Ions score 70)
72 - 99	991.8158	2972.4256	2972.4138	4	0	K.EPVGDDESVPENVLSFDDLTADALANLK.V	(Ions score 100)
72 - 99	991.8160	2972.4261	2972.4138	4	0	K.EPVGDDESVPENVLSFDDLTADALANLK.V	(Ions score 71)
72 - 99	991.8161	2972.4264	2972.4138	4	0	K.EPVGDDESVPENVLSFDDLTADALANLK.V	(Ions score 103)
72 - 99	991.8161	2972.4265	2972.4138	4	0	K.EPVGDDESVPENVLSFDDLTADALANLK.V	(Ions score 94)
72 - 99	991.8161	2972.4266	2972.4138	4	0	K.EPVGDDESVPENVLSFDDLTADALANLK.V	(Ions score 87)
72 - 99	991.8162	2972.4267	2972.4138	4	0	K.EPVGDDESVPENVLSFDDLTADALANLK.V	(Ions score 65)
72 - 99	1487.2206	2972.4267	2972.4138	4	0	K.EPVGDDESVPENVLSFDDLTADALANLK.V	(Ions score 68)
72 - 99	991.8163	2972.4272	2972.4138	4	0	K.EPVGDDESVPENVLSFDDLTADALANLK.V	(Ions score 100)
72 - 99	991.8164	2972.4272	2972.4138	5	0	K.EPVGDDESVPENVLSFDDLTADALANLK.V	(Ions score 96)
72 - 99	1487.2209	2972.4273	2972.4138	5	0	K.EPVGDDESVPENVLSFDDLTADALANLK.V	(Ions score 66)
72 - 99	991.8165	2972.4276	2972.4138	5	0	K.EPVGDDESVPENVLSFDDLTADALANLK.V	(Ions score 99)
72 - 99	991.8165	2972.4277	2972.4138	5	0	K.EPVGDDESVPENVLSFDDLTADALANLK.V	(Ions score 94)
72 - 99	991.8166	2972.4278	2972.4138	5	0	K.EPVGDDESVPENVLSFDDLTADALANLK.V	(Ions score 97)
72 - 99	991.8168	2972.4285	2972.4138	5	0	K.EPVGDDESVPENVLSFDDLTADALANLK.V	(Ions score 63)
72 - 104	882.9468	3527.7581	3527.7519	2	1	K.EPVGDDESVPENVLSFDDLTADALANLKVSQIK.K	(Ions score 33)
72 - 104	1176.9278	3527.7615	3527.7519	3	1	K.EPVGDDESVPENVLSFDDLTADALANLKVSQIK.K	(Ions score 34)
100 - 104	574.3553	573.3481	573.3486	-1	0	K.VSQIK.K	(Ions score 22)
100 - 104	574.3560	573.3487	573.3486	0	0	K.VSQIK.K	(Ions score 23)
100 - 104	574.3561	573.3489	573.3486	0	0	K.VSQIK.K	(Ions score 23)
100 - 104	574.3563	573.3490	573.3486	1	0	K.VSQIK.K	(Ions score 23)
100 - 104	574.3566	573.3493	573.3486	1	0	K.VSQIK.K	(Ions score 27)
100 - 104	574.3566	573.3493	573.3486	1	0	K.VSQIK.K	(Ions score 21)
100 - 104	574.3567	573.3494	573.3486	1	0	K.VSQIK.K	(Ions score 22)
100 - 104	574.3568	573.3495	573.3486	2	0	K.VSQIK.K	(Ions score 21)
100 - 104	574.3568	573.3495	573.3486	2	0	K.VSQIK.K	(Ions score 23)
100 - 104	574.3569	573.3497	573.3486	2	0	K.VSQIK.K	(Ions score 21)
100 - 104	574.3571	573.3498	573.3486	2	0	K.VSQIK.K	(Ions score 21)
106 - 116	428.2764	1281.8073	1281.8020	4	1	K.VRLLIDEAILK.C	(Ions score 41)
108 - 116	514.3231	1026.6317	1026.6325	-1	0	R.LLIDEAILK.C	(Ions score 23)
108 - 116	514.3234	1026.6322	1026.6325	-0	0	R.LLIDEAILK.C	(Ions score 23)
108 - 116	514.3236	1026.6327	1026.6325	0	0	R.LLIDEAILK.C	(Ions score 32)
108 - 116	514.3236	1026.6327	1026.6325	0	0	R.LLIDEAILK.C	(Ions score 31)
108 - 116	514.3236	1026.6327	1026.6325	0	0	R.LLIDEAILK.C	(Ions score 24)
108 - 116	514.3237	1026.6328	1026.6325	0	0	R.LLIDEAILK.C	(Ions score 24)
108 - 116	514.3237	1026.6328	1026.6325	0	0	R.LLIDEAILK.C	(Ions score 21)
108 - 116	514.3237	1026.6329	1026.6325	0	0	R.LLIDEAILK.C	(Ions score 24)
108 - 116	514.3238	1026.6330	1026.6325	1	0	R.LLIDEAILK.C	(Ions score 24)
108 - 116	514.3238	1026.6331	1026.6325	1	0	R.LLIDEAILK.C	(Ions score 23)
108 - 116	514.3238	1026.6331	1026.6325	1	0	R.LLIDEAILK.C	(Ions score 23)
108 - 116	514.3239	1026.6332	1026.6325	1	0	R.LLIDEAILK.C	(Ions score 32)
108 - 116	514.3239	1026.6333	1026.6325	1	0	R.LLIDEAILK.C	(Ions score 30)
108 - 116	514.3240	1026.6334	1026.6325	1	0	R.LLIDEAILK.C	(Ions score 23)
108 - 116	514.3240	1026.6334	1026.6325	1	0	R.LLIDEAILK.C	(Ions score 32)
108 - 116	514.3240	1026.6334	1026.6325	1	0	R.LLIDEAILK.C	(Ions score 32)
108 - 116	514.3240	1026.6334	1026.6325	1	0	R.LLIDEAILK.C	(Ions score 32)
108 - 116	514.3240	1026.6334	1026.6325	1	0	R.LLIDEAILK.C	(Ions score 32)
108 - 116	514.3240	1026.6334	1026.6325	1	0	R.LLIDEAILK.C	(Ions score 23)
108 - 116	514.3240	1026.6334	1026.6325	1	0	R.LLIDEAILK.C	(Ions score 32)
108 - 116	514.3240	1026.6335	1026.6325	1	0	R.LLIDEAILK.C	(Ions score 24)
108 - 116	514.3240	1026.6335	1026.6325	1	0	R.LLIDEAILK.C	(Ions score 32)
108 - 116	514.3240	1026.6335	1026.6325	1	0	R.LLIDEAILK.C	(Ions score 44)
108 - 116	514.3240	1026.6335	1026.6325	1	0	R.LLIDEAILK.C	(Ions score 23)
108 - 116	514.3240	1026.6335	1026.6325	1	0	R.LLIDEAILK.C	(Ions score 32)
108 - 116	514.3240	1026.6335	1026.6325	1	0	R.LLIDEAILK.C	(Ions score 24)
108 - 116	514.3241	1026.6336	1026.6325	1	0	R.LLIDEAILK.C	(Ions score 32)
108 - 116	514.3241	1026.6336	1026.6325	1	0	R.LLIDEAILK.C	(Ions score 24)

108 - 116	514.3241	1026.6336	1026.6325	1	0	R.LLIDEAILK.C	(Ions score 23)
108 - 116	514.3241	1026.6336	1026.6325	1	0	R.LLIDEAILK.C	(Ions score 32)
108 - 116	514.3241	1026.6337	1026.6325	1	0	R.LLIDEAILK.C	(Ions score 24)
108 - 116	514.3242	1026.6338	1026.6325	1	0	R.LLIDEAILK.C	(Ions score 32)
108 - 116	514.3242	1026.6338	1026.6325	1	0	R.LLIDEAILK.C	(Ions score 31)
108 - 116	514.3242	1026.6338	1026.6325	1	0	R.LLIDEAILK.C	(Ions score 21)
108 - 116	514.3242	1026.6338	1026.6325	1	0	R.LLIDEAILK.C	(Ions score 32)
108 - 116	514.3242	1026.6338	1026.6325	1	0	R.LLIDEAILK.C	(Ions score 31)
108 - 116	514.3242	1026.6339	1026.6325	1	0	R.LLIDEAILK.C	(Ions score 21)
108 - 116	514.3242	1026.6339	1026.6325	1	0	R.LLIDEAILK.C	(Ions score 32)
108 - 116	514.3242	1026.6339	1026.6325	1	0	R.LLIDEAILK.C	(Ions score 31)
108 - 116	514.3242	1026.6339	1026.6325	1	0	R.LLIDEAILK.C	(Ions score 23)
108 - 116	514.3243	1026.6340	1026.6325	1	0	R.LLIDEAILK.C	(Ions score 23)
108 - 116	514.3243	1026.6340	1026.6325	1	0	R.LLIDEAILK.C	(Ions score 32)
108 - 116	514.3243	1026.6340	1026.6325	1	0	R.LLIDEAILK.C	(Ions score 23)
108 - 116	514.3243	1026.6341	1026.6325	2	0	R.LLIDEAILK.C	(Ions score 24)
108 - 116	514.3243	1026.6341	1026.6325	2	0	R.LLIDEAILK.C	(Ions score 23)
108 - 116	514.3243	1026.6341	1026.6325	2	0	R.LLIDEAILK.C	(Ions score 31)
108 - 116	514.3243	1026.6341	1026.6325	2	0	R.LLIDEAILK.C	(Ions score 32)
108 - 116	514.3243	1026.6341	1026.6325	2	0	R.LLIDEAILK.C	(Ions score 23)
108 - 116	514.3244	1026.6342	1026.6325	2	0	R.LLIDEAILK.C	(Ions score 25)
108 - 116	514.3244	1026.6342	1026.6325	2	0	R.LLIDEAILK.C	(Ions score 24)
108 - 116	514.3244	1026.6343	1026.6325	2	0	R.LLIDEAILK.C	(Ions score 23)
108 - 116	514.3244	1026.6343	1026.6325	2	0	R.LLIDEAILK.C	(Ions score 23)
108 - 116	514.3245	1026.6344	1026.6325	2	0	R.LLIDEAILK.C	(Ions score 32)
108 - 116	514.3245	1026.6344	1026.6325	2	0	R.LLIDEAILK.C	(Ions score 31)
108 - 116	514.3245	1026.6344	1026.6325	2	0	R.LLIDEAILK.C	(Ions score 24)
108 - 116	514.3245	1026.6344	1026.6325	2	0	R.LLIDEAILK.C	(Ions score 24)
108 - 116	514.3245	1026.6344	1026.6325	2	0	R.LLIDEAILK.C	(Ions score 32)
108 - 116	514.3245	1026.6344	1026.6325	2	0	R.LLIDEAILK.C	(Ions score 24)
108 - 116	514.3245	1026.6345	1026.6325	2	0	R.LLIDEAILK.C	(Ions score 32)
108 - 116	514.3245	1026.6345	1026.6325	2	0	R.LLIDEAILK.C	(Ions score 23)
108 - 116	514.3246	1026.6346	1026.6325	2	0	R.LLIDEAILK.C	(Ions score 32)
108 - 116	514.3246	1026.6346	1026.6325	2	0	R.LLIDEAILK.C	(Ions score 32)
108 - 116	514.3246	1026.6347	1026.6325	2	0	R.LLIDEAILK.C	(Ions score 24)
108 - 116	514.3246	1026.6347	1026.6325	2	0	R.LLIDEAILK.C	(Ions score 24)
108 - 116	514.3246	1026.6347	1026.6325	2	0	R.LLIDEAILK.C	(Ions score 24)
108 - 116	514.3246	1026.6347	1026.6325	2	0	R.LLIDEAILK.C	(Ions score 24)
108 - 116	514.3246	1026.6347	1026.6325	2	0	R.LLIDEAILK.C	(Ions score 24)
108 - 116	514.3246	1026.6347	1026.6325	2	0	R.LLIDEAILK.C	(Ions score 23)
108 - 116	514.3246	1026.6347	1026.6325	2	0	R.LLIDEAILK.C	(Ions score 31)
108 - 116	514.3246	1026.6347	1026.6325	2	0	R.LLIDEAILK.C	(Ions score 24)
108 - 116	514.3246	1026.6347	1026.6325	2	0	R.LLIDEAILK.C	(Ions score 32)
108 - 116	514.3247	1026.6348	1026.6325	2	0	R.LLIDEAILK.C	(Ions score 31)
108 - 116	514.3247	1026.6349	1026.6325	2	0	R.LLIDEAILK.C	(Ions score 42)
108 - 116	514.3247	1026.6349	1026.6325	2	0	R.LLIDEAILK.C	(Ions score 32)
108 - 116	514.3247	1026.6349	1026.6325	2	0	R.LLIDEAILK.C	(Ions score 21)
108 - 116	514.3247	1026.6349	1026.6325	2	0	R.LLIDEAILK.C	(Ions score 32)
108 - 116	514.3247	1026.6349	1026.6325	2	0	R.LLIDEAILK.C	(Ions score 23)
108 - 116	514.3247	1026.6349	1026.6325	2	0	R.LLIDEAILK.C	(Ions score 24)
108 - 116	514.3248	1026.6350	1026.6325	3	0	R.LLIDEAILK.C	(Ions score 32)
108 - 116	514.3248	1026.6351	1026.6325	3	0	R.LLIDEAILK.C	(Ions score 32)
108 - 116	514.3248	1026.6351	1026.6325	3	0	R.LLIDEAILK.C	(Ions score 23)
108 - 116	514.3248	1026.6351	1026.6325	3	0	R.LLIDEAILK.C	(Ions score 32)
108 - 116	514.3248	1026.6351	1026.6325	3	0	R.LLIDEAILK.C	(Ions score 32)
108 - 116	514.3248	1026.6351	1026.6325	3	0	R.LLIDEAILK.C	(Ions score 32)
108 - 116	514.3248	1026.6351	1026.6325	3	0	R.LLIDEAILK.C	(Ions score 23)
108 - 116	514.3249	1026.6352	1026.6325	3	0	R.LLIDEAILK.C	(Ions score 46)

108 - 116	514.3249	1026.6352	1026.6325	3	0	R.LLIDEAILK.C	(Ions score 30)
108 - 116	514.3249	1026.6352	1026.6325	3	0	R.LLIDEAILK.C	(Ions score 32)
108 - 116	514.3249	1026.6352	1026.6325	3	0	R.LLIDEAILK.C	(Ions score 32)
108 - 116	514.3249	1026.6352	1026.6325	3	0	R.LLIDEAILK.C	(Ions score 49)
108 - 116	514.3249	1026.6352	1026.6325	3	0	R.LLIDEAILK.C	(Ions score 46)
108 - 116	514.3249	1026.6352	1026.6325	3	0	R.LLIDEAILK.C	(Ions score 29)
108 - 116	514.3249	1026.6352	1026.6325	3	0	R.LLIDEAILK.C	(Ions score 32)
108 - 116	514.3249	1026.6353	1026.6325	3	0	R.LLIDEAILK.C	(Ions score 32)
108 - 116	514.3249	1026.6353	1026.6325	3	0	R.LLIDEAILK.C	(Ions score 49)
108 - 116	514.3249	1026.6353	1026.6325	3	0	R.LLIDEAILK.C	(Ions score 34)
108 - 116	514.3250	1026.6354	1026.6325	3	0	R.LLIDEAILK.C	(Ions score 45)
108 - 116	514.3250	1026.6354	1026.6325	3	0	R.LLIDEAILK.C	(Ions score 32)
108 - 116	514.3250	1026.6354	1026.6325	3	0	R.LLIDEAILK.C	(Ions score 32)
108 - 116	514.3250	1026.6354	1026.6325	3	0	R.LLIDEAILK.C	(Ions score 35)
108 - 116	514.3250	1026.6354	1026.6325	3	0	R.LLIDEAILK.C	(Ions score 31)
108 - 116	514.3250	1026.6354	1026.6325	3	0	R.LLIDEAILK.C	(Ions score 32)
108 - 116	514.3250	1026.6354	1026.6325	3	0	R.LLIDEAILK.C	(Ions score 46)
108 - 116	514.3250	1026.6355	1026.6325	3	0	R.LLIDEAILK.C	(Ions score 24)
108 - 116	514.3250	1026.6355	1026.6325	3	0	R.LLIDEAILK.C	(Ions score 32)
108 - 116	514.3250	1026.6355	1026.6325	3	0	R.LLIDEAILK.C	(Ions score 32)
108 - 116	514.3250	1026.6355	1026.6325	3	0	R.LLIDEAILK.C	(Ions score 32)
108 - 116	514.3251	1026.6356	1026.6325	3	0	R.LLIDEAILK.C	(Ions score 24)
108 - 116	514.3251	1026.6356	1026.6325	3	0	R.LLIDEAILK.C	(Ions score 50)
108 - 116	514.3251	1026.6356	1026.6325	3	0	R.LLIDEAILK.C	(Ions score 45)
108 - 116	514.3251	1026.6356	1026.6325	3	0	R.LLIDEAILK.C	(Ions score 33)
108 - 116	514.3251	1026.6356	1026.6325	3	0	R.LLIDEAILK.C	(Ions score 24)
108 - 116	514.3251	1026.6356	1026.6325	3	0	R.LLIDEAILK.C	(Ions score 43)
108 - 116	514.3251	1026.6356	1026.6325	3	0	R.LLIDEAILK.C	(Ions score 23)
108 - 116	514.3251	1026.6356	1026.6325	3	0	R.LLIDEAILK.C	(Ions score 46)
108 - 116	514.3251	1026.6357	1026.6325	3	0	R.LLIDEAILK.C	(Ions score 50)
108 - 116	514.3251	1026.6357	1026.6325	3	0	R.LLIDEAILK.C	(Ions score 32)
108 - 116	514.3251	1026.6357	1026.6325	3	0	R.LLIDEAILK.C	(Ions score 24)
108 - 116	514.3251	1026.6357	1026.6325	3	0	R.LLIDEAILK.C	(Ions score 32)
108 - 116	514.3252	1026.6358	1026.6325	3	0	R.LLIDEAILK.C	(Ions score 21)
108 - 116	514.3252	1026.6359	1026.6325	3	0	R.LLIDEAILK.C	(Ions score 30)
108 - 116	514.3253	1026.6360	1026.6325	3	0	R.LLIDEAILK.C	(Ions score 24)
108 - 116	514.3253	1026.6360	1026.6325	3	0	R.LLIDEAILK.C	(Ions score 21)
108 - 116	514.3253	1026.6360	1026.6325	3	0	R.LLIDEAILK.C	(Ions score 23)
108 - 116	514.3253	1026.6360	1026.6325	3	0	R.LLIDEAILK.C	(Ions score 32)
108 - 116	514.3253	1026.6361	1026.6325	4	0	R.LLIDEAILK.C	(Ions score 32)
108 - 116	514.3253	1026.6361	1026.6325	4	0	R.LLIDEAILK.C	(Ions score 32)
108 - 116	514.3253	1026.6361	1026.6325	4	0	R.LLIDEAILK.C	(Ions score 45)
108 - 116	514.3254	1026.6362	1026.6325	4	0	R.LLIDEAILK.C	(Ions score 31)
108 - 116	514.3254	1026.6362	1026.6325	4	0	R.LLIDEAILK.C	(Ions score 33)
108 - 116	514.3254	1026.6362	1026.6325	4	0	R.LLIDEAILK.C	(Ions score 32)
108 - 116	514.3254	1026.6363	1026.6325	4	0	R.LLIDEAILK.C	(Ions score 32)
108 - 116	514.3254	1026.6363	1026.6325	4	0	R.LLIDEAILK.C	(Ions score 32)
108 - 116	514.3254	1026.6363	1026.6325	4	0	R.LLIDEAILK.C	(Ions score 32)
108 - 116	514.3254	1026.6363	1026.6325	4	0	R.LLIDEAILK.C	(Ions score 31)
108 - 116	514.3254	1026.6363	1026.6325	4	0	R.LLIDEAILK.C	(Ions score 32)
108 - 116	514.3254	1026.6363	1026.6325	4	0	R.LLIDEAILK.C	(Ions score 33)
108 - 116	514.3255	1026.6364	1026.6325	4	0	R.LLIDEAILK.C	(Ions score 32)
108 - 116	514.3255	1026.6364	1026.6325	4	0	R.LLIDEAILK.C	(Ions score 32)
108 - 116	514.3255	1026.6364	1026.6325	4	0	R.LLIDEAILK.C	(Ions score 35)
108 - 116	514.3255	1026.6364	1026.6325	4	0	R.LLIDEAILK.C	(Ions score 29)
108 - 116	514.3255	1026.6365	1026.6325	4	0	R.LLIDEAILK.C	(Ions score 32)
108 - 116	514.3255	1026.6365	1026.6325	4	0	R.LLIDEAILK.C	(Ions score 22)
108 - 116	514.3256	1026.6366	1026.6325	4	0	R.LLIDEAILK.C	(Ions score 25)

108 - 116	514.3256	1026.6366	1026.6325	4	0	R.LLIDEAILK.C	(Ions score 33)
108 - 116	514.3256	1026.6366	1026.6325	4	0	R.LLIDEAILK.C	(Ions score 23)
108 - 116	514.3256	1026.6366	1026.6325	4	0	R.LLIDEAILK.C	(Ions score 32)
108 - 116	514.3256	1026.6367	1026.6325	4	0	R.LLIDEAILK.C	(Ions score 34)
108 - 116	514.3257	1026.6368	1026.6325	4	0	R.LLIDEAILK.C	(Ions score 32)
108 - 116	514.3257	1026.6369	1026.6325	4	0	R.LLIDEAILK.C	(Ions score 32)
108 - 116	514.3257	1026.6369	1026.6325	4	0	R.LLIDEAILK.C	(Ions score 32)
108 - 116	514.3258	1026.6371	1026.6325	5	0	R.LLIDEAILK.C	(Ions score 24)
108 - 116	514.3259	1026.6372	1026.6325	5	0	R.LLIDEAILK.C	(Ions score 30)
108 - 116	514.3259	1026.6372	1026.6325	5	0	R.LLIDEAILK.C	(Ions score 24)
108 - 116	514.3259	1026.6373	1026.6325	5	0	R.LLIDEAILK.C	(Ions score 26)
108 - 121	553.6330	1657.8773	1657.8709	4	1	R.LLIDEAILKCDAER.I	(Ions score 47)
108 - 121	553.6335	1657.8788	1657.8709	5	1	R.LLIDEAILKCDAER.I	(Ions score 60)
108 - 121	850.9478	1699.8811	1699.8814	-0	1	R.LLIDEAILKCDAER.I	Acetylation (K) (Ions score 59)
108 - 121	850.9484	1699.8823	1699.8814	1	1	R.LLIDEAILKCDAER.I	Acetylation (K) (Ions score 59)
108 - 121	567.6362	1699.8867	1699.8814	3	1	R.LLIDEAILKCDAER.I	Acetylation (K) (Ions score 53)
122 - 128	429.7555	857.4964	857.4970	-1	1	R.IKLEAER.F	(Ions score 24)
122 - 128	429.7557	857.4969	857.4970	-0	1	R.IKLEAER.F	(Ions score 23)
122 - 128	429.7562	857.4979	857.4970	1	1	R.IKLEAER.F	(Ions score 23)
122 - 128	429.7564	857.4982	857.4970	1	1	R.IKLEAER.F	(Ions score 21)
122 - 128	429.7564	857.4982	857.4970	1	1	R.IKLEAER.F	(Ions score 27)
122 - 128	429.7566	857.4986	857.4970	2	1	R.IKLEAER.F	(Ions score 28)
122 - 128	429.7566	857.4986	857.4970	2	1	R.IKLEAER.F	(Ions score 25)
122 - 128	429.7566	857.4987	857.4970	2	1	R.IKLEAER.F	(Ions score 25)
122 - 128	429.7567	857.4988	857.4970	2	1	R.IKLEAER.F	(Ions score 21)
122 - 128	429.7570	857.4994	857.4970	3	1	R.IKLEAER.F	(Ions score 21)
122 - 128	429.7570	857.4995	857.4970	3	1	R.IKLEAER.F	(Ions score 25)
122 - 128	429.7571	857.4996	857.4970	3	1	R.IKLEAER.F	(Ions score 26)
122 - 128	429.7573	857.5001	857.4970	4	1	R.IKLEAER.F	(Ions score 24)
122 - 128	429.7574	857.5002	857.4970	4	1	R.IKLEAER.F	(Ions score 26)
122 - 128	450.7612	899.5078	899.5076	0	1	R.IKLEAER.F	Acetylation (K) (Ions score 25)
122 - 128	450.7612	899.5079	899.5076	0	1	R.IKLEAER.F	Acetylation (K) (Ions score 32)
122 - 128	450.7618	899.5090	899.5076	2	1	R.IKLEAER.F	Acetylation (K) (Ions score 23)
122 - 128	450.7624	899.5103	899.5076	3	1	R.IKLEAER.F	Acetylation (K) (Ions score 25)
124 - 133	638.8374	1275.6603	1275.6571	2	1	K.LEAERFENLR.E	(Ions score 31)
124 - 133	426.2275	1275.6607	1275.6571	3	1	K.LEAERFENLR.E	(Ions score 21)
124 - 133	638.8382	1275.6618	1275.6571	4	1	K.LEAERFENLR.E	(Ions score 22)
124 - 133	426.2279	1275.6619	1275.6571	4	1	K.LEAERFENLR.E	(Ions score 22)
134 - 154	577.0388	2304.1262	2304.1234	1	0	R.EIGNLLHPSVPISNDEDVDNK.V	(Ions score 23)
134 - 154	1153.0705	2304.1265	2304.1234	1	0	R.EIGNLLHPSVPISNDEDVDNK.V	(Ions score 71)
134 - 154	1153.0706	2304.1266	2304.1234	1	0	R.EIGNLLHPSVPISNDEDVDNK.V	(Ions score 63)
134 - 154	1153.0707	2304.1269	2304.1234	2	0	R.EIGNLLHPSVPISNDEDVDNK.V	(Ions score 75)
134 - 154	769.0497	2304.1272	2304.1234	2	0	R.EIGNLLHPSVPISNDEDVDNK.V	(Ions score 24)
134 - 154	1153.0711	2304.1277	2304.1234	2	0	R.EIGNLLHPSVPISNDEDVDNK.V	(Ions score 71)
134 - 154	769.0499	2304.1279	2304.1234	2	0	R.EIGNLLHPSVPISNDEDVDNK.V	(Ions score 57)
134 - 154	1153.0713	2304.1280	2304.1234	2	0	R.EIGNLLHPSVPISNDEDVDNK.V	(Ions score 63)
134 - 154	1153.0717	2304.1288	2304.1234	2	0	R.EIGNLLHPSVPISNDEDVDNK.V	(Ions score 61)
134 - 154	1153.0718	2304.1290	2304.1234	2	0	R.EIGNLLHPSVPISNDEDVDNK.V	(Ions score 70)
134 - 154	769.0503	2304.1292	2304.1234	3	0	R.EIGNLLHPSVPISNDEDVDNK.V	(Ions score 63)
134 - 154	769.0503	2304.1292	2304.1234	3	0	R.EIGNLLHPSVPISNDEDVDNK.V	(Ions score 58)
134 - 154	769.0503	2304.1292	2304.1234	3	0	R.EIGNLLHPSVPISNDEDVDNK.V	(Ions score 82)
134 - 154	1153.0719	2304.1292	2304.1234	3	0	R.EIGNLLHPSVPISNDEDVDNK.V	(Ions score 58)
134 - 154	769.0504	2304.1294	2304.1234	3	0	R.EIGNLLHPSVPISNDEDVDNK.V	(Ions score 78)
134 - 154	769.0505	2304.1295	2304.1234	3	0	R.EIGNLLHPSVPISNDEDVDNK.V	(Ions score 65)
134 - 154	1153.0722	2304.1298	2304.1234	3	0	R.EIGNLLHPSVPISNDEDVDNK.V	(Ions score 71)
134 - 154	769.0508	2304.1307	2304.1234	3	0	R.EIGNLLHPSVPISNDEDVDNK.V	(Ions score 63)
134 - 154	769.0509	2304.1308	2304.1234	3	0	R.EIGNLLHPSVPISNDEDVDNK.V	(Ions score 66)
134 - 154	769.0511	2304.1314	2304.1234	3	0	R.EIGNLLHPSVPISNDEDVDNK.V	(Ions score 70)

134 - 154	769.0512	2304.1316	2304.1234	4	0	R.EIGNLLHPSVPISNDEDVDNK.V	(Ions score 69)
134 - 157	897.1178	2688.3315	2688.3355	-1	1	R.EIGNLLHPSVPISNDEDVDNKVER.I	(Ions score 77)
134 - 157	1345.1733	2688.3321	2688.3355	-1	1	R.EIGNLLHPSVPISNDEDVDNKVER.I	(Ions score 51)
134 - 157	897.1181	2688.3326	2688.3355	-1	1	R.EIGNLLHPSVPISNDEDVDNKVER.I	(Ions score 24)
134 - 157	673.0907	2688.3337	2688.3355	-1	1	R.EIGNLLHPSVPISNDEDVDNKVER.I	(Ions score 60)
134 - 157	673.0908	2688.3339	2688.3355	-1	1	R.EIGNLLHPSVPISNDEDVDNKVER.I	(Ions score 61)
134 - 157	673.0908	2688.3342	2688.3355	-0	1	R.EIGNLLHPSVPISNDEDVDNKVER.I	(Ions score 34)
134 - 157	897.1189	2688.3348	2688.3355	-0	1	R.EIGNLLHPSVPISNDEDVDNKVER.I	(Ions score 93)
134 - 157	897.1189	2688.3348	2688.3355	-0	1	R.EIGNLLHPSVPISNDEDVDNKVER.I	(Ions score 59)
134 - 157	673.0910	2688.3350	2688.3355	-0	1	R.EIGNLLHPSVPISNDEDVDNKVER.I	(Ions score 23)
134 - 157	673.0911	2688.3352	2688.3355	-0	1	R.EIGNLLHPSVPISNDEDVDNKVER.I	(Ions score 23)
134 - 157	673.0912	2688.3359	2688.3355	0	1	R.EIGNLLHPSVPISNDEDVDNKVER.I	(Ions score 31)
134 - 157	673.0913	2688.3360	2688.3355	0	1	R.EIGNLLHPSVPISNDEDVDNKVER.I	(Ions score 34)
134 - 157	1345.1753	2688.3361	2688.3355	0	1	R.EIGNLLHPSVPISNDEDVDNKVER.I	(Ions score 74)
134 - 157	897.1194	2688.3365	2688.3355	0	1	R.EIGNLLHPSVPISNDEDVDNKVER.I	(Ions score 35)
134 - 157	1345.1756	2688.3366	2688.3355	0	1	R.EIGNLLHPSVPISNDEDVDNKVER.I	(Ions score 53)
134 - 157	897.1195	2688.3367	2688.3355	0	1	R.EIGNLLHPSVPISNDEDVDNKVER.I	(Ions score 81)
134 - 157	673.0915	2688.3367	2688.3355	0	1	R.EIGNLLHPSVPISNDEDVDNKVER.I	(Ions score 36)
134 - 157	897.1196	2688.3370	2688.3355	1	1	R.EIGNLLHPSVPISNDEDVDNKVER.I	(Ions score 28)
134 - 157	673.0916	2688.3372	2688.3355	1	1	R.EIGNLLHPSVPISNDEDVDNKVER.I	(Ions score 51)
134 - 157	673.0916	2688.3373	2688.3355	1	1	R.EIGNLLHPSVPISNDEDVDNKVER.I	(Ions score 48)
134 - 157	1345.1759	2688.3373	2688.3355	1	1	R.EIGNLLHPSVPISNDEDVDNKVER.I	(Ions score 38)
134 - 157	1345.1760	2688.3374	2688.3355	1	1	R.EIGNLLHPSVPISNDEDVDNKVER.I	(Ions score 69)
134 - 157	897.1198	2688.3375	2688.3355	1	1	R.EIGNLLHPSVPISNDEDVDNKVER.I	(Ions score 79)
134 - 157	1345.1761	2688.3377	2688.3355	1	1	R.EIGNLLHPSVPISNDEDVDNKVER.I	(Ions score 75)
134 - 157	673.0917	2688.3379	2688.3355	1	1	R.EIGNLLHPSVPISNDEDVDNKVER.I	(Ions score 33)
134 - 157	673.0918	2688.3380	2688.3355	1	1	R.EIGNLLHPSVPISNDEDVDNKVER.I	(Ions score 51)
134 - 157	673.0918	2688.3380	2688.3355	1	1	R.EIGNLLHPSVPISNDEDVDNKVER.I	(Ions score 61)
134 - 157	673.0918	2688.3381	2688.3355	1	1	R.EIGNLLHPSVPISNDEDVDNKVER.I	(Ions score 41)
134 - 157	673.0918	2688.3383	2688.3355	1	1	R.EIGNLLHPSVPISNDEDVDNKVER.I	(Ions score 38)
134 - 157	673.0919	2688.3385	2688.3355	1	1	R.EIGNLLHPSVPISNDEDVDNKVER.I	(Ions score 38)
134 - 157	673.0919	2688.3385	2688.3355	1	1	R.EIGNLLHPSVPISNDEDVDNKVER.I	(Ions score 26)
134 - 157	673.0919	2688.3385	2688.3355	1	1	R.EIGNLLHPSVPISNDEDVDNKVER.I	(Ions score 44)
134 - 157	1345.1765	2688.3385	2688.3355	1	1	R.EIGNLLHPSVPISNDEDVDNKVER.I	(Ions score 67)
134 - 157	897.1202	2688.3386	2688.3355	1	1	R.EIGNLLHPSVPISNDEDVDNKVER.I	(Ions score 46)
134 - 157	673.0919	2688.3387	2688.3355	1	1	R.EIGNLLHPSVPISNDEDVDNKVER.I	(Ions score 34)
134 - 157	673.0920	2688.3388	2688.3355	1	1	R.EIGNLLHPSVPISNDEDVDNKVER.I	(Ions score 38)
134 - 157	897.1202	2688.3388	2688.3355	1	1	R.EIGNLLHPSVPISNDEDVDNKVER.I	(Ions score 64)
134 - 157	897.1203	2688.3389	2688.3355	1	1	R.EIGNLLHPSVPISNDEDVDNKVER.I	(Ions score 22)
134 - 157	673.0920	2688.3390	2688.3355	1	1	R.EIGNLLHPSVPISNDEDVDNKVER.I	(Ions score 45)
134 - 157	673.0920	2688.3390	2688.3355	1	1	R.EIGNLLHPSVPISNDEDVDNKVER.I	(Ions score 27)
134 - 157	673.0920	2688.3391	2688.3355	1	1	R.EIGNLLHPSVPISNDEDVDNKVER.I	(Ions score 48)
134 - 157	673.0920	2688.3391	2688.3355	1	1	R.EIGNLLHPSVPISNDEDVDNKVER.I	(Ions score 61)
134 - 157	673.0921	2688.3392	2688.3355	1	1	R.EIGNLLHPSVPISNDEDVDNKVER.I	(Ions score 46)
134 - 157	673.0921	2688.3392	2688.3355	1	1	R.EIGNLLHPSVPISNDEDVDNKVER.I	(Ions score 41)
134 - 157	897.1204	2688.3393	2688.3355	1	1	R.EIGNLLHPSVPISNDEDVDNKVER.I	(Ions score 57)
134 - 157	897.1204	2688.3393	2688.3355	1	1	R.EIGNLLHPSVPISNDEDVDNKVER.I	(Ions score 28)
134 - 157	673.0921	2688.3393	2688.3355	1	1	R.EIGNLLHPSVPISNDEDVDNKVER.I	(Ions score 39)
134 - 157	897.1205	2688.3397	2688.3355	2	1	R.EIGNLLHPSVPISNDEDVDNKVER.I	(Ions score 93)
134 - 157	673.0922	2688.3398	2688.3355	2	1	R.EIGNLLHPSVPISNDEDVDNKVER.I	(Ions score 35)
134 - 157	673.0922	2688.3398	2688.3355	2	1	R.EIGNLLHPSVPISNDEDVDNKVER.I	(Ions score 37)
134 - 157	673.0923	2688.3399	2688.3355	2	1	R.EIGNLLHPSVPISNDEDVDNKVER.I	(Ions score 51)
134 - 157	673.0923	2688.3399	2688.3355	2	1	R.EIGNLLHPSVPISNDEDVDNKVER.I	(Ions score 35)
134 - 157	673.0923	2688.3400	2688.3355	2	1	R.EIGNLLHPSVPISNDEDVDNKVER.I	(Ions score 48)
134 - 157	673.0923	2688.3401	2688.3355	2	1	R.EIGNLLHPSVPISNDEDVDNKVER.I	(Ions score 48)
134 - 157	897.1206	2688.3401	2688.3355	2	1	R.EIGNLLHPSVPISNDEDVDNKVER.I	(Ions score 63)
134 - 157	673.0923	2688.3402	2688.3355	2	1	R.EIGNLLHPSVPISNDEDVDNKVER.I	(Ions score 61)
134 - 157	673.0924	2688.3403	2688.3355	2	1	R.EIGNLLHPSVPISNDEDVDNKVER.I	(Ions score 43)

134 - 157	673.0924	2688.3404	2688.3355	2	1	R.EIGNLLHPSVPISNDEDVDNKVER.I	(Ions score 53)
134 - 157	673.0924	2688.3404	2688.3355	2	1	R.EIGNLLHPSVPISNDEDVDNKVER.I	(Ions score 48)
134 - 157	897.1208	2688.3404	2688.3355	2	1	R.EIGNLLHPSVPISNDEDVDNKVER.I	(Ions score 36)
134 - 157	673.0924	2688.3404	2688.3355	2	1	R.EIGNLLHPSVPISNDEDVDNKVER.I	(Ions score 21)
134 - 157	897.1208	2688.3405	2688.3355	2	1	R.EIGNLLHPSVPISNDEDVDNKVER.I	(Ions score 52)
134 - 157	673.0924	2688.3406	2688.3355	2	1	R.EIGNLLHPSVPISNDEDVDNKVER.I	(Ions score 48)
134 - 157	673.0924	2688.3406	2688.3355	2	1	R.EIGNLLHPSVPISNDEDVDNKVER.I	(Ions score 32)
134 - 157	673.0925	2688.3408	2688.3355	2	1	R.EIGNLLHPSVPISNDEDVDNKVER.I	(Ions score 44)
134 - 157	673.0925	2688.3408	2688.3355	2	1	R.EIGNLLHPSVPISNDEDVDNKVER.I	(Ions score 48)
134 - 157	673.0925	2688.3409	2688.3355	2	1	R.EIGNLLHPSVPISNDEDVDNKVER.I	(Ions score 42)
134 - 157	897.1209	2688.3410	2688.3355	2	1	R.EIGNLLHPSVPISNDEDVDNKVER.I	(Ions score 65)
134 - 157	673.0925	2688.3410	2688.3355	2	1	R.EIGNLLHPSVPISNDEDVDNKVER.I	(Ions score 43)
134 - 157	673.0925	2688.3410	2688.3355	2	1	R.EIGNLLHPSVPISNDEDVDNKVER.I	(Ions score 50)
134 - 157	897.1210	2688.3411	2688.3355	2	1	R.EIGNLLHPSVPISNDEDVDNKVER.I	(Ions score 85)
134 - 157	673.0925	2688.3411	2688.3355	2	1	R.EIGNLLHPSVPISNDEDVDNKVER.I	(Ions score 51)
134 - 157	897.1210	2688.3411	2688.3355	2	1	R.EIGNLLHPSVPISNDEDVDNKVER.I	(Ions score 87)
134 - 157	673.0926	2688.3412	2688.3355	2	1	R.EIGNLLHPSVPISNDEDVDNKVER.I	(Ions score 30)
134 - 157	673.0926	2688.3412	2688.3355	2	1	R.EIGNLLHPSVPISNDEDVDNKVER.I	(Ions score 27)
134 - 157	673.0926	2688.3412	2688.3355	2	1	R.EIGNLLHPSVPISNDEDVDNKVER.I	(Ions score 39)
134 - 157	673.0926	2688.3412	2688.3355	2	1	R.EIGNLLHPSVPISNDEDVDNKVER.I	(Ions score 46)
134 - 157	673.0926	2688.3413	2688.3355	2	1	R.EIGNLLHPSVPISNDEDVDNKVER.I	(Ions score 51)
134 - 157	673.0926	2688.3413	2688.3355	2	1	R.EIGNLLHPSVPISNDEDVDNKVER.I	(Ions score 31)
134 - 157	673.0926	2688.3413	2688.3355	2	1	R.EIGNLLHPSVPISNDEDVDNKVER.I	(Ions score 48)
134 - 157	673.0926	2688.3413	2688.3355	2	1	R.EIGNLLHPSVPISNDEDVDNKVER.I	(Ions score 27)
134 - 157	673.0926	2688.3413	2688.3355	2	1	R.EIGNLLHPSVPISNDEDVDNKVER.I	(Ions score 34)
134 - 157	673.0926	2688.3413	2688.3355	2	1	R.EIGNLLHPSVPISNDEDVDNKVER.I	(Ions score 37)
134 - 157	673.0926	2688.3413	2688.3355	2	1	R.EIGNLLHPSVPISNDEDVDNKVER.I	(Ions score 48)
134 - 157	673.0926	2688.3414	2688.3355	2	1	R.EIGNLLHPSVPISNDEDVDNKVER.I	(Ions score 40)
134 - 157	538.6756	2688.3414	2688.3355	2	1	R.EIGNLLHPSVPISNDEDVDNKVER.I	(Ions score 42)
134 - 157	673.0926	2688.3414	2688.3355	2	1	R.EIGNLLHPSVPISNDEDVDNKVER.I	(Ions score 38)
134 - 157	673.0926	2688.3415	2688.3355	2	1	R.EIGNLLHPSVPISNDEDVDNKVER.I	(Ions score 51)
134 - 157	673.0927	2688.3415	2688.3355	2	1	R.EIGNLLHPSVPISNDEDVDNKVER.I	(Ions score 33)
134 - 157	673.0927	2688.3415	2688.3355	2	1	R.EIGNLLHPSVPISNDEDVDNKVER.I	(Ions score 29)
134 - 157	673.0927	2688.3416	2688.3355	2	1	R.EIGNLLHPSVPISNDEDVDNKVER.I	(Ions score 47)
134 - 157	673.0927	2688.3416	2688.3355	2	1	R.EIGNLLHPSVPISNDEDVDNKVER.I	(Ions score 45)
134 - 157	673.0927	2688.3416	2688.3355	2	1	R.EIGNLLHPSVPISNDEDVDNKVER.I	(Ions score 48)
134 - 157	673.0927	2688.3416	2688.3355	2	1	R.EIGNLLHPSVPISNDEDVDNKVER.I	(Ions score 46)
134 - 157	673.0927	2688.3417	2688.3355	2	1	R.EIGNLLHPSVPISNDEDVDNKVER.I	(Ions score 50)
134 - 157	673.0927	2688.3417	2688.3355	2	1	R.EIGNLLHPSVPISNDEDVDNKVER.I	(Ions score 46)
134 - 157	673.0927	2688.3417	2688.3355	2	1	R.EIGNLLHPSVPISNDEDVDNKVER.I	(Ions score 51)
134 - 157	673.0927	2688.3417	2688.3355	2	1	R.EIGNLLHPSVPISNDEDVDNKVER.I	(Ions score 48)
134 - 157	673.0927	2688.3418	2688.3355	2	1	R.EIGNLLHPSVPISNDEDVDNKVER.I	(Ions score 51)
134 - 157	673.0927	2688.3418	2688.3355	2	1	R.EIGNLLHPSVPISNDEDVDNKVER.I	(Ions score 51)
134 - 157	673.0927	2688.3418	2688.3355	2	1	R.EIGNLLHPSVPISNDEDVDNKVER.I	(Ions score 36)
134 - 157	1345.1782	2688.3418	2688.3355	2	1	R.EIGNLLHPSVPISNDEDVDNKVER.I	(Ions score 66)
134 - 157	673.0927	2688.3419	2688.3355	2	1	R.EIGNLLHPSVPISNDEDVDNKVER.I	(Ions score 51)
134 - 157	1345.1782	2688.3419	2688.3355	2	1	R.EIGNLLHPSVPISNDEDVDNKVER.I	(Ions score 75)
134 - 157	673.0928	2688.3420	2688.3355	2	1	R.EIGNLLHPSVPISNDEDVDNKVER.I	(Ions score 49)
134 - 157	673.0928	2688.3420	2688.3355	2	1	R.EIGNLLHPSVPISNDEDVDNKVER.I	(Ions score 51)
134 - 157	538.6757	2688.3421	2688.3355	2	1	R.EIGNLLHPSVPISNDEDVDNKVER.I	(Ions score 36)
134 - 157	673.0928	2688.3421	2688.3355	2	1	R.EIGNLLHPSVPISNDEDVDNKVER.I	(Ions score 46)
134 - 157	673.0928	2688.3421	2688.3355	2	1	R.EIGNLLHPSVPISNDEDVDNKVER.I	(Ions score 48)
134 - 157	673.0928	2688.3421	2688.3355	2	1	R.EIGNLLHPSVPISNDEDVDNKVER.I	(Ions score 35)
134 - 157	897.1213	2688.3421	2688.3355	2	1	R.EIGNLLHPSVPISNDEDVDNKVER.I	(Ions score 85)
134 - 157	673.0928	2688.3422	2688.3355	3	1	R.EIGNLLHPSVPISNDEDVDNKVER.I	(Ions score 44)
134 - 157	897.1213	2688.3422	2688.3355	3	1	R.EIGNLLHPSVPISNDEDVDNKVER.I	(Ions score 87)
134 - 157	673.0928	2688.3423	2688.3355	3	1	R.EIGNLLHPSVPISNDEDVDNKVER.I	(Ions score 60)
134 - 157	673.0928	2688.3423	2688.3355	3	1	R.EIGNLLHPSVPISNDEDVDNKVER.I	(Ions score 36)

134 - 157	673.0928	2688.3423	2688.3355	3	1	R.EIGNLLHPSVPISNDEDVDNKVER.I	(Ions score 50)
134 - 157	1345.1784	2688.3423	2688.3355	3	1	R.EIGNLLHPSVPISNDEDVDNKVER.I	(Ions score 61)
134 - 157	897.1214	2688.3424	2688.3355	3	1	R.EIGNLLHPSVPISNDEDVDNKVER.I	(Ions score 28)
134 - 157	897.1214	2688.3424	2688.3355	3	1	R.EIGNLLHPSVPISNDEDVDNKVER.I	(Ions score 53)
134 - 157	897.1214	2688.3424	2688.3355	3	1	R.EIGNLLHPSVPISNDEDVDNKVER.I	(Ions score 85)
134 - 157	673.0929	2688.3424	2688.3355	3	1	R.EIGNLLHPSVPISNDEDVDNKVER.I	(Ions score 38)
134 - 157	673.0929	2688.3424	2688.3355	3	1	R.EIGNLLHPSVPISNDEDVDNKVER.I	(Ions score 38)
134 - 157	673.0929	2688.3425	2688.3355	3	1	R.EIGNLLHPSVPISNDEDVDNKVER.I	(Ions score 45)
134 - 157	897.1214	2688.3425	2688.3355	3	1	R.EIGNLLHPSVPISNDEDVDNKVER.I	(Ions score 89)
134 - 157	673.0929	2688.3426	2688.3355	3	1	R.EIGNLLHPSVPISNDEDVDNKVER.I	(Ions score 33)
134 - 157	897.1215	2688.3426	2688.3355	3	1	R.EIGNLLHPSVPISNDEDVDNKVER.I	(Ions score 88)
134 - 157	673.0929	2688.3426	2688.3355	3	1	R.EIGNLLHPSVPISNDEDVDNKVER.I	(Ions score 37)
134 - 157	673.0929	2688.3426	2688.3355	3	1	R.EIGNLLHPSVPISNDEDVDNKVER.I	(Ions score 29)
134 - 157	673.0929	2688.3426	2688.3355	3	1	R.EIGNLLHPSVPISNDEDVDNKVER.I	(Ions score 34)
134 - 157	673.0929	2688.3426	2688.3355	3	1	R.EIGNLLHPSVPISNDEDVDNKVER.I	(Ions score 51)
134 - 157	897.1215	2688.3426	2688.3355	3	1	R.EIGNLLHPSVPISNDEDVDNKVER.I	(Ions score 85)
134 - 157	673.0929	2688.3426	2688.3355	3	1	R.EIGNLLHPSVPISNDEDVDNKVER.I	(Ions score 54)
134 - 157	897.1215	2688.3427	2688.3355	3	1	R.EIGNLLHPSVPISNDEDVDNKVER.I	(Ions score 94)
134 - 157	673.0930	2688.3427	2688.3355	3	1	R.EIGNLLHPSVPISNDEDVDNKVER.I	(Ions score 48)
134 - 157	673.0930	2688.3427	2688.3355	3	1	R.EIGNLLHPSVPISNDEDVDNKVER.I	(Ions score 36)
134 - 157	897.1215	2688.3427	2688.3355	3	1	R.EIGNLLHPSVPISNDEDVDNKVER.I	(Ions score 85)
134 - 157	673.0930	2688.3427	2688.3355	3	1	R.EIGNLLHPSVPISNDEDVDNKVER.I	(Ions score 48)
134 - 157	897.1215	2688.3427	2688.3355	3	1	R.EIGNLLHPSVPISNDEDVDNKVER.I	(Ions score 105)
134 - 157	897.1215	2688.3428	2688.3355	3	1	R.EIGNLLHPSVPISNDEDVDNKVER.I	(Ions score 118)
134 - 157	673.0930	2688.3428	2688.3355	3	1	R.EIGNLLHPSVPISNDEDVDNKVER.I	(Ions score 36)
134 - 157	673.0930	2688.3428	2688.3355	3	1	R.EIGNLLHPSVPISNDEDVDNKVER.I	(Ions score 51)
134 - 157	673.0930	2688.3429	2688.3355	3	1	R.EIGNLLHPSVPISNDEDVDNKVER.I	(Ions score 37)
134 - 157	673.0930	2688.3429	2688.3355	3	1	R.EIGNLLHPSVPISNDEDVDNKVER.I	(Ions score 48)
134 - 157	673.0930	2688.3430	2688.3355	3	1	R.EIGNLLHPSVPISNDEDVDNKVER.I	(Ions score 36)
134 - 157	673.0930	2688.3430	2688.3355	3	1	R.EIGNLLHPSVPISNDEDVDNKVER.I	(Ions score 41)
134 - 157	897.1216	2688.3430	2688.3355	3	1	R.EIGNLLHPSVPISNDEDVDNKVER.I	(Ions score 101)
134 - 157	673.0930	2688.3430	2688.3355	3	1	R.EIGNLLHPSVPISNDEDVDNKVER.I	(Ions score 51)
134 - 157	673.0930	2688.3431	2688.3355	3	1	R.EIGNLLHPSVPISNDEDVDNKVER.I	(Ions score 50)
134 - 157	897.1216	2688.3431	2688.3355	3	1	R.EIGNLLHPSVPISNDEDVDNKVER.I	(Ions score 93)
134 - 157	673.0931	2688.3431	2688.3355	3	1	R.EIGNLLHPSVPISNDEDVDNKVER.I	(Ions score 37)
134 - 157	1345.1788	2688.3431	2688.3355	3	1	R.EIGNLLHPSVPISNDEDVDNKVER.I	(Ions score 65)
134 - 157	673.0931	2688.3432	2688.3355	3	1	R.EIGNLLHPSVPISNDEDVDNKVER.I	(Ions score 50)
134 - 157	673.0931	2688.3432	2688.3355	3	1	R.EIGNLLHPSVPISNDEDVDNKVER.I	(Ions score 45)
134 - 157	897.1217	2688.3432	2688.3355	3	1	R.EIGNLLHPSVPISNDEDVDNKVER.I	(Ions score 63)
134 - 157	897.1217	2688.3433	2688.3355	3	1	R.EIGNLLHPSVPISNDEDVDNKVER.I	(Ions score 85)
134 - 157	673.0931	2688.3433	2688.3355	3	1	R.EIGNLLHPSVPISNDEDVDNKVER.I	(Ions score 31)
134 - 157	897.1217	2688.3434	2688.3355	3	1	R.EIGNLLHPSVPISNDEDVDNKVER.I	(Ions score 101)
134 - 157	673.0931	2688.3434	2688.3355	3	1	R.EIGNLLHPSVPISNDEDVDNKVER.I	(Ions score 51)
134 - 157	673.0931	2688.3434	2688.3355	3	1	R.EIGNLLHPSVPISNDEDVDNKVER.I	(Ions score 38)
134 - 157	673.0931	2688.3434	2688.3355	3	1	R.EIGNLLHPSVPISNDEDVDNKVER.I	(Ions score 36)
134 - 157	673.0931	2688.3435	2688.3355	3	1	R.EIGNLLHPSVPISNDEDVDNKVER.I	(Ions score 32)
134 - 157	1345.1790	2688.3435	2688.3355	3	1	R.EIGNLLHPSVPISNDEDVDNKVER.I	(Ions score 63)
134 - 157	673.0931	2688.3435	2688.3355	3	1	R.EIGNLLHPSVPISNDEDVDNKVER.I	(Ions score 32)
134 - 157	673.0931	2688.3435	2688.3355	3	1	R.EIGNLLHPSVPISNDEDVDNKVER.I	(Ions score 38)
134 - 157	673.0932	2688.3435	2688.3355	3	1	R.EIGNLLHPSVPISNDEDVDNKVER.I	(Ions score 48)
134 - 157	673.0932	2688.3435	2688.3355	3	1	R.EIGNLLHPSVPISNDEDVDNKVER.I	(Ions score 30)
134 - 157	673.0932	2688.3436	2688.3355	3	1	R.EIGNLLHPSVPISNDEDVDNKVER.I	(Ions score 50)
134 - 157	673.0932	2688.3436	2688.3355	3	1	R.EIGNLLHPSVPISNDEDVDNKVER.I	(Ions score 47)
134 - 157	673.0932	2688.3436	2688.3355	3	1	R.EIGNLLHPSVPISNDEDVDNKVER.I	(Ions score 47)
134 - 157	673.0932	2688.3436	2688.3355	3	1	R.EIGNLLHPSVPISNDEDVDNKVER.I	(Ions score 35)
134 - 157	897.1218	2688.3436	2688.3355	3	1	R.EIGNLLHPSVPISNDEDVDNKVER.I	(Ions score 30)
134 - 157	673.0932	2688.3437	2688.3355	3	1	R.EIGNLLHPSVPISNDEDVDNKVER.I	(Ions score 48)
134 - 157	897.1218	2688.3437	2688.3355	3	1	R.EIGNLLHPSVPISNDEDVDNKVER.I	(Ions score 71)

134 - 157	673.0932	2688.3438	2688.3355	3	1	R.EIGNLLHPSVPISNDEDVDNKVER.I	(Ions score 38)
134 - 157	673.0932	2688.3438	2688.3355	3	1	R.EIGNLLHPSVPISNDEDVDNKVER.I	(Ions score 35)
134 - 157	897.1219	2688.3438	2688.3355	3	1	R.EIGNLLHPSVPISNDEDVDNKVER.I	(Ions score 101)
134 - 157	673.0933	2688.3440	2688.3355	3	1	R.EIGNLLHPSVPISNDEDVDNKVER.I	(Ions score 38)
134 - 157	673.0933	2688.3440	2688.3355	3	1	R.EIGNLLHPSVPISNDEDVDNKVER.I	(Ions score 48)
134 - 157	673.0933	2688.3441	2688.3355	3	1	R.EIGNLLHPSVPISNDEDVDNKVER.I	(Ions score 32)
134 - 157	897.1220	2688.3442	2688.3355	3	1	R.EIGNLLHPSVPISNDEDVDNKVER.I	(Ions score 101)
134 - 157	673.0933	2688.3442	2688.3355	3	1	R.EIGNLLHPSVPISNDEDVDNKVER.I	(Ions score 58)
134 - 157	897.1220	2688.3443	2688.3355	3	1	R.EIGNLLHPSVPISNDEDVDNKVER.I	(Ions score 101)
134 - 157	897.1220	2688.3443	2688.3355	3	1	R.EIGNLLHPSVPISNDEDVDNKVER.I	(Ions score 89)
134 - 157	673.0934	2688.3443	2688.3355	3	1	R.EIGNLLHPSVPISNDEDVDNKVER.I	(Ions score 50)
134 - 157	673.0934	2688.3443	2688.3355	3	1	R.EIGNLLHPSVPISNDEDVDNKVER.I	(Ions score 30)
134 - 157	673.0934	2688.3443	2688.3355	3	1	R.EIGNLLHPSVPISNDEDVDNKVER.I	(Ions score 61)
134 - 157	673.0934	2688.3444	2688.3355	3	1	R.EIGNLLHPSVPISNDEDVDNKVER.I	(Ions score 33)
134 - 157	673.0934	2688.3444	2688.3355	3	1	R.EIGNLLHPSVPISNDEDVDNKVER.I	(Ions score 47)
134 - 157	673.0934	2688.3444	2688.3355	3	1	R.EIGNLLHPSVPISNDEDVDNKVER.I	(Ions score 35)
134 - 157	673.0934	2688.3444	2688.3355	3	1	R.EIGNLLHPSVPISNDEDVDNKVER.I	(Ions score 46)
134 - 157	673.0934	2688.3445	2688.3355	3	1	R.EIGNLLHPSVPISNDEDVDNKVER.I	(Ions score 58)
134 - 157	673.0934	2688.3446	2688.3355	3	1	R.EIGNLLHPSVPISNDEDVDNKVER.I	(Ions score 48)
134 - 157	673.0934	2688.3446	2688.3355	3	1	R.EIGNLLHPSVPISNDEDVDNKVER.I	(Ions score 38)
134 - 157	897.1221	2688.3446	2688.3355	3	1	R.EIGNLLHPSVPISNDEDVDNKVER.I	(Ions score 62)
134 - 157	673.0934	2688.3446	2688.3355	3	1	R.EIGNLLHPSVPISNDEDVDNKVER.I	(Ions score 48)
134 - 157	897.1221	2688.3446	2688.3355	3	1	R.EIGNLLHPSVPISNDEDVDNKVER.I	(Ions score 100)
134 - 157	673.0934	2688.3446	2688.3355	3	1	R.EIGNLLHPSVPISNDEDVDNKVER.I	(Ions score 61)
134 - 157	673.0934	2688.3447	2688.3355	3	1	R.EIGNLLHPSVPISNDEDVDNKVER.I	(Ions score 61)
134 - 157	897.1222	2688.3447	2688.3355	3	1	R.EIGNLLHPSVPISNDEDVDNKVER.I	(Ions score 101)
134 - 157	673.0934	2688.3447	2688.3355	3	1	R.EIGNLLHPSVPISNDEDVDNKVER.I	(Ions score 50)
134 - 157	673.0935	2688.3448	2688.3355	3	1	R.EIGNLLHPSVPISNDEDVDNKVER.I	(Ions score 36)
134 - 157	673.0935	2688.3448	2688.3355	3	1	R.EIGNLLHPSVPISNDEDVDNKVER.I	(Ions score 48)
134 - 157	1345.1797	2688.3449	2688.3355	4	1	R.EIGNLLHPSVPISNDEDVDNKVER.I	(Ions score 56)
134 - 157	673.0935	2688.3449	2688.3355	4	1	R.EIGNLLHPSVPISNDEDVDNKVER.I	(Ions score 31)
134 - 157	897.1223	2688.3449	2688.3355	4	1	R.EIGNLLHPSVPISNDEDVDNKVER.I	(Ions score 71)
134 - 157	673.0935	2688.3450	2688.3355	4	1	R.EIGNLLHPSVPISNDEDVDNKVER.I	(Ions score 48)
134 - 157	673.0935	2688.3450	2688.3355	4	1	R.EIGNLLHPSVPISNDEDVDNKVER.I	(Ions score 47)
134 - 157	673.0935	2688.3450	2688.3355	4	1	R.EIGNLLHPSVPISNDEDVDNKVER.I	(Ions score 33)
134 - 157	673.0935	2688.3450	2688.3355	4	1	R.EIGNLLHPSVPISNDEDVDNKVER.I	(Ions score 46)
134 - 157	673.0935	2688.3451	2688.3355	4	1	R.EIGNLLHPSVPISNDEDVDNKVER.I	(Ions score 61)
134 - 157	897.1223	2688.3451	2688.3355	4	1	R.EIGNLLHPSVPISNDEDVDNKVER.I	(Ions score 93)
134 - 157	897.1223	2688.3451	2688.3355	4	1	R.EIGNLLHPSVPISNDEDVDNKVER.I	(Ions score 85)
134 - 157	1345.1798	2688.3451	2688.3355	4	1	R.EIGNLLHPSVPISNDEDVDNKVER.I	(Ions score 44)
134 - 157	673.0936	2688.3452	2688.3355	4	1	R.EIGNLLHPSVPISNDEDVDNKVER.I	(Ions score 50)
134 - 157	673.0936	2688.3452	2688.3355	4	1	R.EIGNLLHPSVPISNDEDVDNKVER.I	(Ions score 32)
134 - 157	673.0936	2688.3452	2688.3355	4	1	R.EIGNLLHPSVPISNDEDVDNKVER.I	(Ions score 22)
134 - 157	673.0936	2688.3453	2688.3355	4	1	R.EIGNLLHPSVPISNDEDVDNKVER.I	(Ions score 58)
134 - 157	673.0936	2688.3453	2688.3355	4	1	R.EIGNLLHPSVPISNDEDVDNKVER.I	(Ions score 48)
134 - 157	673.0936	2688.3453	2688.3355	4	1	R.EIGNLLHPSVPISNDEDVDNKVER.I	(Ions score 50)
134 - 157	673.0936	2688.3453	2688.3355	4	1	R.EIGNLLHPSVPISNDEDVDNKVER.I	(Ions score 48)
134 - 157	1345.1800	2688.3454	2688.3355	4	1	R.EIGNLLHPSVPISNDEDVDNKVER.I	(Ions score 72)
134 - 157	673.0936	2688.3454	2688.3355	4	1	R.EIGNLLHPSVPISNDEDVDNKVER.I	(Ions score 57)
134 - 157	897.1224	2688.3454	2688.3355	4	1	R.EIGNLLHPSVPISNDEDVDNKVER.I	(Ions score 72)
134 - 157	673.0936	2688.3454	2688.3355	4	1	R.EIGNLLHPSVPISNDEDVDNKVER.I	(Ions score 38)
134 - 157	897.1224	2688.3454	2688.3355	4	1	R.EIGNLLHPSVPISNDEDVDNKVER.I	(Ions score 101)
134 - 157	673.0936	2688.3455	2688.3355	4	1	R.EIGNLLHPSVPISNDEDVDNKVER.I	(Ions score 40)
134 - 157	897.1224	2688.3455	2688.3355	4	1	R.EIGNLLHPSVPISNDEDVDNKVER.I	(Ions score 59)
134 - 157	673.0937	2688.3455	2688.3355	4	1	R.EIGNLLHPSVPISNDEDVDNKVER.I	(Ions score 51)
134 - 157	673.0937	2688.3455	2688.3355	4	1	R.EIGNLLHPSVPISNDEDVDNKVER.I	(Ions score 47)
134 - 157	673.0937	2688.3455	2688.3355	4	1	R.EIGNLLHPSVPISNDEDVDNKVER.I	(Ions score 31)
134 - 157	673.0937	2688.3456	2688.3355	4	1	R.EIGNLLHPSVPISNDEDVDNKVER.I	(Ions score 50)

134 - 157	673.0937	2688.3456	2688.3355	4	1	R.EIGNLLHPSVPISNDEDVDNKVER.I	(Ions score 39)
134 - 157	897.1225	2688.3457	2688.3355	4	1	R.EIGNLLHPSVPISNDEDVDNKVER.I	(Ions score 105)
134 - 157	673.0937	2688.3458	2688.3355	4	1	R.EIGNLLHPSVPISNDEDVDNKVER.I	(Ions score 43)
134 - 157	1345.1802	2688.3458	2688.3355	4	1	R.EIGNLLHPSVPISNDEDVDNKVER.I	(Ions score 83)
134 - 157	673.0937	2688.3458	2688.3355	4	1	R.EIGNLLHPSVPISNDEDVDNKVER.I	(Ions score 51)
134 - 157	897.1226	2688.3459	2688.3355	4	1	R.EIGNLLHPSVPISNDEDVDNKVER.I	(Ions score 67)
134 - 157	673.0938	2688.3459	2688.3355	4	1	R.EIGNLLHPSVPISNDEDVDNKVER.I	(Ions score 51)
134 - 157	673.0938	2688.3460	2688.3355	4	1	R.EIGNLLHPSVPISNDEDVDNKVER.I	(Ions score 38)
134 - 157	897.1226	2688.3460	2688.3355	4	1	R.EIGNLLHPSVPISNDEDVDNKVER.I	(Ions score 72)
134 - 157	673.0938	2688.3461	2688.3355	4	1	R.EIGNLLHPSVPISNDEDVDNKVER.I	(Ions score 24)
134 - 157	897.1227	2688.3462	2688.3355	4	1	R.EIGNLLHPSVPISNDEDVDNKVER.I	(Ions score 62)
134 - 157	673.0939	2688.3463	2688.3355	4	1	R.EIGNLLHPSVPISNDEDVDNKVER.I	(Ions score 42)
134 - 157	673.0939	2688.3464	2688.3355	4	1	R.EIGNLLHPSVPISNDEDVDNKVER.I	(Ions score 44)
134 - 157	1345.1805	2688.3464	2688.3355	4	1	R.EIGNLLHPSVPISNDEDVDNKVER.I	(Ions score 72)
134 - 157	673.0939	2688.3464	2688.3355	4	1	R.EIGNLLHPSVPISNDEDVDNKVER.I	(Ions score 39)
134 - 157	673.0939	2688.3465	2688.3355	4	1	R.EIGNLLHPSVPISNDEDVDNKVER.I	(Ions score 26)
134 - 157	673.0939	2688.3465	2688.3355	4	1	R.EIGNLLHPSVPISNDEDVDNKVER.I	(Ions score 50)
134 - 157	673.0939	2688.3465	2688.3355	4	1	R.EIGNLLHPSVPISNDEDVDNKVER.I	(Ions score 50)
134 - 157	673.0939	2688.3466	2688.3355	4	1	R.EIGNLLHPSVPISNDEDVDNKVER.I	(Ions score 30)
134 - 157	673.0939	2688.3466	2688.3355	4	1	R.EIGNLLHPSVPISNDEDVDNKVER.I	(Ions score 45)
134 - 157	673.0939	2688.3466	2688.3355	4	1	R.EIGNLLHPSVPISNDEDVDNKVER.I	(Ions score 38)
134 - 157	673.0939	2688.3466	2688.3355	4	1	R.EIGNLLHPSVPISNDEDVDNKVER.I	(Ions score 48)
134 - 157	673.0939	2688.3466	2688.3355	4	1	R.EIGNLLHPSVPISNDEDVDNKVER.I	(Ions score 25)
134 - 157	673.0939	2688.3467	2688.3355	4	1	R.EIGNLLHPSVPISNDEDVDNKVER.I	(Ions score 45)
134 - 157	673.0939	2688.3467	2688.3355	4	1	R.EIGNLLHPSVPISNDEDVDNKVER.I	(Ions score 65)
134 - 157	673.0940	2688.3467	2688.3355	4	1	R.EIGNLLHPSVPISNDEDVDNKVER.I	(Ions score 35)
134 - 157	673.0940	2688.3467	2688.3355	4	1	R.EIGNLLHPSVPISNDEDVDNKVER.I	(Ions score 51)
134 - 157	673.0940	2688.3468	2688.3355	4	1	R.EIGNLLHPSVPISNDEDVDNKVER.I	(Ions score 36)
134 - 157	673.0940	2688.3468	2688.3355	4	1	R.EIGNLLHPSVPISNDEDVDNKVER.I	(Ions score 45)
134 - 157	673.0940	2688.3468	2688.3355	4	1	R.EIGNLLHPSVPISNDEDVDNKVER.I	(Ions score 51)
134 - 157	673.0940	2688.3468	2688.3355	4	1	R.EIGNLLHPSVPISNDEDVDNKVER.I	(Ions score 36)
134 - 157	673.0940	2688.3469	2688.3355	4	1	R.EIGNLLHPSVPISNDEDVDNKVER.I	(Ions score 31)
134 - 157	673.0940	2688.3469	2688.3355	4	1	R.EIGNLLHPSVPISNDEDVDNKVER.I	(Ions score 49)
134 - 157	897.1229	2688.3469	2688.3355	4	1	R.EIGNLLHPSVPISNDEDVDNKVER.I	(Ions score 85)
134 - 157	673.0940	2688.3470	2688.3355	4	1	R.EIGNLLHPSVPISNDEDVDNKVER.I	(Ions score 48)
134 - 157	673.0940	2688.3470	2688.3355	4	1	R.EIGNLLHPSVPISNDEDVDNKVER.I	(Ions score 24)
134 - 157	673.0940	2688.3471	2688.3355	4	1	R.EIGNLLHPSVPISNDEDVDNKVER.I	(Ions score 32)
134 - 157	897.1230	2688.3472	2688.3355	4	1	R.EIGNLLHPSVPISNDEDVDNKVER.I	(Ions score 57)
134 - 157	673.0941	2688.3472	2688.3355	4	1	R.EIGNLLHPSVPISNDEDVDNKVER.I	(Ions score 57)
134 - 157	897.1230	2688.3473	2688.3355	4	1	R.EIGNLLHPSVPISNDEDVDNKVER.I	(Ions score 68)
134 - 157	673.0941	2688.3473	2688.3355	4	1	R.EIGNLLHPSVPISNDEDVDNKVER.I	(Ions score 48)
134 - 157	673.0941	2688.3475	2688.3355	4	1	R.EIGNLLHPSVPISNDEDVDNKVER.I	(Ions score 38)
134 - 157	673.0942	2688.3475	2688.3355	4	1	R.EIGNLLHPSVPISNDEDVDNKVER.I	(Ions score 48)
134 - 157	673.0942	2688.3475	2688.3355	4	1	R.EIGNLLHPSVPISNDEDVDNKVER.I	(Ions score 36)
134 - 157	673.0942	2688.3476	2688.3355	5	1	R.EIGNLLHPSVPISNDEDVDNKVER.I	(Ions score 51)
134 - 157	897.1232	2688.3478	2688.3355	5	1	R.EIGNLLHPSVPISNDEDVDNKVER.I	(Ions score 69)
134 - 157	673.0942	2688.3478	2688.3355	5	1	R.EIGNLLHPSVPISNDEDVDNKVER.I	(Ions score 48)
134 - 157	673.0943	2688.3479	2688.3355	5	1	R.EIGNLLHPSVPISNDEDVDNKVER.I	(Ions score 47)
134 - 157	673.0943	2688.3482	2688.3355	5	1	R.EIGNLLHPSVPISNDEDVDNKVER.I	(Ions score 48)
134 - 157	673.0943	2688.3482	2688.3355	5	1	R.EIGNLLHPSVPISNDEDVDNKVER.I	(Ions score 24)
134 - 157	673.0943	2688.3482	2688.3355	5	1	R.EIGNLLHPSVPISNDEDVDNKVER.I	(Ions score 61)
134 - 157	897.1234	2688.3484	2688.3355	5	1	R.EIGNLLHPSVPISNDEDVDNKVER.I	(Ions score 89)
134 - 157	673.0944	2688.3484	2688.3355	5	1	R.EIGNLLHPSVPISNDEDVDNKVER.I	(Ions score 48)
134 - 157	673.0944	2688.3485	2688.3355	5	1	R.EIGNLLHPSVPISNDEDVDNKVER.I	(Ions score 52)
134 - 157	673.0944	2688.3485	2688.3355	5	1	R.EIGNLLHPSVPISNDEDVDNKVER.I	(Ions score 33)
134 - 157	897.1234	2688.3485	2688.3355	5	1	R.EIGNLLHPSVPISNDEDVDNKVER.I	(Ions score 77)
134 - 157	673.0944	2688.3485	2688.3355	5	1	R.EIGNLLHPSVPISNDEDVDNKVER.I	(Ions score 45)
134 - 157	673.0944	2688.3486	2688.3355	5	1	R.EIGNLLHPSVPISNDEDVDNKVER.I	(Ions score 38)

134 - 157	673.0944	2688.3486	2688.3355	5	1	R.EIGNLLHPSVPISNDEDVDNKVER.I	(Ions score 25)
134 - 157	897.1235	2688.3487	2688.3355	5	1	R.EIGNLLHPSVPISNDEDVDNKVER.I	(Ions score 35)
134 - 157	673.0945	2688.3488	2688.3355	5	1	R.EIGNLLHPSVPISNDEDVDNKVER.I	(Ions score 48)
134 - 157	673.0945	2688.3488	2688.3355	5	1	R.EIGNLLHPSVPISNDEDVDNKVER.I	(Ions score 36)
134 - 157	911.1255	2730.3548	2730.3460	3	1	R.EIGNLLHPSVPISNDEDVDNKVER.I	Acetylation (K) (Ions score 67)
134 - 157	911.1261	2730.3566	2730.3460	4	1	R.EIGNLLHPSVPISNDEDVDNKVER.I	Acetylation (K) (Ions score 69)
158 - 165	503.7420	1005.4694	1005.4702	-1	0	R.IWGDCTVR.K	(Ions score 35)
158 - 165	503.7423	1005.4699	1005.4702	-0	0	R.IWGDCTVR.K	(Ions score 35)
158 - 165	503.7426	1005.4706	1005.4702	0	0	R.IWGDCTVR.K	(Ions score 39)
158 - 165	503.7427	1005.4709	1005.4702	1	0	R.IWGDCTVR.K	(Ions score 38)
158 - 165	503.7428	1005.4711	1005.4702	1	0	R.IWGDCTVR.K	(Ions score 35)
158 - 165	503.7429	1005.4713	1005.4702	1	0	R.IWGDCTVR.K	(Ions score 35)
158 - 165	503.7431	1005.4716	1005.4702	1	0	R.IWGDCTVR.K	(Ions score 35)
158 - 165	503.7433	1005.4720	1005.4702	2	0	R.IWGDCTVR.K	(Ions score 36)
158 - 165	503.7433	1005.4720	1005.4702	2	0	R.IWGDCTVR.K	(Ions score 39)
158 - 165	503.7434	1005.4722	1005.4702	2	0	R.IWGDCTVR.K	(Ions score 36)
158 - 165	503.7434	1005.4722	1005.4702	2	0	R.IWGDCTVR.K	(Ions score 33)
158 - 165	503.7434	1005.4722	1005.4702	2	0	R.IWGDCTVR.K	(Ions score 36)
158 - 165	503.7434	1005.4722	1005.4702	2	0	R.IWGDCTVR.K	(Ions score 35)
158 - 165	503.7436	1005.4726	1005.4702	2	0	R.IWGDCTVR.K	(Ions score 40)
158 - 165	503.7436	1005.4726	1005.4702	2	0	R.IWGDCTVR.K	(Ions score 28)
158 - 165	503.7436	1005.4727	1005.4702	2	0	R.IWGDCTVR.K	(Ions score 35)
158 - 165	503.7437	1005.4728	1005.4702	3	0	R.IWGDCTVR.K	(Ions score 39)
158 - 165	503.7437	1005.4729	1005.4702	3	0	R.IWGDCTVR.K	(Ions score 33)
158 - 165	503.7438	1005.4730	1005.4702	3	0	R.IWGDCTVR.K	(Ions score 39)
158 - 165	503.7438	1005.4731	1005.4702	3	0	R.IWGDCTVR.K	(Ions score 26)
158 - 165	503.7439	1005.4732	1005.4702	3	0	R.IWGDCTVR.K	(Ions score 30)
158 - 165	503.7442	1005.4738	1005.4702	4	0	R.IWGDCTVR.K	(Ions score 34)
158 - 165	503.7442	1005.4739	1005.4702	4	0	R.IWGDCTVR.K	(Ions score 30)
158 - 165	503.7443	1005.4740	1005.4702	4	0	R.IWGDCTVR.K	(Ions score 39)
158 - 165	503.7445	1005.4745	1005.4702	4	0	R.IWGDCTVR.K	(Ions score 40)
158 - 165	503.7447	1005.4748	1005.4702	5	0	R.IWGDCTVR.K	(Ions score 39)
158 - 165	503.7447	1005.4748	1005.4702	5	0	R.IWGDCTVR.K	(Ions score 22)
158 - 165	503.7449	1005.4752	1005.4702	5	0	R.IWGDCTVR.K	(Ions score 35)
167 - 184	684.6748	2051.0026	2051.0034	-0	1	K.KYSHVDLVVMVDGFEGEK.G	(Ions score 50)
167 - 184	684.6750	2051.0033	2051.0034	-0	1	K.KYSHVDLVVMVDGFEGEK.G	(Ions score 38)
167 - 184	684.6753	2051.0039	2051.0034	0	1	K.KYSHVDLVVMVDGFEGEK.G	(Ions score 37)
167 - 184	684.6753	2051.0041	2051.0034	0	1	K.KYSHVDLVVMVDGFEGEK.G	(Ions score 50)
167 - 184	684.6753	2051.0041	2051.0034	0	1	K.KYSHVDLVVMVDGFEGEK.G	(Ions score 39)
167 - 184	684.6753	2051.0041	2051.0034	0	1	K.KYSHVDLVVMVDGFEGEK.G	(Ions score 64)
167 - 184	684.6754	2051.0044	2051.0034	0	1	K.KYSHVDLVVMVDGFEGEK.G	(Ions score 45)
167 - 184	684.6754	2051.0044	2051.0034	1	1	K.KYSHVDLVVMVDGFEGEK.G	(Ions score 21)
167 - 184	684.6754	2051.0045	2051.0034	1	1	K.KYSHVDLVVMVDGFEGEK.G	(Ions score 45)
167 - 184	684.6756	2051.0049	2051.0034	1	1	K.KYSHVDLVVMVDGFEGEK.G	(Ions score 31)
167 - 184	684.6757	2051.0051	2051.0034	1	1	K.KYSHVDLVVMVDGFEGEK.G	(Ions score 54)
167 - 184	684.6757	2051.0053	2051.0034	1	1	K.KYSHVDLVVMVDGFEGEK.G	(Ions score 44)
167 - 184	684.6758	2051.0055	2051.0034	1	1	K.KYSHVDLVVMVDGFEGEK.G	(Ions score 22)
167 - 184	684.6759	2051.0058	2051.0034	1	1	K.KYSHVDLVVMVDGFEGEK.G	(Ions score 39)
167 - 184	684.6759	2051.0060	2051.0034	1	1	K.KYSHVDLVVMVDGFEGEK.G	(Ions score 35)
167 - 184	684.6760	2051.0062	2051.0034	1	1	K.KYSHVDLVVMVDGFEGEK.G	(Ions score 49)
167 - 184	513.7588	2051.0062	2051.0034	1	1	K.KYSHVDLVVMVDGFEGEK.G	(Ions score 40)
167 - 184	684.6760	2051.0062	2051.0034	1	1	K.KYSHVDLVVMVDGFEGEK.G	(Ions score 53)
167 - 184	684.6760	2051.0062	2051.0034	1	1	K.KYSHVDLVVMVDGFEGEK.G	(Ions score 34)
167 - 184	684.6760	2051.0063	2051.0034	1	1	K.KYSHVDLVVMVDGFEGEK.G	(Ions score 29)
167 - 184	684.6761	2051.0063	2051.0034	1	1	K.KYSHVDLVVMVDGFEGEK.G	(Ions score 58)
167 - 184	684.6761	2051.0066	2051.0034	2	1	K.KYSHVDLVVMVDGFEGEK.G	(Ions score 52)
167 - 184	684.6762	2051.0067	2051.0034	2	1	K.KYSHVDLVVMVDGFEGEK.G	(Ions score 39)
167 - 184	684.6762	2051.0068	2051.0034	2	1	K.KYSHVDLVVMVDGFEGEK.G	(Ions score 38)

167 - 184	684.6762	2051.0069	2051.0034	2	1	K.KYSHVDLVVMVDGFEGEK.G	(Ions score 31)
167 - 184	1026.5107	2051.0069	2051.0034	2	1	K.KYSHVDLVVMVDGFEGEK.G	(Ions score 80)
167 - 184	684.6763	2051.0071	2051.0034	2	1	K.KYSHVDLVVMVDGFEGEK.G	(Ions score 38)
167 - 184	684.6763	2051.0072	2051.0034	2	1	K.KYSHVDLVVMVDGFEGEK.G	(Ions score 48)
167 - 184	684.6764	2051.0072	2051.0034	2	1	K.KYSHVDLVVMVDGFEGEK.G	(Ions score 36)
167 - 184	684.6764	2051.0073	2051.0034	2	1	K.KYSHVDLVVMVDGFEGEK.G	(Ions score 39)
167 - 184	513.7591	2051.0074	2051.0034	2	1	K.KYSHVDLVVMVDGFEGEK.G	(Ions score 26)
167 - 184	684.6764	2051.0074	2051.0034	2	1	K.KYSHVDLVVMVDGFEGEK.G	(Ions score 54)
167 - 184	684.6764	2051.0074	2051.0034	2	1	K.KYSHVDLVVMVDGFEGEK.G	(Ions score 53)
167 - 184	513.7592	2051.0076	2051.0034	2	1	K.KYSHVDLVVMVDGFEGEK.G	(Ions score 32)
167 - 184	684.6765	2051.0076	2051.0034	2	1	K.KYSHVDLVVMVDGFEGEK.G	(Ions score 40)
167 - 184	513.7592	2051.0077	2051.0034	2	1	K.KYSHVDLVVMVDGFEGEK.G	(Ions score 32)
167 - 184	684.6765	2051.0077	2051.0034	2	1	K.KYSHVDLVVMVDGFEGEK.G	(Ions score 47)
167 - 184	684.6765	2051.0078	2051.0034	2	1	K.KYSHVDLVVMVDGFEGEK.G	(Ions score 43)
167 - 184	684.6765	2051.0078	2051.0034	2	1	K.KYSHVDLVVMVDGFEGEK.G	(Ions score 31)
167 - 184	684.6766	2051.0078	2051.0034	2	1	K.KYSHVDLVVMVDGFEGEK.G	(Ions score 58)
167 - 184	684.6766	2051.0079	2051.0034	2	1	K.KYSHVDLVVMVDGFEGEK.G	(Ions score 44)
167 - 184	684.6766	2051.0079	2051.0034	2	1	K.KYSHVDLVVMVDGFEGEK.G	(Ions score 35)
167 - 184	684.6766	2051.0079	2051.0034	2	1	K.KYSHVDLVVMVDGFEGEK.G	(Ions score 47)
167 - 184	684.6766	2051.0079	2051.0034	2	1	K.KYSHVDLVVMVDGFEGEK.G	(Ions score 56)
167 - 184	684.6766	2051.0080	2051.0034	2	1	K.KYSHVDLVVMVDGFEGEK.G	(Ions score 34)
167 - 184	684.6766	2051.0080	2051.0034	2	1	K.KYSHVDLVVMVDGFEGEK.G	(Ions score 39)
167 - 184	1026.5113	2051.0080	2051.0034	2	1	K.KYSHVDLVVMVDGFEGEK.G	(Ions score 72)
167 - 184	684.6767	2051.0082	2051.0034	2	1	K.KYSHVDLVVMVDGFEGEK.G	(Ions score 46)
167 - 184	684.6767	2051.0082	2051.0034	2	1	K.KYSHVDLVVMVDGFEGEK.G	(Ions score 43)
167 - 184	684.6767	2051.0082	2051.0034	2	1	K.KYSHVDLVVMVDGFEGEK.G	(Ions score 54)
167 - 184	684.6767	2051.0083	2051.0034	2	1	K.KYSHVDLVVMVDGFEGEK.G	(Ions score 45)
167 - 184	684.6767	2051.0083	2051.0034	2	1	K.KYSHVDLVVMVDGFEGEK.G	(Ions score 58)
167 - 184	1026.5114	2051.0083	2051.0034	2	1	K.KYSHVDLVVMVDGFEGEK.G	(Ions score 89)
167 - 184	684.6767	2051.0083	2051.0034	2	1	K.KYSHVDLVVMVDGFEGEK.G	(Ions score 43)
167 - 184	684.6767	2051.0084	2051.0034	2	1	K.KYSHVDLVVMVDGFEGEK.G	(Ions score 44)
167 - 184	684.6768	2051.0085	2051.0034	3	1	K.KYSHVDLVVMVDGFEGEK.G	(Ions score 42)
167 - 184	1026.5116	2051.0086	2051.0034	3	1	K.KYSHVDLVVMVDGFEGEK.G	(Ions score 80)
167 - 184	684.6768	2051.0086	2051.0034	3	1	K.KYSHVDLVVMVDGFEGEK.G	(Ions score 49)
167 - 184	684.6768	2051.0086	2051.0034	3	1	K.KYSHVDLVVMVDGFEGEK.G	(Ions score 47)
167 - 184	684.6768	2051.0087	2051.0034	3	1	K.KYSHVDLVVMVDGFEGEK.G	(Ions score 49)
167 - 184	684.6769	2051.0089	2051.0034	3	1	K.KYSHVDLVVMVDGFEGEK.G	(Ions score 39)
167 - 184	684.6769	2051.0089	2051.0034	3	1	K.KYSHVDLVVMVDGFEGEK.G	(Ions score 41)
167 - 184	684.6769	2051.0089	2051.0034	3	1	K.KYSHVDLVVMVDGFEGEK.G	(Ions score 53)
167 - 184	684.6769	2051.0090	2051.0034	3	1	K.KYSHVDLVVMVDGFEGEK.G	(Ions score 46)
167 - 184	684.6769	2051.0090	2051.0034	3	1	K.KYSHVDLVVMVDGFEGEK.G	(Ions score 59)
167 - 184	684.6770	2051.0090	2051.0034	3	1	K.KYSHVDLVVMVDGFEGEK.G	(Ions score 46)
167 - 184	684.6770	2051.0091	2051.0034	3	1	K.KYSHVDLVVMVDGFEGEK.G	(Ions score 52)
167 - 184	684.6770	2051.0091	2051.0034	3	1	K.KYSHVDLVVMVDGFEGEK.G	(Ions score 59)
167 - 184	684.6770	2051.0092	2051.0034	3	1	K.KYSHVDLVVMVDGFEGEK.G	(Ions score 52)
167 - 184	684.6770	2051.0092	2051.0034	3	1	K.KYSHVDLVVMVDGFEGEK.G	(Ions score 47)
167 - 184	684.6770	2051.0092	2051.0034	3	1	K.KYSHVDLVVMVDGFEGEK.G	(Ions score 56)
167 - 184	684.6770	2051.0092	2051.0034	3	1	K.KYSHVDLVVMVDGFEGEK.G	(Ions score 28)
167 - 184	684.6770	2051.0093	2051.0034	3	1	K.KYSHVDLVVMVDGFEGEK.G	(Ions score 53)
167 - 184	684.6770	2051.0093	2051.0034	3	1	K.KYSHVDLVVMVDGFEGEK.G	(Ions score 42)
167 - 184	684.6771	2051.0094	2051.0034	3	1	K.KYSHVDLVVMVDGFEGEK.G	(Ions score 36)
167 - 184	684.6771	2051.0094	2051.0034	3	1	K.KYSHVDLVVMVDGFEGEK.G	(Ions score 50)
167 - 184	684.6771	2051.0094	2051.0034	3	1	K.KYSHVDLVVMVDGFEGEK.G	(Ions score 43)
167 - 184	684.6771	2051.0095	2051.0034	3	1	K.KYSHVDLVVMVDGFEGEK.G	(Ions score 45)
167 - 184	684.6771	2051.0095	2051.0034	3	1	K.KYSHVDLVVMVDGFEGEK.G	(Ions score 47)
167 - 184	684.6771	2051.0095	2051.0034	3	1	K.KYSHVDLVVMVDGFEGEK.G	(Ions score 47)
167 - 184	684.6771	2051.0096	2051.0034	3	1	K.KYSHVDLVVMVDGFEGEK.G	(Ions score 29)
167 - 184	684.6771	2051.0096	2051.0034	3	1	K.KYSHVDLVVMVDGFEGEK.G	(Ions score 26)

167 - 184	1026.5121	2051.0096	2051.0034	3	1	K.KYSHVDLVVMVDGFEGEK.G		(Ions score 95)
167 - 184	684.6772	2051.0096	2051.0034	3	1	K.KYSHVDLVVMVDGFEGEK.G		(Ions score 42)
167 - 184	684.6772	2051.0096	2051.0034	3	1	K.KYSHVDLVVMVDGFEGEK.G		(Ions score 43)
167 - 184	684.6772	2051.0097	2051.0034	3	1	K.KYSHVDLVVMVDGFEGEK.G		(Ions score 41)
167 - 184	684.6772	2051.0097	2051.0034	3	1	K.KYSHVDLVVMVDGFEGEK.G		(Ions score 53)
167 - 184	684.6772	2051.0098	2051.0034	3	1	K.KYSHVDLVVMVDGFEGEK.G		(Ions score 50)
167 - 184	684.6772	2051.0099	2051.0034	3	1	K.KYSHVDLVVMVDGFEGEK.G		(Ions score 52)
167 - 184	684.6772	2051.0099	2051.0034	3	1	K.KYSHVDLVVMVDGFEGEK.G		(Ions score 35)
167 - 184	684.6773	2051.0099	2051.0034	3	1	K.KYSHVDLVVMVDGFEGEK.G		(Ions score 58)
167 - 184	684.6773	2051.0100	2051.0034	3	1	K.KYSHVDLVVMVDGFEGEK.G		(Ions score 41)
167 - 184	684.6773	2051.0100	2051.0034	3	1	K.KYSHVDLVVMVDGFEGEK.G		(Ions score 53)
167 - 184	684.6773	2051.0101	2051.0034	3	1	K.KYSHVDLVVMVDGFEGEK.G		(Ions score 49)
167 - 184	684.6773	2051.0101	2051.0034	3	1	K.KYSHVDLVVMVDGFEGEK.G		(Ions score 60)
167 - 184	684.6774	2051.0104	2051.0034	3	1	K.KYSHVDLVVMVDGFEGEK.G		(Ions score 40)
167 - 184	684.6774	2051.0105	2051.0034	3	1	K.KYSHVDLVVMVDGFEGEK.G		(Ions score 41)
167 - 184	684.6774	2051.0105	2051.0034	3	1	K.KYSHVDLVVMVDGFEGEK.G		(Ions score 52)
167 - 184	684.6775	2051.0105	2051.0034	3	1	K.KYSHVDLVVMVDGFEGEK.G		(Ions score 45)
167 - 184	684.6775	2051.0106	2051.0034	4	1	K.KYSHVDLVVMVDGFEGEK.G		(Ions score 25)
167 - 184	684.6775	2051.0106	2051.0034	4	1	K.KYSHVDLVVMVDGFEGEK.G		(Ions score 47)
167 - 184	684.6775	2051.0106	2051.0034	4	1	K.KYSHVDLVVMVDGFEGEK.G		(Ions score 49)
167 - 184	684.6776	2051.0111	2051.0034	4	1	K.KYSHVDLVVMVDGFEGEK.G		(Ions score 38)
167 - 184	684.6776	2051.0111	2051.0034	4	1	K.KYSHVDLVVMVDGFEGEK.G		(Ions score 38)
167 - 184	684.6777	2051.0111	2051.0034	4	1	K.KYSHVDLVVMVDGFEGEK.G		(Ions score 29)
167 - 184	684.6777	2051.0111	2051.0034	4	1	K.KYSHVDLVVMVDGFEGEK.G		(Ions score 34)
167 - 184	1026.5128	2051.0111	2051.0034	4	1	K.KYSHVDLVVMVDGFEGEK.G		(Ions score 75)
167 - 184	684.6777	2051.0112	2051.0034	4	1	K.KYSHVDLVVMVDGFEGEK.G		(Ions score 36)
167 - 184	513.7601	2051.0112	2051.0034	4	1	K.KYSHVDLVVMVDGFEGEK.G		(Ions score 23)
167 - 184	513.7601	2051.0113	2051.0034	4	1	K.KYSHVDLVVMVDGFEGEK.G		(Ions score 31)
167 - 184	684.6777	2051.0114	2051.0034	4	1	K.KYSHVDLVVMVDGFEGEK.G		(Ions score 39)
167 - 184	684.6778	2051.0115	2051.0034	4	1	K.KYSHVDLVVMVDGFEGEK.G		(Ions score 46)
167 - 184	684.6778	2051.0115	2051.0034	4	1	K.KYSHVDLVVMVDGFEGEK.G		(Ions score 47)
167 - 184	684.6778	2051.0116	2051.0034	4	1	K.KYSHVDLVVMVDGFEGEK.G		(Ions score 27)
167 - 184	684.6778	2051.0117	2051.0034	4	1	K.KYSHVDLVVMVDGFEGEK.G		(Ions score 46)
167 - 184	684.6779	2051.0117	2051.0034	4	1	K.KYSHVDLVVMVDGFEGEK.G		(Ions score 37)
167 - 184	684.6779	2051.0120	2051.0034	4	1	K.KYSHVDLVVMVDGFEGEK.G		(Ions score 36)
167 - 184	684.6780	2051.0121	2051.0034	4	1	K.KYSHVDLVVMVDGFEGEK.G		(Ions score 33)
167 - 184	684.6780	2051.0121	2051.0034	4	1	K.KYSHVDLVVMVDGFEGEK.G		(Ions score 27)
167 - 184	1026.5134	2051.0123	2051.0034	4	1	K.KYSHVDLVVMVDGFEGEK.G		(Ions score 66)
167 - 184	684.6781	2051.0124	2051.0034	4	1	K.KYSHVDLVVMVDGFEGEK.G		(Ions score 53)
167 - 184	684.6781	2051.0124	2051.0034	4	1	K.KYSHVDLVVMVDGFEGEK.G		(Ions score 44)
167 - 184	684.6782	2051.0127	2051.0034	5	1	K.KYSHVDLVVMVDGFEGEK.G		(Ions score 26)
167 - 184	684.6782	2051.0128	2051.0034	5	1	K.KYSHVDLVVMVDGFEGEK.G		(Ions score 36)
167 - 184	684.6782	2051.0129	2051.0034	5	1	K.KYSHVDLVVMVDGFEGEK.G		(Ions score 42)
167 - 184	684.6783	2051.0130	2051.0034	5	1	K.KYSHVDLVVMVDGFEGEK.G		(Ions score 43)
167 - 184	684.6783	2051.0132	2051.0034	5	1	K.KYSHVDLVVMVDGFEGEK.G		(Ions score 58)
167 - 184	684.6784	2051.0133	2051.0034	5	1	K.KYSHVDLVVMVDGFEGEK.G		(Ions score 44)
167 - 184	684.6784	2051.0133	2051.0034	5	1	K.KYSHVDLVVMVDGFEGEK.G		(Ions score 47)
167 - 184	684.6785	2051.0136	2051.0034	5	1	K.KYSHVDLVVMVDGFEGEK.G		(Ions score 40)
167 - 184	517.7558	2066.9940	2066.9983	-2	1	K.KYSHVDLVVMVDGFEGEK.G	Oxidation (M)	(Ions score 47)
167 - 184	690.0054	2066.9944	2066.9983	-2	1	K.KYSHVDLVVMVDGFEGEK.G	Oxidation (M)	(Ions score 28)
167 - 184	517.7559	2066.9946	2066.9983	-2	1	K.KYSHVDLVVMVDGFEGEK.G	Oxidation (M)	(Ions score 32)
167 - 184	517.7560	2066.9947	2066.9983	-2	1	K.KYSHVDLVVMVDGFEGEK.G	Oxidation (M)	(Ions score 36)
167 - 184	517.7560	2066.9949	2066.9983	-2	1	K.KYSHVDLVVMVDGFEGEK.G	Oxidation (M)	(Ions score 34)
167 - 184	517.7562	2066.9956	2066.9983	-1	1	K.KYSHVDLVVMVDGFEGEK.G	Oxidation (M)	(Ions score 40)
167 - 184	517.7562	2066.9958	2066.9983	-1	1	K.KYSHVDLVVMVDGFEGEK.G	Oxidation (M)	(Ions score 32)
167 - 184	517.7562	2066.9958	2066.9983	-1	1	K.KYSHVDLVVMVDGFEGEK.G	Oxidation (M)	(Ions score 30)
167 - 184	690.0060	2066.9961	2066.9983	-1	1	K.KYSHVDLVVMVDGFEGEK.G	Oxidation (M)	(Ions score 54)
167 - 184	517.7563	2066.9961	2066.9983	-1	1	K.KYSHVDLVVMVDGFEGEK.G	Oxidation (M)	(Ions score 33)

167 - 184	517.7563	2066.9963	2066.9983	-1	1	K.KYSHVDLVVMVDGFEGEK.G	Oxidation (M)	(Ions score 35)
167 - 184	690.0060	2066.9963	2066.9983	-1	1	K.KYSHVDLVVMVDGFEGEK.G	Oxidation (M)	(Ions score 45)
167 - 184	517.7564	2066.9965	2066.9983	-1	1	K.KYSHVDLVVMVDGFEGEK.G	Oxidation (M)	(Ions score 33)
167 - 184	690.0061	2066.9965	2066.9983	-1	1	K.KYSHVDLVVMVDGFEGEK.G	Oxidation (M)	(Ions score 40)
167 - 184	517.7564	2066.9966	2066.9983	-1	1	K.KYSHVDLVVMVDGFEGEK.G	Oxidation (M)	(Ions score 38)
167 - 184	690.0062	2066.9968	2066.9983	-1	1	K.KYSHVDLVVMVDGFEGEK.G	Oxidation (M)	(Ions score 35)
167 - 184	690.0062	2066.9969	2066.9983	-1	1	K.KYSHVDLVVMVDGFEGEK.G	Oxidation (M)	(Ions score 29)
167 - 184	690.0062	2066.9969	2066.9983	-1	1	K.KYSHVDLVVMVDGFEGEK.G	Oxidation (M)	(Ions score 25)
167 - 184	690.0065	2066.9976	2066.9983	-0	1	K.KYSHVDLVVMVDGFEGEK.G	Oxidation (M)	(Ions score 34)
167 - 184	517.7567	2066.9978	2066.9983	-0	1	K.KYSHVDLVVMVDGFEGEK.G	Oxidation (M)	(Ions score 45)
167 - 184	517.7567	2066.9978	2066.9983	-0	1	K.KYSHVDLVVMVDGFEGEK.G	Oxidation (M)	(Ions score 30)
167 - 184	690.0067	2066.9982	2066.9983	-0	1	K.KYSHVDLVVMVDGFEGEK.G	Oxidation (M)	(Ions score 25)
167 - 184	690.0067	2066.9982	2066.9983	-0	1	K.KYSHVDLVVMVDGFEGEK.G	Oxidation (M)	(Ions score 47)
167 - 184	517.7568	2066.9982	2066.9983	-0	1	K.KYSHVDLVVMVDGFEGEK.G	Oxidation (M)	(Ions score 38)
167 - 184	690.0067	2066.9984	2066.9983	0	1	K.KYSHVDLVVMVDGFEGEK.G	Oxidation (M)	(Ions score 37)
167 - 184	517.7569	2066.9985	2066.9983	0	1	K.KYSHVDLVVMVDGFEGEK.G	Oxidation (M)	(Ions score 33)
167 - 184	517.7569	2066.9985	2066.9983	0	1	K.KYSHVDLVVMVDGFEGEK.G	Oxidation (M)	(Ions score 39)
167 - 184	690.0068	2066.9985	2066.9983	0	1	K.KYSHVDLVVMVDGFEGEK.G	Oxidation (M)	(Ions score 22)
167 - 184	690.0068	2066.9985	2066.9983	0	1	K.KYSHVDLVVMVDGFEGEK.G	Oxidation (M)	(Ions score 45)
167 - 184	690.0068	2066.9985	2066.9983	0	1	K.KYSHVDLVVMVDGFEGEK.G	Oxidation (M)	(Ions score 36)
167 - 184	690.0068	2066.9986	2066.9983	0	1	K.KYSHVDLVVMVDGFEGEK.G	Oxidation (M)	(Ions score 46)
167 - 184	517.7570	2066.9987	2066.9983	0	1	K.KYSHVDLVVMVDGFEGEK.G	Oxidation (M)	(Ions score 41)
167 - 184	517.7570	2066.9988	2066.9983	0	1	K.KYSHVDLVVMVDGFEGEK.G	Oxidation (M)	(Ions score 30)
167 - 184	517.7571	2066.9992	2066.9983	0	1	K.KYSHVDLVVMVDGFEGEK.G	Oxidation (M)	(Ions score 39)
167 - 184	517.7571	2066.9992	2066.9983	0	1	K.KYSHVDLVVMVDGFEGEK.G	Oxidation (M)	(Ions score 39)
167 - 184	690.0070	2066.9992	2066.9983	0	1	K.KYSHVDLVVMVDGFEGEK.G	Oxidation (M)	(Ions score 38)
167 - 184	1034.5069	2066.9993	2066.9983	0	1	K.KYSHVDLVVMVDGFEGEK.G	Oxidation (M)	(Ions score 64)
167 - 184	517.7571	2066.9993	2066.9983	0	1	K.KYSHVDLVVMVDGFEGEK.G	Oxidation (M)	(Ions score 28)
167 - 184	690.0071	2066.9994	2066.9983	1	1	K.KYSHVDLVVMVDGFEGEK.G	Oxidation (M)	(Ions score 41)
167 - 184	517.7572	2066.9996	2066.9983	1	1	K.KYSHVDLVVMVDGFEGEK.G	Oxidation (M)	(Ions score 31)
167 - 184	690.0071	2066.9996	2066.9983	1	1	K.KYSHVDLVVMVDGFEGEK.G	Oxidation (M)	(Ions score 36)
167 - 184	690.0071	2066.9996	2066.9983	1	1	K.KYSHVDLVVMVDGFEGEK.G	Oxidation (M)	(Ions score 45)
167 - 184	517.7572	2066.9997	2066.9983	1	1	K.KYSHVDLVVMVDGFEGEK.G	Oxidation (M)	(Ions score 32)
167 - 184	690.0072	2066.9997	2066.9983	1	1	K.KYSHVDLVVMVDGFEGEK.G	Oxidation (M)	(Ions score 51)
167 - 184	1034.5072	2066.9998	2066.9983	1	1	K.KYSHVDLVVMVDGFEGEK.G	Oxidation (M)	(Ions score 49)
167 - 184	690.0072	2066.9998	2066.9983	1	1	K.KYSHVDLVVMVDGFEGEK.G	Oxidation (M)	(Ions score 29)
167 - 184	690.0072	2066.9999	2066.9983	1	1	K.KYSHVDLVVMVDGFEGEK.G	Oxidation (M)	(Ions score 41)
167 - 184	690.0072	2066.9999	2066.9983	1	1	K.KYSHVDLVVMVDGFEGEK.G	Oxidation (M)	(Ions score 44)
167 - 184	517.7573	2066.9999	2066.9983	1	1	K.KYSHVDLVVMVDGFEGEK.G	Oxidation (M)	(Ions score 33)
167 - 184	690.0073	2067.0000	2066.9983	1	1	K.KYSHVDLVVMVDGFEGEK.G	Oxidation (M)	(Ions score 30)
167 - 184	690.0073	2067.0000	2066.9983	1	1	K.KYSHVDLVVMVDGFEGEK.G	Oxidation (M)	(Ions score 35)
167 - 184	517.7573	2067.0000	2066.9983	1	1	K.KYSHVDLVVMVDGFEGEK.G	Oxidation (M)	(Ions score 34)
167 - 184	517.7573	2067.0001	2066.9983	1	1	K.KYSHVDLVVMVDGFEGEK.G	Oxidation (M)	(Ions score 36)
167 - 184	517.7573	2067.0001	2066.9983	1	1	K.KYSHVDLVVMVDGFEGEK.G	Oxidation (M)	(Ions score 38)
167 - 184	690.0073	2067.0001	2066.9983	1	1	K.KYSHVDLVVMVDGFEGEK.G	Oxidation (M)	(Ions score 43)
167 - 184	690.0074	2067.0002	2066.9983	1	1	K.KYSHVDLVVMVDGFEGEK.G	Oxidation (M)	(Ions score 55)
167 - 184	690.0074	2067.0003	2066.9983	1	1	K.KYSHVDLVVMVDGFEGEK.G	Oxidation (M)	(Ions score 39)
167 - 184	690.0074	2067.0003	2066.9983	1	1	K.KYSHVDLVVMVDGFEGEK.G	Oxidation (M)	(Ions score 54)
167 - 184	690.0074	2067.0003	2066.9983	1	1	K.KYSHVDLVVMVDGFEGEK.G	Oxidation (M)	(Ions score 41)
167 - 184	517.7574	2067.0003	2066.9983	1	1	K.KYSHVDLVVMVDGFEGEK.G	Oxidation (M)	(Ions score 32)
167 - 184	690.0074	2067.0004	2066.9983	1	1	K.KYSHVDLVVMVDGFEGEK.G	Oxidation (M)	(Ions score 41)
167 - 184	690.0074	2067.0004	2066.9983	1	1	K.KYSHVDLVVMVDGFEGEK.G	Oxidation (M)	(Ions score 32)
167 - 184	690.0074	2067.0004	2066.9983	1	1	K.KYSHVDLVVMVDGFEGEK.G	Oxidation (M)	(Ions score 28)
167 - 184	517.7574	2067.0004	2066.9983	1	1	K.KYSHVDLVVMVDGFEGEK.G	Oxidation (M)	(Ions score 32)
167 - 184	517.7574	2067.0004	2066.9983	1	1	K.KYSHVDLVVMVDGFEGEK.G	Oxidation (M)	(Ions score 36)
167 - 184	690.0074	2067.0004	2066.9983	1	1	K.KYSHVDLVVMVDGFEGEK.G	Oxidation (M)	(Ions score 46)
167 - 184	690.0074	2067.0004	2066.9983	1	1	K.KYSHVDLVVMVDGFEGEK.G	Oxidation (M)	(Ions score 29)
167 - 184	690.0074	2067.0005	2066.9983	1	1	K.KYSHVDLVVMVDGFEGEK.G	Oxidation (M)	(Ions score 23)

167 - 184	517.7574	2067.0005	2066.9983	1	1	K.KYSHVDLVVMVDGFEGEK.G	Oxidation (M)	(Ions score 34)
167 - 184	690.0075	2067.0006	2066.9983	1	1	K.KYSHVDLVVMVDGFEGEK.G	Oxidation (M)	(Ions score 38)
167 - 184	517.7574	2067.0006	2066.9983	1	1	K.KYSHVDLVVMVDGFEGEK.G	Oxidation (M)	(Ions score 24)
167 - 184	690.0075	2067.0006	2066.9983	1	1	K.KYSHVDLVVMVDGFEGEK.G	Oxidation (M)	(Ions score 26)
167 - 184	1034.5076	2067.0007	2066.9983	1	1	K.KYSHVDLVVMVDGFEGEK.G	Oxidation (M)	(Ions score 48)
167 - 184	517.7574	2067.0007	2066.9983	1	1	K.KYSHVDLVVMVDGFEGEK.G	Oxidation (M)	(Ions score 27)
167 - 184	690.0075	2067.0007	2066.9983	1	1	K.KYSHVDLVVMVDGFEGEK.G	Oxidation (M)	(Ions score 68)
167 - 184	690.0075	2067.0007	2066.9983	1	1	K.KYSHVDLVVMVDGFEGEK.G	Oxidation (M)	(Ions score 31)
167 - 184	690.0075	2067.0008	2066.9983	1	1	K.KYSHVDLVVMVDGFEGEK.G	Oxidation (M)	(Ions score 43)
167 - 184	690.0076	2067.0008	2066.9983	1	1	K.KYSHVDLVVMVDGFEGEK.G	Oxidation (M)	(Ions score 60)
167 - 184	517.7575	2067.0009	2066.9983	1	1	K.KYSHVDLVVMVDGFEGEK.G	Oxidation (M)	(Ions score 35)
167 - 184	517.7575	2067.0009	2066.9983	1	1	K.KYSHVDLVVMVDGFEGEK.G	Oxidation (M)	(Ions score 29)
167 - 184	690.0076	2067.0010	2066.9983	1	1	K.KYSHVDLVVMVDGFEGEK.G	Oxidation (M)	(Ions score 45)
167 - 184	517.7575	2067.0010	2066.9983	1	1	K.KYSHVDLVVMVDGFEGEK.G	Oxidation (M)	(Ions score 34)
167 - 184	517.7575	2067.0010	2066.9983	1	1	K.KYSHVDLVVMVDGFEGEK.G	Oxidation (M)	(Ions score 34)
167 - 184	517.7575	2067.0011	2066.9983	1	1	K.KYSHVDLVVMVDGFEGEK.G	Oxidation (M)	(Ions score 35)
167 - 184	690.0076	2067.0011	2066.9983	1	1	K.KYSHVDLVVMVDGFEGEK.G	Oxidation (M)	(Ions score 54)
167 - 184	517.7576	2067.0011	2066.9983	1	1	K.KYSHVDLVVMVDGFEGEK.G	Oxidation (M)	(Ions score 36)
167 - 184	517.7576	2067.0011	2066.9983	1	1	K.KYSHVDLVVMVDGFEGEK.G	Oxidation (M)	(Ions score 33)
167 - 184	517.7576	2067.0011	2066.9983	1	1	K.KYSHVDLVVMVDGFEGEK.G	Oxidation (M)	(Ions score 36)
167 - 184	517.7576	2067.0012	2066.9983	1	1	K.KYSHVDLVVMVDGFEGEK.G	Oxidation (M)	(Ions score 35)
167 - 184	517.7576	2067.0013	2066.9983	1	1	K.KYSHVDLVVMVDGFEGEK.G	Oxidation (M)	(Ions score 33)
167 - 184	517.7576	2067.0013	2066.9983	1	1	K.KYSHVDLVVMVDGFEGEK.G	Oxidation (M)	(Ions score 33)
167 - 184	690.0077	2067.0014	2066.9983	1	1	K.KYSHVDLVVMVDGFEGEK.G	Oxidation (M)	(Ions score 26)
167 - 184	690.0077	2067.0014	2066.9983	1	1	K.KYSHVDLVVMVDGFEGEK.G	Oxidation (M)	(Ions score 28)
167 - 184	517.7576	2067.0014	2066.9983	2	1	K.KYSHVDLVVMVDGFEGEK.G	Oxidation (M)	(Ions score 36)
167 - 184	517.7576	2067.0014	2066.9983	2	1	K.KYSHVDLVVMVDGFEGEK.G	Oxidation (M)	(Ions score 31)
167 - 184	690.0078	2067.0015	2066.9983	2	1	K.KYSHVDLVVMVDGFEGEK.G	Oxidation (M)	(Ions score 44)
167 - 184	690.0078	2067.0016	2066.9983	2	1	K.KYSHVDLVVMVDGFEGEK.G	Oxidation (M)	(Ions score 53)
167 - 184	690.0078	2067.0016	2066.9983	2	1	K.KYSHVDLVVMVDGFEGEK.G	Oxidation (M)	(Ions score 30)
167 - 184	690.0078	2067.0016	2066.9983	2	1	K.KYSHVDLVVMVDGFEGEK.G	Oxidation (M)	(Ions score 22)
167 - 184	690.0078	2067.0016	2066.9983	2	1	K.KYSHVDLVVMVDGFEGEK.G	Oxidation (M)	(Ions score 39)
167 - 184	1034.5081	2067.0016	2066.9983	2	1	K.KYSHVDLVVMVDGFEGEK.G	Oxidation (M)	(Ions score 48)
167 - 184	690.0078	2067.0017	2066.9983	2	1	K.KYSHVDLVVMVDGFEGEK.G	Oxidation (M)	(Ions score 53)
167 - 184	517.7577	2067.0017	2066.9983	2	1	K.KYSHVDLVVMVDGFEGEK.G	Oxidation (M)	(Ions score 40)
167 - 184	690.0078	2067.0017	2066.9983	2	1	K.KYSHVDLVVMVDGFEGEK.G	Oxidation (M)	(Ions score 32)
167 - 184	517.7577	2067.0017	2066.9983	2	1	K.KYSHVDLVVMVDGFEGEK.G	Oxidation (M)	(Ions score 37)
167 - 184	517.7577	2067.0017	2066.9983	2	1	K.KYSHVDLVVMVDGFEGEK.G	Oxidation (M)	(Ions score 30)
167 - 184	690.0078	2067.0017	2066.9983	2	1	K.KYSHVDLVVMVDGFEGEK.G	Oxidation (M)	(Ions score 55)
167 - 184	690.0079	2067.0017	2066.9983	2	1	K.KYSHVDLVVMVDGFEGEK.G	Oxidation (M)	(Ions score 64)
167 - 184	690.0079	2067.0017	2066.9983	2	1	K.KYSHVDLVVMVDGFEGEK.G	Oxidation (M)	(Ions score 49)
167 - 184	690.0079	2067.0018	2066.9983	2	1	K.KYSHVDLVVMVDGFEGEK.G	Oxidation (M)	(Ions score 42)
167 - 184	517.7577	2067.0018	2066.9983	2	1	K.KYSHVDLVVMVDGFEGEK.G	Oxidation (M)	(Ions score 34)
167 - 184	690.0079	2067.0018	2066.9983	2	1	K.KYSHVDLVVMVDGFEGEK.G	Oxidation (M)	(Ions score 32)
167 - 184	690.0079	2067.0018	2066.9983	2	1	K.KYSHVDLVVMVDGFEGEK.G	Oxidation (M)	(Ions score 41)
167 - 184	517.7577	2067.0019	2066.9983	2	1	K.KYSHVDLVVMVDGFEGEK.G	Oxidation (M)	(Ions score 34)
167 - 184	517.7578	2067.0019	2066.9983	2	1	K.KYSHVDLVVMVDGFEGEK.G	Oxidation (M)	(Ions score 31)
167 - 184	517.7578	2067.0019	2066.9983	2	1	K.KYSHVDLVVMVDGFEGEK.G	Oxidation (M)	(Ions score 28)
167 - 184	1034.5083	2067.0019	2066.9983	2	1	K.KYSHVDLVVMVDGFEGEK.G	Oxidation (M)	(Ions score 48)
167 - 184	690.0079	2067.0020	2066.9983	2	1	K.KYSHVDLVVMVDGFEGEK.G	Oxidation (M)	(Ions score 54)
167 - 184	690.0080	2067.0020	2066.9983	2	1	K.KYSHVDLVVMVDGFEGEK.G	Oxidation (M)	(Ions score 36)
167 - 184	517.7578	2067.0021	2066.9983	2	1	K.KYSHVDLVVMVDGFEGEK.G	Oxidation (M)	(Ions score 34)
167 - 184	690.0080	2067.0021	2066.9983	2	1	K.KYSHVDLVVMVDGFEGEK.G	Oxidation (M)	(Ions score 54)
167 - 184	690.0080	2067.0021	2066.9983	2	1	K.KYSHVDLVVMVDGFEGEK.G	Oxidation (M)	(Ions score 52)
167 - 184	517.7578	2067.0021	2066.9983	2	1	K.KYSHVDLVVMVDGFEGEK.G	Oxidation (M)	(Ions score 32)
167 - 184	517.7578	2067.0021	2066.9983	2	1	K.KYSHVDLVVMVDGFEGEK.G	Oxidation (M)	(Ions score 35)
167 - 184	517.7578	2067.0021	2066.9983	2	1	K.KYSHVDLVVMVDGFEGEK.G	Oxidation (M)	(Ions score 36)
167 - 184	517.7578	2067.0022	2066.9983	2	1	K.KYSHVDLVVMVDGFEGEK.G	Oxidation (M)	(Ions score 24)

167 - 184	690.0080	2067.0022	2066.9983	2	1	K.KYSHVDLVVMVDGFEGEK.G	Oxidation (M)	(Ions score 53)
167 - 184	517.7578	2067.0022	2066.9983	2	1	K.KYSHVDLVVMVDGFEGEK.G	Oxidation (M)	(Ions score 23)
167 - 184	517.7578	2067.0022	2066.9983	2	1	K.KYSHVDLVVMVDGFEGEK.G	Oxidation (M)	(Ions score 37)
167 - 184	690.0080	2067.0023	2066.9983	2	1	K.KYSHVDLVVMVDGFEGEK.G	Oxidation (M)	(Ions score 60)
167 - 184	517.7578	2067.0023	2066.9983	2	1	K.KYSHVDLVVMVDGFEGEK.G	Oxidation (M)	(Ions score 37)
167 - 184	517.7578	2067.0023	2066.9983	2	1	K.KYSHVDLVVMVDGFEGEK.G	Oxidation (M)	(Ions score 30)
167 - 184	690.0080	2067.0023	2066.9983	2	1	K.KYSHVDLVVMVDGFEGEK.G	Oxidation (M)	(Ions score 36)
167 - 184	690.0080	2067.0023	2066.9983	2	1	K.KYSHVDLVVMVDGFEGEK.G	Oxidation (M)	(Ions score 37)
167 - 184	690.0081	2067.0023	2066.9983	2	1	K.KYSHVDLVVMVDGFEGEK.G	Oxidation (M)	(Ions score 57)
167 - 184	690.0081	2067.0023	2066.9983	2	1	K.KYSHVDLVVMVDGFEGEK.G	Oxidation (M)	(Ions score 32)
167 - 184	517.7579	2067.0024	2066.9983	2	1	K.KYSHVDLVVMVDGFEGEK.G	Oxidation (M)	(Ions score 34)
167 - 184	690.0081	2067.0024	2066.9983	2	1	K.KYSHVDLVVMVDGFEGEK.G	Oxidation (M)	(Ions score 34)
167 - 184	517.7579	2067.0024	2066.9983	2	1	K.KYSHVDLVVMVDGFEGEK.G	Oxidation (M)	(Ions score 37)
167 - 184	517.7579	2067.0024	2066.9983	2	1	K.KYSHVDLVVMVDGFEGEK.G	Oxidation (M)	(Ions score 34)
167 - 184	517.7579	2067.0024	2066.9983	2	1	K.KYSHVDLVVMVDGFEGEK.G	Oxidation (M)	(Ions score 31)
167 - 184	517.7579	2067.0025	2066.9983	2	1	K.KYSHVDLVVMVDGFEGEK.G	Oxidation (M)	(Ions score 40)
167 - 184	690.0081	2067.0025	2066.9983	2	1	K.KYSHVDLVVMVDGFEGEK.G	Oxidation (M)	(Ions score 54)
167 - 184	517.7579	2067.0025	2066.9983	2	1	K.KYSHVDLVVMVDGFEGEK.G	Oxidation (M)	(Ions score 24)
167 - 184	517.7579	2067.0025	2066.9983	2	1	K.KYSHVDLVVMVDGFEGEK.G	Oxidation (M)	(Ions score 37)
167 - 184	1034.5086	2067.0026	2066.9983	2	1	K.KYSHVDLVVMVDGFEGEK.G	Oxidation (M)	(Ions score 21)
167 - 184	517.7579	2067.0026	2066.9983	2	1	K.KYSHVDLVVMVDGFEGEK.G	Oxidation (M)	(Ions score 35)
167 - 184	517.7579	2067.0026	2066.9983	2	1	K.KYSHVDLVVMVDGFEGEK.G	Oxidation (M)	(Ions score 35)
167 - 184	690.0082	2067.0026	2066.9983	2	1	K.KYSHVDLVVMVDGFEGEK.G	Oxidation (M)	(Ions score 48)
167 - 184	517.7579	2067.0027	2066.9983	2	1	K.KYSHVDLVVMVDGFEGEK.G	Oxidation (M)	(Ions score 31)
167 - 184	690.0082	2067.0027	2066.9983	2	1	K.KYSHVDLVVMVDGFEGEK.G	Oxidation (M)	(Ions score 52)
167 - 184	690.0082	2067.0027	2066.9983	2	1	K.KYSHVDLVVMVDGFEGEK.G	Oxidation (M)	(Ions score 31)
167 - 184	517.7580	2067.0028	2066.9983	2	1	K.KYSHVDLVVMVDGFEGEK.G	Oxidation (M)	(Ions score 25)
167 - 184	517.7580	2067.0028	2066.9983	2	1	K.KYSHVDLVVMVDGFEGEK.G	Oxidation (M)	(Ions score 37)
167 - 184	517.7580	2067.0028	2066.9983	2	1	K.KYSHVDLVVMVDGFEGEK.G	Oxidation (M)	(Ions score 43)
167 - 184	517.7580	2067.0028	2066.9983	2	1	K.KYSHVDLVVMVDGFEGEK.G	Oxidation (M)	(Ions score 41)
167 - 184	690.0082	2067.0028	2066.9983	2	1	K.KYSHVDLVVMVDGFEGEK.G	Oxidation (M)	(Ions score 32)
167 - 184	690.0082	2067.0028	2066.9983	2	1	K.KYSHVDLVVMVDGFEGEK.G	Oxidation (M)	(Ions score 25)
167 - 184	690.0082	2067.0029	2066.9983	2	1	K.KYSHVDLVVMVDGFEGEK.G	Oxidation (M)	(Ions score 32)
167 - 184	690.0082	2067.0029	2066.9983	2	1	K.KYSHVDLVVMVDGFEGEK.G	Oxidation (M)	(Ions score 32)
167 - 184	690.0082	2067.0029	2066.9983	2	1	K.KYSHVDLVVMVDGFEGEK.G	Oxidation (M)	(Ions score 34)
167 - 184	690.0082	2067.0029	2066.9983	2	1	K.KYSHVDLVVMVDGFEGEK.G	Oxidation (M)	(Ions score 24)
167 - 184	690.0082	2067.0029	2066.9983	2	1	K.KYSHVDLVVMVDGFEGEK.G	Oxidation (M)	(Ions score 45)
167 - 184	517.7580	2067.0029	2066.9983	2	1	K.KYSHVDLVVMVDGFEGEK.G	Oxidation (M)	(Ions score 34)
167 - 184	690.0082	2067.0029	2066.9983	2	1	K.KYSHVDLVVMVDGFEGEK.G	Oxidation (M)	(Ions score 32)
167 - 184	690.0082	2067.0029	2066.9983	2	1	K.KYSHVDLVVMVDGFEGEK.G	Oxidation (M)	(Ions score 65)
167 - 184	517.7580	2067.0029	2066.9983	2	1	K.KYSHVDLVVMVDGFEGEK.G	Oxidation (M)	(Ions score 41)
167 - 184	690.0083	2067.0029	2066.9983	2	1	K.KYSHVDLVVMVDGFEGEK.G	Oxidation (M)	(Ions score 52)
167 - 184	517.7580	2067.0030	2066.9983	2	1	K.KYSHVDLVVMVDGFEGEK.G	Oxidation (M)	(Ions score 29)
167 - 184	690.0083	2067.0030	2066.9983	2	1	K.KYSHVDLVVMVDGFEGEK.G	Oxidation (M)	(Ions score 67)
167 - 184	517.7580	2067.0030	2066.9983	2	1	K.KYSHVDLVVMVDGFEGEK.G	Oxidation (M)	(Ions score 31)
167 - 184	690.0083	2067.0030	2066.9983	2	1	K.KYSHVDLVVMVDGFEGEK.G	Oxidation (M)	(Ions score 45)
167 - 184	690.0083	2067.0030	2066.9983	2	1	K.KYSHVDLVVMVDGFEGEK.G	Oxidation (M)	(Ions score 45)
167 - 184	690.0083	2067.0031	2066.9983	2	1	K.KYSHVDLVVMVDGFEGEK.G	Oxidation (M)	(Ions score 23)
167 - 184	690.0083	2067.0031	2066.9983	2	1	K.KYSHVDLVVMVDGFEGEK.G	Oxidation (M)	(Ions score 33)
167 - 184	690.0083	2067.0031	2066.9983	2	1	K.KYSHVDLVVMVDGFEGEK.G	Oxidation (M)	(Ions score 44)
167 - 184	690.0083	2067.0031	2066.9983	2	1	K.KYSHVDLVVMVDGFEGEK.G	Oxidation (M)	(Ions score 41)
167 - 184	517.7581	2067.0031	2066.9983	2	1	K.KYSHVDLVVMVDGFEGEK.G	Oxidation (M)	(Ions score 31)
167 - 184	517.7581	2067.0031	2066.9983	2	1	K.KYSHVDLVVMVDGFEGEK.G	Oxidation (M)	(Ions score 33)
167 - 184	690.0083	2067.0031	2066.9983	2	1	K.KYSHVDLVVMVDGFEGEK.G	Oxidation (M)	(Ions score 44)
167 - 184	690.0083	2067.0032	2066.9983	2	1	K.KYSHVDLVVMVDGFEGEK.G	Oxidation (M)	(Ions score 34)
167 - 184	517.7581	2067.0032	2066.9983	2	1	K.KYSHVDLVVMVDGFEGEK.G	Oxidation (M)	(Ions score 49)
167 - 184	517.7581	2067.0032	2066.9983	2	1	K.KYSHVDLVVMVDGFEGEK.G	Oxidation (M)	(Ions score 34)
167 - 184	517.7581	2067.0032	2066.9983	2	1	K.KYSHVDLVVMVDGFEGEK.G	Oxidation (M)	(Ions score 34)

167 - 184	517.7581	2067.0032	2066.9983	2	1	K.KYSHVDLVVMVDGFEGEK.G	Oxidation (M)	(Ions score 33)
167 - 184	517.7581	2067.0032	2066.9983	2	1	K.KYSHVDLVVMVDGFEGEK.G	Oxidation (M)	(Ions score 33)
167 - 184	517.7581	2067.0032	2066.9983	2	1	K.KYSHVDLVVMVDGFEGEK.G	Oxidation (M)	(Ions score 35)
167 - 184	690.0084	2067.0033	2066.9983	2	1	K.KYSHVDLVVMVDGFEGEK.G	Oxidation (M)	(Ions score 31)
167 - 184	517.7581	2067.0033	2066.9983	2	1	K.KYSHVDLVVMVDGFEGEK.G	Oxidation (M)	(Ions score 33)
167 - 184	517.7581	2067.0033	2066.9983	2	1	K.KYSHVDLVVMVDGFEGEK.G	Oxidation (M)	(Ions score 34)
167 - 184	517.7581	2067.0034	2066.9983	2	1	K.KYSHVDLVVMVDGFEGEK.G	Oxidation (M)	(Ions score 32)
167 - 184	517.7581	2067.0034	2066.9983	2	1	K.KYSHVDLVVMVDGFEGEK.G	Oxidation (M)	(Ions score 32)
167 - 184	690.0084	2067.0034	2066.9983	2	1	K.KYSHVDLVVMVDGFEGEK.G	Oxidation (M)	(Ions score 54)
167 - 184	517.7581	2067.0034	2066.9983	2	1	K.KYSHVDLVVMVDGFEGEK.G	Oxidation (M)	(Ions score 27)
167 - 184	517.7581	2067.0034	2066.9983	2	1	K.KYSHVDLVVMVDGFEGEK.G	Oxidation (M)	(Ions score 41)
167 - 184	517.7581	2067.0034	2066.9983	2	1	K.KYSHVDLVVMVDGFEGEK.G	Oxidation (M)	(Ions score 35)
167 - 184	517.7581	2067.0035	2066.9983	2	1	K.KYSHVDLVVMVDGFEGEK.G	Oxidation (M)	(Ions score 32)
167 - 184	517.7581	2067.0035	2066.9983	3	1	K.KYSHVDLVVMVDGFEGEK.G	Oxidation (M)	(Ions score 33)
167 - 184	517.7581	2067.0035	2066.9983	3	1	K.KYSHVDLVVMVDGFEGEK.G	Oxidation (M)	(Ions score 32)
167 - 184	517.7582	2067.0035	2066.9983	3	1	K.KYSHVDLVVMVDGFEGEK.G	Oxidation (M)	(Ions score 33)
167 - 184	690.0084	2067.0035	2066.9983	3	1	K.KYSHVDLVVMVDGFEGEK.G	Oxidation (M)	(Ions score 28)
167 - 184	690.0085	2067.0035	2066.9983	3	1	K.KYSHVDLVVMVDGFEGEK.G	Oxidation (M)	(Ions score 54)
167 - 184	517.7582	2067.0036	2066.9983	3	1	K.KYSHVDLVVMVDGFEGEK.G	Oxidation (M)	(Ions score 36)
167 - 184	1034.5091	2067.0036	2066.9983	3	1	K.KYSHVDLVVMVDGFEGEK.G	Oxidation (M)	(Ions score 29)
167 - 184	517.7582	2067.0036	2066.9983	3	1	K.KYSHVDLVVMVDGFEGEK.G	Oxidation (M)	(Ions score 26)
167 - 184	517.7582	2067.0036	2066.9983	3	1	K.KYSHVDLVVMVDGFEGEK.G	Oxidation (M)	(Ions score 34)
167 - 184	690.0085	2067.0036	2066.9983	3	1	K.KYSHVDLVVMVDGFEGEK.G	Oxidation (M)	(Ions score 47)
167 - 184	517.7582	2067.0036	2066.9983	3	1	K.KYSHVDLVVMVDGFEGEK.G	Oxidation (M)	(Ions score 36)
167 - 184	517.7582	2067.0036	2066.9983	3	1	K.KYSHVDLVVMVDGFEGEK.G	Oxidation (M)	(Ions score 32)
167 - 184	517.7582	2067.0036	2066.9983	3	1	K.KYSHVDLVVMVDGFEGEK.G	Oxidation (M)	(Ions score 31)
167 - 184	517.7582	2067.0036	2066.9983	3	1	K.KYSHVDLVVMVDGFEGEK.G	Oxidation (M)	(Ions score 38)
167 - 184	517.7582	2067.0037	2066.9983	3	1	K.KYSHVDLVVMVDGFEGEK.G	Oxidation (M)	(Ions score 39)
167 - 184	690.0085	2067.0037	2066.9983	3	1	K.KYSHVDLVVMVDGFEGEK.G	Oxidation (M)	(Ions score 44)
167 - 184	517.7582	2067.0037	2066.9983	3	1	K.KYSHVDLVVMVDGFEGEK.G	Oxidation (M)	(Ions score 34)
167 - 184	690.0085	2067.0038	2066.9983	3	1	K.KYSHVDLVVMVDGFEGEK.G	Oxidation (M)	(Ions score 37)
167 - 184	517.7582	2067.0038	2066.9983	3	1	K.KYSHVDLVVMVDGFEGEK.G	Oxidation (M)	(Ions score 34)
167 - 184	690.0085	2067.0038	2066.9983	3	1	K.KYSHVDLVVMVDGFEGEK.G	Oxidation (M)	(Ions score 46)
167 - 184	517.7582	2067.0039	2066.9983	3	1	K.KYSHVDLVVMVDGFEGEK.G	Oxidation (M)	(Ions score 33)
167 - 184	690.0086	2067.0039	2066.9983	3	1	K.KYSHVDLVVMVDGFEGEK.G	Oxidation (M)	(Ions score 26)
167 - 184	517.7582	2067.0039	2066.9983	3	1	K.KYSHVDLVVMVDGFEGEK.G	Oxidation (M)	(Ions score 31)
167 - 184	517.7583	2067.0039	2066.9983	3	1	K.KYSHVDLVVMVDGFEGEK.G	Oxidation (M)	(Ions score 34)
167 - 184	517.7583	2067.0039	2066.9983	3	1	K.KYSHVDLVVMVDGFEGEK.G	Oxidation (M)	(Ions score 30)
167 - 184	690.0086	2067.0039	2066.9983	3	1	K.KYSHVDLVVMVDGFEGEK.G	Oxidation (M)	(Ions score 48)
167 - 184	517.7583	2067.0039	2066.9983	3	1	K.KYSHVDLVVMVDGFEGEK.G	Oxidation (M)	(Ions score 36)
167 - 184	690.0086	2067.0040	2066.9983	3	1	K.KYSHVDLVVMVDGFEGEK.G	Oxidation (M)	(Ions score 43)
167 - 184	690.0086	2067.0040	2066.9983	3	1	K.KYSHVDLVVMVDGFEGEK.G	Oxidation (M)	(Ions score 45)
167 - 184	517.7583	2067.0040	2066.9983	3	1	K.KYSHVDLVVMVDGFEGEK.G	Oxidation (M)	(Ions score 32)
167 - 184	517.7583	2067.0040	2066.9983	3	1	K.KYSHVDLVVMVDGFEGEK.G	Oxidation (M)	(Ions score 35)
167 - 184	517.7583	2067.0040	2066.9983	3	1	K.KYSHVDLVVMVDGFEGEK.G	Oxidation (M)	(Ions score 31)
167 - 184	517.7583	2067.0041	2066.9983	3	1	K.KYSHVDLVVMVDGFEGEK.G	Oxidation (M)	(Ions score 35)
167 - 184	517.7583	2067.0041	2066.9983	3	1	K.KYSHVDLVVMVDGFEGEK.G	Oxidation (M)	(Ions score 30)
167 - 184	517.7583	2067.0041	2066.9983	3	1	K.KYSHVDLVVMVDGFEGEK.G	Oxidation (M)	(Ions score 47)
167 - 184	690.0086	2067.0041	2066.9983	3	1	K.KYSHVDLVVMVDGFEGEK.G	Oxidation (M)	(Ions score 45)
167 - 184	517.7583	2067.0041	2066.9983	3	1	K.KYSHVDLVVMVDGFEGEK.G	Oxidation (M)	(Ions score 25)
167 - 184	690.0087	2067.0042	2066.9983	3	1	K.KYSHVDLVVMVDGFEGEK.G	Oxidation (M)	(Ions score 44)
167 - 184	690.0087	2067.0042	2066.9983	3	1	K.KYSHVDLVVMVDGFEGEK.G	Oxidation (M)	(Ions score 30)
167 - 184	690.0087	2067.0042	2066.9983	3	1	K.KYSHVDLVVMVDGFEGEK.G	Oxidation (M)	(Ions score 66)
167 - 184	517.7583	2067.0042	2066.9983	3	1	K.KYSHVDLVVMVDGFEGEK.G	Oxidation (M)	(Ions score 25)
167 - 184	517.7583	2067.0042	2066.9983	3	1	K.KYSHVDLVVMVDGFEGEK.G	Oxidation (M)	(Ions score 34)
167 - 184	690.0087	2067.0042	2066.9983	3	1	K.KYSHVDLVVMVDGFEGEK.G	Oxidation (M)	(Ions score 37)
167 - 184	690.0087	2067.0042	2066.9983	3	1	K.KYSHVDLVVMVDGFEGEK.G	Oxidation (M)	(Ions score 37)
167 - 184	690.0087	2067.0043	2066.9983	3	1	K.KYSHVDLVVMVDGFEGEK.G	Oxidation (M)	(Ions score 55)

167 - 184	517.7583	2067.0043	2066.9983	3	1	K.KYSHVDLVVMVDGFEGEK.G	Oxidation (M)	(Ions score 34)
167 - 184	690.0087	2067.0043	2066.9983	3	1	K.KYSHVDLVVMVDGFEGEK.G	Oxidation (M)	(Ions score 51)
167 - 184	690.0087	2067.0043	2066.9983	3	1	K.KYSHVDLVVMVDGFEGEK.G	Oxidation (M)	(Ions score 44)
167 - 184	517.7583	2067.0043	2066.9983	3	1	K.KYSHVDLVVMVDGFEGEK.G	Oxidation (M)	(Ions score 25)
167 - 184	517.7584	2067.0043	2066.9983	3	1	K.KYSHVDLVVMVDGFEGEK.G	Oxidation (M)	(Ions score 31)
167 - 184	517.7584	2067.0043	2066.9983	3	1	K.KYSHVDLVVMVDGFEGEK.G	Oxidation (M)	(Ions score 43)
167 - 184	690.0087	2067.0043	2066.9983	3	1	K.KYSHVDLVVMVDGFEGEK.G	Oxidation (M)	(Ions score 41)
167 - 184	517.7584	2067.0043	2066.9983	3	1	K.KYSHVDLVVMVDGFEGEK.G	Oxidation (M)	(Ions score 33)
167 - 184	517.7584	2067.0043	2066.9983	3	1	K.KYSHVDLVVMVDGFEGEK.G	Oxidation (M)	(Ions score 36)
167 - 184	517.7584	2067.0043	2066.9983	3	1	K.KYSHVDLVVMVDGFEGEK.G	Oxidation (M)	(Ions score 52)
167 - 184	690.0087	2067.0044	2066.9983	3	1	K.KYSHVDLVVMVDGFEGEK.G	Oxidation (M)	(Ions score 49)
167 - 184	690.0087	2067.0044	2066.9983	3	1	K.KYSHVDLVVMVDGFEGEK.G	Oxidation (M)	(Ions score 50)
167 - 184	690.0087	2067.0044	2066.9983	3	1	K.KYSHVDLVVMVDGFEGEK.G	Oxidation (M)	(Ions score 56)
167 - 184	517.7584	2067.0044	2066.9983	3	1	K.KYSHVDLVVMVDGFEGEK.G	Oxidation (M)	(Ions score 24)
167 - 184	517.7584	2067.0045	2066.9983	3	1	K.KYSHVDLVVMVDGFEGEK.G	Oxidation (M)	(Ions score 31)
167 - 184	690.0088	2067.0045	2066.9983	3	1	K.KYSHVDLVVMVDGFEGEK.G	Oxidation (M)	(Ions score 33)
167 - 184	690.0088	2067.0045	2066.9983	3	1	K.KYSHVDLVVMVDGFEGEK.G	Oxidation (M)	(Ions score 35)
167 - 184	690.0088	2067.0045	2066.9983	3	1	K.KYSHVDLVVMVDGFEGEK.G	Oxidation (M)	(Ions score 35)
167 - 184	690.0088	2067.0045	2066.9983	3	1	K.KYSHVDLVVMVDGFEGEK.G	Oxidation (M)	(Ions score 31)
167 - 184	517.7584	2067.0045	2066.9983	3	1	K.KYSHVDLVVMVDGFEGEK.G	Oxidation (M)	(Ions score 39)
167 - 184	690.0088	2067.0045	2066.9983	3	1	K.KYSHVDLVVMVDGFEGEK.G	Oxidation (M)	(Ions score 44)
167 - 184	517.7584	2067.0045	2066.9983	3	1	K.KYSHVDLVVMVDGFEGEK.G	Oxidation (M)	(Ions score 34)
167 - 184	690.0088	2067.0045	2066.9983	3	1	K.KYSHVDLVVMVDGFEGEK.G	Oxidation (M)	(Ions score 35)
167 - 184	517.7584	2067.0045	2066.9983	3	1	K.KYSHVDLVVMVDGFEGEK.G	Oxidation (M)	(Ions score 29)
167 - 184	517.7584	2067.0046	2066.9983	3	1	K.KYSHVDLVVMVDGFEGEK.G	Oxidation (M)	(Ions score 43)
167 - 184	690.0088	2067.0046	2066.9983	3	1	K.KYSHVDLVVMVDGFEGEK.G	Oxidation (M)	(Ions score 45)
167 - 184	517.7584	2067.0046	2066.9983	3	1	K.KYSHVDLVVMVDGFEGEK.G	Oxidation (M)	(Ions score 37)
167 - 184	517.7584	2067.0046	2066.9983	3	1	K.KYSHVDLVVMVDGFEGEK.G	Oxidation (M)	(Ions score 36)
167 - 184	690.0088	2067.0046	2066.9983	3	1	K.KYSHVDLVVMVDGFEGEK.G	Oxidation (M)	(Ions score 55)
167 - 184	517.7584	2067.0046	2066.9983	3	1	K.KYSHVDLVVMVDGFEGEK.G	Oxidation (M)	(Ions score 24)
167 - 184	517.7584	2067.0047	2066.9983	3	1	K.KYSHVDLVVMVDGFEGEK.G	Oxidation (M)	(Ions score 36)
167 - 184	690.0088	2067.0047	2066.9983	3	1	K.KYSHVDLVVMVDGFEGEK.G	Oxidation (M)	(Ions score 28)
167 - 184	690.0088	2067.0047	2066.9983	3	1	K.KYSHVDLVVMVDGFEGEK.G	Oxidation (M)	(Ions score 65)
167 - 184	690.0088	2067.0047	2066.9983	3	1	K.KYSHVDLVVMVDGFEGEK.G	Oxidation (M)	(Ions score 38)
167 - 184	690.0088	2067.0047	2066.9983	3	1	K.KYSHVDLVVMVDGFEGEK.G	Oxidation (M)	(Ions score 34)
167 - 184	517.7585	2067.0047	2066.9983	3	1	K.KYSHVDLVVMVDGFEGEK.G	Oxidation (M)	(Ions score 34)
167 - 184	1034.5097	2067.0048	2066.9983	3	1	K.KYSHVDLVVMVDGFEGEK.G	Oxidation (M)	(Ions score 46)
167 - 184	690.0089	2067.0048	2066.9983	3	1	K.KYSHVDLVVMVDGFEGEK.G	Oxidation (M)	(Ions score 22)
167 - 184	517.7585	2067.0048	2066.9983	3	1	K.KYSHVDLVVMVDGFEGEK.G	Oxidation (M)	(Ions score 33)
167 - 184	517.7585	2067.0048	2066.9983	3	1	K.KYSHVDLVVMVDGFEGEK.G	Oxidation (M)	(Ions score 36)
167 - 184	517.7585	2067.0048	2066.9983	3	1	K.KYSHVDLVVMVDGFEGEK.G	Oxidation (M)	(Ions score 24)
167 - 184	517.7585	2067.0048	2066.9983	3	1	K.KYSHVDLVVMVDGFEGEK.G	Oxidation (M)	(Ions score 39)
167 - 184	517.7585	2067.0049	2066.9983	3	1	K.KYSHVDLVVMVDGFEGEK.G	Oxidation (M)	(Ions score 36)
167 - 184	517.7585	2067.0049	2066.9983	3	1	K.KYSHVDLVVMVDGFEGEK.G	Oxidation (M)	(Ions score 32)
167 - 184	690.0089	2067.0049	2066.9983	3	1	K.KYSHVDLVVMVDGFEGEK.G	Oxidation (M)	(Ions score 42)
167 - 184	517.7585	2067.0049	2066.9983	3	1	K.KYSHVDLVVMVDGFEGEK.G	Oxidation (M)	(Ions score 24)
167 - 184	690.0089	2067.0049	2066.9983	3	1	K.KYSHVDLVVMVDGFEGEK.G	Oxidation (M)	(Ions score 44)
167 - 184	517.7585	2067.0049	2066.9983	3	1	K.KYSHVDLVVMVDGFEGEK.G	Oxidation (M)	(Ions score 25)
167 - 184	517.7585	2067.0049	2066.9983	3	1	K.KYSHVDLVVMVDGFEGEK.G	Oxidation (M)	(Ions score 41)
167 - 184	517.7585	2067.0049	2066.9983	3	1	K.KYSHVDLVVMVDGFEGEK.G	Oxidation (M)	(Ions score 36)
167 - 184	690.0089	2067.0049	2066.9983	3	1	K.KYSHVDLVVMVDGFEGEK.G	Oxidation (M)	(Ions score 67)
167 - 184	517.7585	2067.0050	2066.9983	3	1	K.KYSHVDLVVMVDGFEGEK.G	Oxidation (M)	(Ions score 34)
167 - 184	690.0090	2067.0050	2066.9983	3	1	K.KYSHVDLVVMVDGFEGEK.G	Oxidation (M)	(Ions score 38)
167 - 184	690.0090	2067.0050	2066.9983	3	1	K.KYSHVDLVVMVDGFEGEK.G	Oxidation (M)	(Ions score 43)
167 - 184	517.7585	2067.0051	2066.9983	3	1	K.KYSHVDLVVMVDGFEGEK.G	Oxidation (M)	(Ions score 34)
167 - 184	517.7585	2067.0051	2066.9983	3	1	K.KYSHVDLVVMVDGFEGEK.G	Oxidation (M)	(Ions score 32)
167 - 184	517.7585	2067.0051	2066.9983	3	1	K.KYSHVDLVVMVDGFEGEK.G	Oxidation (M)	(Ions score 30)
167 - 184	690.0090	2067.0051	2066.9983	3	1	K.KYSHVDLVVMVDGFEGEK.G	Oxidation (M)	(Ions score 38)

167 - 184	517.7586	2067.0051	2066.9983	3	1	K.KYSHVDLVVMVDGFEGEK.G	Oxidation (M)	(Ions score 30)
167 - 184	517.7586	2067.0051	2066.9983	3	1	K.KYSHVDLVVMVDGFEGEK.G	Oxidation (M)	(Ions score 35)
167 - 184	517.7586	2067.0052	2066.9983	3	1	K.KYSHVDLVVMVDGFEGEK.G	Oxidation (M)	(Ions score 37)
167 - 184	690.0090	2067.0052	2066.9983	3	1	K.KYSHVDLVVMVDGFEGEK.G	Oxidation (M)	(Ions score 38)
167 - 184	517.7586	2067.0052	2066.9983	3	1	K.KYSHVDLVVMVDGFEGEK.G	Oxidation (M)	(Ions score 37)
167 - 184	517.7586	2067.0052	2066.9983	3	1	K.KYSHVDLVVMVDGFEGEK.G	Oxidation (M)	(Ions score 40)
167 - 184	690.0090	2067.0053	2066.9983	3	1	K.KYSHVDLVVMVDGFEGEK.G	Oxidation (M)	(Ions score 39)
167 - 184	690.0090	2067.0053	2066.9983	3	1	K.KYSHVDLVVMVDGFEGEK.G	Oxidation (M)	(Ions score 45)
167 - 184	517.7586	2067.0053	2066.9983	3	1	K.KYSHVDLVVMVDGFEGEK.G	Oxidation (M)	(Ions score 39)
167 - 184	517.7586	2067.0053	2066.9983	3	1	K.KYSHVDLVVMVDGFEGEK.G	Oxidation (M)	(Ions score 32)
167 - 184	690.0091	2067.0053	2066.9983	3	1	K.KYSHVDLVVMVDGFEGEK.G	Oxidation (M)	(Ions score 39)
167 - 184	690.0091	2067.0054	2066.9983	3	1	K.KYSHVDLVVMVDGFEGEK.G	Oxidation (M)	(Ions score 48)
167 - 184	517.7586	2067.0054	2066.9983	3	1	K.KYSHVDLVVMVDGFEGEK.G	Oxidation (M)	(Ions score 39)
167 - 184	517.7586	2067.0054	2066.9983	3	1	K.KYSHVDLVVMVDGFEGEK.G	Oxidation (M)	(Ions score 30)
167 - 184	517.7586	2067.0054	2066.9983	3	1	K.KYSHVDLVVMVDGFEGEK.G	Oxidation (M)	(Ions score 32)
167 - 184	517.7586	2067.0054	2066.9983	3	1	K.KYSHVDLVVMVDGFEGEK.G	Oxidation (M)	(Ions score 37)
167 - 184	517.7586	2067.0054	2066.9983	3	1	K.KYSHVDLVVMVDGFEGEK.G	Oxidation (M)	(Ions score 35)
167 - 184	690.0091	2067.0055	2066.9983	3	1	K.KYSHVDLVVMVDGFEGEK.G	Oxidation (M)	(Ions score 66)
167 - 184	517.7586	2067.0055	2066.9983	3	1	K.KYSHVDLVVMVDGFEGEK.G	Oxidation (M)	(Ions score 33)
167 - 184	517.7586	2067.0055	2066.9983	3	1	K.KYSHVDLVVMVDGFEGEK.G	Oxidation (M)	(Ions score 32)
167 - 184	690.0091	2067.0055	2066.9983	3	1	K.KYSHVDLVVMVDGFEGEK.G	Oxidation (M)	(Ions score 55)
167 - 184	517.7586	2067.0055	2066.9983	3	1	K.KYSHVDLVVMVDGFEGEK.G	Oxidation (M)	(Ions score 23)
167 - 184	517.7586	2067.0055	2066.9983	3	1	K.KYSHVDLVVMVDGFEGEK.G	Oxidation (M)	(Ions score 33)
167 - 184	517.7586	2067.0055	2066.9983	3	1	K.KYSHVDLVVMVDGFEGEK.G	Oxidation (M)	(Ions score 31)
167 - 184	690.0091	2067.0055	2066.9983	3	1	K.KYSHVDLVVMVDGFEGEK.G	Oxidation (M)	(Ions score 58)
167 - 184	517.7587	2067.0055	2066.9983	3	1	K.KYSHVDLVVMVDGFEGEK.G	Oxidation (M)	(Ions score 41)
167 - 184	517.7587	2067.0055	2066.9983	3	1	K.KYSHVDLVVMVDGFEGEK.G	Oxidation (M)	(Ions score 32)
167 - 184	690.0091	2067.0055	2066.9983	3	1	K.KYSHVDLVVMVDGFEGEK.G	Oxidation (M)	(Ions score 53)
167 - 184	690.0091	2067.0056	2066.9983	4	1	K.KYSHVDLVVMVDGFEGEK.G	Oxidation (M)	(Ions score 45)
167 - 184	517.7587	2067.0056	2066.9983	4	1	K.KYSHVDLVVMVDGFEGEK.G	Oxidation (M)	(Ions score 36)
167 - 184	517.7587	2067.0056	2066.9983	4	1	K.KYSHVDLVVMVDGFEGEK.G	Oxidation (M)	(Ions score 33)
167 - 184	690.0092	2067.0056	2066.9983	4	1	K.KYSHVDLVVMVDGFEGEK.G	Oxidation (M)	(Ions score 29)
167 - 184	517.7587	2067.0057	2066.9983	4	1	K.KYSHVDLVVMVDGFEGEK.G	Oxidation (M)	(Ions score 31)
167 - 184	690.0092	2067.0057	2066.9983	4	1	K.KYSHVDLVVMVDGFEGEK.G	Oxidation (M)	(Ions score 22)
167 - 184	517.7587	2067.0057	2066.9983	4	1	K.KYSHVDLVVMVDGFEGEK.G	Oxidation (M)	(Ions score 36)
167 - 184	690.0092	2067.0057	2066.9983	4	1	K.KYSHVDLVVMVDGFEGEK.G	Oxidation (M)	(Ions score 41)
167 - 184	517.7587	2067.0057	2066.9983	4	1	K.KYSHVDLVVMVDGFEGEK.G	Oxidation (M)	(Ions score 25)
167 - 184	690.0092	2067.0057	2066.9983	4	1	K.KYSHVDLVVMVDGFEGEK.G	Oxidation (M)	(Ions score 51)
167 - 184	690.0092	2067.0057	2066.9983	4	1	K.KYSHVDLVVMVDGFEGEK.G	Oxidation (M)	(Ions score 54)
167 - 184	517.7587	2067.0057	2066.9983	4	1	K.KYSHVDLVVMVDGFEGEK.G	Oxidation (M)	(Ions score 36)
167 - 184	690.0092	2067.0057	2066.9983	4	1	K.KYSHVDLVVMVDGFEGEK.G	Oxidation (M)	(Ions score 24)
167 - 184	517.7587	2067.0058	2066.9983	4	1	K.KYSHVDLVVMVDGFEGEK.G	Oxidation (M)	(Ions score 36)
167 - 184	517.7587	2067.0058	2066.9983	4	1	K.KYSHVDLVVMVDGFEGEK.G	Oxidation (M)	(Ions score 31)
167 - 184	690.0092	2067.0059	2066.9983	4	1	K.KYSHVDLVVMVDGFEGEK.G	Oxidation (M)	(Ions score 46)
167 - 184	517.7587	2067.0059	2066.9983	4	1	K.KYSHVDLVVMVDGFEGEK.G	Oxidation (M)	(Ions score 32)
167 - 184	517.7587	2067.0059	2066.9983	4	1	K.KYSHVDLVVMVDGFEGEK.G	Oxidation (M)	(Ions score 32)
167 - 184	690.0092	2067.0059	2066.9983	4	1	K.KYSHVDLVVMVDGFEGEK.G	Oxidation (M)	(Ions score 41)
167 - 184	690.0092	2067.0059	2066.9983	4	1	K.KYSHVDLVVMVDGFEGEK.G	Oxidation (M)	(Ions score 42)
167 - 184	517.7588	2067.0059	2066.9983	4	1	K.KYSHVDLVVMVDGFEGEK.G	Oxidation (M)	(Ions score 36)
167 - 184	690.0093	2067.0060	2066.9983	4	1	K.KYSHVDLVVMVDGFEGEK.G	Oxidation (M)	(Ions score 45)
167 - 184	517.7588	2067.0060	2066.9983	4	1	K.KYSHVDLVVMVDGFEGEK.G	Oxidation (M)	(Ions score 42)
167 - 184	517.7588	2067.0060	2066.9983	4	1	K.KYSHVDLVVMVDGFEGEK.G	Oxidation (M)	(Ions score 37)
167 - 184	690.0093	2067.0061	2066.9983	4	1	K.KYSHVDLVVMVDGFEGEK.G	Oxidation (M)	(Ions score 46)
167 - 184	517.7588	2067.0061	2066.9983	4	1	K.KYSHVDLVVMVDGFEGEK.G	Oxidation (M)	(Ions score 29)
167 - 184	517.7588	2067.0061	2066.9983	4	1	K.KYSHVDLVVMVDGFEGEK.G	Oxidation (M)	(Ions score 34)
167 - 184	517.7588	2067.0062	2066.9983	4	1	K.KYSHVDLVVMVDGFEGEK.G	Oxidation (M)	(Ions score 41)
167 - 184	517.7588	2067.0062	2066.9983	4	1	K.KYSHVDLVVMVDGFEGEK.G	Oxidation (M)	(Ions score 31)
167 - 184	517.7588	2067.0063	2066.9983	4	1	K.KYSHVDLVVMVDGFEGEK.G	Oxidation (M)	(Ions score 36)

167 - 184	690.0094	2067.0063	2066.9983	4	1	K.KYSHVDLVVMVDGFEGEK.G	Oxidation (M)	(Ions score 64)
167 - 184	517.7589	2067.0063	2066.9983	4	1	K.KYSHVDLVVMVDGFEGEK.G	Oxidation (M)	(Ions score 37)
167 - 184	690.0094	2067.0063	2066.9983	4	1	K.KYSHVDLVVMVDGFEGEK.G	Oxidation (M)	(Ions score 77)
167 - 184	517.7589	2067.0063	2066.9983	4	1	K.KYSHVDLVVMVDGFEGEK.G	Oxidation (M)	(Ions score 34)
167 - 184	690.0094	2067.0063	2066.9983	4	1	K.KYSHVDLVVMVDGFEGEK.G	Oxidation (M)	(Ions score 54)
167 - 184	690.0094	2067.0064	2066.9983	4	1	K.KYSHVDLVVMVDGFEGEK.G	Oxidation (M)	(Ions score 37)
167 - 184	517.7589	2067.0064	2066.9983	4	1	K.KYSHVDLVVMVDGFEGEK.G	Oxidation (M)	(Ions score 37)
167 - 184	690.0094	2067.0064	2066.9983	4	1	K.KYSHVDLVVMVDGFEGEK.G	Oxidation (M)	(Ions score 36)
167 - 184	690.0094	2067.0064	2066.9983	4	1	K.KYSHVDLVVMVDGFEGEK.G	Oxidation (M)	(Ions score 45)
167 - 184	517.7589	2067.0064	2066.9983	4	1	K.KYSHVDLVVMVDGFEGEK.G	Oxidation (M)	(Ions score 25)
167 - 184	1034.5105	2067.0065	2066.9983	4	1	K.KYSHVDLVVMVDGFEGEK.G	Oxidation (M)	(Ions score 42)
167 - 184	1034.5105	2067.0065	2066.9983	4	1	K.KYSHVDLVVMVDGFEGEK.G	Oxidation (M)	(Ions score 57)
167 - 184	690.0094	2067.0065	2066.9983	4	1	K.KYSHVDLVVMVDGFEGEK.G	Oxidation (M)	(Ions score 42)
167 - 184	690.0095	2067.0066	2066.9983	4	1	K.KYSHVDLVVMVDGFEGEK.G	Oxidation (M)	(Ions score 34)
167 - 184	517.7589	2067.0066	2066.9983	4	1	K.KYSHVDLVVMVDGFEGEK.G	Oxidation (M)	(Ions score 31)
167 - 184	690.0095	2067.0066	2066.9983	4	1	K.KYSHVDLVVMVDGFEGEK.G	Oxidation (M)	(Ions score 43)
167 - 184	517.7589	2067.0067	2066.9983	4	1	K.KYSHVDLVVMVDGFEGEK.G	Oxidation (M)	(Ions score 42)
167 - 184	517.7589	2067.0067	2066.9983	4	1	K.KYSHVDLVVMVDGFEGEK.G	Oxidation (M)	(Ions score 37)
167 - 184	517.7590	2067.0068	2066.9983	4	1	K.KYSHVDLVVMVDGFEGEK.G	Oxidation (M)	(Ions score 26)
167 - 184	517.7590	2067.0068	2066.9983	4	1	K.KYSHVDLVVMVDGFEGEK.G	Oxidation (M)	(Ions score 34)
167 - 184	690.0095	2067.0068	2066.9983	4	1	K.KYSHVDLVVMVDGFEGEK.G	Oxidation (M)	(Ions score 46)
167 - 184	690.0095	2067.0068	2066.9983	4	1	K.KYSHVDLVVMVDGFEGEK.G	Oxidation (M)	(Ions score 43)
167 - 184	517.7590	2067.0068	2066.9983	4	1	K.KYSHVDLVVMVDGFEGEK.G	Oxidation (M)	(Ions score 31)
167 - 184	690.0095	2067.0068	2066.9983	4	1	K.KYSHVDLVVMVDGFEGEK.G	Oxidation (M)	(Ions score 31)
167 - 184	517.7590	2067.0068	2066.9983	4	1	K.KYSHVDLVVMVDGFEGEK.G	Oxidation (M)	(Ions score 28)
167 - 184	690.0095	2067.0068	2066.9983	4	1	K.KYSHVDLVVMVDGFEGEK.G	Oxidation (M)	(Ions score 42)
167 - 184	690.0095	2067.0068	2066.9983	4	1	K.KYSHVDLVVMVDGFEGEK.G	Oxidation (M)	(Ions score 34)
167 - 184	690.0095	2067.0068	2066.9983	4	1	K.KYSHVDLVVMVDGFEGEK.G	Oxidation (M)	(Ions score 22)
167 - 184	517.7590	2067.0068	2066.9983	4	1	K.KYSHVDLVVMVDGFEGEK.G	Oxidation (M)	(Ions score 36)
167 - 184	517.7590	2067.0068	2066.9983	4	1	K.KYSHVDLVVMVDGFEGEK.G	Oxidation (M)	(Ions score 30)
167 - 184	517.7590	2067.0068	2066.9983	4	1	K.KYSHVDLVVMVDGFEGEK.G	Oxidation (M)	(Ions score 34)
167 - 184	690.0096	2067.0069	2066.9983	4	1	K.KYSHVDLVVMVDGFEGEK.G	Oxidation (M)	(Ions score 34)
167 - 184	517.7590	2067.0069	2066.9983	4	1	K.KYSHVDLVVMVDGFEGEK.G	Oxidation (M)	(Ions score 34)
167 - 184	517.7590	2067.0069	2066.9983	4	1	K.KYSHVDLVVMVDGFEGEK.G	Oxidation (M)	(Ions score 38)
167 - 184	517.7590	2067.0069	2066.9983	4	1	K.KYSHVDLVVMVDGFEGEK.G	Oxidation (M)	(Ions score 28)
167 - 184	1034.5108	2067.0070	2066.9983	4	1	K.KYSHVDLVVMVDGFEGEK.G	Oxidation (M)	(Ions score 50)
167 - 184	517.7590	2067.0070	2066.9983	4	1	K.KYSHVDLVVMVDGFEGEK.G	Oxidation (M)	(Ions score 38)
167 - 184	690.0096	2067.0070	2066.9983	4	1	K.KYSHVDLVVMVDGFEGEK.G	Oxidation (M)	(Ions score 36)
167 - 184	517.7591	2067.0071	2066.9983	4	1	K.KYSHVDLVVMVDGFEGEK.G	Oxidation (M)	(Ions score 42)
167 - 184	690.0097	2067.0071	2066.9983	4	1	K.KYSHVDLVVMVDGFEGEK.G	Oxidation (M)	(Ions score 50)
167 - 184	690.0097	2067.0072	2066.9983	4	1	K.KYSHVDLVVMVDGFEGEK.G	Oxidation (M)	(Ions score 50)
167 - 184	517.7591	2067.0073	2066.9983	4	1	K.KYSHVDLVVMVDGFEGEK.G	Oxidation (M)	(Ions score 37)
167 - 184	517.7591	2067.0073	2066.9983	4	1	K.KYSHVDLVVMVDGFEGEK.G	Oxidation (M)	(Ions score 33)
167 - 184	517.7591	2067.0073	2066.9983	4	1	K.KYSHVDLVVMVDGFEGEK.G	Oxidation (M)	(Ions score 36)
167 - 184	517.7591	2067.0073	2066.9983	4	1	K.KYSHVDLVVMVDGFEGEK.G	Oxidation (M)	(Ions score 36)
167 - 184	517.7591	2067.0074	2066.9983	4	1	K.KYSHVDLVVMVDGFEGEK.G	Oxidation (M)	(Ions score 36)
167 - 184	517.7591	2067.0074	2066.9983	4	1	K.KYSHVDLVVMVDGFEGEK.G	Oxidation (M)	(Ions score 42)
167 - 184	690.0098	2067.0076	2066.9983	5	1	K.KYSHVDLVVMVDGFEGEK.G	Oxidation (M)	(Ions score 30)
167 - 184	517.7592	2067.0076	2066.9983	5	1	K.KYSHVDLVVMVDGFEGEK.G	Oxidation (M)	(Ions score 26)
167 - 184	517.7592	2067.0076	2066.9983	5	1	K.KYSHVDLVVMVDGFEGEK.G	Oxidation (M)	(Ions score 34)
167 - 184	517.7592	2067.0077	2066.9983	5	1	K.KYSHVDLVVMVDGFEGEK.G	Oxidation (M)	(Ions score 36)
167 - 184	690.0099	2067.0077	2066.9983	5	1	K.KYSHVDLVVMVDGFEGEK.G	Oxidation (M)	(Ions score 53)
167 - 184	690.0099	2067.0078	2066.9983	5	1	K.KYSHVDLVVMVDGFEGEK.G	Oxidation (M)	(Ions score 53)
167 - 184	690.0099	2067.0078	2066.9983	5	1	K.KYSHVDLVVMVDGFEGEK.G	Oxidation (M)	(Ions score 43)
167 - 184	517.7593	2067.0079	2066.9983	5	1	K.KYSHVDLVVMVDGFEGEK.G	Oxidation (M)	(Ions score 36)
167 - 184	517.7593	2067.0079	2066.9983	5	1	K.KYSHVDLVVMVDGFEGEK.G	Oxidation (M)	(Ions score 29)
167 - 184	517.7593	2067.0080	2066.9983	5	1	K.KYSHVDLVVMVDGFEGEK.G	Oxidation (M)	(Ions score 31)
167 - 184	517.7593	2067.0080	2066.9983	5	1	K.KYSHVDLVVMVDGFEGEK.G	Oxidation (M)	(Ions score 31)

167 - 184	690.0099	2067.0080	2066.9983	5	1	K.KYSHVDLVVMVDGFEGEK.G	Oxidation (M)	(Ions score 26)
167 - 184	517.7593	2067.0081	2066.9983	5	1	K.KYSHVDLVVMVDGFEGEK.G	Oxidation (M)	(Ions score 38)
167 - 184	690.0100	2067.0081	2066.9983	5	1	K.KYSHVDLVVMVDGFEGEK.G	Oxidation (M)	(Ions score 44)
167 - 184	517.7593	2067.0081	2066.9983	5	1	K.KYSHVDLVVMVDGFEGEK.G	Oxidation (M)	(Ions score 39)
167 - 184	517.7593	2067.0081	2066.9983	5	1	K.KYSHVDLVVMVDGFEGEK.G	Oxidation (M)	(Ions score 30)
167 - 184	690.0100	2067.0082	2066.9983	5	1	K.KYSHVDLVVMVDGFEGEK.G	Oxidation (M)	(Ions score 33)
167 - 184	690.0100	2067.0082	2066.9983	5	1	K.KYSHVDLVVMVDGFEGEK.G	Oxidation (M)	(Ions score 57)
167 - 184	690.0100	2067.0083	2066.9983	5	1	K.KYSHVDLVVMVDGFEGEK.G	Oxidation (M)	(Ions score 55)
167 - 184	1034.5114	2067.0083	2066.9983	5	1	K.KYSHVDLVVMVDGFEGEK.G	Oxidation (M)	(Ions score 38)
167 - 184	690.0100	2067.0083	2066.9983	5	1	K.KYSHVDLVVMVDGFEGEK.G	Oxidation (M)	(Ions score 37)
167 - 184	517.7594	2067.0083	2066.9983	5	1	K.KYSHVDLVVMVDGFEGEK.G	Oxidation (M)	(Ions score 27)
167 - 184	517.7594	2067.0083	2066.9983	5	1	K.KYSHVDLVVMVDGFEGEK.G	Oxidation (M)	(Ions score 37)
167 - 184	690.0101	2067.0084	2066.9983	5	1	K.KYSHVDLVVMVDGFEGEK.G	Oxidation (M)	(Ions score 32)
167 - 184	690.0101	2067.0085	2066.9983	5	1	K.KYSHVDLVVMVDGFEGEK.G	Oxidation (M)	(Ions score 55)
167 - 184	690.0101	2067.0085	2066.9983	5	1	K.KYSHVDLVVMVDGFEGEK.G	Oxidation (M)	(Ions score 56)
167 - 184	698.6784	2093.0132	2093.0139	-0	1	K.KYSHVDLVVMVDGFEGEK.G	Acetylation (K)	(Ions score 53)
167 - 184	698.6792	2093.0156	2093.0139	1	1	K.KYSHVDLVVMVDGFEGEK.G	Acetylation (K)	(Ions score 65)
167 - 184	698.6794	2093.0162	2093.0139	1	1	K.KYSHVDLVVMVDGFEGEK.G	Acetylation (K)	(Ions score 41)
167 - 184	524.2614	2093.0166	2093.0139	1	1	K.KYSHVDLVVMVDGFEGEK.G	Acetylation (K)	(Ions score 49)
167 - 184	698.6799	2093.0180	2093.0139	2	1	K.KYSHVDLVVMVDGFEGEK.G	Acetylation (K)	(Ions score 34)
167 - 184	524.2619	2093.0186	2093.0139	2	1	K.KYSHVDLVVMVDGFEGEK.G	Acetylation (K)	(Ions score 58)
167 - 184	524.2623	2093.0201	2093.0139	3	1	K.KYSHVDLVVMVDGFEGEK.G	Acetylation (K)	(Ions score 57)
167 - 184	704.0107	2109.0102	2109.0089	1	1	K.KYSHVDLVVMVDGFEGEK.G	Acetylation (K); Oxidation (M)	(Ions score 59)
167 - 184	704.0134	2109.0185	2109.0089	5	1	K.KYSHVDLVVMVDGFEGEK.G	Acetylation (K); Oxidation (M)	(Ions score 29)
167 - 184	704.0135	2109.0187	2109.0089	5	1	K.KYSHVDLVVMVDGFEGEK.G	Acetylation (K); Oxidation (M)	(Ions score 60)
168 - 184	962.4569	1922.8992	1922.9084	-5	0	K.YSHVDLVVMVDGFEGEK.G		(Ions score 63)
168 - 184	962.4572	1922.8999	1922.9084	-4	0	K.YSHVDLVVMVDGFEGEK.G		(Ions score 98)
168 - 184	962.4580	1922.9014	1922.9084	-4	0	K.YSHVDLVVMVDGFEGEK.G		(Ions score 75)
168 - 184	962.4581	1922.9016	1922.9084	-4	0	K.YSHVDLVVMVDGFEGEK.G		(Ions score 67)
168 - 184	962.4586	1922.9027	1922.9084	-3	0	K.YSHVDLVVMVDGFEGEK.G		(Ions score 66)
168 - 184	962.4588	1922.9031	1922.9084	-3	0	K.YSHVDLVVMVDGFEGEK.G		(Ions score 73)
168 - 184	962.4590	1922.9035	1922.9084	-3	0	K.YSHVDLVVMVDGFEGEK.G		(Ions score 62)
168 - 184	962.4592	1922.9038	1922.9084	-2	0	K.YSHVDLVVMVDGFEGEK.G		(Ions score 86)
168 - 184	641.9753	1922.9041	1922.9084	-2	0	K.YSHVDLVVMVDGFEGEK.G		(Ions score 49)
168 - 184	962.4594	1922.9043	1922.9084	-2	0	K.YSHVDLVVMVDGFEGEK.G		(Ions score 39)
168 - 184	962.4596	1922.9047	1922.9084	-2	0	K.YSHVDLVVMVDGFEGEK.G		(Ions score 74)
168 - 184	641.9755	1922.9047	1922.9084	-2	0	K.YSHVDLVVMVDGFEGEK.G		(Ions score 24)
168 - 184	962.4598	1922.9050	1922.9084	-2	0	K.YSHVDLVVMVDGFEGEK.G		(Ions score 44)
168 - 184	962.4598	1922.9050	1922.9084	-2	0	K.YSHVDLVVMVDGFEGEK.G		(Ions score 87)
168 - 184	962.4599	1922.9053	1922.9084	-2	0	K.YSHVDLVVMVDGFEGEK.G		(Ions score 70)
168 - 184	962.4600	1922.9055	1922.9084	-2	0	K.YSHVDLVVMVDGFEGEK.G		(Ions score 82)
168 - 184	962.4601	1922.9057	1922.9084	-1	0	K.YSHVDLVVMVDGFEGEK.G		(Ions score 74)
168 - 184	962.4603	1922.9060	1922.9084	-1	0	K.YSHVDLVVMVDGFEGEK.G		(Ions score 91)
168 - 184	962.4603	1922.9061	1922.9084	-1	0	K.YSHVDLVVMVDGFEGEK.G		(Ions score 91)
168 - 184	962.4603	1922.9061	1922.9084	-1	0	K.YSHVDLVVMVDGFEGEK.G		(Ions score 89)
168 - 184	962.4605	1922.9065	1922.9084	-1	0	K.YSHVDLVVMVDGFEGEK.G		(Ions score 88)
168 - 184	641.9763	1922.9071	1922.9084	-1	0	K.YSHVDLVVMVDGFEGEK.G		(Ions score 25)
168 - 184	641.9764	1922.9073	1922.9084	-1	0	K.YSHVDLVVMVDGFEGEK.G		(Ions score 31)
168 - 184	962.4610	1922.9075	1922.9084	-1	0	K.YSHVDLVVMVDGFEGEK.G		(Ions score 91)
168 - 184	962.4611	1922.9076	1922.9084	-0	0	K.YSHVDLVVMVDGFEGEK.G		(Ions score 92)
168 - 184	962.4612	1922.9078	1922.9084	-0	0	K.YSHVDLVVMVDGFEGEK.G		(Ions score 92)
168 - 184	962.4612	1922.9078	1922.9084	-0	0	K.YSHVDLVVMVDGFEGEK.G		(Ions score 79)
168 - 184	962.4612	1922.9079	1922.9084	-0	0	K.YSHVDLVVMVDGFEGEK.G		(Ions score 65)
168 - 184	962.4613	1922.9080	1922.9084	-0	0	K.YSHVDLVVMVDGFEGEK.G		(Ions score 108)
168 - 184	641.9766	1922.9080	1922.9084	-0	0	K.YSHVDLVVMVDGFEGEK.G		(Ions score 60)
168 - 184	962.4613	1922.9081	1922.9084	-0	0	K.YSHVDLVVMVDGFEGEK.G		(Ions score 60)
168 - 184	962.4613	1922.9081	1922.9084	-0	0	K.YSHVDLVVMVDGFEGEK.G		(Ions score 74)
168 - 184	962.4613	1922.9081	1922.9084	-0	0	K.YSHVDLVVMVDGFEGEK.G		(Ions score 74)

168 - 184	641.9767	1922.9082	1922.9084	-0	0	K.YSHVDLVVMVDGFEGEK.G	(Ions score 39)
168 - 184	641.9767	1922.9083	1922.9084	-0	0	K.YSHVDLVVMVDGFEGEK.G	(Ions score 68)
168 - 184	962.4615	1922.9084	1922.9084	-0	0	K.YSHVDLVVMVDGFEGEK.G	(Ions score 72)
168 - 184	962.4615	1922.9085	1922.9084	0	0	K.YSHVDLVVMVDGFEGEK.G	(Ions score 74)
168 - 184	962.4615	1922.9085	1922.9084	0	0	K.YSHVDLVVMVDGFEGEK.G	(Ions score 71)
168 - 184	641.9768	1922.9086	1922.9084	0	0	K.YSHVDLVVMVDGFEGEK.G	(Ions score 76)
168 - 184	641.9768	1922.9086	1922.9084	0	0	K.YSHVDLVVMVDGFEGEK.G	(Ions score 68)
168 - 184	962.4616	1922.9087	1922.9084	0	0	K.YSHVDLVVMVDGFEGEK.G	(Ions score 73)
168 - 184	962.4616	1922.9087	1922.9084	0	0	K.YSHVDLVVMVDGFEGEK.G	(Ions score 91)
168 - 184	962.4616	1922.9087	1922.9084	0	0	K.YSHVDLVVMVDGFEGEK.G	(Ions score 77)
168 - 184	962.4617	1922.9088	1922.9084	0	0	K.YSHVDLVVMVDGFEGEK.G	(Ions score 76)
168 - 184	962.4617	1922.9088	1922.9084	0	0	K.YSHVDLVVMVDGFEGEK.G	(Ions score 93)
168 - 184	962.4617	1922.9088	1922.9084	0	0	K.YSHVDLVVMVDGFEGEK.G	(Ions score 92)
168 - 184	962.4618	1922.9090	1922.9084	0	0	K.YSHVDLVVMVDGFEGEK.G	(Ions score 78)
168 - 184	962.4618	1922.9090	1922.9084	0	0	K.YSHVDLVVMVDGFEGEK.G	(Ions score 85)
168 - 184	641.9769	1922.9090	1922.9084	0	0	K.YSHVDLVVMVDGFEGEK.G	(Ions score 76)
168 - 184	962.4618	1922.9090	1922.9084	0	0	K.YSHVDLVVMVDGFEGEK.G	(Ions score 100)
168 - 184	641.9770	1922.9091	1922.9084	0	0	K.YSHVDLVVMVDGFEGEK.G	(Ions score 44)
168 - 184	641.9770	1922.9091	1922.9084	0	0	K.YSHVDLVVMVDGFEGEK.G	(Ions score 42)
168 - 184	641.9770	1922.9092	1922.9084	0	0	K.YSHVDLVVMVDGFEGEK.G	(Ions score 73)
168 - 184	962.4619	1922.9093	1922.9084	0	0	K.YSHVDLVVMVDGFEGEK.G	(Ions score 97)
168 - 184	962.4619	1922.9093	1922.9084	0	0	K.YSHVDLVVMVDGFEGEK.G	(Ions score 76)
168 - 184	962.4619	1922.9093	1922.9084	0	0	K.YSHVDLVVMVDGFEGEK.G	(Ions score 71)
168 - 184	962.4619	1922.9093	1922.9084	0	0	K.YSHVDLVVMVDGFEGEK.G	(Ions score 86)
168 - 184	962.4620	1922.9094	1922.9084	0	0	K.YSHVDLVVMVDGFEGEK.G	(Ions score 76)
168 - 184	641.9771	1922.9095	1922.9084	1	0	K.YSHVDLVVMVDGFEGEK.G	(Ions score 76)
168 - 184	962.4620	1922.9095	1922.9084	1	0	K.YSHVDLVVMVDGFEGEK.G	(Ions score 76)
168 - 184	641.9771	1922.9095	1922.9084	1	0	K.YSHVDLVVMVDGFEGEK.G	(Ions score 25)
168 - 184	962.4621	1922.9096	1922.9084	1	0	K.YSHVDLVVMVDGFEGEK.G	(Ions score 88)
168 - 184	962.4621	1922.9096	1922.9084	1	0	K.YSHVDLVVMVDGFEGEK.G	(Ions score 103)
168 - 184	962.4621	1922.9096	1922.9084	1	0	K.YSHVDLVVMVDGFEGEK.G	(Ions score 92)
168 - 184	962.4621	1922.9097	1922.9084	1	0	K.YSHVDLVVMVDGFEGEK.G	(Ions score 73)
168 - 184	962.4622	1922.9098	1922.9084	1	0	K.YSHVDLVVMVDGFEGEK.G	(Ions score 94)
168 - 184	641.9772	1922.9098	1922.9084	1	0	K.YSHVDLVVMVDGFEGEK.G	(Ions score 55)
168 - 184	962.4622	1922.9098	1922.9084	1	0	K.YSHVDLVVMVDGFEGEK.G	(Ions score 88)
168 - 184	962.4622	1922.9098	1922.9084	1	0	K.YSHVDLVVMVDGFEGEK.G	(Ions score 104)
168 - 184	641.9772	1922.9098	1922.9084	1	0	K.YSHVDLVVMVDGFEGEK.G	(Ions score 64)
168 - 184	962.4622	1922.9098	1922.9084	1	0	K.YSHVDLVVMVDGFEGEK.G	(Ions score 92)
168 - 184	962.4622	1922.9098	1922.9084	1	0	K.YSHVDLVVMVDGFEGEK.G	(Ions score 91)
168 - 184	641.9772	1922.9098	1922.9084	1	0	K.YSHVDLVVMVDGFEGEK.G	(Ions score 41)
168 - 184	641.9772	1922.9099	1922.9084	1	0	K.YSHVDLVVMVDGFEGEK.G	(Ions score 65)
168 - 184	641.9772	1922.9099	1922.9084	1	0	K.YSHVDLVVMVDGFEGEK.G	(Ions score 48)
168 - 184	962.4623	1922.9100	1922.9084	1	0	K.YSHVDLVVMVDGFEGEK.G	(Ions score 64)
168 - 184	962.4623	1922.9100	1922.9084	1	0	K.YSHVDLVVMVDGFEGEK.G	(Ions score 90)
168 - 184	962.4623	1922.9100	1922.9084	1	0	K.YSHVDLVVMVDGFEGEK.G	(Ions score 75)
168 - 184	962.4623	1922.9100	1922.9084	1	0	K.YSHVDLVVMVDGFEGEK.G	(Ions score 92)
168 - 184	641.9773	1922.9100	1922.9084	1	0	K.YSHVDLVVMVDGFEGEK.G	(Ions score 70)
168 - 184	641.9773	1922.9100	1922.9084	1	0	K.YSHVDLVVMVDGFEGEK.G	(Ions score 76)
168 - 184	962.4623	1922.9101	1922.9084	1	0	K.YSHVDLVVMVDGFEGEK.G	(Ions score 105)
168 - 184	641.9773	1922.9101	1922.9084	1	0	K.YSHVDLVVMVDGFEGEK.G	(Ions score 60)
168 - 184	641.9773	1922.9101	1922.9084	1	0	K.YSHVDLVVMVDGFEGEK.G	(Ions score 73)
168 - 184	962.4623	1922.9101	1922.9084	1	0	K.YSHVDLVVMVDGFEGEK.G	(Ions score 60)
168 - 184	641.9773	1922.9102	1922.9084	1	0	K.YSHVDLVVMVDGFEGEK.G	(Ions score 59)
168 - 184	641.9773	1922.9102	1922.9084	1	0	K.YSHVDLVVMVDGFEGEK.G	(Ions score 70)
168 - 184	641.9773	1922.9102	1922.9084	1	0	K.YSHVDLVVMVDGFEGEK.G	(Ions score 64)
168 - 184	641.9773	1922.9102	1922.9084	1	0	K.YSHVDLVVMVDGFEGEK.G	(Ions score 73)
168 - 184	641.9773	1922.9102	1922.9084	1	0	K.YSHVDLVVMVDGFEGEK.G	(Ions score 75)
168 - 184	962.4624	1922.9102	1922.9084	1	0	K.YSHVDLVVMVDGFEGEK.G	(Ions score 80)

168 - 184	962.4624	1922.9102	1922.9084	1	0	K.YSHVDLVVMVDGFEGEK.G	(Ions score 85)
168 - 184	641.9774	1922.9103	1922.9084	1	0	K.YSHVDLVVMVDGFEGEK.G	(Ions score 73)
168 - 184	962.4624	1922.9103	1922.9084	1	0	K.YSHVDLVVMVDGFEGEK.G	(Ions score 90)
168 - 184	962.4624	1922.9103	1922.9084	1	0	K.YSHVDLVVMVDGFEGEK.G	(Ions score 90)
168 - 184	641.9774	1922.9103	1922.9084	1	0	K.YSHVDLVVMVDGFEGEK.G	(Ions score 59)
168 - 184	641.9774	1922.9104	1922.9084	1	0	K.YSHVDLVVMVDGFEGEK.G	(Ions score 73)
168 - 184	641.9774	1922.9104	1922.9084	1	0	K.YSHVDLVVMVDGFEGEK.G	(Ions score 73)
168 - 184	641.9774	1922.9104	1922.9084	1	0	K.YSHVDLVVMVDGFEGEK.G	(Ions score 38)
168 - 184	641.9774	1922.9104	1922.9084	1	0	K.YSHVDLVVMVDGFEGEK.G	(Ions score 89)
168 - 184	641.9774	1922.9104	1922.9084	1	0	K.YSHVDLVVMVDGFEGEK.G	(Ions score 73)
168 - 184	962.4625	1922.9104	1922.9084	1	0	K.YSHVDLVVMVDGFEGEK.G	(Ions score 78)
168 - 184	962.4625	1922.9104	1922.9084	1	0	K.YSHVDLVVMVDGFEGEK.G	(Ions score 64)
168 - 184	641.9774	1922.9105	1922.9084	1	0	K.YSHVDLVVMVDGFEGEK.G	(Ions score 76)
168 - 184	641.9774	1922.9105	1922.9084	1	0	K.YSHVDLVVMVDGFEGEK.G	(Ions score 40)
168 - 184	641.9774	1922.9105	1922.9084	1	0	K.YSHVDLVVMVDGFEGEK.G	(Ions score 76)
168 - 184	962.4625	1922.9105	1922.9084	1	0	K.YSHVDLVVMVDGFEGEK.G	(Ions score 75)
168 - 184	641.9774	1922.9105	1922.9084	1	0	K.YSHVDLVVMVDGFEGEK.G	(Ions score 73)
168 - 184	962.4625	1922.9105	1922.9084	1	0	K.YSHVDLVVMVDGFEGEK.G	(Ions score 81)
168 - 184	962.4625	1922.9105	1922.9084	1	0	K.YSHVDLVVMVDGFEGEK.G	(Ions score 105)
168 - 184	962.4625	1922.9105	1922.9084	1	0	K.YSHVDLVVMVDGFEGEK.G	(Ions score 89)
168 - 184	641.9775	1922.9105	1922.9084	1	0	K.YSHVDLVVMVDGFEGEK.G	(Ions score 70)
168 - 184	641.9775	1922.9105	1922.9084	1	0	K.YSHVDLVVMVDGFEGEK.G	(Ions score 76)
168 - 184	962.4626	1922.9106	1922.9084	1	0	K.YSHVDLVVMVDGFEGEK.G	(Ions score 97)
168 - 184	641.9775	1922.9106	1922.9084	1	0	K.YSHVDLVVMVDGFEGEK.G	(Ions score 76)
168 - 184	962.4626	1922.9106	1922.9084	1	0	K.YSHVDLVVMVDGFEGEK.G	(Ions score 99)
168 - 184	641.9775	1922.9106	1922.9084	1	0	K.YSHVDLVVMVDGFEGEK.G	(Ions score 59)
168 - 184	962.4626	1922.9106	1922.9084	1	0	K.YSHVDLVVMVDGFEGEK.G	(Ions score 72)
168 - 184	962.4626	1922.9107	1922.9084	1	0	K.YSHVDLVVMVDGFEGEK.G	(Ions score 81)
168 - 184	641.9775	1922.9107	1922.9084	1	0	K.YSHVDLVVMVDGFEGEK.G	(Ions score 64)
168 - 184	641.9775	1922.9107	1922.9084	1	0	K.YSHVDLVVMVDGFEGEK.G	(Ions score 59)
168 - 184	962.4626	1922.9107	1922.9084	1	0	K.YSHVDLVVMVDGFEGEK.G	(Ions score 97)
168 - 184	962.4626	1922.9107	1922.9084	1	0	K.YSHVDLVVMVDGFEGEK.G	(Ions score 92)
168 - 184	641.9775	1922.9107	1922.9084	1	0	K.YSHVDLVVMVDGFEGEK.G	(Ions score 66)
168 - 184	641.9775	1922.9108	1922.9084	1	0	K.YSHVDLVVMVDGFEGEK.G	(Ions score 73)
168 - 184	962.4627	1922.9108	1922.9084	1	0	K.YSHVDLVVMVDGFEGEK.G	(Ions score 80)
168 - 184	962.4627	1922.9109	1922.9084	1	0	K.YSHVDLVVMVDGFEGEK.G	(Ions score 78)
168 - 184	641.9776	1922.9109	1922.9084	1	0	K.YSHVDLVVMVDGFEGEK.G	(Ions score 67)
168 - 184	962.4627	1922.9109	1922.9084	1	0	K.YSHVDLVVMVDGFEGEK.G	(Ions score 92)
168 - 184	641.9776	1922.9109	1922.9084	1	0	K.YSHVDLVVMVDGFEGEK.G	(Ions score 55)
168 - 184	641.9776	1922.9109	1922.9084	1	0	K.YSHVDLVVMVDGFEGEK.G	(Ions score 51)
168 - 184	641.9776	1922.9109	1922.9084	1	0	K.YSHVDLVVMVDGFEGEK.G	(Ions score 39)
168 - 184	962.4628	1922.9109	1922.9084	1	0	K.YSHVDLVVMVDGFEGEK.G	(Ions score 79)
168 - 184	641.9776	1922.9110	1922.9084	1	0	K.YSHVDLVVMVDGFEGEK.G	(Ions score 56)
168 - 184	962.4628	1922.9110	1922.9084	1	0	K.YSHVDLVVMVDGFEGEK.G	(Ions score 75)
168 - 184	641.9776	1922.9110	1922.9084	1	0	K.YSHVDLVVMVDGFEGEK.G	(Ions score 73)
168 - 184	641.9776	1922.9110	1922.9084	1	0	K.YSHVDLVVMVDGFEGEK.G	(Ions score 76)
168 - 184	641.9776	1922.9110	1922.9084	1	0	K.YSHVDLVVMVDGFEGEK.G	(Ions score 73)
168 - 184	641.9776	1922.9110	1922.9084	1	0	K.YSHVDLVVMVDGFEGEK.G	(Ions score 69)
168 - 184	962.4628	1922.9111	1922.9084	1	0	K.YSHVDLVVMVDGFEGEK.G	(Ions score 76)
168 - 184	962.4628	1922.9111	1922.9084	1	0	K.YSHVDLVVMVDGFEGEK.G	(Ions score 80)
168 - 184	641.9776	1922.9111	1922.9084	1	0	K.YSHVDLVVMVDGFEGEK.G	(Ions score 64)
168 - 184	962.4628	1922.9111	1922.9084	1	0	K.YSHVDLVVMVDGFEGEK.G	(Ions score 85)
168 - 184	962.4628	1922.9111	1922.9084	1	0	K.YSHVDLVVMVDGFEGEK.G	(Ions score 104)
168 - 184	962.4628	1922.9111	1922.9084	1	0	K.YSHVDLVVMVDGFEGEK.G	(Ions score 83)
168 - 184	641.9776	1922.9111	1922.9084	1	0	K.YSHVDLVVMVDGFEGEK.G	(Ions score 73)
168 - 184	962.4628	1922.9111	1922.9084	1	0	K.YSHVDLVVMVDGFEGEK.G	(Ions score 72)
168 - 184	962.4628	1922.9111	1922.9084	1	0	K.YSHVDLVVMVDGFEGEK.G	(Ions score 88)
168 - 184	641.9777	1922.9111	1922.9084	1	0	K.YSHVDLVVMVDGFEGEK.G	(Ions score 76)

168 - 184	641.9777	1922.9111	1922.9084	1	0	K.YSHVDLVVMVDGFEGEK.G	(Ions score 73)
168 - 184	641.9777	1922.9111	1922.9084	1	0	K.YSHVDLVVMVDGFEGEK.G	(Ions score 49)
168 - 184	641.9777	1922.9111	1922.9084	1	0	K.YSHVDLVVMVDGFEGEK.G	(Ions score 57)
168 - 184	962.4628	1922.9111	1922.9084	1	0	K.YSHVDLVVMVDGFEGEK.G	(Ions score 91)
168 - 184	641.9777	1922.9111	1922.9084	1	0	K.YSHVDLVVMVDGFEGEK.G	(Ions score 79)
168 - 184	962.4629	1922.9112	1922.9084	1	0	K.YSHVDLVVMVDGFEGEK.G	(Ions score 75)
168 - 184	641.9777	1922.9112	1922.9084	1	0	K.YSHVDLVVMVDGFEGEK.G	(Ions score 76)
168 - 184	962.4629	1922.9112	1922.9084	1	0	K.YSHVDLVVMVDGFEGEK.G	(Ions score 85)
168 - 184	641.9777	1922.9112	1922.9084	1	0	K.YSHVDLVVMVDGFEGEK.G	(Ions score 73)
168 - 184	641.9777	1922.9112	1922.9084	1	0	K.YSHVDLVVMVDGFEGEK.G	(Ions score 55)
168 - 184	962.4629	1922.9112	1922.9084	1	0	K.YSHVDLVVMVDGFEGEK.G	(Ions score 74)
168 - 184	641.9777	1922.9112	1922.9084	1	0	K.YSHVDLVVMVDGFEGEK.G	(Ions score 70)
168 - 184	962.4629	1922.9112	1922.9084	1	0	K.YSHVDLVVMVDGFEGEK.G	(Ions score 91)
168 - 184	962.4629	1922.9112	1922.9084	1	0	K.YSHVDLVVMVDGFEGEK.G	(Ions score 79)
168 - 184	641.9777	1922.9113	1922.9084	1	0	K.YSHVDLVVMVDGFEGEK.G	(Ions score 76)
168 - 184	962.4629	1922.9113	1922.9084	1	0	K.YSHVDLVVMVDGFEGEK.G	(Ions score 102)
168 - 184	641.9777	1922.9113	1922.9084	1	0	K.YSHVDLVVMVDGFEGEK.G	(Ions score 73)
168 - 184	641.9777	1922.9113	1922.9084	2	0	K.YSHVDLVVMVDGFEGEK.G	(Ions score 73)
168 - 184	641.9777	1922.9114	1922.9084	2	0	K.YSHVDLVVMVDGFEGEK.G	(Ions score 73)
168 - 184	641.9777	1922.9114	1922.9084	2	0	K.YSHVDLVVMVDGFEGEK.G	(Ions score 74)
168 - 184	641.9777	1922.9114	1922.9084	2	0	K.YSHVDLVVMVDGFEGEK.G	(Ions score 76)
168 - 184	641.9777	1922.9114	1922.9084	2	0	K.YSHVDLVVMVDGFEGEK.G	(Ions score 73)
168 - 184	641.9777	1922.9114	1922.9084	2	0	K.YSHVDLVVMVDGFEGEK.G	(Ions score 73)
168 - 184	962.4630	1922.9114	1922.9084	2	0	K.YSHVDLVVMVDGFEGEK.G	(Ions score 69)
168 - 184	641.9777	1922.9114	1922.9084	2	0	K.YSHVDLVVMVDGFEGEK.G	(Ions score 57)
168 - 184	641.9778	1922.9114	1922.9084	2	0	K.YSHVDLVVMVDGFEGEK.G	(Ions score 66)
168 - 184	641.9778	1922.9114	1922.9084	2	0	K.YSHVDLVVMVDGFEGEK.G	(Ions score 77)
168 - 184	962.4630	1922.9115	1922.9084	2	0	K.YSHVDLVVMVDGFEGEK.G	(Ions score 71)
168 - 184	641.9778	1922.9115	1922.9084	2	0	K.YSHVDLVVMVDGFEGEK.G	(Ions score 69)
168 - 184	962.4630	1922.9115	1922.9084	2	0	K.YSHVDLVVMVDGFEGEK.G	(Ions score 108)
168 - 184	641.9778	1922.9115	1922.9084	2	0	K.YSHVDLVVMVDGFEGEK.G	(Ions score 76)
168 - 184	641.9778	1922.9115	1922.9084	2	0	K.YSHVDLVVMVDGFEGEK.G	(Ions score 53)
168 - 184	641.9778	1922.9115	1922.9084	2	0	K.YSHVDLVVMVDGFEGEK.G	(Ions score 73)
168 - 184	962.4631	1922.9116	1922.9084	2	0	K.YSHVDLVVMVDGFEGEK.G	(Ions score 76)
168 - 184	962.4631	1922.9116	1922.9084	2	0	K.YSHVDLVVMVDGFEGEK.G	(Ions score 102)
168 - 184	962.4631	1922.9116	1922.9084	2	0	K.YSHVDLVVMVDGFEGEK.G	(Ions score 102)
168 - 184	641.9778	1922.9116	1922.9084	2	0	K.YSHVDLVVMVDGFEGEK.G	(Ions score 57)
168 - 184	962.4631	1922.9116	1922.9084	2	0	K.YSHVDLVVMVDGFEGEK.G	(Ions score 74)
168 - 184	641.9778	1922.9116	1922.9084	2	0	K.YSHVDLVVMVDGFEGEK.G	(Ions score 48)
168 - 184	962.4631	1922.9116	1922.9084	2	0	K.YSHVDLVVMVDGFEGEK.G	(Ions score 62)
168 - 184	962.4631	1922.9117	1922.9084	2	0	K.YSHVDLVVMVDGFEGEK.G	(Ions score 62)
168 - 184	962.4631	1922.9117	1922.9084	2	0	K.YSHVDLVVMVDGFEGEK.G	(Ions score 100)
168 - 184	641.9778	1922.9117	1922.9084	2	0	K.YSHVDLVVMVDGFEGEK.G	(Ions score 73)
168 - 184	641.9778	1922.9117	1922.9084	2	0	K.YSHVDLVVMVDGFEGEK.G	(Ions score 77)
168 - 184	641.9778	1922.9117	1922.9084	2	0	K.YSHVDLVVMVDGFEGEK.G	(Ions score 73)
168 - 184	962.4631	1922.9117	1922.9084	2	0	K.YSHVDLVVMVDGFEGEK.G	(Ions score 92)
168 - 184	641.9779	1922.9117	1922.9084	2	0	K.YSHVDLVVMVDGFEGEK.G	(Ions score 76)
168 - 184	641.9779	1922.9117	1922.9084	2	0	K.YSHVDLVVMVDGFEGEK.G	(Ions score 73)
168 - 184	962.4631	1922.9117	1922.9084	2	0	K.YSHVDLVVMVDGFEGEK.G	(Ions score 105)
168 - 184	962.4632	1922.9117	1922.9084	2	0	K.YSHVDLVVMVDGFEGEK.G	(Ions score 76)
168 - 184	962.4632	1922.9118	1922.9084	2	0	K.YSHVDLVVMVDGFEGEK.G	(Ions score 74)
168 - 184	641.9779	1922.9118	1922.9084	2	0	K.YSHVDLVVMVDGFEGEK.G	(Ions score 70)
168 - 184	641.9779	1922.9118	1922.9084	2	0	K.YSHVDLVVMVDGFEGEK.G	(Ions score 73)
168 - 184	962.4632	1922.9118	1922.9084	2	0	K.YSHVDLVVMVDGFEGEK.G	(Ions score 95)
168 - 184	641.9779	1922.9118	1922.9084	2	0	K.YSHVDLVVMVDGFEGEK.G	(Ions score 73)
168 - 184	962.4632	1922.9118	1922.9084	2	0	K.YSHVDLVVMVDGFEGEK.G	(Ions score 97)
168 - 184	962.4632	1922.9119	1922.9084	2	0	K.YSHVDLVVMVDGFEGEK.G	(Ions score 78)
168 - 184	962.4632	1922.9119	1922.9084	2	0	K.YSHVDLVVMVDGFEGEK.G	(Ions score 82)

168 - 184	641.9779	1922.9119	1922.9084	2	0	K.YSHVDLVVMVDGFEGEK.G	(Ions score 57)
168 - 184	641.9779	1922.9119	1922.9084	2	0	K.YSHVDLVVMVDGFEGEK.G	(Ions score 76)
168 - 184	962.4632	1922.9119	1922.9084	2	0	K.YSHVDLVVMVDGFEGEK.G	(Ions score 79)
168 - 184	641.9779	1922.9119	1922.9084	2	0	K.YSHVDLVVMVDGFEGEK.G	(Ions score 73)
168 - 184	641.9779	1922.9119	1922.9084	2	0	K.YSHVDLVVMVDGFEGEK.G	(Ions score 68)
168 - 184	641.9779	1922.9119	1922.9084	2	0	K.YSHVDLVVMVDGFEGEK.G	(Ions score 70)
168 - 184	641.9779	1922.9119	1922.9084	2	0	K.YSHVDLVVMVDGFEGEK.G	(Ions score 73)
168 - 184	641.9779	1922.9119	1922.9084	2	0	K.YSHVDLVVMVDGFEGEK.G	(Ions score 68)
168 - 184	641.9779	1922.9119	1922.9084	2	0	K.YSHVDLVVMVDGFEGEK.G	(Ions score 65)
168 - 184	641.9779	1922.9119	1922.9084	2	0	K.YSHVDLVVMVDGFEGEK.G	(Ions score 76)
168 - 184	641.9779	1922.9120	1922.9084	2	0	K.YSHVDLVVMVDGFEGEK.G	(Ions score 76)
168 - 184	641.9779	1922.9120	1922.9084	2	0	K.YSHVDLVVMVDGFEGEK.G	(Ions score 66)
168 - 184	641.9779	1922.9120	1922.9084	2	0	K.YSHVDLVVMVDGFEGEK.G	(Ions score 70)
168 - 184	962.4633	1922.9120	1922.9084	2	0	K.YSHVDLVVMVDGFEGEK.G	(Ions score 71)
168 - 184	641.9779	1922.9120	1922.9084	2	0	K.YSHVDLVVMVDGFEGEK.G	(Ions score 68)
168 - 184	641.9779	1922.9120	1922.9084	2	0	K.YSHVDLVVMVDGFEGEK.G	(Ions score 68)
168 - 184	641.9780	1922.9120	1922.9084	2	0	K.YSHVDLVVMVDGFEGEK.G	(Ions score 62)
168 - 184	641.9780	1922.9120	1922.9084	2	0	K.YSHVDLVVMVDGFEGEK.G	(Ions score 64)
168 - 184	962.4633	1922.9120	1922.9084	2	0	K.YSHVDLVVMVDGFEGEK.G	(Ions score 82)
168 - 184	641.9780	1922.9121	1922.9084	2	0	K.YSHVDLVVMVDGFEGEK.G	(Ions score 69)
168 - 184	641.9780	1922.9121	1922.9084	2	0	K.YSHVDLVVMVDGFEGEK.G	(Ions score 57)
168 - 184	962.4633	1922.9121	1922.9084	2	0	K.YSHVDLVVMVDGFEGEK.G	(Ions score 76)
168 - 184	641.9780	1922.9121	1922.9084	2	0	K.YSHVDLVVMVDGFEGEK.G	(Ions score 65)
168 - 184	641.9780	1922.9121	1922.9084	2	0	K.YSHVDLVVMVDGFEGEK.G	(Ions score 66)
168 - 184	962.4633	1922.9121	1922.9084	2	0	K.YSHVDLVVMVDGFEGEK.G	(Ions score 84)
168 - 184	641.9780	1922.9121	1922.9084	2	0	K.YSHVDLVVMVDGFEGEK.G	(Ions score 73)
168 - 184	641.9780	1922.9121	1922.9084	2	0	K.YSHVDLVVMVDGFEGEK.G	(Ions score 76)
168 - 184	641.9780	1922.9121	1922.9084	2	0	K.YSHVDLVVMVDGFEGEK.G	(Ions score 76)
168 - 184	962.4634	1922.9122	1922.9084	2	0	K.YSHVDLVVMVDGFEGEK.G	(Ions score 61)
168 - 184	962.4634	1922.9122	1922.9084	2	0	K.YSHVDLVVMVDGFEGEK.G	(Ions score 92)
168 - 184	962.4634	1922.9122	1922.9084	2	0	K.YSHVDLVVMVDGFEGEK.G	(Ions score 92)
168 - 184	641.9780	1922.9122	1922.9084	2	0	K.YSHVDLVVMVDGFEGEK.G	(Ions score 76)
168 - 184	641.9780	1922.9122	1922.9084	2	0	K.YSHVDLVVMVDGFEGEK.G	(Ions score 69)
168 - 184	962.4634	1922.9122	1922.9084	2	0	K.YSHVDLVVMVDGFEGEK.G	(Ions score 83)
168 - 184	641.9780	1922.9123	1922.9084	2	0	K.YSHVDLVVMVDGFEGEK.G	(Ions score 30)
168 - 184	641.9780	1922.9123	1922.9084	2	0	K.YSHVDLVVMVDGFEGEK.G	(Ions score 68)
168 - 184	641.9780	1922.9123	1922.9084	2	0	K.YSHVDLVVMVDGFEGEK.G	(Ions score 66)
168 - 184	962.4634	1922.9123	1922.9084	2	0	K.YSHVDLVVMVDGFEGEK.G	(Ions score 90)
168 - 184	641.9780	1922.9123	1922.9084	2	0	K.YSHVDLVVMVDGFEGEK.G	(Ions score 68)
168 - 184	962.4634	1922.9123	1922.9084	2	0	K.YSHVDLVVMVDGFEGEK.G	(Ions score 80)
168 - 184	962.4634	1922.9123	1922.9084	2	0	K.YSHVDLVVMVDGFEGEK.G	(Ions score 64)
168 - 184	641.9781	1922.9124	1922.9084	2	0	K.YSHVDLVVMVDGFEGEK.G	(Ions score 76)
168 - 184	962.4635	1922.9124	1922.9084	2	0	K.YSHVDLVVMVDGFEGEK.G	(Ions score 92)
168 - 184	641.9781	1922.9124	1922.9084	2	0	K.YSHVDLVVMVDGFEGEK.G	(Ions score 76)
168 - 184	641.9781	1922.9124	1922.9084	2	0	K.YSHVDLVVMVDGFEGEK.G	(Ions score 73)
168 - 184	962.4635	1922.9124	1922.9084	2	0	K.YSHVDLVVMVDGFEGEK.G	(Ions score 80)
168 - 184	641.9781	1922.9124	1922.9084	2	0	K.YSHVDLVVMVDGFEGEK.G	(Ions score 29)
168 - 184	641.9781	1922.9125	1922.9084	2	0	K.YSHVDLVVMVDGFEGEK.G	(Ions score 70)
168 - 184	641.9781	1922.9125	1922.9084	2	0	K.YSHVDLVVMVDGFEGEK.G	(Ions score 69)
168 - 184	641.9781	1922.9125	1922.9084	2	0	K.YSHVDLVVMVDGFEGEK.G	(Ions score 76)
168 - 184	641.9781	1922.9125	1922.9084	2	0	K.YSHVDLVVMVDGFEGEK.G	(Ions score 34)
168 - 184	641.9781	1922.9125	1922.9084	2	0	K.YSHVDLVVMVDGFEGEK.G	(Ions score 70)
168 - 184	641.9781	1922.9125	1922.9084	2	0	K.YSHVDLVVMVDGFEGEK.G	(Ions score 73)
168 - 184	962.4636	1922.9125	1922.9084	2	0	K.YSHVDLVVMVDGFEGEK.G	(Ions score 92)
168 - 184	641.9781	1922.9126	1922.9084	2	0	K.YSHVDLVVMVDGFEGEK.G	(Ions score 55)
168 - 184	962.4636	1922.9126	1922.9084	2	0	K.YSHVDLVVMVDGFEGEK.G	(Ions score 76)
168 - 184	641.9781	1922.9126	1922.9084	2	0	K.YSHVDLVVMVDGFEGEK.G	(Ions score 76)
168 - 184	641.9781	1922.9126	1922.9084	2	0	K.YSHVDLVVMVDGFEGEK.G	(Ions score 73)

168 - 184	641.9781	1922.9126	1922.9084	2	0	K.YSHVDLVVMVDGFEGEK.G	(Ions score 73)
168 - 184	641.9781	1922.9126	1922.9084	2	0	K.YSHVDLVVMVDGFEGEK.G	(Ions score 76)
168 - 184	962.4636	1922.9126	1922.9084	2	0	K.YSHVDLVVMVDGFEGEK.G	(Ions score 97)
168 - 184	641.9782	1922.9126	1922.9084	2	0	K.YSHVDLVVMVDGFEGEK.G	(Ions score 76)
168 - 184	641.9782	1922.9126	1922.9084	2	0	K.YSHVDLVVMVDGFEGEK.G	(Ions score 77)
168 - 184	962.4636	1922.9126	1922.9084	2	0	K.YSHVDLVVMVDGFEGEK.G	(Ions score 97)
168 - 184	962.4636	1922.9127	1922.9084	2	0	K.YSHVDLVVMVDGFEGEK.G	(Ions score 85)
168 - 184	641.9782	1922.9127	1922.9084	2	0	K.YSHVDLVVMVDGFEGEK.G	(Ions score 73)
168 - 184	641.9782	1922.9127	1922.9084	2	0	K.YSHVDLVVMVDGFEGEK.G	(Ions score 65)
168 - 184	641.9782	1922.9127	1922.9084	2	0	K.YSHVDLVVMVDGFEGEK.G	(Ions score 70)
168 - 184	962.4636	1922.9127	1922.9084	2	0	K.YSHVDLVVMVDGFEGEK.G	(Ions score 72)
168 - 184	641.9782	1922.9127	1922.9084	2	0	K.YSHVDLVVMVDGFEGEK.G	(Ions score 76)
168 - 184	962.4637	1922.9127	1922.9084	2	0	K.YSHVDLVVMVDGFEGEK.G	(Ions score 75)
168 - 184	962.4636	1922.9127	1922.9084	2	0	K.YSHVDLVVMVDGFEGEK.G	(Ions score 63)
168 - 184	641.9782	1922.9128	1922.9084	2	0	K.YSHVDLVVMVDGFEGEK.G	(Ions score 70)
168 - 184	962.4637	1922.9128	1922.9084	2	0	K.YSHVDLVVMVDGFEGEK.G	(Ions score 76)
168 - 184	641.9782	1922.9128	1922.9084	2	0	K.YSHVDLVVMVDGFEGEK.G	(Ions score 64)
168 - 184	641.9782	1922.9129	1922.9084	2	0	K.YSHVDLVVMVDGFEGEK.G	(Ions score 73)
168 - 184	641.9782	1922.9129	1922.9084	2	0	K.YSHVDLVVMVDGFEGEK.G	(Ions score 73)
168 - 184	641.9782	1922.9129	1922.9084	2	0	K.YSHVDLVVMVDGFEGEK.G	(Ions score 76)
168 - 184	641.9782	1922.9129	1922.9084	2	0	K.YSHVDLVVMVDGFEGEK.G	(Ions score 76)
168 - 184	962.4637	1922.9129	1922.9084	2	0	K.YSHVDLVVMVDGFEGEK.G	(Ions score 76)
168 - 184	962.4637	1922.9129	1922.9084	2	0	K.YSHVDLVVMVDGFEGEK.G	(Ions score 79)
168 - 184	641.9782	1922.9129	1922.9084	2	0	K.YSHVDLVVMVDGFEGEK.G	(Ions score 42)
168 - 184	962.4637	1922.9129	1922.9084	2	0	K.YSHVDLVVMVDGFEGEK.G	(Ions score 72)
168 - 184	641.9783	1922.9129	1922.9084	2	0	K.YSHVDLVVMVDGFEGEK.G	(Ions score 73)
168 - 184	641.9783	1922.9129	1922.9084	2	0	K.YSHVDLVVMVDGFEGEK.G	(Ions score 60)
168 - 184	962.4638	1922.9129	1922.9084	2	0	K.YSHVDLVVMVDGFEGEK.G	(Ions score 90)
168 - 184	641.9783	1922.9130	1922.9084	2	0	K.YSHVDLVVMVDGFEGEK.G	(Ions score 76)
168 - 184	962.4638	1922.9130	1922.9084	2	0	K.YSHVDLVVMVDGFEGEK.G	(Ions score 64)
168 - 184	641.9783	1922.9130	1922.9084	2	0	K.YSHVDLVVMVDGFEGEK.G	(Ions score 73)
168 - 184	962.4638	1922.9130	1922.9084	2	0	K.YSHVDLVVMVDGFEGEK.G	(Ions score 102)
168 - 184	641.9783	1922.9130	1922.9084	2	0	K.YSHVDLVVMVDGFEGEK.G	(Ions score 73)
168 - 184	641.9783	1922.9131	1922.9084	2	0	K.YSHVDLVVMVDGFEGEK.G	(Ions score 76)
168 - 184	962.4638	1922.9131	1922.9084	2	0	K.YSHVDLVVMVDGFEGEK.G	(Ions score 84)
168 - 184	641.9783	1922.9131	1922.9084	2	0	K.YSHVDLVVMVDGFEGEK.G	(Ions score 73)
168 - 184	962.4639	1922.9132	1922.9084	2	0	K.YSHVDLVVMVDGFEGEK.G	(Ions score 79)
168 - 184	641.9783	1922.9132	1922.9084	2	0	K.YSHVDLVVMVDGFEGEK.G	(Ions score 76)
168 - 184	641.9784	1922.9132	1922.9084	3	0	K.YSHVDLVVMVDGFEGEK.G	(Ions score 76)
168 - 184	962.4639	1922.9132	1922.9084	3	0	K.YSHVDLVVMVDGFEGEK.G	(Ions score 90)
168 - 184	641.9784	1922.9133	1922.9084	3	0	K.YSHVDLVVMVDGFEGEK.G	(Ions score 73)
168 - 184	962.4639	1922.9133	1922.9084	3	0	K.YSHVDLVVMVDGFEGEK.G	(Ions score 88)
168 - 184	641.9784	1922.9133	1922.9084	3	0	K.YSHVDLVVMVDGFEGEK.G	(Ions score 76)
168 - 184	641.9784	1922.9133	1922.9084	3	0	K.YSHVDLVVMVDGFEGEK.G	(Ions score 70)
168 - 184	962.4639	1922.9133	1922.9084	3	0	K.YSHVDLVVMVDGFEGEK.G	(Ions score 72)
168 - 184	641.9784	1922.9133	1922.9084	3	0	K.YSHVDLVVMVDGFEGEK.G	(Ions score 72)
168 - 184	962.4640	1922.9134	1922.9084	3	0	K.YSHVDLVVMVDGFEGEK.G	(Ions score 90)
168 - 184	962.4640	1922.9134	1922.9084	3	0	K.YSHVDLVVMVDGFEGEK.G	(Ions score 97)
168 - 184	641.9784	1922.9134	1922.9084	3	0	K.YSHVDLVVMVDGFEGEK.G	(Ions score 73)
168 - 184	641.9784	1922.9134	1922.9084	3	0	K.YSHVDLVVMVDGFEGEK.G	(Ions score 67)
168 - 184	641.9784	1922.9134	1922.9084	3	0	K.YSHVDLVVMVDGFEGEK.G	(Ions score 56)
168 - 184	962.4640	1922.9134	1922.9084	3	0	K.YSHVDLVVMVDGFEGEK.G	(Ions score 76)
168 - 184	641.9784	1922.9134	1922.9084	3	0	K.YSHVDLVVMVDGFEGEK.G	(Ions score 73)
168 - 184	962.4640	1922.9135	1922.9084	3	0	K.YSHVDLVVMVDGFEGEK.G	(Ions score 85)
168 - 184	641.9785	1922.9135	1922.9084	3	0	K.YSHVDLVVMVDGFEGEK.G	(Ions score 69)
168 - 184	641.9785	1922.9135	1922.9084	3	0	K.YSHVDLVVMVDGFEGEK.G	(Ions score 69)
168 - 184	641.9785	1922.9136	1922.9084	3	0	K.YSHVDLVVMVDGFEGEK.G	(Ions score 62)
168 - 184	641.9785	1922.9136	1922.9084	3	0	K.YSHVDLVVMVDGFEGEK.G	(Ions score 76)

168 - 184	962.4641	1922.9136	1922.9084	3	0	K.YSHVDLVVMVDGFEGEK.G	(Ions score 75)
168 - 184	641.9785	1922.9136	1922.9084	3	0	K.YSHVDLVVMVDGFEGEK.G	(Ions score 73)
168 - 184	962.4641	1922.9136	1922.9084	3	0	K.YSHVDLVVMVDGFEGEK.G	(Ions score 76)
168 - 184	641.9785	1922.9136	1922.9084	3	0	K.YSHVDLVVMVDGFEGEK.G	(Ions score 76)
168 - 184	641.9785	1922.9136	1922.9084	3	0	K.YSHVDLVVMVDGFEGEK.G	(Ions score 68)
168 - 184	641.9785	1922.9136	1922.9084	3	0	K.YSHVDLVVMVDGFEGEK.G	(Ions score 65)
168 - 184	962.4641	1922.9136	1922.9084	3	0	K.YSHVDLVVMVDGFEGEK.G	(Ions score 94)
168 - 184	641.9785	1922.9136	1922.9084	3	0	K.YSHVDLVVMVDGFEGEK.G	(Ions score 76)
168 - 184	641.9785	1922.9137	1922.9084	3	0	K.YSHVDLVVMVDGFEGEK.G	(Ions score 73)
168 - 184	641.9785	1922.9137	1922.9084	3	0	K.YSHVDLVVMVDGFEGEK.G	(Ions score 65)
168 - 184	962.4641	1922.9137	1922.9084	3	0	K.YSHVDLVVMVDGFEGEK.G	(Ions score 97)
168 - 184	962.4641	1922.9137	1922.9084	3	0	K.YSHVDLVVMVDGFEGEK.G	(Ions score 78)
168 - 184	962.4641	1922.9137	1922.9084	3	0	K.YSHVDLVVMVDGFEGEK.G	(Ions score 62)
168 - 184	962.4642	1922.9138	1922.9084	3	0	K.YSHVDLVVMVDGFEGEK.G	(Ions score 79)
168 - 184	641.9786	1922.9138	1922.9084	3	0	K.YSHVDLVVMVDGFEGEK.G	(Ions score 76)
168 - 184	641.9786	1922.9139	1922.9084	3	0	K.YSHVDLVVMVDGFEGEK.G	(Ions score 73)
168 - 184	962.4642	1922.9139	1922.9084	3	0	K.YSHVDLVVMVDGFEGEK.G	(Ions score 108)
168 - 184	962.4642	1922.9139	1922.9084	3	0	K.YSHVDLVVMVDGFEGEK.G	(Ions score 83)
168 - 184	962.4642	1922.9139	1922.9084	3	0	K.YSHVDLVVMVDGFEGEK.G	(Ions score 76)
168 - 184	641.9786	1922.9139	1922.9084	3	0	K.YSHVDLVVMVDGFEGEK.G	(Ions score 73)
168 - 184	641.9786	1922.9139	1922.9084	3	0	K.YSHVDLVVMVDGFEGEK.G	(Ions score 76)
168 - 184	962.4642	1922.9139	1922.9084	3	0	K.YSHVDLVVMVDGFEGEK.G	(Ions score 56)
168 - 184	962.4642	1922.9139	1922.9084	3	0	K.YSHVDLVVMVDGFEGEK.G	(Ions score 83)
168 - 184	641.9786	1922.9140	1922.9084	3	0	K.YSHVDLVVMVDGFEGEK.G	(Ions score 76)
168 - 184	962.4643	1922.9140	1922.9084	3	0	K.YSHVDLVVMVDGFEGEK.G	(Ions score 76)
168 - 184	962.4643	1922.9140	1922.9084	3	0	K.YSHVDLVVMVDGFEGEK.G	(Ions score 84)
168 - 184	641.9786	1922.9140	1922.9084	3	0	K.YSHVDLVVMVDGFEGEK.G	(Ions score 76)
168 - 184	962.4643	1922.9141	1922.9084	3	0	K.YSHVDLVVMVDGFEGEK.G	(Ions score 79)
168 - 184	962.4644	1922.9142	1922.9084	3	0	K.YSHVDLVVMVDGFEGEK.G	(Ions score 75)
168 - 184	641.9787	1922.9142	1922.9084	3	0	K.YSHVDLVVMVDGFEGEK.G	(Ions score 76)
168 - 184	962.4644	1922.9142	1922.9084	3	0	K.YSHVDLVVMVDGFEGEK.G	(Ions score 98)
168 - 184	962.4644	1922.9143	1922.9084	3	0	K.YSHVDLVVMVDGFEGEK.G	(Ions score 97)
168 - 184	962.4644	1922.9143	1922.9084	3	0	K.YSHVDLVVMVDGFEGEK.G	(Ions score 108)
168 - 184	641.9787	1922.9143	1922.9084	3	0	K.YSHVDLVVMVDGFEGEK.G	(Ions score 76)
168 - 184	641.9788	1922.9144	1922.9084	3	0	K.YSHVDLVVMVDGFEGEK.G	(Ions score 70)
168 - 184	962.4645	1922.9144	1922.9084	3	0	K.YSHVDLVVMVDGFEGEK.G	(Ions score 90)
168 - 184	641.9788	1922.9144	1922.9084	3	0	K.YSHVDLVVMVDGFEGEK.G	(Ions score 73)
168 - 184	962.4645	1922.9144	1922.9084	3	0	K.YSHVDLVVMVDGFEGEK.G	(Ions score 97)
168 - 184	962.4645	1922.9145	1922.9084	3	0	K.YSHVDLVVMVDGFEGEK.G	(Ions score 72)
168 - 184	641.9788	1922.9145	1922.9084	3	0	K.YSHVDLVVMVDGFEGEK.G	(Ions score 73)
168 - 184	962.4646	1922.9145	1922.9084	3	0	K.YSHVDLVVMVDGFEGEK.G	(Ions score 98)
168 - 184	641.9788	1922.9146	1922.9084	3	0	K.YSHVDLVVMVDGFEGEK.G	(Ions score 76)
168 - 184	641.9788	1922.9146	1922.9084	3	0	K.YSHVDLVVMVDGFEGEK.G	(Ions score 55)
168 - 184	641.9788	1922.9146	1922.9084	3	0	K.YSHVDLVVMVDGFEGEK.G	(Ions score 73)
168 - 184	641.9788	1922.9146	1922.9084	3	0	K.YSHVDLVVMVDGFEGEK.G	(Ions score 36)
168 - 184	962.4646	1922.9146	1922.9084	3	0	K.YSHVDLVVMVDGFEGEK.G	(Ions score 69)
168 - 184	962.4646	1922.9146	1922.9084	3	0	K.YSHVDLVVMVDGFEGEK.G	(Ions score 77)
168 - 184	962.4646	1922.9146	1922.9084	3	0	K.YSHVDLVVMVDGFEGEK.G	(Ions score 90)
168 - 184	641.9788	1922.9146	1922.9084	3	0	K.YSHVDLVVMVDGFEGEK.G	(Ions score 80)
168 - 184	962.4646	1922.9146	1922.9084	3	0	K.YSHVDLVVMVDGFEGEK.G	(Ions score 100)
168 - 184	962.4646	1922.9147	1922.9084	3	0	K.YSHVDLVVMVDGFEGEK.G	(Ions score 78)
168 - 184	641.9788	1922.9147	1922.9084	3	0	K.YSHVDLVVMVDGFEGEK.G	(Ions score 75)
168 - 184	641.9788	1922.9147	1922.9084	3	0	K.YSHVDLVVMVDGFEGEK.G	(Ions score 76)
168 - 184	641.9789	1922.9147	1922.9084	3	0	K.YSHVDLVVMVDGFEGEK.G	(Ions score 44)
168 - 184	962.4647	1922.9148	1922.9084	3	0	K.YSHVDLVVMVDGFEGEK.G	(Ions score 74)
168 - 184	641.9789	1922.9148	1922.9084	3	0	K.YSHVDLVVMVDGFEGEK.G	(Ions score 68)
168 - 184	641.9789	1922.9148	1922.9084	3	0	K.YSHVDLVVMVDGFEGEK.G	(Ions score 76)
168 - 184	641.9789	1922.9148	1922.9084	3	0	K.YSHVDLVVMVDGFEGEK.G	(Ions score 70)

168 - 184	962.4647	1922.9149	1922.9084	3	0	K.YSHVDLVVMVDGFEGEK.G	(Ions score 73)
168 - 184	641.9789	1922.9149	1922.9084	3	0	K.YSHVDLVVMVDGFEGEK.G	(Ions score 55)
168 - 184	962.4647	1922.9149	1922.9084	3	0	K.YSHVDLVVMVDGFEGEK.G	(Ions score 87)
168 - 184	962.4647	1922.9149	1922.9084	3	0	K.YSHVDLVVMVDGFEGEK.G	(Ions score 84)
168 - 184	962.4648	1922.9150	1922.9084	3	0	K.YSHVDLVVMVDGFEGEK.G	(Ions score 75)
168 - 184	641.9790	1922.9150	1922.9084	3	0	K.YSHVDLVVMVDGFEGEK.G	(Ions score 76)
168 - 184	962.4648	1922.9150	1922.9084	3	0	K.YSHVDLVVMVDGFEGEK.G	(Ions score 72)
168 - 184	962.4648	1922.9151	1922.9084	3	0	K.YSHVDLVVMVDGFEGEK.G	(Ions score 78)
168 - 184	962.4648	1922.9151	1922.9084	3	0	K.YSHVDLVVMVDGFEGEK.G	(Ions score 110)
168 - 184	962.4648	1922.9151	1922.9084	3	0	K.YSHVDLVVMVDGFEGEK.G	(Ions score 77)
168 - 184	641.9790	1922.9151	1922.9084	3	0	K.YSHVDLVVMVDGFEGEK.G	(Ions score 73)
168 - 184	962.4648	1922.9151	1922.9084	3	0	K.YSHVDLVVMVDGFEGEK.G	(Ions score 92)
168 - 184	641.9790	1922.9151	1922.9084	3	0	K.YSHVDLVVMVDGFEGEK.G	(Ions score 76)
168 - 184	641.9790	1922.9152	1922.9084	3	0	K.YSHVDLVVMVDGFEGEK.G	(Ions score 76)
168 - 184	641.9790	1922.9152	1922.9084	4	0	K.YSHVDLVVMVDGFEGEK.G	(Ions score 73)
168 - 184	962.4649	1922.9152	1922.9084	4	0	K.YSHVDLVVMVDGFEGEK.G	(Ions score 68)
168 - 184	962.4649	1922.9152	1922.9084	4	0	K.YSHVDLVVMVDGFEGEK.G	(Ions score 84)
168 - 184	962.4649	1922.9153	1922.9084	4	0	K.YSHVDLVVMVDGFEGEK.G	(Ions score 83)
168 - 184	962.4649	1922.9153	1922.9084	4	0	K.YSHVDLVVMVDGFEGEK.G	(Ions score 92)
168 - 184	641.9791	1922.9153	1922.9084	4	0	K.YSHVDLVVMVDGFEGEK.G	(Ions score 73)
168 - 184	641.9791	1922.9154	1922.9084	4	0	K.YSHVDLVVMVDGFEGEK.G	(Ions score 73)
168 - 184	641.9791	1922.9154	1922.9084	4	0	K.YSHVDLVVMVDGFEGEK.G	(Ions score 73)
168 - 184	641.9791	1922.9154	1922.9084	4	0	K.YSHVDLVVMVDGFEGEK.G	(Ions score 76)
168 - 184	641.9791	1922.9154	1922.9084	4	0	K.YSHVDLVVMVDGFEGEK.G	(Ions score 67)
168 - 184	962.4650	1922.9154	1922.9084	4	0	K.YSHVDLVVMVDGFEGEK.G	(Ions score 72)
168 - 184	962.4650	1922.9155	1922.9084	4	0	K.YSHVDLVVMVDGFEGEK.G	(Ions score 63)
168 - 184	641.9791	1922.9155	1922.9084	4	0	K.YSHVDLVVMVDGFEGEK.G	(Ions score 76)
168 - 184	962.4650	1922.9155	1922.9084	4	0	K.YSHVDLVVMVDGFEGEK.G	(Ions score 73)
168 - 184	641.9791	1922.9155	1922.9084	4	0	K.YSHVDLVVMVDGFEGEK.G	(Ions score 76)
168 - 184	641.9791	1922.9155	1922.9084	4	0	K.YSHVDLVVMVDGFEGEK.G	(Ions score 26)
168 - 184	641.9791	1922.9156	1922.9084	4	0	K.YSHVDLVVMVDGFEGEK.G	(Ions score 66)
168 - 184	962.4651	1922.9156	1922.9084	4	0	K.YSHVDLVVMVDGFEGEK.G	(Ions score 68)
168 - 184	641.9792	1922.9157	1922.9084	4	0	K.YSHVDLVVMVDGFEGEK.G	(Ions score 73)
168 - 184	962.4651	1922.9157	1922.9084	4	0	K.YSHVDLVVMVDGFEGEK.G	(Ions score 92)
168 - 184	641.9792	1922.9157	1922.9084	4	0	K.YSHVDLVVMVDGFEGEK.G	(Ions score 68)
168 - 184	962.4651	1922.9157	1922.9084	4	0	K.YSHVDLVVMVDGFEGEK.G	(Ions score 92)
168 - 184	641.9792	1922.9158	1922.9084	4	0	K.YSHVDLVVMVDGFEGEK.G	(Ions score 76)
168 - 184	641.9792	1922.9158	1922.9084	4	0	K.YSHVDLVVMVDGFEGEK.G	(Ions score 77)
168 - 184	641.9792	1922.9158	1922.9084	4	0	K.YSHVDLVVMVDGFEGEK.G	(Ions score 76)
168 - 184	641.9792	1922.9158	1922.9084	4	0	K.YSHVDLVVMVDGFEGEK.G	(Ions score 76)
168 - 184	641.9792	1922.9158	1922.9084	4	0	K.YSHVDLVVMVDGFEGEK.G	(Ions score 70)
168 - 184	962.4652	1922.9159	1922.9084	4	0	K.YSHVDLVVMVDGFEGEK.G	(Ions score 36)
168 - 184	641.9792	1922.9159	1922.9084	4	0	K.YSHVDLVVMVDGFEGEK.G	(Ions score 73)
168 - 184	962.4652	1922.9159	1922.9084	4	0	K.YSHVDLVVMVDGFEGEK.G	(Ions score 64)
168 - 184	641.9792	1922.9159	1922.9084	4	0	K.YSHVDLVVMVDGFEGEK.G	(Ions score 76)
168 - 184	962.4652	1922.9159	1922.9084	4	0	K.YSHVDLVVMVDGFEGEK.G	(Ions score 87)
168 - 184	962.4652	1922.9159	1922.9084	4	0	K.YSHVDLVVMVDGFEGEK.G	(Ions score 95)
168 - 184	962.4653	1922.9160	1922.9084	4	0	K.YSHVDLVVMVDGFEGEK.G	(Ions score 88)
168 - 184	962.4653	1922.9160	1922.9084	4	0	K.YSHVDLVVMVDGFEGEK.G	(Ions score 61)
168 - 184	962.4653	1922.9161	1922.9084	4	0	K.YSHVDLVVMVDGFEGEK.G	(Ions score 80)
168 - 184	962.4653	1922.9161	1922.9084	4	0	K.YSHVDLVVMVDGFEGEK.G	(Ions score 80)
168 - 184	641.9793	1922.9161	1922.9084	4	0	K.YSHVDLVVMVDGFEGEK.G	(Ions score 76)
168 - 184	962.4653	1922.9161	1922.9084	4	0	K.YSHVDLVVMVDGFEGEK.G	(Ions score 92)
168 - 184	641.9793	1922.9161	1922.9084	4	0	K.YSHVDLVVMVDGFEGEK.G	(Ions score 76)
168 - 184	641.9793	1922.9161	1922.9084	4	0	K.YSHVDLVVMVDGFEGEK.G	(Ions score 37)
168 - 184	641.9793	1922.9161	1922.9084	4	0	K.YSHVDLVVMVDGFEGEK.G	(Ions score 64)
168 - 184	962.4654	1922.9162	1922.9084	4	0	K.YSHVDLVVMVDGFEGEK.G	(Ions score 107)
168 - 184	641.9793	1922.9162	1922.9084	4	0	K.YSHVDLVVMVDGFEGEK.G	(Ions score 76)

168 - 184	641.9793	1922.9162	1922.9084	4	0	K.YSHVDLVVMVDGFEGEK.G	(Ions score 63)
168 - 184	641.9794	1922.9162	1922.9084	4	0	K.YSHVDLVVMVDGFEGEK.G	(Ions score 76)
168 - 184	962.4654	1922.9162	1922.9084	4	0	K.YSHVDLVVMVDGFEGEK.G	(Ions score 82)
168 - 184	641.9794	1922.9162	1922.9084	4	0	K.YSHVDLVVMVDGFEGEK.G	(Ions score 76)
168 - 184	641.9794	1922.9163	1922.9084	4	0	K.YSHVDLVVMVDGFEGEK.G	(Ions score 73)
168 - 184	641.9794	1922.9163	1922.9084	4	0	K.YSHVDLVVMVDGFEGEK.G	(Ions score 66)
168 - 184	641.9794	1922.9163	1922.9084	4	0	K.YSHVDLVVMVDGFEGEK.G	(Ions score 73)
168 - 184	641.9794	1922.9164	1922.9084	4	0	K.YSHVDLVVMVDGFEGEK.G	(Ions score 89)
168 - 184	641.9795	1922.9165	1922.9084	4	0	K.YSHVDLVVMVDGFEGEK.G	(Ions score 73)
168 - 184	962.4656	1922.9166	1922.9084	4	0	K.YSHVDLVVMVDGFEGEK.G	(Ions score 79)
168 - 184	641.9795	1922.9166	1922.9084	4	0	K.YSHVDLVVMVDGFEGEK.G	(Ions score 73)
168 - 184	641.9795	1922.9166	1922.9084	4	0	K.YSHVDLVVMVDGFEGEK.G	(Ions score 66)
168 - 184	962.4656	1922.9166	1922.9084	4	0	K.YSHVDLVVMVDGFEGEK.G	(Ions score 78)
168 - 184	641.9795	1922.9166	1922.9084	4	0	K.YSHVDLVVMVDGFEGEK.G	(Ions score 73)
168 - 184	641.9795	1922.9166	1922.9084	4	0	K.YSHVDLVVMVDGFEGEK.G	(Ions score 89)
168 - 184	641.9795	1922.9166	1922.9084	4	0	K.YSHVDLVVMVDGFEGEK.G	(Ions score 76)
168 - 184	641.9795	1922.9167	1922.9084	4	0	K.YSHVDLVVMVDGFEGEK.G	(Ions score 70)
168 - 184	641.9795	1922.9167	1922.9084	4	0	K.YSHVDLVVMVDGFEGEK.G	(Ions score 70)
168 - 184	641.9795	1922.9168	1922.9084	4	0	K.YSHVDLVVMVDGFEGEK.G	(Ions score 65)
168 - 184	641.9795	1922.9168	1922.9084	4	0	K.YSHVDLVVMVDGFEGEK.G	(Ions score 80)
168 - 184	641.9795	1922.9168	1922.9084	4	0	K.YSHVDLVVMVDGFEGEK.G	(Ions score 56)
168 - 184	641.9796	1922.9169	1922.9084	4	0	K.YSHVDLVVMVDGFEGEK.G	(Ions score 66)
168 - 184	962.4657	1922.9169	1922.9084	4	0	K.YSHVDLVVMVDGFEGEK.G	(Ions score 84)
168 - 184	641.9796	1922.9169	1922.9084	4	0	K.YSHVDLVVMVDGFEGEK.G	(Ions score 76)
168 - 184	962.4657	1922.9169	1922.9084	4	0	K.YSHVDLVVMVDGFEGEK.G	(Ions score 69)
168 - 184	641.9796	1922.9170	1922.9084	4	0	K.YSHVDLVVMVDGFEGEK.G	(Ions score 62)
168 - 184	641.9796	1922.9170	1922.9084	4	0	K.YSHVDLVVMVDGFEGEK.G	(Ions score 77)
168 - 184	962.4658	1922.9170	1922.9084	4	0	K.YSHVDLVVMVDGFEGEK.G	(Ions score 74)
168 - 184	641.9796	1922.9170	1922.9084	4	0	K.YSHVDLVVMVDGFEGEK.G	(Ions score 23)
168 - 184	641.9796	1922.9170	1922.9084	4	0	K.YSHVDLVVMVDGFEGEK.G	(Ions score 66)
168 - 184	641.9796	1922.9170	1922.9084	4	0	K.YSHVDLVVMVDGFEGEK.G	(Ions score 73)
168 - 184	962.4658	1922.9170	1922.9084	4	0	K.YSHVDLVVMVDGFEGEK.G	(Ions score 107)
168 - 184	641.9796	1922.9171	1922.9084	4	0	K.YSHVDLVVMVDGFEGEK.G	(Ions score 70)
168 - 184	641.9796	1922.9171	1922.9084	4	0	K.YSHVDLVVMVDGFEGEK.G	(Ions score 76)
168 - 184	641.9796	1922.9171	1922.9084	4	0	K.YSHVDLVVMVDGFEGEK.G	(Ions score 62)
168 - 184	641.9796	1922.9171	1922.9084	5	0	K.YSHVDLVVMVDGFEGEK.G	(Ions score 58)
168 - 184	641.9796	1922.9171	1922.9084	5	0	K.YSHVDLVVMVDGFEGEK.G	(Ions score 73)
168 - 184	641.9796	1922.9171	1922.9084	5	0	K.YSHVDLVVMVDGFEGEK.G	(Ions score 76)
168 - 184	962.4659	1922.9172	1922.9084	5	0	K.YSHVDLVVMVDGFEGEK.G	(Ions score 84)
168 - 184	641.9797	1922.9172	1922.9084	5	0	K.YSHVDLVVMVDGFEGEK.G	(Ions score 85)
168 - 184	641.9797	1922.9173	1922.9084	5	0	K.YSHVDLVVMVDGFEGEK.G	(Ions score 80)
168 - 184	641.9797	1922.9173	1922.9084	5	0	K.YSHVDLVVMVDGFEGEK.G	(Ions score 76)
168 - 184	641.9797	1922.9173	1922.9084	5	0	K.YSHVDLVVMVDGFEGEK.G	(Ions score 60)
168 - 184	962.4660	1922.9174	1922.9084	5	0	K.YSHVDLVVMVDGFEGEK.G	(Ions score 98)
168 - 184	641.9797	1922.9174	1922.9084	5	0	K.YSHVDLVVMVDGFEGEK.G	(Ions score 76)
168 - 184	641.9797	1922.9174	1922.9084	5	0	K.YSHVDLVVMVDGFEGEK.G	(Ions score 54)
168 - 184	962.4660	1922.9175	1922.9084	5	0	K.YSHVDLVVMVDGFEGEK.G	(Ions score 98)
168 - 184	962.4660	1922.9175	1922.9084	5	0	K.YSHVDLVVMVDGFEGEK.G	(Ions score 95)
168 - 184	962.4661	1922.9176	1922.9084	5	0	K.YSHVDLVVMVDGFEGEK.G	(Ions score 100)
168 - 184	641.9798	1922.9176	1922.9084	5	0	K.YSHVDLVVMVDGFEGEK.G	(Ions score 56)
168 - 184	641.9798	1922.9176	1922.9084	5	0	K.YSHVDLVVMVDGFEGEK.G	(Ions score 76)
168 - 184	641.9798	1922.9177	1922.9084	5	0	K.YSHVDLVVMVDGFEGEK.G	(Ions score 59)
168 - 184	962.4661	1922.9177	1922.9084	5	0	K.YSHVDLVVMVDGFEGEK.G	(Ions score 79)
168 - 184	962.4662	1922.9178	1922.9084	5	0	K.YSHVDLVVMVDGFEGEK.G	(Ions score 100)
168 - 184	641.9799	1922.9178	1922.9084	5	0	K.YSHVDLVVMVDGFEGEK.G	(Ions score 66)
168 - 184	962.4662	1922.9178	1922.9084	5	0	K.YSHVDLVVMVDGFEGEK.G	(Ions score 73)
168 - 184	641.9799	1922.9178	1922.9084	5	0	K.YSHVDLVVMVDGFEGEK.G	(Ions score 57)
168 - 184	641.9799	1922.9179	1922.9084	5	0	K.YSHVDLVVMVDGFEGEK.G	(Ions score 76)

168 - 184	641.9799	1922.9179	1922.9084	5	0	K.YSHVDLVVMVDGFEGEK.G		(Ions score 73)
168 - 184	641.9799	1922.9179	1922.9084	5	0	K.YSHVDLVVMVDGFEGEK.G		(Ions score 69)
168 - 184	962.4663	1922.9180	1922.9084	5	0	K.YSHVDLVVMVDGFEGEK.G		(Ions score 76)
168 - 184	641.9799	1922.9180	1922.9084	5	0	K.YSHVDLVVMVDGFEGEK.G		(Ions score 76)
168 - 184	970.4571	1938.8996	1938.9034	-2	0	K.YSHVDLVVMVDGFEGEK.G	Oxidation (M)	(Ions score 59)
168 - 184	970.4575	1938.9004	1938.9034	-2	0	K.YSHVDLVVMVDGFEGEK.G	Oxidation (M)	(Ions score 61)
168 - 184	970.4577	1938.9008	1938.9034	-1	0	K.YSHVDLVVMVDGFEGEK.G	Oxidation (M)	(Ions score 53)
168 - 184	970.4578	1938.9011	1938.9034	-1	0	K.YSHVDLVVMVDGFEGEK.G	Oxidation (M)	(Ions score 40)
168 - 184	970.4580	1938.9014	1938.9034	-1	0	K.YSHVDLVVMVDGFEGEK.G	Oxidation (M)	(Ions score 57)
168 - 184	970.4581	1938.9017	1938.9034	-1	0	K.YSHVDLVVMVDGFEGEK.G	Oxidation (M)	(Ions score 39)
168 - 184	970.4581	1938.9017	1938.9034	-1	0	K.YSHVDLVVMVDGFEGEK.G	Oxidation (M)	(Ions score 56)
168 - 184	647.3079	1938.9018	1938.9034	-1	0	K.YSHVDLVVMVDGFEGEK.G	Oxidation (M)	(Ions score 46)
168 - 184	970.4584	1938.9022	1938.9034	-1	0	K.YSHVDLVVMVDGFEGEK.G	Oxidation (M)	(Ions score 53)
168 - 184	647.3080	1938.9023	1938.9034	-1	0	K.YSHVDLVVMVDGFEGEK.G	Oxidation (M)	(Ions score 43)
168 - 184	970.4585	1938.9024	1938.9034	-1	0	K.YSHVDLVVMVDGFEGEK.G	Oxidation (M)	(Ions score 61)
168 - 184	970.4585	1938.9024	1938.9034	-1	0	K.YSHVDLVVMVDGFEGEK.G	Oxidation (M)	(Ions score 41)
168 - 184	647.3081	1938.9024	1938.9034	-1	0	K.YSHVDLVVMVDGFEGEK.G	Oxidation (M)	(Ions score 52)
168 - 184	970.4585	1938.9024	1938.9034	-0	0	K.YSHVDLVVMVDGFEGEK.G	Oxidation (M)	(Ions score 49)
168 - 184	647.3082	1938.9028	1938.9034	-0	0	K.YSHVDLVVMVDGFEGEK.G	Oxidation (M)	(Ions score 72)
168 - 184	970.4587	1938.9029	1938.9034	-0	0	K.YSHVDLVVMVDGFEGEK.G	Oxidation (M)	(Ions score 52)
168 - 184	647.3083	1938.9029	1938.9034	-0	0	K.YSHVDLVVMVDGFEGEK.G	Oxidation (M)	(Ions score 56)
168 - 184	970.4588	1938.9030	1938.9034	-0	0	K.YSHVDLVVMVDGFEGEK.G	Oxidation (M)	(Ions score 59)
168 - 184	647.3083	1938.9030	1938.9034	-0	0	K.YSHVDLVVMVDGFEGEK.G	Oxidation (M)	(Ions score 38)
168 - 184	647.3083	1938.9031	1938.9034	-0	0	K.YSHVDLVVMVDGFEGEK.G	Oxidation (M)	(Ions score 52)
168 - 184	647.3083	1938.9031	1938.9034	-0	0	K.YSHVDLVVMVDGFEGEK.G	Oxidation (M)	(Ions score 66)
168 - 184	970.4588	1938.9031	1938.9034	-0	0	K.YSHVDLVVMVDGFEGEK.G	Oxidation (M)	(Ions score 56)
168 - 184	970.4588	1938.9031	1938.9034	-0	0	K.YSHVDLVVMVDGFEGEK.G	Oxidation (M)	(Ions score 63)
168 - 184	970.4589	1938.9032	1938.9034	-0	0	K.YSHVDLVVMVDGFEGEK.G	Oxidation (M)	(Ions score 61)
168 - 184	970.4589	1938.9033	1938.9034	-0	0	K.YSHVDLVVMVDGFEGEK.G	Oxidation (M)	(Ions score 52)
168 - 184	970.4589	1938.9033	1938.9034	-0	0	K.YSHVDLVVMVDGFEGEK.G	Oxidation (M)	(Ions score 52)
168 - 184	970.4590	1938.9034	1938.9034	0	0	K.YSHVDLVVMVDGFEGEK.G	Oxidation (M)	(Ions score 54)
168 - 184	647.3084	1938.9034	1938.9034	0	0	K.YSHVDLVVMVDGFEGEK.G	Oxidation (M)	(Ions score 41)
168 - 184	970.4590	1938.9035	1938.9034	0	0	K.YSHVDLVVMVDGFEGEK.G	Oxidation (M)	(Ions score 62)
168 - 184	970.4590	1938.9035	1938.9034	0	0	K.YSHVDLVVMVDGFEGEK.G	Oxidation (M)	(Ions score 63)
168 - 184	970.4590	1938.9035	1938.9034	0	0	K.YSHVDLVVMVDGFEGEK.G	Oxidation (M)	(Ions score 72)
168 - 184	970.4591	1938.9036	1938.9034	0	0	K.YSHVDLVVMVDGFEGEK.G	Oxidation (M)	(Ions score 57)
168 - 184	647.3085	1938.9037	1938.9034	0	0	K.YSHVDLVVMVDGFEGEK.G	Oxidation (M)	(Ions score 56)
168 - 184	970.4591	1938.9037	1938.9034	0	0	K.YSHVDLVVMVDGFEGEK.G	Oxidation (M)	(Ions score 58)
168 - 184	970.4591	1938.9037	1938.9034	0	0	K.YSHVDLVVMVDGFEGEK.G	Oxidation (M)	(Ions score 61)
168 - 184	647.3086	1938.9038	1938.9034	0	0	K.YSHVDLVVMVDGFEGEK.G	Oxidation (M)	(Ions score 61)
168 - 184	970.4592	1938.9039	1938.9034	0	0	K.YSHVDLVVMVDGFEGEK.G	Oxidation (M)	(Ions score 40)
168 - 184	970.4592	1938.9039	1938.9034	0	0	K.YSHVDLVVMVDGFEGEK.G	Oxidation (M)	(Ions score 53)
168 - 184	970.4593	1938.9040	1938.9034	0	0	K.YSHVDLVVMVDGFEGEK.G	Oxidation (M)	(Ions score 63)
168 - 184	647.3086	1938.9040	1938.9034	0	0	K.YSHVDLVVMVDGFEGEK.G	Oxidation (M)	(Ions score 53)
168 - 184	647.3086	1938.9041	1938.9034	0	0	K.YSHVDLVVMVDGFEGEK.G	Oxidation (M)	(Ions score 58)
168 - 184	647.3086	1938.9041	1938.9034	0	0	K.YSHVDLVVMVDGFEGEK.G	Oxidation (M)	(Ions score 43)
168 - 184	970.4593	1938.9041	1938.9034	0	0	K.YSHVDLVVMVDGFEGEK.G	Oxidation (M)	(Ions score 49)
168 - 184	647.3087	1938.9042	1938.9034	0	0	K.YSHVDLVVMVDGFEGEK.G	Oxidation (M)	(Ions score 58)
168 - 184	970.4594	1938.9042	1938.9034	0	0	K.YSHVDLVVMVDGFEGEK.G	Oxidation (M)	(Ions score 65)
168 - 184	970.4594	1938.9042	1938.9034	0	0	K.YSHVDLVVMVDGFEGEK.G	Oxidation (M)	(Ions score 57)
168 - 184	970.4594	1938.9042	1938.9034	0	0	K.YSHVDLVVMVDGFEGEK.G	Oxidation (M)	(Ions score 52)
168 - 184	647.3087	1938.9042	1938.9034	0	0	K.YSHVDLVVMVDGFEGEK.G	Oxidation (M)	(Ions score 58)
168 - 184	970.4594	1938.9043	1938.9034	0	0	K.YSHVDLVVMVDGFEGEK.G	Oxidation (M)	(Ions score 58)
168 - 184	970.4594	1938.9043	1938.9034	1	0	K.YSHVDLVVMVDGFEGEK.G	Oxidation (M)	(Ions score 74)
168 - 184	647.3087	1938.9044	1938.9034	1	0	K.YSHVDLVVMVDGFEGEK.G	Oxidation (M)	(Ions score 58)
168 - 184	970.4595	1938.9044	1938.9034	1	0	K.YSHVDLVVMVDGFEGEK.G	Oxidation (M)	(Ions score 52)
168 - 184	970.4595	1938.9044	1938.9034	1	0	K.YSHVDLVVMVDGFEGEK.G	Oxidation (M)	(Ions score 55)
168 - 184	970.4595	1938.9044	1938.9034	1	0	K.YSHVDLVVMVDGFEGEK.G	Oxidation (M)	(Ions score 58)

168 - 184	647.3087	1938.9044	1938.9034	1	0	K.YSHVDLVVMVDGFEGEK.G	Oxidation (M)	(Ions score 58)
168 - 184	970.4595	1938.9044	1938.9034	1	0	K.YSHVDLVVMVDGFEGEK.G	Oxidation (M)	(Ions score 73)
168 - 184	970.4595	1938.9044	1938.9034	1	0	K.YSHVDLVVMVDGFEGEK.G	Oxidation (M)	(Ions score 57)
168 - 184	970.4595	1938.9044	1938.9034	1	0	K.YSHVDLVVMVDGFEGEK.G	Oxidation (M)	(Ions score 63)
168 - 184	647.3088	1938.9044	1938.9034	1	0	K.YSHVDLVVMVDGFEGEK.G	Oxidation (M)	(Ions score 58)
168 - 184	970.4595	1938.9044	1938.9034	1	0	K.YSHVDLVVMVDGFEGEK.G	Oxidation (M)	(Ions score 59)
168 - 184	970.4595	1938.9045	1938.9034	1	0	K.YSHVDLVVMVDGFEGEK.G	Oxidation (M)	(Ions score 68)
168 - 184	647.3088	1938.9045	1938.9034	1	0	K.YSHVDLVVMVDGFEGEK.G	Oxidation (M)	(Ions score 69)
168 - 184	647.3088	1938.9045	1938.9034	1	0	K.YSHVDLVVMVDGFEGEK.G	Oxidation (M)	(Ions score 58)
168 - 184	970.4595	1938.9045	1938.9034	1	0	K.YSHVDLVVMVDGFEGEK.G	Oxidation (M)	(Ions score 67)
168 - 184	647.3088	1938.9045	1938.9034	1	0	K.YSHVDLVVMVDGFEGEK.G	Oxidation (M)	(Ions score 65)
168 - 184	647.3088	1938.9045	1938.9034	1	0	K.YSHVDLVVMVDGFEGEK.G	Oxidation (M)	(Ions score 46)
168 - 184	970.4595	1938.9045	1938.9034	1	0	K.YSHVDLVVMVDGFEGEK.G	Oxidation (M)	(Ions score 52)
168 - 184	647.3088	1938.9045	1938.9034	1	0	K.YSHVDLVVMVDGFEGEK.G	Oxidation (M)	(Ions score 47)
168 - 184	647.3088	1938.9046	1938.9034	1	0	K.YSHVDLVVMVDGFEGEK.G	Oxidation (M)	(Ions score 51)
168 - 184	647.3088	1938.9046	1938.9034	1	0	K.YSHVDLVVMVDGFEGEK.G	Oxidation (M)	(Ions score 42)
168 - 184	647.3088	1938.9046	1938.9034	1	0	K.YSHVDLVVMVDGFEGEK.G	Oxidation (M)	(Ions score 50)
168 - 184	647.3088	1938.9046	1938.9034	1	0	K.YSHVDLVVMVDGFEGEK.G	Oxidation (M)	(Ions score 69)
168 - 184	647.3088	1938.9047	1938.9034	1	0	K.YSHVDLVVMVDGFEGEK.G	Oxidation (M)	(Ions score 69)
168 - 184	647.3088	1938.9047	1938.9034	1	0	K.YSHVDLVVMVDGFEGEK.G	Oxidation (M)	(Ions score 69)
168 - 184	647.3088	1938.9047	1938.9034	1	0	K.YSHVDLVVMVDGFEGEK.G	Oxidation (M)	(Ions score 50)
168 - 184	970.4596	1938.9047	1938.9034	1	0	K.YSHVDLVVMVDGFEGEK.G	Oxidation (M)	(Ions score 72)
168 - 184	647.3089	1938.9047	1938.9034	1	0	K.YSHVDLVVMVDGFEGEK.G	Oxidation (M)	(Ions score 64)
168 - 184	647.3089	1938.9047	1938.9034	1	0	K.YSHVDLVVMVDGFEGEK.G	Oxidation (M)	(Ions score 56)
168 - 184	970.4597	1938.9048	1938.9034	1	0	K.YSHVDLVVMVDGFEGEK.G	Oxidation (M)	(Ions score 72)
168 - 184	970.4597	1938.9048	1938.9034	1	0	K.YSHVDLVVMVDGFEGEK.G	Oxidation (M)	(Ions score 59)
168 - 184	647.3089	1938.9048	1938.9034	1	0	K.YSHVDLVVMVDGFEGEK.G	Oxidation (M)	(Ions score 38)
168 - 184	970.4597	1938.9048	1938.9034	1	0	K.YSHVDLVVMVDGFEGEK.G	Oxidation (M)	(Ions score 58)
168 - 184	647.3089	1938.9048	1938.9034	1	0	K.YSHVDLVVMVDGFEGEK.G	Oxidation (M)	(Ions score 54)
168 - 184	647.3089	1938.9048	1938.9034	1	0	K.YSHVDLVVMVDGFEGEK.G	Oxidation (M)	(Ions score 69)
168 - 184	647.3089	1938.9048	1938.9034	1	0	K.YSHVDLVVMVDGFEGEK.G	Oxidation (M)	(Ions score 69)
168 - 184	647.3089	1938.9048	1938.9034	1	0	K.YSHVDLVVMVDGFEGEK.G	Oxidation (M)	(Ions score 53)
168 - 184	647.3089	1938.9048	1938.9034	1	0	K.YSHVDLVVMVDGFEGEK.G	Oxidation (M)	(Ions score 59)
168 - 184	647.3089	1938.9048	1938.9034	1	0	K.YSHVDLVVMVDGFEGEK.G	Oxidation (M)	(Ions score 69)
168 - 184	970.4597	1938.9048	1938.9034	1	0	K.YSHVDLVVMVDGFEGEK.G	Oxidation (M)	(Ions score 86)
168 - 184	970.4597	1938.9049	1938.9034	1	0	K.YSHVDLVVMVDGFEGEK.G	Oxidation (M)	(Ions score 52)
168 - 184	970.4597	1938.9049	1938.9034	1	0	K.YSHVDLVVMVDGFEGEK.G	Oxidation (M)	(Ions score 59)
168 - 184	970.4597	1938.9049	1938.9034	1	0	K.YSHVDLVVMVDGFEGEK.G	Oxidation (M)	(Ions score 63)
168 - 184	647.3089	1938.9049	1938.9034	1	0	K.YSHVDLVVMVDGFEGEK.G	Oxidation (M)	(Ions score 56)
168 - 184	647.3089	1938.9049	1938.9034	1	0	K.YSHVDLVVMVDGFEGEK.G	Oxidation (M)	(Ions score 58)
168 - 184	647.3089	1938.9049	1938.9034	1	0	K.YSHVDLVVMVDGFEGEK.G	Oxidation (M)	(Ions score 61)
168 - 184	647.3089	1938.9049	1938.9034	1	0	K.YSHVDLVVMVDGFEGEK.G	Oxidation (M)	(Ions score 58)
168 - 184	970.4597	1938.9049	1938.9034	1	0	K.YSHVDLVVMVDGFEGEK.G	Oxidation (M)	(Ions score 40)
168 - 184	970.4598	1938.9050	1938.9034	1	0	K.YSHVDLVVMVDGFEGEK.G	Oxidation (M)	(Ions score 56)
168 - 184	647.3089	1938.9050	1938.9034	1	0	K.YSHVDLVVMVDGFEGEK.G	Oxidation (M)	(Ions score 66)
168 - 184	970.4598	1938.9050	1938.9034	1	0	K.YSHVDLVVMVDGFEGEK.G	Oxidation (M)	(Ions score 53)
168 - 184	647.3090	1938.9051	1938.9034	1	0	K.YSHVDLVVMVDGFEGEK.G	Oxidation (M)	(Ions score 46)
168 - 184	970.4598	1938.9051	1938.9034	1	0	K.YSHVDLVVMVDGFEGEK.G	Oxidation (M)	(Ions score 63)
168 - 184	647.3090	1938.9051	1938.9034	1	0	K.YSHVDLVVMVDGFEGEK.G	Oxidation (M)	(Ions score 66)
168 - 184	647.3090	1938.9051	1938.9034	1	0	K.YSHVDLVVMVDGFEGEK.G	Oxidation (M)	(Ions score 59)
168 - 184	647.3090	1938.9051	1938.9034	1	0	K.YSHVDLVVMVDGFEGEK.G	Oxidation (M)	(Ions score 58)
168 - 184	647.3090	1938.9051	1938.9034	1	0	K.YSHVDLVVMVDGFEGEK.G	Oxidation (M)	(Ions score 39)
168 - 184	970.4598	1938.9051	1938.9034	1	0	K.YSHVDLVVMVDGFEGEK.G	Oxidation (M)	(Ions score 56)
168 - 184	970.4599	1938.9051	1938.9034	1	0	K.YSHVDLVVMVDGFEGEK.G	Oxidation (M)	(Ions score 52)
168 - 184	647.3090	1938.9052	1938.9034	1	0	K.YSHVDLVVMVDGFEGEK.G	Oxidation (M)	(Ions score 55)
168 - 184	647.3090	1938.9052	1938.9034	1	0	K.YSHVDLVVMVDGFEGEK.G	Oxidation (M)	(Ions score 59)
168 - 184	647.3090	1938.9052	1938.9034	1	0	K.YSHVDLVVMVDGFEGEK.G	Oxidation (M)	(Ions score 56)
168 - 184	647.3090	1938.9052	1938.9034	1	0	K.YSHVDLVVMVDGFEGEK.G	Oxidation (M)	(Ions score 50)

168 - 184	970.4599	1938.9052	1938.9034	1	0	K.YSHVDLVVMVDGFEGEK.G	Oxidation (M)	(Ions score 52)
168 - 184	647.3090	1938.9052	1938.9034	1	0	K.YSHVDLVVMVDGFEGEK.G	Oxidation (M)	(Ions score 58)
168 - 184	647.3090	1938.9052	1938.9034	1	0	K.YSHVDLVVMVDGFEGEK.G	Oxidation (M)	(Ions score 58)
168 - 184	970.4599	1938.9052	1938.9034	1	0	K.YSHVDLVVMVDGFEGEK.G	Oxidation (M)	(Ions score 58)
168 - 184	647.3090	1938.9052	1938.9034	1	0	K.YSHVDLVVMVDGFEGEK.G	Oxidation (M)	(Ions score 58)
168 - 184	970.4599	1938.9053	1938.9034	1	0	K.YSHVDLVVMVDGFEGEK.G	Oxidation (M)	(Ions score 45)
168 - 184	647.3090	1938.9053	1938.9034	1	0	K.YSHVDLVVMVDGFEGEK.G	Oxidation (M)	(Ions score 61)
168 - 184	647.3090	1938.9053	1938.9034	1	0	K.YSHVDLVVMVDGFEGEK.G	Oxidation (M)	(Ions score 58)
168 - 184	970.4599	1938.9053	1938.9034	1	0	K.YSHVDLVVMVDGFEGEK.G	Oxidation (M)	(Ions score 58)
168 - 184	647.3091	1938.9053	1938.9034	1	0	K.YSHVDLVVMVDGFEGEK.G	Oxidation (M)	(Ions score 58)
168 - 184	970.4600	1938.9054	1938.9034	1	0	K.YSHVDLVVMVDGFEGEK.G	Oxidation (M)	(Ions score 71)
168 - 184	970.4600	1938.9054	1938.9034	1	0	K.YSHVDLVVMVDGFEGEK.G	Oxidation (M)	(Ions score 68)
168 - 184	647.3091	1938.9054	1938.9034	1	0	K.YSHVDLVVMVDGFEGEK.G	Oxidation (M)	(Ions score 52)
168 - 184	647.3091	1938.9054	1938.9034	1	0	K.YSHVDLVVMVDGFEGEK.G	Oxidation (M)	(Ions score 62)
168 - 184	647.3091	1938.9054	1938.9034	1	0	K.YSHVDLVVMVDGFEGEK.G	Oxidation (M)	(Ions score 58)
168 - 184	970.4600	1938.9054	1938.9034	1	0	K.YSHVDLVVMVDGFEGEK.G	Oxidation (M)	(Ions score 52)
168 - 184	647.3091	1938.9054	1938.9034	1	0	K.YSHVDLVVMVDGFEGEK.G	Oxidation (M)	(Ions score 61)
168 - 184	647.3091	1938.9054	1938.9034	1	0	K.YSHVDLVVMVDGFEGEK.G	Oxidation (M)	(Ions score 69)
168 - 184	647.3091	1938.9055	1938.9034	1	0	K.YSHVDLVVMVDGFEGEK.G	Oxidation (M)	(Ions score 53)
168 - 184	970.4600	1938.9055	1938.9034	1	0	K.YSHVDLVVMVDGFEGEK.G	Oxidation (M)	(Ions score 52)
168 - 184	647.3091	1938.9055	1938.9034	1	0	K.YSHVDLVVMVDGFEGEK.G	Oxidation (M)	(Ions score 64)
168 - 184	970.4600	1938.9055	1938.9034	1	0	K.YSHVDLVVMVDGFEGEK.G	Oxidation (M)	(Ions score 55)
168 - 184	970.4600	1938.9055	1938.9034	1	0	K.YSHVDLVVMVDGFEGEK.G	Oxidation (M)	(Ions score 59)
168 - 184	970.4600	1938.9055	1938.9034	1	0	K.YSHVDLVVMVDGFEGEK.G	Oxidation (M)	(Ions score 68)
168 - 184	647.3091	1938.9055	1938.9034	1	0	K.YSHVDLVVMVDGFEGEK.G	Oxidation (M)	(Ions score 50)
168 - 184	647.3091	1938.9055	1938.9034	1	0	K.YSHVDLVVMVDGFEGEK.G	Oxidation (M)	(Ions score 69)
168 - 184	970.4600	1938.9055	1938.9034	1	0	K.YSHVDLVVMVDGFEGEK.G	Oxidation (M)	(Ions score 60)
168 - 184	647.3091	1938.9055	1938.9034	1	0	K.YSHVDLVVMVDGFEGEK.G	Oxidation (M)	(Ions score 60)
168 - 184	647.3091	1938.9055	1938.9034	1	0	K.YSHVDLVVMVDGFEGEK.G	Oxidation (M)	(Ions score 57)
168 - 184	647.3091	1938.9056	1938.9034	1	0	K.YSHVDLVVMVDGFEGEK.G	Oxidation (M)	(Ions score 42)
168 - 184	647.3091	1938.9056	1938.9034	1	0	K.YSHVDLVVMVDGFEGEK.G	Oxidation (M)	(Ions score 47)
168 - 184	647.3091	1938.9056	1938.9034	1	0	K.YSHVDLVVMVDGFEGEK.G	Oxidation (M)	(Ions score 58)
168 - 184	647.3091	1938.9056	1938.9034	1	0	K.YSHVDLVVMVDGFEGEK.G	Oxidation (M)	(Ions score 53)
168 - 184	970.4601	1938.9056	1938.9034	1	0	K.YSHVDLVVMVDGFEGEK.G	Oxidation (M)	(Ions score 51)
168 - 184	970.4601	1938.9056	1938.9034	1	0	K.YSHVDLVVMVDGFEGEK.G	Oxidation (M)	(Ions score 60)
168 - 184	647.3091	1938.9056	1938.9034	1	0	K.YSHVDLVVMVDGFEGEK.G	Oxidation (M)	(Ions score 55)
168 - 184	647.3091	1938.9056	1938.9034	1	0	K.YSHVDLVVMVDGFEGEK.G	Oxidation (M)	(Ions score 58)
168 - 184	647.3092	1938.9056	1938.9034	1	0	K.YSHVDLVVMVDGFEGEK.G	Oxidation (M)	(Ions score 61)
168 - 184	970.4601	1938.9056	1938.9034	1	0	K.YSHVDLVVMVDGFEGEK.G	Oxidation (M)	(Ions score 61)
168 - 184	647.3092	1938.9056	1938.9034	1	0	K.YSHVDLVVMVDGFEGEK.G	Oxidation (M)	(Ions score 60)
168 - 184	970.4601	1938.9056	1938.9034	1	0	K.YSHVDLVVMVDGFEGEK.G	Oxidation (M)	(Ions score 53)
168 - 184	647.3092	1938.9056	1938.9034	1	0	K.YSHVDLVVMVDGFEGEK.G	Oxidation (M)	(Ions score 63)
168 - 184	647.3092	1938.9056	1938.9034	1	0	K.YSHVDLVVMVDGFEGEK.G	Oxidation (M)	(Ions score 63)
168 - 184	647.3092	1938.9056	1938.9034	1	0	K.YSHVDLVVMVDGFEGEK.G	Oxidation (M)	(Ions score 40)
168 - 184	970.4601	1938.9056	1938.9034	1	0	K.YSHVDLVVMVDGFEGEK.G	Oxidation (M)	(Ions score 60)
168 - 184	647.3092	1938.9056	1938.9034	1	0	K.YSHVDLVVMVDGFEGEK.G	Oxidation (M)	(Ions score 55)
168 - 184	647.3092	1938.9057	1938.9034	1	0	K.YSHVDLVVMVDGFEGEK.G	Oxidation (M)	(Ions score 46)
168 - 184	970.4601	1938.9057	1938.9034	1	0	K.YSHVDLVVMVDGFEGEK.G	Oxidation (M)	(Ions score 57)
168 - 184	647.3092	1938.9057	1938.9034	1	0	K.YSHVDLVVMVDGFEGEK.G	Oxidation (M)	(Ions score 65)
168 - 184	647.3092	1938.9057	1938.9034	1	0	K.YSHVDLVVMVDGFEGEK.G	Oxidation (M)	(Ions score 66)
168 - 184	970.4601	1938.9057	1938.9034	1	0	K.YSHVDLVVMVDGFEGEK.G	Oxidation (M)	(Ions score 56)
168 - 184	970.4601	1938.9057	1938.9034	1	0	K.YSHVDLVVMVDGFEGEK.G	Oxidation (M)	(Ions score 52)
168 - 184	647.3092	1938.9057	1938.9034	1	0	K.YSHVDLVVMVDGFEGEK.G	Oxidation (M)	(Ions score 56)
168 - 184	647.3092	1938.9057	1938.9034	1	0	K.YSHVDLVVMVDGFEGEK.G	Oxidation (M)	(Ions score 56)
168 - 184	970.4601	1938.9057	1938.9034	1	0	K.YSHVDLVVMVDGFEGEK.G	Oxidation (M)	(Ions score 58)
168 - 184	647.3092	1938.9057	1938.9034	1	0	K.YSHVDLVVMVDGFEGEK.G	Oxidation (M)	(Ions score 56)
168 - 184	647.3092	1938.9057	1938.9034	1	0	K.YSHVDLVVMVDGFEGEK.G	Oxidation (M)	(Ions score 58)
168 - 184	647.3092	1938.9057	1938.9034	1	0	K.YSHVDLVVMVDGFEGEK.G	Oxidation (M)	(Ions score 61)

168 - 184	647.3092	1938.9057	1938.9034	1	0	K.YSHVDLVVMVDGFEGEK.G	Oxidation (M)	(Ions score 50)
168 - 184	970.4601	1938.9057	1938.9034	1	0	K.YSHVDLVVMVDGFEGEK.G	Oxidation (M)	(Ions score 70)
168 - 184	647.3092	1938.9057	1938.9034	1	0	K.YSHVDLVVMVDGFEGEK.G	Oxidation (M)	(Ions score 58)
168 - 184	970.4601	1938.9057	1938.9034	1	0	K.YSHVDLVVMVDGFEGEK.G	Oxidation (M)	(Ions score 70)
168 - 184	647.3092	1938.9057	1938.9034	1	0	K.YSHVDLVVMVDGFEGEK.G	Oxidation (M)	(Ions score 62)
168 - 184	647.3092	1938.9058	1938.9034	1	0	K.YSHVDLVVMVDGFEGEK.G	Oxidation (M)	(Ions score 58)
168 - 184	647.3092	1938.9058	1938.9034	1	0	K.YSHVDLVVMVDGFEGEK.G	Oxidation (M)	(Ions score 60)
168 - 184	970.4602	1938.9058	1938.9034	1	0	K.YSHVDLVVMVDGFEGEK.G	Oxidation (M)	(Ions score 61)
168 - 184	647.3092	1938.9058	1938.9034	1	0	K.YSHVDLVVMVDGFEGEK.G	Oxidation (M)	(Ions score 59)
168 - 184	970.4602	1938.9058	1938.9034	1	0	K.YSHVDLVVMVDGFEGEK.G	Oxidation (M)	(Ions score 50)
168 - 184	647.3092	1938.9058	1938.9034	1	0	K.YSHVDLVVMVDGFEGEK.G	Oxidation (M)	(Ions score 63)
168 - 184	970.4602	1938.9058	1938.9034	1	0	K.YSHVDLVVMVDGFEGEK.G	Oxidation (M)	(Ions score 58)
168 - 184	647.3092	1938.9058	1938.9034	1	0	K.YSHVDLVVMVDGFEGEK.G	Oxidation (M)	(Ions score 56)
168 - 184	647.3092	1938.9058	1938.9034	1	0	K.YSHVDLVVMVDGFEGEK.G	Oxidation (M)	(Ions score 55)
168 - 184	647.3092	1938.9059	1938.9034	1	0	K.YSHVDLVVMVDGFEGEK.G	Oxidation (M)	(Ions score 51)
168 - 184	647.3092	1938.9059	1938.9034	1	0	K.YSHVDLVVMVDGFEGEK.G	Oxidation (M)	(Ions score 66)
168 - 184	647.3092	1938.9059	1938.9034	1	0	K.YSHVDLVVMVDGFEGEK.G	Oxidation (M)	(Ions score 62)
168 - 184	647.3092	1938.9059	1938.9034	1	0	K.YSHVDLVVMVDGFEGEK.G	Oxidation (M)	(Ions score 58)
168 - 184	970.4602	1938.9059	1938.9034	1	0	K.YSHVDLVVMVDGFEGEK.G	Oxidation (M)	(Ions score 58)
168 - 184	970.4602	1938.9059	1938.9034	1	0	K.YSHVDLVVMVDGFEGEK.G	Oxidation (M)	(Ions score 55)
168 - 184	647.3093	1938.9060	1938.9034	1	0	K.YSHVDLVVMVDGFEGEK.G	Oxidation (M)	(Ions score 41)
168 - 184	647.3093	1938.9060	1938.9034	1	0	K.YSHVDLVVMVDGFEGEK.G	Oxidation (M)	(Ions score 57)
168 - 184	647.3093	1938.9060	1938.9034	1	0	K.YSHVDLVVMVDGFEGEK.G	Oxidation (M)	(Ions score 53)
168 - 184	970.4603	1938.9060	1938.9034	1	0	K.YSHVDLVVMVDGFEGEK.G	Oxidation (M)	(Ions score 49)
168 - 184	647.3093	1938.9060	1938.9034	1	0	K.YSHVDLVVMVDGFEGEK.G	Oxidation (M)	(Ions score 62)
168 - 184	970.4603	1938.9060	1938.9034	1	0	K.YSHVDLVVMVDGFEGEK.G	Oxidation (M)	(Ions score 56)
168 - 184	647.3093	1938.9060	1938.9034	1	0	K.YSHVDLVVMVDGFEGEK.G	Oxidation (M)	(Ions score 58)
168 - 184	647.3093	1938.9060	1938.9034	1	0	K.YSHVDLVVMVDGFEGEK.G	Oxidation (M)	(Ions score 65)
168 - 184	647.3093	1938.9060	1938.9034	1	0	K.YSHVDLVVMVDGFEGEK.G	Oxidation (M)	(Ions score 64)
168 - 184	647.3093	1938.9060	1938.9034	1	0	K.YSHVDLVVMVDGFEGEK.G	Oxidation (M)	(Ions score 56)
168 - 184	970.4603	1938.9061	1938.9034	1	0	K.YSHVDLVVMVDGFEGEK.G	Oxidation (M)	(Ions score 59)
168 - 184	647.3093	1938.9061	1938.9034	1	0	K.YSHVDLVVMVDGFEGEK.G	Oxidation (M)	(Ions score 49)
168 - 184	970.4603	1938.9061	1938.9034	1	0	K.YSHVDLVVMVDGFEGEK.G	Oxidation (M)	(Ions score 58)
168 - 184	647.3093	1938.9061	1938.9034	1	0	K.YSHVDLVVMVDGFEGEK.G	Oxidation (M)	(Ions score 66)
168 - 184	647.3093	1938.9061	1938.9034	1	0	K.YSHVDLVVMVDGFEGEK.G	Oxidation (M)	(Ions score 61)
168 - 184	970.4603	1938.9061	1938.9034	1	0	K.YSHVDLVVMVDGFEGEK.G	Oxidation (M)	(Ions score 70)
168 - 184	970.4603	1938.9061	1938.9034	1	0	K.YSHVDLVVMVDGFEGEK.G	Oxidation (M)	(Ions score 56)
168 - 184	647.3093	1938.9061	1938.9034	1	0	K.YSHVDLVVMVDGFEGEK.G	Oxidation (M)	(Ions score 55)
168 - 184	647.3093	1938.9061	1938.9034	1	0	K.YSHVDLVVMVDGFEGEK.G	Oxidation (M)	(Ions score 61)
168 - 184	970.4603	1938.9061	1938.9034	1	0	K.YSHVDLVVMVDGFEGEK.G	Oxidation (M)	(Ions score 46)
168 - 184	647.3093	1938.9061	1938.9034	1	0	K.YSHVDLVVMVDGFEGEK.G	Oxidation (M)	(Ions score 63)
168 - 184	970.4603	1938.9061	1938.9034	1	0	K.YSHVDLVVMVDGFEGEK.G	Oxidation (M)	(Ions score 56)
168 - 184	647.3093	1938.9061	1938.9034	1	0	K.YSHVDLVVMVDGFEGEK.G	Oxidation (M)	(Ions score 61)
168 - 184	970.4603	1938.9061	1938.9034	1	0	K.YSHVDLVVMVDGFEGEK.G	Oxidation (M)	(Ions score 52)
168 - 184	970.4603	1938.9061	1938.9034	1	0	K.YSHVDLVVMVDGFEGEK.G	Oxidation (M)	(Ions score 55)
168 - 184	970.4603	1938.9061	1938.9034	1	0	K.YSHVDLVVMVDGFEGEK.G	Oxidation (M)	(Ions score 60)
168 - 184	647.3093	1938.9062	1938.9034	1	0	K.YSHVDLVVMVDGFEGEK.G	Oxidation (M)	(Ions score 53)
168 - 184	647.3093	1938.9062	1938.9034	1	0	K.YSHVDLVVMVDGFEGEK.G	Oxidation (M)	(Ions score 58)
168 - 184	647.3093	1938.9062	1938.9034	1	0	K.YSHVDLVVMVDGFEGEK.G	Oxidation (M)	(Ions score 66)
168 - 184	970.4604	1938.9062	1938.9034	1	0	K.YSHVDLVVMVDGFEGEK.G	Oxidation (M)	(Ions score 74)
168 - 184	970.4604	1938.9062	1938.9034	1	0	K.YSHVDLVVMVDGFEGEK.G	Oxidation (M)	(Ions score 62)
168 - 184	647.3093	1938.9062	1938.9034	1	0	K.YSHVDLVVMVDGFEGEK.G	Oxidation (M)	(Ions score 56)
168 - 184	970.4604	1938.9062	1938.9034	1	0	K.YSHVDLVVMVDGFEGEK.G	Oxidation (M)	(Ions score 60)
168 - 184	647.3094	1938.9062	1938.9034	1	0	K.YSHVDLVVMVDGFEGEK.G	Oxidation (M)	(Ions score 60)
168 - 184	647.3094	1938.9062	1938.9034	1	0	K.YSHVDLVVMVDGFEGEK.G	Oxidation (M)	(Ions score 42)
168 - 184	970.4604	1938.9062	1938.9034	1	0	K.YSHVDLVVMVDGFEGEK.G	Oxidation (M)	(Ions score 58)
168 - 184	647.3094	1938.9062	1938.9034	1	0	K.YSHVDLVVMVDGFEGEK.G	Oxidation (M)	(Ions score 56)
168 - 184	647.3094	1938.9062	1938.9034	1	0	K.YSHVDLVVMVDGFEGEK.G	Oxidation (M)	(Ions score 66)

168 - 184	647.3094	1938.9063	1938.9034	2	0	K.YSHVDLVVMVDGFEGEK.G	Oxidation (M)	(Ions score 58)
168 - 184	647.3094	1938.9063	1938.9034	2	0	K.YSHVDLVVMVDGFEGEK.G	Oxidation (M)	(Ions score 58)
168 - 184	647.3094	1938.9063	1938.9034	2	0	K.YSHVDLVVMVDGFEGEK.G	Oxidation (M)	(Ions score 60)
168 - 184	970.4604	1938.9063	1938.9034	2	0	K.YSHVDLVVMVDGFEGEK.G	Oxidation (M)	(Ions score 58)
168 - 184	647.3094	1938.9063	1938.9034	2	0	K.YSHVDLVVMVDGFEGEK.G	Oxidation (M)	(Ions score 61)
168 - 184	970.4604	1938.9063	1938.9034	2	0	K.YSHVDLVVMVDGFEGEK.G	Oxidation (M)	(Ions score 54)
168 - 184	970.4604	1938.9063	1938.9034	2	0	K.YSHVDLVVMVDGFEGEK.G	Oxidation (M)	(Ions score 57)
168 - 184	970.4604	1938.9063	1938.9034	2	0	K.YSHVDLVVMVDGFEGEK.G	Oxidation (M)	(Ions score 53)
168 - 184	647.3094	1938.9063	1938.9034	2	0	K.YSHVDLVVMVDGFEGEK.G	Oxidation (M)	(Ions score 72)
168 - 184	647.3094	1938.9063	1938.9034	2	0	K.YSHVDLVVMVDGFEGEK.G	Oxidation (M)	(Ions score 57)
168 - 184	970.4604	1938.9063	1938.9034	2	0	K.YSHVDLVVMVDGFEGEK.G	Oxidation (M)	(Ions score 52)
168 - 184	970.4604	1938.9063	1938.9034	2	0	K.YSHVDLVVMVDGFEGEK.G	Oxidation (M)	(Ions score 42)
168 - 184	970.4604	1938.9063	1938.9034	2	0	K.YSHVDLVVMVDGFEGEK.G	Oxidation (M)	(Ions score 64)
168 - 184	970.4604	1938.9063	1938.9034	2	0	K.YSHVDLVVMVDGFEGEK.G	Oxidation (M)	(Ions score 43)
168 - 184	647.3094	1938.9063	1938.9034	2	0	K.YSHVDLVVMVDGFEGEK.G	Oxidation (M)	(Ions score 58)
168 - 184	970.4604	1938.9063	1938.9034	2	0	K.YSHVDLVVMVDGFEGEK.G	Oxidation (M)	(Ions score 56)
168 - 184	970.4604	1938.9063	1938.9034	2	0	K.YSHVDLVVMVDGFEGEK.G	Oxidation (M)	(Ions score 55)
168 - 184	970.4604	1938.9063	1938.9034	2	0	K.YSHVDLVVMVDGFEGEK.G	Oxidation (M)	(Ions score 55)
168 - 184	970.4605	1938.9064	1938.9034	2	0	K.YSHVDLVVMVDGFEGEK.G	Oxidation (M)	(Ions score 57)
168 - 184	970.4605	1938.9064	1938.9034	2	0	K.YSHVDLVVMVDGFEGEK.G	Oxidation (M)	(Ions score 56)
168 - 184	970.4605	1938.9064	1938.9034	2	0	K.YSHVDLVVMVDGFEGEK.G	Oxidation (M)	(Ions score 50)
168 - 184	647.3094	1938.9064	1938.9034	2	0	K.YSHVDLVVMVDGFEGEK.G	Oxidation (M)	(Ions score 59)
168 - 184	970.4605	1938.9064	1938.9034	2	0	K.YSHVDLVVMVDGFEGEK.G	Oxidation (M)	(Ions score 59)
168 - 184	970.4605	1938.9064	1938.9034	2	0	K.YSHVDLVVMVDGFEGEK.G	Oxidation (M)	(Ions score 44)
168 - 184	647.3094	1938.9064	1938.9034	2	0	K.YSHVDLVVMVDGFEGEK.G	Oxidation (M)	(Ions score 56)
168 - 184	970.4605	1938.9064	1938.9034	2	0	K.YSHVDLVVMVDGFEGEK.G	Oxidation (M)	(Ions score 70)
168 - 184	647.3094	1938.9064	1938.9034	2	0	K.YSHVDLVVMVDGFEGEK.G	Oxidation (M)	(Ions score 49)
168 - 184	970.4605	1938.9064	1938.9034	2	0	K.YSHVDLVVMVDGFEGEK.G	Oxidation (M)	(Ions score 57)
168 - 184	647.3094	1938.9064	1938.9034	2	0	K.YSHVDLVVMVDGFEGEK.G	Oxidation (M)	(Ions score 81)
168 - 184	647.3094	1938.9065	1938.9034	2	0	K.YSHVDLVVMVDGFEGEK.G	Oxidation (M)	(Ions score 69)
168 - 184	647.3094	1938.9065	1938.9034	2	0	K.YSHVDLVVMVDGFEGEK.G	Oxidation (M)	(Ions score 58)
168 - 184	970.4605	1938.9065	1938.9034	2	0	K.YSHVDLVVMVDGFEGEK.G	Oxidation (M)	(Ions score 54)
168 - 184	647.3094	1938.9065	1938.9034	2	0	K.YSHVDLVVMVDGFEGEK.G	Oxidation (M)	(Ions score 57)
168 - 184	647.3094	1938.9065	1938.9034	2	0	K.YSHVDLVVMVDGFEGEK.G	Oxidation (M)	(Ions score 61)
168 - 184	647.3095	1938.9065	1938.9034	2	0	K.YSHVDLVVMVDGFEGEK.G	Oxidation (M)	(Ions score 58)
168 - 184	970.4605	1938.9065	1938.9034	2	0	K.YSHVDLVVMVDGFEGEK.G	Oxidation (M)	(Ions score 49)
168 - 184	970.4606	1938.9065	1938.9034	2	0	K.YSHVDLVVMVDGFEGEK.G	Oxidation (M)	(Ions score 55)
168 - 184	970.4606	1938.9066	1938.9034	2	0	K.YSHVDLVVMVDGFEGEK.G	Oxidation (M)	(Ions score 73)
168 - 184	970.4606	1938.9066	1938.9034	2	0	K.YSHVDLVVMVDGFEGEK.G	Oxidation (M)	(Ions score 70)
168 - 184	647.3095	1938.9066	1938.9034	2	0	K.YSHVDLVVMVDGFEGEK.G	Oxidation (M)	(Ions score 45)
168 - 184	970.4606	1938.9066	1938.9034	2	0	K.YSHVDLVVMVDGFEGEK.G	Oxidation (M)	(Ions score 70)
168 - 184	647.3095	1938.9066	1938.9034	2	0	K.YSHVDLVVMVDGFEGEK.G	Oxidation (M)	(Ions score 58)
168 - 184	647.3095	1938.9066	1938.9034	2	0	K.YSHVDLVVMVDGFEGEK.G	Oxidation (M)	(Ions score 67)
168 - 184	647.3095	1938.9066	1938.9034	2	0	K.YSHVDLVVMVDGFEGEK.G	Oxidation (M)	(Ions score 63)
168 - 184	970.4606	1938.9066	1938.9034	2	0	K.YSHVDLVVMVDGFEGEK.G	Oxidation (M)	(Ions score 74)
168 - 184	647.3095	1938.9066	1938.9034	2	0	K.YSHVDLVVMVDGFEGEK.G	Oxidation (M)	(Ions score 50)
168 - 184	970.4606	1938.9067	1938.9034	2	0	K.YSHVDLVVMVDGFEGEK.G	Oxidation (M)	(Ions score 57)
168 - 184	970.4606	1938.9067	1938.9034	2	0	K.YSHVDLVVMVDGFEGEK.G	Oxidation (M)	(Ions score 70)
168 - 184	647.3095	1938.9067	1938.9034	2	0	K.YSHVDLVVMVDGFEGEK.G	Oxidation (M)	(Ions score 61)
168 - 184	647.3095	1938.9067	1938.9034	2	0	K.YSHVDLVVMVDGFEGEK.G	Oxidation (M)	(Ions score 53)
168 - 184	970.4606	1938.9067	1938.9034	2	0	K.YSHVDLVVMVDGFEGEK.G	Oxidation (M)	(Ions score 54)
168 - 184	647.3095	1938.9067	1938.9034	2	0	K.YSHVDLVVMVDGFEGEK.G	Oxidation (M)	(Ions score 61)
168 - 184	647.3095	1938.9067	1938.9034	2	0	K.YSHVDLVVMVDGFEGEK.G	Oxidation (M)	(Ions score 58)
168 - 184	970.4606	1938.9067	1938.9034	2	0	K.YSHVDLVVMVDGFEGEK.G	Oxidation (M)	(Ions score 49)
168 - 184	970.4606	1938.9067	1938.9034	2	0	K.YSHVDLVVMVDGFEGEK.G	Oxidation (M)	(Ions score 74)
168 - 184	970.4607	1938.9068	1938.9034	2	0	K.YSHVDLVVMVDGFEGEK.G	Oxidation (M)	(Ions score 48)
168 - 184	647.3095	1938.9068	1938.9034	2	0	K.YSHVDLVVMVDGFEGEK.G	Oxidation (M)	(Ions score 58)
168 - 184	647.3095	1938.9068	1938.9034	2	0	K.YSHVDLVVMVDGFEGEK.G	Oxidation (M)	(Ions score 58)

168 - 184	647.3095	1938.9068	1938.9034	2	0	K.YSHVDLVVMVDGFEGEK.G	Oxidation (M)	(Ions score 45)
168 - 184	647.3096	1938.9068	1938.9034	2	0	K.YSHVDLVVMVDGFEGEK.G	Oxidation (M)	(Ions score 73)
168 - 184	647.3096	1938.9068	1938.9034	2	0	K.YSHVDLVVMVDGFEGEK.G	Oxidation (M)	(Ions score 66)
168 - 184	970.4607	1938.9068	1938.9034	2	0	K.YSHVDLVVMVDGFEGEK.G	Oxidation (M)	(Ions score 62)
168 - 184	647.3096	1938.9069	1938.9034	2	0	K.YSHVDLVVMVDGFEGEK.G	Oxidation (M)	(Ions score 58)
168 - 184	970.4607	1938.9069	1938.9034	2	0	K.YSHVDLVVMVDGFEGEK.G	Oxidation (M)	(Ions score 51)
168 - 184	647.3096	1938.9069	1938.9034	2	0	K.YSHVDLVVMVDGFEGEK.G	Oxidation (M)	(Ions score 58)
168 - 184	647.3096	1938.9069	1938.9034	2	0	K.YSHVDLVVMVDGFEGEK.G	Oxidation (M)	(Ions score 78)
168 - 184	647.3096	1938.9069	1938.9034	2	0	K.YSHVDLVVMVDGFEGEK.G	Oxidation (M)	(Ions score 58)
168 - 184	647.3096	1938.9069	1938.9034	2	0	K.YSHVDLVVMVDGFEGEK.G	Oxidation (M)	(Ions score 61)
168 - 184	970.4607	1938.9069	1938.9034	2	0	K.YSHVDLVVMVDGFEGEK.G	Oxidation (M)	(Ions score 39)
168 - 184	970.4607	1938.9069	1938.9034	2	0	K.YSHVDLVVMVDGFEGEK.G	Oxidation (M)	(Ions score 62)
168 - 184	647.3096	1938.9069	1938.9034	2	0	K.YSHVDLVVMVDGFEGEK.G	Oxidation (M)	(Ions score 53)
168 - 184	970.4607	1938.9069	1938.9034	2	0	K.YSHVDLVVMVDGFEGEK.G	Oxidation (M)	(Ions score 62)
168 - 184	647.3096	1938.9069	1938.9034	2	0	K.YSHVDLVVMVDGFEGEK.G	Oxidation (M)	(Ions score 43)
168 - 184	647.3096	1938.9070	1938.9034	2	0	K.YSHVDLVVMVDGFEGEK.G	Oxidation (M)	(Ions score 53)
168 - 184	970.4608	1938.9070	1938.9034	2	0	K.YSHVDLVVMVDGFEGEK.G	Oxidation (M)	(Ions score 61)
168 - 184	647.3096	1938.9070	1938.9034	2	0	K.YSHVDLVVMVDGFEGEK.G	Oxidation (M)	(Ions score 51)
168 - 184	970.4608	1938.9070	1938.9034	2	0	K.YSHVDLVVMVDGFEGEK.G	Oxidation (M)	(Ions score 72)
168 - 184	970.4608	1938.9070	1938.9034	2	0	K.YSHVDLVVMVDGFEGEK.G	Oxidation (M)	(Ions score 68)
168 - 184	970.4608	1938.9070	1938.9034	2	0	K.YSHVDLVVMVDGFEGEK.G	Oxidation (M)	(Ions score 70)
168 - 184	647.3096	1938.9070	1938.9034	2	0	K.YSHVDLVVMVDGFEGEK.G	Oxidation (M)	(Ions score 53)
168 - 184	970.4608	1938.9070	1938.9034	2	0	K.YSHVDLVVMVDGFEGEK.G	Oxidation (M)	(Ions score 58)
168 - 184	647.3096	1938.9071	1938.9034	2	0	K.YSHVDLVVMVDGFEGEK.G	Oxidation (M)	(Ions score 78)
168 - 184	970.4608	1938.9071	1938.9034	2	0	K.YSHVDLVVMVDGFEGEK.G	Oxidation (M)	(Ions score 58)
168 - 184	647.3096	1938.9071	1938.9034	2	0	K.YSHVDLVVMVDGFEGEK.G	Oxidation (M)	(Ions score 61)
168 - 184	970.4608	1938.9071	1938.9034	2	0	K.YSHVDLVVMVDGFEGEK.G	Oxidation (M)	(Ions score 59)
168 - 184	647.3096	1938.9071	1938.9034	2	0	K.YSHVDLVVMVDGFEGEK.G	Oxidation (M)	(Ions score 61)
168 - 184	970.4608	1938.9071	1938.9034	2	0	K.YSHVDLVVMVDGFEGEK.G	Oxidation (M)	(Ions score 65)
168 - 184	647.3097	1938.9071	1938.9034	2	0	K.YSHVDLVVMVDGFEGEK.G	Oxidation (M)	(Ions score 51)
168 - 184	970.4609	1938.9072	1938.9034	2	0	K.YSHVDLVVMVDGFEGEK.G	Oxidation (M)	(Ions score 62)
168 - 184	970.4609	1938.9072	1938.9034	2	0	K.YSHVDLVVMVDGFEGEK.G	Oxidation (M)	(Ions score 64)
168 - 184	970.4609	1938.9072	1938.9034	2	0	K.YSHVDLVVMVDGFEGEK.G	Oxidation (M)	(Ions score 53)
168 - 184	970.4609	1938.9072	1938.9034	2	0	K.YSHVDLVVMVDGFEGEK.G	Oxidation (M)	(Ions score 53)
168 - 184	970.4609	1938.9072	1938.9034	2	0	K.YSHVDLVVMVDGFEGEK.G	Oxidation (M)	(Ions score 63)
168 - 184	647.3097	1938.9072	1938.9034	2	0	K.YSHVDLVVMVDGFEGEK.G	Oxidation (M)	(Ions score 66)
168 - 184	647.3097	1938.9072	1938.9034	2	0	K.YSHVDLVVMVDGFEGEK.G	Oxidation (M)	(Ions score 75)
168 - 184	970.4609	1938.9072	1938.9034	2	0	K.YSHVDLVVMVDGFEGEK.G	Oxidation (M)	(Ions score 51)
168 - 184	647.3097	1938.9072	1938.9034	2	0	K.YSHVDLVVMVDGFEGEK.G	Oxidation (M)	(Ions score 62)
168 - 184	970.4609	1938.9072	1938.9034	2	0	K.YSHVDLVVMVDGFEGEK.G	Oxidation (M)	(Ions score 49)
168 - 184	647.3097	1938.9072	1938.9034	2	0	K.YSHVDLVVMVDGFEGEK.G	Oxidation (M)	(Ions score 56)
168 - 184	647.3097	1938.9073	1938.9034	2	0	K.YSHVDLVVMVDGFEGEK.G	Oxidation (M)	(Ions score 66)
168 - 184	647.3097	1938.9073	1938.9034	2	0	K.YSHVDLVVMVDGFEGEK.G	Oxidation (M)	(Ions score 66)
168 - 184	970.4609	1938.9073	1938.9034	2	0	K.YSHVDLVVMVDGFEGEK.G	Oxidation (M)	(Ions score 58)
168 - 184	647.3097	1938.9073	1938.9034	2	0	K.YSHVDLVVMVDGFEGEK.G	Oxidation (M)	(Ions score 66)
168 - 184	970.4609	1938.9073	1938.9034	2	0	K.YSHVDLVVMVDGFEGEK.G	Oxidation (M)	(Ions score 57)
168 - 184	647.3097	1938.9073	1938.9034	2	0	K.YSHVDLVVMVDGFEGEK.G	Oxidation (M)	(Ions score 59)
168 - 184	970.4609	1938.9073	1938.9034	2	0	K.YSHVDLVVMVDGFEGEK.G	Oxidation (M)	(Ions score 70)
168 - 184	970.4609	1938.9073	1938.9034	2	0	K.YSHVDLVVMVDGFEGEK.G	Oxidation (M)	(Ions score 71)
168 - 184	647.3097	1938.9073	1938.9034	2	0	K.YSHVDLVVMVDGFEGEK.G	Oxidation (M)	(Ions score 58)
168 - 184	970.4609	1938.9073	1938.9034	2	0	K.YSHVDLVVMVDGFEGEK.G	Oxidation (M)	(Ions score 70)
168 - 184	647.3097	1938.9073	1938.9034	2	0	K.YSHVDLVVMVDGFEGEK.G	Oxidation (M)	(Ions score 63)
168 - 184	647.3097	1938.9073	1938.9034	2	0	K.YSHVDLVVMVDGFEGEK.G	Oxidation (M)	(Ions score 61)
168 - 184	647.3097	1938.9073	1938.9034	2	0	K.YSHVDLVVMVDGFEGEK.G	Oxidation (M)	(Ions score 58)
168 - 184	970.4609	1938.9073	1938.9034	2	0	K.YSHVDLVVMVDGFEGEK.G	Oxidation (M)	(Ions score 61)
168 - 184	647.3097	1938.9074	1938.9034	2	0	K.YSHVDLVVMVDGFEGEK.G	Oxidation (M)	(Ions score 56)
168 - 184	647.3097	1938.9074	1938.9034	2	0	K.YSHVDLVVMVDGFEGEK.G	Oxidation (M)	(Ions score 72)
168 - 184	970.4610	1938.9074	1938.9034	2	0	K.YSHVDLVVMVDGFEGEK.G	Oxidation (M)	(Ions score 70)

168 - 184	970.4610	1938.9074	1938.9034	2	0	K.YSHVDLVVMVDGFEGEK.G	Oxidation (M)	(Ions score 58)
168 - 184	970.4610	1938.9074	1938.9034	2	0	K.YSHVDLVVMVDGFEGEK.G	Oxidation (M)	(Ions score 53)
168 - 184	970.4610	1938.9074	1938.9034	2	0	K.YSHVDLVVMVDGFEGEK.G	Oxidation (M)	(Ions score 59)
168 - 184	970.4610	1938.9074	1938.9034	2	0	K.YSHVDLVVMVDGFEGEK.G	Oxidation (M)	(Ions score 56)
168 - 184	647.3097	1938.9074	1938.9034	2	0	K.YSHVDLVVMVDGFEGEK.G	Oxidation (M)	(Ions score 54)
168 - 184	647.3097	1938.9074	1938.9034	2	0	K.YSHVDLVVMVDGFEGEK.G	Oxidation (M)	(Ions score 56)
168 - 184	970.4610	1938.9074	1938.9034	2	0	K.YSHVDLVVMVDGFEGEK.G	Oxidation (M)	(Ions score 57)
168 - 184	970.4610	1938.9074	1938.9034	2	0	K.YSHVDLVVMVDGFEGEK.G	Oxidation (M)	(Ions score 55)
168 - 184	647.3098	1938.9074	1938.9034	2	0	K.YSHVDLVVMVDGFEGEK.G	Oxidation (M)	(Ions score 53)
168 - 184	647.3098	1938.9075	1938.9034	2	0	K.YSHVDLVVMVDGFEGEK.G	Oxidation (M)	(Ions score 61)
168 - 184	647.3098	1938.9075	1938.9034	2	0	K.YSHVDLVVMVDGFEGEK.G	Oxidation (M)	(Ions score 60)
168 - 184	647.3098	1938.9075	1938.9034	2	0	K.YSHVDLVVMVDGFEGEK.G	Oxidation (M)	(Ions score 72)
168 - 184	647.3098	1938.9075	1938.9034	2	0	K.YSHVDLVVMVDGFEGEK.G	Oxidation (M)	(Ions score 51)
168 - 184	970.4610	1938.9075	1938.9034	2	0	K.YSHVDLVVMVDGFEGEK.G	Oxidation (M)	(Ions score 50)
168 - 184	647.3098	1938.9075	1938.9034	2	0	K.YSHVDLVVMVDGFEGEK.G	Oxidation (M)	(Ions score 61)
168 - 184	647.3098	1938.9075	1938.9034	2	0	K.YSHVDLVVMVDGFEGEK.G	Oxidation (M)	(Ions score 68)
168 - 184	647.3098	1938.9075	1938.9034	2	0	K.YSHVDLVVMVDGFEGEK.G	Oxidation (M)	(Ions score 51)
168 - 184	970.4611	1938.9076	1938.9034	2	0	K.YSHVDLVVMVDGFEGEK.G	Oxidation (M)	(Ions score 66)
168 - 184	970.4611	1938.9076	1938.9034	2	0	K.YSHVDLVVMVDGFEGEK.G	Oxidation (M)	(Ions score 54)
168 - 184	970.4611	1938.9076	1938.9034	2	0	K.YSHVDLVVMVDGFEGEK.G	Oxidation (M)	(Ions score 55)
168 - 184	647.3098	1938.9076	1938.9034	2	0	K.YSHVDLVVMVDGFEGEK.G	Oxidation (M)	(Ions score 61)
168 - 184	647.3098	1938.9076	1938.9034	2	0	K.YSHVDLVVMVDGFEGEK.G	Oxidation (M)	(Ions score 50)
168 - 184	647.3098	1938.9076	1938.9034	2	0	K.YSHVDLVVMVDGFEGEK.G	Oxidation (M)	(Ions score 56)
168 - 184	647.3098	1938.9076	1938.9034	2	0	K.YSHVDLVVMVDGFEGEK.G	Oxidation (M)	(Ions score 64)
168 - 184	970.4611	1938.9076	1938.9034	2	0	K.YSHVDLVVMVDGFEGEK.G	Oxidation (M)	(Ions score 52)
168 - 184	970.4611	1938.9076	1938.9034	2	0	K.YSHVDLVVMVDGFEGEK.G	Oxidation (M)	(Ions score 57)
168 - 184	647.3098	1938.9077	1938.9034	2	0	K.YSHVDLVVMVDGFEGEK.G	Oxidation (M)	(Ions score 51)
168 - 184	647.3098	1938.9077	1938.9034	2	0	K.YSHVDLVVMVDGFEGEK.G	Oxidation (M)	(Ions score 66)
168 - 184	970.4611	1938.9077	1938.9034	2	0	K.YSHVDLVVMVDGFEGEK.G	Oxidation (M)	(Ions score 58)
168 - 184	647.3098	1938.9077	1938.9034	2	0	K.YSHVDLVVMVDGFEGEK.G	Oxidation (M)	(Ions score 82)
168 - 184	970.4611	1938.9077	1938.9034	2	0	K.YSHVDLVVMVDGFEGEK.G	Oxidation (M)	(Ions score 73)
168 - 184	970.4611	1938.9077	1938.9034	2	0	K.YSHVDLVVMVDGFEGEK.G	Oxidation (M)	(Ions score 50)
168 - 184	647.3099	1938.9077	1938.9034	2	0	K.YSHVDLVVMVDGFEGEK.G	Oxidation (M)	(Ions score 58)
168 - 184	970.4611	1938.9077	1938.9034	2	0	K.YSHVDLVVMVDGFEGEK.G	Oxidation (M)	(Ions score 50)
168 - 184	647.3099	1938.9077	1938.9034	2	0	K.YSHVDLVVMVDGFEGEK.G	Oxidation (M)	(Ions score 66)
168 - 184	647.3099	1938.9077	1938.9034	2	0	K.YSHVDLVVMVDGFEGEK.G	Oxidation (M)	(Ions score 50)
168 - 184	970.4611	1938.9077	1938.9034	2	0	K.YSHVDLVVMVDGFEGEK.G	Oxidation (M)	(Ions score 56)
168 - 184	647.3099	1938.9077	1938.9034	2	0	K.YSHVDLVVMVDGFEGEK.G	Oxidation (M)	(Ions score 81)
168 - 184	970.4612	1938.9078	1938.9034	2	0	K.YSHVDLVVMVDGFEGEK.G	Oxidation (M)	(Ions score 70)
168 - 184	647.3099	1938.9078	1938.9034	2	0	K.YSHVDLVVMVDGFEGEK.G	Oxidation (M)	(Ions score 61)
168 - 184	970.4612	1938.9078	1938.9034	2	0	K.YSHVDLVVMVDGFEGEK.G	Oxidation (M)	(Ions score 57)
168 - 184	647.3099	1938.9078	1938.9034	2	0	K.YSHVDLVVMVDGFEGEK.G	Oxidation (M)	(Ions score 66)
168 - 184	647.3099	1938.9078	1938.9034	2	0	K.YSHVDLVVMVDGFEGEK.G	Oxidation (M)	(Ions score 64)
168 - 184	647.3099	1938.9078	1938.9034	2	0	K.YSHVDLVVMVDGFEGEK.G	Oxidation (M)	(Ions score 39)
168 - 184	647.3099	1938.9078	1938.9034	2	0	K.YSHVDLVVMVDGFEGEK.G	Oxidation (M)	(Ions score 58)
168 - 184	647.3099	1938.9078	1938.9034	2	0	K.YSHVDLVVMVDGFEGEK.G	Oxidation (M)	(Ions score 54)
168 - 184	647.3099	1938.9078	1938.9034	2	0	K.YSHVDLVVMVDGFEGEK.G	Oxidation (M)	(Ions score 58)
168 - 184	970.4612	1938.9078	1938.9034	2	0	K.YSHVDLVVMVDGFEGEK.G	Oxidation (M)	(Ions score 57)
168 - 184	970.4612	1938.9078	1938.9034	2	0	K.YSHVDLVVMVDGFEGEK.G	Oxidation (M)	(Ions score 41)
168 - 184	647.3099	1938.9078	1938.9034	2	0	K.YSHVDLVVMVDGFEGEK.G	Oxidation (M)	(Ions score 61)
168 - 184	970.4612	1938.9079	1938.9034	2	0	K.YSHVDLVVMVDGFEGEK.G	Oxidation (M)	(Ions score 74)
168 - 184	647.3099	1938.9079	1938.9034	2	0	K.YSHVDLVVMVDGFEGEK.G	Oxidation (M)	(Ions score 69)
168 - 184	970.4612	1938.9079	1938.9034	2	0	K.YSHVDLVVMVDGFEGEK.G	Oxidation (M)	(Ions score 56)
168 - 184	647.3099	1938.9079	1938.9034	2	0	K.YSHVDLVVMVDGFEGEK.G	Oxidation (M)	(Ions score 56)
168 - 184	647.3099	1938.9079	1938.9034	2	0	K.YSHVDLVVMVDGFEGEK.G	Oxidation (M)	(Ions score 51)
168 - 184	647.3099	1938.9079	1938.9034	2	0	K.YSHVDLVVMVDGFEGEK.G	Oxidation (M)	(Ions score 52)
168 - 184	647.3099	1938.9079	1938.9034	2	0	K.YSHVDLVVMVDGFEGEK.G	Oxidation (M)	(Ions score 53)
168 - 184	647.3099	1938.9079	1938.9034	2	0	K.YSHVDLVVMVDGFEGEK.G	Oxidation (M)	(Ions score 66)

168 - 184	970.4612	1938.9079	1938.9034	2	0	K.YSHVDLVVMVDGFEGEK.G	Oxidation (M)	(Ions score 58)
168 - 184	970.4612	1938.9079	1938.9034	2	0	K.YSHVDLVVMVDGFEGEK.G	Oxidation (M)	(Ions score 61)
168 - 184	970.4613	1938.9080	1938.9034	2	0	K.YSHVDLVVMVDGFEGEK.G	Oxidation (M)	(Ions score 47)
168 - 184	647.3099	1938.9080	1938.9034	2	0	K.YSHVDLVVMVDGFEGEK.G	Oxidation (M)	(Ions score 78)
168 - 184	647.3099	1938.9080	1938.9034	2	0	K.YSHVDLVVMVDGFEGEK.G	Oxidation (M)	(Ions score 49)
168 - 184	647.3099	1938.9080	1938.9034	2	0	K.YSHVDLVVMVDGFEGEK.G	Oxidation (M)	(Ions score 60)
168 - 184	970.4613	1938.9080	1938.9034	2	0	K.YSHVDLVVMVDGFEGEK.G	Oxidation (M)	(Ions score 71)
168 - 184	647.3099	1938.9080	1938.9034	2	0	K.YSHVDLVVMVDGFEGEK.G	Oxidation (M)	(Ions score 58)
168 - 184	647.3099	1938.9080	1938.9034	2	0	K.YSHVDLVVMVDGFEGEK.G	Oxidation (M)	(Ions score 78)
168 - 184	970.4613	1938.9080	1938.9034	2	0	K.YSHVDLVVMVDGFEGEK.G	Oxidation (M)	(Ions score 58)
168 - 184	970.4613	1938.9080	1938.9034	2	0	K.YSHVDLVVMVDGFEGEK.G	Oxidation (M)	(Ions score 57)
168 - 184	647.3099	1938.9080	1938.9034	2	0	K.YSHVDLVVMVDGFEGEK.G	Oxidation (M)	(Ions score 52)
168 - 184	647.3100	1938.9080	1938.9034	2	0	K.YSHVDLVVMVDGFEGEK.G	Oxidation (M)	(Ions score 56)
168 - 184	970.4613	1938.9080	1938.9034	2	0	K.YSHVDLVVMVDGFEGEK.G	Oxidation (M)	(Ions score 56)
168 - 184	647.3100	1938.9080	1938.9034	2	0	K.YSHVDLVVMVDGFEGEK.G	Oxidation (M)	(Ions score 64)
168 - 184	647.3100	1938.9080	1938.9034	2	0	K.YSHVDLVVMVDGFEGEK.G	Oxidation (M)	(Ions score 51)
168 - 184	970.4613	1938.9081	1938.9034	2	0	K.YSHVDLVVMVDGFEGEK.G	Oxidation (M)	(Ions score 49)
168 - 184	970.4613	1938.9081	1938.9034	2	0	K.YSHVDLVVMVDGFEGEK.G	Oxidation (M)	(Ions score 57)
168 - 184	647.3100	1938.9081	1938.9034	2	0	K.YSHVDLVVMVDGFEGEK.G	Oxidation (M)	(Ions score 51)
168 - 184	970.4613	1938.9081	1938.9034	2	0	K.YSHVDLVVMVDGFEGEK.G	Oxidation (M)	(Ions score 55)
168 - 184	647.3100	1938.9081	1938.9034	2	0	K.YSHVDLVVMVDGFEGEK.G	Oxidation (M)	(Ions score 74)
168 - 184	647.3100	1938.9081	1938.9034	2	0	K.YSHVDLVVMVDGFEGEK.G	Oxidation (M)	(Ions score 53)
168 - 184	647.3100	1938.9081	1938.9034	2	0	K.YSHVDLVVMVDGFEGEK.G	Oxidation (M)	(Ions score 51)
168 - 184	970.4613	1938.9081	1938.9034	2	0	K.YSHVDLVVMVDGFEGEK.G	Oxidation (M)	(Ions score 57)
168 - 184	647.3100	1938.9081	1938.9034	2	0	K.YSHVDLVVMVDGFEGEK.G	Oxidation (M)	(Ions score 55)
168 - 184	970.4613	1938.9081	1938.9034	2	0	K.YSHVDLVVMVDGFEGEK.G	Oxidation (M)	(Ions score 55)
168 - 184	970.4614	1938.9082	1938.9034	2	0	K.YSHVDLVVMVDGFEGEK.G	Oxidation (M)	(Ions score 62)
168 - 184	647.3100	1938.9082	1938.9034	2	0	K.YSHVDLVVMVDGFEGEK.G	Oxidation (M)	(Ions score 66)
168 - 184	647.3100	1938.9082	1938.9034	3	0	K.YSHVDLVVMVDGFEGEK.G	Oxidation (M)	(Ions score 56)
168 - 184	647.3100	1938.9082	1938.9034	3	0	K.YSHVDLVVMVDGFEGEK.G	Oxidation (M)	(Ions score 69)
168 - 184	647.3100	1938.9082	1938.9034	3	0	K.YSHVDLVVMVDGFEGEK.G	Oxidation (M)	(Ions score 56)
168 - 184	647.3100	1938.9083	1938.9034	3	0	K.YSHVDLVVMVDGFEGEK.G	Oxidation (M)	(Ions score 53)
168 - 184	647.3100	1938.9083	1938.9034	3	0	K.YSHVDLVVMVDGFEGEK.G	Oxidation (M)	(Ions score 56)
168 - 184	970.4614	1938.9083	1938.9034	3	0	K.YSHVDLVVMVDGFEGEK.G	Oxidation (M)	(Ions score 56)
168 - 184	647.3100	1938.9083	1938.9034	3	0	K.YSHVDLVVMVDGFEGEK.G	Oxidation (M)	(Ions score 51)
168 - 184	647.3100	1938.9083	1938.9034	3	0	K.YSHVDLVVMVDGFEGEK.G	Oxidation (M)	(Ions score 70)
168 - 184	970.4614	1938.9083	1938.9034	3	0	K.YSHVDLVVMVDGFEGEK.G	Oxidation (M)	(Ions score 63)
168 - 184	647.3100	1938.9083	1938.9034	3	0	K.YSHVDLVVMVDGFEGEK.G	Oxidation (M)	(Ions score 56)
168 - 184	970.4614	1938.9083	1938.9034	3	0	K.YSHVDLVVMVDGFEGEK.G	Oxidation (M)	(Ions score 60)
168 - 184	970.4614	1938.9083	1938.9034	3	0	K.YSHVDLVVMVDGFEGEK.G	Oxidation (M)	(Ions score 54)
168 - 184	647.3100	1938.9083	1938.9034	3	0	K.YSHVDLVVMVDGFEGEK.G	Oxidation (M)	(Ions score 58)
168 - 184	647.3101	1938.9083	1938.9034	3	0	K.YSHVDLVVMVDGFEGEK.G	Oxidation (M)	(Ions score 56)
168 - 184	647.3101	1938.9084	1938.9034	3	0	K.YSHVDLVVMVDGFEGEK.G	Oxidation (M)	(Ions score 66)
168 - 184	647.3101	1938.9084	1938.9034	3	0	K.YSHVDLVVMVDGFEGEK.G	Oxidation (M)	(Ions score 59)
168 - 184	970.4615	1938.9084	1938.9034	3	0	K.YSHVDLVVMVDGFEGEK.G	Oxidation (M)	(Ions score 47)
168 - 184	970.4615	1938.9085	1938.9034	3	0	K.YSHVDLVVMVDGFEGEK.G	Oxidation (M)	(Ions score 40)
168 - 184	970.4615	1938.9085	1938.9034	3	0	K.YSHVDLVVMVDGFEGEK.G	Oxidation (M)	(Ions score 60)
168 - 184	970.4615	1938.9085	1938.9034	3	0	K.YSHVDLVVMVDGFEGEK.G	Oxidation (M)	(Ions score 58)
168 - 184	970.4615	1938.9085	1938.9034	3	0	K.YSHVDLVVMVDGFEGEK.G	Oxidation (M)	(Ions score 54)
168 - 184	970.4615	1938.9085	1938.9034	3	0	K.YSHVDLVVMVDGFEGEK.G	Oxidation (M)	(Ions score 70)
168 - 184	647.3101	1938.9085	1938.9034	3	0	K.YSHVDLVVMVDGFEGEK.G	Oxidation (M)	(Ions score 58)
168 - 184	647.3101	1938.9085	1938.9034	3	0	K.YSHVDLVVMVDGFEGEK.G	Oxidation (M)	(Ions score 56)
168 - 184	647.3101	1938.9085	1938.9034	3	0	K.YSHVDLVVMVDGFEGEK.G	Oxidation (M)	(Ions score 58)
168 - 184	647.3101	1938.9085	1938.9034	3	0	K.YSHVDLVVMVDGFEGEK.G	Oxidation (M)	(Ions score 62)
168 - 184	970.4615	1938.9085	1938.9034	3	0	K.YSHVDLVVMVDGFEGEK.G	Oxidation (M)	(Ions score 72)
168 - 184	970.4615	1938.9085	1938.9034	3	0	K.YSHVDLVVMVDGFEGEK.G	Oxidation (M)	(Ions score 58)
168 - 184	970.4616	1938.9086	1938.9034	3	0	K.YSHVDLVVMVDGFEGEK.G	Oxidation (M)	(Ions score 50)
168 - 184	647.3101	1938.9086	1938.9034	3	0	K.YSHVDLVVMVDGFEGEK.G	Oxidation (M)	(Ions score 56)

168 - 184	647.3101	1938.9086	1938.9034	3	0	K.YSHVDLVVMVDGFEGEK.G	Oxidation (M)	(Ions score 58)
168 - 184	647.3102	1938.9086	1938.9034	3	0	K.YSHVDLVVMVDGFEGEK.G	Oxidation (M)	(Ions score 64)
168 - 184	970.4616	1938.9086	1938.9034	3	0	K.YSHVDLVVMVDGFEGEK.G	Oxidation (M)	(Ions score 49)
168 - 184	647.3102	1938.9087	1938.9034	3	0	K.YSHVDLVVMVDGFEGEK.G	Oxidation (M)	(Ions score 61)
168 - 184	647.3102	1938.9087	1938.9034	3	0	K.YSHVDLVVMVDGFEGEK.G	Oxidation (M)	(Ions score 61)
168 - 184	970.4616	1938.9087	1938.9034	3	0	K.YSHVDLVVMVDGFEGEK.G	Oxidation (M)	(Ions score 43)
168 - 184	647.3102	1938.9087	1938.9034	3	0	K.YSHVDLVVMVDGFEGEK.G	Oxidation (M)	(Ions score 56)
168 - 184	647.3102	1938.9087	1938.9034	3	0	K.YSHVDLVVMVDGFEGEK.G	Oxidation (M)	(Ions score 66)
168 - 184	647.3102	1938.9087	1938.9034	3	0	K.YSHVDLVVMVDGFEGEK.G	Oxidation (M)	(Ions score 58)
168 - 184	647.3102	1938.9087	1938.9034	3	0	K.YSHVDLVVMVDGFEGEK.G	Oxidation (M)	(Ions score 63)
168 - 184	970.4617	1938.9088	1938.9034	3	0	K.YSHVDLVVMVDGFEGEK.G	Oxidation (M)	(Ions score 53)
168 - 184	647.3102	1938.9088	1938.9034	3	0	K.YSHVDLVVMVDGFEGEK.G	Oxidation (M)	(Ions score 60)
168 - 184	647.3102	1938.9088	1938.9034	3	0	K.YSHVDLVVMVDGFEGEK.G	Oxidation (M)	(Ions score 50)
168 - 184	647.3102	1938.9088	1938.9034	3	0	K.YSHVDLVVMVDGFEGEK.G	Oxidation (M)	(Ions score 56)
168 - 184	970.4617	1938.9088	1938.9034	3	0	K.YSHVDLVVMVDGFEGEK.G	Oxidation (M)	(Ions score 74)
168 - 184	970.4617	1938.9088	1938.9034	3	0	K.YSHVDLVVMVDGFEGEK.G	Oxidation (M)	(Ions score 57)
168 - 184	970.4617	1938.9088	1938.9034	3	0	K.YSHVDLVVMVDGFEGEK.G	Oxidation (M)	(Ions score 54)
168 - 184	970.4617	1938.9088	1938.9034	3	0	K.YSHVDLVVMVDGFEGEK.G	Oxidation (M)	(Ions score 57)
168 - 184	970.4617	1938.9088	1938.9034	3	0	K.YSHVDLVVMVDGFEGEK.G	Oxidation (M)	(Ions score 50)
168 - 184	970.4617	1938.9089	1938.9034	3	0	K.YSHVDLVVMVDGFEGEK.G	Oxidation (M)	(Ions score 49)
168 - 184	647.3102	1938.9089	1938.9034	3	0	K.YSHVDLVVMVDGFEGEK.G	Oxidation (M)	(Ions score 66)
168 - 184	647.3102	1938.9089	1938.9034	3	0	K.YSHVDLVVMVDGFEGEK.G	Oxidation (M)	(Ions score 49)
168 - 184	970.4617	1938.9089	1938.9034	3	0	K.YSHVDLVVMVDGFEGEK.G	Oxidation (M)	(Ions score 65)
168 - 184	647.3102	1938.9089	1938.9034	3	0	K.YSHVDLVVMVDGFEGEK.G	Oxidation (M)	(Ions score 63)
168 - 184	970.4617	1938.9089	1938.9034	3	0	K.YSHVDLVVMVDGFEGEK.G	Oxidation (M)	(Ions score 56)
168 - 184	647.3102	1938.9089	1938.9034	3	0	K.YSHVDLVVMVDGFEGEK.G	Oxidation (M)	(Ions score 78)
168 - 184	647.3103	1938.9089	1938.9034	3	0	K.YSHVDLVVMVDGFEGEK.G	Oxidation (M)	(Ions score 66)
168 - 184	970.4617	1938.9089	1938.9034	3	0	K.YSHVDLVVMVDGFEGEK.G	Oxidation (M)	(Ions score 73)
168 - 184	647.3103	1938.9090	1938.9034	3	0	K.YSHVDLVVMVDGFEGEK.G	Oxidation (M)	(Ions score 49)
168 - 184	970.4618	1938.9090	1938.9034	3	0	K.YSHVDLVVMVDGFEGEK.G	Oxidation (M)	(Ions score 56)
168 - 184	647.3103	1938.9090	1938.9034	3	0	K.YSHVDLVVMVDGFEGEK.G	Oxidation (M)	(Ions score 55)
168 - 184	970.4618	1938.9090	1938.9034	3	0	K.YSHVDLVVMVDGFEGEK.G	Oxidation (M)	(Ions score 51)
168 - 184	647.3103	1938.9090	1938.9034	3	0	K.YSHVDLVVMVDGFEGEK.G	Oxidation (M)	(Ions score 58)
168 - 184	970.4618	1938.9090	1938.9034	3	0	K.YSHVDLVVMVDGFEGEK.G	Oxidation (M)	(Ions score 64)
168 - 184	970.4618	1938.9090	1938.9034	3	0	K.YSHVDLVVMVDGFEGEK.G	Oxidation (M)	(Ions score 55)
168 - 184	647.3103	1938.9090	1938.9034	3	0	K.YSHVDLVVMVDGFEGEK.G	Oxidation (M)	(Ions score 58)
168 - 184	647.3103	1938.9091	1938.9034	3	0	K.YSHVDLVVMVDGFEGEK.G	Oxidation (M)	(Ions score 61)
168 - 184	970.4618	1938.9091	1938.9034	3	0	K.YSHVDLVVMVDGFEGEK.G	Oxidation (M)	(Ions score 55)
168 - 184	970.4618	1938.9091	1938.9034	3	0	K.YSHVDLVVMVDGFEGEK.G	Oxidation (M)	(Ions score 59)
168 - 184	647.3103	1938.9091	1938.9034	3	0	K.YSHVDLVVMVDGFEGEK.G	Oxidation (M)	(Ions score 56)
168 - 184	647.3103	1938.9091	1938.9034	3	0	K.YSHVDLVVMVDGFEGEK.G	Oxidation (M)	(Ions score 55)
168 - 184	970.4618	1938.9091	1938.9034	3	0	K.YSHVDLVVMVDGFEGEK.G	Oxidation (M)	(Ions score 68)
168 - 184	647.3103	1938.9091	1938.9034	3	0	K.YSHVDLVVMVDGFEGEK.G	Oxidation (M)	(Ions score 56)
168 - 184	970.4619	1938.9092	1938.9034	3	0	K.YSHVDLVVMVDGFEGEK.G	Oxidation (M)	(Ions score 67)
168 - 184	647.3103	1938.9092	1938.9034	3	0	K.YSHVDLVVMVDGFEGEK.G	Oxidation (M)	(Ions score 46)
168 - 184	647.3104	1938.9092	1938.9034	3	0	K.YSHVDLVVMVDGFEGEK.G	Oxidation (M)	(Ions score 61)
168 - 184	970.4619	1938.9092	1938.9034	3	0	K.YSHVDLVVMVDGFEGEK.G	Oxidation (M)	(Ions score 55)
168 - 184	647.3104	1938.9092	1938.9034	3	0	K.YSHVDLVVMVDGFEGEK.G	Oxidation (M)	(Ions score 59)
168 - 184	970.4619	1938.9093	1938.9034	3	0	K.YSHVDLVVMVDGFEGEK.G	Oxidation (M)	(Ions score 67)
168 - 184	647.3104	1938.9093	1938.9034	3	0	K.YSHVDLVVMVDGFEGEK.G	Oxidation (M)	(Ions score 56)
168 - 184	970.4619	1938.9093	1938.9034	3	0	K.YSHVDLVVMVDGFEGEK.G	Oxidation (M)	(Ions score 52)
168 - 184	647.3104	1938.9093	1938.9034	3	0	K.YSHVDLVVMVDGFEGEK.G	Oxidation (M)	(Ions score 66)
168 - 184	647.3104	1938.9093	1938.9034	3	0	K.YSHVDLVVMVDGFEGEK.G	Oxidation (M)	(Ions score 66)
168 - 184	647.3104	1938.9093	1938.9034	3	0	K.YSHVDLVVMVDGFEGEK.G	Oxidation (M)	(Ions score 61)
168 - 184	647.3104	1938.9094	1938.9034	3	0	K.YSHVDLVVMVDGFEGEK.G	Oxidation (M)	(Ions score 54)
168 - 184	647.3104	1938.9094	1938.9034	3	0	K.YSHVDLVVMVDGFEGEK.G	Oxidation (M)	(Ions score 56)
168 - 184	647.3104	1938.9094	1938.9034	3	0	K.YSHVDLVVMVDGFEGEK.G	Oxidation (M)	(Ions score 78)
168 - 184	970.4620	1938.9094	1938.9034	3	0	K.YSHVDLVVMVDGFEGEK.G	Oxidation (M)	(Ions score 74)

168 - 184	970.4620	1938.9094	1938.9034	3	0	K.YSHVDLVVMVDGFEGEK.G	Oxidation (M)	(Ions score 57)
168 - 184	647.3104	1938.9094	1938.9034	3	0	K.YSHVDLVVMVDGFEGEK.G	Oxidation (M)	(Ions score 69)
168 - 184	970.4620	1938.9095	1938.9034	3	0	K.YSHVDLVVMVDGFEGEK.G	Oxidation (M)	(Ions score 58)
168 - 184	647.3104	1938.9095	1938.9034	3	0	K.YSHVDLVVMVDGFEGEK.G	Oxidation (M)	(Ions score 56)
168 - 184	647.3104	1938.9095	1938.9034	3	0	K.YSHVDLVVMVDGFEGEK.G	Oxidation (M)	(Ions score 56)
168 - 184	970.4620	1938.9095	1938.9034	3	0	K.YSHVDLVVMVDGFEGEK.G	Oxidation (M)	(Ions score 58)
168 - 184	970.4620	1938.9095	1938.9034	3	0	K.YSHVDLVVMVDGFEGEK.G	Oxidation (M)	(Ions score 62)
168 - 184	647.3104	1938.9095	1938.9034	3	0	K.YSHVDLVVMVDGFEGEK.G	Oxidation (M)	(Ions score 56)
168 - 184	647.3104	1938.9095	1938.9034	3	0	K.YSHVDLVVMVDGFEGEK.G	Oxidation (M)	(Ions score 59)
168 - 184	970.4620	1938.9095	1938.9034	3	0	K.YSHVDLVVMVDGFEGEK.G	Oxidation (M)	(Ions score 60)
168 - 184	970.4620	1938.9095	1938.9034	3	0	K.YSHVDLVVMVDGFEGEK.G	Oxidation (M)	(Ions score 53)
168 - 184	647.3105	1938.9095	1938.9034	3	0	K.YSHVDLVVMVDGFEGEK.G	Oxidation (M)	(Ions score 63)
168 - 184	647.3105	1938.9095	1938.9034	3	0	K.YSHVDLVVMVDGFEGEK.G	Oxidation (M)	(Ions score 66)
168 - 184	647.3105	1938.9096	1938.9034	3	0	K.YSHVDLVVMVDGFEGEK.G	Oxidation (M)	(Ions score 64)
168 - 184	970.4621	1938.9096	1938.9034	3	0	K.YSHVDLVVMVDGFEGEK.G	Oxidation (M)	(Ions score 56)
168 - 184	970.4621	1938.9096	1938.9034	3	0	K.YSHVDLVVMVDGFEGEK.G	Oxidation (M)	(Ions score 47)
168 - 184	647.3105	1938.9096	1938.9034	3	0	K.YSHVDLVVMVDGFEGEK.G	Oxidation (M)	(Ions score 49)
168 - 184	970.4621	1938.9096	1938.9034	3	0	K.YSHVDLVVMVDGFEGEK.G	Oxidation (M)	(Ions score 57)
168 - 184	647.3105	1938.9096	1938.9034	3	0	K.YSHVDLVVMVDGFEGEK.G	Oxidation (M)	(Ions score 61)
168 - 184	647.3105	1938.9096	1938.9034	3	0	K.YSHVDLVVMVDGFEGEK.G	Oxidation (M)	(Ions score 64)
168 - 184	647.3105	1938.9096	1938.9034	3	0	K.YSHVDLVVMVDGFEGEK.G	Oxidation (M)	(Ions score 58)
168 - 184	647.3105	1938.9096	1938.9034	3	0	K.YSHVDLVVMVDGFEGEK.G	Oxidation (M)	(Ions score 51)
168 - 184	970.4621	1938.9096	1938.9034	3	0	K.YSHVDLVVMVDGFEGEK.G	Oxidation (M)	(Ions score 57)
168 - 184	970.4621	1938.9096	1938.9034	3	0	K.YSHVDLVVMVDGFEGEK.G	Oxidation (M)	(Ions score 59)
168 - 184	647.3105	1938.9096	1938.9034	3	0	K.YSHVDLVVMVDGFEGEK.G	Oxidation (M)	(Ions score 69)
168 - 184	970.4621	1938.9096	1938.9034	3	0	K.YSHVDLVVMVDGFEGEK.G	Oxidation (M)	(Ions score 63)
168 - 184	970.4621	1938.9096	1938.9034	3	0	K.YSHVDLVVMVDGFEGEK.G	Oxidation (M)	(Ions score 52)
168 - 184	970.4621	1938.9097	1938.9034	3	0	K.YSHVDLVVMVDGFEGEK.G	Oxidation (M)	(Ions score 54)
168 - 184	970.4621	1938.9097	1938.9034	3	0	K.YSHVDLVVMVDGFEGEK.G	Oxidation (M)	(Ions score 70)
168 - 184	970.4621	1938.9097	1938.9034	3	0	K.YSHVDLVVMVDGFEGEK.G	Oxidation (M)	(Ions score 63)
168 - 184	970.4621	1938.9097	1938.9034	3	0	K.YSHVDLVVMVDGFEGEK.G	Oxidation (M)	(Ions score 58)
168 - 184	970.4621	1938.9097	1938.9034	3	0	K.YSHVDLVVMVDGFEGEK.G	Oxidation (M)	(Ions score 57)
168 - 184	970.4621	1938.9097	1938.9034	3	0	K.YSHVDLVVMVDGFEGEK.G	Oxidation (M)	(Ions score 49)
168 - 184	970.4621	1938.9097	1938.9034	3	0	K.YSHVDLVVMVDGFEGEK.G	Oxidation (M)	(Ions score 54)
168 - 184	647.3105	1938.9097	1938.9034	3	0	K.YSHVDLVVMVDGFEGEK.G	Oxidation (M)	(Ions score 58)
168 - 184	970.4621	1938.9097	1938.9034	3	0	K.YSHVDLVVMVDGFEGEK.G	Oxidation (M)	(Ions score 67)
168 - 184	647.3105	1938.9097	1938.9034	3	0	K.YSHVDLVVMVDGFEGEK.G	Oxidation (M)	(Ions score 69)
168 - 184	647.3105	1938.9097	1938.9034	3	0	K.YSHVDLVVMVDGFEGEK.G	Oxidation (M)	(Ions score 56)
168 - 184	647.3105	1938.9098	1938.9034	3	0	K.YSHVDLVVMVDGFEGEK.G	Oxidation (M)	(Ions score 64)
168 - 184	647.3105	1938.9098	1938.9034	3	0	K.YSHVDLVVMVDGFEGEK.G	Oxidation (M)	(Ions score 75)
168 - 184	970.4622	1938.9098	1938.9034	3	0	K.YSHVDLVVMVDGFEGEK.G	Oxidation (M)	(Ions score 61)
168 - 184	970.4622	1938.9098	1938.9034	3	0	K.YSHVDLVVMVDGFEGEK.G	Oxidation (M)	(Ions score 44)
168 - 184	970.4622	1938.9098	1938.9034	3	0	K.YSHVDLVVMVDGFEGEK.G	Oxidation (M)	(Ions score 57)
168 - 184	970.4622	1938.9098	1938.9034	3	0	K.YSHVDLVVMVDGFEGEK.G	Oxidation (M)	(Ions score 45)
168 - 184	647.3105	1938.9098	1938.9034	3	0	K.YSHVDLVVMVDGFEGEK.G	Oxidation (M)	(Ions score 69)
168 - 184	970.4622	1938.9098	1938.9034	3	0	K.YSHVDLVVMVDGFEGEK.G	Oxidation (M)	(Ions score 47)
168 - 184	970.4622	1938.9098	1938.9034	3	0	K.YSHVDLVVMVDGFEGEK.G	Oxidation (M)	(Ions score 58)
168 - 184	970.4622	1938.9099	1938.9034	3	0	K.YSHVDLVVMVDGFEGEK.G	Oxidation (M)	(Ions score 52)
168 - 184	647.3106	1938.9099	1938.9034	3	0	K.YSHVDLVVMVDGFEGEK.G	Oxidation (M)	(Ions score 58)
168 - 184	970.4622	1938.9099	1938.9034	3	0	K.YSHVDLVVMVDGFEGEK.G	Oxidation (M)	(Ions score 59)
168 - 184	970.4622	1938.9099	1938.9034	3	0	K.YSHVDLVVMVDGFEGEK.G	Oxidation (M)	(Ions score 50)
168 - 184	647.3106	1938.9099	1938.9034	3	0	K.YSHVDLVVMVDGFEGEK.G	Oxidation (M)	(Ions score 56)
168 - 184	970.4622	1938.9099	1938.9034	3	0	K.YSHVDLVVMVDGFEGEK.G	Oxidation (M)	(Ions score 57)
168 - 184	970.4622	1938.9099	1938.9034	3	0	K.YSHVDLVVMVDGFEGEK.G	Oxidation (M)	(Ions score 76)
168 - 184	647.3106	1938.9099	1938.9034	3	0	K.YSHVDLVVMVDGFEGEK.G	Oxidation (M)	(Ions score 66)
168 - 184	970.4623	1938.9100	1938.9034	3	0	K.YSHVDLVVMVDGFEGEK.G	Oxidation (M)	(Ions score 62)
168 - 184	647.3106	1938.9100	1938.9034	3	0	K.YSHVDLVVMVDGFEGEK.G	Oxidation (M)	(Ions score 56)
168 - 184	970.4623	1938.9100	1938.9034	3	0	K.YSHVDLVVMVDGFEGEK.G	Oxidation (M)	(Ions score 61)

168 - 184	647.3106	1938.9100	1938.9034	3	0	K.YSHVDLVVMVDGFEGEK.G	Oxidation (M)	(Ions score 66)
168 - 184	970.4623	1938.9100	1938.9034	3	0	K.YSHVDLVVMVDGFEGEK.G	Oxidation (M)	(Ions score 63)
168 - 184	970.4623	1938.9100	1938.9034	3	0	K.YSHVDLVVMVDGFEGEK.G	Oxidation (M)	(Ions score 55)
168 - 184	647.3106	1938.9100	1938.9034	3	0	K.YSHVDLVVMVDGFEGEK.G	Oxidation (M)	(Ions score 69)
168 - 184	970.4623	1938.9100	1938.9034	3	0	K.YSHVDLVVMVDGFEGEK.G	Oxidation (M)	(Ions score 56)
168 - 184	970.4623	1938.9101	1938.9034	3	0	K.YSHVDLVVMVDGFEGEK.G	Oxidation (M)	(Ions score 55)
168 - 184	970.4623	1938.9101	1938.9034	3	0	K.YSHVDLVVMVDGFEGEK.G	Oxidation (M)	(Ions score 51)
168 - 184	970.4623	1938.9101	1938.9034	3	0	K.YSHVDLVVMVDGFEGEK.G	Oxidation (M)	(Ions score 52)
168 - 184	647.3106	1938.9101	1938.9034	3	0	K.YSHVDLVVMVDGFEGEK.G	Oxidation (M)	(Ions score 60)
168 - 184	647.3106	1938.9101	1938.9034	3	0	K.YSHVDLVVMVDGFEGEK.G	Oxidation (M)	(Ions score 69)
168 - 184	647.3106	1938.9101	1938.9034	3	0	K.YSHVDLVVMVDGFEGEK.G	Oxidation (M)	(Ions score 38)
168 - 184	970.4623	1938.9101	1938.9034	3	0	K.YSHVDLVVMVDGFEGEK.G	Oxidation (M)	(Ions score 72)
168 - 184	647.3107	1938.9101	1938.9034	4	0	K.YSHVDLVVMVDGFEGEK.G	Oxidation (M)	(Ions score 58)
168 - 184	970.4624	1938.9102	1938.9034	4	0	K.YSHVDLVVMVDGFEGEK.G	Oxidation (M)	(Ions score 78)
168 - 184	647.3107	1938.9102	1938.9034	4	0	K.YSHVDLVVMVDGFEGEK.G	Oxidation (M)	(Ions score 52)
168 - 184	647.3107	1938.9102	1938.9034	4	0	K.YSHVDLVVMVDGFEGEK.G	Oxidation (M)	(Ions score 72)
168 - 184	970.4624	1938.9102	1938.9034	4	0	K.YSHVDLVVMVDGFEGEK.G	Oxidation (M)	(Ions score 56)
168 - 184	970.4624	1938.9102	1938.9034	4	0	K.YSHVDLVVMVDGFEGEK.G	Oxidation (M)	(Ions score 59)
168 - 184	970.4624	1938.9102	1938.9034	4	0	K.YSHVDLVVMVDGFEGEK.G	Oxidation (M)	(Ions score 72)
168 - 184	647.3107	1938.9102	1938.9034	4	0	K.YSHVDLVVMVDGFEGEK.G	Oxidation (M)	(Ions score 58)
168 - 184	970.4624	1938.9102	1938.9034	4	0	K.YSHVDLVVMVDGFEGEK.G	Oxidation (M)	(Ions score 57)
168 - 184	647.3107	1938.9103	1938.9034	4	0	K.YSHVDLVVMVDGFEGEK.G	Oxidation (M)	(Ions score 66)
168 - 184	970.4624	1938.9103	1938.9034	4	0	K.YSHVDLVVMVDGFEGEK.G	Oxidation (M)	(Ions score 60)
168 - 184	970.4624	1938.9103	1938.9034	4	0	K.YSHVDLVVMVDGFEGEK.G	Oxidation (M)	(Ions score 56)
168 - 184	970.4625	1938.9104	1938.9034	4	0	K.YSHVDLVVMVDGFEGEK.G	Oxidation (M)	(Ions score 54)
168 - 184	647.3107	1938.9104	1938.9034	4	0	K.YSHVDLVVMVDGFEGEK.G	Oxidation (M)	(Ions score 61)
168 - 184	970.4625	1938.9104	1938.9034	4	0	K.YSHVDLVVMVDGFEGEK.G	Oxidation (M)	(Ions score 50)
168 - 184	647.3108	1938.9105	1938.9034	4	0	K.YSHVDLVVMVDGFEGEK.G	Oxidation (M)	(Ions score 63)
168 - 184	970.4625	1938.9105	1938.9034	4	0	K.YSHVDLVVMVDGFEGEK.G	Oxidation (M)	(Ions score 55)
168 - 184	970.4625	1938.9105	1938.9034	4	0	K.YSHVDLVVMVDGFEGEK.G	Oxidation (M)	(Ions score 68)
168 - 184	647.3108	1938.9105	1938.9034	4	0	K.YSHVDLVVMVDGFEGEK.G	Oxidation (M)	(Ions score 56)
168 - 184	647.3108	1938.9105	1938.9034	4	0	K.YSHVDLVVMVDGFEGEK.G	Oxidation (M)	(Ions score 36)
168 - 184	970.4625	1938.9105	1938.9034	4	0	K.YSHVDLVVMVDGFEGEK.G	Oxidation (M)	(Ions score 56)
168 - 184	647.3108	1938.9106	1938.9034	4	0	K.YSHVDLVVMVDGFEGEK.G	Oxidation (M)	(Ions score 69)
168 - 184	970.4626	1938.9106	1938.9034	4	0	K.YSHVDLVVMVDGFEGEK.G	Oxidation (M)	(Ions score 61)
168 - 184	970.4626	1938.9106	1938.9034	4	0	K.YSHVDLVVMVDGFEGEK.G	Oxidation (M)	(Ions score 60)
168 - 184	647.3108	1938.9106	1938.9034	4	0	K.YSHVDLVVMVDGFEGEK.G	Oxidation (M)	(Ions score 61)
168 - 184	970.4626	1938.9107	1938.9034	4	0	K.YSHVDLVVMVDGFEGEK.G	Oxidation (M)	(Ions score 55)
168 - 184	970.4626	1938.9107	1938.9034	4	0	K.YSHVDLVVMVDGFEGEK.G	Oxidation (M)	(Ions score 46)
168 - 184	647.3108	1938.9107	1938.9034	4	0	K.YSHVDLVVMVDGFEGEK.G	Oxidation (M)	(Ions score 58)
168 - 184	647.3109	1938.9107	1938.9034	4	0	K.YSHVDLVVMVDGFEGEK.G	Oxidation (M)	(Ions score 58)
168 - 184	647.3109	1938.9108	1938.9034	4	0	K.YSHVDLVVMVDGFEGEK.G	Oxidation (M)	(Ions score 66)
168 - 184	647.3109	1938.9108	1938.9034	4	0	K.YSHVDLVVMVDGFEGEK.G	Oxidation (M)	(Ions score 66)
168 - 184	970.4627	1938.9108	1938.9034	4	0	K.YSHVDLVVMVDGFEGEK.G	Oxidation (M)	(Ions score 57)
168 - 184	647.3109	1938.9108	1938.9034	4	0	K.YSHVDLVVMVDGFEGEK.G	Oxidation (M)	(Ions score 54)
168 - 184	970.4627	1938.9108	1938.9034	4	0	K.YSHVDLVVMVDGFEGEK.G	Oxidation (M)	(Ions score 50)
168 - 184	970.4627	1938.9109	1938.9034	4	0	K.YSHVDLVVMVDGFEGEK.G	Oxidation (M)	(Ions score 57)
168 - 184	647.3109	1938.9109	1938.9034	4	0	K.YSHVDLVVMVDGFEGEK.G	Oxidation (M)	(Ions score 62)
168 - 184	970.4627	1938.9109	1938.9034	4	0	K.YSHVDLVVMVDGFEGEK.G	Oxidation (M)	(Ions score 69)
168 - 184	970.4627	1938.9109	1938.9034	4	0	K.YSHVDLVVMVDGFEGEK.G	Oxidation (M)	(Ions score 65)
168 - 184	647.3109	1938.9109	1938.9034	4	0	K.YSHVDLVVMVDGFEGEK.G	Oxidation (M)	(Ions score 55)
168 - 184	647.3109	1938.9109	1938.9034	4	0	K.YSHVDLVVMVDGFEGEK.G	Oxidation (M)	(Ions score 58)
168 - 184	970.4627	1938.9109	1938.9034	4	0	K.YSHVDLVVMVDGFEGEK.G	Oxidation (M)	(Ions score 58)
168 - 184	647.3109	1938.9110	1938.9034	4	0	K.YSHVDLVVMVDGFEGEK.G	Oxidation (M)	(Ions score 54)
168 - 184	647.3109	1938.9110	1938.9034	4	0	K.YSHVDLVVMVDGFEGEK.G	Oxidation (M)	(Ions score 53)
168 - 184	647.3110	1938.9111	1938.9034	4	0	K.YSHVDLVVMVDGFEGEK.G	Oxidation (M)	(Ions score 59)
168 - 184	970.4628	1938.9111	1938.9034	4	0	K.YSHVDLVVMVDGFEGEK.G	Oxidation (M)	(Ions score 61)
168 - 184	970.4628	1938.9111	1938.9034	4	0	K.YSHVDLVVMVDGFEGEK.G	Oxidation (M)	(Ions score 56)

168 - 184	970.4629	1938.9112	1938.9034	4	0	K.YSHVDLVVMVDGFEGEK.G	Oxidation (M)	(Ions score 60)
168 - 184	647.3110	1938.9112	1938.9034	4	0	K.YSHVDLVVMVDGFEGEK.G	Oxidation (M)	(Ions score 49)
168 - 184	647.3110	1938.9112	1938.9034	4	0	K.YSHVDLVVMVDGFEGEK.G	Oxidation (M)	(Ions score 58)
168 - 184	647.3110	1938.9112	1938.9034	4	0	K.YSHVDLVVMVDGFEGEK.G	Oxidation (M)	(Ions score 58)
168 - 184	647.3110	1938.9113	1938.9034	4	0	K.YSHVDLVVMVDGFEGEK.G	Oxidation (M)	(Ions score 62)
168 - 184	970.4629	1938.9113	1938.9034	4	0	K.YSHVDLVVMVDGFEGEK.G	Oxidation (M)	(Ions score 54)
168 - 184	970.4629	1938.9113	1938.9034	4	0	K.YSHVDLVVMVDGFEGEK.G	Oxidation (M)	(Ions score 51)
168 - 184	970.4629	1938.9113	1938.9034	4	0	K.YSHVDLVVMVDGFEGEK.G	Oxidation (M)	(Ions score 56)
168 - 184	647.3111	1938.9114	1938.9034	4	0	K.YSHVDLVVMVDGFEGEK.G	Oxidation (M)	(Ions score 53)
168 - 184	970.4630	1938.9114	1938.9034	4	0	K.YSHVDLVVMVDGFEGEK.G	Oxidation (M)	(Ions score 49)
168 - 184	647.3111	1938.9114	1938.9034	4	0	K.YSHVDLVVMVDGFEGEK.G	Oxidation (M)	(Ions score 58)
168 - 184	647.3111	1938.9114	1938.9034	4	0	K.YSHVDLVVMVDGFEGEK.G	Oxidation (M)	(Ions score 56)
168 - 184	970.4630	1938.9114	1938.9034	4	0	K.YSHVDLVVMVDGFEGEK.G	Oxidation (M)	(Ions score 52)
168 - 184	970.4630	1938.9114	1938.9034	4	0	K.YSHVDLVVMVDGFEGEK.G	Oxidation (M)	(Ions score 51)
168 - 184	647.3111	1938.9114	1938.9034	4	0	K.YSHVDLVVMVDGFEGEK.G	Oxidation (M)	(Ions score 66)
168 - 184	970.4630	1938.9115	1938.9034	4	0	K.YSHVDLVVMVDGFEGEK.G	Oxidation (M)	(Ions score 67)
168 - 184	647.3111	1938.9115	1938.9034	4	0	K.YSHVDLVVMVDGFEGEK.G	Oxidation (M)	(Ions score 56)
168 - 184	647.3111	1938.9115	1938.9034	4	0	K.YSHVDLVVMVDGFEGEK.G	Oxidation (M)	(Ions score 50)
168 - 184	970.4631	1938.9116	1938.9034	4	0	K.YSHVDLVVMVDGFEGEK.G	Oxidation (M)	(Ions score 59)
168 - 184	970.4631	1938.9116	1938.9034	4	0	K.YSHVDLVVMVDGFEGEK.G	Oxidation (M)	(Ions score 48)
168 - 184	970.4631	1938.9116	1938.9034	4	0	K.YSHVDLVVMVDGFEGEK.G	Oxidation (M)	(Ions score 52)
168 - 184	647.3112	1938.9116	1938.9034	4	0	K.YSHVDLVVMVDGFEGEK.G	Oxidation (M)	(Ions score 53)
168 - 184	970.4631	1938.9116	1938.9034	4	0	K.YSHVDLVVMVDGFEGEK.G	Oxidation (M)	(Ions score 58)
168 - 184	970.4631	1938.9116	1938.9034	4	0	K.YSHVDLVVMVDGFEGEK.G	Oxidation (M)	(Ions score 65)
168 - 184	970.4631	1938.9117	1938.9034	4	0	K.YSHVDLVVMVDGFEGEK.G	Oxidation (M)	(Ions score 54)
168 - 184	970.4631	1938.9117	1938.9034	4	0	K.YSHVDLVVMVDGFEGEK.G	Oxidation (M)	(Ions score 49)
168 - 184	647.3112	1938.9117	1938.9034	4	0	K.YSHVDLVVMVDGFEGEK.G	Oxidation (M)	(Ions score 50)
168 - 184	970.4632	1938.9118	1938.9034	4	0	K.YSHVDLVVMVDGFEGEK.G	Oxidation (M)	(Ions score 57)
168 - 184	970.4632	1938.9118	1938.9034	4	0	K.YSHVDLVVMVDGFEGEK.G	Oxidation (M)	(Ions score 56)
168 - 184	647.3112	1938.9118	1938.9034	4	0	K.YSHVDLVVMVDGFEGEK.G	Oxidation (M)	(Ions score 56)
168 - 184	970.4632	1938.9119	1938.9034	4	0	K.YSHVDLVVMVDGFEGEK.G	Oxidation (M)	(Ions score 60)
168 - 184	647.3113	1938.9120	1938.9034	4	0	K.YSHVDLVVMVDGFEGEK.G	Oxidation (M)	(Ions score 32)
168 - 184	970.4633	1938.9121	1938.9034	4	0	K.YSHVDLVVMVDGFEGEK.G	Oxidation (M)	(Ions score 67)
168 - 184	970.4633	1938.9121	1938.9034	4	0	K.YSHVDLVVMVDGFEGEK.G	Oxidation (M)	(Ions score 56)
168 - 184	647.3113	1938.9121	1938.9034	5	0	K.YSHVDLVVMVDGFEGEK.G	Oxidation (M)	(Ions score 58)
168 - 184	970.4633	1938.9121	1938.9034	5	0	K.YSHVDLVVMVDGFEGEK.G	Oxidation (M)	(Ions score 56)
168 - 184	970.4634	1938.9122	1938.9034	5	0	K.YSHVDLVVMVDGFEGEK.G	Oxidation (M)	(Ions score 60)
168 - 184	970.4634	1938.9122	1938.9034	5	0	K.YSHVDLVVMVDGFEGEK.G	Oxidation (M)	(Ions score 54)
168 - 184	647.3113	1938.9122	1938.9034	5	0	K.YSHVDLVVMVDGFEGEK.G	Oxidation (M)	(Ions score 61)
168 - 184	647.3113	1938.9122	1938.9034	5	0	K.YSHVDLVVMVDGFEGEK.G	Oxidation (M)	(Ions score 55)
168 - 184	647.3114	1938.9122	1938.9034	5	0	K.YSHVDLVVMVDGFEGEK.G	Oxidation (M)	(Ions score 58)
168 - 184	647.3114	1938.9124	1938.9034	5	0	K.YSHVDLVVMVDGFEGEK.G	Oxidation (M)	(Ions score 50)
168 - 184	970.4635	1938.9124	1938.9034	5	0	K.YSHVDLVVMVDGFEGEK.G	Oxidation (M)	(Ions score 48)
168 - 184	970.4635	1938.9124	1938.9034	5	0	K.YSHVDLVVMVDGFEGEK.G	Oxidation (M)	(Ions score 58)
168 - 184	647.3114	1938.9124	1938.9034	5	0	K.YSHVDLVVMVDGFEGEK.G	Oxidation (M)	(Ions score 61)
168 - 184	970.4635	1938.9125	1938.9034	5	0	K.YSHVDLVVMVDGFEGEK.G	Oxidation (M)	(Ions score 57)
168 - 184	647.3115	1938.9126	1938.9034	5	0	K.YSHVDLVVMVDGFEGEK.G	Oxidation (M)	(Ions score 58)
168 - 184	647.3115	1938.9127	1938.9034	5	0	K.YSHVDLVVMVDGFEGEK.G	Oxidation (M)	(Ions score 61)
168 - 184	970.4636	1938.9127	1938.9034	5	0	K.YSHVDLVVMVDGFEGEK.G	Oxidation (M)	(Ions score 52)
168 - 184	970.4636	1938.9127	1938.9034	5	0	K.YSHVDLVVMVDGFEGEK.G	Oxidation (M)	(Ions score 72)
168 - 184	970.4637	1938.9128	1938.9034	5	0	K.YSHVDLVVMVDGFEGEK.G	Oxidation (M)	(Ions score 55)
168 - 184	647.3116	1938.9129	1938.9034	5	0	K.YSHVDLVVMVDGFEGEK.G	Oxidation (M)	(Ions score 54)
168 - 184	970.4637	1938.9129	1938.9034	5	0	K.YSHVDLVVMVDGFEGEK.G	Oxidation (M)	(Ions score 63)
168 - 184	970.4637	1938.9129	1938.9034	5	0	K.YSHVDLVVMVDGFEGEK.G	Oxidation (M)	(Ions score 52)
168 - 184	970.4638	1938.9130	1938.9034	5	0	K.YSHVDLVVMVDGFEGEK.G	Oxidation (M)	(Ions score 57)
168 - 184	647.3116	1938.9130	1938.9034	5	0	K.YSHVDLVVMVDGFEGEK.G	Oxidation (M)	(Ions score 60)
168 - 184	970.4638	1938.9130	1938.9034	5	0	K.YSHVDLVVMVDGFEGEK.G	Oxidation (M)	(Ions score 50)
168 - 192	656.0831	2620.3031	2620.2956	3	1	K.YSHVDLVVMVDGFEGEKGAVVAGSR.G		(Ions score 26)

185 - 192	716.4048	715.3976	715.3977	-0	0	K.GAVVAGSR.G	(Ions score 39)
193 - 197	627.3505	626.3432	626.3428	1	0	R.GYFLK.G	(Ions score 29)
193 - 213	814.4741	2440.4003	2440.3882	5	1	R.GYFLKGVLVFLEQALIQYALR.T	(Ions score 75)
198 - 213	611.6907	1832.0504	1832.0560	-3	0	K.GVLVFLEQALIQYALR.T	(Ions score 46)
198 - 213	917.0357	1832.0568	1832.0560	0	0	K.GVLVFLEQALIQYALR.T	(Ions score 97)
198 - 213	611.6929	1832.0568	1832.0560	0	0	K.GVLVFLEQALIQYALR.T	(Ions score 93)
198 - 213	1833.0643	1832.0571	1832.0560	1	0	K.GVLVFLEQALIQYALR.T	(Ions score 78)
198 - 213	611.6930	1832.0571	1832.0560	1	0	K.GVLVFLEQALIQYALR.T	(Ions score 93)
198 - 213	611.6932	1832.0578	1832.0560	1	0	K.GVLVFLEQALIQYALR.T	(Ions score 75)
198 - 213	917.0363	1832.0580	1832.0560	1	0	K.GVLVFLEQALIQYALR.T	(Ions score 113)
198 - 213	917.0366	1832.0586	1832.0560	1	0	K.GVLVFLEQALIQYALR.T	(Ions score 113)
198 - 213	917.0366	1832.0586	1832.0560	1	0	K.GVLVFLEQALIQYALR.T	(Ions score 85)
198 - 213	611.6936	1832.0589	1832.0560	2	0	K.GVLVFLEQALIQYALR.T	(Ions score 76)
198 - 213	611.6936	1832.0589	1832.0560	2	0	K.GVLVFLEQALIQYALR.T	(Ions score 93)
198 - 213	917.0368	1832.0591	1832.0560	2	0	K.GVLVFLEQALIQYALR.T	(Ions score 107)
198 - 213	917.0369	1832.0592	1832.0560	2	0	K.GVLVFLEQALIQYALR.T	(Ions score 55)
198 - 213	917.0369	1832.0592	1832.0560	2	0	K.GVLVFLEQALIQYALR.T	(Ions score 107)
198 - 213	917.0370	1832.0595	1832.0560	2	0	K.GVLVFLEQALIQYALR.T	(Ions score 113)
198 - 213	611.6938	1832.0595	1832.0560	2	0	K.GVLVFLEQALIQYALR.T	(Ions score 75)
198 - 213	611.6938	1832.0597	1832.0560	2	0	K.GVLVFLEQALIQYALR.T	(Ions score 63)
198 - 213	611.6939	1832.0599	1832.0560	2	0	K.GVLVFLEQALIQYALR.T	(Ions score 93)
198 - 213	917.0372	1832.0599	1832.0560	2	0	K.GVLVFLEQALIQYALR.T	(Ions score 78)
198 - 213	611.6940	1832.0601	1832.0560	2	0	K.GVLVFLEQALIQYALR.T	(Ions score 76)
198 - 213	917.0374	1832.0603	1832.0560	2	0	K.GVLVFLEQALIQYALR.T	(Ions score 107)
198 - 213	917.0377	1832.0609	1832.0560	3	0	K.GVLVFLEQALIQYALR.T	(Ions score 107)
198 - 213	611.6944	1832.0613	1832.0560	3	0	K.GVLVFLEQALIQYALR.T	(Ions score 60)
198 - 213	611.6944	1832.0613	1832.0560	3	0	K.GVLVFLEQALIQYALR.T	(Ions score 65)
198 - 213	917.0381	1832.0617	1832.0560	3	0	K.GVLVFLEQALIQYALR.T	(Ions score 107)
198 - 213	611.6946	1832.0618	1832.0560	3	0	K.GVLVFLEQALIQYALR.T	(Ions score 76)
198 - 213	917.0383	1832.0620	1832.0560	3	0	K.GVLVFLEQALIQYALR.T	(Ions score 103)
198 - 213	917.0383	1832.0620	1832.0560	3	0	K.GVLVFLEQALIQYALR.T	(Ions score 103)
198 - 213	611.6947	1832.0621	1832.0560	3	0	K.GVLVFLEQALIQYALR.T	(Ions score 75)
198 - 213	917.0385	1832.0624	1832.0560	3	0	K.GVLVFLEQALIQYALR.T	(Ions score 107)
198 - 213	611.6947	1832.0624	1832.0560	4	0	K.GVLVFLEQALIQYALR.T	(Ions score 80)
198 - 213	917.0385	1832.0624	1832.0560	4	0	K.GVLVFLEQALIQYALR.T	(Ions score 107)
198 - 213	917.0385	1832.0625	1832.0560	4	0	K.GVLVFLEQALIQYALR.T	(Ions score 113)
198 - 213	917.0386	1832.0626	1832.0560	4	0	K.GVLVFLEQALIQYALR.T	(Ions score 107)
198 - 213	917.0387	1832.0628	1832.0560	4	0	K.GVLVFLEQALIQYALR.T	(Ions score 107)
198 - 213	917.0388	1832.0630	1832.0560	4	0	K.GVLVFLEQALIQYALR.T	(Ions score 107)
198 - 213	917.0388	1832.0631	1832.0560	4	0	K.GVLVFLEQALIQYALR.T	(Ions score 107)
198 - 213	611.6950	1832.0631	1832.0560	4	0	K.GVLVFLEQALIQYALR.T	(Ions score 93)
198 - 213	917.0388	1832.0631	1832.0560	4	0	K.GVLVFLEQALIQYALR.T	(Ions score 107)
198 - 213	611.6950	1832.0633	1832.0560	4	0	K.GVLVFLEQALIQYALR.T	(Ions score 76)
198 - 213	611.6951	1832.0636	1832.0560	4	0	K.GVLVFLEQALIQYALR.T	(Ions score 76)
198 - 213	611.6951	1832.0636	1832.0560	4	0	K.GVLVFLEQALIQYALR.T	(Ions score 93)
198 - 213	917.0391	1832.0636	1832.0560	4	0	K.GVLVFLEQALIQYALR.T	(Ions score 107)
198 - 213	611.6952	1832.0638	1832.0560	4	0	K.GVLVFLEQALIQYALR.T	(Ions score 75)
198 - 213	611.6953	1832.0640	1832.0560	4	0	K.GVLVFLEQALIQYALR.T	(Ions score 93)
198 - 213	611.6953	1832.0640	1832.0560	4	0	K.GVLVFLEQALIQYALR.T	(Ions score 68)
198 - 213	917.0393	1832.0640	1832.0560	4	0	K.GVLVFLEQALIQYALR.T	(Ions score 127)
198 - 213	611.6953	1832.0641	1832.0560	4	0	K.GVLVFLEQALIQYALR.T	(Ions score 76)
198 - 213	917.0393	1832.0641	1832.0560	4	0	K.GVLVFLEQALIQYALR.T	(Ions score 107)
198 - 213	917.0393	1832.0641	1832.0560	4	0	K.GVLVFLEQALIQYALR.T	(Ions score 127)
198 - 213	917.0394	1832.0643	1832.0560	5	0	K.GVLVFLEQALIQYALR.T	(Ions score 121)
198 - 213	611.6954	1832.0644	1832.0560	5	0	K.GVLVFLEQALIQYALR.T	(Ions score 72)
198 - 213	611.6954	1832.0645	1832.0560	5	0	K.GVLVFLEQALIQYALR.T	(Ions score 72)
198 - 213	611.6955	1832.0645	1832.0560	5	0	K.GVLVFLEQALIQYALR.T	(Ions score 57)
198 - 213	611.6955	1832.0647	1832.0560	5	0	K.GVLVFLEQALIQYALR.T	(Ions score 93)

198 - 213	611.6955	1832.0647	1832.0560	5	0	K.GVLVFLEQALIQYALR.T		(Ions score 76)
198 - 213	917.0397	1832.0648	1832.0560	5	0	K.GVLVFLEQALIQYALR.T		(Ions score 107)
198 - 213	611.6955	1832.0648	1832.0560	5	0	K.GVLVFLEQALIQYALR.T		(Ions score 76)
198 - 213	611.6955	1832.0648	1832.0560	5	0	K.GVLVFLEQALIQYALR.T		(Ions score 76)
198 - 213	917.0398	1832.0650	1832.0560	5	0	K.GVLVFLEQALIQYALR.T		(Ions score 121)
219 - 230	752.8863	1503.7581	1503.7584	-0	0	R.GYIPIYTPFFMR.K		(Ions score 22)
219 - 230	502.2602	1503.7589	1503.7584	0	0	R.GYIPIYTPFFMR.K		(Ions score 39)
219 - 230	752.8870	1503.7595	1503.7584	1	0	R.GYIPIYTPFFMR.K		(Ions score 22)
219 - 230	752.8871	1503.7597	1503.7584	1	0	R.GYIPIYTPFFMR.K		(Ions score 23)
219 - 230	752.8872	1503.7599	1503.7584	1	0	R.GYIPIYTPFFMR.K		(Ions score 25)
219 - 230	502.2607	1503.7602	1503.7584	1	0	R.GYIPIYTPFFMR.K		(Ions score 39)
219 - 230	752.8876	1503.7606	1503.7584	1	0	R.GYIPIYTPFFMR.K		(Ions score 40)
219 - 230	752.8876	1503.7607	1503.7584	1	0	R.GYIPIYTPFFMR.K		(Ions score 40)
219 - 230	502.2609	1503.7607	1503.7584	2	0	R.GYIPIYTPFFMR.K		(Ions score 47)
219 - 230	502.2609	1503.7608	1503.7584	2	0	R.GYIPIYTPFFMR.K		(Ions score 53)
219 - 230	752.8879	1503.7613	1503.7584	2	0	R.GYIPIYTPFFMR.K		(Ions score 34)
219 - 230	752.8882	1503.7619	1503.7584	2	0	R.GYIPIYTPFFMR.K		(Ions score 23)
219 - 230	752.8883	1503.7621	1503.7584	2	0	R.GYIPIYTPFFMR.K		(Ions score 38)
219 - 230	752.8885	1503.7624	1503.7584	3	0	R.GYIPIYTPFFMR.K		(Ions score 23)
219 - 230	752.8887	1503.7628	1503.7584	3	0	R.GYIPIYTPFFMR.K		(Ions score 24)
219 - 230	752.8889	1503.7633	1503.7584	3	0	R.GYIPIYTPFFMR.K		(Ions score 24)
219 - 230	752.8893	1503.7641	1503.7584	4	0	R.GYIPIYTPFFMR.K		(Ions score 23)
219 - 230	1520.7626	1519.7553	1519.7534	1	0	R.GYIPIYTPFFMR.K	Oxidation (M)	(Ions score 24)
219 - 230	1520.7627	1519.7554	1519.7534	1	0	R.GYIPIYTPFFMR.K	Oxidation (M)	(Ions score 23)
219 - 230	507.5925	1519.7556	1519.7534	1	0	R.GYIPIYTPFFMR.K	Oxidation (M)	(Ions score 26)
219 - 230	507.5925	1519.7557	1519.7534	2	0	R.GYIPIYTPFFMR.K	Oxidation (M)	(Ions score 36)
219 - 230	760.8852	1519.7558	1519.7534	2	0	R.GYIPIYTPFFMR.K	Oxidation (M)	(Ions score 22)
219 - 230	760.8852	1519.7558	1519.7534	2	0	R.GYIPIYTPFFMR.K	Oxidation (M)	(Ions score 23)
219 - 230	760.8852	1519.7559	1519.7534	2	0	R.GYIPIYTPFFMR.K	Oxidation (M)	(Ions score 23)
219 - 230	507.5926	1519.7559	1519.7534	2	0	R.GYIPIYTPFFMR.K	Oxidation (M)	(Ions score 25)
219 - 230	507.5926	1519.7560	1519.7534	2	0	R.GYIPIYTPFFMR.K	Oxidation (M)	(Ions score 29)
219 - 230	507.5927	1519.7563	1519.7534	2	0	R.GYIPIYTPFFMR.K	Oxidation (M)	(Ions score 26)
219 - 230	507.5927	1519.7563	1519.7534	2	0	R.GYIPIYTPFFMR.K	Oxidation (M)	(Ions score 29)
219 - 230	507.5927	1519.7564	1519.7534	2	0	R.GYIPIYTPFFMR.K	Oxidation (M)	(Ions score 22)
219 - 230	507.5927	1519.7564	1519.7534	2	0	R.GYIPIYTPFFMR.K	Oxidation (M)	(Ions score 22)
219 - 230	507.5928	1519.7567	1519.7534	2	0	R.GYIPIYTPFFMR.K	Oxidation (M)	(Ions score 27)
219 - 230	507.5929	1519.7568	1519.7534	2	0	R.GYIPIYTPFFMR.K	Oxidation (M)	(Ions score 29)
219 - 230	507.5930	1519.7572	1519.7534	3	0	R.GYIPIYTPFFMR.K	Oxidation (M)	(Ions score 38)
219 - 230	507.5931	1519.7575	1519.7534	3	0	R.GYIPIYTPFFMR.K	Oxidation (M)	(Ions score 23)
219 - 230	760.8871	1519.7597	1519.7534	4	0	R.GYIPIYTPFFMR.K	Oxidation (M)	(Ions score 23)
219 - 230	760.8872	1519.7598	1519.7534	4	0	R.GYIPIYTPFFMR.K	Oxidation (M)	(Ions score 22)
219 - 230	507.5942	1519.7606	1519.7534	5	0	R.GYIPIYTPFFMR.K	Oxidation (M)	(Ions score 22)
231 - 249	772.0450	2313.1131	2313.1198	-3	1	R.KEVMQEVAQLSQFDEELYK.V		(Ions score 31)
231 - 249	772.0451	2313.1134	2313.1198	-3	1	R.KEVMQEVAQLSQFDEELYK.V		(Ions score 51)
231 - 249	772.0467	2313.1182	2313.1198	-1	1	R.KEVMQEVAQLSQFDEELYK.V		(Ions score 32)
231 - 249	1157.5669	2313.1192	2313.1198	-0	1	R.KEVMQEVAQLSQFDEELYK.V		(Ions score 86)
231 - 249	772.0472	2313.1199	2313.1198	0	1	R.KEVMQEVAQLSQFDEELYK.V		(Ions score 78)
231 - 249	772.0472	2313.1199	2313.1198	0	1	R.KEVMQEVAQLSQFDEELYK.V		(Ions score 36)
231 - 249	772.0477	2313.1213	2313.1198	1	1	R.KEVMQEVAQLSQFDEELYK.V		(Ions score 55)
231 - 249	772.0478	2313.1215	2313.1198	1	1	R.KEVMQEVAQLSQFDEELYK.V		(Ions score 54)
231 - 249	772.0479	2313.1220	2313.1198	1	1	R.KEVMQEVAQLSQFDEELYK.V		(Ions score 53)
231 - 249	579.2879	2313.1225	2313.1198	1	1	R.KEVMQEVAQLSQFDEELYK.V		(Ions score 61)
231 - 249	772.0482	2313.1228	2313.1198	1	1	R.KEVMQEVAQLSQFDEELYK.V		(Ions score 36)
231 - 249	772.0482	2313.1228	2313.1198	1	1	R.KEVMQEVAQLSQFDEELYK.V		(Ions score 27)
231 - 249	772.0482	2313.1229	2313.1198	1	1	R.KEVMQEVAQLSQFDEELYK.V		(Ions score 63)
231 - 249	772.0484	2313.1233	2313.1198	1	1	R.KEVMQEVAQLSQFDEELYK.V		(Ions score 52)
231 - 249	1157.5691	2313.1236	2313.1198	2	1	R.KEVMQEVAQLSQFDEELYK.V		(Ions score 83)
231 - 249	1157.5692	2313.1239	2313.1198	2	1	R.KEVMQEVAQLSQFDEELYK.V		(Ions score 87)

231 - 249	772.0486	2313.1239	2313.1198	2	1	R.KEVMQEVAQLSQFDEELYK.V		(Ions score 31)
231 - 249	772.0486	2313.1239	2313.1198	2	1	R.KEVMQEVAQLSQFDEELYK.V		(Ions score 45)
231 - 249	772.0486	2313.1239	2313.1198	2	1	R.KEVMQEVAQLSQFDEELYK.V		(Ions score 44)
231 - 249	772.0486	2313.1239	2313.1198	2	1	R.KEVMQEVAQLSQFDEELYK.V		(Ions score 36)
231 - 249	1157.5693	2313.1241	2313.1198	2	1	R.KEVMQEVAQLSQFDEELYK.V		(Ions score 77)
231 - 249	772.0487	2313.1242	2313.1198	2	1	R.KEVMQEVAQLSQFDEELYK.V		(Ions score 35)
231 - 249	772.0487	2313.1242	2313.1198	2	1	R.KEVMQEVAQLSQFDEELYK.V		(Ions score 47)
231 - 249	772.0487	2313.1242	2313.1198	2	1	R.KEVMQEVAQLSQFDEELYK.V		(Ions score 26)
231 - 249	772.0487	2313.1242	2313.1198	2	1	R.KEVMQEVAQLSQFDEELYK.V		(Ions score 59)
231 - 249	1157.5694	2313.1243	2313.1198	2	1	R.KEVMQEVAQLSQFDEELYK.V		(Ions score 93)
231 - 249	1157.5695	2313.1244	2313.1198	2	1	R.KEVMQEVAQLSQFDEELYK.V		(Ions score 85)
231 - 249	772.0487	2313.1244	2313.1198	2	1	R.KEVMQEVAQLSQFDEELYK.V		(Ions score 62)
231 - 249	1157.5695	2313.1245	2313.1198	2	1	R.KEVMQEVAQLSQFDEELYK.V		(Ions score 72)
231 - 249	772.0488	2313.1247	2313.1198	2	1	R.KEVMQEVAQLSQFDEELYK.V		(Ions score 29)
231 - 249	1157.5696	2313.1247	2313.1198	2	1	R.KEVMQEVAQLSQFDEELYK.V		(Ions score 104)
231 - 249	772.0489	2313.1249	2313.1198	2	1	R.KEVMQEVAQLSQFDEELYK.V		(Ions score 78)
231 - 249	772.0490	2313.1250	2313.1198	2	1	R.KEVMQEVAQLSQFDEELYK.V		(Ions score 72)
231 - 249	1157.5699	2313.1252	2313.1198	2	1	R.KEVMQEVAQLSQFDEELYK.V		(Ions score 78)
231 - 249	1157.5699	2313.1252	2313.1198	2	1	R.KEVMQEVAQLSQFDEELYK.V		(Ions score 92)
231 - 249	772.0490	2313.1253	2313.1198	2	1	R.KEVMQEVAQLSQFDEELYK.V		(Ions score 75)
231 - 249	772.0491	2313.1255	2313.1198	2	1	R.KEVMQEVAQLSQFDEELYK.V		(Ions score 42)
231 - 249	772.0492	2313.1257	2313.1198	3	1	R.KEVMQEVAQLSQFDEELYK.V		(Ions score 59)
231 - 249	772.0492	2313.1258	2313.1198	3	1	R.KEVMQEVAQLSQFDEELYK.V		(Ions score 51)
231 - 249	1157.5702	2313.1258	2313.1198	3	1	R.KEVMQEVAQLSQFDEELYK.V		(Ions score 75)
231 - 249	772.0492	2313.1259	2313.1198	3	1	R.KEVMQEVAQLSQFDEELYK.V		(Ions score 54)
231 - 249	772.0492	2313.1259	2313.1198	3	1	R.KEVMQEVAQLSQFDEELYK.V		(Ions score 66)
231 - 249	772.0493	2313.1260	2313.1198	3	1	R.KEVMQEVAQLSQFDEELYK.V		(Ions score 80)
231 - 249	772.0494	2313.1262	2313.1198	3	1	R.KEVMQEVAQLSQFDEELYK.V		(Ions score 51)
231 - 249	1157.5705	2313.1265	2313.1198	3	1	R.KEVMQEVAQLSQFDEELYK.V		(Ions score 83)
231 - 249	772.0495	2313.1266	2313.1198	3	1	R.KEVMQEVAQLSQFDEELYK.V		(Ions score 64)
231 - 249	772.0495	2313.1267	2313.1198	3	1	R.KEVMQEVAQLSQFDEELYK.V		(Ions score 45)
231 - 249	772.0495	2313.1267	2313.1198	3	1	R.KEVMQEVAQLSQFDEELYK.V		(Ions score 61)
231 - 249	772.0495	2313.1267	2313.1198	3	1	R.KEVMQEVAQLSQFDEELYK.V		(Ions score 68)
231 - 249	772.0496	2313.1269	2313.1198	3	1	R.KEVMQEVAQLSQFDEELYK.V		(Ions score 57)
231 - 249	772.0496	2313.1269	2313.1198	3	1	R.KEVMQEVAQLSQFDEELYK.V		(Ions score 62)
231 - 249	772.0496	2313.1270	2313.1198	3	1	R.KEVMQEVAQLSQFDEELYK.V		(Ions score 65)
231 - 249	772.0496	2313.1271	2313.1198	3	1	R.KEVMQEVAQLSQFDEELYK.V		(Ions score 59)
231 - 249	772.0498	2313.1275	2313.1198	3	1	R.KEVMQEVAQLSQFDEELYK.V		(Ions score 43)
231 - 249	772.0499	2313.1278	2313.1198	3	1	R.KEVMQEVAQLSQFDEELYK.V		(Ions score 65)
231 - 249	772.0499	2313.1280	2313.1198	4	1	R.KEVMQEVAQLSQFDEELYK.V		(Ions score 43)
231 - 249	772.0500	2313.1283	2313.1198	4	1	R.KEVMQEVAQLSQFDEELYK.V		(Ions score 44)
231 - 249	772.0501	2313.1284	2313.1198	4	1	R.KEVMQEVAQLSQFDEELYK.V		(Ions score 74)
231 - 249	772.0501	2313.1284	2313.1198	4	1	R.KEVMQEVAQLSQFDEELYK.V		(Ions score 40)
231 - 249	772.0501	2313.1286	2313.1198	4	1	R.KEVMQEVAQLSQFDEELYK.V		(Ions score 45)
231 - 249	772.0502	2313.1289	2313.1198	4	1	R.KEVMQEVAQLSQFDEELYK.V		(Ions score 61)
231 - 249	1157.5718	2313.1291	2313.1198	4	1	R.KEVMQEVAQLSQFDEELYK.V		(Ions score 49)
231 - 249	1157.5719	2313.1292	2313.1198	4	1	R.KEVMQEVAQLSQFDEELYK.V		(Ions score 100)
231 - 249	772.0504	2313.1293	2313.1198	4	1	R.KEVMQEVAQLSQFDEELYK.V		(Ions score 80)
231 - 249	772.0504	2313.1295	2313.1198	4	1	R.KEVMQEVAQLSQFDEELYK.V		(Ions score 51)
231 - 249	772.0505	2313.1295	2313.1198	4	1	R.KEVMQEVAQLSQFDEELYK.V		(Ions score 58)
231 - 249	772.0505	2313.1296	2313.1198	4	1	R.KEVMQEVAQLSQFDEELYK.V		(Ions score 58)
231 - 249	772.0509	2313.1309	2313.1198	5	1	R.KEVMQEVAQLSQFDEELYK.V		(Ions score 43)
231 - 249	777.3765	2329.1076	2329.1147	-3	1	R.KEVMQEVAQLSQFDEELYK.V	Oxidation (M)	(Ions score 37)
231 - 249	777.3769	2329.1089	2329.1147	-3	1	R.KEVMQEVAQLSQFDEELYK.V	Oxidation (M)	(Ions score 21)
231 - 249	777.3771	2329.1095	2329.1147	-2	1	R.KEVMQEVAQLSQFDEELYK.V	Oxidation (M)	(Ions score 39)
231 - 249	1165.5624	2329.1103	2329.1147	-2	1	R.KEVMQEVAQLSQFDEELYK.V	Oxidation (M)	(Ions score 47)
231 - 249	777.3774	2329.1105	2329.1147	-2	1	R.KEVMQEVAQLSQFDEELYK.V	Oxidation (M)	(Ions score 23)
231 - 249	777.3774	2329.1105	2329.1147	-2	1	R.KEVMQEVAQLSQFDEELYK.V	Oxidation (M)	(Ions score 22)

231 - 249	777.3776	2329.1111	2329.1147	-2	1	R.KEVMQEVAQLSQFDEELYK.V	Oxidation (M)	(Ions score 22)
231 - 249	1165.5631	2329.1116	2329.1147	-1	1	R.KEVMQEVAQLSQFDEELYK.V	Oxidation (M)	(Ions score 40)
231 - 249	777.3778	2329.1116	2329.1147	-1	1	R.KEVMQEVAQLSQFDEELYK.V	Oxidation (M)	(Ions score 23)
231 - 249	1165.5633	2329.1121	2329.1147	-1	1	R.KEVMQEVAQLSQFDEELYK.V	Oxidation (M)	(Ions score 64)
231 - 249	777.3781	2329.1123	2329.1147	-1	1	R.KEVMQEVAQLSQFDEELYK.V	Oxidation (M)	(Ions score 38)
231 - 249	777.3782	2329.1128	2329.1147	-1	1	R.KEVMQEVAQLSQFDEELYK.V	Oxidation (M)	(Ions score 24)
231 - 249	777.3782	2329.1129	2329.1147	-1	1	R.KEVMQEVAQLSQFDEELYK.V	Oxidation (M)	(Ions score 29)
231 - 249	777.3783	2329.1130	2329.1147	-1	1	R.KEVMQEVAQLSQFDEELYK.V	Oxidation (M)	(Ions score 46)
231 - 249	777.3783	2329.1131	2329.1147	-1	1	R.KEVMQEVAQLSQFDEELYK.V	Oxidation (M)	(Ions score 25)
231 - 249	777.3784	2329.1132	2329.1147	-1	1	R.KEVMQEVAQLSQFDEELYK.V	Oxidation (M)	(Ions score 25)
231 - 249	777.3785	2329.1136	2329.1147	-0	1	R.KEVMQEVAQLSQFDEELYK.V	Oxidation (M)	(Ions score 24)
231 - 249	777.3785	2329.1138	2329.1147	-0	1	R.KEVMQEVAQLSQFDEELYK.V	Oxidation (M)	(Ions score 63)
231 - 249	777.3785	2329.1138	2329.1147	-0	1	R.KEVMQEVAQLSQFDEELYK.V	Oxidation (M)	(Ions score 27)
231 - 249	1165.5643	2329.1141	2329.1147	-0	1	R.KEVMQEVAQLSQFDEELYK.V	Oxidation (M)	(Ions score 59)
231 - 249	777.3789	2329.1148	2329.1147	0	1	R.KEVMQEVAQLSQFDEELYK.V	Oxidation (M)	(Ions score 51)
231 - 249	777.3789	2329.1149	2329.1147	0	1	R.KEVMQEVAQLSQFDEELYK.V	Oxidation (M)	(Ions score 31)
231 - 249	1165.5649	2329.1153	2329.1147	0	1	R.KEVMQEVAQLSQFDEELYK.V	Oxidation (M)	(Ions score 102)
231 - 249	777.3791	2329.1154	2329.1147	0	1	R.KEVMQEVAQLSQFDEELYK.V	Oxidation (M)	(Ions score 25)
231 - 249	1165.5652	2329.1158	2329.1147	0	1	R.KEVMQEVAQLSQFDEELYK.V	Oxidation (M)	(Ions score 61)
231 - 249	1165.5653	2329.1160	2329.1147	1	1	R.KEVMQEVAQLSQFDEELYK.V	Oxidation (M)	(Ions score 55)
231 - 249	777.3793	2329.1160	2329.1147	1	1	R.KEVMQEVAQLSQFDEELYK.V	Oxidation (M)	(Ions score 37)
231 - 249	777.3793	2329.1162	2329.1147	1	1	R.KEVMQEVAQLSQFDEELYK.V	Oxidation (M)	(Ions score 36)
231 - 249	777.3794	2329.1165	2329.1147	1	1	R.KEVMQEVAQLSQFDEELYK.V	Oxidation (M)	(Ions score 37)
231 - 249	777.3795	2329.1167	2329.1147	1	1	R.KEVMQEVAQLSQFDEELYK.V	Oxidation (M)	(Ions score 23)
231 - 249	1165.5657	2329.1168	2329.1147	1	1	R.KEVMQEVAQLSQFDEELYK.V	Oxidation (M)	(Ions score 41)
231 - 249	1165.5657	2329.1168	2329.1147	1	1	R.KEVMQEVAQLSQFDEELYK.V	Oxidation (M)	(Ions score 55)
231 - 249	1165.5659	2329.1172	2329.1147	1	1	R.KEVMQEVAQLSQFDEELYK.V	Oxidation (M)	(Ions score 62)
231 - 249	1165.5659	2329.1173	2329.1147	1	1	R.KEVMQEVAQLSQFDEELYK.V	Oxidation (M)	(Ions score 38)
231 - 249	1165.5659	2329.1173	2329.1147	1	1	R.KEVMQEVAQLSQFDEELYK.V	Oxidation (M)	(Ions score 46)
231 - 249	777.3798	2329.1175	2329.1147	1	1	R.KEVMQEVAQLSQFDEELYK.V	Oxidation (M)	(Ions score 47)
231 - 249	777.3798	2329.1175	2329.1147	1	1	R.KEVMQEVAQLSQFDEELYK.V	Oxidation (M)	(Ions score 24)
231 - 249	777.3798	2329.1175	2329.1147	1	1	R.KEVMQEVAQLSQFDEELYK.V	Oxidation (M)	(Ions score 29)
231 - 249	777.3798	2329.1175	2329.1147	1	1	R.KEVMQEVAQLSQFDEELYK.V	Oxidation (M)	(Ions score 40)
231 - 249	1165.5662	2329.1178	2329.1147	1	1	R.KEVMQEVAQLSQFDEELYK.V	Oxidation (M)	(Ions score 61)
231 - 249	1165.5662	2329.1179	2329.1147	1	1	R.KEVMQEVAQLSQFDEELYK.V	Oxidation (M)	(Ions score 46)
231 - 249	777.3799	2329.1180	2329.1147	1	1	R.KEVMQEVAQLSQFDEELYK.V	Oxidation (M)	(Ions score 40)
231 - 249	777.3799	2329.1180	2329.1147	1	1	R.KEVMQEVAQLSQFDEELYK.V	Oxidation (M)	(Ions score 29)
231 - 249	1165.5663	2329.1181	2329.1147	1	1	R.KEVMQEVAQLSQFDEELYK.V	Oxidation (M)	(Ions score 83)
231 - 249	1165.5664	2329.1182	2329.1147	1	1	R.KEVMQEVAQLSQFDEELYK.V	Oxidation (M)	(Ions score 58)
231 - 249	583.2868	2329.1183	2329.1147	2	1	R.KEVMQEVAQLSQFDEELYK.V	Oxidation (M)	(Ions score 25)
231 - 249	777.3801	2329.1185	2329.1147	2	1	R.KEVMQEVAQLSQFDEELYK.V	Oxidation (M)	(Ions score 50)
231 - 249	1165.5666	2329.1186	2329.1147	2	1	R.KEVMQEVAQLSQFDEELYK.V	Oxidation (M)	(Ions score 58)
231 - 249	583.2869	2329.1186	2329.1147	2	1	R.KEVMQEVAQLSQFDEELYK.V	Oxidation (M)	(Ions score 33)
231 - 249	777.3802	2329.1188	2329.1147	2	1	R.KEVMQEVAQLSQFDEELYK.V	Oxidation (M)	(Ions score 37)
231 - 249	1165.5667	2329.1188	2329.1147	2	1	R.KEVMQEVAQLSQFDEELYK.V	Oxidation (M)	(Ions score 58)
231 - 249	777.3802	2329.1189	2329.1147	2	1	R.KEVMQEVAQLSQFDEELYK.V	Oxidation (M)	(Ions score 53)
231 - 249	1165.5669	2329.1192	2329.1147	2	1	R.KEVMQEVAQLSQFDEELYK.V	Oxidation (M)	(Ions score 68)
231 - 249	777.3804	2329.1193	2329.1147	2	1	R.KEVMQEVAQLSQFDEELYK.V	Oxidation (M)	(Ions score 37)
231 - 249	777.3804	2329.1193	2329.1147	2	1	R.KEVMQEVAQLSQFDEELYK.V	Oxidation (M)	(Ions score 49)
231 - 249	777.3804	2329.1194	2329.1147	2	1	R.KEVMQEVAQLSQFDEELYK.V	Oxidation (M)	(Ions score 36)
231 - 249	777.3805	2329.1196	2329.1147	2	1	R.KEVMQEVAQLSQFDEELYK.V	Oxidation (M)	(Ions score 47)
231 - 249	777.3805	2329.1197	2329.1147	2	1	R.KEVMQEVAQLSQFDEELYK.V	Oxidation (M)	(Ions score 35)
231 - 249	777.3806	2329.1200	2329.1147	2	1	R.KEVMQEVAQLSQFDEELYK.V	Oxidation (M)	(Ions score 34)
231 - 249	777.3806	2329.1200	2329.1147	2	1	R.KEVMQEVAQLSQFDEELYK.V	Oxidation (M)	(Ions score 46)
231 - 249	1165.5673	2329.1200	2329.1147	2	1	R.KEVMQEVAQLSQFDEELYK.V	Oxidation (M)	(Ions score 68)
231 - 249	777.3806	2329.1201	2329.1147	2	1	R.KEVMQEVAQLSQFDEELYK.V	Oxidation (M)	(Ions score 51)
231 - 249	1165.5674	2329.1202	2329.1147	2	1	R.KEVMQEVAQLSQFDEELYK.V	Oxidation (M)	(Ions score 82)
231 - 249	777.3807	2329.1202	2329.1147	2	1	R.KEVMQEVAQLSQFDEELYK.V	Oxidation (M)	(Ions score 41)

231 - 249	1165.5674	2329.1203	2329.1147	2	1	R.KEVMQEVAQLSQFDEELYK.V	Oxidation (M)	(Ions score 42)
231 - 249	777.3807	2329.1204	2329.1147	2	1	R.KEVMQEVAQLSQFDEELYK.V	Oxidation (M)	(Ions score 21)
231 - 249	1165.5675	2329.1205	2329.1147	2	1	R.KEVMQEVAQLSQFDEELYK.V	Oxidation (M)	(Ions score 68)
231 - 249	777.3808	2329.1205	2329.1147	2	1	R.KEVMQEVAQLSQFDEELYK.V	Oxidation (M)	(Ions score 32)
231 - 249	777.3809	2329.1209	2329.1147	3	1	R.KEVMQEVAQLSQFDEELYK.V	Oxidation (M)	(Ions score 24)
231 - 249	1165.5678	2329.1210	2329.1147	3	1	R.KEVMQEVAQLSQFDEELYK.V	Oxidation (M)	(Ions score 57)
231 - 249	777.3809	2329.1210	2329.1147	3	1	R.KEVMQEVAQLSQFDEELYK.V	Oxidation (M)	(Ions score 28)
231 - 249	777.3810	2329.1211	2329.1147	3	1	R.KEVMQEVAQLSQFDEELYK.V	Oxidation (M)	(Ions score 21)
231 - 249	777.3810	2329.1212	2329.1147	3	1	R.KEVMQEVAQLSQFDEELYK.V	Oxidation (M)	(Ions score 37)
231 - 249	777.3810	2329.1213	2329.1147	3	1	R.KEVMQEVAQLSQFDEELYK.V	Oxidation (M)	(Ions score 51)
231 - 249	777.3811	2329.1214	2329.1147	3	1	R.KEVMQEVAQLSQFDEELYK.V	Oxidation (M)	(Ions score 38)
231 - 249	777.3811	2329.1215	2329.1147	3	1	R.KEVMQEVAQLSQFDEELYK.V	Oxidation (M)	(Ions score 47)
231 - 249	777.3812	2329.1216	2329.1147	3	1	R.KEVMQEVAQLSQFDEELYK.V	Oxidation (M)	(Ions score 31)
231 - 249	1165.5682	2329.1218	2329.1147	3	1	R.KEVMQEVAQLSQFDEELYK.V	Oxidation (M)	(Ions score 83)
231 - 249	777.3812	2329.1219	2329.1147	3	1	R.KEVMQEVAQLSQFDEELYK.V	Oxidation (M)	(Ions score 38)
231 - 249	777.3813	2329.1221	2329.1147	3	1	R.KEVMQEVAQLSQFDEELYK.V	Oxidation (M)	(Ions score 38)
231 - 249	777.3814	2329.1222	2329.1147	3	1	R.KEVMQEVAQLSQFDEELYK.V	Oxidation (M)	(Ions score 22)
231 - 249	777.3814	2329.1222	2329.1147	3	1	R.KEVMQEVAQLSQFDEELYK.V	Oxidation (M)	(Ions score 48)
231 - 249	777.3815	2329.1226	2329.1147	3	1	R.KEVMQEVAQLSQFDEELYK.V	Oxidation (M)	(Ions score 27)
231 - 249	777.3816	2329.1229	2329.1147	3	1	R.KEVMQEVAQLSQFDEELYK.V	Oxidation (M)	(Ions score 28)
231 - 249	1165.5689	2329.1233	2329.1147	4	1	R.KEVMQEVAQLSQFDEELYK.V	Oxidation (M)	(Ions score 68)
231 - 249	777.3817	2329.1234	2329.1147	4	1	R.KEVMQEVAQLSQFDEELYK.V	Oxidation (M)	(Ions score 30)
231 - 249	777.3818	2329.1234	2329.1147	4	1	R.KEVMQEVAQLSQFDEELYK.V	Oxidation (M)	(Ions score 48)
231 - 249	777.3818	2329.1235	2329.1147	4	1	R.KEVMQEVAQLSQFDEELYK.V	Oxidation (M)	(Ions score 22)
231 - 249	777.3818	2329.1235	2329.1147	4	1	R.KEVMQEVAQLSQFDEELYK.V	Oxidation (M)	(Ions score 35)
231 - 249	777.3818	2329.1235	2329.1147	4	1	R.KEVMQEVAQLSQFDEELYK.V	Oxidation (M)	(Ions score 49)
231 - 249	777.3818	2329.1237	2329.1147	4	1	R.KEVMQEVAQLSQFDEELYK.V	Oxidation (M)	(Ions score 22)
231 - 249	777.3819	2329.1237	2329.1147	4	1	R.KEVMQEVAQLSQFDEELYK.V	Oxidation (M)	(Ions score 43)
231 - 249	777.3820	2329.1241	2329.1147	4	1	R.KEVMQEVAQLSQFDEELYK.V	Oxidation (M)	(Ions score 51)
231 - 249	777.3820	2329.1241	2329.1147	4	1	R.KEVMQEVAQLSQFDEELYK.V	Oxidation (M)	(Ions score 48)
231 - 249	777.3820	2329.1243	2329.1147	4	1	R.KEVMQEVAQLSQFDEELYK.V	Oxidation (M)	(Ions score 49)
231 - 249	777.3821	2329.1243	2329.1147	4	1	R.KEVMQEVAQLSQFDEELYK.V	Oxidation (M)	(Ions score 30)
231 - 249	777.3821	2329.1244	2329.1147	4	1	R.KEVMQEVAQLSQFDEELYK.V	Oxidation (M)	(Ions score 37)
231 - 249	777.3824	2329.1255	2329.1147	5	1	R.KEVMQEVAQLSQFDEELYK.V	Oxidation (M)	(Ions score 31)
231 - 249	777.3824	2329.1255	2329.1147	5	1	R.KEVMQEVAQLSQFDEELYK.V	Oxidation (M)	(Ions score 24)
231 - 249	786.0511	2355.1314	2355.1304	0	1	R.KEVMQEVAQLSQFDEELYK.V	Acetylation (K)	(Ions score 59)
231 - 249	786.0514	2355.1322	2355.1304	1	1	R.KEVMQEVAQLSQFDEELYK.V	Acetylation (K)	(Ions score 27)
231 - 249	786.0514	2355.1323	2355.1304	1	1	R.KEVMQEVAQLSQFDEELYK.V	Acetylation (K)	(Ions score 47)
231 - 249	786.0514	2355.1323	2355.1304	1	1	R.KEVMQEVAQLSQFDEELYK.V	Acetylation (K)	(Ions score 58)
231 - 249	786.0514	2355.1325	2355.1304	1	1	R.KEVMQEVAQLSQFDEELYK.V	Acetylation (K)	(Ions score 50)
231 - 249	786.0516	2355.1329	2355.1304	1	1	R.KEVMQEVAQLSQFDEELYK.V	Acetylation (K)	(Ions score 67)
231 - 249	1178.5738	2355.1330	2355.1304	1	1	R.KEVMQEVAQLSQFDEELYK.V	Acetylation (K)	(Ions score 53)
231 - 249	786.0521	2355.1344	2355.1304	2	1	R.KEVMQEVAQLSQFDEELYK.V	Acetylation (K)	(Ions score 47)
231 - 249	786.0527	2355.1362	2355.1304	2	1	R.KEVMQEVAQLSQFDEELYK.V	Acetylation (K)	(Ions score 59)
231 - 249	1178.5755	2355.1364	2355.1304	3	1	R.KEVMQEVAQLSQFDEELYK.V	Acetylation (K)	(Ions score 87)
231 - 249	1178.5759	2355.1373	2355.1304	3	1	R.KEVMQEVAQLSQFDEELYK.V	Acetylation (K)	(Ions score 50)
231 - 249	1178.5777	2355.1409	2355.1304	4	1	R.KEVMQEVAQLSQFDEELYK.V	Acetylation (K)	(Ions score 53)
231 - 249	786.0544	2355.1413	2355.1304	5	1	R.KEVMQEVAQLSQFDEELYK.V	Acetylation (K)	(Ions score 54)
231 - 249	791.3817	2371.1231	2371.1253	-1	1	R.KEVMQEVAQLSQFDEELYK.V	Acetylation (K); Oxidation (M)	(Ions score 22)
231 - 249	791.3826	2371.1259	2371.1253	0	1	R.KEVMQEVAQLSQFDEELYK.V	Acetylation (K); Oxidation (M)	(Ions score 22)
231 - 249	1186.5727	2371.1309	2371.1253	2	1	R.KEVMQEVAQLSQFDEELYK.V	Acetylation (K); Oxidation (M)	(Ions score 49)
231 - 249	1186.5732	2371.1318	2371.1253	3	1	R.KEVMQEVAQLSQFDEELYK.V	Acetylation (K); Oxidation (M)	(Ions score 43)
231 - 249	1186.5733	2371.1320	2371.1253	3	1	R.KEVMQEVAQLSQFDEELYK.V	Acetylation (K); Oxidation (M)	(Ions score 105)
231 - 249	1186.5738	2371.1331	2371.1253	3	1	R.KEVMQEVAQLSQFDEELYK.V	Acetylation (K); Oxidation (M)	(Ions score 63)
232 - 249	1093.5163	2185.0181	2185.0249	-3	0	K.EVMQEVAQLSQFDEELYK.V	(Ions score 67)	
232 - 249	1093.5179	2185.0213	2185.0249	-2	0	K.EVMQEVAQLSQFDEELYK.V	(Ions score 85)	
232 - 249	1093.5181	2185.0216	2185.0249	-2	0	K.EVMQEVAQLSQFDEELYK.V	(Ions score 69)	
232 - 249	729.3479	2185.0217	2185.0249	-1	0	K.EVMQEVAQLSQFDEELYK.V	(Ions score 63)	

232 - 249	729.3479	2185.0219	2185.0249	-1	0	K.EVMQEVAQLSQFDEELYK.V	(Ions score 66)
232 - 249	1093.5186	2185.0226	2185.0249	-1	0	K.EVMQEVAQLSQFDEELYK.V	(Ions score 88)
232 - 249	1093.5187	2185.0228	2185.0249	-1	0	K.EVMQEVAQLSQFDEELYK.V	(Ions score 73)
232 - 249	1093.5191	2185.0236	2185.0249	-1	0	K.EVMQEVAQLSQFDEELYK.V	(Ions score 98)
232 - 249	729.3485	2185.0237	2185.0249	-1	0	K.EVMQEVAQLSQFDEELYK.V	(Ions score 58)
232 - 249	729.3485	2185.0237	2185.0249	-1	0	K.EVMQEVAQLSQFDEELYK.V	(Ions score 77)
232 - 249	1093.5192	2185.0237	2185.0249	-1	0	K.EVMQEVAQLSQFDEELYK.V	(Ions score 86)
232 - 249	1093.5193	2185.0240	2185.0249	-0	0	K.EVMQEVAQLSQFDEELYK.V	(Ions score 95)
232 - 249	1093.5193	2185.0240	2185.0249	-0	0	K.EVMQEVAQLSQFDEELYK.V	(Ions score 71)
232 - 249	1093.5193	2185.0241	2185.0249	-0	0	K.EVMQEVAQLSQFDEELYK.V	(Ions score 105)
232 - 249	729.3489	2185.0250	2185.0249	0	0	K.EVMQEVAQLSQFDEELYK.V	(Ions score 63)
232 - 249	1093.5198	2185.0250	2185.0249	0	0	K.EVMQEVAQLSQFDEELYK.V	(Ions score 68)
232 - 249	1093.5198	2185.0250	2185.0249	0	0	K.EVMQEVAQLSQFDEELYK.V	(Ions score 82)
232 - 249	1093.5198	2185.0251	2185.0249	0	0	K.EVMQEVAQLSQFDEELYK.V	(Ions score 56)
232 - 249	729.3490	2185.0253	2185.0249	0	0	K.EVMQEVAQLSQFDEELYK.V	(Ions score 69)
232 - 249	729.3491	2185.0254	2185.0249	0	0	K.EVMQEVAQLSQFDEELYK.V	(Ions score 75)
232 - 249	1093.5200	2185.0254	2185.0249	0	0	K.EVMQEVAQLSQFDEELYK.V	(Ions score 72)
232 - 249	1093.5200	2185.0255	2185.0249	0	0	K.EVMQEVAQLSQFDEELYK.V	(Ions score 87)
232 - 249	1093.5200	2185.0255	2185.0249	0	0	K.EVMQEVAQLSQFDEELYK.V	(Ions score 84)
232 - 249	729.3491	2185.0255	2185.0249	0	0	K.EVMQEVAQLSQFDEELYK.V	(Ions score 62)
232 - 249	1093.5201	2185.0256	2185.0249	0	0	K.EVMQEVAQLSQFDEELYK.V	(Ions score 86)
232 - 249	729.3492	2185.0259	2185.0249	0	0	K.EVMQEVAQLSQFDEELYK.V	(Ions score 40)
232 - 249	1093.5204	2185.0262	2185.0249	1	0	K.EVMQEVAQLSQFDEELYK.V	(Ions score 88)
232 - 249	1093.5205	2185.0265	2185.0249	1	0	K.EVMQEVAQLSQFDEELYK.V	(Ions score 80)
232 - 249	1093.5206	2185.0266	2185.0249	1	0	K.EVMQEVAQLSQFDEELYK.V	(Ions score 105)
232 - 249	1093.5206	2185.0267	2185.0249	1	0	K.EVMQEVAQLSQFDEELYK.V	(Ions score 71)
232 - 249	1093.5206	2185.0267	2185.0249	1	0	K.EVMQEVAQLSQFDEELYK.V	(Ions score 87)
232 - 249	1093.5207	2185.0268	2185.0249	1	0	K.EVMQEVAQLSQFDEELYK.V	(Ions score 87)
232 - 249	729.3495	2185.0268	2185.0249	1	0	K.EVMQEVAQLSQFDEELYK.V	(Ions score 67)
232 - 249	1093.5207	2185.0269	2185.0249	1	0	K.EVMQEVAQLSQFDEELYK.V	(Ions score 87)
232 - 249	729.3496	2185.0271	2185.0249	1	0	K.EVMQEVAQLSQFDEELYK.V	(Ions score 66)
232 - 249	1093.5208	2185.0271	2185.0249	1	0	K.EVMQEVAQLSQFDEELYK.V	(Ions score 73)
232 - 249	1093.5209	2185.0272	2185.0249	1	0	K.EVMQEVAQLSQFDEELYK.V	(Ions score 82)
232 - 249	1093.5209	2185.0272	2185.0249	1	0	K.EVMQEVAQLSQFDEELYK.V	(Ions score 86)
232 - 249	1093.5209	2185.0272	2185.0249	1	0	K.EVMQEVAQLSQFDEELYK.V	(Ions score 80)
232 - 249	729.3497	2185.0272	2185.0249	1	0	K.EVMQEVAQLSQFDEELYK.V	(Ions score 63)
232 - 249	1093.5209	2185.0272	2185.0249	1	0	K.EVMQEVAQLSQFDEELYK.V	(Ions score 86)
232 - 249	1093.5209	2185.0273	2185.0249	1	0	K.EVMQEVAQLSQFDEELYK.V	(Ions score 83)
232 - 249	729.3497	2185.0273	2185.0249	1	0	K.EVMQEVAQLSQFDEELYK.V	(Ions score 71)
232 - 249	1093.5210	2185.0274	2185.0249	1	0	K.EVMQEVAQLSQFDEELYK.V	(Ions score 100)
232 - 249	1093.5210	2185.0274	2185.0249	1	0	K.EVMQEVAQLSQFDEELYK.V	(Ions score 98)
232 - 249	1093.5210	2185.0275	2185.0249	1	0	K.EVMQEVAQLSQFDEELYK.V	(Ions score 96)
232 - 249	729.3498	2185.0275	2185.0249	1	0	K.EVMQEVAQLSQFDEELYK.V	(Ions score 67)
232 - 249	729.3498	2185.0275	2185.0249	1	0	K.EVMQEVAQLSQFDEELYK.V	(Ions score 62)
232 - 249	729.3498	2185.0276	2185.0249	1	0	K.EVMQEVAQLSQFDEELYK.V	(Ions score 57)
232 - 249	1093.5211	2185.0276	2185.0249	1	0	K.EVMQEVAQLSQFDEELYK.V	(Ions score 84)
232 - 249	1093.5211	2185.0277	2185.0249	1	0	K.EVMQEVAQLSQFDEELYK.V	(Ions score 88)
232 - 249	1093.5211	2185.0277	2185.0249	1	0	K.EVMQEVAQLSQFDEELYK.V	(Ions score 83)
232 - 249	729.3498	2185.0277	2185.0249	1	0	K.EVMQEVAQLSQFDEELYK.V	(Ions score 77)
232 - 249	1093.5212	2185.0278	2185.0249	1	0	K.EVMQEVAQLSQFDEELYK.V	(Ions score 114)
232 - 249	729.3499	2185.0278	2185.0249	1	0	K.EVMQEVAQLSQFDEELYK.V	(Ions score 64)
232 - 249	1093.5212	2185.0278	2185.0249	1	0	K.EVMQEVAQLSQFDEELYK.V	(Ions score 81)
232 - 249	729.3499	2185.0279	2185.0249	1	0	K.EVMQEVAQLSQFDEELYK.V	(Ions score 61)
232 - 249	1093.5212	2185.0279	2185.0249	1	0	K.EVMQEVAQLSQFDEELYK.V	(Ions score 91)
232 - 249	729.3500	2185.0280	2185.0249	1	0	K.EVMQEVAQLSQFDEELYK.V	(Ions score 64)
232 - 249	729.3500	2185.0281	2185.0249	1	0	K.EVMQEVAQLSQFDEELYK.V	(Ions score 74)
232 - 249	729.3500	2185.0282	2185.0249	2	0	K.EVMQEVAQLSQFDEELYK.V	(Ions score 63)
232 - 249	729.3500	2185.0282	2185.0249	2	0	K.EVMQEVAQLSQFDEELYK.V	(Ions score 66)

232 - 249	729.3500	2185.0282	2185.0249	2	0	K.EVMQEVAQLSQFDEELYK.V	(Ions score 57)
232 - 249	1093.5214	2185.0282	2185.0249	2	0	K.EVMQEVAQLSQFDEELYK.V	(Ions score 101)
232 - 249	1093.5214	2185.0282	2185.0249	2	0	K.EVMQEVAQLSQFDEELYK.V	(Ions score 99)
232 - 249	1093.5214	2185.0283	2185.0249	2	0	K.EVMQEVAQLSQFDEELYK.V	(Ions score 86)
232 - 249	729.3500	2185.0283	2185.0249	2	0	K.EVMQEVAQLSQFDEELYK.V	(Ions score 49)
232 - 249	729.3501	2185.0284	2185.0249	2	0	K.EVMQEVAQLSQFDEELYK.V	(Ions score 51)
232 - 249	729.3501	2185.0284	2185.0249	2	0	K.EVMQEVAQLSQFDEELYK.V	(Ions score 63)
232 - 249	1093.5215	2185.0284	2185.0249	2	0	K.EVMQEVAQLSQFDEELYK.V	(Ions score 26)
232 - 249	729.3501	2185.0284	2185.0249	2	0	K.EVMQEVAQLSQFDEELYK.V	(Ions score 61)
232 - 249	729.3501	2185.0285	2185.0249	2	0	K.EVMQEVAQLSQFDEELYK.V	(Ions score 92)
232 - 249	729.3501	2185.0285	2185.0249	2	0	K.EVMQEVAQLSQFDEELYK.V	(Ions score 80)
232 - 249	729.3501	2185.0285	2185.0249	2	0	K.EVMQEVAQLSQFDEELYK.V	(Ions score 73)
232 - 249	1093.5216	2185.0286	2185.0249	2	0	K.EVMQEVAQLSQFDEELYK.V	(Ions score 102)
232 - 249	729.3502	2185.0286	2185.0249	2	0	K.EVMQEVAQLSQFDEELYK.V	(Ions score 38)
232 - 249	1093.5216	2185.0287	2185.0249	2	0	K.EVMQEVAQLSQFDEELYK.V	(Ions score 87)
232 - 249	1093.5217	2185.0288	2185.0249	2	0	K.EVMQEVAQLSQFDEELYK.V	(Ions score 88)
232 - 249	729.3502	2185.0288	2185.0249	2	0	K.EVMQEVAQLSQFDEELYK.V	(Ions score 64)
232 - 249	1093.5217	2185.0289	2185.0249	2	0	K.EVMQEVAQLSQFDEELYK.V	(Ions score 80)
232 - 249	1093.5217	2185.0289	2185.0249	2	0	K.EVMQEVAQLSQFDEELYK.V	(Ions score 93)
232 - 249	1093.5217	2185.0289	2185.0249	2	0	K.EVMQEVAQLSQFDEELYK.V	(Ions score 71)
232 - 249	1093.5217	2185.0289	2185.0249	2	0	K.EVMQEVAQLSQFDEELYK.V	(Ions score 78)
232 - 249	729.3502	2185.0289	2185.0249	2	0	K.EVMQEVAQLSQFDEELYK.V	(Ions score 64)
232 - 249	729.3503	2185.0291	2185.0249	2	0	K.EVMQEVAQLSQFDEELYK.V	(Ions score 58)
232 - 249	1093.5219	2185.0291	2185.0249	2	0	K.EVMQEVAQLSQFDEELYK.V	(Ions score 96)
232 - 249	1093.5219	2185.0291	2185.0249	2	0	K.EVMQEVAQLSQFDEELYK.V	(Ions score 94)
232 - 249	1093.5219	2185.0292	2185.0249	2	0	K.EVMQEVAQLSQFDEELYK.V	(Ions score 84)
232 - 249	1093.5219	2185.0292	2185.0249	2	0	K.EVMQEVAQLSQFDEELYK.V	(Ions score 84)
232 - 249	729.3503	2185.0292	2185.0249	2	0	K.EVMQEVAQLSQFDEELYK.V	(Ions score 47)
232 - 249	1093.5219	2185.0292	2185.0249	2	0	K.EVMQEVAQLSQFDEELYK.V	(Ions score 99)
232 - 249	729.3504	2185.0292	2185.0249	2	0	K.EVMQEVAQLSQFDEELYK.V	(Ions score 65)
232 - 249	729.3504	2185.0293	2185.0249	2	0	K.EVMQEVAQLSQFDEELYK.V	(Ions score 62)
232 - 249	1093.5219	2185.0293	2185.0249	2	0	K.EVMQEVAQLSQFDEELYK.V	(Ions score 67)
232 - 249	729.3504	2185.0293	2185.0249	2	0	K.EVMQEVAQLSQFDEELYK.V	(Ions score 66)
232 - 249	729.3504	2185.0293	2185.0249	2	0	K.EVMQEVAQLSQFDEELYK.V	(Ions score 66)
232 - 249	1093.5220	2185.0294	2185.0249	2	0	K.EVMQEVAQLSQFDEELYK.V	(Ions score 87)
232 - 249	1093.5220	2185.0295	2185.0249	2	0	K.EVMQEVAQLSQFDEELYK.V	(Ions score 80)
232 - 249	1093.5220	2185.0295	2185.0249	2	0	K.EVMQEVAQLSQFDEELYK.V	(Ions score 99)
232 - 249	1093.5221	2185.0296	2185.0249	2	0	K.EVMQEVAQLSQFDEELYK.V	(Ions score 59)
232 - 249	729.3505	2185.0296	2185.0249	2	0	K.EVMQEVAQLSQFDEELYK.V	(Ions score 63)
232 - 249	729.3505	2185.0297	2185.0249	2	0	K.EVMQEVAQLSQFDEELYK.V	(Ions score 73)
232 - 249	1093.5221	2185.0297	2185.0249	2	0	K.EVMQEVAQLSQFDEELYK.V	(Ions score 84)
232 - 249	729.3505	2185.0298	2185.0249	2	0	K.EVMQEVAQLSQFDEELYK.V	(Ions score 56)
232 - 249	1093.5222	2185.0298	2185.0249	2	0	K.EVMQEVAQLSQFDEELYK.V	(Ions score 80)
232 - 249	729.3506	2185.0298	2185.0249	2	0	K.EVMQEVAQLSQFDEELYK.V	(Ions score 59)
232 - 249	729.3506	2185.0298	2185.0249	2	0	K.EVMQEVAQLSQFDEELYK.V	(Ions score 64)
232 - 249	729.3506	2185.0299	2185.0249	2	0	K.EVMQEVAQLSQFDEELYK.V	(Ions score 68)
232 - 249	729.3506	2185.0300	2185.0249	2	0	K.EVMQEVAQLSQFDEELYK.V	(Ions score 65)
232 - 249	1093.5223	2185.0300	2185.0249	2	0	K.EVMQEVAQLSQFDEELYK.V	(Ions score 102)
232 - 249	1093.5223	2185.0300	2185.0249	2	0	K.EVMQEVAQLSQFDEELYK.V	(Ions score 83)
232 - 249	1093.5223	2185.0300	2185.0249	2	0	K.EVMQEVAQLSQFDEELYK.V	(Ions score 84)
232 - 249	729.3506	2185.0301	2185.0249	2	0	K.EVMQEVAQLSQFDEELYK.V	(Ions score 65)
232 - 249	729.3506	2185.0301	2185.0249	2	0	K.EVMQEVAQLSQFDEELYK.V	(Ions score 64)
232 - 249	1093.5224	2185.0302	2185.0249	2	0	K.EVMQEVAQLSQFDEELYK.V	(Ions score 113)
232 - 249	1093.5224	2185.0303	2185.0249	2	0	K.EVMQEVAQLSQFDEELYK.V	(Ions score 59)
232 - 249	729.3507	2185.0303	2185.0249	2	0	K.EVMQEVAQLSQFDEELYK.V	(Ions score 57)
232 - 249	729.3507	2185.0303	2185.0249	2	0	K.EVMQEVAQLSQFDEELYK.V	(Ions score 82)
232 - 249	729.3507	2185.0303	2185.0249	3	0	K.EVMQEVAQLSQFDEELYK.V	(Ions score 75)
232 - 249	729.3507	2185.0304	2185.0249	3	0	K.EVMQEVAQLSQFDEELYK.V	(Ions score 77)

232 - 249	1093.5225	2185.0304	2185.0249	3	0	K.EVMQEVAQLSQFDEELYK.V	(Ions score 80)
232 - 249	1093.5225	2185.0304	2185.0249	3	0	K.EVMQEVAQLSQFDEELYK.V	(Ions score 96)
232 - 249	1093.5225	2185.0304	2185.0249	3	0	K.EVMQEVAQLSQFDEELYK.V	(Ions score 90)
232 - 249	1093.5225	2185.0304	2185.0249	3	0	K.EVMQEVAQLSQFDEELYK.V	(Ions score 81)
232 - 249	729.3508	2185.0305	2185.0249	3	0	K.EVMQEVAQLSQFDEELYK.V	(Ions score 70)
232 - 249	1093.5225	2185.0305	2185.0249	3	0	K.EVMQEVAQLSQFDEELYK.V	(Ions score 79)
232 - 249	729.3508	2185.0305	2185.0249	3	0	K.EVMQEVAQLSQFDEELYK.V	(Ions score 75)
232 - 249	729.3508	2185.0305	2185.0249	3	0	K.EVMQEVAQLSQFDEELYK.V	(Ions score 67)
232 - 249	729.3508	2185.0306	2185.0249	3	0	K.EVMQEVAQLSQFDEELYK.V	(Ions score 66)
232 - 249	729.3508	2185.0307	2185.0249	3	0	K.EVMQEVAQLSQFDEELYK.V	(Ions score 73)
232 - 249	729.3508	2185.0307	2185.0249	3	0	K.EVMQEVAQLSQFDEELYK.V	(Ions score 74)
232 - 249	1093.5226	2185.0307	2185.0249	3	0	K.EVMQEVAQLSQFDEELYK.V	(Ions score 84)
232 - 249	1093.5227	2185.0308	2185.0249	3	0	K.EVMQEVAQLSQFDEELYK.V	(Ions score 85)
232 - 249	729.3509	2185.0308	2185.0249	3	0	K.EVMQEVAQLSQFDEELYK.V	(Ions score 62)
232 - 249	729.3509	2185.0308	2185.0249	3	0	K.EVMQEVAQLSQFDEELYK.V	(Ions score 77)
232 - 249	1093.5227	2185.0309	2185.0249	3	0	K.EVMQEVAQLSQFDEELYK.V	(Ions score 76)
232 - 249	729.3509	2185.0310	2185.0249	3	0	K.EVMQEVAQLSQFDEELYK.V	(Ions score 63)
232 - 249	729.3509	2185.0310	2185.0249	3	0	K.EVMQEVAQLSQFDEELYK.V	(Ions score 75)
232 - 249	1093.5228	2185.0310	2185.0249	3	0	K.EVMQEVAQLSQFDEELYK.V	(Ions score 78)
232 - 249	729.3510	2185.0310	2185.0249	3	0	K.EVMQEVAQLSQFDEELYK.V	(Ions score 60)
232 - 249	729.3510	2185.0311	2185.0249	3	0	K.EVMQEVAQLSQFDEELYK.V	(Ions score 72)
232 - 249	1093.5228	2185.0311	2185.0249	3	0	K.EVMQEVAQLSQFDEELYK.V	(Ions score 79)
232 - 249	1093.5228	2185.0311	2185.0249	3	0	K.EVMQEVAQLSQFDEELYK.V	(Ions score 89)
232 - 249	729.3510	2185.0312	2185.0249	3	0	K.EVMQEVAQLSQFDEELYK.V	(Ions score 54)
232 - 249	1093.5229	2185.0312	2185.0249	3	0	K.EVMQEVAQLSQFDEELYK.V	(Ions score 88)
232 - 249	729.3510	2185.0312	2185.0249	3	0	K.EVMQEVAQLSQFDEELYK.V	(Ions score 77)
232 - 249	729.3510	2185.0312	2185.0249	3	0	K.EVMQEVAQLSQFDEELYK.V	(Ions score 92)
232 - 249	1093.5230	2185.0314	2185.0249	3	0	K.EVMQEVAQLSQFDEELYK.V	(Ions score 97)
232 - 249	1093.5230	2185.0315	2185.0249	3	0	K.EVMQEVAQLSQFDEELYK.V	(Ions score 84)
232 - 249	1093.5230	2185.0315	2185.0249	3	0	K.EVMQEVAQLSQFDEELYK.V	(Ions score 83)
232 - 249	1093.5230	2185.0315	2185.0249	3	0	K.EVMQEVAQLSQFDEELYK.V	(Ions score 88)
232 - 249	729.3511	2185.0316	2185.0249	3	0	K.EVMQEVAQLSQFDEELYK.V	(Ions score 62)
232 - 249	729.3511	2185.0316	2185.0249	3	0	K.EVMQEVAQLSQFDEELYK.V	(Ions score 77)
232 - 249	729.3511	2185.0316	2185.0249	3	0	K.EVMQEVAQLSQFDEELYK.V	(Ions score 83)
232 - 249	729.3511	2185.0316	2185.0249	3	0	K.EVMQEVAQLSQFDEELYK.V	(Ions score 66)
232 - 249	729.3512	2185.0316	2185.0249	3	0	K.EVMQEVAQLSQFDEELYK.V	(Ions score 75)
232 - 249	729.3512	2185.0317	2185.0249	3	0	K.EVMQEVAQLSQFDEELYK.V	(Ions score 62)
232 - 249	729.3512	2185.0317	2185.0249	3	0	K.EVMQEVAQLSQFDEELYK.V	(Ions score 64)
232 - 249	729.3512	2185.0317	2185.0249	3	0	K.EVMQEVAQLSQFDEELYK.V	(Ions score 60)
232 - 249	729.3512	2185.0317	2185.0249	3	0	K.EVMQEVAQLSQFDEELYK.V	(Ions score 65)
232 - 249	1093.5231	2185.0317	2185.0249	3	0	K.EVMQEVAQLSQFDEELYK.V	(Ions score 85)
232 - 249	729.3512	2185.0318	2185.0249	3	0	K.EVMQEVAQLSQFDEELYK.V	(Ions score 61)
232 - 249	1093.5232	2185.0318	2185.0249	3	0	K.EVMQEVAQLSQFDEELYK.V	(Ions score 100)
232 - 249	1093.5232	2185.0319	2185.0249	3	0	K.EVMQEVAQLSQFDEELYK.V	(Ions score 95)
232 - 249	1093.5232	2185.0319	2185.0249	3	0	K.EVMQEVAQLSQFDEELYK.V	(Ions score 95)
232 - 249	729.3513	2185.0320	2185.0249	3	0	K.EVMQEVAQLSQFDEELYK.V	(Ions score 54)
232 - 249	1093.5233	2185.0320	2185.0249	3	0	K.EVMQEVAQLSQFDEELYK.V	(Ions score 84)
232 - 249	729.3513	2185.0320	2185.0249	3	0	K.EVMQEVAQLSQFDEELYK.V	(Ions score 60)
232 - 249	1093.5233	2185.0321	2185.0249	3	0	K.EVMQEVAQLSQFDEELYK.V	(Ions score 83)
232 - 249	729.3513	2185.0321	2185.0249	3	0	K.EVMQEVAQLSQFDEELYK.V	(Ions score 100)
232 - 249	729.3513	2185.0321	2185.0249	3	0	K.EVMQEVAQLSQFDEELYK.V	(Ions score 64)
232 - 249	729.3513	2185.0322	2185.0249	3	0	K.EVMQEVAQLSQFDEELYK.V	(Ions score 52)
232 - 249	729.3514	2185.0322	2185.0249	3	0	K.EVMQEVAQLSQFDEELYK.V	(Ions score 63)
232 - 249	1093.5234	2185.0322	2185.0249	3	0	K.EVMQEVAQLSQFDEELYK.V	(Ions score 80)
232 - 249	729.3514	2185.0323	2185.0249	3	0	K.EVMQEVAQLSQFDEELYK.V	(Ions score 68)
232 - 249	729.3514	2185.0323	2185.0249	3	0	K.EVMQEVAQLSQFDEELYK.V	(Ions score 63)
232 - 249	729.3514	2185.0323	2185.0249	3	0	K.EVMQEVAQLSQFDEELYK.V	(Ions score 77)
232 - 249	729.3514	2185.0324	2185.0249	3	0	K.EVMQEVAQLSQFDEELYK.V	(Ions score 64)

232 - 249	1093.5235	2185.0324	2185.0249	3	0	K.EVMQEVAQLSQFDEELYK.V	(Ions score 88)
232 - 249	729.3514	2185.0325	2185.0249	3	0	K.EVMQEVAQLSQFDEELYK.V	(Ions score 63)
232 - 249	1093.5235	2185.0325	2185.0249	3	0	K.EVMQEVAQLSQFDEELYK.V	(Ions score 96)
232 - 249	729.3514	2185.0325	2185.0249	3	0	K.EVMQEVAQLSQFDEELYK.V	(Ions score 63)
232 - 249	729.3515	2185.0325	2185.0249	4	0	K.EVMQEVAQLSQFDEELYK.V	(Ions score 64)
232 - 249	1093.5236	2185.0326	2185.0249	4	0	K.EVMQEVAQLSQFDEELYK.V	(Ions score 89)
232 - 249	1093.5236	2185.0326	2185.0249	4	0	K.EVMQEVAQLSQFDEELYK.V	(Ions score 80)
232 - 249	729.3515	2185.0326	2185.0249	4	0	K.EVMQEVAQLSQFDEELYK.V	(Ions score 80)
232 - 249	729.3515	2185.0326	2185.0249	4	0	K.EVMQEVAQLSQFDEELYK.V	(Ions score 62)
232 - 249	1093.5236	2185.0327	2185.0249	4	0	K.EVMQEVAQLSQFDEELYK.V	(Ions score 79)
232 - 249	729.3515	2185.0327	2185.0249	4	0	K.EVMQEVAQLSQFDEELYK.V	(Ions score 65)
232 - 249	729.3515	2185.0327	2185.0249	4	0	K.EVMQEVAQLSQFDEELYK.V	(Ions score 52)
232 - 249	1093.5236	2185.0327	2185.0249	4	0	K.EVMQEVAQLSQFDEELYK.V	(Ions score 84)
232 - 249	729.3515	2185.0327	2185.0249	4	0	K.EVMQEVAQLSQFDEELYK.V	(Ions score 64)
232 - 249	1093.5237	2185.0328	2185.0249	4	0	K.EVMQEVAQLSQFDEELYK.V	(Ions score 96)
232 - 249	1093.5237	2185.0328	2185.0249	4	0	K.EVMQEVAQLSQFDEELYK.V	(Ions score 98)
232 - 249	729.3516	2185.0329	2185.0249	4	0	K.EVMQEVAQLSQFDEELYK.V	(Ions score 72)
232 - 249	729.3516	2185.0329	2185.0249	4	0	K.EVMQEVAQLSQFDEELYK.V	(Ions score 77)
232 - 249	1093.5237	2185.0329	2185.0249	4	0	K.EVMQEVAQLSQFDEELYK.V	(Ions score 96)
232 - 249	1093.5237	2185.0329	2185.0249	4	0	K.EVMQEVAQLSQFDEELYK.V	(Ions score 76)
232 - 249	729.3516	2185.0330	2185.0249	4	0	K.EVMQEVAQLSQFDEELYK.V	(Ions score 79)
232 - 249	729.3516	2185.0331	2185.0249	4	0	K.EVMQEVAQLSQFDEELYK.V	(Ions score 89)
232 - 249	729.3516	2185.0331	2185.0249	4	0	K.EVMQEVAQLSQFDEELYK.V	(Ions score 64)
232 - 249	729.3516	2185.0331	2185.0249	4	0	K.EVMQEVAQLSQFDEELYK.V	(Ions score 92)
232 - 249	729.3517	2185.0331	2185.0249	4	0	K.EVMQEVAQLSQFDEELYK.V	(Ions score 53)
232 - 249	729.3517	2185.0331	2185.0249	4	0	K.EVMQEVAQLSQFDEELYK.V	(Ions score 77)
232 - 249	1093.5238	2185.0331	2185.0249	4	0	K.EVMQEVAQLSQFDEELYK.V	(Ions score 100)
232 - 249	729.3517	2185.0331	2185.0249	4	0	K.EVMQEVAQLSQFDEELYK.V	(Ions score 61)
232 - 249	1093.5239	2185.0332	2185.0249	4	0	K.EVMQEVAQLSQFDEELYK.V	(Ions score 80)
232 - 249	729.3517	2185.0332	2185.0249	4	0	K.EVMQEVAQLSQFDEELYK.V	(Ions score 48)
232 - 249	1093.5239	2185.0332	2185.0249	4	0	K.EVMQEVAQLSQFDEELYK.V	(Ions score 92)
232 - 249	729.3517	2185.0333	2185.0249	4	0	K.EVMQEVAQLSQFDEELYK.V	(Ions score 57)
232 - 249	729.3517	2185.0333	2185.0249	4	0	K.EVMQEVAQLSQFDEELYK.V	(Ions score 64)
232 - 249	729.3517	2185.0333	2185.0249	4	0	K.EVMQEVAQLSQFDEELYK.V	(Ions score 66)
232 - 249	729.3517	2185.0334	2185.0249	4	0	K.EVMQEVAQLSQFDEELYK.V	(Ions score 72)
232 - 249	729.3517	2185.0334	2185.0249	4	0	K.EVMQEVAQLSQFDEELYK.V	(Ions score 79)
232 - 249	1093.5240	2185.0335	2185.0249	4	0	K.EVMQEVAQLSQFDEELYK.V	(Ions score 80)
232 - 249	729.3518	2185.0336	2185.0249	4	0	K.EVMQEVAQLSQFDEELYK.V	(Ions score 89)
232 - 249	1093.5241	2185.0336	2185.0249	4	0	K.EVMQEVAQLSQFDEELYK.V	(Ions score 90)
232 - 249	729.3518	2185.0336	2185.0249	4	0	K.EVMQEVAQLSQFDEELYK.V	(Ions score 64)
232 - 249	1093.5241	2185.0336	2185.0249	4	0	K.EVMQEVAQLSQFDEELYK.V	(Ions score 76)
232 - 249	1093.5241	2185.0337	2185.0249	4	0	K.EVMQEVAQLSQFDEELYK.V	(Ions score 89)
232 - 249	729.3519	2185.0338	2185.0249	4	0	K.EVMQEVAQLSQFDEELYK.V	(Ions score 21)
232 - 249	1093.5242	2185.0338	2185.0249	4	0	K.EVMQEVAQLSQFDEELYK.V	(Ions score 96)
232 - 249	1093.5242	2185.0338	2185.0249	4	0	K.EVMQEVAQLSQFDEELYK.V	(Ions score 84)
232 - 249	729.3519	2185.0338	2185.0249	4	0	K.EVMQEVAQLSQFDEELYK.V	(Ions score 66)
232 - 249	729.3519	2185.0339	2185.0249	4	0	K.EVMQEVAQLSQFDEELYK.V	(Ions score 53)
232 - 249	1093.5242	2185.0339	2185.0249	4	0	K.EVMQEVAQLSQFDEELYK.V	(Ions score 86)
232 - 249	729.3519	2185.0339	2185.0249	4	0	K.EVMQEVAQLSQFDEELYK.V	(Ions score 66)
232 - 249	729.3519	2185.0339	2185.0249	4	0	K.EVMQEVAQLSQFDEELYK.V	(Ions score 78)
232 - 249	1093.5243	2185.0340	2185.0249	4	0	K.EVMQEVAQLSQFDEELYK.V	(Ions score 83)
232 - 249	729.3519	2185.0340	2185.0249	4	0	K.EVMQEVAQLSQFDEELYK.V	(Ions score 68)
232 - 249	1093.5243	2185.0340	2185.0249	4	0	K.EVMQEVAQLSQFDEELYK.V	(Ions score 83)
232 - 249	1093.5243	2185.0340	2185.0249	4	0	K.EVMQEVAQLSQFDEELYK.V	(Ions score 97)
232 - 249	729.3520	2185.0342	2185.0249	4	0	K.EVMQEVAQLSQFDEELYK.V	(Ions score 68)
232 - 249	1093.5244	2185.0342	2185.0249	4	0	K.EVMQEVAQLSQFDEELYK.V	(Ions score 72)
232 - 249	729.3520	2185.0342	2185.0249	4	0	K.EVMQEVAQLSQFDEELYK.V	(Ions score 74)
232 - 249	729.3520	2185.0342	2185.0249	4	0	K.EVMQEVAQLSQFDEELYK.V	(Ions score 60)

232 - 249	1093.5244	2185.0343	2185.0249	4	0	K.EVMQEVAQLSQFDEELYK.V		(Ions score 68)
232 - 249	729.3521	2185.0344	2185.0249	4	0	K.EVMQEVAQLSQFDEELYK.V		(Ions score 66)
232 - 249	1093.5245	2185.0344	2185.0249	4	0	K.EVMQEVAQLSQFDEELYK.V		(Ions score 85)
232 - 249	729.3521	2185.0344	2185.0249	4	0	K.EVMQEVAQLSQFDEELYK.V		(Ions score 59)
232 - 249	729.3521	2185.0344	2185.0249	4	0	K.EVMQEVAQLSQFDEELYK.V		(Ions score 64)
232 - 249	729.3521	2185.0345	2185.0249	4	0	K.EVMQEVAQLSQFDEELYK.V		(Ions score 58)
232 - 249	729.3521	2185.0345	2185.0249	4	0	K.EVMQEVAQLSQFDEELYK.V		(Ions score 64)
232 - 249	729.3522	2185.0346	2185.0249	4	0	K.EVMQEVAQLSQFDEELYK.V		(Ions score 67)
232 - 249	729.3522	2185.0346	2185.0249	4	0	K.EVMQEVAQLSQFDEELYK.V		(Ions score 78)
232 - 249	729.3522	2185.0347	2185.0249	4	0	K.EVMQEVAQLSQFDEELYK.V		(Ions score 77)
232 - 249	729.3522	2185.0347	2185.0249	5	0	K.EVMQEVAQLSQFDEELYK.V		(Ions score 60)
232 - 249	1093.5247	2185.0349	2185.0249	5	0	K.EVMQEVAQLSQFDEELYK.V		(Ions score 113)
232 - 249	729.3522	2185.0349	2185.0249	5	0	K.EVMQEVAQLSQFDEELYK.V		(Ions score 73)
232 - 249	729.3522	2185.0349	2185.0249	5	0	K.EVMQEVAQLSQFDEELYK.V		(Ions score 77)
232 - 249	729.3522	2185.0349	2185.0249	5	0	K.EVMQEVAQLSQFDEELYK.V		(Ions score 63)
232 - 249	729.3523	2185.0349	2185.0249	5	0	K.EVMQEVAQLSQFDEELYK.V		(Ions score 70)
232 - 249	729.3523	2185.0350	2185.0249	5	0	K.EVMQEVAQLSQFDEELYK.V		(Ions score 75)
232 - 249	729.3523	2185.0350	2185.0249	5	0	K.EVMQEVAQLSQFDEELYK.V		(Ions score 65)
232 - 249	729.3523	2185.0350	2185.0249	5	0	K.EVMQEVAQLSQFDEELYK.V		(Ions score 67)
232 - 249	729.3523	2185.0351	2185.0249	5	0	K.EVMQEVAQLSQFDEELYK.V		(Ions score 78)
232 - 249	729.3523	2185.0351	2185.0249	5	0	K.EVMQEVAQLSQFDEELYK.V		(Ions score 55)
232 - 249	1093.5249	2185.0352	2185.0249	5	0	K.EVMQEVAQLSQFDEELYK.V		(Ions score 88)
232 - 249	1093.5249	2185.0353	2185.0249	5	0	K.EVMQEVAQLSQFDEELYK.V		(Ions score 79)
232 - 249	729.3524	2185.0353	2185.0249	5	0	K.EVMQEVAQLSQFDEELYK.V		(Ions score 67)
232 - 249	729.3524	2185.0353	2185.0249	5	0	K.EVMQEVAQLSQFDEELYK.V		(Ions score 65)
232 - 249	729.3524	2185.0353	2185.0249	5	0	K.EVMQEVAQLSQFDEELYK.V		(Ions score 75)
232 - 249	1093.5250	2185.0355	2185.0249	5	0	K.EVMQEVAQLSQFDEELYK.V		(Ions score 79)
232 - 249	729.3525	2185.0356	2185.0249	5	0	K.EVMQEVAQLSQFDEELYK.V		(Ions score 38)
232 - 249	729.3525	2185.0357	2185.0249	5	0	K.EVMQEVAQLSQFDEELYK.V		(Ions score 94)
232 - 249	729.3525	2185.0357	2185.0249	5	0	K.EVMQEVAQLSQFDEELYK.V		(Ions score 73)
232 - 249	1093.5251	2185.0357	2185.0249	5	0	K.EVMQEVAQLSQFDEELYK.V		(Ions score 85)
232 - 249	729.3525	2185.0357	2185.0249	5	0	K.EVMQEVAQLSQFDEELYK.V		(Ions score 94)
232 - 249	729.3525	2185.0358	2185.0249	5	0	K.EVMQEVAQLSQFDEELYK.V		(Ions score 62)
232 - 249	1101.5126	2201.0106	2201.0198	-4	0	K.EVMQEVAQLSQFDEELYK.V	Oxidation (M)	(Ions score 50)
232 - 249	1101.5155	2201.0165	2201.0198	-1	0	K.EVMQEVAQLSQFDEELYK.V	Oxidation (M)	(Ions score 70)
232 - 249	1101.5156	2201.0166	2201.0198	-1	0	K.EVMQEVAQLSQFDEELYK.V	Oxidation (M)	(Ions score 57)
232 - 249	734.6796	2201.0169	2201.0198	-1	0	K.EVMQEVAQLSQFDEELYK.V	Oxidation (M)	(Ions score 48)
232 - 249	1101.5157	2201.0169	2201.0198	-1	0	K.EVMQEVAQLSQFDEELYK.V	Oxidation (M)	(Ions score 59)
232 - 249	734.6797	2201.0174	2201.0198	-1	0	K.EVMQEVAQLSQFDEELYK.V	Oxidation (M)	(Ions score 38)
232 - 249	734.6797	2201.0174	2201.0198	-1	0	K.EVMQEVAQLSQFDEELYK.V	Oxidation (M)	(Ions score 36)
232 - 249	1101.5161	2201.0176	2201.0198	-1	0	K.EVMQEVAQLSQFDEELYK.V	Oxidation (M)	(Ions score 66)
232 - 249	1101.5161	2201.0177	2201.0198	-1	0	K.EVMQEVAQLSQFDEELYK.V	Oxidation (M)	(Ions score 85)
232 - 249	1101.5161	2201.0177	2201.0198	-1	0	K.EVMQEVAQLSQFDEELYK.V	Oxidation (M)	(Ions score 70)
232 - 249	1101.5162	2201.0178	2201.0198	-1	0	K.EVMQEVAQLSQFDEELYK.V	Oxidation (M)	(Ions score 58)
232 - 249	734.6799	2201.0178	2201.0198	-1	0	K.EVMQEVAQLSQFDEELYK.V	Oxidation (M)	(Ions score 32)
232 - 249	734.6801	2201.0183	2201.0198	-1	0	K.EVMQEVAQLSQFDEELYK.V	Oxidation (M)	(Ions score 29)
232 - 249	734.6801	2201.0186	2201.0198	-1	0	K.EVMQEVAQLSQFDEELYK.V	Oxidation (M)	(Ions score 37)
232 - 249	734.6801	2201.0186	2201.0198	-1	0	K.EVMQEVAQLSQFDEELYK.V	Oxidation (M)	(Ions score 39)
232 - 249	734.6802	2201.0187	2201.0198	-0	0	K.EVMQEVAQLSQFDEELYK.V	Oxidation (M)	(Ions score 59)
232 - 249	1101.5167	2201.0189	2201.0198	-0	0	K.EVMQEVAQLSQFDEELYK.V	Oxidation (M)	(Ions score 70)
232 - 249	734.6802	2201.0189	2201.0198	-0	0	K.EVMQEVAQLSQFDEELYK.V	Oxidation (M)	(Ions score 58)
232 - 249	734.6802	2201.0189	2201.0198	-0	0	K.EVMQEVAQLSQFDEELYK.V	Oxidation (M)	(Ions score 76)
232 - 249	1101.5168	2201.0190	2201.0198	-0	0	K.EVMQEVAQLSQFDEELYK.V	Oxidation (M)	(Ions score 56)
232 - 249	734.6803	2201.0191	2201.0198	-0	0	K.EVMQEVAQLSQFDEELYK.V	Oxidation (M)	(Ions score 47)
232 - 249	734.6804	2201.0192	2201.0198	-0	0	K.EVMQEVAQLSQFDEELYK.V	Oxidation (M)	(Ions score 47)
232 - 249	734.6804	2201.0192	2201.0198	-0	0	K.EVMQEVAQLSQFDEELYK.V	Oxidation (M)	(Ions score 61)
232 - 249	1101.5170	2201.0194	2201.0198	-0	0	K.EVMQEVAQLSQFDEELYK.V	Oxidation (M)	(Ions score 56)
232 - 249	734.6805	2201.0195	2201.0198	-0	0	K.EVMQEVAQLSQFDEELYK.V	Oxidation (M)	(Ions score 34)

232 - 249	734.6805	2201.0196	2201.0198	-0	0	K.EVMQEVAQLSQFDEELYK.V	Oxidation (M)	(Ions score 40)
232 - 249	1101.5171	2201.0196	2201.0198	-0	0	K.EVMQEVAQLSQFDEELYK.V	Oxidation (M)	(Ions score 75)
232 - 249	734.6805	2201.0197	2201.0198	-0	0	K.EVMQEVAQLSQFDEELYK.V	Oxidation (M)	(Ions score 25)
232 - 249	1101.5171	2201.0197	2201.0198	-0	0	K.EVMQEVAQLSQFDEELYK.V	Oxidation (M)	(Ions score 82)
232 - 249	1101.5172	2201.0198	2201.0198	-0	0	K.EVMQEVAQLSQFDEELYK.V	Oxidation (M)	(Ions score 70)
232 - 249	1101.5172	2201.0198	2201.0198	-0	0	K.EVMQEVAQLSQFDEELYK.V	Oxidation (M)	(Ions score 58)
232 - 249	1101.5172	2201.0199	2201.0198	0	0	K.EVMQEVAQLSQFDEELYK.V	Oxidation (M)	(Ions score 86)
232 - 249	1101.5172	2201.0199	2201.0198	0	0	K.EVMQEVAQLSQFDEELYK.V	Oxidation (M)	(Ions score 81)
232 - 249	734.6806	2201.0200	2201.0198	0	0	K.EVMQEVAQLSQFDEELYK.V	Oxidation (M)	(Ions score 47)
232 - 249	1101.5173	2201.0200	2201.0198	0	0	K.EVMQEVAQLSQFDEELYK.V	Oxidation (M)	(Ions score 72)
232 - 249	1101.5173	2201.0200	2201.0198	0	0	K.EVMQEVAQLSQFDEELYK.V	Oxidation (M)	(Ions score 66)
232 - 249	734.6806	2201.0201	2201.0198	0	0	K.EVMQEVAQLSQFDEELYK.V	Oxidation (M)	(Ions score 34)
232 - 249	734.6807	2201.0202	2201.0198	0	0	K.EVMQEVAQLSQFDEELYK.V	Oxidation (M)	(Ions score 53)
232 - 249	734.6807	2201.0202	2201.0198	0	0	K.EVMQEVAQLSQFDEELYK.V	Oxidation (M)	(Ions score 26)
232 - 249	1101.5174	2201.0203	2201.0198	0	0	K.EVMQEVAQLSQFDEELYK.V	Oxidation (M)	(Ions score 67)
232 - 249	734.6807	2201.0203	2201.0198	0	0	K.EVMQEVAQLSQFDEELYK.V	Oxidation (M)	(Ions score 26)
232 - 249	734.6807	2201.0203	2201.0198	0	0	K.EVMQEVAQLSQFDEELYK.V	Oxidation (M)	(Ions score 41)
232 - 249	1101.5175	2201.0204	2201.0198	0	0	K.EVMQEVAQLSQFDEELYK.V	Oxidation (M)	(Ions score 70)
232 - 249	1101.5175	2201.0205	2201.0198	0	0	K.EVMQEVAQLSQFDEELYK.V	Oxidation (M)	(Ions score 71)
232 - 249	734.6808	2201.0205	2201.0198	0	0	K.EVMQEVAQLSQFDEELYK.V	Oxidation (M)	(Ions score 39)
232 - 249	1101.5176	2201.0206	2201.0198	0	0	K.EVMQEVAQLSQFDEELYK.V	Oxidation (M)	(Ions score 58)
232 - 249	1101.5176	2201.0206	2201.0198	0	0	K.EVMQEVAQLSQFDEELYK.V	Oxidation (M)	(Ions score 81)
232 - 249	734.6808	2201.0206	2201.0198	0	0	K.EVMQEVAQLSQFDEELYK.V	Oxidation (M)	(Ions score 44)
232 - 249	1101.5176	2201.0207	2201.0198	0	0	K.EVMQEVAQLSQFDEELYK.V	Oxidation (M)	(Ions score 56)
232 - 249	1101.5177	2201.0208	2201.0198	0	0	K.EVMQEVAQLSQFDEELYK.V	Oxidation (M)	(Ions score 67)
232 - 249	734.6809	2201.0209	2201.0198	0	0	K.EVMQEVAQLSQFDEELYK.V	Oxidation (M)	(Ions score 29)
232 - 249	1101.5177	2201.0209	2201.0198	0	0	K.EVMQEVAQLSQFDEELYK.V	Oxidation (M)	(Ions score 66)
232 - 249	734.6810	2201.0211	2201.0198	1	0	K.EVMQEVAQLSQFDEELYK.V	Oxidation (M)	(Ions score 47)
232 - 249	1101.5178	2201.0211	2201.0198	1	0	K.EVMQEVAQLSQFDEELYK.V	Oxidation (M)	(Ions score 56)
232 - 249	1101.5179	2201.0212	2201.0198	1	0	K.EVMQEVAQLSQFDEELYK.V	Oxidation (M)	(Ions score 56)
232 - 249	734.6810	2201.0212	2201.0198	1	0	K.EVMQEVAQLSQFDEELYK.V	Oxidation (M)	(Ions score 27)
232 - 249	1101.5179	2201.0213	2201.0198	1	0	K.EVMQEVAQLSQFDEELYK.V	Oxidation (M)	(Ions score 60)
232 - 249	734.6810	2201.0213	2201.0198	1	0	K.EVMQEVAQLSQFDEELYK.V	Oxidation (M)	(Ions score 30)
232 - 249	1101.5179	2201.0213	2201.0198	1	0	K.EVMQEVAQLSQFDEELYK.V	Oxidation (M)	(Ions score 66)
232 - 249	734.6810	2201.0213	2201.0198	1	0	K.EVMQEVAQLSQFDEELYK.V	Oxidation (M)	(Ions score 38)
232 - 249	1101.5180	2201.0214	2201.0198	1	0	K.EVMQEVAQLSQFDEELYK.V	Oxidation (M)	(Ions score 60)
232 - 249	734.6811	2201.0214	2201.0198	1	0	K.EVMQEVAQLSQFDEELYK.V	Oxidation (M)	(Ions score 38)
232 - 249	1101.5180	2201.0214	2201.0198	1	0	K.EVMQEVAQLSQFDEELYK.V	Oxidation (M)	(Ions score 66)
232 - 249	1101.5180	2201.0214	2201.0198	1	0	K.EVMQEVAQLSQFDEELYK.V	Oxidation (M)	(Ions score 70)
232 - 249	1101.5180	2201.0214	2201.0198	1	0	K.EVMQEVAQLSQFDEELYK.V	Oxidation (M)	(Ions score 74)
232 - 249	1101.5180	2201.0215	2201.0198	1	0	K.EVMQEVAQLSQFDEELYK.V	Oxidation (M)	(Ions score 71)
232 - 249	734.6811	2201.0216	2201.0198	1	0	K.EVMQEVAQLSQFDEELYK.V	Oxidation (M)	(Ions score 21)
232 - 249	1101.5181	2201.0216	2201.0198	1	0	K.EVMQEVAQLSQFDEELYK.V	Oxidation (M)	(Ions score 78)
232 - 249	1101.5181	2201.0217	2201.0198	1	0	K.EVMQEVAQLSQFDEELYK.V	Oxidation (M)	(Ions score 53)
232 - 249	734.6812	2201.0218	2201.0198	1	0	K.EVMQEVAQLSQFDEELYK.V	Oxidation (M)	(Ions score 26)
232 - 249	1101.5182	2201.0218	2201.0198	1	0	K.EVMQEVAQLSQFDEELYK.V	Oxidation (M)	(Ions score 71)
232 - 249	734.6813	2201.0220	2201.0198	1	0	K.EVMQEVAQLSQFDEELYK.V	Oxidation (M)	(Ions score 38)
232 - 249	1101.5183	2201.0220	2201.0198	1	0	K.EVMQEVAQLSQFDEELYK.V	Oxidation (M)	(Ions score 70)
232 - 249	1101.5183	2201.0221	2201.0198	1	0	K.EVMQEVAQLSQFDEELYK.V	Oxidation (M)	(Ions score 67)
232 - 249	734.6813	2201.0222	2201.0198	1	0	K.EVMQEVAQLSQFDEELYK.V	Oxidation (M)	(Ions score 31)
232 - 249	734.6813	2201.0222	2201.0198	1	0	K.EVMQEVAQLSQFDEELYK.V	Oxidation (M)	(Ions score 32)
232 - 249	734.6813	2201.0222	2201.0198	1	0	K.EVMQEVAQLSQFDEELYK.V	Oxidation (M)	(Ions score 41)
232 - 249	1101.5184	2201.0222	2201.0198	1	0	K.EVMQEVAQLSQFDEELYK.V	Oxidation (M)	(Ions score 50)
232 - 249	1101.5184	2201.0222	2201.0198	1	0	K.EVMQEVAQLSQFDEELYK.V	Oxidation (M)	(Ions score 38)
232 - 249	734.6814	2201.0222	2201.0198	1	0	K.EVMQEVAQLSQFDEELYK.V	Oxidation (M)	(Ions score 39)
232 - 249	1101.5184	2201.0222	2201.0198	1	0	K.EVMQEVAQLSQFDEELYK.V	Oxidation (M)	(Ions score 56)
232 - 249	1101.5184	2201.0223	2201.0198	1	0	K.EVMQEVAQLSQFDEELYK.V	Oxidation (M)	(Ions score 70)
232 - 249	734.6814	2201.0223	2201.0198	1	0	K.EVMQEVAQLSQFDEELYK.V	Oxidation (M)	(Ions score 30)

232 - 249	1101.5184	2201.0223	2201.0198	1	0	K.EVMQEVAQLSQFDEELYK.V	Oxidation (M)	(Ions score 66)
232 - 249	734.6814	2201.0223	2201.0198	1	0	K.EVMQEVAQLSQFDEELYK.V	Oxidation (M)	(Ions score 40)
232 - 249	734.6814	2201.0224	2201.0198	1	0	K.EVMQEVAQLSQFDEELYK.V	Oxidation (M)	(Ions score 43)
232 - 249	734.6814	2201.0224	2201.0198	1	0	K.EVMQEVAQLSQFDEELYK.V	Oxidation (M)	(Ions score 38)
232 - 249	1101.5185	2201.0224	2201.0198	1	0	K.EVMQEVAQLSQFDEELYK.V	Oxidation (M)	(Ions score 82)
232 - 249	1101.5185	2201.0225	2201.0198	1	0	K.EVMQEVAQLSQFDEELYK.V	Oxidation (M)	(Ions score 74)
232 - 249	1101.5185	2201.0225	2201.0198	1	0	K.EVMQEVAQLSQFDEELYK.V	Oxidation (M)	(Ions score 67)
232 - 249	734.6815	2201.0225	2201.0198	1	0	K.EVMQEVAQLSQFDEELYK.V	Oxidation (M)	(Ions score 27)
232 - 249	1101.5186	2201.0226	2201.0198	1	0	K.EVMQEVAQLSQFDEELYK.V	Oxidation (M)	(Ions score 70)
232 - 249	1101.5186	2201.0226	2201.0198	1	0	K.EVMQEVAQLSQFDEELYK.V	Oxidation (M)	(Ions score 59)
232 - 249	1101.5186	2201.0226	2201.0198	1	0	K.EVMQEVAQLSQFDEELYK.V	Oxidation (M)	(Ions score 70)
232 - 249	1101.5186	2201.0227	2201.0198	1	0	K.EVMQEVAQLSQFDEELYK.V	Oxidation (M)	(Ions score 58)
232 - 249	1101.5187	2201.0228	2201.0198	1	0	K.EVMQEVAQLSQFDEELYK.V	Oxidation (M)	(Ions score 64)
232 - 249	734.6815	2201.0228	2201.0198	1	0	K.EVMQEVAQLSQFDEELYK.V	Oxidation (M)	(Ions score 45)
232 - 249	734.6815	2201.0228	2201.0198	1	0	K.EVMQEVAQLSQFDEELYK.V	Oxidation (M)	(Ions score 32)
232 - 249	1101.5187	2201.0228	2201.0198	1	0	K.EVMQEVAQLSQFDEELYK.V	Oxidation (M)	(Ions score 47)
232 - 249	734.6816	2201.0229	2201.0198	1	0	K.EVMQEVAQLSQFDEELYK.V	Oxidation (M)	(Ions score 29)
232 - 249	1101.5187	2201.0229	2201.0198	1	0	K.EVMQEVAQLSQFDEELYK.V	Oxidation (M)	(Ions score 49)
232 - 249	1101.5187	2201.0229	2201.0198	1	0	K.EVMQEVAQLSQFDEELYK.V	Oxidation (M)	(Ions score 58)
232 - 249	1101.5187	2201.0229	2201.0198	1	0	K.EVMQEVAQLSQFDEELYK.V	Oxidation (M)	(Ions score 66)
232 - 249	1101.5188	2201.0230	2201.0198	1	0	K.EVMQEVAQLSQFDEELYK.V	Oxidation (M)	(Ions score 62)
232 - 249	734.6816	2201.0230	2201.0198	1	0	K.EVMQEVAQLSQFDEELYK.V	Oxidation (M)	(Ions score 27)
232 - 249	734.6816	2201.0230	2201.0198	1	0	K.EVMQEVAQLSQFDEELYK.V	Oxidation (M)	(Ions score 39)
232 - 249	1101.5188	2201.0230	2201.0198	1	0	K.EVMQEVAQLSQFDEELYK.V	Oxidation (M)	(Ions score 71)
232 - 249	1101.5188	2201.0231	2201.0198	1	0	K.EVMQEVAQLSQFDEELYK.V	Oxidation (M)	(Ions score 70)
232 - 249	1101.5188	2201.0231	2201.0198	1	0	K.EVMQEVAQLSQFDEELYK.V	Oxidation (M)	(Ions score 67)
232 - 249	1101.5188	2201.0231	2201.0198	2	0	K.EVMQEVAQLSQFDEELYK.V	Oxidation (M)	(Ions score 51)
232 - 249	1101.5188	2201.0231	2201.0198	2	0	K.EVMQEVAQLSQFDEELYK.V	Oxidation (M)	(Ions score 81)
232 - 249	1101.5188	2201.0231	2201.0198	2	0	K.EVMQEVAQLSQFDEELYK.V	Oxidation (M)	(Ions score 67)
232 - 249	1101.5188	2201.0231	2201.0198	2	0	K.EVMQEVAQLSQFDEELYK.V	Oxidation (M)	(Ions score 86)
232 - 249	734.6817	2201.0232	2201.0198	2	0	K.EVMQEVAQLSQFDEELYK.V	Oxidation (M)	(Ions score 48)
232 - 249	1101.5189	2201.0232	2201.0198	2	0	K.EVMQEVAQLSQFDEELYK.V	Oxidation (M)	(Ions score 81)
232 - 249	1101.5189	2201.0232	2201.0198	2	0	K.EVMQEVAQLSQFDEELYK.V	Oxidation (M)	(Ions score 67)
232 - 249	734.6817	2201.0232	2201.0198	2	0	K.EVMQEVAQLSQFDEELYK.V	Oxidation (M)	(Ions score 26)
232 - 249	734.6817	2201.0232	2201.0198	2	0	K.EVMQEVAQLSQFDEELYK.V	Oxidation (M)	(Ions score 31)
232 - 249	1101.5189	2201.0233	2201.0198	2	0	K.EVMQEVAQLSQFDEELYK.V	Oxidation (M)	(Ions score 66)
232 - 249	1101.5189	2201.0233	2201.0198	2	0	K.EVMQEVAQLSQFDEELYK.V	Oxidation (M)	(Ions score 53)
232 - 249	1101.5189	2201.0233	2201.0198	2	0	K.EVMQEVAQLSQFDEELYK.V	Oxidation (M)	(Ions score 67)
232 - 249	734.6817	2201.0233	2201.0198	2	0	K.EVMQEVAQLSQFDEELYK.V	Oxidation (M)	(Ions score 41)
232 - 249	1101.5189	2201.0233	2201.0198	2	0	K.EVMQEVAQLSQFDEELYK.V	Oxidation (M)	(Ions score 82)
232 - 249	734.6817	2201.0234	2201.0198	2	0	K.EVMQEVAQLSQFDEELYK.V	Oxidation (M)	(Ions score 27)
232 - 249	1101.5190	2201.0234	2201.0198	2	0	K.EVMQEVAQLSQFDEELYK.V	Oxidation (M)	(Ions score 86)
232 - 249	1101.5190	2201.0234	2201.0198	2	0	K.EVMQEVAQLSQFDEELYK.V	Oxidation (M)	(Ions score 66)
232 - 249	1101.5190	2201.0234	2201.0198	2	0	K.EVMQEVAQLSQFDEELYK.V	Oxidation (M)	(Ions score 67)
232 - 249	734.6818	2201.0234	2201.0198	2	0	K.EVMQEVAQLSQFDEELYK.V	Oxidation (M)	(Ions score 47)
232 - 249	734.6818	2201.0234	2201.0198	2	0	K.EVMQEVAQLSQFDEELYK.V	Oxidation (M)	(Ions score 28)
232 - 249	1101.5190	2201.0235	2201.0198	2	0	K.EVMQEVAQLSQFDEELYK.V	Oxidation (M)	(Ions score 68)
232 - 249	1101.5190	2201.0235	2201.0198	2	0	K.EVMQEVAQLSQFDEELYK.V	Oxidation (M)	(Ions score 67)
232 - 249	1101.5190	2201.0235	2201.0198	2	0	K.EVMQEVAQLSQFDEELYK.V	Oxidation (M)	(Ions score 82)
232 - 249	1101.5190	2201.0235	2201.0198	2	0	K.EVMQEVAQLSQFDEELYK.V	Oxidation (M)	(Ions score 74)
232 - 249	734.6818	2201.0236	2201.0198	2	0	K.EVMQEVAQLSQFDEELYK.V	Oxidation (M)	(Ions score 44)
232 - 249	1101.5191	2201.0236	2201.0198	2	0	K.EVMQEVAQLSQFDEELYK.V	Oxidation (M)	(Ions score 36)
232 - 249	1101.5191	2201.0236	2201.0198	2	0	K.EVMQEVAQLSQFDEELYK.V	Oxidation (M)	(Ions score 52)
232 - 249	1101.5191	2201.0236	2201.0198	2	0	K.EVMQEVAQLSQFDEELYK.V	Oxidation (M)	(Ions score 83)
232 - 249	1101.5191	2201.0236	2201.0198	2	0	K.EVMQEVAQLSQFDEELYK.V	Oxidation (M)	(Ions score 74)
232 - 249	1101.5191	2201.0236	2201.0198	2	0	K.EVMQEVAQLSQFDEELYK.V	Oxidation (M)	(Ions score 53)
232 - 249	734.6818	2201.0236	2201.0198	2	0	K.EVMQEVAQLSQFDEELYK.V	Oxidation (M)	(Ions score 38)
232 - 249	734.6818	2201.0237	2201.0198	2	0	K.EVMQEVAQLSQFDEELYK.V	Oxidation (M)	(Ions score 26)

232 - 249	1101.5191	2201.0237	2201.0198	2	0	K.EVMQEVAQLSQFDEELYK.V	Oxidation (M)	(Ions score 67)
232 - 249	1101.5191	2201.0237	2201.0198	2	0	K.EVMQEVAQLSQFDEELYK.V	Oxidation (M)	(Ions score 70)
232 - 249	1101.5191	2201.0237	2201.0198	2	0	K.EVMQEVAQLSQFDEELYK.V	Oxidation (M)	(Ions score 70)
232 - 249	1101.5191	2201.0237	2201.0198	2	0	K.EVMQEVAQLSQFDEELYK.V	Oxidation (M)	(Ions score 59)
232 - 249	1101.5191	2201.0237	2201.0198	2	0	K.EVMQEVAQLSQFDEELYK.V	Oxidation (M)	(Ions score 69)
232 - 249	734.6818	2201.0237	2201.0198	2	0	K.EVMQEVAQLSQFDEELYK.V	Oxidation (M)	(Ions score 26)
232 - 249	734.6819	2201.0238	2201.0198	2	0	K.EVMQEVAQLSQFDEELYK.V	Oxidation (M)	(Ions score 24)
232 - 249	734.6819	2201.0238	2201.0198	2	0	K.EVMQEVAQLSQFDEELYK.V	Oxidation (M)	(Ions score 43)
232 - 249	734.6819	2201.0239	2201.0198	2	0	K.EVMQEVAQLSQFDEELYK.V	Oxidation (M)	(Ions score 33)
232 - 249	1101.5192	2201.0239	2201.0198	2	0	K.EVMQEVAQLSQFDEELYK.V	Oxidation (M)	(Ions score 54)
232 - 249	1101.5192	2201.0239	2201.0198	2	0	K.EVMQEVAQLSQFDEELYK.V	Oxidation (M)	(Ions score 56)
232 - 249	734.6819	2201.0239	2201.0198	2	0	K.EVMQEVAQLSQFDEELYK.V	Oxidation (M)	(Ions score 38)
232 - 249	1101.5193	2201.0240	2201.0198	2	0	K.EVMQEVAQLSQFDEELYK.V	Oxidation (M)	(Ions score 86)
232 - 249	1101.5193	2201.0240	2201.0198	2	0	K.EVMQEVAQLSQFDEELYK.V	Oxidation (M)	(Ions score 71)
232 - 249	734.6819	2201.0240	2201.0198	2	0	K.EVMQEVAQLSQFDEELYK.V	Oxidation (M)	(Ions score 41)
232 - 249	734.6819	2201.0240	2201.0198	2	0	K.EVMQEVAQLSQFDEELYK.V	Oxidation (M)	(Ions score 29)
232 - 249	734.6819	2201.0240	2201.0198	2	0	K.EVMQEVAQLSQFDEELYK.V	Oxidation (M)	(Ions score 31)
232 - 249	1101.5193	2201.0240	2201.0198	2	0	K.EVMQEVAQLSQFDEELYK.V	Oxidation (M)	(Ions score 70)
232 - 249	1101.5193	2201.0240	2201.0198	2	0	K.EVMQEVAQLSQFDEELYK.V	Oxidation (M)	(Ions score 64)
232 - 249	1101.5193	2201.0240	2201.0198	2	0	K.EVMQEVAQLSQFDEELYK.V	Oxidation (M)	(Ions score 81)
232 - 249	734.6820	2201.0240	2201.0198	2	0	K.EVMQEVAQLSQFDEELYK.V	Oxidation (M)	(Ions score 30)
232 - 249	1101.5193	2201.0240	2201.0198	2	0	K.EVMQEVAQLSQFDEELYK.V	Oxidation (M)	(Ions score 56)
232 - 249	1101.5193	2201.0240	2201.0198	2	0	K.EVMQEVAQLSQFDEELYK.V	Oxidation (M)	(Ions score 54)
232 - 249	1101.5193	2201.0240	2201.0198	2	0	K.EVMQEVAQLSQFDEELYK.V	Oxidation (M)	(Ions score 64)
232 - 249	1101.5193	2201.0240	2201.0198	2	0	K.EVMQEVAQLSQFDEELYK.V	Oxidation (M)	(Ions score 74)
232 - 249	734.6820	2201.0240	2201.0198	2	0	K.EVMQEVAQLSQFDEELYK.V	Oxidation (M)	(Ions score 23)
232 - 249	734.6820	2201.0241	2201.0198	2	0	K.EVMQEVAQLSQFDEELYK.V	Oxidation (M)	(Ions score 28)
232 - 249	1101.5193	2201.0241	2201.0198	2	0	K.EVMQEVAQLSQFDEELYK.V	Oxidation (M)	(Ions score 86)
232 - 249	734.6820	2201.0241	2201.0198	2	0	K.EVMQEVAQLSQFDEELYK.V	Oxidation (M)	(Ions score 41)
232 - 249	1101.5193	2201.0241	2201.0198	2	0	K.EVMQEVAQLSQFDEELYK.V	Oxidation (M)	(Ions score 81)
232 - 249	734.6820	2201.0241	2201.0198	2	0	K.EVMQEVAQLSQFDEELYK.V	Oxidation (M)	(Ions score 42)
232 - 249	734.6820	2201.0241	2201.0198	2	0	K.EVMQEVAQLSQFDEELYK.V	Oxidation (M)	(Ions score 30)
232 - 249	734.6820	2201.0242	2201.0198	2	0	K.EVMQEVAQLSQFDEELYK.V	Oxidation (M)	(Ions score 34)
232 - 249	1101.5194	2201.0242	2201.0198	2	0	K.EVMQEVAQLSQFDEELYK.V	Oxidation (M)	(Ions score 86)
232 - 249	734.6820	2201.0242	2201.0198	2	0	K.EVMQEVAQLSQFDEELYK.V	Oxidation (M)	(Ions score 27)
232 - 249	1101.5194	2201.0242	2201.0198	2	0	K.EVMQEVAQLSQFDEELYK.V	Oxidation (M)	(Ions score 70)
232 - 249	1101.5194	2201.0242	2201.0198	2	0	K.EVMQEVAQLSQFDEELYK.V	Oxidation (M)	(Ions score 67)
232 - 249	1101.5194	2201.0242	2201.0198	2	0	K.EVMQEVAQLSQFDEELYK.V	Oxidation (M)	(Ions score 60)
232 - 249	734.6820	2201.0242	2201.0198	2	0	K.EVMQEVAQLSQFDEELYK.V	Oxidation (M)	(Ions score 45)
232 - 249	1101.5194	2201.0242	2201.0198	2	0	K.EVMQEVAQLSQFDEELYK.V	Oxidation (M)	(Ions score 70)
232 - 249	734.6820	2201.0242	2201.0198	2	0	K.EVMQEVAQLSQFDEELYK.V	Oxidation (M)	(Ions score 39)
232 - 249	734.6820	2201.0242	2201.0198	2	0	K.EVMQEVAQLSQFDEELYK.V	Oxidation (M)	(Ions score 31)
232 - 249	1101.5194	2201.0242	2201.0198	2	0	K.EVMQEVAQLSQFDEELYK.V	Oxidation (M)	(Ions score 53)
232 - 249	734.6820	2201.0243	2201.0198	2	0	K.EVMQEVAQLSQFDEELYK.V	Oxidation (M)	(Ions score 28)
232 - 249	734.6820	2201.0243	2201.0198	2	0	K.EVMQEVAQLSQFDEELYK.V	Oxidation (M)	(Ions score 43)
232 - 249	1101.5194	2201.0243	2201.0198	2	0	K.EVMQEVAQLSQFDEELYK.V	Oxidation (M)	(Ions score 70)
232 - 249	734.6820	2201.0243	2201.0198	2	0	K.EVMQEVAQLSQFDEELYK.V	Oxidation (M)	(Ions score 35)
232 - 249	1101.5194	2201.0243	2201.0198	2	0	K.EVMQEVAQLSQFDEELYK.V	Oxidation (M)	(Ions score 66)
232 - 249	734.6820	2201.0243	2201.0198	2	0	K.EVMQEVAQLSQFDEELYK.V	Oxidation (M)	(Ions score 36)
232 - 249	1101.5194	2201.0243	2201.0198	2	0	K.EVMQEVAQLSQFDEELYK.V	Oxidation (M)	(Ions score 81)
232 - 249	1101.5194	2201.0243	2201.0198	2	0	K.EVMQEVAQLSQFDEELYK.V	Oxidation (M)	(Ions score 86)
232 - 249	734.6821	2201.0245	2201.0198	2	0	K.EVMQEVAQLSQFDEELYK.V	Oxidation (M)	(Ions score 35)
232 - 249	734.6821	2201.0245	2201.0198	2	0	K.EVMQEVAQLSQFDEELYK.V	Oxidation (M)	(Ions score 27)
232 - 249	1101.5195	2201.0245	2201.0198	2	0	K.EVMQEVAQLSQFDEELYK.V	Oxidation (M)	(Ions score 70)
232 - 249	734.6821	2201.0245	2201.0198	2	0	K.EVMQEVAQLSQFDEELYK.V	Oxidation (M)	(Ions score 37)
232 - 249	1101.5195	2201.0245	2201.0198	2	0	K.EVMQEVAQLSQFDEELYK.V	Oxidation (M)	(Ions score 66)
232 - 249	734.6821	2201.0246	2201.0198	2	0	K.EVMQEVAQLSQFDEELYK.V	Oxidation (M)	(Ions score 28)
232 - 249	734.6821	2201.0246	2201.0198	2	0	K.EVMQEVAQLSQFDEELYK.V	Oxidation (M)	(Ions score 27)

232 - 249	734.6821	2201.0246	2201.0198	2	0	K.EVMQEVAQLSQFDEELYK.V	Oxidation (M)	(Ions score 39)
232 - 249	734.6821	2201.0246	2201.0198	2	0	K.EVMQEVAQLSQFDEELYK.V	Oxidation (M)	(Ions score 36)
232 - 249	734.6821	2201.0246	2201.0198	2	0	K.EVMQEVAQLSQFDEELYK.V	Oxidation (M)	(Ions score 43)
232 - 249	1101.5196	2201.0246	2201.0198	2	0	K.EVMQEVAQLSQFDEELYK.V	Oxidation (M)	(Ions score 64)
232 - 249	1101.5196	2201.0246	2201.0198	2	0	K.EVMQEVAQLSQFDEELYK.V	Oxidation (M)	(Ions score 63)
232 - 249	1101.5196	2201.0246	2201.0198	2	0	K.EVMQEVAQLSQFDEELYK.V	Oxidation (M)	(Ions score 49)
232 - 249	734.6821	2201.0246	2201.0198	2	0	K.EVMQEVAQLSQFDEELYK.V	Oxidation (M)	(Ions score 33)
232 - 249	734.6822	2201.0246	2201.0198	2	0	K.EVMQEVAQLSQFDEELYK.V	Oxidation (M)	(Ions score 45)
232 - 249	1101.5196	2201.0246	2201.0198	2	0	K.EVMQEVAQLSQFDEELYK.V	Oxidation (M)	(Ions score 74)
232 - 249	734.6822	2201.0247	2201.0198	2	0	K.EVMQEVAQLSQFDEELYK.V	Oxidation (M)	(Ions score 53)
232 - 249	734.6822	2201.0247	2201.0198	2	0	K.EVMQEVAQLSQFDEELYK.V	Oxidation (M)	(Ions score 36)
232 - 249	1101.5196	2201.0247	2201.0198	2	0	K.EVMQEVAQLSQFDEELYK.V	Oxidation (M)	(Ions score 60)
232 - 249	1101.5196	2201.0247	2201.0198	2	0	K.EVMQEVAQLSQFDEELYK.V	Oxidation (M)	(Ions score 81)
232 - 249	734.6822	2201.0247	2201.0198	2	0	K.EVMQEVAQLSQFDEELYK.V	Oxidation (M)	(Ions score 35)
232 - 249	734.6822	2201.0248	2201.0198	2	0	K.EVMQEVAQLSQFDEELYK.V	Oxidation (M)	(Ions score 24)
232 - 249	734.6822	2201.0248	2201.0198	2	0	K.EVMQEVAQLSQFDEELYK.V	Oxidation (M)	(Ions score 22)
232 - 249	1101.5197	2201.0248	2201.0198	2	0	K.EVMQEVAQLSQFDEELYK.V	Oxidation (M)	(Ions score 67)
232 - 249	734.6822	2201.0248	2201.0198	2	0	K.EVMQEVAQLSQFDEELYK.V	Oxidation (M)	(Ions score 30)
232 - 249	734.6822	2201.0249	2201.0198	2	0	K.EVMQEVAQLSQFDEELYK.V	Oxidation (M)	(Ions score 32)
232 - 249	734.6822	2201.0249	2201.0198	2	0	K.EVMQEVAQLSQFDEELYK.V	Oxidation (M)	(Ions score 39)
232 - 249	1101.5197	2201.0249	2201.0198	2	0	K.EVMQEVAQLSQFDEELYK.V	Oxidation (M)	(Ions score 64)
232 - 249	1101.5197	2201.0249	2201.0198	2	0	K.EVMQEVAQLSQFDEELYK.V	Oxidation (M)	(Ions score 67)
232 - 249	1101.5197	2201.0249	2201.0198	2	0	K.EVMQEVAQLSQFDEELYK.V	Oxidation (M)	(Ions score 67)
232 - 249	734.6822	2201.0249	2201.0198	2	0	K.EVMQEVAQLSQFDEELYK.V	Oxidation (M)	(Ions score 29)
232 - 249	734.6822	2201.0249	2201.0198	2	0	K.EVMQEVAQLSQFDEELYK.V	Oxidation (M)	(Ions score 29)
232 - 249	734.6823	2201.0249	2201.0198	2	0	K.EVMQEVAQLSQFDEELYK.V	Oxidation (M)	(Ions score 40)
232 - 249	734.6823	2201.0250	2201.0198	2	0	K.EVMQEVAQLSQFDEELYK.V	Oxidation (M)	(Ions score 36)
232 - 249	1101.5198	2201.0250	2201.0198	2	0	K.EVMQEVAQLSQFDEELYK.V	Oxidation (M)	(Ions score 66)
232 - 249	1101.5198	2201.0250	2201.0198	2	0	K.EVMQEVAQLSQFDEELYK.V	Oxidation (M)	(Ions score 74)
232 - 249	1101.5198	2201.0250	2201.0198	2	0	K.EVMQEVAQLSQFDEELYK.V	Oxidation (M)	(Ions score 49)
232 - 249	734.6823	2201.0250	2201.0198	2	0	K.EVMQEVAQLSQFDEELYK.V	Oxidation (M)	(Ions score 29)
232 - 249	1101.5198	2201.0251	2201.0198	2	0	K.EVMQEVAQLSQFDEELYK.V	Oxidation (M)	(Ions score 70)
232 - 249	1101.5198	2201.0251	2201.0198	2	0	K.EVMQEVAQLSQFDEELYK.V	Oxidation (M)	(Ions score 66)
232 - 249	734.6823	2201.0251	2201.0198	2	0	K.EVMQEVAQLSQFDEELYK.V	Oxidation (M)	(Ions score 26)
232 - 249	1101.5198	2201.0251	2201.0198	2	0	K.EVMQEVAQLSQFDEELYK.V	Oxidation (M)	(Ions score 81)
232 - 249	734.6823	2201.0251	2201.0198	2	0	K.EVMQEVAQLSQFDEELYK.V	Oxidation (M)	(Ions score 22)
232 - 249	1101.5198	2201.0251	2201.0198	2	0	K.EVMQEVAQLSQFDEELYK.V	Oxidation (M)	(Ions score 70)
232 - 249	734.6823	2201.0252	2201.0198	2	0	K.EVMQEVAQLSQFDEELYK.V	Oxidation (M)	(Ions score 29)
232 - 249	1101.5199	2201.0252	2201.0198	2	0	K.EVMQEVAQLSQFDEELYK.V	Oxidation (M)	(Ions score 67)
232 - 249	734.6823	2201.0252	2201.0198	2	0	K.EVMQEVAQLSQFDEELYK.V	Oxidation (M)	(Ions score 48)
232 - 249	1101.5199	2201.0252	2201.0198	2	0	K.EVMQEVAQLSQFDEELYK.V	Oxidation (M)	(Ions score 81)
232 - 249	1101.5199	2201.0252	2201.0198	2	0	K.EVMQEVAQLSQFDEELYK.V	Oxidation (M)	(Ions score 74)
232 - 249	1101.5199	2201.0253	2201.0198	2	0	K.EVMQEVAQLSQFDEELYK.V	Oxidation (M)	(Ions score 64)
232 - 249	734.6824	2201.0253	2201.0198	2	0	K.EVMQEVAQLSQFDEELYK.V	Oxidation (M)	(Ions score 35)
232 - 249	1101.5199	2201.0253	2201.0198	3	0	K.EVMQEVAQLSQFDEELYK.V	Oxidation (M)	(Ions score 64)
232 - 249	734.6824	2201.0253	2201.0198	3	0	K.EVMQEVAQLSQFDEELYK.V	Oxidation (M)	(Ions score 40)
232 - 249	734.6824	2201.0254	2201.0198	3	0	K.EVMQEVAQLSQFDEELYK.V	Oxidation (M)	(Ions score 41)
232 - 249	734.6824	2201.0254	2201.0198	3	0	K.EVMQEVAQLSQFDEELYK.V	Oxidation (M)	(Ions score 25)
232 - 249	734.6824	2201.0255	2201.0198	3	0	K.EVMQEVAQLSQFDEELYK.V	Oxidation (M)	(Ions score 36)
232 - 249	734.6824	2201.0255	2201.0198	3	0	K.EVMQEVAQLSQFDEELYK.V	Oxidation (M)	(Ions score 35)
232 - 249	734.6825	2201.0255	2201.0198	3	0	K.EVMQEVAQLSQFDEELYK.V	Oxidation (M)	(Ions score 29)
232 - 249	734.6825	2201.0256	2201.0198	3	0	K.EVMQEVAQLSQFDEELYK.V	Oxidation (M)	(Ions score 30)
232 - 249	1101.5201	2201.0256	2201.0198	3	0	K.EVMQEVAQLSQFDEELYK.V	Oxidation (M)	(Ions score 57)
232 - 249	734.6825	2201.0256	2201.0198	3	0	K.EVMQEVAQLSQFDEELYK.V	Oxidation (M)	(Ions score 28)
232 - 249	734.6825	2201.0257	2201.0198	3	0	K.EVMQEVAQLSQFDEELYK.V	Oxidation (M)	(Ions score 22)
232 - 249	1101.5202	2201.0258	2201.0198	3	0	K.EVMQEVAQLSQFDEELYK.V	Oxidation (M)	(Ions score 63)
232 - 249	734.6826	2201.0259	2201.0198	3	0	K.EVMQEVAQLSQFDEELYK.V	Oxidation (M)	(Ions score 45)
232 - 249	734.6826	2201.0259	2201.0198	3	0	K.EVMQEVAQLSQFDEELYK.V	Oxidation (M)	(Ions score 41)

232 - 249	734.6826	2201.0259	2201.0198	3	0	K.EVMQEVAQLSQFDEELYK.V	Oxidation (M)	(Ions score 33)
232 - 249	734.6826	2201.0259	2201.0198	3	0	K.EVMQEVAQLSQFDEELYK.V	Oxidation (M)	(Ions score 37)
232 - 249	734.6826	2201.0259	2201.0198	3	0	K.EVMQEVAQLSQFDEELYK.V	Oxidation (M)	(Ions score 25)
232 - 249	1101.5202	2201.0259	2201.0198	3	0	K.EVMQEVAQLSQFDEELYK.V	Oxidation (M)	(Ions score 86)
232 - 249	734.6826	2201.0259	2201.0198	3	0	K.EVMQEVAQLSQFDEELYK.V	Oxidation (M)	(Ions score 43)
232 - 249	1101.5202	2201.0259	2201.0198	3	0	K.EVMQEVAQLSQFDEELYK.V	Oxidation (M)	(Ions score 81)
232 - 249	734.6826	2201.0260	2201.0198	3	0	K.EVMQEVAQLSQFDEELYK.V	Oxidation (M)	(Ions score 36)
232 - 249	734.6826	2201.0260	2201.0198	3	0	K.EVMQEVAQLSQFDEELYK.V	Oxidation (M)	(Ions score 24)
232 - 249	734.6826	2201.0260	2201.0198	3	0	K.EVMQEVAQLSQFDEELYK.V	Oxidation (M)	(Ions score 28)
232 - 249	734.6826	2201.0260	2201.0198	3	0	K.EVMQEVAQLSQFDEELYK.V	Oxidation (M)	(Ions score 38)
232 - 249	1101.5203	2201.0260	2201.0198	3	0	K.EVMQEVAQLSQFDEELYK.V	Oxidation (M)	(Ions score 74)
232 - 249	734.6826	2201.0261	2201.0198	3	0	K.EVMQEVAQLSQFDEELYK.V	Oxidation (M)	(Ions score 30)
232 - 249	734.6826	2201.0261	2201.0198	3	0	K.EVMQEVAQLSQFDEELYK.V	Oxidation (M)	(Ions score 33)
232 - 249	734.6826	2201.0261	2201.0198	3	0	K.EVMQEVAQLSQFDEELYK.V	Oxidation (M)	(Ions score 33)
232 - 249	734.6826	2201.0261	2201.0198	3	0	K.EVMQEVAQLSQFDEELYK.V	Oxidation (M)	(Ions score 39)
232 - 249	734.6827	2201.0261	2201.0198	3	0	K.EVMQEVAQLSQFDEELYK.V	Oxidation (M)	(Ions score 42)
232 - 249	1101.5203	2201.0261	2201.0198	3	0	K.EVMQEVAQLSQFDEELYK.V	Oxidation (M)	(Ions score 67)
232 - 249	734.6827	2201.0261	2201.0198	3	0	K.EVMQEVAQLSQFDEELYK.V	Oxidation (M)	(Ions score 39)
232 - 249	1101.5204	2201.0262	2201.0198	3	0	K.EVMQEVAQLSQFDEELYK.V	Oxidation (M)	(Ions score 74)
232 - 249	1101.5204	2201.0262	2201.0198	3	0	K.EVMQEVAQLSQFDEELYK.V	Oxidation (M)	(Ions score 66)
232 - 249	734.6827	2201.0262	2201.0198	3	0	K.EVMQEVAQLSQFDEELYK.V	Oxidation (M)	(Ions score 37)
232 - 249	734.6827	2201.0262	2201.0198	3	0	K.EVMQEVAQLSQFDEELYK.V	Oxidation (M)	(Ions score 27)
232 - 249	734.6827	2201.0262	2201.0198	3	0	K.EVMQEVAQLSQFDEELYK.V	Oxidation (M)	(Ions score 27)
232 - 249	1101.5204	2201.0262	2201.0198	3	0	K.EVMQEVAQLSQFDEELYK.V	Oxidation (M)	(Ions score 81)
232 - 249	734.6827	2201.0262	2201.0198	3	0	K.EVMQEVAQLSQFDEELYK.V	Oxidation (M)	(Ions score 32)
232 - 249	1101.5204	2201.0262	2201.0198	3	0	K.EVMQEVAQLSQFDEELYK.V	Oxidation (M)	(Ions score 68)
232 - 249	734.6827	2201.0263	2201.0198	3	0	K.EVMQEVAQLSQFDEELYK.V	Oxidation (M)	(Ions score 50)
232 - 249	734.6827	2201.0263	2201.0198	3	0	K.EVMQEVAQLSQFDEELYK.V	Oxidation (M)	(Ions score 38)
232 - 249	734.6827	2201.0263	2201.0198	3	0	K.EVMQEVAQLSQFDEELYK.V	Oxidation (M)	(Ions score 39)
232 - 249	734.6827	2201.0263	2201.0198	3	0	K.EVMQEVAQLSQFDEELYK.V	Oxidation (M)	(Ions score 26)
232 - 249	734.6827	2201.0263	2201.0198	3	0	K.EVMQEVAQLSQFDEELYK.V	Oxidation (M)	(Ions score 36)
232 - 249	734.6827	2201.0263	2201.0198	3	0	K.EVMQEVAQLSQFDEELYK.V	Oxidation (M)	(Ions score 44)
232 - 249	734.6827	2201.0264	2201.0198	3	0	K.EVMQEVAQLSQFDEELYK.V	Oxidation (M)	(Ions score 29)
232 - 249	1101.5205	2201.0264	2201.0198	3	0	K.EVMQEVAQLSQFDEELYK.V	Oxidation (M)	(Ions score 58)
232 - 249	1101.5205	2201.0264	2201.0198	3	0	K.EVMQEVAQLSQFDEELYK.V	Oxidation (M)	(Ions score 67)
232 - 249	1101.5205	2201.0264	2201.0198	3	0	K.EVMQEVAQLSQFDEELYK.V	Oxidation (M)	(Ions score 86)
232 - 249	1101.5205	2201.0264	2201.0198	3	0	K.EVMQEVAQLSQFDEELYK.V	Oxidation (M)	(Ions score 70)
232 - 249	1101.5205	2201.0264	2201.0198	3	0	K.EVMQEVAQLSQFDEELYK.V	Oxidation (M)	(Ions score 78)
232 - 249	734.6828	2201.0264	2201.0198	3	0	K.EVMQEVAQLSQFDEELYK.V	Oxidation (M)	(Ions score 38)
232 - 249	734.6828	2201.0264	2201.0198	3	0	K.EVMQEVAQLSQFDEELYK.V	Oxidation (M)	(Ions score 28)
232 - 249	734.6828	2201.0264	2201.0198	3	0	K.EVMQEVAQLSQFDEELYK.V	Oxidation (M)	(Ions score 41)
232 - 249	734.6828	2201.0265	2201.0198	3	0	K.EVMQEVAQLSQFDEELYK.V	Oxidation (M)	(Ions score 31)
232 - 249	734.6828	2201.0265	2201.0198	3	0	K.EVMQEVAQLSQFDEELYK.V	Oxidation (M)	(Ions score 38)
232 - 249	734.6828	2201.0265	2201.0198	3	0	K.EVMQEVAQLSQFDEELYK.V	Oxidation (M)	(Ions score 26)
232 - 249	734.6828	2201.0266	2201.0198	3	0	K.EVMQEVAQLSQFDEELYK.V	Oxidation (M)	(Ions score 31)
232 - 249	734.6828	2201.0266	2201.0198	3	0	K.EVMQEVAQLSQFDEELYK.V	Oxidation (M)	(Ions score 36)
232 - 249	1101.5206	2201.0267	2201.0198	3	0	K.EVMQEVAQLSQFDEELYK.V	Oxidation (M)	(Ions score 74)
232 - 249	734.6828	2201.0267	2201.0198	3	0	K.EVMQEVAQLSQFDEELYK.V	Oxidation (M)	(Ions score 33)
232 - 249	1101.5206	2201.0267	2201.0198	3	0	K.EVMQEVAQLSQFDEELYK.V	Oxidation (M)	(Ions score 78)
232 - 249	1101.5207	2201.0268	2201.0198	3	0	K.EVMQEVAQLSQFDEELYK.V	Oxidation (M)	(Ions score 86)
232 - 249	734.6829	2201.0268	2201.0198	3	0	K.EVMQEVAQLSQFDEELYK.V	Oxidation (M)	(Ions score 39)
232 - 249	734.6829	2201.0268	2201.0198	3	0	K.EVMQEVAQLSQFDEELYK.V	Oxidation (M)	(Ions score 36)
232 - 249	1101.5207	2201.0269	2201.0198	3	0	K.EVMQEVAQLSQFDEELYK.V	Oxidation (M)	(Ions score 67)
232 - 249	734.6829	2201.0269	2201.0198	3	0	K.EVMQEVAQLSQFDEELYK.V	Oxidation (M)	(Ions score 42)
232 - 249	734.6829	2201.0269	2201.0198	3	0	K.EVMQEVAQLSQFDEELYK.V	Oxidation (M)	(Ions score 38)
232 - 249	734.6829	2201.0269	2201.0198	3	0	K.EVMQEVAQLSQFDEELYK.V	Oxidation (M)	(Ions score 25)
232 - 249	734.6829	2201.0269	2201.0198	3	0	K.EVMQEVAQLSQFDEELYK.V	Oxidation (M)	(Ions score 41)
232 - 249	734.6829	2201.0269	2201.0198	3	0	K.EVMQEVAQLSQFDEELYK.V	Oxidation (M)	(Ions score 50)

232 - 249	734.6829	2201.0270	2201.0198	3	0	K.EVMQEVAQLSQFDEELYK.V	Oxidation (M)	(Ions score 26)
232 - 249	734.6829	2201.0270	2201.0198	3	0	K.EVMQEVAQLSQFDEELYK.V	Oxidation (M)	(Ions score 38)
232 - 249	1101.5208	2201.0270	2201.0198	3	0	K.EVMQEVAQLSQFDEELYK.V	Oxidation (M)	(Ions score 86)
232 - 249	734.6829	2201.0270	2201.0198	3	0	K.EVMQEVAQLSQFDEELYK.V	Oxidation (M)	(Ions score 34)
232 - 249	734.6829	2201.0270	2201.0198	3	0	K.EVMQEVAQLSQFDEELYK.V	Oxidation (M)	(Ions score 46)
232 - 249	734.6829	2201.0270	2201.0198	3	0	K.EVMQEVAQLSQFDEELYK.V	Oxidation (M)	(Ions score 33)
232 - 249	734.6830	2201.0271	2201.0198	3	0	K.EVMQEVAQLSQFDEELYK.V	Oxidation (M)	(Ions score 51)
232 - 249	1101.5208	2201.0271	2201.0198	3	0	K.EVMQEVAQLSQFDEELYK.V	Oxidation (M)	(Ions score 70)
232 - 249	1101.5208	2201.0271	2201.0198	3	0	K.EVMQEVAQLSQFDEELYK.V	Oxidation (M)	(Ions score 86)
232 - 249	734.6830	2201.0271	2201.0198	3	0	K.EVMQEVAQLSQFDEELYK.V	Oxidation (M)	(Ions score 34)
232 - 249	1101.5208	2201.0271	2201.0198	3	0	K.EVMQEVAQLSQFDEELYK.V	Oxidation (M)	(Ions score 56)
232 - 249	734.6830	2201.0272	2201.0198	3	0	K.EVMQEVAQLSQFDEELYK.V	Oxidation (M)	(Ions score 32)
232 - 249	1101.5209	2201.0272	2201.0198	3	0	K.EVMQEVAQLSQFDEELYK.V	Oxidation (M)	(Ions score 86)
232 - 249	1101.5209	2201.0272	2201.0198	3	0	K.EVMQEVAQLSQFDEELYK.V	Oxidation (M)	(Ions score 63)
232 - 249	734.6830	2201.0273	2201.0198	3	0	K.EVMQEVAQLSQFDEELYK.V	Oxidation (M)	(Ions score 35)
232 - 249	734.6830	2201.0273	2201.0198	3	0	K.EVMQEVAQLSQFDEELYK.V	Oxidation (M)	(Ions score 46)
232 - 249	734.6830	2201.0273	2201.0198	3	0	K.EVMQEVAQLSQFDEELYK.V	Oxidation (M)	(Ions score 46)
232 - 249	1101.5210	2201.0274	2201.0198	3	0	K.EVMQEVAQLSQFDEELYK.V	Oxidation (M)	(Ions score 66)
232 - 249	734.6831	2201.0274	2201.0198	3	0	K.EVMQEVAQLSQFDEELYK.V	Oxidation (M)	(Ions score 36)
232 - 249	734.6831	2201.0274	2201.0198	3	0	K.EVMQEVAQLSQFDEELYK.V	Oxidation (M)	(Ions score 30)
232 - 249	1101.5210	2201.0274	2201.0198	3	0	K.EVMQEVAQLSQFDEELYK.V	Oxidation (M)	(Ions score 56)
232 - 249	1101.5210	2201.0275	2201.0198	3	0	K.EVMQEVAQLSQFDEELYK.V	Oxidation (M)	(Ions score 71)
232 - 249	734.6831	2201.0275	2201.0198	3	0	K.EVMQEVAQLSQFDEELYK.V	Oxidation (M)	(Ions score 27)
232 - 249	734.6831	2201.0275	2201.0198	3	0	K.EVMQEVAQLSQFDEELYK.V	Oxidation (M)	(Ions score 40)
232 - 249	734.6831	2201.0275	2201.0198	4	0	K.EVMQEVAQLSQFDEELYK.V	Oxidation (M)	(Ions score 31)
232 - 249	734.6831	2201.0275	2201.0198	4	0	K.EVMQEVAQLSQFDEELYK.V	Oxidation (M)	(Ions score 46)
232 - 249	734.6831	2201.0275	2201.0198	4	0	K.EVMQEVAQLSQFDEELYK.V	Oxidation (M)	(Ions score 39)
232 - 249	734.6831	2201.0275	2201.0198	4	0	K.EVMQEVAQLSQFDEELYK.V	Oxidation (M)	(Ions score 27)
232 - 249	734.6831	2201.0276	2201.0198	4	0	K.EVMQEVAQLSQFDEELYK.V	Oxidation (M)	(Ions score 36)
232 - 249	1101.5211	2201.0276	2201.0198	4	0	K.EVMQEVAQLSQFDEELYK.V	Oxidation (M)	(Ions score 56)
232 - 249	734.6832	2201.0276	2201.0198	4	0	K.EVMQEVAQLSQFDEELYK.V	Oxidation (M)	(Ions score 44)
232 - 249	734.6832	2201.0277	2201.0198	4	0	K.EVMQEVAQLSQFDEELYK.V	Oxidation (M)	(Ions score 51)
232 - 249	734.6832	2201.0277	2201.0198	4	0	K.EVMQEVAQLSQFDEELYK.V	Oxidation (M)	(Ions score 43)
232 - 249	734.6832	2201.0277	2201.0198	4	0	K.EVMQEVAQLSQFDEELYK.V	Oxidation (M)	(Ions score 40)
232 - 249	1101.5211	2201.0277	2201.0198	4	0	K.EVMQEVAQLSQFDEELYK.V	Oxidation (M)	(Ions score 81)
232 - 249	734.6832	2201.0277	2201.0198	4	0	K.EVMQEVAQLSQFDEELYK.V	Oxidation (M)	(Ions score 29)
232 - 249	734.6832	2201.0277	2201.0198	4	0	K.EVMQEVAQLSQFDEELYK.V	Oxidation (M)	(Ions score 22)
232 - 249	734.6832	2201.0277	2201.0198	4	0	K.EVMQEVAQLSQFDEELYK.V	Oxidation (M)	(Ions score 37)
232 - 249	1101.5212	2201.0278	2201.0198	4	0	K.EVMQEVAQLSQFDEELYK.V	Oxidation (M)	(Ions score 74)
232 - 249	1101.5212	2201.0279	2201.0198	4	0	K.EVMQEVAQLSQFDEELYK.V	Oxidation (M)	(Ions score 86)
232 - 249	1101.5212	2201.0279	2201.0198	4	0	K.EVMQEVAQLSQFDEELYK.V	Oxidation (M)	(Ions score 66)
232 - 249	1101.5212	2201.0279	2201.0198	4	0	K.EVMQEVAQLSQFDEELYK.V	Oxidation (M)	(Ions score 66)
232 - 249	734.6832	2201.0279	2201.0198	4	0	K.EVMQEVAQLSQFDEELYK.V	Oxidation (M)	(Ions score 26)
232 - 249	1101.5212	2201.0279	2201.0198	4	0	K.EVMQEVAQLSQFDEELYK.V	Oxidation (M)	(Ions score 67)
232 - 249	1101.5213	2201.0280	2201.0198	4	0	K.EVMQEVAQLSQFDEELYK.V	Oxidation (M)	(Ions score 70)
232 - 249	734.6833	2201.0280	2201.0198	4	0	K.EVMQEVAQLSQFDEELYK.V	Oxidation (M)	(Ions score 38)
232 - 249	1101.5213	2201.0280	2201.0198	4	0	K.EVMQEVAQLSQFDEELYK.V	Oxidation (M)	(Ions score 66)
232 - 249	734.6833	2201.0280	2201.0198	4	0	K.EVMQEVAQLSQFDEELYK.V	Oxidation (M)	(Ions score 45)
232 - 249	1101.5213	2201.0280	2201.0198	4	0	K.EVMQEVAQLSQFDEELYK.V	Oxidation (M)	(Ions score 70)
232 - 249	734.6833	2201.0280	2201.0198	4	0	K.EVMQEVAQLSQFDEELYK.V	Oxidation (M)	(Ions score 46)
232 - 249	1101.5213	2201.0280	2201.0198	4	0	K.EVMQEVAQLSQFDEELYK.V	Oxidation (M)	(Ions score 70)
232 - 249	734.6833	2201.0280	2201.0198	4	0	K.EVMQEVAQLSQFDEELYK.V	Oxidation (M)	(Ions score 35)
232 - 249	734.6833	2201.0281	2201.0198	4	0	K.EVMQEVAQLSQFDEELYK.V	Oxidation (M)	(Ions score 45)
232 - 249	1101.5213	2201.0281	2201.0198	4	0	K.EVMQEVAQLSQFDEELYK.V	Oxidation (M)	(Ions score 81)
232 - 249	734.6833	2201.0282	2201.0198	4	0	K.EVMQEVAQLSQFDEELYK.V	Oxidation (M)	(Ions score 35)
232 - 249	734.6833	2201.0282	2201.0198	4	0	K.EVMQEVAQLSQFDEELYK.V	Oxidation (M)	(Ions score 50)
232 - 249	1101.5214	2201.0282	2201.0198	4	0	K.EVMQEVAQLSQFDEELYK.V	Oxidation (M)	(Ions score 86)
232 - 249	1101.5214	2201.0282	2201.0198	4	0	K.EVMQEVAQLSQFDEELYK.V	Oxidation (M)	(Ions score 52)

232 - 249	734.6833	2201.0282	2201.0198	4	0	K.EVMQEVAQLSQFDEELYK.V	Oxidation (M)	(Ions score 44)
232 - 249	1101.5214	2201.0282	2201.0198	4	0	K.EVMQEVAQLSQFDEELYK.V	Oxidation (M)	(Ions score 56)
232 - 249	1101.5214	2201.0282	2201.0198	4	0	K.EVMQEVAQLSQFDEELYK.V	Oxidation (M)	(Ions score 81)
232 - 249	1101.5214	2201.0283	2201.0198	4	0	K.EVMQEVAQLSQFDEELYK.V	Oxidation (M)	(Ions score 70)
232 - 249	1101.5214	2201.0283	2201.0198	4	0	K.EVMQEVAQLSQFDEELYK.V	Oxidation (M)	(Ions score 70)
232 - 249	1101.5214	2201.0283	2201.0198	4	0	K.EVMQEVAQLSQFDEELYK.V	Oxidation (M)	(Ions score 66)
232 - 249	1101.5215	2201.0284	2201.0198	4	0	K.EVMQEVAQLSQFDEELYK.V	Oxidation (M)	(Ions score 52)
232 - 249	734.6834	2201.0284	2201.0198	4	0	K.EVMQEVAQLSQFDEELYK.V	Oxidation (M)	(Ions score 35)
232 - 249	734.6834	2201.0284	2201.0198	4	0	K.EVMQEVAQLSQFDEELYK.V	Oxidation (M)	(Ions score 52)
232 - 249	734.6834	2201.0284	2201.0198	4	0	K.EVMQEVAQLSQFDEELYK.V	Oxidation (M)	(Ions score 37)
232 - 249	734.6834	2201.0284	2201.0198	4	0	K.EVMQEVAQLSQFDEELYK.V	Oxidation (M)	(Ions score 36)
232 - 249	734.6834	2201.0284	2201.0198	4	0	K.EVMQEVAQLSQFDEELYK.V	Oxidation (M)	(Ions score 25)
232 - 249	1101.5215	2201.0284	2201.0198	4	0	K.EVMQEVAQLSQFDEELYK.V	Oxidation (M)	(Ions score 74)
232 - 249	1101.5215	2201.0285	2201.0198	4	0	K.EVMQEVAQLSQFDEELYK.V	Oxidation (M)	(Ions score 78)
232 - 249	734.6835	2201.0286	2201.0198	4	0	K.EVMQEVAQLSQFDEELYK.V	Oxidation (M)	(Ions score 43)
232 - 249	734.6835	2201.0286	2201.0198	4	0	K.EVMQEVAQLSQFDEELYK.V	Oxidation (M)	(Ions score 35)
232 - 249	1101.5216	2201.0286	2201.0198	4	0	K.EVMQEVAQLSQFDEELYK.V	Oxidation (M)	(Ions score 64)
232 - 249	734.6835	2201.0286	2201.0198	4	0	K.EVMQEVAQLSQFDEELYK.V	Oxidation (M)	(Ions score 34)
232 - 249	734.6835	2201.0286	2201.0198	4	0	K.EVMQEVAQLSQFDEELYK.V	Oxidation (M)	(Ions score 39)
232 - 249	734.6835	2201.0287	2201.0198	4	0	K.EVMQEVAQLSQFDEELYK.V	Oxidation (M)	(Ions score 39)
232 - 249	734.6835	2201.0287	2201.0198	4	0	K.EVMQEVAQLSQFDEELYK.V	Oxidation (M)	(Ions score 38)
232 - 249	734.6835	2201.0287	2201.0198	4	0	K.EVMQEVAQLSQFDEELYK.V	Oxidation (M)	(Ions score 41)
232 - 249	734.6836	2201.0288	2201.0198	4	0	K.EVMQEVAQLSQFDEELYK.V	Oxidation (M)	(Ions score 31)
232 - 249	734.6836	2201.0288	2201.0198	4	0	K.EVMQEVAQLSQFDEELYK.V	Oxidation (M)	(Ions score 24)
232 - 249	734.6836	2201.0289	2201.0198	4	0	K.EVMQEVAQLSQFDEELYK.V	Oxidation (M)	(Ions score 38)
232 - 249	734.6836	2201.0289	2201.0198	4	0	K.EVMQEVAQLSQFDEELYK.V	Oxidation (M)	(Ions score 41)
232 - 249	734.6836	2201.0289	2201.0198	4	0	K.EVMQEVAQLSQFDEELYK.V	Oxidation (M)	(Ions score 35)
232 - 249	734.6836	2201.0290	2201.0198	4	0	K.EVMQEVAQLSQFDEELYK.V	Oxidation (M)	(Ions score 47)
232 - 249	734.6836	2201.0290	2201.0198	4	0	K.EVMQEVAQLSQFDEELYK.V	Oxidation (M)	(Ions score 29)
232 - 249	734.6836	2201.0291	2201.0198	4	0	K.EVMQEVAQLSQFDEELYK.V	Oxidation (M)	(Ions score 37)
232 - 249	1101.5218	2201.0291	2201.0198	4	0	K.EVMQEVAQLSQFDEELYK.V	Oxidation (M)	(Ions score 56)
232 - 249	734.6837	2201.0291	2201.0198	4	0	K.EVMQEVAQLSQFDEELYK.V	Oxidation (M)	(Ions score 37)
232 - 249	1101.5219	2201.0292	2201.0198	4	0	K.EVMQEVAQLSQFDEELYK.V	Oxidation (M)	(Ions score 53)
232 - 249	734.6837	2201.0292	2201.0198	4	0	K.EVMQEVAQLSQFDEELYK.V	Oxidation (M)	(Ions score 39)
232 - 249	734.6837	2201.0292	2201.0198	4	0	K.EVMQEVAQLSQFDEELYK.V	Oxidation (M)	(Ions score 34)
232 - 249	1101.5219	2201.0292	2201.0198	4	0	K.EVMQEVAQLSQFDEELYK.V	Oxidation (M)	(Ions score 67)
232 - 249	1101.5220	2201.0294	2201.0198	4	0	K.EVMQEVAQLSQFDEELYK.V	Oxidation (M)	(Ions score 70)
232 - 249	1101.5220	2201.0294	2201.0198	4	0	K.EVMQEVAQLSQFDEELYK.V	Oxidation (M)	(Ions score 81)
232 - 249	734.6838	2201.0295	2201.0198	4	0	K.EVMQEVAQLSQFDEELYK.V	Oxidation (M)	(Ions score 54)
232 - 249	1101.5220	2201.0295	2201.0198	4	0	K.EVMQEVAQLSQFDEELYK.V	Oxidation (M)	(Ions score 81)
232 - 249	1101.5221	2201.0296	2201.0198	4	0	K.EVMQEVAQLSQFDEELYK.V	Oxidation (M)	(Ions score 74)
232 - 249	734.6838	2201.0297	2201.0198	4	0	K.EVMQEVAQLSQFDEELYK.V	Oxidation (M)	(Ions score 53)
232 - 249	734.6838	2201.0297	2201.0198	5	0	K.EVMQEVAQLSQFDEELYK.V	Oxidation (M)	(Ions score 43)
232 - 249	1101.5221	2201.0297	2201.0198	5	0	K.EVMQEVAQLSQFDEELYK.V	Oxidation (M)	(Ions score 78)
232 - 249	734.6839	2201.0297	2201.0198	5	0	K.EVMQEVAQLSQFDEELYK.V	Oxidation (M)	(Ions score 24)
232 - 249	734.6839	2201.0297	2201.0198	5	0	K.EVMQEVAQLSQFDEELYK.V	Oxidation (M)	(Ions score 38)
232 - 249	734.6839	2201.0298	2201.0198	5	0	K.EVMQEVAQLSQFDEELYK.V	Oxidation (M)	(Ions score 50)
232 - 249	734.6839	2201.0298	2201.0198	5	0	K.EVMQEVAQLSQFDEELYK.V	Oxidation (M)	(Ions score 45)
232 - 249	1101.5222	2201.0299	2201.0198	5	0	K.EVMQEVAQLSQFDEELYK.V	Oxidation (M)	(Ions score 63)
232 - 249	1101.5222	2201.0299	2201.0198	5	0	K.EVMQEVAQLSQFDEELYK.V	Oxidation (M)	(Ions score 82)
232 - 249	734.6839	2201.0300	2201.0198	5	0	K.EVMQEVAQLSQFDEELYK.V	Oxidation (M)	(Ions score 29)
232 - 249	734.6840	2201.0301	2201.0198	5	0	K.EVMQEVAQLSQFDEELYK.V	Oxidation (M)	(Ions score 44)
232 - 249	1101.5223	2201.0301	2201.0198	5	0	K.EVMQEVAQLSQFDEELYK.V	Oxidation (M)	(Ions score 53)
232 - 249	734.6840	2201.0302	2201.0198	5	0	K.EVMQEVAQLSQFDEELYK.V	Oxidation (M)	(Ions score 45)
232 - 249	734.6840	2201.0302	2201.0198	5	0	K.EVMQEVAQLSQFDEELYK.V	Oxidation (M)	(Ions score 50)
232 - 249	1101.5224	2201.0302	2201.0198	5	0	K.EVMQEVAQLSQFDEELYK.V	Oxidation (M)	(Ions score 74)
232 - 249	734.6840	2201.0302	2201.0198	5	0	K.EVMQEVAQLSQFDEELYK.V	Oxidation (M)	(Ions score 37)
232 - 249	734.6841	2201.0305	2201.0198	5	0	K.EVMQEVAQLSQFDEELYK.V	Oxidation (M)	(Ions score 46)

232 - 249	734.6841	2201.0306	2201.0198	5	0	K.EVMQEVAQLSQFDEELYK.V	Oxidation (M) (Ions score 42)
232 - 249	1101.5226	2201.0307	2201.0198	5	0	K.EVMQEVAQLSQFDEELYK.V	Oxidation (M) (Ions score 74)
232 - 249	734.6842	2201.0307	2201.0198	5	0	K.EVMQEVAQLSQFDEELYK.V	Oxidation (M) (Ions score 39)
232 - 249	734.6842	2201.0308	2201.0198	5	0	K.EVMQEVAQLSQFDEELYK.V	Oxidation (M) (Ions score 41)
232 - 249	1122.5259	2243.0373	2243.0303	3	0	K.EVMQEVAQLSQFDEELYK.V	Acetylation (K); Oxidation (M) (Ions score 32)
232 - 253	861.7755	2582.3047	2582.2938	4	1	K.EVMQEVAQLSQFDEELYKVIGK.G	(Ions score 41)
232 - 253	861.7757	2582.3053	2582.2938	4	1	K.EVMQEVAQLSQFDEELYKVIGK.G	(Ions score 59)
232 - 253	1300.1531	2598.2916	2598.2887	1	1	K.EVMQEVAQLSQFDEELYKVIGK.G	Oxidation (M) (Ions score 34)
232 - 253	867.1060	2598.2962	2598.2887	3	1	K.EVMQEVAQLSQFDEELYKVIGK.G	Oxidation (M) (Ions score 64)
232 - 253	867.1063	2598.2969	2598.2887	3	1	K.EVMQEVAQLSQFDEELYKVIGK.G	Oxidation (M) (Ions score 44)
232 - 253	867.1071	2598.2993	2598.2887	4	1	K.EVMQEVAQLSQFDEELYKVIGK.G	Oxidation (M) (Ions score 40)
250 - 257	430.2490	858.4834	858.4811	3	1	K.VIGKGSEK.S	Acetylation (K) (Ions score 26)
258 - 266	536.7058	1071.3970	1071.3992	-2	0	K.SDDNSYDEK.Y	(Ions score 55)
258 - 266	536.7059	1071.3973	1071.3992	-2	0	K.SDDNSYDEK.Y	(Ions score 51)
258 - 266	536.7061	1071.3976	1071.3992	-2	0	K.SDDNSYDEK.Y	(Ions score 41)
258 - 266	536.7063	1071.3980	1071.3992	-1	0	K.SDDNSYDEK.Y	(Ions score 57)
258 - 266	536.7063	1071.3980	1071.3992	-1	0	K.SDDNSYDEK.Y	(Ions score 50)
258 - 266	536.7063	1071.3981	1071.3992	-1	0	K.SDDNSYDEK.Y	(Ions score 34)
258 - 266	536.7067	1071.3989	1071.3992	-0	0	K.SDDNSYDEK.Y	(Ions score 35)
258 - 266	536.7069	1071.3992	1071.3992	-0	0	K.SDDNSYDEK.Y	(Ions score 50)
258 - 266	536.7069	1071.3993	1071.3992	0	0	K.SDDNSYDEK.Y	(Ions score 45)
258 - 266	536.7071	1071.3997	1071.3992	0	0	K.SDDNSYDEK.Y	(Ions score 54)
258 - 266	536.7072	1071.3999	1071.3992	1	0	K.SDDNSYDEK.Y	(Ions score 25)
258 - 266	536.7073	1071.4001	1071.3992	1	0	K.SDDNSYDEK.Y	(Ions score 37)
258 - 266	536.7075	1071.4004	1071.3992	1	0	K.SDDNSYDEK.Y	(Ions score 54)
258 - 266	536.7075	1071.4004	1071.3992	1	0	K.SDDNSYDEK.Y	(Ions score 58)
258 - 266	536.7075	1071.4005	1071.3992	1	0	K.SDDNSYDEK.Y	(Ions score 55)
258 - 266	536.7076	1071.4006	1071.3992	1	0	K.SDDNSYDEK.Y	(Ions score 40)
258 - 266	536.7076	1071.4006	1071.3992	1	0	K.SDDNSYDEK.Y	(Ions score 49)
258 - 266	536.7076	1071.4007	1071.3992	1	0	K.SDDNSYDEK.Y	(Ions score 47)
258 - 266	536.7076	1071.4007	1071.3992	1	0	K.SDDNSYDEK.Y	(Ions score 22)
258 - 266	536.7077	1071.4008	1071.3992	1	0	K.SDDNSYDEK.Y	(Ions score 50)
258 - 266	536.7077	1071.4008	1071.3992	1	0	K.SDDNSYDEK.Y	(Ions score 46)
258 - 266	536.7077	1071.4008	1071.3992	1	0	K.SDDNSYDEK.Y	(Ions score 57)
258 - 266	536.7077	1071.4009	1071.3992	2	0	K.SDDNSYDEK.Y	(Ions score 56)
258 - 266	536.7077	1071.4009	1071.3992	2	0	K.SDDNSYDEK.Y	(Ions score 51)
258 - 266	536.7078	1071.4010	1071.3992	2	0	K.SDDNSYDEK.Y	(Ions score 50)
258 - 266	536.7078	1071.4010	1071.3992	2	0	K.SDDNSYDEK.Y	(Ions score 47)
258 - 266	536.7078	1071.4010	1071.3992	2	0	K.SDDNSYDEK.Y	(Ions score 56)
258 - 266	536.7078	1071.4010	1071.3992	2	0	K.SDDNSYDEK.Y	(Ions score 55)
258 - 266	536.7078	1071.4010	1071.3992	2	0	K.SDDNSYDEK.Y	(Ions score 59)
258 - 266	536.7078	1071.4010	1071.3992	2	0	K.SDDNSYDEK.Y	(Ions score 45)
258 - 266	536.7078	1071.4010	1071.3992	2	0	K.SDDNSYDEK.Y	(Ions score 39)
258 - 266	536.7078	1071.4011	1071.3992	2	0	K.SDDNSYDEK.Y	(Ions score 31)
258 - 266	536.7078	1071.4011	1071.3992	2	0	K.SDDNSYDEK.Y	(Ions score 39)
258 - 266	536.7078	1071.4011	1071.3992	2	0	K.SDDNSYDEK.Y	(Ions score 54)
258 - 266	536.7079	1071.4012	1071.3992	2	0	K.SDDNSYDEK.Y	(Ions score 47)
258 - 266	536.7079	1071.4012	1071.3992	2	0	K.SDDNSYDEK.Y	(Ions score 32)
258 - 266	536.7079	1071.4012	1071.3992	2	0	K.SDDNSYDEK.Y	(Ions score 44)
258 - 266	536.7079	1071.4012	1071.3992	2	0	K.SDDNSYDEK.Y	(Ions score 53)
258 - 266	536.7079	1071.4012	1071.3992	2	0	K.SDDNSYDEK.Y	(Ions score 46)
258 - 266	536.7079	1071.4012	1071.3992	2	0	K.SDDNSYDEK.Y	(Ions score 42)
258 - 266	536.7079	1071.4012	1071.3992	2	0	K.SDDNSYDEK.Y	(Ions score 57)
258 - 266	536.7079	1071.4013	1071.3992	2	0	K.SDDNSYDEK.Y	(Ions score 51)
258 - 266	536.7079	1071.4013	1071.3992	2	0	K.SDDNSYDEK.Y	(Ions score 35)
258 - 266	536.7079	1071.4013	1071.3992	2	0	K.SDDNSYDEK.Y	(Ions score 42)
258 - 266	536.7080	1071.4014	1071.3992	2	0	K.SDDNSYDEK.Y	(Ions score 31)
258 - 266	536.7081	1071.4017	1071.3992	2	0	K.SDDNSYDEK.Y	(Ions score 39)

258 - 266	536.7082	1071.4019	1071.3992	2	0	K.SDDNSYDEK.Y		(Ions score 38)
258 - 266	536.7083	1071.4020	1071.3992	3	0	K.SDDNSYDEK.Y		(Ions score 50)
258 - 266	536.7083	1071.4020	1071.3992	3	0	K.SDDNSYDEK.Y		(Ions score 49)
258 - 266	536.7083	1071.4021	1071.3992	3	0	K.SDDNSYDEK.Y		(Ions score 29)
258 - 266	536.7084	1071.4022	1071.3992	3	0	K.SDDNSYDEK.Y		(Ions score 38)
258 - 266	536.7085	1071.4024	1071.3992	3	0	K.SDDNSYDEK.Y		(Ions score 39)
258 - 266	536.7085	1071.4024	1071.3992	3	0	K.SDDNSYDEK.Y		(Ions score 35)
258 - 266	536.7087	1071.4028	1071.3992	3	0	K.SDDNSYDEK.Y		(Ions score 31)
258 - 266	536.7087	1071.4029	1071.3992	3	0	K.SDDNSYDEK.Y		(Ions score 37)
258 - 266	536.7087	1071.4029	1071.3992	3	0	K.SDDNSYDEK.Y		(Ions score 32)
258 - 266	536.7088	1071.4030	1071.3992	4	0	K.SDDNSYDEK.Y		(Ions score 47)
258 - 266	536.7091	1071.4036	1071.3992	4	0	K.SDDNSYDEK.Y		(Ions score 57)
258 - 266	536.7095	1071.4044	1071.3992	5	0	K.SDDNSYDEK.Y		(Ions score 42)
258 - 281	684.8343	2735.3080	2735.3038	2	1	K.SDDNSYDEKYLIATSEQPIAALHR.D		(Ions score 30)
258 - 281	684.8345	2735.3090	2735.3038	2	1	K.SDDNSYDEKYLIATSEQPIAALHR.D		(Ions score 30)
258 - 281	926.7803	2777.3191	2777.3144	2	1	K.SDDNSYDEKYLIATSEQPIAALHR.D	Acetylation (K)	(Ions score 31)
258 - 281	695.3375	2777.3208	2777.3144	2	1	K.SDDNSYDEKYLIATSEQPIAALHR.D	Acetylation (K)	(Ions score 40)
258 - 281	695.3375	2777.3209	2777.3144	2	1	K.SDDNSYDEKYLIATSEQPIAALHR.D	Acetylation (K)	(Ions score 45)
258 - 281	926.7823	2777.3251	2777.3144	4	1	K.SDDNSYDEKYLIATSEQPIAALHR.D	Acetylation (K)	(Ions score 30)
267 - 281	561.6445	1681.9118	1681.9151	-2	0	K.YLIATSEQPIAALHR.D		(Ions score 79)
267 - 281	561.6449	1681.9129	1681.9151	-1	0	K.YLIATSEQPIAALHR.D		(Ions score 47)
267 - 281	561.6449	1681.9129	1681.9151	-1	0	K.YLIATSEQPIAALHR.D		(Ions score 66)
267 - 281	561.6450	1681.9130	1681.9151	-1	0	K.YLIATSEQPIAALHR.D		(Ions score 69)
267 - 281	561.6450	1681.9132	1681.9151	-1	0	K.YLIATSEQPIAALHR.D		(Ions score 63)
267 - 281	561.6450	1681.9132	1681.9151	-1	0	K.YLIATSEQPIAALHR.D		(Ions score 68)
267 - 281	561.6450	1681.9133	1681.9151	-1	0	K.YLIATSEQPIAALHR.D		(Ions score 66)
267 - 281	561.6451	1681.9134	1681.9151	-1	0	K.YLIATSEQPIAALHR.D		(Ions score 54)
267 - 281	561.6451	1681.9134	1681.9151	-1	0	K.YLIATSEQPIAALHR.D		(Ions score 72)
267 - 281	561.6451	1681.9134	1681.9151	-1	0	K.YLIATSEQPIAALHR.D		(Ions score 67)
267 - 281	561.6451	1681.9134	1681.9151	-1	0	K.YLIATSEQPIAALHR.D		(Ions score 69)
267 - 281	561.6452	1681.9137	1681.9151	-1	0	K.YLIATSEQPIAALHR.D		(Ions score 64)
267 - 281	561.6453	1681.9140	1681.9151	-1	0	K.YLIATSEQPIAALHR.D		(Ions score 67)
267 - 281	561.6453	1681.9141	1681.9151	-1	0	K.YLIATSEQPIAALHR.D		(Ions score 58)
267 - 281	561.6454	1681.9143	1681.9151	-0	0	K.YLIATSEQPIAALHR.D		(Ions score 78)
267 - 281	561.6454	1681.9143	1681.9151	-0	0	K.YLIATSEQPIAALHR.D		(Ions score 63)
267 - 281	561.6455	1681.9147	1681.9151	-0	0	K.YLIATSEQPIAALHR.D		(Ions score 70)
267 - 281	561.6455	1681.9147	1681.9151	-0	0	K.YLIATSEQPIAALHR.D		(Ions score 56)
267 - 281	561.6455	1681.9147	1681.9151	-0	0	K.YLIATSEQPIAALHR.D		(Ions score 64)
267 - 281	561.6455	1681.9147	1681.9151	-0	0	K.YLIATSEQPIAALHR.D		(Ions score 70)
267 - 281	561.6455	1681.9148	1681.9151	-0	0	K.YLIATSEQPIAALHR.D		(Ions score 62)
267 - 281	561.6457	1681.9152	1681.9151	0	0	K.YLIATSEQPIAALHR.D		(Ions score 63)
267 - 281	841.9649	1681.9152	1681.9151	0	0	K.YLIATSEQPIAALHR.D		(Ions score 80)
267 - 281	561.6457	1681.9153	1681.9151	0	0	K.YLIATSEQPIAALHR.D		(Ions score 67)
267 - 281	421.4861	1681.9153	1681.9151	0	0	K.YLIATSEQPIAALHR.D		(Ions score 50)
267 - 281	561.6457	1681.9154	1681.9151	0	0	K.YLIATSEQPIAALHR.D		(Ions score 60)
267 - 281	561.6458	1681.9155	1681.9151	0	0	K.YLIATSEQPIAALHR.D		(Ions score 67)
267 - 281	841.9650	1681.9155	1681.9151	0	0	K.YLIATSEQPIAALHR.D		(Ions score 57)
267 - 281	561.6458	1681.9155	1681.9151	0	0	K.YLIATSEQPIAALHR.D		(Ions score 63)
267 - 281	561.6458	1681.9156	1681.9151	0	0	K.YLIATSEQPIAALHR.D		(Ions score 47)
267 - 281	561.6459	1681.9158	1681.9151	0	0	K.YLIATSEQPIAALHR.D		(Ions score 61)
267 - 281	561.6459	1681.9160	1681.9151	1	0	K.YLIATSEQPIAALHR.D		(Ions score 77)
267 - 281	561.6459	1681.9160	1681.9151	1	0	K.YLIATSEQPIAALHR.D		(Ions score 79)
267 - 281	561.6459	1681.9160	1681.9151	1	0	K.YLIATSEQPIAALHR.D		(Ions score 76)
267 - 281	561.6459	1681.9160	1681.9151	1	0	K.YLIATSEQPIAALHR.D		(Ions score 73)
267 - 281	561.6460	1681.9161	1681.9151	1	0	K.YLIATSEQPIAALHR.D		(Ions score 80)
267 - 281	561.6460	1681.9161	1681.9151	1	0	K.YLIATSEQPIAALHR.D		(Ions score 70)
267 - 281	561.6460	1681.9161	1681.9151	1	0	K.YLIATSEQPIAALHR.D		(Ions score 48)
267 - 281	561.6460	1681.9162	1681.9151	1	0	K.YLIATSEQPIAALHR.D		(Ions score 59)

267 - 281	561.6460	1681.9163	1681.9151	1	0	K.YLIATSEQPIAALHR.D	(Ions score 64)
267 - 281	841.9654	1681.9163	1681.9151	1	0	K.YLIATSEQPIAALHR.D	(Ions score 54)
267 - 281	561.6461	1681.9163	1681.9151	1	0	K.YLIATSEQPIAALHR.D	(Ions score 77)
267 - 281	561.6461	1681.9164	1681.9151	1	0	K.YLIATSEQPIAALHR.D	(Ions score 63)
267 - 281	421.4864	1681.9165	1681.9151	1	0	K.YLIATSEQPIAALHR.D	(Ions score 37)
267 - 281	561.6461	1681.9166	1681.9151	1	0	K.YLIATSEQPIAALHR.D	(Ions score 75)
267 - 281	561.6461	1681.9166	1681.9151	1	0	K.YLIATSEQPIAALHR.D	(Ions score 66)
267 - 281	561.6461	1681.9166	1681.9151	1	0	K.YLIATSEQPIAALHR.D	(Ions score 60)
267 - 281	561.6462	1681.9166	1681.9151	1	0	K.YLIATSEQPIAALHR.D	(Ions score 64)
267 - 281	561.6462	1681.9166	1681.9151	1	0	K.YLIATSEQPIAALHR.D	(Ions score 66)
267 - 281	561.6462	1681.9167	1681.9151	1	0	K.YLIATSEQPIAALHR.D	(Ions score 72)
267 - 281	561.6462	1681.9167	1681.9151	1	0	K.YLIATSEQPIAALHR.D	(Ions score 60)
267 - 281	561.6462	1681.9168	1681.9151	1	0	K.YLIATSEQPIAALHR.D	(Ions score 64)
267 - 281	561.6462	1681.9168	1681.9151	1	0	K.YLIATSEQPIAALHR.D	(Ions score 69)
267 - 281	561.6462	1681.9168	1681.9151	1	0	K.YLIATSEQPIAALHR.D	(Ions score 74)
267 - 281	561.6462	1681.9169	1681.9151	1	0	K.YLIATSEQPIAALHR.D	(Ions score 66)
267 - 281	561.6462	1681.9169	1681.9151	1	0	K.YLIATSEQPIAALHR.D	(Ions score 59)
267 - 281	561.6462	1681.9169	1681.9151	1	0	K.YLIATSEQPIAALHR.D	(Ions score 60)
267 - 281	561.6462	1681.9169	1681.9151	1	0	K.YLIATSEQPIAALHR.D	(Ions score 79)
267 - 281	561.6462	1681.9169	1681.9151	1	0	K.YLIATSEQPIAALHR.D	(Ions score 79)
267 - 281	561.6463	1681.9169	1681.9151	1	0	K.YLIATSEQPIAALHR.D	(Ions score 75)
267 - 281	561.6463	1681.9171	1681.9151	1	0	K.YLIATSEQPIAALHR.D	(Ions score 61)
267 - 281	561.6463	1681.9171	1681.9151	1	0	K.YLIATSEQPIAALHR.D	(Ions score 26)
267 - 281	561.6463	1681.9171	1681.9151	1	0	K.YLIATSEQPIAALHR.D	(Ions score 69)
267 - 281	561.6463	1681.9171	1681.9151	1	0	K.YLIATSEQPIAALHR.D	(Ions score 65)
267 - 281	561.6463	1681.9171	1681.9151	1	0	K.YLIATSEQPIAALHR.D	(Ions score 69)
267 - 281	561.6463	1681.9171	1681.9151	1	0	K.YLIATSEQPIAALHR.D	(Ions score 65)
267 - 281	561.6463	1681.9171	1681.9151	1	0	K.YLIATSEQPIAALHR.D	(Ions score 71)
267 - 281	561.6463	1681.9172	1681.9151	1	0	K.YLIATSEQPIAALHR.D	(Ions score 22)
267 - 281	561.6464	1681.9172	1681.9151	1	0	K.YLIATSEQPIAALHR.D	(Ions score 22)
267 - 281	561.6464	1681.9173	1681.9151	1	0	K.YLIATSEQPIAALHR.D	(Ions score 84)
267 - 281	561.6464	1681.9174	1681.9151	1	0	K.YLIATSEQPIAALHR.D	(Ions score 79)
267 - 281	561.6464	1681.9175	1681.9151	1	0	K.YLIATSEQPIAALHR.D	(Ions score 73)
267 - 281	561.6464	1681.9175	1681.9151	1	0	K.YLIATSEQPIAALHR.D	(Ions score 75)
267 - 281	561.6465	1681.9175	1681.9151	1	0	K.YLIATSEQPIAALHR.D	(Ions score 83)
267 - 281	561.6465	1681.9175	1681.9151	1	0	K.YLIATSEQPIAALHR.D	(Ions score 73)
267 - 281	561.6465	1681.9176	1681.9151	1	0	K.YLIATSEQPIAALHR.D	(Ions score 60)
267 - 281	561.6465	1681.9176	1681.9151	1	0	K.YLIATSEQPIAALHR.D	(Ions score 25)
267 - 281	561.6465	1681.9176	1681.9151	1	0	K.YLIATSEQPIAALHR.D	(Ions score 65)
267 - 281	561.6465	1681.9176	1681.9151	1	0	K.YLIATSEQPIAALHR.D	(Ions score 67)
267 - 281	561.6465	1681.9176	1681.9151	1	0	K.YLIATSEQPIAALHR.D	(Ions score 68)
267 - 281	561.6465	1681.9177	1681.9151	2	0	K.YLIATSEQPIAALHR.D	(Ions score 75)
267 - 281	561.6465	1681.9177	1681.9151	2	0	K.YLIATSEQPIAALHR.D	(Ions score 72)
267 - 281	561.6465	1681.9177	1681.9151	2	0	K.YLIATSEQPIAALHR.D	(Ions score 67)
267 - 281	561.6465	1681.9177	1681.9151	2	0	K.YLIATSEQPIAALHR.D	(Ions score 79)
267 - 281	561.6465	1681.9177	1681.9151	2	0	K.YLIATSEQPIAALHR.D	(Ions score 81)
267 - 281	561.6465	1681.9177	1681.9151	2	0	K.YLIATSEQPIAALHR.D	(Ions score 74)
267 - 281	561.6465	1681.9177	1681.9151	2	0	K.YLIATSEQPIAALHR.D	(Ions score 75)
267 - 281	561.6465	1681.9178	1681.9151	2	0	K.YLIATSEQPIAALHR.D	(Ions score 79)
267 - 281	561.6466	1681.9178	1681.9151	2	0	K.YLIATSEQPIAALHR.D	(Ions score 72)
267 - 281	561.6466	1681.9179	1681.9151	2	0	K.YLIATSEQPIAALHR.D	(Ions score 77)
267 - 281	841.9662	1681.9179	1681.9151	2	0	K.YLIATSEQPIAALHR.D	(Ions score 96)
267 - 281	561.6466	1681.9179	1681.9151	2	0	K.YLIATSEQPIAALHR.D	(Ions score 75)
267 - 281	841.9662	1681.9179	1681.9151	2	0	K.YLIATSEQPIAALHR.D	(Ions score 101)
267 - 281	561.6466	1681.9179	1681.9151	2	0	K.YLIATSEQPIAALHR.D	(Ions score 56)
267 - 281	561.6466	1681.9179	1681.9151	2	0	K.YLIATSEQPIAALHR.D	(Ions score 77)
267 - 281	561.6466	1681.9180	1681.9151	2	0	K.YLIATSEQPIAALHR.D	(Ions score 86)
267 - 281	561.6466	1681.9180	1681.9151	2	0	K.YLIATSEQPIAALHR.D	(Ions score 29)

267 - 281	421.4868	1681.9180	1681.9151	2	0	K.YLIATSEQPIAALHR.D	(Ions score 29)
267 - 281	561.6466	1681.9180	1681.9151	2	0	K.YLIATSEQPIAALHR.D	(Ions score 79)
267 - 281	561.6466	1681.9180	1681.9151	2	0	K.YLIATSEQPIAALHR.D	(Ions score 68)
267 - 281	561.6466	1681.9180	1681.9151	2	0	K.YLIATSEQPIAALHR.D	(Ions score 84)
267 - 281	561.6466	1681.9180	1681.9151	2	0	K.YLIATSEQPIAALHR.D	(Ions score 45)
267 - 281	841.9663	1681.9181	1681.9151	2	0	K.YLIATSEQPIAALHR.D	(Ions score 42)
267 - 281	561.6466	1681.9181	1681.9151	2	0	K.YLIATSEQPIAALHR.D	(Ions score 21)
267 - 281	561.6466	1681.9181	1681.9151	2	0	K.YLIATSEQPIAALHR.D	(Ions score 69)
267 - 281	561.6467	1681.9182	1681.9151	2	0	K.YLIATSEQPIAALHR.D	(Ions score 65)
267 - 281	561.6467	1681.9182	1681.9151	2	0	K.YLIATSEQPIAALHR.D	(Ions score 71)
267 - 281	561.6467	1681.9182	1681.9151	2	0	K.YLIATSEQPIAALHR.D	(Ions score 36)
267 - 281	561.6467	1681.9182	1681.9151	2	0	K.YLIATSEQPIAALHR.D	(Ions score 82)
267 - 281	561.6467	1681.9182	1681.9151	2	0	K.YLIATSEQPIAALHR.D	(Ions score 73)
267 - 281	561.6467	1681.9183	1681.9151	2	0	K.YLIATSEQPIAALHR.D	(Ions score 70)
267 - 281	561.6467	1681.9183	1681.9151	2	0	K.YLIATSEQPIAALHR.D	(Ions score 38)
267 - 281	841.9665	1681.9184	1681.9151	2	0	K.YLIATSEQPIAALHR.D	(Ions score 108)
267 - 281	561.6467	1681.9184	1681.9151	2	0	K.YLIATSEQPIAALHR.D	(Ions score 56)
267 - 281	561.6468	1681.9185	1681.9151	2	0	K.YLIATSEQPIAALHR.D	(Ions score 75)
267 - 281	561.6468	1681.9185	1681.9151	2	0	K.YLIATSEQPIAALHR.D	(Ions score 65)
267 - 281	561.6468	1681.9185	1681.9151	2	0	K.YLIATSEQPIAALHR.D	(Ions score 63)
267 - 281	561.6468	1681.9185	1681.9151	2	0	K.YLIATSEQPIAALHR.D	(Ions score 68)
267 - 281	561.6468	1681.9186	1681.9151	2	0	K.YLIATSEQPIAALHR.D	(Ions score 69)
267 - 281	561.6468	1681.9186	1681.9151	2	0	K.YLIATSEQPIAALHR.D	(Ions score 79)
267 - 281	561.6468	1681.9186	1681.9151	2	0	K.YLIATSEQPIAALHR.D	(Ions score 51)
267 - 281	561.6468	1681.9186	1681.9151	2	0	K.YLIATSEQPIAALHR.D	(Ions score 70)
267 - 281	561.6468	1681.9187	1681.9151	2	0	K.YLIATSEQPIAALHR.D	(Ions score 78)
267 - 281	561.6468	1681.9187	1681.9151	2	0	K.YLIATSEQPIAALHR.D	(Ions score 74)
267 - 281	561.6468	1681.9187	1681.9151	2	0	K.YLIATSEQPIAALHR.D	(Ions score 65)
267 - 281	561.6468	1681.9187	1681.9151	2	0	K.YLIATSEQPIAALHR.D	(Ions score 57)
267 - 281	561.6468	1681.9187	1681.9151	2	0	K.YLIATSEQPIAALHR.D	(Ions score 91)
267 - 281	561.6468	1681.9187	1681.9151	2	0	K.YLIATSEQPIAALHR.D	(Ions score 30)
267 - 281	561.6469	1681.9187	1681.9151	2	0	K.YLIATSEQPIAALHR.D	(Ions score 66)
267 - 281	841.9667	1681.9188	1681.9151	2	0	K.YLIATSEQPIAALHR.D	(Ions score 87)
267 - 281	561.6469	1681.9189	1681.9151	2	0	K.YLIATSEQPIAALHR.D	(Ions score 47)
267 - 281	561.6469	1681.9189	1681.9151	2	0	K.YLIATSEQPIAALHR.D	(Ions score 73)
267 - 281	561.6469	1681.9189	1681.9151	2	0	K.YLIATSEQPIAALHR.D	(Ions score 75)
267 - 281	561.6469	1681.9189	1681.9151	2	0	K.YLIATSEQPIAALHR.D	(Ions score 63)
267 - 281	561.6469	1681.9189	1681.9151	2	0	K.YLIATSEQPIAALHR.D	(Ions score 65)
267 - 281	561.6469	1681.9189	1681.9151	2	0	K.YLIATSEQPIAALHR.D	(Ions score 79)
267 - 281	561.6469	1681.9190	1681.9151	2	0	K.YLIATSEQPIAALHR.D	(Ions score 72)
267 - 281	561.6469	1681.9190	1681.9151	2	0	K.YLIATSEQPIAALHR.D	(Ions score 70)
267 - 281	561.6469	1681.9190	1681.9151	2	0	K.YLIATSEQPIAALHR.D	(Ions score 79)
267 - 281	561.6469	1681.9190	1681.9151	2	0	K.YLIATSEQPIAALHR.D	(Ions score 57)
267 - 281	561.6469	1681.9190	1681.9151	2	0	K.YLIATSEQPIAALHR.D	(Ions score 77)
267 - 281	561.6469	1681.9190	1681.9151	2	0	K.YLIATSEQPIAALHR.D	(Ions score 71)
267 - 281	561.6469	1681.9190	1681.9151	2	0	K.YLIATSEQPIAALHR.D	(Ions score 75)
267 - 281	561.6470	1681.9190	1681.9151	2	0	K.YLIATSEQPIAALHR.D	(Ions score 79)
267 - 281	841.9668	1681.9190	1681.9151	2	0	K.YLIATSEQPIAALHR.D	(Ions score 87)
267 - 281	561.6470	1681.9191	1681.9151	2	0	K.YLIATSEQPIAALHR.D	(Ions score 57)
267 - 281	561.6470	1681.9191	1681.9151	2	0	K.YLIATSEQPIAALHR.D	(Ions score 68)
267 - 281	561.6470	1681.9191	1681.9151	2	0	K.YLIATSEQPIAALHR.D	(Ions score 64)
267 - 281	561.6470	1681.9191	1681.9151	2	0	K.YLIATSEQPIAALHR.D	(Ions score 38)
267 - 281	561.6470	1681.9191	1681.9151	2	0	K.YLIATSEQPIAALHR.D	(Ions score 80)
267 - 281	561.6470	1681.9191	1681.9151	2	0	K.YLIATSEQPIAALHR.D	(Ions score 79)
267 - 281	561.6470	1681.9192	1681.9151	2	0	K.YLIATSEQPIAALHR.D	(Ions score 60)
267 - 281	841.9669	1681.9192	1681.9151	2	0	K.YLIATSEQPIAALHR.D	(Ions score 45)
267 - 281	561.6470	1681.9193	1681.9151	2	0	K.YLIATSEQPIAALHR.D	(Ions score 88)
267 - 281	561.6470	1681.9193	1681.9151	2	0	K.YLIATSEQPIAALHR.D	(Ions score 40)

267 - 281	561.6470	1681.9193	1681.9151	2	0	K.YLIATSEQPIAALHR.D	(Ions score 82)
267 - 281	561.6470	1681.9193	1681.9151	2	0	K.YLIATSEQPIAALHR.D	(Ions score 42)
267 - 281	561.6470	1681.9193	1681.9151	2	0	K.YLIATSEQPIAALHR.D	(Ions score 71)
267 - 281	841.9669	1681.9193	1681.9151	2	0	K.YLIATSEQPIAALHR.D	(Ions score 98)
267 - 281	561.6470	1681.9193	1681.9151	2	0	K.YLIATSEQPIAALHR.D	(Ions score 77)
267 - 281	561.6471	1681.9193	1681.9151	3	0	K.YLIATSEQPIAALHR.D	(Ions score 77)
267 - 281	561.6471	1681.9193	1681.9151	3	0	K.YLIATSEQPIAALHR.D	(Ions score 72)
267 - 281	561.6471	1681.9194	1681.9151	3	0	K.YLIATSEQPIAALHR.D	(Ions score 65)
267 - 281	841.9670	1681.9194	1681.9151	3	0	K.YLIATSEQPIAALHR.D	(Ions score 103)
267 - 281	561.6471	1681.9194	1681.9151	3	0	K.YLIATSEQPIAALHR.D	(Ions score 75)
267 - 281	561.6471	1681.9194	1681.9151	3	0	K.YLIATSEQPIAALHR.D	(Ions score 77)
267 - 281	561.6471	1681.9195	1681.9151	3	0	K.YLIATSEQPIAALHR.D	(Ions score 67)
267 - 281	561.6471	1681.9195	1681.9151	3	0	K.YLIATSEQPIAALHR.D	(Ions score 55)
267 - 281	561.6471	1681.9195	1681.9151	3	0	K.YLIATSEQPIAALHR.D	(Ions score 75)
267 - 281	561.6471	1681.9195	1681.9151	3	0	K.YLIATSEQPIAALHR.D	(Ions score 67)
267 - 281	561.6471	1681.9195	1681.9151	3	0	K.YLIATSEQPIAALHR.D	(Ions score 53)
267 - 281	561.6471	1681.9195	1681.9151	3	0	K.YLIATSEQPIAALHR.D	(Ions score 77)
267 - 281	561.6471	1681.9195	1681.9151	3	0	K.YLIATSEQPIAALHR.D	(Ions score 69)
267 - 281	841.9670	1681.9195	1681.9151	3	0	K.YLIATSEQPIAALHR.D	(Ions score 86)
267 - 281	561.6471	1681.9195	1681.9151	3	0	K.YLIATSEQPIAALHR.D	(Ions score 83)
267 - 281	561.6471	1681.9195	1681.9151	3	0	K.YLIATSEQPIAALHR.D	(Ions score 71)
267 - 281	841.9670	1681.9195	1681.9151	3	0	K.YLIATSEQPIAALHR.D	(Ions score 42)
267 - 281	561.6471	1681.9195	1681.9151	3	0	K.YLIATSEQPIAALHR.D	(Ions score 76)
267 - 281	561.6471	1681.9196	1681.9151	3	0	K.YLIATSEQPIAALHR.D	(Ions score 66)
267 - 281	561.6471	1681.9196	1681.9151	3	0	K.YLIATSEQPIAALHR.D	(Ions score 69)
267 - 281	561.6471	1681.9196	1681.9151	3	0	K.YLIATSEQPIAALHR.D	(Ions score 68)
267 - 281	841.9671	1681.9196	1681.9151	3	0	K.YLIATSEQPIAALHR.D	(Ions score 82)
267 - 281	561.6472	1681.9196	1681.9151	3	0	K.YLIATSEQPIAALHR.D	(Ions score 32)
267 - 281	561.6472	1681.9196	1681.9151	3	0	K.YLIATSEQPIAALHR.D	(Ions score 67)
267 - 281	561.6472	1681.9197	1681.9151	3	0	K.YLIATSEQPIAALHR.D	(Ions score 76)
267 - 281	561.6472	1681.9197	1681.9151	3	0	K.YLIATSEQPIAALHR.D	(Ions score 62)
267 - 281	561.6472	1681.9197	1681.9151	3	0	K.YLIATSEQPIAALHR.D	(Ions score 76)
267 - 281	561.6472	1681.9198	1681.9151	3	0	K.YLIATSEQPIAALHR.D	(Ions score 67)
267 - 281	561.6472	1681.9198	1681.9151	3	0	K.YLIATSEQPIAALHR.D	(Ions score 74)
267 - 281	561.6472	1681.9198	1681.9151	3	0	K.YLIATSEQPIAALHR.D	(Ions score 72)
267 - 281	561.6472	1681.9198	1681.9151	3	0	K.YLIATSEQPIAALHR.D	(Ions score 56)
267 - 281	561.6472	1681.9198	1681.9151	3	0	K.YLIATSEQPIAALHR.D	(Ions score 73)
267 - 281	561.6472	1681.9198	1681.9151	3	0	K.YLIATSEQPIAALHR.D	(Ions score 53)
267 - 281	561.6472	1681.9198	1681.9151	3	0	K.YLIATSEQPIAALHR.D	(Ions score 72)
267 - 281	561.6472	1681.9198	1681.9151	3	0	K.YLIATSEQPIAALHR.D	(Ions score 71)
267 - 281	561.6472	1681.9198	1681.9151	3	0	K.YLIATSEQPIAALHR.D	(Ions score 70)
267 - 281	841.9672	1681.9198	1681.9151	3	0	K.YLIATSEQPIAALHR.D	(Ions score 33)
267 - 281	561.6472	1681.9199	1681.9151	3	0	K.YLIATSEQPIAALHR.D	(Ions score 27)
267 - 281	561.6472	1681.9199	1681.9151	3	0	K.YLIATSEQPIAALHR.D	(Ions score 55)
267 - 281	561.6473	1681.9200	1681.9151	3	0	K.YLIATSEQPIAALHR.D	(Ions score 73)
267 - 281	841.9673	1681.9200	1681.9151	3	0	K.YLIATSEQPIAALHR.D	(Ions score 59)
267 - 281	561.6473	1681.9200	1681.9151	3	0	K.YLIATSEQPIAALHR.D	(Ions score 73)
267 - 281	561.6473	1681.9200	1681.9151	3	0	K.YLIATSEQPIAALHR.D	(Ions score 53)
267 - 281	561.6473	1681.9200	1681.9151	3	0	K.YLIATSEQPIAALHR.D	(Ions score 70)
267 - 281	561.6473	1681.9201	1681.9151	3	0	K.YLIATSEQPIAALHR.D	(Ions score 77)
267 - 281	841.9673	1681.9201	1681.9151	3	0	K.YLIATSEQPIAALHR.D	(Ions score 90)
267 - 281	561.6473	1681.9201	1681.9151	3	0	K.YLIATSEQPIAALHR.D	(Ions score 71)
267 - 281	561.6473	1681.9201	1681.9151	3	0	K.YLIATSEQPIAALHR.D	(Ions score 66)
267 - 281	561.6473	1681.9201	1681.9151	3	0	K.YLIATSEQPIAALHR.D	(Ions score 78)
267 - 281	561.6473	1681.9201	1681.9151	3	0	K.YLIATSEQPIAALHR.D	(Ions score 79)
267 - 281	561.6473	1681.9201	1681.9151	3	0	K.YLIATSEQPIAALHR.D	(Ions score 61)
267 - 281	561.6473	1681.9201	1681.9151	3	0	K.YLIATSEQPIAALHR.D	(Ions score 73)
267 - 281	561.6473	1681.9201	1681.9151	3	0	K.YLIATSEQPIAALHR.D	(Ions score 64)

267 - 281	561.6473	1681.9202	1681.9151	3	0	K.YLIATSEQPIAALHR.D	(Ions score 76)
267 - 281	561.6474	1681.9202	1681.9151	3	0	K.YLIATSEQPIAALHR.D	(Ions score 65)
267 - 281	561.6474	1681.9202	1681.9151	3	0	K.YLIATSEQPIAALHR.D	(Ions score 54)
267 - 281	561.6474	1681.9203	1681.9151	3	0	K.YLIATSEQPIAALHR.D	(Ions score 68)
267 - 281	561.6474	1681.9203	1681.9151	3	0	K.YLIATSEQPIAALHR.D	(Ions score 75)
267 - 281	561.6474	1681.9203	1681.9151	3	0	K.YLIATSEQPIAALHR.D	(Ions score 75)
267 - 281	561.6474	1681.9203	1681.9151	3	0	K.YLIATSEQPIAALHR.D	(Ions score 65)
267 - 281	561.6474	1681.9203	1681.9151	3	0	K.YLIATSEQPIAALHR.D	(Ions score 61)
267 - 281	561.6474	1681.9203	1681.9151	3	0	K.YLIATSEQPIAALHR.D	(Ions score 71)
267 - 281	561.6474	1681.9203	1681.9151	3	0	K.YLIATSEQPIAALHR.D	(Ions score 67)
267 - 281	561.6474	1681.9204	1681.9151	3	0	K.YLIATSEQPIAALHR.D	(Ions score 73)
267 - 281	561.6474	1681.9204	1681.9151	3	0	K.YLIATSEQPIAALHR.D	(Ions score 74)
267 - 281	561.6474	1681.9204	1681.9151	3	0	K.YLIATSEQPIAALHR.D	(Ions score 69)
267 - 281	561.6474	1681.9204	1681.9151	3	0	K.YLIATSEQPIAALHR.D	(Ions score 69)
267 - 281	561.6474	1681.9204	1681.9151	3	0	K.YLIATSEQPIAALHR.D	(Ions score 63)
267 - 281	561.6474	1681.9205	1681.9151	3	0	K.YLIATSEQPIAALHR.D	(Ions score 58)
267 - 281	561.6474	1681.9205	1681.9151	3	0	K.YLIATSEQPIAALHR.D	(Ions score 77)
267 - 281	561.6474	1681.9205	1681.9151	3	0	K.YLIATSEQPIAALHR.D	(Ions score 57)
267 - 281	561.6475	1681.9205	1681.9151	3	0	K.YLIATSEQPIAALHR.D	(Ions score 73)
267 - 281	561.6475	1681.9205	1681.9151	3	0	K.YLIATSEQPIAALHR.D	(Ions score 57)
267 - 281	561.6475	1681.9205	1681.9151	3	0	K.YLIATSEQPIAALHR.D	(Ions score 75)
267 - 281	561.6475	1681.9205	1681.9151	3	0	K.YLIATSEQPIAALHR.D	(Ions score 67)
267 - 281	561.6475	1681.9206	1681.9151	3	0	K.YLIATSEQPIAALHR.D	(Ions score 30)
267 - 281	561.6475	1681.9206	1681.9151	3	0	K.YLIATSEQPIAALHR.D	(Ions score 78)
267 - 281	561.6475	1681.9206	1681.9151	3	0	K.YLIATSEQPIAALHR.D	(Ions score 83)
267 - 281	561.6475	1681.9206	1681.9151	3	0	K.YLIATSEQPIAALHR.D	(Ions score 34)
267 - 281	561.6475	1681.9206	1681.9151	3	0	K.YLIATSEQPIAALHR.D	(Ions score 69)
267 - 281	561.6475	1681.9207	1681.9151	3	0	K.YLIATSEQPIAALHR.D	(Ions score 67)
267 - 281	561.6475	1681.9207	1681.9151	3	0	K.YLIATSEQPIAALHR.D	(Ions score 66)
267 - 281	561.6475	1681.9207	1681.9151	3	0	K.YLIATSEQPIAALHR.D	(Ions score 78)
267 - 281	561.6475	1681.9207	1681.9151	3	0	K.YLIATSEQPIAALHR.D	(Ions score 91)
267 - 281	561.6475	1681.9207	1681.9151	3	0	K.YLIATSEQPIAALHR.D	(Ions score 72)
267 - 281	561.6475	1681.9208	1681.9151	3	0	K.YLIATSEQPIAALHR.D	(Ions score 64)
267 - 281	561.6475	1681.9208	1681.9151	3	0	K.YLIATSEQPIAALHR.D	(Ions score 72)
267 - 281	841.9677	1681.9208	1681.9151	3	0	K.YLIATSEQPIAALHR.D	(Ions score 91)
267 - 281	561.6476	1681.9208	1681.9151	3	0	K.YLIATSEQPIAALHR.D	(Ions score 62)
267 - 281	841.9677	1681.9209	1681.9151	3	0	K.YLIATSEQPIAALHR.D	(Ions score 81)
267 - 281	561.6476	1681.9209	1681.9151	3	0	K.YLIATSEQPIAALHR.D	(Ions score 79)
267 - 281	561.6476	1681.9209	1681.9151	3	0	K.YLIATSEQPIAALHR.D	(Ions score 57)
267 - 281	561.6476	1681.9209	1681.9151	3	0	K.YLIATSEQPIAALHR.D	(Ions score 76)
267 - 281	561.6476	1681.9209	1681.9151	3	0	K.YLIATSEQPIAALHR.D	(Ions score 72)
267 - 281	561.6476	1681.9209	1681.9151	3	0	K.YLIATSEQPIAALHR.D	(Ions score 70)
267 - 281	561.6476	1681.9209	1681.9151	3	0	K.YLIATSEQPIAALHR.D	(Ions score 65)
267 - 281	561.6476	1681.9210	1681.9151	4	0	K.YLIATSEQPIAALHR.D	(Ions score 78)
267 - 281	561.6476	1681.9210	1681.9151	4	0	K.YLIATSEQPIAALHR.D	(Ions score 63)
267 - 281	561.6476	1681.9211	1681.9151	4	0	K.YLIATSEQPIAALHR.D	(Ions score 79)
267 - 281	561.6476	1681.9211	1681.9151	4	0	K.YLIATSEQPIAALHR.D	(Ions score 55)
267 - 281	561.6476	1681.9211	1681.9151	4	0	K.YLIATSEQPIAALHR.D	(Ions score 70)
267 - 281	561.6476	1681.9211	1681.9151	4	0	K.YLIATSEQPIAALHR.D	(Ions score 60)
267 - 281	561.6476	1681.9211	1681.9151	4	0	K.YLIATSEQPIAALHR.D	(Ions score 70)
267 - 281	561.6476	1681.9211	1681.9151	4	0	K.YLIATSEQPIAALHR.D	(Ions score 63)
267 - 281	561.6477	1681.9211	1681.9151	4	0	K.YLIATSEQPIAALHR.D	(Ions score 57)
267 - 281	561.6477	1681.9212	1681.9151	4	0	K.YLIATSEQPIAALHR.D	(Ions score 66)
267 - 281	561.6477	1681.9212	1681.9151	4	0	K.YLIATSEQPIAALHR.D	(Ions score 73)
267 - 281	561.6477	1681.9212	1681.9151	4	0	K.YLIATSEQPIAALHR.D	(Ions score 75)
267 - 281	561.6477	1681.9213	1681.9151	4	0	K.YLIATSEQPIAALHR.D	(Ions score 67)
267 - 281	561.6477	1681.9213	1681.9151	4	0	K.YLIATSEQPIAALHR.D	(Ions score 54)
267 - 281	561.6477	1681.9213	1681.9151	4	0	K.YLIATSEQPIAALHR.D	(Ions score 77)

267 - 281	561.6477	1681.9213	1681.9151	4	0	K.YLIATSEQPIAALHR.D	(Ions score 61)
267 - 281	561.6477	1681.9213	1681.9151	4	0	K.YLIATSEQPIAALHR.D	(Ions score 73)
267 - 281	561.6477	1681.9214	1681.9151	4	0	K.YLIATSEQPIAALHR.D	(Ions score 69)
267 - 281	841.9680	1681.9214	1681.9151	4	0	K.YLIATSEQPIAALHR.D	(Ions score 46)
267 - 281	841.9680	1681.9214	1681.9151	4	0	K.YLIATSEQPIAALHR.D	(Ions score 60)
267 - 281	561.6477	1681.9214	1681.9151	4	0	K.YLIATSEQPIAALHR.D	(Ions score 75)
267 - 281	561.6478	1681.9214	1681.9151	4	0	K.YLIATSEQPIAALHR.D	(Ions score 70)
267 - 281	561.6478	1681.9215	1681.9151	4	0	K.YLIATSEQPIAALHR.D	(Ions score 87)
267 - 281	561.6478	1681.9215	1681.9151	4	0	K.YLIATSEQPIAALHR.D	(Ions score 68)
267 - 281	561.6478	1681.9215	1681.9151	4	0	K.YLIATSEQPIAALHR.D	(Ions score 76)
267 - 281	561.6478	1681.9215	1681.9151	4	0	K.YLIATSEQPIAALHR.D	(Ions score 45)
267 - 281	561.6478	1681.9215	1681.9151	4	0	K.YLIATSEQPIAALHR.D	(Ions score 70)
267 - 281	561.6478	1681.9216	1681.9151	4	0	K.YLIATSEQPIAALHR.D	(Ions score 68)
267 - 281	561.6478	1681.9216	1681.9151	4	0	K.YLIATSEQPIAALHR.D	(Ions score 64)
267 - 281	561.6478	1681.9216	1681.9151	4	0	K.YLIATSEQPIAALHR.D	(Ions score 76)
267 - 281	561.6478	1681.9217	1681.9151	4	0	K.YLIATSEQPIAALHR.D	(Ions score 72)
267 - 281	561.6478	1681.9217	1681.9151	4	0	K.YLIATSEQPIAALHR.D	(Ions score 72)
267 - 281	561.6478	1681.9217	1681.9151	4	0	K.YLIATSEQPIAALHR.D	(Ions score 75)
267 - 281	561.6479	1681.9217	1681.9151	4	0	K.YLIATSEQPIAALHR.D	(Ions score 70)
267 - 281	561.6479	1681.9217	1681.9151	4	0	K.YLIATSEQPIAALHR.D	(Ions score 26)
267 - 281	561.6479	1681.9218	1681.9151	4	0	K.YLIATSEQPIAALHR.D	(Ions score 77)
267 - 281	561.6479	1681.9218	1681.9151	4	0	K.YLIATSEQPIAALHR.D	(Ions score 60)
267 - 281	561.6479	1681.9219	1681.9151	4	0	K.YLIATSEQPIAALHR.D	(Ions score 55)
267 - 281	561.6479	1681.9219	1681.9151	4	0	K.YLIATSEQPIAALHR.D	(Ions score 71)
267 - 281	561.6479	1681.9219	1681.9151	4	0	K.YLIATSEQPIAALHR.D	(Ions score 63)
267 - 281	561.6479	1681.9220	1681.9151	4	0	K.YLIATSEQPIAALHR.D	(Ions score 65)
267 - 281	841.9683	1681.9221	1681.9151	4	0	K.YLIATSEQPIAALHR.D	(Ions score 65)
267 - 281	561.6480	1681.9221	1681.9151	4	0	K.YLIATSEQPIAALHR.D	(Ions score 69)
267 - 281	561.6480	1681.9221	1681.9151	4	0	K.YLIATSEQPIAALHR.D	(Ions score 76)
267 - 281	561.6480	1681.9223	1681.9151	4	0	K.YLIATSEQPIAALHR.D	(Ions score 67)
267 - 281	561.6480	1681.9223	1681.9151	4	0	K.YLIATSEQPIAALHR.D	(Ions score 87)
267 - 281	561.6481	1681.9223	1681.9151	4	0	K.YLIATSEQPIAALHR.D	(Ions score 66)
267 - 281	561.6481	1681.9223	1681.9151	4	0	K.YLIATSEQPIAALHR.D	(Ions score 43)
267 - 281	561.6481	1681.9223	1681.9151	4	0	K.YLIATSEQPIAALHR.D	(Ions score 67)
267 - 281	561.6481	1681.9224	1681.9151	4	0	K.YLIATSEQPIAALHR.D	(Ions score 59)
267 - 281	561.6481	1681.9224	1681.9151	4	0	K.YLIATSEQPIAALHR.D	(Ions score 75)
267 - 281	561.6481	1681.9225	1681.9151	4	0	K.YLIATSEQPIAALHR.D	(Ions score 70)
267 - 281	561.6481	1681.9225	1681.9151	4	0	K.YLIATSEQPIAALHR.D	(Ions score 58)
267 - 281	561.6481	1681.9225	1681.9151	4	0	K.YLIATSEQPIAALHR.D	(Ions score 58)
267 - 281	561.6481	1681.9226	1681.9151	4	0	K.YLIATSEQPIAALHR.D	(Ions score 75)
267 - 281	561.6481	1681.9226	1681.9151	4	0	K.YLIATSEQPIAALHR.D	(Ions score 69)
267 - 281	561.6481	1681.9226	1681.9151	4	0	K.YLIATSEQPIAALHR.D	(Ions score 42)
267 - 281	561.6482	1681.9226	1681.9151	4	0	K.YLIATSEQPIAALHR.D	(Ions score 61)
267 - 281	561.6482	1681.9226	1681.9151	4	0	K.YLIATSEQPIAALHR.D	(Ions score 63)
267 - 281	841.9686	1681.9227	1681.9151	5	0	K.YLIATSEQPIAALHR.D	(Ions score 65)
267 - 281	561.6482	1681.9228	1681.9151	5	0	K.YLIATSEQPIAALHR.D	(Ions score 73)
267 - 281	561.6482	1681.9228	1681.9151	5	0	K.YLIATSEQPIAALHR.D	(Ions score 74)
267 - 281	561.6482	1681.9229	1681.9151	5	0	K.YLIATSEQPIAALHR.D	(Ions score 67)
267 - 281	561.6482	1681.9229	1681.9151	5	0	K.YLIATSEQPIAALHR.D	(Ions score 85)
267 - 281	561.6482	1681.9229	1681.9151	5	0	K.YLIATSEQPIAALHR.D	(Ions score 58)
267 - 281	561.6482	1681.9229	1681.9151	5	0	K.YLIATSEQPIAALHR.D	(Ions score 78)
267 - 281	561.6482	1681.9229	1681.9151	5	0	K.YLIATSEQPIAALHR.D	(Ions score 54)
267 - 281	561.6482	1681.9229	1681.9151	5	0	K.YLIATSEQPIAALHR.D	(Ions score 79)
267 - 281	841.9688	1681.9230	1681.9151	5	0	K.YLIATSEQPIAALHR.D	(Ions score 66)
267 - 281	841.9688	1681.9230	1681.9151	5	0	K.YLIATSEQPIAALHR.D	(Ions score 56)
267 - 281	561.6483	1681.9230	1681.9151	5	0	K.YLIATSEQPIAALHR.D	(Ions score 67)
267 - 281	561.6483	1681.9230	1681.9151	5	0	K.YLIATSEQPIAALHR.D	(Ions score 70)
267 - 281	561.6483	1681.9230	1681.9151	5	0	K.YLIATSEQPIAALHR.D	(Ions score 48)

267 - 281	561.6483	1681.9231	1681.9151	5	0	K.YLIATSEQPIAALHR.D	(Ions score 66)
267 - 281	561.6483	1681.9231	1681.9151	5	0	K.YLIATSEQPIAALHR.D	(Ions score 72)
267 - 281	561.6483	1681.9231	1681.9151	5	0	K.YLIATSEQPIAALHR.D	(Ions score 64)
267 - 281	561.6483	1681.9231	1681.9151	5	0	K.YLIATSEQPIAALHR.D	(Ions score 63)
267 - 281	561.6484	1681.9232	1681.9151	5	0	K.YLIATSEQPIAALHR.D	(Ions score 39)
267 - 281	561.6484	1681.9233	1681.9151	5	0	K.YLIATSEQPIAALHR.D	(Ions score 69)
267 - 281	561.6484	1681.9234	1681.9151	5	0	K.YLIATSEQPIAALHR.D	(Ions score 69)
267 - 281	561.6484	1681.9234	1681.9151	5	0	K.YLIATSEQPIAALHR.D	(Ions score 62)
267 - 281	561.6484	1681.9235	1681.9151	5	0	K.YLIATSEQPIAALHR.D	(Ions score 52)
267 - 281	561.6484	1681.9235	1681.9151	5	0	K.YLIATSEQPIAALHR.D	(Ions score 70)
282 - 293	755.8979	1509.7813	1509.7827	-1	0	R.DEWLRPEDLPIK.Y	(Ions score 26)
282 - 293	755.8982	1509.7818	1509.7827	-1	0	R.DEWLRPEDLPIK.Y	(Ions score 48)
282 - 293	755.8983	1509.7820	1509.7827	-0	0	R.DEWLRPEDLPIK.Y	(Ions score 26)
282 - 293	755.8983	1509.7821	1509.7827	-0	0	R.DEWLRPEDLPIK.Y	(Ions score 51)
282 - 293	755.8989	1509.7832	1509.7827	0	0	R.DEWLRPEDLPIK.Y	(Ions score 37)
282 - 293	755.8989	1509.7832	1509.7827	0	0	R.DEWLRPEDLPIK.Y	(Ions score 26)
282 - 293	755.8990	1509.7834	1509.7827	0	0	R.DEWLRPEDLPIK.Y	(Ions score 40)
282 - 293	755.8991	1509.7836	1509.7827	1	0	R.DEWLRPEDLPIK.Y	(Ions score 37)
282 - 293	755.8991	1509.7836	1509.7827	1	0	R.DEWLRPEDLPIK.Y	(Ions score 26)
282 - 293	755.8991	1509.7837	1509.7827	1	0	R.DEWLRPEDLPIK.Y	(Ions score 48)
282 - 293	755.8992	1509.7839	1509.7827	1	0	R.DEWLRPEDLPIK.Y	(Ions score 30)
282 - 293	755.8993	1509.7841	1509.7827	1	0	R.DEWLRPEDLPIK.Y	(Ions score 46)
282 - 293	755.8995	1509.7844	1509.7827	1	0	R.DEWLRPEDLPIK.Y	(Ions score 43)
282 - 293	755.8995	1509.7844	1509.7827	1	0	R.DEWLRPEDLPIK.Y	(Ions score 44)
282 - 293	755.8996	1509.7846	1509.7827	1	0	R.DEWLRPEDLPIK.Y	(Ions score 39)
282 - 293	755.8996	1509.7847	1509.7827	1	0	R.DEWLRPEDLPIK.Y	(Ions score 37)
282 - 293	755.8996	1509.7847	1509.7827	1	0	R.DEWLRPEDLPIK.Y	(Ions score 39)
282 - 293	755.8997	1509.7849	1509.7827	1	0	R.DEWLRPEDLPIK.Y	(Ions score 33)
282 - 293	504.2689	1509.7849	1509.7827	1	0	R.DEWLRPEDLPIK.Y	(Ions score 22)
282 - 293	755.8998	1509.7851	1509.7827	2	0	R.DEWLRPEDLPIK.Y	(Ions score 52)
282 - 293	755.8998	1509.7851	1509.7827	2	0	R.DEWLRPEDLPIK.Y	(Ions score 48)
282 - 293	755.8998	1509.7851	1509.7827	2	0	R.DEWLRPEDLPIK.Y	(Ions score 44)
282 - 293	755.8999	1509.7852	1509.7827	2	0	R.DEWLRPEDLPIK.Y	(Ions score 41)
282 - 293	755.8999	1509.7852	1509.7827	2	0	R.DEWLRPEDLPIK.Y	(Ions score 37)
282 - 293	755.8999	1509.7852	1509.7827	2	0	R.DEWLRPEDLPIK.Y	(Ions score 39)
282 - 293	755.8999	1509.7853	1509.7827	2	0	R.DEWLRPEDLPIK.Y	(Ions score 39)
282 - 293	504.2691	1509.7854	1509.7827	2	0	R.DEWLRPEDLPIK.Y	(Ions score 22)
282 - 293	755.9000	1509.7854	1509.7827	2	0	R.DEWLRPEDLPIK.Y	(Ions score 45)
282 - 293	755.9000	1509.7854	1509.7827	2	0	R.DEWLRPEDLPIK.Y	(Ions score 36)
282 - 293	755.9001	1509.7857	1509.7827	2	0	R.DEWLRPEDLPIK.Y	(Ions score 26)
282 - 293	504.2692	1509.7858	1509.7827	2	0	R.DEWLRPEDLPIK.Y	(Ions score 21)
282 - 293	755.9002	1509.7858	1509.7827	2	0	R.DEWLRPEDLPIK.Y	(Ions score 29)
282 - 293	755.9003	1509.7860	1509.7827	2	0	R.DEWLRPEDLPIK.Y	(Ions score 53)
282 - 293	755.9003	1509.7860	1509.7827	2	0	R.DEWLRPEDLPIK.Y	(Ions score 48)
282 - 293	755.9003	1509.7860	1509.7827	2	0	R.DEWLRPEDLPIK.Y	(Ions score 52)
282 - 293	755.9003	1509.7860	1509.7827	2	0	R.DEWLRPEDLPIK.Y	(Ions score 53)
282 - 293	755.9003	1509.7860	1509.7827	2	0	R.DEWLRPEDLPIK.Y	(Ions score 54)
282 - 293	755.9003	1509.7861	1509.7827	2	0	R.DEWLRPEDLPIK.Y	(Ions score 42)
282 - 293	755.9004	1509.7862	1509.7827	2	0	R.DEWLRPEDLPIK.Y	(Ions score 50)
282 - 293	755.9004	1509.7862	1509.7827	2	0	R.DEWLRPEDLPIK.Y	(Ions score 54)
282 - 293	755.9004	1509.7862	1509.7827	2	0	R.DEWLRPEDLPIK.Y	(Ions score 53)
282 - 293	755.9004	1509.7862	1509.7827	2	0	R.DEWLRPEDLPIK.Y	(Ions score 53)
282 - 293	755.9004	1509.7863	1509.7827	2	0	R.DEWLRPEDLPIK.Y	(Ions score 57)
282 - 293	755.9004	1509.7863	1509.7827	2	0	R.DEWLRPEDLPIK.Y	(Ions score 43)
282 - 293	755.9005	1509.7864	1509.7827	2	0	R.DEWLRPEDLPIK.Y	(Ions score 52)
282 - 293	755.9005	1509.7864	1509.7827	2	0	R.DEWLRPEDLPIK.Y	(Ions score 45)
282 - 293	755.9005	1509.7864	1509.7827	2	0	R.DEWLRPEDLPIK.Y	(Ions score 64)
282 - 293	755.9005	1509.7864	1509.7827	2	0	R.DEWLRPEDLPIK.Y	(Ions score 37)

282 - 293	755.9005	1509.7865	1509.7827	3	0	R.DEWLRPEDLPIK.Y	(Ions score 57)
282 - 293	755.9006	1509.7866	1509.7827	3	0	R.DEWLRPEDLPIK.Y	(Ions score 55)
282 - 293	755.9006	1509.7866	1509.7827	3	0	R.DEWLRPEDLPIK.Y	(Ions score 40)
282 - 293	755.9006	1509.7867	1509.7827	3	0	R.DEWLRPEDLPIK.Y	(Ions score 44)
282 - 293	755.9006	1509.7867	1509.7827	3	0	R.DEWLRPEDLPIK.Y	(Ions score 57)
282 - 293	755.9006	1509.7867	1509.7827	3	0	R.DEWLRPEDLPIK.Y	(Ions score 28)
282 - 293	755.9006	1509.7867	1509.7827	3	0	R.DEWLRPEDLPIK.Y	(Ions score 45)
282 - 293	755.9006	1509.7867	1509.7827	3	0	R.DEWLRPEDLPIK.Y	(Ions score 44)
282 - 293	755.9007	1509.7868	1509.7827	3	0	R.DEWLRPEDLPIK.Y	(Ions score 39)
282 - 293	755.9007	1509.7868	1509.7827	3	0	R.DEWLRPEDLPIK.Y	(Ions score 49)
282 - 293	755.9007	1509.7868	1509.7827	3	0	R.DEWLRPEDLPIK.Y	(Ions score 23)
282 - 293	755.9007	1509.7868	1509.7827	3	0	R.DEWLRPEDLPIK.Y	(Ions score 59)
282 - 293	504.2696	1509.7868	1509.7827	3	0	R.DEWLRPEDLPIK.Y	(Ions score 21)
282 - 293	755.9007	1509.7868	1509.7827	3	0	R.DEWLRPEDLPIK.Y	(Ions score 48)
282 - 293	755.9007	1509.7869	1509.7827	3	0	R.DEWLRPEDLPIK.Y	(Ions score 57)
282 - 293	755.9008	1509.7870	1509.7827	3	0	R.DEWLRPEDLPIK.Y	(Ions score 50)
282 - 293	755.9008	1509.7870	1509.7827	3	0	R.DEWLRPEDLPIK.Y	(Ions score 24)
282 - 293	755.9008	1509.7870	1509.7827	3	0	R.DEWLRPEDLPIK.Y	(Ions score 54)
282 - 293	755.9008	1509.7870	1509.7827	3	0	R.DEWLRPEDLPIK.Y	(Ions score 23)
282 - 293	755.9008	1509.7871	1509.7827	3	0	R.DEWLRPEDLPIK.Y	(Ions score 31)
282 - 293	755.9008	1509.7871	1509.7827	3	0	R.DEWLRPEDLPIK.Y	(Ions score 41)
282 - 293	504.2697	1509.7872	1509.7827	3	0	R.DEWLRPEDLPIK.Y	(Ions score 23)
282 - 293	755.9009	1509.7872	1509.7827	3	0	R.DEWLRPEDLPIK.Y	(Ions score 41)
282 - 293	755.9009	1509.7872	1509.7827	3	0	R.DEWLRPEDLPIK.Y	(Ions score 41)
282 - 293	755.9009	1509.7873	1509.7827	3	0	R.DEWLRPEDLPIK.Y	(Ions score 42)
282 - 293	755.9010	1509.7874	1509.7827	3	0	R.DEWLRPEDLPIK.Y	(Ions score 44)
282 - 293	755.9011	1509.7876	1509.7827	3	0	R.DEWLRPEDLPIK.Y	(Ions score 41)
282 - 293	755.9011	1509.7876	1509.7827	3	0	R.DEWLRPEDLPIK.Y	(Ions score 37)
282 - 293	755.9011	1509.7876	1509.7827	3	0	R.DEWLRPEDLPIK.Y	(Ions score 40)
282 - 293	755.9011	1509.7877	1509.7827	3	0	R.DEWLRPEDLPIK.Y	(Ions score 44)
282 - 293	755.9011	1509.7877	1509.7827	3	0	R.DEWLRPEDLPIK.Y	(Ions score 41)
282 - 293	755.9012	1509.7878	1509.7827	3	0	R.DEWLRPEDLPIK.Y	(Ions score 41)
282 - 293	755.9012	1509.7878	1509.7827	3	0	R.DEWLRPEDLPIK.Y	(Ions score 49)
282 - 293	755.9012	1509.7878	1509.7827	3	0	R.DEWLRPEDLPIK.Y	(Ions score 50)
282 - 293	755.9012	1509.7879	1509.7827	3	0	R.DEWLRPEDLPIK.Y	(Ions score 34)
282 - 293	504.2699	1509.7879	1509.7827	3	0	R.DEWLRPEDLPIK.Y	(Ions score 23)
282 - 293	755.9013	1509.7880	1509.7827	3	0	R.DEWLRPEDLPIK.Y	(Ions score 37)
282 - 293	755.9013	1509.7880	1509.7827	4	0	R.DEWLRPEDLPIK.Y	(Ions score 30)
282 - 293	755.9013	1509.7881	1509.7827	4	0	R.DEWLRPEDLPIK.Y	(Ions score 23)
282 - 293	755.9014	1509.7883	1509.7827	4	0	R.DEWLRPEDLPIK.Y	(Ions score 44)
282 - 293	755.9015	1509.7884	1509.7827	4	0	R.DEWLRPEDLPIK.Y	(Ions score 39)
282 - 293	755.9015	1509.7884	1509.7827	4	0	R.DEWLRPEDLPIK.Y	(Ions score 42)
282 - 293	755.9015	1509.7884	1509.7827	4	0	R.DEWLRPEDLPIK.Y	(Ions score 36)
282 - 293	755.9015	1509.7884	1509.7827	4	0	R.DEWLRPEDLPIK.Y	(Ions score 37)
282 - 293	504.2701	1509.7885	1509.7827	4	0	R.DEWLRPEDLPIK.Y	(Ions score 21)
282 - 293	755.9016	1509.7885	1509.7827	4	0	R.DEWLRPEDLPIK.Y	(Ions score 37)
282 - 293	755.9016	1509.7886	1509.7827	4	0	R.DEWLRPEDLPIK.Y	(Ions score 23)
282 - 293	755.9016	1509.7886	1509.7827	4	0	R.DEWLRPEDLPIK.Y	(Ions score 41)
282 - 293	755.9016	1509.7886	1509.7827	4	0	R.DEWLRPEDLPIK.Y	(Ions score 43)
282 - 293	755.9016	1509.7886	1509.7827	4	0	R.DEWLRPEDLPIK.Y	(Ions score 39)
282 - 293	755.9016	1509.7887	1509.7827	4	0	R.DEWLRPEDLPIK.Y	(Ions score 45)
282 - 293	755.9017	1509.7888	1509.7827	4	0	R.DEWLRPEDLPIK.Y	(Ions score 30)
282 - 293	755.9017	1509.7888	1509.7827	4	0	R.DEWLRPEDLPIK.Y	(Ions score 31)
282 - 293	755.9017	1509.7888	1509.7827	4	0	R.DEWLRPEDLPIK.Y	(Ions score 39)
282 - 293	755.9017	1509.7889	1509.7827	4	0	R.DEWLRPEDLPIK.Y	(Ions score 42)
282 - 293	755.9017	1509.7889	1509.7827	4	0	R.DEWLRPEDLPIK.Y	(Ions score 38)
282 - 293	755.9017	1509.7889	1509.7827	4	0	R.DEWLRPEDLPIK.Y	(Ions score 32)
282 - 293	755.9017	1509.7889	1509.7827	4	0	R.DEWLRPEDLPIK.Y	(Ions score 51)

282 - 293	755.9017	1509.7889	1509.7827	4	0	R.DEWLRPEDLPIK.Y		(Ions score 31)
282 - 293	755.9017	1509.7889	1509.7827	4	0	R.DEWLRPEDLPIK.Y		(Ions score 56)
282 - 293	755.9018	1509.7890	1509.7827	4	0	R.DEWLRPEDLPIK.Y		(Ions score 24)
282 - 293	755.9018	1509.7890	1509.7827	4	0	R.DEWLRPEDLPIK.Y		(Ions score 31)
282 - 293	755.9018	1509.7891	1509.7827	4	0	R.DEWLRPEDLPIK.Y		(Ions score 41)
282 - 293	755.9020	1509.7894	1509.7827	4	0	R.DEWLRPEDLPIK.Y		(Ions score 30)
282 - 293	504.2704	1509.7894	1509.7827	4	0	R.DEWLRPEDLPIK.Y		(Ions score 24)
282 - 293	755.9021	1509.7896	1509.7827	5	0	R.DEWLRPEDLPIK.Y		(Ions score 34)
282 - 293	755.9021	1509.7896	1509.7827	5	0	R.DEWLRPEDLPIK.Y		(Ions score 49)
282 - 293	755.9022	1509.7898	1509.7827	5	0	R.DEWLRPEDLPIK.Y		(Ions score 34)
282 - 293	755.9022	1509.7898	1509.7827	5	0	R.DEWLRPEDLPIK.Y		(Ions score 25)
282 - 293	755.9022	1509.7898	1509.7827	5	0	R.DEWLRPEDLPIK.Y		(Ions score 22)
282 - 293	755.9022	1509.7899	1509.7827	5	0	R.DEWLRPEDLPIK.Y		(Ions score 50)
282 - 293	755.9022	1509.7899	1509.7827	5	0	R.DEWLRPEDLPIK.Y		(Ions score 27)
282 - 293	755.9023	1509.7900	1509.7827	5	0	R.DEWLRPEDLPIK.Y		(Ions score 55)
282 - 293	755.9023	1509.7900	1509.7827	5	0	R.DEWLRPEDLPIK.Y		(Ions score 41)
282 - 293	755.9023	1509.7901	1509.7827	5	0	R.DEWLRPEDLPIK.Y		(Ions score 41)
282 - 293	755.9023	1509.7901	1509.7827	5	0	R.DEWLRPEDLPIK.Y		(Ions score 53)
282 - 293	755.9024	1509.7902	1509.7827	5	0	R.DEWLRPEDLPIK.Y		(Ions score 36)
282 - 293	755.9024	1509.7902	1509.7827	5	0	R.DEWLRPEDLPIK.Y		(Ions score 39)
282 - 302	1283.6433	2565.2721	2565.2686	1	1	R.DEWLRPEDLPIKYAGLSTCFR.Q		(Ions score 30)
282 - 302	856.0985	2565.2737	2565.2686	2	1	R.DEWLRPEDLPIKYAGLSTCFR.Q		(Ions score 43)
282 - 302	856.0988	2565.2745	2565.2686	2	1	R.DEWLRPEDLPIKYAGLSTCFR.Q		(Ions score 40)
282 - 302	856.0993	2565.2761	2565.2686	3	1	R.DEWLRPEDLPIKYAGLSTCFR.Q		(Ions score 40)
282 - 302	870.1023	2607.2852	2607.2791	2	1	R.DEWLRPEDLPIKYAGLSTCFR.Q	Acetylation (K)	(Ions score 43)
294 - 302	537.7533	1073.4921	1073.4964	-4	0	K.YAGLSTCFR.Q		(Ions score 37)
294 - 302	537.7537	1073.4928	1073.4964	-3	0	K.YAGLSTCFR.Q		(Ions score 33)
294 - 302	537.7539	1073.4932	1073.4964	-3	0	K.YAGLSTCFR.Q		(Ions score 24)
294 - 302	537.7542	1073.4938	1073.4964	-2	0	K.YAGLSTCFR.Q		(Ions score 37)
294 - 302	537.7542	1073.4939	1073.4964	-2	0	K.YAGLSTCFR.Q		(Ions score 36)
294 - 302	537.7543	1073.4940	1073.4964	-2	0	K.YAGLSTCFR.Q		(Ions score 28)
294 - 302	537.7546	1073.4947	1073.4964	-2	0	K.YAGLSTCFR.Q		(Ions score 28)
294 - 302	537.7548	1073.4950	1073.4964	-1	0	K.YAGLSTCFR.Q		(Ions score 23)
294 - 302	537.7548	1073.4951	1073.4964	-1	0	K.YAGLSTCFR.Q		(Ions score 25)
294 - 302	537.7549	1073.4953	1073.4964	-1	0	K.YAGLSTCFR.Q		(Ions score 29)
294 - 302	537.7551	1073.4956	1073.4964	-1	0	K.YAGLSTCFR.Q		(Ions score 48)
294 - 302	537.7551	1073.4957	1073.4964	-1	0	K.YAGLSTCFR.Q		(Ions score 25)
294 - 302	537.7551	1073.4957	1073.4964	-1	0	K.YAGLSTCFR.Q		(Ions score 27)
294 - 302	537.7553	1073.4960	1073.4964	-0	0	K.YAGLSTCFR.Q		(Ions score 37)
294 - 302	537.7553	1073.4960	1073.4964	-0	0	K.YAGLSTCFR.Q		(Ions score 26)
294 - 302	537.7554	1073.4962	1073.4964	-0	0	K.YAGLSTCFR.Q		(Ions score 34)
294 - 302	537.7554	1073.4962	1073.4964	-0	0	K.YAGLSTCFR.Q		(Ions score 23)
294 - 302	537.7554	1073.4962	1073.4964	-0	0	K.YAGLSTCFR.Q		(Ions score 35)
294 - 302	537.7554	1073.4963	1073.4964	-0	0	K.YAGLSTCFR.Q		(Ions score 30)
294 - 302	537.7555	1073.4964	1073.4964	-0	0	K.YAGLSTCFR.Q		(Ions score 24)
294 - 302	537.7555	1073.4964	1073.4964	0	0	K.YAGLSTCFR.Q		(Ions score 24)
294 - 302	537.7555	1073.4964	1073.4964	0	0	K.YAGLSTCFR.Q		(Ions score 36)
294 - 302	537.7555	1073.4964	1073.4964	0	0	K.YAGLSTCFR.Q		(Ions score 36)
294 - 302	537.7555	1073.4965	1073.4964	0	0	K.YAGLSTCFR.Q		(Ions score 21)
294 - 302	537.7556	1073.4966	1073.4964	0	0	K.YAGLSTCFR.Q		(Ions score 38)
294 - 302	537.7556	1073.4966	1073.4964	0	0	K.YAGLSTCFR.Q		(Ions score 37)
294 - 302	537.7556	1073.4967	1073.4964	0	0	K.YAGLSTCFR.Q		(Ions score 26)
294 - 302	537.7556	1073.4967	1073.4964	0	0	K.YAGLSTCFR.Q		(Ions score 23)
294 - 302	537.7556	1073.4967	1073.4964	0	0	K.YAGLSTCFR.Q		(Ions score 27)
294 - 302	537.7556	1073.4967	1073.4964	0	0	K.YAGLSTCFR.Q		(Ions score 35)
294 - 302	537.7557	1073.4968	1073.4964	0	0	K.YAGLSTCFR.Q		(Ions score 35)
294 - 302	537.7557	1073.4968	1073.4964	0	0	K.YAGLSTCFR.Q		(Ions score 32)
294 - 302	537.7557	1073.4968	1073.4964	0	0	K.YAGLSTCFR.Q		(Ions score 33)

294 - 302	537.7557	1073.4968	1073.4964	0	0	K.YAGLSTCFR.Q	(Ions score 38)
294 - 302	537.7558	1073.4970	1073.4964	1	0	K.YAGLSTCFR.Q	(Ions score 50)
294 - 302	537.7558	1073.4971	1073.4964	1	0	K.YAGLSTCFR.Q	(Ions score 37)
294 - 302	537.7558	1073.4971	1073.4964	1	0	K.YAGLSTCFR.Q	(Ions score 38)
294 - 302	537.7558	1073.4971	1073.4964	1	0	K.YAGLSTCFR.Q	(Ions score 24)
294 - 302	537.7558	1073.4971	1073.4964	1	0	K.YAGLSTCFR.Q	(Ions score 22)
294 - 302	537.7559	1073.4972	1073.4964	1	0	K.YAGLSTCFR.Q	(Ions score 47)
294 - 302	537.7559	1073.4972	1073.4964	1	0	K.YAGLSTCFR.Q	(Ions score 24)
294 - 302	537.7559	1073.4972	1073.4964	1	0	K.YAGLSTCFR.Q	(Ions score 27)
294 - 302	537.7559	1073.4973	1073.4964	1	0	K.YAGLSTCFR.Q	(Ions score 26)
294 - 302	537.7559	1073.4973	1073.4964	1	0	K.YAGLSTCFR.Q	(Ions score 56)
294 - 302	537.7560	1073.4974	1073.4964	1	0	K.YAGLSTCFR.Q	(Ions score 25)
294 - 302	537.7560	1073.4974	1073.4964	1	0	K.YAGLSTCFR.Q	(Ions score 25)
294 - 302	537.7560	1073.4974	1073.4964	1	0	K.YAGLSTCFR.Q	(Ions score 36)
294 - 302	537.7560	1073.4974	1073.4964	1	0	K.YAGLSTCFR.Q	(Ions score 26)
294 - 302	537.7560	1073.4975	1073.4964	1	0	K.YAGLSTCFR.Q	(Ions score 28)
294 - 302	537.7560	1073.4975	1073.4964	1	0	K.YAGLSTCFR.Q	(Ions score 27)
294 - 302	537.7560	1073.4975	1073.4964	1	0	K.YAGLSTCFR.Q	(Ions score 36)
294 - 302	537.7561	1073.4976	1073.4964	1	0	K.YAGLSTCFR.Q	(Ions score 28)
294 - 302	537.7561	1073.4976	1073.4964	1	0	K.YAGLSTCFR.Q	(Ions score 24)
294 - 302	537.7561	1073.4976	1073.4964	1	0	K.YAGLSTCFR.Q	(Ions score 25)
294 - 302	537.7561	1073.4977	1073.4964	1	0	K.YAGLSTCFR.Q	(Ions score 40)
294 - 302	537.7561	1073.4977	1073.4964	1	0	K.YAGLSTCFR.Q	(Ions score 36)
294 - 302	537.7561	1073.4977	1073.4964	1	0	K.YAGLSTCFR.Q	(Ions score 30)
294 - 302	537.7561	1073.4977	1073.4964	1	0	K.YAGLSTCFR.Q	(Ions score 29)
294 - 302	537.7562	1073.4978	1073.4964	1	0	K.YAGLSTCFR.Q	(Ions score 26)
294 - 302	537.7562	1073.4978	1073.4964	1	0	K.YAGLSTCFR.Q	(Ions score 25)
294 - 302	537.7562	1073.4978	1073.4964	1	0	K.YAGLSTCFR.Q	(Ions score 29)
294 - 302	537.7562	1073.4978	1073.4964	1	0	K.YAGLSTCFR.Q	(Ions score 50)
294 - 302	537.7562	1073.4979	1073.4964	1	0	K.YAGLSTCFR.Q	(Ions score 22)
294 - 302	537.7562	1073.4979	1073.4964	1	0	K.YAGLSTCFR.Q	(Ions score 38)
294 - 302	537.7562	1073.4979	1073.4964	1	0	K.YAGLSTCFR.Q	(Ions score 26)
294 - 302	537.7563	1073.4979	1073.4964	1	0	K.YAGLSTCFR.Q	(Ions score 22)
294 - 302	537.7563	1073.4979	1073.4964	1	0	K.YAGLSTCFR.Q	(Ions score 27)
294 - 302	537.7563	1073.4980	1073.4964	1	0	K.YAGLSTCFR.Q	(Ions score 34)
294 - 302	537.7563	1073.4980	1073.4964	1	0	K.YAGLSTCFR.Q	(Ions score 41)
294 - 302	537.7563	1073.4980	1073.4964	1	0	K.YAGLSTCFR.Q	(Ions score 31)
294 - 302	537.7563	1073.4981	1073.4964	2	0	K.YAGLSTCFR.Q	(Ions score 27)
294 - 302	537.7563	1073.4981	1073.4964	2	0	K.YAGLSTCFR.Q	(Ions score 23)
294 - 302	537.7563	1073.4981	1073.4964	2	0	K.YAGLSTCFR.Q	(Ions score 34)
294 - 302	537.7563	1073.4981	1073.4964	2	0	K.YAGLSTCFR.Q	(Ions score 26)
294 - 302	537.7563	1073.4981	1073.4964	2	0	K.YAGLSTCFR.Q	(Ions score 34)
294 - 302	537.7563	1073.4981	1073.4964	2	0	K.YAGLSTCFR.Q	(Ions score 29)
294 - 302	537.7564	1073.4982	1073.4964	2	0	K.YAGLSTCFR.Q	(Ions score 25)
294 - 302	537.7564	1073.4982	1073.4964	2	0	K.YAGLSTCFR.Q	(Ions score 22)
294 - 302	537.7564	1073.4983	1073.4964	2	0	K.YAGLSTCFR.Q	(Ions score 25)
294 - 302	537.7564	1073.4983	1073.4964	2	0	K.YAGLSTCFR.Q	(Ions score 24)
294 - 302	537.7564	1073.4983	1073.4964	2	0	K.YAGLSTCFR.Q	(Ions score 25)
294 - 302	537.7564	1073.4983	1073.4964	2	0	K.YAGLSTCFR.Q	(Ions score 23)
294 - 302	537.7565	1073.4984	1073.4964	2	0	K.YAGLSTCFR.Q	(Ions score 34)
294 - 302	537.7565	1073.4984	1073.4964	2	0	K.YAGLSTCFR.Q	(Ions score 26)
294 - 302	537.7565	1073.4984	1073.4964	2	0	K.YAGLSTCFR.Q	(Ions score 38)
294 - 302	537.7565	1073.4985	1073.4964	2	0	K.YAGLSTCFR.Q	(Ions score 48)
294 - 302	537.7565	1073.4985	1073.4964	2	0	K.YAGLSTCFR.Q	(Ions score 28)
294 - 302	537.7565	1073.4985	1073.4964	2	0	K.YAGLSTCFR.Q	(Ions score 32)
294 - 302	537.7565	1073.4985	1073.4964	2	0	K.YAGLSTCFR.Q	(Ions score 29)
294 - 302	537.7565	1073.4985	1073.4964	2	0	K.YAGLSTCFR.Q	(Ions score 27)
294 - 302	537.7565	1073.4985	1073.4964	2	0	K.YAGLSTCFR.Q	(Ions score 26)

294 - 302	537.7565	1073.4985	1073.4964	2	0	K.YAGLSTCFR.Q	(Ions score 30)
294 - 302	537.7565	1073.4985	1073.4964	2	0	K.YAGLSTCFR.Q	(Ions score 31)
294 - 302	537.7566	1073.4986	1073.4964	2	0	K.YAGLSTCFR.Q	(Ions score 37)
294 - 302	537.7566	1073.4986	1073.4964	2	0	K.YAGLSTCFR.Q	(Ions score 25)
294 - 302	537.7566	1073.4986	1073.4964	2	0	K.YAGLSTCFR.Q	(Ions score 24)
294 - 302	537.7566	1073.4986	1073.4964	2	0	K.YAGLSTCFR.Q	(Ions score 24)
294 - 302	537.7566	1073.4987	1073.4964	2	0	K.YAGLSTCFR.Q	(Ions score 26)
294 - 302	537.7566	1073.4987	1073.4964	2	0	K.YAGLSTCFR.Q	(Ions score 25)
294 - 302	537.7566	1073.4987	1073.4964	2	0	K.YAGLSTCFR.Q	(Ions score 23)
294 - 302	537.7566	1073.4987	1073.4964	2	0	K.YAGLSTCFR.Q	(Ions score 22)
294 - 302	537.7567	1073.4988	1073.4964	2	0	K.YAGLSTCFR.Q	(Ions score 23)
294 - 302	537.7567	1073.4988	1073.4964	2	0	K.YAGLSTCFR.Q	(Ions score 34)
294 - 302	537.7567	1073.4989	1073.4964	2	0	K.YAGLSTCFR.Q	(Ions score 25)
294 - 302	537.7567	1073.4989	1073.4964	2	0	K.YAGLSTCFR.Q	(Ions score 30)
294 - 302	537.7567	1073.4989	1073.4964	2	0	K.YAGLSTCFR.Q	(Ions score 48)
294 - 302	537.7568	1073.4990	1073.4964	2	0	K.YAGLSTCFR.Q	(Ions score 27)
294 - 302	537.7568	1073.4990	1073.4964	2	0	K.YAGLSTCFR.Q	(Ions score 25)
294 - 302	537.7568	1073.4990	1073.4964	2	0	K.YAGLSTCFR.Q	(Ions score 42)
294 - 302	537.7568	1073.4990	1073.4964	2	0	K.YAGLSTCFR.Q	(Ions score 24)
294 - 302	537.7568	1073.4990	1073.4964	2	0	K.YAGLSTCFR.Q	(Ions score 35)
294 - 302	537.7568	1073.4991	1073.4964	3	0	K.YAGLSTCFR.Q	(Ions score 26)
294 - 302	537.7568	1073.4991	1073.4964	3	0	K.YAGLSTCFR.Q	(Ions score 27)
294 - 302	537.7569	1073.4992	1073.4964	3	0	K.YAGLSTCFR.Q	(Ions score 26)
294 - 302	537.7569	1073.4992	1073.4964	3	0	K.YAGLSTCFR.Q	(Ions score 26)
294 - 302	537.7569	1073.4992	1073.4964	3	0	K.YAGLSTCFR.Q	(Ions score 23)
294 - 302	537.7569	1073.4993	1073.4964	3	0	K.YAGLSTCFR.Q	(Ions score 22)
294 - 302	537.7569	1073.4993	1073.4964	3	0	K.YAGLSTCFR.Q	(Ions score 34)
294 - 302	537.7569	1073.4993	1073.4964	3	0	K.YAGLSTCFR.Q	(Ions score 24)
294 - 302	537.7569	1073.4993	1073.4964	3	0	K.YAGLSTCFR.Q	(Ions score 27)
294 - 302	537.7570	1073.4994	1073.4964	3	0	K.YAGLSTCFR.Q	(Ions score 37)
294 - 302	537.7570	1073.4994	1073.4964	3	0	K.YAGLSTCFR.Q	(Ions score 26)
294 - 302	537.7570	1073.4994	1073.4964	3	0	K.YAGLSTCFR.Q	(Ions score 37)
294 - 302	537.7570	1073.4994	1073.4964	3	0	K.YAGLSTCFR.Q	(Ions score 35)
294 - 302	537.7570	1073.4995	1073.4964	3	0	K.YAGLSTCFR.Q	(Ions score 25)
294 - 302	537.7570	1073.4995	1073.4964	3	0	K.YAGLSTCFR.Q	(Ions score 54)
294 - 302	537.7570	1073.4995	1073.4964	3	0	K.YAGLSTCFR.Q	(Ions score 27)
294 - 302	537.7570	1073.4995	1073.4964	3	0	K.YAGLSTCFR.Q	(Ions score 33)
294 - 302	537.7571	1073.4996	1073.4964	3	0	K.YAGLSTCFR.Q	(Ions score 35)
294 - 302	537.7571	1073.4996	1073.4964	3	0	K.YAGLSTCFR.Q	(Ions score 33)
294 - 302	537.7571	1073.4996	1073.4964	3	0	K.YAGLSTCFR.Q	(Ions score 47)
294 - 302	537.7571	1073.4996	1073.4964	3	0	K.YAGLSTCFR.Q	(Ions score 24)
294 - 302	537.7571	1073.4996	1073.4964	3	0	K.YAGLSTCFR.Q	(Ions score 28)
294 - 302	537.7571	1073.4997	1073.4964	3	0	K.YAGLSTCFR.Q	(Ions score 32)
294 - 302	537.7572	1073.4998	1073.4964	3	0	K.YAGLSTCFR.Q	(Ions score 32)
294 - 302	537.7572	1073.4998	1073.4964	3	0	K.YAGLSTCFR.Q	(Ions score 55)
294 - 302	537.7572	1073.4998	1073.4964	3	0	K.YAGLSTCFR.Q	(Ions score 34)
294 - 302	537.7572	1073.4998	1073.4964	3	0	K.YAGLSTCFR.Q	(Ions score 24)
294 - 302	537.7572	1073.4998	1073.4964	3	0	K.YAGLSTCFR.Q	(Ions score 38)
294 - 302	537.7572	1073.4999	1073.4964	3	0	K.YAGLSTCFR.Q	(Ions score 30)
294 - 302	537.7572	1073.4999	1073.4964	3	0	K.YAGLSTCFR.Q	(Ions score 54)
294 - 302	537.7573	1073.5000	1073.4964	3	0	K.YAGLSTCFR.Q	(Ions score 22)
294 - 302	537.7573	1073.5000	1073.4964	3	0	K.YAGLSTCFR.Q	(Ions score 25)
294 - 302	537.7573	1073.5000	1073.4964	3	0	K.YAGLSTCFR.Q	(Ions score 30)
294 - 302	537.7573	1073.5000	1073.4964	3	0	K.YAGLSTCFR.Q	(Ions score 23)
294 - 302	537.7573	1073.5000	1073.4964	3	0	K.YAGLSTCFR.Q	(Ions score 24)
294 - 302	537.7573	1073.5000	1073.4964	3	0	K.YAGLSTCFR.Q	(Ions score 27)
294 - 302	537.7573	1073.5000	1073.4964	3	0	K.YAGLSTCFR.Q	(Ions score 28)
294 - 302	537.7573	1073.5000	1073.4964	3	0	K.YAGLSTCFR.Q	(Ions score 25)

294 - 302	537.7574	1073.5001	1073.4964	3	0	K.YAGLSTCFR.Q	(Ions score 25)
294 - 302	537.7574	1073.5002	1073.4964	3	0	K.YAGLSTCFR.Q	(Ions score 23)
294 - 302	537.7574	1073.5002	1073.4964	4	0	K.YAGLSTCFR.Q	(Ions score 41)
294 - 302	537.7574	1073.5002	1073.4964	4	0	K.YAGLSTCFR.Q	(Ions score 28)
294 - 302	537.7574	1073.5002	1073.4964	4	0	K.YAGLSTCFR.Q	(Ions score 25)
294 - 302	537.7574	1073.5002	1073.4964	4	0	K.YAGLSTCFR.Q	(Ions score 24)
294 - 302	537.7574	1073.5003	1073.4964	4	0	K.YAGLSTCFR.Q	(Ions score 24)
294 - 302	537.7574	1073.5003	1073.4964	4	0	K.YAGLSTCFR.Q	(Ions score 28)
294 - 302	537.7574	1073.5003	1073.4964	4	0	K.YAGLSTCFR.Q	(Ions score 23)
294 - 302	537.7574	1073.5003	1073.4964	4	0	K.YAGLSTCFR.Q	(Ions score 24)
294 - 302	537.7574	1073.5003	1073.4964	4	0	K.YAGLSTCFR.Q	(Ions score 25)
294 - 302	537.7575	1073.5004	1073.4964	4	0	K.YAGLSTCFR.Q	(Ions score 54)
294 - 302	537.7575	1073.5005	1073.4964	4	0	K.YAGLSTCFR.Q	(Ions score 24)
294 - 302	537.7575	1073.5005	1073.4964	4	0	K.YAGLSTCFR.Q	(Ions score 47)
294 - 302	537.7575	1073.5005	1073.4964	4	0	K.YAGLSTCFR.Q	(Ions score 25)
294 - 302	537.7575	1073.5005	1073.4964	4	0	K.YAGLSTCFR.Q	(Ions score 24)
294 - 302	537.7576	1073.5006	1073.4964	4	0	K.YAGLSTCFR.Q	(Ions score 24)
294 - 302	537.7576	1073.5006	1073.4964	4	0	K.YAGLSTCFR.Q	(Ions score 25)
294 - 302	537.7576	1073.5006	1073.4964	4	0	K.YAGLSTCFR.Q	(Ions score 24)
294 - 302	537.7576	1073.5007	1073.4964	4	0	K.YAGLSTCFR.Q	(Ions score 46)
294 - 302	537.7576	1073.5007	1073.4964	4	0	K.YAGLSTCFR.Q	(Ions score 24)
294 - 302	537.7576	1073.5007	1073.4964	4	0	K.YAGLSTCFR.Q	(Ions score 25)
294 - 302	537.7577	1073.5008	1073.4964	4	0	K.YAGLSTCFR.Q	(Ions score 30)
294 - 302	537.7577	1073.5008	1073.4964	4	0	K.YAGLSTCFR.Q	(Ions score 41)
294 - 302	537.7577	1073.5008	1073.4964	4	0	K.YAGLSTCFR.Q	(Ions score 34)
294 - 302	537.7577	1073.5009	1073.4964	4	0	K.YAGLSTCFR.Q	(Ions score 27)
294 - 302	537.7577	1073.5009	1073.4964	4	0	K.YAGLSTCFR.Q	(Ions score 51)
294 - 302	537.7577	1073.5009	1073.4964	4	0	K.YAGLSTCFR.Q	(Ions score 27)
294 - 302	537.7577	1073.5009	1073.4964	4	0	K.YAGLSTCFR.Q	(Ions score 27)
294 - 302	537.7578	1073.5010	1073.4964	4	0	K.YAGLSTCFR.Q	(Ions score 57)
294 - 302	537.7578	1073.5010	1073.4964	4	0	K.YAGLSTCFR.Q	(Ions score 40)
294 - 302	537.7578	1073.5010	1073.4964	4	0	K.YAGLSTCFR.Q	(Ions score 34)
294 - 302	537.7578	1073.5010	1073.4964	4	0	K.YAGLSTCFR.Q	(Ions score 35)
294 - 302	537.7578	1073.5010	1073.4964	4	0	K.YAGLSTCFR.Q	(Ions score 31)
294 - 302	537.7578	1073.5011	1073.4964	4	0	K.YAGLSTCFR.Q	(Ions score 34)
294 - 302	537.7578	1073.5011	1073.4964	4	0	K.YAGLSTCFR.Q	(Ions score 24)
294 - 302	537.7578	1073.5011	1073.4964	4	0	K.YAGLSTCFR.Q	(Ions score 28)
294 - 302	537.7579	1073.5012	1073.4964	4	0	K.YAGLSTCFR.Q	(Ions score 27)
294 - 302	537.7579	1073.5012	1073.4964	4	0	K.YAGLSTCFR.Q	(Ions score 32)
294 - 302	537.7579	1073.5013	1073.4964	5	0	K.YAGLSTCFR.Q	(Ions score 36)
294 - 302	537.7579	1073.5013	1073.4964	5	0	K.YAGLSTCFR.Q	(Ions score 25)
294 - 302	537.7579	1073.5013	1073.4964	5	0	K.YAGLSTCFR.Q	(Ions score 54)
294 - 302	537.7579	1073.5013	1073.4964	5	0	K.YAGLSTCFR.Q	(Ions score 30)
294 - 302	537.7580	1073.5014	1073.4964	5	0	K.YAGLSTCFR.Q	(Ions score 52)
294 - 302	537.7581	1073.5016	1073.4964	5	0	K.YAGLSTCFR.Q	(Ions score 24)
294 - 302	537.7581	1073.5016	1073.4964	5	0	K.YAGLSTCFR.Q	(Ions score 24)
303 - 310	435.2127	868.4109	868.4151	-5	0	R.QEVGSHGR.D	(Ions score 42)
303 - 310	435.2129	868.4112	868.4151	-5	0	R.QEVGSHGR.D	(Ions score 28)
303 - 310	435.2143	868.4141	868.4151	-1	0	R.QEVGSHGR.D	(Ions score 21)
303 - 310	435.2145	868.4144	868.4151	-1	0	R.QEVGSHGR.D	(Ions score 22)
303 - 310	435.2148	868.4151	868.4151	-0	0	R.QEVGSHGR.D	(Ions score 44)
303 - 310	435.2149	868.4153	868.4151	0	0	R.QEVGSHGR.D	(Ions score 38)
303 - 310	435.2149	868.4153	868.4151	0	0	R.QEVGSHGR.D	(Ions score 41)
303 - 310	435.2150	868.4155	868.4151	0	0	R.QEVGSHGR.D	(Ions score 37)
303 - 310	435.2151	868.4156	868.4151	1	0	R.QEVGSHGR.D	(Ions score 48)
303 - 310	435.2151	868.4156	868.4151	1	0	R.QEVGSHGR.D	(Ions score 44)
303 - 310	435.2151	868.4157	868.4151	1	0	R.QEVGSHGR.D	(Ions score 37)
303 - 310	435.2152	868.4158	868.4151	1	0	R.QEVGSHGR.D	(Ions score 28)

303 - 310	435.2152	868.4158	868.4151	1	0	R.QEVGSHGR.D		(Ions score 22)
303 - 310	435.2152	868.4159	868.4151	1	0	R.QEVGSHGR.D		(Ions score 22)
303 - 310	435.2152	868.4159	868.4151	1	0	R.QEVGSHGR.D		(Ions score 47)
303 - 310	435.2152	868.4159	868.4151	1	0	R.QEVGSHGR.D		(Ions score 40)
303 - 310	435.2152	868.4159	868.4151	1	0	R.QEVGSHGR.D		(Ions score 44)
303 - 310	435.2152	868.4159	868.4151	1	0	R.QEVGSHGR.D		(Ions score 37)
303 - 310	435.2152	868.4159	868.4151	1	0	R.QEVGSHGR.D		(Ions score 42)
303 - 310	435.2152	868.4159	868.4151	1	0	R.QEVGSHGR.D		(Ions score 48)
303 - 310	435.2152	868.4159	868.4151	1	0	R.QEVGSHGR.D		(Ions score 39)
303 - 310	435.2152	868.4159	868.4151	1	0	R.QEVGSHGR.D		(Ions score 39)
303 - 310	435.2153	868.4160	868.4151	1	0	R.QEVGSHGR.D		(Ions score 47)
303 - 310	435.2153	868.4160	868.4151	1	0	R.QEVGSHGR.D		(Ions score 40)
303 - 310	435.2153	868.4160	868.4151	1	0	R.QEVGSHGR.D		(Ions score 37)
303 - 310	435.2154	868.4162	868.4151	1	0	R.QEVGSHGR.D		(Ions score 27)
303 - 310	435.2154	868.4162	868.4151	1	0	R.QEVGSHGR.D		(Ions score 40)
303 - 310	435.2154	868.4162	868.4151	1	0	R.QEVGSHGR.D		(Ions score 39)
303 - 310	435.2154	868.4163	868.4151	1	0	R.QEVGSHGR.D		(Ions score 37)
303 - 310	435.2154	868.4163	868.4151	1	0	R.QEVGSHGR.D		(Ions score 31)
303 - 310	435.2155	868.4164	868.4151	1	0	R.QEVGSHGR.D		(Ions score 25)
303 - 310	435.2155	868.4164	868.4151	1	0	R.QEVGSHGR.D		(Ions score 40)
303 - 310	435.2155	868.4164	868.4151	1	0	R.QEVGSHGR.D		(Ions score 38)
303 - 310	435.2155	868.4165	868.4151	2	0	R.QEVGSHGR.D		(Ions score 31)
303 - 310	435.2157	868.4168	868.4151	2	0	R.QEVGSHGR.D		(Ions score 46)
303 - 310	435.2158	868.4170	868.4151	2	0	R.QEVGSHGR.D		(Ions score 26)
303 - 310	435.2163	868.4180	868.4151	3	0	R.QEVGSHGR.D		(Ions score 40)
303 - 310	435.2163	868.4181	868.4151	3	0	R.QEVGSHGR.D		(Ions score 24)
318 - 336	1166.5702	2331.1258	2331.1284	-1	1	R.VHQFEKIEQFVYSSPHDNK.S		(Ions score 88)
318 - 336	583.7900	2331.1307	2331.1284	1	1	R.VHQFEKIEQFVYSSPHDNK.S		(Ions score 40)
318 - 336	778.0511	2331.1316	2331.1284	1	1	R.VHQFEKIEQFVYSSPHDNK.S		(Ions score 50)
318 - 336	467.2337	2331.1322	2331.1284	2	1	R.VHQFEKIEQFVYSSPHDNK.S		(Ions score 31)
318 - 336	583.7904	2331.1325	2331.1284	2	1	R.VHQFEKIEQFVYSSPHDNK.S		(Ions score 44)
318 - 336	778.0515	2331.1327	2331.1284	2	1	R.VHQFEKIEQFVYSSPHDNK.S		(Ions score 81)
318 - 336	467.2338	2331.1328	2331.1284	2	1	R.VHQFEKIEQFVYSSPHDNK.S		(Ions score 45)
318 - 336	1187.5806	2373.1467	2373.1389	3	1	R.VHQFEKIEQFVYSSPHDNK.S	Acetylation (K)	(Ions score 76)
318 - 336	594.2942	2373.1479	2373.1389	4	1	R.VHQFEKIEQFVYSSPHDNK.S	Acetylation (K)	(Ions score 69)
318 - 336	594.2944	2373.1483	2373.1389	4	1	R.VHQFEKIEQFVYSSPHDNK.S	Acetylation (K)	(Ions score 71)
318 - 336	1187.5815	2373.1485	2373.1389	4	1	R.VHQFEKIEQFVYSSPHDNK.S	Acetylation (K)	(Ions score 37)
324 - 336	521.9177	1562.7312	1562.7365	-3	0	K.IEQFVYSSPHDNK.S		(Ions score 42)
324 - 336	521.9182	1562.7329	1562.7365	-2	0	K.IEQFVYSSPHDNK.S		(Ions score 38)
324 - 336	521.9184	1562.7334	1562.7365	-2	0	K.IEQFVYSSPHDNK.S		(Ions score 25)
324 - 336	782.3741	1562.7336	1562.7365	-2	0	K.IEQFVYSSPHDNK.S		(Ions score 44)
324 - 336	782.3741	1562.7337	1562.7365	-2	0	K.IEQFVYSSPHDNK.S		(Ions score 52)
324 - 336	521.9186	1562.7338	1562.7365	-2	0	K.IEQFVYSSPHDNK.S		(Ions score 24)
324 - 336	521.9186	1562.7341	1562.7365	-2	0	K.IEQFVYSSPHDNK.S		(Ions score 39)
324 - 336	521.9188	1562.7345	1562.7365	-1	0	K.IEQFVYSSPHDNK.S		(Ions score 45)
324 - 336	521.9190	1562.7352	1562.7365	-1	0	K.IEQFVYSSPHDNK.S		(Ions score 33)
324 - 336	521.9192	1562.7358	1562.7365	-0	0	K.IEQFVYSSPHDNK.S		(Ions score 45)
324 - 336	521.9192	1562.7358	1562.7365	-0	0	K.IEQFVYSSPHDNK.S		(Ions score 32)
324 - 336	521.9192	1562.7359	1562.7365	-0	0	K.IEQFVYSSPHDNK.S		(Ions score 39)
324 - 336	782.3753	1562.7361	1562.7365	-0	0	K.IEQFVYSSPHDNK.S		(Ions score 66)
324 - 336	521.9194	1562.7363	1562.7365	-0	0	K.IEQFVYSSPHDNK.S		(Ions score 50)
324 - 336	521.9194	1562.7364	1562.7365	-0	0	K.IEQFVYSSPHDNK.S		(Ions score 45)
324 - 336	521.9195	1562.7367	1562.7365	0	0	K.IEQFVYSSPHDNK.S		(Ions score 41)
324 - 336	521.9195	1562.7367	1562.7365	0	0	K.IEQFVYSSPHDNK.S		(Ions score 42)
324 - 336	521.9195	1562.7367	1562.7365	0	0	K.IEQFVYSSPHDNK.S		(Ions score 34)
324 - 336	521.9195	1562.7368	1562.7365	0	0	K.IEQFVYSSPHDNK.S		(Ions score 31)
324 - 336	521.9196	1562.7368	1562.7365	0	0	K.IEQFVYSSPHDNK.S		(Ions score 43)
324 - 336	521.9196	1562.7368	1562.7365	0	0	K.IEQFVYSSPHDNK.S		(Ions score 38)

324 - 336	521.9196	1562.7369	1562.7365	0	0	K.IEQFVYSSPHDNK.S	(Ions score 42)
324 - 336	521.9197	1562.7373	1562.7365	0	0	K.IEQFVYSSPHDNK.S	(Ions score 37)
324 - 336	521.9197	1562.7373	1562.7365	0	0	K.IEQFVYSSPHDNK.S	(Ions score 24)
324 - 336	521.9197	1562.7374	1562.7365	1	0	K.IEQFVYSSPHDNK.S	(Ions score 31)
324 - 336	521.9197	1562.7374	1562.7365	1	0	K.IEQFVYSSPHDNK.S	(Ions score 40)
324 - 336	521.9198	1562.7375	1562.7365	1	0	K.IEQFVYSSPHDNK.S	(Ions score 38)
324 - 336	521.9198	1562.7375	1562.7365	1	0	K.IEQFVYSSPHDNK.S	(Ions score 43)
324 - 336	521.9198	1562.7375	1562.7365	1	0	K.IEQFVYSSPHDNK.S	(Ions score 42)
324 - 336	521.9198	1562.7376	1562.7365	1	0	K.IEQFVYSSPHDNK.S	(Ions score 39)
324 - 336	521.9198	1562.7376	1562.7365	1	0	K.IEQFVYSSPHDNK.S	(Ions score 41)
324 - 336	521.9199	1562.7377	1562.7365	1	0	K.IEQFVYSSPHDNK.S	(Ions score 39)
324 - 336	521.9199	1562.7378	1562.7365	1	0	K.IEQFVYSSPHDNK.S	(Ions score 28)
324 - 336	521.9199	1562.7378	1562.7365	1	0	K.IEQFVYSSPHDNK.S	(Ions score 41)
324 - 336	521.9199	1562.7378	1562.7365	1	0	K.IEQFVYSSPHDNK.S	(Ions score 30)
324 - 336	782.3762	1562.7379	1562.7365	1	0	K.IEQFVYSSPHDNK.S	(Ions score 60)
324 - 336	521.9199	1562.7379	1562.7365	1	0	K.IEQFVYSSPHDNK.S	(Ions score 46)
324 - 336	521.9199	1562.7380	1562.7365	1	0	K.IEQFVYSSPHDNK.S	(Ions score 38)
324 - 336	521.9200	1562.7381	1562.7365	1	0	K.IEQFVYSSPHDNK.S	(Ions score 42)
324 - 336	521.9200	1562.7381	1562.7365	1	0	K.IEQFVYSSPHDNK.S	(Ions score 37)
324 - 336	521.9200	1562.7381	1562.7365	1	0	K.IEQFVYSSPHDNK.S	(Ions score 22)
324 - 336	782.3764	1562.7382	1562.7365	1	0	K.IEQFVYSSPHDNK.S	(Ions score 70)
324 - 336	521.9200	1562.7383	1562.7365	1	0	K.IEQFVYSSPHDNK.S	(Ions score 33)
324 - 336	521.9201	1562.7384	1562.7365	1	0	K.IEQFVYSSPHDNK.S	(Ions score 36)
324 - 336	521.9201	1562.7384	1562.7365	1	0	K.IEQFVYSSPHDNK.S	(Ions score 37)
324 - 336	782.3765	1562.7384	1562.7365	1	0	K.IEQFVYSSPHDNK.S	(Ions score 60)
324 - 336	521.9201	1562.7384	1562.7365	1	0	K.IEQFVYSSPHDNK.S	(Ions score 37)
324 - 336	521.9201	1562.7385	1562.7365	1	0	K.IEQFVYSSPHDNK.S	(Ions score 43)
324 - 336	521.9201	1562.7385	1562.7365	1	0	K.IEQFVYSSPHDNK.S	(Ions score 34)
324 - 336	521.9201	1562.7385	1562.7365	1	0	K.IEQFVYSSPHDNK.S	(Ions score 39)
324 - 336	521.9201	1562.7386	1562.7365	1	0	K.IEQFVYSSPHDNK.S	(Ions score 44)
324 - 336	521.9201	1562.7386	1562.7365	1	0	K.IEQFVYSSPHDNK.S	(Ions score 37)
324 - 336	521.9202	1562.7388	1562.7365	1	0	K.IEQFVYSSPHDNK.S	(Ions score 46)
324 - 336	521.9202	1562.7389	1562.7365	1	0	K.IEQFVYSSPHDNK.S	(Ions score 39)
324 - 336	521.9202	1562.7389	1562.7365	1	0	K.IEQFVYSSPHDNK.S	(Ions score 42)
324 - 336	521.9202	1562.7389	1562.7365	2	0	K.IEQFVYSSPHDNK.S	(Ions score 41)
324 - 336	521.9202	1562.7389	1562.7365	2	0	K.IEQFVYSSPHDNK.S	(Ions score 29)
324 - 336	521.9202	1562.7389	1562.7365	2	0	K.IEQFVYSSPHDNK.S	(Ions score 36)
324 - 336	521.9202	1562.7389	1562.7365	2	0	K.IEQFVYSSPHDNK.S	(Ions score 41)
324 - 336	521.9203	1562.7389	1562.7365	2	0	K.IEQFVYSSPHDNK.S	(Ions score 41)
324 - 336	782.3768	1562.7390	1562.7365	2	0	K.IEQFVYSSPHDNK.S	(Ions score 35)
324 - 336	521.9203	1562.7390	1562.7365	2	0	K.IEQFVYSSPHDNK.S	(Ions score 42)
324 - 336	521.9203	1562.7391	1562.7365	2	0	K.IEQFVYSSPHDNK.S	(Ions score 41)
324 - 336	521.9203	1562.7391	1562.7365	2	0	K.IEQFVYSSPHDNK.S	(Ions score 29)
324 - 336	521.9203	1562.7391	1562.7365	2	0	K.IEQFVYSSPHDNK.S	(Ions score 34)
324 - 336	521.9203	1562.7392	1562.7365	2	0	K.IEQFVYSSPHDNK.S	(Ions score 38)
324 - 336	521.9203	1562.7392	1562.7365	2	0	K.IEQFVYSSPHDNK.S	(Ions score 45)
324 - 336	521.9203	1562.7392	1562.7365	2	0	K.IEQFVYSSPHDNK.S	(Ions score 37)
324 - 336	521.9203	1562.7392	1562.7365	2	0	K.IEQFVYSSPHDNK.S	(Ions score 44)
324 - 336	521.9203	1562.7392	1562.7365	2	0	K.IEQFVYSSPHDNK.S	(Ions score 39)
324 - 336	521.9204	1562.7392	1562.7365	2	0	K.IEQFVYSSPHDNK.S	(Ions score 40)
324 - 336	521.9204	1562.7393	1562.7365	2	0	K.IEQFVYSSPHDNK.S	(Ions score 38)
324 - 336	521.9204	1562.7393	1562.7365	2	0	K.IEQFVYSSPHDNK.S	(Ions score 42)
324 - 336	521.9204	1562.7393	1562.7365	2	0	K.IEQFVYSSPHDNK.S	(Ions score 41)
324 - 336	521.9204	1562.7393	1562.7365	2	0	K.IEQFVYSSPHDNK.S	(Ions score 29)
324 - 336	521.9204	1562.7394	1562.7365	2	0	K.IEQFVYSSPHDNK.S	(Ions score 37)
324 - 336	521.9204	1562.7394	1562.7365	2	0	K.IEQFVYSSPHDNK.S	(Ions score 45)
324 - 336	782.3770	1562.7395	1562.7365	2	0	K.IEQFVYSSPHDNK.S	(Ions score 55)
324 - 336	521.9204	1562.7395	1562.7365	2	0	K.IEQFVYSSPHDNK.S	(Ions score 48)

324 - 336	782.3770	1562.7395	1562.7365	2	0	K.IEQFVYSSPHDNK.S	(Ions score 64)
324 - 336	521.9205	1562.7395	1562.7365	2	0	K.IEQFVYSSPHDNK.S	(Ions score 40)
324 - 336	521.9205	1562.7396	1562.7365	2	0	K.IEQFVYSSPHDNK.S	(Ions score 36)
324 - 336	521.9205	1562.7396	1562.7365	2	0	K.IEQFVYSSPHDNK.S	(Ions score 38)
324 - 336	521.9205	1562.7396	1562.7365	2	0	K.IEQFVYSSPHDNK.S	(Ions score 47)
324 - 336	521.9205	1562.7397	1562.7365	2	0	K.IEQFVYSSPHDNK.S	(Ions score 34)
324 - 336	521.9205	1562.7398	1562.7365	2	0	K.IEQFVYSSPHDNK.S	(Ions score 34)
324 - 336	521.9205	1562.7398	1562.7365	2	0	K.IEQFVYSSPHDNK.S	(Ions score 41)
324 - 336	521.9205	1562.7398	1562.7365	2	0	K.IEQFVYSSPHDNK.S	(Ions score 37)
324 - 336	521.9205	1562.7398	1562.7365	2	0	K.IEQFVYSSPHDNK.S	(Ions score 47)
324 - 336	521.9205	1562.7398	1562.7365	2	0	K.IEQFVYSSPHDNK.S	(Ions score 21)
324 - 336	521.9206	1562.7398	1562.7365	2	0	K.IEQFVYSSPHDNK.S	(Ions score 42)
324 - 336	521.9206	1562.7398	1562.7365	2	0	K.IEQFVYSSPHDNK.S	(Ions score 34)
324 - 336	521.9206	1562.7398	1562.7365	2	0	K.IEQFVYSSPHDNK.S	(Ions score 35)
324 - 336	521.9206	1562.7399	1562.7365	2	0	K.IEQFVYSSPHDNK.S	(Ions score 38)
324 - 336	782.3772	1562.7399	1562.7365	2	0	K.IEQFVYSSPHDNK.S	(Ions score 55)
324 - 336	521.9206	1562.7399	1562.7365	2	0	K.IEQFVYSSPHDNK.S	(Ions score 38)
324 - 336	521.9206	1562.7399	1562.7365	2	0	K.IEQFVYSSPHDNK.S	(Ions score 45)
324 - 336	782.3772	1562.7399	1562.7365	2	0	K.IEQFVYSSPHDNK.S	(Ions score 68)
324 - 336	521.9206	1562.7399	1562.7365	2	0	K.IEQFVYSSPHDNK.S	(Ions score 33)
324 - 336	521.9206	1562.7399	1562.7365	2	0	K.IEQFVYSSPHDNK.S	(Ions score 43)
324 - 336	521.9206	1562.7400	1562.7365	2	0	K.IEQFVYSSPHDNK.S	(Ions score 32)
324 - 336	521.9206	1562.7400	1562.7365	2	0	K.IEQFVYSSPHDNK.S	(Ions score 40)
324 - 336	521.9206	1562.7401	1562.7365	2	0	K.IEQFVYSSPHDNK.S	(Ions score 40)
324 - 336	521.9207	1562.7401	1562.7365	2	0	K.IEQFVYSSPHDNK.S	(Ions score 41)
324 - 336	521.9207	1562.7402	1562.7365	2	0	K.IEQFVYSSPHDNK.S	(Ions score 32)
324 - 336	521.9207	1562.7402	1562.7365	2	0	K.IEQFVYSSPHDNK.S	(Ions score 22)
324 - 336	521.9207	1562.7403	1562.7365	2	0	K.IEQFVYSSPHDNK.S	(Ions score 45)
324 - 336	521.9207	1562.7403	1562.7365	2	0	K.IEQFVYSSPHDNK.S	(Ions score 36)
324 - 336	782.3774	1562.7403	1562.7365	2	0	K.IEQFVYSSPHDNK.S	(Ions score 74)
324 - 336	521.9207	1562.7403	1562.7365	2	0	K.IEQFVYSSPHDNK.S	(Ions score 22)
324 - 336	521.9207	1562.7403	1562.7365	2	0	K.IEQFVYSSPHDNK.S	(Ions score 47)
324 - 336	521.9207	1562.7403	1562.7365	2	0	K.IEQFVYSSPHDNK.S	(Ions score 35)
324 - 336	521.9207	1562.7403	1562.7365	2	0	K.IEQFVYSSPHDNK.S	(Ions score 22)
324 - 336	521.9207	1562.7403	1562.7365	2	0	K.IEQFVYSSPHDNK.S	(Ions score 42)
324 - 336	521.9207	1562.7404	1562.7365	2	0	K.IEQFVYSSPHDNK.S	(Ions score 45)
324 - 336	521.9207	1562.7404	1562.7365	2	0	K.IEQFVYSSPHDNK.S	(Ions score 41)
324 - 336	782.3775	1562.7404	1562.7365	2	0	K.IEQFVYSSPHDNK.S	(Ions score 49)
324 - 336	521.9208	1562.7404	1562.7365	3	0	K.IEQFVYSSPHDNK.S	(Ions score 35)
324 - 336	521.9208	1562.7404	1562.7365	3	0	K.IEQFVYSSPHDNK.S	(Ions score 36)
324 - 336	521.9208	1562.7405	1562.7365	3	0	K.IEQFVYSSPHDNK.S	(Ions score 39)
324 - 336	521.9208	1562.7405	1562.7365	3	0	K.IEQFVYSSPHDNK.S	(Ions score 39)
324 - 336	521.9208	1562.7405	1562.7365	3	0	K.IEQFVYSSPHDNK.S	(Ions score 39)
324 - 336	521.9208	1562.7405	1562.7365	3	0	K.IEQFVYSSPHDNK.S	(Ions score 51)
324 - 336	521.9208	1562.7406	1562.7365	3	0	K.IEQFVYSSPHDNK.S	(Ions score 45)
324 - 336	521.9208	1562.7406	1562.7365	3	0	K.IEQFVYSSPHDNK.S	(Ions score 34)
324 - 336	521.9208	1562.7406	1562.7365	3	0	K.IEQFVYSSPHDNK.S	(Ions score 39)
324 - 336	521.9208	1562.7406	1562.7365	3	0	K.IEQFVYSSPHDNK.S	(Ions score 34)
324 - 336	521.9208	1562.7406	1562.7365	3	0	K.IEQFVYSSPHDNK.S	(Ions score 41)
324 - 336	521.9208	1562.7407	1562.7365	3	0	K.IEQFVYSSPHDNK.S	(Ions score 36)
324 - 336	521.9209	1562.7407	1562.7365	3	0	K.IEQFVYSSPHDNK.S	(Ions score 43)
324 - 336	521.9209	1562.7408	1562.7365	3	0	K.IEQFVYSSPHDNK.S	(Ions score 43)
324 - 336	521.9209	1562.7408	1562.7365	3	0	K.IEQFVYSSPHDNK.S	(Ions score 51)
324 - 336	521.9209	1562.7408	1562.7365	3	0	K.IEQFVYSSPHDNK.S	(Ions score 43)
324 - 336	782.3777	1562.7409	1562.7365	3	0	K.IEQFVYSSPHDNK.S	(Ions score 58)
324 - 336	521.9209	1562.7409	1562.7365	3	0	K.IEQFVYSSPHDNK.S	(Ions score 51)
324 - 336	521.9209	1562.7409	1562.7365	3	0	K.IEQFVYSSPHDNK.S	(Ions score 32)
324 - 336	521.9209	1562.7410	1562.7365	3	0	K.IEQFVYSSPHDNK.S	(Ions score 36)

324 - 336	521.9209	1562.7410	1562.7365	3	0	K.IEQFVYSSPHDNK.S	(Ions score 24)
324 - 336	782.3778	1562.7410	1562.7365	3	0	K.IEQFVYSSPHDNK.S	(Ions score 37)
324 - 336	521.9209	1562.7410	1562.7365	3	0	K.IEQFVYSSPHDNK.S	(Ions score 50)
324 - 336	521.9209	1562.7410	1562.7365	3	0	K.IEQFVYSSPHDNK.S	(Ions score 37)
324 - 336	521.9210	1562.7410	1562.7365	3	0	K.IEQFVYSSPHDNK.S	(Ions score 38)
324 - 336	521.9210	1562.7411	1562.7365	3	0	K.IEQFVYSSPHDNK.S	(Ions score 44)
324 - 336	521.9210	1562.7411	1562.7365	3	0	K.IEQFVYSSPHDNK.S	(Ions score 42)
324 - 336	521.9210	1562.7411	1562.7365	3	0	K.IEQFVYSSPHDNK.S	(Ions score 29)
324 - 336	521.9210	1562.7411	1562.7365	3	0	K.IEQFVYSSPHDNK.S	(Ions score 34)
324 - 336	521.9210	1562.7411	1562.7365	3	0	K.IEQFVYSSPHDNK.S	(Ions score 40)
324 - 336	521.9210	1562.7411	1562.7365	3	0	K.IEQFVYSSPHDNK.S	(Ions score 29)
324 - 336	521.9210	1562.7412	1562.7365	3	0	K.IEQFVYSSPHDNK.S	(Ions score 36)
324 - 336	521.9210	1562.7412	1562.7365	3	0	K.IEQFVYSSPHDNK.S	(Ions score 45)
324 - 336	521.9210	1562.7412	1562.7365	3	0	K.IEQFVYSSPHDNK.S	(Ions score 36)
324 - 336	782.3779	1562.7413	1562.7365	3	0	K.IEQFVYSSPHDNK.S	(Ions score 27)
324 - 336	521.9210	1562.7413	1562.7365	3	0	K.IEQFVYSSPHDNK.S	(Ions score 32)
324 - 336	521.9211	1562.7413	1562.7365	3	0	K.IEQFVYSSPHDNK.S	(Ions score 46)
324 - 336	521.9211	1562.7413	1562.7365	3	0	K.IEQFVYSSPHDNK.S	(Ions score 50)
324 - 336	521.9211	1562.7413	1562.7365	3	0	K.IEQFVYSSPHDNK.S	(Ions score 36)
324 - 336	521.9211	1562.7413	1562.7365	3	0	K.IEQFVYSSPHDNK.S	(Ions score 25)
324 - 336	521.9211	1562.7414	1562.7365	3	0	K.IEQFVYSSPHDNK.S	(Ions score 45)
324 - 336	521.9211	1562.7414	1562.7365	3	0	K.IEQFVYSSPHDNK.S	(Ions score 40)
324 - 336	782.3780	1562.7415	1562.7365	3	0	K.IEQFVYSSPHDNK.S	(Ions score 76)
324 - 336	782.3780	1562.7415	1562.7365	3	0	K.IEQFVYSSPHDNK.S	(Ions score 48)
324 - 336	521.9211	1562.7415	1562.7365	3	0	K.IEQFVYSSPHDNK.S	(Ions score 42)
324 - 336	521.9211	1562.7415	1562.7365	3	0	K.IEQFVYSSPHDNK.S	(Ions score 28)
324 - 336	521.9211	1562.7415	1562.7365	3	0	K.IEQFVYSSPHDNK.S	(Ions score 36)
324 - 336	521.9211	1562.7416	1562.7365	3	0	K.IEQFVYSSPHDNK.S	(Ions score 40)
324 - 336	521.9211	1562.7416	1562.7365	3	0	K.IEQFVYSSPHDNK.S	(Ions score 39)
324 - 336	521.9212	1562.7416	1562.7365	3	0	K.IEQFVYSSPHDNK.S	(Ions score 28)
324 - 336	521.9212	1562.7416	1562.7365	3	0	K.IEQFVYSSPHDNK.S	(Ions score 40)
324 - 336	521.9212	1562.7416	1562.7365	3	0	K.IEQFVYSSPHDNK.S	(Ions score 30)
324 - 336	521.9212	1562.7416	1562.7365	3	0	K.IEQFVYSSPHDNK.S	(Ions score 33)
324 - 336	782.3781	1562.7417	1562.7365	3	0	K.IEQFVYSSPHDNK.S	(Ions score 68)
324 - 336	521.9212	1562.7417	1562.7365	3	0	K.IEQFVYSSPHDNK.S	(Ions score 47)
324 - 336	521.9212	1562.7417	1562.7365	3	0	K.IEQFVYSSPHDNK.S	(Ions score 48)
324 - 336	521.9212	1562.7418	1562.7365	3	0	K.IEQFVYSSPHDNK.S	(Ions score 43)
324 - 336	782.3782	1562.7418	1562.7365	3	0	K.IEQFVYSSPHDNK.S	(Ions score 66)
324 - 336	521.9212	1562.7418	1562.7365	3	0	K.IEQFVYSSPHDNK.S	(Ions score 45)
324 - 336	521.9212	1562.7418	1562.7365	3	0	K.IEQFVYSSPHDNK.S	(Ions score 31)
324 - 336	521.9212	1562.7418	1562.7365	3	0	K.IEQFVYSSPHDNK.S	(Ions score 39)
324 - 336	521.9212	1562.7418	1562.7365	3	0	K.IEQFVYSSPHDNK.S	(Ions score 39)
324 - 336	521.9212	1562.7419	1562.7365	3	0	K.IEQFVYSSPHDNK.S	(Ions score 45)
324 - 336	782.3782	1562.7419	1562.7365	3	0	K.IEQFVYSSPHDNK.S	(Ions score 85)
324 - 336	521.9213	1562.7419	1562.7365	3	0	K.IEQFVYSSPHDNK.S	(Ions score 30)
324 - 336	521.9213	1562.7419	1562.7365	3	0	K.IEQFVYSSPHDNK.S	(Ions score 52)
324 - 336	521.9213	1562.7420	1562.7365	3	0	K.IEQFVYSSPHDNK.S	(Ions score 21)
324 - 336	521.9213	1562.7420	1562.7365	3	0	K.IEQFVYSSPHDNK.S	(Ions score 33)
324 - 336	521.9213	1562.7420	1562.7365	4	0	K.IEQFVYSSPHDNK.S	(Ions score 21)
324 - 336	521.9213	1562.7420	1562.7365	4	0	K.IEQFVYSSPHDNK.S	(Ions score 36)
324 - 336	521.9213	1562.7420	1562.7365	4	0	K.IEQFVYSSPHDNK.S	(Ions score 36)
324 - 336	521.9213	1562.7421	1562.7365	4	0	K.IEQFVYSSPHDNK.S	(Ions score 28)
324 - 336	521.9213	1562.7421	1562.7365	4	0	K.IEQFVYSSPHDNK.S	(Ions score 42)
324 - 336	521.9213	1562.7421	1562.7365	4	0	K.IEQFVYSSPHDNK.S	(Ions score 41)
324 - 336	521.9213	1562.7421	1562.7365	4	0	K.IEQFVYSSPHDNK.S	(Ions score 45)
324 - 336	521.9213	1562.7422	1562.7365	4	0	K.IEQFVYSSPHDNK.S	(Ions score 41)
324 - 336	521.9213	1562.7422	1562.7365	4	0	K.IEQFVYSSPHDNK.S	(Ions score 33)
324 - 336	521.9213	1562.7422	1562.7365	4	0	K.IEQFVYSSPHDNK.S	(Ions score 44)

324 - 336	521.9213	1562.7422	1562.7365	4	0	K.IEQFVYSSPHDNK.S	(Ions score 45)
324 - 336	782.3784	1562.7422	1562.7365	4	0	K.IEQFVYSSPHDNK.S	(Ions score 51)
324 - 336	521.9214	1562.7422	1562.7365	4	0	K.IEQFVYSSPHDNK.S	(Ions score 42)
324 - 336	521.9214	1562.7423	1562.7365	4	0	K.IEQFVYSSPHDNK.S	(Ions score 40)
324 - 336	521.9214	1562.7423	1562.7365	4	0	K.IEQFVYSSPHDNK.S	(Ions score 23)
324 - 336	521.9214	1562.7423	1562.7365	4	0	K.IEQFVYSSPHDNK.S	(Ions score 35)
324 - 336	521.9214	1562.7423	1562.7365	4	0	K.IEQFVYSSPHDNK.S	(Ions score 37)
324 - 336	521.9214	1562.7423	1562.7365	4	0	K.IEQFVYSSPHDNK.S	(Ions score 36)
324 - 336	521.9214	1562.7424	1562.7365	4	0	K.IEQFVYSSPHDNK.S	(Ions score 36)
324 - 336	782.3785	1562.7424	1562.7365	4	0	K.IEQFVYSSPHDNK.S	(Ions score 59)
324 - 336	521.9214	1562.7424	1562.7365	4	0	K.IEQFVYSSPHDNK.S	(Ions score 41)
324 - 336	521.9214	1562.7425	1562.7365	4	0	K.IEQFVYSSPHDNK.S	(Ions score 45)
324 - 336	521.9214	1562.7425	1562.7365	4	0	K.IEQFVYSSPHDNK.S	(Ions score 45)
324 - 336	521.9215	1562.7426	1562.7365	4	0	K.IEQFVYSSPHDNK.S	(Ions score 37)
324 - 336	521.9215	1562.7426	1562.7365	4	0	K.IEQFVYSSPHDNK.S	(Ions score 35)
324 - 336	782.3786	1562.7426	1562.7365	4	0	K.IEQFVYSSPHDNK.S	(Ions score 55)
324 - 336	521.9215	1562.7426	1562.7365	4	0	K.IEQFVYSSPHDNK.S	(Ions score 39)
324 - 336	521.9215	1562.7426	1562.7365	4	0	K.IEQFVYSSPHDNK.S	(Ions score 25)
324 - 336	521.9215	1562.7426	1562.7365	4	0	K.IEQFVYSSPHDNK.S	(Ions score 27)
324 - 336	521.9215	1562.7426	1562.7365	4	0	K.IEQFVYSSPHDNK.S	(Ions score 30)
324 - 336	521.9215	1562.7426	1562.7365	4	0	K.IEQFVYSSPHDNK.S	(Ions score 24)
324 - 336	521.9215	1562.7427	1562.7365	4	0	K.IEQFVYSSPHDNK.S	(Ions score 42)
324 - 336	521.9215	1562.7427	1562.7365	4	0	K.IEQFVYSSPHDNK.S	(Ions score 22)
324 - 336	521.9215	1562.7427	1562.7365	4	0	K.IEQFVYSSPHDNK.S	(Ions score 31)
324 - 336	521.9215	1562.7427	1562.7365	4	0	K.IEQFVYSSPHDNK.S	(Ions score 25)
324 - 336	521.9215	1562.7428	1562.7365	4	0	K.IEQFVYSSPHDNK.S	(Ions score 46)
324 - 336	521.9215	1562.7428	1562.7365	4	0	K.IEQFVYSSPHDNK.S	(Ions score 39)
324 - 336	782.3787	1562.7429	1562.7365	4	0	K.IEQFVYSSPHDNK.S	(Ions score 57)
324 - 336	521.9216	1562.7429	1562.7365	4	0	K.IEQFVYSSPHDNK.S	(Ions score 38)
324 - 336	521.9216	1562.7429	1562.7365	4	0	K.IEQFVYSSPHDNK.S	(Ions score 30)
324 - 336	521.9216	1562.7429	1562.7365	4	0	K.IEQFVYSSPHDNK.S	(Ions score 42)
324 - 336	521.9216	1562.7430	1562.7365	4	0	K.IEQFVYSSPHDNK.S	(Ions score 33)
324 - 336	521.9216	1562.7431	1562.7365	4	0	K.IEQFVYSSPHDNK.S	(Ions score 47)
324 - 336	521.9216	1562.7431	1562.7365	4	0	K.IEQFVYSSPHDNK.S	(Ions score 40)
324 - 336	521.9217	1562.7431	1562.7365	4	0	K.IEQFVYSSPHDNK.S	(Ions score 43)
324 - 336	521.9217	1562.7431	1562.7365	4	0	K.IEQFVYSSPHDNK.S	(Ions score 40)
324 - 336	521.9217	1562.7431	1562.7365	4	0	K.IEQFVYSSPHDNK.S	(Ions score 36)
324 - 336	521.9217	1562.7433	1562.7365	4	0	K.IEQFVYSSPHDNK.S	(Ions score 36)
324 - 336	521.9217	1562.7433	1562.7365	4	0	K.IEQFVYSSPHDNK.S	(Ions score 39)
324 - 336	521.9217	1562.7434	1562.7365	4	0	K.IEQFVYSSPHDNK.S	(Ions score 40)
324 - 336	521.9217	1562.7434	1562.7365	4	0	K.IEQFVYSSPHDNK.S	(Ions score 41)
324 - 336	521.9217	1562.7434	1562.7365	4	0	K.IEQFVYSSPHDNK.S	(Ions score 41)
324 - 336	521.9218	1562.7434	1562.7365	4	0	K.IEQFVYSSPHDNK.S	(Ions score 21)
324 - 336	521.9218	1562.7435	1562.7365	4	0	K.IEQFVYSSPHDNK.S	(Ions score 40)
324 - 336	521.9218	1562.7435	1562.7365	4	0	K.IEQFVYSSPHDNK.S	(Ions score 42)
324 - 336	782.3791	1562.7436	1562.7365	5	0	K.IEQFVYSSPHDNK.S	(Ions score 60)
324 - 336	521.9218	1562.7437	1562.7365	5	0	K.IEQFVYSSPHDNK.S	(Ions score 30)
324 - 336	521.9218	1562.7437	1562.7365	5	0	K.IEQFVYSSPHDNK.S	(Ions score 37)
324 - 336	521.9219	1562.7438	1562.7365	5	0	K.IEQFVYSSPHDNK.S	(Ions score 31)
324 - 336	521.9219	1562.7438	1562.7365	5	0	K.IEQFVYSSPHDNK.S	(Ions score 50)
324 - 336	521.9219	1562.7438	1562.7365	5	0	K.IEQFVYSSPHDNK.S	(Ions score 23)
324 - 336	521.9219	1562.7439	1562.7365	5	0	K.IEQFVYSSPHDNK.S	(Ions score 36)
324 - 336	782.3793	1562.7440	1562.7365	5	0	K.IEQFVYSSPHDNK.S	(Ions score 29)
324 - 336	521.9220	1562.7440	1562.7365	5	0	K.IEQFVYSSPHDNK.S	(Ions score 23)
324 - 336	521.9220	1562.7441	1562.7365	5	0	K.IEQFVYSSPHDNK.S	(Ions score 40)
324 - 336	521.9220	1562.7441	1562.7365	5	0	K.IEQFVYSSPHDNK.S	(Ions score 40)
324 - 336	521.9220	1562.7442	1562.7365	5	0	K.IEQFVYSSPHDNK.S	(Ions score 36)
324 - 336	521.9220	1562.7442	1562.7365	5	0	K.IEQFVYSSPHDNK.S	(Ions score 32)

324 - 336	782.3794	1562.7442	1562.7365	5	0	K.IEQFVYSSPHDNK.S	(Ions score 81)
337 - 375	741.6934	4444.1166	4444.1031	3	0	K.SWEMFEEMITTAEEFYQSLGIPYHIVNIVSGSLNHAASK.K	Oxidation (M) (Ions score 33)
337 - 375	1487.7072	4460.0997	4460.0981	0	0	K.SWEMFEEMITTAEEFYQSLGIPYHIVNIVSGSLNHAASK.K	2 Oxidation (M) (Ions score 29)
337 - 375	1487.7078	4460.1015	4460.0981	1	0	K.SWEMFEEMITTAEEFYQSLGIPYHIVNIVSGSLNHAASK.K	2 Oxidation (M) (Ions score 22)
337 - 375	1487.7085	4460.1036	4460.0981	1	0	K.SWEMFEEMITTAEEFYQSLGIPYHIVNIVSGSLNHAASK.K	2 Oxidation (M) (Ions score 25)
337 - 375	1487.7086	4460.1041	4460.0981	1	0	K.SWEMFEEMITTAEEFYQSLGIPYHIVNIVSGSLNHAASK.K	2 Oxidation (M) (Ions score 24)
337 - 375	1116.0334	4460.1046	4460.0981	1	0	K.SWEMFEEMITTAEEFYQSLGIPYHIVNIVSGSLNHAASK.K	2 Oxidation (M) (Ions score 23)
337 - 375	1116.0335	4460.1047	4460.0981	1	0	K.SWEMFEEMITTAEEFYQSLGIPYHIVNIVSGSLNHAASK.K	2 Oxidation (M) (Ions score 25)
337 - 375	1487.7094	4460.1063	4460.0981	2	0	K.SWEMFEEMITTAEEFYQSLGIPYHIVNIVSGSLNHAASK.K	2 Oxidation (M) (Ions score 25)
337 - 375	893.0288	4460.1075	4460.0981	2	0	K.SWEMFEEMITTAEEFYQSLGIPYHIVNIVSGSLNHAASK.K	2 Oxidation (M) (Ions score 23)
337 - 375	1116.0346	4460.1093	4460.0981	3	0	K.SWEMFEEMITTAEEFYQSLGIPYHIVNIVSGSLNHAASK.K	2 Oxidation (M) (Ions score 26)
337 - 375	1116.0348	4460.1102	4460.0981	3	0	K.SWEMFEEMITTAEEFYQSLGIPYHIVNIVSGSLNHAASK.K	2 Oxidation (M) (Ions score 23)
337 - 375	893.0304	4460.1157	4460.0981	4	0	K.SWEMFEEMITTAEEFYQSLGIPYHIVNIVSGSLNHAASK.K	2 Oxidation (M) (Ions score 28)
337 - 375	1116.0363	4460.1162	4460.0981	4	0	K.SWEMFEEMITTAEEFYQSLGIPYHIVNIVSGSLNHAASK.K	2 Oxidation (M) (Ions score 23)
337 - 375	893.0306	4460.1165	4460.0981	4	0	K.SWEMFEEMITTAEEFYQSLGIPYHIVNIVSGSLNHAASK.K	2 Oxidation (M) (Ions score 30)
337 - 375	1116.0365	4460.1168	4460.0981	4	0	K.SWEMFEEMITTAEEFYQSLGIPYHIVNIVSGSLNHAASK.K	2 Oxidation (M) (Ions score 28)
376 - 390	847.4364	1692.8583	1692.8624	-2	1	K.KLDLEAWFPGSGAFR.E	(Ions score 44)
376 - 390	424.2219	1692.8583	1692.8624	-2	1	K.KLDLEAWFPGSGAFR.E	(Ions score 50)
376 - 390	1693.8671	1692.8598	1692.8624	-2	1	K.KLDLEAWFPGSGAFR.E	(Ions score 84)
376 - 390	565.2939	1692.8600	1692.8624	-1	1	K.KLDLEAWFPGSGAFR.E	(Ions score 29)
376 - 390	424.2223	1692.8601	1692.8624	-1	1	K.KLDLEAWFPGSGAFR.E	(Ions score 41)
376 - 390	847.4374	1692.8602	1692.8624	-1	1	K.KLDLEAWFPGSGAFR.E	(Ions score 62)
376 - 390	847.4374	1692.8602	1692.8624	-1	1	K.KLDLEAWFPGSGAFR.E	(Ions score 62)
376 - 390	424.2225	1692.8607	1692.8624	-1	1	K.KLDLEAWFPGSGAFR.E	(Ions score 49)
376 - 390	424.2225	1692.8608	1692.8624	-1	1	K.KLDLEAWFPGSGAFR.E	(Ions score 52)
376 - 390	424.2225	1692.8609	1692.8624	-1	1	K.KLDLEAWFPGSGAFR.E	(Ions score 46)
376 - 390	847.4377	1692.8609	1692.8624	-1	1	K.KLDLEAWFPGSGAFR.E	(Ions score 61)
376 - 390	424.2226	1692.8611	1692.8624	-1	1	K.KLDLEAWFPGSGAFR.E	(Ions score 47)
376 - 390	424.2226	1692.8612	1692.8624	-1	1	K.KLDLEAWFPGSGAFR.E	(Ions score 42)
376 - 390	424.2226	1692.8614	1692.8624	-1	1	K.KLDLEAWFPGSGAFR.E	(Ions score 50)
376 - 390	424.2227	1692.8617	1692.8624	-0	1	K.KLDLEAWFPGSGAFR.E	(Ions score 47)
376 - 390	424.2227	1692.8619	1692.8624	-0	1	K.KLDLEAWFPGSGAFR.E	(Ions score 40)
376 - 390	424.2228	1692.8619	1692.8624	-0	1	K.KLDLEAWFPGSGAFR.E	(Ions score 39)
376 - 390	424.2228	1692.8619	1692.8624	-0	1	K.KLDLEAWFPGSGAFR.E	(Ions score 55)
376 - 390	424.2228	1692.8620	1692.8624	-0	1	K.KLDLEAWFPGSGAFR.E	(Ions score 58)
376 - 390	847.4383	1692.8621	1692.8624	-0	1	K.KLDLEAWFPGSGAFR.E	(Ions score 43)
376 - 390	1693.8695	1692.8622	1692.8624	-0	1	K.KLDLEAWFPGSGAFR.E	(Ions score 62)
376 - 390	847.4384	1692.8623	1692.8624	-0	1	K.KLDLEAWFPGSGAFR.E	(Ions score 75)
376 - 390	424.2229	1692.8624	1692.8624	0	1	K.KLDLEAWFPGSGAFR.E	(Ions score 54)
376 - 390	424.2229	1692.8624	1692.8624	0	1	K.KLDLEAWFPGSGAFR.E	(Ions score 29)
376 - 390	424.2229	1692.8626	1692.8624	0	1	K.KLDLEAWFPGSGAFR.E	(Ions score 51)
376 - 390	847.4386	1692.8627	1692.8624	0	1	K.KLDLEAWFPGSGAFR.E	(Ions score 52)
376 - 390	565.2948	1692.8627	1692.8624	0	1	K.KLDLEAWFPGSGAFR.E	(Ions score 31)
376 - 390	424.2230	1692.8628	1692.8624	0	1	K.KLDLEAWFPGSGAFR.E	(Ions score 45)
376 - 390	424.2230	1692.8628	1692.8624	0	1	K.KLDLEAWFPGSGAFR.E	(Ions score 47)
376 - 390	424.2230	1692.8629	1692.8624	0	1	K.KLDLEAWFPGSGAFR.E	(Ions score 51)
376 - 390	847.4387	1692.8629	1692.8624	0	1	K.KLDLEAWFPGSGAFR.E	(Ions score 62)
376 - 390	565.2949	1692.8630	1692.8624	0	1	K.KLDLEAWFPGSGAFR.E	(Ions score 26)
376 - 390	565.2949	1692.8630	1692.8624	0	1	K.KLDLEAWFPGSGAFR.E	(Ions score 24)
376 - 390	424.2230	1692.8631	1692.8624	0	1	K.KLDLEAWFPGSGAFR.E	(Ions score 51)
376 - 390	1693.8704	1692.8631	1692.8624	0	1	K.KLDLEAWFPGSGAFR.E	(Ions score 78)
376 - 390	847.4388	1692.8631	1692.8624	0	1	K.KLDLEAWFPGSGAFR.E	(Ions score 46)
376 - 390	424.2231	1692.8631	1692.8624	0	1	K.KLDLEAWFPGSGAFR.E	(Ions score 32)
376 - 390	847.4388	1692.8631	1692.8624	0	1	K.KLDLEAWFPGSGAFR.E	(Ions score 32)
376 - 390	424.2231	1692.8632	1692.8624	0	1	K.KLDLEAWFPGSGAFR.E	(Ions score 50)
376 - 390	565.2950	1692.8632	1692.8624	1	1	K.KLDLEAWFPGSGAFR.E	(Ions score 28)
376 - 390	847.4389	1692.8633	1692.8624	1	1	K.KLDLEAWFPGSGAFR.E	(Ions score 56)
376 - 390	847.4389	1692.8633	1692.8624	1	1	K.KLDLEAWFPGSGAFR.E	(Ions score 30)

376 - 390	424.2231	1692.8633	1692.8624	1	1	K.KLDLEAWFPGSGAFR.E	(Ions score 53)
376 - 390	565.2950	1692.8633	1692.8624	1	1	K.KLDLEAWFPGSGAFR.E	(Ions score 30)
376 - 390	565.2951	1692.8635	1692.8624	1	1	K.KLDLEAWFPGSGAFR.E	(Ions score 25)
376 - 390	565.2951	1692.8636	1692.8624	1	1	K.KLDLEAWFPGSGAFR.E	(Ions score 34)
376 - 390	565.2951	1692.8636	1692.8624	1	1	K.KLDLEAWFPGSGAFR.E	(Ions score 29)
376 - 390	565.2952	1692.8637	1692.8624	1	1	K.KLDLEAWFPGSGAFR.E	(Ions score 23)
376 - 390	424.2232	1692.8638	1692.8624	1	1	K.KLDLEAWFPGSGAFR.E	(Ions score 46)
376 - 390	847.4392	1692.8638	1692.8624	1	1	K.KLDLEAWFPGSGAFR.E	(Ions score 48)
376 - 390	565.2952	1692.8638	1692.8624	1	1	K.KLDLEAWFPGSGAFR.E	(Ions score 23)
376 - 390	565.2952	1692.8639	1692.8624	1	1	K.KLDLEAWFPGSGAFR.E	(Ions score 28)
376 - 390	565.2953	1692.8639	1692.8624	1	1	K.KLDLEAWFPGSGAFR.E	(Ions score 23)
376 - 390	1693.8712	1692.8639	1692.8624	1	1	K.KLDLEAWFPGSGAFR.E	(Ions score 93)
376 - 390	847.4393	1692.8640	1692.8624	1	1	K.KLDLEAWFPGSGAFR.E	(Ions score 58)
376 - 390	565.2953	1692.8641	1692.8624	1	1	K.KLDLEAWFPGSGAFR.E	(Ions score 26)
376 - 390	565.2953	1692.8641	1692.8624	1	1	K.KLDLEAWFPGSGAFR.E	(Ions score 23)
376 - 390	565.2953	1692.8642	1692.8624	1	1	K.KLDLEAWFPGSGAFR.E	(Ions score 25)
376 - 390	424.2233	1692.8642	1692.8624	1	1	K.KLDLEAWFPGSGAFR.E	(Ions score 47)
376 - 390	565.2953	1692.8642	1692.8624	1	1	K.KLDLEAWFPGSGAFR.E	(Ions score 28)
376 - 390	847.4394	1692.8642	1692.8624	1	1	K.KLDLEAWFPGSGAFR.E	(Ions score 71)
376 - 390	565.2954	1692.8643	1692.8624	1	1	K.KLDLEAWFPGSGAFR.E	(Ions score 27)
376 - 390	565.2954	1692.8643	1692.8624	1	1	K.KLDLEAWFPGSGAFR.E	(Ions score 27)
376 - 390	565.2954	1692.8643	1692.8624	1	1	K.KLDLEAWFPGSGAFR.E	(Ions score 31)
376 - 390	565.2954	1692.8644	1692.8624	1	1	K.KLDLEAWFPGSGAFR.E	(Ions score 28)
376 - 390	847.4395	1692.8644	1692.8624	1	1	K.KLDLEAWFPGSGAFR.E	(Ions score 61)
376 - 390	847.4395	1692.8644	1692.8624	1	1	K.KLDLEAWFPGSGAFR.E	(Ions score 53)
376 - 390	565.2954	1692.8645	1692.8624	1	1	K.KLDLEAWFPGSGAFR.E	(Ions score 26)
376 - 390	847.4395	1692.8645	1692.8624	1	1	K.KLDLEAWFPGSGAFR.E	(Ions score 65)
376 - 390	847.4395	1692.8645	1692.8624	1	1	K.KLDLEAWFPGSGAFR.E	(Ions score 54)
376 - 390	1693.8718	1692.8646	1692.8624	1	1	K.KLDLEAWFPGSGAFR.E	(Ions score 66)
376 - 390	565.2955	1692.8646	1692.8624	1	1	K.KLDLEAWFPGSGAFR.E	(Ions score 26)
376 - 390	565.2955	1692.8646	1692.8624	1	1	K.KLDLEAWFPGSGAFR.E	(Ions score 31)
376 - 390	847.4396	1692.8646	1692.8624	1	1	K.KLDLEAWFPGSGAFR.E	(Ions score 66)
376 - 390	847.4396	1692.8646	1692.8624	1	1	K.KLDLEAWFPGSGAFR.E	(Ions score 64)
376 - 390	847.4396	1692.8647	1692.8624	1	1	K.KLDLEAWFPGSGAFR.E	(Ions score 62)
376 - 390	565.2955	1692.8647	1692.8624	1	1	K.KLDLEAWFPGSGAFR.E	(Ions score 23)
376 - 390	847.4396	1692.8647	1692.8624	1	1	K.KLDLEAWFPGSGAFR.E	(Ions score 65)
376 - 390	847.4396	1692.8647	1692.8624	1	1	K.KLDLEAWFPGSGAFR.E	(Ions score 55)
376 - 390	847.4396	1692.8647	1692.8624	1	1	K.KLDLEAWFPGSGAFR.E	(Ions score 58)
376 - 390	847.4396	1692.8647	1692.8624	1	1	K.KLDLEAWFPGSGAFR.E	(Ions score 59)
376 - 390	565.2955	1692.8647	1692.8624	1	1	K.KLDLEAWFPGSGAFR.E	(Ions score 25)
376 - 390	565.2955	1692.8648	1692.8624	1	1	K.KLDLEAWFPGSGAFR.E	(Ions score 24)
376 - 390	847.4397	1692.8648	1692.8624	1	1	K.KLDLEAWFPGSGAFR.E	(Ions score 59)
376 - 390	847.4397	1692.8648	1692.8624	1	1	K.KLDLEAWFPGSGAFR.E	(Ions score 63)
376 - 390	565.2956	1692.8648	1692.8624	1	1	K.KLDLEAWFPGSGAFR.E	(Ions score 27)
376 - 390	847.4397	1692.8649	1692.8624	1	1	K.KLDLEAWFPGSGAFR.E	(Ions score 70)
376 - 390	847.4397	1692.8649	1692.8624	1	1	K.KLDLEAWFPGSGAFR.E	(Ions score 64)
376 - 390	847.4397	1692.8649	1692.8624	1	1	K.KLDLEAWFPGSGAFR.E	(Ions score 50)
376 - 390	847.4397	1692.8649	1692.8624	1	1	K.KLDLEAWFPGSGAFR.E	(Ions score 66)
376 - 390	847.4397	1692.8649	1692.8624	1	1	K.KLDLEAWFPGSGAFR.E	(Ions score 59)
376 - 390	847.4397	1692.8649	1692.8624	1	1	K.KLDLEAWFPGSGAFR.E	(Ions score 66)
376 - 390	847.4397	1692.8649	1692.8624	1	1	K.KLDLEAWFPGSGAFR.E	(Ions score 72)
376 - 390	565.2956	1692.8649	1692.8624	1	1	K.KLDLEAWFPGSGAFR.E	(Ions score 29)
376 - 390	565.2956	1692.8649	1692.8624	1	1	K.KLDLEAWFPGSGAFR.E	(Ions score 32)
376 - 390	847.4397	1692.8649	1692.8624	2	1	K.KLDLEAWFPGSGAFR.E	(Ions score 76)
376 - 390	847.4397	1692.8649	1692.8624	2	1	K.KLDLEAWFPGSGAFR.E	(Ions score 70)
376 - 390	847.4397	1692.8649	1692.8624	2	1	K.KLDLEAWFPGSGAFR.E	(Ions score 72)
376 - 390	847.4397	1692.8649	1692.8624	2	1	K.KLDLEAWFPGSGAFR.E	(Ions score 36)
376 - 390	847.4398	1692.8650	1692.8624	2	1	K.KLDLEAWFPGSGAFR.E	(Ions score 47)

376 - 390	847.4398	1692.8650	1692.8624	2	1	K.KLDLEAWFPGSGAFR.E	(Ions score 65)
376 - 390	847.4398	1692.8650	1692.8624	2	1	K.KLDLEAWFPGSGAFR.E	(Ions score 72)
376 - 390	847.4398	1692.8650	1692.8624	2	1	K.KLDLEAWFPGSGAFR.E	(Ions score 73)
376 - 390	424.2235	1692.8650	1692.8624	2	1	K.KLDLEAWFPGSGAFR.E	(Ions score 52)
376 - 390	847.4398	1692.8650	1692.8624	2	1	K.KLDLEAWFPGSGAFR.E	(Ions score 64)
376 - 390	565.2956	1692.8650	1692.8624	2	1	K.KLDLEAWFPGSGAFR.E	(Ions score 26)
376 - 390	847.4398	1692.8650	1692.8624	2	1	K.KLDLEAWFPGSGAFR.E	(Ions score 62)
376 - 390	565.2956	1692.8651	1692.8624	2	1	K.KLDLEAWFPGSGAFR.E	(Ions score 29)
376 - 390	847.4398	1692.8651	1692.8624	2	1	K.KLDLEAWFPGSGAFR.E	(Ions score 47)
376 - 390	565.2957	1692.8652	1692.8624	2	1	K.KLDLEAWFPGSGAFR.E	(Ions score 22)
376 - 390	565.2957	1692.8652	1692.8624	2	1	K.KLDLEAWFPGSGAFR.E	(Ions score 27)
376 - 390	847.4399	1692.8652	1692.8624	2	1	K.KLDLEAWFPGSGAFR.E	(Ions score 40)
376 - 390	565.2957	1692.8652	1692.8624	2	1	K.KLDLEAWFPGSGAFR.E	(Ions score 28)
376 - 390	565.2957	1692.8652	1692.8624	2	1	K.KLDLEAWFPGSGAFR.E	(Ions score 31)
376 - 390	847.4399	1692.8652	1692.8624	2	1	K.KLDLEAWFPGSGAFR.E	(Ions score 67)
376 - 390	847.4399	1692.8652	1692.8624	2	1	K.KLDLEAWFPGSGAFR.E	(Ions score 48)
376 - 390	565.2957	1692.8652	1692.8624	2	1	K.KLDLEAWFPGSGAFR.E	(Ions score 28)
376 - 390	847.4399	1692.8652	1692.8624	2	1	K.KLDLEAWFPGSGAFR.E	(Ions score 70)
376 - 390	565.2957	1692.8652	1692.8624	2	1	K.KLDLEAWFPGSGAFR.E	(Ions score 24)
376 - 390	847.4399	1692.8653	1692.8624	2	1	K.KLDLEAWFPGSGAFR.E	(Ions score 33)
376 - 390	847.4399	1692.8653	1692.8624	2	1	K.KLDLEAWFPGSGAFR.E	(Ions score 73)
376 - 390	565.2957	1692.8653	1692.8624	2	1	K.KLDLEAWFPGSGAFR.E	(Ions score 27)
376 - 390	847.4399	1692.8653	1692.8624	2	1	K.KLDLEAWFPGSGAFR.E	(Ions score 30)
376 - 390	565.2957	1692.8653	1692.8624	2	1	K.KLDLEAWFPGSGAFR.E	(Ions score 29)
376 - 390	847.4400	1692.8654	1692.8624	2	1	K.KLDLEAWFPGSGAFR.E	(Ions score 70)
376 - 390	847.4400	1692.8654	1692.8624	2	1	K.KLDLEAWFPGSGAFR.E	(Ions score 65)
376 - 390	847.4400	1692.8654	1692.8624	2	1	K.KLDLEAWFPGSGAFR.E	(Ions score 82)
376 - 390	424.2236	1692.8654	1692.8624	2	1	K.KLDLEAWFPGSGAFR.E	(Ions score 51)
376 - 390	565.2957	1692.8654	1692.8624	2	1	K.KLDLEAWFPGSGAFR.E	(Ions score 28)
376 - 390	847.4400	1692.8654	1692.8624	2	1	K.KLDLEAWFPGSGAFR.E	(Ions score 72)
376 - 390	847.4400	1692.8654	1692.8624	2	1	K.KLDLEAWFPGSGAFR.E	(Ions score 62)
376 - 390	565.2958	1692.8654	1692.8624	2	1	K.KLDLEAWFPGSGAFR.E	(Ions score 29)
376 - 390	565.2958	1692.8656	1692.8624	2	1	K.KLDLEAWFPGSGAFR.E	(Ions score 24)
376 - 390	565.2958	1692.8656	1692.8624	2	1	K.KLDLEAWFPGSGAFR.E	(Ions score 28)
376 - 390	847.4401	1692.8656	1692.8624	2	1	K.KLDLEAWFPGSGAFR.E	(Ions score 32)
376 - 390	565.2958	1692.8656	1692.8624	2	1	K.KLDLEAWFPGSGAFR.E	(Ions score 28)
376 - 390	565.2958	1692.8657	1692.8624	2	1	K.KLDLEAWFPGSGAFR.E	(Ions score 31)
376 - 390	847.4401	1692.8657	1692.8624	2	1	K.KLDLEAWFPGSGAFR.E	(Ions score 68)
376 - 390	847.4402	1692.8658	1692.8624	2	1	K.KLDLEAWFPGSGAFR.E	(Ions score 69)
376 - 390	565.2959	1692.8659	1692.8624	2	1	K.KLDLEAWFPGSGAFR.E	(Ions score 28)
376 - 390	565.2959	1692.8659	1692.8624	2	1	K.KLDLEAWFPGSGAFR.E	(Ions score 33)
376 - 390	847.4402	1692.8659	1692.8624	2	1	K.KLDLEAWFPGSGAFR.E	(Ions score 62)
376 - 390	847.4402	1692.8659	1692.8624	2	1	K.KLDLEAWFPGSGAFR.E	(Ions score 42)
376 - 390	565.2959	1692.8659	1692.8624	2	1	K.KLDLEAWFPGSGAFR.E	(Ions score 26)
376 - 390	565.2959	1692.8659	1692.8624	2	1	K.KLDLEAWFPGSGAFR.E	(Ions score 26)
376 - 390	847.4403	1692.8660	1692.8624	2	1	K.KLDLEAWFPGSGAFR.E	(Ions score 65)
376 - 390	847.4403	1692.8660	1692.8624	2	1	K.KLDLEAWFPGSGAFR.E	(Ions score 49)
376 - 390	565.2960	1692.8661	1692.8624	2	1	K.KLDLEAWFPGSGAFR.E	(Ions score 25)
376 - 390	565.2960	1692.8661	1692.8624	2	1	K.KLDLEAWFPGSGAFR.E	(Ions score 26)
376 - 390	565.2960	1692.8661	1692.8624	2	1	K.KLDLEAWFPGSGAFR.E	(Ions score 29)
376 - 390	565.2960	1692.8661	1692.8624	2	1	K.KLDLEAWFPGSGAFR.E	(Ions score 28)
376 - 390	565.2960	1692.8661	1692.8624	2	1	K.KLDLEAWFPGSGAFR.E	(Ions score 26)
376 - 390	424.2238	1692.8661	1692.8624	2	1	K.KLDLEAWFPGSGAFR.E	(Ions score 50)
376 - 390	847.4404	1692.8662	1692.8624	2	1	K.KLDLEAWFPGSGAFR.E	(Ions score 64)
376 - 390	565.2960	1692.8662	1692.8624	2	1	K.KLDLEAWFPGSGAFR.E	(Ions score 31)
376 - 390	565.2960	1692.8662	1692.8624	2	1	K.KLDLEAWFPGSGAFR.E	(Ions score 26)
376 - 390	565.2960	1692.8662	1692.8624	2	1	K.KLDLEAWFPGSGAFR.E	(Ions score 30)
376 - 390	565.2960	1692.8662	1692.8624	2	1	K.KLDLEAWFPGSGAFR.E	(Ions score 28)

376 - 390	565.2960	1692.8662	1692.8624	2	1	K.KLDLEAWFPGSGAFR.E	(Ions score 27)
376 - 390	565.2960	1692.8662	1692.8624	2	1	K.KLDLEAWFPGSGAFR.E	(Ions score 24)
376 - 390	847.4404	1692.8663	1692.8624	2	1	K.KLDLEAWFPGSGAFR.E	(Ions score 64)
376 - 390	565.2960	1692.8663	1692.8624	2	1	K.KLDLEAWFPGSGAFR.E	(Ions score 31)
376 - 390	565.2960	1692.8663	1692.8624	2	1	K.KLDLEAWFPGSGAFR.E	(Ions score 29)
376 - 390	565.2961	1692.8663	1692.8624	2	1	K.KLDLEAWFPGSGAFR.E	(Ions score 27)
376 - 390	565.2961	1692.8663	1692.8624	2	1	K.KLDLEAWFPGSGAFR.E	(Ions score 31)
376 - 390	565.2961	1692.8664	1692.8624	2	1	K.KLDLEAWFPGSGAFR.E	(Ions score 28)
376 - 390	565.2961	1692.8664	1692.8624	2	1	K.KLDLEAWFPGSGAFR.E	(Ions score 26)
376 - 390	565.2961	1692.8664	1692.8624	2	1	K.KLDLEAWFPGSGAFR.E	(Ions score 27)
376 - 390	565.2961	1692.8664	1692.8624	2	1	K.KLDLEAWFPGSGAFR.E	(Ions score 31)
376 - 390	565.2961	1692.8664	1692.8624	2	1	K.KLDLEAWFPGSGAFR.E	(Ions score 28)
376 - 390	565.2961	1692.8664	1692.8624	2	1	K.KLDLEAWFPGSGAFR.E	(Ions score 27)
376 - 390	565.2961	1692.8664	1692.8624	2	1	K.KLDLEAWFPGSGAFR.E	(Ions score 31)
376 - 390	565.2961	1692.8665	1692.8624	2	1	K.KLDLEAWFPGSGAFR.E	(Ions score 27)
376 - 390	565.2961	1692.8665	1692.8624	2	1	K.KLDLEAWFPGSGAFR.E	(Ions score 30)
376 - 390	847.4405	1692.8665	1692.8624	2	1	K.KLDLEAWFPGSGAFR.E	(Ions score 65)
376 - 390	847.4405	1692.8665	1692.8624	2	1	K.KLDLEAWFPGSGAFR.E	(Ions score 58)
376 - 390	424.2239	1692.8665	1692.8624	2	1	K.KLDLEAWFPGSGAFR.E	(Ions score 50)
376 - 390	847.4406	1692.8666	1692.8624	2	1	K.KLDLEAWFPGSGAFR.E	(Ions score 50)
376 - 390	565.2961	1692.8666	1692.8624	2	1	K.KLDLEAWFPGSGAFR.E	(Ions score 26)
376 - 390	565.2961	1692.8666	1692.8624	3	1	K.KLDLEAWFPGSGAFR.E	(Ions score 27)
376 - 390	847.4406	1692.8666	1692.8624	3	1	K.KLDLEAWFPGSGAFR.E	(Ions score 69)
376 - 390	565.2962	1692.8667	1692.8624	3	1	K.KLDLEAWFPGSGAFR.E	(Ions score 30)
376 - 390	565.2962	1692.8667	1692.8624	3	1	K.KLDLEAWFPGSGAFR.E	(Ions score 32)
376 - 390	565.2962	1692.8667	1692.8624	3	1	K.KLDLEAWFPGSGAFR.E	(Ions score 29)
376 - 390	565.2962	1692.8667	1692.8624	3	1	K.KLDLEAWFPGSGAFR.E	(Ions score 24)
376 - 390	847.4406	1692.8667	1692.8624	3	1	K.KLDLEAWFPGSGAFR.E	(Ions score 57)
376 - 390	565.2962	1692.8668	1692.8624	3	1	K.KLDLEAWFPGSGAFR.E	(Ions score 28)
376 - 390	565.2962	1692.8668	1692.8624	3	1	K.KLDLEAWFPGSGAFR.E	(Ions score 29)
376 - 390	565.2962	1692.8668	1692.8624	3	1	K.KLDLEAWFPGSGAFR.E	(Ions score 31)
376 - 390	565.2962	1692.8668	1692.8624	3	1	K.KLDLEAWFPGSGAFR.E	(Ions score 26)
376 - 390	847.4407	1692.8668	1692.8624	3	1	K.KLDLEAWFPGSGAFR.E	(Ions score 61)
376 - 390	565.2962	1692.8668	1692.8624	3	1	K.KLDLEAWFPGSGAFR.E	(Ions score 27)
376 - 390	565.2962	1692.8668	1692.8624	3	1	K.KLDLEAWFPGSGAFR.E	(Ions score 28)
376 - 390	565.2962	1692.8669	1692.8624	3	1	K.KLDLEAWFPGSGAFR.E	(Ions score 29)
376 - 390	565.2962	1692.8669	1692.8624	3	1	K.KLDLEAWFPGSGAFR.E	(Ions score 33)
376 - 390	847.4407	1692.8669	1692.8624	3	1	K.KLDLEAWFPGSGAFR.E	(Ions score 42)
376 - 390	847.4407	1692.8669	1692.8624	3	1	K.KLDLEAWFPGSGAFR.E	(Ions score 73)
376 - 390	565.2963	1692.8670	1692.8624	3	1	K.KLDLEAWFPGSGAFR.E	(Ions score 25)
376 - 390	565.2963	1692.8670	1692.8624	3	1	K.KLDLEAWFPGSGAFR.E	(Ions score 29)
376 - 390	565.2963	1692.8670	1692.8624	3	1	K.KLDLEAWFPGSGAFR.E	(Ions score 27)
376 - 390	565.2963	1692.8671	1692.8624	3	1	K.KLDLEAWFPGSGAFR.E	(Ions score 27)
376 - 390	847.4408	1692.8671	1692.8624	3	1	K.KLDLEAWFPGSGAFR.E	(Ions score 31)
376 - 390	565.2963	1692.8671	1692.8624	3	1	K.KLDLEAWFPGSGAFR.E	(Ions score 26)
376 - 390	565.2963	1692.8672	1692.8624	3	1	K.KLDLEAWFPGSGAFR.E	(Ions score 30)
376 - 390	565.2964	1692.8672	1692.8624	3	1	K.KLDLEAWFPGSGAFR.E	(Ions score 23)
376 - 390	847.4409	1692.8673	1692.8624	3	1	K.KLDLEAWFPGSGAFR.E	(Ions score 47)
376 - 390	565.2964	1692.8673	1692.8624	3	1	K.KLDLEAWFPGSGAFR.E	(Ions score 28)
376 - 390	565.2964	1692.8673	1692.8624	3	1	K.KLDLEAWFPGSGAFR.E	(Ions score 25)
376 - 390	565.2964	1692.8673	1692.8624	3	1	K.KLDLEAWFPGSGAFR.E	(Ions score 25)
376 - 390	565.2964	1692.8673	1692.8624	3	1	K.KLDLEAWFPGSGAFR.E	(Ions score 23)
376 - 390	847.4410	1692.8674	1692.8624	3	1	K.KLDLEAWFPGSGAFR.E	(Ions score 62)
376 - 390	847.4410	1692.8674	1692.8624	3	1	K.KLDLEAWFPGSGAFR.E	(Ions score 52)
376 - 390	565.2964	1692.8674	1692.8624	3	1	K.KLDLEAWFPGSGAFR.E	(Ions score 32)
376 - 390	565.2964	1692.8674	1692.8624	3	1	K.KLDLEAWFPGSGAFR.E	(Ions score 27)
376 - 390	565.2964	1692.8674	1692.8624	3	1	K.KLDLEAWFPGSGAFR.E	(Ions score 28)
376 - 390	565.2964	1692.8675	1692.8624	3	1	K.KLDLEAWFPGSGAFR.E	(Ions score 26)

376 - 390	565.2964	1692.8675	1692.8624	3	1	K.KLDLEAWFPGSGAFR.E	(Ions score 27)
376 - 390	847.4410	1692.8675	1692.8624	3	1	K.KLDLEAWFPGSGAFR.E	(Ions score 49)
376 - 390	565.2965	1692.8676	1692.8624	3	1	K.KLDLEAWFPGSGAFR.E	(Ions score 25)
376 - 390	565.2965	1692.8676	1692.8624	3	1	K.KLDLEAWFPGSGAFR.E	(Ions score 27)
376 - 390	565.2965	1692.8676	1692.8624	3	1	K.KLDLEAWFPGSGAFR.E	(Ions score 23)
376 - 390	847.4411	1692.8677	1692.8624	3	1	K.KLDLEAWFPGSGAFR.E	(Ions score 51)
376 - 390	565.2965	1692.8677	1692.8624	3	1	K.KLDLEAWFPGSGAFR.E	(Ions score 31)
376 - 390	565.2965	1692.8678	1692.8624	3	1	K.KLDLEAWFPGSGAFR.E	(Ions score 23)
376 - 390	565.2965	1692.8678	1692.8624	3	1	K.KLDLEAWFPGSGAFR.E	(Ions score 28)
376 - 390	565.2966	1692.8679	1692.8624	3	1	K.KLDLEAWFPGSGAFR.E	(Ions score 25)
376 - 390	565.2966	1692.8679	1692.8624	3	1	K.KLDLEAWFPGSGAFR.E	(Ions score 21)
376 - 390	565.2966	1692.8679	1692.8624	3	1	K.KLDLEAWFPGSGAFR.E	(Ions score 29)
376 - 390	847.4412	1692.8679	1692.8624	3	1	K.KLDLEAWFPGSGAFR.E	(Ions score 48)
376 - 390	847.4412	1692.8679	1692.8624	3	1	K.KLDLEAWFPGSGAFR.E	(Ions score 65)
376 - 390	565.2966	1692.8679	1692.8624	3	1	K.KLDLEAWFPGSGAFR.E	(Ions score 30)
376 - 390	847.4412	1692.8679	1692.8624	3	1	K.KLDLEAWFPGSGAFR.E	(Ions score 55)
376 - 390	565.2966	1692.8679	1692.8624	3	1	K.KLDLEAWFPGSGAFR.E	(Ions score 25)
376 - 390	565.2966	1692.8680	1692.8624	3	1	K.KLDLEAWFPGSGAFR.E	(Ions score 27)
376 - 390	847.4413	1692.8680	1692.8624	3	1	K.KLDLEAWFPGSGAFR.E	(Ions score 49)
376 - 390	847.4413	1692.8680	1692.8624	3	1	K.KLDLEAWFPGSGAFR.E	(Ions score 32)
376 - 390	565.2966	1692.8680	1692.8624	3	1	K.KLDLEAWFPGSGAFR.E	(Ions score 25)
376 - 390	565.2966	1692.8680	1692.8624	3	1	K.KLDLEAWFPGSGAFR.E	(Ions score 30)
376 - 390	565.2966	1692.8680	1692.8624	3	1	K.KLDLEAWFPGSGAFR.E	(Ions score 27)
376 - 390	565.2966	1692.8680	1692.8624	3	1	K.KLDLEAWFPGSGAFR.E	(Ions score 31)
376 - 390	565.2966	1692.8680	1692.8624	3	1	K.KLDLEAWFPGSGAFR.E	(Ions score 24)
376 - 390	565.2966	1692.8681	1692.8624	3	1	K.KLDLEAWFPGSGAFR.E	(Ions score 28)
376 - 390	565.2966	1692.8681	1692.8624	3	1	K.KLDLEAWFPGSGAFR.E	(Ions score 21)
376 - 390	565.2966	1692.8681	1692.8624	3	1	K.KLDLEAWFPGSGAFR.E	(Ions score 27)
376 - 390	565.2966	1692.8681	1692.8624	3	1	K.KLDLEAWFPGSGAFR.E	(Ions score 25)
376 - 390	847.4414	1692.8682	1692.8624	3	1	K.KLDLEAWFPGSGAFR.E	(Ions score 66)
376 - 390	565.2967	1692.8682	1692.8624	3	1	K.KLDLEAWFPGSGAFR.E	(Ions score 25)
376 - 390	565.2967	1692.8683	1692.8624	3	1	K.KLDLEAWFPGSGAFR.E	(Ions score 35)
376 - 390	847.4414	1692.8683	1692.8624	3	1	K.KLDLEAWFPGSGAFR.E	(Ions score 62)
376 - 390	847.4414	1692.8683	1692.8624	3	1	K.KLDLEAWFPGSGAFR.E	(Ions score 67)
376 - 390	565.2967	1692.8683	1692.8624	4	1	K.KLDLEAWFPGSGAFR.E	(Ions score 27)
376 - 390	565.2967	1692.8683	1692.8624	4	1	K.KLDLEAWFPGSGAFR.E	(Ions score 23)
376 - 390	565.2967	1692.8684	1692.8624	4	1	K.KLDLEAWFPGSGAFR.E	(Ions score 31)
376 - 390	565.2967	1692.8684	1692.8624	4	1	K.KLDLEAWFPGSGAFR.E	(Ions score 26)
376 - 390	565.2968	1692.8684	1692.8624	4	1	K.KLDLEAWFPGSGAFR.E	(Ions score 25)
376 - 390	847.4415	1692.8685	1692.8624	4	1	K.KLDLEAWFPGSGAFR.E	(Ions score 64)
376 - 390	847.4415	1692.8685	1692.8624	4	1	K.KLDLEAWFPGSGAFR.E	(Ions score 62)
376 - 390	565.2968	1692.8686	1692.8624	4	1	K.KLDLEAWFPGSGAFR.E	(Ions score 21)
376 - 390	565.2968	1692.8686	1692.8624	4	1	K.KLDLEAWFPGSGAFR.E	(Ions score 26)
376 - 390	565.2968	1692.8686	1692.8624	4	1	K.KLDLEAWFPGSGAFR.E	(Ions score 32)
376 - 390	565.2968	1692.8686	1692.8624	4	1	K.KLDLEAWFPGSGAFR.E	(Ions score 28)
376 - 390	565.2968	1692.8687	1692.8624	4	1	K.KLDLEAWFPGSGAFR.E	(Ions score 28)
376 - 390	847.4416	1692.8687	1692.8624	4	1	K.KLDLEAWFPGSGAFR.E	(Ions score 44)
376 - 390	565.2968	1692.8687	1692.8624	4	1	K.KLDLEAWFPGSGAFR.E	(Ions score 34)
376 - 390	565.2969	1692.8687	1692.8624	4	1	K.KLDLEAWFPGSGAFR.E	(Ions score 27)
376 - 390	847.4417	1692.8688	1692.8624	4	1	K.KLDLEAWFPGSGAFR.E	(Ions score 52)
376 - 390	565.2969	1692.8689	1692.8624	4	1	K.KLDLEAWFPGSGAFR.E	(Ions score 28)
376 - 390	565.2969	1692.8689	1692.8624	4	1	K.KLDLEAWFPGSGAFR.E	(Ions score 24)
376 - 390	565.2969	1692.8689	1692.8624	4	1	K.KLDLEAWFPGSGAFR.E	(Ions score 31)
376 - 390	565.2969	1692.8690	1692.8624	4	1	K.KLDLEAWFPGSGAFR.E	(Ions score 24)
376 - 390	565.2969	1692.8690	1692.8624	4	1	K.KLDLEAWFPGSGAFR.E	(Ions score 31)
376 - 390	847.4418	1692.8690	1692.8624	4	1	K.KLDLEAWFPGSGAFR.E	(Ions score 64)
376 - 390	847.4418	1692.8690	1692.8624	4	1	K.KLDLEAWFPGSGAFR.E	(Ions score 62)
376 - 390	565.2970	1692.8690	1692.8624	4	1	K.KLDLEAWFPGSGAFR.E	(Ions score 34)

376 - 390	847.4418	1692.8691	1692.8624	4	1	K.KLDLEAWFPGSGAFR.E		(Ions score 65)
376 - 390	847.4418	1692.8691	1692.8624	4	1	K.KLDLEAWFPGSGAFR.E		(Ions score 51)
376 - 390	847.4418	1692.8691	1692.8624	4	1	K.KLDLEAWFPGSGAFR.E		(Ions score 36)
376 - 390	565.2970	1692.8691	1692.8624	4	1	K.KLDLEAWFPGSGAFR.E		(Ions score 25)
376 - 390	565.2970	1692.8692	1692.8624	4	1	K.KLDLEAWFPGSGAFR.E		(Ions score 25)
376 - 390	565.2970	1692.8692	1692.8624	4	1	K.KLDLEAWFPGSGAFR.E		(Ions score 22)
376 - 390	565.2970	1692.8692	1692.8624	4	1	K.KLDLEAWFPGSGAFR.E		(Ions score 29)
376 - 390	565.2970	1692.8692	1692.8624	4	1	K.KLDLEAWFPGSGAFR.E		(Ions score 31)
376 - 390	565.2970	1692.8693	1692.8624	4	1	K.KLDLEAWFPGSGAFR.E		(Ions score 32)
376 - 390	565.2970	1692.8693	1692.8624	4	1	K.KLDLEAWFPGSGAFR.E		(Ions score 29)
376 - 390	565.2971	1692.8693	1692.8624	4	1	K.KLDLEAWFPGSGAFR.E		(Ions score 29)
376 - 390	847.4419	1692.8693	1692.8624	4	1	K.KLDLEAWFPGSGAFR.E		(Ions score 43)
376 - 390	565.2971	1692.8694	1692.8624	4	1	K.KLDLEAWFPGSGAFR.E		(Ions score 29)
376 - 390	565.2971	1692.8694	1692.8624	4	1	K.KLDLEAWFPGSGAFR.E		(Ions score 23)
376 - 390	565.2971	1692.8695	1692.8624	4	1	K.KLDLEAWFPGSGAFR.E		(Ions score 25)
376 - 390	847.4420	1692.8695	1692.8624	4	1	K.KLDLEAWFPGSGAFR.E		(Ions score 65)
376 - 390	847.4420	1692.8695	1692.8624	4	1	K.KLDLEAWFPGSGAFR.E		(Ions score 59)
376 - 390	565.2971	1692.8696	1692.8624	4	1	K.KLDLEAWFPGSGAFR.E		(Ions score 31)
376 - 390	565.2971	1692.8696	1692.8624	4	1	K.KLDLEAWFPGSGAFR.E		(Ions score 28)
376 - 390	847.4421	1692.8696	1692.8624	4	1	K.KLDLEAWFPGSGAFR.E		(Ions score 62)
376 - 390	847.4421	1692.8696	1692.8624	4	1	K.KLDLEAWFPGSGAFR.E		(Ions score 48)
376 - 390	847.4421	1692.8697	1692.8624	4	1	K.KLDLEAWFPGSGAFR.E		(Ions score 59)
376 - 390	847.4421	1692.8697	1692.8624	4	1	K.KLDLEAWFPGSGAFR.E		(Ions score 61)
376 - 390	847.4421	1692.8697	1692.8624	4	1	K.KLDLEAWFPGSGAFR.E		(Ions score 53)
376 - 390	847.4421	1692.8697	1692.8624	4	1	K.KLDLEAWFPGSGAFR.E		(Ions score 63)
376 - 390	565.2972	1692.8697	1692.8624	4	1	K.KLDLEAWFPGSGAFR.E		(Ions score 29)
376 - 390	565.2972	1692.8698	1692.8624	4	1	K.KLDLEAWFPGSGAFR.E		(Ions score 28)
376 - 390	847.4422	1692.8698	1692.8624	4	1	K.KLDLEAWFPGSGAFR.E		(Ions score 54)
376 - 390	847.4422	1692.8698	1692.8624	4	1	K.KLDLEAWFPGSGAFR.E		(Ions score 34)
376 - 390	565.2972	1692.8698	1692.8624	4	1	K.KLDLEAWFPGSGAFR.E		(Ions score 43)
376 - 390	565.2972	1692.8699	1692.8624	4	1	K.KLDLEAWFPGSGAFR.E		(Ions score 26)
376 - 390	565.2972	1692.8699	1692.8624	4	1	K.KLDLEAWFPGSGAFR.E		(Ions score 35)
376 - 390	565.2972	1692.8699	1692.8624	4	1	K.KLDLEAWFPGSGAFR.E		(Ions score 29)
376 - 390	847.4422	1692.8699	1692.8624	4	1	K.KLDLEAWFPGSGAFR.E		(Ions score 57)
376 - 390	847.4422	1692.8699	1692.8624	4	1	K.KLDLEAWFPGSGAFR.E		(Ions score 62)
376 - 390	565.2973	1692.8700	1692.8624	4	1	K.KLDLEAWFPGSGAFR.E		(Ions score 28)
376 - 390	847.4423	1692.8701	1692.8624	5	1	K.KLDLEAWFPGSGAFR.E		(Ions score 62)
376 - 390	565.2973	1692.8701	1692.8624	5	1	K.KLDLEAWFPGSGAFR.E		(Ions score 29)
376 - 390	847.4423	1692.8701	1692.8624	5	1	K.KLDLEAWFPGSGAFR.E		(Ions score 54)
376 - 390	565.2973	1692.8702	1692.8624	5	1	K.KLDLEAWFPGSGAFR.E		(Ions score 28)
376 - 390	847.4424	1692.8702	1692.8624	5	1	K.KLDLEAWFPGSGAFR.E		(Ions score 58)
376 - 390	565.2974	1692.8702	1692.8624	5	1	K.KLDLEAWFPGSGAFR.E		(Ions score 26)
376 - 390	847.4424	1692.8702	1692.8624	5	1	K.KLDLEAWFPGSGAFR.E		(Ions score 64)
376 - 390	565.2974	1692.8703	1692.8624	5	1	K.KLDLEAWFPGSGAFR.E		(Ions score 29)
376 - 390	565.2974	1692.8703	1692.8624	5	1	K.KLDLEAWFPGSGAFR.E		(Ions score 38)
376 - 390	847.4425	1692.8704	1692.8624	5	1	K.KLDLEAWFPGSGAFR.E		(Ions score 50)
376 - 390	847.4425	1692.8704	1692.8624	5	1	K.KLDLEAWFPGSGAFR.E		(Ions score 43)
376 - 390	565.2974	1692.8705	1692.8624	5	1	K.KLDLEAWFPGSGAFR.E		(Ions score 32)
376 - 390	565.2975	1692.8705	1692.8624	5	1	K.KLDLEAWFPGSGAFR.E		(Ions score 30)
376 - 390	847.4426	1692.8706	1692.8624	5	1	K.KLDLEAWFPGSGAFR.E		(Ions score 53)
376 - 390	847.4426	1692.8707	1692.8624	5	1	K.KLDLEAWFPGSGAFR.E		(Ions score 43)
376 - 390	847.4426	1692.8707	1692.8624	5	1	K.KLDLEAWFPGSGAFR.E		(Ions score 65)
376 - 390	565.2975	1692.8708	1692.8624	5	1	K.KLDLEAWFPGSGAFR.E		(Ions score 30)
376 - 390	847.4427	1692.8708	1692.8624	5	1	K.KLDLEAWFPGSGAFR.E		(Ions score 46)
376 - 390	868.4433	1734.8720	1734.8729	-1	1	K.KLDLEAWFPGSGAFR.E	Acetylation (K)	(Ions score 31)
376 - 390	868.4445	1734.8744	1734.8729	1	1	K.KLDLEAWFPGSGAFR.E	Acetylation (K)	(Ions score 68)
376 - 390	868.4446	1734.8746	1734.8729	1	1	K.KLDLEAWFPGSGAFR.E	Acetylation (K)	(Ions score 65)
376 - 390	579.2989	1734.8748	1734.8729	1	1	K.KLDLEAWFPGSGAFR.E	Acetylation (K)	(Ions score 27)

376 - 390	868.4448	1734.8751	1734.8729	1	1	K.KLDLEAWFPGSGAFR.E	Acetylation (K)	(Ions score 28)
376 - 390	579.2991	1734.8754	1734.8729	1	1	K.KLDLEAWFPGSGAFR.E	Acetylation (K)	(Ions score 31)
376 - 390	868.4452	1734.8758	1734.8729	2	1	K.KLDLEAWFPGSGAFR.E	Acetylation (K)	(Ions score 65)
376 - 390	579.2992	1734.8758	1734.8729	2	1	K.KLDLEAWFPGSGAFR.E	Acetylation (K)	(Ions score 30)
376 - 390	579.2993	1734.8760	1734.8729	2	1	K.KLDLEAWFPGSGAFR.E	Acetylation (K)	(Ions score 32)
376 - 390	579.2995	1734.8766	1734.8729	2	1	K.KLDLEAWFPGSGAFR.E	Acetylation (K)	(Ions score 30)
376 - 390	868.4459	1734.8772	1734.8729	2	1	K.KLDLEAWFPGSGAFR.E	Acetylation (K)	(Ions score 63)
376 - 390	579.2998	1734.8775	1734.8729	3	1	K.KLDLEAWFPGSGAFR.E	Acetylation (K)	(Ions score 28)
376 - 390	868.4461	1734.8777	1734.8729	3	1	K.KLDLEAWFPGSGAFR.E	Acetylation (K)	(Ions score 65)
376 - 390	579.2999	1734.8778	1734.8729	3	1	K.KLDLEAWFPGSGAFR.E	Acetylation (K)	(Ions score 29)
376 - 390	579.2999	1734.8778	1734.8729	3	1	K.KLDLEAWFPGSGAFR.E	Acetylation (K)	(Ions score 30)
376 - 390	579.3000	1734.8782	1734.8729	3	1	K.KLDLEAWFPGSGAFR.E	Acetylation (K)	(Ions score 27)
376 - 390	579.3000	1734.8782	1734.8729	3	1	K.KLDLEAWFPGSGAFR.E	Acetylation (K)	(Ions score 22)
376 - 390	868.4464	1734.8783	1734.8729	3	1	K.KLDLEAWFPGSGAFR.E	Acetylation (K)	(Ions score 66)
376 - 390	868.4466	1734.8787	1734.8729	3	1	K.KLDLEAWFPGSGAFR.E	Acetylation (K)	(Ions score 48)
376 - 390	579.3002	1734.8789	1734.8729	3	1	K.KLDLEAWFPGSGAFR.E	Acetylation (K)	(Ions score 25)
376 - 390	868.4468	1734.8790	1734.8729	4	1	K.KLDLEAWFPGSGAFR.E	Acetylation (K)	(Ions score 65)
376 - 390	868.4472	1734.8798	1734.8729	4	1	K.KLDLEAWFPGSGAFR.E	Acetylation (K)	(Ions score 57)
376 - 390	868.4472	1734.8799	1734.8729	4	1	K.KLDLEAWFPGSGAFR.E	Acetylation (K)	(Ions score 58)
376 - 390	868.4473	1734.8800	1734.8729	4	1	K.KLDLEAWFPGSGAFR.E	Acetylation (K)	(Ions score 60)
376 - 390	868.4475	1734.8805	1734.8729	4	1	K.KLDLEAWFPGSGAFR.E	Acetylation (K)	(Ions score 70)
377 - 390	783.3904	1564.7662	1564.7674	-1	0	K.LDLEAWFPGSGAFR.E	(Ions score 26)	
377 - 390	783.3917	1564.7688	1564.7674	1	0	K.LDLEAWFPGSGAFR.E	(Ions score 32)	
377 - 390	522.5970	1564.7690	1564.7674	1	0	K.LDLEAWFPGSGAFR.E	(Ions score 31)	
377 - 390	783.3918	1564.7691	1564.7674	1	0	K.LDLEAWFPGSGAFR.E	(Ions score 26)	
377 - 390	783.3918	1564.7691	1564.7674	1	0	K.LDLEAWFPGSGAFR.E	(Ions score 45)	
377 - 390	522.5970	1564.7691	1564.7674	1	0	K.LDLEAWFPGSGAFR.E	(Ions score 31)	
377 - 390	522.5970	1564.7692	1564.7674	1	0	K.LDLEAWFPGSGAFR.E	(Ions score 39)	
377 - 390	522.5971	1564.7694	1564.7674	1	0	K.LDLEAWFPGSGAFR.E	(Ions score 37)	
377 - 390	783.3920	1564.7695	1564.7674	1	0	K.LDLEAWFPGSGAFR.E	(Ions score 34)	
377 - 390	522.5971	1564.7696	1564.7674	1	0	K.LDLEAWFPGSGAFR.E	(Ions score 38)	
377 - 390	522.5972	1564.7696	1564.7674	1	0	K.LDLEAWFPGSGAFR.E	(Ions score 28)	
377 - 390	783.3921	1564.7696	1564.7674	1	0	K.LDLEAWFPGSGAFR.E	(Ions score 37)	
377 - 390	522.5972	1564.7697	1564.7674	1	0	K.LDLEAWFPGSGAFR.E	(Ions score 38)	
377 - 390	783.3922	1564.7699	1564.7674	2	0	K.LDLEAWFPGSGAFR.E	(Ions score 48)	
377 - 390	783.3923	1564.7700	1564.7674	2	0	K.LDLEAWFPGSGAFR.E	(Ions score 38)	
377 - 390	522.5973	1564.7702	1564.7674	2	0	K.LDLEAWFPGSGAFR.E	(Ions score 37)	
377 - 390	783.3924	1564.7702	1564.7674	2	0	K.LDLEAWFPGSGAFR.E	(Ions score 49)	
377 - 390	783.3924	1564.7703	1564.7674	2	0	K.LDLEAWFPGSGAFR.E	(Ions score 27)	
377 - 390	783.3925	1564.7704	1564.7674	2	0	K.LDLEAWFPGSGAFR.E	(Ions score 47)	
377 - 390	783.3925	1564.7705	1564.7674	2	0	K.LDLEAWFPGSGAFR.E	(Ions score 28)	
377 - 390	783.3925	1564.7705	1564.7674	2	0	K.LDLEAWFPGSGAFR.E	(Ions score 28)	
377 - 390	783.3926	1564.7707	1564.7674	2	0	K.LDLEAWFPGSGAFR.E	(Ions score 34)	
377 - 390	783.3927	1564.7708	1564.7674	2	0	K.LDLEAWFPGSGAFR.E	(Ions score 30)	
377 - 390	522.5976	1564.7709	1564.7674	2	0	K.LDLEAWFPGSGAFR.E	(Ions score 38)	
377 - 390	783.3927	1564.7709	1564.7674	2	0	K.LDLEAWFPGSGAFR.E	(Ions score 44)	
377 - 390	783.3928	1564.7710	1564.7674	2	0	K.LDLEAWFPGSGAFR.E	(Ions score 55)	
377 - 390	783.3929	1564.7712	1564.7674	2	0	K.LDLEAWFPGSGAFR.E	(Ions score 44)	
377 - 390	783.3929	1564.7712	1564.7674	2	0	K.LDLEAWFPGSGAFR.E	(Ions score 29)	
377 - 390	783.3929	1564.7713	1564.7674	2	0	K.LDLEAWFPGSGAFR.E	(Ions score 40)	
377 - 390	783.3931	1564.7715	1564.7674	3	0	K.LDLEAWFPGSGAFR.E	(Ions score 34)	
377 - 390	783.3931	1564.7717	1564.7674	3	0	K.LDLEAWFPGSGAFR.E	(Ions score 34)	
377 - 390	783.3932	1564.7718	1564.7674	3	0	K.LDLEAWFPGSGAFR.E	(Ions score 23)	
377 - 390	783.3932	1564.7718	1564.7674	3	0	K.LDLEAWFPGSGAFR.E	(Ions score 61)	
377 - 390	783.3932	1564.7718	1564.7674	3	0	K.LDLEAWFPGSGAFR.E	(Ions score 54)	
377 - 390	783.3932	1564.7719	1564.7674	3	0	K.LDLEAWFPGSGAFR.E	(Ions score 31)	
377 - 390	783.3932	1564.7719	1564.7674	3	0	K.LDLEAWFPGSGAFR.E	(Ions score 38)	
377 - 390	783.3932	1564.7719	1564.7674	3	0	K.LDLEAWFPGSGAFR.E	(Ions score 45)	

377 - 390	783.3933	1564.7720	1564.7674	3	0	K.LDLEAWFPGSGAFR.E	(Ions score 29)
377 - 390	783.3933	1564.7720	1564.7674	3	0	K.LDLEAWFPGSGAFR.E	(Ions score 48)
377 - 390	783.3933	1564.7720	1564.7674	3	0	K.LDLEAWFPGSGAFR.E	(Ions score 57)
377 - 390	783.3933	1564.7720	1564.7674	3	0	K.LDLEAWFPGSGAFR.E	(Ions score 55)
377 - 390	783.3933	1564.7721	1564.7674	3	0	K.LDLEAWFPGSGAFR.E	(Ions score 52)
377 - 390	783.3933	1564.7721	1564.7674	3	0	K.LDLEAWFPGSGAFR.E	(Ions score 56)
377 - 390	783.3933	1564.7721	1564.7674	3	0	K.LDLEAWFPGSGAFR.E	(Ions score 60)
377 - 390	783.3934	1564.7722	1564.7674	3	0	K.LDLEAWFPGSGAFR.E	(Ions score 27)
377 - 390	783.3934	1564.7722	1564.7674	3	0	K.LDLEAWFPGSGAFR.E	(Ions score 58)
377 - 390	783.3934	1564.7723	1564.7674	3	0	K.LDLEAWFPGSGAFR.E	(Ions score 27)
377 - 390	783.3935	1564.7724	1564.7674	3	0	K.LDLEAWFPGSGAFR.E	(Ions score 33)
377 - 390	783.3935	1564.7725	1564.7674	3	0	K.LDLEAWFPGSGAFR.E	(Ions score 55)
377 - 390	783.3935	1564.7725	1564.7674	3	0	K.LDLEAWFPGSGAFR.E	(Ions score 61)
377 - 390	783.3936	1564.7726	1564.7674	3	0	K.LDLEAWFPGSGAFR.E	(Ions score 40)
377 - 390	783.3936	1564.7727	1564.7674	3	0	K.LDLEAWFPGSGAFR.E	(Ions score 53)
377 - 390	783.3936	1564.7727	1564.7674	3	0	K.LDLEAWFPGSGAFR.E	(Ions score 44)
377 - 390	783.3936	1564.7727	1564.7674	3	0	K.LDLEAWFPGSGAFR.E	(Ions score 46)
377 - 390	783.3937	1564.7728	1564.7674	3	0	K.LDLEAWFPGSGAFR.E	(Ions score 33)
377 - 390	783.3937	1564.7728	1564.7674	3	0	K.LDLEAWFPGSGAFR.E	(Ions score 43)
377 - 390	783.3937	1564.7729	1564.7674	3	0	K.LDLEAWFPGSGAFR.E	(Ions score 27)
377 - 390	783.3937	1564.7729	1564.7674	4	0	K.LDLEAWFPGSGAFR.E	(Ions score 39)
377 - 390	783.3938	1564.7730	1564.7674	4	0	K.LDLEAWFPGSGAFR.E	(Ions score 40)
377 - 390	783.3938	1564.7730	1564.7674	4	0	K.LDLEAWFPGSGAFR.E	(Ions score 34)
377 - 390	783.3938	1564.7731	1564.7674	4	0	K.LDLEAWFPGSGAFR.E	(Ions score 52)
377 - 390	783.3939	1564.7732	1564.7674	4	0	K.LDLEAWFPGSGAFR.E	(Ions score 29)
377 - 390	783.3939	1564.7732	1564.7674	4	0	K.LDLEAWFPGSGAFR.E	(Ions score 30)
377 - 390	783.3939	1564.7732	1564.7674	4	0	K.LDLEAWFPGSGAFR.E	(Ions score 32)
377 - 390	783.3939	1564.7733	1564.7674	4	0	K.LDLEAWFPGSGAFR.E	(Ions score 59)
377 - 390	783.3940	1564.7733	1564.7674	4	0	K.LDLEAWFPGSGAFR.E	(Ions score 47)
377 - 390	783.3940	1564.7734	1564.7674	4	0	K.LDLEAWFPGSGAFR.E	(Ions score 25)
377 - 390	783.3940	1564.7735	1564.7674	4	0	K.LDLEAWFPGSGAFR.E	(Ions score 68)
377 - 390	783.3940	1564.7735	1564.7674	4	0	K.LDLEAWFPGSGAFR.E	(Ions score 32)
377 - 390	783.3941	1564.7736	1564.7674	4	0	K.LDLEAWFPGSGAFR.E	(Ions score 43)
377 - 390	783.3941	1564.7737	1564.7674	4	0	K.LDLEAWFPGSGAFR.E	(Ions score 32)
377 - 390	783.3942	1564.7737	1564.7674	4	0	K.LDLEAWFPGSGAFR.E	(Ions score 43)
377 - 390	783.3942	1564.7738	1564.7674	4	0	K.LDLEAWFPGSGAFR.E	(Ions score 21)
377 - 390	783.3942	1564.7738	1564.7674	4	0	K.LDLEAWFPGSGAFR.E	(Ions score 31)
377 - 390	783.3943	1564.7740	1564.7674	4	0	K.LDLEAWFPGSGAFR.E	(Ions score 32)
377 - 390	783.3943	1564.7741	1564.7674	4	0	K.LDLEAWFPGSGAFR.E	(Ions score 59)
377 - 390	783.3945	1564.7744	1564.7674	4	0	K.LDLEAWFPGSGAFR.E	(Ions score 44)
377 - 390	783.3945	1564.7744	1564.7674	4	0	K.LDLEAWFPGSGAFR.E	(Ions score 29)
377 - 390	783.3948	1564.7750	1564.7674	5	0	K.LDLEAWFPGSGAFR.E	(Ions score 37)
377 - 390	783.3948	1564.7751	1564.7674	5	0	K.LDLEAWFPGSGAFR.E	(Ions score 44)
377 - 390	783.3949	1564.7752	1564.7674	5	0	K.LDLEAWFPGSGAFR.E	(Ions score 57)
391 - 404	851.8599	1701.7053	1701.7087	-2	0	R.ELVSCSNCTDYQAR.R	(Ions score 48)
391 - 404	851.8601	1701.7057	1701.7087	-2	0	R.ELVSCSNCTDYQAR.R	(Ions score 37)
391 - 404	851.8602	1701.7058	1701.7087	-2	0	R.ELVSCSNCTDYQAR.R	(Ions score 77)
391 - 404	851.8604	1701.7063	1701.7087	-1	0	R.ELVSCSNCTDYQAR.R	(Ions score 48)
391 - 404	851.8605	1701.7064	1701.7087	-1	0	R.ELVSCSNCTDYQAR.R	(Ions score 82)
391 - 404	851.8608	1701.7070	1701.7087	-1	0	R.ELVSCSNCTDYQAR.R	(Ions score 85)
391 - 404	851.8609	1701.7073	1701.7087	-1	0	R.ELVSCSNCTDYQAR.R	(Ions score 82)
391 - 404	851.8613	1701.7081	1701.7087	-0	0	R.ELVSCSNCTDYQAR.R	(Ions score 85)
391 - 404	851.8615	1701.7085	1701.7087	-0	0	R.ELVSCSNCTDYQAR.R	(Ions score 40)
391 - 404	851.8617	1701.7089	1701.7087	0	0	R.ELVSCSNCTDYQAR.R	(Ions score 74)
391 - 404	851.8619	1701.7093	1701.7087	0	0	R.ELVSCSNCTDYQAR.R	(Ions score 95)
391 - 404	851.8621	1701.7096	1701.7087	1	0	R.ELVSCSNCTDYQAR.R	(Ions score 77)
391 - 404	851.8621	1701.7097	1701.7087	1	0	R.ELVSCSNCTDYQAR.R	(Ions score 54)
391 - 404	851.8621	1701.7097	1701.7087	1	0	R.ELVSCSNCTDYQAR.R	(Ions score 77)

391 - 404	851.8622	1701.7098	1701.7087	1	0	R.ELVSCSNCTDYQAR.R	(Ions score 65)
391 - 404	851.8622	1701.7098	1701.7087	1	0	R.ELVSCSNCTDYQAR.R	(Ions score 30)
391 - 404	851.8622	1701.7099	1701.7087	1	0	R.ELVSCSNCTDYQAR.R	(Ions score 82)
391 - 404	851.8623	1701.7100	1701.7087	1	0	R.ELVSCSNCTDYQAR.R	(Ions score 95)
391 - 404	851.8623	1701.7101	1701.7087	1	0	R.ELVSCSNCTDYQAR.R	(Ions score 95)
391 - 404	568.2440	1701.7101	1701.7087	1	0	R.ELVSCSNCTDYQAR.R	(Ions score 43)
391 - 404	851.8624	1701.7102	1701.7087	1	0	R.ELVSCSNCTDYQAR.R	(Ions score 56)
391 - 404	851.8624	1701.7103	1701.7087	1	0	R.ELVSCSNCTDYQAR.R	(Ions score 61)
391 - 404	851.8626	1701.7106	1701.7087	1	0	R.ELVSCSNCTDYQAR.R	(Ions score 72)
391 - 404	851.8627	1701.7109	1701.7087	1	0	R.ELVSCSNCTDYQAR.R	(Ions score 59)
391 - 404	851.8628	1701.7110	1701.7087	1	0	R.ELVSCSNCTDYQAR.R	(Ions score 53)
391 - 404	851.8628	1701.7111	1701.7087	1	0	R.ELVSCSNCTDYQAR.R	(Ions score 33)
391 - 404	851.8629	1701.7113	1701.7087	2	0	R.ELVSCSNCTDYQAR.R	(Ions score 77)
391 - 404	851.8630	1701.7114	1701.7087	2	0	R.ELVSCSNCTDYQAR.R	(Ions score 95)
391 - 404	851.8630	1701.7115	1701.7087	2	0	R.ELVSCSNCTDYQAR.R	(Ions score 62)
391 - 404	568.2444	1701.7115	1701.7087	2	0	R.ELVSCSNCTDYQAR.R	(Ions score 42)
391 - 404	851.8631	1701.7116	1701.7087	2	0	R.ELVSCSNCTDYQAR.R	(Ions score 72)
391 - 404	851.8631	1701.7116	1701.7087	2	0	R.ELVSCSNCTDYQAR.R	(Ions score 88)
391 - 404	851.8631	1701.7117	1701.7087	2	0	R.ELVSCSNCTDYQAR.R	(Ions score 26)
391 - 404	851.8632	1701.7117	1701.7087	2	0	R.ELVSCSNCTDYQAR.R	(Ions score 88)
391 - 404	851.8632	1701.7118	1701.7087	2	0	R.ELVSCSNCTDYQAR.R	(Ions score 57)
391 - 404	851.8632	1701.7119	1701.7087	2	0	R.ELVSCSNCTDYQAR.R	(Ions score 85)
391 - 404	851.8633	1701.7120	1701.7087	2	0	R.ELVSCSNCTDYQAR.R	(Ions score 99)
391 - 404	568.2446	1701.7120	1701.7087	2	0	R.ELVSCSNCTDYQAR.R	(Ions score 34)
391 - 404	851.8633	1701.7120	1701.7087	2	0	R.ELVSCSNCTDYQAR.R	(Ions score 85)
391 - 404	851.8633	1701.7121	1701.7087	2	0	R.ELVSCSNCTDYQAR.R	(Ions score 85)
391 - 404	851.8634	1701.7122	1701.7087	2	0	R.ELVSCSNCTDYQAR.R	(Ions score 52)
391 - 404	851.8634	1701.7123	1701.7087	2	0	R.ELVSCSNCTDYQAR.R	(Ions score 65)
391 - 404	851.8635	1701.7124	1701.7087	2	0	R.ELVSCSNCTDYQAR.R	(Ions score 66)
391 - 404	851.8635	1701.7124	1701.7087	2	0	R.ELVSCSNCTDYQAR.R	(Ions score 31)
391 - 404	851.8636	1701.7126	1701.7087	2	0	R.ELVSCSNCTDYQAR.R	(Ions score 58)
391 - 404	851.8636	1701.7126	1701.7087	2	0	R.ELVSCSNCTDYQAR.R	(Ions score 88)
391 - 404	851.8636	1701.7127	1701.7087	2	0	R.ELVSCSNCTDYQAR.R	(Ions score 41)
391 - 404	851.8637	1701.7128	1701.7087	2	0	R.ELVSCSNCTDYQAR.R	(Ions score 95)
391 - 404	568.2449	1701.7129	1701.7087	2	0	R.ELVSCSNCTDYQAR.R	(Ions score 49)
391 - 404	568.2449	1701.7130	1701.7087	3	0	R.ELVSCSNCTDYQAR.R	(Ions score 40)
391 - 404	851.8638	1701.7130	1701.7087	3	0	R.ELVSCSNCTDYQAR.R	(Ions score 57)
391 - 404	851.8638	1701.7130	1701.7087	3	0	R.ELVSCSNCTDYQAR.R	(Ions score 70)
391 - 404	851.8638	1701.7130	1701.7087	3	0	R.ELVSCSNCTDYQAR.R	(Ions score 73)
391 - 404	851.8638	1701.7130	1701.7087	3	0	R.ELVSCSNCTDYQAR.R	(Ions score 53)
391 - 404	851.8638	1701.7131	1701.7087	3	0	R.ELVSCSNCTDYQAR.R	(Ions score 76)
391 - 404	851.8638	1701.7131	1701.7087	3	0	R.ELVSCSNCTDYQAR.R	(Ions score 56)
391 - 404	851.8639	1701.7132	1701.7087	3	0	R.ELVSCSNCTDYQAR.R	(Ions score 59)
391 - 404	851.8639	1701.7132	1701.7087	3	0	R.ELVSCSNCTDYQAR.R	(Ions score 85)
391 - 404	851.8639	1701.7132	1701.7087	3	0	R.ELVSCSNCTDYQAR.R	(Ions score 68)
391 - 404	851.8639	1701.7133	1701.7087	3	0	R.ELVSCSNCTDYQAR.R	(Ions score 28)
391 - 404	851.8640	1701.7134	1701.7087	3	0	R.ELVSCSNCTDYQAR.R	(Ions score 73)
391 - 404	851.8640	1701.7134	1701.7087	3	0	R.ELVSCSNCTDYQAR.R	(Ions score 76)
391 - 404	851.8640	1701.7134	1701.7087	3	0	R.ELVSCSNCTDYQAR.R	(Ions score 81)
391 - 404	851.8640	1701.7135	1701.7087	3	0	R.ELVSCSNCTDYQAR.R	(Ions score 53)
391 - 404	851.8641	1701.7137	1701.7087	3	0	R.ELVSCSNCTDYQAR.R	(Ions score 68)
391 - 404	851.8641	1701.7137	1701.7087	3	0	R.ELVSCSNCTDYQAR.R	(Ions score 71)
391 - 404	851.8642	1701.7138	1701.7087	3	0	R.ELVSCSNCTDYQAR.R	(Ions score 84)
391 - 404	851.8642	1701.7139	1701.7087	3	0	R.ELVSCSNCTDYQAR.R	(Ions score 57)
391 - 404	568.2453	1701.7139	1701.7087	3	0	R.ELVSCSNCTDYQAR.R	(Ions score 63)
391 - 404	851.8643	1701.7140	1701.7087	3	0	R.ELVSCSNCTDYQAR.R	(Ions score 76)
391 - 404	851.8644	1701.7142	1701.7087	3	0	R.ELVSCSNCTDYQAR.R	(Ions score 60)
391 - 404	851.8644	1701.7143	1701.7087	3	0	R.ELVSCSNCTDYQAR.R	(Ions score 72)

391 - 404	851.8644	1701.7143	1701.7087	3	0	R.ELVSCSNCTDYQAR.R	(Ions score 45)
391 - 404	851.8644	1701.7143	1701.7087	3	0	R.ELVSCSNCTDYQAR.R	(Ions score 88)
391 - 404	851.8645	1701.7144	1701.7087	3	0	R.ELVSCSNCTDYQAR.R	(Ions score 96)
391 - 404	851.8645	1701.7145	1701.7087	3	0	R.ELVSCSNCTDYQAR.R	(Ions score 56)
391 - 404	851.8646	1701.7145	1701.7087	3	0	R.ELVSCSNCTDYQAR.R	(Ions score 56)
391 - 404	851.8646	1701.7146	1701.7087	3	0	R.ELVSCSNCTDYQAR.R	(Ions score 68)
391 - 404	851.8646	1701.7147	1701.7087	4	0	R.ELVSCSNCTDYQAR.R	(Ions score 85)
391 - 404	851.8646	1701.7147	1701.7087	4	0	R.ELVSCSNCTDYQAR.R	(Ions score 51)
391 - 404	851.8646	1701.7147	1701.7087	4	0	R.ELVSCSNCTDYQAR.R	(Ions score 56)
391 - 404	851.8647	1701.7149	1701.7087	4	0	R.ELVSCSNCTDYQAR.R	(Ions score 91)
391 - 404	851.8647	1701.7149	1701.7087	4	0	R.ELVSCSNCTDYQAR.R	(Ions score 53)
391 - 404	851.8647	1701.7149	1701.7087	4	0	R.ELVSCSNCTDYQAR.R	(Ions score 53)
391 - 404	851.8648	1701.7151	1701.7087	4	0	R.ELVSCSNCTDYQAR.R	(Ions score 60)
391 - 404	851.8648	1701.7151	1701.7087	4	0	R.ELVSCSNCTDYQAR.R	(Ions score 73)
391 - 404	851.8648	1701.7151	1701.7087	4	0	R.ELVSCSNCTDYQAR.R	(Ions score 56)
391 - 404	851.8648	1701.7151	1701.7087	4	0	R.ELVSCSNCTDYQAR.R	(Ions score 77)
391 - 404	851.8649	1701.7152	1701.7087	4	0	R.ELVSCSNCTDYQAR.R	(Ions score 59)
391 - 404	851.8649	1701.7153	1701.7087	4	0	R.ELVSCSNCTDYQAR.R	(Ions score 53)
391 - 404	851.8650	1701.7154	1701.7087	4	0	R.ELVSCSNCTDYQAR.R	(Ions score 47)
391 - 404	851.8650	1701.7154	1701.7087	4	0	R.ELVSCSNCTDYQAR.R	(Ions score 88)
391 - 404	851.8650	1701.7155	1701.7087	4	0	R.ELVSCSNCTDYQAR.R	(Ions score 73)
391 - 404	851.8651	1701.7156	1701.7087	4	0	R.ELVSCSNCTDYQAR.R	(Ions score 77)
391 - 404	851.8651	1701.7157	1701.7087	4	0	R.ELVSCSNCTDYQAR.R	(Ions score 81)
391 - 404	851.8651	1701.7157	1701.7087	4	0	R.ELVSCSNCTDYQAR.R	(Ions score 69)
391 - 404	851.8652	1701.7158	1701.7087	4	0	R.ELVSCSNCTDYQAR.R	(Ions score 93)
391 - 404	851.8652	1701.7159	1701.7087	4	0	R.ELVSCSNCTDYQAR.R	(Ions score 56)
391 - 404	851.8653	1701.7161	1701.7087	4	0	R.ELVSCSNCTDYQAR.R	(Ions score 95)
391 - 404	851.8655	1701.7164	1701.7087	5	0	R.ELVSCSNCTDYQAR.R	(Ions score 75)
391 - 404	851.8655	1701.7164	1701.7087	5	0	R.ELVSCSNCTDYQAR.R	(Ions score 97)
391 - 404	851.8655	1701.7165	1701.7087	5	0	R.ELVSCSNCTDYQAR.R	(Ions score 95)
391 - 404	851.8655	1701.7165	1701.7087	5	0	R.ELVSCSNCTDYQAR.R	(Ions score 76)
391 - 404	851.8655	1701.7165	1701.7087	5	0	R.ELVSCSNCTDYQAR.R	(Ions score 56)
391 - 404	851.8656	1701.7167	1701.7087	5	0	R.ELVSCSNCTDYQAR.R	(Ions score 59)
391 - 404	851.8657	1701.7168	1701.7087	5	0	R.ELVSCSNCTDYQAR.R	(Ions score 98)
391 - 404	851.8657	1701.7168	1701.7087	5	0	R.ELVSCSNCTDYQAR.R	(Ions score 100)
391 - 404	851.8657	1701.7168	1701.7087	5	0	R.ELVSCSNCTDYQAR.R	(Ions score 76)
391 - 404	851.8657	1701.7169	1701.7087	5	0	R.ELVSCSNCTDYQAR.R	(Ions score 95)
391 - 404	851.8657	1701.7169	1701.7087	5	0	R.ELVSCSNCTDYQAR.R	(Ions score 57)
391 - 404	851.8658	1701.7170	1701.7087	5	0	R.ELVSCSNCTDYQAR.R	(Ions score 60)
410 - 414	596.3039	595.2966	595.2966	0	0	R.YGQTK.K	(Ions score 23)
410 - 414	596.3039	595.2966	595.2966	0	0	R.YGQTK.K	(Ions score 28)
410 - 414	596.3039	595.2966	595.2966	0	0	R.YGQTK.K	(Ions score 21)
410 - 414	596.3039	595.2966	595.2966	0	0	R.YGQTK.K	(Ions score 25)
410 - 414	596.3039	595.2966	595.2966	0	0	R.YGQTK.K	(Ions score 25)
410 - 414	596.3039	595.2966	595.2966	0	0	R.YGQTK.K	(Ions score 22)
410 - 414	596.3039	595.2966	595.2966	0	0	R.YGQTK.K	(Ions score 21)
410 - 414	596.3039	595.2966	595.2966	0	0	R.YGQTK.K	(Ions score 21)
410 - 414	596.3039	595.2966	595.2966	0	0	R.YGQTK.K	(Ions score 22)
410 - 414	596.3039	595.2966	595.2966	0	0	R.YGQTK.K	(Ions score 23)
410 - 414	596.3039	595.2966	595.2966	0	0	R.YGQTK.K	(Ions score 21)
410 - 414	596.3039	595.2966	595.2966	0	0	R.YGQTK.K	(Ions score 23)
410 - 414	596.3039	595.2966	595.2966	0	0	R.YGQTK.K	(Ions score 25)
410 - 414	596.3039	595.2966	595.2966	0	0	R.YGQTK.K	(Ions score 23)
410 - 414	596.3039	595.2966	595.2966	0	0	R.YGQTK.K	(Ions score 23)
410 - 414	596.3039	595.2966	595.2966	0	0	R.YGQTK.K	(Ions score 22)
410 - 414	596.3039	595.2966	595.2966	0	0	R.YGQTK.K	(Ions score 21)
410 - 414	596.3039	595.2966	595.2966	0	0	R.YGQTK.K	(Ions score 21)
410 - 414	596.3039	595.2966	595.2966	0	0	R.YGQTK.K	(Ions score 22)

410 - 414	596.3039	595.2966	595.2966	0	0	R.YGQTK.K		(Ions score 23)
410 - 414	596.3039	595.2966	595.2966	0	0	R.YGQTK.K		(Ions score 23)
416 - 435	597.2737	2385.0656	2385.0772	-5	1	K.MMDKVEFVHMLNATMCATTR.T		(Ions score 47)
416 - 435	796.0294	2385.0664	2385.0772	-5	1	K.MMDKVEFVHMLNATMCATTR.T		(Ions score 44)
416 - 435	597.2745	2385.0690	2385.0772	-3	1	K.MMDKVEFVHMLNATMCATTR.T		(Ions score 58)
416 - 435	597.2745	2385.0690	2385.0772	-3	1	K.MMDKVEFVHMLNATMCATTR.T		(Ions score 47)
416 - 435	796.0307	2385.0703	2385.0772	-3	1	K.MMDKVEFVHMLNATMCATTR.T		(Ions score 63)
416 - 435	796.0323	2385.0750	2385.0772	-1	1	K.MMDKVEFVHMLNATMCATTR.T		(Ions score 31)
416 - 435	796.0323	2385.0752	2385.0772	-1	1	K.MMDKVEFVHMLNATMCATTR.T		(Ions score 38)
416 - 435	597.2765	2385.0769	2385.0772	-0	1	K.MMDKVEFVHMLNATMCATTR.T		(Ions score 35)
416 - 435	796.0329	2385.0770	2385.0772	-0	1	K.MMDKVEFVHMLNATMCATTR.T		(Ions score 24)
416 - 435	597.2766	2385.0771	2385.0772	-0	1	K.MMDKVEFVHMLNATMCATTR.T		(Ions score 49)
416 - 435	597.2766	2385.0774	2385.0772	0	1	K.MMDKVEFVHMLNATMCATTR.T		(Ions score 34)
416 - 435	597.2767	2385.0778	2385.0772	0	1	K.MMDKVEFVHMLNATMCATTR.T		(Ions score 46)
416 - 435	796.0333	2385.0782	2385.0772	0	1	K.MMDKVEFVHMLNATMCATTR.T		(Ions score 32)
416 - 435	796.0336	2385.0789	2385.0772	1	1	K.MMDKVEFVHMLNATMCATTR.T		(Ions score 50)
416 - 435	597.2771	2385.0793	2385.0772	1	1	K.MMDKVEFVHMLNATMCATTR.T		(Ions score 46)
416 - 435	597.2772	2385.0796	2385.0772	1	1	K.MMDKVEFVHMLNATMCATTR.T		(Ions score 23)
416 - 435	597.2772	2385.0797	2385.0772	1	1	K.MMDKVEFVHMLNATMCATTR.T		(Ions score 57)
416 - 435	597.2773	2385.0801	2385.0772	1	1	K.MMDKVEFVHMLNATMCATTR.T		(Ions score 38)
416 - 435	597.2774	2385.0805	2385.0772	1	1	K.MMDKVEFVHMLNATMCATTR.T		(Ions score 35)
416 - 435	796.0344	2385.0812	2385.0772	2	1	K.MMDKVEFVHMLNATMCATTR.T		(Ions score 77)
416 - 435	597.2777	2385.0815	2385.0772	2	1	K.MMDKVEFVHMLNATMCATTR.T		(Ions score 39)
416 - 435	597.2777	2385.0818	2385.0772	2	1	K.MMDKVEFVHMLNATMCATTR.T		(Ions score 35)
416 - 435	597.2778	2385.0820	2385.0772	2	1	K.MMDKVEFVHMLNATMCATTR.T		(Ions score 34)
416 - 435	597.2779	2385.0825	2385.0772	2	1	K.MMDKVEFVHMLNATMCATTR.T		(Ions score 46)
416 - 435	597.2781	2385.0832	2385.0772	3	1	K.MMDKVEFVHMLNATMCATTR.T		(Ions score 33)
416 - 435	796.0353	2385.0840	2385.0772	3	1	K.MMDKVEFVHMLNATMCATTR.T		(Ions score 81)
416 - 435	597.2783	2385.0840	2385.0772	3	1	K.MMDKVEFVHMLNATMCATTR.T		(Ions score 39)
416 - 435	597.2786	2385.0852	2385.0772	3	1	K.MMDKVEFVHMLNATMCATTR.T		(Ions score 35)
416 - 435	597.2788	2385.0863	2385.0772	4	1	K.MMDKVEFVHMLNATMCATTR.T		(Ions score 25)
416 - 435	1193.5505	2385.0865	2385.0772	4	1	K.MMDKVEFVHMLNATMCATTR.T		(Ions score 83)
416 - 435	601.2729	2401.0623	2401.0721	-4	1	K.MMDKVEFVHMLNATMCATTR.T	Oxidation (M)	(Ions score 62)
416 - 435	601.2731	2401.0634	2401.0721	-4	1	K.MMDKVEFVHMLNATMCATTR.T	Oxidation (M)	(Ions score 62)
416 - 435	601.2737	2401.0656	2401.0721	-3	1	K.MMDKVEFVHMLNATMCATTR.T	Oxidation (M)	(Ions score 24)
416 - 435	801.3629	2401.0667	2401.0721	-2	1	K.MMDKVEFVHMLNATMCATTR.T	Oxidation (M)	(Ions score 61)
416 - 435	801.3635	2401.0686	2401.0721	-1	1	K.MMDKVEFVHMLNATMCATTR.T	Oxidation (M)	(Ions score 51)
416 - 435	801.3636	2401.0689	2401.0721	-1	1	K.MMDKVEFVHMLNATMCATTR.T	Oxidation (M)	(Ions score 30)
416 - 435	801.3636	2401.0689	2401.0721	-1	1	K.MMDKVEFVHMLNATMCATTR.T	Oxidation (M)	(Ions score 58)
416 - 435	801.3640	2401.0701	2401.0721	-1	1	K.MMDKVEFVHMLNATMCATTR.T	Oxidation (M)	(Ions score 76)
416 - 435	801.3640	2401.0702	2401.0721	-1	1	K.MMDKVEFVHMLNATMCATTR.T	Oxidation (M)	(Ions score 30)
416 - 435	801.3641	2401.0704	2401.0721	-1	1	K.MMDKVEFVHMLNATMCATTR.T	Oxidation (M)	(Ions score 35)
416 - 435	801.3641	2401.0704	2401.0721	-1	1	K.MMDKVEFVHMLNATMCATTR.T	Oxidation (M)	(Ions score 66)
416 - 435	801.3644	2401.0713	2401.0721	-0	1	K.MMDKVEFVHMLNATMCATTR.T	Oxidation (M)	(Ions score 53)
416 - 435	801.3645	2401.0717	2401.0721	-0	1	K.MMDKVEFVHMLNATMCATTR.T	Oxidation (M)	(Ions score 26)
416 - 435	801.3645	2401.0717	2401.0721	-0	1	K.MMDKVEFVHMLNATMCATTR.T	Oxidation (M)	(Ions score 34)
416 - 435	801.3647	2401.0722	2401.0721	0	1	K.MMDKVEFVHMLNATMCATTR.T	Oxidation (M)	(Ions score 64)
416 - 435	801.3648	2401.0725	2401.0721	0	1	K.MMDKVEFVHMLNATMCATTR.T	Oxidation (M)	(Ions score 30)
416 - 435	801.3648	2401.0726	2401.0721	0	1	K.MMDKVEFVHMLNATMCATTR.T	Oxidation (M)	(Ions score 58)
416 - 435	801.3648	2401.0726	2401.0721	0	1	K.MMDKVEFVHMLNATMCATTR.T	Oxidation (M)	(Ions score 64)
416 - 435	801.3648	2401.0727	2401.0721	0	1	K.MMDKVEFVHMLNATMCATTR.T	Oxidation (M)	(Ions score 50)
416 - 435	801.3648	2401.0727	2401.0721	0	1	K.MMDKVEFVHMLNATMCATTR.T	Oxidation (M)	(Ions score 47)
416 - 435	801.3649	2401.0728	2401.0721	0	1	K.MMDKVEFVHMLNATMCATTR.T	Oxidation (M)	(Ions score 35)
416 - 435	801.3649	2401.0730	2401.0721	0	1	K.MMDKVEFVHMLNATMCATTR.T	Oxidation (M)	(Ions score 37)
416 - 435	801.3651	2401.0733	2401.0721	1	1	K.MMDKVEFVHMLNATMCATTR.T	Oxidation (M)	(Ions score 44)
416 - 435	801.3651	2401.0734	2401.0721	1	1	K.MMDKVEFVHMLNATMCATTR.T	Oxidation (M)	(Ions score 31)
416 - 435	801.3651	2401.0735	2401.0721	1	1	K.MMDKVEFVHMLNATMCATTR.T	Oxidation (M)	(Ions score 58)
416 - 435	801.3651	2401.0736	2401.0721	1	1	K.MMDKVEFVHMLNATMCATTR.T	Oxidation (M)	(Ions score 88)

416 - 435	801.3652	2401.0737	2401.0721	1	1	K.MMDKVEFVHMLNATMCATTR.T	Oxidation (M)	(Ions score 48)
416 - 435	801.3653	2401.0741	2401.0721	1	1	K.MMDKVEFVHMLNATMCATTR.T	Oxidation (M)	(Ions score 38)
416 - 435	801.3653	2401.0741	2401.0721	1	1	K.MMDKVEFVHMLNATMCATTR.T	Oxidation (M)	(Ions score 45)
416 - 435	601.2758	2401.0742	2401.0721	1	1	K.MMDKVEFVHMLNATMCATTR.T	Oxidation (M)	(Ions score 25)
416 - 435	801.3654	2401.0743	2401.0721	1	1	K.MMDKVEFVHMLNATMCATTR.T	Oxidation (M)	(Ions score 76)
416 - 435	601.2758	2401.0743	2401.0721	1	1	K.MMDKVEFVHMLNATMCATTR.T	Oxidation (M)	(Ions score 23)
416 - 435	801.3654	2401.0743	2401.0721	1	1	K.MMDKVEFVHMLNATMCATTR.T	Oxidation (M)	(Ions score 61)
416 - 435	801.3654	2401.0743	2401.0721	1	1	K.MMDKVEFVHMLNATMCATTR.T	Oxidation (M)	(Ions score 47)
416 - 435	601.2759	2401.0745	2401.0721	1	1	K.MMDKVEFVHMLNATMCATTR.T	Oxidation (M)	(Ions score 22)
416 - 435	801.3655	2401.0746	2401.0721	1	1	K.MMDKVEFVHMLNATMCATTR.T	Oxidation (M)	(Ions score 36)
416 - 435	801.3655	2401.0748	2401.0721	1	1	K.MMDKVEFVHMLNATMCATTR.T	Oxidation (M)	(Ions score 27)
416 - 435	601.2760	2401.0748	2401.0721	1	1	K.MMDKVEFVHMLNATMCATTR.T	Oxidation (M)	(Ions score 28)
416 - 435	801.3656	2401.0750	2401.0721	1	1	K.MMDKVEFVHMLNATMCATTR.T	Oxidation (M)	(Ions score 50)
416 - 435	801.3656	2401.0750	2401.0721	1	1	K.MMDKVEFVHMLNATMCATTR.T	Oxidation (M)	(Ions score 41)
416 - 435	801.3657	2401.0752	2401.0721	1	1	K.MMDKVEFVHMLNATMCATTR.T	Oxidation (M)	(Ions score 45)
416 - 435	801.3657	2401.0752	2401.0721	1	1	K.MMDKVEFVHMLNATMCATTR.T	Oxidation (M)	(Ions score 42)
416 - 435	1201.5449	2401.0752	2401.0721	1	1	K.MMDKVEFVHMLNATMCATTR.T	Oxidation (M)	(Ions score 55)
416 - 435	1201.5449	2401.0752	2401.0721	1	1	K.MMDKVEFVHMLNATMCATTR.T	Oxidation (M)	(Ions score 96)
416 - 435	801.3658	2401.0755	2401.0721	1	1	K.MMDKVEFVHMLNATMCATTR.T	Oxidation (M)	(Ions score 37)
416 - 435	801.3658	2401.0755	2401.0721	1	1	K.MMDKVEFVHMLNATMCATTR.T	Oxidation (M)	(Ions score 61)
416 - 435	801.3659	2401.0758	2401.0721	2	1	K.MMDKVEFVHMLNATMCATTR.T	Oxidation (M)	(Ions score 32)
416 - 435	601.2762	2401.0758	2401.0721	2	1	K.MMDKVEFVHMLNATMCATTR.T	Oxidation (M)	(Ions score 22)
416 - 435	801.3659	2401.0758	2401.0721	2	1	K.MMDKVEFVHMLNATMCATTR.T	Oxidation (M)	(Ions score 37)
416 - 435	601.2762	2401.0758	2401.0721	2	1	K.MMDKVEFVHMLNATMCATTR.T	Oxidation (M)	(Ions score 22)
416 - 435	801.3659	2401.0758	2401.0721	2	1	K.MMDKVEFVHMLNATMCATTR.T	Oxidation (M)	(Ions score 35)
416 - 435	1201.5452	2401.0759	2401.0721	2	1	K.MMDKVEFVHMLNATMCATTR.T	Oxidation (M)	(Ions score 106)
416 - 435	1201.5452	2401.0759	2401.0721	2	1	K.MMDKVEFVHMLNATMCATTR.T	Oxidation (M)	(Ions score 82)
416 - 435	601.2763	2401.0760	2401.0721	2	1	K.MMDKVEFVHMLNATMCATTR.T	Oxidation (M)	(Ions score 23)
416 - 435	601.2763	2401.0762	2401.0721	2	1	K.MMDKVEFVHMLNATMCATTR.T	Oxidation (M)	(Ions score 22)
416 - 435	601.2763	2401.0763	2401.0721	2	1	K.MMDKVEFVHMLNATMCATTR.T	Oxidation (M)	(Ions score 23)
416 - 435	1201.5456	2401.0766	2401.0721	2	1	K.MMDKVEFVHMLNATMCATTR.T	Oxidation (M)	(Ions score 108)
416 - 435	801.3662	2401.0767	2401.0721	2	1	K.MMDKVEFVHMLNATMCATTR.T	Oxidation (M)	(Ions score 58)
416 - 435	801.3663	2401.0770	2401.0721	2	1	K.MMDKVEFVHMLNATMCATTR.T	Oxidation (M)	(Ions score 61)
416 - 435	801.3663	2401.0770	2401.0721	2	1	K.MMDKVEFVHMLNATMCATTR.T	Oxidation (M)	(Ions score 59)
416 - 435	601.2767	2401.0776	2401.0721	2	1	K.MMDKVEFVHMLNATMCATTR.T	Oxidation (M)	(Ions score 21)
416 - 435	801.3665	2401.0776	2401.0721	2	1	K.MMDKVEFVHMLNATMCATTR.T	Oxidation (M)	(Ions score 40)
416 - 435	801.3665	2401.0777	2401.0721	2	1	K.MMDKVEFVHMLNATMCATTR.T	Oxidation (M)	(Ions score 61)
416 - 435	1201.5461	2401.0777	2401.0721	2	1	K.MMDKVEFVHMLNATMCATTR.T	Oxidation (M)	(Ions score 93)
416 - 435	801.3666	2401.0779	2401.0721	2	1	K.MMDKVEFVHMLNATMCATTR.T	Oxidation (M)	(Ions score 52)
416 - 435	801.3667	2401.0782	2401.0721	3	1	K.MMDKVEFVHMLNATMCATTR.T	Oxidation (M)	(Ions score 45)
416 - 435	801.3667	2401.0783	2401.0721	3	1	K.MMDKVEFVHMLNATMCATTR.T	Oxidation (M)	(Ions score 50)
416 - 435	601.2769	2401.0786	2401.0721	3	1	K.MMDKVEFVHMLNATMCATTR.T	Oxidation (M)	(Ions score 26)
416 - 435	801.3670	2401.0791	2401.0721	3	1	K.MMDKVEFVHMLNATMCATTR.T	Oxidation (M)	(Ions score 58)
416 - 435	801.3671	2401.0794	2401.0721	3	1	K.MMDKVEFVHMLNATMCATTR.T	Oxidation (M)	(Ions score 48)
416 - 435	601.2772	2401.0796	2401.0721	3	1	K.MMDKVEFVHMLNATMCATTR.T	Oxidation (M)	(Ions score 22)
416 - 435	1201.5472	2401.0798	2401.0721	3	1	K.MMDKVEFVHMLNATMCATTR.T	Oxidation (M)	(Ions score 81)
416 - 435	801.3672	2401.0799	2401.0721	3	1	K.MMDKVEFVHMLNATMCATTR.T	Oxidation (M)	(Ions score 36)
416 - 435	801.3673	2401.0800	2401.0721	3	1	K.MMDKVEFVHMLNATMCATTR.T	Oxidation (M)	(Ions score 45)
416 - 435	801.3673	2401.0800	2401.0721	3	1	K.MMDKVEFVHMLNATMCATTR.T	Oxidation (M)	(Ions score 51)
416 - 435	801.3675	2401.0807	2401.0721	4	1	K.MMDKVEFVHMLNATMCATTR.T	Oxidation (M)	(Ions score 38)
416 - 435	801.3676	2401.0809	2401.0721	4	1	K.MMDKVEFVHMLNATMCATTR.T	Oxidation (M)	(Ions score 48)
416 - 435	601.2775	2401.0810	2401.0721	4	1	K.MMDKVEFVHMLNATMCATTR.T	Oxidation (M)	(Ions score 21)
416 - 435	801.3676	2401.0810	2401.0721	4	1	K.MMDKVEFVHMLNATMCATTR.T	Oxidation (M)	(Ions score 47)
416 - 435	801.3677	2401.0814	2401.0721	4	1	K.MMDKVEFVHMLNATMCATTR.T	Oxidation (M)	(Ions score 39)
416 - 435	601.2777	2401.0816	2401.0721	4	1	K.MMDKVEFVHMLNATMCATTR.T	Oxidation (M)	(Ions score 32)
416 - 435	801.3678	2401.0816	2401.0721	4	1	K.MMDKVEFVHMLNATMCATTR.T	Oxidation (M)	(Ions score 37)
416 - 435	601.2777	2401.0817	2401.0721	4	1	K.MMDKVEFVHMLNATMCATTR.T	Oxidation (M)	(Ions score 21)
416 - 435	601.2779	2401.0823	2401.0721	4	1	K.MMDKVEFVHMLNATMCATTR.T	Oxidation (M)	(Ions score 23)

416 - 435	601.2780	2401.0830	2401.0721	5	1	K.MMDKVEFVHMLNATMCATTR.T	Oxidation (M) (Ions score 30)
416 - 435	801.3683	2401.0831	2401.0721	5	1	K.MMDKVEFVHMLNATMCATTR.T	Oxidation (M) (Ions score 25)
416 - 435	806.6938	2417.0596	2417.0670	-3	1	K.MMDKVEFVHMLNATMCATTR.T	2 Oxidation (M) (Ions score 45)
416 - 435	1209.5374	2417.0602	2417.0670	-3	1	K.MMDKVEFVHMLNATMCATTR.T	2 Oxidation (M) (Ions score 59)
416 - 435	1209.5374	2417.0602	2417.0670	-3	1	K.MMDKVEFVHMLNATMCATTR.T	2 Oxidation (M) (Ions score 79)
416 - 435	806.6944	2417.0614	2417.0670	-2	1	K.MMDKVEFVHMLNATMCATTR.T	2 Oxidation (M) (Ions score 35)
416 - 435	1209.5389	2417.0633	2417.0670	-2	1	K.MMDKVEFVHMLNATMCATTR.T	2 Oxidation (M) (Ions score 34)
416 - 435	605.2732	2417.0635	2417.0670	-1	1	K.MMDKVEFVHMLNATMCATTR.T	2 Oxidation (M) (Ions score 21)
416 - 435	806.6951	2417.0636	2417.0670	-1	1	K.MMDKVEFVHMLNATMCATTR.T	2 Oxidation (M) (Ions score 45)
416 - 435	806.6952	2417.0639	2417.0670	-1	1	K.MMDKVEFVHMLNATMCATTR.T	2 Oxidation (M) (Ions score 25)
416 - 435	806.6954	2417.0645	2417.0670	-1	1	K.MMDKVEFVHMLNATMCATTR.T	2 Oxidation (M) (Ions score 38)
416 - 435	806.6955	2417.0647	2417.0670	-1	1	K.MMDKVEFVHMLNATMCATTR.T	2 Oxidation (M) (Ions score 58)
416 - 435	806.6956	2417.0648	2417.0670	-1	1	K.MMDKVEFVHMLNATMCATTR.T	2 Oxidation (M) (Ions score 59)
416 - 435	806.6956	2417.0649	2417.0670	-1	1	K.MMDKVEFVHMLNATMCATTR.T	2 Oxidation (M) (Ions score 35)
416 - 435	605.2735	2417.0649	2417.0670	-1	1	K.MMDKVEFVHMLNATMCATTR.T	2 Oxidation (M) (Ions score 22)
416 - 435	806.6956	2417.0650	2417.0670	-1	1	K.MMDKVEFVHMLNATMCATTR.T	2 Oxidation (M) (Ions score 48)
416 - 435	806.6957	2417.0652	2417.0670	-1	1	K.MMDKVEFVHMLNATMCATTR.T	2 Oxidation (M) (Ions score 49)
416 - 435	806.6957	2417.0652	2417.0670	-1	1	K.MMDKVEFVHMLNATMCATTR.T	2 Oxidation (M) (Ions score 35)
416 - 435	806.6957	2417.0653	2417.0670	-1	1	K.MMDKVEFVHMLNATMCATTR.T	2 Oxidation (M) (Ions score 45)
416 - 435	806.6957	2417.0654	2417.0670	-1	1	K.MMDKVEFVHMLNATMCATTR.T	2 Oxidation (M) (Ions score 49)
416 - 435	806.6958	2417.0656	2417.0670	-1	1	K.MMDKVEFVHMLNATMCATTR.T	2 Oxidation (M) (Ions score 63)
416 - 435	806.6958	2417.0657	2417.0670	-1	1	K.MMDKVEFVHMLNATMCATTR.T	2 Oxidation (M) (Ions score 48)
416 - 435	806.6959	2417.0658	2417.0670	-1	1	K.MMDKVEFVHMLNATMCATTR.T	2 Oxidation (M) (Ions score 45)
416 - 435	806.6960	2417.0663	2417.0670	-0	1	K.MMDKVEFVHMLNATMCATTR.T	2 Oxidation (M) (Ions score 37)
416 - 435	806.6961	2417.0665	2417.0670	-0	1	K.MMDKVEFVHMLNATMCATTR.T	2 Oxidation (M) (Ions score 32)
416 - 435	806.6961	2417.0665	2417.0670	-0	1	K.MMDKVEFVHMLNATMCATTR.T	2 Oxidation (M) (Ions score 59)
416 - 435	806.6962	2417.0667	2417.0670	-0	1	K.MMDKVEFVHMLNATMCATTR.T	2 Oxidation (M) (Ions score 47)
416 - 435	806.6963	2417.0670	2417.0670	-0	1	K.MMDKVEFVHMLNATMCATTR.T	2 Oxidation (M) (Ions score 43)
416 - 435	806.6963	2417.0670	2417.0670	0	1	K.MMDKVEFVHMLNATMCATTR.T	2 Oxidation (M) (Ions score 28)
416 - 435	806.6964	2417.0672	2417.0670	0	1	K.MMDKVEFVHMLNATMCATTR.T	2 Oxidation (M) (Ions score 75)
416 - 435	605.2741	2417.0673	2417.0670	0	1	K.MMDKVEFVHMLNATMCATTR.T	2 Oxidation (M) (Ions score 22)
416 - 435	806.6964	2417.0674	2417.0670	0	1	K.MMDKVEFVHMLNATMCATTR.T	2 Oxidation (M) (Ions score 42)
416 - 435	806.6965	2417.0676	2417.0670	0	1	K.MMDKVEFVHMLNATMCATTR.T	2 Oxidation (M) (Ions score 60)
416 - 435	806.6966	2417.0678	2417.0670	0	1	K.MMDKVEFVHMLNATMCATTR.T	2 Oxidation (M) (Ions score 25)
416 - 435	605.2743	2417.0680	2417.0670	0	1	K.MMDKVEFVHMLNATMCATTR.T	2 Oxidation (M) (Ions score 25)
416 - 435	1209.5414	2417.0683	2417.0670	1	1	K.MMDKVEFVHMLNATMCATTR.T	2 Oxidation (M) (Ions score 94)
416 - 435	806.6967	2417.0683	2417.0670	1	1	K.MMDKVEFVHMLNATMCATTR.T	2 Oxidation (M) (Ions score 47)
416 - 435	806.6968	2417.0684	2417.0670	1	1	K.MMDKVEFVHMLNATMCATTR.T	2 Oxidation (M) (Ions score 50)
416 - 435	806.6968	2417.0685	2417.0670	1	1	K.MMDKVEFVHMLNATMCATTR.T	2 Oxidation (M) (Ions score 22)
416 - 435	806.6968	2417.0685	2417.0670	1	1	K.MMDKVEFVHMLNATMCATTR.T	2 Oxidation (M) (Ions score 43)
416 - 435	806.6968	2417.0685	2417.0670	1	1	K.MMDKVEFVHMLNATMCATTR.T	2 Oxidation (M) (Ions score 52)
416 - 435	605.2745	2417.0688	2417.0670	1	1	K.MMDKVEFVHMLNATMCATTR.T	2 Oxidation (M) (Ions score 22)
416 - 435	806.6969	2417.0689	2417.0670	1	1	K.MMDKVEFVHMLNATMCATTR.T	2 Oxidation (M) (Ions score 56)
416 - 435	806.6969	2417.0689	2417.0670	1	1	K.MMDKVEFVHMLNATMCATTR.T	2 Oxidation (M) (Ions score 56)
416 - 435	605.2745	2417.0689	2417.0670	1	1	K.MMDKVEFVHMLNATMCATTR.T	2 Oxidation (M) (Ions score 27)
416 - 435	806.6969	2417.0690	2417.0670	1	1	K.MMDKVEFVHMLNATMCATTR.T	2 Oxidation (M) (Ions score 66)
416 - 435	1209.5418	2417.0690	2417.0670	1	1	K.MMDKVEFVHMLNATMCATTR.T	2 Oxidation (M) (Ions score 50)
416 - 435	806.6969	2417.0690	2417.0670	1	1	K.MMDKVEFVHMLNATMCATTR.T	2 Oxidation (M) (Ions score 53)
416 - 435	605.2745	2417.0690	2417.0670	1	1	K.MMDKVEFVHMLNATMCATTR.T	2 Oxidation (M) (Ions score 27)
416 - 435	806.6970	2417.0691	2417.0670	1	1	K.MMDKVEFVHMLNATMCATTR.T	2 Oxidation (M) (Ions score 47)
416 - 435	806.6970	2417.0693	2417.0670	1	1	K.MMDKVEFVHMLNATMCATTR.T	2 Oxidation (M) (Ions score 66)
416 - 435	806.6971	2417.0694	2417.0670	1	1	K.MMDKVEFVHMLNATMCATTR.T	2 Oxidation (M) (Ions score 59)
416 - 435	806.6971	2417.0696	2417.0670	1	1	K.MMDKVEFVHMLNATMCATTR.T	2 Oxidation (M) (Ions score 33)
416 - 435	1209.5421	2417.0696	2417.0670	1	1	K.MMDKVEFVHMLNATMCATTR.T	2 Oxidation (M) (Ions score 83)
416 - 435	1209.5421	2417.0697	2417.0670	1	1	K.MMDKVEFVHMLNATMCATTR.T	2 Oxidation (M) (Ions score 62)
416 - 435	1209.5421	2417.0697	2417.0670	1	1	K.MMDKVEFVHMLNATMCATTR.T	2 Oxidation (M) (Ions score 51)
416 - 435	806.6972	2417.0697	2417.0670	1	1	K.MMDKVEFVHMLNATMCATTR.T	2 Oxidation (M) (Ions score 52)
416 - 435	806.6972	2417.0698	2417.0670	1	1	K.MMDKVEFVHMLNATMCATTR.T	2 Oxidation (M) (Ions score 33)

416 - 435	806.6972	2417.0699	2417.0670	1	1	K.MMDKVEFVHMLNATMCATTR.T	2 Oxidation (M)	(Ions score 24)
416 - 435	806.6973	2417.0700	2417.0670	1	1	K.MMDKVEFVHMLNATMCATTR.T	2 Oxidation (M)	(Ions score 44)
416 - 435	806.6973	2417.0700	2417.0670	1	1	K.MMDKVEFVHMLNATMCATTR.T	2 Oxidation (M)	(Ions score 36)
416 - 435	806.6973	2417.0700	2417.0670	1	1	K.MMDKVEFVHMLNATMCATTR.T	2 Oxidation (M)	(Ions score 39)
416 - 435	806.6973	2417.0700	2417.0670	1	1	K.MMDKVEFVHMLNATMCATTR.T	2 Oxidation (M)	(Ions score 39)
416 - 435	806.6973	2417.0700	2417.0670	1	1	K.MMDKVEFVHMLNATMCATTR.T	2 Oxidation (M)	(Ions score 38)
416 - 435	1209.5423	2417.0700	2417.0670	1	1	K.MMDKVEFVHMLNATMCATTR.T	2 Oxidation (M)	(Ions score 70)
416 - 435	806.6973	2417.0702	2417.0670	1	1	K.MMDKVEFVHMLNATMCATTR.T	2 Oxidation (M)	(Ions score 37)
416 - 435	605.2748	2417.0702	2417.0670	1	1	K.MMDKVEFVHMLNATMCATTR.T	2 Oxidation (M)	(Ions score 22)
416 - 435	806.6974	2417.0703	2417.0670	1	1	K.MMDKVEFVHMLNATMCATTR.T	2 Oxidation (M)	(Ions score 25)
416 - 435	806.6974	2417.0703	2417.0670	1	1	K.MMDKVEFVHMLNATMCATTR.T	2 Oxidation (M)	(Ions score 35)
416 - 435	806.6974	2417.0704	2417.0670	1	1	K.MMDKVEFVHMLNATMCATTR.T	2 Oxidation (M)	(Ions score 39)
416 - 435	1209.5425	2417.0705	2417.0670	1	1	K.MMDKVEFVHMLNATMCATTR.T	2 Oxidation (M)	(Ions score 65)
416 - 435	806.6975	2417.0707	2417.0670	2	1	K.MMDKVEFVHMLNATMCATTR.T	2 Oxidation (M)	(Ions score 24)
416 - 435	806.6975	2417.0707	2417.0670	2	1	K.MMDKVEFVHMLNATMCATTR.T	2 Oxidation (M)	(Ions score 58)
416 - 435	605.2750	2417.0707	2417.0670	2	1	K.MMDKVEFVHMLNATMCATTR.T	2 Oxidation (M)	(Ions score 27)
416 - 435	806.6976	2417.0711	2417.0670	2	1	K.MMDKVEFVHMLNATMCATTR.T	2 Oxidation (M)	(Ions score 40)
416 - 435	806.6976	2417.0711	2417.0670	2	1	K.MMDKVEFVHMLNATMCATTR.T	2 Oxidation (M)	(Ions score 33)
416 - 435	806.6976	2417.0711	2417.0670	2	1	K.MMDKVEFVHMLNATMCATTR.T	2 Oxidation (M)	(Ions score 27)
416 - 435	806.6976	2417.0711	2417.0670	2	1	K.MMDKVEFVHMLNATMCATTR.T	2 Oxidation (M)	(Ions score 49)
416 - 435	806.6977	2417.0711	2417.0670	2	1	K.MMDKVEFVHMLNATMCATTR.T	2 Oxidation (M)	(Ions score 40)
416 - 435	806.6977	2417.0713	2417.0670	2	1	K.MMDKVEFVHMLNATMCATTR.T	2 Oxidation (M)	(Ions score 42)
416 - 435	806.6977	2417.0713	2417.0670	2	1	K.MMDKVEFVHMLNATMCATTR.T	2 Oxidation (M)	(Ions score 26)
416 - 435	806.6977	2417.0713	2417.0670	2	1	K.MMDKVEFVHMLNATMCATTR.T	2 Oxidation (M)	(Ions score 39)
416 - 435	806.6977	2417.0713	2417.0670	2	1	K.MMDKVEFVHMLNATMCATTR.T	2 Oxidation (M)	(Ions score 50)
416 - 435	605.2751	2417.0713	2417.0670	2	1	K.MMDKVEFVHMLNATMCATTR.T	2 Oxidation (M)	(Ions score 22)
416 - 435	1209.5429	2417.0713	2417.0670	2	1	K.MMDKVEFVHMLNATMCATTR.T	2 Oxidation (M)	(Ions score 42)
416 - 435	806.6978	2417.0714	2417.0670	2	1	K.MMDKVEFVHMLNATMCATTR.T	2 Oxidation (M)	(Ions score 36)
416 - 435	806.6978	2417.0714	2417.0670	2	1	K.MMDKVEFVHMLNATMCATTR.T	2 Oxidation (M)	(Ions score 37)
416 - 435	806.6978	2417.0714	2417.0670	2	1	K.MMDKVEFVHMLNATMCATTR.T	2 Oxidation (M)	(Ions score 45)
416 - 435	605.2751	2417.0714	2417.0670	2	1	K.MMDKVEFVHMLNATMCATTR.T	2 Oxidation (M)	(Ions score 22)
416 - 435	806.6978	2417.0715	2417.0670	2	1	K.MMDKVEFVHMLNATMCATTR.T	2 Oxidation (M)	(Ions score 56)
416 - 435	806.6978	2417.0715	2417.0670	2	1	K.MMDKVEFVHMLNATMCATTR.T	2 Oxidation (M)	(Ions score 42)
416 - 435	806.6978	2417.0716	2417.0670	2	1	K.MMDKVEFVHMLNATMCATTR.T	2 Oxidation (M)	(Ions score 26)
416 - 435	806.6978	2417.0717	2417.0670	2	1	K.MMDKVEFVHMLNATMCATTR.T	2 Oxidation (M)	(Ions score 64)
416 - 435	1209.5431	2417.0717	2417.0670	2	1	K.MMDKVEFVHMLNATMCATTR.T	2 Oxidation (M)	(Ions score 57)
416 - 435	806.6979	2417.0718	2417.0670	2	1	K.MMDKVEFVHMLNATMCATTR.T	2 Oxidation (M)	(Ions score 39)
416 - 435	806.6979	2417.0718	2417.0670	2	1	K.MMDKVEFVHMLNATMCATTR.T	2 Oxidation (M)	(Ions score 26)
416 - 435	605.2753	2417.0719	2417.0670	2	1	K.MMDKVEFVHMLNATMCATTR.T	2 Oxidation (M)	(Ions score 28)
416 - 435	605.2753	2417.0720	2417.0670	2	1	K.MMDKVEFVHMLNATMCATTR.T	2 Oxidation (M)	(Ions score 24)
416 - 435	806.6979	2417.0720	2417.0670	2	1	K.MMDKVEFVHMLNATMCATTR.T	2 Oxidation (M)	(Ions score 23)
416 - 435	806.6979	2417.0720	2417.0670	2	1	K.MMDKVEFVHMLNATMCATTR.T	2 Oxidation (M)	(Ions score 60)
416 - 435	806.6980	2417.0720	2417.0670	2	1	K.MMDKVEFVHMLNATMCATTR.T	2 Oxidation (M)	(Ions score 38)
416 - 435	806.6980	2417.0721	2417.0670	2	1	K.MMDKVEFVHMLNATMCATTR.T	2 Oxidation (M)	(Ions score 54)
416 - 435	806.6980	2417.0721	2417.0670	2	1	K.MMDKVEFVHMLNATMCATTR.T	2 Oxidation (M)	(Ions score 48)
416 - 435	806.6980	2417.0722	2417.0670	2	1	K.MMDKVEFVHMLNATMCATTR.T	2 Oxidation (M)	(Ions score 54)
416 - 435	605.2753	2417.0722	2417.0670	2	1	K.MMDKVEFVHMLNATMCATTR.T	2 Oxidation (M)	(Ions score 27)
416 - 435	605.2753	2417.0722	2417.0670	2	1	K.MMDKVEFVHMLNATMCATTR.T	2 Oxidation (M)	(Ions score 21)
416 - 435	605.2754	2417.0724	2417.0670	2	1	K.MMDKVEFVHMLNATMCATTR.T	2 Oxidation (M)	(Ions score 22)
416 - 435	605.2754	2417.0725	2417.0670	2	1	K.MMDKVEFVHMLNATMCATTR.T	2 Oxidation (M)	(Ions score 26)
416 - 435	806.6981	2417.0725	2417.0670	2	1	K.MMDKVEFVHMLNATMCATTR.T	2 Oxidation (M)	(Ions score 49)
416 - 435	605.2754	2417.0727	2417.0670	2	1	K.MMDKVEFVHMLNATMCATTR.T	2 Oxidation (M)	(Ions score 23)
416 - 435	605.2755	2417.0727	2417.0670	2	1	K.MMDKVEFVHMLNATMCATTR.T	2 Oxidation (M)	(Ions score 22)
416 - 435	806.6982	2417.0727	2417.0670	2	1	K.MMDKVEFVHMLNATMCATTR.T	2 Oxidation (M)	(Ions score 46)
416 - 435	806.6982	2417.0728	2417.0670	2	1	K.MMDKVEFVHMLNATMCATTR.T	2 Oxidation (M)	(Ions score 67)
416 - 435	605.2755	2417.0729	2417.0670	2	1	K.MMDKVEFVHMLNATMCATTR.T	2 Oxidation (M)	(Ions score 23)
416 - 435	605.2755	2417.0729	2417.0670	2	1	K.MMDKVEFVHMLNATMCATTR.T	2 Oxidation (M)	(Ions score 22)
416 - 435	1209.5438	2417.0730	2417.0670	2	1	K.MMDKVEFVHMLNATMCATTR.T	2 Oxidation (M)	(Ions score 64)

416 - 435	605.2755	2417.0730	2417.0670	2	1	K.MMDKVEFVHMLNATMCATTR.T	2 Oxidation (M)	(Ions score 24)
416 - 435	806.6983	2417.0731	2417.0670	3	1	K.MMDKVEFVHMLNATMCATTR.T	2 Oxidation (M)	(Ions score 23)
416 - 435	806.6983	2417.0732	2417.0670	3	1	K.MMDKVEFVHMLNATMCATTR.T	2 Oxidation (M)	(Ions score 36)
416 - 435	1209.5439	2417.0732	2417.0670	3	1	K.MMDKVEFVHMLNATMCATTR.T	2 Oxidation (M)	(Ions score 90)
416 - 435	605.2756	2417.0732	2417.0670	3	1	K.MMDKVEFVHMLNATMCATTR.T	2 Oxidation (M)	(Ions score 23)
416 - 435	806.6984	2417.0733	2417.0670	3	1	K.MMDKVEFVHMLNATMCATTR.T	2 Oxidation (M)	(Ions score 30)
416 - 435	806.6984	2417.0733	2417.0670	3	1	K.MMDKVEFVHMLNATMCATTR.T	2 Oxidation (M)	(Ions score 46)
416 - 435	806.6984	2417.0734	2417.0670	3	1	K.MMDKVEFVHMLNATMCATTR.T	2 Oxidation (M)	(Ions score 45)
416 - 435	806.6984	2417.0734	2417.0670	3	1	K.MMDKVEFVHMLNATMCATTR.T	2 Oxidation (M)	(Ions score 34)
416 - 435	605.2757	2417.0736	2417.0670	3	1	K.MMDKVEFVHMLNATMCATTR.T	2 Oxidation (M)	(Ions score 21)
416 - 435	806.6985	2417.0736	2417.0670	3	1	K.MMDKVEFVHMLNATMCATTR.T	2 Oxidation (M)	(Ions score 54)
416 - 435	806.6985	2417.0736	2417.0670	3	1	K.MMDKVEFVHMLNATMCATTR.T	2 Oxidation (M)	(Ions score 33)
416 - 435	605.2757	2417.0737	2417.0670	3	1	K.MMDKVEFVHMLNATMCATTR.T	2 Oxidation (M)	(Ions score 31)
416 - 435	1209.5441	2417.0737	2417.0670	3	1	K.MMDKVEFVHMLNATMCATTR.T	2 Oxidation (M)	(Ions score 74)
416 - 435	806.6985	2417.0738	2417.0670	3	1	K.MMDKVEFVHMLNATMCATTR.T	2 Oxidation (M)	(Ions score 40)
416 - 435	806.6985	2417.0738	2417.0670	3	1	K.MMDKVEFVHMLNATMCATTR.T	2 Oxidation (M)	(Ions score 22)
416 - 435	806.6985	2417.0738	2417.0670	3	1	K.MMDKVEFVHMLNATMCATTR.T	2 Oxidation (M)	(Ions score 24)
416 - 435	605.2758	2417.0739	2417.0670	3	1	K.MMDKVEFVHMLNATMCATTR.T	2 Oxidation (M)	(Ions score 23)
416 - 435	806.6986	2417.0740	2417.0670	3	1	K.MMDKVEFVHMLNATMCATTR.T	2 Oxidation (M)	(Ions score 31)
416 - 435	806.6986	2417.0740	2417.0670	3	1	K.MMDKVEFVHMLNATMCATTR.T	2 Oxidation (M)	(Ions score 46)
416 - 435	806.6986	2417.0741	2417.0670	3	1	K.MMDKVEFVHMLNATMCATTR.T	2 Oxidation (M)	(Ions score 63)
416 - 435	1209.5443	2417.0741	2417.0670	3	1	K.MMDKVEFVHMLNATMCATTR.T	2 Oxidation (M)	(Ions score 66)
416 - 435	806.6987	2417.0742	2417.0670	3	1	K.MMDKVEFVHMLNATMCATTR.T	2 Oxidation (M)	(Ions score 33)
416 - 435	806.6987	2417.0742	2417.0670	3	1	K.MMDKVEFVHMLNATMCATTR.T	2 Oxidation (M)	(Ions score 43)
416 - 435	605.2759	2417.0743	2417.0670	3	1	K.MMDKVEFVHMLNATMCATTR.T	2 Oxidation (M)	(Ions score 24)
416 - 435	605.2759	2417.0744	2417.0670	3	1	K.MMDKVEFVHMLNATMCATTR.T	2 Oxidation (M)	(Ions score 23)
416 - 435	605.2759	2417.0744	2417.0670	3	1	K.MMDKVEFVHMLNATMCATTR.T	2 Oxidation (M)	(Ions score 24)
416 - 435	806.6988	2417.0745	2417.0670	3	1	K.MMDKVEFVHMLNATMCATTR.T	2 Oxidation (M)	(Ions score 41)
416 - 435	605.2759	2417.0745	2417.0670	3	1	K.MMDKVEFVHMLNATMCATTR.T	2 Oxidation (M)	(Ions score 22)
416 - 435	1209.5446	2417.0746	2417.0670	3	1	K.MMDKVEFVHMLNATMCATTR.T	2 Oxidation (M)	(Ions score 32)
416 - 435	806.6989	2417.0747	2417.0670	3	1	K.MMDKVEFVHMLNATMCATTR.T	2 Oxidation (M)	(Ions score 34)
416 - 435	806.6989	2417.0747	2417.0670	3	1	K.MMDKVEFVHMLNATMCATTR.T	2 Oxidation (M)	(Ions score 33)
416 - 435	605.2760	2417.0748	2417.0670	3	1	K.MMDKVEFVHMLNATMCATTR.T	2 Oxidation (M)	(Ions score 23)
416 - 435	1209.5447	2417.0749	2417.0670	3	1	K.MMDKVEFVHMLNATMCATTR.T	2 Oxidation (M)	(Ions score 83)
416 - 435	806.6989	2417.0749	2417.0670	3	1	K.MMDKVEFVHMLNATMCATTR.T	2 Oxidation (M)	(Ions score 34)
416 - 435	806.6990	2417.0751	2417.0670	3	1	K.MMDKVEFVHMLNATMCATTR.T	2 Oxidation (M)	(Ions score 39)
416 - 435	1209.5449	2417.0753	2417.0670	3	1	K.MMDKVEFVHMLNATMCATTR.T	2 Oxidation (M)	(Ions score 65)
416 - 435	605.2761	2417.0754	2417.0670	3	1	K.MMDKVEFVHMLNATMCATTR.T	2 Oxidation (M)	(Ions score 22)
416 - 435	806.6991	2417.0754	2417.0670	3	1	K.MMDKVEFVHMLNATMCATTR.T	2 Oxidation (M)	(Ions score 57)
416 - 435	605.2761	2417.0754	2417.0670	3	1	K.MMDKVEFVHMLNATMCATTR.T	2 Oxidation (M)	(Ions score 25)
416 - 435	605.2762	2417.0755	2417.0670	4	1	K.MMDKVEFVHMLNATMCATTR.T	2 Oxidation (M)	(Ions score 21)
416 - 435	605.2762	2417.0755	2417.0670	4	1	K.MMDKVEFVHMLNATMCATTR.T	2 Oxidation (M)	(Ions score 23)
416 - 435	605.2762	2417.0756	2417.0670	4	1	K.MMDKVEFVHMLNATMCATTR.T	2 Oxidation (M)	(Ions score 23)
416 - 435	806.6991	2417.0756	2417.0670	4	1	K.MMDKVEFVHMLNATMCATTR.T	2 Oxidation (M)	(Ions score 55)
416 - 435	806.6992	2417.0756	2417.0670	4	1	K.MMDKVEFVHMLNATMCATTR.T	2 Oxidation (M)	(Ions score 32)
416 - 435	605.2762	2417.0758	2417.0670	4	1	K.MMDKVEFVHMLNATMCATTR.T	2 Oxidation (M)	(Ions score 23)
416 - 435	605.2762	2417.0758	2417.0670	4	1	K.MMDKVEFVHMLNATMCATTR.T	2 Oxidation (M)	(Ions score 23)
416 - 435	1209.5452	2417.0758	2417.0670	4	1	K.MMDKVEFVHMLNATMCATTR.T	2 Oxidation (M)	(Ions score 111)
416 - 435	1209.5452	2417.0758	2417.0670	4	1	K.MMDKVEFVHMLNATMCATTR.T	2 Oxidation (M)	(Ions score 58)
416 - 435	605.2762	2417.0759	2417.0670	4	1	K.MMDKVEFVHMLNATMCATTR.T	2 Oxidation (M)	(Ions score 27)
416 - 435	605.2763	2417.0760	2417.0670	4	1	K.MMDKVEFVHMLNATMCATTR.T	2 Oxidation (M)	(Ions score 25)
416 - 435	605.2763	2417.0760	2417.0670	4	1	K.MMDKVEFVHMLNATMCATTR.T	2 Oxidation (M)	(Ions score 22)
416 - 435	605.2763	2417.0761	2417.0670	4	1	K.MMDKVEFVHMLNATMCATTR.T	2 Oxidation (M)	(Ions score 23)
416 - 435	605.2763	2417.0761	2417.0670	4	1	K.MMDKVEFVHMLNATMCATTR.T	2 Oxidation (M)	(Ions score 26)
416 - 435	806.6994	2417.0763	2417.0670	4	1	K.MMDKVEFVHMLNATMCATTR.T	2 Oxidation (M)	(Ions score 30)
416 - 435	605.2764	2417.0764	2417.0670	4	1	K.MMDKVEFVHMLNATMCATTR.T	2 Oxidation (M)	(Ions score 26)
416 - 435	806.6994	2417.0764	2417.0670	4	1	K.MMDKVEFVHMLNATMCATTR.T	2 Oxidation (M)	(Ions score 33)
416 - 435	605.2764	2417.0765	2417.0670	4	1	K.MMDKVEFVHMLNATMCATTR.T	2 Oxidation (M)	(Ions score 23)

416 - 435	605.2764	2417.0765	2417.0670	4	1	K.MMDKVEFVHMLNATMCATTR.T	2 Oxidation (M)	(Ions score 21)
416 - 435	605.2765	2417.0770	2417.0670	4	1	K.MMDKVEFVHMLNATMCATTR.T	2 Oxidation (M)	(Ions score 23)
416 - 435	806.6996	2417.0771	2417.0670	4	1	K.MMDKVEFVHMLNATMCATTR.T	2 Oxidation (M)	(Ions score 35)
416 - 435	605.2766	2417.0773	2417.0670	4	1	K.MMDKVEFVHMLNATMCATTR.T	2 Oxidation (M)	(Ions score 26)
416 - 435	806.6997	2417.0773	2417.0670	4	1	K.MMDKVEFVHMLNATMCATTR.T	2 Oxidation (M)	(Ions score 46)
416 - 435	605.2768	2417.0782	2417.0670	5	1	K.MMDKVEFVHMLNATMCATTR.T	2 Oxidation (M)	(Ions score 25)
416 - 435	806.7001	2417.0784	2417.0670	5	1	K.MMDKVEFVHMLNATMCATTR.T	2 Oxidation (M)	(Ions score 38)
416 - 435	806.7001	2417.0786	2417.0670	5	1	K.MMDKVEFVHMLNATMCATTR.T	2 Oxidation (M)	(Ions score 37)
416 - 435	806.7002	2417.0786	2417.0670	5	1	K.MMDKVEFVHMLNATMCATTR.T	2 Oxidation (M)	(Ions score 29)
416 - 435	607.7765	2427.0769	2427.0877	-4	1	K.MMDKVEFVHMLNATMCATTR.T	Acetylation (K)	(Ions score 30)
416 - 435	812.0250	2433.0531	2433.0619	-4	1	K.MMDKVEFVHMLNATMCATTR.T	3 Oxidation (M)	(Ions score 25)
416 - 435	609.2706	2433.0532	2433.0619	-4	1	K.MMDKVEFVHMLNATMCATTR.T	3 Oxidation (M)	(Ions score 26)
416 - 435	812.0254	2433.0542	2433.0619	-3	1	K.MMDKVEFVHMLNATMCATTR.T	3 Oxidation (M)	(Ions score 30)
416 - 435	812.0255	2433.0547	2433.0619	-3	1	K.MMDKVEFVHMLNATMCATTR.T	3 Oxidation (M)	(Ions score 30)
416 - 435	609.2711	2433.0551	2433.0619	-3	1	K.MMDKVEFVHMLNATMCATTR.T	3 Oxidation (M)	(Ions score 22)
416 - 435	812.0264	2433.0572	2433.0619	-2	1	K.MMDKVEFVHMLNATMCATTR.T	3 Oxidation (M)	(Ions score 35)
416 - 435	812.0264	2433.0574	2433.0619	-2	1	K.MMDKVEFVHMLNATMCATTR.T	3 Oxidation (M)	(Ions score 24)
416 - 435	812.0264	2433.0575	2433.0619	-2	1	K.MMDKVEFVHMLNATMCATTR.T	3 Oxidation (M)	(Ions score 32)
416 - 435	812.0266	2433.0578	2433.0619	-2	1	K.MMDKVEFVHMLNATMCATTR.T	3 Oxidation (M)	(Ions score 23)
416 - 435	1217.5364	2433.0583	2433.0619	-1	1	K.MMDKVEFVHMLNATMCATTR.T	3 Oxidation (M)	(Ions score 36)
416 - 435	812.0267	2433.0584	2433.0619	-1	1	K.MMDKVEFVHMLNATMCATTR.T	3 Oxidation (M)	(Ions score 22)
416 - 435	812.0268	2433.0585	2433.0619	-1	1	K.MMDKVEFVHMLNATMCATTR.T	3 Oxidation (M)	(Ions score 35)
416 - 435	609.2720	2433.0588	2433.0619	-1	1	K.MMDKVEFVHMLNATMCATTR.T	3 Oxidation (M)	(Ions score 29)
416 - 435	812.0270	2433.0592	2433.0619	-1	1	K.MMDKVEFVHMLNATMCATTR.T	3 Oxidation (M)	(Ions score 45)
416 - 435	812.0270	2433.0593	2433.0619	-1	1	K.MMDKVEFVHMLNATMCATTR.T	3 Oxidation (M)	(Ions score 37)
416 - 435	812.0271	2433.0593	2433.0619	-1	1	K.MMDKVEFVHMLNATMCATTR.T	3 Oxidation (M)	(Ions score 35)
416 - 435	812.0271	2433.0595	2433.0619	-1	1	K.MMDKVEFVHMLNATMCATTR.T	3 Oxidation (M)	(Ions score 33)
416 - 435	812.0271	2433.0595	2433.0619	-1	1	K.MMDKVEFVHMLNATMCATTR.T	3 Oxidation (M)	(Ions score 22)
416 - 435	812.0272	2433.0597	2433.0619	-1	1	K.MMDKVEFVHMLNATMCATTR.T	3 Oxidation (M)	(Ions score 44)
416 - 435	812.0273	2433.0599	2433.0619	-1	1	K.MMDKVEFVHMLNATMCATTR.T	3 Oxidation (M)	(Ions score 26)
416 - 435	812.0273	2433.0600	2433.0619	-1	1	K.MMDKVEFVHMLNATMCATTR.T	3 Oxidation (M)	(Ions score 29)
416 - 435	609.2723	2433.0601	2433.0619	-1	1	K.MMDKVEFVHMLNATMCATTR.T	3 Oxidation (M)	(Ions score 21)
416 - 435	1217.5373	2433.0601	2433.0619	-1	1	K.MMDKVEFVHMLNATMCATTR.T	3 Oxidation (M)	(Ions score 32)
416 - 435	609.2723	2433.0603	2433.0619	-1	1	K.MMDKVEFVHMLNATMCATTR.T	3 Oxidation (M)	(Ions score 23)
416 - 435	609.2723	2433.0603	2433.0619	-1	1	K.MMDKVEFVHMLNATMCATTR.T	3 Oxidation (M)	(Ions score 21)
416 - 435	812.0274	2433.0603	2433.0619	-1	1	K.MMDKVEFVHMLNATMCATTR.T	3 Oxidation (M)	(Ions score 40)
416 - 435	609.2724	2433.0605	2433.0619	-1	1	K.MMDKVEFVHMLNATMCATTR.T	3 Oxidation (M)	(Ions score 28)
416 - 435	812.0275	2433.0606	2433.0619	-1	1	K.MMDKVEFVHMLNATMCATTR.T	3 Oxidation (M)	(Ions score 33)
416 - 435	812.0275	2433.0608	2433.0619	-0	1	K.MMDKVEFVHMLNATMCATTR.T	3 Oxidation (M)	(Ions score 30)
416 - 435	812.0276	2433.0611	2433.0619	-0	1	K.MMDKVEFVHMLNATMCATTR.T	3 Oxidation (M)	(Ions score 31)
416 - 435	812.0278	2433.0616	2433.0619	-0	1	K.MMDKVEFVHMLNATMCATTR.T	3 Oxidation (M)	(Ions score 33)
416 - 435	812.0278	2433.0617	2433.0619	-0	1	K.MMDKVEFVHMLNATMCATTR.T	3 Oxidation (M)	(Ions score 42)
416 - 435	812.0278	2433.0617	2433.0619	-0	1	K.MMDKVEFVHMLNATMCATTR.T	3 Oxidation (M)	(Ions score 45)
416 - 435	812.0278	2433.0617	2433.0619	-0	1	K.MMDKVEFVHMLNATMCATTR.T	3 Oxidation (M)	(Ions score 44)
416 - 435	812.0279	2433.0619	2433.0619	-0	1	K.MMDKVEFVHMLNATMCATTR.T	3 Oxidation (M)	(Ions score 44)
416 - 435	812.0279	2433.0619	2433.0619	-0	1	K.MMDKVEFVHMLNATMCATTR.T	3 Oxidation (M)	(Ions score 21)
416 - 435	609.2728	2433.0620	2433.0619	0	1	K.MMDKVEFVHMLNATMCATTR.T	3 Oxidation (M)	(Ions score 26)
416 - 435	609.2728	2433.0620	2433.0619	0	1	K.MMDKVEFVHMLNATMCATTR.T	3 Oxidation (M)	(Ions score 27)
416 - 435	812.0280	2433.0620	2433.0619	0	1	K.MMDKVEFVHMLNATMCATTR.T	3 Oxidation (M)	(Ions score 40)
416 - 435	812.0280	2433.0620	2433.0619	0	1	K.MMDKVEFVHMLNATMCATTR.T	3 Oxidation (M)	(Ions score 33)
416 - 435	609.2728	2433.0621	2433.0619	0	1	K.MMDKVEFVHMLNATMCATTR.T	3 Oxidation (M)	(Ions score 29)
416 - 435	812.0280	2433.0622	2433.0619	0	1	K.MMDKVEFVHMLNATMCATTR.T	3 Oxidation (M)	(Ions score 38)
416 - 435	812.0280	2433.0622	2433.0619	0	1	K.MMDKVEFVHMLNATMCATTR.T	3 Oxidation (M)	(Ions score 22)
416 - 435	609.2728	2433.0623	2433.0619	0	1	K.MMDKVEFVHMLNATMCATTR.T	3 Oxidation (M)	(Ions score 29)
416 - 435	609.2729	2433.0623	2433.0619	0	1	K.MMDKVEFVHMLNATMCATTR.T	3 Oxidation (M)	(Ions score 28)
416 - 435	812.0281	2433.0623	2433.0619	0	1	K.MMDKVEFVHMLNATMCATTR.T	3 Oxidation (M)	(Ions score 42)
416 - 435	609.2729	2433.0624	2433.0619	0	1	K.MMDKVEFVHMLNATMCATTR.T	3 Oxidation (M)	(Ions score 26)
416 - 435	609.2729	2433.0625	2433.0619	0	1	K.MMDKVEFVHMLNATMCATTR.T	3 Oxidation (M)	(Ions score 29)

416 - 435	812.0282	2433.0626	2433.0619	0	1	K.MMDKVEFVHMLNATMCATTR.T	3 Oxidation (M)	(Ions score 31)
416 - 435	812.0282	2433.0627	2433.0619	0	1	K.MMDKVEFVHMLNATMCATTR.T	3 Oxidation (M)	(Ions score 23)
416 - 435	812.0282	2433.0627	2433.0619	0	1	K.MMDKVEFVHMLNATMCATTR.T	3 Oxidation (M)	(Ions score 44)
416 - 435	812.0282	2433.0629	2433.0619	0	1	K.MMDKVEFVHMLNATMCATTR.T	3 Oxidation (M)	(Ions score 42)
416 - 435	609.2730	2433.0629	2433.0619	0	1	K.MMDKVEFVHMLNATMCATTR.T	3 Oxidation (M)	(Ions score 26)
416 - 435	609.2730	2433.0629	2433.0619	0	1	K.MMDKVEFVHMLNATMCATTR.T	3 Oxidation (M)	(Ions score 34)
416 - 435	812.0283	2433.0631	2433.0619	0	1	K.MMDKVEFVHMLNATMCATTR.T	3 Oxidation (M)	(Ions score 23)
416 - 435	812.0283	2433.0631	2433.0619	0	1	K.MMDKVEFVHMLNATMCATTR.T	3 Oxidation (M)	(Ions score 33)
416 - 435	609.2731	2433.0633	2433.0619	1	1	K.MMDKVEFVHMLNATMCATTR.T	3 Oxidation (M)	(Ions score 22)
416 - 435	609.2731	2433.0633	2433.0619	1	1	K.MMDKVEFVHMLNATMCATTR.T	3 Oxidation (M)	(Ions score 34)
416 - 435	609.2731	2433.0634	2433.0619	1	1	K.MMDKVEFVHMLNATMCATTR.T	3 Oxidation (M)	(Ions score 28)
416 - 435	609.2731	2433.0634	2433.0619	1	1	K.MMDKVEFVHMLNATMCATTR.T	3 Oxidation (M)	(Ions score 22)
416 - 435	609.2732	2433.0635	2433.0619	1	1	K.MMDKVEFVHMLNATMCATTR.T	3 Oxidation (M)	(Ions score 23)
416 - 435	609.2732	2433.0636	2433.0619	1	1	K.MMDKVEFVHMLNATMCATTR.T	3 Oxidation (M)	(Ions score 23)
416 - 435	609.2732	2433.0636	2433.0619	1	1	K.MMDKVEFVHMLNATMCATTR.T	3 Oxidation (M)	(Ions score 28)
416 - 435	812.0285	2433.0636	2433.0619	1	1	K.MMDKVEFVHMLNATMCATTR.T	3 Oxidation (M)	(Ions score 33)
416 - 435	812.0285	2433.0637	2433.0619	1	1	K.MMDKVEFVHMLNATMCATTR.T	3 Oxidation (M)	(Ions score 32)
416 - 435	812.0285	2433.0637	2433.0619	1	1	K.MMDKVEFVHMLNATMCATTR.T	3 Oxidation (M)	(Ions score 37)
416 - 435	812.0285	2433.0637	2433.0619	1	1	K.MMDKVEFVHMLNATMCATTR.T	3 Oxidation (M)	(Ions score 30)
416 - 435	1217.5392	2433.0638	2433.0619	1	1	K.MMDKVEFVHMLNATMCATTR.T	3 Oxidation (M)	(Ions score 49)
416 - 435	812.0285	2433.0638	2433.0619	1	1	K.MMDKVEFVHMLNATMCATTR.T	3 Oxidation (M)	(Ions score 31)
416 - 435	1217.5392	2433.0638	2433.0619	1	1	K.MMDKVEFVHMLNATMCATTR.T	3 Oxidation (M)	(Ions score 37)
416 - 435	812.0286	2433.0639	2433.0619	1	1	K.MMDKVEFVHMLNATMCATTR.T	3 Oxidation (M)	(Ions score 25)
416 - 435	812.0286	2433.0640	2433.0619	1	1	K.MMDKVEFVHMLNATMCATTR.T	3 Oxidation (M)	(Ions score 44)
416 - 435	609.2733	2433.0640	2433.0619	1	1	K.MMDKVEFVHMLNATMCATTR.T	3 Oxidation (M)	(Ions score 27)
416 - 435	812.0286	2433.0640	2433.0619	1	1	K.MMDKVEFVHMLNATMCATTR.T	3 Oxidation (M)	(Ions score 44)
416 - 435	812.0286	2433.0640	2433.0619	1	1	K.MMDKVEFVHMLNATMCATTR.T	3 Oxidation (M)	(Ions score 24)
416 - 435	609.2733	2433.0641	2433.0619	1	1	K.MMDKVEFVHMLNATMCATTR.T	3 Oxidation (M)	(Ions score 23)
416 - 435	609.2733	2433.0642	2433.0619	1	1	K.MMDKVEFVHMLNATMCATTR.T	3 Oxidation (M)	(Ions score 23)
416 - 435	609.2733	2433.0642	2433.0619	1	1	K.MMDKVEFVHMLNATMCATTR.T	3 Oxidation (M)	(Ions score 24)
416 - 435	812.0287	2433.0642	2433.0619	1	1	K.MMDKVEFVHMLNATMCATTR.T	3 Oxidation (M)	(Ions score 32)
416 - 435	812.0287	2433.0642	2433.0619	1	1	K.MMDKVEFVHMLNATMCATTR.T	3 Oxidation (M)	(Ions score 32)
416 - 435	812.0287	2433.0644	2433.0619	1	1	K.MMDKVEFVHMLNATMCATTR.T	3 Oxidation (M)	(Ions score 40)
416 - 435	609.2734	2433.0644	2433.0619	1	1	K.MMDKVEFVHMLNATMCATTR.T	3 Oxidation (M)	(Ions score 33)
416 - 435	812.0287	2433.0644	2433.0619	1	1	K.MMDKVEFVHMLNATMCATTR.T	3 Oxidation (M)	(Ions score 23)
416 - 435	609.2734	2433.0644	2433.0619	1	1	K.MMDKVEFVHMLNATMCATTR.T	3 Oxidation (M)	(Ions score 30)
416 - 435	812.0288	2433.0645	2433.0619	1	1	K.MMDKVEFVHMLNATMCATTR.T	3 Oxidation (M)	(Ions score 43)
416 - 435	812.0288	2433.0645	2433.0619	1	1	K.MMDKVEFVHMLNATMCATTR.T	3 Oxidation (M)	(Ions score 30)
416 - 435	812.0288	2433.0646	2433.0619	1	1	K.MMDKVEFVHMLNATMCATTR.T	3 Oxidation (M)	(Ions score 30)
416 - 435	812.0288	2433.0647	2433.0619	1	1	K.MMDKVEFVHMLNATMCATTR.T	3 Oxidation (M)	(Ions score 34)
416 - 435	609.2735	2433.0647	2433.0619	1	1	K.MMDKVEFVHMLNATMCATTR.T	3 Oxidation (M)	(Ions score 29)
416 - 435	812.0289	2433.0648	2433.0619	1	1	K.MMDKVEFVHMLNATMCATTR.T	3 Oxidation (M)	(Ions score 45)
416 - 435	812.0289	2433.0648	2433.0619	1	1	K.MMDKVEFVHMLNATMCATTR.T	3 Oxidation (M)	(Ions score 43)
416 - 435	812.0289	2433.0649	2433.0619	1	1	K.MMDKVEFVHMLNATMCATTR.T	3 Oxidation (M)	(Ions score 33)
416 - 435	609.2735	2433.0649	2433.0619	1	1	K.MMDKVEFVHMLNATMCATTR.T	3 Oxidation (M)	(Ions score 23)
416 - 435	609.2735	2433.0649	2433.0619	1	1	K.MMDKVEFVHMLNATMCATTR.T	3 Oxidation (M)	(Ions score 30)
416 - 435	609.2735	2433.0649	2433.0619	1	1	K.MMDKVEFVHMLNATMCATTR.T	3 Oxidation (M)	(Ions score 31)
416 - 435	812.0289	2433.0650	2433.0619	1	1	K.MMDKVEFVHMLNATMCATTR.T	3 Oxidation (M)	(Ions score 37)
416 - 435	812.0289	2433.0650	2433.0619	1	1	K.MMDKVEFVHMLNATMCATTR.T	3 Oxidation (M)	(Ions score 33)
416 - 435	812.0289	2433.0650	2433.0619	1	1	K.MMDKVEFVHMLNATMCATTR.T	3 Oxidation (M)	(Ions score 31)
416 - 435	812.0289	2433.0650	2433.0619	1	1	K.MMDKVEFVHMLNATMCATTR.T	3 Oxidation (M)	(Ions score 47)
416 - 435	812.0290	2433.0651	2433.0619	1	1	K.MMDKVEFVHMLNATMCATTR.T	3 Oxidation (M)	(Ions score 42)
416 - 435	609.2736	2433.0651	2433.0619	1	1	K.MMDKVEFVHMLNATMCATTR.T	3 Oxidation (M)	(Ions score 26)
416 - 435	812.0290	2433.0652	2433.0619	1	1	K.MMDKVEFVHMLNATMCATTR.T	3 Oxidation (M)	(Ions score 31)
416 - 435	812.0290	2433.0653	2433.0619	1	1	K.MMDKVEFVHMLNATMCATTR.T	3 Oxidation (M)	(Ions score 40)
416 - 435	812.0291	2433.0653	2433.0619	1	1	K.MMDKVEFVHMLNATMCATTR.T	3 Oxidation (M)	(Ions score 29)
416 - 435	812.0291	2433.0653	2433.0619	1	1	K.MMDKVEFVHMLNATMCATTR.T	3 Oxidation (M)	(Ions score 38)
416 - 435	812.0291	2433.0654	2433.0619	1	1	K.MMDKVEFVHMLNATMCATTR.T	3 Oxidation (M)	(Ions score 41)

416 - 435	609.2736	2433.0654	2433.0619	1	1	K.MMDKVEFVHMLNATMCATTR.T	3 Oxidation (M)	(Ions score 22)
416 - 435	609.2736	2433.0654	2433.0619	1	1	K.MMDKVEFVHMLNATMCATTR.T	3 Oxidation (M)	(Ions score 31)
416 - 435	609.2736	2433.0655	2433.0619	1	1	K.MMDKVEFVHMLNATMCATTR.T	3 Oxidation (M)	(Ions score 25)
416 - 435	812.0291	2433.0655	2433.0619	1	1	K.MMDKVEFVHMLNATMCATTR.T	3 Oxidation (M)	(Ions score 31)
416 - 435	812.0291	2433.0655	2433.0619	1	1	K.MMDKVEFVHMLNATMCATTR.T	3 Oxidation (M)	(Ions score 38)
416 - 435	812.0291	2433.0655	2433.0619	1	1	K.MMDKVEFVHMLNATMCATTR.T	3 Oxidation (M)	(Ions score 44)
416 - 435	812.0291	2433.0655	2433.0619	1	1	K.MMDKVEFVHMLNATMCATTR.T	3 Oxidation (M)	(Ions score 51)
416 - 435	812.0291	2433.0655	2433.0619	1	1	K.MMDKVEFVHMLNATMCATTR.T	3 Oxidation (M)	(Ions score 34)
416 - 435	609.2737	2433.0655	2433.0619	1	1	K.MMDKVEFVHMLNATMCATTR.T	3 Oxidation (M)	(Ions score 28)
416 - 435	609.2737	2433.0656	2433.0619	2	1	K.MMDKVEFVHMLNATMCATTR.T	3 Oxidation (M)	(Ions score 31)
416 - 435	812.0291	2433.0656	2433.0619	2	1	K.MMDKVEFVHMLNATMCATTR.T	3 Oxidation (M)	(Ions score 48)
416 - 435	609.2737	2433.0656	2433.0619	2	1	K.MMDKVEFVHMLNATMCATTR.T	3 Oxidation (M)	(Ions score 26)
416 - 435	609.2737	2433.0656	2433.0619	2	1	K.MMDKVEFVHMLNATMCATTR.T	3 Oxidation (M)	(Ions score 31)
416 - 435	609.2737	2433.0656	2433.0619	2	1	K.MMDKVEFVHMLNATMCATTR.T	3 Oxidation (M)	(Ions score 32)
416 - 435	812.0292	2433.0657	2433.0619	2	1	K.MMDKVEFVHMLNATMCATTR.T	3 Oxidation (M)	(Ions score 31)
416 - 435	609.2737	2433.0657	2433.0619	2	1	K.MMDKVEFVHMLNATMCATTR.T	3 Oxidation (M)	(Ions score 27)
416 - 435	812.0292	2433.0658	2433.0619	2	1	K.MMDKVEFVHMLNATMCATTR.T	3 Oxidation (M)	(Ions score 34)
416 - 435	609.2737	2433.0658	2433.0619	2	1	K.MMDKVEFVHMLNATMCATTR.T	3 Oxidation (M)	(Ions score 34)
416 - 435	609.2737	2433.0659	2433.0619	2	1	K.MMDKVEFVHMLNATMCATTR.T	3 Oxidation (M)	(Ions score 28)
416 - 435	609.2737	2433.0659	2433.0619	2	1	K.MMDKVEFVHMLNATMCATTR.T	3 Oxidation (M)	(Ions score 23)
416 - 435	609.2738	2433.0659	2433.0619	2	1	K.MMDKVEFVHMLNATMCATTR.T	3 Oxidation (M)	(Ions score 21)
416 - 435	812.0293	2433.0659	2433.0619	2	1	K.MMDKVEFVHMLNATMCATTR.T	3 Oxidation (M)	(Ions score 34)
416 - 435	609.2738	2433.0660	2433.0619	2	1	K.MMDKVEFVHMLNATMCATTR.T	3 Oxidation (M)	(Ions score 26)
416 - 435	812.0293	2433.0661	2433.0619	2	1	K.MMDKVEFVHMLNATMCATTR.T	3 Oxidation (M)	(Ions score 35)
416 - 435	609.2738	2433.0661	2433.0619	2	1	K.MMDKVEFVHMLNATMCATTR.T	3 Oxidation (M)	(Ions score 29)
416 - 435	609.2738	2433.0661	2433.0619	2	1	K.MMDKVEFVHMLNATMCATTR.T	3 Oxidation (M)	(Ions score 22)
416 - 435	609.2738	2433.0661	2433.0619	2	1	K.MMDKVEFVHMLNATMCATTR.T	3 Oxidation (M)	(Ions score 26)
416 - 435	609.2738	2433.0662	2433.0619	2	1	K.MMDKVEFVHMLNATMCATTR.T	3 Oxidation (M)	(Ions score 27)
416 - 435	812.0294	2433.0663	2433.0619	2	1	K.MMDKVEFVHMLNATMCATTR.T	3 Oxidation (M)	(Ions score 39)
416 - 435	609.2738	2433.0663	2433.0619	2	1	K.MMDKVEFVHMLNATMCATTR.T	3 Oxidation (M)	(Ions score 31)
416 - 435	609.2739	2433.0663	2433.0619	2	1	K.MMDKVEFVHMLNATMCATTR.T	3 Oxidation (M)	(Ions score 25)
416 - 435	609.2739	2433.0664	2433.0619	2	1	K.MMDKVEFVHMLNATMCATTR.T	3 Oxidation (M)	(Ions score 21)
416 - 435	812.0294	2433.0664	2433.0619	2	1	K.MMDKVEFVHMLNATMCATTR.T	3 Oxidation (M)	(Ions score 48)
416 - 435	812.0294	2433.0664	2433.0619	2	1	K.MMDKVEFVHMLNATMCATTR.T	3 Oxidation (M)	(Ions score 42)
416 - 435	609.2739	2433.0664	2433.0619	2	1	K.MMDKVEFVHMLNATMCATTR.T	3 Oxidation (M)	(Ions score 28)
416 - 435	812.0294	2433.0665	2433.0619	2	1	K.MMDKVEFVHMLNATMCATTR.T	3 Oxidation (M)	(Ions score 27)
416 - 435	609.2739	2433.0665	2433.0619	2	1	K.MMDKVEFVHMLNATMCATTR.T	3 Oxidation (M)	(Ions score 23)
416 - 435	609.2739	2433.0666	2433.0619	2	1	K.MMDKVEFVHMLNATMCATTR.T	3 Oxidation (M)	(Ions score 29)
416 - 435	609.2740	2433.0667	2433.0619	2	1	K.MMDKVEFVHMLNATMCATTR.T	3 Oxidation (M)	(Ions score 22)
416 - 435	609.2740	2433.0668	2433.0619	2	1	K.MMDKVEFVHMLNATMCATTR.T	3 Oxidation (M)	(Ions score 29)
416 - 435	812.0295	2433.0668	2433.0619	2	1	K.MMDKVEFVHMLNATMCATTR.T	3 Oxidation (M)	(Ions score 37)
416 - 435	812.0296	2433.0668	2433.0619	2	1	K.MMDKVEFVHMLNATMCATTR.T	3 Oxidation (M)	(Ions score 36)
416 - 435	609.2740	2433.0669	2433.0619	2	1	K.MMDKVEFVHMLNATMCATTR.T	3 Oxidation (M)	(Ions score 28)
416 - 435	609.2740	2433.0670	2433.0619	2	1	K.MMDKVEFVHMLNATMCATTR.T	3 Oxidation (M)	(Ions score 27)
416 - 435	812.0296	2433.0670	2433.0619	2	1	K.MMDKVEFVHMLNATMCATTR.T	3 Oxidation (M)	(Ions score 39)
416 - 435	812.0296	2433.0671	2433.0619	2	1	K.MMDKVEFVHMLNATMCATTR.T	3 Oxidation (M)	(Ions score 43)
416 - 435	609.2741	2433.0672	2433.0619	2	1	K.MMDKVEFVHMLNATMCATTR.T	3 Oxidation (M)	(Ions score 29)
416 - 435	812.0297	2433.0672	2433.0619	2	1	K.MMDKVEFVHMLNATMCATTR.T	3 Oxidation (M)	(Ions score 29)
416 - 435	609.2741	2433.0672	2433.0619	2	1	K.MMDKVEFVHMLNATMCATTR.T	3 Oxidation (M)	(Ions score 24)
416 - 435	609.2741	2433.0672	2433.0619	2	1	K.MMDKVEFVHMLNATMCATTR.T	3 Oxidation (M)	(Ions score 29)
416 - 435	812.0297	2433.0672	2433.0619	2	1	K.MMDKVEFVHMLNATMCATTR.T	3 Oxidation (M)	(Ions score 40)
416 - 435	812.0297	2433.0672	2433.0619	2	1	K.MMDKVEFVHMLNATMCATTR.T	3 Oxidation (M)	(Ions score 34)
416 - 435	609.2741	2433.0673	2433.0619	2	1	K.MMDKVEFVHMLNATMCATTR.T	3 Oxidation (M)	(Ions score 27)
416 - 435	812.0297	2433.0673	2433.0619	2	1	K.MMDKVEFVHMLNATMCATTR.T	3 Oxidation (M)	(Ions score 43)
416 - 435	812.0297	2433.0673	2433.0619	2	1	K.MMDKVEFVHMLNATMCATTR.T	3 Oxidation (M)	(Ions score 30)
416 - 435	609.2741	2433.0673	2433.0619	2	1	K.MMDKVEFVHMLNATMCATTR.T	3 Oxidation (M)	(Ions score 21)
416 - 435	812.0297	2433.0674	2433.0619	2	1	K.MMDKVEFVHMLNATMCATTR.T	3 Oxidation (M)	(Ions score 42)
416 - 435	609.2741	2433.0674	2433.0619	2	1	K.MMDKVEFVHMLNATMCATTR.T	3 Oxidation (M)	(Ions score 26)

416 - 435	609.2741	2433.0674	2433.0619	2	1	K.MMDKVEFVHMLNATMCATTR.T	3 Oxidation (M)	(Ions score 29)
416 - 435	609.2741	2433.0675	2433.0619	2	1	K.MMDKVEFVHMLNATMCATTR.T	3 Oxidation (M)	(Ions score 28)
416 - 435	609.2741	2433.0675	2433.0619	2	1	K.MMDKVEFVHMLNATMCATTR.T	3 Oxidation (M)	(Ions score 22)
416 - 435	609.2742	2433.0675	2433.0619	2	1	K.MMDKVEFVHMLNATMCATTR.T	3 Oxidation (M)	(Ions score 31)
416 - 435	609.2742	2433.0676	2433.0619	2	1	K.MMDKVEFVHMLNATMCATTR.T	3 Oxidation (M)	(Ions score 29)
416 - 435	609.2742	2433.0676	2433.0619	2	1	K.MMDKVEFVHMLNATMCATTR.T	3 Oxidation (M)	(Ions score 27)
416 - 435	812.0298	2433.0676	2433.0619	2	1	K.MMDKVEFVHMLNATMCATTR.T	3 Oxidation (M)	(Ions score 42)
416 - 435	812.0298	2433.0676	2433.0619	2	1	K.MMDKVEFVHMLNATMCATTR.T	3 Oxidation (M)	(Ions score 51)
416 - 435	609.2742	2433.0677	2433.0619	2	1	K.MMDKVEFVHMLNATMCATTR.T	3 Oxidation (M)	(Ions score 26)
416 - 435	812.0299	2433.0677	2433.0619	2	1	K.MMDKVEFVHMLNATMCATTR.T	3 Oxidation (M)	(Ions score 32)
416 - 435	609.2742	2433.0677	2433.0619	2	1	K.MMDKVEFVHMLNATMCATTR.T	3 Oxidation (M)	(Ions score 21)
416 - 435	609.2742	2433.0678	2433.0619	2	1	K.MMDKVEFVHMLNATMCATTR.T	3 Oxidation (M)	(Ions score 23)
416 - 435	812.0299	2433.0678	2433.0619	2	1	K.MMDKVEFVHMLNATMCATTR.T	3 Oxidation (M)	(Ions score 32)
416 - 435	609.2742	2433.0679	2433.0619	2	1	K.MMDKVEFVHMLNATMCATTR.T	3 Oxidation (M)	(Ions score 29)
416 - 435	609.2743	2433.0679	2433.0619	2	1	K.MMDKVEFVHMLNATMCATTR.T	3 Oxidation (M)	(Ions score 22)
416 - 435	609.2743	2433.0679	2433.0619	2	1	K.MMDKVEFVHMLNATMCATTR.T	3 Oxidation (M)	(Ions score 31)
416 - 435	609.2743	2433.0680	2433.0619	2	1	K.MMDKVEFVHMLNATMCATTR.T	3 Oxidation (M)	(Ions score 28)
416 - 435	812.0299	2433.0680	2433.0619	2	1	K.MMDKVEFVHMLNATMCATTR.T	3 Oxidation (M)	(Ions score 25)
416 - 435	609.2743	2433.0681	2433.0619	3	1	K.MMDKVEFVHMLNATMCATTR.T	3 Oxidation (M)	(Ions score 29)
416 - 435	609.2743	2433.0681	2433.0619	3	1	K.MMDKVEFVHMLNATMCATTR.T	3 Oxidation (M)	(Ions score 27)
416 - 435	812.0300	2433.0681	2433.0619	3	1	K.MMDKVEFVHMLNATMCATTR.T	3 Oxidation (M)	(Ions score 31)
416 - 435	609.2743	2433.0681	2433.0619	3	1	K.MMDKVEFVHMLNATMCATTR.T	3 Oxidation (M)	(Ions score 29)
416 - 435	609.2743	2433.0681	2433.0619	3	1	K.MMDKVEFVHMLNATMCATTR.T	3 Oxidation (M)	(Ions score 39)
416 - 435	812.0300	2433.0681	2433.0619	3	1	K.MMDKVEFVHMLNATMCATTR.T	3 Oxidation (M)	(Ions score 45)
416 - 435	609.2743	2433.0682	2433.0619	3	1	K.MMDKVEFVHMLNATMCATTR.T	3 Oxidation (M)	(Ions score 29)
416 - 435	609.2743	2433.0682	2433.0619	3	1	K.MMDKVEFVHMLNATMCATTR.T	3 Oxidation (M)	(Ions score 34)
416 - 435	812.0300	2433.0682	2433.0619	3	1	K.MMDKVEFVHMLNATMCATTR.T	3 Oxidation (M)	(Ions score 35)
416 - 435	609.2743	2433.0683	2433.0619	3	1	K.MMDKVEFVHMLNATMCATTR.T	3 Oxidation (M)	(Ions score 28)
416 - 435	609.2743	2433.0683	2433.0619	3	1	K.MMDKVEFVHMLNATMCATTR.T	3 Oxidation (M)	(Ions score 24)
416 - 435	812.0300	2433.0683	2433.0619	3	1	K.MMDKVEFVHMLNATMCATTR.T	3 Oxidation (M)	(Ions score 31)
416 - 435	609.2744	2433.0683	2433.0619	3	1	K.MMDKVEFVHMLNATMCATTR.T	3 Oxidation (M)	(Ions score 28)
416 - 435	609.2744	2433.0683	2433.0619	3	1	K.MMDKVEFVHMLNATMCATTR.T	3 Oxidation (M)	(Ions score 21)
416 - 435	609.2744	2433.0683	2433.0619	3	1	K.MMDKVEFVHMLNATMCATTR.T	3 Oxidation (M)	(Ions score 28)
416 - 435	609.2744	2433.0684	2433.0619	3	1	K.MMDKVEFVHMLNATMCATTR.T	3 Oxidation (M)	(Ions score 23)
416 - 435	609.2744	2433.0684	2433.0619	3	1	K.MMDKVEFVHMLNATMCATTR.T	3 Oxidation (M)	(Ions score 30)
416 - 435	812.0301	2433.0684	2433.0619	3	1	K.MMDKVEFVHMLNATMCATTR.T	3 Oxidation (M)	(Ions score 39)
416 - 435	609.2744	2433.0684	2433.0619	3	1	K.MMDKVEFVHMLNATMCATTR.T	3 Oxidation (M)	(Ions score 31)
416 - 435	812.0301	2433.0684	2433.0619	3	1	K.MMDKVEFVHMLNATMCATTR.T	3 Oxidation (M)	(Ions score 48)
416 - 435	812.0301	2433.0685	2433.0619	3	1	K.MMDKVEFVHMLNATMCATTR.T	3 Oxidation (M)	(Ions score 39)
416 - 435	609.2744	2433.0685	2433.0619	3	1	K.MMDKVEFVHMLNATMCATTR.T	3 Oxidation (M)	(Ions score 34)
416 - 435	609.2744	2433.0685	2433.0619	3	1	K.MMDKVEFVHMLNATMCATTR.T	3 Oxidation (M)	(Ions score 30)
416 - 435	609.2744	2433.0686	2433.0619	3	1	K.MMDKVEFVHMLNATMCATTR.T	3 Oxidation (M)	(Ions score 25)
416 - 435	812.0302	2433.0686	2433.0619	3	1	K.MMDKVEFVHMLNATMCATTR.T	3 Oxidation (M)	(Ions score 31)
416 - 435	812.0302	2433.0687	2433.0619	3	1	K.MMDKVEFVHMLNATMCATTR.T	3 Oxidation (M)	(Ions score 45)
416 - 435	812.0302	2433.0687	2433.0619	3	1	K.MMDKVEFVHMLNATMCATTR.T	3 Oxidation (M)	(Ions score 42)
416 - 435	812.0302	2433.0687	2433.0619	3	1	K.MMDKVEFVHMLNATMCATTR.T	3 Oxidation (M)	(Ions score 54)
416 - 435	609.2745	2433.0688	2433.0619	3	1	K.MMDKVEFVHMLNATMCATTR.T	3 Oxidation (M)	(Ions score 26)
416 - 435	609.2745	2433.0688	2433.0619	3	1	K.MMDKVEFVHMLNATMCATTR.T	3 Oxidation (M)	(Ions score 25)
416 - 435	812.0302	2433.0688	2433.0619	3	1	K.MMDKVEFVHMLNATMCATTR.T	3 Oxidation (M)	(Ions score 31)
416 - 435	609.2745	2433.0688	2433.0619	3	1	K.MMDKVEFVHMLNATMCATTR.T	3 Oxidation (M)	(Ions score 34)
416 - 435	609.2745	2433.0688	2433.0619	3	1	K.MMDKVEFVHMLNATMCATTR.T	3 Oxidation (M)	(Ions score 31)
416 - 435	609.2745	2433.0689	2433.0619	3	1	K.MMDKVEFVHMLNATMCATTR.T	3 Oxidation (M)	(Ions score 23)
416 - 435	609.2745	2433.0689	2433.0619	3	1	K.MMDKVEFVHMLNATMCATTR.T	3 Oxidation (M)	(Ions score 22)
416 - 435	609.2745	2433.0689	2433.0619	3	1	K.MMDKVEFVHMLNATMCATTR.T	3 Oxidation (M)	(Ions score 30)
416 - 435	609.2745	2433.0690	2433.0619	3	1	K.MMDKVEFVHMLNATMCATTR.T	3 Oxidation (M)	(Ions score 31)
416 - 435	812.0303	2433.0690	2433.0619	3	1	K.MMDKVEFVHMLNATMCATTR.T	3 Oxidation (M)	(Ions score 28)
416 - 435	812.0303	2433.0690	2433.0619	3	1	K.MMDKVEFVHMLNATMCATTR.T	3 Oxidation (M)	(Ions score 42)
416 - 435	812.0303	2433.0690	2433.0619	3	1	K.MMDKVEFVHMLNATMCATTR.T	3 Oxidation (M)	(Ions score 35)

416 - 435	812.0303	2433.0690	2433.0619	3	1	K.MMDKVEFVHMLNATMCATTR.T	3 Oxidation (M) (Ions score 44)
416 - 435	812.0303	2433.0691	2433.0619	3	1	K.MMDKVEFVHMLNATMCATTR.T	3 Oxidation (M) (Ions score 42)
416 - 435	609.2746	2433.0692	2433.0619	3	1	K.MMDKVEFVHMLNATMCATTR.T	3 Oxidation (M) (Ions score 22)
416 - 435	812.0303	2433.0692	2433.0619	3	1	K.MMDKVEFVHMLNATMCATTR.T	3 Oxidation (M) (Ions score 44)
416 - 435	609.2746	2433.0692	2433.0619	3	1	K.MMDKVEFVHMLNATMCATTR.T	3 Oxidation (M) (Ions score 29)
416 - 435	609.2746	2433.0692	2433.0619	3	1	K.MMDKVEFVHMLNATMCATTR.T	3 Oxidation (M) (Ions score 28)
416 - 435	812.0304	2433.0693	2433.0619	3	1	K.MMDKVEFVHMLNATMCATTR.T	3 Oxidation (M) (Ions score 44)
416 - 435	812.0304	2433.0693	2433.0619	3	1	K.MMDKVEFVHMLNATMCATTR.T	3 Oxidation (M) (Ions score 32)
416 - 435	812.0304	2433.0693	2433.0619	3	1	K.MMDKVEFVHMLNATMCATTR.T	3 Oxidation (M) (Ions score 29)
416 - 435	609.2746	2433.0693	2433.0619	3	1	K.MMDKVEFVHMLNATMCATTR.T	3 Oxidation (M) (Ions score 22)
416 - 435	812.0304	2433.0693	2433.0619	3	1	K.MMDKVEFVHMLNATMCATTR.T	3 Oxidation (M) (Ions score 35)
416 - 435	609.2746	2433.0694	2433.0619	3	1	K.MMDKVEFVHMLNATMCATTR.T	3 Oxidation (M) (Ions score 32)
416 - 435	812.0304	2433.0694	2433.0619	3	1	K.MMDKVEFVHMLNATMCATTR.T	3 Oxidation (M) (Ions score 31)
416 - 435	812.0304	2433.0695	2433.0619	3	1	K.MMDKVEFVHMLNATMCATTR.T	3 Oxidation (M) (Ions score 32)
416 - 435	812.0304	2433.0695	2433.0619	3	1	K.MMDKVEFVHMLNATMCATTR.T	3 Oxidation (M) (Ions score 45)
416 - 435	609.2747	2433.0695	2433.0619	3	1	K.MMDKVEFVHMLNATMCATTR.T	3 Oxidation (M) (Ions score 30)
416 - 435	812.0304	2433.0695	2433.0619	3	1	K.MMDKVEFVHMLNATMCATTR.T	3 Oxidation (M) (Ions score 42)
416 - 435	609.2747	2433.0695	2433.0619	3	1	K.MMDKVEFVHMLNATMCATTR.T	3 Oxidation (M) (Ions score 21)
416 - 435	609.2747	2433.0696	2433.0619	3	1	K.MMDKVEFVHMLNATMCATTR.T	3 Oxidation (M) (Ions score 24)
416 - 435	609.2747	2433.0696	2433.0619	3	1	K.MMDKVEFVHMLNATMCATTR.T	3 Oxidation (M) (Ions score 23)
416 - 435	609.2747	2433.0697	2433.0619	3	1	K.MMDKVEFVHMLNATMCATTR.T	3 Oxidation (M) (Ions score 28)
416 - 435	812.0305	2433.0697	2433.0619	3	1	K.MMDKVEFVHMLNATMCATTR.T	3 Oxidation (M) (Ions score 44)
416 - 435	609.2747	2433.0697	2433.0619	3	1	K.MMDKVEFVHMLNATMCATTR.T	3 Oxidation (M) (Ions score 29)
416 - 435	609.2747	2433.0698	2433.0619	3	1	K.MMDKVEFVHMLNATMCATTR.T	3 Oxidation (M) (Ions score 29)
416 - 435	609.2747	2433.0698	2433.0619	3	1	K.MMDKVEFVHMLNATMCATTR.T	3 Oxidation (M) (Ions score 31)
416 - 435	812.0306	2433.0699	2433.0619	3	1	K.MMDKVEFVHMLNATMCATTR.T	3 Oxidation (M) (Ions score 44)
416 - 435	609.2748	2433.0700	2433.0619	3	1	K.MMDKVEFVHMLNATMCATTR.T	3 Oxidation (M) (Ions score 23)
416 - 435	609.2748	2433.0700	2433.0619	3	1	K.MMDKVEFVHMLNATMCATTR.T	3 Oxidation (M) (Ions score 31)
416 - 435	609.2748	2433.0701	2433.0619	3	1	K.MMDKVEFVHMLNATMCATTR.T	3 Oxidation (M) (Ions score 34)
416 - 435	609.2748	2433.0702	2433.0619	3	1	K.MMDKVEFVHMLNATMCATTR.T	3 Oxidation (M) (Ions score 29)
416 - 435	609.2748	2433.0702	2433.0619	3	1	K.MMDKVEFVHMLNATMCATTR.T	3 Oxidation (M) (Ions score 26)
416 - 435	609.2748	2433.0702	2433.0619	3	1	K.MMDKVEFVHMLNATMCATTR.T	3 Oxidation (M) (Ions score 34)
416 - 435	812.0307	2433.0703	2433.0619	3	1	K.MMDKVEFVHMLNATMCATTR.T	3 Oxidation (M) (Ions score 21)
416 - 435	609.2749	2433.0703	2433.0619	3	1	K.MMDKVEFVHMLNATMCATTR.T	3 Oxidation (M) (Ions score 28)
416 - 435	812.0307	2433.0703	2433.0619	3	1	K.MMDKVEFVHMLNATMCATTR.T	3 Oxidation (M) (Ions score 35)
416 - 435	609.2749	2433.0704	2433.0619	3	1	K.MMDKVEFVHMLNATMCATTR.T	3 Oxidation (M) (Ions score 30)
416 - 435	609.2749	2433.0704	2433.0619	3	1	K.MMDKVEFVHMLNATMCATTR.T	3 Oxidation (M) (Ions score 31)
416 - 435	812.0308	2433.0705	2433.0619	4	1	K.MMDKVEFVHMLNATMCATTR.T	3 Oxidation (M) (Ions score 33)
416 - 435	609.2749	2433.0705	2433.0619	4	1	K.MMDKVEFVHMLNATMCATTR.T	3 Oxidation (M) (Ions score 34)
416 - 435	609.2749	2433.0706	2433.0619	4	1	K.MMDKVEFVHMLNATMCATTR.T	3 Oxidation (M) (Ions score 29)
416 - 435	609.2749	2433.0707	2433.0619	4	1	K.MMDKVEFVHMLNATMCATTR.T	3 Oxidation (M) (Ions score 31)
416 - 435	812.0309	2433.0707	2433.0619	4	1	K.MMDKVEFVHMLNATMCATTR.T	3 Oxidation (M) (Ions score 34)
416 - 435	812.0309	2433.0708	2433.0619	4	1	K.MMDKVEFVHMLNATMCATTR.T	3 Oxidation (M) (Ions score 37)
416 - 435	812.0309	2433.0709	2433.0619	4	1	K.MMDKVEFVHMLNATMCATTR.T	3 Oxidation (M) (Ions score 43)
416 - 435	609.2750	2433.0709	2433.0619	4	1	K.MMDKVEFVHMLNATMCATTR.T	3 Oxidation (M) (Ions score 29)
416 - 435	609.2750	2433.0709	2433.0619	4	1	K.MMDKVEFVHMLNATMCATTR.T	3 Oxidation (M) (Ions score 29)
416 - 435	609.2750	2433.0710	2433.0619	4	1	K.MMDKVEFVHMLNATMCATTR.T	3 Oxidation (M) (Ions score 28)
416 - 435	609.2750	2433.0710	2433.0619	4	1	K.MMDKVEFVHMLNATMCATTR.T	3 Oxidation (M) (Ions score 29)
416 - 435	609.2751	2433.0713	2433.0619	4	1	K.MMDKVEFVHMLNATMCATTR.T	3 Oxidation (M) (Ions score 26)
416 - 435	812.0311	2433.0714	2433.0619	4	1	K.MMDKVEFVHMLNATMCATTR.T	3 Oxidation (M) (Ions score 26)
416 - 435	609.2751	2433.0714	2433.0619	4	1	K.MMDKVEFVHMLNATMCATTR.T	3 Oxidation (M) (Ions score 27)
416 - 435	812.0311	2433.0714	2433.0619	4	1	K.MMDKVEFVHMLNATMCATTR.T	3 Oxidation (M) (Ions score 49)
416 - 435	812.0311	2433.0714	2433.0619	4	1	K.MMDKVEFVHMLNATMCATTR.T	3 Oxidation (M) (Ions score 35)
416 - 435	609.2751	2433.0715	2433.0619	4	1	K.MMDKVEFVHMLNATMCATTR.T	3 Oxidation (M) (Ions score 34)
416 - 435	609.2752	2433.0716	2433.0619	4	1	K.MMDKVEFVHMLNATMCATTR.T	3 Oxidation (M) (Ions score 31)
416 - 435	1217.5431	2433.0717	2433.0619	4	1	K.MMDKVEFVHMLNATMCATTR.T	3 Oxidation (M) (Ions score 25)
416 - 435	812.0312	2433.0719	2433.0619	4	1	K.MMDKVEFVHMLNATMCATTR.T	3 Oxidation (M) (Ions score 53)
416 - 435	609.2753	2433.0719	2433.0619	4	1	K.MMDKVEFVHMLNATMCATTR.T	3 Oxidation (M) (Ions score 21)

416 - 435	812.0312	2433.0719	2433.0619	4	1	K.MMDKVEFVHMLNATMCATTR.T	3 Oxidation (M)	(Ions score 34)
416 - 435	609.2753	2433.0720	2433.0619	4	1	K.MMDKVEFVHMLNATMCATTR.T	3 Oxidation (M)	(Ions score 30)
416 - 435	609.2753	2433.0720	2433.0619	4	1	K.MMDKVEFVHMLNATMCATTR.T	3 Oxidation (M)	(Ions score 23)
416 - 435	609.2753	2433.0720	2433.0619	4	1	K.MMDKVEFVHMLNATMCATTR.T	3 Oxidation (M)	(Ions score 22)
416 - 435	812.0313	2433.0720	2433.0619	4	1	K.MMDKVEFVHMLNATMCATTR.T	3 Oxidation (M)	(Ions score 36)
416 - 435	609.2753	2433.0721	2433.0619	4	1	K.MMDKVEFVHMLNATMCATTR.T	3 Oxidation (M)	(Ions score 28)
416 - 435	609.2753	2433.0721	2433.0619	4	1	K.MMDKVEFVHMLNATMCATTR.T	3 Oxidation (M)	(Ions score 27)
416 - 435	812.0313	2433.0722	2433.0619	4	1	K.MMDKVEFVHMLNATMCATTR.T	3 Oxidation (M)	(Ions score 40)
416 - 435	812.0314	2433.0722	2433.0619	4	1	K.MMDKVEFVHMLNATMCATTR.T	3 Oxidation (M)	(Ions score 47)
416 - 435	812.0314	2433.0722	2433.0619	4	1	K.MMDKVEFVHMLNATMCATTR.T	3 Oxidation (M)	(Ions score 42)
416 - 435	609.2754	2433.0723	2433.0619	4	1	K.MMDKVEFVHMLNATMCATTR.T	3 Oxidation (M)	(Ions score 27)
416 - 435	609.2754	2433.0724	2433.0619	4	1	K.MMDKVEFVHMLNATMCATTR.T	3 Oxidation (M)	(Ions score 31)
416 - 435	812.0314	2433.0724	2433.0619	4	1	K.MMDKVEFVHMLNATMCATTR.T	3 Oxidation (M)	(Ions score 35)
416 - 435	812.0314	2433.0724	2433.0619	4	1	K.MMDKVEFVHMLNATMCATTR.T	3 Oxidation (M)	(Ions score 35)
416 - 435	609.2754	2433.0725	2433.0619	4	1	K.MMDKVEFVHMLNATMCATTR.T	3 Oxidation (M)	(Ions score 29)
416 - 435	812.0315	2433.0728	2433.0619	4	1	K.MMDKVEFVHMLNATMCATTR.T	3 Oxidation (M)	(Ions score 22)
416 - 435	812.0316	2433.0729	2433.0619	4	1	K.MMDKVEFVHMLNATMCATTR.T	3 Oxidation (M)	(Ions score 32)
416 - 435	812.0316	2433.0729	2433.0619	4	1	K.MMDKVEFVHMLNATMCATTR.T	3 Oxidation (M)	(Ions score 38)
416 - 435	812.0316	2433.0729	2433.0619	5	1	K.MMDKVEFVHMLNATMCATTR.T	3 Oxidation (M)	(Ions score 42)
416 - 435	812.0316	2433.0730	2433.0619	5	1	K.MMDKVEFVHMLNATMCATTR.T	3 Oxidation (M)	(Ions score 55)
416 - 435	812.0317	2433.0732	2433.0619	5	1	K.MMDKVEFVHMLNATMCATTR.T	3 Oxidation (M)	(Ions score 42)
416 - 435	812.0317	2433.0733	2433.0619	5	1	K.MMDKVEFVHMLNATMCATTR.T	3 Oxidation (M)	(Ions score 42)
416 - 435	812.0318	2433.0736	2433.0619	5	1	K.MMDKVEFVHMLNATMCATTR.T	3 Oxidation (M)	(Ions score 35)
416 - 435	812.0319	2433.0737	2433.0619	5	1	K.MMDKVEFVHMLNATMCATTR.T	3 Oxidation (M)	(Ions score 41)
416 - 435	812.0319	2433.0739	2433.0619	5	1	K.MMDKVEFVHMLNATMCATTR.T	3 Oxidation (M)	(Ions score 26)
416 - 435	812.0319	2433.0740	2433.0619	5	1	K.MMDKVEFVHMLNATMCATTR.T	3 Oxidation (M)	(Ions score 40)
416 - 435	611.7761	2443.0754	2443.0826	-3	1	K.MMDKVEFVHMLNATMCATTR.T	Acetylation (K); Oxidation (M)	(Ions score 61)
416 - 435	611.7786	2443.0854	2443.0826	1	1	K.MMDKVEFVHMLNATMCATTR.T	Acetylation (K); Oxidation (M)	(Ions score 23)
416 - 435	815.3691	2443.0855	2443.0826	1	1	K.MMDKVEFVHMLNATMCATTR.T	Acetylation (K); Oxidation (M)	(Ions score 56)
416 - 435	815.3697	2443.0873	2443.0826	2	1	K.MMDKVEFVHMLNATMCATTR.T	Acetylation (K); Oxidation (M)	(Ions score 39)
416 - 435	815.3702	2443.0887	2443.0826	2	1	K.MMDKVEFVHMLNATMCATTR.T	Acetylation (K); Oxidation (M)	(Ions score 27)
416 - 435	815.3702	2443.0888	2443.0826	3	1	K.MMDKVEFVHMLNATMCATTR.T	Acetylation (K); Oxidation (M)	(Ions score 42)
416 - 435	815.3710	2443.0912	2443.0826	4	1	K.MMDKVEFVHMLNATMCATTR.T	Acetylation (K); Oxidation (M)	(Ions score 37)
416 - 435	817.3590	2449.0552	2449.0568	-1	1	K.MMDKVEFVHMLNATMCATTR.T	4 Oxidation (M)	(Ions score 32)
416 - 435	817.3596	2449.0570	2449.0568	0	1	K.MMDKVEFVHMLNATMCATTR.T	4 Oxidation (M)	(Ions score 23)
416 - 435	817.3597	2449.0572	2449.0568	0	1	K.MMDKVEFVHMLNATMCATTR.T	4 Oxidation (M)	(Ions score 28)
416 - 435	613.2716	2449.0573	2449.0568	0	1	K.MMDKVEFVHMLNATMCATTR.T	4 Oxidation (M)	(Ions score 21)
416 - 435	1225.5360	2449.0575	2449.0568	0	1	K.MMDKVEFVHMLNATMCATTR.T	4 Oxidation (M)	(Ions score 37)
416 - 435	613.2717	2449.0578	2449.0568	0	1	K.MMDKVEFVHMLNATMCATTR.T	4 Oxidation (M)	(Ions score 21)
416 - 435	613.2718	2449.0579	2449.0568	0	1	K.MMDKVEFVHMLNATMCATTR.T	4 Oxidation (M)	(Ions score 21)
416 - 435	613.2718	2449.0582	2449.0568	1	1	K.MMDKVEFVHMLNATMCATTR.T	4 Oxidation (M)	(Ions score 21)
416 - 435	817.3602	2449.0587	2449.0568	1	1	K.MMDKVEFVHMLNATMCATTR.T	4 Oxidation (M)	(Ions score 22)
416 - 435	817.3603	2449.0590	2449.0568	1	1	K.MMDKVEFVHMLNATMCATTR.T	4 Oxidation (M)	(Ions score 42)
416 - 435	613.2721	2449.0591	2449.0568	1	1	K.MMDKVEFVHMLNATMCATTR.T	4 Oxidation (M)	(Ions score 21)
416 - 435	613.2721	2449.0593	2449.0568	1	1	K.MMDKVEFVHMLNATMCATTR.T	4 Oxidation (M)	(Ions score 22)
416 - 435	1225.5369	2449.0593	2449.0568	1	1	K.MMDKVEFVHMLNATMCATTR.T	4 Oxidation (M)	(Ions score 31)
416 - 435	613.2722	2449.0598	2449.0568	1	1	K.MMDKVEFVHMLNATMCATTR.T	4 Oxidation (M)	(Ions score 22)
416 - 435	613.2728	2449.0619	2449.0568	2	1	K.MMDKVEFVHMLNATMCATTR.T	4 Oxidation (M)	(Ions score 21)
416 - 435	817.3614	2449.0623	2449.0568	2	1	K.MMDKVEFVHMLNATMCATTR.T	4 Oxidation (M)	(Ions score 27)
416 - 435	817.3615	2449.0628	2449.0568	2	1	K.MMDKVEFVHMLNATMCATTR.T	4 Oxidation (M)	(Ions score 23)
416 - 435	613.2730	2449.0629	2449.0568	2	1	K.MMDKVEFVHMLNATMCATTR.T	4 Oxidation (M)	(Ions score 22)
416 - 435	613.2730	2449.0630	2449.0568	3	1	K.MMDKVEFVHMLNATMCATTR.T	4 Oxidation (M)	(Ions score 22)
416 - 435	613.2730	2449.0630	2449.0568	3	1	K.MMDKVEFVHMLNATMCATTR.T	4 Oxidation (M)	(Ions score 21)
416 - 435	613.2731	2449.0632	2449.0568	3	1	K.MMDKVEFVHMLNATMCATTR.T	4 Oxidation (M)	(Ions score 21)
416 - 435	613.2731	2449.0633	2449.0568	3	1	K.MMDKVEFVHMLNATMCATTR.T	4 Oxidation (M)	(Ions score 21)
416 - 435	817.3618	2449.0635	2449.0568	3	1	K.MMDKVEFVHMLNATMCATTR.T	4 Oxidation (M)	(Ions score 24)
416 - 435	817.3618	2449.0635	2449.0568	3	1	K.MMDKVEFVHMLNATMCATTR.T	4 Oxidation (M)	(Ions score 42)
416 - 435	817.3619	2449.0638	2449.0568	3	1	K.MMDKVEFVHMLNATMCATTR.T	4 Oxidation (M)	(Ions score 23)

416 - 435	613.2732	2449.0639	2449.0568	3	1	K.MMDKVEFVHMLNATMCATTR.T	4 Oxidation (M) (Ions score 21)
416 - 435	613.2733	2449.0640	2449.0568	3	1	K.MMDKVEFVHMLNATMCATTR.T	4 Oxidation (M) (Ions score 22)
416 - 435	817.3621	2449.0644	2449.0568	3	1	K.MMDKVEFVHMLNATMCATTR.T	4 Oxidation (M) (Ions score 33)
416 - 435	613.2734	2449.0644	2449.0568	3	1	K.MMDKVEFVHMLNATMCATTR.T	4 Oxidation (M) (Ions score 22)
416 - 435	613.2734	2449.0645	2449.0568	3	1	K.MMDKVEFVHMLNATMCATTR.T	4 Oxidation (M) (Ions score 21)
416 - 435	613.2736	2449.0655	2449.0568	4	1	K.MMDKVEFVHMLNATMCATTR.T	4 Oxidation (M) (Ions score 22)
416 - 435	613.2737	2449.0655	2449.0568	4	1	K.MMDKVEFVHMLNATMCATTR.T	4 Oxidation (M) (Ions score 22)
416 - 435	613.2737	2449.0656	2449.0568	4	1	K.MMDKVEFVHMLNATMCATTR.T	4 Oxidation (M) (Ions score 22)
416 - 435	613.2737	2449.0657	2449.0568	4	1	K.MMDKVEFVHMLNATMCATTR.T	4 Oxidation (M) (Ions score 22)
416 - 435	817.3625	2449.0658	2449.0568	4	1	K.MMDKVEFVHMLNATMCATTR.T	4 Oxidation (M) (Ions score 21)
416 - 435	613.2738	2449.0660	2449.0568	4	1	K.MMDKVEFVHMLNATMCATTR.T	4 Oxidation (M) (Ions score 22)
416 - 435	613.2739	2449.0667	2449.0568	4	1	K.MMDKVEFVHMLNATMCATTR.T	4 Oxidation (M) (Ions score 21)
416 - 435	613.2740	2449.0671	2449.0568	4	1	K.MMDKVEFVHMLNATMCATTR.T	4 Oxidation (M) (Ions score 22)
416 - 435	817.3630	2449.0673	2449.0568	4	1	K.MMDKVEFVHMLNATMCATTR.T	4 Oxidation (M) (Ions score 31)
416 - 435	613.2745	2449.0691	2449.0568	5	1	K.MMDKVEFVHMLNATMCATTR.T	4 Oxidation (M) (Ions score 22)
416 - 435	820.6987	2459.0742	2459.0776	-1	1	K.MMDKVEFVHMLNATMCATTR.T	Acetylation (K); 2 Oxidation (M) (Ions score 47)
416 - 435	820.6993	2459.0760	2459.0776	-1	1	K.MMDKVEFVHMLNATMCATTR.T	Acetylation (K); 2 Oxidation (M) (Ions score 74)
416 - 435	615.7765	2459.0768	2459.0776	-0	1	K.MMDKVEFVHMLNATMCATTR.T	Acetylation (K); 2 Oxidation (M) (Ions score 34)
416 - 435	820.7001	2459.0784	2459.0776	0	1	K.MMDKVEFVHMLNATMCATTR.T	Acetylation (K); 2 Oxidation (M) (Ions score 51)
416 - 435	820.7004	2459.0794	2459.0776	1	1	K.MMDKVEFVHMLNATMCATTR.T	Acetylation (K); 2 Oxidation (M) (Ions score 87)
416 - 435	820.7005	2459.0798	2459.0776	1	1	K.MMDKVEFVHMLNATMCATTR.T	Acetylation (K); 2 Oxidation (M) (Ions score 77)
416 - 435	820.7007	2459.0802	2459.0776	1	1	K.MMDKVEFVHMLNATMCATTR.T	Acetylation (K); 2 Oxidation (M) (Ions score 39)
416 - 435	820.7009	2459.0808	2459.0776	1	1	K.MMDKVEFVHMLNATMCATTR.T	Acetylation (K); 2 Oxidation (M) (Ions score 62)
416 - 435	820.7010	2459.0811	2459.0776	1	1	K.MMDKVEFVHMLNATMCATTR.T	Acetylation (K); 2 Oxidation (M) (Ions score 51)
416 - 435	820.7016	2459.0830	2459.0776	2	1	K.MMDKVEFVHMLNATMCATTR.T	Acetylation (K); 2 Oxidation (M) (Ions score 32)
416 - 435	820.7016	2459.0831	2459.0776	2	1	K.MMDKVEFVHMLNATMCATTR.T	Acetylation (K); 2 Oxidation (M) (Ions score 34)
416 - 435	615.7781	2459.0833	2459.0776	2	1	K.MMDKVEFVHMLNATMCATTR.T	Acetylation (K); 2 Oxidation (M) (Ions score 25)
416 - 435	820.7018	2459.0837	2459.0776	2	1	K.MMDKVEFVHMLNATMCATTR.T	Acetylation (K); 2 Oxidation (M) (Ions score 44)
416 - 435	615.7785	2459.0847	2459.0776	3	1	K.MMDKVEFVHMLNATMCATTR.T	Acetylation (K); 2 Oxidation (M) (Ions score 51)
416 - 435	615.7785	2459.0847	2459.0776	3	1	K.MMDKVEFVHMLNATMCATTR.T	Acetylation (K); 2 Oxidation (M) (Ions score 45)
416 - 435	615.7788	2459.0861	2459.0776	3	1	K.MMDKVEFVHMLNATMCATTR.T	Acetylation (K); 2 Oxidation (M) (Ions score 25)
416 - 435	820.7029	2459.0869	2459.0776	4	1	K.MMDKVEFVHMLNATMCATTR.T	Acetylation (K); 2 Oxidation (M) (Ions score 36)
416 - 435	615.7790	2459.0869	2459.0776	4	1	K.MMDKVEFVHMLNATMCATTR.T	Acetylation (K); 2 Oxidation (M) (Ions score 24)
416 - 435	615.7793	2459.0880	2459.0776	4	1	K.MMDKVEFVHMLNATMCATTR.T	Acetylation (K); 2 Oxidation (M) (Ions score 25)
416 - 435	615.7794	2459.0883	2459.0776	4	1	K.MMDKVEFVHMLNATMCATTR.T	Acetylation (K); 2 Oxidation (M) (Ions score 29)
416 - 435	615.7795	2459.0891	2459.0776	5	1	K.MMDKVEFVHMLNATMCATTR.T	Acetylation (K); 2 Oxidation (M) (Ions score 24)
416 - 435	615.7797	2459.0897	2459.0776	5	1	K.MMDKVEFVHMLNATMCATTR.T	Acetylation (K); 2 Oxidation (M) (Ions score 29)
416 - 435	619.7747	2475.0695	2475.0725	-1	1	K.MMDKVEFVHMLNATMCATTR.T	Acetylation (K); 3 Oxidation (M) (Ions score 28)
416 - 435	826.0308	2475.0706	2475.0725	-1	1	K.MMDKVEFVHMLNATMCATTR.T	Acetylation (K); 3 Oxidation (M) (Ions score 24)
416 - 435	826.0310	2475.0712	2475.0725	-1	1	K.MMDKVEFVHMLNATMCATTR.T	Acetylation (K); 3 Oxidation (M) (Ions score 38)
416 - 435	619.7751	2475.0715	2475.0725	-0	1	K.MMDKVEFVHMLNATMCATTR.T	Acetylation (K); 3 Oxidation (M) (Ions score 24)
416 - 435	826.0315	2475.0728	2475.0725	0	1	K.MMDKVEFVHMLNATMCATTR.T	Acetylation (K); 3 Oxidation (M) (Ions score 47)
416 - 435	826.0321	2475.0746	2475.0725	1	1	K.MMDKVEFVHMLNATMCATTR.T	Acetylation (K); 3 Oxidation (M) (Ions score 31)
416 - 435	826.0323	2475.0752	2475.0725	1	1	K.MMDKVEFVHMLNATMCATTR.T	Acetylation (K); 3 Oxidation (M) (Ions score 62)
416 - 435	826.0331	2475.0776	2475.0725	2	1	K.MMDKVEFVHMLNATMCATTR.T	Acetylation (K); 3 Oxidation (M) (Ions score 24)
416 - 435	826.0335	2475.0788	2475.0725	3	1	K.MMDKVEFVHMLNATMCATTR.T	Acetylation (K); 3 Oxidation (M) (Ions score 78)
416 - 435	826.0337	2475.0794	2475.0725	3	1	K.MMDKVEFVHMLNATMCATTR.T	Acetylation (K); 3 Oxidation (M) (Ions score 47)
416 - 435	619.7774	2475.0805	2475.0725	3	1	K.MMDKVEFVHMLNATMCATTR.T	Acetylation (K); 3 Oxidation (M) (Ions score 22)
416 - 435	826.0342	2475.0809	2475.0725	3	1	K.MMDKVEFVHMLNATMCATTR.T	Acetylation (K); 3 Oxidation (M) (Ions score 50)
416 - 435	826.0347	2475.0821	2475.0725	4	1	K.MMDKVEFVHMLNATMCATTR.T	Acetylation (K); 3 Oxidation (M) (Ions score 72)
416 - 435	826.0347	2475.0824	2475.0725	4	1	K.MMDKVEFVHMLNATMCATTR.T	Acetylation (K); 3 Oxidation (M) (Ions score 23)
416 - 435	826.0348	2475.0826	2475.0725	4	1	K.MMDKVEFVHMLNATMCATTR.T	Acetylation (K); 3 Oxidation (M) (Ions score 77)
416 - 435	619.7780	2475.0827	2475.0725	4	1	K.MMDKVEFVHMLNATMCATTR.T	Acetylation (K); 3 Oxidation (M) (Ions score 28)
416 - 435	619.7781	2475.0832	2475.0725	4	1	K.MMDKVEFVHMLNATMCATTR.T	Acetylation (K); 3 Oxidation (M) (Ions score 28)
416 - 435	826.0354	2475.0844	2475.0725	5	1	K.MMDKVEFVHMLNATMCATTR.T	Acetylation (K); 3 Oxidation (M) (Ions score 66)
416 - 435	831.3638	2491.0697	2491.0674	1	1	K.MMDKVEFVHMLNATMCATTR.T	Acetylation (K); 4 Oxidation (M) (Ions score 24)
420 - 435	940.9414	1879.8683	1879.8743	-3	0	K.VEFVHMLNATMCATTR.T	(Ions score 77)
420 - 435	627.6304	1879.8695	1879.8743	-3	0	K.VEFVHMLNATMCATTR.T	(Ions score 73)

420 - 435	940.9429	1879.8713	1879.8743	-2	0	K.VEFVHMLNATMCATTR.T	(Ions score 77)
420 - 435	940.9430	1879.8715	1879.8743	-1	0	K.VEFVHMLNATMCATTR.T	(Ions score 40)
420 - 435	627.6312	1879.8716	1879.8743	-1	0	K.VEFVHMLNATMCATTR.T	(Ions score 64)
420 - 435	940.9431	1879.8717	1879.8743	-1	0	K.VEFVHMLNATMCATTR.T	(Ions score 54)
420 - 435	940.9431	1879.8717	1879.8743	-1	0	K.VEFVHMLNATMCATTR.T	(Ions score 54)
420 - 435	940.9432	1879.8719	1879.8743	-1	0	K.VEFVHMLNATMCATTR.T	(Ions score 45)
420 - 435	940.9432	1879.8719	1879.8743	-1	0	K.VEFVHMLNATMCATTR.T	(Ions score 36)
420 - 435	940.9433	1879.8721	1879.8743	-1	0	K.VEFVHMLNATMCATTR.T	(Ions score 31)
420 - 435	627.6313	1879.8722	1879.8743	-1	0	K.VEFVHMLNATMCATTR.T	(Ions score 75)
420 - 435	627.6314	1879.8722	1879.8743	-1	0	K.VEFVHMLNATMCATTR.T	(Ions score 62)
420 - 435	627.6314	1879.8723	1879.8743	-1	0	K.VEFVHMLNATMCATTR.T	(Ions score 62)
420 - 435	627.6315	1879.8726	1879.8743	-1	0	K.VEFVHMLNATMCATTR.T	(Ions score 81)
420 - 435	627.6316	1879.8730	1879.8743	-1	0	K.VEFVHMLNATMCATTR.T	(Ions score 83)
420 - 435	627.6317	1879.8733	1879.8743	-1	0	K.VEFVHMLNATMCATTR.T	(Ions score 65)
420 - 435	627.6317	1879.8733	1879.8743	-1	0	K.VEFVHMLNATMCATTR.T	(Ions score 76)
420 - 435	940.9440	1879.8735	1879.8743	-0	0	K.VEFVHMLNATMCATTR.T	(Ions score 76)
420 - 435	627.6318	1879.8736	1879.8743	-0	0	K.VEFVHMLNATMCATTR.T	(Ions score 68)
420 - 435	940.9441	1879.8736	1879.8743	-0	0	K.VEFVHMLNATMCATTR.T	(Ions score 57)
420 - 435	627.6318	1879.8737	1879.8743	-0	0	K.VEFVHMLNATMCATTR.T	(Ions score 79)
420 - 435	627.6319	1879.8739	1879.8743	-0	0	K.VEFVHMLNATMCATTR.T	(Ions score 59)
420 - 435	627.6320	1879.8740	1879.8743	-0	0	K.VEFVHMLNATMCATTR.T	(Ions score 65)
420 - 435	627.6320	1879.8741	1879.8743	-0	0	K.VEFVHMLNATMCATTR.T	(Ions score 90)
420 - 435	940.9443	1879.8741	1879.8743	-0	0	K.VEFVHMLNATMCATTR.T	(Ions score 61)
420 - 435	627.6320	1879.8742	1879.8743	-0	0	K.VEFVHMLNATMCATTR.T	(Ions score 54)
420 - 435	940.9444	1879.8742	1879.8743	-0	0	K.VEFVHMLNATMCATTR.T	(Ions score 67)
420 - 435	627.6321	1879.8743	1879.8743	0	0	K.VEFVHMLNATMCATTR.T	(Ions score 31)
420 - 435	627.6321	1879.8743	1879.8743	0	0	K.VEFVHMLNATMCATTR.T	(Ions score 63)
420 - 435	627.6321	1879.8744	1879.8743	0	0	K.VEFVHMLNATMCATTR.T	(Ions score 79)
420 - 435	940.9445	1879.8745	1879.8743	0	0	K.VEFVHMLNATMCATTR.T	(Ions score 58)
420 - 435	940.9445	1879.8745	1879.8743	0	0	K.VEFVHMLNATMCATTR.T	(Ions score 74)
420 - 435	627.6321	1879.8745	1879.8743	0	0	K.VEFVHMLNATMCATTR.T	(Ions score 89)
420 - 435	627.6321	1879.8746	1879.8743	0	0	K.VEFVHMLNATMCATTR.T	(Ions score 78)
420 - 435	940.9446	1879.8746	1879.8743	0	0	K.VEFVHMLNATMCATTR.T	(Ions score 76)
420 - 435	940.9446	1879.8746	1879.8743	0	0	K.VEFVHMLNATMCATTR.T	(Ions score 52)
420 - 435	627.6322	1879.8748	1879.8743	0	0	K.VEFVHMLNATMCATTR.T	(Ions score 75)
420 - 435	627.6323	1879.8749	1879.8743	0	0	K.VEFVHMLNATMCATTR.T	(Ions score 85)
420 - 435	940.9448	1879.8750	1879.8743	0	0	K.VEFVHMLNATMCATTR.T	(Ions score 59)
420 - 435	940.9448	1879.8751	1879.8743	0	0	K.VEFVHMLNATMCATTR.T	(Ions score 62)
420 - 435	940.9449	1879.8752	1879.8743	0	0	K.VEFVHMLNATMCATTR.T	(Ions score 49)
420 - 435	627.6324	1879.8753	1879.8743	1	0	K.VEFVHMLNATMCATTR.T	(Ions score 74)
420 - 435	940.9449	1879.8753	1879.8743	1	0	K.VEFVHMLNATMCATTR.T	(Ions score 52)
420 - 435	627.6324	1879.8753	1879.8743	1	0	K.VEFVHMLNATMCATTR.T	(Ions score 78)
420 - 435	627.6324	1879.8754	1879.8743	1	0	K.VEFVHMLNATMCATTR.T	(Ions score 74)
420 - 435	940.9450	1879.8755	1879.8743	1	0	K.VEFVHMLNATMCATTR.T	(Ions score 58)
420 - 435	627.6324	1879.8755	1879.8743	1	0	K.VEFVHMLNATMCATTR.T	(Ions score 63)
420 - 435	627.6324	1879.8755	1879.8743	1	0	K.VEFVHMLNATMCATTR.T	(Ions score 91)
420 - 435	627.6325	1879.8756	1879.8743	1	0	K.VEFVHMLNATMCATTR.T	(Ions score 57)
420 - 435	940.9451	1879.8756	1879.8743	1	0	K.VEFVHMLNATMCATTR.T	(Ions score 64)
420 - 435	940.9451	1879.8756	1879.8743	1	0	K.VEFVHMLNATMCATTR.T	(Ions score 65)
420 - 435	627.6325	1879.8757	1879.8743	1	0	K.VEFVHMLNATMCATTR.T	(Ions score 83)
420 - 435	627.6325	1879.8757	1879.8743	1	0	K.VEFVHMLNATMCATTR.T	(Ions score 75)
420 - 435	627.6325	1879.8757	1879.8743	1	0	K.VEFVHMLNATMCATTR.T	(Ions score 49)
420 - 435	627.6325	1879.8757	1879.8743	1	0	K.VEFVHMLNATMCATTR.T	(Ions score 64)
420 - 435	940.9451	1879.8757	1879.8743	1	0	K.VEFVHMLNATMCATTR.T	(Ions score 61)
420 - 435	940.9452	1879.8758	1879.8743	1	0	K.VEFVHMLNATMCATTR.T	(Ions score 22)
420 - 435	627.6325	1879.8758	1879.8743	1	0	K.VEFVHMLNATMCATTR.T	(Ions score 90)
420 - 435	627.6325	1879.8758	1879.8743	1	0	K.VEFVHMLNATMCATTR.T	(Ions score 79)
420 - 435	940.9452	1879.8758	1879.8743	1	0	K.VEFVHMLNATMCATTR.T	(Ions score 76)

420 - 435	940.9452	1879.8758	1879.8743	1	0	K.VEFVHMLNATMCATTR.T	(Ions score 70)
420 - 435	627.6325	1879.8758	1879.8743	1	0	K.VEFVHMLNATMCATTR.T	(Ions score 79)
420 - 435	940.9452	1879.8759	1879.8743	1	0	K.VEFVHMLNATMCATTR.T	(Ions score 56)
420 - 435	940.9453	1879.8760	1879.8743	1	0	K.VEFVHMLNATMCATTR.T	(Ions score 81)
420 - 435	627.6326	1879.8760	1879.8743	1	0	K.VEFVHMLNATMCATTR.T	(Ions score 92)
420 - 435	940.9453	1879.8760	1879.8743	1	0	K.VEFVHMLNATMCATTR.T	(Ions score 89)
420 - 435	627.6326	1879.8760	1879.8743	1	0	K.VEFVHMLNATMCATTR.T	(Ions score 73)
420 - 435	627.6326	1879.8761	1879.8743	1	0	K.VEFVHMLNATMCATTR.T	(Ions score 79)
420 - 435	627.6326	1879.8761	1879.8743	1	0	K.VEFVHMLNATMCATTR.T	(Ions score 71)
420 - 435	627.6327	1879.8762	1879.8743	1	0	K.VEFVHMLNATMCATTR.T	(Ions score 75)
420 - 435	627.6327	1879.8762	1879.8743	1	0	K.VEFVHMLNATMCATTR.T	(Ions score 71)
420 - 435	627.6327	1879.8762	1879.8743	1	0	K.VEFVHMLNATMCATTR.T	(Ions score 81)
420 - 435	940.9454	1879.8762	1879.8743	1	0	K.VEFVHMLNATMCATTR.T	(Ions score 53)
420 - 435	940.9454	1879.8762	1879.8743	1	0	K.VEFVHMLNATMCATTR.T	(Ions score 65)
420 - 435	627.6327	1879.8762	1879.8743	1	0	K.VEFVHMLNATMCATTR.T	(Ions score 79)
420 - 435	627.6327	1879.8762	1879.8743	1	0	K.VEFVHMLNATMCATTR.T	(Ions score 69)
420 - 435	627.6327	1879.8763	1879.8743	1	0	K.VEFVHMLNATMCATTR.T	(Ions score 73)
420 - 435	627.6327	1879.8763	1879.8743	1	0	K.VEFVHMLNATMCATTR.T	(Ions score 70)
420 - 435	940.9454	1879.8763	1879.8743	1	0	K.VEFVHMLNATMCATTR.T	(Ions score 74)
420 - 435	940.9454	1879.8763	1879.8743	1	0	K.VEFVHMLNATMCATTR.T	(Ions score 36)
420 - 435	627.6327	1879.8763	1879.8743	1	0	K.VEFVHMLNATMCATTR.T	(Ions score 74)
420 - 435	940.9455	1879.8765	1879.8743	1	0	K.VEFVHMLNATMCATTR.T	(Ions score 80)
420 - 435	940.9455	1879.8765	1879.8743	1	0	K.VEFVHMLNATMCATTR.T	(Ions score 78)
420 - 435	940.9455	1879.8765	1879.8743	1	0	K.VEFVHMLNATMCATTR.T	(Ions score 66)
420 - 435	627.6328	1879.8765	1879.8743	1	0	K.VEFVHMLNATMCATTR.T	(Ions score 59)
420 - 435	627.6328	1879.8766	1879.8743	1	0	K.VEFVHMLNATMCATTR.T	(Ions score 57)
420 - 435	627.6328	1879.8766	1879.8743	1	0	K.VEFVHMLNATMCATTR.T	(Ions score 92)
420 - 435	627.6328	1879.8766	1879.8743	1	0	K.VEFVHMLNATMCATTR.T	(Ions score 59)
420 - 435	627.6328	1879.8766	1879.8743	1	0	K.VEFVHMLNATMCATTR.T	(Ions score 83)
420 - 435	627.6328	1879.8766	1879.8743	1	0	K.VEFVHMLNATMCATTR.T	(Ions score 85)
420 - 435	627.6328	1879.8767	1879.8743	1	0	K.VEFVHMLNATMCATTR.T	(Ions score 76)
420 - 435	627.6328	1879.8767	1879.8743	1	0	K.VEFVHMLNATMCATTR.T	(Ions score 61)
420 - 435	627.6329	1879.8768	1879.8743	1	0	K.VEFVHMLNATMCATTR.T	(Ions score 89)
420 - 435	940.9457	1879.8768	1879.8743	1	0	K.VEFVHMLNATMCATTR.T	(Ions score 71)
420 - 435	627.6329	1879.8768	1879.8743	1	0	K.VEFVHMLNATMCATTR.T	(Ions score 71)
420 - 435	940.9457	1879.8769	1879.8743	1	0	K.VEFVHMLNATMCATTR.T	(Ions score 36)
420 - 435	627.6329	1879.8769	1879.8743	1	0	K.VEFVHMLNATMCATTR.T	(Ions score 89)
420 - 435	627.6329	1879.8769	1879.8743	1	0	K.VEFVHMLNATMCATTR.T	(Ions score 79)
420 - 435	627.6329	1879.8770	1879.8743	1	0	K.VEFVHMLNATMCATTR.T	(Ions score 73)
420 - 435	627.6329	1879.8770	1879.8743	1	0	K.VEFVHMLNATMCATTR.T	(Ions score 87)
420 - 435	627.6329	1879.8770	1879.8743	1	0	K.VEFVHMLNATMCATTR.T	(Ions score 73)
420 - 435	627.6330	1879.8770	1879.8743	1	0	K.VEFVHMLNATMCATTR.T	(Ions score 73)
420 - 435	940.9458	1879.8771	1879.8743	1	0	K.VEFVHMLNATMCATTR.T	(Ions score 76)
420 - 435	940.9458	1879.8771	1879.8743	1	0	K.VEFVHMLNATMCATTR.T	(Ions score 43)
420 - 435	627.6330	1879.8771	1879.8743	2	0	K.VEFVHMLNATMCATTR.T	(Ions score 79)
420 - 435	940.9459	1879.8772	1879.8743	2	0	K.VEFVHMLNATMCATTR.T	(Ions score 69)
420 - 435	627.6330	1879.8772	1879.8743	2	0	K.VEFVHMLNATMCATTR.T	(Ions score 46)
420 - 435	627.6330	1879.8772	1879.8743	2	0	K.VEFVHMLNATMCATTR.T	(Ions score 80)
420 - 435	627.6330	1879.8772	1879.8743	2	0	K.VEFVHMLNATMCATTR.T	(Ions score 91)
420 - 435	627.6330	1879.8772	1879.8743	2	0	K.VEFVHMLNATMCATTR.T	(Ions score 69)
420 - 435	627.6330	1879.8772	1879.8743	2	0	K.VEFVHMLNATMCATTR.T	(Ions score 89)
420 - 435	940.9459	1879.8773	1879.8743	2	0	K.VEFVHMLNATMCATTR.T	(Ions score 62)
420 - 435	627.6330	1879.8773	1879.8743	2	0	K.VEFVHMLNATMCATTR.T	(Ions score 66)
420 - 435	940.9460	1879.8773	1879.8743	2	0	K.VEFVHMLNATMCATTR.T	(Ions score 79)
420 - 435	627.6331	1879.8774	1879.8743	2	0	K.VEFVHMLNATMCATTR.T	(Ions score 46)
420 - 435	940.9460	1879.8774	1879.8743	2	0	K.VEFVHMLNATMCATTR.T	(Ions score 54)
420 - 435	627.6331	1879.8774	1879.8743	2	0	K.VEFVHMLNATMCATTR.T	(Ions score 75)
420 - 435	627.6331	1879.8775	1879.8743	2	0	K.VEFVHMLNATMCATTR.T	(Ions score 71)

420 - 435	627.6331	1879.8775	1879.8743	2	0	K.VEFVHMLNATMCATTR.T	(Ions score 73)
420 - 435	627.6331	1879.8775	1879.8743	2	0	K.VEFVHMLNATMCATTR.T	(Ions score 87)
420 - 435	627.6331	1879.8775	1879.8743	2	0	K.VEFVHMLNATMCATTR.T	(Ions score 78)
420 - 435	627.6331	1879.8775	1879.8743	2	0	K.VEFVHMLNATMCATTR.T	(Ions score 82)
420 - 435	627.6331	1879.8775	1879.8743	2	0	K.VEFVHMLNATMCATTR.T	(Ions score 80)
420 - 435	940.9461	1879.8776	1879.8743	2	0	K.VEFVHMLNATMCATTR.T	(Ions score 61)
420 - 435	627.6332	1879.8776	1879.8743	2	0	K.VEFVHMLNATMCATTR.T	(Ions score 83)
420 - 435	627.6332	1879.8777	1879.8743	2	0	K.VEFVHMLNATMCATTR.T	(Ions score 63)
420 - 435	627.6332	1879.8777	1879.8743	2	0	K.VEFVHMLNATMCATTR.T	(Ions score 74)
420 - 435	627.6332	1879.8777	1879.8743	2	0	K.VEFVHMLNATMCATTR.T	(Ions score 83)
420 - 435	627.6332	1879.8777	1879.8743	2	0	K.VEFVHMLNATMCATTR.T	(Ions score 60)
420 - 435	627.6332	1879.8777	1879.8743	2	0	K.VEFVHMLNATMCATTR.T	(Ions score 75)
420 - 435	940.9461	1879.8777	1879.8743	2	0	K.VEFVHMLNATMCATTR.T	(Ions score 48)
420 - 435	940.9462	1879.8778	1879.8743	2	0	K.VEFVHMLNATMCATTR.T	(Ions score 79)
420 - 435	627.6332	1879.8778	1879.8743	2	0	K.VEFVHMLNATMCATTR.T	(Ions score 91)
420 - 435	940.9462	1879.8778	1879.8743	2	0	K.VEFVHMLNATMCATTR.T	(Ions score 62)
420 - 435	940.9462	1879.8778	1879.8743	2	0	K.VEFVHMLNATMCATTR.T	(Ions score 61)
420 - 435	627.6332	1879.8778	1879.8743	2	0	K.VEFVHMLNATMCATTR.T	(Ions score 73)
420 - 435	627.6332	1879.8778	1879.8743	2	0	K.VEFVHMLNATMCATTR.T	(Ions score 90)
420 - 435	627.6332	1879.8778	1879.8743	2	0	K.VEFVHMLNATMCATTR.T	(Ions score 79)
420 - 435	627.6332	1879.8778	1879.8743	2	0	K.VEFVHMLNATMCATTR.T	(Ions score 69)
420 - 435	627.6332	1879.8779	1879.8743	2	0	K.VEFVHMLNATMCATTR.T	(Ions score 65)
420 - 435	627.6332	1879.8779	1879.8743	2	0	K.VEFVHMLNATMCATTR.T	(Ions score 71)
420 - 435	627.6332	1879.8779	1879.8743	2	0	K.VEFVHMLNATMCATTR.T	(Ions score 42)
420 - 435	627.6332	1879.8779	1879.8743	2	0	K.VEFVHMLNATMCATTR.T	(Ions score 83)
420 - 435	627.6333	1879.8779	1879.8743	2	0	K.VEFVHMLNATMCATTR.T	(Ions score 83)
420 - 435	940.9463	1879.8780	1879.8743	2	0	K.VEFVHMLNATMCATTR.T	(Ions score 58)
420 - 435	940.9463	1879.8780	1879.8743	2	0	K.VEFVHMLNATMCATTR.T	(Ions score 34)
420 - 435	627.6333	1879.8780	1879.8743	2	0	K.VEFVHMLNATMCATTR.T	(Ions score 70)
420 - 435	627.6333	1879.8780	1879.8743	2	0	K.VEFVHMLNATMCATTR.T	(Ions score 54)
420 - 435	940.9463	1879.8780	1879.8743	2	0	K.VEFVHMLNATMCATTR.T	(Ions score 41)
420 - 435	627.6333	1879.8780	1879.8743	2	0	K.VEFVHMLNATMCATTR.T	(Ions score 87)
420 - 435	940.9463	1879.8781	1879.8743	2	0	K.VEFVHMLNATMCATTR.T	(Ions score 55)
420 - 435	940.9463	1879.8781	1879.8743	2	0	K.VEFVHMLNATMCATTR.T	(Ions score 53)
420 - 435	627.6333	1879.8781	1879.8743	2	0	K.VEFVHMLNATMCATTR.T	(Ions score 79)
420 - 435	940.9463	1879.8781	1879.8743	2	0	K.VEFVHMLNATMCATTR.T	(Ions score 60)
420 - 435	940.9463	1879.8781	1879.8743	2	0	K.VEFVHMLNATMCATTR.T	(Ions score 79)
420 - 435	627.6333	1879.8781	1879.8743	2	0	K.VEFVHMLNATMCATTR.T	(Ions score 69)
420 - 435	627.6333	1879.8782	1879.8743	2	0	K.VEFVHMLNATMCATTR.T	(Ions score 75)
420 - 435	627.6333	1879.8782	1879.8743	2	0	K.VEFVHMLNATMCATTR.T	(Ions score 87)
420 - 435	627.6333	1879.8782	1879.8743	2	0	K.VEFVHMLNATMCATTR.T	(Ions score 77)
420 - 435	627.6333	1879.8782	1879.8743	2	0	K.VEFVHMLNATMCATTR.T	(Ions score 70)
420 - 435	627.6333	1879.8782	1879.8743	2	0	K.VEFVHMLNATMCATTR.T	(Ions score 70)
420 - 435	627.6334	1879.8782	1879.8743	2	0	K.VEFVHMLNATMCATTR.T	(Ions score 82)
420 - 435	627.6334	1879.8783	1879.8743	2	0	K.VEFVHMLNATMCATTR.T	(Ions score 57)
420 - 435	627.6334	1879.8783	1879.8743	2	0	K.VEFVHMLNATMCATTR.T	(Ions score 66)
420 - 435	627.6334	1879.8783	1879.8743	2	0	K.VEFVHMLNATMCATTR.T	(Ions score 70)
420 - 435	627.6334	1879.8783	1879.8743	2	0	K.VEFVHMLNATMCATTR.T	(Ions score 55)
420 - 435	940.9464	1879.8783	1879.8743	2	0	K.VEFVHMLNATMCATTR.T	(Ions score 64)
420 - 435	627.6334	1879.8783	1879.8743	2	0	K.VEFVHMLNATMCATTR.T	(Ions score 71)
420 - 435	940.9464	1879.8783	1879.8743	2	0	K.VEFVHMLNATMCATTR.T	(Ions score 45)
420 - 435	940.9464	1879.8783	1879.8743	2	0	K.VEFVHMLNATMCATTR.T	(Ions score 79)
420 - 435	627.6334	1879.8784	1879.8743	2	0	K.VEFVHMLNATMCATTR.T	(Ions score 79)
420 - 435	627.6334	1879.8784	1879.8743	2	0	K.VEFVHMLNATMCATTR.T	(Ions score 79)
420 - 435	627.6334	1879.8784	1879.8743	2	0	K.VEFVHMLNATMCATTR.T	(Ions score 76)
420 - 435	627.6334	1879.8784	1879.8743	2	0	K.VEFVHMLNATMCATTR.T	(Ions score 72)
420 - 435	627.6334	1879.8784	1879.8743	2	0	K.VEFVHMLNATMCATTR.T	(Ions score 69)
420 - 435	627.6334	1879.8785	1879.8743	2	0	K.VEFVHMLNATMCATTR.T	(Ions score 75)

420 - 435	627.6334	1879.8785	1879.8743	2	0	K.VEFVHMLNATMCATTR.T	(Ions score 79)
420 - 435	940.9465	1879.8785	1879.8743	2	0	K.VEFVHMLNATMCATTR.T	(Ions score 80)
420 - 435	627.6334	1879.8785	1879.8743	2	0	K.VEFVHMLNATMCATTR.T	(Ions score 74)
420 - 435	627.6334	1879.8785	1879.8743	2	0	K.VEFVHMLNATMCATTR.T	(Ions score 74)
420 - 435	627.6335	1879.8785	1879.8743	2	0	K.VEFVHMLNATMCATTR.T	(Ions score 69)
420 - 435	627.6335	1879.8786	1879.8743	2	0	K.VEFVHMLNATMCATTR.T	(Ions score 72)
420 - 435	627.6335	1879.8786	1879.8743	2	0	K.VEFVHMLNATMCATTR.T	(Ions score 79)
420 - 435	627.6335	1879.8786	1879.8743	2	0	K.VEFVHMLNATMCATTR.T	(Ions score 64)
420 - 435	627.6335	1879.8786	1879.8743	2	0	K.VEFVHMLNATMCATTR.T	(Ions score 85)
420 - 435	627.6335	1879.8786	1879.8743	2	0	K.VEFVHMLNATMCATTR.T	(Ions score 60)
420 - 435	940.9466	1879.8786	1879.8743	2	0	K.VEFVHMLNATMCATTR.T	(Ions score 46)
420 - 435	627.6335	1879.8786	1879.8743	2	0	K.VEFVHMLNATMCATTR.T	(Ions score 70)
420 - 435	940.9466	1879.8787	1879.8743	2	0	K.VEFVHMLNATMCATTR.T	(Ions score 68)
420 - 435	627.6335	1879.8787	1879.8743	2	0	K.VEFVHMLNATMCATTR.T	(Ions score 72)
420 - 435	940.9466	1879.8787	1879.8743	2	0	K.VEFVHMLNATMCATTR.T	(Ions score 35)
420 - 435	627.6335	1879.8787	1879.8743	2	0	K.VEFVHMLNATMCATTR.T	(Ions score 69)
420 - 435	627.6335	1879.8787	1879.8743	2	0	K.VEFVHMLNATMCATTR.T	(Ions score 87)
420 - 435	627.6335	1879.8787	1879.8743	2	0	K.VEFVHMLNATMCATTR.T	(Ions score 71)
420 - 435	627.6335	1879.8787	1879.8743	2	0	K.VEFVHMLNATMCATTR.T	(Ions score 77)
420 - 435	627.6335	1879.8787	1879.8743	2	0	K.VEFVHMLNATMCATTR.T	(Ions score 79)
420 - 435	627.6335	1879.8787	1879.8743	2	0	K.VEFVHMLNATMCATTR.T	(Ions score 71)
420 - 435	627.6335	1879.8788	1879.8743	2	0	K.VEFVHMLNATMCATTR.T	(Ions score 64)
420 - 435	627.6335	1879.8788	1879.8743	2	0	K.VEFVHMLNATMCATTR.T	(Ions score 60)
420 - 435	940.9467	1879.8789	1879.8743	2	0	K.VEFVHMLNATMCATTR.T	(Ions score 67)
420 - 435	940.9467	1879.8789	1879.8743	2	0	K.VEFVHMLNATMCATTR.T	(Ions score 76)
420 - 435	627.6336	1879.8789	1879.8743	2	0	K.VEFVHMLNATMCATTR.T	(Ions score 84)
420 - 435	940.9467	1879.8789	1879.8743	2	0	K.VEFVHMLNATMCATTR.T	(Ions score 59)
420 - 435	940.9468	1879.8790	1879.8743	2	0	K.VEFVHMLNATMCATTR.T	(Ions score 57)
420 - 435	940.9468	1879.8790	1879.8743	2	0	K.VEFVHMLNATMCATTR.T	(Ions score 81)
420 - 435	940.9468	1879.8790	1879.8743	2	0	K.VEFVHMLNATMCATTR.T	(Ions score 67)
420 - 435	627.6336	1879.8790	1879.8743	2	0	K.VEFVHMLNATMCATTR.T	(Ions score 66)
420 - 435	627.6336	1879.8790	1879.8743	2	0	K.VEFVHMLNATMCATTR.T	(Ions score 79)
420 - 435	627.6336	1879.8790	1879.8743	2	0	K.VEFVHMLNATMCATTR.T	(Ions score 43)
420 - 435	627.6336	1879.8790	1879.8743	3	0	K.VEFVHMLNATMCATTR.T	(Ions score 63)
420 - 435	627.6336	1879.8790	1879.8743	3	0	K.VEFVHMLNATMCATTR.T	(Ions score 82)
420 - 435	940.9468	1879.8791	1879.8743	3	0	K.VEFVHMLNATMCATTR.T	(Ions score 51)
420 - 435	940.9468	1879.8791	1879.8743	3	0	K.VEFVHMLNATMCATTR.T	(Ions score 68)
420 - 435	940.9468	1879.8791	1879.8743	3	0	K.VEFVHMLNATMCATTR.T	(Ions score 91)
420 - 435	940.9468	1879.8791	1879.8743	3	0	K.VEFVHMLNATMCATTR.T	(Ions score 68)
420 - 435	940.9468	1879.8791	1879.8743	3	0	K.VEFVHMLNATMCATTR.T	(Ions score 67)
420 - 435	627.6336	1879.8791	1879.8743	3	0	K.VEFVHMLNATMCATTR.T	(Ions score 87)
420 - 435	627.6337	1879.8791	1879.8743	3	0	K.VEFVHMLNATMCATTR.T	(Ions score 69)
420 - 435	627.6337	1879.8792	1879.8743	3	0	K.VEFVHMLNATMCATTR.T	(Ions score 74)
420 - 435	627.6337	1879.8792	1879.8743	3	0	K.VEFVHMLNATMCATTR.T	(Ions score 70)
420 - 435	940.9469	1879.8792	1879.8743	3	0	K.VEFVHMLNATMCATTR.T	(Ions score 26)
420 - 435	627.6337	1879.8792	1879.8743	3	0	K.VEFVHMLNATMCATTR.T	(Ions score 89)
420 - 435	627.6337	1879.8792	1879.8743	3	0	K.VEFVHMLNATMCATTR.T	(Ions score 49)
420 - 435	940.9469	1879.8792	1879.8743	3	0	K.VEFVHMLNATMCATTR.T	(Ions score 58)
420 - 435	940.9469	1879.8792	1879.8743	3	0	K.VEFVHMLNATMCATTR.T	(Ions score 78)
420 - 435	627.6337	1879.8792	1879.8743	3	0	K.VEFVHMLNATMCATTR.T	(Ions score 84)
420 - 435	627.6337	1879.8792	1879.8743	3	0	K.VEFVHMLNATMCATTR.T	(Ions score 75)
420 - 435	627.6337	1879.8792	1879.8743	3	0	K.VEFVHMLNATMCATTR.T	(Ions score 79)
420 - 435	627.6337	1879.8793	1879.8743	3	0	K.VEFVHMLNATMCATTR.T	(Ions score 83)
420 - 435	627.6337	1879.8793	1879.8743	3	0	K.VEFVHMLNATMCATTR.T	(Ions score 70)
420 - 435	627.6337	1879.8793	1879.8743	3	0	K.VEFVHMLNATMCATTR.T	(Ions score 82)
420 - 435	940.9469	1879.8793	1879.8743	3	0	K.VEFVHMLNATMCATTR.T	(Ions score 53)
420 - 435	627.6337	1879.8794	1879.8743	3	0	K.VEFVHMLNATMCATTR.T	(Ions score 60)
420 - 435	627.6337	1879.8794	1879.8743	3	0	K.VEFVHMLNATMCATTR.T	(Ions score 72)

420 - 435	627.6337	1879.8794	1879.8743	3	0	K.VEFVHMLNATMCATTR.T	(Ions score 74)
420 - 435	627.6337	1879.8794	1879.8743	3	0	K.VEFVHMLNATMCATTR.T	(Ions score 57)
420 - 435	940.9470	1879.8794	1879.8743	3	0	K.VEFVHMLNATMCATTR.T	(Ions score 53)
420 - 435	627.6338	1879.8794	1879.8743	3	0	K.VEFVHMLNATMCATTR.T	(Ions score 81)
420 - 435	627.6338	1879.8794	1879.8743	3	0	K.VEFVHMLNATMCATTR.T	(Ions score 85)
420 - 435	627.6338	1879.8795	1879.8743	3	0	K.VEFVHMLNATMCATTR.T	(Ions score 90)
420 - 435	940.9470	1879.8795	1879.8743	3	0	K.VEFVHMLNATMCATTR.T	(Ions score 63)
420 - 435	627.6338	1879.8795	1879.8743	3	0	K.VEFVHMLNATMCATTR.T	(Ions score 70)
420 - 435	627.6338	1879.8795	1879.8743	3	0	K.VEFVHMLNATMCATTR.T	(Ions score 89)
420 - 435	627.6338	1879.8796	1879.8743	3	0	K.VEFVHMLNATMCATTR.T	(Ions score 86)
420 - 435	627.6338	1879.8796	1879.8743	3	0	K.VEFVHMLNATMCATTR.T	(Ions score 69)
420 - 435	940.9471	1879.8796	1879.8743	3	0	K.VEFVHMLNATMCATTR.T	(Ions score 77)
420 - 435	940.9471	1879.8796	1879.8743	3	0	K.VEFVHMLNATMCATTR.T	(Ions score 52)
420 - 435	627.6338	1879.8796	1879.8743	3	0	K.VEFVHMLNATMCATTR.T	(Ions score 79)
420 - 435	627.6338	1879.8797	1879.8743	3	0	K.VEFVHMLNATMCATTR.T	(Ions score 76)
420 - 435	940.9472	1879.8798	1879.8743	3	0	K.VEFVHMLNATMCATTR.T	(Ions score 59)
420 - 435	940.9472	1879.8798	1879.8743	3	0	K.VEFVHMLNATMCATTR.T	(Ions score 78)
420 - 435	627.6339	1879.8798	1879.8743	3	0	K.VEFVHMLNATMCATTR.T	(Ions score 72)
420 - 435	627.6339	1879.8798	1879.8743	3	0	K.VEFVHMLNATMCATTR.T	(Ions score 79)
420 - 435	940.9472	1879.8798	1879.8743	3	0	K.VEFVHMLNATMCATTR.T	(Ions score 46)
420 - 435	940.9472	1879.8799	1879.8743	3	0	K.VEFVHMLNATMCATTR.T	(Ions score 50)
420 - 435	627.6339	1879.8799	1879.8743	3	0	K.VEFVHMLNATMCATTR.T	(Ions score 79)
420 - 435	627.6339	1879.8799	1879.8743	3	0	K.VEFVHMLNATMCATTR.T	(Ions score 83)
420 - 435	627.6339	1879.8799	1879.8743	3	0	K.VEFVHMLNATMCATTR.T	(Ions score 82)
420 - 435	627.6339	1879.8799	1879.8743	3	0	K.VEFVHMLNATMCATTR.T	(Ions score 92)
420 - 435	627.6339	1879.8800	1879.8743	3	0	K.VEFVHMLNATMCATTR.T	(Ions score 79)
420 - 435	627.6339	1879.8800	1879.8743	3	0	K.VEFVHMLNATMCATTR.T	(Ions score 67)
420 - 435	627.6339	1879.8800	1879.8743	3	0	K.VEFVHMLNATMCATTR.T	(Ions score 75)
420 - 435	627.6340	1879.8801	1879.8743	3	0	K.VEFVHMLNATMCATTR.T	(Ions score 80)
420 - 435	627.6340	1879.8801	1879.8743	3	0	K.VEFVHMLNATMCATTR.T	(Ions score 78)
420 - 435	627.6340	1879.8801	1879.8743	3	0	K.VEFVHMLNATMCATTR.T	(Ions score 58)
420 - 435	627.6340	1879.8801	1879.8743	3	0	K.VEFVHMLNATMCATTR.T	(Ions score 72)
420 - 435	627.6340	1879.8802	1879.8743	3	0	K.VEFVHMLNATMCATTR.T	(Ions score 63)
420 - 435	627.6340	1879.8802	1879.8743	3	0	K.VEFVHMLNATMCATTR.T	(Ions score 30)
420 - 435	627.6340	1879.8803	1879.8743	3	0	K.VEFVHMLNATMCATTR.T	(Ions score 79)
420 - 435	627.6340	1879.8803	1879.8743	3	0	K.VEFVHMLNATMCATTR.T	(Ions score 72)
420 - 435	627.6340	1879.8803	1879.8743	3	0	K.VEFVHMLNATMCATTR.T	(Ions score 80)
420 - 435	627.6340	1879.8803	1879.8743	3	0	K.VEFVHMLNATMCATTR.T	(Ions score 63)
420 - 435	940.9474	1879.8803	1879.8743	3	0	K.VEFVHMLNATMCATTR.T	(Ions score 64)
420 - 435	627.6341	1879.8803	1879.8743	3	0	K.VEFVHMLNATMCATTR.T	(Ions score 82)
420 - 435	627.6341	1879.8804	1879.8743	3	0	K.VEFVHMLNATMCATTR.T	(Ions score 75)
420 - 435	627.6341	1879.8804	1879.8743	3	0	K.VEFVHMLNATMCATTR.T	(Ions score 71)
420 - 435	627.6341	1879.8804	1879.8743	3	0	K.VEFVHMLNATMCATTR.T	(Ions score 87)
420 - 435	627.6341	1879.8804	1879.8743	3	0	K.VEFVHMLNATMCATTR.T	(Ions score 79)
420 - 435	940.9475	1879.8804	1879.8743	3	0	K.VEFVHMLNATMCATTR.T	(Ions score 59)
420 - 435	627.6341	1879.8805	1879.8743	3	0	K.VEFVHMLNATMCATTR.T	(Ions score 76)
420 - 435	627.6341	1879.8805	1879.8743	3	0	K.VEFVHMLNATMCATTR.T	(Ions score 85)
420 - 435	627.6341	1879.8805	1879.8743	3	0	K.VEFVHMLNATMCATTR.T	(Ions score 75)
420 - 435	627.6341	1879.8805	1879.8743	3	0	K.VEFVHMLNATMCATTR.T	(Ions score 79)
420 - 435	627.6341	1879.8805	1879.8743	3	0	K.VEFVHMLNATMCATTR.T	(Ions score 67)
420 - 435	940.9476	1879.8806	1879.8743	3	0	K.VEFVHMLNATMCATTR.T	(Ions score 64)
420 - 435	627.6341	1879.8806	1879.8743	3	0	K.VEFVHMLNATMCATTR.T	(Ions score 61)
420 - 435	940.9476	1879.8806	1879.8743	3	0	K.VEFVHMLNATMCATTR.T	(Ions score 51)
420 - 435	627.6341	1879.8806	1879.8743	3	0	K.VEFVHMLNATMCATTR.T	(Ions score 56)
420 - 435	627.6341	1879.8806	1879.8743	3	0	K.VEFVHMLNATMCATTR.T	(Ions score 75)
420 - 435	627.6342	1879.8807	1879.8743	3	0	K.VEFVHMLNATMCATTR.T	(Ions score 79)
420 - 435	627.6342	1879.8807	1879.8743	3	0	K.VEFVHMLNATMCATTR.T	(Ions score 75)
420 - 435	627.6342	1879.8807	1879.8743	3	0	K.VEFVHMLNATMCATTR.T	(Ions score 82)

420 - 435	627.6342	1879.8808	1879.8743	3	0	K.VEFVHMLNATMCATTR.T	(Ions score 92)
420 - 435	627.6342	1879.8808	1879.8743	3	0	K.VEFVHMLNATMCATTR.T	(Ions score 86)
420 - 435	940.9477	1879.8808	1879.8743	3	0	K.VEFVHMLNATMCATTR.T	(Ions score 24)
420 - 435	627.6342	1879.8809	1879.8743	3	0	K.VEFVHMLNATMCATTR.T	(Ions score 67)
420 - 435	627.6342	1879.8809	1879.8743	4	0	K.VEFVHMLNATMCATTR.T	(Ions score 90)
420 - 435	940.9478	1879.8810	1879.8743	4	0	K.VEFVHMLNATMCATTR.T	(Ions score 51)
420 - 435	627.6343	1879.8811	1879.8743	4	0	K.VEFVHMLNATMCATTR.T	(Ions score 78)
420 - 435	627.6343	1879.8811	1879.8743	4	0	K.VEFVHMLNATMCATTR.T	(Ions score 69)
420 - 435	627.6343	1879.8811	1879.8743	4	0	K.VEFVHMLNATMCATTR.T	(Ions score 79)
420 - 435	940.9478	1879.8811	1879.8743	4	0	K.VEFVHMLNATMCATTR.T	(Ions score 67)
420 - 435	627.6343	1879.8811	1879.8743	4	0	K.VEFVHMLNATMCATTR.T	(Ions score 54)
420 - 435	627.6343	1879.8812	1879.8743	4	0	K.VEFVHMLNATMCATTR.T	(Ions score 79)
420 - 435	627.6343	1879.8812	1879.8743	4	0	K.VEFVHMLNATMCATTR.T	(Ions score 29)
420 - 435	940.9479	1879.8812	1879.8743	4	0	K.VEFVHMLNATMCATTR.T	(Ions score 73)
420 - 435	627.6343	1879.8812	1879.8743	4	0	K.VEFVHMLNATMCATTR.T	(Ions score 81)
420 - 435	627.6344	1879.8812	1879.8743	4	0	K.VEFVHMLNATMCATTR.T	(Ions score 61)
420 - 435	627.6344	1879.8812	1879.8743	4	0	K.VEFVHMLNATMCATTR.T	(Ions score 58)
420 - 435	627.6344	1879.8813	1879.8743	4	0	K.VEFVHMLNATMCATTR.T	(Ions score 90)
420 - 435	627.6344	1879.8813	1879.8743	4	0	K.VEFVHMLNATMCATTR.T	(Ions score 79)
420 - 435	627.6344	1879.8813	1879.8743	4	0	K.VEFVHMLNATMCATTR.T	(Ions score 79)
420 - 435	627.6344	1879.8814	1879.8743	4	0	K.VEFVHMLNATMCATTR.T	(Ions score 81)
420 - 435	627.6344	1879.8814	1879.8743	4	0	K.VEFVHMLNATMCATTR.T	(Ions score 70)
420 - 435	627.6344	1879.8814	1879.8743	4	0	K.VEFVHMLNATMCATTR.T	(Ions score 74)
420 - 435	627.6344	1879.8815	1879.8743	4	0	K.VEFVHMLNATMCATTR.T	(Ions score 71)
420 - 435	627.6344	1879.8815	1879.8743	4	0	K.VEFVHMLNATMCATTR.T	(Ions score 74)
420 - 435	627.6345	1879.8816	1879.8743	4	0	K.VEFVHMLNATMCATTR.T	(Ions score 87)
420 - 435	627.6345	1879.8816	1879.8743	4	0	K.VEFVHMLNATMCATTR.T	(Ions score 76)
420 - 435	627.6345	1879.8816	1879.8743	4	0	K.VEFVHMLNATMCATTR.T	(Ions score 76)
420 - 435	627.6345	1879.8816	1879.8743	4	0	K.VEFVHMLNATMCATTR.T	(Ions score 79)
420 - 435	627.6345	1879.8817	1879.8743	4	0	K.VEFVHMLNATMCATTR.T	(Ions score 80)
420 - 435	627.6345	1879.8817	1879.8743	4	0	K.VEFVHMLNATMCATTR.T	(Ions score 71)
420 - 435	627.6345	1879.8817	1879.8743	4	0	K.VEFVHMLNATMCATTR.T	(Ions score 82)
420 - 435	627.6345	1879.8818	1879.8743	4	0	K.VEFVHMLNATMCATTR.T	(Ions score 79)
420 - 435	627.6345	1879.8818	1879.8743	4	0	K.VEFVHMLNATMCATTR.T	(Ions score 77)
420 - 435	940.9482	1879.8818	1879.8743	4	0	K.VEFVHMLNATMCATTR.T	(Ions score 43)
420 - 435	627.6346	1879.8818	1879.8743	4	0	K.VEFVHMLNATMCATTR.T	(Ions score 63)
420 - 435	627.6346	1879.8819	1879.8743	4	0	K.VEFVHMLNATMCATTR.T	(Ions score 41)
420 - 435	627.6346	1879.8819	1879.8743	4	0	K.VEFVHMLNATMCATTR.T	(Ions score 68)
420 - 435	940.9482	1879.8819	1879.8743	4	0	K.VEFVHMLNATMCATTR.T	(Ions score 58)
420 - 435	627.6346	1879.8819	1879.8743	4	0	K.VEFVHMLNATMCATTR.T	(Ions score 69)
420 - 435	627.6346	1879.8821	1879.8743	4	0	K.VEFVHMLNATMCATTR.T	(Ions score 63)
420 - 435	627.6346	1879.8821	1879.8743	4	0	K.VEFVHMLNATMCATTR.T	(Ions score 57)
420 - 435	627.6346	1879.8821	1879.8743	4	0	K.VEFVHMLNATMCATTR.T	(Ions score 85)
420 - 435	940.9484	1879.8822	1879.8743	4	0	K.VEFVHMLNATMCATTR.T	(Ions score 44)
420 - 435	940.9484	1879.8823	1879.8743	4	0	K.VEFVHMLNATMCATTR.T	(Ions score 39)
420 - 435	627.6347	1879.8823	1879.8743	4	0	K.VEFVHMLNATMCATTR.T	(Ions score 46)
420 - 435	627.6347	1879.8823	1879.8743	4	0	K.VEFVHMLNATMCATTR.T	(Ions score 67)
420 - 435	627.6347	1879.8824	1879.8743	4	0	K.VEFVHMLNATMCATTR.T	(Ions score 79)
420 - 435	627.6347	1879.8824	1879.8743	4	0	K.VEFVHMLNATMCATTR.T	(Ions score 75)
420 - 435	627.6348	1879.8825	1879.8743	4	0	K.VEFVHMLNATMCATTR.T	(Ions score 78)
420 - 435	627.6348	1879.8825	1879.8743	4	0	K.VEFVHMLNATMCATTR.T	(Ions score 69)
420 - 435	627.6348	1879.8825	1879.8743	4	0	K.VEFVHMLNATMCATTR.T	(Ions score 89)
420 - 435	627.6348	1879.8825	1879.8743	4	0	K.VEFVHMLNATMCATTR.T	(Ions score 85)
420 - 435	627.6348	1879.8826	1879.8743	4	0	K.VEFVHMLNATMCATTR.T	(Ions score 79)
420 - 435	940.9486	1879.8826	1879.8743	4	0	K.VEFVHMLNATMCATTR.T	(Ions score 54)
420 - 435	627.6348	1879.8827	1879.8743	4	0	K.VEFVHMLNATMCATTR.T	(Ions score 79)
420 - 435	627.6349	1879.8828	1879.8743	5	0	K.VEFVHMLNATMCATTR.T	(Ions score 83)
420 - 435	627.6349	1879.8829	1879.8743	5	0	K.VEFVHMLNATMCATTR.T	(Ions score 33)

420 - 435	627.6349	1879.8830	1879.8743	5	0	K.VEFVHMLNATMCATTR.T		(Ions score 63)
420 - 435	627.6350	1879.8831	1879.8743	5	0	K.VEFVHMLNATMCATTR.T		(Ions score 79)
420 - 435	627.6350	1879.8832	1879.8743	5	0	K.VEFVHMLNATMCATTR.T		(Ions score 79)
420 - 435	627.6350	1879.8833	1879.8743	5	0	K.VEFVHMLNATMCATTR.T		(Ions score 89)
420 - 435	627.6351	1879.8834	1879.8743	5	0	K.VEFVHMLNATMCATTR.T		(Ions score 31)
420 - 435	627.6351	1879.8834	1879.8743	5	0	K.VEFVHMLNATMCATTR.T		(Ions score 62)
420 - 435	627.6351	1879.8834	1879.8743	5	0	K.VEFVHMLNATMCATTR.T		(Ions score 60)
420 - 435	940.9490	1879.8834	1879.8743	5	0	K.VEFVHMLNATMCATTR.T		(Ions score 31)
420 - 435	627.6351	1879.8834	1879.8743	5	0	K.VEFVHMLNATMCATTR.T		(Ions score 73)
420 - 435	627.6351	1879.8835	1879.8743	5	0	K.VEFVHMLNATMCATTR.T		(Ions score 61)
420 - 435	627.6351	1879.8835	1879.8743	5	0	K.VEFVHMLNATMCATTR.T		(Ions score 76)
420 - 435	627.6351	1879.8835	1879.8743	5	0	K.VEFVHMLNATMCATTR.T		(Ions score 74)
420 - 435	940.9490	1879.8835	1879.8743	5	0	K.VEFVHMLNATMCATTR.T		(Ions score 37)
420 - 435	627.6351	1879.8836	1879.8743	5	0	K.VEFVHMLNATMCATTR.T		(Ions score 78)
420 - 435	940.9491	1879.8837	1879.8743	5	0	K.VEFVHMLNATMCATTR.T		(Ions score 28)
420 - 435	632.9612	1895.8619	1895.8692	-4	0	K.VEFVHMLNATMCATTR.T	Oxidation (M)	(Ions score 49)
420 - 435	632.9617	1895.8633	1895.8692	-3	0	K.VEFVHMLNATMCATTR.T	Oxidation (M)	(Ions score 37)
420 - 435	948.9396	1895.8646	1895.8692	-2	0	K.VEFVHMLNATMCATTR.T	Oxidation (M)	(Ions score 29)
420 - 435	948.9396	1895.8647	1895.8692	-2	0	K.VEFVHMLNATMCATTR.T	Oxidation (M)	(Ions score 57)
420 - 435	632.9624	1895.8655	1895.8692	-2	0	K.VEFVHMLNATMCATTR.T	Oxidation (M)	(Ions score 59)
420 - 435	632.9624	1895.8655	1895.8692	-2	0	K.VEFVHMLNATMCATTR.T	Oxidation (M)	(Ions score 55)
420 - 435	632.9625	1895.8655	1895.8692	-2	0	K.VEFVHMLNATMCATTR.T	Oxidation (M)	(Ions score 43)
420 - 435	632.9625	1895.8656	1895.8692	-2	0	K.VEFVHMLNATMCATTR.T	Oxidation (M)	(Ions score 69)
420 - 435	948.9403	1895.8661	1895.8692	-2	0	K.VEFVHMLNATMCATTR.T	Oxidation (M)	(Ions score 30)
420 - 435	632.9628	1895.8665	1895.8692	-1	0	K.VEFVHMLNATMCATTR.T	Oxidation (M)	(Ions score 66)
420 - 435	632.9628	1895.8665	1895.8692	-1	0	K.VEFVHMLNATMCATTR.T	Oxidation (M)	(Ions score 41)
420 - 435	948.9406	1895.8666	1895.8692	-1	0	K.VEFVHMLNATMCATTR.T	Oxidation (M)	(Ions score 32)
420 - 435	632.9628	1895.8667	1895.8692	-1	0	K.VEFVHMLNATMCATTR.T	Oxidation (M)	(Ions score 58)
420 - 435	948.9406	1895.8667	1895.8692	-1	0	K.VEFVHMLNATMCATTR.T	Oxidation (M)	(Ions score 30)
420 - 435	948.9407	1895.8668	1895.8692	-1	0	K.VEFVHMLNATMCATTR.T	Oxidation (M)	(Ions score 63)
420 - 435	632.9629	1895.8669	1895.8692	-1	0	K.VEFVHMLNATMCATTR.T	Oxidation (M)	(Ions score 44)
420 - 435	632.9630	1895.8672	1895.8692	-1	0	K.VEFVHMLNATMCATTR.T	Oxidation (M)	(Ions score 60)
420 - 435	948.9409	1895.8672	1895.8692	-1	0	K.VEFVHMLNATMCATTR.T	Oxidation (M)	(Ions score 45)
420 - 435	948.9409	1895.8673	1895.8692	-1	0	K.VEFVHMLNATMCATTR.T	Oxidation (M)	(Ions score 36)
420 - 435	948.9410	1895.8675	1895.8692	-1	0	K.VEFVHMLNATMCATTR.T	Oxidation (M)	(Ions score 38)
420 - 435	948.9411	1895.8677	1895.8692	-1	0	K.VEFVHMLNATMCATTR.T	Oxidation (M)	(Ions score 37)
420 - 435	948.9411	1895.8677	1895.8692	-1	0	K.VEFVHMLNATMCATTR.T	Oxidation (M)	(Ions score 30)
420 - 435	632.9632	1895.8678	1895.8692	-1	0	K.VEFVHMLNATMCATTR.T	Oxidation (M)	(Ions score 60)
420 - 435	948.9412	1895.8679	1895.8692	-1	0	K.VEFVHMLNATMCATTR.T	Oxidation (M)	(Ions score 48)
420 - 435	632.9633	1895.8679	1895.8692	-1	0	K.VEFVHMLNATMCATTR.T	Oxidation (M)	(Ions score 48)
420 - 435	948.9413	1895.8680	1895.8692	-1	0	K.VEFVHMLNATMCATTR.T	Oxidation (M)	(Ions score 49)
420 - 435	632.9633	1895.8681	1895.8692	-1	0	K.VEFVHMLNATMCATTR.T	Oxidation (M)	(Ions score 55)
420 - 435	948.9413	1895.8681	1895.8692	-1	0	K.VEFVHMLNATMCATTR.T	Oxidation (M)	(Ions score 46)
420 - 435	632.9633	1895.8681	1895.8692	-1	0	K.VEFVHMLNATMCATTR.T	Oxidation (M)	(Ions score 54)
420 - 435	948.9413	1895.8681	1895.8692	-1	0	K.VEFVHMLNATMCATTR.T	Oxidation (M)	(Ions score 24)
420 - 435	632.9633	1895.8682	1895.8692	-1	0	K.VEFVHMLNATMCATTR.T	Oxidation (M)	(Ions score 69)
420 - 435	948.9414	1895.8682	1895.8692	-1	0	K.VEFVHMLNATMCATTR.T	Oxidation (M)	(Ions score 23)
420 - 435	632.9633	1895.8682	1895.8692	-1	0	K.VEFVHMLNATMCATTR.T	Oxidation (M)	(Ions score 70)
420 - 435	632.9634	1895.8682	1895.8692	-1	0	K.VEFVHMLNATMCATTR.T	Oxidation (M)	(Ions score 70)
420 - 435	632.9634	1895.8683	1895.8692	-0	0	K.VEFVHMLNATMCATTR.T	Oxidation (M)	(Ions score 43)
420 - 435	948.9415	1895.8684	1895.8692	-0	0	K.VEFVHMLNATMCATTR.T	Oxidation (M)	(Ions score 34)
420 - 435	948.9415	1895.8684	1895.8692	-0	0	K.VEFVHMLNATMCATTR.T	Oxidation (M)	(Ions score 33)
420 - 435	632.9634	1895.8685	1895.8692	-0	0	K.VEFVHMLNATMCATTR.T	Oxidation (M)	(Ions score 61)
420 - 435	948.9416	1895.8687	1895.8692	-0	0	K.VEFVHMLNATMCATTR.T	Oxidation (M)	(Ions score 25)
420 - 435	632.9635	1895.8688	1895.8692	-0	0	K.VEFVHMLNATMCATTR.T	Oxidation (M)	(Ions score 58)
420 - 435	632.9635	1895.8688	1895.8692	-0	0	K.VEFVHMLNATMCATTR.T	Oxidation (M)	(Ions score 65)
420 - 435	632.9636	1895.8689	1895.8692	-0	0	K.VEFVHMLNATMCATTR.T	Oxidation (M)	(Ions score 74)
420 - 435	632.9636	1895.8689	1895.8692	-0	0	K.VEFVHMLNATMCATTR.T	Oxidation (M)	(Ions score 51)

420 - 435	948.9417	1895.8689	1895.8692	-0	0	K.VEFVHMLNATMCATTR.T	Oxidation (M)	(Ions score 63)
420 - 435	948.9418	1895.8690	1895.8692	-0	0	K.VEFVHMLNATMCATTR.T	Oxidation (M)	(Ions score 42)
420 - 435	632.9636	1895.8690	1895.8692	-0	0	K.VEFVHMLNATMCATTR.T	Oxidation (M)	(Ions score 71)
420 - 435	632.9636	1895.8690	1895.8692	-0	0	K.VEFVHMLNATMCATTR.T	Oxidation (M)	(Ions score 53)
420 - 435	948.9418	1895.8690	1895.8692	-0	0	K.VEFVHMLNATMCATTR.T	Oxidation (M)	(Ions score 63)
420 - 435	948.9418	1895.8691	1895.8692	-0	0	K.VEFVHMLNATMCATTR.T	Oxidation (M)	(Ions score 31)
420 - 435	632.9636	1895.8691	1895.8692	-0	0	K.VEFVHMLNATMCATTR.T	Oxidation (M)	(Ions score 61)
420 - 435	948.9418	1895.8691	1895.8692	-0	0	K.VEFVHMLNATMCATTR.T	Oxidation (M)	(Ions score 35)
420 - 435	632.9637	1895.8692	1895.8692	-0	0	K.VEFVHMLNATMCATTR.T	Oxidation (M)	(Ions score 66)
420 - 435	948.9419	1895.8692	1895.8692	-0	0	K.VEFVHMLNATMCATTR.T	Oxidation (M)	(Ions score 35)
420 - 435	632.9637	1895.8693	1895.8692	0	0	K.VEFVHMLNATMCATTR.T	Oxidation (M)	(Ions score 58)
420 - 435	948.9419	1895.8693	1895.8692	0	0	K.VEFVHMLNATMCATTR.T	Oxidation (M)	(Ions score 36)
420 - 435	632.9637	1895.8693	1895.8692	0	0	K.VEFVHMLNATMCATTR.T	Oxidation (M)	(Ions score 68)
420 - 435	632.9637	1895.8694	1895.8692	0	0	K.VEFVHMLNATMCATTR.T	Oxidation (M)	(Ions score 71)
420 - 435	632.9637	1895.8694	1895.8692	0	0	K.VEFVHMLNATMCATTR.T	Oxidation (M)	(Ions score 55)
420 - 435	948.9420	1895.8694	1895.8692	0	0	K.VEFVHMLNATMCATTR.T	Oxidation (M)	(Ions score 35)
420 - 435	632.9638	1895.8695	1895.8692	0	0	K.VEFVHMLNATMCATTR.T	Oxidation (M)	(Ions score 40)
420 - 435	632.9638	1895.8696	1895.8692	0	0	K.VEFVHMLNATMCATTR.T	Oxidation (M)	(Ions score 66)
420 - 435	948.9421	1895.8696	1895.8692	0	0	K.VEFVHMLNATMCATTR.T	Oxidation (M)	(Ions score 27)
420 - 435	632.9638	1895.8696	1895.8692	0	0	K.VEFVHMLNATMCATTR.T	Oxidation (M)	(Ions score 60)
420 - 435	632.9638	1895.8697	1895.8692	0	0	K.VEFVHMLNATMCATTR.T	Oxidation (M)	(Ions score 72)
420 - 435	632.9638	1895.8697	1895.8692	0	0	K.VEFVHMLNATMCATTR.T	Oxidation (M)	(Ions score 43)
420 - 435	632.9639	1895.8698	1895.8692	0	0	K.VEFVHMLNATMCATTR.T	Oxidation (M)	(Ions score 61)
420 - 435	632.9639	1895.8698	1895.8692	0	0	K.VEFVHMLNATMCATTR.T	Oxidation (M)	(Ions score 52)
420 - 435	632.9639	1895.8699	1895.8692	0	0	K.VEFVHMLNATMCATTR.T	Oxidation (M)	(Ions score 67)
420 - 435	948.9422	1895.8699	1895.8692	0	0	K.VEFVHMLNATMCATTR.T	Oxidation (M)	(Ions score 50)
420 - 435	632.9639	1895.8699	1895.8692	0	0	K.VEFVHMLNATMCATTR.T	Oxidation (M)	(Ions score 57)
420 - 435	632.9639	1895.8699	1895.8692	0	0	K.VEFVHMLNATMCATTR.T	Oxidation (M)	(Ions score 74)
420 - 435	632.9639	1895.8699	1895.8692	0	0	K.VEFVHMLNATMCATTR.T	Oxidation (M)	(Ions score 58)
420 - 435	632.9639	1895.8700	1895.8692	0	0	K.VEFVHMLNATMCATTR.T	Oxidation (M)	(Ions score 73)
420 - 435	632.9639	1895.8700	1895.8692	0	0	K.VEFVHMLNATMCATTR.T	Oxidation (M)	(Ions score 61)
420 - 435	948.9423	1895.8700	1895.8692	0	0	K.VEFVHMLNATMCATTR.T	Oxidation (M)	(Ions score 48)
420 - 435	632.9640	1895.8700	1895.8692	0	0	K.VEFVHMLNATMCATTR.T	Oxidation (M)	(Ions score 63)
420 - 435	632.9640	1895.8701	1895.8692	0	0	K.VEFVHMLNATMCATTR.T	Oxidation (M)	(Ions score 37)
420 - 435	632.9640	1895.8701	1895.8692	0	0	K.VEFVHMLNATMCATTR.T	Oxidation (M)	(Ions score 64)
420 - 435	632.9640	1895.8701	1895.8692	0	0	K.VEFVHMLNATMCATTR.T	Oxidation (M)	(Ions score 68)
420 - 435	632.9640	1895.8701	1895.8692	0	0	K.VEFVHMLNATMCATTR.T	Oxidation (M)	(Ions score 53)
420 - 435	632.9640	1895.8701	1895.8692	0	0	K.VEFVHMLNATMCATTR.T	Oxidation (M)	(Ions score 72)
420 - 435	632.9640	1895.8702	1895.8692	1	0	K.VEFVHMLNATMCATTR.T	Oxidation (M)	(Ions score 56)
420 - 435	632.9640	1895.8702	1895.8692	1	0	K.VEFVHMLNATMCATTR.T	Oxidation (M)	(Ions score 57)
420 - 435	632.9640	1895.8702	1895.8692	1	0	K.VEFVHMLNATMCATTR.T	Oxidation (M)	(Ions score 57)
420 - 435	632.9640	1895.8702	1895.8692	1	0	K.VEFVHMLNATMCATTR.T	Oxidation (M)	(Ions score 66)
420 - 435	632.9640	1895.8702	1895.8692	1	0	K.VEFVHMLNATMCATTR.T	Oxidation (M)	(Ions score 63)
420 - 435	632.9640	1895.8703	1895.8692	1	0	K.VEFVHMLNATMCATTR.T	Oxidation (M)	(Ions score 61)
420 - 435	632.9640	1895.8703	1895.8692	1	0	K.VEFVHMLNATMCATTR.T	Oxidation (M)	(Ions score 65)
420 - 435	632.9641	1895.8703	1895.8692	1	0	K.VEFVHMLNATMCATTR.T	Oxidation (M)	(Ions score 68)
420 - 435	632.9641	1895.8703	1895.8692	1	0	K.VEFVHMLNATMCATTR.T	Oxidation (M)	(Ions score 49)
420 - 435	632.9641	1895.8704	1895.8692	1	0	K.VEFVHMLNATMCATTR.T	Oxidation (M)	(Ions score 54)
420 - 435	632.9641	1895.8704	1895.8692	1	0	K.VEFVHMLNATMCATTR.T	Oxidation (M)	(Ions score 59)
420 - 435	948.9425	1895.8705	1895.8692	1	0	K.VEFVHMLNATMCATTR.T	Oxidation (M)	(Ions score 24)
420 - 435	948.9425	1895.8705	1895.8692	1	0	K.VEFVHMLNATMCATTR.T	Oxidation (M)	(Ions score 45)
420 - 435	632.9641	1895.8706	1895.8692	1	0	K.VEFVHMLNATMCATTR.T	Oxidation (M)	(Ions score 62)
420 - 435	632.9641	1895.8706	1895.8692	1	0	K.VEFVHMLNATMCATTR.T	Oxidation (M)	(Ions score 71)
420 - 435	948.9426	1895.8706	1895.8692	1	0	K.VEFVHMLNATMCATTR.T	Oxidation (M)	(Ions score 43)
420 - 435	632.9641	1895.8706	1895.8692	1	0	K.VEFVHMLNATMCATTR.T	Oxidation (M)	(Ions score 46)
420 - 435	632.9642	1895.8706	1895.8692	1	0	K.VEFVHMLNATMCATTR.T	Oxidation (M)	(Ions score 63)
420 - 435	632.9642	1895.8706	1895.8692	1	0	K.VEFVHMLNATMCATTR.T	Oxidation (M)	(Ions score 73)
420 - 435	632.9642	1895.8706	1895.8692	1	0	K.VEFVHMLNATMCATTR.T	Oxidation (M)	(Ions score 68)

420 - 435	632.9642	1895.8707	1895.8692	1	0	K.VEFVHMLNATMCATTR.T	Oxidation (M)	(Ions score 58)
420 - 435	948.9426	1895.8707	1895.8692	1	0	K.VEFVHMLNATMCATTR.T	Oxidation (M)	(Ions score 53)
420 - 435	632.9642	1895.8707	1895.8692	1	0	K.VEFVHMLNATMCATTR.T	Oxidation (M)	(Ions score 58)
420 - 435	948.9426	1895.8707	1895.8692	1	0	K.VEFVHMLNATMCATTR.T	Oxidation (M)	(Ions score 55)
420 - 435	632.9642	1895.8708	1895.8692	1	0	K.VEFVHMLNATMCATTR.T	Oxidation (M)	(Ions score 56)
420 - 435	632.9642	1895.8708	1895.8692	1	0	K.VEFVHMLNATMCATTR.T	Oxidation (M)	(Ions score 47)
420 - 435	632.9642	1895.8708	1895.8692	1	0	K.VEFVHMLNATMCATTR.T	Oxidation (M)	(Ions score 54)
420 - 435	632.9642	1895.8708	1895.8692	1	0	K.VEFVHMLNATMCATTR.T	Oxidation (M)	(Ions score 60)
420 - 435	632.9642	1895.8708	1895.8692	1	0	K.VEFVHMLNATMCATTR.T	Oxidation (M)	(Ions score 57)
420 - 435	632.9642	1895.8708	1895.8692	1	0	K.VEFVHMLNATMCATTR.T	Oxidation (M)	(Ions score 71)
420 - 435	948.9427	1895.8709	1895.8692	1	0	K.VEFVHMLNATMCATTR.T	Oxidation (M)	(Ions score 62)
420 - 435	632.9642	1895.8709	1895.8692	1	0	K.VEFVHMLNATMCATTR.T	Oxidation (M)	(Ions score 54)
420 - 435	632.9642	1895.8709	1895.8692	1	0	K.VEFVHMLNATMCATTR.T	Oxidation (M)	(Ions score 75)
420 - 435	632.9642	1895.8709	1895.8692	1	0	K.VEFVHMLNATMCATTR.T	Oxidation (M)	(Ions score 41)
420 - 435	632.9642	1895.8709	1895.8692	1	0	K.VEFVHMLNATMCATTR.T	Oxidation (M)	(Ions score 57)
420 - 435	948.9427	1895.8709	1895.8692	1	0	K.VEFVHMLNATMCATTR.T	Oxidation (M)	(Ions score 44)
420 - 435	632.9643	1895.8709	1895.8692	1	0	K.VEFVHMLNATMCATTR.T	Oxidation (M)	(Ions score 59)
420 - 435	948.9427	1895.8709	1895.8692	1	0	K.VEFVHMLNATMCATTR.T	Oxidation (M)	(Ions score 44)
420 - 435	632.9643	1895.8710	1895.8692	1	0	K.VEFVHMLNATMCATTR.T	Oxidation (M)	(Ions score 43)
420 - 435	632.9643	1895.8711	1895.8692	1	0	K.VEFVHMLNATMCATTR.T	Oxidation (M)	(Ions score 53)
420 - 435	632.9643	1895.8711	1895.8692	1	0	K.VEFVHMLNATMCATTR.T	Oxidation (M)	(Ions score 65)
420 - 435	948.9428	1895.8711	1895.8692	1	0	K.VEFVHMLNATMCATTR.T	Oxidation (M)	(Ions score 44)
420 - 435	632.9643	1895.8711	1895.8692	1	0	K.VEFVHMLNATMCATTR.T	Oxidation (M)	(Ions score 31)
420 - 435	632.9643	1895.8711	1895.8692	1	0	K.VEFVHMLNATMCATTR.T	Oxidation (M)	(Ions score 56)
420 - 435	632.9643	1895.8711	1895.8692	1	0	K.VEFVHMLNATMCATTR.T	Oxidation (M)	(Ions score 49)
420 - 435	632.9643	1895.8712	1895.8692	1	0	K.VEFVHMLNATMCATTR.T	Oxidation (M)	(Ions score 59)
420 - 435	632.9643	1895.8712	1895.8692	1	0	K.VEFVHMLNATMCATTR.T	Oxidation (M)	(Ions score 41)
420 - 435	632.9644	1895.8713	1895.8692	1	0	K.VEFVHMLNATMCATTR.T	Oxidation (M)	(Ions score 70)
420 - 435	632.9644	1895.8713	1895.8692	1	0	K.VEFVHMLNATMCATTR.T	Oxidation (M)	(Ions score 61)
420 - 435	632.9644	1895.8713	1895.8692	1	0	K.VEFVHMLNATMCATTR.T	Oxidation (M)	(Ions score 21)
420 - 435	632.9644	1895.8713	1895.8692	1	0	K.VEFVHMLNATMCATTR.T	Oxidation (M)	(Ions score 61)
420 - 435	632.9644	1895.8713	1895.8692	1	0	K.VEFVHMLNATMCATTR.T	Oxidation (M)	(Ions score 71)
420 - 435	632.9644	1895.8714	1895.8692	1	0	K.VEFVHMLNATMCATTR.T	Oxidation (M)	(Ions score 38)
420 - 435	632.9644	1895.8714	1895.8692	1	0	K.VEFVHMLNATMCATTR.T	Oxidation (M)	(Ions score 63)
420 - 435	632.9644	1895.8714	1895.8692	1	0	K.VEFVHMLNATMCATTR.T	Oxidation (M)	(Ions score 73)
420 - 435	632.9644	1895.8714	1895.8692	1	0	K.VEFVHMLNATMCATTR.T	Oxidation (M)	(Ions score 63)
420 - 435	632.9644	1895.8714	1895.8692	1	0	K.VEFVHMLNATMCATTR.T	Oxidation (M)	(Ions score 60)
420 - 435	948.9430	1895.8714	1895.8692	1	0	K.VEFVHMLNATMCATTR.T	Oxidation (M)	(Ions score 45)
420 - 435	632.9644	1895.8715	1895.8692	1	0	K.VEFVHMLNATMCATTR.T	Oxidation (M)	(Ions score 46)
420 - 435	632.9644	1895.8715	1895.8692	1	0	K.VEFVHMLNATMCATTR.T	Oxidation (M)	(Ions score 57)
420 - 435	632.9645	1895.8715	1895.8692	1	0	K.VEFVHMLNATMCATTR.T	Oxidation (M)	(Ions score 71)
420 - 435	632.9645	1895.8716	1895.8692	1	0	K.VEFVHMLNATMCATTR.T	Oxidation (M)	(Ions score 58)
420 - 435	948.9431	1895.8716	1895.8692	1	0	K.VEFVHMLNATMCATTR.T	Oxidation (M)	(Ions score 40)
420 - 435	632.9645	1895.8716	1895.8692	1	0	K.VEFVHMLNATMCATTR.T	Oxidation (M)	(Ions score 70)
420 - 435	632.9645	1895.8716	1895.8692	1	0	K.VEFVHMLNATMCATTR.T	Oxidation (M)	(Ions score 72)
420 - 435	948.9431	1895.8717	1895.8692	1	0	K.VEFVHMLNATMCATTR.T	Oxidation (M)	(Ions score 45)
420 - 435	632.9645	1895.8717	1895.8692	1	0	K.VEFVHMLNATMCATTR.T	Oxidation (M)	(Ions score 73)
420 - 435	948.9431	1895.8717	1895.8692	1	0	K.VEFVHMLNATMCATTR.T	Oxidation (M)	(Ions score 42)
420 - 435	948.9431	1895.8717	1895.8692	1	0	K.VEFVHMLNATMCATTR.T	Oxidation (M)	(Ions score 43)
420 - 435	948.9431	1895.8717	1895.8692	1	0	K.VEFVHMLNATMCATTR.T	Oxidation (M)	(Ions score 42)
420 - 435	632.9645	1895.8717	1895.8692	1	0	K.VEFVHMLNATMCATTR.T	Oxidation (M)	(Ions score 39)
420 - 435	632.9645	1895.8718	1895.8692	1	0	K.VEFVHMLNATMCATTR.T	Oxidation (M)	(Ions score 59)
420 - 435	632.9645	1895.8718	1895.8692	1	0	K.VEFVHMLNATMCATTR.T	Oxidation (M)	(Ions score 43)
420 - 435	632.9645	1895.8718	1895.8692	1	0	K.VEFVHMLNATMCATTR.T	Oxidation (M)	(Ions score 48)
420 - 435	632.9645	1895.8718	1895.8692	1	0	K.VEFVHMLNATMCATTR.T	Oxidation (M)	(Ions score 42)
420 - 435	632.9645	1895.8718	1895.8692	1	0	K.VEFVHMLNATMCATTR.T	Oxidation (M)	(Ions score 61)
420 - 435	632.9646	1895.8718	1895.8692	1	0	K.VEFVHMLNATMCATTR.T	Oxidation (M)	(Ions score 69)
420 - 435	632.9646	1895.8718	1895.8692	1	0	K.VEFVHMLNATMCATTR.T	Oxidation (M)	(Ions score 72)

420 - 435	632.9646	1895.8718	1895.8692	1	0	K.VEFVHMLNATMCATTR.T	Oxidation (M)	(Ions score 58)
420 - 435	948.9432	1895.8718	1895.8692	1	0	K.VEFVHMLNATMCATTR.T	Oxidation (M)	(Ions score 28)
420 - 435	632.9646	1895.8719	1895.8692	1	0	K.VEFVHMLNATMCATTR.T	Oxidation (M)	(Ions score 58)
420 - 435	948.9432	1895.8719	1895.8692	1	0	K.VEFVHMLNATMCATTR.T	Oxidation (M)	(Ions score 47)
420 - 435	632.9646	1895.8719	1895.8692	1	0	K.VEFVHMLNATMCATTR.T	Oxidation (M)	(Ions score 61)
420 - 435	948.9432	1895.8719	1895.8692	1	0	K.VEFVHMLNATMCATTR.T	Oxidation (M)	(Ions score 42)
420 - 435	632.9646	1895.8719	1895.8692	1	0	K.VEFVHMLNATMCATTR.T	Oxidation (M)	(Ions score 65)
420 - 435	948.9433	1895.8720	1895.8692	1	0	K.VEFVHMLNATMCATTR.T	Oxidation (M)	(Ions score 44)
420 - 435	632.9646	1895.8720	1895.8692	1	0	K.VEFVHMLNATMCATTR.T	Oxidation (M)	(Ions score 59)
420 - 435	948.9433	1895.8720	1895.8692	1	0	K.VEFVHMLNATMCATTR.T	Oxidation (M)	(Ions score 42)
420 - 435	632.9646	1895.8720	1895.8692	1	0	K.VEFVHMLNATMCATTR.T	Oxidation (M)	(Ions score 59)
420 - 435	632.9646	1895.8720	1895.8692	1	0	K.VEFVHMLNATMCATTR.T	Oxidation (M)	(Ions score 51)
420 - 435	632.9646	1895.8720	1895.8692	1	0	K.VEFVHMLNATMCATTR.T	Oxidation (M)	(Ions score 58)
420 - 435	632.9646	1895.8720	1895.8692	1	0	K.VEFVHMLNATMCATTR.T	Oxidation (M)	(Ions score 59)
420 - 435	632.9646	1895.8721	1895.8692	1	0	K.VEFVHMLNATMCATTR.T	Oxidation (M)	(Ions score 65)
420 - 435	948.9433	1895.8721	1895.8692	2	0	K.VEFVHMLNATMCATTR.T	Oxidation (M)	(Ions score 37)
420 - 435	632.9646	1895.8721	1895.8692	2	0	K.VEFVHMLNATMCATTR.T	Oxidation (M)	(Ions score 60)
420 - 435	632.9646	1895.8721	1895.8692	2	0	K.VEFVHMLNATMCATTR.T	Oxidation (M)	(Ions score 54)
420 - 435	632.9647	1895.8721	1895.8692	2	0	K.VEFVHMLNATMCATTR.T	Oxidation (M)	(Ions score 61)
420 - 435	632.9647	1895.8721	1895.8692	2	0	K.VEFVHMLNATMCATTR.T	Oxidation (M)	(Ions score 47)
420 - 435	632.9647	1895.8722	1895.8692	2	0	K.VEFVHMLNATMCATTR.T	Oxidation (M)	(Ions score 64)
420 - 435	632.9647	1895.8722	1895.8692	2	0	K.VEFVHMLNATMCATTR.T	Oxidation (M)	(Ions score 60)
420 - 435	948.9434	1895.8722	1895.8692	2	0	K.VEFVHMLNATMCATTR.T	Oxidation (M)	(Ions score 54)
420 - 435	948.9434	1895.8722	1895.8692	2	0	K.VEFVHMLNATMCATTR.T	Oxidation (M)	(Ions score 44)
420 - 435	632.9647	1895.8722	1895.8692	2	0	K.VEFVHMLNATMCATTR.T	Oxidation (M)	(Ions score 45)
420 - 435	632.9647	1895.8723	1895.8692	2	0	K.VEFVHMLNATMCATTR.T	Oxidation (M)	(Ions score 63)
420 - 435	632.9647	1895.8723	1895.8692	2	0	K.VEFVHMLNATMCATTR.T	Oxidation (M)	(Ions score 41)
420 - 435	632.9647	1895.8723	1895.8692	2	0	K.VEFVHMLNATMCATTR.T	Oxidation (M)	(Ions score 59)
420 - 435	948.9434	1895.8723	1895.8692	2	0	K.VEFVHMLNATMCATTR.T	Oxidation (M)	(Ions score 36)
420 - 435	948.9434	1895.8723	1895.8692	2	0	K.VEFVHMLNATMCATTR.T	Oxidation (M)	(Ions score 29)
420 - 435	632.9647	1895.8723	1895.8692	2	0	K.VEFVHMLNATMCATTR.T	Oxidation (M)	(Ions score 71)
420 - 435	632.9647	1895.8723	1895.8692	2	0	K.VEFVHMLNATMCATTR.T	Oxidation (M)	(Ions score 58)
420 - 435	632.9647	1895.8723	1895.8692	2	0	K.VEFVHMLNATMCATTR.T	Oxidation (M)	(Ions score 64)
420 - 435	632.9647	1895.8723	1895.8692	2	0	K.VEFVHMLNATMCATTR.T	Oxidation (M)	(Ions score 73)
420 - 435	948.9434	1895.8723	1895.8692	2	0	K.VEFVHMLNATMCATTR.T	Oxidation (M)	(Ions score 43)
420 - 435	632.9647	1895.8724	1895.8692	2	0	K.VEFVHMLNATMCATTR.T	Oxidation (M)	(Ions score 64)
420 - 435	948.9435	1895.8724	1895.8692	2	0	K.VEFVHMLNATMCATTR.T	Oxidation (M)	(Ions score 54)
420 - 435	632.9647	1895.8724	1895.8692	2	0	K.VEFVHMLNATMCATTR.T	Oxidation (M)	(Ions score 74)
420 - 435	632.9647	1895.8724	1895.8692	2	0	K.VEFVHMLNATMCATTR.T	Oxidation (M)	(Ions score 58)
420 - 435	632.9647	1895.8724	1895.8692	2	0	K.VEFVHMLNATMCATTR.T	Oxidation (M)	(Ions score 57)
420 - 435	632.9647	1895.8724	1895.8692	2	0	K.VEFVHMLNATMCATTR.T	Oxidation (M)	(Ions score 63)
420 - 435	632.9647	1895.8724	1895.8692	2	0	K.VEFVHMLNATMCATTR.T	Oxidation (M)	(Ions score 76)
420 - 435	632.9648	1895.8724	1895.8692	2	0	K.VEFVHMLNATMCATTR.T	Oxidation (M)	(Ions score 71)
420 - 435	632.9648	1895.8724	1895.8692	2	0	K.VEFVHMLNATMCATTR.T	Oxidation (M)	(Ions score 67)
420 - 435	632.9648	1895.8724	1895.8692	2	0	K.VEFVHMLNATMCATTR.T	Oxidation (M)	(Ions score 62)
420 - 435	948.9435	1895.8725	1895.8692	2	0	K.VEFVHMLNATMCATTR.T	Oxidation (M)	(Ions score 47)
420 - 435	632.9648	1895.8725	1895.8692	2	0	K.VEFVHMLNATMCATTR.T	Oxidation (M)	(Ions score 45)
420 - 435	632.9648	1895.8725	1895.8692	2	0	K.VEFVHMLNATMCATTR.T	Oxidation (M)	(Ions score 57)
420 - 435	948.9435	1895.8725	1895.8692	2	0	K.VEFVHMLNATMCATTR.T	Oxidation (M)	(Ions score 36)
420 - 435	948.9436	1895.8726	1895.8692	2	0	K.VEFVHMLNATMCATTR.T	Oxidation (M)	(Ions score 44)
420 - 435	632.9648	1895.8726	1895.8692	2	0	K.VEFVHMLNATMCATTR.T	Oxidation (M)	(Ions score 52)
420 - 435	948.9436	1895.8726	1895.8692	2	0	K.VEFVHMLNATMCATTR.T	Oxidation (M)	(Ions score 49)
420 - 435	948.9436	1895.8726	1895.8692	2	0	K.VEFVHMLNATMCATTR.T	Oxidation (M)	(Ions score 44)
420 - 435	948.9436	1895.8726	1895.8692	2	0	K.VEFVHMLNATMCATTR.T	Oxidation (M)	(Ions score 38)
420 - 435	948.9436	1895.8727	1895.8692	2	0	K.VEFVHMLNATMCATTR.T	Oxidation (M)	(Ions score 25)
420 - 435	948.9436	1895.8727	1895.8692	2	0	K.VEFVHMLNATMCATTR.T	Oxidation (M)	(Ions score 32)
420 - 435	632.9648	1895.8727	1895.8692	2	0	K.VEFVHMLNATMCATTR.T	Oxidation (M)	(Ions score 64)
420 - 435	632.9648	1895.8727	1895.8692	2	0	K.VEFVHMLNATMCATTR.T	Oxidation (M)	(Ions score 73)

420 - 435	632.9648	1895.8727	1895.8692	2	0	K.VEFVHMLNATMCATTR.T	Oxidation (M)	(Ions score 68)
420 - 435	948.9436	1895.8727	1895.8692	2	0	K.VEFVHMLNATMCATTR.T	Oxidation (M)	(Ions score 28)
420 - 435	948.9436	1895.8727	1895.8692	2	0	K.VEFVHMLNATMCATTR.T	Oxidation (M)	(Ions score 44)
420 - 435	632.9649	1895.8727	1895.8692	2	0	K.VEFVHMLNATMCATTR.T	Oxidation (M)	(Ions score 62)
420 - 435	632.9649	1895.8728	1895.8692	2	0	K.VEFVHMLNATMCATTR.T	Oxidation (M)	(Ions score 77)
420 - 435	948.9437	1895.8728	1895.8692	2	0	K.VEFVHMLNATMCATTR.T	Oxidation (M)	(Ions score 54)
420 - 435	632.9649	1895.8728	1895.8692	2	0	K.VEFVHMLNATMCATTR.T	Oxidation (M)	(Ions score 75)
420 - 435	632.9649	1895.8728	1895.8692	2	0	K.VEFVHMLNATMCATTR.T	Oxidation (M)	(Ions score 71)
420 - 435	632.9649	1895.8728	1895.8692	2	0	K.VEFVHMLNATMCATTR.T	Oxidation (M)	(Ions score 57)
420 - 435	948.9437	1895.8728	1895.8692	2	0	K.VEFVHMLNATMCATTR.T	Oxidation (M)	(Ions score 43)
420 - 435	632.9649	1895.8728	1895.8692	2	0	K.VEFVHMLNATMCATTR.T	Oxidation (M)	(Ions score 58)
420 - 435	632.9649	1895.8728	1895.8692	2	0	K.VEFVHMLNATMCATTR.T	Oxidation (M)	(Ions score 71)
420 - 435	948.9437	1895.8729	1895.8692	2	0	K.VEFVHMLNATMCATTR.T	Oxidation (M)	(Ions score 66)
420 - 435	632.9649	1895.8729	1895.8692	2	0	K.VEFVHMLNATMCATTR.T	Oxidation (M)	(Ions score 66)
420 - 435	948.9437	1895.8729	1895.8692	2	0	K.VEFVHMLNATMCATTR.T	Oxidation (M)	(Ions score 42)
420 - 435	632.9649	1895.8729	1895.8692	2	0	K.VEFVHMLNATMCATTR.T	Oxidation (M)	(Ions score 37)
420 - 435	948.9437	1895.8729	1895.8692	2	0	K.VEFVHMLNATMCATTR.T	Oxidation (M)	(Ions score 40)
420 - 435	632.9649	1895.8729	1895.8692	2	0	K.VEFVHMLNATMCATTR.T	Oxidation (M)	(Ions score 73)
420 - 435	632.9649	1895.8729	1895.8692	2	0	K.VEFVHMLNATMCATTR.T	Oxidation (M)	(Ions score 58)
420 - 435	632.9649	1895.8729	1895.8692	2	0	K.VEFVHMLNATMCATTR.T	Oxidation (M)	(Ions score 64)
420 - 435	632.9649	1895.8729	1895.8692	2	0	K.VEFVHMLNATMCATTR.T	Oxidation (M)	(Ions score 69)
420 - 435	948.9438	1895.8729	1895.8692	2	0	K.VEFVHMLNATMCATTR.T	Oxidation (M)	(Ions score 37)
420 - 435	948.9438	1895.8730	1895.8692	2	0	K.VEFVHMLNATMCATTR.T	Oxidation (M)	(Ions score 29)
420 - 435	632.9649	1895.8730	1895.8692	2	0	K.VEFVHMLNATMCATTR.T	Oxidation (M)	(Ions score 60)
420 - 435	948.9438	1895.8730	1895.8692	2	0	K.VEFVHMLNATMCATTR.T	Oxidation (M)	(Ions score 27)
420 - 435	948.9438	1895.8730	1895.8692	2	0	K.VEFVHMLNATMCATTR.T	Oxidation (M)	(Ions score 34)
420 - 435	948.9438	1895.8731	1895.8692	2	0	K.VEFVHMLNATMCATTR.T	Oxidation (M)	(Ions score 25)
420 - 435	632.9650	1895.8731	1895.8692	2	0	K.VEFVHMLNATMCATTR.T	Oxidation (M)	(Ions score 73)
420 - 435	632.9650	1895.8731	1895.8692	2	0	K.VEFVHMLNATMCATTR.T	Oxidation (M)	(Ions score 58)
420 - 435	948.9438	1895.8731	1895.8692	2	0	K.VEFVHMLNATMCATTR.T	Oxidation (M)	(Ions score 49)
420 - 435	632.9650	1895.8731	1895.8692	2	0	K.VEFVHMLNATMCATTR.T	Oxidation (M)	(Ions score 63)
420 - 435	948.9438	1895.8731	1895.8692	2	0	K.VEFVHMLNATMCATTR.T	Oxidation (M)	(Ions score 39)
420 - 435	948.9438	1895.8731	1895.8692	2	0	K.VEFVHMLNATMCATTR.T	Oxidation (M)	(Ions score 52)
420 - 435	632.9650	1895.8731	1895.8692	2	0	K.VEFVHMLNATMCATTR.T	Oxidation (M)	(Ions score 63)
420 - 435	632.9650	1895.8732	1895.8692	2	0	K.VEFVHMLNATMCATTR.T	Oxidation (M)	(Ions score 59)
420 - 435	632.9650	1895.8732	1895.8692	2	0	K.VEFVHMLNATMCATTR.T	Oxidation (M)	(Ions score 45)
420 - 435	632.9650	1895.8732	1895.8692	2	0	K.VEFVHMLNATMCATTR.T	Oxidation (M)	(Ions score 73)
420 - 435	948.9439	1895.8732	1895.8692	2	0	K.VEFVHMLNATMCATTR.T	Oxidation (M)	(Ions score 40)
420 - 435	632.9650	1895.8733	1895.8692	2	0	K.VEFVHMLNATMCATTR.T	Oxidation (M)	(Ions score 52)
420 - 435	948.9439	1895.8733	1895.8692	2	0	K.VEFVHMLNATMCATTR.T	Oxidation (M)	(Ions score 52)
420 - 435	632.9650	1895.8733	1895.8692	2	0	K.VEFVHMLNATMCATTR.T	Oxidation (M)	(Ions score 61)
420 - 435	632.9650	1895.8733	1895.8692	2	0	K.VEFVHMLNATMCATTR.T	Oxidation (M)	(Ions score 73)
420 - 435	948.9439	1895.8733	1895.8692	2	0	K.VEFVHMLNATMCATTR.T	Oxidation (M)	(Ions score 22)
420 - 435	632.9651	1895.8734	1895.8692	2	0	K.VEFVHMLNATMCATTR.T	Oxidation (M)	(Ions score 60)
420 - 435	632.9651	1895.8734	1895.8692	2	0	K.VEFVHMLNATMCATTR.T	Oxidation (M)	(Ions score 47)
420 - 435	632.9651	1895.8734	1895.8692	2	0	K.VEFVHMLNATMCATTR.T	Oxidation (M)	(Ions score 61)
420 - 435	948.9440	1895.8735	1895.8692	2	0	K.VEFVHMLNATMCATTR.T	Oxidation (M)	(Ions score 38)
420 - 435	632.9651	1895.8735	1895.8692	2	0	K.VEFVHMLNATMCATTR.T	Oxidation (M)	(Ions score 57)
420 - 435	632.9651	1895.8735	1895.8692	2	0	K.VEFVHMLNATMCATTR.T	Oxidation (M)	(Ions score 70)
420 - 435	948.9440	1895.8735	1895.8692	2	0	K.VEFVHMLNATMCATTR.T	Oxidation (M)	(Ions score 41)
420 - 435	632.9651	1895.8735	1895.8692	2	0	K.VEFVHMLNATMCATTR.T	Oxidation (M)	(Ions score 58)
420 - 435	632.9651	1895.8735	1895.8692	2	0	K.VEFVHMLNATMCATTR.T	Oxidation (M)	(Ions score 48)
420 - 435	948.9440	1895.8735	1895.8692	2	0	K.VEFVHMLNATMCATTR.T	Oxidation (M)	(Ions score 33)
420 - 435	948.9441	1895.8736	1895.8692	2	0	K.VEFVHMLNATMCATTR.T	Oxidation (M)	(Ions score 54)
420 - 435	948.9441	1895.8736	1895.8692	2	0	K.VEFVHMLNATMCATTR.T	Oxidation (M)	(Ions score 39)
420 - 435	632.9652	1895.8736	1895.8692	2	0	K.VEFVHMLNATMCATTR.T	Oxidation (M)	(Ions score 43)
420 - 435	948.9441	1895.8737	1895.8692	2	0	K.VEFVHMLNATMCATTR.T	Oxidation (M)	(Ions score 32)
420 - 435	632.9652	1895.8737	1895.8692	2	0	K.VEFVHMLNATMCATTR.T	Oxidation (M)	(Ions score 68)

420 - 435	632.9652	1895.8737	1895.8692	2	0	K.VEFVHMLNATMCATTR.T	Oxidation (M)	(Ions score 71)
420 - 435	632.9652	1895.8737	1895.8692	2	0	K.VEFVHMLNATMCATTR.T	Oxidation (M)	(Ions score 74)
420 - 435	632.9652	1895.8737	1895.8692	2	0	K.VEFVHMLNATMCATTR.T	Oxidation (M)	(Ions score 62)
420 - 435	632.9652	1895.8737	1895.8692	2	0	K.VEFVHMLNATMCATTR.T	Oxidation (M)	(Ions score 49)
420 - 435	632.9652	1895.8737	1895.8692	2	0	K.VEFVHMLNATMCATTR.T	Oxidation (M)	(Ions score 52)
420 - 435	632.9652	1895.8737	1895.8692	2	0	K.VEFVHMLNATMCATTR.T	Oxidation (M)	(Ions score 62)
420 - 435	632.9652	1895.8737	1895.8692	2	0	K.VEFVHMLNATMCATTR.T	Oxidation (M)	(Ions score 66)
420 - 435	632.9652	1895.8738	1895.8692	2	0	K.VEFVHMLNATMCATTR.T	Oxidation (M)	(Ions score 75)
420 - 435	632.9652	1895.8738	1895.8692	2	0	K.VEFVHMLNATMCATTR.T	Oxidation (M)	(Ions score 61)
420 - 435	632.9652	1895.8738	1895.8692	2	0	K.VEFVHMLNATMCATTR.T	Oxidation (M)	(Ions score 59)
420 - 435	632.9652	1895.8738	1895.8692	2	0	K.VEFVHMLNATMCATTR.T	Oxidation (M)	(Ions score 57)
420 - 435	632.9652	1895.8738	1895.8692	2	0	K.VEFVHMLNATMCATTR.T	Oxidation (M)	(Ions score 55)
420 - 435	632.9652	1895.8738	1895.8692	2	0	K.VEFVHMLNATMCATTR.T	Oxidation (M)	(Ions score 47)
420 - 435	632.9652	1895.8738	1895.8692	2	0	K.VEFVHMLNATMCATTR.T	Oxidation (M)	(Ions score 63)
420 - 435	948.9442	1895.8738	1895.8692	2	0	K.VEFVHMLNATMCATTR.T	Oxidation (M)	(Ions score 50)
420 - 435	632.9652	1895.8738	1895.8692	2	0	K.VEFVHMLNATMCATTR.T	Oxidation (M)	(Ions score 61)
420 - 435	632.9652	1895.8739	1895.8692	2	0	K.VEFVHMLNATMCATTR.T	Oxidation (M)	(Ions score 55)
420 - 435	632.9652	1895.8739	1895.8692	2	0	K.VEFVHMLNATMCATTR.T	Oxidation (M)	(Ions score 71)
420 - 435	632.9652	1895.8739	1895.8692	2	0	K.VEFVHMLNATMCATTR.T	Oxidation (M)	(Ions score 60)
420 - 435	632.9653	1895.8739	1895.8692	2	0	K.VEFVHMLNATMCATTR.T	Oxidation (M)	(Ions score 60)
420 - 435	632.9653	1895.8740	1895.8692	2	0	K.VEFVHMLNATMCATTR.T	Oxidation (M)	(Ions score 69)
420 - 435	948.9443	1895.8740	1895.8692	3	0	K.VEFVHMLNATMCATTR.T	Oxidation (M)	(Ions score 54)
420 - 435	632.9653	1895.8741	1895.8692	3	0	K.VEFVHMLNATMCATTR.T	Oxidation (M)	(Ions score 64)
420 - 435	948.9443	1895.8741	1895.8692	3	0	K.VEFVHMLNATMCATTR.T	Oxidation (M)	(Ions score 44)
420 - 435	632.9653	1895.8741	1895.8692	3	0	K.VEFVHMLNATMCATTR.T	Oxidation (M)	(Ions score 72)
420 - 435	948.9443	1895.8741	1895.8692	3	0	K.VEFVHMLNATMCATTR.T	Oxidation (M)	(Ions score 30)
420 - 435	632.9653	1895.8741	1895.8692	3	0	K.VEFVHMLNATMCATTR.T	Oxidation (M)	(Ions score 57)
420 - 435	632.9653	1895.8742	1895.8692	3	0	K.VEFVHMLNATMCATTR.T	Oxidation (M)	(Ions score 74)
420 - 435	632.9653	1895.8742	1895.8692	3	0	K.VEFVHMLNATMCATTR.T	Oxidation (M)	(Ions score 75)
420 - 435	632.9653	1895.8742	1895.8692	3	0	K.VEFVHMLNATMCATTR.T	Oxidation (M)	(Ions score 61)
420 - 435	632.9654	1895.8742	1895.8692	3	0	K.VEFVHMLNATMCATTR.T	Oxidation (M)	(Ions score 46)
420 - 435	948.9444	1895.8743	1895.8692	3	0	K.VEFVHMLNATMCATTR.T	Oxidation (M)	(Ions score 41)
420 - 435	632.9654	1895.8743	1895.8692	3	0	K.VEFVHMLNATMCATTR.T	Oxidation (M)	(Ions score 69)
420 - 435	632.9654	1895.8743	1895.8692	3	0	K.VEFVHMLNATMCATTR.T	Oxidation (M)	(Ions score 59)
420 - 435	948.9444	1895.8743	1895.8692	3	0	K.VEFVHMLNATMCATTR.T	Oxidation (M)	(Ions score 57)
420 - 435	632.9654	1895.8743	1895.8692	3	0	K.VEFVHMLNATMCATTR.T	Oxidation (M)	(Ions score 49)
420 - 435	632.9654	1895.8743	1895.8692	3	0	K.VEFVHMLNATMCATTR.T	Oxidation (M)	(Ions score 59)
420 - 435	632.9654	1895.8743	1895.8692	3	0	K.VEFVHMLNATMCATTR.T	Oxidation (M)	(Ions score 42)
420 - 435	948.9444	1895.8743	1895.8692	3	0	K.VEFVHMLNATMCATTR.T	Oxidation (M)	(Ions score 35)
420 - 435	632.9654	1895.8743	1895.8692	3	0	K.VEFVHMLNATMCATTR.T	Oxidation (M)	(Ions score 62)
420 - 435	632.9654	1895.8744	1895.8692	3	0	K.VEFVHMLNATMCATTR.T	Oxidation (M)	(Ions score 57)
420 - 435	632.9654	1895.8744	1895.8692	3	0	K.VEFVHMLNATMCATTR.T	Oxidation (M)	(Ions score 68)
420 - 435	948.9445	1895.8744	1895.8692	3	0	K.VEFVHMLNATMCATTR.T	Oxidation (M)	(Ions score 23)
420 - 435	632.9654	1895.8744	1895.8692	3	0	K.VEFVHMLNATMCATTR.T	Oxidation (M)	(Ions score 59)
420 - 435	948.9445	1895.8744	1895.8692	3	0	K.VEFVHMLNATMCATTR.T	Oxidation (M)	(Ions score 44)
420 - 435	632.9654	1895.8744	1895.8692	3	0	K.VEFVHMLNATMCATTR.T	Oxidation (M)	(Ions score 58)
420 - 435	948.9445	1895.8745	1895.8692	3	0	K.VEFVHMLNATMCATTR.T	Oxidation (M)	(Ions score 53)
420 - 435	632.9654	1895.8745	1895.8692	3	0	K.VEFVHMLNATMCATTR.T	Oxidation (M)	(Ions score 66)
420 - 435	632.9654	1895.8745	1895.8692	3	0	K.VEFVHMLNATMCATTR.T	Oxidation (M)	(Ions score 62)
420 - 435	948.9445	1895.8745	1895.8692	3	0	K.VEFVHMLNATMCATTR.T	Oxidation (M)	(Ions score 41)
420 - 435	632.9654	1895.8745	1895.8692	3	0	K.VEFVHMLNATMCATTR.T	Oxidation (M)	(Ions score 60)
420 - 435	632.9654	1895.8745	1895.8692	3	0	K.VEFVHMLNATMCATTR.T	Oxidation (M)	(Ions score 73)
420 - 435	632.9655	1895.8745	1895.8692	3	0	K.VEFVHMLNATMCATTR.T	Oxidation (M)	(Ions score 64)
420 - 435	632.9655	1895.8745	1895.8692	3	0	K.VEFVHMLNATMCATTR.T	Oxidation (M)	(Ions score 74)
420 - 435	632.9655	1895.8746	1895.8692	3	0	K.VEFVHMLNATMCATTR.T	Oxidation (M)	(Ions score 63)
420 - 435	948.9446	1895.8746	1895.8692	3	0	K.VEFVHMLNATMCATTR.T	Oxidation (M)	(Ions score 35)
420 - 435	632.9655	1895.8746	1895.8692	3	0	K.VEFVHMLNATMCATTR.T	Oxidation (M)	(Ions score 63)
420 - 435	632.9655	1895.8746	1895.8692	3	0	K.VEFVHMLNATMCATTR.T	Oxidation (M)	(Ions score 58)

420 - 435	632.9655	1895.8746	1895.8692	3	0	K.VEFVHMLNATMCATTR.T	Oxidation (M)	(Ions score 64)
420 - 435	632.9655	1895.8746	1895.8692	3	0	K.VEFVHMLNATMCATTR.T	Oxidation (M)	(Ions score 73)
420 - 435	632.9655	1895.8746	1895.8692	3	0	K.VEFVHMLNATMCATTR.T	Oxidation (M)	(Ions score 56)
420 - 435	632.9655	1895.8747	1895.8692	3	0	K.VEFVHMLNATMCATTR.T	Oxidation (M)	(Ions score 59)
420 - 435	632.9655	1895.8747	1895.8692	3	0	K.VEFVHMLNATMCATTR.T	Oxidation (M)	(Ions score 42)
420 - 435	632.9655	1895.8747	1895.8692	3	0	K.VEFVHMLNATMCATTR.T	Oxidation (M)	(Ions score 62)
420 - 435	632.9655	1895.8747	1895.8692	3	0	K.VEFVHMLNATMCATTR.T	Oxidation (M)	(Ions score 57)
420 - 435	948.9446	1895.8747	1895.8692	3	0	K.VEFVHMLNATMCATTR.T	Oxidation (M)	(Ions score 34)
420 - 435	632.9655	1895.8747	1895.8692	3	0	K.VEFVHMLNATMCATTR.T	Oxidation (M)	(Ions score 64)
420 - 435	948.9446	1895.8747	1895.8692	3	0	K.VEFVHMLNATMCATTR.T	Oxidation (M)	(Ions score 64)
420 - 435	632.9655	1895.8748	1895.8692	3	0	K.VEFVHMLNATMCATTR.T	Oxidation (M)	(Ions score 68)
420 - 435	948.9447	1895.8748	1895.8692	3	0	K.VEFVHMLNATMCATTR.T	Oxidation (M)	(Ions score 47)
420 - 435	632.9656	1895.8748	1895.8692	3	0	K.VEFVHMLNATMCATTR.T	Oxidation (M)	(Ions score 75)
420 - 435	632.9656	1895.8749	1895.8692	3	0	K.VEFVHMLNATMCATTR.T	Oxidation (M)	(Ions score 52)
420 - 435	632.9656	1895.8749	1895.8692	3	0	K.VEFVHMLNATMCATTR.T	Oxidation (M)	(Ions score 63)
420 - 435	948.9447	1895.8749	1895.8692	3	0	K.VEFVHMLNATMCATTR.T	Oxidation (M)	(Ions score 38)
420 - 435	632.9656	1895.8749	1895.8692	3	0	K.VEFVHMLNATMCATTR.T	Oxidation (M)	(Ions score 59)
420 - 435	632.9656	1895.8749	1895.8692	3	0	K.VEFVHMLNATMCATTR.T	Oxidation (M)	(Ions score 70)
420 - 435	632.9656	1895.8749	1895.8692	3	0	K.VEFVHMLNATMCATTR.T	Oxidation (M)	(Ions score 58)
420 - 435	632.9656	1895.8749	1895.8692	3	0	K.VEFVHMLNATMCATTR.T	Oxidation (M)	(Ions score 48)
420 - 435	632.9656	1895.8749	1895.8692	3	0	K.VEFVHMLNATMCATTR.T	Oxidation (M)	(Ions score 71)
420 - 435	948.9447	1895.8749	1895.8692	3	0	K.VEFVHMLNATMCATTR.T	Oxidation (M)	(Ions score 38)
420 - 435	948.9447	1895.8749	1895.8692	3	0	K.VEFVHMLNATMCATTR.T	Oxidation (M)	(Ions score 25)
420 - 435	632.9656	1895.8749	1895.8692	3	0	K.VEFVHMLNATMCATTR.T	Oxidation (M)	(Ions score 58)
420 - 435	948.9447	1895.8749	1895.8692	3	0	K.VEFVHMLNATMCATTR.T	Oxidation (M)	(Ions score 43)
420 - 435	948.9448	1895.8750	1895.8692	3	0	K.VEFVHMLNATMCATTR.T	Oxidation (M)	(Ions score 50)
420 - 435	948.9448	1895.8750	1895.8692	3	0	K.VEFVHMLNATMCATTR.T	Oxidation (M)	(Ions score 44)
420 - 435	632.9656	1895.8750	1895.8692	3	0	K.VEFVHMLNATMCATTR.T	Oxidation (M)	(Ions score 68)
420 - 435	632.9656	1895.8750	1895.8692	3	0	K.VEFVHMLNATMCATTR.T	Oxidation (M)	(Ions score 42)
420 - 435	632.9656	1895.8750	1895.8692	3	0	K.VEFVHMLNATMCATTR.T	Oxidation (M)	(Ions score 53)
420 - 435	632.9656	1895.8750	1895.8692	3	0	K.VEFVHMLNATMCATTR.T	Oxidation (M)	(Ions score 74)
420 - 435	948.9448	1895.8750	1895.8692	3	0	K.VEFVHMLNATMCATTR.T	Oxidation (M)	(Ions score 44)
420 - 435	632.9656	1895.8750	1895.8692	3	0	K.VEFVHMLNATMCATTR.T	Oxidation (M)	(Ions score 60)
420 - 435	948.9448	1895.8751	1895.8692	3	0	K.VEFVHMLNATMCATTR.T	Oxidation (M)	(Ions score 25)
420 - 435	632.9656	1895.8751	1895.8692	3	0	K.VEFVHMLNATMCATTR.T	Oxidation (M)	(Ions score 62)
420 - 435	632.9657	1895.8751	1895.8692	3	0	K.VEFVHMLNATMCATTR.T	Oxidation (M)	(Ions score 52)
420 - 435	948.9449	1895.8752	1895.8692	3	0	K.VEFVHMLNATMCATTR.T	Oxidation (M)	(Ions score 44)
420 - 435	632.9657	1895.8752	1895.8692	3	0	K.VEFVHMLNATMCATTR.T	Oxidation (M)	(Ions score 64)
420 - 435	948.9449	1895.8752	1895.8692	3	0	K.VEFVHMLNATMCATTR.T	Oxidation (M)	(Ions score 39)
420 - 435	632.9657	1895.8752	1895.8692	3	0	K.VEFVHMLNATMCATTR.T	Oxidation (M)	(Ions score 61)
420 - 435	948.9449	1895.8752	1895.8692	3	0	K.VEFVHMLNATMCATTR.T	Oxidation (M)	(Ions score 33)
420 - 435	632.9657	1895.8752	1895.8692	3	0	K.VEFVHMLNATMCATTR.T	Oxidation (M)	(Ions score 50)
420 - 435	632.9657	1895.8752	1895.8692	3	0	K.VEFVHMLNATMCATTR.T	Oxidation (M)	(Ions score 61)
420 - 435	632.9657	1895.8752	1895.8692	3	0	K.VEFVHMLNATMCATTR.T	Oxidation (M)	(Ions score 29)
420 - 435	948.9449	1895.8752	1895.8692	3	0	K.VEFVHMLNATMCATTR.T	Oxidation (M)	(Ions score 40)
420 - 435	632.9657	1895.8753	1895.8692	3	0	K.VEFVHMLNATMCATTR.T	Oxidation (M)	(Ions score 62)
420 - 435	632.9657	1895.8753	1895.8692	3	0	K.VEFVHMLNATMCATTR.T	Oxidation (M)	(Ions score 64)
420 - 435	948.9449	1895.8753	1895.8692	3	0	K.VEFVHMLNATMCATTR.T	Oxidation (M)	(Ions score 36)
420 - 435	632.9657	1895.8753	1895.8692	3	0	K.VEFVHMLNATMCATTR.T	Oxidation (M)	(Ions score 74)
420 - 435	632.9657	1895.8753	1895.8692	3	0	K.VEFVHMLNATMCATTR.T	Oxidation (M)	(Ions score 63)
420 - 435	632.9657	1895.8754	1895.8692	3	0	K.VEFVHMLNATMCATTR.T	Oxidation (M)	(Ions score 54)
420 - 435	632.9657	1895.8754	1895.8692	3	0	K.VEFVHMLNATMCATTR.T	Oxidation (M)	(Ions score 65)
420 - 435	632.9657	1895.8754	1895.8692	3	0	K.VEFVHMLNATMCATTR.T	Oxidation (M)	(Ions score 71)
420 - 435	632.9657	1895.8754	1895.8692	3	0	K.VEFVHMLNATMCATTR.T	Oxidation (M)	(Ions score 74)
420 - 435	632.9658	1895.8754	1895.8692	3	0	K.VEFVHMLNATMCATTR.T	Oxidation (M)	(Ions score 57)
420 - 435	632.9658	1895.8754	1895.8692	3	0	K.VEFVHMLNATMCATTR.T	Oxidation (M)	(Ions score 73)
420 - 435	948.9450	1895.8755	1895.8692	3	0	K.VEFVHMLNATMCATTR.T	Oxidation (M)	(Ions score 28)
420 - 435	948.9450	1895.8755	1895.8692	3	0	K.VEFVHMLNATMCATTR.T	Oxidation (M)	(Ions score 32)

420 - 435	948.9450	1895.8755	1895.8692	3	0	K.VEFVHMLNATMCATTR.T	Oxidation (M)	(Ions score 35)
420 - 435	632.9658	1895.8755	1895.8692	3	0	K.VEFVHMLNATMCATTR.T	Oxidation (M)	(Ions score 63)
420 - 435	948.9450	1895.8755	1895.8692	3	0	K.VEFVHMLNATMCATTR.T	Oxidation (M)	(Ions score 27)
420 - 435	948.9450	1895.8755	1895.8692	3	0	K.VEFVHMLNATMCATTR.T	Oxidation (M)	(Ions score 34)
420 - 435	632.9658	1895.8756	1895.8692	3	0	K.VEFVHMLNATMCATTR.T	Oxidation (M)	(Ions score 59)
420 - 435	948.9451	1895.8756	1895.8692	3	0	K.VEFVHMLNATMCATTR.T	Oxidation (M)	(Ions score 41)
420 - 435	632.9658	1895.8756	1895.8692	3	0	K.VEFVHMLNATMCATTR.T	Oxidation (M)	(Ions score 64)
420 - 435	632.9658	1895.8756	1895.8692	3	0	K.VEFVHMLNATMCATTR.T	Oxidation (M)	(Ions score 71)
420 - 435	948.9451	1895.8756	1895.8692	3	0	K.VEFVHMLNATMCATTR.T	Oxidation (M)	(Ions score 42)
420 - 435	948.9451	1895.8757	1895.8692	3	0	K.VEFVHMLNATMCATTR.T	Oxidation (M)	(Ions score 33)
420 - 435	948.9452	1895.8758	1895.8692	3	0	K.VEFVHMLNATMCATTR.T	Oxidation (M)	(Ions score 31)
420 - 435	948.9452	1895.8758	1895.8692	3	0	K.VEFVHMLNATMCATTR.T	Oxidation (M)	(Ions score 27)
420 - 435	632.9659	1895.8758	1895.8692	3	0	K.VEFVHMLNATMCATTR.T	Oxidation (M)	(Ions score 65)
420 - 435	632.9659	1895.8758	1895.8692	3	0	K.VEFVHMLNATMCATTR.T	Oxidation (M)	(Ions score 57)
420 - 435	632.9659	1895.8758	1895.8692	3	0	K.VEFVHMLNATMCATTR.T	Oxidation (M)	(Ions score 58)
420 - 435	632.9659	1895.8758	1895.8692	3	0	K.VEFVHMLNATMCATTR.T	Oxidation (M)	(Ions score 72)
420 - 435	632.9659	1895.8758	1895.8692	3	0	K.VEFVHMLNATMCATTR.T	Oxidation (M)	(Ions score 57)
420 - 435	632.9659	1895.8759	1895.8692	4	0	K.VEFVHMLNATMCATTR.T	Oxidation (M)	(Ions score 54)
420 - 435	632.9659	1895.8759	1895.8692	4	0	K.VEFVHMLNATMCATTR.T	Oxidation (M)	(Ions score 61)
420 - 435	632.9659	1895.8760	1895.8692	4	0	K.VEFVHMLNATMCATTR.T	Oxidation (M)	(Ions score 65)
420 - 435	948.9453	1895.8760	1895.8692	4	0	K.VEFVHMLNATMCATTR.T	Oxidation (M)	(Ions score 36)
420 - 435	632.9659	1895.8760	1895.8692	4	0	K.VEFVHMLNATMCATTR.T	Oxidation (M)	(Ions score 49)
420 - 435	632.9659	1895.8760	1895.8692	4	0	K.VEFVHMLNATMCATTR.T	Oxidation (M)	(Ions score 56)
420 - 435	948.9453	1895.8760	1895.8692	4	0	K.VEFVHMLNATMCATTR.T	Oxidation (M)	(Ions score 43)
420 - 435	632.9660	1895.8761	1895.8692	4	0	K.VEFVHMLNATMCATTR.T	Oxidation (M)	(Ions score 74)
420 - 435	632.9660	1895.8761	1895.8692	4	0	K.VEFVHMLNATMCATTR.T	Oxidation (M)	(Ions score 52)
420 - 435	632.9660	1895.8761	1895.8692	4	0	K.VEFVHMLNATMCATTR.T	Oxidation (M)	(Ions score 35)
420 - 435	632.9660	1895.8761	1895.8692	4	0	K.VEFVHMLNATMCATTR.T	Oxidation (M)	(Ions score 50)
420 - 435	632.9660	1895.8761	1895.8692	4	0	K.VEFVHMLNATMCATTR.T	Oxidation (M)	(Ions score 75)
420 - 435	948.9453	1895.8761	1895.8692	4	0	K.VEFVHMLNATMCATTR.T	Oxidation (M)	(Ions score 34)
420 - 435	948.9453	1895.8761	1895.8692	4	0	K.VEFVHMLNATMCATTR.T	Oxidation (M)	(Ions score 46)
420 - 435	632.9660	1895.8762	1895.8692	4	0	K.VEFVHMLNATMCATTR.T	Oxidation (M)	(Ions score 40)
420 - 435	632.9660	1895.8762	1895.8692	4	0	K.VEFVHMLNATMCATTR.T	Oxidation (M)	(Ions score 71)
420 - 435	632.9660	1895.8762	1895.8692	4	0	K.VEFVHMLNATMCATTR.T	Oxidation (M)	(Ions score 61)
420 - 435	632.9660	1895.8762	1895.8692	4	0	K.VEFVHMLNATMCATTR.T	Oxidation (M)	(Ions score 73)
420 - 435	948.9454	1895.8762	1895.8692	4	0	K.VEFVHMLNATMCATTR.T	Oxidation (M)	(Ions score 48)
420 - 435	632.9660	1895.8763	1895.8692	4	0	K.VEFVHMLNATMCATTR.T	Oxidation (M)	(Ions score 74)
420 - 435	632.9660	1895.8763	1895.8692	4	0	K.VEFVHMLNATMCATTR.T	Oxidation (M)	(Ions score 70)
420 - 435	948.9454	1895.8763	1895.8692	4	0	K.VEFVHMLNATMCATTR.T	Oxidation (M)	(Ions score 23)
420 - 435	948.9455	1895.8764	1895.8692	4	0	K.VEFVHMLNATMCATTR.T	Oxidation (M)	(Ions score 27)
420 - 435	632.9661	1895.8764	1895.8692	4	0	K.VEFVHMLNATMCATTR.T	Oxidation (M)	(Ions score 59)
420 - 435	474.9764	1895.8764	1895.8692	4	0	K.VEFVHMLNATMCATTR.T	Oxidation (M)	(Ions score 39)
420 - 435	632.9661	1895.8764	1895.8692	4	0	K.VEFVHMLNATMCATTR.T	Oxidation (M)	(Ions score 42)
420 - 435	632.9661	1895.8764	1895.8692	4	0	K.VEFVHMLNATMCATTR.T	Oxidation (M)	(Ions score 73)
420 - 435	948.9455	1895.8764	1895.8692	4	0	K.VEFVHMLNATMCATTR.T	Oxidation (M)	(Ions score 34)
420 - 435	632.9661	1895.8764	1895.8692	4	0	K.VEFVHMLNATMCATTR.T	Oxidation (M)	(Ions score 65)
420 - 435	632.9661	1895.8764	1895.8692	4	0	K.VEFVHMLNATMCATTR.T	Oxidation (M)	(Ions score 63)
420 - 435	632.9661	1895.8765	1895.8692	4	0	K.VEFVHMLNATMCATTR.T	Oxidation (M)	(Ions score 77)
420 - 435	632.9661	1895.8765	1895.8692	4	0	K.VEFVHMLNATMCATTR.T	Oxidation (M)	(Ions score 72)
420 - 435	632.9661	1895.8765	1895.8692	4	0	K.VEFVHMLNATMCATTR.T	Oxidation (M)	(Ions score 40)
420 - 435	632.9661	1895.8765	1895.8692	4	0	K.VEFVHMLNATMCATTR.T	Oxidation (M)	(Ions score 75)
420 - 435	632.9661	1895.8765	1895.8692	4	0	K.VEFVHMLNATMCATTR.T	Oxidation (M)	(Ions score 40)
420 - 435	632.9661	1895.8766	1895.8692	4	0	K.VEFVHMLNATMCATTR.T	Oxidation (M)	(Ions score 65)
420 - 435	948.9456	1895.8766	1895.8692	4	0	K.VEFVHMLNATMCATTR.T	Oxidation (M)	(Ions score 30)
420 - 435	948.9456	1895.8767	1895.8692	4	0	K.VEFVHMLNATMCATTR.T	Oxidation (M)	(Ions score 35)
420 - 435	632.9662	1895.8767	1895.8692	4	0	K.VEFVHMLNATMCATTR.T	Oxidation (M)	(Ions score 64)
420 - 435	948.9456	1895.8767	1895.8692	4	0	K.VEFVHMLNATMCATTR.T	Oxidation (M)	(Ions score 29)
420 - 435	632.9662	1895.8767	1895.8692	4	0	K.VEFVHMLNATMCATTR.T	Oxidation (M)	(Ions score 56)

420 - 435	632.9662	1895.8767	1895.8692	4	0	K.VEFVHMLNATMCATTR.T	Oxidation (M)	(Ions score 59)
420 - 435	632.9662	1895.8768	1895.8692	4	0	K.VEFVHMLNATMCATTR.T	Oxidation (M)	(Ions score 73)
420 - 435	632.9662	1895.8768	1895.8692	4	0	K.VEFVHMLNATMCATTR.T	Oxidation (M)	(Ions score 58)
420 - 435	632.9662	1895.8769	1895.8692	4	0	K.VEFVHMLNATMCATTR.T	Oxidation (M)	(Ions score 75)
420 - 435	632.9662	1895.8769	1895.8692	4	0	K.VEFVHMLNATMCATTR.T	Oxidation (M)	(Ions score 58)
420 - 435	632.9662	1895.8769	1895.8692	4	0	K.VEFVHMLNATMCATTR.T	Oxidation (M)	(Ions score 46)
420 - 435	948.9457	1895.8769	1895.8692	4	0	K.VEFVHMLNATMCATTR.T	Oxidation (M)	(Ions score 48)
420 - 435	948.9457	1895.8769	1895.8692	4	0	K.VEFVHMLNATMCATTR.T	Oxidation (M)	(Ions score 49)
420 - 435	632.9662	1895.8769	1895.8692	4	0	K.VEFVHMLNATMCATTR.T	Oxidation (M)	(Ions score 37)
420 - 435	632.9663	1895.8769	1895.8692	4	0	K.VEFVHMLNATMCATTR.T	Oxidation (M)	(Ions score 66)
420 - 435	632.9663	1895.8770	1895.8692	4	0	K.VEFVHMLNATMCATTR.T	Oxidation (M)	(Ions score 79)
420 - 435	632.9663	1895.8770	1895.8692	4	0	K.VEFVHMLNATMCATTR.T	Oxidation (M)	(Ions score 58)
420 - 435	632.9663	1895.8770	1895.8692	4	0	K.VEFVHMLNATMCATTR.T	Oxidation (M)	(Ions score 41)
420 - 435	632.9663	1895.8770	1895.8692	4	0	K.VEFVHMLNATMCATTR.T	Oxidation (M)	(Ions score 60)
420 - 435	948.9458	1895.8770	1895.8692	4	0	K.VEFVHMLNATMCATTR.T	Oxidation (M)	(Ions score 32)
420 - 435	632.9663	1895.8771	1895.8692	4	0	K.VEFVHMLNATMCATTR.T	Oxidation (M)	(Ions score 68)
420 - 435	632.9663	1895.8771	1895.8692	4	0	K.VEFVHMLNATMCATTR.T	Oxidation (M)	(Ions score 62)
420 - 435	632.9663	1895.8772	1895.8692	4	0	K.VEFVHMLNATMCATTR.T	Oxidation (M)	(Ions score 63)
420 - 435	632.9663	1895.8772	1895.8692	4	0	K.VEFVHMLNATMCATTR.T	Oxidation (M)	(Ions score 58)
420 - 435	948.9459	1895.8772	1895.8692	4	0	K.VEFVHMLNATMCATTR.T	Oxidation (M)	(Ions score 33)
420 - 435	632.9664	1895.8772	1895.8692	4	0	K.VEFVHMLNATMCATTR.T	Oxidation (M)	(Ions score 58)
420 - 435	948.9459	1895.8772	1895.8692	4	0	K.VEFVHMLNATMCATTR.T	Oxidation (M)	(Ions score 34)
420 - 435	948.9459	1895.8773	1895.8692	4	0	K.VEFVHMLNATMCATTR.T	Oxidation (M)	(Ions score 32)
420 - 435	948.9459	1895.8773	1895.8692	4	0	K.VEFVHMLNATMCATTR.T	Oxidation (M)	(Ions score 36)
420 - 435	632.9664	1895.8773	1895.8692	4	0	K.VEFVHMLNATMCATTR.T	Oxidation (M)	(Ions score 40)
420 - 435	632.9664	1895.8773	1895.8692	4	0	K.VEFVHMLNATMCATTR.T	Oxidation (M)	(Ions score 37)
420 - 435	632.9664	1895.8773	1895.8692	4	0	K.VEFVHMLNATMCATTR.T	Oxidation (M)	(Ions score 52)
420 - 435	632.9664	1895.8773	1895.8692	4	0	K.VEFVHMLNATMCATTR.T	Oxidation (M)	(Ions score 23)
420 - 435	632.9664	1895.8773	1895.8692	4	0	K.VEFVHMLNATMCATTR.T	Oxidation (M)	(Ions score 55)
420 - 435	632.9664	1895.8773	1895.8692	4	0	K.VEFVHMLNATMCATTR.T	Oxidation (M)	(Ions score 59)
420 - 435	632.9664	1895.8774	1895.8692	4	0	K.VEFVHMLNATMCATTR.T	Oxidation (M)	(Ions score 73)
420 - 435	632.9664	1895.8774	1895.8692	4	0	K.VEFVHMLNATMCATTR.T	Oxidation (M)	(Ions score 57)
420 - 435	632.9664	1895.8774	1895.8692	4	0	K.VEFVHMLNATMCATTR.T	Oxidation (M)	(Ions score 69)
420 - 435	632.9664	1895.8774	1895.8692	4	0	K.VEFVHMLNATMCATTR.T	Oxidation (M)	(Ions score 62)
420 - 435	632.9664	1895.8774	1895.8692	4	0	K.VEFVHMLNATMCATTR.T	Oxidation (M)	(Ions score 28)
420 - 435	632.9664	1895.8775	1895.8692	4	0	K.VEFVHMLNATMCATTR.T	Oxidation (M)	(Ions score 73)
420 - 435	632.9664	1895.8775	1895.8692	4	0	K.VEFVHMLNATMCATTR.T	Oxidation (M)	(Ions score 73)
420 - 435	948.9461	1895.8776	1895.8692	4	0	K.VEFVHMLNATMCATTR.T	Oxidation (M)	(Ions score 51)
420 - 435	948.9462	1895.8778	1895.8692	5	0	K.VEFVHMLNATMCATTR.T	Oxidation (M)	(Ions score 29)
420 - 435	632.9666	1895.8778	1895.8692	5	0	K.VEFVHMLNATMCATTR.T	Oxidation (M)	(Ions score 72)
420 - 435	632.9666	1895.8778	1895.8692	5	0	K.VEFVHMLNATMCATTR.T	Oxidation (M)	(Ions score 36)
420 - 435	632.9666	1895.8779	1895.8692	5	0	K.VEFVHMLNATMCATTR.T	Oxidation (M)	(Ions score 75)
420 - 435	632.9666	1895.8780	1895.8692	5	0	K.VEFVHMLNATMCATTR.T	Oxidation (M)	(Ions score 73)
420 - 435	632.9666	1895.8780	1895.8692	5	0	K.VEFVHMLNATMCATTR.T	Oxidation (M)	(Ions score 57)
420 - 435	632.9666	1895.8780	1895.8692	5	0	K.VEFVHMLNATMCATTR.T	Oxidation (M)	(Ions score 61)
420 - 435	632.9666	1895.8780	1895.8692	5	0	K.VEFVHMLNATMCATTR.T	Oxidation (M)	(Ions score 62)
420 - 435	632.9666	1895.8780	1895.8692	5	0	K.VEFVHMLNATMCATTR.T	Oxidation (M)	(Ions score 37)
420 - 435	632.9666	1895.8780	1895.8692	5	0	K.VEFVHMLNATMCATTR.T	Oxidation (M)	(Ions score 72)
420 - 435	948.9463	1895.8781	1895.8692	5	0	K.VEFVHMLNATMCATTR.T	Oxidation (M)	(Ions score 37)
420 - 435	632.9667	1895.8782	1895.8692	5	0	K.VEFVHMLNATMCATTR.T	Oxidation (M)	(Ions score 59)
420 - 435	948.9464	1895.8782	1895.8692	5	0	K.VEFVHMLNATMCATTR.T	Oxidation (M)	(Ions score 38)
420 - 435	632.9667	1895.8782	1895.8692	5	0	K.VEFVHMLNATMCATTR.T	Oxidation (M)	(Ions score 67)
420 - 435	948.9464	1895.8783	1895.8692	5	0	K.VEFVHMLNATMCATTR.T	Oxidation (M)	(Ions score 45)
420 - 435	632.9667	1895.8783	1895.8692	5	0	K.VEFVHMLNATMCATTR.T	Oxidation (M)	(Ions score 64)
420 - 435	632.9667	1895.8783	1895.8692	5	0	K.VEFVHMLNATMCATTR.T	Oxidation (M)	(Ions score 62)
420 - 435	948.9465	1895.8784	1895.8692	5	0	K.VEFVHMLNATMCATTR.T	Oxidation (M)	(Ions score 29)
420 - 435	632.9667	1895.8784	1895.8692	5	0	K.VEFVHMLNATMCATTR.T	Oxidation (M)	(Ions score 39)
420 - 435	632.9668	1895.8784	1895.8692	5	0	K.VEFVHMLNATMCATTR.T	Oxidation (M)	(Ions score 76)

420 - 435	948.9466	1895.8786	1895.8692	5	0	K.VEFVHMLNATMCATTR.T	Oxidation (M)	(Ions score 62)
420 - 435	948.9466	1895.8786	1895.8692	5	0	K.VEFVHMLNATMCATTR.T	Oxidation (M)	(Ions score 38)
420 - 435	638.2934	1911.8584	1911.8641	-3	0	K.VEFVHMLNATMCATTR.T	2 Oxidation (M)	(Ions score 34)
420 - 435	956.9375	1911.8605	1911.8641	-2	0	K.VEFVHMLNATMCATTR.T	2 Oxidation (M)	(Ions score 27)
420 - 435	638.2945	1911.8617	1911.8641	-1	0	K.VEFVHMLNATMCATTR.T	2 Oxidation (M)	(Ions score 28)
420 - 435	638.2946	1911.8619	1911.8641	-1	0	K.VEFVHMLNATMCATTR.T	2 Oxidation (M)	(Ions score 32)
420 - 435	638.2946	1911.8621	1911.8641	-1	0	K.VEFVHMLNATMCATTR.T	2 Oxidation (M)	(Ions score 23)
420 - 435	638.2947	1911.8623	1911.8641	-1	0	K.VEFVHMLNATMCATTR.T	2 Oxidation (M)	(Ions score 26)
420 - 435	638.2947	1911.8624	1911.8641	-1	0	K.VEFVHMLNATMCATTR.T	2 Oxidation (M)	(Ions score 56)
420 - 435	638.2948	1911.8624	1911.8641	-1	0	K.VEFVHMLNATMCATTR.T	2 Oxidation (M)	(Ions score 45)
420 - 435	956.9385	1911.8625	1911.8641	-1	0	K.VEFVHMLNATMCATTR.T	2 Oxidation (M)	(Ions score 35)
420 - 435	638.2948	1911.8625	1911.8641	-1	0	K.VEFVHMLNATMCATTR.T	2 Oxidation (M)	(Ions score 42)
420 - 435	638.2949	1911.8629	1911.8641	-1	0	K.VEFVHMLNATMCATTR.T	2 Oxidation (M)	(Ions score 31)
420 - 435	638.2949	1911.8630	1911.8641	-1	0	K.VEFVHMLNATMCATTR.T	2 Oxidation (M)	(Ions score 29)
420 - 435	638.2949	1911.8630	1911.8641	-1	0	K.VEFVHMLNATMCATTR.T	2 Oxidation (M)	(Ions score 29)
420 - 435	956.9388	1911.8630	1911.8641	-1	0	K.VEFVHMLNATMCATTR.T	2 Oxidation (M)	(Ions score 25)
420 - 435	638.2950	1911.8631	1911.8641	-1	0	K.VEFVHMLNATMCATTR.T	2 Oxidation (M)	(Ions score 44)
420 - 435	638.2950	1911.8632	1911.8641	-0	0	K.VEFVHMLNATMCATTR.T	2 Oxidation (M)	(Ions score 27)
420 - 435	956.9390	1911.8634	1911.8641	-0	0	K.VEFVHMLNATMCATTR.T	2 Oxidation (M)	(Ions score 29)
420 - 435	638.2951	1911.8635	1911.8641	-0	0	K.VEFVHMLNATMCATTR.T	2 Oxidation (M)	(Ions score 36)
420 - 435	638.2951	1911.8635	1911.8641	-0	0	K.VEFVHMLNATMCATTR.T	2 Oxidation (M)	(Ions score 27)
420 - 435	638.2951	1911.8636	1911.8641	-0	0	K.VEFVHMLNATMCATTR.T	2 Oxidation (M)	(Ions score 37)
420 - 435	638.2951	1911.8636	1911.8641	-0	0	K.VEFVHMLNATMCATTR.T	2 Oxidation (M)	(Ions score 42)
420 - 435	638.2952	1911.8636	1911.8641	-0	0	K.VEFVHMLNATMCATTR.T	2 Oxidation (M)	(Ions score 37)
420 - 435	638.2952	1911.8637	1911.8641	-0	0	K.VEFVHMLNATMCATTR.T	2 Oxidation (M)	(Ions score 36)
420 - 435	638.2952	1911.8637	1911.8641	-0	0	K.VEFVHMLNATMCATTR.T	2 Oxidation (M)	(Ions score 34)
420 - 435	956.9392	1911.8638	1911.8641	-0	0	K.VEFVHMLNATMCATTR.T	2 Oxidation (M)	(Ions score 24)
420 - 435	956.9392	1911.8638	1911.8641	-0	0	K.VEFVHMLNATMCATTR.T	2 Oxidation (M)	(Ions score 22)
420 - 435	638.2952	1911.8638	1911.8641	-0	0	K.VEFVHMLNATMCATTR.T	2 Oxidation (M)	(Ions score 27)
420 - 435	638.2952	1911.8638	1911.8641	-0	0	K.VEFVHMLNATMCATTR.T	2 Oxidation (M)	(Ions score 30)
420 - 435	638.2952	1911.8638	1911.8641	-0	0	K.VEFVHMLNATMCATTR.T	2 Oxidation (M)	(Ions score 44)
420 - 435	638.2952	1911.8639	1911.8641	-0	0	K.VEFVHMLNATMCATTR.T	2 Oxidation (M)	(Ions score 41)
420 - 435	638.2954	1911.8644	1911.8641	0	0	K.VEFVHMLNATMCATTR.T	2 Oxidation (M)	(Ions score 30)
420 - 435	638.2954	1911.8644	1911.8641	0	0	K.VEFVHMLNATMCATTR.T	2 Oxidation (M)	(Ions score 29)
420 - 435	638.2954	1911.8644	1911.8641	0	0	K.VEFVHMLNATMCATTR.T	2 Oxidation (M)	(Ions score 42)
420 - 435	956.9395	1911.8645	1911.8641	0	0	K.VEFVHMLNATMCATTR.T	2 Oxidation (M)	(Ions score 23)
420 - 435	638.2955	1911.8645	1911.8641	0	0	K.VEFVHMLNATMCATTR.T	2 Oxidation (M)	(Ions score 33)
420 - 435	638.2955	1911.8646	1911.8641	0	0	K.VEFVHMLNATMCATTR.T	2 Oxidation (M)	(Ions score 29)
420 - 435	638.2955	1911.8646	1911.8641	0	0	K.VEFVHMLNATMCATTR.T	2 Oxidation (M)	(Ions score 25)
420 - 435	638.2955	1911.8647	1911.8641	0	0	K.VEFVHMLNATMCATTR.T	2 Oxidation (M)	(Ions score 56)
420 - 435	956.9396	1911.8647	1911.8641	0	0	K.VEFVHMLNATMCATTR.T	2 Oxidation (M)	(Ions score 23)
420 - 435	638.2955	1911.8647	1911.8641	0	0	K.VEFVHMLNATMCATTR.T	2 Oxidation (M)	(Ions score 27)
420 - 435	638.2957	1911.8652	1911.8641	1	0	K.VEFVHMLNATMCATTR.T	2 Oxidation (M)	(Ions score 85)
420 - 435	638.2957	1911.8652	1911.8641	1	0	K.VEFVHMLNATMCATTR.T	2 Oxidation (M)	(Ions score 56)
420 - 435	638.2957	1911.8652	1911.8641	1	0	K.VEFVHMLNATMCATTR.T	2 Oxidation (M)	(Ions score 21)
420 - 435	638.2957	1911.8653	1911.8641	1	0	K.VEFVHMLNATMCATTR.T	2 Oxidation (M)	(Ions score 22)
420 - 435	638.2957	1911.8653	1911.8641	1	0	K.VEFVHMLNATMCATTR.T	2 Oxidation (M)	(Ions score 26)
420 - 435	638.2957	1911.8654	1911.8641	1	0	K.VEFVHMLNATMCATTR.T	2 Oxidation (M)	(Ions score 32)
420 - 435	956.9400	1911.8655	1911.8641	1	0	K.VEFVHMLNATMCATTR.T	2 Oxidation (M)	(Ions score 39)
420 - 435	638.2958	1911.8655	1911.8641	1	0	K.VEFVHMLNATMCATTR.T	2 Oxidation (M)	(Ions score 23)
420 - 435	638.2958	1911.8655	1911.8641	1	0	K.VEFVHMLNATMCATTR.T	2 Oxidation (M)	(Ions score 25)
420 - 435	638.2958	1911.8655	1911.8641	1	0	K.VEFVHMLNATMCATTR.T	2 Oxidation (M)	(Ions score 25)
420 - 435	638.2958	1911.8656	1911.8641	1	0	K.VEFVHMLNATMCATTR.T	2 Oxidation (M)	(Ions score 22)
420 - 435	638.2958	1911.8656	1911.8641	1	0	K.VEFVHMLNATMCATTR.T	2 Oxidation (M)	(Ions score 37)
420 - 435	638.2958	1911.8657	1911.8641	1	0	K.VEFVHMLNATMCATTR.T	2 Oxidation (M)	(Ions score 27)
420 - 435	638.2959	1911.8659	1911.8641	1	0	K.VEFVHMLNATMCATTR.T	2 Oxidation (M)	(Ions score 56)
420 - 435	638.2959	1911.8659	1911.8641	1	0	K.VEFVHMLNATMCATTR.T	2 Oxidation (M)	(Ions score 55)
420 - 435	956.9402	1911.8659	1911.8641	1	0	K.VEFVHMLNATMCATTR.T	2 Oxidation (M)	(Ions score 28)

420 - 435	638.2959	1911.8660	1911.8641	1	0	K.VEFVHMLNATMCATTR.T	2 Oxidation (M)	(Ions score 37)
420 - 435	956.9403	1911.8661	1911.8641	1	0	K.VEFVHMLNATMCATTR.T	2 Oxidation (M)	(Ions score 40)
420 - 435	956.9403	1911.8661	1911.8641	1	0	K.VEFVHMLNATMCATTR.T	2 Oxidation (M)	(Ions score 40)
420 - 435	956.9404	1911.8662	1911.8641	1	0	K.VEFVHMLNATMCATTR.T	2 Oxidation (M)	(Ions score 23)
420 - 435	638.2960	1911.8662	1911.8641	1	0	K.VEFVHMLNATMCATTR.T	2 Oxidation (M)	(Ions score 35)
420 - 435	638.2960	1911.8662	1911.8641	1	0	K.VEFVHMLNATMCATTR.T	2 Oxidation (M)	(Ions score 47)
420 - 435	638.2960	1911.8662	1911.8641	1	0	K.VEFVHMLNATMCATTR.T	2 Oxidation (M)	(Ions score 35)
420 - 435	638.2960	1911.8663	1911.8641	1	0	K.VEFVHMLNATMCATTR.T	2 Oxidation (M)	(Ions score 45)
420 - 435	638.2961	1911.8664	1911.8641	1	0	K.VEFVHMLNATMCATTR.T	2 Oxidation (M)	(Ions score 42)
420 - 435	638.2961	1911.8664	1911.8641	1	0	K.VEFVHMLNATMCATTR.T	2 Oxidation (M)	(Ions score 44)
420 - 435	638.2961	1911.8665	1911.8641	1	0	K.VEFVHMLNATMCATTR.T	2 Oxidation (M)	(Ions score 36)
420 - 435	638.2961	1911.8665	1911.8641	1	0	K.VEFVHMLNATMCATTR.T	2 Oxidation (M)	(Ions score 29)
420 - 435	956.9406	1911.8665	1911.8641	1	0	K.VEFVHMLNATMCATTR.T	2 Oxidation (M)	(Ions score 27)
420 - 435	638.2961	1911.8666	1911.8641	1	0	K.VEFVHMLNATMCATTR.T	2 Oxidation (M)	(Ions score 28)
420 - 435	638.2961	1911.8666	1911.8641	1	0	K.VEFVHMLNATMCATTR.T	2 Oxidation (M)	(Ions score 27)
420 - 435	638.2962	1911.8667	1911.8641	1	0	K.VEFVHMLNATMCATTR.T	2 Oxidation (M)	(Ions score 57)
420 - 435	638.2962	1911.8667	1911.8641	1	0	K.VEFVHMLNATMCATTR.T	2 Oxidation (M)	(Ions score 41)
420 - 435	638.2962	1911.8668	1911.8641	1	0	K.VEFVHMLNATMCATTR.T	2 Oxidation (M)	(Ions score 52)
420 - 435	638.2962	1911.8668	1911.8641	1	0	K.VEFVHMLNATMCATTR.T	2 Oxidation (M)	(Ions score 38)
420 - 435	638.2962	1911.8668	1911.8641	1	0	K.VEFVHMLNATMCATTR.T	2 Oxidation (M)	(Ions score 50)
420 - 435	956.9407	1911.8668	1911.8641	1	0	K.VEFVHMLNATMCATTR.T	2 Oxidation (M)	(Ions score 23)
420 - 435	956.9407	1911.8669	1911.8641	1	0	K.VEFVHMLNATMCATTR.T	2 Oxidation (M)	(Ions score 33)
420 - 435	638.2962	1911.8669	1911.8641	1	0	K.VEFVHMLNATMCATTR.T	2 Oxidation (M)	(Ions score 49)
420 - 435	638.2963	1911.8669	1911.8641	1	0	K.VEFVHMLNATMCATTR.T	2 Oxidation (M)	(Ions score 45)
420 - 435	638.2963	1911.8669	1911.8641	1	0	K.VEFVHMLNATMCATTR.T	2 Oxidation (M)	(Ions score 29)
420 - 435	638.2963	1911.8670	1911.8641	1	0	K.VEFVHMLNATMCATTR.T	2 Oxidation (M)	(Ions score 24)
420 - 435	638.2963	1911.8670	1911.8641	1	0	K.VEFVHMLNATMCATTR.T	2 Oxidation (M)	(Ions score 31)
420 - 435	956.9408	1911.8670	1911.8641	1	0	K.VEFVHMLNATMCATTR.T	2 Oxidation (M)	(Ions score 29)
420 - 435	638.2963	1911.8670	1911.8641	2	0	K.VEFVHMLNATMCATTR.T	2 Oxidation (M)	(Ions score 30)
420 - 435	638.2963	1911.8671	1911.8641	2	0	K.VEFVHMLNATMCATTR.T	2 Oxidation (M)	(Ions score 35)
420 - 435	638.2963	1911.8671	1911.8641	2	0	K.VEFVHMLNATMCATTR.T	2 Oxidation (M)	(Ions score 38)
420 - 435	638.2963	1911.8671	1911.8641	2	0	K.VEFVHMLNATMCATTR.T	2 Oxidation (M)	(Ions score 29)
420 - 435	956.9409	1911.8672	1911.8641	2	0	K.VEFVHMLNATMCATTR.T	2 Oxidation (M)	(Ions score 43)
420 - 435	638.2963	1911.8672	1911.8641	2	0	K.VEFVHMLNATMCATTR.T	2 Oxidation (M)	(Ions score 54)
420 - 435	638.2963	1911.8672	1911.8641	2	0	K.VEFVHMLNATMCATTR.T	2 Oxidation (M)	(Ions score 26)
420 - 435	638.2964	1911.8672	1911.8641	2	0	K.VEFVHMLNATMCATTR.T	2 Oxidation (M)	(Ions score 21)
420 - 435	638.2964	1911.8672	1911.8641	2	0	K.VEFVHMLNATMCATTR.T	2 Oxidation (M)	(Ions score 41)
420 - 435	638.2964	1911.8673	1911.8641	2	0	K.VEFVHMLNATMCATTR.T	2 Oxidation (M)	(Ions score 26)
420 - 435	956.9409	1911.8673	1911.8641	2	0	K.VEFVHMLNATMCATTR.T	2 Oxidation (M)	(Ions score 27)
420 - 435	638.2964	1911.8673	1911.8641	2	0	K.VEFVHMLNATMCATTR.T	2 Oxidation (M)	(Ions score 49)
420 - 435	638.2964	1911.8674	1911.8641	2	0	K.VEFVHMLNATMCATTR.T	2 Oxidation (M)	(Ions score 37)
420 - 435	638.2964	1911.8674	1911.8641	2	0	K.VEFVHMLNATMCATTR.T	2 Oxidation (M)	(Ions score 40)
420 - 435	638.2964	1911.8675	1911.8641	2	0	K.VEFVHMLNATMCATTR.T	2 Oxidation (M)	(Ions score 24)
420 - 435	638.2964	1911.8675	1911.8641	2	0	K.VEFVHMLNATMCATTR.T	2 Oxidation (M)	(Ions score 31)
420 - 435	638.2965	1911.8676	1911.8641	2	0	K.VEFVHMLNATMCATTR.T	2 Oxidation (M)	(Ions score 44)
420 - 435	956.9411	1911.8676	1911.8641	2	0	K.VEFVHMLNATMCATTR.T	2 Oxidation (M)	(Ions score 34)
420 - 435	638.2965	1911.8677	1911.8641	2	0	K.VEFVHMLNATMCATTR.T	2 Oxidation (M)	(Ions score 46)
420 - 435	638.2965	1911.8677	1911.8641	2	0	K.VEFVHMLNATMCATTR.T	2 Oxidation (M)	(Ions score 39)
420 - 435	956.9411	1911.8677	1911.8641	2	0	K.VEFVHMLNATMCATTR.T	2 Oxidation (M)	(Ions score 23)
420 - 435	638.2965	1911.8678	1911.8641	2	0	K.VEFVHMLNATMCATTR.T	2 Oxidation (M)	(Ions score 72)
420 - 435	956.9412	1911.8678	1911.8641	2	0	K.VEFVHMLNATMCATTR.T	2 Oxidation (M)	(Ions score 29)
420 - 435	956.9412	1911.8679	1911.8641	2	0	K.VEFVHMLNATMCATTR.T	2 Oxidation (M)	(Ions score 24)
420 - 435	638.2966	1911.8679	1911.8641	2	0	K.VEFVHMLNATMCATTR.T	2 Oxidation (M)	(Ions score 33)
420 - 435	638.2966	1911.8680	1911.8641	2	0	K.VEFVHMLNATMCATTR.T	2 Oxidation (M)	(Ions score 34)
420 - 435	638.2966	1911.8680	1911.8641	2	0	K.VEFVHMLNATMCATTR.T	2 Oxidation (M)	(Ions score 54)
420 - 435	638.2966	1911.8681	1911.8641	2	0	K.VEFVHMLNATMCATTR.T	2 Oxidation (M)	(Ions score 38)
420 - 435	638.2966	1911.8681	1911.8641	2	0	K.VEFVHMLNATMCATTR.T	2 Oxidation (M)	(Ions score 36)
420 - 435	638.2967	1911.8681	1911.8641	2	0	K.VEFVHMLNATMCATTR.T	2 Oxidation (M)	(Ions score 45)

420 - 435	956.9414	1911.8682	1911.8641	2	0	K.VEFVHMLNATMCATTR.T	2 Oxidation (M) (Ions score 40)
420 - 435	638.2967	1911.8682	1911.8641	2	0	K.VEFVHMLNATMCATTR.T	2 Oxidation (M) (Ions score 25)
420 - 435	638.2967	1911.8682	1911.8641	2	0	K.VEFVHMLNATMCATTR.T	2 Oxidation (M) (Ions score 27)
420 - 435	638.2967	1911.8683	1911.8641	2	0	K.VEFVHMLNATMCATTR.T	2 Oxidation (M) (Ions score 35)
420 - 435	638.2967	1911.8683	1911.8641	2	0	K.VEFVHMLNATMCATTR.T	2 Oxidation (M) (Ions score 58)
420 - 435	638.2967	1911.8684	1911.8641	2	0	K.VEFVHMLNATMCATTR.T	2 Oxidation (M) (Ions score 25)
420 - 435	638.2968	1911.8684	1911.8641	2	0	K.VEFVHMLNATMCATTR.T	2 Oxidation (M) (Ions score 54)
420 - 435	638.2968	1911.8685	1911.8641	2	0	K.VEFVHMLNATMCATTR.T	2 Oxidation (M) (Ions score 41)
420 - 435	638.2968	1911.8687	1911.8641	2	0	K.VEFVHMLNATMCATTR.T	2 Oxidation (M) (Ions score 27)
420 - 435	956.9416	1911.8687	1911.8641	2	0	K.VEFVHMLNATMCATTR.T	2 Oxidation (M) (Ions score 27)
420 - 435	638.2968	1911.8687	1911.8641	2	0	K.VEFVHMLNATMCATTR.T	2 Oxidation (M) (Ions score 56)
420 - 435	638.2969	1911.8688	1911.8641	2	0	K.VEFVHMLNATMCATTR.T	2 Oxidation (M) (Ions score 36)
420 - 435	638.2969	1911.8688	1911.8641	2	0	K.VEFVHMLNATMCATTR.T	2 Oxidation (M) (Ions score 43)
420 - 435	638.2969	1911.8688	1911.8641	2	0	K.VEFVHMLNATMCATTR.T	2 Oxidation (M) (Ions score 38)
420 - 435	638.2969	1911.8688	1911.8641	2	0	K.VEFVHMLNATMCATTR.T	2 Oxidation (M) (Ions score 30)
420 - 435	956.9417	1911.8688	1911.8641	2	0	K.VEFVHMLNATMCATTR.T	2 Oxidation (M) (Ions score 43)
420 - 435	638.2969	1911.8689	1911.8641	2	0	K.VEFVHMLNATMCATTR.T	2 Oxidation (M) (Ions score 25)
420 - 435	956.9417	1911.8689	1911.8641	3	0	K.VEFVHMLNATMCATTR.T	2 Oxidation (M) (Ions score 22)
420 - 435	638.2969	1911.8690	1911.8641	3	0	K.VEFVHMLNATMCATTR.T	2 Oxidation (M) (Ions score 33)
420 - 435	638.2969	1911.8690	1911.8641	3	0	K.VEFVHMLNATMCATTR.T	2 Oxidation (M) (Ions score 61)
420 - 435	638.2969	1911.8690	1911.8641	3	0	K.VEFVHMLNATMCATTR.T	2 Oxidation (M) (Ions score 34)
420 - 435	956.9418	1911.8691	1911.8641	3	0	K.VEFVHMLNATMCATTR.T	2 Oxidation (M) (Ions score 42)
420 - 435	638.2970	1911.8691	1911.8641	3	0	K.VEFVHMLNATMCATTR.T	2 Oxidation (M) (Ions score 57)
420 - 435	638.2970	1911.8691	1911.8641	3	0	K.VEFVHMLNATMCATTR.T	2 Oxidation (M) (Ions score 23)
420 - 435	638.2970	1911.8691	1911.8641	3	0	K.VEFVHMLNATMCATTR.T	2 Oxidation (M) (Ions score 57)
420 - 435	638.2970	1911.8691	1911.8641	3	0	K.VEFVHMLNATMCATTR.T	2 Oxidation (M) (Ions score 56)
420 - 435	638.2970	1911.8691	1911.8641	3	0	K.VEFVHMLNATMCATTR.T	2 Oxidation (M) (Ions score 57)
420 - 435	638.2970	1911.8692	1911.8641	3	0	K.VEFVHMLNATMCATTR.T	2 Oxidation (M) (Ions score 56)
420 - 435	638.2970	1911.8692	1911.8641	3	0	K.VEFVHMLNATMCATTR.T	2 Oxidation (M) (Ions score 36)
420 - 435	638.2970	1911.8692	1911.8641	3	0	K.VEFVHMLNATMCATTR.T	2 Oxidation (M) (Ions score 25)
420 - 435	638.2970	1911.8693	1911.8641	3	0	K.VEFVHMLNATMCATTR.T	2 Oxidation (M) (Ions score 54)
420 - 435	638.2970	1911.8693	1911.8641	3	0	K.VEFVHMLNATMCATTR.T	2 Oxidation (M) (Ions score 26)
420 - 435	638.2970	1911.8693	1911.8641	3	0	K.VEFVHMLNATMCATTR.T	2 Oxidation (M) (Ions score 38)
420 - 435	638.2970	1911.8693	1911.8641	3	0	K.VEFVHMLNATMCATTR.T	2 Oxidation (M) (Ions score 43)
420 - 435	638.2971	1911.8693	1911.8641	3	0	K.VEFVHMLNATMCATTR.T	2 Oxidation (M) (Ions score 45)
420 - 435	956.9420	1911.8694	1911.8641	3	0	K.VEFVHMLNATMCATTR.T	2 Oxidation (M) (Ions score 43)
420 - 435	638.2971	1911.8694	1911.8641	3	0	K.VEFVHMLNATMCATTR.T	2 Oxidation (M) (Ions score 54)
420 - 435	638.2971	1911.8694	1911.8641	3	0	K.VEFVHMLNATMCATTR.T	2 Oxidation (M) (Ions score 57)
420 - 435	956.9420	1911.8694	1911.8641	3	0	K.VEFVHMLNATMCATTR.T	2 Oxidation (M) (Ions score 43)
420 - 435	638.2971	1911.8695	1911.8641	3	0	K.VEFVHMLNATMCATTR.T	2 Oxidation (M) (Ions score 32)
420 - 435	638.2971	1911.8695	1911.8641	3	0	K.VEFVHMLNATMCATTR.T	2 Oxidation (M) (Ions score 54)
420 - 435	638.2971	1911.8695	1911.8641	3	0	K.VEFVHMLNATMCATTR.T	2 Oxidation (M) (Ions score 45)
420 - 435	638.2971	1911.8695	1911.8641	3	0	K.VEFVHMLNATMCATTR.T	2 Oxidation (M) (Ions score 32)
420 - 435	638.2971	1911.8696	1911.8641	3	0	K.VEFVHMLNATMCATTR.T	2 Oxidation (M) (Ions score 55)
420 - 435	638.2971	1911.8696	1911.8641	3	0	K.VEFVHMLNATMCATTR.T	2 Oxidation (M) (Ions score 57)
420 - 435	638.2972	1911.8696	1911.8641	3	0	K.VEFVHMLNATMCATTR.T	2 Oxidation (M) (Ions score 47)
420 - 435	638.2972	1911.8696	1911.8641	3	0	K.VEFVHMLNATMCATTR.T	2 Oxidation (M) (Ions score 39)
420 - 435	638.2972	1911.8697	1911.8641	3	0	K.VEFVHMLNATMCATTR.T	2 Oxidation (M) (Ions score 37)
420 - 435	638.2972	1911.8697	1911.8641	3	0	K.VEFVHMLNATMCATTR.T	2 Oxidation (M) (Ions score 32)
420 - 435	638.2972	1911.8697	1911.8641	3	0	K.VEFVHMLNATMCATTR.T	2 Oxidation (M) (Ions score 57)
420 - 435	638.2972	1911.8697	1911.8641	3	0	K.VEFVHMLNATMCATTR.T	2 Oxidation (M) (Ions score 28)
420 - 435	638.2972	1911.8698	1911.8641	3	0	K.VEFVHMLNATMCATTR.T	2 Oxidation (M) (Ions score 29)
420 - 435	956.9422	1911.8699	1911.8641	3	0	K.VEFVHMLNATMCATTR.T	2 Oxidation (M) (Ions score 32)
420 - 435	638.2973	1911.8700	1911.8641	3	0	K.VEFVHMLNATMCATTR.T	2 Oxidation (M) (Ions score 34)
420 - 435	638.2973	1911.8700	1911.8641	3	0	K.VEFVHMLNATMCATTR.T	2 Oxidation (M) (Ions score 41)
420 - 435	956.9423	1911.8701	1911.8641	3	0	K.VEFVHMLNATMCATTR.T	2 Oxidation (M) (Ions score 43)
420 - 435	956.9424	1911.8701	1911.8641	3	0	K.VEFVHMLNATMCATTR.T	2 Oxidation (M) (Ions score 32)
420 - 435	638.2973	1911.8702	1911.8641	3	0	K.VEFVHMLNATMCATTR.T	2 Oxidation (M) (Ions score 58)

420 - 435	638.2973	1911.8702	1911.8641	3	0	K.VEFVHMLNATMCATTR.T	2 Oxidation (M)	(Ions score 56)
420 - 435	956.9424	1911.8702	1911.8641	3	0	K.VEFVHMLNATMCATTR.T	2 Oxidation (M)	(Ions score 29)
420 - 435	638.2974	1911.8702	1911.8641	3	0	K.VEFVHMLNATMCATTR.T	2 Oxidation (M)	(Ions score 48)
420 - 435	638.2974	1911.8703	1911.8641	3	0	K.VEFVHMLNATMCATTR.T	2 Oxidation (M)	(Ions score 32)
420 - 435	638.2974	1911.8704	1911.8641	3	0	K.VEFVHMLNATMCATTR.T	2 Oxidation (M)	(Ions score 36)
420 - 435	638.2974	1911.8705	1911.8641	3	0	K.VEFVHMLNATMCATTR.T	2 Oxidation (M)	(Ions score 58)
420 - 435	638.2974	1911.8705	1911.8641	3	0	K.VEFVHMLNATMCATTR.T	2 Oxidation (M)	(Ions score 55)
420 - 435	638.2975	1911.8706	1911.8641	3	0	K.VEFVHMLNATMCATTR.T	2 Oxidation (M)	(Ions score 59)
420 - 435	638.2975	1911.8706	1911.8641	3	0	K.VEFVHMLNATMCATTR.T	2 Oxidation (M)	(Ions score 57)
420 - 435	638.2975	1911.8707	1911.8641	3	0	K.VEFVHMLNATMCATTR.T	2 Oxidation (M)	(Ions score 24)
420 - 435	638.2976	1911.8709	1911.8641	4	0	K.VEFVHMLNATMCATTR.T	2 Oxidation (M)	(Ions score 25)
420 - 435	638.2976	1911.8709	1911.8641	4	0	K.VEFVHMLNATMCATTR.T	2 Oxidation (M)	(Ions score 61)
420 - 435	956.9427	1911.8709	1911.8641	4	0	K.VEFVHMLNATMCATTR.T	2 Oxidation (M)	(Ions score 44)
420 - 435	956.9428	1911.8710	1911.8641	4	0	K.VEFVHMLNATMCATTR.T	2 Oxidation (M)	(Ions score 37)
420 - 435	638.2976	1911.8711	1911.8641	4	0	K.VEFVHMLNATMCATTR.T	2 Oxidation (M)	(Ions score 22)
420 - 435	638.2977	1911.8712	1911.8641	4	0	K.VEFVHMLNATMCATTR.T	2 Oxidation (M)	(Ions score 39)
420 - 435	638.2977	1911.8713	1911.8641	4	0	K.VEFVHMLNATMCATTR.T	2 Oxidation (M)	(Ions score 27)
420 - 435	638.2977	1911.8713	1911.8641	4	0	K.VEFVHMLNATMCATTR.T	2 Oxidation (M)	(Ions score 59)
420 - 435	638.2977	1911.8714	1911.8641	4	0	K.VEFVHMLNATMCATTR.T	2 Oxidation (M)	(Ions score 65)
420 - 435	638.2977	1911.8714	1911.8641	4	0	K.VEFVHMLNATMCATTR.T	2 Oxidation (M)	(Ions score 58)
420 - 435	956.9430	1911.8714	1911.8641	4	0	K.VEFVHMLNATMCATTR.T	2 Oxidation (M)	(Ions score 29)
420 - 435	638.2978	1911.8715	1911.8641	4	0	K.VEFVHMLNATMCATTR.T	2 Oxidation (M)	(Ions score 59)
420 - 435	638.2978	1911.8715	1911.8641	4	0	K.VEFVHMLNATMCATTR.T	2 Oxidation (M)	(Ions score 21)
420 - 435	638.2978	1911.8715	1911.8641	4	0	K.VEFVHMLNATMCATTR.T	2 Oxidation (M)	(Ions score 55)
420 - 435	638.2978	1911.8716	1911.8641	4	0	K.VEFVHMLNATMCATTR.T	2 Oxidation (M)	(Ions score 37)
420 - 435	956.9431	1911.8717	1911.8641	4	0	K.VEFVHMLNATMCATTR.T	2 Oxidation (M)	(Ions score 32)
420 - 435	638.2979	1911.8717	1911.8641	4	0	K.VEFVHMLNATMCATTR.T	2 Oxidation (M)	(Ions score 35)
420 - 435	638.2979	1911.8718	1911.8641	4	0	K.VEFVHMLNATMCATTR.T	2 Oxidation (M)	(Ions score 41)
420 - 435	638.2979	1911.8718	1911.8641	4	0	K.VEFVHMLNATMCATTR.T	2 Oxidation (M)	(Ions score 31)
420 - 435	638.2979	1911.8719	1911.8641	4	0	K.VEFVHMLNATMCATTR.T	2 Oxidation (M)	(Ions score 22)
420 - 435	638.2979	1911.8720	1911.8641	4	0	K.VEFVHMLNATMCATTR.T	2 Oxidation (M)	(Ions score 29)
420 - 435	638.2980	1911.8720	1911.8641	4	0	K.VEFVHMLNATMCATTR.T	2 Oxidation (M)	(Ions score 37)
420 - 435	638.2980	1911.8721	1911.8641	4	0	K.VEFVHMLNATMCATTR.T	2 Oxidation (M)	(Ions score 23)
420 - 435	638.2980	1911.8721	1911.8641	4	0	K.VEFVHMLNATMCATTR.T	2 Oxidation (M)	(Ions score 45)
420 - 435	956.9434	1911.8722	1911.8641	4	0	K.VEFVHMLNATMCATTR.T	2 Oxidation (M)	(Ions score 29)
420 - 435	638.2980	1911.8722	1911.8641	4	0	K.VEFVHMLNATMCATTR.T	2 Oxidation (M)	(Ions score 29)
420 - 435	956.9435	1911.8724	1911.8641	4	0	K.VEFVHMLNATMCATTR.T	2 Oxidation (M)	(Ions score 28)
420 - 435	638.2981	1911.8726	1911.8641	4	0	K.VEFVHMLNATMCATTR.T	2 Oxidation (M)	(Ions score 59)
420 - 435	638.2981	1911.8726	1911.8641	4	0	K.VEFVHMLNATMCATTR.T	2 Oxidation (M)	(Ions score 35)
420 - 435	638.2982	1911.8727	1911.8641	4	0	K.VEFVHMLNATMCATTR.T	2 Oxidation (M)	(Ions score 22)
420 - 435	638.2982	1911.8728	1911.8641	5	0	K.VEFVHMLNATMCATTR.T	2 Oxidation (M)	(Ions score 31)
420 - 435	638.2982	1911.8728	1911.8641	5	0	K.VEFVHMLNATMCATTR.T	2 Oxidation (M)	(Ions score 22)
420 - 435	638.2983	1911.8730	1911.8641	5	0	K.VEFVHMLNATMCATTR.T	2 Oxidation (M)	(Ions score 48)
420 - 435	638.2983	1911.8731	1911.8641	5	0	K.VEFVHMLNATMCATTR.T	2 Oxidation (M)	(Ions score 27)
420 - 435	638.2984	1911.8732	1911.8641	5	0	K.VEFVHMLNATMCATTR.T	2 Oxidation (M)	(Ions score 31)
420 - 435	638.2984	1911.8733	1911.8641	5	0	K.VEFVHMLNATMCATTR.T	2 Oxidation (M)	(Ions score 28)
420 - 435	638.2984	1911.8734	1911.8641	5	0	K.VEFVHMLNATMCATTR.T	2 Oxidation (M)	(Ions score 26)
420 - 435	638.2984	1911.8735	1911.8641	5	0	K.VEFVHMLNATMCATTR.T	2 Oxidation (M)	(Ions score 36)
420 - 435	638.2985	1911.8735	1911.8641	5	0	K.VEFVHMLNATMCATTR.T	2 Oxidation (M)	(Ions score 26)
420 - 435	638.2985	1911.8736	1911.8641	5	0	K.VEFVHMLNATMCATTR.T	2 Oxidation (M)	(Ions score 64)
420 - 435	638.2985	1911.8737	1911.8641	5	0	K.VEFVHMLNATMCATTR.T	2 Oxidation (M)	(Ions score 54)
436 - 448	791.8893	1581.7641	1581.7708	-4	0	R.TICAILENYQTEK.G		(Ions score 27)
436 - 448	791.8895	1581.7643	1581.7708	-4	0	R.TICAILENYQTEK.G		(Ions score 50)
436 - 448	791.8904	1581.7662	1581.7708	-3	0	R.TICAILENYQTEK.G		(Ions score 39)
436 - 448	791.8905	1581.7665	1581.7708	-3	0	R.TICAILENYQTEK.G		(Ions score 39)
436 - 448	791.8907	1581.7668	1581.7708	-3	0	R.TICAILENYQTEK.G		(Ions score 52)
436 - 448	791.8908	1581.7671	1581.7708	-2	0	R.TICAILENYQTEK.G		(Ions score 37)
436 - 448	791.8908	1581.7671	1581.7708	-2	0	R.TICAILENYQTEK.G		(Ions score 45)

436 - 448	791.8909	1581.7672	1581.7708	-2	0	R.TICAILENYQTEK.G	(Ions score 47)
436 - 448	791.8911	1581.7677	1581.7708	-2	0	R.TICAILENYQTEK.G	(Ions score 46)
436 - 448	791.8912	1581.7678	1581.7708	-2	0	R.TICAILENYQTEK.G	(Ions score 32)
436 - 448	791.8913	1581.7680	1581.7708	-2	0	R.TICAILENYQTEK.G	(Ions score 36)
436 - 448	791.8914	1581.7683	1581.7708	-2	0	R.TICAILENYQTEK.G	(Ions score 43)
436 - 448	791.8914	1581.7683	1581.7708	-2	0	R.TICAILENYQTEK.G	(Ions score 44)
436 - 448	791.8915	1581.7684	1581.7708	-2	0	R.TICAILENYQTEK.G	(Ions score 30)
436 - 448	528.2635	1581.7687	1581.7708	-1	0	R.TICAILENYQTEK.G	(Ions score 27)
436 - 448	791.8917	1581.7688	1581.7708	-1	0	R.TICAILENYQTEK.G	(Ions score 49)
436 - 448	791.8918	1581.7690	1581.7708	-1	0	R.TICAILENYQTEK.G	(Ions score 43)
436 - 448	791.8918	1581.7691	1581.7708	-1	0	R.TICAILENYQTEK.G	(Ions score 39)
436 - 448	791.8918	1581.7691	1581.7708	-1	0	R.TICAILENYQTEK.G	(Ions score 45)
436 - 448	791.8919	1581.7693	1581.7708	-1	0	R.TICAILENYQTEK.G	(Ions score 35)
436 - 448	791.8919	1581.7693	1581.7708	-1	0	R.TICAILENYQTEK.G	(Ions score 46)
436 - 448	791.8920	1581.7694	1581.7708	-1	0	R.TICAILENYQTEK.G	(Ions score 37)
436 - 448	791.8920	1581.7694	1581.7708	-1	0	R.TICAILENYQTEK.G	(Ions score 46)
436 - 448	791.8920	1581.7694	1581.7708	-1	0	R.TICAILENYQTEK.G	(Ions score 41)
436 - 448	791.8920	1581.7694	1581.7708	-1	0	R.TICAILENYQTEK.G	(Ions score 31)
436 - 448	791.8920	1581.7694	1581.7708	-1	0	R.TICAILENYQTEK.G	(Ions score 28)
436 - 448	791.8920	1581.7694	1581.7708	-1	0	R.TICAILENYQTEK.G	(Ions score 45)
436 - 448	791.8920	1581.7695	1581.7708	-1	0	R.TICAILENYQTEK.G	(Ions score 34)
436 - 448	791.8920	1581.7695	1581.7708	-1	0	R.TICAILENYQTEK.G	(Ions score 28)
436 - 448	791.8920	1581.7695	1581.7708	-1	0	R.TICAILENYQTEK.G	(Ions score 27)
436 - 448	791.8920	1581.7695	1581.7708	-1	0	R.TICAILENYQTEK.G	(Ions score 44)
436 - 448	791.8920	1581.7695	1581.7708	-1	0	R.TICAILENYQTEK.G	(Ions score 35)
436 - 448	528.2638	1581.7696	1581.7708	-1	0	R.TICAILENYQTEK.G	(Ions score 23)
436 - 448	791.8922	1581.7697	1581.7708	-1	0	R.TICAILENYQTEK.G	(Ions score 39)
436 - 448	791.8922	1581.7699	1581.7708	-1	0	R.TICAILENYQTEK.G	(Ions score 41)
436 - 448	791.8922	1581.7699	1581.7708	-1	0	R.TICAILENYQTEK.G	(Ions score 44)
436 - 448	791.8923	1581.7700	1581.7708	-1	0	R.TICAILENYQTEK.G	(Ions score 42)
436 - 448	791.8924	1581.7702	1581.7708	-0	0	R.TICAILENYQTEK.G	(Ions score 26)
436 - 448	791.8925	1581.7704	1581.7708	-0	0	R.TICAILENYQTEK.G	(Ions score 45)
436 - 448	791.8925	1581.7705	1581.7708	-0	0	R.TICAILENYQTEK.G	(Ions score 51)
436 - 448	528.2642	1581.7706	1581.7708	-0	0	R.TICAILENYQTEK.G	(Ions score 28)
436 - 448	791.8927	1581.7708	1581.7708	-0	0	R.TICAILENYQTEK.G	(Ions score 51)
436 - 448	791.8927	1581.7708	1581.7708	-0	0	R.TICAILENYQTEK.G	(Ions score 50)
436 - 448	791.8927	1581.7708	1581.7708	-0	0	R.TICAILENYQTEK.G	(Ions score 42)
436 - 448	791.8927	1581.7709	1581.7708	0	0	R.TICAILENYQTEK.G	(Ions score 49)
436 - 448	528.2642	1581.7709	1581.7708	0	0	R.TICAILENYQTEK.G	(Ions score 24)
436 - 448	791.8928	1581.7710	1581.7708	0	0	R.TICAILENYQTEK.G	(Ions score 45)
436 - 448	791.8928	1581.7710	1581.7708	0	0	R.TICAILENYQTEK.G	(Ions score 40)
436 - 448	791.8928	1581.7711	1581.7708	0	0	R.TICAILENYQTEK.G	(Ions score 29)
436 - 448	791.8929	1581.7713	1581.7708	0	0	R.TICAILENYQTEK.G	(Ions score 42)
436 - 448	528.2644	1581.7715	1581.7708	0	0	R.TICAILENYQTEK.G	(Ions score 23)
436 - 448	791.8930	1581.7715	1581.7708	0	0	R.TICAILENYQTEK.G	(Ions score 37)
436 - 448	791.8931	1581.7715	1581.7708	0	0	R.TICAILENYQTEK.G	(Ions score 46)
436 - 448	791.8931	1581.7716	1581.7708	0	0	R.TICAILENYQTEK.G	(Ions score 57)
436 - 448	791.8931	1581.7716	1581.7708	1	0	R.TICAILENYQTEK.G	(Ions score 71)
436 - 448	791.8931	1581.7717	1581.7708	1	0	R.TICAILENYQTEK.G	(Ions score 31)
436 - 448	791.8931	1581.7717	1581.7708	1	0	R.TICAILENYQTEK.G	(Ions score 47)
436 - 448	791.8931	1581.7717	1581.7708	1	0	R.TICAILENYQTEK.G	(Ions score 47)
436 - 448	528.2645	1581.7718	1581.7708	1	0	R.TICAILENYQTEK.G	(Ions score 25)
436 - 448	791.8932	1581.7718	1581.7708	1	0	R.TICAILENYQTEK.G	(Ions score 51)
436 - 448	791.8932	1581.7718	1581.7708	1	0	R.TICAILENYQTEK.G	(Ions score 48)
436 - 448	791.8932	1581.7718	1581.7708	1	0	R.TICAILENYQTEK.G	(Ions score 42)
436 - 448	791.8932	1581.7718	1581.7708	1	0	R.TICAILENYQTEK.G	(Ions score 44)
436 - 448	791.8932	1581.7718	1581.7708	1	0	R.TICAILENYQTEK.G	(Ions score 43)
436 - 448	791.8932	1581.7719	1581.7708	1	0	R.TICAILENYQTEK.G	(Ions score 54)

436 - 448	791.8933	1581.7720	1581.7708	1	0	R.TICAILENYQTEK.G	(Ions score 52)
436 - 448	791.8933	1581.7721	1581.7708	1	0	R.TICAILENYQTEK.G	(Ions score 43)
436 - 448	791.8933	1581.7721	1581.7708	1	0	R.TICAILENYQTEK.G	(Ions score 42)
436 - 448	791.8933	1581.7721	1581.7708	1	0	R.TICAILENYQTEK.G	(Ions score 60)
436 - 448	791.8934	1581.7722	1581.7708	1	0	R.TICAILENYQTEK.G	(Ions score 66)
436 - 448	791.8934	1581.7722	1581.7708	1	0	R.TICAILENYQTEK.G	(Ions score 47)
436 - 448	791.8934	1581.7722	1581.7708	1	0	R.TICAILENYQTEK.G	(Ions score 64)
436 - 448	791.8934	1581.7722	1581.7708	1	0	R.TICAILENYQTEK.G	(Ions score 39)
436 - 448	528.2647	1581.7723	1581.7708	1	0	R.TICAILENYQTEK.G	(Ions score 24)
436 - 448	791.8934	1581.7723	1581.7708	1	0	R.TICAILENYQTEK.G	(Ions score 52)
436 - 448	791.8934	1581.7723	1581.7708	1	0	R.TICAILENYQTEK.G	(Ions score 35)
436 - 448	528.2647	1581.7723	1581.7708	1	0	R.TICAILENYQTEK.G	(Ions score 26)
436 - 448	791.8934	1581.7723	1581.7708	1	0	R.TICAILENYQTEK.G	(Ions score 46)
436 - 448	791.8935	1581.7724	1581.7708	1	0	R.TICAILENYQTEK.G	(Ions score 41)
436 - 448	528.2648	1581.7725	1581.7708	1	0	R.TICAILENYQTEK.G	(Ions score 43)
436 - 448	791.8935	1581.7725	1581.7708	1	0	R.TICAILENYQTEK.G	(Ions score 45)
436 - 448	791.8936	1581.7726	1581.7708	1	0	R.TICAILENYQTEK.G	(Ions score 43)
436 - 448	528.2648	1581.7726	1581.7708	1	0	R.TICAILENYQTEK.G	(Ions score 48)
436 - 448	791.8936	1581.7726	1581.7708	1	0	R.TICAILENYQTEK.G	(Ions score 44)
436 - 448	528.2648	1581.7726	1581.7708	1	0	R.TICAILENYQTEK.G	(Ions score 39)
436 - 448	791.8936	1581.7727	1581.7708	1	0	R.TICAILENYQTEK.G	(Ions score 45)
436 - 448	791.8936	1581.7727	1581.7708	1	0	R.TICAILENYQTEK.G	(Ions score 45)
436 - 448	791.8936	1581.7727	1581.7708	1	0	R.TICAILENYQTEK.G	(Ions score 44)
436 - 448	791.8936	1581.7727	1581.7708	1	0	R.TICAILENYQTEK.G	(Ions score 56)
436 - 448	791.8937	1581.7728	1581.7708	1	0	R.TICAILENYQTEK.G	(Ions score 47)
436 - 448	791.8937	1581.7728	1581.7708	1	0	R.TICAILENYQTEK.G	(Ions score 29)
436 - 448	791.8937	1581.7728	1581.7708	1	0	R.TICAILENYQTEK.G	(Ions score 42)
436 - 448	528.2649	1581.7729	1581.7708	1	0	R.TICAILENYQTEK.G	(Ions score 45)
436 - 448	791.8937	1581.7729	1581.7708	1	0	R.TICAILENYQTEK.G	(Ions score 58)
436 - 448	528.2649	1581.7730	1581.7708	1	0	R.TICAILENYQTEK.G	(Ions score 37)
436 - 448	791.8938	1581.7730	1581.7708	1	0	R.TICAILENYQTEK.G	(Ions score 41)
436 - 448	791.8938	1581.7731	1581.7708	1	0	R.TICAILENYQTEK.G	(Ions score 46)
436 - 448	791.8939	1581.7732	1581.7708	1	0	R.TICAILENYQTEK.G	(Ions score 50)
436 - 448	791.8939	1581.7732	1581.7708	1	0	R.TICAILENYQTEK.G	(Ions score 45)
436 - 448	791.8939	1581.7732	1581.7708	2	0	R.TICAILENYQTEK.G	(Ions score 48)
436 - 448	528.2650	1581.7732	1581.7708	2	0	R.TICAILENYQTEK.G	(Ions score 23)
436 - 448	791.8939	1581.7733	1581.7708	2	0	R.TICAILENYQTEK.G	(Ions score 50)
436 - 448	791.8939	1581.7733	1581.7708	2	0	R.TICAILENYQTEK.G	(Ions score 42)
436 - 448	528.2651	1581.7734	1581.7708	2	0	R.TICAILENYQTEK.G	(Ions score 28)
436 - 448	791.8940	1581.7734	1581.7708	2	0	R.TICAILENYQTEK.G	(Ions score 51)
436 - 448	528.2651	1581.7734	1581.7708	2	0	R.TICAILENYQTEK.G	(Ions score 44)
436 - 448	791.8940	1581.7734	1581.7708	2	0	R.TICAILENYQTEK.G	(Ions score 34)
436 - 448	791.8940	1581.7735	1581.7708	2	0	R.TICAILENYQTEK.G	(Ions score 44)
436 - 448	791.8940	1581.7735	1581.7708	2	0	R.TICAILENYQTEK.G	(Ions score 35)
436 - 448	791.8941	1581.7736	1581.7708	2	0	R.TICAILENYQTEK.G	(Ions score 39)
436 - 448	791.8941	1581.7736	1581.7708	2	0	R.TICAILENYQTEK.G	(Ions score 37)
436 - 448	791.8941	1581.7737	1581.7708	2	0	R.TICAILENYQTEK.G	(Ions score 28)
436 - 448	791.8941	1581.7737	1581.7708	2	0	R.TICAILENYQTEK.G	(Ions score 41)
436 - 448	791.8941	1581.7737	1581.7708	2	0	R.TICAILENYQTEK.G	(Ions score 42)
436 - 448	528.2652	1581.7737	1581.7708	2	0	R.TICAILENYQTEK.G	(Ions score 41)
436 - 448	791.8942	1581.7737	1581.7708	2	0	R.TICAILENYQTEK.G	(Ions score 64)
436 - 448	528.2652	1581.7738	1581.7708	2	0	R.TICAILENYQTEK.G	(Ions score 42)
436 - 448	791.8942	1581.7738	1581.7708	2	0	R.TICAILENYQTEK.G	(Ions score 48)
436 - 448	791.8942	1581.7738	1581.7708	2	0	R.TICAILENYQTEK.G	(Ions score 29)
436 - 448	791.8942	1581.7738	1581.7708	2	0	R.TICAILENYQTEK.G	(Ions score 46)
436 - 448	791.8942	1581.7738	1581.7708	2	0	R.TICAILENYQTEK.G	(Ions score 45)
436 - 448	791.8942	1581.7739	1581.7708	2	0	R.TICAILENYQTEK.G	(Ions score 55)
436 - 448	791.8942	1581.7739	1581.7708	2	0	R.TICAILENYQTEK.G	(Ions score 49)

436 - 448	791.8943	1581.7740	1581.7708	2	0	R.TICAILENYQTEK.G	(Ions score 46)
436 - 448	791.8943	1581.7740	1581.7708	2	0	R.TICAILENYQTEK.G	(Ions score 71)
436 - 448	791.8943	1581.7740	1581.7708	2	0	R.TICAILENYQTEK.G	(Ions score 60)
436 - 448	791.8943	1581.7740	1581.7708	2	0	R.TICAILENYQTEK.G	(Ions score 44)
436 - 448	791.8943	1581.7740	1581.7708	2	0	R.TICAILENYQTEK.G	(Ions score 44)
436 - 448	791.8943	1581.7741	1581.7708	2	0	R.TICAILENYQTEK.G	(Ions score 56)
436 - 448	791.8943	1581.7741	1581.7708	2	0	R.TICAILENYQTEK.G	(Ions score 36)
436 - 448	791.8944	1581.7742	1581.7708	2	0	R.TICAILENYQTEK.G	(Ions score 36)
436 - 448	791.8944	1581.7742	1581.7708	2	0	R.TICAILENYQTEK.G	(Ions score 51)
436 - 448	791.8944	1581.7742	1581.7708	2	0	R.TICAILENYQTEK.G	(Ions score 44)
436 - 448	528.2654	1581.7742	1581.7708	2	0	R.TICAILENYQTEK.G	(Ions score 41)
436 - 448	528.2654	1581.7743	1581.7708	2	0	R.TICAILENYQTEK.G	(Ions score 41)
436 - 448	528.2654	1581.7743	1581.7708	2	0	R.TICAILENYQTEK.G	(Ions score 41)
436 - 448	791.8944	1581.7743	1581.7708	2	0	R.TICAILENYQTEK.G	(Ions score 50)
436 - 448	791.8945	1581.7744	1581.7708	2	0	R.TICAILENYQTEK.G	(Ions score 27)
436 - 448	791.8945	1581.7744	1581.7708	2	0	R.TICAILENYQTEK.G	(Ions score 51)
436 - 448	791.8945	1581.7744	1581.7708	2	0	R.TICAILENYQTEK.G	(Ions score 38)
436 - 448	791.8945	1581.7744	1581.7708	2	0	R.TICAILENYQTEK.G	(Ions score 41)
436 - 448	791.8945	1581.7744	1581.7708	2	0	R.TICAILENYQTEK.G	(Ions score 42)
436 - 448	791.8945	1581.7745	1581.7708	2	0	R.TICAILENYQTEK.G	(Ions score 60)
436 - 448	528.2654	1581.7745	1581.7708	2	0	R.TICAILENYQTEK.G	(Ions score 25)
436 - 448	791.8945	1581.7745	1581.7708	2	0	R.TICAILENYQTEK.G	(Ions score 38)
436 - 448	791.8945	1581.7745	1581.7708	2	0	R.TICAILENYQTEK.G	(Ions score 65)
436 - 448	528.2655	1581.7745	1581.7708	2	0	R.TICAILENYQTEK.G	(Ions score 27)
436 - 448	528.2655	1581.7746	1581.7708	2	0	R.TICAILENYQTEK.G	(Ions score 43)
436 - 448	528.2655	1581.7746	1581.7708	2	0	R.TICAILENYQTEK.G	(Ions score 33)
436 - 448	791.8946	1581.7746	1581.7708	2	0	R.TICAILENYQTEK.G	(Ions score 48)
436 - 448	528.2655	1581.7746	1581.7708	2	0	R.TICAILENYQTEK.G	(Ions score 49)
436 - 448	528.2655	1581.7746	1581.7708	2	0	R.TICAILENYQTEK.G	(Ions score 44)
436 - 448	528.2655	1581.7746	1581.7708	2	0	R.TICAILENYQTEK.G	(Ions score 46)
436 - 448	791.8946	1581.7747	1581.7708	2	0	R.TICAILENYQTEK.G	(Ions score 41)
436 - 448	528.2655	1581.7747	1581.7708	2	0	R.TICAILENYQTEK.G	(Ions score 44)
436 - 448	528.2655	1581.7747	1581.7708	2	0	R.TICAILENYQTEK.G	(Ions score 39)
436 - 448	528.2655	1581.7747	1581.7708	2	0	R.TICAILENYQTEK.G	(Ions score 45)
436 - 448	528.2655	1581.7747	1581.7708	2	0	R.TICAILENYQTEK.G	(Ions score 45)
436 - 448	791.8947	1581.7748	1581.7708	2	0	R.TICAILENYQTEK.G	(Ions score 46)
436 - 448	791.8947	1581.7748	1581.7708	2	0	R.TICAILENYQTEK.G	(Ions score 26)
436 - 448	791.8947	1581.7748	1581.7708	2	0	R.TICAILENYQTEK.G	(Ions score 44)
436 - 448	791.8947	1581.7748	1581.7708	3	0	R.TICAILENYQTEK.G	(Ions score 48)
436 - 448	791.8947	1581.7748	1581.7708	3	0	R.TICAILENYQTEK.G	(Ions score 44)
436 - 448	791.8947	1581.7748	1581.7708	3	0	R.TICAILENYQTEK.G	(Ions score 46)
436 - 448	791.8947	1581.7748	1581.7708	3	0	R.TICAILENYQTEK.G	(Ions score 53)
436 - 448	791.8947	1581.7749	1581.7708	3	0	R.TICAILENYQTEK.G	(Ions score 42)
436 - 448	791.8947	1581.7749	1581.7708	3	0	R.TICAILENYQTEK.G	(Ions score 55)
436 - 448	791.8947	1581.7749	1581.7708	3	0	R.TICAILENYQTEK.G	(Ions score 56)
436 - 448	791.8947	1581.7749	1581.7708	3	0	R.TICAILENYQTEK.G	(Ions score 45)
436 - 448	791.8947	1581.7749	1581.7708	3	0	R.TICAILENYQTEK.G	(Ions score 26)
436 - 448	528.2656	1581.7749	1581.7708	3	0	R.TICAILENYQTEK.G	(Ions score 28)
436 - 448	528.2656	1581.7749	1581.7708	3	0	R.TICAILENYQTEK.G	(Ions score 43)
436 - 448	528.2656	1581.7749	1581.7708	3	0	R.TICAILENYQTEK.G	(Ions score 25)
436 - 448	528.2656	1581.7750	1581.7708	3	0	R.TICAILENYQTEK.G	(Ions score 44)
436 - 448	791.8948	1581.7750	1581.7708	3	0	R.TICAILENYQTEK.G	(Ions score 61)
436 - 448	528.2656	1581.7750	1581.7708	3	0	R.TICAILENYQTEK.G	(Ions score 28)
436 - 448	791.8948	1581.7750	1581.7708	3	0	R.TICAILENYQTEK.G	(Ions score 47)
436 - 448	791.8948	1581.7750	1581.7708	3	0	R.TICAILENYQTEK.G	(Ions score 47)
436 - 448	528.2656	1581.7751	1581.7708	3	0	R.TICAILENYQTEK.G	(Ions score 50)
436 - 448	528.2656	1581.7751	1581.7708	3	0	R.TICAILENYQTEK.G	(Ions score 49)
436 - 448	791.8948	1581.7751	1581.7708	3	0	R.TICAILENYQTEK.G	(Ions score 27)

436 - 448	791.8948	1581.7751	1581.7708	3	0	R.TICAILENYQTEK.G	(Ions score 44)
436 - 448	791.8948	1581.7751	1581.7708	3	0	R.TICAILENYQTEK.G	(Ions score 62)
436 - 448	791.8949	1581.7752	1581.7708	3	0	R.TICAILENYQTEK.G	(Ions score 46)
436 - 448	791.8949	1581.7752	1581.7708	3	0	R.TICAILENYQTEK.G	(Ions score 48)
436 - 448	528.2657	1581.7752	1581.7708	3	0	R.TICAILENYQTEK.G	(Ions score 33)
436 - 448	528.2657	1581.7752	1581.7708	3	0	R.TICAILENYQTEK.G	(Ions score 32)
436 - 448	528.2657	1581.7752	1581.7708	3	0	R.TICAILENYQTEK.G	(Ions score 25)
436 - 448	791.8949	1581.7753	1581.7708	3	0	R.TICAILENYQTEK.G	(Ions score 68)
436 - 448	528.2657	1581.7753	1581.7708	3	0	R.TICAILENYQTEK.G	(Ions score 48)
436 - 448	791.8950	1581.7754	1581.7708	3	0	R.TICAILENYQTEK.G	(Ions score 45)
436 - 448	791.8950	1581.7754	1581.7708	3	0	R.TICAILENYQTEK.G	(Ions score 43)
436 - 448	791.8950	1581.7754	1581.7708	3	0	R.TICAILENYQTEK.G	(Ions score 47)
436 - 448	791.8950	1581.7755	1581.7708	3	0	R.TICAILENYQTEK.G	(Ions score 49)
436 - 448	528.2658	1581.7755	1581.7708	3	0	R.TICAILENYQTEK.G	(Ions score 37)
436 - 448	791.8950	1581.7755	1581.7708	3	0	R.TICAILENYQTEK.G	(Ions score 47)
436 - 448	791.8950	1581.7755	1581.7708	3	0	R.TICAILENYQTEK.G	(Ions score 43)
436 - 448	528.2658	1581.7756	1581.7708	3	0	R.TICAILENYQTEK.G	(Ions score 43)
436 - 448	528.2658	1581.7756	1581.7708	3	0	R.TICAILENYQTEK.G	(Ions score 51)
436 - 448	791.8951	1581.7756	1581.7708	3	0	R.TICAILENYQTEK.G	(Ions score 40)
436 - 448	528.2658	1581.7756	1581.7708	3	0	R.TICAILENYQTEK.G	(Ions score 26)
436 - 448	528.2658	1581.7756	1581.7708	3	0	R.TICAILENYQTEK.G	(Ions score 44)
436 - 448	791.8951	1581.7757	1581.7708	3	0	R.TICAILENYQTEK.G	(Ions score 41)
436 - 448	791.8951	1581.7757	1581.7708	3	0	R.TICAILENYQTEK.G	(Ions score 68)
436 - 448	791.8951	1581.7757	1581.7708	3	0	R.TICAILENYQTEK.G	(Ions score 50)
436 - 448	791.8951	1581.7757	1581.7708	3	0	R.TICAILENYQTEK.G	(Ions score 48)
436 - 448	791.8952	1581.7758	1581.7708	3	0	R.TICAILENYQTEK.G	(Ions score 45)
436 - 448	791.8952	1581.7758	1581.7708	3	0	R.TICAILENYQTEK.G	(Ions score 52)
436 - 448	528.2659	1581.7758	1581.7708	3	0	R.TICAILENYQTEK.G	(Ions score 28)
436 - 448	791.8952	1581.7758	1581.7708	3	0	R.TICAILENYQTEK.G	(Ions score 46)
436 - 448	791.8952	1581.7759	1581.7708	3	0	R.TICAILENYQTEK.G	(Ions score 38)
436 - 448	791.8953	1581.7760	1581.7708	3	0	R.TICAILENYQTEK.G	(Ions score 22)
436 - 448	791.8953	1581.7760	1581.7708	3	0	R.TICAILENYQTEK.G	(Ions score 45)
436 - 448	791.8953	1581.7760	1581.7708	3	0	R.TICAILENYQTEK.G	(Ions score 43)
436 - 448	791.8953	1581.7760	1581.7708	3	0	R.TICAILENYQTEK.G	(Ions score 59)
436 - 448	791.8953	1581.7760	1581.7708	3	0	R.TICAILENYQTEK.G	(Ions score 50)
436 - 448	791.8953	1581.7760	1581.7708	3	0	R.TICAILENYQTEK.G	(Ions score 52)
436 - 448	791.8953	1581.7760	1581.7708	3	0	R.TICAILENYQTEK.G	(Ions score 48)
436 - 448	791.8953	1581.7760	1581.7708	3	0	R.TICAILENYQTEK.G	(Ions score 44)
436 - 448	528.2660	1581.7760	1581.7708	3	0	R.TICAILENYQTEK.G	(Ions score 44)
436 - 448	791.8953	1581.7761	1581.7708	3	0	R.TICAILENYQTEK.G	(Ions score 48)
436 - 448	791.8953	1581.7761	1581.7708	3	0	R.TICAILENYQTEK.G	(Ions score 61)
436 - 448	791.8954	1581.7762	1581.7708	3	0	R.TICAILENYQTEK.G	(Ions score 51)
436 - 448	528.2660	1581.7762	1581.7708	3	0	R.TICAILENYQTEK.G	(Ions score 46)
436 - 448	528.2660	1581.7762	1581.7708	3	0	R.TICAILENYQTEK.G	(Ions score 43)
436 - 448	528.2660	1581.7762	1581.7708	3	0	R.TICAILENYQTEK.G	(Ions score 46)
436 - 448	791.8954	1581.7762	1581.7708	3	0	R.TICAILENYQTEK.G	(Ions score 27)
436 - 448	791.8954	1581.7763	1581.7708	3	0	R.TICAILENYQTEK.G	(Ions score 48)
436 - 448	791.8954	1581.7763	1581.7708	3	0	R.TICAILENYQTEK.G	(Ions score 68)
436 - 448	791.8954	1581.7763	1581.7708	3	0	R.TICAILENYQTEK.G	(Ions score 45)
436 - 448	791.8954	1581.7763	1581.7708	3	0	R.TICAILENYQTEK.G	(Ions score 53)
436 - 448	528.2661	1581.7763	1581.7708	3	0	R.TICAILENYQTEK.G	(Ions score 41)
436 - 448	791.8955	1581.7764	1581.7708	3	0	R.TICAILENYQTEK.G	(Ions score 53)
436 - 448	528.2661	1581.7764	1581.7708	3	0	R.TICAILENYQTEK.G	(Ions score 40)
436 - 448	791.8955	1581.7764	1581.7708	4	0	R.TICAILENYQTEK.G	(Ions score 46)
436 - 448	791.8955	1581.7765	1581.7708	4	0	R.TICAILENYQTEK.G	(Ions score 43)
436 - 448	791.8955	1581.7765	1581.7708	4	0	R.TICAILENYQTEK.G	(Ions score 70)
436 - 448	791.8955	1581.7765	1581.7708	4	0	R.TICAILENYQTEK.G	(Ions score 53)
436 - 448	791.8955	1581.7765	1581.7708	4	0	R.TICAILENYQTEK.G	(Ions score 59)

436 - 448	791.8955	1581.7765	1581.7708	4	0	R.TICAILENYQTEK.G	(Ions score 45)
436 - 448	791.8955	1581.7765	1581.7708	4	0	R.TICAILENYQTEK.G	(Ions score 45)
436 - 448	528.2661	1581.7766	1581.7708	4	0	R.TICAILENYQTEK.G	(Ions score 28)
436 - 448	791.8956	1581.7766	1581.7708	4	0	R.TICAILENYQTEK.G	(Ions score 50)
436 - 448	791.8956	1581.7766	1581.7708	4	0	R.TICAILENYQTEK.G	(Ions score 24)
436 - 448	791.8956	1581.7766	1581.7708	4	0	R.TICAILENYQTEK.G	(Ions score 42)
436 - 448	791.8956	1581.7766	1581.7708	4	0	R.TICAILENYQTEK.G	(Ions score 43)
436 - 448	528.2661	1581.7766	1581.7708	4	0	R.TICAILENYQTEK.G	(Ions score 25)
436 - 448	791.8956	1581.7766	1581.7708	4	0	R.TICAILENYQTEK.G	(Ions score 39)
436 - 448	791.8956	1581.7767	1581.7708	4	0	R.TICAILENYQTEK.G	(Ions score 57)
436 - 448	528.2662	1581.7767	1581.7708	4	0	R.TICAILENYQTEK.G	(Ions score 28)
436 - 448	791.8956	1581.7767	1581.7708	4	0	R.TICAILENYQTEK.G	(Ions score 68)
436 - 448	791.8956	1581.7767	1581.7708	4	0	R.TICAILENYQTEK.G	(Ions score 45)
436 - 448	528.2662	1581.7768	1581.7708	4	0	R.TICAILENYQTEK.G	(Ions score 39)
436 - 448	791.8957	1581.7768	1581.7708	4	0	R.TICAILENYQTEK.G	(Ions score 39)
436 - 448	791.8957	1581.7768	1581.7708	4	0	R.TICAILENYQTEK.G	(Ions score 49)
436 - 448	791.8957	1581.7769	1581.7708	4	0	R.TICAILENYQTEK.G	(Ions score 25)
436 - 448	791.8957	1581.7769	1581.7708	4	0	R.TICAILENYQTEK.G	(Ions score 60)
436 - 448	791.8957	1581.7769	1581.7708	4	0	R.TICAILENYQTEK.G	(Ions score 48)
436 - 448	528.2662	1581.7769	1581.7708	4	0	R.TICAILENYQTEK.G	(Ions score 25)
436 - 448	791.8957	1581.7769	1581.7708	4	0	R.TICAILENYQTEK.G	(Ions score 41)
436 - 448	791.8957	1581.7769	1581.7708	4	0	R.TICAILENYQTEK.G	(Ions score 48)
436 - 448	791.8957	1581.7769	1581.7708	4	0	R.TICAILENYQTEK.G	(Ions score 46)
436 - 448	791.8958	1581.7770	1581.7708	4	0	R.TICAILENYQTEK.G	(Ions score 45)
436 - 448	791.8958	1581.7770	1581.7708	4	0	R.TICAILENYQTEK.G	(Ions score 66)
436 - 448	791.8958	1581.7770	1581.7708	4	0	R.TICAILENYQTEK.G	(Ions score 53)
436 - 448	791.8958	1581.7770	1581.7708	4	0	R.TICAILENYQTEK.G	(Ions score 72)
436 - 448	791.8958	1581.7770	1581.7708	4	0	R.TICAILENYQTEK.G	(Ions score 41)
436 - 448	791.8958	1581.7770	1581.7708	4	0	R.TICAILENYQTEK.G	(Ions score 47)
436 - 448	791.8958	1581.7770	1581.7708	4	0	R.TICAILENYQTEK.G	(Ions score 40)
436 - 448	791.8958	1581.7770	1581.7708	4	0	R.TICAILENYQTEK.G	(Ions score 43)
436 - 448	528.2663	1581.7771	1581.7708	4	0	R.TICAILENYQTEK.G	(Ions score 44)
436 - 448	791.8958	1581.7771	1581.7708	4	0	R.TICAILENYQTEK.G	(Ions score 53)
436 - 448	791.8958	1581.7771	1581.7708	4	0	R.TICAILENYQTEK.G	(Ions score 40)
436 - 448	791.8958	1581.7771	1581.7708	4	0	R.TICAILENYQTEK.G	(Ions score 47)
436 - 448	791.8958	1581.7771	1581.7708	4	0	R.TICAILENYQTEK.G	(Ions score 70)
436 - 448	791.8958	1581.7771	1581.7708	4	0	R.TICAILENYQTEK.G	(Ions score 55)
436 - 448	791.8958	1581.7771	1581.7708	4	0	R.TICAILENYQTEK.G	(Ions score 56)
436 - 448	528.2663	1581.7771	1581.7708	4	0	R.TICAILENYQTEK.G	(Ions score 23)
436 - 448	528.2663	1581.7771	1581.7708	4	0	R.TICAILENYQTEK.G	(Ions score 27)
436 - 448	791.8959	1581.7772	1581.7708	4	0	R.TICAILENYQTEK.G	(Ions score 65)
436 - 448	791.8959	1581.7772	1581.7708	4	0	R.TICAILENYQTEK.G	(Ions score 59)
436 - 448	791.8959	1581.7772	1581.7708	4	0	R.TICAILENYQTEK.G	(Ions score 51)
436 - 448	791.8959	1581.7772	1581.7708	4	0	R.TICAILENYQTEK.G	(Ions score 45)
436 - 448	791.8959	1581.7772	1581.7708	4	0	R.TICAILENYQTEK.G	(Ions score 27)
436 - 448	528.2663	1581.7772	1581.7708	4	0	R.TICAILENYQTEK.G	(Ions score 28)
436 - 448	791.8959	1581.7773	1581.7708	4	0	R.TICAILENYQTEK.G	(Ions score 59)
436 - 448	791.8959	1581.7773	1581.7708	4	0	R.TICAILENYQTEK.G	(Ions score 41)
436 - 448	791.8959	1581.7773	1581.7708	4	0	R.TICAILENYQTEK.G	(Ions score 57)
436 - 448	791.8959	1581.7773	1581.7708	4	0	R.TICAILENYQTEK.G	(Ions score 36)
436 - 448	791.8959	1581.7773	1581.7708	4	0	R.TICAILENYQTEK.G	(Ions score 57)
436 - 448	528.2664	1581.7774	1581.7708	4	0	R.TICAILENYQTEK.G	(Ions score 44)
436 - 448	791.8960	1581.7774	1581.7708	4	0	R.TICAILENYQTEK.G	(Ions score 43)
436 - 448	791.8960	1581.7774	1581.7708	4	0	R.TICAILENYQTEK.G	(Ions score 35)
436 - 448	791.8960	1581.7774	1581.7708	4	0	R.TICAILENYQTEK.G	(Ions score 33)
436 - 448	791.8960	1581.7775	1581.7708	4	0	R.TICAILENYQTEK.G	(Ions score 66)
436 - 448	791.8960	1581.7775	1581.7708	4	0	R.TICAILENYQTEK.G	(Ions score 41)
436 - 448	791.8961	1581.7777	1581.7708	4	0	R.TICAILENYQTEK.G	(Ions score 73)

436 - 448	791.8961	1581.7777	1581.7708	4	0	R.TICAILENYQTEK.G	(Ions score 49)
436 - 448	528.2665	1581.7777	1581.7708	4	0	R.TICAILENYQTEK.G	(Ions score 23)
436 - 448	791.8961	1581.7777	1581.7708	4	0	R.TICAILENYQTEK.G	(Ions score 44)
436 - 448	791.8962	1581.7778	1581.7708	4	0	R.TICAILENYQTEK.G	(Ions score 48)
436 - 448	791.8962	1581.7778	1581.7708	4	0	R.TICAILENYQTEK.G	(Ions score 49)
436 - 448	791.8962	1581.7778	1581.7708	4	0	R.TICAILENYQTEK.G	(Ions score 36)
436 - 448	791.8962	1581.7778	1581.7708	4	0	R.TICAILENYQTEK.G	(Ions score 38)
436 - 448	791.8962	1581.7779	1581.7708	4	0	R.TICAILENYQTEK.G	(Ions score 47)
436 - 448	528.2666	1581.7780	1581.7708	5	0	R.TICAILENYQTEK.G	(Ions score 25)
436 - 448	791.8963	1581.7780	1581.7708	5	0	R.TICAILENYQTEK.G	(Ions score 66)
436 - 448	791.8963	1581.7780	1581.7708	5	0	R.TICAILENYQTEK.G	(Ions score 46)
436 - 448	791.8963	1581.7780	1581.7708	5	0	R.TICAILENYQTEK.G	(Ions score 48)
436 - 448	791.8963	1581.7780	1581.7708	5	0	R.TICAILENYQTEK.G	(Ions score 28)
436 - 448	528.2666	1581.7781	1581.7708	5	0	R.TICAILENYQTEK.G	(Ions score 27)
436 - 448	791.8963	1581.7781	1581.7708	5	0	R.TICAILENYQTEK.G	(Ions score 50)
436 - 448	791.8963	1581.7781	1581.7708	5	0	R.TICAILENYQTEK.G	(Ions score 44)
436 - 448	528.2667	1581.7782	1581.7708	5	0	R.TICAILENYQTEK.G	(Ions score 22)
436 - 448	528.2667	1581.7782	1581.7708	5	0	R.TICAILENYQTEK.G	(Ions score 26)
436 - 448	528.2667	1581.7782	1581.7708	5	0	R.TICAILENYQTEK.G	(Ions score 23)
436 - 448	791.8964	1581.7782	1581.7708	5	0	R.TICAILENYQTEK.G	(Ions score 51)
436 - 448	791.8964	1581.7782	1581.7708	5	0	R.TICAILENYQTEK.G	(Ions score 46)
436 - 448	528.2667	1581.7783	1581.7708	5	0	R.TICAILENYQTEK.G	(Ions score 31)
436 - 448	791.8964	1581.7783	1581.7708	5	0	R.TICAILENYQTEK.G	(Ions score 41)
436 - 448	791.8964	1581.7783	1581.7708	5	0	R.TICAILENYQTEK.G	(Ions score 45)
436 - 448	528.2667	1581.7783	1581.7708	5	0	R.TICAILENYQTEK.G	(Ions score 38)
436 - 448	528.2667	1581.7784	1581.7708	5	0	R.TICAILENYQTEK.G	(Ions score 24)
436 - 448	791.8965	1581.7784	1581.7708	5	0	R.TICAILENYQTEK.G	(Ions score 36)
436 - 448	791.8965	1581.7784	1581.7708	5	0	R.TICAILENYQTEK.G	(Ions score 45)
436 - 448	791.8965	1581.7785	1581.7708	5	0	R.TICAILENYQTEK.G	(Ions score 47)
436 - 448	791.8965	1581.7785	1581.7708	5	0	R.TICAILENYQTEK.G	(Ions score 44)
436 - 448	791.8966	1581.7786	1581.7708	5	0	R.TICAILENYQTEK.G	(Ions score 53)
436 - 455	1154.1020	2306.1894	2306.1828	3	1	R.TICAILENYQTEKGITVPEK.L	(Ions score 68)
436 - 455	1154.1023	2306.1900	2306.1828	3	1	R.TICAILENYQTEKGITVPEK.L	(Ions score 74)
436 - 455	769.7381	2306.1924	2306.1828	4	1	R.TICAILENYQTEKGITVPEK.L	(Ions score 45)
436 - 455	769.7385	2306.1938	2306.1828	5	1	R.TICAILENYQTEKGITVPEK.L	(Ions score 48)
436 - 455	783.7399	2348.1978	2348.1933	2	1	R.TICAILENYQTEKGITVPEK.L	Acetylation (K) (Ions score 67)
436 - 455	1175.1075	2348.2005	2348.1933	3	1	R.TICAILENYQTEKGITVPEK.L	Acetylation (K) (Ions score 75)
436 - 455	1175.1078	2348.2010	2348.1933	3	1	R.TICAILENYQTEKGITVPEK.L	Acetylation (K) (Ions score 53)
436 - 455	783.7416	2348.2031	2348.1933	4	1	R.TICAILENYQTEKGITVPEK.L	Acetylation (K) (Ions score 57)
449 - 455	743.4289	742.4216	742.4225	-1	0	K.GITVPEK.L	(Ions score 24)
449 - 457	492.8083	983.6020	983.6015	1	1	K.GITVPEKLK.E	(Ions score 32)
449 - 457	513.8157	1025.6169	1025.6121	5	1	K.GITVPEKLK.E	Acetylation (K) (Ions score 30)
456 - 482	1021.5606	3061.6600	3061.6562	1	1	K.LKEFMPPGLQELIPFVKPAPIEQEPSK.K	(Ions score 23)
456 - 482	766.4228	3061.6621	3061.6562	2	1	K.LKEFMPPGLQELIPFVKPAPIEQEPSK.K	(Ions score 26)
456 - 482	613.3400	3061.6635	3061.6562	2	1	K.LKEFMPPGLQELIPFVKPAPIEQEPSK.K	(Ions score 23)
456 - 482	766.4244	3061.6687	3061.6562	4	1	K.LKEFMPPGLQELIPFVKPAPIEQEPSK.K	(Ions score 31)
456 - 482	770.4210	3077.6548	3077.6511	1	1	K.LKEFMPPGLQELIPFVKPAPIEQEPSK.K	Oxidation (M) (Ions score 23)
456 - 482	1539.8353	3077.6561	3077.6511	2	1	K.LKEFMPPGLQELIPFVKPAPIEQEPSK.K	Oxidation (M) (Ions score 34)
456 - 482	1026.8928	3077.6565	3077.6511	2	1	K.LKEFMPPGLQELIPFVKPAPIEQEPSK.K	Oxidation (M) (Ions score 27)
456 - 482	770.4217	3077.6575	3077.6511	2	1	K.LKEFMPPGLQELIPFVKPAPIEQEPSK.K	Oxidation (M) (Ions score 28)
456 - 482	1026.8932	3077.6577	3077.6511	2	1	K.LKEFMPPGLQELIPFVKPAPIEQEPSK.K	Oxidation (M) (Ions score 28)
456 - 482	770.4218	3077.6582	3077.6511	2	1	K.LKEFMPPGLQELIPFVKPAPIEQEPSK.K	Oxidation (M) (Ions score 23)
456 - 482	1026.8934	3077.6583	3077.6511	2	1	K.LKEFMPPGLQELIPFVKPAPIEQEPSK.K	Oxidation (M) (Ions score 39)
456 - 482	770.4219	3077.6586	3077.6511	2	1	K.LKEFMPPGLQELIPFVKPAPIEQEPSK.K	Oxidation (M) (Ions score 31)
456 - 482	770.4221	3077.6591	3077.6511	3	1	K.LKEFMPPGLQELIPFVKPAPIEQEPSK.K	Oxidation (M) (Ions score 26)
456 - 482	770.4225	3077.6607	3077.6511	3	1	K.LKEFMPPGLQELIPFVKPAPIEQEPSK.K	Oxidation (M) (Ions score 25)
456 - 482	1539.8382	3077.6619	3077.6511	3	1	K.LKEFMPPGLQELIPFVKPAPIEQEPSK.K	Oxidation (M) (Ions score 38)
456 - 482	770.4230	3077.6628	3077.6511	4	1	K.LKEFMPPGLQELIPFVKPAPIEQEPSK.K	Oxidation (M) (Ions score 24)

456 - 482	770.4235	3077.6648	3077.6511	4	1	K.LKEFMPPGLQELIPFVKPAPIEQEPSK.K	Oxidation (M) (Ions score 22)
456 - 482	770.4236	3077.6651	3077.6511	5	1	K.LKEFMPPGLQELIPFVKPAPIEQEPSK.K	Oxidation (M) (Ions score 24)
456 - 482	776.9251	3103.6713	3103.6667	1	1	K.LKEFMPPGLQELIPFVKPAPIEQEPSK.K	Acetylation (K) (Ions score 31)
456 - 482	776.9261	3103.6753	3103.6667	3	1	K.LKEFMPPGLQELIPFVKPAPIEQEPSK.K	Acetylation (K) (Ions score 39)
456 - 482	776.9272	3103.6796	3103.6667	4	1	K.LKEFMPPGLQELIPFVKPAPIEQEPSK.K	Acetylation (K) (Ions score 36)
456 - 482	1040.8978	3119.6715	3119.6617	3	1	K.LKEFMPPGLQELIPFVKPAPIEQEPSK.K	Acetylation (K); Oxidation (M) (Ions score 28)
456 - 482	1040.8981	3119.6726	3119.6617	3	1	K.LKEFMPPGLQELIPFVKPAPIEQEPSK.K	Acetylation (K); Oxidation (M) (Ions score 30)
456 - 482	1040.8983	3119.6731	3119.6617	4	1	K.LKEFMPPGLQELIPFVKPAPIEQEPSK.K	Acetylation (K); Oxidation (M) (Ions score 22)
456 - 482	1040.8986	3119.6739	3119.6617	4	1	K.LKEFMPPGLQELIPFVKPAPIEQEPSK.K	Acetylation (K); Oxidation (M) (Ions score 37)
458 - 482	1411.2476	2820.4807	2820.4772	1	0	K.EFMPPGLQELIPFVKPAPIEQEPSK.K	(Ions score 44)
458 - 482	941.1677	2820.4812	2820.4772	1	0	K.EFMPPGLQELIPFVKPAPIEQEPSK.K	(Ions score 32)
458 - 482	706.1277	2820.4817	2820.4772	2	0	K.EFMPPGLQELIPFVKPAPIEQEPSK.K	(Ions score 32)
458 - 482	706.1279	2820.4826	2820.4772	2	0	K.EFMPPGLQELIPFVKPAPIEQEPSK.K	(Ions score 31)
458 - 482	941.1684	2820.4834	2820.4772	2	0	K.EFMPPGLQELIPFVKPAPIEQEPSK.K	(Ions score 28)
458 - 482	1419.2447	2836.4749	2836.4721	1	0	K.EFMPPGLQELIPFVKPAPIEQEPSK.K	Oxidation (M) (Ions score 48)
458 - 482	1419.2448	2836.4750	2836.4721	1	0	K.EFMPPGLQELIPFVKPAPIEQEPSK.K	Oxidation (M) (Ions score 32)
458 - 482	1419.2453	2836.4761	2836.4721	1	0	K.EFMPPGLQELIPFVKPAPIEQEPSK.K	Oxidation (M) (Ions score 25)
458 - 482	946.4993	2836.4762	2836.4721	1	0	K.EFMPPGLQELIPFVKPAPIEQEPSK.K	Oxidation (M) (Ions score 27)
458 - 482	710.1264	2836.4764	2836.4721	2	0	K.EFMPPGLQELIPFVKPAPIEQEPSK.K	Oxidation (M) (Ions score 24)
458 - 482	946.4995	2836.4768	2836.4721	2	0	K.EFMPPGLQELIPFVKPAPIEQEPSK.K	Oxidation (M) (Ions score 30)
458 - 482	1419.2457	2836.4768	2836.4721	2	0	K.EFMPPGLQELIPFVKPAPIEQEPSK.K	Oxidation (M) (Ions score 45)
458 - 482	946.4996	2836.4768	2836.4721	2	0	K.EFMPPGLQELIPFVKPAPIEQEPSK.K	Oxidation (M) (Ions score 23)
458 - 482	710.1265	2836.4770	2836.4721	2	0	K.EFMPPGLQELIPFVKPAPIEQEPSK.K	Oxidation (M) (Ions score 23)
458 - 482	946.4996	2836.4771	2836.4721	2	0	K.EFMPPGLQELIPFVKPAPIEQEPSK.K	Oxidation (M) (Ions score 33)
458 - 482	946.4997	2836.4773	2836.4721	2	0	K.EFMPPGLQELIPFVKPAPIEQEPSK.K	Oxidation (M) (Ions score 29)
458 - 482	1419.2460	2836.4775	2836.4721	2	0	K.EFMPPGLQELIPFVKPAPIEQEPSK.K	Oxidation (M) (Ions score 49)
458 - 482	946.4999	2836.4777	2836.4721	2	0	K.EFMPPGLQELIPFVKPAPIEQEPSK.K	Oxidation (M) (Ions score 28)
458 - 482	710.1268	2836.4780	2836.4721	2	0	K.EFMPPGLQELIPFVKPAPIEQEPSK.K	Oxidation (M) (Ions score 27)
458 - 482	946.5001	2836.4785	2836.4721	2	0	K.EFMPPGLQELIPFVKPAPIEQEPSK.K	Oxidation (M) (Ions score 24)
458 - 482	946.5002	2836.4786	2836.4721	2	0	K.EFMPPGLQELIPFVKPAPIEQEPSK.K	Oxidation (M) (Ions score 25)
458 - 482	946.5002	2836.4787	2836.4721	2	0	K.EFMPPGLQELIPFVKPAPIEQEPSK.K	Oxidation (M) (Ions score 33)
458 - 482	946.5002	2836.4788	2836.4721	2	0	K.EFMPPGLQELIPFVKPAPIEQEPSK.K	Oxidation (M) (Ions score 23)
458 - 482	946.5002	2836.4789	2836.4721	2	0	K.EFMPPGLQELIPFVKPAPIEQEPSK.K	Oxidation (M) (Ions score 24)
458 - 482	946.5002	2836.4789	2836.4721	2	0	K.EFMPPGLQELIPFVKPAPIEQEPSK.K	Oxidation (M) (Ions score 34)
458 - 482	946.5003	2836.4792	2836.4721	2	0	K.EFMPPGLQELIPFVKPAPIEQEPSK.K	Oxidation (M) (Ions score 24)
458 - 482	946.5004	2836.4795	2836.4721	3	0	K.EFMPPGLQELIPFVKPAPIEQEPSK.K	Oxidation (M) (Ions score 34)
458 - 482	946.5005	2836.4795	2836.4721	3	0	K.EFMPPGLQELIPFVKPAPIEQEPSK.K	Oxidation (M) (Ions score 22)
458 - 482	946.5005	2836.4798	2836.4721	3	0	K.EFMPPGLQELIPFVKPAPIEQEPSK.K	Oxidation (M) (Ions score 21)
458 - 482	946.5006	2836.4800	2836.4721	3	0	K.EFMPPGLQELIPFVKPAPIEQEPSK.K	Oxidation (M) (Ions score 34)
458 - 482	946.5006	2836.4801	2836.4721	3	0	K.EFMPPGLQELIPFVKPAPIEQEPSK.K	Oxidation (M) (Ions score 25)
458 - 482	946.5007	2836.4804	2836.4721	3	0	K.EFMPPGLQELIPFVKPAPIEQEPSK.K	Oxidation (M) (Ions score 25)
458 - 482	946.5010	2836.4810	2836.4721	3	0	K.EFMPPGLQELIPFVKPAPIEQEPSK.K	Oxidation (M) (Ions score 28)
458 - 482	946.5010	2836.4813	2836.4721	3	0	K.EFMPPGLQELIPFVKPAPIEQEPSK.K	Oxidation (M) (Ions score 28)
458 - 482	946.5010	2836.4813	2836.4721	3	0	K.EFMPPGLQELIPFVKPAPIEQEPSK.K	Oxidation (M) (Ions score 23)
458 - 482	946.5013	2836.4821	2836.4721	4	0	K.EFMPPGLQELIPFVKPAPIEQEPSK.K	Oxidation (M) (Ions score 24)
458 - 483	1475.2932	2948.5719	2948.5721	-0	1	K.EFMPPGLQELIPFVKPAPIEQEPSKK.Q	(Ions score 25)
458 - 483	1475.2935	2948.5725	2948.5721	0	1	K.EFMPPGLQELIPFVKPAPIEQEPSKK.Q	(Ions score 38)
458 - 483	983.8663	2948.5770	2948.5721	2	1	K.EFMPPGLQELIPFVKPAPIEQEPSKK.Q	(Ions score 31)
458 - 483	1483.2900	2964.5655	2964.5670	-1	1	K.EFMPPGLQELIPFVKPAPIEQEPSKK.Q	Oxidation (M) (Ions score 35)
458 - 483	1483.2914	2964.5682	2964.5670	0	1	K.EFMPPGLQELIPFVKPAPIEQEPSKK.Q	Oxidation (M) (Ions score 26)
458 - 483	1483.2933	2964.5721	2964.5670	2	1	K.EFMPPGLQELIPFVKPAPIEQEPSKK.Q	Oxidation (M) (Ions score 27)
458 - 483	1483.2937	2964.5728	2964.5670	2	1	K.EFMPPGLQELIPFVKPAPIEQEPSKK.Q	Oxidation (M) (Ions score 45)
458 - 483	989.1988	2964.5744	2964.5670	2	1	K.EFMPPGLQELIPFVKPAPIEQEPSKK.Q	Oxidation (M) (Ions score 23)
499 - 505	423.7183	845.4221	845.4243	-3	0	R.DVTLENR.L	(Ions score 24)
499 - 505	423.7187	845.4228	845.4243	-2	0	R.DVTLENR.L	(Ions score 28)
499 - 505	423.7189	845.4233	845.4243	-1	0	R.DVTLENR.L	(Ions score 26)
499 - 505	423.7190	845.4235	845.4243	-1	0	R.DVTLENR.L	(Ions score 24)
499 - 505	423.7190	845.4235	845.4243	-1	0	R.DVTLENR.L	(Ions score 26)

499 - 505	423.7190	845.4235	845.4243	-1	0	R.DVTLENR.L	(Ions score 26)
499 - 505	423.7190	845.4235	845.4243	-1	0	R.DVTLENR.L	(Ions score 25)
499 - 505	423.7191	845.4236	845.4243	-1	0	R.DVTLENR.L	(Ions score 27)
499 - 505	423.7191	845.4236	845.4243	-1	0	R.DVTLENR.L	(Ions score 24)
499 - 505	423.7191	845.4237	845.4243	-1	0	R.DVTLENR.L	(Ions score 22)
499 - 505	423.7192	845.4238	845.4243	-1	0	R.DVTLENR.L	(Ions score 24)
499 - 505	423.7192	845.4238	845.4243	-1	0	R.DVTLENR.L	(Ions score 22)
499 - 505	423.7192	845.4239	845.4243	-0	0	R.DVTLENR.L	(Ions score 25)
499 - 505	423.7192	845.4239	845.4243	-0	0	R.DVTLENR.L	(Ions score 25)
499 - 505	423.7192	845.4239	845.4243	-0	0	R.DVTLENR.L	(Ions score 22)
499 - 505	423.7192	845.4239	845.4243	-0	0	R.DVTLENR.L	(Ions score 22)
499 - 505	423.7193	845.4240	845.4243	-0	0	R.DVTLENR.L	(Ions score 26)
499 - 505	423.7193	845.4240	845.4243	-0	0	R.DVTLENR.L	(Ions score 26)
499 - 505	423.7193	845.4240	845.4243	-0	0	R.DVTLENR.L	(Ions score 24)
499 - 505	423.7193	845.4240	845.4243	-0	0	R.DVTLENR.L	(Ions score 25)
499 - 505	423.7193	845.4240	845.4243	-0	0	R.DVTLENR.L	(Ions score 24)
499 - 505	423.7193	845.4241	845.4243	-0	0	R.DVTLENR.L	(Ions score 22)
499 - 505	423.7193	845.4241	845.4243	-0	0	R.DVTLENR.L	(Ions score 22)
499 - 505	423.7193	845.4241	845.4243	-0	0	R.DVTLENR.L	(Ions score 25)
499 - 505	423.7193	845.4241	845.4243	-0	0	R.DVTLENR.L	(Ions score 27)
499 - 505	423.7193	845.4241	845.4243	-0	0	R.DVTLENR.L	(Ions score 25)
499 - 505	423.7193	845.4241	845.4243	-0	0	R.DVTLENR.L	(Ions score 23)
499 - 505	423.7194	845.4242	845.4243	-0	0	R.DVTLENR.L	(Ions score 24)
499 - 505	423.7194	845.4242	845.4243	-0	0	R.DVTLENR.L	(Ions score 24)
499 - 505	423.7194	845.4242	845.4243	-0	0	R.DVTLENR.L	(Ions score 22)
499 - 505	423.7194	845.4242	845.4243	-0	0	R.DVTLENR.L	(Ions score 22)
499 - 505	423.7194	845.4243	845.4243	-0	0	R.DVTLENR.L	(Ions score 26)
499 - 505	423.7194	845.4243	845.4243	0	0	R.DVTLENR.L	(Ions score 23)
499 - 505	423.7194	845.4243	845.4243	0	0	R.DVTLENR.L	(Ions score 25)
499 - 505	423.7194	845.4243	845.4243	0	0	R.DVTLENR.L	(Ions score 24)
499 - 505	423.7195	845.4244	845.4243	0	0	R.DVTLENR.L	(Ions score 27)
499 - 505	423.7195	845.4244	845.4243	0	0	R.DVTLENR.L	(Ions score 27)
499 - 505	423.7195	845.4244	845.4243	0	0	R.DVTLENR.L	(Ions score 28)
499 - 505	423.7195	845.4245	845.4243	0	0	R.DVTLENR.L	(Ions score 24)
499 - 505	423.7195	845.4245	845.4243	0	0	R.DVTLENR.L	(Ions score 25)
499 - 505	423.7195	845.4245	845.4243	0	0	R.DVTLENR.L	(Ions score 24)
499 - 505	423.7195	845.4245	845.4243	0	0	R.DVTLENR.L	(Ions score 24)
499 - 505	423.7195	845.4245	845.4243	0	0	R.DVTLENR.L	(Ions score 26)
499 - 505	423.7195	845.4245	845.4243	0	0	R.DVTLENR.L	(Ions score 22)
499 - 505	423.7195	845.4245	845.4243	0	0	R.DVTLENR.L	(Ions score 23)
499 - 505	423.7195	845.4245	845.4243	0	0	R.DVTLENR.L	(Ions score 24)
499 - 505	423.7196	845.4246	845.4243	0	0	R.DVTLENR.L	(Ions score 26)
499 - 505	423.7196	845.4246	845.4243	0	0	R.DVTLENR.L	(Ions score 26)
499 - 505	423.7196	845.4246	845.4243	0	0	R.DVTLENR.L	(Ions score 26)
499 - 505	423.7196	845.4246	845.4243	0	0	R.DVTLENR.L	(Ions score 25)
499 - 505	423.7196	845.4246	845.4243	0	0	R.DVTLENR.L	(Ions score 25)
499 - 505	423.7196	845.4246	845.4243	0	0	R.DVTLENR.L	(Ions score 25)
499 - 505	423.7196	845.4247	845.4243	0	0	R.DVTLENR.L	(Ions score 24)
499 - 505	423.7196	845.4247	845.4243	0	0	R.DVTLENR.L	(Ions score 25)
499 - 505	423.7196	845.4247	845.4243	0	0	R.DVTLENR.L	(Ions score 24)
499 - 505	423.7196	845.4247	845.4243	0	0	R.DVTLENR.L	(Ions score 24)
499 - 505	423.7196	845.4247	845.4243	1	0	R.DVTLENR.L	(Ions score 28)
499 - 505	423.7196	845.4247	845.4243	1	0	R.DVTLENR.L	(Ions score 27)
499 - 505	423.7196	845.4247	845.4243	1	0	R.DVTLENR.L	(Ions score 25)
499 - 505	423.7196	845.4247	845.4243	1	0	R.DVTLENR.L	(Ions score 29)
499 - 505	423.7196	845.4247	845.4243	1	0	R.DVTLENR.L	(Ions score 24)
499 - 505	423.7197	845.4248	845.4243	1	0	R.DVTLENR.L	(Ions score 24)
499 - 505	423.7197	845.4248	845.4243	1	0	R.DVTLENR.L	(Ions score 25)

499 - 505	423.7197	845.4248	845.4243	1	0	R.DVTLENR.L	(Ions score 25)
499 - 505	423.7197	845.4248	845.4243	1	0	R.DVTLENR.L	(Ions score 24)
499 - 505	423.7197	845.4248	845.4243	1	0	R.DVTLENR.L	(Ions score 24)
499 - 505	423.7197	845.4248	845.4243	1	0	R.DVTLENR.L	(Ions score 25)
499 - 505	423.7197	845.4249	845.4243	1	0	R.DVTLENR.L	(Ions score 26)
499 - 505	423.7197	845.4249	845.4243	1	0	R.DVTLENR.L	(Ions score 25)
499 - 505	423.7197	845.4249	845.4243	1	0	R.DVTLENR.L	(Ions score 24)
499 - 505	423.7197	845.4249	845.4243	1	0	R.DVTLENR.L	(Ions score 24)
499 - 505	423.7197	845.4249	845.4243	1	0	R.DVTLENR.L	(Ions score 27)
499 - 505	423.7197	845.4249	845.4243	1	0	R.DVTLENR.L	(Ions score 24)
499 - 505	423.7197	845.4249	845.4243	1	0	R.DVTLENR.L	(Ions score 24)
499 - 505	423.7197	845.4249	845.4243	1	0	R.DVTLENR.L	(Ions score 22)
499 - 505	423.7198	845.4250	845.4243	1	0	R.DVTLENR.L	(Ions score 23)
499 - 505	423.7198	845.4250	845.4243	1	0	R.DVTLENR.L	(Ions score 25)
499 - 505	423.7198	845.4250	845.4243	1	0	R.DVTLENR.L	(Ions score 26)
499 - 505	423.7198	845.4250	845.4243	1	0	R.DVTLENR.L	(Ions score 24)
499 - 505	423.7198	845.4250	845.4243	1	0	R.DVTLENR.L	(Ions score 25)
499 - 505	423.7198	845.4250	845.4243	1	0	R.DVTLENR.L	(Ions score 26)
499 - 505	423.7198	845.4250	845.4243	1	0	R.DVTLENR.L	(Ions score 22)
499 - 505	423.7198	845.4250	845.4243	1	0	R.DVTLENR.L	(Ions score 24)
499 - 505	423.7198	845.4251	845.4243	1	0	R.DVTLENR.L	(Ions score 25)
499 - 505	423.7198	845.4251	845.4243	1	0	R.DVTLENR.L	(Ions score 24)
499 - 505	423.7198	845.4251	845.4243	1	0	R.DVTLENR.L	(Ions score 27)
499 - 505	423.7198	845.4251	845.4243	1	0	R.DVTLENR.L	(Ions score 24)
499 - 505	423.7198	845.4251	845.4243	1	0	R.DVTLENR.L	(Ions score 25)
499 - 505	423.7198	845.4251	845.4243	1	0	R.DVTLENR.L	(Ions score 26)
499 - 505	423.7198	845.4251	845.4243	1	0	R.DVTLENR.L	(Ions score 22)
499 - 505	423.7198	845.4251	845.4243	1	0	R.DVTLENR.L	(Ions score 26)
499 - 505	423.7199	845.4252	845.4243	1	0	R.DVTLENR.L	(Ions score 25)
499 - 505	423.7199	845.4252	845.4243	1	0	R.DVTLENR.L	(Ions score 23)
499 - 505	423.7199	845.4252	845.4243	1	0	R.DVTLENR.L	(Ions score 24)
499 - 505	423.7199	845.4252	845.4243	1	0	R.DVTLENR.L	(Ions score 26)
499 - 505	423.7199	845.4252	845.4243	1	0	R.DVTLENR.L	(Ions score 26)
499 - 505	423.7199	845.4252	845.4243	1	0	R.DVTLENR.L	(Ions score 26)
499 - 505	423.7199	845.4252	845.4243	1	0	R.DVTLENR.L	(Ions score 24)
499 - 505	423.7199	845.4252	845.4243	1	0	R.DVTLENR.L	(Ions score 25)
499 - 505	423.7199	845.4252	845.4243	1	0	R.DVTLENR.L	(Ions score 25)
499 - 505	423.7199	845.4252	845.4243	1	0	R.DVTLENR.L	(Ions score 24)
499 - 505	423.7199	845.4252	845.4243	1	0	R.DVTLENR.L	(Ions score 22)
499 - 505	423.7199	845.4252	845.4243	1	0	R.DVTLENR.L	(Ions score 22)
499 - 505	423.7199	845.4252	845.4243	1	0	R.DVTLENR.L	(Ions score 24)
499 - 505	423.7199	845.4252	845.4243	1	0	R.DVTLENR.L	(Ions score 24)
499 - 505	423.7199	845.4252	845.4243	1	0	R.DVTLENR.L	(Ions score 28)
499 - 505	423.7199	845.4253	845.4243	1	0	R.DVTLENR.L	(Ions score 25)
499 - 505	423.7199	845.4253	845.4243	1	0	R.DVTLENR.L	(Ions score 22)
499 - 505	423.7199	845.4253	845.4243	1	0	R.DVTLENR.L	(Ions score 24)
499 - 505	423.7199	845.4253	845.4243	1	0	R.DVTLENR.L	(Ions score 24)
499 - 505	423.7199	845.4253	845.4243	1	0	R.DVTLENR.L	(Ions score 22)
499 - 505	423.7199	845.4253	845.4243	1	0	R.DVTLENR.L	(Ions score 26)
499 - 505	423.7199	845.4253	845.4243	1	0	R.DVTLENR.L	(Ions score 26)
499 - 505	423.7199	845.4253	845.4243	1	0	R.DVTLENR.L	(Ions score 25)
499 - 505	423.7199	845.4253	845.4243	1	0	R.DVTLENR.L	(Ions score 23)
499 - 505	423.7199	845.4253	845.4243	1	0	R.DVTLENR.L	(Ions score 22)
499 - 505	423.7199	845.4253	845.4243	1	0	R.DVTLENR.L	(Ions score 24)
499 - 505	423.7199	845.4253	845.4243	1	0	R.DVTLENR.L	(Ions score 24)
499 - 505	423.7200	845.4254	845.4243	1	0	R.DVTLENR.L	(Ions score 25)
499 - 505	423.7200	845.4254	845.4243	1	0	R.DVTLENR.L	(Ions score 24)
499 - 505	423.7200	845.4254	845.4243	1	0	R.DVTLENR.L	(Ions score 28)

499 - 505	423.7200	845.4254	845.4243	1	0	R.DVTLENR.L	(Ions score 23)
499 - 505	423.7200	845.4254	845.4243	1	0	R.DVTLENR.L	(Ions score 24)
499 - 505	423.7200	845.4255	845.4243	1	0	R.DVTLENR.L	(Ions score 24)
499 - 505	423.7200	845.4255	845.4243	1	0	R.DVTLENR.L	(Ions score 25)
499 - 505	423.7200	845.4255	845.4243	1	0	R.DVTLENR.L	(Ions score 25)
499 - 505	423.7200	845.4255	845.4243	1	0	R.DVTLENR.L	(Ions score 24)
499 - 505	423.7200	845.4255	845.4243	1	0	R.DVTLENR.L	(Ions score 27)
499 - 505	423.7200	845.4255	845.4243	1	0	R.DVTLENR.L	(Ions score 28)
499 - 505	423.7200	845.4255	845.4243	1	0	R.DVTLENR.L	(Ions score 24)
499 - 505	423.7200	845.4255	845.4243	1	0	R.DVTLENR.L	(Ions score 25)
499 - 505	423.7200	845.4255	845.4243	1	0	R.DVTLENR.L	(Ions score 27)
499 - 505	423.7200	845.4255	845.4243	1	0	R.DVTLENR.L	(Ions score 22)
499 - 505	423.7200	845.4255	845.4243	1	0	R.DVTLENR.L	(Ions score 24)
499 - 505	423.7201	845.4256	845.4243	2	0	R.DVTLENR.L	(Ions score 26)
499 - 505	423.7201	845.4256	845.4243	2	0	R.DVTLENR.L	(Ions score 24)
499 - 505	423.7201	845.4256	845.4243	2	0	R.DVTLENR.L	(Ions score 30)
499 - 505	423.7201	845.4256	845.4243	2	0	R.DVTLENR.L	(Ions score 25)
499 - 505	423.7201	845.4256	845.4243	2	0	R.DVTLENR.L	(Ions score 25)
499 - 505	423.7201	845.4256	845.4243	2	0	R.DVTLENR.L	(Ions score 21)
499 - 505	423.7201	845.4256	845.4243	2	0	R.DVTLENR.L	(Ions score 26)
499 - 505	423.7201	845.4256	845.4243	2	0	R.DVTLENR.L	(Ions score 21)
499 - 505	423.7201	845.4256	845.4243	2	0	R.DVTLENR.L	(Ions score 24)
499 - 505	423.7201	845.4256	845.4243	2	0	R.DVTLENR.L	(Ions score 29)
499 - 505	423.7201	845.4257	845.4243	2	0	R.DVTLENR.L	(Ions score 24)
499 - 505	423.7201	845.4257	845.4243	2	0	R.DVTLENR.L	(Ions score 26)
499 - 505	423.7201	845.4257	845.4243	2	0	R.DVTLENR.L	(Ions score 23)
499 - 505	423.7201	845.4257	845.4243	2	0	R.DVTLENR.L	(Ions score 23)
499 - 505	423.7201	845.4257	845.4243	2	0	R.DVTLENR.L	(Ions score 28)
499 - 505	423.7201	845.4257	845.4243	2	0	R.DVTLENR.L	(Ions score 22)
499 - 505	423.7201	845.4257	845.4243	2	0	R.DVTLENR.L	(Ions score 25)
499 - 505	423.7201	845.4257	845.4243	2	0	R.DVTLENR.L	(Ions score 25)
499 - 505	423.7201	845.4257	845.4243	2	0	R.DVTLENR.L	(Ions score 25)
499 - 505	423.7201	845.4257	845.4243	2	0	R.DVTLENR.L	(Ions score 26)
499 - 505	423.7201	845.4257	845.4243	2	0	R.DVTLENR.L	(Ions score 24)
499 - 505	423.7201	845.4257	845.4243	2	0	R.DVTLENR.L	(Ions score 29)
499 - 505	423.7201	845.4257	845.4243	2	0	R.DVTLENR.L	(Ions score 23)
499 - 505	423.7201	845.4257	845.4243	2	0	R.DVTLENR.L	(Ions score 25)
499 - 505	423.7201	845.4257	845.4243	2	0	R.DVTLENR.L	(Ions score 29)
499 - 505	423.7202	845.4258	845.4243	2	0	R.DVTLENR.L	(Ions score 25)
499 - 505	423.7202	845.4258	845.4243	2	0	R.DVTLENR.L	(Ions score 27)
499 - 505	423.7202	845.4258	845.4243	2	0	R.DVTLENR.L	(Ions score 26)
499 - 505	423.7202	845.4258	845.4243	2	0	R.DVTLENR.L	(Ions score 25)
499 - 505	423.7202	845.4258	845.4243	2	0	R.DVTLENR.L	(Ions score 27)
499 - 505	423.7202	845.4258	845.4243	2	0	R.DVTLENR.L	(Ions score 22)
499 - 505	423.7202	845.4258	845.4243	2	0	R.DVTLENR.L	(Ions score 26)
499 - 505	423.7202	845.4258	845.4243	2	0	R.DVTLENR.L	(Ions score 28)
499 - 505	423.7202	845.4259	845.4243	2	0	R.DVTLENR.L	(Ions score 23)
499 - 505	423.7202	845.4259	845.4243	2	0	R.DVTLENR.L	(Ions score 24)
499 - 505	423.7202	845.4259	845.4243	2	0	R.DVTLENR.L	(Ions score 26)
499 - 505	423.7202	845.4259	845.4243	2	0	R.DVTLENR.L	(Ions score 25)
499 - 505	423.7202	845.4259	845.4243	2	0	R.DVTLENR.L	(Ions score 27)
499 - 505	423.7202	845.4259	845.4243	2	0	R.DVTLENR.L	(Ions score 22)
499 - 505	423.7202	845.4259	845.4243	2	0	R.DVTLENR.L	(Ions score 24)
499 - 505	423.7202	845.4259	845.4243	2	0	R.DVTLENR.L	(Ions score 24)
499 - 505	423.7202	845.4259	845.4243	2	0	R.DVTLENR.L	(Ions score 27)
499 - 505	423.7202	845.4259	845.4243	2	0	R.DVTLENR.L	(Ions score 24)
499 - 505	423.7202	845.4259	845.4243	2	0	R.DVTLENR.L	(Ions score 25)
499 - 505	423.7202	845.4259	845.4243	2	0	R.DVTLENR.L	(Ions score 22)

499 - 505	423.7202	845.4259	845.4243	2	0	R.DVTLENR.L	(Ions score 27)
499 - 505	423.7202	845.4259	845.4243	2	0	R.DVTLENR.L	(Ions score 26)
499 - 505	423.7202	845.4259	845.4243	2	0	R.DVTLENR.L	(Ions score 27)
499 - 505	423.7203	845.4260	845.4243	2	0	R.DVTLENR.L	(Ions score 29)
499 - 505	423.7203	845.4260	845.4243	2	0	R.DVTLENR.L	(Ions score 26)
499 - 505	423.7203	845.4260	845.4243	2	0	R.DVTLENR.L	(Ions score 24)
499 - 505	423.7203	845.4260	845.4243	2	0	R.DVTLENR.L	(Ions score 24)
499 - 505	423.7203	845.4260	845.4243	2	0	R.DVTLENR.L	(Ions score 25)
499 - 505	423.7203	845.4260	845.4243	2	0	R.DVTLENR.L	(Ions score 24)
499 - 505	423.7203	845.4260	845.4243	2	0	R.DVTLENR.L	(Ions score 29)
499 - 505	423.7203	845.4260	845.4243	2	0	R.DVTLENR.L	(Ions score 24)
499 - 505	423.7203	845.4260	845.4243	2	0	R.DVTLENR.L	(Ions score 25)
499 - 505	423.7203	845.4260	845.4243	2	0	R.DVTLENR.L	(Ions score 25)
499 - 505	423.7203	845.4260	845.4243	2	0	R.DVTLENR.L	(Ions score 24)
499 - 505	423.7203	845.4261	845.4243	2	0	R.DVTLENR.L	(Ions score 24)
499 - 505	423.7203	845.4261	845.4243	2	0	R.DVTLENR.L	(Ions score 24)
499 - 505	423.7203	845.4261	845.4243	2	0	R.DVTLENR.L	(Ions score 25)
499 - 505	423.7203	845.4261	845.4243	2	0	R.DVTLENR.L	(Ions score 27)
499 - 505	423.7203	845.4261	845.4243	2	0	R.DVTLENR.L	(Ions score 24)
499 - 505	423.7204	845.4262	845.4243	2	0	R.DVTLENR.L	(Ions score 27)
499 - 505	423.7204	845.4262	845.4243	2	0	R.DVTLENR.L	(Ions score 24)
499 - 505	423.7204	845.4262	845.4243	2	0	R.DVTLENR.L	(Ions score 24)
499 - 505	423.7204	845.4262	845.4243	2	0	R.DVTLENR.L	(Ions score 24)
499 - 505	423.7204	845.4262	845.4243	2	0	R.DVTLENR.L	(Ions score 23)
499 - 505	423.7204	845.4263	845.4243	2	0	R.DVTLENR.L	(Ions score 23)
499 - 505	423.7204	845.4263	845.4243	2	0	R.DVTLENR.L	(Ions score 25)
499 - 505	423.7204	845.4263	845.4243	2	0	R.DVTLENR.L	(Ions score 22)
499 - 505	423.7204	845.4263	845.4243	2	0	R.DVTLENR.L	(Ions score 24)
499 - 505	423.7204	845.4263	845.4243	2	0	R.DVTLENR.L	(Ions score 24)
499 - 505	423.7204	845.4263	845.4243	2	0	R.DVTLENR.L	(Ions score 26)
499 - 505	423.7204	845.4263	845.4243	2	0	R.DVTLENR.L	(Ions score 23)
499 - 505	423.7204	845.4263	845.4243	2	0	R.DVTLENR.L	(Ions score 22)
499 - 505	423.7204	845.4263	845.4243	2	0	R.DVTLENR.L	(Ions score 27)
499 - 505	423.7204	845.4263	845.4243	2	0	R.DVTLENR.L	(Ions score 28)
499 - 505	423.7204	845.4263	845.4243	2	0	R.DVTLENR.L	(Ions score 23)
499 - 505	423.7204	845.4263	845.4243	2	0	R.DVTLENR.L	(Ions score 23)
499 - 505	423.7204	845.4263	845.4243	2	0	R.DVTLENR.L	(Ions score 25)
499 - 505	423.7204	845.4263	845.4243	2	0	R.DVTLENR.L	(Ions score 28)
499 - 505	423.7205	845.4263	845.4243	2	0	R.DVTLENR.L	(Ions score 25)
499 - 505	423.7205	845.4264	845.4243	2	0	R.DVTLENR.L	(Ions score 25)
499 - 505	423.7205	845.4264	845.4243	2	0	R.DVTLENR.L	(Ions score 30)
499 - 505	423.7205	845.4264	845.4243	2	0	R.DVTLENR.L	(Ions score 24)
499 - 505	423.7205	845.4264	845.4243	3	0	R.DVTLENR.L	(Ions score 24)
499 - 505	423.7205	845.4264	845.4243	3	0	R.DVTLENR.L	(Ions score 27)
499 - 505	423.7205	845.4264	845.4243	3	0	R.DVTLENR.L	(Ions score 24)
499 - 505	423.7205	845.4264	845.4243	3	0	R.DVTLENR.L	(Ions score 24)
499 - 505	423.7205	845.4265	845.4243	3	0	R.DVTLENR.L	(Ions score 22)
499 - 505	423.7205	845.4265	845.4243	3	0	R.DVTLENR.L	(Ions score 22)
499 - 505	423.7205	845.4265	845.4243	3	0	R.DVTLENR.L	(Ions score 24)
499 - 505	423.7205	845.4265	845.4243	3	0	R.DVTLENR.L	(Ions score 24)
499 - 505	423.7205	845.4265	845.4243	3	0	R.DVTLENR.L	(Ions score 28)
499 - 505	423.7206	845.4266	845.4243	3	0	R.DVTLENR.L	(Ions score 26)
499 - 505	423.7206	845.4266	845.4243	3	0	R.DVTLENR.L	(Ions score 27)
499 - 505	423.7206	845.4266	845.4243	3	0	R.DVTLENR.L	(Ions score 25)
499 - 505	423.7206	845.4266	845.4243	3	0	R.DVTLENR.L	(Ions score 27)
499 - 505	423.7206	845.4266	845.4243	3	0	R.DVTLENR.L	(Ions score 22)
499 - 505	423.7206	845.4266	845.4243	3	0	R.DVTLENR.L	(Ions score 26)
499 - 505	423.7206	845.4266	845.4243	3	0	R.DVTLENR.L	(Ions score 24)

499 - 505	423.7206	845.4266	845.4243	3	0	R.DVTLENR.L	(Ions score 27)
499 - 505	423.7206	845.4266	845.4243	3	0	R.DVTLENR.L	(Ions score 25)
499 - 505	423.7206	845.4267	845.4243	3	0	R.DVTLENR.L	(Ions score 25)
499 - 505	423.7206	845.4267	845.4243	3	0	R.DVTLENR.L	(Ions score 25)
499 - 505	423.7206	845.4267	845.4243	3	0	R.DVTLENR.L	(Ions score 25)
499 - 505	423.7206	845.4267	845.4243	3	0	R.DVTLENR.L	(Ions score 27)
499 - 505	423.7206	845.4267	845.4243	3	0	R.DVTLENR.L	(Ions score 23)
499 - 505	423.7206	845.4267	845.4243	3	0	R.DVTLENR.L	(Ions score 25)
499 - 505	423.7206	845.4267	845.4243	3	0	R.DVTLENR.L	(Ions score 25)
499 - 505	423.7207	845.4268	845.4243	3	0	R.DVTLENR.L	(Ions score 22)
499 - 505	423.7207	845.4268	845.4243	3	0	R.DVTLENR.L	(Ions score 24)
499 - 505	423.7207	845.4268	845.4243	3	0	R.DVTLENR.L	(Ions score 25)
499 - 505	423.7207	845.4268	845.4243	3	0	R.DVTLENR.L	(Ions score 26)
499 - 505	423.7207	845.4269	845.4243	3	0	R.DVTLENR.L	(Ions score 28)
499 - 505	423.7207	845.4269	845.4243	3	0	R.DVTLENR.L	(Ions score 25)
499 - 505	423.7208	845.4270	845.4243	3	0	R.DVTLENR.L	(Ions score 23)
499 - 505	423.7208	845.4270	845.4243	3	0	R.DVTLENR.L	(Ions score 24)
499 - 505	423.7208	845.4270	845.4243	3	0	R.DVTLENR.L	(Ions score 21)
499 - 505	423.7208	845.4270	845.4243	3	0	R.DVTLENR.L	(Ions score 24)
499 - 505	423.7208	845.4270	845.4243	3	0	R.DVTLENR.L	(Ions score 24)
499 - 505	423.7208	845.4270	845.4243	3	0	R.DVTLENR.L	(Ions score 25)
499 - 505	423.7208	845.4270	845.4243	3	0	R.DVTLENR.L	(Ions score 24)
499 - 505	423.7208	845.4270	845.4243	3	0	R.DVTLENR.L	(Ions score 24)
499 - 505	423.7208	845.4270	845.4243	3	0	R.DVTLENR.L	(Ions score 24)
499 - 505	423.7208	845.4271	845.4243	3	0	R.DVTLENR.L	(Ions score 25)
499 - 505	423.7208	845.4271	845.4243	3	0	R.DVTLENR.L	(Ions score 24)
499 - 505	423.7208	845.4271	845.4243	3	0	R.DVTLENR.L	(Ions score 27)
499 - 505	423.7209	845.4272	845.4243	3	0	R.DVTLENR.L	(Ions score 25)
499 - 505	423.7209	845.4273	845.4243	4	0	R.DVTLENR.L	(Ions score 25)
499 - 505	423.7209	845.4273	845.4243	4	0	R.DVTLENR.L	(Ions score 26)
499 - 505	423.7209	845.4273	845.4243	4	0	R.DVTLENR.L	(Ions score 29)
499 - 505	423.7210	845.4274	845.4243	4	0	R.DVTLENR.L	(Ions score 24)
499 - 505	423.7210	845.4274	845.4243	4	0	R.DVTLENR.L	(Ions score 22)
499 - 505	423.7210	845.4275	845.4243	4	0	R.DVTLENR.L	(Ions score 28)
499 - 505	423.7210	845.4275	845.4243	4	0	R.DVTLENR.L	(Ions score 22)
499 - 505	423.7210	845.4275	845.4243	4	0	R.DVTLENR.L	(Ions score 25)
499 - 505	423.7210	845.4275	845.4243	4	0	R.DVTLENR.L	(Ions score 22)
499 - 505	423.7211	845.4276	845.4243	4	0	R.DVTLENR.L	(Ions score 28)
499 - 505	423.7211	845.4276	845.4243	4	0	R.DVTLENR.L	(Ions score 25)
499 - 505	423.7211	845.4276	845.4243	4	0	R.DVTLENR.L	(Ions score 25)
499 - 505	423.7211	845.4276	845.4243	4	0	R.DVTLENR.L	(Ions score 25)
499 - 505	423.7211	845.4277	845.4243	4	0	R.DVTLENR.L	(Ions score 26)
499 - 505	423.7211	845.4277	845.4243	4	0	R.DVTLENR.L	(Ions score 25)
499 - 505	423.7211	845.4277	845.4243	4	0	R.DVTLENR.L	(Ions score 27)
499 - 505	423.7211	845.4277	845.4243	4	0	R.DVTLENR.L	(Ions score 26)
499 - 505	423.7211	845.4277	845.4243	4	0	R.DVTLENR.L	(Ions score 23)
499 - 505	423.7212	845.4278	845.4243	4	0	R.DVTLENR.L	(Ions score 26)
499 - 505	423.7212	845.4278	845.4243	4	0	R.DVTLENR.L	(Ions score 23)
499 - 505	423.7212	845.4278	845.4243	4	0	R.DVTLENR.L	(Ions score 27)
499 - 505	423.7212	845.4278	845.4243	4	0	R.DVTLENR.L	(Ions score 25)
499 - 505	423.7212	845.4279	845.4243	4	0	R.DVTLENR.L	(Ions score 28)
499 - 505	423.7213	845.4280	845.4243	4	0	R.DVTLENR.L	(Ions score 26)
499 - 505	423.7213	845.4280	845.4243	4	0	R.DVTLENR.L	(Ions score 24)
499 - 505	423.7214	845.4282	845.4243	5	0	R.DVTLENR.L	(Ions score 22)
499 - 505	423.7215	845.4284	845.4243	5	0	R.DVTLENR.L	(Ions score 22)
506 - 514	510.7373	1019.4601	1019.4594	1	0	R.LQNMEVTDA.-	(Ions score 30)
506 - 514	510.7376	1019.4606	1019.4594	1	0	R.LQNMEVTDA.-	(Ions score 29)
506 - 514	510.7377	1019.4609	1019.4594	2	0	R.LQNMEVTDA.-	(Ions score 22)

506 - 514	510.7380	1019.4615	1019.4594	2	0	R.LQNMEVTDA.-	(Ions score 25)
506 - 514	510.7381	1019.4616	1019.4594	2	0	R.LQNMEVTDA.-	(Ions score 24)
506 - 514	510.7384	1019.4623	1019.4594	3	0	R.LQNMEVTDA.-	(Ions score 28)
506 - 514	510.7387	1019.4628	1019.4594	3	0	R.LQNMEVTDA.-	(Ions score 30)
506 - 514	510.7391	1019.4636	1019.4594	4	0	R.LQNMEVTDA.-	(Ions score 25)

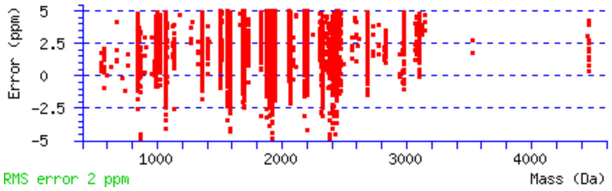

Mascot: http://www.matrixscience.com/

MATRIX SCIENCE Mascot Search Results

User	: Mascot Daemon
Email	: daemon@localhost
Search title	: 7571 7572 Human (D:\Method_Mascot\NCBI_DB_human-decoy.par), submitted from Daemon on MS-BRUKER-WORK
MS data file	: \\scarletvault.scripps.edu\userdata4\QE_data\2022\Nov 2022\11302022\AABT7572.raw
Database	: NCBInr3 (57851050 sequences; 20740007484 residues)
Taxonomy	: Homo sapiens (human) (273087 sequences)
Timestamp	: 16 Dec 2022 at 20:05:46 GMT
Enzyme	: Trypsin
Fixed modifications	: Carbamidomethyl (C)
Variable modifications	: Oxidation (M)
Mass values	: Monoisotopic
Protein Mass	: Unrestricted
Peptide Mass Tolerance	: ± 10 ppm
Fragment Mass Tolerance	: ± 0.01 Da
Max Missed Cleavages	: 1
Instrument type	: ESI-TRAP
Number of queries	: 62214
Protein hits	: gi|16306548 serine--tRNA ligase, cytoplasmic [Homo sapiens]
	gi|13775600 NAD-dependent protein deacetylase sirtuin-2 isoform 1 [Homo sapiens]
	gi|21961605 Keratin 10 [Homo sapiens]
	gi|7331218 keratin 1 [Homo sapiens]
	gi|375314779 keratin 1 [Homo sapiens]
	gi|181402 epidermal cytokeratin 2 [Homo sapiens]
	gi|435476 cytokeratin 9 [Homo sapiens]
	gi|37460 unnamed protein product [Homo sapiens]
	gi|51873887 DNAJC14 protein, partial [Homo sapiens]
	gi|28592 serum albumin [Homo sapiens]
	gi|119625937 hCG2040199, partial [Homo sapiens]
	gi|475157 XP-C repair complementing protein (p125) [Homo sapiens]

	NCBInr3	Decoy	False discovery rate
Peptide matches above identity threshold	7443	67	0.90 %
Peptide matches above homology or identity threshold	7680	95	1.24 %

Select Summary Report

Format As [Select Summary (protein hits)] Help

Significance threshold p< 0.05 Max. number of hits AUTO Show Percolator scores ☐

Standard scoring ◉ MudPIT scoring ○ Ions score or expect cut-off 36 Show sub-sets 1

Show pop-ups ◉ Suppress pop-ups ○ Require bold red ☑

Re-Search ◉ All queries ○ Unassigned ○ Below homology threshold ○ Below identity threshold

1. gi|16306548 **Mass:** 59253 **Score:** 3088 **Matches:** 5929(5929) **Sequences:** 42(42) **emPAI:** 810.68
serine--tRNA ligase, cytoplasmic [Homo sapiens]

Query	Observed	Mr(expt)	Mr(calc)	ppm	Miss	Score	Expect	Rank	Unique	Peptide
16097	716.4048	715.3976	715.3977	-0.17	0	39	0.014	1	U	K.GAVVAGSR.G
3325	435.2151	868.4156	868.4151	0.57	0	48	0.0013	1	U	R.QEVGSHGR.D 21256 21259 3322 3323 3324 3326 3327 21261 21262 21263 21264 3330 3331 3332 3333 21265 21266 3334 3336 3337 3338 3339
22658	458.2675	914.5205	914.5185	2.19	1	43	0.0042	1	U	R.ADNLNKLK.N
6118	495.7850	989.5555	989.5546	0.88	0	72	2.1e-06	1	U	M.VLDLDLFR.V 24388 6079 6081 6084 6085 6087 24389 24391 24392 6091 6092 6093 24393 24394 6094 6096 6097 6099 24395 6100 6102 6104 2[illegible]
24796	503.7436	1005.4726	1005.4702	2.39	0	40	0.0062	1	U	R.IWGDCTVR.K 24781 24782 24789 24790 24793 24800 24802 24808 24810 24812 24818
25428	514.3251	1026.6357	1026.6325	3.14	0	50	0.00015	1	U	R.LLIDEAILK.C 25311 7820 25394 25398 25399 25404 25407 25413 25422 25423 25426 25427 25441
26647	536.7078	1071.4010	1071.3992	1.64	0	59	1.8e-06	1	U	K.SDDNSYDEK.Y 26618 26619 26620 26621 26622 26625 26626 26627 26628 26630 26631 26632 26633 26634 26635 26637 26638 26639 26640 2[illegible]
26955	537.7578	1073.5010	1073.4964	4.24	0	57	7.7e-05	1	U	K.YAGLSTCFR.Q 26733 26737 26746 26749 26763 26765 26774 26776 26777 26778 26782 26787 26791 26802 26808 26810 26816 26841 26843 2[illegible]
27224	542.3074	1082.6002	1082.5972	2.77	0	54	0.00017	1	U	K.DPGLVDQLVK.A 27210 27213 27228 27231 27234
28227	561.3062	1120.5979	1120.5951	2.51	0	51	0.00046	1	U	-.MVLDLDLFR.V 28224 28225 28226 10480
10868	569.3008	1136.5870	1136.5900	-2.61	0	(42)	0.0095	1	U	-.MVLDLDLFR.V 28486 28487
28556	570.8252	1139.6358	1139.6299	5.18	1	52	0.00021	1	U	R.VDKGGDPALIR.E 28543 28544 28547 28548 28551 28552

30070	428.2764	1281.8073	1281.8020	4.16	1	41	0.00023	1	U	K.VRLLIDEAILK.C
30879	444.9231	1331.7475	1331.7449	1.92	1	44	0.0012	1	U	M.VLDLDLFRVDK.G 30880
31362	1358.7689	1357.7616	1357.7606	0.80	1	(42)	0.0027	1	U	R.FKDPGLVDQLVK.A
31423	453.5947	1357.7624	1357.7606	1.36	1	(57)	7.4e-05	1	U	R.FKDPGLVDQLVK.A 31250 31251 31253 31254 31255 31258 31260 31263 31264 31266 31267 31269 31270 31271 31272 31273 31274 31276 3127
31632	679.8899	1357.7653	1357.7606	3.46	1	57	6.1e-05	1	U	R.FKDPGLVDQLVK.A 31256 31262 31286 31358 31364 31400 31441 31450 31522 31528 31549 31584 31587 31591 31623 31627 31630 31655 3165
33620	752.8876	1503.7607	1503.7584	1.49	0	(40)	0.007	1	U	R.GYIPIYTPFFMR.K 33619 33627
33622	502.2609	1503.7608	1503.7584	1.54	0	53	0.00034	1	U	R.GYIPIYTPFFMR.K 33613 33617 33621
33984	755.9005	1509.7864	1509.7827	2.46	0	64	2.4e-05	1	U	R.DEWLRPEDLPIK.Y 33753 33758 33786 33794 33804 33808 33832 33848 33850 33860 33863 33866 33890 33893 33894 33895 33900 33901 3390
34387	507.5930	1519.7572	1519.7534	2.54	0	(38)	0.009	1	U	R.GYIPIYTPFFMR.K
35687	782.3782	1562.7419	1562.7365	3.44	0	85	1.5e-07	1	U	K.IEQFVYSSPHDNK.S 35486 35487 35497 35524 35533 35536 35569 35571 35589 35593 35606 35616 35636 35641 35663 35665 35675 35679 357
35789	521.9229	1562.7469	1562.7365	6.63	0	(61)	5.1e-05	1	U	K.IEQFVYSSPHDNK.S 35481 35483 35490 35491 35493 35496 35498 35499 35500 35501 35504 35505 35506 35509 35512 35513 35514 35515 355
35858	522.5970	1564.7692	1564.7674	1.16	0	(39)	0.016	1	U	K.LDLEAWFPGSGAFR.E 35859 35861 35864 35867 35875
35922	783.3940	1564.7735	1564.7674	3.85	0	68	2e-05	1	U	K.LDLEAWFPGSGAFR.E 17552 35863 35865 35866 35868 35870 35876 35877 35878 35880 35884 35885 35888 35889 35891 35892 35893 35894 35
36574	528.2658	1581.7756	1581.7708	2.99	0	(51)	0.00039	1	U	R.TICAILENYQTEK.G 36463 36466 36468 36476 36479 36489 36496 36498 36512 36513 36514 36526 36529 36530 36531 36533 36534 36535 365
36671	791.8961	1581.7777	1581.7708	4.33	0	73	2.9e-06	1	U	R.TICAILENYQTEK.G 36384 36387 36388 36389 17752 36390 36391 36392 36396 36397 36401 36402 36403 36404 36406 36407 36408 36409 364
38468	553.6335	1657.8788	1657.8709	4.79	1	(60)	4.5e-05	1	U	R.LLIDEAILKCDAER.I 38467 38469
38470	829.9475	1657.8805	1657.8709	5.81	1	82	2.9e-07	1	U	R.LLIDEAILKCDAER.I
38932	421.4861	1681.9153	1681.9151	0.12	0	(50)	0.00031	1	U	K.YLIATSEQPIAALHR.D 38951
39017	841.9665	1681.9184	1681.9151	1.93	0	108	4.8e-10	1	U	K.YLIATSEQPIAALHR.D 38930 38935 38948 38997 38999 39008 39034 39049 39056 39061 39066 39076 39079 39084 39103 39108 39149 39151 3
39031	561.6468	1681.9187	1681.9151	2.14	0	(91)	2e-08	1	U	K.YLIATSEQPIAALHR.D 38909 38910 38911 38912 38913 38914 38915 10503 38916 38917 38918 38919 38920 38921 38922 38923 38924 38925 3
39442	424.2228	1692.8620	1692.8624	-0.22	1	(58)	7.9e-05	1	U	K.KLDLEAWFPGSGAFR.E 39424 39428 39431 39432 39433 39435 39436 39437 39438 39439 39440 39441 39447 39449 39452 39453 39455 39460 3
39482	1693.8712	1692.8639	1692.8624	0.92	1	93	4.1e-08	1	U	K.KLDLEAWFPGSGAFR.E 39426 39445 39461 39501
39555	847.4400	1692.8654	1692.8624	1.77	1	(82)	2.8e-07	1	U	K.KLDLEAWFPGSGAFR.E 39423 39429 39430 39434 39443 39446 39450 39456 39462 39467 39478 39483 39490 39495 39496 39498 39499 39504 3
39737	565.2972	1692.8698	1692.8624	4.39	1	(43)	0.0024	1	U	K.KLDLEAWFPGSGAFR.E 39752 39777
40058	568.2453	1701.7139	1701.7087	3.09	0	(63)	5e-06	1	U	R.ELVSCSNCTDYQAR.R 40007 40017 40038 40039
40098	851.8657	1701.7168	1701.7087	4.79	0	100	1.4e-09	1	U	R.ELVSCSNCTDYQAR.R 39987 39988 39989 39991 39992 39993 39994 39995 39996 39997 39998 39999 40000 40001 40002 40004 40005 40006 40
43037	1833.0643	1832.0571	1832.0560	0.58	0	(78)	1.5e-07	1	U	K.GVLVFLEQALIQYALR.T
43075	611.6951	1832.0636	1832.0560	4.16	0	(93)	2.9e-09	1	U	K.GVLVFLEQALIQYALR.T 13272 43036 43038 43039 43043 43044 43049 43050 43051 43053 43056 43057 43059 43062 43064 43071 43073 43074
43080	917.0393	1832.0640	1832.0560	4.39	0	127	1e-12	1	U	K.GVLVFLEQALIQYALR.T 43035 43040 43041 43042 43045 43046 43047 43048 43052 43054 43055 43058 43060 43061 43063 43065 43066 43067
43801	940.9468	1879.8791	1879.8743	2.55	0	(91)	3.9e-08	1	U	K.VEFVHMLNATMCATTR.T 43595 43598 43599 43601 43602 43603 43604 43613 43615 43620 43621 43625 43626 43629 43630 43633 43634 43635
43879	627.6342	1879.8808	1879.8743	3.46	0	92	3.7e-08	1	U	K.VEFVHMLNATMCATTR.T 43596 43600 43606 43607 43608 43609 43610 43611 43612 43614 43616 43617 43618 43619 13712 43623 43624 43627
44359	948.9437	1895.8729	1895.8692	1.92	0	(66)	8.9e-06	1	U	K.VEFVHMLNATMCATTR.T 44128 44138 44141 44143 44144 44148 44150 44152 44169 44170 44173 44196 44202 44221 44230 44232 44239 44244
44380	948.9438	1895.8731	1895.8692	2.07	0	(52)	0.00026	1	U	K.VEFVHMLNATMCATTR.T 44224 44340 44486 44503
44571	474.9764	1895.8764	1895.8692	3.78	0	(39)	0.0053	1	U	K.VEFVHMLNATMCATTR.T
44601	632.9663	1895.8770	1895.8692	4.09	0	(79)	6.1e-07	1	U	K.VEFVHMLNATMCATTR.T 44124 44125 44129 44130 44131 44132 44134 44136 44139 44140 44146 44149 44151 44153 44155 44157 44158 44159
44653	632.9669	1895.8789	1895.8692	5.12	0	(71)	3.6e-06	1	U	K.VEFVHMLNATMCATTR.T 13850 44499 44524 44621
44891	638.2957	1911.8652	1911.8641	0.55	0	(85)	1.2e-07	1	U	K.VEFVHMLNATMCATTR.T 44855 44856 44858 44863 44866 44868 44869 44870 44878 44879 44882 44887 44892 44903 44905 44906 44908 13988
45135	956.9451	1911.8756	1911.8641	6.01	0	(44)	0.0014	1	U	K.VEFVHMLNATMCATTR.T 44898 44911 44912 44944 44974 44990 44999 45016 45019 45040 45059 45061
45607	962.4648	1922.9151	1922.9084	3.45	0	110	5.5e-10	1	U	K.YSHVDLVVMVDGFEGEK.G 45224 45226 45227 45228 45230 45232 45233 45234 45235 45236 45238 45239 23849 45240 45241 45242 45243 4524
45668	641.9795	1922.9166	1922.9084	4.26	0	(89)	6.7e-08	1	U	K.YSHVDLVVMVDGFEGEK.G 45237 45253 45256 45257 45261 45262 45272 45274 45275 45276 45282 45289 45292 45295 45296 45297 45302 4530
46020	970.4597	1938.9048	1938.9034	0.77	0	(86)	1.1e-07	1	U	K.YSHVDLVVMVDGFEGEK.G 45936 45937 45938 45939 45940 45941 45942 45943 45944 45945 45947 45949 45951 45954 45955 45956 45957 4595
46301	647.3098	1938.9077	1938.9034	2.24	0	(82)	2.9e-07	1	U	K.YSHVDLVVMVDGFEGEK.G 14531 14532 45946 45948 45950 14533 45952 45953 14534 45964 45967 45971 45972 45973 45975 45979 45982 4598
48241	684.6753	2051.0041	2051.0034	0.37	1	(64)	1.9e-05	1	U	K.KYSHVDLVVMVDGFEGEK.G 48235 48237 48238 48239 48240 48242 48244 48248 48250 48254 48259 48261 48263 48264 48266 48267 48271 482
48260	513.7588	2051.0062	2051.0034	1.39	1	(40)	0.0043	1	U	K.KYSHVDLVVMVDGFEGEK.G
48333	1026.5121	2051.0096	2051.0034	3.04	1	95	1.6e-08	1	U	K.KYSHVDLVVMVDGFEGEK.G 48269 48293 48300 48305 48360 48374
48510	1034.5069	2066.9993	2066.9983	0.49	1	(64)	1.8e-05	1	U	K.KYSHVDLVVMVDGFEGEK.G 48520 48550 48581 48598 48771 48865 48866 48892 48928 48953
48742	517.7584	2067.0043	2066.9983	2.92	1	(52)	0.00026	1	U	K.KYSHVDLVVMVDGFEGEK.G 48465 48468 48471 48482 48489 48493 48498 48503 48506 48508 48531 48565 48583 48585 48606 48610 48612 486
48857	690.0094	2067.0063	2066.9983	3.87	1	(77)	9.4e-07	1	U	K.KYSHVDLVVMVDGFEGEK.G 48476 48479 48481 48492 48495 48500 48501 48502 48509 48513 48517 48519 48522 48523 48532 48533 48534 485
49560	1093.5212	2185.0278	2185.0249	1.33	0	114	1.8e-10	1	U	K.EVMQEVAQLSQFDEELYK.V 49507 49508 49509 49512 49513 49514 49517 49518 49519 49520 49522 49523 49524 49527 49528 49529 49531 495
49671	729.3513	2185.0321	2185.0249	3.32	0	(100)	4e-09	1	U	K.EVMQEVAQLSQFDEELYK.V 49510 49511 49515 49516 49521 49525 49526 49530 49532 49539 49541 49546 49549 49553 49554 49555 49559 495
16255	734.6802	2201.0189	2201.0198	-0.41	0	(76)	6.4e-07	1	U	K.EVMQEVAQLSQFDEELYK.V 16250 16251 16252 49894 49895 16253 16254 16256 16257 16258 49901 16259 16260 16261 16262 49919 16263 499
50248	1101.5214	2201.0282	2201.0198	3.80	0	(86)	8.3e-08	1	U	K.EVMQEVAQLSQFDEELYK.V 49882 49883 27715 49885 49886 49887 49888 49889 49890 49891 49897 49898 49899 49902 49904 49905 49906 499
51507	1153.0707	2304.1269	2304.1234	1.52	0	(75)	1.9e-06	1	U	R.EIGNLLHPSVPISNDEDVDNK.V 51505 51506 51509 51511 51512 51514 51518 51521
51517	769.0503	2304.1292	2304.1234	2.52	0	82	4.1e-07	1	U	R.EIGNLLHPSVPISNDEDVDNK.V 51510 51515 51516 51519 51520 51522 51523 51524 51525 51526
51546	1154.1023	2306.1900	2306.1828	3.14	1	74	1.3e-06	1	U	R.TICAILENYQTEKGITVPEK.L 51545
51548	769.7385	2306.1938	2306.1828	4.79	1	(48)	0.00047	1	U	R.TICAILENYQTEKGITVPEK.L 51547
51583	579.2879	2313.1225	2313.1198	1.13	1	(61)	3.6e-05	1	U	R.KEVMQEVAQLSQFDEELYK.V
51606	1157.5696	2313.1247	2313.1198	2.12	1	104	1.7e-09	1	U	R.KEVMQEVAQLSQFDEELYK.V 51576 51589 51590 51595 51600 51601 51603 51609 51610 51615 51620 51638 51639
51618	772.0493	2313.1260	2313.1198	2.69	1	(80)	4.1e-07	1	U	R.KEVMQEVAQLSQFDEELYK.V 51572 51577 51579 51581 51582 51584 51586 51588 51592 51593 51594 51597 51599 51602 51607 51608 51611 516
17305	777.3785	2329.1138	2329.1147	-0.41	1	(63)	7.9e-05	1	U	R.KEVMQEVAQLSQFDEELYK.V 51763 51766 17300 51775 51778 51782 51783 51785 51794 51797 51800 51808 51811 17313 51816 51817 51819 518
51779	1165.5649	2329.1153	2329.1147	0.24	1	(102)	2.4e-09	1	U	R.KEVMQEVAQLSQFDEELYK.V 51769 51772 51773 51776 51780 51781 51788 51789 51791 51792 51793 51798 51799 51801 51802 51809 51812 518

51899	1166.5702	2331.1258	2331.1284	-1.11	1	88	7e-08	1	U	R.VHQFEKIEQFVYSSPHDNK.S	
51903	583.7904	2331.1325	2331.1284	1.77	1	(44)	0.0016	1	U	R.VHQFEKIEQFVYSSPHDNK.S	51900
51904	778.0515	2331.1327	2331.1284	1.86	1	(81)	3e-07	1	U	R.VHQFEKIEQFVYSSPHDNK.S	51901
51905	467.2338	2331.1328	2331.1284	1.91	1	(45)	0.0013	1	U	R.VHQFEKIEQFVYSSPHDNK.S	
52378	597.2745	2385.0690	2385.0772	-3.41	1	(58)	3.7e-05	1	U	K.MMDKVEFVHMLNATMCATTR.T	52375 52379 52389 52391 52394 52396 52397 52400 52403 52407
52406	796.0353	2385.0840	2385.0772	2.85	1	(81)	2.2e-07	1	U	K.MMDKVEFVHMLNATMCATTR.T	52376 52381 52386 52393 52399
52410	1193.5505	2385.0865	2385.0772	3.93	1	(83)	1.4e-07	1	U	K.MMDKVEFVHMLNATMCATTR.T	
52547	601.2729	2401.0623	2401.0721	-4.07	1	(62)	1.3e-05	1	U	K.MMDKVEFVHMLNATMCATTR.T	52549
52589	801.3651	2401.0736	2401.0721	0.64	1	(88)	4.2e-08	1	U	K.MMDKVEFVHMLNATMCATTR.T	52552 52556 52558 52559 52562 52567 52571 52574 52575 52579 52580 52582 52586 52588 52591 52595 52596 52
52616	1201.5449	2401.0752	2401.0721	1.31	1	(55)	7e-05	1	U	K.MMDKVEFVHMLNATMCATTR.T	
52650	1201.5456	2401.0766	2401.0721	1.87	1	(108)	4.2e-10	1	U	K.MMDKVEFVHMLNATMCATTR.T	52617 52637 52638 52672 52707
52677	801.3666	2401.0779	2401.0721	2.42	1	(52)	0.00016	1	U	K.MMDKVEFVHMLNATMCATTR.T	
52952	806.6964	2417.0672	2417.0670	0.09	1	(75)	6.3e-07	1	U	K.MMDKVEFVHMLNATMCATTR.T	52917 52924 52927 52928 52929 52932 52933 52935 52936 52937 52939 52944 18380 18381 52949 52959 52960 52
53003	1209.5421	2417.0697	2417.0670	1.11	1	(51)	0.00014	1	U	K.MMDKVEFVHMLNATMCATTR.T	
53047	1209.5429	2417.0713	2417.0670	1.78	1	(42)	0.0013	1	U	K.MMDKVEFVHMLNATMCATTR.T	
53052	806.6978	2417.0715	2417.0670	1.85	1	(42)	0.0013	1	U	K.MMDKVEFVHMLNATMCATTR.T	
53055	806.6978	2417.0717	2417.0670	1.93	1	(64)	8.4e-06	1	U	K.MMDKVEFVHMLNATMCATTR.T	
53082	806.6982	2417.0728	2417.0670	2.41	1	(67)	4e-06	1	U	K.MMDKVEFVHMLNATMCATTR.T	18379 52985 52988 52996 53004 18387 18393 18396 53044 53051 53060 53068 18405 18409 18412 18415 53152 18
53119	1209.5443	2417.0741	2417.0670	2.95	1	(66)	5.2e-06	1	U	K.MMDKVEFVHMLNATMCATTR.T	53002 53025 53056 53087 53144 53157
53156	1209.5452	2417.0758	2417.0670	3.64	1	111	1.5e-10	1	U	K.MMDKVEFVHMLNATMCATTR.T	52918 52919 52968 52989 53000 53012 53096 53109 53136
53544	1217.5392	2433.0638	2433.0619	0.76	1	(49)	0.00019	1	U	K.MMDKVEFVHMLNATMCATTR.T	
53546	1217.5392	2433.0638	2433.0619	0.77	1	(37)	0.0032	1	U	K.MMDKVEFVHMLNATMCATTR.T	
53703	609.2743	2433.0681	2433.0619	2.55	1	(39)	0.0026	1	U	K.MMDKVEFVHMLNATMCATTR.T	
53842	812.0316	2433.0730	2433.0619	4.56	1	(55)	5.7e-05	1	U	K.MMDKVEFVHMLNATMCATTR.T	53474 53475 53479 53487 53499 53500 53501 53502 53507 53511 53515 53520 53521 53540 53551 53553 53562 53
54127	814.4741	2440.4003	2440.3882	4.96	1	(75)	1.3e-07	1	U	R.GYFLKGVLVFLEQALIQYALR.T	
54128	1221.2080	2440.4014	2440.3882	5.39	1	78	6.3e-08	1	U	R.GYFLKGVLVFLEQALIQYALR.T	
54204	1225.5360	2449.0575	2449.0568	0.26	1	(37)	0.0025	1	U	K.MMDKVEFVHMLNATMCATTR.T	
54277	817.3618	2449.0635	2449.0568	2.73	1	(42)	0.00095	1	U	K.MMDKVEFVHMLNATMCATTR.T	54215
55353	856.0985	2565.2737	2565.2686	2.02	1	43	0.0026	1	U	R.DEWLRPEDLPIKYAGLSTCFR.Q	55354 55355
55440	861.7757	2582.3053	2582.2938	4.46	1	(59)	4.9e-05	1	U	K.EVMQEVAQLSQFDEELYKVIGK.G	55439
55489	867.1060	2598.2962	2598.2887	2.90	1	64	1.6e-05	1	U	K.EVMQEVAQLSQFDEELYKVIGK.G	55490 21187
56005	538.6756	2688.3414	2688.3355	2.20	1	(42)	0.002	1	U	R.EIGNLLHPSVPISNDEDVDNKVER.I	
56057	897.1215	2688.3428	2688.3355	2.72	1	118	5.2e-11	1	U	R.EIGNLLHPSVPISNDEDVDNKVER.I	55917 55924 55925 55932 55940 55951 55954 55962 55966 55974 55981 55987 55990 55992 56031 56033 221
56153	1345.1802	2688.3458	2688.3355	3.84	1	(83)	1.6e-07	1	U	R.EIGNLLHPSVPISNDEDVDNKVER.I	55918 55930 55931 55938 55939 55941 55950 56022 56024 56037 56069 56080 56121 56131 56139 56163 562
56175	673.0939	2688.3467	2688.3355	4.18	1	(65)	1.2e-05	1	U	R.EIGNLLHPSVPISNDEDVDNKVER.I	55920 55921 55936 55937 55943 55944 55945 55946 55947 55949 55953 55956 55958 55959 55960 55961 559
57259	1411.2476	2820.4807	2820.4772	1.26	0	(44)	0.00089	1	U	K.EFMPPGLQELIPFVKPAPIEQEPSK.K	
57337	1419.2460	2836.4775	2836.4721	1.91	0	49	0.00027	1	U	K.EFMPPGLQELIPFVKPAPIEQEPSK.K	57322 57331
57661	1475.2935	2948.5725	2948.5721	0.13	1	(38)	0.0019	1	U	K.EFMPPGLQELIPFVKPAPIEQEPSKK.Q	
57713	1483.2937	2964.5728	2964.5670	1.96	1	45	0.00062	1	U	K.EFMPPGLQELIPFVKPAPIEQEPSKK.Q	
57785	744.1111	2972.4153	2972.4138	0.50	0	(87)	6.8e-08	1	U	K.EPVGDDESVPENVLSFDDLTADALANLK.V	57790 57803
57795	1487.2169	2972.4193	2972.4138	1.82	0	116	8.5e-11	1	U	K.EPVGDDESVPENVLSFDDLTADALANLK.V	57782 57783 57787 57788 57789 57791 57792 57793 57794 57813 57827 57830 57832 57835 57843 5784
57814	991.8147	2972.4223	2972.4138	2.84	0	(108)	4.8e-10	1	U	K.EPVGDDESVPENVLSFDDLTADALANLK.V	57781 57784 57786 57796 57798 57799 57800 57801 57802 57804 57805 57806 57807 57808 57809 5781
58293	1026.8934	3077.6583	3077.6511	2.34	1	39	0.0011	1	U	K.LKEFMPPGLQELIPFVKPAPIEQEPSK.K	
58298	1539.8382	3077.6619	3077.6511	3.50	1	(38)	0.0016	1	U	K.LKEFMPPGLQELIPFVKPAPIEQEPSK.K	
35174	1551.2617	3100.5089	3100.5088	0.03	1	(98)	5.7e-09	1	U	K.KEPVGDDESVPENVLSFDDLTADALANLK.V	35175 35176 35177 35178 58379 58383 58384 58392 58395 58406 58407 58434
58374	776.1353	3100.5122	3100.5088	1.09	1	(96)	8.1e-09	1	U	K.KEPVGDDESVPENVLSFDDLTADALANLK.V	58376 58386 58387 58389 58390 58391 58403 58404 58412 58459
58414	621.1110	3100.5185	3100.5088	3.13	1	(60)	3.3e-05	1	U	K.KEPVGDDESVPENVLSFDDLTADALANLK.V	58370 58394 58397
58440	1034.5143	3100.5209	3100.5088	3.91	1	108	6.1e-10	1	U	K.KEPVGDDESVPENVLSFDDLTADALANLK.V	58369 58371 58372 58373 58375 58377 58378 58380 58381 58382 58385 58388 58393 58396 58398 583

Proteins matching the same set of peptides:
gi\|62896913 **Mass:** 59166 **Score:** 3088 **Matches:** 5929(5929) **Sequences:** 42(42)
seryl-tRNA synthetase variant [Homo sapiens]
gi\|119576775 **Mass:** 59986 **Score:** 3088 **Matches:** 5929(5929) **Sequences:** 42(42)
seryl-tRNA synthetase, isoform CRA_g [Homo sapiens]
gi\|378792521 **Mass:** 60318 **Score:** 3088 **Matches:** 5929(5929) **Sequences:** 42(42)
Chain A, Crystal Structure Of Seryl-Trna Synthetase From Human At 2.9 Angstroms

Proteins matching a subset of these peptides:
gi\|1050527 **Mass:** 59226 **Score:** 3045 **Matches:** 5928(5928) **Sequences:** 41(41)
seryl-tRNA synthetase [Homo sapiens]

gi|111494145 **Mass:** 58883 **Score:** 2955 **Matches:** 5921(5921) **Sequences:** 39(39)
SARS protein [Homo sapiens]
gi|550545083 **Mass:** 54920 **Score:** 2904 **Matches:** 5913(5913) **Sequences:** 38(38)
Chain A, Crystal Structure Of The Human Seryl-trna Synthetase In Complex With Ser-sa At 2.9 Angstrom Resolution
gi|119576772 **Mass:** 62172 **Score:** 2884 **Matches:** 4728(4728) **Sequences:** 40(40)
seryl-tRNA synthetase, isoform CRA_d [Homo sapiens]
gi|14424755 **Mass:** 59257 **Score:** 2738 **Matches:** 4456(4456) **Sequences:** 38(38)
Seryl-tRNA synthetase [Homo sapiens]
gi|119576771 **Mass:** 51371 **Score:** 2714 **Matches:** 5472(5472) **Sequences:** 35(35)
seryl-tRNA synthetase, isoform CRA_c [Homo sapiens]
gi|119576769 **Mass:** 53844 **Score:** 2399 **Matches:** 4019(4019) **Sequences:** 34(34)
seryl-tRNA synthetase, isoform CRA_a [Homo sapiens]
gi|119576774 **Mass:** 48467 **Score:** 2064 **Matches:** 3249(3249) **Sequences:** 30(30)
seryl-tRNA synthetase, isoform CRA_f [Homo sapiens]
gi|119576770 **Mass:** 23967 **Score:** 903 **Matches:** 662(662) **Sequences:** 15(15)
seryl-tRNA synthetase, isoform CRA_b [Homo sapiens]

---

2. gi|13775600 **Mass:** 43782 **Score:** 1621 **Matches:** 163(163) **Sequences:** 22(22) **emPAI:** 28.02
NAD-dependent protein deacetylase sirtuin-2 isoform 1 [Homo sapiens]

Query	Observed	Mr(expt)	Mr(calc)	ppm	Miss	Score	Expect	Rank	Unique	Peptide
21400	437.2402	872.4658	872.4603	6.29	0	42	0.0068	1	U	K.ELEDLVR.R 21401 21402
22818	460.7593	919.5040	919.5015	2.77	0	38	0.011	1	U	K.IFSEVTPK.C
28765	578.8119	1155.6093	1155.6077	1.45	0	56	0.00012	1	U	K.HPEPFFALAK.E 28764 28766 28767
29191	595.7875	1189.5604	1189.5590	1.21	0	40	0.0051	1	U	R.HEYPLSWMK.E
30075	428.9089	1283.7047	1283.7026	1.66	1	(47)	0.00092	1	U	K.KHPEPFFALAK.E 30078
30077	642.8620	1283.7094	1283.7026	5.25	1	55	0.00016	1	U	K.KHPEPFFALAK.E
30787	443.5860	1327.7361	1327.7347	1.07	0	(49)	0.00056	1	U	R.LLDELTLEGVAR.Y 30791
30800	664.8764	1327.7382	1327.7347	2.66	0	90	4.6e-08	1	U	R.LLDELTLEGVAR.Y 30783 30784 30785 30786 30789 30790 30792 30793 30794 30795 14959 30796 30797 30798 14960 14961 30799 30801 3080
16005	705.3161	1408.6177	1408.6156	1.49	0	66	2.4e-05	1	U	R.FFSCMQSDFLK.V 32390 32391 16006
32445	706.8269	1411.6393	1411.6402	-0.63	0	63	1.8e-05	1	U	R.CYTQNIDTLER.I 32446 32448 32449
32599	711.8845	1421.7544	1421.7514	2.11	0	70	4.5e-06	1	U	R.NLFSQTLSLGSQK.E 32598 32600 32602 32605
16059	713.3140	1424.6135	1424.6105	2.12	0	(43)	0.002	1	U	R.FFSCMQSDFLK.V 32623 16060
36242	788.8840	1575.7534	1575.7529	0.35	0	80	4.2e-07	1	U	M.AEPDPSHPLETQAGK.V 36243 36247 36249
36244	526.2594	1575.7562	1575.7529	2.12	0	(44)	0.0019	1	U	M.AEPDPSHPLETQAGK.V
45871	645.0097	1932.0071	1932.0139	-3.50	0	(62)	3.3e-05	1	U	R.VICLVGAGISTSAGIPDFR.S 14303 14304 45872 14305 45876
45874	967.0148	1932.0149	1932.0139	0.54	0	130	3.5e-12	1	U	R.VICLVGAGISTSAGIPDFR.S 45873 45875 45877 45878 45879 45880 45881 45882
48075	1009.0103	2016.0061	2016.0033	1.42	0	(48)	0.00084	1	U	K.YHLPYPEAIFEISYFK.K 48076
48077	673.0095	2016.0067	2016.0033	1.72	0	66	1.4e-05	1	U	K.YHLPYPEAIFEISYFK.K 48074 15174
49126	697.0471	2088.1194	2088.1150	2.12	1	68	5.7e-06	1	U	R.RVICLVGAGISTSAGIPDFR.S 49125
49836	547.5208	2186.0542	2186.0441	4.61	0	45	0.0017	1	U	K.ELYPGQFKPTICHYFMR.L 49835
50401	736.6914	2207.0525	2207.0454	3.19	0	(82)	2.8e-07	1	U	R.EHASIDAQSGAGVPNPSTSASPK.K 50397 50399 50404
50402	1104.5341	2207.0537	2207.0454	3.75	0	89	6e-08	1	U	R.EHASIDAQSGAGVPNPSTSASPK.K 50396
51878	1166.0287	2330.0429	2330.0381	2.06	0	100	2e-09	1	U	K.AGQSDPFLGMIMGLGGGMDFDSK.K
52022	1174.0274	2346.0403	2346.0331	3.08	0	(74)	9.2e-07	1	U	K.AGQSDPFLGMIMGLGGGMDFDSK.K
52164	1180.1712	2358.3278	2358.3233	1.94	0	82	3e-08	1	U	K.VDLLLVMGTSLQVQPFASLISK.A 52169
52168	787.1180	2358.3322	2358.3233	3.78	0	(39)	0.0005	1	U	K.VDLLLVMGTSLQVQPFASLISK.A
52204	1182.0239	2362.0333	2362.0280	2.24	0	(51)	0.00011	1	U	K.AGQSDPFLGMIMGLGGGMDFDSK.K 52200 52201
52205	788.3522	2362.0347	2362.0280	2.84	0	(41)	0.0012	1	U	K.AGQSDPFLGMIMGLGGGMDFDSK.K 52203
52215	788.7243	2363.1511	2363.1465	1.92	1	61	3.3e-05	1	U	R.REHASIDAQSGAGVPNPSTSASPK.K 52214 52216
52218	591.7995	2363.1689	2363.1465	9.45	1	(60)	4.7e-05	1	U	R.REHASIDAQSGAGVPNPSTSASPK.K
52316	1188.1708	2374.3270	2374.3182	3.70	0	(68)	1.6e-06	1	U	K.VDLLLVMGTSLQVQPFASLISK.A
55057	1252.6048	2503.1950	2503.1876	2.99	0	130	4.5e-12	1	U	R.DVAWLGECDQGCLALAELLGWK.K 55048 55052 55058 55063 55065 55070 55071
55061	835.4063	2503.1971	2503.1876	3.81	0	(97)	8.9e-09	1	U	R.DVAWLGECDQGCLALAELLGWK.K 55049 55051 55054 55055 55056 55059 55060 55062 55064 55066 55067 55068
55358	1284.1207	2566.2269	2566.2196	2.85	0	(48)	0.00063	1	U	K.CEDCQSLVKPDIVFFGESLPAR.F 55357
55360	856.4170	2566.2291	2566.2196	3.70	0	76	1e-06	1	U	K.CEDCQSLVKPDIVFFGESLPAR.F 55359
55682	1329.0495	2656.0845	2656.0831	0.54	0	110	1.1e-11	1	U	K.VQEAQDSDSDSEGGAAGGEADMDFLR.N 55681 55685 55686 55688 55693 55696
55697	886.3713	2656.0921	2656.0831	3.38	0	(107)	2.5e-11	1	U	K.VQEAQDSDSDSEGGAAGGEADMDFLR.N 55683 55684 55687 55689 55690 55691 55692 55694 55695 55698
55815	1337.0539	2672.0932	2672.0780	5.68	0	(59)	2.9e-06	1	U	K.VQEAQDSDSDSEGGAAGGEADMDFLR.N 55812 55817
55816	891.7051	2672.0935	2672.0780	5.78	0	(63)	1.1e-06	1	U	K.VQEAQDSDSDSEGGAAGGEADMDFLR.N 55804 55807 55809 55813 55818
58169	1013.1324	3036.3755	3036.3706	1.62	0	(65)	8.1e-06	1	U	R.IAGLEQEDLVEAHGTFYTSHCVSASCR.H 58175

58185	760.1059	3036.3943	3036.3706	7.82	0	66	5.4e-06	1	U	R.IAGLEQEDLVEAHGTFYTSHCVSASCR.H 58168 58172 58173 58176 58177 58178 58179

Proteins matching a subset of these peptides:

gi|13775602 **Mass:** 40117 **Score:** 1431 **Matches:** 131(131) **Sequences:** 20(20)
NAD-dependent protein deacetylase sirtuin-2 isoform 2 [Homo sapiens]
gi|119577240 **Mass:** 43727 **Score:** 1423 **Matches:** 146(146) **Sequences:** 20(20)
sirtuin (silent mating type information regulation 2 homolog) 2 (S. cerevisiae), isoform CRA_d [Homo sapiens]
gi|24474785 **Mass:** 42140 **Score:** 1393 **Matches:** 130(130) **Sequences:** 19(19)
sirtuin type 2 [Homo sapiens]
gi|119577238 **Mass:** 39989 **Score:** 1387 **Matches:** 129(129) **Sequences:** 19(19)
sirtuin (silent mating type information regulation 2 homolog) 2 (S. cerevisiae), isoform CRA_b [Homo sapiens]
gi|15826438 **Mass:** 37093 **Score:** 1281 **Matches:** 121(121) **Sequences:** 18(18)
Chain C, Human Sirt2 Histone Deacetylase
gi|407280316 **Mass:** 37147 **Score:** 1281 **Matches:** 121(121) **Sequences:** 18(18)
Chain A, Human Sirt2 Histone Deacetylase
gi|460417411 **Mass:** 37237 **Score:** 1281 **Matches:** 121(121) **Sequences:** 18(18)
Chain A, Structure Of Human Sirt2 In Complex With Adp-ribose
gi|675717284 **Mass:** 36201 **Score:** 1272 **Matches:** 103(103) **Sequences:** 18(18)
PREDICTED: NAD-dependent protein deacetylase sirtuin-2 isoform X3 [Pan paniscus]
gi|586500238 **Mass:** 34782 **Score:** 1211 **Matches:** 116(116) **Sequences:** 17(17)
Chain A, Crystal Structure Of Sirt2 In Complex With The Macrocyclic Peptide S2il5
gi|469474239 **Mass:** 37221 **Score:** 1205 **Matches:** 117(117) **Sequences:** 17(17)
Chain A, Re-refined Structure Of The Human Sirt2 Apoform
gi|7677070 **Mass:** 41225 **Score:** 1065 **Matches:** 102(102) **Sequences:** 14(14)
silent information regulator 2 homolog [Homo sapiens]
gi|156713213 **Mass:** 43813 **Score:** 974 **Matches:** 91(91) **Sequences:** 14(14)
silent information regulator 2 [Homo sapiens]
gi|4406630 **Mass:** 29072 **Score:** 927 **Matches:** 86(86) **Sequences:** 14(14)
Similar to rat 5E5 antigen [Homo sapiens]
gi|300797705 **Mass:** 27208 **Score:** 927 **Matches:** 86(86) **Sequences:** 14(14)
NAD-dependent protein deacetylase sirtuin-2 isoform 3 [Homo sapiens]

---

3. gi|21961605 **Mass:** 59020 **Score:** 609 **Matches:** 16(16) **Sequences:** 8(8) **emPAI:** 0.73
Keratin 10 [Homo sapiens]

Query	Observed	Mr(expt)	Mr(calc)	ppm	Miss	Score	Expect	Rank	Unique	Peptide
27914	553.7667	1105.5189	1105.5186	0.27	0	57	0.0001	1	U	K.VTMQNLNDR.L 27912 27913 27915
29323	601.3124	1200.6103	1200.6098	0.38	0	73	3e-06	1	U	R.QSVEADINGLR.R 29324
29895	631.8037	1261.5928	1261.5899	2.33	0	78	8.7e-07	1	U	R.SLLEGEGSSGGGGR.G 29896
32027	691.3291	1380.6437	1380.6408	2.06	0	64	3.9e-05	1	U	R.ALEESNYELEGK.I
32128	695.8462	1389.6778	1389.6736	3.09	0	101	4e-09	1	U	K.QSLEASLAETEGR.Y 15879
33537	747.3729	1492.7313	1492.7270	2.91	1	43	0.0028	1	U	R.SQYEQLAEQNRK.D
49258	1056.5287	2111.0428	2111.0343	4.03	0	(77)	3.4e-06	1	U	K.ADLEMQIESLTEELAYLK.K
49260	704.6889	2111.0449	2111.0343	4.99	0	99	1.8e-08	1	U	K.ADLEMQIESLTEELAYLK.K 49255
56862	916.1457	2745.4153	2745.4119	1.24	0	94	1.1e-08	1	U	R.YCVQLSQIQAQISALEEQLQQIR.A

Proteins matching the same set of peptides:

gi|119581085 **Mass:** 63536 **Score:** 609 **Matches:** 16(16) **Sequences:** 8(8)
keratin 10 (epidermolytic hyperkeratosis; keratosis palmaris et plantaris), isoform CRA_b [Homo sapiens]
gi|195972866 **Mass:** 58994 **Score:** 609 **Matches:** 16(16) **Sequences:** 8(8)
keratin, type I cytoskeletal 10 [Homo sapiens]
gi|269849769 **Mass:** 59020 **Score:** 609 **Matches:** 16(16) **Sequences:** 8(8)
RecName: Full=Keratin, type I cytoskeletal 10; AltName: Full=Cytokeratin-10; Short=CK-10; AltName: Full=Keratin-10; Short=K10 [Homo sapiens]
gi|530412176 **Mass:** 63536 **Score:** 609 **Matches:** 16(16) **Sequences:** 8(8)
PREDICTED: keratin, type I cytoskeletal 10 isoform X1 [Homo sapiens]

Proteins matching a subset of these peptides:

gi|28317 **Mass:** 59720 **Score:** 515 **Matches:** 15(15) **Sequences:** 7(7)
unnamed protein product [Homo sapiens]
gi|186629 **Mass:** 39832 **Score:** 411 **Matches:** 9(9) **Sequences:** 5(5)

keratin 10, partial [Homo sapiens]
gi|307086 **Mass:** 46473 **Score:** 375 **Matches:** 7(7) **Sequences:** 5(5)
keratin-10 [Homo sapiens]
gi|623409 **Mass:** 57384 **Score:** 373 **Matches:** 11(11) **Sequences:** 5(5)
keratin 10 [Homo sapiens]
gi|30377 **Mass:** 46179 **Score:** 73 **Matches:** 2(2) **Sequences:** 1(1)
cytokeratin 13 [Homo sapiens]
gi|34033 **Mass:** 49956 **Score:** 73 **Matches:** 2(2) **Sequences:** 1(1)
keratin 13 [Homo sapiens]
gi|7020236 **Mass:** 55625 **Score:** 73 **Matches:** 2(2) **Sequences:** 1(1)
unnamed protein product [Homo sapiens]
gi|21750830 **Mass:** 38783 **Score:** 73 **Matches:** 2(2) **Sequences:** 1(1)
unnamed protein product [Homo sapiens]
gi|62897663 **Mass:** 49997 **Score:** 73 **Matches:** 2(2) **Sequences:** 1(1)
keratin 13 isoform a variant [Homo sapiens]
gi|118835468 **Mass:** 49898 **Score:** 73 **Matches:** 2(2) **Sequences:** 1(1)
Keratin 13 [Homo sapiens]
gi|119581079 **Mass:** 55550 **Score:** 73 **Matches:** 2(2) **Sequences:** 1(1)
keratin 24 [Homo sapiens]
gi|131412225 **Mass:** 49870 **Score:** 73 **Matches:** 2(2) **Sequences:** 1(1)
keratin, type I cytoskeletal 13 isoform a [Homo sapiens]
gi|131412228 **Mass:** 46151 **Score:** 73 **Matches:** 2(2) **Sequences:** 1(1)
keratin, type I cytoskeletal 13 isoform b [Homo sapiens]
gi|158261511 **Mass:** 49828 **Score:** 73 **Matches:** 2(2) **Sequences:** 1(1)
unnamed protein product [Homo sapiens]
gi|192807292 **Mass:** 55567 **Score:** 73 **Matches:** 2(2) **Sequences:** 1(1)
keratin, type I cytoskeletal 24 [Homo sapiens]
gi|269849755 **Mass:** 49900 **Score:** 73 **Matches:** 2(2) **Sequences:** 1(1)
RecName: Full=Keratin, type I cytoskeletal 13; AltName: Full=Cytokeratin-13; Short=CK-13; AltName: Full=Keratin-13; Short=K13 [Homo sapiens]
gi|578830527 **Mass:** 46458 **Score:** 73 **Matches:** 2(2) **Sequences:** 1(1)
PREDICTED: keratin, type I cytoskeletal 24 isoform X1 [Homo sapiens]
gi|186685 **Mass:** 51010 **Score:** 57 **Matches:** 4(4) **Sequences:** 1(1)
keratin type 16 [Homo sapiens]
gi|386848 **Mass:** 51916 **Score:** 57 **Matches:** 4(4) **Sequences:** 1(1)
keratin [Homo sapiens]
gi|1000377 **Mass:** 1992 **Score:** 57 **Matches:** 4(4) **Sequences:** 1(1)
type I keratin K16, partial [Homo sapiens]
gi|1195531 **Mass:** 51548 **Score:** 57 **Matches:** 4(4) **Sequences:** 1(1)
type I keratin 16 [Homo sapiens]
gi|1332731 **Mass:** 1832 **Score:** 57 **Matches:** 4(4) **Sequences:** 1(1)
keratin 14, K14 {Y129D, rod domain} [human, Dowling-Meara epidermolysis bullosa simplex patient, blood, Peptide Partial Mutant, 16 aa]
gi|12803709 **Mass:** 51905 **Score:** 57 **Matches:** 4(4) **Sequences:** 1(1)
Keratin 14 [Homo sapiens]
gi|15431310 **Mass:** 51875 **Score:** 57 **Matches:** 4(4) **Sequences:** 1(1)
keratin, type I cytoskeletal 14 [Homo sapiens]
gi|24430192 **Mass:** 51578 **Score:** 57 **Matches:** 4(4) **Sequences:** 1(1)
keratin, type I cytoskeletal 16 [Homo sapiens]
gi|31074639 **Mass:** 52578 **Score:** 57 **Matches:** 4(4) **Sequences:** 1(1)
type I inner root sheath specific keratin 25 irs2 [Homo sapiens]
gi|31074641 **Mass:** 50420 **Score:** 57 **Matches:** 4(4) **Sequences:** 1(1)
type I inner root sheath specific keratin 25 irs3 [Homo sapiens]
gi|31074643 **Mass:** 51191 **Score:** 57 **Matches:** 4(4) **Sequences:** 1(1)
type I inner root sheath specific keratin 25 irs4 [Homo sapiens]
gi|31559829 **Mass:** 49858 **Score:** 57 **Matches:** 4(4) **Sequences:** 1(1)
keratin, type I cytoskeletal 25 [Homo sapiens]
gi|34526053 **Mass:** 41924 **Score:** 57 **Matches:** 4(4) **Sequences:** 1(1)
unnamed protein product [Homo sapiens]
gi|34526448 **Mass:** 53733 **Score:** 57 **Matches:** 4(4) **Sequences:** 1(1)
unnamed protein product [Homo sapiens]
gi|114431246 **Mass:** 51163 **Score:** 57 **Matches:** 4(4) **Sequences:** 1(1)

keratin, type I cytoskeletal 28 [Homo sapiens]
gi|119581082 **Mass:** 50421 **Score:** 57 **Matches:** 4(4) **Sequences:** 1(1)
keratin 25C [Homo sapiens]
gi|119581150 **Mass:** 44865 **Score:** 57 **Matches:** 4(4) **Sequences:** 1(1)
keratin 14 (epidermolysis bullosa simplex, Dowling-Meara, Koebner), isoform CRA_b [Homo sapiens]
gi|153945736 **Mass:** 50419 **Score:** 57 **Matches:** 4(4) **Sequences:** 1(1)
keratin, type I cytoskeletal 27 [Homo sapiens]
gi|229463044 **Mass:** 51872 **Score:** 57 **Matches:** 4(4) **Sequences:** 1(1)
RecName: Full=Keratin, type I cytoskeletal 14; AltName: Full=Cytokeratin-14; Short=CK-14; AltName: Full=Keratin-14; Short=K14 [Homo sapiens]
gi|254675185 **Mass:** 52620 **Score:** 57 **Matches:** 4(4) **Sequences:** 1(1)
keratin, type I cytoskeletal 26 [Homo sapiens]

---

4. gi|7331218 **Mass:** 66149 **Score:** 567 **Matches:** 26(26) **Sequences:** 10(10) **emPAI:** 0.82
keratin 1 [Homo sapiens]

Query	Observed	Mr(expt)	Mr(calc)	ppm	Miss	Score	Expect	Rank	Unique	Peptide
24103	487.2714	972.5283	972.5240	4.46	0	44	0.0038	1		K.IEISELNR.V 24095 24105
29072	590.3067	1178.5989	1178.5931	4.90	0	43	0.003	1		K.YEELQITAGR.H
29904	633.3247	1264.6348	1264.6299	3.86	0	41	0.0046	1		R.TNAENEFVTIK.K
30022	639.3612	1276.7079	1276.7027	4.10	0	37	0.01	1		K.LALDLEIATYR.T
14797	651.8619	1301.7092	1301.7078	1.06	0	51	0.00041	1	U	R.SLDLDSIIAEVK.A 30265 30266
30869	666.7680	1331.5215	1331.5122	6.98	0	74	2.4e-07	1		K.NMQDMVEDYR.N 30868
32043	692.3512	1382.6878	1382.6830	3.48	0	53	0.00031	1		K.SLNNQFASFIDK.V
33265	738.3963	1474.7780	1474.7780	0.04	0	63	3.5e-05	1		R.FLEQQNQVLQTK.W 33263 33264
40554	858.9296	1715.8447	1715.8438	0.54	0	88	8.4e-08	1		K.QISNLQQSISDAEQR.G 40557
40555	572.9558	1715.8456	1715.8438	1.04	0	(37)	0.013	1		K.QISNLQQSISDAEQR.G
47782	665.3320	1992.9741	1992.9693	2.37	0	71	4.7e-06	1		R.THNLEPYFESFINNLR.R 47780 47781 47783 47784 47785 47786 47787

Proteins matching the same set of peptides:
gi|11935049 **Mass:** 66198 **Score:** 567 **Matches:** 26(26) **Sequences:** 10(10)
keratin 1 [Homo sapiens]
gi|119395750 **Mass:** 66170 **Score:** 566 **Matches:** 26(26) **Sequences:** 10(10)
keratin, type II cytoskeletal 1 [Homo sapiens]
gi|375314771 **Mass:** 66184 **Score:** 566 **Matches:** 26(26) **Sequences:** 10(10)
keratin 1 [Homo sapiens]
gi|375314773 **Mass:** 66200 **Score:** 566 **Matches:** 26(26) **Sequences:** 10(10)
keratin 1 [Homo sapiens]
gi|375314775 **Mass:** 66101 **Score:** 566 **Matches:** 26(26) **Sequences:** 10(10)
keratin 1 [Homo sapiens]
gi|375314777 **Mass:** 66257 **Score:** 566 **Matches:** 26(26) **Sequences:** 10(10)
keratin 1 [Homo sapiens]
gi|375314781 **Mass:** 66242 **Score:** 566 **Matches:** 26(26) **Sequences:** 10(10)
keratin 1 [Homo sapiens]

Proteins matching a subset of these peptides:
gi|189054178 **Mass:** 66151 **Score:** 522 **Matches:** 23(23) **Sequences:** 9(9)
unnamed protein product [Homo sapiens]
gi|386854 **Mass:** 52928 **Score:** 381 **Matches:** 12(12) **Sequences:** 7(7)
type II keratin subunit protein, partial [Homo sapiens]
gi|31074631 **Mass:** 62049 **Score:** 63 **Matches:** 3(3) **Sequences:** 1(1)
keratin 1b [Homo sapiens]
gi|113414871 **Mass:** 62050 **Score:** 63 **Matches:** 3(3) **Sequences:** 1(1)
Keratin 77 [Homo sapiens]
gi|119617049 **Mass:** 62183 **Score:** 63 **Matches:** 3(3) **Sequences:** 1(1)
keratin 1B [Homo sapiens]
gi|194018511 **Mass:** 62149 **Score:** 63 **Matches:** 3(3) **Sequences:** 1(1)
keratin, type II cytoskeletal 1b [Homo sapiens]

---

5. gi|375314779 **Mass:** 66197 **Score:** 555 **Matches:** 24(24) **Sequences:** 10(10) **emPAI:** 0.82
keratin 1 [Homo sapiens]

Query	Observed	Mr(expt)	Mr(calc)	ppm	Miss	Score	Expect	Rank	Unique	Peptide

24103	487.2714	972.5283	972.5240	4.46	0	44	0.0038	1	K.IEISELNR.V 24095 24105
29072	590.3067	1178.5989	1178.5931	4.90	0	43	0.003	1	K.YEELQITAGR.H
29904	633.3247	1264.6348	1264.6299	3.86	0	41	0.0046	1	R.TNAENEFVTIK.K
30022	639.3612	1276.7079	1276.7027	4.10	0	37	0.01	1	K.LALDLEIATYR.T
30813	**665.3677**	**1328.7208**	**1328.7187**	**1.54**	**0**	**40**	**0.0051**	**1**	**R.NLDLDSIIAEVK.A**
30869	666.7680	1331.5215	1331.5122	6.98	0	74	2.4e-07	1	K.NMQDMVEDYR.N 30868
32043	692.3512	1382.6878	1382.6830	3.48	0	53	0.00031	1	K.SLNNQFASFIDK.V
33265	738.3963	1474.7780	1474.7780	0.04	0	63	3.5e-05	1	R.FLEQQNQVLQTK.W 33263 33264
40554	858.9296	1715.8447	1715.8438	0.54	0	88	8.4e-08	1	K.QISNLQQSISDAEQR.G 40557
40555	572.9558	1715.8456	1715.8438	1.04	0	(37)	0.013	1	K.QISNLQQSISDAEQR.G
47782	665.3320	1992.9741	1992.9693	2.37	0	71	4.7e-06	1	R.THNLEPYFESFINNLR.R 47780 47781 47783 47784 47785 47786 47787

Proteins matching a subset of these peptides:

gi|119617032 **Mass:** 60159 **Score:** 83 **Matches:** 2(2) **Sequences:** 2(2)
keratin 6B, isoform CRA_a [Homo sapiens]
gi|119617033 **Mass:** 60288 **Score:** 83 **Matches:** 2(2) **Sequences:** 2(2)
keratin 6B, isoform CRA_b [Homo sapiens]
gi|119703753 **Mass:** 60315 **Score:** 83 **Matches:** 2(2) **Sequences:** 2(2)
keratin, type II cytoskeletal 6B [Homo sapiens]
gi|34069 **Mass:** 39309 **Score:** 40 **Matches:** 1(1) **Sequences:** 1(1)
unnamed protein product [Homo sapiens]
gi|386849 **Mass:** 60258 **Score:** 40 **Matches:** 1(1) **Sequences:** 1(1)
keratin type II, partial [Homo sapiens]
gi|386850 **Mass:** 54649 **Score:** 40 **Matches:** 1(1) **Sequences:** 1(1)
keratin K5, partial [Homo sapiens]
gi|908790 **Mass:** 60247 **Score:** 40 **Matches:** 1(1) **Sequences:** 1(1)
keratin type II [Homo sapiens]
gi|908801 **Mass:** 60448 **Score:** 40 **Matches:** 1(1) **Sequences:** 1(1)
keratin type II [Homo sapiens]
gi|908803 **Mass:** 60472 **Score:** 40 **Matches:** 1(1) **Sequences:** 1(1)
keratin type II [Homo sapiens]
gi|914833 **Mass:** 42557 **Score:** 40 **Matches:** 1(1) **Sequences:** 1(1)
keratin type II [Homo sapiens]
gi|3901030 **Mass:** 59753 **Score:** 40 **Matches:** 1(1) **Sequences:** 1(1)
cytokeratin type II [Homo sapiens]
gi|5031839 **Mass:** 60293 **Score:** 40 **Matches:** 1(1) **Sequences:** 1(1)
keratin, type II cytoskeletal 6A [Homo sapiens]
gi|7161776 **Mass:** 59779 **Score:** 40 **Matches:** 1(1) **Sequences:** 1(1)
cytokeratin [Homo sapiens]
gi|9739163 **Mass:** 62651 **Score:** 40 **Matches:** 1(1) **Sequences:** 1(1)
keratin 5 [Homo sapiens]
gi|18999435 **Mass:** 62568 **Score:** 40 **Matches:** 1(1) **Sequences:** 1(1)
Keratin 5 [Homo sapiens]
gi|32567786 **Mass:** 58085 **Score:** 40 **Matches:** 1(1) **Sequences:** 1(1)
keratin, type II cytoskeletal 79 [Homo sapiens]
gi|39795269 **Mass:** 58025 **Score:** 40 **Matches:** 1(1) **Sequences:** 1(1)
Keratin 79 [Homo sapiens]
gi|46812692 **Mass:** 60323 **Score:** 40 **Matches:** 1(1) **Sequences:** 1(1)
Keratin 6A [Homo sapiens]
gi|54035279 **Mass:** 58059 **Score:** 40 **Matches:** 1(1) **Sequences:** 1(1)
Keratin 79 [Homo sapiens]
gi|84040267 **Mass:** 60245 **Score:** 40 **Matches:** 1(1) **Sequences:** 1(1)
Keratin 6C [Homo sapiens]
gi|114644568 **Mass:** 60266 **Score:** 40 **Matches:** 1(1) **Sequences:** 1(1)
PREDICTED: keratin, type II cytoskeletal 6A [Pan troglodytes]
gi|119395754 **Mass:** 62568 **Score:** 40 **Matches:** 1(1) **Sequences:** 1(1)
keratin, type II cytoskeletal 5 [Homo sapiens]
gi|119617035 **Mass:** 60144 **Score:** 40 **Matches:** 1(1) **Sequences:** 1(1)
keratin 6E, isoform CRA_b [Homo sapiens]

gi|119617036 **Mass:** 60164 **Score:** 40 **Matches:** 1(1) **Sequences:** 1(1)
hCG2039812, isoform CRA_a [Homo sapiens]
gi|119617038 **Mass:** 38781 **Score:** 40 **Matches:** 1(1) **Sequences:** 1(1)
keratin 5 (epidermolysis bullosa simplex, Dowling-Meara/Kobner/Weber-Cockayne types), isoform CRA_a [Homo sapiens]
gi|119617040 **Mass:** 60317 **Score:** 40 **Matches:** 1(1) **Sequences:** 1(1)
keratin 5 (epidermolysis bullosa simplex, Dowling-Meara/Kobner/Weber-Cockayne types), isoform CRA_c [Homo sapiens]
gi|119617041 **Mass:** 59853 **Score:** 40 **Matches:** 1(1) **Sequences:** 1(1)
keratin 5 (epidermolysis bullosa simplex, Dowling-Meara/Kobner/Weber-Cockayne types), isoform CRA_d [Homo sapiens]
gi|153791158 **Mass:** 59809 **Score:** 40 **Matches:** 1(1) **Sequences:** 1(1)
keratin, type II cytoskeletal 75 [Homo sapiens]
gi|155969697 **Mass:** 60273 **Score:** 40 **Matches:** 1(1) **Sequences:** 1(1)
keratin, type II cytoskeletal 6C [Homo sapiens]
gi|158261513 **Mass:** 60279 **Score:** 40 **Matches:** 1(1) **Sequences:** 1(1)
unnamed protein product [Homo sapiens]
gi|189053798 **Mass:** 60275 **Score:** 40 **Matches:** 1(1) **Sequences:** 1(1)
unnamed protein product [Homo sapiens]
gi|194377194 **Mass:** 55993 **Score:** 40 **Matches:** 1(1) **Sequences:** 1(1)
unnamed protein product [Homo sapiens]
gi|194383916 **Mass:** 55983 **Score:** 40 **Matches:** 1(1) **Sequences:** 1(1)
unnamed protein product [Homo sapiens]
gi|194384240 **Mass:** 59042 **Score:** 40 **Matches:** 1(1) **Sequences:** 1(1)
unnamed protein product [Homo sapiens]
gi|194384468 **Mass:** 27409 **Score:** 40 **Matches:** 1(1) **Sequences:** 1(1)
unnamed protein product [Homo sapiens]
gi|194386492 **Mass:** 65720 **Score:** 40 **Matches:** 1(1) **Sequences:** 1(1)
unnamed protein product [Homo sapiens]
gi|194387918 **Mass:** 58088 **Score:** 40 **Matches:** 1(1) **Sequences:** 1(1)
unnamed protein product [Homo sapiens]
gi|194387990 **Mass:** 58786 **Score:** 40 **Matches:** 1(1) **Sequences:** 1(1)
unnamed protein product [Homo sapiens]
gi|194388850 **Mass:** 58928 **Score:** 40 **Matches:** 1(1) **Sequences:** 1(1)
unnamed protein product [Homo sapiens]

---

6. gi|181402 **Mass:** 66110 **Score:** 184 **Matches:** 8(8) **Sequences:** 4(4) **emPAI:** 0.24
epidermal cytokeratin 2 [Homo sapiens]

Query	Observed	Mr(expt)	Mr(calc)	ppm	Miss	Score	Expect	Rank	Unique	Peptide
24103	487.2714	972.5283	972.5240	4.46	0	44	0.0038	1		K.IEISELNR.V 24095 24105
30813	665.3677	1328.7208	1328.7187	1.54	0	40	0.0051	1		R.NLDLDSIIAEVK.A
**33143**	**730.9060**	**1459.7975**	**1459.7922**	**3.59**	**0**	**37**	**0.0074**	**1**	**U**	**K.VDLLNQEIEFLK.V**
33265	738.3963	1474.7780	1474.7780	0.04	0	63	3.5e-05	1		R.FLEQQNQVLQTK.W 33263 33264

Proteins matching the same set of peptides:
gi|47132620 **Mass:** 65678 **Score:** 184 **Matches:** 8(8) **Sequences:** 4(4)
keratin, type II cytoskeletal 2 epidermal [Homo sapiens]

---

7. gi|435476 **Mass:** 62320 **Score:** 77 **Matches:** 2(2) **Sequences:** 2(2) **emPAI:** 0.12
cytokeratin 9 [Homo sapiens]

Query	Observed	Mr(expt)	Mr(calc)	ppm	Miss	Score	Expect	Rank	Unique	Peptide
**27165**	**541.2521**	**1080.4897**	**1080.4870**	**2.52**	**0**	**39**	**0.0039**	**1**	**U**	**K.STMQELNSR.L**
**43139**	**613.3287**	**1836.9643**	**1836.9581**	**3.35**	**0**	**37**	**0.0084**	**1**	**U**	**R.HGVQELEIELQSQLSK.K**

Proteins matching the same set of peptides:
gi|453155 **Mass:** 62178 **Score:** 77 **Matches:** 2(2) **Sequences:** 2(2)
keratin 9 [Homo sapiens]
gi|55956899 **Mass:** 62255 **Score:** 77 **Matches:** 2(2) **Sequences:** 2(2)
keratin, type I cytoskeletal 9 [Homo sapiens]
gi|119581148 **Mass:** 57754 **Score:** 77 **Matches:** 2(2) **Sequences:** 2(2)
keratin 9 (epidermolytic palmoplantar keratoderma) [Homo sapiens]

Proteins matching a subset of these peptides:
gi|113197968 **Mass:** 48171 **Score:** 37 **Matches:** 1(1) **Sequences:** 1(1)
KRT9 protein, partial [Homo sapiens]

---

8. gi|37460 **Mass:** 27329 **Score:** 58 **Matches:** 1(1) **Sequences:** 1(1) **emPAI:** 0.14
unnamed protein product [Homo sapiens]

Query	Observed	Mr(expt)	Mr(calc)	ppm	Miss	Score	Expect	Rank	Unique	Peptide
32732	716.8771	1431.7397	1431.7358	2.73	0	58	9.9e-05	1	U	K.VLEGNEQFINAAK.I

Proteins matching the same set of peptides:
gi|2136330 **Mass:** 28593 **Score:** 58 **Matches:** 1(1) **Sequences:** 1(1)
trypsin (EC 3.4.21.4) IV form b precursor - human (fragment)
gi|3980129 **Mass:** 28611 **Score:** 58 **Matches:** 1(1) **Sequences:** 1(1)
trypsinogen IV b-form [Homo sapiens]
gi|6066378 **Mass:** 28779 **Score:** 58 **Matches:** 1(1) **Sequences:** 1(1)
trypsinogen IV a-form [Homo sapiens]
gi|20988417 **Mass:** 27702 **Score:** 58 **Matches:** 1(1) **Sequences:** 1(1)
PRSS3 protein, partial [Homo sapiens]
gi|21536452 **Mass:** 27250 **Score:** 58 **Matches:** 1(1) **Sequences:** 1(1)
trypsin-3 isoform 2 preproprotein [Homo sapiens]
gi|29649713 **Mass:** 9218 **Score:** 58 **Matches:** 1(1) **Sequences:** 1(1)
protease serine 1 [Homo sapiens]
gi|33126535 **Mass:** 28751 **Score:** 58 **Matches:** 1(1) **Sequences:** 1(1)
protease serine 4 isoform B [Homo sapiens]
gi|118763987 **Mass:** 27040 **Score:** 58 **Matches:** 1(1) **Sequences:** 1(1)
PRSS3 protein [Homo sapiens]
gi|162330095 **Mass:** 24788 **Score:** 58 **Matches:** 1(1) **Sequences:** 1(1)
Chain A, Human Mesotrypsin Complexed With Bovine Pancreatic Trypsin Inhibitor(Bpti)
gi|170296790 **Mass:** 33276 **Score:** 58 **Matches:** 1(1) **Sequences:** 1(1)
trypsin-3 isoform 1 preproprotein [Homo sapiens]
gi|209572698 **Mass:** 33306 **Score:** 58 **Matches:** 1(1) **Sequences:** 1(1)
RecName: Full=Trypsin-3; AltName: Full=Brain trypsinogen; AltName: Full=Mesotrypsinogen; AltName: Full=Serine protease 3; AltName: Full=Serine protease 4; AltName: Full=Trypsin III; AltName: Full=Try
gi|308193325 **Mass:** 26448 **Score:** 58 **Matches:** 1(1) **Sequences:** 1(1)
trypsin-3 isoform 4 preproprotein [Homo sapiens]
gi|342672030 **Mass:** 28924 **Score:** 58 **Matches:** 1(1) **Sequences:** 1(1)
trypsin-3 isoform 3 preproprotein [Homo sapiens]
gi|404312861 **Mass:** 24864 **Score:** 58 **Matches:** 1(1) **Sequences:** 1(1)
Chain A, Human Mesotrypsin-s39y Complexed With Bovine Pancreatic Trypsin Inhibitor (bpti)

---

9. gi|51873887 **Mass:** 48402 **Score:** 51 **Matches:** 16(16) **Sequences:** 1(1) **emPAI:** 0.08
DNAJC14 protein, partial [Homo sapiens]

Query	Observed	Mr(expt)	Mr(calc)	ppm	Miss	Score	Expect	Rank	Unique	Peptide
17388	778.4097	777.4025	777.4021	0.45	0	51	0.0013	1	U	-.DLDLFR.V 17384 17385 17386 17387 17389 17390 17391 17392 17393 17394 17395 17396 17397 17398 17399

---

10. gi|28592 **Mass:** 71316 **Score:** 43 **Matches:** 1(1) **Sequences:** 1(1) **emPAI:** 0.05
serum albumin [Homo sapiens]

Query	Observed	Mr(expt)	Mr(calc)	ppm	Miss	Score	Expect	Rank	Unique	Peptide
38060	547.3163	1638.9271	1638.9305	-2.06	1	43	0.00073	1	U	K.KVPQVSTPTLVEVSR.N

Proteins matching the same set of peptides:
gi|178345 **Mass:** 71177 **Score:** 43 **Matches:** 1(1) **Sequences:** 1(1)
alloalbumin Venezia [Homo sapiens]
gi|763431 **Mass:** 53416 **Score:** 43 **Matches:** 1(1) **Sequences:** 1(1)
albumin-like [Homo sapiens]
gi|3212456 **Mass:** 68425 **Score:** 43 **Matches:** 1(1) **Sequences:** 1(1)
Chain A, Crystal Structure Of Human Serum Albumin
gi|4502027 **Mass:** 71317 **Score:** 43 **Matches:** 1(1) **Sequences:** 1(1)
serum albumin preproprotein [Homo sapiens]

gi|6013427 **Mass:** 71176 **Score:** 43 **Matches:** 1(1) **Sequences:** 1(1)
serum albumin precursor [Homo sapiens]
gi|6650826 **Mass:** 30084 **Score:** 43 **Matches:** 1(1) **Sequences:** 1(1)
PRO2044 [Homo sapiens]
gi|7770217 **Mass:** 33466 **Score:** 43 **Matches:** 1(1) **Sequences:** 1(1)
PRO2675 [Homo sapiens]
gi|7959791 **Mass:** 33120 **Score:** 43 **Matches:** 1(1) **Sequences:** 1(1)
PRO1708 [Homo sapiens]
gi|11493459 **Mass:** 58513 **Score:** 43 **Matches:** 1(1) **Sequences:** 1(1)
PRO2619 [Homo sapiens]
gi|15679996 **Mass:** 23322 **Score:** 43 **Matches:** 1(1) **Sequences:** 1(1)
Unknown (protein for IMAGE:3934797), partial [Homo sapiens]
gi|23241675 **Mass:** 46442 **Score:** 43 **Matches:** 1(1) **Sequences:** 1(1)
ALB protein [Homo sapiens]
gi|23307793 **Mass:** 71344 **Score:** 43 **Matches:** 1(1) **Sequences:** 1(1)
serum albumin [Homo sapiens]
gi|27692693 **Mass:** 48641 **Score:** 43 **Matches:** 1(1) **Sequences:** 1(1)
ALB protein [Homo sapiens]
gi|31615330 **Mass:** 68406 **Score:** 43 **Matches:** 1(1) **Sequences:** 1(1)
Chain A, Human Serum Albumin Mutant R218h Complexed With Thyroxine (3,3',5,5'-Tetraiodo-L-Thyronine)
gi|31615331 **Mass:** 68366 **Score:** 43 **Matches:** 1(1) **Sequences:** 1(1)
Chain A, Human Serum Albumin Mutant R218p Complexed With Thyroxine (3,3',5,5'-Tetraiodo-L-Thyronine)
gi|51476390 **Mass:** 71353 **Score:** 43 **Matches:** 1(1) **Sequences:** 1(1)
hypothetical protein [Homo sapiens]
gi|55669910 **Mass:** 67174 **Score:** 43 **Matches:** 1(1) **Sequences:** 1(1)
Chain A, Crystal Structure Of The Ga Module Complexed With Human Serum Albumin
gi|62113341 **Mass:** 71092 **Score:** 43 **Matches:** 1(1) **Sequences:** 1(1)
serum albumin [Homo sapiens]
gi|71042087 **Mass:** 23321 **Score:** 43 **Matches:** 1(1) **Sequences:** 1(1)
Chain A, Solution Structure Of Domain 3 From Human Serum Albumin Complexed To An Anti-Apoptotic Ligand Directed Against Bcl- Xl And Bcl-2
gi|78101694 **Mass:** 68398 **Score:** 43 **Matches:** 1(1) **Sequences:** 1(1)
Chain A, Human Serum Albumin Complexed With Myristate And Azapropazone
gi|119626065 **Mass:** 61122 **Score:** 43 **Matches:** 1(1) **Sequences:** 1(1)
albumin, isoform CRA_b [Homo sapiens]
gi|119626068 **Mass:** 47098 **Score:** 43 **Matches:** 1(1) **Sequences:** 1(1)
albumin, isoform CRA_e [Homo sapiens]
gi|119626071 **Mass:** 70564 **Score:** 43 **Matches:** 1(1) **Sequences:** 1(1)
albumin, isoform CRA_h [Homo sapiens]
gi|119626074 **Mass:** 48568 **Score:** 43 **Matches:** 1(1) **Sequences:** 1(1)
albumin, isoform CRA_k [Homo sapiens]
gi|119626079 **Mass:** 23183 **Score:** 43 **Matches:** 1(1) **Sequences:** 1(1)
albumin, isoform CRA_p [Homo sapiens]
gi|119626083 **Mass:** 60211 **Score:** 43 **Matches:** 1(1) **Sequences:** 1(1)
albumin, isoform CRA_t [Homo sapiens]
gi|122920512 **Mass:** 68408 **Score:** 43 **Matches:** 1(1) **Sequences:** 1(1)
Chain A, Human Serum Albumin Complexed With Myristate And Aspirin
gi|157830361 **Mass:** 67988 **Score:** 43 **Matches:** 1(1) **Sequences:** 1(1)
Chain A, Human Serum Albumin In A Complex With Myristic Acid And Tri- Iodobenzoic Acid
gi|158258947 **Mass:** 71218 **Score:** 43 **Matches:** 1(1) **Sequences:** 1(1)
unnamed protein product [Homo sapiens]
gi|168988718 **Mass:** 67773 **Score:** 43 **Matches:** 1(1) **Sequences:** 1(1)
Chain A, Structure Of Human Serum Albumin With S-Naproxen And The Ga Module
gi|189054552 **Mass:** 71343 **Score:** 43 **Matches:** 1(1) **Sequences:** 1(1)
unnamed protein product [Homo sapiens]
gi|194391080 **Mass:** 72370 **Score:** 43 **Matches:** 1(1) **Sequences:** 1(1)
unnamed protein product [Homo sapiens]
gi|194391112 **Mass:** 61132 **Score:** 43 **Matches:** 1(1) **Sequences:** 1(1)
unnamed protein product [Homo sapiens]
gi|228311905 **Mass:** 68126 **Score:** 43 **Matches:** 1(1) **Sequences:** 1(1)
Chain A, Crystal Structure Of Human Serum Albumin Complexed With Myristic Acid And Lysophosphatidylethanolamine

gi|332356380 **Mass:** 68484 **Score:** 43 **Matches:** 1(1) **Sequences:** 1(1)
albumin [Homo sapiens]
gi|582045552 **Mass:** 68239 **Score:** 43 **Matches:** 1(1) **Sequences:** 1(1)
Chain D, Ternary Complex Between Neonatal Fc Receptor, Serum Albumin And Fc

---

11. gi|119625937 **Mass:** 35112 **Score:** 40 **Matches:** 8(8) **Sequences:** 1(1) **emPAI:** 0.11
hCG2040199, partial [Homo sapiens]

Query	Observed	Mr(expt)	Mr(calc)	ppm	Miss	Score	Expect	Rank	Unique	Peptide
18175	801.4734	800.4662	800.4643	2.29	0	40	0.0057	1	U	R.VEEALLK.E 18166 18167 18168 18170 18171 18172 18174

---

12. gi|475157 **Score:** 38 **Matches:** 1(1) **Sequences:** 1(1) **emPAI:** 0.04
XP-C repair complementing protein (p125) [Homo sapiens]

Query	Observed	Mr(expt)	Mr(calc)	ppm	Miss	Score	Expect	Rank	Unique	Peptide
9832	547.3455	546.3382	546.3377	0.90	0	38	0.0087	1	U	K.SLLSK.V

Proteins matching the same set of peptides:
gi|499072 **Score:** 38 **Matches:** 1(1) **Sequences:** 1(1)
gi|4507917 **Score:** 38 **Matches:** 1(1) **Sequences:** 1(1)
gi|16741636 **Score:** 38 **Matches:** 1(1) **Sequences:** 1(1)
gi|30354631 **Score:** 38 **Matches:** 1(1) **Sequences:** 1(1)
gi|34364945 **Score:** 38 **Matches:** 1(1) **Sequences:** 1(1)
gi|47123300 **Score:** 38 **Matches:** 1(1) **Sequences:** 1(1)
gi|71841618 **Score:** 38 **Matches:** 1(1) **Sequences:** 1(1)
gi|71841628 **Score:** 38 **Matches:** 1(1) **Sequences:** 1(1)
gi|71841638 **Score:** 38 **Matches:** 1(1) **Sequences:** 1(1)
gi|158260545 **Score:** 38 **Matches:** 1(1) **Sequences:** 1(1)
gi|190335108 **Score:** 38 **Matches:** 1(1) **Sequences:** 1(1)
gi|190335111 **Score:** 38 **Matches:** 1(1) **Sequences:** 1(1)
gi|190335114 **Score:** 38 **Matches:** 1(1) **Sequences:** 1(1)
gi|190335117 **Score:** 38 **Matches:** 1(1) **Sequences:** 1(1)
gi|194380804 **Score:** 38 **Matches:** 1(1) **Sequences:** 1(1)
gi|224809295 **Score:** 38 **Matches:** 1(1) **Sequences:** 1(1)
gi|226529650 **Score:** 38 **Matches:** 1(1) **Sequences:** 1(1)
gi|297718104 **Score:** 38 **Matches:** 1(1) **Sequences:** 1(1)
gi|608785776 **Score:** 38 **Matches:** 1(1) **Sequences:** 1(1)

Mascot: http://www.matrixscience.com/
